# Blood epigenome-wide association studies of suicide attempt in adults with bipolar disorder

**DOI:** 10.1038/s41398-024-02760-y

**Published:** 2024-01-31

**Authors:** Salahudeen Mirza, Camila N. C. Lima, Alexandra Del Favero-Campbell, Alexandre Rubinstein, Natasha Topolski, Brenda Cabrera-Mendoza, Emese H. C. Kovács, Hilary P. Blumberg, Jenny Gringer Richards, Aislinn J. Williams, John A. Wemmie, Vincent A. Magnotta, Jess G. Fiedorowicz, Marie E. Gaine, Consuelo Walss-Bass, Joao Quevedo, Jair C. Soares, Gabriel R. Fries

**Affiliations:** 1 Translational Psychiatry Program, Faillace Department of Psychiatry and Behavioral Sciences, McGovern Medical School, University of Texas Health Science Center at Houston, (UTHealth), 77054 Houston, TX USA; 2 Institute of Child Development, University of Minnesota, 55455 Minneapolis, MN USA; 3 Department of Psychiatry, Yale School of Medicine, 06510 New Haven, CT USA; 4 Neuroscience Graduate Program, The University of Texas MD Anderson Cancer Center UTHealth Graduate School of Biomedical Sciences, 77054 Houston, TX USA; 5 Department of Neuroscience and Pharmacology, The University of Iowa, 51 Newton Rd, 52242 Iowa City, IA USA; 6 Department of Radiology, The University of Iowa, 200 Hawkins Dr, 52242 Iowa City, IA USA; 7 Department of Psychiatry, The University of Iowa, 200 Hawkins Dr, 52242 Iowa City, IA USA; 8 Iowa Neuroscience Institute, The University of Iowa, 169 Newton Rd, 52242 Iowa City, IA USA; 9 Department of Veterans Affairs Medical Center, Iowa City, IA USA; 10 University of Ottawa Brain and Mind Research Institute, Ottawa Hospital Research Institute, 501 Smyth, K1H 8L6 Ottawa, ON Canada; 11 Pharmaceutical Sciences and Experimental Therapeutics, The University of Iowa, 180 South Grand Ave, 52242 Iowa City, IA USA; 12 Center of Excellence in Mood Disorders, Faillace Department of Psychiatry and Behavioral Sciences, McGovern Medical School, The University of Texas Health Science Center at Houston, 1941 East Rd, 77054 Houston, TX USA; 13 Center for Interventional Psychiatry, Faillace Department of Psychiatry and Behavioral Sciences, McGovern Medical School, The University of Texas Health Science Center at Houston (UTHealth), Houston, 1941 East Rd, 77054 Houston, TX USA

**Keywords:** Predictive markers, Personalized medicine

## Abstract

Suicide attempt (SA) risk is elevated in individuals with bipolar disorder (BD), and DNA methylation patterns may serve as possible biomarkers of SA. We conducted epigenome-wide association studies (EWAS) of blood DNA methylation associated with BD and SA. DNA methylation was measured at >700,000 positions in a discovery cohort of *n* = 84 adults with BD with a history of SA (BD/SA), *n* = 79 adults with BD without history of SA (BD/non-SA), and *n* = 76 non-psychiatric controls (CON). EWAS revealed six differentially methylated positions (DMPs) and seven differentially methylated regions (DMRs) between BD/SA and BD/non-SA, with multiple immune-related genes implicated. There were no epigenome-wide significant differences when BD/SA and BD/non-SA were each compared to CON, and patterns suggested that epigenetics differentiating BD/SA from BD/non-SA do not differentiate BD/non-SA from CON. Weighted gene co-methylation network analysis and trait enrichment analysis of the BD/SA vs. BD/non-SA contrast further corroborated immune system involvement, while gene ontology analysis implicated calcium signalling. In an independent replication cohort of *n* = 48 BD/SA and *n* = 47 BD/non-SA, fold changes at the discovery cohort’s significant sites showed moderate correlation across cohorts and agreement on direction. In both cohorts, classification accuracy for SA history among individuals with BD was highest when methylation at the significant CpG sites as well as information from clinical interviews were combined, with an AUC of 88.8% (CI = 83.8–93.8%) and 82.1% (CI = 73.6–90.5%) for the combined epigenetic-clinical classifier in the discovery and replication cohorts, respectively. Our results provide novel insight to the role of immune system functioning in SA and BD and also suggest that integrating information from multiple levels of analysis holds promise to improve risk assessment for SA in adults with BD.

## Introduction

Suicide is a leading cause of death, with over 47,000 suicide deaths (SD) in the United States in 2021. Many more people attempt suicide each year, with an estimated 1,400,000 suicide attempts (SA) in the US in 2021 [1]. Improving detection of SA risk could be critical to reducing injuries, as well as intervening early to prevent SD. There have been various efforts to identify robust and easily obtainable clinical predictors. For example, in a study from our group with mood disorders subjects, a combination of risk factors including previous hospitalisations for depression, history of psychosis, cocaine dependence, and post-traumatic stress disorder (PTSD) has shown potential for classifying patients based on SA history [2]. However, such classification tools are still nascent, and a recent meta-analysis of canonical risk factors for SA and other suicidal thoughts and behaviours found that there are no strong longitudinal predictors [3].

Applying a multiple-levels-of-analysis approach may be key to understanding the aetiology of SA [4, 5] and identifying signs of risk in vulnerable populations. Integration of information across the biological and clinical domains, when taken together, could illuminate novel pathways and opportunities for intervention. Suicidal behaviour has been shown to have a biological component [6, 7]. Emerging genome-wide association studies (GWAS) are beginning to identify associated genes, but the variance explained is low [8, 9]. Beyond the contributions of the genotype itself, mechanisms which regulate gene expression may also be relevant. DNA methylation, the reversible addition of methyl groups to the DNA sequence, can modulate gene expression and ultimately shape larger biological networks [10]. Accordingly, DNA methylation alterations have been proposed as possible mediators between early life adversity and the development of psychopathology, including suicide risk [11]. Of note, some of the earliest work in psychiatric epigenetics demonstrated DNA methylation alterations related to childhood abuse and SD [12].

Patterns in DNA methylation may also capture genetic and environmental contributions to SA. Epigenome-wide association studies (EWAS), which attempt to surveille DNA methylation across the entire (epi)genome, improve upon many of the limitations of previous studies focused on specific candidate genes [9]. The majority of suicide-focused EWAS have considered SD, as opposed to a few which studied SA [13–15]. There are several indicators that the epigenetic basis may differ between SA and SD [9]. Aside from a variety of epidemiological differences [16], the existing EWAS of SD are predominantly based in brain tissue [9], which is generally inaccessible in living individuals. DNA methylation patterns vary by cell type and can differ between peripheral tissues and the brain [17]. In particular, understanding the contributions of peripherally-measured DNA methylation to SA in vivo would best inform preventive and interventional strategies, such as screening tools. Recently, a large blood EWAS of SA in U.S. military veterans identified three significant differentially methylated positions (DMPs) associated with SA, implicating nervous system-related alterations and overlapping DNA methylation patterns with a variety of risk traits [15]. Carrying this momentum forward, it is important to understand the associated peripheral DNA methylation patterns in other focused subgroups, including specific psychiatric diagnoses, given evidence that the molecular underpinnings of SA may differ across diagnostic populations [9].

Risk for SA is substantially elevated in individuals with bipolar disorder (BD), with 20–60% of individuals having at least one lifetime SA [18]. This elevated risk could be accounted for by patterns of shared biological vulnerability, with recent GWAS suggesting genetic correlation [19]. BD is also associated with other psychiatric comorbidities and early adversities which are known to drive suicide risk [20]. One of the challenges of the extant literature on epigenetics of suicidal behaviour is that a suicidal case group is often compared only to a non-psychiatric control group. A central limitation of this approach is that any pathophysiological alterations described in the cases cannot be ascribed to suicide *vs*. the elevated burden of psychopathology [9]. Therefore, there is a need to carefully study the epigenetics of SA using well-matched reference groups, such as comparing individuals with BD and a history of SA to individuals with BD without such history. Extending the study to also include non-psychiatric controls can inform on the extent to which SA-associated alterations are unique from BD-associated pathology (e.g., whether individuals with BD and SA are showing an exacerbation of BD-associated pathology). Results from EWAS of SA in BD could provide insights to the underlying biology, as well as provide possible diagnostic aids. Critically, this information from the biological domain, instead of being considered independently from the clinical domain, could be integrated with existing measures, such as clinical interviews [21].

In this study, we leveraged a richly phenotyped discovery cohort of individuals with BD stratified by lifetime history of SA, as well as non-psychiatric controls, to understand the underlying epigenetic patterns linked to SA. We conducted EWAS to identify differentially methylated positions (DMPs) and regions (DMRs) associated with BD and SA. We measured the association of these DNA methylation patterns with other traits and biological pathways. To address concerns of replicability, we attempted to validate our findings in an independent replication cohort of individuals with BD and SA. Finally, to demonstrate the potential of integrating information across multiple levels of analysis to inform risk assessment, we tested the ability of various models to classify individuals according to SA history based on DNA methylation and clinical measures both independently and jointly in the discovery and replication cohorts.

## Method

### Discovery cohort

This sample has been previously described [22]. One hundred and sixty-one adults with BD (79 BD/non-SA, 84 BD/SA) and 76 non-psychiatric controls without a lifetime history of SA (CON) were recruited at the Center of Excellence in Mood Disorders, Houston, TX. BD diagnosis was ascertained in the Structured Clinical Interview for DSM-IV Axis I Disorders (SCID-I) [23]. Lifetime history of SA was assessed with Columbia Suicide History Form (CSHF) [24]. Other demographic and clinical characteristics (e.g., substance use, previous hospitalisations, psychiatric comorbidities) were obtained by demographic questionnaire and clinical interview. Interviews were administered by trained evaluators and reviewed by a board-certified psychiatrist. Young Mania Rating Scale (YMRS) [25] and Montgomery-Asberg Depression Rating Scale (MADRS) [26] were administered for assessing manic and depressive symptomatology. Exclusion criteria for all participants included neurological disorders and traumatic brain injury, schizophrenia, developmental disorders, eating disorders, intellectual disability, and recent illicit drug use by urine drug screen. Exclusion criteria for CON included a history of any Axis I disorder in first-degree relatives or if they had taken a prescribed psychotropic medication at any point in their lives. The study protocol was approved by the local institutional review board (IRB), and informed consent was obtained from all participants at enrolment and prior to any procedure.

### Replication cohort

This sample has been previously described [22]. Ninety-five adults with BD (48 BD/SA, 47 BD/non-SA) were recruited through the IRB-approved Iowa Neuroscience Institute Bipolar Disorder Research Program of Excellence (BD-RPOE). There were no CON members in the replication cohort. Participants between 18 and 70 years with a confirmed SCID-I diagnosis of BD-I provided informed consent. History of SA and number of lifetime attempts were recorded with the Columbia Suicide Severity Rating Scale (C-SSRS) [24]. Demographic measures and clinical interviews, including YMRS and MADRS, were administered. Exclusion criteria included a history of loss of consciousness for more than 10 min, seizure disorder, brain damage or other neurological problems, coronary or cerebral artery disease, alcohol or drug dependence within the past 3 months, current pregnancy, or contraindication for magnetic resonance imaging.

### Methylation assay

All participants provided peripheral blood by venipuncture, which was stored in EDTA-containing vacutainers at −80 degrees Celsius. In the discovery cohort, DNA was isolated from the buffy coat using the DNeasy Blood & Tissue Mini Kit (Qiagen, Hilden, Germany). In the replication cohort, 1 mL of whole blood per sample was used with the Puregene Blood Kit with RNase A solution (Qiagen, Hilden, Germany). Elution Buffer CDB-02 (Kurabo Industries Ltd, Osaka, Japan) was used instead of DNA Hydration Solution. In both cohorts, five hundred nanograms of DNA were bisulfite-converted using the EZ DNA Methylation Kit (Zymo Research, Irvine, CA, USA). Genome-wide DNA methylation was measured using the Infinium EPICMethylation BeadChip version 1.0 (Illumina, San Diego, CA, USA), according to manufacturer’s instructions.

### Information for ancestry controls

In the discovery cohort, DNA was hybridised to the Infinium Global Screening Array v1.0 and v3.0 (Illumina, San Diego, CA, USA) to measure common genetic variation. Prior to principal components analysis, pruning was performed in PLINK v1.9 [27] with a window size of 200 variants, step size of 50 variants, and LD r^2^ threshold of 0.25. The first three principal components of genotyping data were extracted for each participant and retained as covariates for genomic ancestry. In the replication cohort, EPISTRUCTURE, which uses information from patterns in DNA methylation rather than genotyping [28], was used to compute the first three principal components used for ancestral adjustment. EPISTRUCTURE has been validated as an alternative to using genotyping information to capture ancestry [28].

### Data pre-processing, quality controls, and filtering

All pre-processing and analysis steps were performed in R version 4.2.0 with the package *minfi* [29]. Cross-reactive probes [30], probes with mean detection *p*-value > 0.01, probes with fewer than 3 beads in >1% of samples, polymorphic probes [30], and probes located on the sex chromosomes were removed. Quality controls were conducted separately for each EWAS carried out. There were four EWAS performed: discovery cohort BD/SA vs. BD/non-SA (719,231 CpG sites), discovery cohort BD/SA vs. CON (725,943 CpG sites), discovery cohort BD/non-SA vs. CON (725,843 CpG sites), and replication cohort BD/SA vs. BD/non-SA (654,448 CpG sites). In the discovery cohort BD/SA vs. BD/non-SA EWAS, one member of the BD/SA group failed quality controls, reducing the size of the BD/SA group in that analysis to *n* = 83.

### Statistical analysis

Methylation values were log-transformed (base 2) from beta-values to M-values [31]. Control for batch effects and technical variability was achieved by the functional normalisation method, which applied noob background correction, dye normalisation, and correction for the first 2 principal components of the internal control probes on the EPIC array to the *M*-values [32]. To address cell type heterogeneity, white blood cell count proportions (CD8 + T cells, CD4 + T cells, natural killer cells, B cells, monocytes, and granulocytes) were estimated [33]. DNA methylation-based smoking scores were estimated based on a validated set of 174 sites in the R package *EpiSmokEr* [34, 35].

### EWAS for identification of DMPs

In both cohorts, epigenome-wide DMPs were measured with linear models adjusted for age, sex, the six white blood cell count proportions, DNA methylation smoking scores, and the first three ancestral principal components, in the R package *limma* [36]. Results for each EWAS were adjusted for false discovery rate (FDR) at significance threshold of *q* < 0.05 [37]. FDR-significant DMPs were annotated to the UCSC Genome Browser hg19 reference genome using the *CruzDB* Python implementation [38]. A sensitivity analysis of the BD/SA vs. BD/non-SA EWAS included further statistical adjustment for YMRS and MADRS total scores. The log2 fold changes were correlated across the discovery cohort EWAS (pairwise) for the FDR-significant DMPs, as well as the DMPs with nominal *p* < 0.001, from the BD/SA vs. BD/non-SA EWAS. This analysis primarily intended to better inform whether the underlying alterations differentiating BD/SA from BD/non-SA also differentiated BD/non-SA from CON.

### Blood-brain correlation of DMPs

The IMAGE-CpG tool, which provides within-person cross-tissue correlations for CpG sites measured on the EPIC BeadChip [17], was consulted to provide insight to the potential similarity of methylation levels at the discovery cohort FDR-significant DMPs from the BD/SA vs. BD/non-SA EWAS between blood (measured in this study) and brain. The *rho* correlation coefficient for within-subject blood and brain methylation concordance was extracted for each FDR-significant DMP. The magnitudes of the coefficients were also averaged across the six DMPs to provide a broader sense of the generalisability of the blood-based results to brain methylation.

### Identification of DMRs

In all discovery and replication cohort EWAS, DMRs were identified using the *comb-p* algorithm in Python 2.7, a method based on autocorrelations between all probe *p*-values from the DMP analysis and subsequent combination as broader DMRs [39]. The following parameters were specified: seed-*p*-value 1 × 10^−4^, minimum of 2 probes, and sliding window 500 base pairs. DMRs which passed a Šidák corrected *p*-value < 0.05 were considered significant.

### Genomic location, trait, and pathway enrichment analyses of DMPs

The EWAS Toolkit [40] was used to test the DMPs from each EWAS with nominal *p* < 0.001 for enrichment for genomic locations (e.g., intergenic, gene body, etc), traits, and gene ontology (GO) pathways. The background used were probes on the EPIC BeadChip.

### Weighted gene co-methylation network analysis (WGCNA)

WGCNA was only performed for the BD/SA vs. BD/non-SA EWAS in the discovery cohort. Functionally normalised CpG probes were filtered based on a nominal *p*-value of <0.05 and location at transcriptional start site (TSS) (*n* = 19,403 probes). Restriction to TSS was implemented to concentrate signal at the potentially most regulatorily impactful CpG sites [41], as done in prior studies [42, 43]. This strategy allowed for identification of possible high-impact regulatory networks among the differentially methylated CpG sites. The WGCNA R package was used to create co-methylated modules [44]. Soft power threshold of 9 was selected according to the criterion of approximate scale-free topology (R_signed_^2^ > 0.90). Eigengenes for each module were tested for association with SA in BD by logit generalised linear models including age, sex, white blood cell count proportions, smoking scores, and the first three ancestral genomic principal components as covariates, and *p*-values were adjusted for FDR by Benjamini-Hochberg procedure. GO analysis with the genes annotated to all CpG sites comprising each module was performed using the EWAS Toolkit. The background used were probes on the EPIC BeadChip. The EWAS Toolkit has a maximum of 5000 probes, so the first 5000 were taken for the turquoise module. Probes related to hub genes were identified by module membership > 0.85 and gene significance > 0.30, with annotation of genes accomplished using *CruzDB* Python implementation.

### Statistical comparison across discovery and replication cohorts

At the FDR-significant DMPs from the BD/SA vs. BD/non-SA EWAS in the discovery cohort, agreement in fold change direction was checked in the replication cohort. The log2 fold changes of the DMPs at *p* < 0.001 from the BD/SA *vs*. BD/non-SA EWAS in the discovery cohort were correlated across the discovery and replication cohorts.

### Development of a clinical-epigenetic classifier of SA history

DMP methylation beta-values and six relevant clinical correlates (age, sex, number of previous hospitalisations for depression, history of psychosis, cocaine dependence, and PTSD) were tested to classify individuals with BD according to SA history. The selected clinical correlates were the most important factors associated with SA in a previous machine learning analysis among individuals with mood disorders [2]. In individuals with BD from both cohorts, generalised linear models were fitted to classify based on SA history from (i) methylation (beta-values) from the six discovery cohort BD/SA vs. BD/non-SA EWAS FDR-significant DMPs alone, (ii) clinical correlates alone, and (iii) methylation at DMPs and clinical correlates together. The generalised linear models were of logistic form, with SA history as the outcome and individual DMPs and clinical correlates included as independent, additive predictors. For example, the model to test classification of SA history from beta-values and clinical correlates together was of the form:

Suicide Attempt ~ Age + Sex + cg20244265 + cg25876840 + cg11476866 + cg15653194 + cg00306112 + cg20242392 + Psychosis History (binary) + Cocaine Dependence (binary) + PTSD (binary) + Number Previous Hospitalisations for Depression.

Area under the curve (AUC) of the receiver operating characteristic (ROC) curve was used to evaluate the predictive performance of the models in R package pROC [45]. Additionally, pairwise differences in AUC among ROC curves were statistically tested using the DeLong test for two correlated ROC curves within cohorts.

### Investigation of correlations between methylation at discovery cohort DMPs associated with SA history and number of SA

Within the discovery cohort BD/SA group (*n* = 84), associations between methylation (beta-values) at the six discovery cohort FDR-significant DMPs from the BD/SA vs. BD/non-SA EWAS and number of lifetime SA were investigated with Pearson’s correlation analysis. This analysis sought to provide further insight to whether markers of SA history in individuals with BD would also be related to other features of SA such as this proxy for cumulative burden, among individuals with BD and SA history.

## Results

### Demographic and clinical characteristics of the discovery and replication cohorts

Detailed information about the discovery and replication cohorts can be found in Table 1.

**Table 1:** Demographics.

	Discovery cohort (*N* = 239)				Replication cohort (*N* = 95)		
Group	BD/non-SA (*n* = 79)	BD/SA (*n* = 84)	CON (*n* = 76)	*p*_BD/SA vs. BD/non-SA_	BD/non-SA (*n* = 47)	BD/SA (*n* = 48)	*p*_BD/SA vs. BD/non-SA_
Age, mean (SD)	36.7 (11.5)	35.3 (10.9)	33.8 (10.5)	0.48	39.6 (13.8)	44.3 (12.9)	0.08
Sex, *n* female (%)	59 (75%)	59 (70%)	49 (64%)	0.60	20 (43%)	14 (29%)	0.20
Race/Ethnicity				0.39			0.22
Non-Hispanic White, *n* (%)	31 (39%)	39 (46%)	17 (22%)	–	39 (83%)	35 (73%)	–
Black African-American, *n* (%)	24 (30%)	24 (29%)	28 (37%)	–	0 (0%)	4 (8%)	–
Hispanic or Latino, *n* (%)	12 (15%)	15 (18%)	19 (25%)	–	4 (9%)	6 (13%)	–
Other race, *n* (%)	12 (15%)	6 (7%)	11 (14%)	–	4 (9%)	3 (6%)	–
Years of education, mean (SD)	14.3 (2.7)	13. 9 (2.5)	15.8 (2.4)	0.10	15.3 (2.1)	14.8 (2.3)	0.26
Self-reported smoking history, *n* (%)	26 (33%)	30 (36%)	2 (3%)	0.74	5 (11%)	14 (29%)	**0.04**
Psychiatric medication, *n* (%)	70 (89%)	71 (85%)	0 (0%)	0.64	42 (89%)	44 (92%)	0.74
Lithium, *n* (%)	18 (23%)	15 (18%)	0 (0%)	0.41	20 (43%)	6 (13%)	**0.0012**
Anticonvulsant, *n* (%)	27 (34%)	31 (37%)	0 (0%)	0.30	19 (40%)	26 (54%)	0.22
Antidepressant, *n* (%)	31 (39%)	28 (33%)	0 (0%)	1.00	26 (55%)	33 (69%)	0.21
Antipsychotic, *n* (%)	35 (44%)	42 (50%)	0 (0%)	0.12	24 (51%)	26 (54%)	0.84
Sedative, *n* (%)	17 (22%)	19 (23%)	0 (0%)	0.56	14 (30%)	25 (52%)	**0.037**
Stimulant, *n* (%)	1 (1%)	4 (5%)	0 (0%)	0.20	4 (9%)	6 (13%)	0.74
Number of SA, mean (SD)	–	1.79 (1.50)	–	–	–	3.98 (5.21)	–
MADRS, mean (SD)	11.0 (9.5)	16.0 (10.3)	0.16 (0.49)	**0.0016**	11.9 (8.7)	16.2 (9.0)	**0.0017**
YMRS, mean (SD)	5.2 (6.4)	7.1 (8.2)	0.20 (0.49)	0.072	5.4 (5.5)	8.2 (8.3)	0.070

Demographic and clinical characteristics of the discovery and replication cohorts.

*p*-values for quantitative variables were calculated using either *t*-test or Mann–Whitney *U*-test, depending on the result of a Shapiro–Wilk test for normality. *p*-values for categorical variables were calculated using the Fisher test. Bolded values indicate *p* < 0.05.

*BD* bipolar disorder, *CON* controls, *MADRS* Montgomery-Asberg Depression Rating Scale, *SA* suicide attempt, *SD* standard deviation, *YMRS* Young Mania Rating Scale.

### Discovery cohort EWAS identifies six DMPs between BD/SA and BD/non-SA

**Fig. 1:**
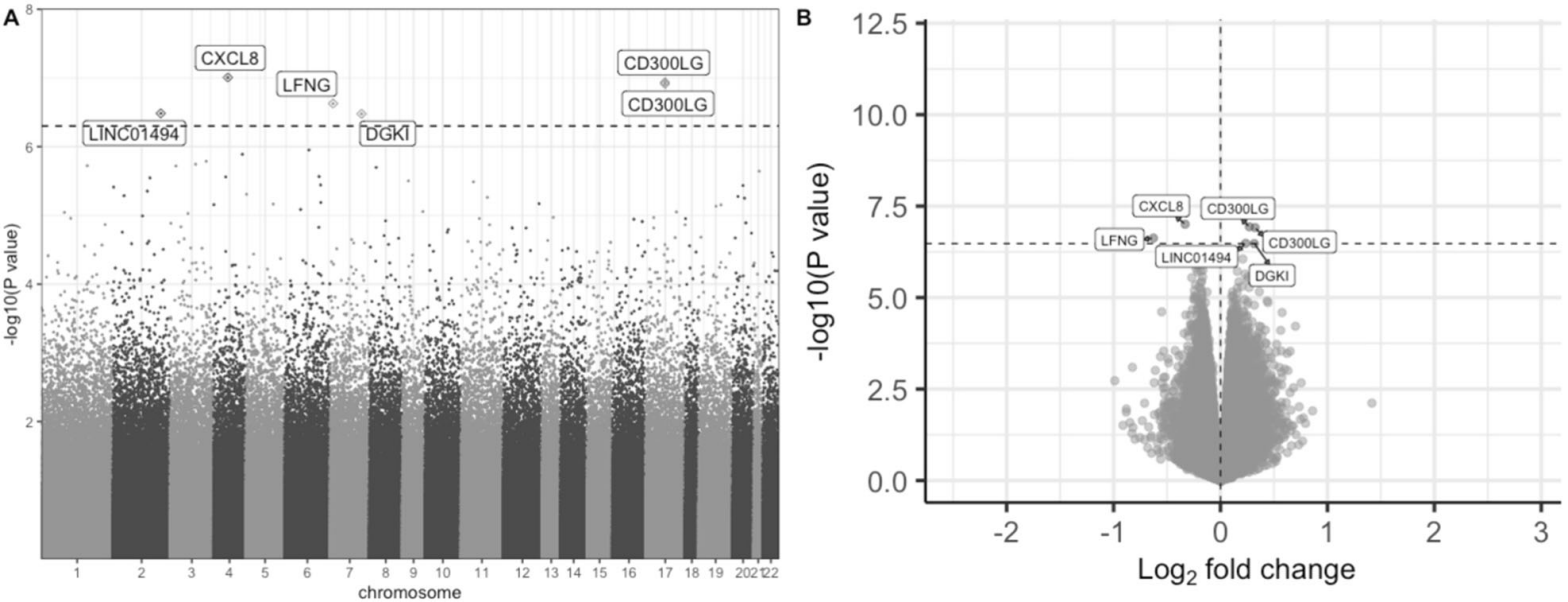
Results from the discovery cohort epigenome-wide association study of BD/SA (*n* = 79) vs. BD/non-SA (*n* = 83). **A** Manhattan plot of significance by chromosomal location. **B** Volcano plot of significance against log2 fold change, where positive and negative log2 fold changes indicate higher methylation and lower methylation in the BD/SA group, respectively. In both plots, the horizontal dotted line demarcates the FDR adjusted *q* < 0.05 threshold, and FDR-significant CpG sites are labelled based on their annotated genes. BD bipolar disorder, FDR false discovery rate, SA suicide attempt.

The discovery cohort EWAS identified six DMPs between BD/SA and BD/non-SA after FDR correction (*q* < 0.05) (Fig. 1 and Table 2). The leading site was annotated to the *CXCL8* gene, which encodes the interleukin-8 cytokine. Two sites showed lower methylation in BD/SA: cg20244265 (*CXCL8*) and cg15653194 (*LFNG*). Four sites showed higher methylation in BD/SA: cg11476866 (*CD300LG*), cg20242392 (*DGKI*), cg25876840 (*CD300LG*), and cg00306112 (*LINC01494*) (Table 2). For all CpG site results at nominal *p* < 0.001, see Supplementary Table S1. After sensitivity adjustment for YMRS and MADRS scores, fold changes at these six sites remained consistent and nominal *p*-values were all < 1 × 10^−5^, though none of the six sites survived the FDR correction (Supplementary Table S2).

**Table 2:** Differentially Methylated Positions.

chr	Probe ID	Log2 FC	*p*	FDR *q*	Gene annotation	Gene distance (bp)	Gene feature
4	cg20244265	−0.33	9.86 × 10^−8^	0.03	*CXCL8*	−22861	Intergenic
17	cg25876840	0.27	1.16 × 10^−7^	0.03	*CD300LG*	−4066	Intergenic
17	cg11476866	0.32	1.22 × 10^−7^	0.03	*CD300LG*	−4128	Intergenic
7	cg15653194	−0.63	2.36 × 10^−7^	0.04	*LFNG*	0;0	Exon+5′UTR; Intron
2	cg00306112	0.23	3.28 × 10^−7^	0.04	*LINC01494*	0	nc_intron
7	cg20242392	0.32	3.33 × 10^−7^	0.04	*DGKI*	0;0	Intron; Intron+5′UTR

Six CpG sites differentially methylated between BD/non-SA and BD/SA at FDR *q* < 0.05 in the discovery cohort (*n* = 162).

*5*′*UTR* 5′ untranslated region, *BD* bipolar disorder, *chr* chromosome, *FC* fold change, *FDR* false discovery rate, *SA* suicide attempt.

The EWAS between BD/SA and CON found no DMPs at FDR correction *q* < 0.05 (full results at nominal *p* < 0.001 in Supplementary Table S3). In addition, the EWAS between BD/non-SA and CON also found no DMPs at FDR correction q < 0.05 (full results at nominal *p* < 0.001 in Supplementary Table S4, Supplementary Fig. S1). Genomic inflation factor lambda was 1.20 for BD/SA vs. BD/non-SA, 0.90 for BD/SA vs. CON, and 1.21 for BD/non-SA vs. CON, suggesting no major issues of inflation (Supplementary Fig. S2).

At the six CpG sites which passed *q* < 0.05 in the BD/SA *vs*. BD/non-SA analysis, fold change direction was generally inconsistent among the three contrasts (BD/SA *vs*. CON, BD/SA *vs*. BD/non-SA, BD/non-SA *vs*. CON), suggesting noncontinuous changes between severity groups (Supplementary Table S5, Supplementary Fig. S3). For the DMPs with nominal *p* < 0.001 in the BD/SA vs. BD/non-SA analysis, the correlations among log2 fold changes between the BD/SA vs. BD/non-SA and BD/SA vs. CON contrasts were *r* = 0.76 (*p* < 2.2 × 10^−16^, df = 2050), with proportion agreeing in fold change direction being 85% (95% CI = 84–87%). The correlation of the fold changes between the BD/SA vs. BD/non-SA and BD/non-SA vs. CON contrasts had an *r* = −0.84 (*p* < 2.2 × 10^−16^, df = 2056), with proportion agreeing in fold change direction being 5% (95% CI = 4–6%). Finally, the correlation of fold changes between the BD/SA vs. CON and BD/non-SA vs. CON contrasts had an *r* = −0.36 (*p* < 2.2 × 10^−16^, df = 2045), with proportion agreeing in fold change direction being 20% (95% CI = 18–22%) (Supplementary Table S6, Supplementary Fig. S4). Twenty CpG probes passed nominal *p* < 0.001 in both the BD/SA vs. BD/non-SA and BD/SA vs. CON analyses, as did 60 CpG probes in both the BD/SA vs. BD/non-SA and BD/non-SA vs. CON analyses, and 147 in the BD/SA vs. CON and BD/non-SA vs. CON analyses (listed in Supplementary Table S7).

### Blood-brain correlation of discovery cohort DMPs

Magnitudes of the blood-brain correlations for the six FDR-significant DMPs in the BD/SA vs. BD/non-SA contrast ranged from rho_abs_ = 0.01 (*CXCL8*) to rho_abs_ = 0.49 (*LFNG*), with a mean average of rho_abs_ = 0.29 and five of the six DMPs of magnitude greater than rho_abs_ = 0.20 (Supplementary Table S8).

### DMRs in the discovery cohort

In the discovery cohort, seven DMRs were identified between BD/SA and BD/non-SA after Šidák correction (Šidák *p* < 0.05) (Supplementary Fig. S5, Table 3). The DMRs were annotated to the genes *TRIM40, RNF14, C14orf93, ETFBKMT, CD300LG, LOC728392*, and *HIVEP3*. There were no Šidák significant DMRs in the discovery cohort analyses of DMPs from BD/SA vs. CON and BD/non-SA vs. CON.

**Table 3:** Differentially Methylated Regions.

chr	Start	End	*n* probes	Šidák *p*	Gene annotation	Gene distance	Gene feature
6	30095136	30095495	17	3.91 × 10^−8^	*TRIM40*	−8421	Intergenic
5	141348202	141348279	5	1.01 × 10^−6^	*RNF14*	0	Intron+5′UTR
14	23479657	23479801	5	2.06 × 10^−5^	*C14orf93*	−305	Intergenic
12	31799111	31799118	3	2.94 × 10^−5^	*ETFBKMT*	−975	Intergenic
17	41920415	41920519	3	3.20 × 10^−5^	*CD300LG*	−4024	Intergenic
17	5403805	5403907	3	4.09 × 10^−5^	*LOC728392*	0	Exon+3′UTR
1	42384390	42384491	4	1.55 × 10^−3^	*HIVEP3*	0;0;0	Exon+5′UTR; nc_intron; nc_exon

Seven regions differentially methylated between BD/non-SA and BD/SA at Šidák’s *p* < 0.05.

*UTR* untranslated region, *BD* bipolar disorder, *chr* chromosome, *FC* fold change, *FDR* false discovery rate, *SA* suicide attempt.

### Genomic location, trait and pathway enrichment analyses of DMPs in the discovery cohort

**Fig. 2:**
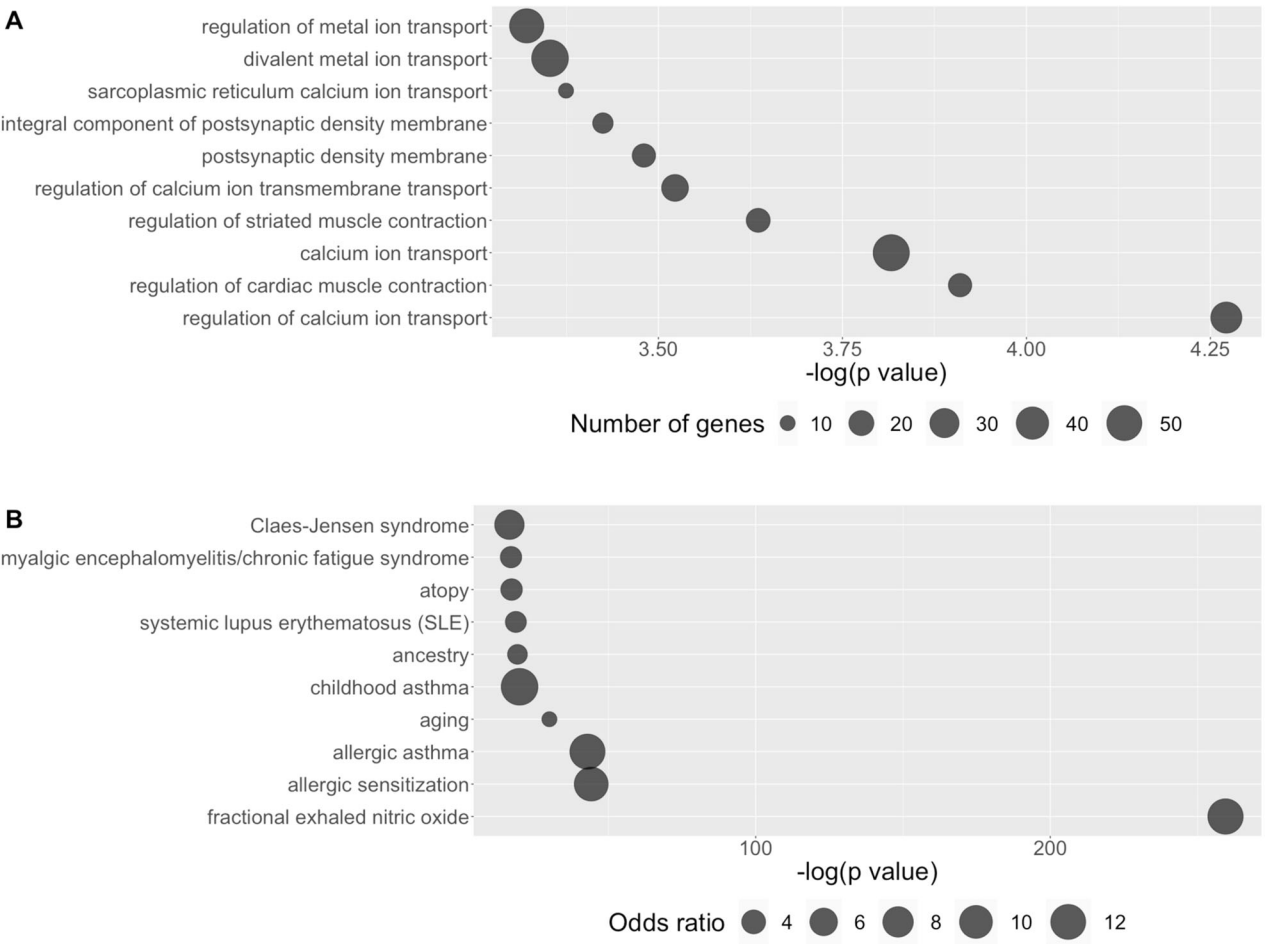
Gene ontology and trait enrichment results for the discovery cohort BD/SA vs. BD/non-SA EWAS using the DMPs at nominal *p* < 0.001. **A** Top ten gene ontology pathways ordered by -log (*p*-value), with the number of genes assigned to each pathway represented by the size of the point. **B** Top ten traits ordered by -log (*p*-value), with the odds ratio for each trait represented by the size of the point. BD bipolar disorder, *DMP* differentially methylated position, *EWAS* epigenome-wide association study, *SA* suicide attempt.

In the discovery cohort BD/SA vs. BD/non-SA EWAS, the DMPs were primarily concentrated in OpenSea (OR = 1.53) and intergenic (OR = 1.32) genomic locations. Top traits were especially enriched for inflammation-related traits. There was also trait enrichment for psychiatric and neurodevelopmental traits, as well as indicators of early life stress. Calcium signalling was implicated in the GO results. The results for the discovery cohort BD/SA vs. BD/non-SA EWAS can be viewed in Fig. 2 and Supplementary Tables S9–S11.

Enrichment for genomic locations, traits, and GO processes for the BD/SA vs. CON and BD/non-SA vs. CON EWAS can be seen in (Supplementary Figs. S6, S7, and Supplementary Tables S9–S11). There were no overlapping significant GO processes between the three contrasts.

### WGCNA in the discovery cohort

The WGCNA analysis returned six modules (blue, turquoise, brown, pink, black, green), with a mean of 2947 probes per module and a range from 188 probes (pink) to 7237 probes (turquoise) (Supplementary Fig. S8). Unassigned CpG sites (*n* = 1718) were clustered in a “grey” module, which was not considered for further analyses. All six modules were significantly associated with SA history in individuals with BD, led by the brown module (all *q* < 0.05) (Supplementary Table S12).

The assignment of probes to modules, including module membership and gene significance, can be found in Supplementary Table S13. The same information after a filter for hub genes is presented in Supplementary Table S14. Significant processes from the GO enrichment analyses performed with CpG probes in each module are shown in Supplementary Table S15. Of note, the top-ranked module, module brown, was substantially enriched for immune-related processes, including immune system process, immune response, and immune effector process (Supplementary Table S15). Relevant hub genes for this module included *RUSC1-AS1, BTG3-AS1, IQCE, SLC26A8, MIR6840, STAG3L5P-PVRIG2P-PILRB*, and *SSBP3* (for other module hub genes see Supplementary Table S14).

### Replication cohort validation of discovery cohort DMPs, DMRs, and enrichment analyses

In the replication cohort DMP analysis, no epigenome-wide significant CpG sites were identified at the FDR adjustment threshold of *q* < 0.05 (full results at nominal *p* < 0.001 in Supplementary Table S16). Replication cohort log2 fold changes and *p*-values for the discovery cohort epigenome-wide significant CpG sites are reported in Supplementary Table S17, with fold change direction being consistent at four of the sites. None of the sites replicated at a nominal *p* < 0.05. Additionally, one of the sites (cg20242392, *DGKI*) failed quality control in the replication cohort so was not included in the DMP analysis. The replication cohort DMR analysis revealed no Šidák significant DMRs.

For the five discovery cohort FDR-significant CpG sites which survived filtering in the replication cohort, the correlation of log2 fold changes across cohorts was *r* = 0.60 (*p* = 0.28, df = 3). For the DMPs with *p* < 0.001 in the discovery cohort, the correlation of log2 fold changes across cohorts was *r* = 0.10 (*p* = 2.0 × 10^−5^, *df* = 1956) (Supplementary Table S18, Supplementary Fig. S9). Additionally, the proportion of DMPs *p* < 0.001 which agreed in fold change direction across cohorts was 51% (95% CI = 49–53%).

Information on genomic locations, trait enrichment, and GO processes in the replication cohort BD/SA vs. BD/non-SA EWAS is available in (Supplementary Tables S19–S21). Some immune system-related traits and pathways arose in enrichment and GO analyses (Supplementary Tables S20, S21). Several replication cohort significant traits overlapped with those from the discovery cohort BD/SA vs. BD/non-SA analysis, including inflammation-related diseases (e.g., perinatally-acquired HIV, Crohn’s disease) and indications of stress (e.g., preterm birth, maternal smoking). However, there was no overlap among GO terms across the discovery cohort and replication cohort contrasts.

### Performance of the clinical-epigenetic classifier of SA history among individuals with BD in the discovery and replication cohorts

**Fig. 3:**
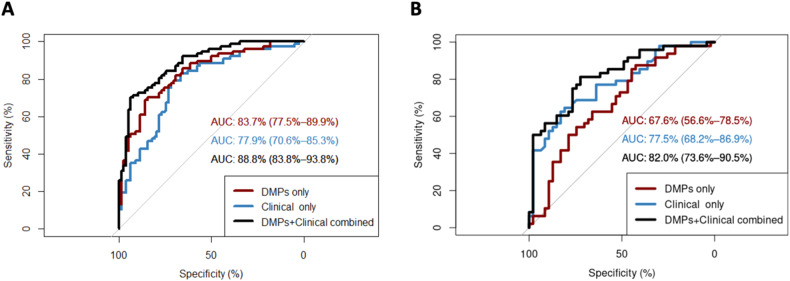
Receiver operating characteristic (ROC) curves for generalised linear models classifying SA history among individuals with BD, based on beta-values of the six FDR-significant discovery cohort DMPs (“DMPs only”), six clinical correlates of SA history in mood disorder (“Clinical only”), and the beta-values and clinical correlates combined (“DMPs + Clinical combined”). AUC of the ROC curves are presented with 95% confidence intervals. **A** ROC curves are presented for classification of SA history in the discovery cohort (n = 162). **B** ROC curves are presented for classification of SA history in the replication cohort (*n* = 95). AUC area under the curve, DMP differentially methylated positions, FDR false discovery rate, SA suicide attempt.

Performance in both cohorts is presented in Fig. 3. In the discovery cohort, the AUC was 83.7% (95% CI = 77.5–89.9%) for classification based on DMP methylation alone, 77.9% (95% CI = 70.6–85.3%) for classification based on clinical measures alone, and 88.8% (95% CI = 83.8–93.8%) for classification on combined methylation and clinical measures. In the replication cohort, the AUC was 67.6% (95% CI = 56.6–78.5%) for classification based on DMP methylation alone, 77.6% (95% CI = 68.2–86.9%) for classification based on clinical measures alone, and 82.1% (95% CI = 73.6–90.5%) for classification on combined methylation and clinical measures. DeLong’s test revealed that in both cohorts, classification based on DMP methylation significantly improved when clinical measures were included; though, adding DMP methylation to clinical measures only significantly improved classification in the discovery cohort (Supplementary Table S22).

### Correlational analysis of DMPs and number of SA in the discovery cohort

Methylation at any of the six DMPs was not significantly correlated with the number of SA among members of the BD/SA group in the discovery cohort (Supplementary Table S23, Supplementary Fig. S10), with the largest correlation being a negative association between methylation at cg25876840 (*CD300LG*) and the number of SA (*r* = −0.15, *p* = 0.22).

## Discussion

Considering the heightened risk for SA in BD, we conducted a series of EWAS of lifetime history of SA and BD to better understand the underlying biological patterns. Further, we leveraged an independent replication cohort of individuals with BD to attempt to validate our findings and test a peripheral methylation biomarker of SA history within BD. In our discovery analysis, we identified six CpG sites and seven genomic regions which were significantly differentially methylated between individuals with BD with and without history of SA at stringent multiple testing thresholds. Importantly, the average blood-brain correlation for methylation at the CpG sites was low to moderate (average rho_abs_ = 0.29), which though higher than the previously identified overall average blood-brain correlation of 0.15 across CpG sites within individuals [17], still suggests that the identified site-specific patterns in DNA methylation may not directly extrapolate to brain.

The top DMP was annotated to *CXCL8*, which encodes for the interleukin-8 (IL-8) cytokine and is a well-recognised participant in the systemic immune response [46]. Further, gene *CD300LG*, which was mapped to two of the top DMPs and also captured by the DMR analysis, is also involved in immune system function, via modulation of cytokine-induced killer cells [47]. *LFNG*, annotated to one of the DMPs, is part of the Notch pathway, which has been implicated in neuro-inflammation and BD [48]. From the DMR analysis, *TRIM40* has recently been associated with inflammation in the context of inflammatory bowel disease [49], *RNF14* regulates mitochondrial and immune-related functioning [50], and *HIVEP3* regulates the development of innate-like T cells [51]. Further, a number of significantly associated traits from enrichment analysis were also associated with immune dysfunction. In addition, the top-ranked module from the WGCNA analysis was enriched for immune responses, suggesting a role for regulatory network organisation of genes related to immune system functioning.

Taken together, these results suggest that alterations in immune-related genes in individuals with BD and SA history could provide an epigenetic mechanism for previously observed peripheral immune activation in SA [52–54]. This includes specific findings related to our identified genes, such as prior evidence for IL-8 dysregulation in suicide attempters [55, 56]. The patterns of immune dysregulation in adults with BD and a history of SA may be attributable to increased exposure to environmental stressors [57]. Whether these alterations are simply correlates of a more stressful life or causal contributors to suicide risk remains to be seen, though it is possible that immune system changes lead to mood changes [58]. There is also the possibility that genetic influences are involved. Of note, the implications of the direction of methylation at these genes remain to be explored, ideally by concerted measurement of both DNA methylation and gene expression in follow-up work. At the functional pathway level, calcium signalling emerged as another possible pathway related to SA history among individuals with BD. The candidate gene *CACNA1C*, a calcium channel subunit, has previously been associated with BD and SA in both genetic and epigenetic analyses [59–62]. There were a number of significant GO processes related to neural functioning as well, though the direct effects are unclear as this study was conducted in peripheral blood.

Within the discovery cohort, we found evidence that the epigenetic pathophysiology differentiating BD/SA from BD/non-SA does not also differentiate BD/non-SA from CON, as supported by the strong negative correlation of log2 fold changes and minimal percentage agreement in fold change direction at the nominally significant DMPs. This suggests that there is a specific epigenetic basis to SA in BD above and beyond the contributions of BD pathology in the absence of SA. In other words, the patterns of differential methylation which characterise SA in BD are not a recapitulation of patterns differentiating BD (without SA) from CON, which provides empirical support for the notion that the epigenetic signature of SA history can be separated from the contribution of psychiatric diagnosis. Further, differences at the FDR-significant DMPs appeared robust to adjustment for manic and depressive symptomatology differences between BD/SA and BD/non-SA, also suggesting greater correspondence to SA than to BD severity. Of note, methylation at the DMPs did not relate to the number of SAs within the BD/SA group, which could suggest that DNA methylation differences mark differences in binary-type “thresholds” for SA rather than dose-response relationships with SA burden. Our study was not designed to provide such granular insight and follow-up is needed. Lack of FDR-significant results in the other contrasts (BD/SA vs. CON, BD/non-SA vs. CON) could be partially due to the greater number of probes surviving quality controls, leading to a more stringent multiple comparisons correction.

In the independent replication cohort, we observed general consistency in the direction of fold change at the FDR-significant sites. Though none of the discovery cohort FDR-significant sites replicated at nominal *p* < 0.05 in the replication cohort, this could be due to a lack of statistical power in the replication cohort. Lack of statistical power could also be responsible for weaker correlation of fold changes across the discovery cohort nominally significant sites and limited convergence across GO processes, though there was some overlap in trait enrichment. An alternative effort to leverage the replication cohort was by inspecting the AUC of the ROC classification curves based on DMP methylation and clinical interviews. In both cohorts, methylation at the FDR-significant DMPs alone performed better than chance at classifying individuals with BD according to SA history. The finding that individual probe results were non-significant in the replication cohort EWAS, though explanatory power was still achieved when methylation at the discovery cohort DMPs was aggregated in the ROC approach, could suggest that combining signal across multiple methylation markers, akin to the theory behind polygenic risk scores, holds the greatest promise for clinical use. Combining information on DNA methylation with information from clinical interviews performed best in both cohorts, though adding DMP methylation to clinical measures only significantly improved classification in the discovery cohort. Overall, the evidence provides a precedent for integrating patterns in DNA methylation to existing risk prediction models in order to improve their performance. The multiple-levels-of-analysis approach to understanding SA risk in BD promises improvements to prevention and possible intervention [21].

When considering past EWAS of suicidal behaviours, there are a few in particular that are important to compare the present results to. The only prior study which we are aware of which also focused on SA specifically in the context of BD was an EWAS comparing white blood cell DNA (as in our discovery cohort) from individuals with BD and high *vs*. low suicidality (a combination of SI and SA) [14]. Within their epigenome-wide and nominally significant results, we do not find our DMPs or DMRs. However, that study focused on only 350,000 CpG sites, in comparison to the >700,000 CpG sites included in our analysis. It may be of interest to also consider EWAS of SA in other psychiatric disorders, to investigate overlap and divergences. In the most significant results of a study based in white blood cell DNA from individuals with schizophrenia (SCZ) and SA and without SA [13], limited to <400,000 CpG sites, we do not find overlap with our DMPs and DMRs. To our knowledge, there are no studies of SA in other focused psychiatric disorders. In the largest EWAS to date of SA in whole blood, which was not focused within a specific psychiatric diagnosis (*N* = 2712 U.S. veterans), three FDR-significant DMPs were identified; they were annotated to genes *SLC4A2/CDK5, PDE3A*, and *RARRES3* [15]. None of those DMPs are present in our results of DMPs at nominal *P* < 0.001 from either the BD/SA vs. BD/non-SA or the BD/SA vs. CON EWAS. However, this limited convergence may be expected when comparing results from a more epidemiologically-informed veteran sample to our psychiatric sample.

Considering relevance to SD is also possible. In a similarly structured study to ours, DNA methylation in brain tissue was compared across individuals with BD and death by suicide (BD/SD), BD and death by a cause other than suicide (BD/non-SD), and controls (CON) [63]. With a specific focus on comparing results from their BD/SD vs. BD/non-SD contrast to our BD/SA vs. BD/non-SA results, we do not find our FDR-significant DMPs or Sidak-significant DMRs in their nominally significant (*P* < 0.05) results. When considering gene-level replication patterns, two CpG probes annotated to *TRIM40*, a gene annotated to a DMR we identified, show decreased methylation in BD/SD. Further, at the pathway level, calcium signalling, which we identified as a pathway enriched among DMPs between BD/SA and BD/non-SA, was the top pathway differentiating BD/SD from CON. Comparing these studies is challenging due to several sources of inter-study heterogeneity, the largest contributors to variation being the possible differing biology underlying SA and SD, as well as the inter-tissue heterogeneity in DNA methylation patterns across brain and blood. Though, they could suggest a common role for *TRIM40* and calcium signalling in BD and the broader bin of suicidal behaviours.

Insight to temporality is the major limitation of this study. As SA was ascertained retrospectively as a lifetime history, it is unclear how relevant this epigenetic signal may be for future SA risk. We did not have information on time elapsed since the most recent SA, which could have helped with this problem in sensitivity analyses. As some risk associated with suicidal behaviour may be temporally enduring [12], there is the possibility that the EWAS are capturing some trait-like risk, which may have been present even before the onset of SA; though, with these data there is no way to tell. Another drawback of this retrospective approach is the possibility that alongside the psychological consequences of SA [64], there may be concomitant biological sequelae, or “after-effects” of surviving a SA. If this were true, then DMPs differentiating BD/SA vs. BD/non-SA would be less related to antecedent risk than to the aftermath of SA, which would not be helpful for initiatives aiming to prevent SA. That information could still be helpful in linking SA history to potential correlates of physical health, considering the heightened illness and mortality burden among individuals with a history of SA [65]. The gold standard approach to “solving” the temporality problem would be to conduct prospective longitudinal studies of high-risk individuals for SA, with the aim of relating baseline methylation patterns to onset of SA. This is no simple feat due to a variety of practical and ethical challenges, and to date there are no such published studies to our knowledge [9]. However, a way forward could be to leverage existing longitudinal cohorts of individuals with BD which have collected DNA methylation at some time point and examine whether changes at the sites that we identified here are also prospectively predictive of SA.

There are several other key limitations to consider in the interpretation of the study findings. High rates of psychiatric medication in both cohorts render it difficult to generalise to unmedicated individuals, given possible background influences of medication use on DNA methylation patterns. However, fortunately in the discovery cohort analysis, use of specific medications did not significantly differ between BD/SA and BD/non-SA, suggesting that confounding effects of medications in this contrast was limited. Though when comparing either of these groups to CON, medication use certainly could explain part of the epigenetic signal. In the replication cohort, BD/SA and BD/non-SA subgroups differed on rates of lithium and sedatives use; this in addition to other factors, such as broader inter-cohort differences in medication subtype use, could be an important contributor to the lack of statistically significant replication for the discovery cohort DMPs. In both cohorts, the EWAS were likely limited by statistical power constraints. Enrichment analyses and use of the replication cohort as a test sample for the AUC analyses were attempts to make the most of these limitations. One factor potentially explaining low overall replication across the two cohorts could be the difference in biological materials from which DNA was extracted, since the discovery cohort DNA was derived from buffy coat and the replication cohort DNA was derived from whole blood; controlling for white blood cell count proportions may not have fully accounted for these differences. Thus, future multi-cohort studies should try to match biological sampling protocols as closely as possible so as to minimise heterogeneity. As the EWAS were performed in peripheral blood, the immune-related findings may not extend to the brain (i.e., neuroinflammation), though this will have to be tested more directly in concerted follow-up studies. Finally, interpretation of DMPs, DMRs, and enrichment results relies on the assumption that DNA methylation alterations lead to changes in gene expression, although future studies incorporating gene expression measurements are needed to directly test this at relevant sites.

In summary, in a series of EWAS, we investigated the epigenetic underpinnings of SA history in BD and provided evidence that alterations in DNA methylation are especially concentrated at immune-relevant genes and related to systemic immune dysfunction. Though multilevel longitudinal research is needed to clarify the associated processes, we propose that these epigenetic modifications may be involved in the development of the atypical immune functioning which is implicated in suicide risk. Additionally, we demonstrate that epigenetic information could be useful in screening approaches and further that the greatest classification accuracy arises when both epigenetic and clinical sources of information are considered together.

### Supplementary information

Supplementary Figures S1-S10

Supplementary Tables S1-S23

## Data Availability

Datasets analysed in this study and bioinformatic code will be made available from the corresponding author upon reasonable request.
