# Supplementary material for: Blood epigenome-wide association studies of suicide attempt in adults with bipolar disorder
Source: Transl Psychiatry. 2024 Jan 31;14:70. doi: 10.1038/s41398-024-02760-y (PMC10831084; doi:10.1038/s41398-024-02760-y)
Supplement: Supplementary file 2 — Supplementary Tables S1-S23 [file 41398_2024_2760_MOESM2_ESM.pdf]

Supplementary Table S1. DMPs at nominal p < 0.001 in the discovery cohort BD/SA vs. BD/non-SA EWAS.

chr	Probe ID	Gene feature	Gene annotation	logFC	p	FDR q
chr4	cg20244265			-0.3302726	9.86E-08	0.0292713
chr17	cg25876840			0.2693119	1.16E-07	0.0292713
chr17	cg11476866			0.3232176	1.22E-07	0.0292713
chr7	cg15653194	Body; TSS200	LFNG;LFNG	-0.6268628	2.36E-07	0.0399152
chr2	cg00306112		AC073128.10	0.2344892	3.28E-07	0.0399152
chr7	cg20242392	Body	DGKI;DGKI;DGKI	0.3159388	3.33E-07	0.0399152
chr1	cg13065624	TSS1500	RP11-297H3.4	-0.285941	7.87E-07	0.0788101
chr2	cg05454076		WNT10A	0.2081813	8.77E-07	0.0788101
chr6	cg08649048			-0.2603638	1.12E-06	0.0895173
chr4	cg27145165	Body	GLRA3	-0.2193758	1.29E-06	0.0925912
chr3	cg05077488	Body	FNDC3B	-0.2277804	1.64E-06	0.0960933
chr3	cg18042859	Body;Body	KALRN	-0.1650394	1.80E-06	0.0960933
chr1	cg23190823	Body;TSS1500;TSS1500;Body;Body;Body;Body	RUSC1-AS1;FDPS;FDPS;RUSC1;RUSC1;FDPS;RUSC1-AS1;RUSC1-AS1;RUSC1-AS1	0.1568636	1.90E-06	0.0960933
chr3	cg23143093	Body	NEK10;NEK10;NEK10;NEK10	-0.1840548	1.91E-06	0.0960933
chr8	cg07058706		SNORA67	-0.2136145	2.00E-06	0.0960933
chr21	cg02088355		TMPRSS3	0.228502	2.27E-06	0.1021404
chr6	cg27148007	Body;Body;Body;Body;Body	EPB41L2;EPB41L2;EPB41L2;EPB41L2	-0.1671539	2.73E-06	0.1076902
chr4	cg11366178	TSS200	LNX1;LNX1-AS2;LNX1-AS2;LNX1;LNX1;LNX1;LNX1;LNX1;LNX1;LNX1	0.2554875	2.75E-06	0.1076902
chr2	cg11328284	Body	STK39	-0.2690633	2.84E-06	0.1076902
chr9	cg05186787	5'UTR;1stExon;1stExon;1stExon;5'UTR;5'UTR;Bod	PRUNE2;PRUNE2;PRUNE2;PRUNE2;PRUNE2;PRUNE2;PRUNE2	0.4757222	3.15E-06	0.1112665

		y;Body;Body;Body				
chr11	cg08026078	5'UTR	PRR5L;PRR5L;PRR5L;PRR5L;PRR5L	0.2524987	3.25E-06	0.1112665
chr6	cg00888507			-0.1760226	3.61E-06	0.1150565
chr20	cg23629264	Body;Body	PPP1R16B;PPP1R16B	0.172311	3.68E-06	0.1150565
chr2	cg07397939	TSS1500	AC113607.2;AC113607.2	-0.1858535	3.89E-06	0.1165589
chr2	cg01942558	TSS200	TNFAIP6	-0.2350465	4.44E-06	0.127814
chr5	cg15282634			0.1988397	4.95E-06	0.1347084
chr2	cg26986629			-0.201111	5.16E-06	0.1347084
chr20	cg26463200	TSS1500	PAX1;PAX1;PAX1	0.268075	5.30E-06	0.1347084
chr11	cg05999592			0.1559178	5.49E-06	0.1347084
chr20	cg13590419	TSS1500;5'UTR;5'UTR;5'UTR;5'UTR	PKIG;PKIG;PKIG;PKIG;PKIG;PKIG	-0.1834079	5.62E-06	0.1347084
chr6	cg21248089	Body;Body	RP11-356I2.1	-0.2447266	6.48E-06	0.1440781
chr12	cg00431564	Body		-0.1732368	6.74E-06	0.1440781
chr5	cg24777028	TSS1500;TSS1500;TSS1500;TSS1500;5'UTR	RNF14;RNF14;RNF14;RNF14;RNF14;RNF14;RNF14;RNF14;RNF14;RNF14;RNF14;RNF14;RNF14;RNF14;RNF14;RNF14;PCDH12;RNF14;RNF14	0.3628555	6.88E-06	0.1440781
chr4	cg23681017	Body	MFSD7;MFSD7;MFSD7;MFSD7	-0.1261616	7.01E-06	0.1440781
chr19	cg00984985	ExonBnd;ExonBnd;Body;Body	BCKDHA;CTC-435M10.3;BCKDHA	0.1112046	7.01E-06	0.1440781
chr19	cg02113493			0.1556088	7.34E-06	0.1465963
chr6	cg01673307	Body	PSMB9;PSMB9	-0.1730423	8.24E-06	0.1602644
chr9	cg13954127	Body	KCNT1;KCNT1	-0.2306245	8.78E-06	0.1629849
chr17	cg12349832	Body	C17orf28;C17orf28	0.1178188	8.94E-06	0.1629849
chr1	cg21902874	Body;Body		0.1049864	9.06E-06	0.1629849
chr3	cg02997497	5'UTR	FAM19A1;FAM19A1	-0.2458791	9.35E-06	0.1639903

chr2	cg14302909			-0.2232178	1.02E-05	0.1710409
chr13	cg17832917			-0.169277	1.07E-05	0.1710409
chr17	cg08564349			-0.1812676	1.08E-05	0.1710409
chr1	cg03124710	5'UTR	RP6-102O10.1;DAB1;DAB1;DAB1;DAB1	-0.2373383	1.10E-05	0.1710409
chr11	cg26150489	Body	AHNAK	0.224385	1.11E-05	0.1710409
chr18	cg13765206	Body		0.1481417	1.12E-05	0.1710409
chr16	cg17770458	TSS200	PSKH1;PSKH1	-0.2503428	1.14E-05	0.1710409
chr8	cg20280337			0.1288803	1.20E-05	0.1759136
chr16	cg22025478	TSS200	RP11-482M8.1;ZCCHC14;RP11-482M8.1;ZCCHC14;ZCCHC14	0.4357619	1.23E-05	0.1768008
chr20	cg04430871			-0.1815242	1.29E-05	0.1795009
chr3	cg14896157			0.2535164	1.30E-05	0.1795009
chr1	cg09856246			-0.1407546	1.36E-05	0.1795009
chr17	cg13054523			0.4438791	1.37E-05	0.1795009
chr11	cg15046793		RP11-452H21.1	0.1086033	1.37E-05	0.1795009
chr3	cg12404216	Body		0.132344	1.47E-05	0.1809684
chr6	cg14789775		RP11-73O6.4	-0.1869881	1.49E-05	0.1809684
chr12	cg20755748	TSS1500	METTL20	0.1594809	1.52E-05	0.1809684
chr17	cg03741118	Body		-0.1787635	1.52E-05	0.1809684
chr12	cg19560014	TSS1500	METTL20	0.2494661	1.53E-05	0.1809684
chr12	cg15239248			-0.2014681	1.53E-05	0.1809684
chr10	cg23504719	Body		0.3166257	1.61E-05	0.1863562
chr10	cg17293125	Body;Body;Body;Body	RP11-429G19.2;RP11-429G19.2;RP11-429G19.2;RP11-429G19.2;RP11-429G19.2;RP11-429G19.2	-0.1606111	1.76E-05	0.1979306
chr2	cg02954577		RN5S89	-0.180619	1.76E-05	0.1979306
chr20	cg23631370	Body	LAMA5	0.155625	1.81E-05	0.2002698

chr8	cg22822599	3'UTR	CHD7;CHD7	-0.2195767	1.97E-05	0.214205
chr19	cg14137282			0.146014	2.07E-05	0.215623
chr17	cg23542477	Body	AC007639.1;AC007639.1	-0.2137709	2.11E-05	0.215623
chr20	cg25349738			-0.2628617	2.13E-05	0.215623
chr8	cg10680133			0.1120015	2.15E-05	0.215623
chr3	cg18094261			0.1054403	2.19E-05	0.215623
chr7	cg00730266	TSS1500;5'UTR;TSS1500;TSS1500;5'UTR	PPP1R9A;PPP1R9A;PPP1R9A;PPP1R9A;PPP1R9A;PPP1R9A;PPP1R9A	-0.1638288	2.25E-05	0.215623
chr13	cg01477959	TSS200	TNFSF11	-0.1586417	2.32E-05	0.215623
chr17	cg23132302	Body;Body		0.1511499	2.33E-05	0.215623
chr2	cg19405799			-0.1654261	2.37E-05	0.215623
chr11	cg21991092			0.2098959	2.40E-05	0.215623
chr11	cg04935656			0.2175118	2.45E-05	0.215623
chr3	cg20041670			-0.5504991	2.45E-05	0.215623
chr15	cg22657448		RP11-315L6.1;CTD-2147F2.1	-0.1536807	2.47E-05	0.215623
chr11	cg14790883	5'UTR	ODZ4;ODZ4;ODZ4	-0.1639913	2.50E-05	0.215623
chr7	cg10289177	Body;Body;Body	ASIC3;ASIC3	0.1342907	2.51E-05	0.215623
chr7	cg25241447			0.1133674	2.53E-05	0.215623
chr2	cg00551617			0.5758892	2.56E-05	0.215623
chr2	cg25420477		AC016700.2	0.1668734	2.58E-05	0.215623
chr8	cg18536819			-0.1521377	2.64E-05	0.215623
chr17	cg16182479	3'UTR	SOST	0.0979533	2.67E-05	0.215623
chr12	cg16788865		RP11-96H19.1;RP11-474P2.4;RP11-96H19.1	-0.2009535	2.68E-05	0.215623
chr11	cg01457573	3'UTR;3'UTR;3'UTR;3'UTR	CLPB;CLPB	0.1027593	2.68E-05	0.215623
chr1	cg12729305			-0.1546547	2.71E-05	0.215623
chr10	cg03412153		CCDC3	0.1233367	2.73E-05	0.215623
chr16	cg06952529	Body;Body	WWOX	-0.1749012	2.75E-05	0.215623

chr18	cg00251102	Body;5'UTR	KCTD1	-0.1515774	2.77E-05	0.215623
chr1	cg02459569	5'UTR;5'UTR	RP11-316M1.3;CERS2;CERS2;CERS2;CERS2;CERS2;CERS2;CERS2	0.1050378	2.79E-05	0.215623
chr8	cg24188870	Body		-0.2227512	2.89E-05	0.2195247
chr3	cg23118377			-0.3248768	2.98E-05	0.2195247
chr3	cg06430695		RP11-206M11.7	-0.2825935	3.00E-05	0.2195247
chr14	cg22723842	Body;Body	SAMD4A	-0.1598849	3.02E-05	0.2195247
chr7	cg21660861	Body;5'UTR;5'UTR	BZW2;BZW2;BZW2;BZW2;BZW2;BZW2;BZW2;BZW2;BZW2;BZW2	-0.1564194	3.05E-05	0.2195247
chr17	cg06893273			0.2496519	3.06E-05	0.2195247
chr17	cg05016698	TSS200;5'UTR;5'UTR;5'UTR	EPN2-IT1;EPN2;EPN2;EPN2;EPN2;EPN2	0.1136611	3.10E-05	0.2195247
chr2	cg11518826	Body		-0.1667247	3.12E-05	0.2195247
chr11	cg10021935	5'UTR;5'UTR	B3GAT1;B3GAT1;B3GAT1	-0.1506018	3.16E-05	0.2195247
chr12	cg27112470	TSS1500	ACVRL1;ACVRL1	0.1410984	3.21E-05	0.2195247
chr14	cg14386691	TSS1500;TSS1500;TSS1500	C14orf93;C14orf93;C14orf93;C14orf93;C14orf93;C14orf93;C14orf93;C14orf93	0.2177193	3.24E-05	0.2195247
chr12	cg19756320	5'UTR	USP30	-0.1767822	3.26E-05	0.2195247
chr11	cg09755924		RP1-17K7.1;RCN1	0.1316618	3.30E-05	0.2195247
chr15	cg10973967	TSS1500;Body	RP11-387D10.3;RP11-387D10.3;RP11-387D10.2	0.1634775	3.33E-05	0.2195247
chr10	cg18759809			0.1407333	3.34E-05	0.2195247
chr4	cg06634862	5'UTR;1stExon	LNX1;LNX1-AS2;LNX1;LNX1;LNX1;LNX1;LNX1;LNX1;LNX1	0.2390319	3.34E-05	0.2195247
chr14	cg14527639	Body	NRXN3	-0.1501484	3.36E-05	0.2195247
chr18	cg14885150	5'UTR	SOCS6	0.1377656	3.41E-05	0.2197662

chr16	cg15705079		SPG7;RP11-104N10.1	0.1449652	3.42E-05	0.2197662
chr15	cg24158019	Body;Body;5'UTR	NARG2;NARG2;NARG2	-0.1648576	3.46E-05	0.2199283
chr22	cg04695090	5'UTR;5'UTR;5'UTR;5'UTR	MAFF;MAFF;MAFF;MAFF;MAFF;MAFF;MAFF	0.1034675	3.49E-05	0.2201312
chr19	cg20962215			0.0981938	3.55E-05	0.2215896
chr12	cg18428489			0.1267933	3.64E-05	0.2215896
chr5	cg12558410	Body;Body;Body	EGFLAM-AS4	-0.1788357	3.66E-05	0.2215896
chr11	cg04224661			0.1515072	3.69E-05	0.2215896
chr2	cg15351895		AC068718.1	-0.1509223	3.71E-05	0.2215896
chr1	cg02014883		RP11-12M5.4	0.0950244	3.73E-05	0.2215896
chr17	cg14343283	Body		0.0955188	3.75E-05	0.2215896
chr9	cg17243528	Body;Body;Body	TGFBR1;TGFBR1;TGFBR1;TGFBR1;TGFBR1;TGFBR1	-0.1465959	3.78E-05	0.2215896
chr12	cg23579306	TSS1500	SLC11A2;SLC11A2	0.1377526	3.79E-05	0.2215896
chr1	cg20263853	5'UTR;5'UTR	RERE;RERE	0.1891849	3.87E-05	0.2236807
chr16	cg16640791	Body;Body;Body	KLHDC4;KLHDC4;KLHDC4;KLHDC4;KLHDC4;KLHDC4;KLHDC4;KLHDC4	-0.1633468	3.89E-05	0.2236807
chr22	cg04898572	Body	RBX1;RBX1;XPNPEP3;XPNPEP3	0.1631946	3.96E-05	0.2263015
chr11	cg09883841	Body;5'UTR	SLC22A12;SLC22A12	0.0969547	4.24E-05	0.2384456
chr5	cg27441580	1stExon;1stExon;1stExon;5'UTR;5'UTR;5'UTR	CREBRF;CREBRF;CREBRF;CREBRF;CREBRF;CREBRF	0.1433989	4.24E-05	0.2384456
chr13	cg06559111	TSS1500;TSS1500	SMAD9;SMAD9;SMAD9	-0.1330631	4.41E-05	0.2401705
chr3	cg17641710	Body	GNAI2;GNAI2;GNAI2	0.1058988	4.42E-05	0.2401705
chr6	cg20636743			0.1508406	4.52E-05	0.2401705
chr15	cg08510096	TSS1500;Body	RP11-387D10.3;RP11-387D10.3;RP11-387D10.2	0.1574588	4.56E-05	0.2401705

chr17	cg06556650	Body		0.296068	4.58E-05	0.2401705
chr8	cg11007075	Body	PEBP4	0.1429672	4.62E-05	0.2401705
chr15	cg14555697		SEMA6D;SEMA6D;SEMA6D;SEMA6D	-0.1429491	4.64E-05	0.2401705
chr12	cg22566355	Body		0.1200445	4.69E-05	0.2401705
chr9	cg08491582	Body;Body	TMOD1	0.1678569	4.69E-05	0.2401705
chr17	cg06169270	TSS1500	TTLL6;TTLL6;TTLL6;TTLL6;TTLL6;TTLL6	0.1058255	4.70E-05	0.2401705
chr7	cg03894891		AC073094.4	-0.213274	4.73E-05	0.2401705
chr15	cg11397301	TSS1500;TSS1500	SCG5;SCG5;SCG5	0.1324067	4.74E-05	0.2401705
chr17	cg03723356	TSS1500	CDK5RAP3;CDK5RAP3	0.1514629	4.74E-05	0.2401705
chr19	cg18763727	Body;Body	BCKDHA;BCKDHA	0.1359618	4.82E-05	0.2401705
chr1	cg13333788	Body	ZDHHC18;ZDHHC18	0.1404523	4.84E-05	0.2401705
chr3	cg11473272			-0.2067107	4.87E-05	0.2401705
chr8	cg09503853	TSS1500	TSPYL5	-0.2030642	4.91E-05	0.2401705
chr3	cg02019125			0.1628423	4.94E-05	0.2401705
chr21	cg16934894	TSS1500;TSS1500	BTG3;BTG3;BTG3;BTG3	0.1096233	4.95E-05	0.2401705
chr6	cg22578433	TSS1500;TSS1500;TSS1500	CCND3;CCND3;CCND3;CCND3;CCND3;CCND3;CCND3;TAF8;TAF8;TAF8;TAF8;TAF8;CCND3;CCND3;TAF8;TAF8;CCND3	0.257178	4.96E-05	0.2401705
chr3	cg16722884			-0.1407572	5.00E-05	0.2401705
chr11	cg03603211	Body;Body;Body	MYO7A	0.2054707	5.14E-05	0.2401705
chr7	cg03935444	3'UTR	AMZ1;AMZ1	-0.168973	5.14E-05	0.2401705
chr15	cg12870052	Body;Body;Body;Body	BLM;BLM;BLM;BLM	-0.2573995	5.24E-05	0.2401705
chr4	cg02233041	TSS200;Body;Body	TNIP3;TNIP3;TNIP3	-0.2815617	5.26E-05	0.2401705
chr4	cg02625241	Body;Body	NFKB1;NFKB1	-0.2316465	5.27E-05	0.2401705

chr4	cg20215176			-0.2166446	5.41E-05	0.2401705
chr20	cg20316542	5'UTR;TSS1500	TCEA2;TCEA2;TCEA2;TCEA2;TCEA2;TCEA2;TCEA2	-0.1432772	5.43E-05	0.2401705
chr6	cg26104293			-0.18123	5.46E-05	0.2401705
chr9	cg05369810			-0.1854433	5.51E-05	0.2401705
chr19	cg09859911	Body		0.1112152	5.54E-05	0.2401705
chr14	cg27504299	TSS1500;TSS1500	TCL1B;TCL1B;RP11-1070N10.6;RP11-1070N10.6	-0.1760711	5.56E-05	0.2401705
chr7	cg10763891		GNGT1	-0.1777126	5.61E-05	0.2401705
chr11	cg17695512	1stExon	OR10AG1	-0.2727632	5.62E-05	0.2401705
chr19	cg16146513	Body		0.1401169	5.66E-05	0.2401705
chr5	cg11919883		RN5S176;RP11-122F24.1	-0.2201051	5.66E-05	0.2401705
chr14	cg10842530	Body;Body;Body	ACTN1;ACTN1;ACTN1	0.1118349	5.66E-05	0.2401705
chr19	cg04660111	1stExon;1stExon;5'UTR;5'UTR	FUT2;FUT2;FUT2;FUT2;FUT2;FUT2	0.1469437	5.75E-05	0.2401705
chr3	cg09007811	Body		-0.1991592	5.82E-05	0.2401705
chr18	cg21320412	Body		-0.1319903	5.84E-05	0.2401705
chr2	cg10079538			-0.1975943	5.85E-05	0.2401705
chr16	cg05539847	TSS1500;TSS1500;TSS1500;Body	HSD3B7;HSD3B7;HSD3B7;HSD3B7;HSD3B7	0.1259354	5.87E-05	0.2401705
chr15	cg02726566			-0.1581877	6.00E-05	0.2401705
chr5	cg22866825	TSS1500	PHAX;PHAX;PHAX;PHAX	0.360166	6.06E-05	0.2401705
chr3	cg04721651	TSS1500;TSS1500;TSS1500	MGLL;MGLL;MGLL	0.1643141	6.06E-05	0.2401705
chr17	cg16558432	1stExon;3'UTR	NLRP1;NLRP1;NLRP1;NLRP1	0.701184	6.06E-05	0.2401705
chr11	cg08458745	Body	ETS1	0.0990348	6.10E-05	0.2401705
chr14	cg15209695	Body	TMED10;TMED10;TMED10;TMED10	0.1589534	6.13E-05	0.2401705
chr16	cg26845395	Body	CIRH1A;RP11-70O5.1	-0.1591572	6.14E-05	0.2401705
chr7	cg14156592	Body;Body;Body	BCAP29;BCAP29;BCAP29;BCAP29	-0.2227892	6.17E-05	0.2401705

chr14	cg04957377		NDRG2	-0.1601038	6.17E-05	0.2401705
chr2	cg14765416	Body	AC018737.1	0.1519566	6.17E-05	0.2401705
chr2	cg04751078			-0.1863306	6.28E-05	0.2401705
chr10	cg02101203	Body	FRMD4A;FRMD4A	0.3783426	6.28E-05	0.2401705
chr2	cg22957691	Body;Body	FEZ2;FEZ2;FEZ2	-0.2303825	6.30E-05	0.2401705
chr1	cg22167396			-0.2096017	6.37E-05	0.2401705
chr22	cg06768071	Body	SCUBE1	0.1402682	6.38E-05	0.2401705
chr22	cg09390308			-0.2002207	6.43E-05	0.2401705
chr15	cg23991388	Body	ADAMTS17	-0.2542202	6.52E-05	0.2401705
chr3	cg20197622			-0.1579141	6.55E-05	0.2401705
chr15	cg01899252			0.1053505	6.55E-05	0.2401705
chr16	cg01067137	Body	ZNF646	0.1464173	6.60E-05	0.2401705
chr17	cg21214508	1stExon	KANSL1;KANSL1;KANSL1;KANSL1;KANSL1;KANSL1;KANSL1;KANSL1	0.2452169	6.63E-05	0.2401705
chr3	cg04324158			-0.1887632	6.65E-05	0.2401705
chr17	cg06496197		SNORA70	0.1062888	6.75E-05	0.2401705
chr17	cg12219401	5'UTR;TSS1500;Body;Body;Body	PEMT;U6;PEMT;PEMT;PEMT	-0.1396135	6.79E-05	0.2401705
chr13	cg20400045	Body	CRYL1	-0.1948689	6.80E-05	0.2401705
chr17	cg13392258			0.2689495	6.86E-05	0.2401705
chr19	cg02445229			0.1054484	6.86E-05	0.2401705
chr20	cg22147003			-0.1215726	6.91E-05	0.2401705
chr2	cg25272883			-0.2190525	6.93E-05	0.2401705
chr6	cg18356679		HEY2;RP11-624M8.1;RP11-624M8.1	-0.2655606	6.98E-05	0.2401705
chr7	cg23055674	5'UTR;TSS1500;5'UTR	PILRB;PILRB;PILRB;PILRB;PILRB;PILRB;PILRB;PILRB;PILRB;PILRB;PILRB;PILRB	0.1050409	7.00E-05	0.2401705
chr1	cg00404394	5'UTR	FGR;FGR;FGR	0.1114574	7.01E-05	0.2401705

chr14	cg02411284	Body	CLMN;CLMN;CLMN	-0.1507542	7.05E-05	0.2401705
chr11	cg05208605			0.1999557	7.05E-05	0.2401705
chr6	cg03217204	TSS1500;TSS1500	ARG1;ARG1;ARG1;ARG1;ARG1;ARG1;ARG1;ARG1	-0.1916049	7.09E-05	0.2401705
chr2	cg22303418		OTX1	0.1201604	7.09E-05	0.2401705
chr21	cg10538440	3'UTR;Body	SIM2;SIM2	-0.1651974	7.17E-05	0.2401705
chr19	cg22023664	5'UTR	GNG7	0.1170779	7.25E-05	0.2401705
chr1	cg26377940		RP11-373J16.1	-0.1969658	7.26E-05	0.2401705
chr19	cg26542091	Body		0.1127254	7.37E-05	0.2401705
chr1	cg04906485	Body		-0.1531284	7.39E-05	0.2401705
chr8	cg17884069	Body;Body;Body;Body;Body;Body;Body;Body		-0.2478801	7.40E-05	0.2401705
chr6	cg09808864	TSS1500	KIAA1244	0.2124844	7.47E-05	0.2401705
chr7	cg16450683	TSS1500	TAS2R4;SSBP1	-0.1642216	7.59E-05	0.2401705
chr4	cg05812836	TSS1500;5'UTR;1stExon	C4orf33;SCLT1;SCLT1;SCLT1;C4orf33;SCLT1;SCLT1;SCLT1;SCLT1;C4orf33;C4orf33	0.2384734	7.59E-05	0.2401705
chr20	cg01363264			0.1308334	7.60E-05	0.2401705
chr19	cg21977234	Body;Body		0.4192799	7.68E-05	0.2401705
chr22	cg05977840	5'UTR;5'UTR;1stExon;1stExon;Body	LIMK2;LIMK2;LIMK2;LIMK2;LIMK2;LIMK2;LIMK2;LIMK2;LIMK2	0.1136428	7.72E-05	0.2401705
chr1	cg14775431			0.1005191	7.76E-05	0.2401705
chr15	cg24164973	Body;Body		0.2478739	7.76E-05	0.2401705
chr20	cg17474349			0.1005185	7.82E-05	0.2401705
chr1	cg26009832	Body;Body	ATP1B1	-0.2041717	7.83E-05	0.2401705
chr16	cg05352821	Body		0.2008033	7.88E-05	0.2401705
chr1	cg21220721	Body;Body;Body;Body	ACOT7;ACOT7;ACOT7	0.5628659	7.88E-05	0.2401705
chr19	cg16715953			0.1660977	7.90E-05	0.2401705
chr8	cg23342367	Body;Body	TPD52;TPD52	-0.2465877	7.94E-05	0.2401705

chr5	cg01610123			-0.1499423	7.98E-05	0.2401705
chr14	cg18731460	Body	SERPINA5	-0.1650366	7.98E-05	0.2401705
chr9	cg03492094	Body;Body	SEMA4D;SEMA4D	0.1462526	8.03E-05	0.2401705
chr12	cg05482226	Body;Body;Body;Body;Body		0.192532	8.04E-05	0.2401705
chr11	cg15236764			0.2643114	8.14E-05	0.2401705
chr14	cg02969500	Body;Body;Body;Body	NPAS3	-0.1419109	8.20E-05	0.2401705
chr14	cg17487559	TSS1500	CATSPERB;CATSPERB;CATSPERB;CATSPERB	-0.1472439	8.20E-05	0.2401705
chr13	cg07175985	TSS200	SACS	0.2178156	8.23E-05	0.2401705
chr10	cg12807368	Body		-0.1842219	8.23E-05	0.2401705
chr1	cg14791530	TSS1500	CTBS;CTBS;CTBS;CTBS	-0.1635032	8.23E-05	0.2401705
chr16	cg02264459			-0.3026294	8.24E-05	0.2401705
chr12	cg22594773	5'UTR;5'UTR	PITPNM2;PITPNM2;PITPNM2;PITPNM2;PITPNM2	0.1438495	8.25E-05	0.2401705
chr14	cg27040968			-0.1466228	8.27E-05	0.2401705
chr17	cg24922045	TSS200	GAS2L2	0.1204364	8.28E-05	0.2401705
chr17	cg19480933	Body;Body		0.118089	8.29E-05	0.2401705
chr2	cg13603638	Body		-0.1452059	8.31E-05	0.2401705
chr3	cg11906641	Body;Body;Body;Body;Body;Body;Body;Body;Body	OPA1;OPA1-AS1;OPA1-AS1;Y_RNA;OPA1	-0.1562661	8.31E-05	0.2401705
chr5	cg20981515	Body	PDGFRB;PDGFRB;PDGFRB	-0.2712373	8.34E-05	0.2401705
chr17	cg13569987	3'UTR	SDF2;SDF2;SDF2	-0.1257859	8.36E-05	0.2401705
chr18	cg02158486	TSS1500	MAPK4;MAPK4	0.2318734	8.38E-05	0.2401705
chr7	cg03528037	3'UTR	MDH2;MDH2;MDH2	-0.1607747	8.41E-05	0.2401705
chr20	cg13676099	Body		0.1147205	8.46E-05	0.2401705
chr14	cg11789185	TSS1500;TSS1500;TSS1500	C14orf93;C14orf93;C14orf93;C14orf93;C14orf93;C14orf93;C14orf93;C14orf93	0.1875541	8.55E-05	0.2401705
chr11	cg16047053	TSS1500	OR1S2	-0.1770229	8.56E-05	0.2401705

chr11	cg10611016	TSS1500	C11orf42	-0.1446558	8.62E-05	0.2401705
chr8	cg25910699			-0.1977986	8.69E-05	0.2401705
chr2	cg02575035	Body	AC007092.1;AC007092.1;AC007092.1;AC007092.1;AC007092.1	-0.1602261	8.70E-05	0.2401705
chr3	cg00449491	Body	ZNF148	-0.1951875	8.70E-05	0.2401705
chr2	cg00143376	Body		0.1226573	8.80E-05	0.2401705
chr7	cg07829731		U6	-0.1959841	8.80E-05	0.2401705
chr7	cg10688790	TSS1500;Body		-0.1602887	8.80E-05	0.2401705
chr2	cg20669090	Body;Body	FAM176A;FAM176A	-0.1585733	8.81E-05	0.2401705
chr12	cg13588483	5'UTR	RP11-444D3.1;RP11-444D3.1;RP11-444D3.1;RP11-444D3.1;RP11-444D3.1	-0.1551865	8.88E-05	0.2401705
chr14	cg05257429	5'UTR	FOXN3;FOXN3;FOXN3;FOXN3;FOXN3;RP11-33N16.3	0.1556796	8.89E-05	0.2401705
chr2	cg12028998			0.1441575	8.92E-05	0.2401705
chr20	cg22853608			0.1237961	8.93E-05	0.2401705
chr6	cg26592496		RP1-167F1.2	-0.198994	8.93E-05	0.2401705
chr17	cg07115820	1stExon	EPX	0.1265109	8.95E-05	0.2401705
chr10	cg01344420			-0.1314424	8.97E-05	0.2401705
chr3	cg04603413			-0.1564895	9.04E-05	0.2401705
chr17	cg19139618	Body;Body		-0.1410335	9.06E-05	0.2401705
chr11	cg09742895	Body;Body;Body;Body	GRIA4	-0.138853	9.12E-05	0.2401705
chr11	cg18571531	Body;Body	SHANK2-AS1	0.115282	9.12E-05	0.2401705
chr14	cg26109393			0.0891353	9.16E-05	0.2401705
chr22	cg20182202	Body;Body;Body	CABIN1;CABIN1	0.1835414	9.19E-05	0.2401705
chr5	cg04897804	1stExon;1stExon	SOX30;SOX30	0.1529027	9.21E-05	0.2401705
chr2	cg07667018	5'UTR;5'UTR;5'UTR;5'UTR;TSS200;TSS200;ExonBnd;ExonBnd;ExonBnd;ExonBnd	TPO;TPO;TPO;TPO;TPO;TPO;TPO;TPO;TPO	-0.1578751	9.23E-05	0.2401705

chr11	cg17134838		RP11-804A23.1	0.1999118	9.25E-05	0.2401705
chr1	cg24474319	Body		0.0932076	9.26E-05	0.2401705
chr14	cg10995649			-0.2024494	9.30E-05	0.2401705
chr22	cg14162806	TSS1500	PDGFB	0.1015697	9.38E-05	0.2401705
chr15	cg04151444			0.1205713	9.39E-05	0.2401705
chr14	cg20267408	5'UTR;5'UTR;5'UTR;5'UTR;5'UTR;5'UTR;5'UTR;5'UTR;5'UTR;5'UTR;5'UTR	SERPINA1;SERPINA1;SERPINA1;SERPINA1;SERPINA1;SERPINA1;SERPINA1;SERPINA1;SERPINA1;SERPINA1;SERPINA1	-0.1642808	9.40E-05	0.2401705
chr17	cg01798813			0.3535299	9.41E-05	0.2401705
chr19	cg04447623	5'UTR	FZR1	0.1194829	9.44E-05	0.2401705
chr17	cg14782678	Body	RP11-104H15.7	0.1756714	9.49E-05	0.2401705
chr11	cg05468843	Body;Body	IL10RA;IL10RA;IL10RA;IL10RA;IL10RA;IL10RA;IL10RA	-0.3408507	9.49E-05	0.2401705
chr2	cg14093610	Body;Body;Body;Body;Body;Body;Body;Body;TSS200	CCDC140	-0.1937788	9.53E-05	0.2401705
chr5	cg09183284		NPR3	-0.1425979	9.59E-05	0.2401705
chr2	cg03574735	Body;Body	MFSD2B;FKBP1B;FKBP1B	0.1727923	9.60E-05	0.2401705
chr1	cg00633339			-0.156829	9.63E-05	0.2401705
chr3	cg00220225	5'UTR	SEMA3F;SEMA3F;SEMA3F;SEMA3F;RP11-493K19.3;RP11-493K19.3;SEMA3F	0.2209975	9.64E-05	0.2401705
chr6	cg25533100			-0.1398795	9.76E-05	0.2401705
chr10	cg18003644	Body;Body;Body;Body;Body;Body	PAX2	-0.1851393	9.79E-05	0.2401705
chr7	cg00292336			0.161808	9.80E-05	0.2401705
chr6	cg17485531	5'UTR	USP49;USP49;USP49;USP49	0.0897347	9.84E-05	0.2401705

chr5	cg18417562	Body;TSS1500	LRRTM2;LRRTM2;LRRTM2;LRRTM2;CTNNA1;CTNNA1;CTNNA1;CTNNA1;CTNNA1;CTNNA1;CTNNA1;CTNNA1;CTNNA1;CTNNA1;CTNNA1;CTNNA1;CTNNA1;CTNNA1;CTNNA1	-0.178843	9.89E-05	0.2401705
chr6	cg22739554			-0.1812786	9.92E-05	0.2401705
chr12	cg18015427		RP11-121J20.1	-0.1818328	9.99E-05	0.2401705
chr7	cg17717565	Body;Body	TRIM4;TRIM4	0.2229714	0.0001	0.2401705
chr1	cg19304100	5'UTR;5'UTR;TSS200	DCST1;DCST2;DCST1;DCST2;DCST1;DCST1;DCST1	-0.1468903	0.0001003	0.2401705
chr14	cg14773011	ExonBnd;ExonBnd;ExonBnd;Body;Body;Body	CTD-2002H8.2;PSMA3;PSMA3;PSMA3;PSMA3;C14orf37;PSMA3;PSMA3;PSMA3	-0.1245625	0.0001004	0.2401705
chr18	cg10418276	Body;Body;Body	ANKRD12	-0.2070833	0.0001006	0.2401705
chr5	cg26318502			-0.1427832	0.0001007	0.2401705
chr11	cg22364991	Body;Body;5'UTR;5'UTR	LRRC4C;LRRC4C;LRRC4C;LRRC4C;LRRC4C	-0.1856966	0.0001021	0.2401705
chr4	cg05386577	Body;Body	BBS7;BBS7	-0.1305419	0.0001023	0.2401705
chr6	cg15458313	TSS1500	GPLD1;GPLD1;GPLD1;GPLD1;GPLD1	-0.1576972	0.0001028	0.2401705
chr13	cg19247877	5'UTR	SACS;SACS	-0.1431425	0.0001037	0.2401705
chr5	cg05876737	TSS1500;Body;Body;Body;Body	PDE4D;PDE4D;PDE4D;PDE4D	0.1417602	0.0001047	0.2401705
chr16	cg14484628	TSS1500;TSS1500;5'UTR;5'UTR	SEPT1;SEPT1;ZNF48;ZNF48;SEPT1;ZNF48	0.0930264	0.0001052	0.2401705
chr17	cg11793449			0.0939633	0.0001053	0.2401705
chr4	cg09444160	TSS1500;TSS1500;TSS1500;TSS1500	LARP1B;LARP1B;LARP1B;LARP1B;LARP1B	0.2533322	0.0001059	0.2401705
chr11	cg07490694	Body	PCSK7	-0.1563985	0.0001068	0.2401705
chr14	cg07960587			-0.1529407	0.000107	0.2401705
chr6	cg26771258		SNORD46	-0.1853363	0.0001071	0.2401705
chr11	cg24425958	Body	RP11-652L8.2	0.3864048	0.0001072	0.2401705

chr9	cg08004835	Body;Body;Body;Body		-0.1815997	0.0001074	0.2401705
chr17	cg16500497	Body		-0.161519	0.0001076	0.2401705
chr12	cg25957306	Body	BAZ2A;BAZ2A;BAZ2A;BAZ2A;BAZ2A;BAZ2A	-0.1290399	0.0001076	0.2401705
chr15	cg23088318	5'UTR;5'UTR;5'UTR	SNRPN	0.331789	0.0001078	0.2401705
chr5	cg11688402		AC091969.1	-0.1478792	0.0001082	0.2401705
chr2	cg01137798			-0.1320127	0.0001084	0.2401705
chr1	cg20358574	Body;Body;Body;Body;Body;Body;Body;Body;Body;Body;Body	TMEM9;TMEM9;TMEM9;TMEM9	-0.1396599	0.0001089	0.2401705
chr9	cg17591980			-0.2064679	0.0001097	0.2401705
chr9	cg14080050	Body		-0.1377946	0.0001098	0.2401705
chr17	cg19265972	Body	ASGR1;ASGR1;ASGR1;ASGR1;ASGR1;ASGR1	0.1568377	0.0001099	0.2401705
chr8	cg20669366		SLC45A4;SLC45A4	-0.1341972	0.0001101	0.2401705
chr4	cg07519811		RP11-217E13.1	-0.1327451	0.0001101	0.2401705
chr2	cg14869618	Body;Body;Body;Body;Body;Body;Body	AC010680.1;AC010680.1	-0.1497993	0.0001104	0.2401705
chr5	cg26181433	Body	WDR55	-0.1506679	0.0001105	0.2401705
chr20	cg26527490			0.1543704	0.000111	0.2401705
chr11	cg09343676			0.1224386	0.0001112	0.2401705
chr20	cg27509634	Body;Body;Body;Body	ZFP64;ZFP64;ZFP64	-0.1297648	0.0001113	0.2401705
chr14	cg27131955	Body		0.1667397	0.0001116	0.2401705
chr6	cg18657788			-0.1601078	0.0001117	0.2401705
chr17	cg26055019	Body;Body		0.1279651	0.000112	0.2401705
chr2	cg24713838	TSS1500;TSS1500;Body	WDR35;WDR35;WDR35;AC079145.4	0.1501584	0.0001123	0.2401705

chr15	cg10778736	TSS200;TSS200	DUOXA2;DUOX2;DUOX2;DUOX2;DUOXA2;DUOXA2	0.130143	0.0001126	0.2401705
chr2	cg10228677	Body;Body;Body;Body	AC096753.1	-0.1667459	0.0001128	0.2401705
chr11	cg19237879	TSS1500;TSS1500	SCUBE2;SCUBE2;SCUBE2;SCUBE2;SCUBE2	0.136261	0.000113	0.2401705
chr11	cg08288223	Body	SHANK2;SHANK2;SHANK2	0.1961714	0.0001131	0.2401705
chr8	cg02141358	Body		-0.1571632	0.0001131	0.2401705
chr7	cg06891344	5'UTR;5'UTR;5'UTR;5'UTR	NRCAM;NRCAM;NRCAM;NRCAM;NRCAM;NRCAM;NRCAM;NRCAM	-0.1679247	0.0001132	0.2401705
chr10	cg25308531	TSS1500;5'UTR;Body;Body;5'UTR;Body;Body;Body	NCOA4;NCOA4;NCOA4;NCOA4;NCOA4;NCOA4;NCOA4	0.1328666	0.0001144	0.2418829
chr14	cg21284750	5'UTR;5'UTR;5'UTR;5'UTR;5'UTR;1stExon;1stExon;1stExon;1stExon;1stExon	CMTM5;CMTM5;CMTM5;CMTM5;CMTM5;CMTM5;CMTM5;CMTM5;CMTM5;CMTM5;CMTM5	0.1064455	0.000115	0.2418829
chr2	cg03946834	Body		0.1368316	0.000115	0.2418829
chr15	cg08291506			0.1082912	0.0001156	0.2424773
chr19	cg20739309		KLK12	-0.3019735	0.0001164	0.2434194
chr14	cg23013137	Body	TSSK4;TSSK4	0.1028048	0.0001182	0.2464513
chr5	cg26834542	Body	SNX24;SNX24;SNX24;SNX24;SNX24;SNX24;SNX24	-0.1672454	0.0001188	0.2466822
chr1	cg24912565	Body	GBAP1;GBAP1;GBAP1;GBAP1;GBAP1	0.1287544	0.0001194	0.2466822
chr11	cg06483739	Body	RCN1;RP1-65P5.3;RCN1;RCN1;RCN1	-0.1586476	0.0001194	0.2466822
chr19	cg05360373			-0.1631172	0.00012	0.2466822
chr11	cg01437998	TSS1500	OR4D10	-0.1403007	0.0001201	0.2466822
chr10	cg04313719			-0.1461883	0.0001204	0.2466822
chr1	cg17909977	3'UTR	ADSS;ADSS;RP11-518L10.2	-0.2330364	0.0001207	0.2466822
chr10	cg17569303	5'UTR	SGMS1	-0.1487387	0.0001213	0.2466822

chr1	cg02086349	5'UTR;1stExon;TSS1500	MTMR11;MTMR11;MTMR11;MTMR11;MTMR11;MTMR11;MTMR11;MTMR11;MTMR11;MTMR11;MTMR11;MTMR11;MTMR11	0.1298406	0.0001219	0.2466822
chr4	cg05054673	TSS200	RP11-317M11.1;LNX1;RP11-317M11.1	-0.1635206	0.000122	0.2466822
chr2	cg11432540			-0.169103	0.0001221	0.2466822
chr5	cg09218258	Body	CTD-2247C11.4;CTD-2247C11.3;CTD-2247C11.3;CTD-2247C11.3	-0.1513914	0.0001234	0.2485742
chr5	cg08969102		CDKL3;CDKL3	0.1795793	0.0001244	0.249637
chr2	cg01109729	Body	FAM110C;FAM110C	-0.1320981	0.000125	0.249637
chr11	cg00188327	5'UTR;5'UTR;5'UTR	MICAL2;MICAL2;MICAL2;MICAL2;MICAL2;MICAL2;MICAL2	-0.1963198	0.0001251	0.249637
chr13	cg25485930	Body	POU4F1-AS1;POU4F1-AS1;POU4F1-AS1	0.2745981	0.000126	0.249637
chr5	cg19565757	TSS1500;TSS200;TSS1500	PAIP1;PAIP1;PAIP1;PAIP1;PAIP1;PAIP1;PAIP1;PAIP1;PAIP1;PAIP1	0.1756594	0.0001268	0.249637
chr17	cg14960238			0.1469595	0.000127	0.249637
chr12	cg09504658	TSS1500;TSS1500;TSS1500;TSS1500;TSS1500;TSS1500	CLEC2D;CLEC2D;CLEC2D;CLEC2D;CLEC2D;CLEC2D;CLEC2D;CLEC2D	-0.1447581	0.0001274	0.249637
chr4	cg12344589	Body;Body	AFAP1	-0.1830612	0.0001276	0.249637
chr7	cg12508286	TSS1500	EN2;AC008060.8	0.2114273	0.0001278	0.249637
chr3	cg16323609	TSS1500	PRKAR2A-AS1;PRKAR2A-AS1;PRKAR2A-AS1;PRKAR2A-AS1;PRKAR2A-AS1;PRKAR2A-AS1;PRKAR2A;PRKAR2A;PRKAR2A;PRKAR2A	0.1184959	0.000128	0.249637
chr1	cg01715384			0.1123616	0.0001282	0.249637
chr9	cg13614383	Body	SHC3	-0.2311797	0.0001283	0.249637
chr16	cg09480190	5'UTR;Body	KIAA0182;KIAA0182	0.3417248	0.0001284	0.249637
chr6	cg11729950	Body;Body	MDN1;RP1-122O8.7	-0.1251594	0.0001297	0.2500993
chr1	cg16219384			-0.147187	0.0001298	0.2500993

chr16	cg04999025	Body	ABCC1	0.1135852	0.0001304	0.2500993
chr6	cg25790531		RP1-124C6.1	-0.1962326	0.000131	0.2500993
chr4	cg21783716	Body;Body	RP11-76G10.1	-0.1408	0.0001313	0.2500993
chr5	cg02326273			-0.1968934	0.0001313	0.2500993
chr19	cg01539133			0.2044953	0.0001316	0.2500993
chr12	cg13584406			-0.2087649	0.0001316	0.2500993
chr1	cg19038540	TSS1500	S1PR1;S1PR1;S1PR1;RP4-575N6.4	0.3455533	0.0001318	0.2500993
chr5	cg00068571	Body;Body;Body;Body;Body	MAST4	-0.174991	0.0001336	0.2519271
chr1	cg04436877			0.1973093	0.0001338	0.2519271
chr6	cg13250847	5'UTR;5'UTR;Body;Body	NKAIN2	0.1346044	0.0001339	0.2519271
chr6	cg22797971			0.1241232	0.0001342	0.2519271
chr16	cg04898487	Body;Body;Body		-0.1864806	0.0001356	0.2539369
chr2	cg08230856	Body	PRKCE;PRKCE	-0.1357859	0.0001369	0.2542534
chr1	cg03668686	Body	PROX1-AS1;PROX1-AS1;PROX1-AS1	-0.1619803	0.0001376	0.2542534
chr11	cg18446861	Body;Body	RP11-708L7.6;DIXDC1;DIXDC1;DIXDC1;DIXDC1	-0.1249813	0.0001378	0.2542534
chr5	cg11142038		RP11-381K20.2	-0.1376933	0.0001379	0.2542534
chr21	cg10335612	Body;Body;Body;Body	TMPRSS3	-0.1438613	0.000138	0.2542534
chr11	cg25070692	Body;Body;Body;Body		-0.1626637	0.0001381	0.2542534
chr4	cg04195136	TSS1500	ELMOD2;ELMOD2;ELMOD2;ELMOD2;ELMOD2	0.2745816	0.0001385	0.2542534
chr5	cg03637218	Body	AP3S1;AP3S1;AP3S1	-0.2055633	0.0001386	0.2542534
chr20	cg05753799	5'UTR	C20orf27;C20orf27;C20orf27;C20orf27;C20orf27	-0.1308637	0.0001399	0.2555143
chr6	cg18068929	Body	LINC00340	-0.1652343	0.00014	0.2555143

chr11	cg12954619	TSS1500;TSS1500;Body	GANAB;GANAB;GANAB;GANAB;GANAB;GANAB;GANAB;GANAB;GANAB;GANAB	0.11806	0.0001404	0.2555583
chr20	cg20393839	TSS1500;TSS1500;TSS1500;Body	CEBPB	0.1351183	0.0001414	0.2567971
chr13	cg05296619	Body		-0.1037809	0.0001423	0.2577725
chr6	cg14823860			0.1467143	0.0001443	0.2577725
chr3	cg18022607	Body		-0.1600004	0.0001444	0.2577725
chr1	cg07019697	3'UTR	ZBTB8B	-0.1284612	0.0001448	0.2577725
chr15	cg06748889			-0.160702	0.000145	0.2577725
chr7	cg06389955	Body;Body;Body		-0.1373115	0.0001453	0.2577725
chr19	cg01656853	TSS200;TSS200	FUT2;FUT2;FUT2	0.1283231	0.0001467	0.2577725
chr1	cg11070531	Body;Body	ZNF692;ZNF692;ZNF692;ZNF692;ZNF692;ZNF692;ZNF692	-0.137246	0.0001474	0.2577725
chr15	cg13645296	Body	DAPK2;DAPK2;DAPK2;DAPK2	0.2162076	0.0001475	0.2577725
chr9	cg13587621	Body;Body		0.1986053	0.0001478	0.2577725
chr11	cg16760899			0.3300931	0.000148	0.2577725
chr8	cg08989195	5'UTR	RP11-1023P17.2;ST18;ST18;ST18;ST18	-0.1341099	0.0001484	0.2577725
chr3	cg16371949			-0.2329192	0.0001489	0.2577725
chr4	cg09275205	TSS1500;Body;Body;Body;Body;Body	GPM6A;GPM6A;GPM6A;GPM6A;GPM6A;GPM6A;GPM6A;GPM6A;GPM6A;GPM6A;GPM6A;GPM6A;GPM6A	-0.2075609	0.0001492	0.2577725
chr16	cg23684449	Body;1stExon;5'UTR	GPT2;GPT2	0.0934388	0.0001495	0.2577725
chr3	cg03060158	TSS1500;TSS1500;TSS1500;TSS1500;TSS1500;TSS1500;TSS1500	IP6K2;IP6K2;IP6K2;IP6K2;IP6K2;IP6K2;IP6K2;IP6K2;IP6K2;IP6K2;IP6K2;IP6K2;IP6K2	0.1462694	0.0001497	0.2577725
chr7	cg26158288	TSS1500;TSS1500;TSS1500;TSS1500	ADAM22;ADAM22;ADAM22;ADAM22;ADAM22;ADAM22;ADAM22	0.162543	0.0001498	0.2577725

chr2	cg04180623	Body;Body	AC013461.1;MLK7-AS1;MLK7-AS1	-0.1246297	0.0001512	0.2577725
chr7	cg23332328	Body	CYP2W1;CYP2W1;CYP2W1	0.1284724	0.0001514	0.2577725
chr16	cg14871186	Body;5'UTR;Body	USP7;USP7;USP7;USP7;USP7;USP7;USP7	0.0856868	0.0001517	0.2577725
chr12	cg02614847	Body	TSPAN31;TSPAN31;TSPAN31;TSPAN31;TSPAN31;TSPAN31	0.0769589	0.0001521	0.2577725
chr11	cg03774275	5'UTR	PKNOX2;PKNOX2;PKNOX2;PKNOX2;PKNOX2;PKNOX2;PKNOX2;PKNOX2;PKNOX2;PKNOX2;PKNOX2;PKNOX2;PKNOX2;PKNOX2;PKNOX2	0.2194152	0.0001524	0.2577725
chr6	cg24527098			0.2926745	0.0001526	0.2577725
chr15	cg03365496			0.1681751	0.0001532	0.2577725
chr2	cg01319403			0.1606185	0.0001532	0.2577725
chr21	cg06490962		RUNX1	-0.1261221	0.0001533	0.2577725
chr2	cg05704183		AC074093.1;AC074093.1;AC074093.1	-0.2064241	0.0001533	0.2577725
chr19	cg20810702	Body;Body		0.1268153	0.0001539	0.2577725
chr19	cg25697727	TSS1500;TSS1500;TSS1500	PPAP2C;PPAP2C	0.3014278	0.0001543	0.2577725
chr17	cg15756507	Body;Body		0.1430343	0.0001544	0.2577725
chr17	cg03023732			-0.2043231	0.0001546	0.2577725
chr6	cg18109427			-0.1570952	0.000155	0.2577725
chr10	cg19043851			-0.1517843	0.0001551	0.2577725
chr7	cg17131606	Body	CYTH3	-0.1326425	0.0001552	0.2577725
chr4	cg24915409	Body	MRPL1;MRPL1	-0.2121445	0.0001553	0.2577725
chr12	cg23457203	TSS200	RP11-1016B18.1;RP11-1016B18.1;RP11-1016B18.1	-0.1584009	0.0001554	0.2577725
chr19	cg23530133	TSS1500	SLC17A7	0.1091004	0.0001562	0.2577725
chr9	cg08709434	TSS1500	ACER2;ACER2	0.1847575	0.0001563	0.2577725
chr4	cg10018232			-0.1905535	0.0001569	0.2577725
chr14	cg07583563	Body	CALM1;CALM1;CALM1;CALM1;CALM1;CALM1;CALM1;CALM1	0.1042206	0.0001569	0.2577725

chr17	cg06559756	Body;Body		0.1275051	0.000157	0.2577725
chr2	cg00933914			-0.1385509	0.0001571	0.2577725
chr3	cg08508763	TSS1500;Body	TRAK1;TRAK1;TRAK1;TRAK1;TRAK1	0.2442249	0.0001576	0.2577725
chr15	cg00593333	Body		0.1010784	0.0001578	0.2577725
chr5	cg16165759	Body	AC091969.1;AC091969.1;AC091969.1	-0.158695	0.0001581	0.2577725
chr17	cg06835483	TSS200	ALOX12B	0.109769	0.0001586	0.2580698
chr1	cg13739348	Body		-0.1855352	0.000159	0.258109
chr14	cg17299745	Body;Body;Body	ITPK1	0.1373821	0.0001606	0.2601352
chr6	cg19412131	5'UTR;5'UTR	ATXN1;ATXN1;ATXN1;ATXN1;ATXN1;ATXN1;ATXN1;ATXN1;ATXN1	-0.2281205	0.0001623	0.2619501
chr6	cg15599182	Body;Body;Body;Body	DST;DST;DST;DST;DST	-0.1407481	0.0001624	0.2619501
chr10	cg23956385	TSS1500;TSS1500	VSTM4	0.0943371	0.000163	0.2623012
chr6	cg14084896	5'UTR	LRFN2	-0.172573	0.0001655	0.2654099
chr2	cg10214540			-0.1919052	0.0001657	0.2654099
chr1	cg10231978			-0.1773126	0.0001679	0.2677144
chr17	cg23007418	Body;Body	CYTH1	0.1206934	0.0001679	0.2677144
chr16	cg04337153	Body	AC004158.3;AC004158.2	-0.1562248	0.0001682	0.2677144
chr3	cg13367729	TSS1500	IQCF6	-0.1619078	0.0001695	0.2690418
chr8	cg27377644			-0.1501812	0.0001703	0.2693899
chr20	cg05282030			0.1336886	0.0001713	0.2693899
chr9	cg09017660		RP11-305L7.6;RP11-305L7.6;RP11-305L7.6;RP11-305L7.6	-0.1337639	0.0001714	0.2693899
chr3	cg25215011	Body;Body	PLCL2-AS1	-0.1295616	0.0001717	0.2693899
chr10	cg06748146	Body;Body;Body;Body;Body	HK1;HK1;HK1	0.1568173	0.0001717	0.2693899
chr2	cg24788999	Body	AC019172.2	-0.1447833	0.0001719	0.2693899
chr1	cg13685026			-0.2011961	0.0001735	0.2701141
chr8	cg05525416	5'UTR	PAG1;PAG1	-0.142525	0.0001739	0.2701141

chr16	cg27402811		RP11-134D3.1	-0.1442277	0.0001741	0.2701141
chr2	cg20205586	TSS1500;TSS1500	SRSF7;SRSF7;SRSF7;SRSF7;SRSF7;SRSF7;SRSF7;SRSF7;GEMIN6	0.1358939	0.0001743	0.2701141
chr10	cg17865604		GPAM;GPAM	-0.2195382	0.0001752	0.2701141
chr12	cg14120215	5'UTR;1stExon	SDS;SDS;SDS;SDS;SDS;SDS;SDS;SDS	0.4197295	0.0001763	0.2701141
chr9	cg13689390	Body	TBC1D2;TBC1D2	-0.129537	0.0001765	0.2701141
chr7	cg07092213	5'UTR	ZFAND2A;ZFAND2A;ZFAND2A;AC091729.9;AC091729.9;AC091729.9;AC091729.9	0.4263887	0.0001767	0.2701141
chr17	cg00860225	Body;Body	SEZ6	0.1307447	0.000177	0.2701141
chr5	cg05685493			0.1672994	0.0001771	0.2701141
chr11	cg04338129	Body	EFEMP2;EFEMP2;EFEMP2;EFEMP2;EFEMP2;EFEMP2;EFEMP2;EFEMP2;EFEMP2	0.1523483	0.0001773	0.2701141
chr22	cg07068187			0.1118053	0.0001776	0.2701141
chr13	cg20382920			-0.1902653	0.0001777	0.2701141
chr2	cg09781514	Body	PRKD3;PRKD3;PRKD3	-0.2003781	0.0001782	0.2701141
chr1	cg01347529	TSS200	EPHX4	0.2242619	0.0001783	0.2701141
chr8	cg07755718		RP11-211C9.1;RP11-211C9.1	-0.1245414	0.0001784	0.2701141
chr2	cg01467882	Body		0.1916345	0.0001791	0.2701738
chr13	cg25084760	Body;Body	PCCA	-0.1994706	0.0001793	0.2701738
chr2	cg26109401			-0.1501142	0.0001796	0.2701738
chr8	cg23791076	5'UTR;5'UTR	TOP1MT;TOP1MT;U6;TOP1MT;TOP1MT;TOP1MT;TOP1MT	0.1485612	0.0001807	0.270766
chr1	cg02290349	5'UTR	FAM46C	0.24183	0.0001807	0.270766
chr22	cg21137052	TSS1500;TSS1500	RPL3;RPL3;RPL3;RPL3;RPL3;RPL3;RPL3;RPL3	0.1784046	0.0001817	0.271612
chr7	cg16276856	Body	VSTM2A;VSTM2A;VSTM2A;VSTM2A	-0.1561282	0.000182	0.271612
chr7	cg09519327	3'UTR;Body	NOS3;NOS3;ATG9B;ATG9B;NOS3;ATG9B;ATG9B;ATG9B	0.3289578	0.0001826	0.271612
chr17	cg18995064	Body;Body;Body;Body	MINK1;MINK1;MINK1;MINK1;MINK1	0.1134528	0.000183	0.271612
chr13	cg02722614	Body;Body		-0.1593763	0.0001832	0.271612

chr18	cg16547027	5'UTR;5'UTR;1st Exon	KCTD1;KCTD1;KCTD1	0.1258808	0.0001846	0.2728692
chr4	cg03336997			-0.189533	0.0001849	0.2728692
chr16	cg24474561	TSS1500;TSS1500;TSS1500;TSS200;Body	CDIPT;CDIPT;CDIPT;CDIPT;CDIPT;CTD-2574D22.5;CDIPT;CTD-2574D22.5;CDIPT	0.1429191	0.0001851	0.2728692
chr13	cg20647118	Body		0.2458967	0.000188	0.2757373
chr19	cg11270237			0.1428749	0.000188	0.2757373
chr12	cg21625740		RP11-319E16.2;RP11-1038A11.3	-0.1309478	0.0001882	0.2757373
chr17	cg04241623	3'UTR;1stExon	NLRP1;NLRP1;NLRP1;NLRP1	0.5248651	0.0001887	0.275798
chr2	cg00429509			-0.1612582	0.0001892	0.2760031
chr13	cg26463289	Body		-0.1162753	0.0001896	0.2760031
chr6	cg00518702			0.1463622	0.0001905	0.2762799
chr16	cg12294213	Body	RP11-473I1.9	0.1382806	0.0001906	0.2762799
chr20	cg16606773	Body	RIN2	-0.1104888	0.0001912	0.2762799
chr8	cg08840751		RP11-100L22.2;RP11-100L22.2;RP11-100L22.2	-0.1460182	0.0001913	0.2762799
chr11	cg06145347	TSS1500;TSS1500;TSS1500	STIP1;STIP1;STIP1;STIP1;STIP1;STIP1;STIP1	0.1150141	0.0001921	0.276936
chr6	cg02876191			-0.1689997	0.0001926	0.2770281
chr6	cg12178807		ZNF292	0.3656537	0.0001943	0.2770754
chr4	cg13213598	5'UTR	FAM53A;Y_RNA;FAM53A;FAM53A;FAM53A;FAM53A	0.3684987	0.0001947	0.2770754
chr10	cg06379721			0.1224961	0.0001954	0.2770754
chr17	cg17808440	Body;Body	VPS53;VPS53;VPS53;VPS53;VPS53	-0.2193141	0.0001954	0.2770754
chr7	cg03094728	Body;Body	AKAP9;AKAP9;AKAP9	-0.179173	0.0001955	0.2770754
chr12	cg24716960			-0.1683042	0.0001956	0.2770754
chr10	cg09255716	TSS200	KIAA1598	-0.121625	0.0001958	0.2770754
chr8	cg00319774	5'UTR	DUSP26;DUSP26;DUSP26;DUSP26	0.1602807	0.0001963	0.2770754
chr2	cg22373593	Body;Body	GALNT13;GALNT13	-0.1505625	0.0001965	0.2770754
chr2	cg21458828			0.1411728	0.000197	0.2770754

chr12	cg05646433	Body;Body		0.1369536	0.0001979	0.2770754
chr3	cg00803692	TSS200;TSS200	CCR5;CCR5;RP11-24F11.2	-0.1559077	0.000198	0.2770754
chr17	cg23261327	Body;Body;Body;Body	BAIAP2;BAIAP2;BAIAP2;BAIAP2;BAIAP2;BAIAP2	0.2385121	0.000198	0.2770754
chr17	cg04303382		CBX8	0.2245856	0.0001995	0.2770754
chr12	cg22098683	TSS1500;TSS1500;TSS1500	HVCN1;HVCN1;HVCN1;HVCN1;HVCN1;HVCN1;HVCN1	0.1386229	0.0002001	0.2770754
chr17	cg15148156	Body;Body	RFFL;RFFL	0.1813633	0.0002002	0.2770754
chr3	cg01838759	Body;Body;Body	NISCH	0.1423124	0.0002005	0.2770754
chr1	cg07851807	Body;Body	PTPRF;PTPRF	0.1074706	0.0002005	0.2770754
chr3	cg18792281	Body;Body;Body	DGKG;DGKG	0.1120432	0.0002006	0.2770754
chr14	cg19387137		RP11-1077A2.1;RP11-108M12.3	-0.1527292	0.0002008	0.2770754
chr6	cg18702715		RP3-468B3.2;RP3-468B3.2	-0.1244592	0.0002008	0.2770754
chr11	cg26906466		RP1-276E15.1	-0.2134743	0.0002015	0.2770754
chr7	cg11214064		AC073130.3;CAV2;CAV2;AC002066.1;CAV2;CAV2;CAV2;CAV2;CAV2;CAV2;CAV2;CAV2;CAV2;CAV2	-0.1192079	0.0002019	0.2770754
chr7	cg16570051	Body	AEBP1	0.1158428	0.0002022	0.2770754
chr16	cg06489965	1stExon;5'UTR	CLDN9;CLDN6;CLDN9	0.1792596	0.0002023	0.2770754
chr11	cg22339837	TSS1500;TSS1500;TSS1500;TSS1500;TSS1500;TSS1500;TSS1500;TSS1500;TSS1500	RTN3;RTN3;RTN3;RTN3;RTN3;RTN3;RTN3;RTN3;RTN3	0.1549837	0.0002028	0.2773076
chr9	cg06782865			-0.1521342	0.0002037	0.2776133
chr20	cg16556868	Body;Body;Body;Body		-0.1591063	0.0002038	0.2776133
chr6	cg07412002	Body;Body	TMEM30A;TMEM30A	-0.1517793	0.0002046	0.2778929
chr3	cg08544192		RP11-71N10.1;RP11-71N10.1	-0.1524598	0.0002048	0.2778929
chr17	cg05558046	Body;Body;TSS1500	GAS7;GAS7	0.0907295	0.0002055	0.2782417

chr18	cg09865411			0.2078965	0.0002058	0.2782417
chr11	cg00797434	Body	CCDC73	-0.1513717	0.0002067	0.2789733
chr5	cg04505045	1stExon;1stExon;Body	SOX30;SOX30	0.1170335	0.0002079	0.279492
chr13	cg10787440			-0.1419393	0.0002083	0.279492
chr19	cg15811468			0.3355962	0.0002083	0.279492
chr3	cg15613488		PLCH1	0.310933	0.0002095	0.280645
chr3	cg19551115	Body;Body;Body	MAGI1;MAGI1	-0.1375832	0.0002114	0.2810933
chr11	cg24466850			0.2156217	0.0002115	0.2810933
chr5	cg26139975		RP11-267A15.1;RP11-267A15.1;RP11-267A15.1	0.2398954	0.0002119	0.2810933
chr12	cg19733736	Body	RDH5;RP11-644F5.10;RDH5;RDH5;RDH5;RDH5;RDH5;RDH5;RDH5;RDH5	0.1419602	0.0002123	0.2810933
chr1	cg26318532			-0.3053628	0.0002124	0.2810933
chr5	cg00292334			-0.2042156	0.0002125	0.2810933
chr3	cg21968765	TSS200	ABI3BP;ABI3BP;ABI3BP;ABI3BP;ABI3BP;ABI3BP	0.1965918	0.0002126	0.2810933
chr4	cg15400629	Body	AC006445.8;AC006445.8	0.1145194	0.0002132	0.2814003
chr15	cg01327313	Body		-0.1377108	0.0002136	0.2814003
chr20	cg01908419	5'UTR;5'UTR;5'UTR	RNF24;RNF24;RNF24	0.1188718	0.0002143	0.2817719
chr14	cg16478616	5'UTR;5'UTR;5'UTR;5'UTR	TRAF3;TRAF3;TRAF3;TRAF3;TRAF3	-0.1436951	0.0002155	0.2818822
chr20	cg26320520			0.1322777	0.000216	0.2818822
chr11	cg16699198	Body	GRAMD1B	-0.1497615	0.0002166	0.2818822
chr22	cg08727202	TSS1500;TSS1500;Body;TSS1500;TSS1500	MPST;TST;MPST;MPST;MPST;MPST;MPST	0.0868734	0.0002171	0.2818822
chr13	cg16543024			-0.1329777	0.000218	0.2818822
chr2	cg14021899			0.0929884	0.000218	0.2818822

chr2	cg18518720			0.1971128	0.000218	0.2818822
chr2	cg14180680	Body		-0.1459479	0.0002183	0.2818822
chr10	cg05572410		GDI2	0.1835908	0.0002188	0.2818822
chr1	cg01945501	Body	LRRC7	-0.1439507	0.0002197	0.2818822
chr16	cg21017385			0.1835847	0.00022	0.2818822
chr7	cg08140389	Body	AC002480.3;STEAP1B;AC002480.5	-0.1426992	0.0002201	0.2818822
chr6	cg15922651			-0.1380856	0.0002201	0.2818822
chr11	cg18367488			0.2172089	0.0002203	0.2818822
chr7	cg12183789	Body;Body;Body;Body;Body		-0.139247	0.0002203	0.2818822
chr10	cg05267294	Body	GRID1;GRID1	-0.1701805	0.0002207	0.2818822
chr1	cg20920045	Body	FAM5C	-0.1578657	0.0002217	0.2818822
chr4	cg26162435			-0.1704067	0.000222	0.2818822
chr1	cg02660895	Body;Body;Body	RP4-794H19.1;RP4-794H19.1;RP4-794H19.2;RP4-794H19.2	-0.134412	0.0002224	0.2818822
chr1	cg12445832			0.1482123	0.0002226	0.2818822
chr13	cg07832591			-0.2092937	0.0002226	0.2818822
chr3	cg20815919	Body;Body;Body	MGLL;MGLL;MGLL;MGLL	0.0956565	0.0002233	0.282248
chr6	cg10148702	Body;Body;Body;Body	PHACTR2;PHACTR2	-0.1838219	0.0002244	0.2824255
chr16	cg10633128	Body	COTL1	0.112889	0.0002244	0.2824255
chr21	cg03232933	TSS1500;TSS1500	BTG3;BTG3;BTG3;BTG3	0.1709835	0.0002251	0.2824255
chr18	cg06856216	Body		0.1806946	0.0002255	0.2824255
chr3	cg07488574	Body	LIPH;LIPH	-0.1319086	0.0002258	0.2824255
chr5	cg21256257	5'UTR;5'UTR;TSS1500;TSS1500;TSS1500;TSS1500	RNF14;RNF14;RNF14;RNF14;RNF14;RNF14;RNF14;RNF14;RNF14;RNF14;RNF14;RNF14;RNF14;RNF14;RNF14;RNF14;PCDH12;RNF14;RNF14	0.2864321	0.0002267	0.2824255
chr6	cg10250828	Body;Body;Body;Body;Body;Body	KCNQ5-IT1	-0.1521718	0.0002272	0.2824255

chr5	cg19218072	Body;Body		-0.1741707	0.0002274	0.2824255
chr20	cg17409313	Body	GDAP1L1;GDAP1L1	0.151584	0.0002281	0.2824255
chr11	cg11845417	1stExon;5'UTR	C11orf52;HSPB2-C11orf52;C11orf52;C11orf52;C11orf52;CRYAB	0.133327	0.0002283	0.2824255
chr8	cg07529715	TSS1500	CTHRC1;CTHRC1;CTHRC1	0.2154825	0.0002283	0.2824255
chr1	cg24666085	5'UTR;5'UTR	PAPPA2	-0.2059996	0.0002289	0.2824255
chr16	cg04223227			0.1539372	0.0002295	0.2824255
chr22	cg27281093	TSS1500	USP18	0.2145304	0.0002295	0.2824255
chr3	cg22897419			-0.1565251	0.0002297	0.2824255
chr15	cg02965553	Body		0.1059332	0.0002297	0.2824255
chr5	cg24558747	Body	ERGIC1;ERGIC1;ERGIC1;ERGIC1	-0.1796487	0.0002319	0.2846499
chr7	cg24014202	Body;Body	DLX6-AS2;DLX6;DLX6;DLX6;DLX6-AS1;DLX6-AS1;DLX6-AS1;DLX6-AS1;DLX6-AS1;DLX6-AS1	0.1703905	0.0002326	0.2848428
chr9	cg02628787	Body	QSOX2	0.1180652	0.000233	0.2848428
chr9	cg05928239	TSS1500	FOXE1	0.1701476	0.0002336	0.2848428
chr2	cg08017142			-0.1727921	0.0002337	0.2848428
chr9	cg13226907	TSS1500;TSS1500	CDC14B	0.1297507	0.0002359	0.2871315
chr19	cg26924223	TSS1500	ZNF563;ZNF563	0.1582924	0.0002377	0.2887016
chr1	cg10806711	Body;Body		0.1462912	0.000238	0.2887016
chr18	cg03974563			-0.1451649	0.0002385	0.2887704
chr3	cg07765814	TSS1500	ATP13A5;ATP13A5	-0.1953587	0.0002414	0.291835
chr1	cg18852096	Body;Body;Body;Body;Body;Body	MIB2;MIB2;MIB2;MIB2;MIB2;MIB2;MIB2;MIB2;MIB2	0.2764237	0.0002425	0.2921398
chr1	cg08223291	TSS200;5'UTR;Body;Body	RP11-10N16.3;RP11-10N16.3;GRHL3;GRHL3;GRHL3;GRHL3;GRHL3	0.1478334	0.0002429	0.2921398
chr18	cg05938218			-0.1254245	0.000243	0.2921398

chr6	cg03771840	3'UTR	TRIM15;TRIM15;TRIM15	0.2307426	0.0002433	0.2921398
chr7	cg07176561	Body;Body;Body		-0.1453384	0.0002438	0.2922999
chr5	cg16051727			-0.1421904	0.0002445	0.2926389
chr1	cg10555800			0.2137573	0.0002457	0.2935727
chr1	cg20678082			0.1908983	0.0002462	0.2936805
chr16	cg21695020	TSS200;TSS200	DPEP3;DPEP3;DPEP3	0.2015501	0.0002474	0.294463
chr16	cg24517604		LITAF	0.1988456	0.0002477	0.294463
chr19	cg25695172	Body;Body		-0.1568201	0.0002482	0.294607
chr8	cg19585816	TSS1500	ANK1	-0.1486846	0.0002493	0.2947861
chr22	cg16406315			0.0856349	0.0002495	0.2947861
chr17	cg02650908	Body;Body;Body	MGAT5B	0.1705259	0.0002497	0.2947861
chr2	cg23702716	Body	TTC21B;TTC21B;TTC21B	-0.1065475	0.00025	0.2947861
chr17	cg02880508	Body		0.1273338	0.0002506	0.2950225
chr6	cg20157127	Body		0.1474643	0.0002523	0.2955189
chr5	cg17944801		RN5S196;CTB-99A3.1	-0.1573619	0.0002525	0.2955189
chr15	cg04858164	Body;Body;Body ;Body	TCF12;TCF12	-0.1919262	0.0002526	0.2955189
chr9	cg21222556	Body;Body	FAM102A;FAM102A;FAM102A;FAM102 A	-0.1557952	0.0002529	0.2955189
chr1	cg15676040	TSS200;3'UTR;3' UTR;3'UTR;TSS 200;3'UTR	RUSC1- AS1;FDPS;FDPS;RUSC1;RUSC1;RUSC1- AS1;FDPS;FDPS;RUSC1-AS1;RUSC1-AS1	0.2129053	0.0002531	0.2955189
chr2	cg11942603	5'UTR	AC092431.1;ANXA4;ANXA4;ANXA4;AN XA4;ANXA4;ANXA4	0.1848363	0.0002542	0.2957316
chr11	cg01914428	Body	SHANK2	0.1282614	0.000255	0.2957316
chr4	cg03090145	Body	C4orf29;C4orf29	-0.2288333	0.0002554	0.2957316
chr6	cg07412562		RP11-307P5.1;RP11-307P5.1;RP11-307P5.1	-0.1657506	0.0002559	0.2957316
chr11	cg12829473		CTC-497E21.5	0.1248138	0.0002559	0.2957316
chr20	cg26334832	5'UTR	IFT52;IFT52	0.2130697	0.0002563	0.2957316
chr1	cg02457145			-0.209868	0.0002565	0.2957316

chr16	cg13767707	TSS200;TSS200;TSS200;Body;Body	SLC9A3R2;SLC9A3R2;SLC9A3R2	0.1469681	0.000257	0.2957316
chr6	cg03045620	TSS200	SLC44A4;SLC44A4;SLC44A4;SLC44A4	0.1463338	0.0002575	0.2957316
chr17	cg15266658			0.0870608	0.0002577	0.2957316
chr3	cg03699904	TSS1500	SLC2A2;SLC2A2	0.1501063	0.0002578	0.2957316
chr19	cg12104982	Body	SAFB2	0.3336726	0.0002586	0.2957316
chr2	cg24343835	TSS200	MIR216A;AC011306.2	-0.1948632	0.0002586	0.2957316
chr13	cg22674251			-0.130868	0.0002613	0.2970609
chr6	cg07357891	Body;Body		-0.1548101	0.0002616	0.2970609
chr15	cg11970192	Body;5'UTR	ST20;AC015871.2;RP11-38G5.1;RP11-38G5.1;C15orf37	0.3038925	0.0002617	0.2970609
chr2	cg10934870	Body		-0.2247429	0.0002623	0.2970609
chr2	cg12073779	TSS200	CRYGD	0.326442	0.0002628	0.2970609
chr18	cg00565113		ZBTB7C;ZBTB7C	-0.1673207	0.0002637	0.2970609
chr2	cg09110314			-0.1343168	0.000264	0.2970609
chr3	cg06303936	Body		-0.1298416	0.000265	0.2970609
chr1	cg01887689		FAM159A;FAM159A;FAM159A	0.1577828	0.0002655	0.2970609
chr14	cg07532405		TRAJ56;AE000661.37;AE000661.37	-0.1160948	0.0002656	0.2970609
chr11	cg11206788		RP11-448P19.1	-0.1356818	0.0002657	0.2970609
chr5	cg22955595	Body		-0.125007	0.000266	0.2970609
chr16	cg00885460	TSS1500	MMP15	0.1698699	0.0002664	0.2970609
chr1	cg22695117			0.2141387	0.0002668	0.2970609
chr20	cg15277588			-0.174601	0.000267	0.2970609
chr18	cg10154487		U6	-0.1576609	0.0002674	0.2970609
chr22	cg20137410	Body	ZNRF3;ZNRF3	0.158919	0.000268	0.2970609
chr1	cg18936052	TSS1500;TSS1500;TSS1500;TSS1500;TSS1500	FAM76A;FAM76A;FAM76A;FAM76A	0.1200969	0.0002685	0.2970609
chr9	cg14478368			-0.1627046	0.0002685	0.2970609

chr15	cg01989456			0.1255211	0.0002688	0.2970609
chr8	cg08979870		RP11-600K15.1	-0.1929246	0.000269	0.2970609
chr7	cg16410508		RP11-797H7.5;ZNF273	0.1087172	0.0002692	0.2970609
chr17	cg17631924			0.1407276	0.0002695	0.2970609
chr4	cg25555847			-0.1504661	0.0002707	0.2970609
chr3	cg01976224	5'UTR;5'UTR	FAM19A4;FAM19A4	-0.3031049	0.0002709	0.2970609
chr5	cg14158827	Body;Body;Body;Body;Body;Body	ACSL6;ACSL6	0.087501	0.000271	0.2970609
chr1	cg15756091	Body	RGSL1;RGSL1;RGSL1	-0.161031	0.0002715	0.2970609
chr4	cg03250742	Body;Body;Body	SPON2;SPON2;SPON2;SPON2;SPON2;SPON2	0.1631596	0.0002719	0.2970609
chr6	cg24515946			-0.1899695	0.0002719	0.2970609
chr6	cg00498529			-0.1338048	0.0002722	0.2970609
chr21	cg26395115			-0.1339976	0.0002736	0.2972463
chr5	cg04458670	TSS1500	KIAA0947;KIAA0947;KIAA0947	0.2190552	0.0002737	0.2972463
chr17	cg19850503	Body;Body;Body	ABCA8	-0.1352188	0.0002738	0.2972463
chr6	cg16251647	1stExon;5'UTR	SLC17A1;SLC17A1;SLC17A1;SLC17A1;SLC17A1;SLC17A1	-0.1409811	0.000274	0.2972463
chr7	cg04339041	Body	ADCY1	-0.1286915	0.0002745	0.2972922
chr13	cg15190788			-0.1291365	0.0002752	0.297641
chr4	cg13859764			0.3126053	0.0002758	0.2978429
chr15	cg23689615	Body		0.1152465	0.0002777	0.2978429
chr19	cg02187276			-0.1203408	0.000278	0.2978429
chr8	cg00575501		CTD-3023L14.1;CTD-3023L14.3;CTD-3023L14.2;CTD-3023L14.2	-0.1345099	0.0002785	0.2978429
chr9	cg13993274	TSS1500	AK3;AK3;AK3	0.1538269	0.0002792	0.2978429
chr7	cg22584127		AC073348.1	-0.1182044	0.0002799	0.2978429
chr13	cg25918510			-0.424506	0.00028	0.2978429
chr11	cg24599329		RP11-945A11.1;RP11-945A11.2	-0.2277327	0.0002809	0.2978429

chr9	cg27625732	TSS1500	TBC1D13;TBC1D13;TBC1D13;TBC1D13;TBC1D13	0.1311872	0.0002815	0.2978429
chr1	cg27539046			0.354771	0.0002823	0.2978429
chr2	cg03958364	Body		-0.1321304	0.0002824	0.2978429
chr10	cg05132851			-0.1741361	0.0002828	0.2978429
chr3	cg00517117	Body	TBL1XR1-AS1	-0.1602352	0.000283	0.2978429
chr5	cg23743252	5'UTR;5'UTR;TSS1500;TSS200;TSS200;TSS200	RNF14;RNF14;RNF14;RNF14;RNF14;RNF14;RNF14;RNF14;RNF14;RNF14;RNF14;RNF14;RNF14;RNF14;RNF14;RNF14;PCDH12;RNF14;RNF14	0.2716451	0.0002831	0.2978429
chr19	cg12463140	TSS1500	GLT25D1;GLT25D1	-0.2652343	0.0002832	0.2978429
chr9	cg21389750	Body	CNTRL;CNTRL	0.1530005	0.0002832	0.2978429
chr19	cg09137630	TSS1500	ZC3H4	0.1589775	0.0002833	0.2978429
chr11	cg15777890	Body	OPCML	-0.1679513	0.0002836	0.2978429
chr16	cg07405121	Body;Body	SLC9A3R2;SLC9A3R2;SLC9A3R2	0.1481114	0.0002836	0.2978429
chr21	cg07945323	TSS1500;TSS1500	KRTAP6-2;KRTAP22-1	-0.1278278	0.0002837	0.2978429
chr11	cg02897707			0.1909998	0.0002844	0.297862
chr7	cg16921950	TSS1500;TSS1500	GBAS;GBAS;GBAS;GBAS;GBAS;GBAS;GBAS	0.1535564	0.0002845	0.297862
chr12	cg24587879	TSS1500;TSS1500;5'UTR	USP44;USP44;USP44;USP44;USP44;USP44	0.2251522	0.0002855	0.2979684
chr1	cg21191743	Body;Body;Body;Body;5'UTR	VTCN1;VTCN1;VTCN1	0.0925879	0.0002863	0.2979684
chr7	cg10475014	Body;Body;Body;Body	SRRT;SRRT;SRRT;SRRT;SRRT;SRRT;SRRT;SRRT;SRRT	0.3788815	0.0002865	0.2979684
chr4	cg03443943			-0.17627	0.000287	0.2979684
chr1	cg00492341	Body	MMEL1	-0.1707048	0.0002873	0.2979684
chr6	cg01031101	TSS200	NKAPL;ZKSCAN4	0.4496277	0.0002874	0.2979684
chr11	cg05377527	TSS1500;TSS1500	NDUFS3;PTPMT1;PTPMT1;PTPMT1;PTPMT1;PTPMT1;CELF1;CELF1;CELF1	0.1054946	0.0002893	0.2979684

chr4	ch.4.12604R	Body;Body	PIGG;PIGG;PIGG;PIGG;PIGG;PIGG;PIGG	-0.531554	0.0002895	0.2979684
chr13	cg15338967			0.115293	0.0002896	0.2979684
chr3	cg17781418	5'UTR;5'UTR;5'UTR;5'UTR;5'UTR;5'UTR	FOXP1;FOXP1;FOXP1;FOXP1;FOXP1;FOXP1;FOXP1	-0.2113438	0.0002898	0.2979684
chr17	cg12309114	Body	TOP3A;TOP3A;TOP3A;TOP3A	-0.1326912	0.0002903	0.2979684
chr2	cg06621309	TSS200;5'UTR	HPCAL1;HPCAL1;HPCAL1	0.5696959	0.0002908	0.2979684
chr2	cg12717533	Body		0.0907804	0.0002914	0.2979684
chr9	cg17927434	Body;Body		0.1232934	0.0002917	0.2979684
chr7	cg17498476	Body;Body;Body		-0.1594643	0.0002919	0.2979684
chr19	cg27110790			0.2038986	0.0002923	0.2979684
chr5	cg05183211	Body	SFXN1	-0.1544808	0.0002927	0.2979684
chr8	cg05106778	Body	SDC2;SDC2;SDC2;SDC2	-0.1514583	0.0002927	0.2979684
chr6	cg02281167	Body	TRIM15	0.1120526	0.0002928	0.2979684
chr8	cg06657841		RP11-480O10.1	-0.1218991	0.000293	0.2979684
chr1	cg16263722	Body;Body	MECR;MECR;MECR;MECR;MECR	0.161145	0.0002941	0.2979684
chr7	cg03060590			0.6514904	0.0002941	0.2979684
chr2	cg10125918	Body;Body;Body	HADHB	-0.2199804	0.0002955	0.2979684
chr12	cg15767529	TSS1500;TSS1500;Body	ITGA7;ITGA7;ITGA7;ITGA7;ITGA7;ITGA7;ITGA7	0.1108382	0.0002959	0.2979684
chr7	cg12423387	Body	MKLN1;MKLN1	-0.1271967	0.0002968	0.2979684
chr22	cg27426267	5'UTR;5'UTR	FAM118A;FAM118A;FAM118A;FAM118A;FAM118A;FAM118A;FAM118A	0.1011307	0.0002972	0.2979684
chr6	cg11427329	TSS1500;TSS1500;TSS1500;Body;Body;Body	SLC29A1;SLC29A1;SLC29A1;SLC29A1;SLC29A1;SLC29A1	0.1079104	0.0002972	0.2979684
chr1	cg04165922	Body;Body		-0.2033128	0.000298	0.2979684
chr17	cg05220510	5'UTR;5'UTR	CLEC10A;CLEC10A;CLEC10A;CLEC10A;CLEC10A	-0.1446657	0.0002984	0.2979684
chr5	cg27628536		CTD-2201E18.3;CTD-2201E18.3;CTD-2035E11.3;CTD-2035E11.3;CTD-	0.18901	0.0002987	0.2979684

			2201E18.3;CTD-2201E18.3;CTD-2201E18.3			
chr6	cg02081431	Body		0.0837853	0.0003013	0.2979684
chr17	cg17110400	TSS1500	WNK4	0.1333144	0.0003014	0.2979684
chr2	cg08334830	Body		0.1518554	0.0003019	0.2979684
chr11	cg15755673		AP002884.2;SDHD	-0.1282187	0.0003024	0.2979684
chr17	cg16435459		CRK	0.1148062	0.0003025	0.2979684
chr3	cg02315509	TSS1500;Body;Body	ZBED2	-0.1425492	0.0003029	0.2979684
chr11	cg09170311	Body;Body	C11orf88;RP11-794P6.6	0.1401554	0.000303	0.2979684
chr10	cg13062960	Body	RP11-507P23.3;RP11-507P23.3;RP11-507P23.3;RP11-507P23.3	0.1763919	0.0003033	0.2979684
chr12	cg22794803	Body;Body;Body	TPCN1;TPCN1	0.1166867	0.0003033	0.2979684
chr15	cg04672210	TSS200	FAM169B	-0.1197999	0.0003035	0.2979684
chr13	cg16479957	Body	MTUS2	0.1401381	0.0003035	0.2979684
chr3	cg26491882	Body;Body;Body	ITPR1;ITPR1	-0.1641312	0.0003035	0.2979684
chr1	cg09126636	Body;Body		0.1441105	0.0003035	0.2979684
chr5	cg04346128	5'UTR	KCTD16;KCTD16	0.1298563	0.0003035	0.2979684
chr6	cg25247859			0.1910337	0.0003039	0.2979684
chr14	cg00726151			-0.1715051	0.0003039	0.2979684
chr6	cg13540225			0.1499863	0.0003042	0.2979684
chr3	cg01650347	TSS1500;Body;Body;Body;Body;Body;Body;5'UTR	C3orf65;C3orf65;C3orf65;IGF2BP2;IGF2BP2;IGF2BP2	-0.1597316	0.0003045	0.2979684
chr14	cg14871588			0.1411241	0.0003049	0.2979727
chr17	cg08554115	1stExon;3'UTR	NLRP1;NLRP1;NLRP1;NLRP1	0.3216246	0.0003057	0.2981604
chr16	cg04715503	1stExon;5'UTR	CLDN9;CLDN6;CLDN9	0.5193013	0.000306	0.2981604
chr3	cg08046836	Body	RP11-91K9.1;RP11-91K9.1	-0.1554695	0.0003068	0.2981604
chr12	cg12784704	5'UTR;5'UTR;5'UTR;TSS200;Body	LMO3;LMO3;LMO3;LMO3;LMO3;LMO3;LMO3;LMO3;LMO3;LMO3;LMO3;LMO3;	0.1555675	0.000307	0.2981604

		;Body;Body;Body;5'UTR;5'UTR	LMO3;LMO3;LMO3;LMO3;LMO3;LMO3;LMO3;LMO3;LMO3;LMO3;LMO3;LMO3;LMO3;LMO3;LMO3;LMO3;LMO3;LMO3;LMO3;LMO3;MGST1			
chr8	cg23494863	TSS1500;TSS1500	STAR;STAR;STAR;RP11-90P5.2	0.1191901	0.0003072	0.2981604
chr3	cg18709144	Body	PVRL3;PVRL3;PVRL3;PVRL3	-0.1180993	0.0003095	0.2995014
chr8	cg12992268		RP11-697M17.1;RP11-697M17.1;RP11-697M17.1;RP11-697M17.1	-0.1645047	0.0003096	0.2995014
chr18	cg17571306			-0.1422069	0.0003098	0.2995014
chr9	cg18655410	5'UTR;5'UTR;5'UTR;5'UTR;5'UTR;5'UTR;5'UTR	GSN;GSN;GSN;GSN;GSN;GSN;GSN;RP11-477J21.6;GSN;GSN	0.1984152	0.0003104	0.2995542
chr7	cg04194173	Body;Body;Body	C7orf50	-0.1411998	0.0003111	0.2995542
chr12	cg18054492	TSS1500	METTL20	0.2789845	0.0003117	0.2995542
chr12	cg17495529	TSS200	KRT85	-0.2204457	0.000312	0.2995542
chr18	cg05533549			-0.1275952	0.0003123	0.2995542
chr8	cg03860720	Body;Body	LRP12;LRP12;LRP12	-0.1435188	0.0003128	0.2995542
chr6	cg26393036			0.1711009	0.0003131	0.2995542
chr12	cg04361103	TSS1500;TSS1500;TSS1500	HVCN1;HVCN1;HVCN1;HVCN1;HVCN1;HVCN1;HVCN1	0.1091759	0.0003132	0.2995542
chr6	cg01192291	TSS1500	ZKSCAN4;ZKSCAN4;ZKSCAN4	-0.2041642	0.0003141	0.3000599
chr15	cg17214263	Body;Body;Body;Body;Body	PCSK6;PCSK6;PCSK6;PCSK6;PCSK6;PCSK6	-0.1556253	0.000315	0.3004288
chr2	cg22079269	5'UTR	HDAC4;HDAC4;HDAC4;HDAC4	0.1709389	0.0003171	0.3013386
chr6	cg16170767			0.1929867	0.0003171	0.3013386
chr11	cg04046848			0.1981232	0.0003173	0.3013386
chr3	cg17941143			0.156554	0.0003177	0.3013386
chr4	cg22805341	5'UTR;Body;Body;Body;Body	LIMCH1;LIMCH1;LIMCH1;LIMCH1;LIMCH1;LIMCH1;LIMCH1;LIMCH1;LIMCH1	-0.1478172	0.000318	0.3013386
chr1	cg07999953	5'UTR;5'UTR	GJB3;GJB3;GJB3;RP1-34M23.5	-0.1466227	0.0003194	0.3021703

chr7	cg07332511	Body;Body;Body	TMEM130	-0.1459678	0.0003197	0.3021703
chr3	cg17219740	Body;3'UTR	GRM7	-0.1462978	0.0003226	0.3045302
chr17	cg07429804			-0.1521577	0.0003233	0.3046024
chr11	cg12703652	Body;Body;Body;Body;Body	DLG2;DLG2	-0.1749795	0.0003236	0.3046024
chr1	cg11788604	Body;Body	AK5;AK5	-0.1854199	0.0003243	0.3046024
chr5	cg05670219	Body		-0.1489845	0.0003247	0.3046024
chr2	cg09128059		PER2	-0.1348744	0.0003248	0.3046024
chr20	cg06794913			0.1716404	0.0003259	0.3048126
chr11	cg00084864			0.0878477	0.0003259	0.3048126
chr2	cg06689758			0.1273416	0.0003265	0.3049425
chr20	cg01756827			0.1733691	0.0003269	0.3049845
chr8	cg09164611			0.1588278	0.000328	0.3053243
chr17	cg07042338	5'UTR;TSS1500	SDF2;SUPT6H;SDF2;SDF2;SDF2	-0.1576937	0.0003285	0.3053243
chr15	cg16362027			-0.175506	0.0003293	0.3053243
chr6	cg22681148	3'UTR	UBR2	-0.1451934	0.0003299	0.3053243
chr11	cg17032689			0.1044773	0.0003307	0.3053243
chr5	cg09006366	Body;Body;Body;Body;Body;Body;Body;Body;Body	RP11-65F13.2;RP11-65F13.2;RP11-65F13.2;RP11-65F13.2;RP11-65F13.2	-0.1941719	0.0003311	0.3053243
chr15	cg25837909	Body;Body	APH1B;APH1B	-0.2148724	0.0003311	0.3053243
chr17	cg15804598	TSS1500	HEXIM1	0.0796689	0.0003313	0.3053243
chr5	cg18466162			-0.1847824	0.0003314	0.3053243
chr19	cg23357789	3'UTR	PRTN3;PRTN3	-0.162767	0.0003316	0.3053243
chr17	cg18733548		CBX8	0.1708981	0.000332	0.3053243
chr9	cg02296661	Body;Body;Body;Body	GDA	-0.1335522	0.000334	0.3063152
chr1	cg20632567		MDS2	0.0805604	0.0003342	0.3063152

chr2	cg27016609	5'UTR;TSS1500	STON1-GTF2A1L;STON1-GTF2A1L;STON1-GTF2A1L;STON1;STON1-GTF2A1L;STON1;STON1	0.1161497	0.0003349	0.3063152
chr19	cg09991298	TSS1500;1stExon;5'UTR	ANKRD27;RGS9BP;RGS9BP	0.1562546	0.0003351	0.3063152
chr5	cg16449581		RP11-321E2.4;RP11-321E2.4;RP11-321E2.4	-0.2024747	0.0003352	0.3063152
chr16	cg26513180	TSS200;TSS200	FANCA;FANCA;FANCA;FANCA;FANCA;SPIRE2;SPIRE2;FANCA;FANCA;FANCA;FANCA	0.6230788	0.0003356	0.3063547
chr18	cg07058998	TSS200;5'UTR	CCDC68;CCDC68	-0.1460712	0.0003377	0.3065434
chr1	cg03984347	Body	C1orf228;C1orf228;RP4-678E16.4;RP4-678E16.4;C1orf228	0.2711897	0.0003378	0.3065434
chr12	cg10640894	Body		-0.1566686	0.0003381	0.3065434
chr20	cg12471376			0.129387	0.0003382	0.3065434
chr17	cg20122123			0.082093	0.0003382	0.3065434
chr10	cg03379552	Body		0.3695441	0.0003384	0.3065434
chr10	cg11445554			-0.1418107	0.000339	0.3067071
chr17	cg10805039	Body	NXN;NXN;NXN;NXN;NXN	0.2605176	0.0003407	0.3075574
chr2	cg23759710	1stExon	OXER1	0.1055001	0.0003412	0.3075574
chr1	cg09255383			0.2799985	0.0003417	0.3075574
chr6	cg10577793			-0.1242307	0.0003419	0.3075574
chr14	cg06920962	TSS1500;TSS1500	PLD4;PLD4;PLD4;PLD4	0.0936882	0.0003421	0.3075574
chr22	cg03805475		GRAMD4;GRAMD4	0.2137909	0.0003428	0.3078503
chr12	cg10769507	Body	PIK3C2G	-0.1303707	0.0003441	0.3081079
chr3	cg26104910	Body;Body	PTH1R	-0.2025742	0.0003443	0.3081079
chr6	cg17034390			0.310652	0.0003446	0.3081079
chr13	cg18832428	TSS1500	RP11-74A12.2	-0.1502963	0.0003453	0.3081079
chr19	cg19773296			-0.1190364	0.0003453	0.3081079

chr16	cg08196561	TSS200	RP11-482M8.1;ZCCHC14;RP11-482M8.1;ZCCHC14;ZCCHC14	0.381346	0.0003459	0.3081893
chr15	cg09131617	3'UTR	TLN2;RP11-1069G10.1;RP11-1069G10.1;RP11-1069G10.1	-0.1519819	0.0003468	0.3081893
chr20	cg04645556	Body;Body	PLCB1	-0.1498509	0.000347	0.3081893
chr8	cg25302050	Body	PLEKHA2;PLEKHA2;PLEKHA2;PLEKHA2	-0.1217436	0.0003475	0.3081893
chr1	cg01259156	5'UTR	RP11-510C10.2;LHX8	0.2116598	0.0003487	0.3081893
chr12	cg03691549	5'UTR;1stExon;5'UTR;Body	TENC1;TENC1;TENC1;TENC1;TENC1;TENC1;RP11-983P16.4;RP11-983P16.4;TENC1;TENC1;TENC1;TENC1	0.1005287	0.0003488	0.3081893
chr14	cg25293251	TSS1500	GOLGA5;GOLGA5;GOLGA5	0.1630287	0.000349	0.3081893
chr1	cg17591816	Body;Body		-0.1434655	0.000349	0.3081893
chr2	cg09459451	Body	DHX57;DHX57	-0.1603127	0.0003492	0.3081893
chr17	cg17118948	Body;Body;Body	CACNB1;CACNB1	-0.1593837	0.0003505	0.3088327
chr14	cg11260662	Body;Body	TDP1;TDP1;TDP1	-0.1946415	0.0003516	0.3088327
chr6	cg19059495			0.1863711	0.0003525	0.3088327
chr10	cg16241033	Body	FRMD4A;FRMD4A	0.3204526	0.0003535	0.3088327
chr4	cg11786320			0.2579645	0.0003537	0.3088327
chr5	cg22804222			-0.2133778	0.0003545	0.3088327
chr5	cg06465076	Body;Body;Body;Body;Body	CAST;CAST;CAST	-0.1662812	0.0003549	0.3088327
chr10	cg19237150			-0.1949399	0.0003549	0.3088327
chr1	cg00178562		CROCCP2;CROCCP2	-0.1448867	0.0003557	0.3088327
chr7	cg07703358			0.3046874	0.0003559	0.3088327
chr19	cg19380072	Body;Body	BCKDHA;BCKDHA	0.1215527	0.0003561	0.3088327
chr1	cg15583642			0.1022898	0.0003563	0.3088327
chr11	cg05083507	TSS200;TSS200	CCDC81;CCDC81;CCDC81	0.1975955	0.0003565	0.3088327
chr10	cg25900192	Body		-0.2076724	0.000357	0.3088327
chr17	cg22968401	5'UTR;1stExon	PIPOX;PIPOX;PIPOX;PIPOX	0.1236965	0.0003571	0.3088327
chr14	cg24765602	TSS1500	HSPA2;HSPA2;HSPA2;RP11-973N13.4	0.1575412	0.0003572	0.3088327

chr3	cg00446046		RP11-132N15.3	-0.22377	0.0003573	0.3088327
chr5	cg14869043		CTD-2113L7.1	-0.1441998	0.0003607	0.310544
chr1	cg17222682		LPHN2;LPHN2;LPHN2	-0.123498	0.0003608	0.310544
chr16	cg17710952	ExonBnd;ExonBnd;ExonBnd;Body;Body;Body	ATP2A1;ATP2A1	0.143624	0.0003621	0.310544
chr16	cg01954600			-0.2230894	0.0003623	0.310544
chr8	cg19997384	Body		0.2180316	0.0003625	0.310544
chr11	cg22960962			-0.1277269	0.0003626	0.310544
chr5	cg16935794	Body;Body;Body	MRPS27;MRPS27;MRPS27	-0.147269	0.0003629	0.310544
chr8	cg25422895		HMBOX1	-0.1354481	0.0003632	0.310544
chr22	cg01493106	TSS1500;TSS1500	SCARF2;SCARF2	0.0786708	0.0003633	0.310544
chr4	cg10533057	Body		-0.1612292	0.0003641	0.310544
chr2	cg00861295	TSS1500	AC018682.6;AC018682.6	-0.110297	0.0003644	0.310544
chr11	cg16734913	1stExon	OR5W2	-0.3621717	0.0003651	0.310544
chr6	cg05509894	TSS1500	C6orf163;C6orf163	-0.1572128	0.0003652	0.310544
chr1	cg19500415	Body		-0.1518348	0.0003653	0.310544
chr1	cg12629991	Body	PBX1;PBX1;PBX1;PBX1;PBX1;PBX1;PBX1	-0.1363104	0.0003666	0.3113244
chr10	cg14529070			0.1124587	0.0003671	0.3113244
chr6	cg18055610			0.2415253	0.0003685	0.3113244
chr11	cg19085165		RP11-148I19.1;RP11-148I19.1	-0.2800765	0.0003689	0.3113244
chr12	cg03028611			0.1280701	0.0003692	0.3113244
chr2	cg26491213	TSS1500	AC012354.5;AC012354.5;SIX3	0.2219094	0.0003699	0.3113244
chr2	cg26277248	Body	GLI2;GLI2;GLI2;GLI2	-0.1783197	0.00037	0.3113244
chr7	cg10207609	TSS1500;TSS1500;5'UTR	CD36;CD36;CD36;CD36;CD36;CD36;CD36;CD36;CD36;CD36;CD36	-0.1623296	0.0003703	0.3113244
chr16	cg00564657			0.1065288	0.0003714	0.3113244
chr19	cg20098710	Body	NDUFA13;YJEFN3	0.1905733	0.0003717	0.3113244

chr1	cg00849335	Body;Body;Body;Body;Body		-0.2023097	0.0003727	0.3113244
chr8	cg19168966	Body		-0.1572058	0.0003729	0.3113244
chr1	cg22012583	TSS1500;TSS1500	CERS2;CERS2;CERS2;CERS2;CERS2;CERS2;RP11-316M1.3	0.2853544	0.0003735	0.3113244
chr14	cg08075719		CTD-2555C10.3	0.14182	0.0003746	0.3113244
chr2	cg26106807			0.0841197	0.0003749	0.3113244
chr14	cg21235823	TSS1500	GOLGA5;GOLGA5;GOLGA5	0.188015	0.0003752	0.3113244
chr17	cg14061051	Body	FXR2;MPDU1	0.1197421	0.0003753	0.3113244
chr15	cg27071234	Body		-0.1511299	0.0003761	0.3113244
chr9	cg13862900	Body;Body;Body	SDCCAG3;SDCCAG3;SDCCAG3;SDCCAG3	-0.1484574	0.0003767	0.3113244
chr4	cg05622862	Body;Body;Body		0.3222331	0.0003769	0.3113244
chr16	cg22828022	TSS200;TSS200	DPEP3;DPEP3;DPEP3	0.1510972	0.0003776	0.3113244
chr5	cg25785733	Body;Body;Body;Body;Body;Body;Body;Body;Body;Body	PPP2R2B;PPP2R2B;PPP2R2B;PPP2R2B;PPP2R2B;PPP2R2B;PPP2R2B;PPP2R2B;PPP2R2B;PPP2R2B;PPP2R2B	-0.1255715	0.000378	0.3113244
chr17	cg27469152	3'UTR	EPX	0.1493964	0.0003792	0.3113244
chr6	cg18528196	Body	U6	-0.1338762	0.0003793	0.3113244
chr17	cg14929207	TSS1500	DHRS13;DHRS13	0.2193866	0.0003795	0.3113244
chr22	cg18512769	5'UTR;5'UTR;5'UTR;5'UTR	PPP6R2;PPP6R2;PPP6R2;PPP6R2	0.1573791	0.0003797	0.3113244
chr12	cg05898280	Body		-0.1225067	0.00038	0.3113244
chr14	cg12820006	TSS1500;TSS1500;TSS1500	MIR1197;MIR323A;MIR758	-0.1425884	0.0003801	0.3113244
chr21	cg17164249			-0.1097499	0.0003806	0.3113244
chr6	cg06392470	Body;Body;Body;Body	MAP3K7	-0.1259905	0.0003811	0.3113244
chr8	cg14475527	Body	HGSNAT;HGSNAT	-0.1611654	0.0003817	0.3113244

chr14	cg24265217	5'UTR;5'UTR;5'UTR;Body;5'UTR;5'UTR;Body	RP11-363J20.1;RP11-363J20.1;RP11-363J20.1;EXD2;EXD2;EXD2;EXD2;EXD2;EXD2;EXD2;EXD2;EXD2;EXD2;EXD2	0.1517163	0.0003827	0.3113244
chr10	cg07347315	Body	PDLIM1;PDLIM1	-0.2323903	0.0003828	0.3113244
chr8	cg01764822	Body;Body	TRAPPC9;TRAPPC9;TRAPPC9;TRAPPC9;TRAPPC9;TRAPPC9	-0.1389669	0.000383	0.3113244
chr6	cg18735214	TSS1500	CRIP3;CRIP3;CRIP3	0.1692013	0.0003833	0.3113244
chr14	cg07914111	Body		-0.1805167	0.0003835	0.3113244
chr1	cg10567964	Body	KLHDC8A;KLHDC8A	0.1159264	0.0003837	0.3113244
chr10	cg18932687			0.1160527	0.0003842	0.3113244
chr16	cg24921262	Body	SMPD3;SMPD3;SMPD3;SMPD3;SMPD3;SMPD3;SMPD3	0.1075122	0.0003843	0.3113244
chr5	cg14002042			-0.144058	0.0003849	0.3113244
chr2	cg18561265	Body	CYP1B1-AS1	-0.1685548	0.0003849	0.3113244
chr2	cg20518572			0.3253117	0.000385	0.3113244
chr1	cg20147290	ExonBnd;ExonBnd;Body;Body	NUP210L;NUP210L	0.1099121	0.000385	0.3113244
chr6	cg10049251		PI16	0.1052999	0.0003866	0.3113244
chr6	cg06206989	Body;Body;Body		-0.1263612	0.0003869	0.3113244
chr11	cg02663352	Body;Body;Body;Body;Body	CD44	-0.1625056	0.0003874	0.3113244
chr1	cg22040874			-0.1508313	0.0003877	0.3113244
chr14	cg18885766	Body	CCDC85C	-0.1465206	0.0003877	0.3113244
chr17	cg15119467			-0.1423914	0.0003877	0.3113244
chr8	cg16898283	Body	RP11-956J14.1;C8orf84	-0.155225	0.0003879	0.3113244
chr2	cg24221559			-0.1602418	0.0003883	0.3113244
chr5	cg10190863		CTB-35F21.1	-0.3904318	0.0003896	0.3120095
chr7	cg11086566	Body;Body;Body;Body	ASIC3;ASIC3;ASIC3;ASIC3	0.1463536	0.0003901	0.3120943
chr19	cg20285546			0.1936481	0.0003911	0.3124644
chr10	cg11892080		RP11-336A10.4;RP11-336A10.4	-0.1281142	0.0003924	0.3124644

chr9	cg22963015			-0.14804	0.0003924	0.3124644
chr5	cg13657884		CTD-2288O8.1	-0.1251796	0.0003932	0.3124644
chr18	cg24595611	Body;5'UTR;5'UTR	DLGAP1	0.0965192	0.0003939	0.3124644
chr2	cg05488527	5'UTR;5'UTR	U6;SLC39A10;SLC39A10;SLC39A10;SLC39A10;SLC39A10;SLC39A10;SLC39A10;SLC39A10	-0.1293194	0.000394	0.3124644
chr15	cg02907319	Body	FAM189A1	-0.1328511	0.000395	0.3124644
chr10	cg11180667			-0.1824543	0.0003955	0.3124644
chr10	cg22635041			-0.1499793	0.0003956	0.3124644
chr15	cg03780875	TSS1500;TSS1500	IDH2;IDH2;IDH2;CTD-2315E11.1	0.1596538	0.0003965	0.3124644
chr3	cg12487205			-0.1423593	0.0003966	0.3124644
chr6	cg11522683		RP1-153P14.8	-0.2076595	0.0003966	0.3124644
chr16	cg06004232		RMI2;RMI2	0.1041541	0.0003969	0.3124644
chr22	cg14943837	Body;Body	TBC1D10A;TBC1D10A;MTMR3	0.1342887	0.000397	0.3124644
chr4	cg08409465	TSS200;Body;Body	FGF5;FGF5	0.2350221	0.0003971	0.3124644
chr12	cg26079959	Body	SLC15A4;SLC15A4;SLC15A4;SLC15A4	-0.2785296	0.0003977	0.3125814
chr20	cg07589968	TSS200	KCNS1;KCNS1	0.0853721	0.0003986	0.3129985
chr5	cg03290223	Body	CTD-2247C11.3;CTD-2247C11.3	-0.1840252	0.0003992	0.3130212
chr10	cg15881077			0.1738424	0.0003995	0.3130212
chr2	cg16215960	Body;Body		-0.1440714	0.0004015	0.3136378
chr16	cg21644701	TSS1500;5'UTR;5'UTR;Body	FAM65A;FAM65A;FAM65A;FAM65A;FAM65A;FAM65A;FAM65A;FAM65A;FAM65A	0.0991046	0.0004021	0.3136378
chr7	cg02473195	TSS1500	TWISTNB	0.1991876	0.0004029	0.3136378
chr9	cg27585268			-0.1283111	0.0004029	0.3136378
chr6	cg22178280		RP11-73O6.4	0.2848595	0.0004032	0.3136378
chr18	cg24468939	Body	CDH2	-0.1301143	0.0004032	0.3136378
chr4	cg27338840			-0.2044889	0.000404	0.3136378

chr10	cg22696426			-0.1645402	0.000404	0.3136378
chr13	cg22411536	TSS1500;TSS1500;Body	KLHL1;KLHL1;ATXN8OS;ATXN8OS	-0.1657017	0.0004044	0.3136378
chr7	cg24501036			0.1011196	0.0004054	0.3136378
chr20	cg10422845			0.1696968	0.0004054	0.3136378
chr1	cg00852816			-0.1239419	0.0004055	0.3136378
chr6	cg08462030	Body;Body	RPS6KA2;RPS6KA2;RPS6KA2;RPS6KA2	-0.1539708	0.0004068	0.3142678
chr1	cg11310044	ExonBnd;ExonBnd;Body;Body	U6atac;PHACTR4;PHACTR4	-0.1179442	0.0004096	0.3148674
chr17	cg17042037	Body;Body;Body	INPP5K;INPP5K;INPP5K;INPP5K;INPP5K	0.2172058	0.0004098	0.3148674
chr3	cg05481881		RP11-373I8.1;CLRN1-AS1;RP11-166N6.3;RP11-166N6.2	-0.1467423	0.0004101	0.3148674
chr1	cg01770296	Body	MIA3	0.1691688	0.0004104	0.3148674
chr4	cg24358089	Body;Body	FAM190A;FAM190A	-0.5614074	0.0004105	0.3148674
chr16	cg11215870	Body;5'UTR;5'UTR	TGFB1I1;TGFB1I1;TGFB1I1;TGFB1I1;TGFB1I1;TGFB1I1;TGFB1I1;TGFB1I1;TGFB1I1;TGFB1I1;TGFB1I1;TGFB1I1;TGFB1I1;TGFB1I1;TGFB1I1	0.1648357	0.0004106	0.3148674
chr8	cg08600456	5'UTR	PAG1;PAG1	-0.1223356	0.0004106	0.3148674
chr8	cg23125737			-0.145729	0.000412	0.3154721
chr6	cg04439077			-0.1601597	0.0004123	0.3154721
chr2	cg13113121	5'UTR	AC011239.1;KLHL29	-0.2080683	0.0004137	0.3160856
chr14	cg00940972		TRAJ52	-0.1393595	0.0004141	0.3160856
chr5	cg18586212	Body;Body;Body;Body;Body;Body;Body	PPP2R2B;PPP2R2B;PPP2R2B;PPP2R2B;PPP2R2B;PPP2R2B;PPP2R2B;PPP2R2B;PPP2R2B;PPP2R2B;PPP2R2B	-0.1530888	0.0004147	0.3160856
chr6	cg07014126			-0.1684488	0.0004149	0.3160856
chr16	cg12358978	TSS1500	RP11-178L8.5;RP11-178L8.5;RP11-178L8.5;C16orf95	-0.1462844	0.0004157	0.3160856
chr10	cg17094269			0.0838346	0.0004158	0.3160856
chr11	cg12245876	Body		0.1276498	0.0004165	0.3160856

chr6	cg02486845	TSS1500;5'UTR;1stExon	AKIRIN2;AKIRIN2	0.1497696	0.0004174	0.3160856
chr19	cg21122437	5'UTR;5'UTR;5'UTR;5'UTR;5'UTR;TSS200	TEAD2;TEAD2;TEAD2	0.1691689	0.0004175	0.3160856
chr17	cg15246895	5'UTR	RAI1-AS1;RAI1	0.0978258	0.0004179	0.3160856
chr9	cg26417468	TSS1500;TSS1500;TSS200;3'UTR;3'UTR	MIR27B;MIR24-1;MIR23B;C9orf3;C9orf3;C9orf3;C9orf3;C9orf3;C9orf3	-0.1504979	0.0004184	0.3160856
chr8	cg04175248	5'UTR;5'UTR	AC108449.3;HMBOX1;HMBOX1;HMBOX1;HMBOX1;HMBOX1;HMBOX1;HMBOX1	-0.125529	0.0004184	0.3160856
chr8	cg07164659	TSS1500	BIN3;BIN3;BIN3;BIN3;BIN3;BIN3;BIN3	0.1479542	0.0004188	0.3160856
chr17	cg14593033	TSS200;TSS1500	MKS1;MKS1;MKS1;MKS1;MKS1	0.1555785	0.0004208	0.3172391
chr5	cg13214422	1stExon;1stExon;5'UTR;5'UTR	MYOZ3;MYOZ3;MYOZ3;MYOZ3	-0.1319177	0.0004213	0.3173271
chr1	cg25159046			0.140236	0.0004222	0.3176012
chr21	cg14626259	Body;Body	SIM2;SIM2	0.2051929	0.0004241	0.3176813
chr4	cg20562823			-0.1609275	0.000425	0.3176813
chr5	cg18484130			-0.1546376	0.0004252	0.3176813
chr20	cg10650353	Body;Body;Body;Body;Body;Body;Body	EIF6;RP4-614O4.11	0.1448698	0.0004255	0.3176813
chr2	cg19423804			-0.1791385	0.0004256	0.3176813
chr16	cg09620131			0.1179263	0.0004256	0.3176813
chr6	cg18844614	5'UTR	USP49;USP49;USP49	0.0838748	0.0004257	0.3176813
chr12	cg09447225			-0.1647536	0.0004258	0.3176813
chr12	cg01173104		U6	-0.2630008	0.0004264	0.3177758
chr8	cg26351746	Body;Body	RP11-152P17.2;RP11-152P17.2;RP11-152P17.2;RP11-152P17.2;RP11-152P17.2;RP11-152P17.2	0.3322848	0.0004275	0.3177758

chr15	cg26692263	TSS1500	ANKDD1A;ANKDD1A;ANKDD1A;ANKDD1A;AC069368.3	0.1467227	0.0004278	0.3177758
chr22	cg14703589	1stExon;1stExon;5'UTR;Body;5'UTR	LIMK2;LIMK2;LIMK2;LIMK2;LIMK2;LIMK2;LIMK2;LIMK2;LIMK2	0.1343551	0.0004281	0.3177758
chr4	cg09722400			-0.1237664	0.0004281	0.3177758
chr4	cg10537627		ANTXR2	-0.2011239	0.0004288	0.3178279
chr7	cg17705735	Body		-0.1308777	0.0004292	0.3178279
chr6	cg19705450	Body	ZNF311	-0.1211154	0.0004295	0.3178279
chr7	cg08563201		AC002066.1;CAV2;CAV2;CAV2;CAV2;CAV2;CAV2;CAV2;CAV2;CAV2;CAV2;CAV2;AC002066.1	-0.1495511	0.0004302	0.3178589
chr11	cg26181894			-0.1503292	0.0004306	0.3178589
chr19	cg13629807			-0.1486161	0.0004309	0.3178589
chr2	cg11967110			-0.1669794	0.0004314	0.3178772
chr2	cg15676528			-0.1373701	0.0004323	0.3182119
chr4	cg06743599	Body;TSS1500;TSS1500	RPL34;RPL34;RPL34;RPL34;RPL34;RPL34;RP11-462C24.1;RP11-462C24.1;RP11-462C24.1;RP11-462C24.1	0.1665922	0.000433	0.3184146
chr3	cg07056242	Body;Body	SLC6A6	-0.1233131	0.0004346	0.3185015
chr3	cg10360845	Body	RP11-197K6.1;RP11-197K6.1	-0.1626351	0.0004363	0.3185015
chr1	cg27230882			-0.1018734	0.0004363	0.3185015
chr20	cg21178389	Body		-0.2792396	0.000437	0.3185015
chr3	cg12410124		AC124944.5;AC124944.5	-0.158873	0.000437	0.3185015
chr2	cg24936411	5'UTR	CSRNP3;CSRNP3;CSRNP3;CSRNP3	-0.1425872	0.0004374	0.3185015
chr16	cg10282491	TSS200	CLDN9;CLDN6	0.206876	0.0004386	0.3185015
chr15	cg10425861			0.1846653	0.0004387	0.3185015
chr10	cg26483332	3'UTR	COMTD1;COMTD1;COMTD1;COMTD1;COMTD1	0.1678747	0.0004388	0.3185015
chr2	cg09036712			0.0794555	0.0004391	0.3185015
chr16	cg05152300	Body	XYLT1;XYLT1	0.2172154	0.0004404	0.3185015

chr7	cg15447685	ExonBnd;Body	ESYT2;ESYT2	-0.138299	0.0004406	0.3185015
chr21	cg07992935	Body	AP000402.3;AP000235.2	-0.2173361	0.0004409	0.3185015
chr8	cg18833383			-0.2490331	0.0004409	0.3185015
chr20	cg18333061	TSS1500	TAF4;TAF4	0.2233157	0.0004416	0.3185015
chr1	cg15178904			-0.1203806	0.0004433	0.3185015
chr8	cg08770998	Body;Body;Body;Body;Body;Body;Body	ZFAT;ZFAT;ZFAT;ZFAT	-0.1010697	0.0004437	0.3185015
chr11	cg26078436	TSS1500	HBBP1;CTD-2643I7.1	-0.1452184	0.0004441	0.3185015
chr17	cg00343839	1stExon;3'UTR	NLRP1;NLRP1;NLRP1;NLRP1	0.4722852	0.0004452	0.3185015
chr5	cg21576256		CTB-11I22.1;CTB-11I22.2	-0.1303347	0.0004465	0.3185015
chr12	cg23170057	5'UTR;1stExon;Body	FAM71C;FAM71C;ANKS1B	-0.1461746	0.0004467	0.3185015
chr12	cg10529804	Body		-0.1463644	0.0004468	0.3185015
chr19	cg25808797	ExonBnd;Body;Body;Body;Body;Body;Body;Body		0.131723	0.0004472	0.3185015
chr20	cg01995281			0.0923537	0.0004476	0.3185015
chr15	cg18476444			-0.1267147	0.0004478	0.3185015
chr7	cg26240231	Body;Body;Body	C7orf50	0.4467914	0.0004479	0.3185015
chr18	cg10334948	5'UTR	L3MBTL4;L3MBTL4	-0.1776141	0.0004485	0.3185015
chr1	cg02151399	TSS1500	CTBS;CTBS;CTBS;CTBS	-0.1623531	0.0004485	0.3185015
chr10	cg25043986		RP11-245J24.1	-0.2161436	0.000449	0.3185015
chr17	cg07505964	Body;Body;TSS1500	MIR301A	-0.129456	0.0004491	0.3185015
chr2	cg03672322	TSS1500	AC114752.3	-0.1157928	0.0004492	0.3185015
chr6	cg25142847			-0.1483971	0.0004494	0.3185015
chr4	cg03847893	Body;Body		-0.1786922	0.0004494	0.3185015
chr7	cg07470570	Body;Body	AUTS2	-0.1654869	0.0004496	0.3185015
chr10	cg02722997			-0.1498495	0.00045	0.3185015
chr14	cg01457080	Body	ANGEL1;ANGEL1	0.145407	0.0004501	0.3185015
chr5	cg12367906		IL7R;IL7R;IL7R	-0.2046024	0.0004502	0.3185015

chr9	cg10016185	Body;Body	ZNF462;ZNF462	-0.1390972	0.0004512	0.3185015
chr20	cg16539768	Body		-0.1603856	0.0004512	0.3185015
chr16	cg00930515		U91319.1;U91319.1	-0.1183461	0.0004512	0.3185015
chr17	cg01132893		HOXB7;RP11-357H14.19;HOXB-AS4	0.2772374	0.0004515	0.3185015
chr1	cg10721728	Body	LRRC7	-0.1245832	0.0004517	0.3185015
chr7	cg04800909	Body;Body;5'UTR	DFNA5;DFNA5;DFNA5;DFNA5;DFNA5;DFNA5;DFNA5	-0.1251789	0.0004543	0.3188317
chr5	cg24478125			-0.1471999	0.0004543	0.3188317
chr12	cg22917372	Body		0.1094793	0.0004545	0.3188317
chr4	cg16214582			-0.162279	0.0004546	0.3188317
chr20	cg06255299			0.2132993	0.0004549	0.3188317
chr5	cg14263957			0.1358175	0.0004551	0.3188317
chr7	cg19722748	Body	DLX6-AS1;DLX6-AS1;DLX6-AS1;DLX6-AS1;DLX6-AS1;DLX6-AS1;DLX6-AS1	0.1751011	0.0004553	0.3188317
chr6	cg20450082	ExonBnd;ExonBnd;Body;Body	COL12A1	-0.1516334	0.0004576	0.319529
chr1	cg07362328	Body		0.1492952	0.0004576	0.319529
chr2	cg14856316			-0.1425409	0.0004582	0.319529
chr20	cg19931348	TSS1500	PI3	-0.1845068	0.0004585	0.319529
chr14	cg12299771			-0.1372646	0.0004593	0.319529
chr4	cg05848434			-0.1496524	0.0004598	0.319529
chr3	cg01285676			-0.1291046	0.0004605	0.319529
chr17	cg19595239			-0.117227	0.0004605	0.319529
chr20	cg18569296	Body;Body;Body		-0.1234662	0.0004611	0.319529
chr6	cg00770599	TSS200;TSS1500;TSS1500	SLC26A8;SLC26A8;SLC26A8;SLC26A8;SLC26A8;SLC26A8;SLC26A8;SLC26A8	0.0831845	0.0004628	0.319529
chr22	cg12117654	5'UTR;Body	ELFN2;ELFN2;RP1-63G5.5;RP1-63G5.5;RP1-63G5.5;RP1-63G5.5;RP1-63G5.5;RP1-63G5.5;RP1-63G5.5	0.145361	0.000463	0.319529

chr16	cg10189135	TSS1500	ATP6V0D1;ATP6V0D1;ATP6V0D1;ATP6V0D1;ATP6V0D1;ATP6V0D1;ATP6V0D1;ATP6V0D1	0.1359811	0.0004636	0.319529
chr1	cg26645700	TSS1500;1stExon;5'UTR	MTMR11;MTMR11;MTMR11;MTMR11;MTMR11;MTMR11;MTMR11;MTMR11;MTMR11	0.1436569	0.0004636	0.319529
chr11	cg09243384			-0.2397405	0.0004646	0.319529
chr9	cg06290690	Body;Body	TRIM14;TRIM14	0.2515638	0.0004655	0.319529
chr1	cg23658362			-0.1364588	0.0004657	0.319529
chr16	cg09535526	Body	ZFPM1;ZFPM1;ZFPM1	0.231112	0.000466	0.319529
chr16	cg10631173	Body;5'UTR	ATMIN;ATMIN;ATMIN;ATMIN;ATMIN	-0.1303403	0.0004666	0.319529
chr2	cg20876248	ExonBnd;Body	EML6;EML6	-0.1553299	0.000467	0.319529
chr20	cg01522717	TSS1500	RP5-1022P6.4	0.1039719	0.0004671	0.319529
chr17	cg21852429	TSS200	C17orf98	0.4145318	0.0004672	0.319529
chr7	cg11616676	Body;Body	SRI;SRI;CTB-167B5.1;SRI	-0.2481452	0.0004679	0.319529
chr12	cg13385981	Body;Body;Body		0.0977955	0.0004685	0.319529
chr7	cg14928241	Body;Body;Body	CADPS2	-0.1296513	0.0004693	0.319529
chr15	cg25959826	Body	RP11-505E24.3;LRRK1;LRRK1;LRRK1	-0.1391483	0.0004695	0.319529
chr5	cg20802710			0.1045586	0.0004701	0.319529
chr5	cg25348888		PAM;PAM;PAM;PAM;PAM	-0.1992214	0.0004704	0.319529
chr1	cg22816626	Body;Body;Body		-0.1215763	0.0004708	0.319529
chr7	cg20127957	Body	CHCHD3;CHCHD3;CHCHD3;CHCHD3	0.1625133	0.0004711	0.319529
chr7	cg03131054	TSS1500;TSS1500;TSS1500;TSS1500;TSS1500	IQCE;IQCE;IQCE;IQCE;IQCE;IQCE;IQCE;IQCE	0.0851692	0.0004713	0.319529
chr4	cg04964428			-0.1978546	0.000472	0.319529
chr13	cg21315358			-0.4888564	0.0004728	0.319529
chr9	cg13628444	Body		0.1896189	0.0004728	0.319529
chr6	cg07836600			-0.1393692	0.0004732	0.319529
chr3	cg21931506			0.1003167	0.0004738	0.319529

chr2	cg03382601		AC010145.4	-0.1187357	0.0004739	0.319529
chr2	cg09715906	TSS1500	SLC4A10;SLC4A10;SLC4A10;SLC4A10;SLC4A10;SLC4A10;SLC4A10;SLC4A10;SLC4A10;SLC4A10	-0.2015592	0.000474	0.319529
chr17	cg18883887	Body	WSCD1;WSCD1	-0.1744995	0.000474	0.319529
chr11	cg18886630	Body	AMPD3;AMPD3;AMPD3;AMPD3	0.1022271	0.0004748	0.319529
chr17	cg12493255	Body;Body;Body;Body	ABR	0.1901816	0.0004751	0.319529
chr21	cg04549141			-0.1567856	0.0004752	0.319529
chr18	cg00452639	Body;Body;Body		0.0721782	0.0004759	0.319529
chr19	cg15649464	Body;Body		0.1276009	0.0004766	0.319529
chr4	cg12341047	Body;Body	FGF5;FGF5	0.1929569	0.0004779	0.319529
chr16	cg23545098	TSS1500;TSS1500	CREBBP;CREBBP	0.1131998	0.0004783	0.319529
chr1	cg13632142			-0.1279421	0.000479	0.319529
chr16	cg05965700	5'UTR;5'UTR	RP11-303E16.6;RP11-303E16.6;RP11-303E16.7;RP11-303E16.8;C16orf46;C16orf46;C16orf46;C16orf46	0.1384564	0.0004793	0.319529
chr12	cg11651209			0.196474	0.0004802	0.319529
chr13	cg17344560	Body;Body;Body		0.0887332	0.0004806	0.319529
chr13	cg20450401	Body;Body	LINC00371;LINC00371	0.2199252	0.0004809	0.319529
chr1	cg19455323		FMN2	-0.1446587	0.0004815	0.319529
chr20	cg08881516	5'UTR;5'UTR	ELMO2;ELMO2;ELMO2;ELMO2;ELMO2;ELMO2;ELMO2;ELMO2;ELMO2;ELMO2;ELMO2	0.1839496	0.0004816	0.319529
chr5	cg10199250	TSS1500	LVRN;LVRN;LVRN	0.1163422	0.0004822	0.319529
chr5	cg00778525	Body	AC008697.1	-0.2489466	0.0004823	0.319529
chr3	cg18161208	Body;Body;Body	BDH1;BDH1	0.1165571	0.0004823	0.319529
chr14	cg21669940	5'UTR	DAAM1;DAAM1;DAAM1;DAAM1	-0.131475	0.0004824	0.319529
chr5	cg00291573			-0.1554952	0.0004826	0.319529

chr6	cg22851977	Body		0.2468564	0.0004827	0.319529
chr3	cg23518541	5'UTR;Body	RARB;RARB;RARB	-0.2079538	0.0004828	0.319529
chr2	cg23189953			0.1208932	0.0004831	0.319529
chr6	cg18030133	5'UTR;1stExon;5'UTR;1stExon	PHACTR2;PHACTR2;PHACTR2;PHACTR2	-0.1265029	0.0004834	0.319529
chr1	cg23879308	Body;Body	TCEANC2	0.094052	0.0004842	0.3197887
chr17	cg18935506	TSS1500	UTP6	-0.1741554	0.0004847	0.3198474
chr3	cg26405475			0.1884327	0.0004863	0.3205899
chr17	cg07529381	TSS1500;TSS1500;TSS1500;TSS1500;TSS200	CDK5RAP3;CDK5RAP3	0.0979972	0.0004882	0.3215738
chr20	cg20543681	3'UTR	SCRT2;SRXN1	0.143803	0.0004891	0.3218225
chr7	cg21932542	Body	RADIL;RADIL;RADIL;RADIL	-0.151277	0.0004909	0.3226546
chr1	cg21497935	TSS200;Body	GIPC2;GIPC2	0.1430876	0.0004913	0.3226546
chr16	cg10202714	Body;Body;Body	ACSF3	-0.1253148	0.0004917	0.3226546
chr3	cg20176212			0.1021054	0.0004935	0.323534
chr3	cg22467301		GNAI2	0.11576	0.000494	0.3236154
chr1	cg01008102	TSS200	RGS18;RGS18;RGS18;RGS18	-0.1825533	0.0004953	0.3240567
chr2	cg23157190	1stExon;5'UTR	HK2;HK2;HK2	0.1103582	0.0004966	0.3240567
chr10	cg18667130	Body;Body;Body	PSAP	-0.1302163	0.0004967	0.3240567
chr11	cg02773624	TSS200	SMTNL1;SMTNL1	0.1529635	0.0004969	0.3240567
chr11	cg22972893	Body	RP11-356J5.12;RP11-356J5.12	0.2339581	0.0004971	0.3240567
chr7	cg17517849	TSS1500	GRM3	-0.1450312	0.0004975	0.3240567
chr20	cg01685428	Body		-0.1709032	0.0004979	0.3240567
chr11	cg23721124	Body;Body;Body		-0.1555198	0.0004987	0.3241488
chr2	cg23717459		U6	-0.1186088	0.0004989	0.3241488
chr3	cg08112841	Body;Body;Body;Body;Body	KCNAB1;KCNAB1	-0.1348196	0.0004999	0.3244558
chr3	cg07206256			-0.1308632	0.0005003	0.3244558

chr5	cg12840325	Body	RP11-267A15.1;RP11-267A15.1;RP11-267A15.1;RP11-267A15.1;RP11-267A15.1	-0.1400073	0.0005009	0.3245024
chr1	cg10970798	3'UTR	CAPZB;CAPZB;CAPZB;CAPZB	-0.1133332	0.0005013	0.3245024
chr7	cg27490143			0.2068388	0.0005041	0.3260273
chr5	cg02711271	Body;Body	SYNPO	0.0752191	0.0005048	0.326178
chr9	cg26063655			-0.1213014	0.0005065	0.326178
chr16	cg05659265	TSS1500;1stExon;5'UTR;1stExon;5'UTR	GNAO1;GNAO1;RP11-461O7.1;GNAO1;GNAO1;GNAO1;GNAO1;GNAO1	0.1287111	0.0005068	0.326178
chr5	cg11136040		WDR41	-0.2028477	0.0005072	0.326178
chr7	cg07620446	5'UTR	GRB10;GRB10;GRB10;GRB10;GRB10;GRB10;GRB10;GRB10;GRB10;GRB10	0.1586314	0.0005077	0.326178
chr4	cg02940093	Body;Body;Body	EGF;EGF	-0.165865	0.0005085	0.326178
chr15	cg23906060	TSS1500	ANKRD34C;RP11-17L5.4;RP11-17L5.4;RP11-17L5.4;RP11-17L5.4;RP11-17L5.4;RP11-17L5.4;RP11-17L5.4	0.1292827	0.0005089	0.326178
chr11	cg23475732	Body;Body	ZBTB16;ZBTB16;RP11-64D24.2	0.1197097	0.0005093	0.326178
chr8	cg01495604	TSS1500;TSS1500;TSS1500	SH2D4A;SH2D4A;SH2D4A	0.117235	0.0005096	0.326178
chr7	cg04666263	Body;Body;Body	ELMO1	0.0784825	0.0005098	0.326178
chr2	cg12525449	Body		-0.2104186	0.0005115	0.326178
chr5	cg26957633			-0.206827	0.0005117	0.326178
chr11	cg11485154			0.1708831	0.0005118	0.326178
chr20	cg09653610			0.2898107	0.000512	0.326178
chr20	cg23574215	TSS1500	XKR7	0.0871274	0.0005126	0.326178
chr8	cg07579284	Body	LINC00535;LINC00535	-0.1254085	0.0005127	0.326178
chr7	cg06079564	Body;Body		0.1789088	0.000513	0.326178
chr13	cg09677330	Body	KLF12	-0.1535834	0.000513	0.326178
chr8	cg15698851	1stExon	TMEM66;TMEM66;TMEM66;TMEM66;TMEM66;TMEM66;TMEM66;TMEM66;TM	-0.2946676	0.0005132	0.326178

			EM66;TMEM66;TMEM66;TMEM66;TMEM66;TMEM66;TMEM66			
chr3	cg21787176	5'UTR	SLITRK3;SLITRK3;SLITRK3	-0.1537802	0.0005137	0.326178
chr11	cg03397616	Body	FAM55B	-0.1644157	0.0005144	0.326178
chr15	cg08401618		RP11-96C21.1	0.072095	0.0005158	0.326178
chr4	cg02665999	5'UTR;5'UTR;5'UTR;Body;Body;Body;Body;Body;Body	SPOCK3;SPOCK3;SPOCK3	-0.1232319	0.0005158	0.326178
chr17	cg08743514	Body		0.1274916	0.0005164	0.326178
chr11	cg16007541	Body	IGSF22;RP11-1081L13.4;IGSF22;RP11-1081L13.4;IGSF22	0.3744396	0.0005165	0.326178
chr17	cg05511978	Body;Body;Body	ANKFY1;ANKFY1;ANKFY1	0.1719036	0.0005176	0.326178
chr3	cg19272144			-0.1235857	0.0005178	0.326178
chr19	cg16773312			0.107584	0.0005182	0.326178
chr6	cg21103269			-0.1499189	0.0005184	0.326178
chr6	cg08279189	Body	TRIM27;TRIM27	-0.2106981	0.0005184	0.326178
chr16	cg18538954		OGFOD1;BBS2;BBS2	-0.1433503	0.0005201	0.326178
chr16	cg08530537	1stExon;5'UTR;5'UTR;1stExon	DPEP3;DPEP3;DPEP3;DPEP3	0.196932	0.0005207	0.326178
chr5	cg08126665		CTC-454M9.1;CTC-454M9.1	-0.1554393	0.0005212	0.326178
chr20	cg09224452			0.2848694	0.0005214	0.326178
chr13	cg21585766	Body	ZIC2;ZIC2;ZIC2;ZIC2	0.12026	0.0005216	0.326178
chr4	cg19934294	Body;Body	MFSD10;MFSD10;MFSD10;MFSD10;MFSD10;MFSD10;MFSD10	0.0947888	0.0005219	0.326178
chr13	cg14311763	TSS1500;TSS1500	CLDN10	0.1554406	0.0005221	0.326178
chr10	cg14803335	Body;Body;Body;Body;Body;Body;Body;Body;Body;Body;Body	FGFR2;FGFR2;FGFR2	-0.1319347	0.0005222	0.326178

chr11	cg07685357	TSS1500;Body	MIR125B1;RP11-166D19.1;RP11-166D19.1;RP11-166D19.1;RP11-166D19.1;RP11-166D19.1;RP11-166D19.1;RP11-166D19.1;RP11-166D19.1;RP11-166D19.1;RP11-166D19.1;RP11-166D19.1;RP11-166D19.1	0.1124822	0.0005223	0.326178
chr3	cg22652435	TSS200;TSS200;TSS200;TSS200;TSS200	IQCJ-SCHIP1;IQCJ-SCHIP1;IQCJ-SCHIP1;IQCJ-SCHIP1;IQCJ-SCHIP1;IQCJ;IQCJ;IQCJ-SCHIP1	-0.261311	0.0005225	0.326178
chr12	cg00785312	Body	RP11-174G6.1	0.1067671	0.0005238	0.326178
chr7	cg25695450			0.1731467	0.000524	0.326178
chr6	cg19866578	ExonBnd;ExonBnd;Body;Body	SYNJ2	-0.1233639	0.0005242	0.326178
chr2	cg10770335			-0.140163	0.0005243	0.326178
chr7	cg05486616			-0.1165578	0.0005248	0.3262479
chr11	cg20143092	TSS200;TSS200;TSS200;TSS200;TSS200	CD44;CD44;CD44;CD44;CD44;CD44;CD44;CD44;CD44;CD44;CD44;RP4-607I7.1;RP4-607I7.1	0.225761	0.0005267	0.3268013
chr1	cg19296400	ExonBnd;ExonBnd;Body;Body;Body	UBXN11;UBXN11;UBXN11;UBXN11;UBXN11;UBXN11;UBXN11;UBXN11;UBXN11;UBXN11	0.1513182	0.0005269	0.3268013
chr4	cg09114528	Body	RP11-503N18.1	0.1861202	0.000528	0.3268013
chr5	cg11352024	Body	C5orf17	-0.1228388	0.0005285	0.3268013
chr4	cg25975995	Body	PET112;PET112;PET112;PET112	0.1174303	0.0005287	0.3268013
chr6	cg09649901	3'UTR;3'UTR;3'UTR	EYA4;EYA4;EYA4	-0.1383774	0.0005295	0.3268013
chr6	cg17612461	5'UTR;5'UTR	TRERF1;TRERF1;TRERF1	0.1067055	0.0005297	0.3268013
chr6	cg13225865	Body;Body;Body;Body;Body;Body	RAB23	-0.1486358	0.0005298	0.3268013
chr5	cg21193966		CTD-2503O16.4	-0.1795462	0.0005298	0.3268013

chr1	cg22989941	Body;Body	TDRD10	-0.136057	0.0005303	0.32684
chr3	cg22915906			-0.1359024	0.0005319	0.3269019
chr19	cg10995397			0.1230094	0.0005322	0.3269019
chr10	cg08135895	Body;Body;Body	NRG3	-0.1529363	0.0005322	0.3269019
chr9	cg11317199	Body;Body;Body;Body	TRIM14;TRIM14	0.2163767	0.0005323	0.3269019
chr12	cg22742001	TSS1500;TSS1500;TSS1500;TSS1500;TSS1500;TSS1500	GIT2;GIT2;GIT2;GIT2;GIT2;GIT2;GIT2;GIT2;GIT2	-0.1738172	0.000533	0.3269019
chr16	cg26828017		CDR2;CDR2;CDR2	-0.2046989	0.0005337	0.3269019
chr13	cg26231071	Body;Body;Body;Body	ITGBL1	-0.1560403	0.0005337	0.3269019
chr12	cg17424856	1stExon;1stExon;5'UTR	ANKRD33;ANKRD33;ANKRD33;ANKRD33;ANKRD33;ANKRD33;ANKRD33;ANKRD33;ANKRD33;ANKRD33	0.1521585	0.0005347	0.3269019
chr7	cg21480319			-0.1383417	0.0005352	0.3269019
chr12	cg07899997			0.2115547	0.0005353	0.3269019
chr12	cg18805734	5'UTR;5'UTR;5'UTR	FAM60A;FAM60A;FAM60A;FAM60A;FAM60A;FAM60A;FAM60A	0.106531	0.0005354	0.3269019
chr19	cg15224011	Body		0.1116181	0.0005374	0.3269489
chr3	cg04333485	Body;Body		-0.1811805	0.0005375	0.3269489
chr2	cg15074047	Body		0.0949085	0.0005377	0.3269489
chr6	cg05544828	3'UTR	PPARD;PPARD	-0.1275911	0.0005379	0.3269489
chr16	cg10922280	TSS1500	DPEP2;DPEP2;DPEP2;DPEP2;DPEP2;DUS2L;DUS2L;DUS2L;DPEP2;DPEP2	0.0978729	0.0005386	0.3269489
chr6	cg01964152			0.1882552	0.000539	0.3269489
chr5	cg07882781	Body	KIAA0825	-0.1981361	0.0005391	0.3269489
chr16	cg04277040	Body;TSS1500	RP11-18F14.1;RP11-18F14.1;RP11-18F14.1;RP11-18F14.1;RP11-18F14.1	0.1130813	0.0005391	0.3269489
chr8	cg16736000	Body;Body;Body	GATA4	-0.1553087	0.0005402	0.3271442

chr1	cg18141902			0.1001041	0.0005407	0.3271442
chr3	cg03624510	TSS1500	KCTD6;KCTD6;KCTD6	0.1402472	0.0005408	0.3271442
chr18	cg09348353	Body;Body		0.0986642	0.0005435	0.3284221
chr19	cg06193788	TSS1500;Body	DPY19L3	-0.1216939	0.0005438	0.3284221
chr13	cg07223180	Body		0.2133794	0.0005453	0.3288152
chr7	cg18005261	Body		-0.1861713	0.0005458	0.3288152
chr1	cg27333706	TSS200	MIR205HG;MIR205HG;MIR205HG;MIR205HG;MIR205HG;MIR205HG;MIR205HG;MIR205HG	0.1094377	0.0005459	0.3288152
chr12	cg15033653	TSS200	CCDC42B	0.0950072	0.0005471	0.3292815
chr7	cg19968134	Body;Body	CCDC136;CCDC136;CCDC136	0.1175781	0.000548	0.3293353
chr2	cg11888727	Body		-0.152242	0.0005481	0.3293353
chr13	cg22561487	Body	POU4F1-AS1;POU4F1-AS1;POU4F1-AS1	0.1405912	0.0005486	0.3293621
chr1	cg00872631	Body		-0.1432489	0.0005501	0.3297303
chr13	cg25385939	Body	MTUS2-AS2;MTUS2	-0.1366802	0.0005505	0.3297303
chr8	cg14635466	Body	SNX31	0.122208	0.0005507	0.3297303
chr6	cg26921068	TSS1500	MCCD1	-0.1150911	0.0005512	0.3297303
chr19	cg25370448	TSS1500	ZNF256	-0.2973172	0.0005515	0.3297303
chr1	cg00646396			0.168469	0.0005525	0.3300727
chr2	cg04071487			-0.2380111	0.0005534	0.3301891
chr2	cg00401161			-0.1295796	0.0005537	0.3301891
chr2	cg16672147	Body;Body;Body		-0.0922448	0.0005545	0.3302681
chr7	cg02308280	Body;Body;Body		0.1642178	0.000555	0.3302681
chr13	cg17044418	Body	RP11-315H15.1	-0.2664546	0.0005552	0.3302681
chr7	cg09900139	Body;Body		-0.1468739	0.0005569	0.3309996
chr2	cg24269863	Body;Body;Body;Body	MTERFD2;MTERFD2;SNED1;MTERFD2;MTERFD2;MTERFD2	-0.1401573	0.0005574	0.3310336
chr21	cg22076474	5'UTR;TSS1500;5'UTR	BACH1;BACH1;BACH1;BACH1;BACH1;BACH1;BACH1;BACH1	-0.1740731	0.0005584	0.3313916
chr10	cg00533891	5'UTR	ZMIZ1	0.1957964	0.00056	0.3319541

chr11	cg23311800	TSS200	ELF5;ELF5;ELF5	-0.1343178	0.0005603	0.3319541
chr1	cg13073141	TSS1500;TSS1500	CERS2;CERS2;CERS2;CERS2;CERS2;CERS2	0.1986741	0.0005628	0.3329051
chr6	cg10471548	5'UTR;Body;1stExon	RIOK1;RIOK1;RIOK1;RIOK1	-0.184521	0.0005629	0.3329051
chr19	cg17139369	TSS200;TSS200;TSS200	2-Mar	0.3361514	0.0005633	0.3329051
chr5	cg21130103		RP11-315A16.1	-0.1763092	0.0005638	0.3329145
chr17	cg19495022	Body		0.1066486	0.0005654	0.3333692
chr22	cg06255037	Body		0.1042545	0.0005655	0.3333692
chr22	cg11570271	TSS200	CECR3	0.1310936	0.0005664	0.3333909
chr22	cg05075268			0.0939767	0.0005665	0.3333909
chr2	cg07936975			-0.2067252	0.000567	0.3333909
chr13	cg18326313	Body;Body	PSPC1-AS1;PSPC1;PSPC1;PSPC1	-0.1741883	0.0005678	0.3333909
chr1	cg13500499			-0.1304206	0.0005685	0.3333909
chr1	cg22033352	Body		-0.122043	0.0005697	0.3333909
chr10	cg18948913	Body;Body	RP11-57C13.3;PAPSS2;PAPSS2;PAPSS2	0.1600967	0.00057	0.3333909
chr3	cg21849126		RP11-134J21.1	-0.1421859	0.0005702	0.3333909
chr3	cg03537184	Body;Body	CLASP2;CLASP2;CLASP2;CLASP2;CLASP2;CLASP2;CLASP2;CLASP2;CLASP2;CLASP2	-0.1551517	0.0005704	0.3333909
chr19	cg10256708	TSS1500	PIK3R2	0.1313368	0.0005709	0.3333909
chr11	cg23636267			0.125519	0.0005714	0.3333909
chr22	cg18161904	TSS1500	RP1-222E13.10;RP1-222E13.10;RP1-222E13.10;RP1-222E13.10	0.2200802	0.0005715	0.3333909
chr4	cg04459751			0.1783958	0.0005715	0.3333909
chr2	cg20989225		GPR55	0.1091692	0.0005724	0.3334129
chr10	cg05944343			-0.1966618	0.0005725	0.3334129
chr20	cg08338597		RP11-560A15.3	-0.1626799	0.0005751	0.3345305
chr6	cg10108296	Body	TRIM15	0.1423671	0.0005766	0.3345305
chr8	cg12477985	5'UTR	ARHGAP39	-0.1221465	0.000577	0.3345305

chr10	cg10017260			-0.1553437	0.0005775	0.3345305
chr6	cg05958778	Body		-0.1408577	0.000578	0.3345305
chr20	cg15402265			0.1347263	0.0005781	0.3345305
chr3	cg01889083			-0.1403333	0.0005788	0.3345305
chr15	cg14374954	Body;Body;Body	CA12	0.1047597	0.0005789	0.3345305
chr3	cg01449754		MIR720	-0.1715415	0.0005789	0.3345305
chr7	cg11102760			-0.1730488	0.0005793	0.3345305
chr17	cg10271836	Body;Body;Body;Body;Body		-0.1305432	0.0005802	0.3345305
chr1	cg22934246			0.1337454	0.0005804	0.3345305
chr1	cg11835197	TSS1500	RP4-728D4.2;RP4-728D4.2;TFAP2E	0.145108	0.0005811	0.3345305
chr1	cg24310780	Body		-0.1687707	0.0005811	0.3345305
chr8	cg09459291	1stExon;5'UTR;1stExon;5'UTR;1stExon;5'UTR	GFRA2;GFRA2;GFRA2;GFRA2;GFRA2;GFRA2;GFRA2;GFRA2;GFRA2;GFRA2;GFRA2;GFRA2	0.1700125	0.0005814	0.3345305
chr5	cg14297914			0.1242786	0.000582	0.3345867
chr18	cg18483766	Body;Body;Body		-0.1234082	0.0005826	0.3346689
chr6	cg23030937	Body;Body;Body	MLIP	-0.135714	0.000583	0.3346689
chr1	cg04023465	Body;Body;Body	AK4;AK4;AK4;AK4;AK4	-0.22075	0.0005845	0.3347639
chr2	cg00759022	Body	IGFBP2;IGFBP2;IGFBP2;IGFBP2	-0.1299385	0.0005861	0.3347639
chr6	cg05849615	Body;Body;Body;Body;Body	DST;DST;DST	-0.1348679	0.0005879	0.3347639
chr13	cg24798968			0.1397487	0.0005882	0.3347639
chr6	cg17345081	TSS200	NMBR;RP11-137J7.2	-0.1747554	0.0005883	0.3347639
chr14	cg03703356	TSS200	CKB;CKB;CKB;CKB;CKB;CKB;CKB;CKB;CKB;CKB	0.1177795	0.0005893	0.3347639
chr2	cg04734865			-0.2015977	0.00059	0.3347639
chr17	cg15321814			0.1137768	0.0005909	0.3347639
chr4	cg10248878	TSS1500	GSX2;GSX2;GSX2	0.1530404	0.0005917	0.3347639
chr7	cg16748520	Body;Body	POU6F2	-0.1190914	0.0005923	0.3347639

chr4	cg12214913	TSS1500;TSS1500	ADH7;ADH7;ADH7;ADH7	-0.1468151	0.0005931	0.3347639
chr8	cg16580197			-0.1916597	0.0005932	0.3347639
chr2	cg17987306	Body;Body		-0.1890888	0.000594	0.3347639
chr10	cg04317263			0.1913015	0.0005941	0.3347639
chr18	cg04995615	Body		-0.142505	0.0005948	0.3347639
chr4	cg04854282	Body	RP11-389E17.1;RP11-162G9.1	-0.1530233	0.0005958	0.3347639
chr7	cg11827925	1stExon;5'UTR	AQP1;AQP1;AQP1;AQP1;AQP1;AQP1	0.1206449	0.0005958	0.3347639
chr12	cg11970289	5'UTR	B3GNT4;B3GNT4;B3GNT4;B3GNT4;B3GNT4;B3GNT4	0.1681377	0.0005959	0.3347639
chr11	cg01664929	Body;Body	PAMR1	-0.1415938	0.000596	0.3347639
chr6	cg21644740	Body;Body	GABBR1	0.2007812	0.0005961	0.3347639
chr1	cg02254587	TSS1500;TSS1500	DDAH1;DDAH1;CYR61;CYR61	0.1379683	0.0005962	0.3347639
chr7	cg23616761	1stExon;TSS1500;1stExon;TSS1500;1stExon	PEG10;PEG10;SGCE;SGCE;SGCE;SGCE;SGCE;SGCE;SGCE	0.3778079	0.0005968	0.3347639
chr7	cg14275933			-0.1569336	0.0005972	0.3347639
chr12	cg16862319	Body		-0.1859982	0.0005976	0.3347639
chr2	cg03106207			0.0946562	0.0005977	0.3347639
chr16	cg25084279	Body	RP11-44I10.6;SIAH1	-0.1066975	0.0005981	0.3347639
chr7	cg25064530			-0.1079554	0.0005981	0.3347639
chr22	cg27201889	Body;Body;Body	SYN3;SYN3	0.1201342	0.0005985	0.3347639
chr8	cg20165746	Body	ERI1;ERI1	-0.1482321	0.0005986	0.3347639
chr9	cg06161398			-0.1472176	0.0005989	0.3347639
chr5	cg14882828	Body		-0.1899405	0.0005989	0.3347639
chr12	cg12917573			-0.1467598	0.0005989	0.3347639
chr11	cg23732576		RP11-672A2.5	0.0905249	0.0005993	0.3347639
chr3	cg02548289	Body;5'UTR	SH3BP5;SH3BP5;SH3BP5;SH3BP5;SH3BP5;SH3BP5;SH3BP5	0.2134888	0.0005997	0.3347639
chr11	cg26647111		DCDC5;DCDC1;DCDC1	0.3231447	0.0005999	0.3347639

chr11	cg13802316	Body;Body	CTTN;CTTN;CTTN	0.21259	0.0006	0.3347639
chr9	cg00424066	TSS1500;TSS1500;TSS1500	MPDZ;MPDZ	-0.1388065	0.0006014	0.3349431
chr2	cg14117065	Body;Body;Body	THADA;THADA;THADA;THADA	-0.1229373	0.0006015	0.3349431
chr5	cg20777670	Body	EFNA5	0.204619	0.0006027	0.3349431
chr1	cg03006527			0.0875359	0.0006027	0.3349431
chr12	cg06161550	Body	GALNT9	-0.4849076	0.0006038	0.3349431
chr13	cg00502575	Body		-0.1325964	0.0006043	0.3349431
chr2	cg04707703	Body	TRIP12;TRIP12	-0.129535	0.0006055	0.3349431
chr16	cg12361705	Body;Body	CMIP	0.1055048	0.0006057	0.3349431
chr1	cg26508681	Body	EPHA10	-0.2157062	0.0006059	0.3349431
chr3	cg04931126			-0.2177531	0.0006063	0.3349431
chr13	cg25990696			-0.1693291	0.0006063	0.3349431
chr1	cg00823843		RP11-278H7.1	0.1980287	0.0006065	0.3349431
chr18	cg06168021			-0.1304438	0.0006067	0.3349431
chr1	cg10716909	TSS1500		-0.1476688	0.000607	0.3349431
chr17	cg17055821	TSS1500	C17orf75	0.2611075	0.0006073	0.3349431
chr7	cg09606588			-0.1429602	0.0006082	0.3349539
chr7	cg12700055	Body;Body;Body;Body;Body		-0.1242603	0.0006083	0.3349539
chr10	cg27523087			0.1507675	0.0006094	0.3349539
chr8	cg15358079			0.1473074	0.0006096	0.3349539
chr2	cg00018635			-0.1135198	0.0006096	0.3349539
chr14	cg02008651	Body;Body		-0.1757686	0.0006128	0.3359057
chr11	cg01491932	Body	SHANK2;SHANK2	-0.1635636	0.0006129	0.3359057
chr14	cg16094352	Body;Body	REC8;REC8;REC8;REC8;REC8	0.1231422	0.0006135	0.3359057
chr8	cg19530281		CTD-2281E23.2	0.1669103	0.0006153	0.3359057
chr12	cg01671341		ACACB;ACACB	0.191618	0.0006159	0.3359057
chr19	cg05398335	Body		-0.1774091	0.000616	0.3359057
chr16	cg06117341	1stExon;5'UTR	CLDN9;CLDN6;CLDN9	0.1874394	0.0006163	0.3359057

chr6	cg09891302		TMEM151B	-0.1381974	0.0006174	0.3359057
chr3	cg19713057	TSS1500;TSS1500	HRASLS	-0.2526334	0.000618	0.3359057
chr8	cg07123118	Body	DLC1;DLC1;DLC1	0.1247412	0.0006181	0.3359057
chr5	cg05462339	Body;Body	SIL1	0.1008246	0.000619	0.3359057
chr3	cg10585661	5'UTR;Body	FAM131A;FAM131A;FAM131A;FAM131A;FAM131A;FAM131A;FAM131A;FAM131A	0.2310591	0.0006199	0.3359057
chr22	cg05815196	TSS200	PLA2G3;PLA2G3	0.1200594	0.0006203	0.3359057
chr2	cg08354007			-0.1383791	0.0006206	0.3359057
chr2	cg23230929		LINC00152;LINC00152;LINC00152	0.1010872	0.0006206	0.3359057
chr1	cg20980494			-0.1250505	0.0006208	0.3359057
chr19	cg19046189	Body		0.0833217	0.0006209	0.3359057
chr20	cg21479898			-0.2245917	0.0006214	0.3359057
chr17	cg14480531	Body;Body		0.1809772	0.0006221	0.3359057
chr19	cg24189904	5'UTR	ZNF177;ZNF177;ZNF177	0.1737868	0.0006222	0.3359057
chr2	cg18577232	Body	LINC00486;LINC00486	-0.1266324	0.0006224	0.3359057
chr12	cg00274203	Body;Body	COQ10A;COQ10A;COQ10A;COQ10A;COQ10A;COQ10A;COQ10A	0.1265053	0.0006225	0.3359057
chr2	cg08114120			-0.1744423	0.0006228	0.3359057
chr1	cg12793044	Body	MEGF6;MEGF6;MEGF6;MEGF6;MEGF6	-0.1207646	0.0006233	0.3359057
chr6	cg25110204			-0.1206824	0.0006233	0.3359057
chr12	cg11881599	3'UTR;TSS1500;TSS1500	CLLU1;CLLU1OS	0.1683309	0.0006235	0.3359057
chr10	cg25021613	Body;Body	PLXDC2	-0.2846557	0.0006249	0.3364136
chr15	cg14164099		CTD-2536I1.1;CTD-2536I1.1	-0.2176703	0.0006261	0.3367956
chr5	cg21987970			-0.1553919	0.0006273	0.3370149
chr10	cg12432168	Body	RP11-67B16.1	-0.1178824	0.0006274	0.3370149
chr3	cg01058983	TSS1500	TBC1D5;TBC1D5;TBC1D5;TBC1D5;TBC1D5;TBC1D5;TBC1D5;TBC1D5;TBC1D5;T	-0.3541402	0.0006285	0.3372378

			BC1D5;TBC1D5;TBC1D5;TBC1D5;TBC1D5			
chr12	cg12718502	TSS1500;TSS1500	TULP3;TULP3;TULP3;TULP3;TULP3;TULP3	0.0982775	0.0006288	0.3372378
chr16	cg16562724	TSS1500	ESRP2;ESRP2;ESRP2;ESRP2;ESRP2;ESRP2;ESRP2	0.1468703	0.0006302	0.3375706
chr7	cg13272526		CTA-254O6.1	-0.1130733	0.0006303	0.3375706
chr9	cg08967503			-0.1760696	0.0006309	0.337647
chr14	cg01172965	TSS1500	KCNK13	0.1764132	0.0006314	0.3376585
chr4	cg06966092	5'UTR;5'UTR;5'UTR	RP11-21G20.3;RBM46;RBM46;RBM46	-0.141362	0.0006327	0.3376817
chr12	cg09755932	TSS200;TSS200;TSS200;TSS200	ANKRD33;ANKRD33;ANKRD33;ANKRD33;ANKRD33;ANKRD33;ANKRD33	0.1564804	0.0006327	0.3376817
chr17	cg12911648	TSS200	KRT17;KRT17;KRT17;KRT17;KRT17	-0.1187402	0.0006329	0.3376817
chr6	cg14880747	Body;Body;Body;Body	GPR126	-0.1629414	0.0006344	0.3382319
chr16	cg27009812	1stExon;5'UTR	CLDN9;CLDN6;CLDN9	0.2364744	0.0006352	0.3382319
chr17	cg00728973	Body;Body;Body		0.0979351	0.0006353	0.3382319
chr10	cg08310058			0.1173218	0.0006359	0.3382946
chr20	cg23301821	5'UTR;TSS1500;Body;Body;Body;Body	SDCBP2;SDCBP2;SDCBP2;FKBP1A-SDCBP2	-0.1186571	0.0006369	0.3384255
chr13	cg09346823			-0.1353612	0.0006372	0.3384255
chr3	cg17731524	Body;Body;Body	NEK10;NEK10;NEK10;NEK10	-0.1548131	0.0006383	0.3384255
chr4	cg23664833		RP11-685F15.1;RP11-4O3.1	-0.1535447	0.0006388	0.3384255
chr7	cg22372849		AC102953.4	0.1449149	0.0006391	0.3384255
chr1	cg24742092	TSS1500	PLA2G2E	-0.1015346	0.0006394	0.3384255
chr8	cg15961904			-0.1852158	0.0006398	0.3384255
chr10	cg07261071	Body;Body	RP11-225H22.5	0.0911856	0.0006399	0.3384255
chr1	cg11881963	Body	EPHX4	-0.1530761	0.0006423	0.3388726
chr4	cg13048201	Body;Body	HERC6;HERC6	-0.0983767	0.0006425	0.3388726

chr10	cg11832000			-0.1717749	0.0006426	0.3388726
chr1	cg07101594	Body	LINC00568	0.1329377	0.0006427	0.3388726
chr18	cg18477104	Body;Body;Body		-0.1374549	0.000645	0.3394901
chr1	cg05425893			-0.1476426	0.0006457	0.3394901
chr7	cg03310874	Body	RADIL;RADIL;RADIL;RADIL	-0.1173179	0.0006459	0.3394901
chr20	cg23518655		RP11-305P22.5	-0.2171956	0.0006465	0.3394901
chr16	cg06743426		LITAF	0.1752068	0.0006466	0.3394901
chr16	cg08514145	Body	U6	-0.136513	0.0006473	0.3394901
chr14	cg15199016	Body;Body	SPATA7;SPATA7;SPATA7;SPATA7;SPATA7;SPATA7;SPATA7	-0.1717672	0.0006482	0.3394901
chr13	cg17308852			-0.1466698	0.0006485	0.3394901
chr19	cg09212407	TSS200	NANOS3;NANOS3	0.089873	0.0006487	0.3394901
chr14	cg14767069	Body;Body;Body;Body;Body	PPP2R5C;PPP2R5C;PPP2R5C;PPP2R5C;PPP2R5C	-0.1054557	0.0006493	0.3394901
chr1	cg06697744	Body	MACF1;MACF1;MACF1	0.0909135	0.0006494	0.3394901
chr7	cg13153834	Body;Body;Body;Body;Body	SUMF2;SUMF2	0.1214364	0.00065	0.3394901
chr10	cg04492166	Body		0.195706	0.00065	0.3394901
chr6	cg27544384		RP5-894D12.3;RP5-894D12.3	-0.1519566	0.0006504	0.3394901
chr9	cg16797474	TSS1500		0.1190083	0.0006513	0.3395363
chr10	cg22763136	Body;Body	CDH23;CDH23;CDH23	-0.1274242	0.0006515	0.3395363
chr10	cg18947261	Body;Body	CDH23	0.067823	0.0006521	0.3395363
chr4	cg26636590	TSS200	COX7B2;COX7B2;COX7B2	-0.1322514	0.000653	0.3395363
chr22	cg24038033			-0.1300931	0.0006535	0.3395363
chr3	cg09439920	TSS1500	TBC1D23;TBC1D23;TBC1D23;TBC1D23	0.1612559	0.0006547	0.3395363
chr8	cg20969199	Body;Body	KIAA1967;KIAA1967	-0.1319472	0.0006556	0.3395363
chr5	cg02946465		CTD-2374C24.1;RP11-6N13.1;CTD-2374C24.1	-0.1399915	0.0006564	0.3395363
chr17	cg07236324	5'UTR	CUEDC1	0.0778808	0.000657	0.3395363
chr4	cg10485816			-0.130167	0.0006572	0.3395363

chr3	cg06900004	TSS1500;TSS1500	NKIRAS1;NR1D2;NR1D2;NR1D2	0.1128292	0.0006572	0.3395363
chr15	cg07879334	TSS1500;TSS1500;TSS1500	SEMA7A;SEMA7A;SEMA7A;SEMA7A;SEMA7A	0.0757865	0.0006574	0.3395363
chr1	cg06002052	TSS1500;Body	C1orf226;C1orf226	-0.1337004	0.0006574	0.3395363
chr8	cg14202477	1stExon;5'UTR	GRHL2;GRHL2;GRHL2;GRHL2;KB-1562D12.1;GRHL2;GRHL2	0.0995724	0.0006575	0.3395363
chr16	cg00749382	Body	AMFR;AMFR;AMFR;AMFR	-0.1345274	0.0006576	0.3395363
chr9	cg02616710	TSS1500	ACER2;ACER2	0.1614576	0.0006586	0.3398162
chr10	cg10827962			0.3928476	0.0006599	0.3398249
chr9	cg08749132	Body;Body;Body;Body	RFX3	-0.1157365	0.0006606	0.3398249
chr7	cg14649827	Body;Body;Body;Body	AP4M1;TAF6	0.1137011	0.0006609	0.3398249
chr4	cg18284616	Body;Body	NFKB1;NFKB1;NFKB1	-0.1281979	0.0006617	0.3398249
chr7	cg12692727	Body;Body;Body	C7orf50	-0.1394859	0.0006618	0.3398249
chr1	cg17850049	Body	FHAD1;RP3-467K16.2;FHAD1;FHAD1;FHAD1;FHAD1	0.127721	0.0006618	0.3398249
chr6	cg04809274	1stExon	HLA-DOA;HLA-DOA;HLA-DOA;HLA-DOA;HLA-DOA;HLA-DOA;HLA-DOA;HLA-DOA;HLA-DOA	0.1810422	0.0006619	0.3398249
chr12	cg08668140	Body;TSS1500;Body;Body	MIR1252;RP1-78O14.1;RP1-78O14.1;RP1-78O14.1	-0.1206551	0.0006634	0.3403104
chr5	cg27348779			-0.1321423	0.0006648	0.3403564
chr3	cg05584597		PLCH1	0.1938544	0.0006648	0.3403564
chr8	cg24540678		ZNF395	0.1729356	0.0006649	0.3403564
chr4	cg12883739	Body;Body;Body	U6;EGF	-0.1235294	0.0006657	0.3404058
chr1	cg14280658			0.17703	0.0006659	0.3404058
chr17	cg25173129	TSS1500	EPX	0.1670194	0.0006668	0.3406233
chr1	cg12559906	Body;Body	DENND2D	0.1843878	0.0006698	0.341426

chr14	cg13995230	TSS1500;TSS1500;TSS1500;TSS1500;Body	MIR654;MIR376C	-0.1451532	0.0006706	0.341426
chr6	cg16832302		XXyac-YX65C7_A.3	-0.1108183	0.0006707	0.341426
chr1	cg24859236	5'UTR	PIK3CD;PIK3CD;PIK3CD;PIK3CD	0.2112488	0.0006708	0.341426
chr5	cg14887344		AC008410.1	-0.1610332	0.0006709	0.341426
chr2	cg07935562	TSS1500;TSS1500;TSS1500;TSS1500;TSS1500;TSS1500	KANSL3;KANSL3;KANSL3;KANSL3;KANSL3;KANSL3;KANSL3;KANSL3;KANSL3	-0.1365836	0.0006726	0.341426
chr2	cg07792332		AC009965.2	-0.1356259	0.0006726	0.341426
chr18	cg17928607	Body	ATP9B;ATP9B	-0.1366075	0.0006726	0.341426
chr2	cg12080566	TSS1500	FAM176A;FAM176A	-0.1350415	0.0006729	0.341426
chr19	cg12261117		MIR1302-10	0.1392421	0.0006747	0.341426
chr9	cg20487632	5'UTR;TSS200;Body	CERCAM;CERCAM;CERCAM;CERCAM;CERCAM;CERCAM;CERCAM;CERCAM	0.1762662	0.0006747	0.341426
chr3	cg03081549	Body		-0.1558922	0.0006747	0.341426
chr16	cg05180206		AC009120.4;AC009120.4;AC009120.4	-0.1056599	0.0006747	0.341426
chr4	cg24974047	Body;Body;Body;Body		-0.1205642	0.0006767	0.341426
chr16	cg06973172	Body;Body	CMIP;CMIP	0.0921975	0.0006768	0.341426
chr1	cg12123924	Body		-0.1748447	0.0006786	0.341426
chr6	cg17119394	TSS200;Body;5'UTR	GFOD1	0.1095591	0.0006792	0.341426
chr17	cg05981034		CRK	0.1481888	0.0006795	0.341426
chr11	cg25977913	Body;Body;Body;Body		0.1120348	0.0006795	0.341426
chr1	cg14452528			0.1766706	0.0006797	0.341426
chr13	cg27452471		snoU13	-0.1411283	0.0006798	0.341426
chr15	cg13234901	TSS1500	OSTBETA	0.0958243	0.0006801	0.341426
chr10	cg22779542		U3	0.2285844	0.0006805	0.341426
chr10	cg05374483	ExonBnd;ExonBnd;ExonBnd;Exo	SEC61A2;SEC61A2;SEC61A2	-0.1054461	0.0006805	0.341426

		nBnd;ExonBnd;Body;Body;Body;Body;Body				
chr11	cg10148841	Body	ROBO4;ROBO4;ROBO4;ROBO4;ROBO4;ROBO4;ROBO4	0.0781954	0.000681	0.341426
chr22	cg11388006	Body;Body	TXNRD2;TXNRD2;TXNRD2;TXNRD2;TXNRD2;TXNRD2;TXNRD2;TXNRD2;TXNRD2	-0.1054414	0.0006813	0.341426
chr16	cg06330621	TSS200	RP11-482M8.1;ZCCHC14;ZCCHC14;RP11-482M8.1;ZCCHC14;ZCCHC14	0.1745576	0.0006816	0.341426
chr4	cg02848389	Body	CLNK;CLNK	-0.1177035	0.0006818	0.341426
chr5	cg19851005	Body		-0.138282	0.0006831	0.341426
chr6	cg25589055	Body;Body	SHPRH;SHPRH	-0.1093393	0.0006836	0.341426
chr6	cg13995568			-0.1614544	0.0006843	0.341426
chr12	cg21269738	Body;Body;Body;Body;Body;Body;Body;Body;Body;Body;Body;Body;Body;Body;Body;Body;Body;Body;Body;Body;Body;Body;Body		-0.1243746	0.0006844	0.341426
chr3	cg24301690	5'UTR	KIAA1257;KIAA1257;KIAA1257;KIAA1257	0.1301453	0.0006845	0.341426
chr4	cg03515346	Body;Body;Body	FAM53A	-0.1292577	0.0006852	0.341426
chr6	cg09151120	TSS1500	SLC16A10;SLC16A10	-0.1321916	0.0006852	0.341426
chr1	cg19365328	TSS1500	CYP2J2;CYP2J2;CYP2J2	0.1763159	0.0006855	0.341426
chr6	cg20134151	TSS1500	PPP1R14C	0.2324411	0.000688	0.3422275
chr12	cg21070941	Body	DNAJC14;RP11-762I7.5	0.0830375	0.0006888	0.3422275
chr5	cg10473171	Body	EFNA5	-0.1255191	0.0006893	0.3422275
chr16	cg11047257			0.1054046	0.0006895	0.3422275
chr4	cg06214925	TSS1500;Body	RP11-558N14.1;RP11-558N14.1;RP11-558N14.1;RP11-558N14.1;RP11-558N14.1	0.1340834	0.0006901	0.3422275
chr12	cg23951683			-0.1452374	0.0006906	0.3422275
chr22	cg09696535	3'UTR	BPIFC;BPIFC	0.2240224	0.0006912	0.3422275

chr1	cg18765270	TSS1500	RP5-952N6.1	-0.147924	0.0006915	0.3422275
chr4	cg00258409	TSS200	CPE;CPE;CPE;CPE	0.1358653	0.0006918	0.3422275
chr5	cg16514544	5'UTR	CDH18;CDH18	-0.1644409	0.0006929	0.3422275
chr19	cg18637476			0.1683567	0.000693	0.3422275
chr11	cg20852226	Body;Body;Body	MYO7A;MYO7A	0.5002838	0.0006935	0.3422275
chr17	cg15835620	5'UTR;1stExon	ALDOC;ALDOC;ALDOC;ALDOC;ALDOC;ALDOC	0.2135793	0.0006936	0.3422275
chr13	cg16021126	TSS1500	SERP2;SERP2;SERP2	0.0928649	0.0006938	0.3422275
chr2	cg06299447	TSS200	FBXO41;FBXO41	0.1197869	0.0006945	0.3422275
chr7	cg00915918		RP11-3P22.2;RP11-3P22.2	0.1317993	0.0006947	0.3422275
chr20	cg07281419		RP11-218C14.5	-0.1191804	0.0006961	0.3422275
chr16	cg19836999	TSS1500	ESRP2;ESRP2;ESRP2;ESRP2;ESRP2;ESRP2;ESRP2	0.1169546	0.000697	0.3422275
chr11	cg25976755	TSS1500;Body	KCNQ1;KCNQ1	-0.1690926	0.0006976	0.3422275
chr9	cg05178561	Body;Body;Body	DOCK8;DOCK8;DOCK8	0.1994109	0.0006976	0.3422275
chr14	cg26099778	Body	TCL6;TCL6;TCL6;TCL6	-0.1131992	0.0006978	0.3422275
chr7	cg22411165	Body		-0.1376764	0.0006987	0.3422275
chr17	cg13053441	Body;Body;Body	GOSR2;GOSR2;GOSR2	-0.1510599	0.0006987	0.3422275
chr3	cg05772206			0.1395401	0.0006995	0.3422275
chr6	cg16071691	Body;Body		-0.1534205	0.0006996	0.3422275
chr2	cg18326616	TSS1500	C2orf78	-0.3157344	0.0006999	0.3422275
chr10	cg05825703			0.1844916	0.0006999	0.3422275
chr19	cg27376984	5'UTR	CDC42EP5	0.2230164	0.0007016	0.3425557
chr6	cg00405086	TSS1500;TSS1500	MED23;MED23;MED23;MED23;MED23;MED23;MED23;MED23;ENPP3;ENPP3;ENPP3;ENPP3	0.2314618	0.0007016	0.3425557
chr17	cg19038804	TSS1500	GAST	-0.1237779	0.0007034	0.3426713
chr10	cg16214148	Body;Body	PRKG1	-0.2547947	0.0007039	0.3426713
chr15	cg14500637	Body	ADAMTS17	-0.1487639	0.0007043	0.3426713
chr2	cg16336556	Body		-0.1553069	0.0007044	0.3426713

chr4	cg05381578	Body	PET112;PET112;RP11-164P12.3;RP11-164P12.3;PET112;PET112;PET112;PET112	0.1433576	0.0007047	0.3426713
chr12	cg18635631	Body	NINJ2;NINJ2;NINJ2	-0.1342689	0.000705	0.3426713
chr8	cg25661986	Body;Body;Body	TRPS1	-0.1882317	0.0007057	0.3426713
chr10	cg25427880			0.1361819	0.000706	0.3426713
chr18	cg20521672	Body		-0.1250855	0.0007062	0.3426713
chr11	cg00026554	TSS1500;TSS1500	NXF1;NXF1;NXF1;NXF1;NXF1;NXF1;NXF1;NXF1	0.1182811	0.0007074	0.3426713
chr11	cg06809604	TSS200;Body;Body;Body;Body;Body;Body;Body;Body	YAP1	0.1738522	0.0007077	0.3426713
chr3	cg15515311	Body	RP11-379K17.4;RP11-379K17.4	-0.1596122	0.0007078	0.3426713
chr7	cg24826151	Body		-0.13572	0.0007084	0.3426713
chr11	cg16160712			-0.1845352	0.0007085	0.3426713
chr2	cg18416734	5'UTR;5'UTR;5'UTR;5'UTR;5'UTR	VIT;VIT;VIT;VIT;VIT;VIT;VIT	-0.2569556	0.000709	0.3426769
chr22	cg08316097	5'UTR;TSS1500;TSS1500;TSS1500	SMCR7L;SMCR7L;SMCR7L;SMCR7L;SMCR7L;SMCR7L;SMCR7L;SMCR7L;SMCR7L;SMCR7L;SMCR7L;SMCR7L;SMCR7L	0.1328607	0.0007124	0.3439563
chr1	cg06695534			-0.1385764	0.0007126	0.3439563
chr22	cg21385983	TSS200	CITF22-24E5.1;PVALB	-0.1615549	0.0007136	0.3441591
chr21	cg04196458			-0.1306865	0.000714	0.3441591
chr10	cg07260003	Body;Body		0.0979243	0.0007146	0.3441591
chr1	cg00407231	Body	KLHDC8A	0.1295549	0.0007157	0.3441591
chr16	cg02729593		RP11-680G10.1	-0.1353312	0.0007171	0.3441591
chr21	cg11949606	Body;Body;Body;Body		0.1574071	0.0007182	0.3441591
chr3	cg07891507		PLCH1	0.18069	0.0007184	0.3441591
chr6	cg08451988	Body;Body;Body	PARK2	-0.1403602	0.0007186	0.3441591
chr2	cg16114629			-0.1168346	0.000719	0.3441591
chr6	cg13051245			-0.2234082	0.00072	0.3441591

chr18	cg24399872	TSS1500;Body	MIR320C2	-0.1204822	0.0007201	0.3441591
chr11	cg01360258	Body	snoU13	-0.1319476	0.000721	0.3441591
chr11	cg18077061	Body		-0.1212978	0.000721	0.3441591
chr14	cg12829286	Body	RIN3;RIN3;RIN3	0.1393063	0.0007212	0.3441591
chr17	cg09786634	Body;Body	RPTOR	0.4180696	0.0007224	0.3441591
chr13	cg16034742		RP11-538C21.1	-0.1152492	0.0007226	0.3441591
chr2	cg09915950	Body	AOX1;AOX1	-0.1357572	0.0007232	0.3441591
chr16	cg17414215	Body		0.1338621	0.0007235	0.3441591
chr17	cg05098880	Body		-0.1279384	0.0007246	0.3441591
chr4	cg06275417	Body	PCGF3;PCGF3;PCGF3	-0.1307983	0.0007257	0.3441591
chr1	cg25492319			-0.1429372	0.0007272	0.3441591
chr8	cg27176043		CTD-3212A4.1;CTD-3212A4.1	-0.1237595	0.0007274	0.3441591
chr14	cg23585420			0.1436277	0.0007276	0.3441591
chr19	cg23136457	Body;Body;Body;Body;Body		0.0939047	0.0007279	0.3441591
chr7	cg10701636			-0.2725831	0.0007281	0.3441591
chr2	cg06669700			0.1192361	0.0007282	0.3441591
chr1	cg10058913			-0.2018854	0.0007285	0.3441591
chr9	cg01166384	5'UTR	NACC2	0.115252	0.0007292	0.3441591
chr9	cg18893685	5'UTR;5'UTR;5'UTR	GAPVD1;GAPVD1;GAPVD1;GAPVD1;GAPVD1;GAPVD1;GAPVD1;GAPVD1	-0.2009426	0.0007297	0.3441591
chr5	cg10424417		CTC-467M3.3;CTC-467M3.1	0.1913938	0.0007318	0.3441591
chr1	cg20825664			-0.1332024	0.000733	0.3441591
chr18	cg26848157	TSS1500		-0.1369028	0.0007335	0.3441591
chr19	cg24168413	5'UTR;TSS200	FXYD1;FXYD1;LGI4	-0.0680131	0.0007337	0.3441591
chr17	cg21468274	Body	RP11-104H15.7	0.265578	0.000734	0.3441591
chr1	cg00852924	Body	TOR3A;RALGPS2	-0.0897277	0.0007341	0.3441591
chr17	cg23864881	TSS200	GAS2L2	0.1163414	0.0007356	0.3441591
chr17	cg26768406	Body;Body;Body;Body;Body		0.1197605	0.0007359	0.3441591
chr14	cg00418695	Body;Body;Body;Body	RAB15;RAB15;CHURC1-FNTB	-0.1469106	0.0007364	0.3441591

chr11	cg05249343	Body;Body	RP11-697H9.2	-0.1177465	0.0007377	0.3441591
chr2	cg24530225	5'UTR	IL1R1;IL1R1	-0.1445001	0.0007387	0.3441591
chr1	cg01420388	TSS1500;Body;TSS1500;TSS200;TSS1500	FBXO44;FBXO2;FBXO2;FBXO44;FBXO44;FBXO44;FBXO44;FBXO2	0.1021485	0.0007409	0.3441591
chr10	cg04526584			0.2486479	0.000741	0.3441591
chr3	cg13476950	5'UTR	DNAH1;DNAH1;DNAH1	0.2012535	0.0007415	0.3441591
chr2	cg14099479			-0.1283102	0.0007419	0.3441591
chr11	cg02023469	TSS200;TSS200	TMEM132A;TMEM132A;TMEM132A;TMEM132A;TMEM132A;TMEM132A;TMEM132A;RP11-881M11.4	0.1279066	0.000742	0.3441591
chr12	cg03632717	5'UTR;5'UTR;5'UTR;Body	SPATS2;SPATS2;SPATS2;SPATS2;SPATS2;SPATS2;SPATS2;SPATS2;SPATS2;SPATS2;SPATS2;SPATS2;SPATS2;SPATS2;SPATS2	0.1145611	0.0007437	0.3441591
chr11	cg01571916	5'UTR	TSPAN18;TSPAN18;TSPAN18;TSPAN18;TSPAN18;TSPAN18;TSPAN18	-0.1334256	0.0007437	0.3441591
chr12	cg25028289	TSS200	SLC11A2;SLC11A2	0.1907773	0.0007439	0.3441591
chr18	cg10690887	TSS1500	ABHD3	0.2047266	0.000744	0.3441591
chr13	cg13171192			-0.1267801	0.0007442	0.3441591
chr12	cg21344159	Body	GPR133;GPR133;GPR133	-0.1468306	0.0007444	0.3441591
chr2	cg00610991	5'UTR	SLC8A1;SLC8A1;SLC8A1;SLC8A1;SLC8A1;SLC8A1	0.2633259	0.000745	0.3441591
chr2	cg11551940			-0.1447335	0.0007462	0.3441591
chr5	cg00660817	TSS1500	KIF3A;KIF3A;KIF3A;KIF3A;KIF3A;KIF3A	0.1007938	0.0007466	0.3441591
chr12	cg19781462		RP11-522N14.1	0.3880363	0.0007467	0.3441591
chr3	cg17365440	Body	MUC13;MUC13	0.1715244	0.0007473	0.3441591
chr8	cg11641916	Body;5'UTR	RP11-439C15.2;RP11-439C15.4;KBTBD11	-0.1407388	0.0007484	0.3441591
chr11	cg16404106	TSS200;TSS200	CCDC81;CCDC81;CCDC81	0.1925863	0.0007486	0.3441591
chr12	cg12113819	TSS1500	MED13L;MED13L	0.093166	0.0007489	0.3441591

chr6	cg14898111		RP11-716O23.1	-0.149529	0.0007491	0.3441591
chr11	cg03871753			-0.1357057	0.0007494	0.3441591
chr6	cg16055406		OSTM1	0.0897025	0.0007495	0.3441591
chr15	cg02906470		RP11-279F6.1;RP11-279F6.1;RP11-279F6.1	-0.2028503	0.0007496	0.3441591
chr7	cg16678001	Body;Body	MAGI2;MAGI2;MAGI2;MAGI2	0.1285448	0.0007501	0.3441591
chr10	cg07252482			-0.1561415	0.0007504	0.3441591
chr17	cg05264101	TSS200		0.0771499	0.0007504	0.3441591
chr8	cg15765497	Body	RP11-662G23.1;NECAB1;NECAB1	-0.1089103	0.0007505	0.3441591
chr3	cg24602369	Body		-0.1077228	0.0007522	0.3441591
chr15	cg10843046	Body;Body	POLG;RP11-217B1.2	-0.1551673	0.0007536	0.3441591
chr2	cg07422110			-0.1722907	0.000754	0.3441591
chr19	cg12669065	5'UTR;TSS200;Body	NUMBL	0.1101452	0.0007543	0.3441591
chr14	cg19477689	Body;Body	JPH4	-0.1493165	0.0007545	0.3441591
chr5	cg26890354	Body;Body;Body;TSS1500;Body;Body;Body;Body;Body;Body;TSS1500;Body;Body;Body;Body;Body;Body;Body;Body;Body;Body;Body;Body;Body	PCDHGC4	0.1756529	0.0007546	0.3441591
chr20	cg19693110	5'UTR	DLGAP4	0.0857975	0.0007557	0.3441591
chr18	cg19186966	Body		-0.1627667	0.0007568	0.3441591
chr7	cg00632863			-0.2028707	0.0007568	0.3441591
chr9	cg14011077		RP11-426A6.7	0.2867252	0.0007586	0.3441591
chr6	cg02833471			-0.1322242	0.0007594	0.3441591
chr17	cg13502414		C17orf51;C17orf51;C17orf51;C17orf51	0.1809631	0.0007602	0.3441591
chr12	cg09521093	Body	RHOF;RP11-347I19.3;RHOF;RP11-347I19.3	-0.203761	0.0007605	0.3441591

chr2	cg25667337		AC012307.2	0.0982319	0.0007607	0.3441591
chr22	cg25225310	Body	PPM1F;LL22NC03-86G7.1;LL22NC03-86G7.1;LL22NC03-86G7.1;PPM1F;PPM1F;PPM1F;PPM1F	0.1010685	0.000762	0.3441591
chr4	cg14017489	Body		-0.1247556	0.0007632	0.3441591
chr1	cg11251215	Body	RNF220;RNF220;RNF220;RNF220;RNF220;RNF220;TMEM53;RNF220	0.1146028	0.0007633	0.3441591
chr1	cg16509736	Body;Body;Body;Body	RBM8A;RP11-315I20.1;RP11-315I20.1;RP11-315I20.1;RP11-315I20.1;RP11-315I20.1	-0.1273506	0.0007636	0.3441591
chr22	cg17527673	Body;Body	SCARF2;SCARF2	0.2621083	0.000764	0.3441591
chr7	cg01017212	Body	C7orf44;C7orf44;C7orf44;STK17A	-0.1393776	0.0007642	0.3441591
chr13	cg14762528	Body		-0.1613237	0.0007644	0.3441591
chr6	cg24529722	TSS200	SLC44A4;SLC44A4;SLC44A4;SLC44A4;SLC44A4	0.101247	0.000765	0.3441591
chr5	cg26117521	Body	EBF1;EBF1;EBF1;EBF1	0.1344744	0.0007654	0.3441591
chr1	cg13987674	TSS200;Body;TSS200	RP11-10N16.3;RP11-10N16.3;GRHL3;GRHL3;GRHL3;GRHL3;GRHL3	0.1562918	0.0007654	0.3441591
chr16	cg04928338			-0.1474158	0.0007663	0.3441591
chr1	cg27452217			0.1624749	0.0007668	0.3441591
chr8	cg01911585	Body;Body;Body;Body		0.1264641	0.0007672	0.3441591
chr13	cg12216419			-0.1637662	0.0007676	0.3441591
chr7	cg26446602			-0.1458226	0.000768	0.3441591
chr6	cg03656583	TSS1500;Body;Body	SGK1;SGK1;SGK1;SGK1;SGK1	0.106519	0.0007681	0.3441591
chr7	cg13552468	Body	U2;GS1-179L18.1	-0.1405008	0.0007684	0.3441591
chr5	cg06017028	TSS200	PGGT1B;PGGT1B;PGGT1B	-0.3979314	0.0007687	0.3441591
chr4	cg04518155	TSS1500	C4orf27;C4orf27	0.1956139	0.0007688	0.3441591
chr17	cg05726450	TSS1500;TSS1500;TSS1500	PIK3R6	0.106381	0.0007697	0.3441591

chr11	cg03826976	TSS1500	CYB5R2;CYB5R2;CYB5R2;CYB5R2;CYB5R2;CYB5R2;CYB5R2;CYB5R2;CYB5R2;CYB5R2;CYB5R2;CYB5R2;CYB5R2;CYB5R2	0.1791866	0.0007701	0.3441591
chr2	cg08860346			-0.1657233	0.0007702	0.3441591
chr6	cg09102573	Body		-0.1050755	0.0007705	0.3441591
chr1	cg14373277	5'UTR;5'UTR;5'UTR;5'UTR;5'UTR;5'UTR	EPB41;EPB41;EPB41;EPB41;EPB41;EPB41	-0.1317577	0.0007707	0.3441591
chr6	cg14106638	Body	NT5DC1	-0.1273592	0.0007722	0.3441591
chr10	cg26781482		TMEM72-AS1	0.1093202	0.0007723	0.3441591
chr7	cg15805330	Body		-0.1333999	0.0007726	0.3441591
chr11	cg17220734	TSS1500	MSANTD4;MSANTD4;MSANTD4	-0.123863	0.0007732	0.3441591
chr17	cg05083423			-0.1054886	0.000774	0.3441591
chr21	cg05620451		SNORA32;TMPRSS3	-0.1273805	0.0007751	0.3441591
chr17	cg22287738		HSD17B1P1	0.0938577	0.0007754	0.3441591
chr9	cg13740078	Body		-0.1177752	0.0007755	0.3441591
chr22	cg18553232		RP11-536P6.3	0.1233308	0.0007757	0.3441591
chr19	cg15645685	TSS1500	PBX4;PBX4;PBX4;PBX4;PBX4	0.2208873	0.0007761	0.3441591
chr13	cg26200980			-0.1811631	0.0007763	0.3441591
chr10	cg06981391	Body	FRMD4A	0.3374343	0.0007768	0.3441591
chr1	cg01942646	TSS200	NR0B2	0.2230239	0.0007768	0.3441591
chr5	cg01084270	3'UTR;3'UTR	CAPSL;CAPSL	-0.1314276	0.0007778	0.3441591
chr3	cg20959189	5'UTR	ZBTB38;ZBTB38;ZBTB38;ZBTB38;ZBTB38;ZBTB38;ZBTB38;ZBTB38;ZBTB38;ZBTB38;ZBTB38	-0.1232017	0.0007781	0.3441591
chr6	cg18836146		RP3-428L16.1	0.1050831	0.0007782	0.3441591
chr5	cg26759568	ExonBnd;Body	RP11-46C20.1	-0.1169094	0.0007785	0.3441591
chr22	cg18747276	Body;Body	XBP1;XBP1	-0.1248234	0.0007791	0.3441591
chr16	cg16248584	Body;Body;Body;Body	CDH13;CDH13	-0.1636358	0.0007792	0.3441591

chr18	cg11401101	Body		-0.1188761	0.0007794	0.3441591
chr10	cg07199534	TSS1500;TSS1500	FAM45A;FAM45A;FAM45A;FAM45A;FAM45A;FAM45A;FAM45A	0.1993447	0.0007795	0.3441591
chr20	cg04188430	5'UTR	SYNDIG1	-0.1376342	0.0007797	0.3441591
chr21	cg09449447	5'UTR;5'UTR	AP000695.4;CLDN14;CLDN14	0.2794165	0.0007802	0.3441591
chr12	cg07349477	Body;Body;Body;Body;Body		-0.2230777	0.0007807	0.3441591
chr6	cg06299718	TSS1500	RP3-523C21.1;RP3-523C21.1;RP3-523C21.1	-0.1220527	0.0007808	0.3441591
chr2	cg00095772	Body		-0.1202929	0.0007816	0.3441591
chr13	cg26744460			-0.2203925	0.000782	0.3441591
chr2	cg11970682	TSS1500;5'UTR;5'UTR;5'UTR;5'UTR;5'UTR;5'UTR	DTNB;DTNB;DTNB;DTNB;DTNB;DTNB;DTNB;DTNB;DTNB;DTNB;DTNB	0.1520336	0.0007822	0.3441591
chr5	cg24424833			-0.1751607	0.0007826	0.3441591
chr1	cg03416592			-0.1365967	0.0007828	0.3441591
chr10	cg26015743			0.1352142	0.0007832	0.3441591
chr2	cg03211711	TSS1500;TSS1500;TSS1500;TSS1500	NLRC4;NLRC4;NLRC4	-0.1410409	0.0007835	0.3441591
chr20	cg12800962			0.1556924	0.0007837	0.3441591
chr13	cg11544434	Body		-0.1833049	0.0007838	0.3441591
chr18	cg27306101	Body;Body	ATP9B	-0.1418003	0.0007841	0.3441591
chr19	cg24808271	TSS1500	ZNF17	0.0917113	0.0007847	0.3441591
chr1	cg13802686		RP11-309H21.3	-0.1245253	0.0007856	0.3441591
chr8	cg20039814	TSS1500;TSS1500	TP53INP1;TP53INP1;RP11-347C18.3;RP11-347C18.3;RP11-347C18.3;C8orf38;C8orf38;C8orf38;C8orf38	0.2817936	0.0007859	0.3441591
chr10	cg20788584	5'UTR	VWA2	0.1338347	0.0007865	0.3441591
chr11	cg03462634	Body;Body;Body;Body	ST3GAL4;ST3GAL4;ST3GAL4;ST3GAL4;ST3GAL4	-0.1364185	0.0007866	0.3441591

chr3	cg13905238			0.242038	0.0007879	0.3441591
chr14	cg24011830		RP11-187E13.2	-0.147979	0.0007888	0.3441591
chr12	cg19898108	3'UTR	HOXC10;HOXC6;HOXC10;HOXC5	0.1389298	0.0007888	0.3441591
chr1	cg05371135	TSS1500	FGR;FGR;FGR	0.1005904	0.0007894	0.3441591
chr15	cg23354714	Body;Body	THSD4	-0.1158185	0.0007895	0.3441591
chr6	cg03554134			-0.118155	0.0007902	0.3441591
chr11	cg00105570	TSS1500	RAG1;RAG1	0.078854	0.0007908	0.3441591
chr11	cg09653626	Body;TSS200;ExonBnd	RP11-712B9.2;RP11-712B9.2;SESN3;RP11-712B9.2;SESN3;SESN3;SESN3;RP11-712B9.2;RP11-712B9.2;RP11-712B9.2	0.2542939	0.0007938	0.3441591
chr13	cg05694908	Body		0.0980935	0.0007942	0.3441591
chr17	cg08970112	Body	KRT25	-0.1715218	0.0007946	0.3441591
chr7	cg08215366	TSS200;TSS200	ZPBP;ZPBP;ZPBP	-0.2058204	0.0007962	0.3441591
chr1	cg15497006			0.2286629	0.0007967	0.3441591
chr7	cg21439214	Body;Body	ZP3	-0.1333361	0.0007989	0.3441591
chr1	cg16685388	5'UTR;5'UTR	HIVEP3;HIVEP3;HIVEP3;HIVEP3	0.3141324	0.0007989	0.3441591
chr10	cg09012269	Body;Body;Body		0.1684634	0.0007995	0.3441591
chr17	cg03839155			-0.1449122	0.0007997	0.3441591
chr9	cg20779473	Body;Body	CACNA1B;CACNA1B	-0.1271434	0.0008002	0.3441591
chr8	cg26764980	Body		0.2142336	0.0008008	0.3441591
chr13	cg02562540	TSS1500;Body;Body;Body	FNDC3A;FNDC3A;FNDC3A	-0.175937	0.000801	0.3441591
chr22	cg03688771	Body;Body;Body	MTMR3	0.1225409	0.0008012	0.3441591
chr8	cg09352738			-0.1619526	0.0008017	0.3441591
chr12	cg02622288		CLLU1	0.1194292	0.0008018	0.3441591
chr19	cg03221702	Body;Body;Body;Body;Body		0.0966913	0.000802	0.3441591
chr14	cg12792931			0.1124916	0.0008033	0.3441591
chr2	cg01822965	Body;Body;Body	FARP2;FARP2;FARP2;FARP2	-0.1074699	0.0008038	0.3441591
chr14	cg11122136			-0.102389	0.0008043	0.3441591

chr18	cg16576811	5'UTR;Body;TSS200;5'UTR;5'UTR;Body;Body;Body;5'UTR	NEDD4L;NEDD4L;NEDD4L;NEDD4L;NEDD4L	0.1032203	0.0008044	0.3441591
chr1	cg25360898	3'UTR	RP4-798P15.2;SEC16B;SEC16B;SEC16B;SEC16B;SEC16B;SEC16B	-0.1118915	0.0008044	0.3441591
chr12	cg09035930	Body	SLC15A4	-0.8241988	0.0008044	0.3441591
chr5	cg12220237	Body	HEATR7B2;HEATR7B2	-0.1849187	0.0008046	0.3441591
chr12	cg06004033	Body	NT5DC3	0.1893256	0.0008052	0.3441591
chr20	cg02725313	Body		0.1221616	0.000806	0.3441591
chr16	cg08859379	TSS1500;TSS1500;TSS1500;Body;Body;Body;Body	RBFOX1	-0.2147191	0.0008063	0.3441591
chr12	cg09908050	Body;Body;Body	TPCN1;TPCN1;TPCN1;TPCN1	0.2038286	0.0008066	0.3441591
chr6	cg11842502		TRAF3IP2-AS1;TRAF3IP2-AS1;TRAF3IP2-AS1;TRAF3IP2-AS1;TRAF3IP2-AS1;TRAF3IP2-AS1	-0.1764619	0.0008071	0.3441591
chr1	cg12016107	TSS1500;TSS1500	UBE2T	0.2843391	0.0008076	0.3441591
chr5	cg22609474		AC091969.1;AC091969.1;AC091969.1	-0.1629276	0.0008082	0.3441591
chr7	cg18654697			-0.0947064	0.0008085	0.3441591
chr7	cg14825462	Body;Body;Body;Body;Body		0.1101637	0.0008088	0.3441591
chr15	cg04875128	Body		0.550556	0.0008092	0.3441591
chr21	cg19498960	Body;Body	SIM2;SIM2	0.1263382	0.0008093	0.3441591
chr11	cg17372806	TSS1500;TSS1500;TSS1500;TSS1500	RTN3;RTN3;RTN3;RTN3;RTN3;RTN3;RTN3;RTN3;RTN3	0.1511394	0.0008097	0.3441591
chr16	cg04653025	Body	LMF1;LMF1;LMF1;LMF1	-0.1297287	0.0008101	0.3441591
chr19	cg25985734			0.1289005	0.0008102	0.3441591
chr1	cg11980897			0.1093384	0.0008102	0.3441591

chr5	cg04580170			-0.1111293	0.0008107	0.3441591
chr5	cg25206709			-0.1208569	0.0008116	0.3441591
chr4	cg11928963			-0.1269648	0.0008117	0.3441591
chr6	cg19819280			-0.1393581	0.0008126	0.3441591
chr5	cg04107129	Body		-0.1540829	0.0008129	0.3441591
chr9	cg07337434	TSS1500	RP11-109D9.3;C9orf135;C9orf135;C9orf135;RP11-109D9.3;C9orf135;C9orf135;RP11-109D9.3	-0.1384239	0.0008136	0.3441591
chr4	cg19676181			0.2273761	0.0008142	0.3441591
chr10	cg04317477			-0.1281492	0.0008152	0.3441591
chr21	cg12767180		PCBP3;PCBP3	-0.15447	0.0008152	0.3441591
chr12	cg21430218	TSS1500	CHPT1;SYCP3;SYCP3;SYCP3;SYCP3;CHPT1	-0.1169061	0.0008155	0.3441591
chr20	cg11841529	TSS200;TSS200	CD40;CD40;CD40;CD40;CD40;CD40	0.2671989	0.0008161	0.3441591
chr16	cg04544898	Body	FTO;FTO	-0.1252911	0.0008162	0.3441591
chr11	cg01178601	Body	ABTB2	-0.1121925	0.0008162	0.3441591
chr19	cg18618355	TSS1500	ZNF599;ZNF599	0.2318273	0.0008175	0.3441591
chr6	cg19139330	Body;Body;Body;Body;Body;Body;Body	U6	-0.1272728	0.0008177	0.3441591
chr3	cg05897316			0.0712594	0.0008179	0.3441591
chr6	cg22281866		NCOA7;NCOA7	0.1686865	0.000818	0.3441591
chr1	cg00272709	TSS200;TSS200	LAX1;LAX1	0.1535388	0.0008184	0.3441591
chr19	cg13256716			-0.1979974	0.0008206	0.3441591
chr16	cg09895527	TSS1500;TSS1500	NUDT7;NUDT7;NUDT7;NUDT7	0.3163402	0.0008207	0.3441591
chr15	cg05215884		ALDH1A2;ALDH1A2	-0.1421443	0.0008223	0.3441591
chr1	cg26924247	Body	RGS7	-0.1671935	0.0008224	0.3441591
chr18	cg03508170	Body;Body;Body;Body	EPB41L3;EPB41L3;EPB41L3	-0.1179867	0.0008225	0.3441591

chr12	cg04123409	5'UTR;1stExon	SDS;SDS;SDS;SDS;SDS;SDS;SDS;SDS	0.2027693	0.0008225	0.3441591
chr21	cg15517335			0.0887676	0.0008226	0.3441591
chr11	cg22338369	Body;Body		-0.3391938	0.0008233	0.3441591
chr8	cg21640732	Body		-0.113556	0.0008236	0.3441591
chr1	cg08774410	TSS200;5'UTR;Body;Body	RP11-10N16.3;RP11-10N16.3;GRHL3;GRHL3;GRHL3;GRHL3;GRHL3	0.1780478	0.0008238	0.3441591
chr6	cg05409540		ZNF292	0.3143065	0.0008243	0.3441591
chr2	cg06484622	Body		0.1866209	0.0008247	0.3441591
chr2	cg15168825		LINC00299	-0.1020252	0.0008256	0.3441591
chr9	cg02150060	5'UTR;1stExon	GLT6D1;GLT6D1	-0.144835	0.0008256	0.3441591
chr20	cg05408680	Body	RP5-955M13.4;RP5-955M13.3;KCNG1	-0.1368443	0.0008258	0.3441591
chr10	cg02932098			0.1254329	0.0008271	0.3441591
chr4	cg20562630	TSS200	GK2	-0.1298456	0.0008273	0.3441591
chr12	cg00845279			-0.2140671	0.0008284	0.3441591
chr1	cg16120196			-0.1191067	0.0008291	0.3441591
chr5	cg09601692		CTD-2066L21.3	-0.1550343	0.0008293	0.3441591
chr1	cg03172058			0.1609211	0.0008295	0.3441591
chr2	cg18301717			-0.1085871	0.0008297	0.3441591
chr1	cg00851361			0.1100256	0.0008301	0.3441591
chr6	cg08955035		HCP5	0.1739533	0.0008308	0.3441591
chr3	cg01999641	Body;Body;Body;Body;Body;Body;5'UTR	IGF2BP2;IGF2BP2;IGF2BP2	0.1082645	0.0008315	0.3441591
chr4	cg16090476	Body;Body;Body;Body	ARHGAP24	-0.1305039	0.0008316	0.3441591
chr14	cg21199629		TRAJ38	-0.2030029	0.0008317	0.3441591
chr14	cg03406313		AL163953.3;AL163953.3;AL163953.3	-0.1463618	0.0008328	0.3441591
chr2	cg01454215	3'UTR	UCN	0.1497278	0.0008335	0.3441591
chr1	cg16821558		PPIE	-0.1746975	0.0008335	0.3441591

chr5	cg11715789	Body;Body;Body	RUFY1;RUFY1;RUFY1	0.1690521	0.000834	0.3441591
chr7	cg20984904	Body;Body;Body		-0.1322894	0.0008343	0.3441591
chr19	cg16992787	Body;Body		0.0905139	0.0008344	0.3441591
chr16	cg07207813	Body	WWOX	0.1365939	0.0008347	0.3441591
chr10	cg05123019	TSS1500;TSS1500	ALDH18A1;ALDH18A1	-0.1431715	0.0008352	0.3441591
chr19	cg14086828	Body	UCA1	0.1343324	0.0008364	0.3441591
chr2	cg22974428		U6	-0.1420431	0.0008366	0.3441591
chr18	cg04616680			-0.171	0.0008368	0.3441591
chr3	cg00841845			0.2437614	0.0008375	0.3441591
chr3	cg05813955	TSS1500	SLC22A14;SLC22A14;SLC22A14;SLC22A14;SLC22A14	-0.1159866	0.0008382	0.3441591
chr3	cg15012214	Body;Body;TSS1500	ARHGEF3;ARHGEF3;ARHGEF3;ARHGEF3;ARHGEF3;ARHGEF3;ARHGEF3	-0.124612	0.0008384	0.3441591
chr3	cg08875089	Body;Body		-0.1774077	0.0008384	0.3441591
chr1	cg16842449			0.1144059	0.0008394	0.3441591
chr12	cg13356734	Body	LPAR5;ACRBP	0.0906221	0.0008403	0.3441591
chr10	cg18389639	Body	PDLIM1	0.1234616	0.0008411	0.3441591
chr6	cg26774971	Body		0.1972191	0.0008411	0.3441591
chr18	cg12778716	Body		0.3217968	0.0008415	0.3441591
chr1	cg22154992	Body	RNF220;RNF220;RNF220;RNF220;RNF220;RNF220;RNF220	-0.1188313	0.0008418	0.3441591
chr11	cg05415146			0.1507867	0.0008425	0.3441591
chr19	cg03540731	1stExon;5'UTR	LRRC25;LRRC25	0.1790445	0.0008428	0.3441591
chr19	cg18462898	Body;5'UTR;TSS1500;Body	MEF2B;MEF2B;MEF2B;MEF2B;MEF2BNB-MEF2B;MEF2B;MEF2B	0.1701889	0.0008431	0.3441591
chr8	cg25768108	Body	EIF3H	-0.1198866	0.0008432	0.3441591
chr16	cg18062490	Body;Body;Body	CCDC135;RP11-405F3.4	0.1407144	0.0008435	0.3441591
chr1	cg05213189			-0.1727494	0.0008448	0.3441591
chr20	cg26667500			0.0800139	0.0008458	0.3441591

chr3	cg24575128	Body	NISCH	0.1072006	0.000846	0.3441591
chr1	cg26371327	Body	EPHA2	0.2582317	0.0008463	0.3441591
chr2	cg08993331			0.3317513	0.0008463	0.3441591
chr8	cg08259755	3'UTR	XKR4;XKR4;RP11-628E19.3	-0.1289372	0.0008466	0.3441591
chr14	cg21304569	Body	RP11-270M14.4	0.1174578	0.0008469	0.3441591
chr6	cg07578986			-0.1191153	0.0008475	0.3441591
chr17	cg18016128	TSS1500;TSS1500	FAM211A;FAM211A	0.0959006	0.0008477	0.3441591
chr2	cg09940349	Body;Body;Body;Body;Body	TGFA-IT1;TGFA	0.1338012	0.000848	0.3441591
chr9	cg04010123	TSS1500;TSS1500;TSS1500	GAPVD1;GAPVD1;GAPVD1;GAPVD1;GAPVD1;GAPVD1;GAPVD1;GAPVD1	0.1780813	0.000849	0.3441591
chr17	cg10433059	TSS1500	AC102948.1	-0.1375195	0.0008491	0.3441591
chr6	cg14878151			-0.1288698	0.0008503	0.3441591
chr10	cg23938419	TSS1500;TSS1500;TSS1500	ZNF365;ZNF365	0.1895334	0.000851	0.3441591
chr9	cg12903948	5'UTR	TMC1;TMC1	-0.2088808	0.000851	0.3441591
chr2	cg03506768			-0.1394272	0.0008515	0.3441591
chr8	cg03617420	Body		0.0918394	0.0008515	0.3441591
chr2	cg12010413			-0.1549162	0.0008518	0.3441591
chr10	cg19511929	Body;Body;Body;Body	CTNNA3;LRRTM3	-0.2056993	0.0008519	0.3441591
chr17	cg26508841	Body;Body		0.1026407	0.0008521	0.3441591
chr17	cg02483931	Body	CCL5;AC015849.2	0.1228276	0.0008523	0.3441591
chr11	cg04880487			-0.132063	0.0008525	0.3441591
chr11	cg03755115	Body;Body;Body	MYO7A	0.1253745	0.000853	0.3441591
chr4	cg05405150			-0.1211671	0.0008535	0.3441591
chr6	cg14082441		U6	-0.1313118	0.0008535	0.3441591
chr7	cg25667676	Body;Body;Body	CADPS2	-0.1419615	0.0008536	0.3441591
chr11	cg01987034	Body	FAM76B;FAM76B	-0.1223542	0.0008545	0.3441591

chr14	cg01267415			-0.1310908	0.0008551	0.3441591
chr7	cg19811609	5'UTR;5'UTR	IMMP2L;IMMP2L;IMMP2L;IMMP2L;IMMP2L;IMMP2L	-0.1404959	0.0008552	0.3441591
chr12	cg07172178			-0.1402405	0.0008552	0.3441591
chr18	cg14434111			-0.1380996	0.0008561	0.3441591
chr6	cg04954726		ZNF292	0.2965547	0.0008573	0.3441591
chr4	cg20345275	Body		0.1382846	0.0008575	0.3441591
chr22	cg07969622	TSS200;TSS200;TSS200;5'UTR;5'UTR;5'UTR	PRAME;PRAME;PRAME;PRAME;PRAME;PRAME;PRAME;PRAME;PRAME;LL22NC03-63E9.3;PRAME	0.1117511	0.0008582	0.3441591
chr16	cg11508935			0.117988	0.0008595	0.3441591
chr3	cg14615449	Body	AC087859.1;AC087859.1;AC087859.1	-0.15433	0.0008603	0.3441591
chr18	cg25653759	TSS1500	SERPINB5;SERPINB5;SERPINB5	-0.1288095	0.0008611	0.3441591
chr9	cg01541867			0.1007697	0.0008616	0.3441591
chr12	cg15359788	Body	RP11-511B23.1;RP11-511B23.2;RP11-511B23.2	-0.1285328	0.0008622	0.3441591
chr7	cg01051024		TRBV7-1	-0.1162951	0.0008625	0.3441591
chr18	cg13357998			-0.1412897	0.0008628	0.3441591
chr4	cg21695476			-0.1488362	0.000863	0.3441591
chr19	cg08634012	5'UTR;5'UTR;TSS1500;TSS1500	STRN4;STRN4;STRN4;FKRP	0.1045109	0.0008634	0.3441591
chr11	cg18468235	Body;Body	AMICA1;AMICA1;AMICA1	-0.1458129	0.0008634	0.3441591
chr4	cg01140719	5'UTR	RBM47;RBM47;RBM47;RBM47;RBM47;RBM47;RBM47;RBM47;RBM47	0.0750425	0.0008646	0.3441591
chr1	cg14975122	Body	HDAC1;HDAC1;HDAC1;HDAC1	0.1934998	0.0008647	0.3441591
chr22	cg16072296		PISD;PISD;PISD	-0.1290166	0.0008649	0.3441591
chr15	cg04482760	TSS1500	SECISBP2L;SECISBP2L;SECISBP2L;SECISBP2L	-0.1177459	0.0008653	0.3441591
chr17	cg06127160	5'UTR	KSR1	0.2651584	0.0008654	0.3441591
chr8	cg17098441			-0.1125776	0.0008659	0.3441591
chr11	cg09090006	Body;Body	CTD-2530H12.7;MAP6;MAP6	0.1100176	0.0008661	0.3441591

chr1	cg01616313			0.0941754	0.0008669	0.3441591
chr11	cg06759780	Body		0.1134566	0.000867	0.3441591
chr17	cg07066019	Body;3'UTR;ExonBnd	C17orf107;CHRNE;CHRNE;CHRNE	0.0928125	0.0008673	0.3441591
chr16	cg11399136	Body		0.115232	0.0008682	0.3441591
chr2	cg05867210			0.1451162	0.0008684	0.3441591
chr5	cg11487037	TSS1500;5'UTR	MATR3;SIL1;SIL1;MATR3;MATR3;MATR3;MATR3;MATR3;MATR3;MATR3;MATR3;SIL1	0.2209575	0.0008686	0.3441591
chr16	cg08787837			0.3345051	0.0008711	0.3441591
chr8	cg05014732	Body	RP11-33I11.2	-0.2046139	0.0008717	0.3441591
chr11	cg02492280	Body;Body;Body;Body;Body;Body;Body	MICAL2;MICAL2;MICAL2;MICAL2;MICAL2	0.0782004	0.0008719	0.3441591
chr20	cg00592995	TSS1500;TSS1500;TSS1500;TSS1500	ZBP1;ZBP1;ZBP1;ZBP1;ZBP1;ZBP1;ZBP1	0.1072525	0.0008721	0.3441591
chr3	cg00974681	Body;Body;Body	CPNE4;CPNE4	-0.1114583	0.000873	0.3441591
chr17	cg26532208	Body;Body		-0.1364679	0.000873	0.3441591
chr6	cg07738234	3'UTR;3'UTR	VGLL2;VGLL2	0.2506814	0.0008733	0.3441591
chr22	cg26478216	TSS1500;TSS1500	TUBA8;TUBA8;TUBA8	0.0920367	0.0008733	0.3441591
chr1	cg02805410	Body	RNU5F-8P	-0.1408351	0.0008736	0.3441591
chr17	cg20538707			0.0886006	0.0008748	0.3441591
chr20	cg11316100	TSS1500;TSS1500;TSS1500	C20orf24;C20orf24;C20orf24;C20orf24;C20orf24;C20orf24;RP5-977B1.11	0.1161715	0.000875	0.3441591
chr11	cg21136371			0.1312639	0.0008752	0.3441591
chr1	cg01226543	Body	ST6GALNAC5	-0.1301468	0.0008753	0.3441591

chr1	cg06161010			0.4014472	0.0008755	0.3441591
chr1	cg01876832			-0.1382332	0.000876	0.3441591
chr5	cg07301795	Body	ADAM19;ADAM19	0.1381951	0.0008779	0.3441591
chr4	cg15600598	Body;Body	SLC2A9;SLC2A9;SLC2A9	-0.1337131	0.0008794	0.3441591
chr2	cg22490405	Body	MEIS1;MEIS1;MEIS1;MEIS1;MEIS1	-0.2319894	0.0008794	0.3441591
chr5	cg02186450			-0.1761467	0.0008801	0.3441591
chr19	cg27093835	5'UTR	FZR1	0.0927175	0.0008802	0.3441591
chr2	cg02603284	Body;Body;Body;Body;Body;Body	TPO;TPO;TPO	-0.151988	0.0008804	0.3441591
chr19	cg19649900	5'UTR	SBNO2	-0.0805837	0.0008824	0.3441591
chr20	cg02107801			0.1465246	0.0008824	0.3441591
chr10	cg20816857	Body;Body;Body;Body;Body;Body;Body		-0.1495099	0.0008833	0.3441591
chr3	cg08926287	TSS1500;TSS1500;TSS1500	SLC6A6;SLC6A6;SLC6A6;SLC6A6;SLC6A6;SLC6A6	0.0890588	0.0008833	0.3441591
chr2	cg08079888			-0.1294064	0.0008837	0.3441591
chr1	cg19362478		HIVEP3	0.1031638	0.0008843	0.3441591
chr4	cg15613153	5'UTR;5'UTR;Body	IL15;IL15;IL15;IL15	-0.1646518	0.0008845	0.3441591
chr21	cg07209338	Body;Body;Body;Body;Body;Body;5'UTR;5'UTR;Body	ERG;ERG;ERG;ERG;ERG;TMPRSS3	0.1383474	0.0008859	0.3441591
chr6	cg15501329			-0.1506179	0.0008859	0.3441591
chr5	cg13687924	Body	CAMK4;CAMK4	0.186053	0.0008865	0.3441591
chr2	cg13969089			-0.1238124	0.0008866	0.3441591
chr1	cg13569053	Body;Body;Body;Body		0.1001431	0.000887	0.3441591
chr3	cg07343039	Body	PTPRG;PTPRG	-0.1518708	0.0008875	0.3441591
chr2	cg00674569	5'UTR;5'UTR;5'UTR;Body;Body	GULP1;GULP1;GULP1;GULP1;GULP1;GULP1;GULP1;GULP1;GULP1;GULP1	-0.144931	0.0008875	0.3441591
chr19	cg23083936	Body;Body	BCKDHA;BCKDHA	0.081451	0.0008875	0.3441591

chr16	cg17638070	Body	LA16c-444G7.2	0.1046029	0.0008878	0.3441591
chr4	cg11469627			-0.1306016	0.0008882	0.3441591
chr8	cg14426030			-0.1303345	0.0008891	0.3441591
chr12	cg06478649	1stExon	MYF5	0.1771229	0.0008894	0.3441591
chr16	cg01701207	Body	SF3B3;SF3B3;SF3B3	-0.2136179	0.0008905	0.3441591
chr12	cg19262963		ARHGEF25;SLC26A10;AC025165.8;AC025165.8	0.125288	0.0008914	0.3441591
chr5	cg21605772			-0.2066866	0.0008924	0.3441591
chr2	cg22039638	TSS1500;TSS1500;TSS1500;TSS1500;TSS1500	ADD2;ADD2;ADD2;ADD2;ADD2;ADD2;ADD2;ADD2;ADD2	-0.2520646	0.0008932	0.3441591
chr1	cg10247328	TSS1500;TSS1500;TSS1500	KCNT2;KCNT2;KCNT2	0.1390742	0.0008943	0.3441591
chr8	cg16699632	Body	MMP16	-0.1959984	0.0008949	0.3441591
chr3	cg03278639			0.1383007	0.0008955	0.3441591
chr3	cg02737861	TSS1500;Body	SLC6A1-AS1;SLC6A1	-0.1298035	0.0008959	0.3441591
chr5	cg05763294	Body	GLRX	-0.1242969	0.0008961	0.3441591
chr11	cg10026995	Body	BRSK2;BRSK2;BRSK2;BRSK2;BRSK2;BRSK2	0.107732	0.0008961	0.3441591
chr1	cg27427318			0.1583448	0.0008962	0.3441591
chr8	cg07120993	TSS1500	PDGFRL;PDGFRL	0.6009251	0.0008962	0.3441591
chr16	cg08461765	Body	RP11-525K10.2;RP11-525K10.3;RP11-525K10.3	-0.2091189	0.0008964	0.3441591
chr6	cg10132858	Body;Body;Body	RNF217;RNF217;RNF217	-0.1139146	0.0008968	0.3441591
chr5	cg27531590			-0.1263336	0.0008968	0.3441591
chr17	cg10718608	Body	SLC47A1;SLC47A1	0.1327039	0.0008972	0.3441591
chr2	cg27134495			-0.1422643	0.0008975	0.3441591
chr12	cg11046544	Body	LPAR5;ACRBP	0.084421	0.0008975	0.3441591
chr12	cg16111073	Body		-0.1864075	0.0008975	0.3441591
chr18	cg00180787	5'UTR	ZNF532	-0.1182439	0.0008978	0.3441591
chr7	cg26062439	Body	GRID2IP	-0.1104816	0.000898	0.3441591

chr12	cg23069718			0.1218078	0.0008985	0.3441591
chr11	cg14620221	1stExon	OR8B8	0.0898325	0.0008985	0.3441591
chr1	cg00851518	Body	SYTL1;SYTL1;SYTL1;SYTL1;SYTL1	0.254274	0.000899	0.3441591
chr12	cg04923758			0.101483	0.000899	0.3441591
chr11	cg01650399	Body	PCNXL3	0.0774701	0.0008991	0.3441591
chr6	cg11755913			-0.111341	0.0008998	0.3441591
chr11	cg02952849	Body		-0.145813	0.0009003	0.3441591
chr15	cg00159951	Body	RP11-382A20.3;RP11-382A20.4	-0.1275906	0.0009007	0.3441591
chr6	cg03665255		RP11-457M11.5	-0.1391091	0.0009009	0.3441591
chr8	cg26708596	Body	RP11-770E5.1;AC026904.1	0.1953895	0.0009016	0.3441591
chr7	cg01058360	Body;Body	PRKAG2;PRKAG2	-0.1616685	0.0009019	0.3441591
chr11	cg26137432	Body;Body	IL18;AP002884.2;IL18;IL18	-0.1156356	0.0009028	0.3441591
chr11	cg15189415			-0.1969043	0.000903	0.3441591
chr11	cg06540283	Body;Body	MAP6;MAP6	-0.1264483	0.0009034	0.3441591
chr16	cg13761842	Body	USP31;USP31	-0.127272	0.0009037	0.3441591
chr21	cg07391141		AP001626.1;AP001626.1	0.0894353	0.0009052	0.3441591
chr19	cg17850597	1stExon;3'UTR	OR10H1;OR10H1	-0.1016919	0.0009053	0.3441591
chr16	cg17768039	Body	RP11-525K10.3;RP11-525K10.3;RP11-525K10.3	-0.125574	0.0009055	0.3441591
chr7	cg04650889	Body	AC090421.1;AC090421.1;AC090421.1	0.144026	0.0009055	0.3441591
chr7	cg06548369			-0.1277445	0.0009064	0.3441591
chr2	cg08035195	Body;Body	RBMS1;RBMS1;RBMS1;RBMS1;RBMS1;RBMS1	-0.1402545	0.0009065	0.3441591
chr2	cg15523907			-0.1628753	0.0009068	0.3441591
chr14	cg11121623	Body	PELI2;PELI2	-0.2597359	0.0009069	0.3441591
chr5	cg22035625			-0.1134188	0.0009074	0.3441591
chr1	cg04767060	Body	PIK3C2B;RP11-739N20.2	0.1768535	0.0009077	0.3441591
chr1	cg04701420	TSS1500	S1PR1;S1PR1;S1PR1;RP4-575N6.4	0.2535776	0.0009078	0.3441591
chr20	cg00239420			-0.1160046	0.0009082	0.3441591

chr2	cg22982930	Body;Body;Body;Body;Body	DIS3L2;AC105461.1;DIS3L2	0.1034987	0.0009083	0.3441591
chr3	cg19525758	Body	ARHGEF3;ARHGEF3;ARHGEF3;ARHGEF3	0.0815285	0.0009092	0.3441591
chr5	cg17762936		RP11-119J18.1	0.0826448	0.0009104	0.3441591
chr4	cg06254804	Body	RP11-362F19.1;RP11-362F19.1;RP11-362F19.1;RP11-362F19.1	-0.1060714	0.0009108	0.3441591
chr10	cg21780171			0.2263784	0.0009112	0.3441591
chr5	cg09896909	Body	RP11-756H20.1	0.1428365	0.0009112	0.3441591
chr11	cg22975913	Body;TSS1500;TSS1500;TSS1500;TSS1500	WT1-AS;WT1;WT1-AS;WT1;WT1;WT1-AS;WT1;WT1-AS;WT1-AS	0.1696421	0.0009113	0.3441591
chr11	cg15838394	TSS1500;5'UTR;5'UTR;Body;Body	PLEKHB1;PLEKHB1;PLEKHB1;PLEKHB1;PLEKHB1;PLEKHB1;PLEKHB1;PLEKHB1;PLEKHB1;PLEKHB1;PLEKHB1;PLEKHB1	0.0792814	0.0009135	0.3441591
chr1	cg24642169		RP11-61J19.4	0.1121787	0.0009137	0.3441591
chr6	cg17337307			-0.114194	0.0009138	0.3441591
chr1	cg21226227		RP4-771M4.3	-0.1382497	0.000914	0.3441591
chr16	cg01832935			-0.1478926	0.0009145	0.3441591
chr16	cg07004313	Body;Body;Body;Body;Body	IQCK;IQCK;IQCK;IQCK;IQCK	-0.1309578	0.000915	0.3441591
chr4	cg06979587	Body;Body;Body;Body;Body;Body	RP11-323J4.1;RAP1GDS1;RAP1GDS1;RAP1GDS1	-0.1288102	0.0009152	0.3441591
chr9	cg13721397	Body	FAM73B;FAM73B;FAM73B;FAM73B;FAM73B	0.1201727	0.0009153	0.3441591
chr1	cg02495604	TSS1500	HPCAL4;PPIE;PPIE	0.1120318	0.0009157	0.3441591
chr11	cg24568187	Body;Body	SMPD1;SMPD1;SMPD1;SMPD1;SMPD1	-0.1458703	0.0009158	0.3441591
chr11	cg12902625	1stExon;1stExon;5'UTR;5'UTR	PYGM;PYGM;PYGM	0.0732604	0.000916	0.3441591

chr1	cg23430652	Body;Body;Body;Body;Body;Body	MIB2;MIB2;MIB2;MIB2;MIB2;MIB2;MIB2;MIB2;MIB2;MIB2	0.1947572	0.0009168	0.3441591
chr17	cg10516317	Body;Body		-0.1250411	0.0009177	0.3441591
chr8	cg08990402			-0.1479758	0.0009178	0.3441591
chr11	cg20986135	1stExon;5'UTR	DCDC5;DCDC5;DCDC5;DCDC5;DCDC5;DCDC5;DCDC1	0.1923504	0.0009185	0.3441591
chr2	cg08787988	TSS1500	IFT172;IFT172;IFT172;IFT172;IFT172;IFT172;IFT172	0.2418001	0.0009191	0.3441591
chr3	cg15562189	Body;Body;Body		0.1201389	0.0009192	0.3441591
chr19	cg24344841			0.0751255	0.0009192	0.3441591
chr3	cg18060685	1stExon	IFRD2;IFRD2;IFRD2;IFRD2;IFRD2;IFRD2;IFRD2;IFRD2;IFRD2;IFRD2;IFRD2;IFRD2;IFRD2;IFRD2	0.1932934	0.000921	0.3442052
chr6	cg03422350	Body;TSS1500;Body;TSS1500;TSS1500	MOCS1;MOCS1;MOCS1;MOCS1;MOCS1;MOCS1;MOCS1	-0.1190683	0.0009211	0.3442052
chr8	cg07018107	Body	MIR1208	0.2142959	0.0009213	0.3442052
chr5	cg16597425	TSS1500;TSS1500;TSS1500;TSS1500	ABLIM3;ABLIM3;ABLIM3;ABLIM3	-0.13076	0.0009215	0.3442052
chr10	cg25633717	Body	LARP4B	0.0688305	0.0009225	0.3442052
chr7	cg16947270	ExonBnd;ExonBnd;ExonBnd;ExonBnd;Body;Body;Body;Body	VIPR2	-0.1363196	0.0009227	0.3442052
chr4	cg07848657		RP11-506N2.1;RP11-506N2.1;RP11-506N2.1;RP11-506N2.1	-0.1383494	0.0009227	0.3442052
chr2	cg22332666	TSS1500	OSGEPL1;OSGEPL1;OSGEPL1-AS1;OSGEPL1-AS1;OSGEPL1-AS1;OSGEPL1;OSGEPL1;OSGEPL1;OSGEPL1	-0.1275824	0.000925	0.344638

chr6	cg09409956	Body	LY6G6D;C6orf25	-0.1180544	0.0009256	0.344638
chr16	cg22320795	Body	CLEC19A;CLEC19A	0.1458997	0.0009262	0.344638
chr5	cg24189734	TSS1500	CTD-2195M18.1;CTD-2195M18.1;CTD-2195M18.1	-0.1537543	0.0009265	0.344638
chr4	cg01469823	Body	G3BP2;G3BP2;G3BP2;G3BP2;USO1	0.1465171	0.0009266	0.344638
chr16	cg02840199	Body	ADCY9;ADCY9	0.1355169	0.0009282	0.344638
chr8	cg10801473	Body	RP11-44K6.3;IDO2	-0.1417339	0.0009289	0.344638
chr5	cg25516542			-0.2034208	0.0009292	0.344638
chr19	cg04351156	Body;Body;Body;TSS1500	PDE4A;PDE4A	0.0859126	0.0009294	0.344638
chr7	cg09342997	Body;Body		-0.2016781	0.0009296	0.344638
chr21	cg02049700	Body	HSF2BP	-0.1184316	0.0009301	0.344638
chr11	cg13207340	Body	MTA2;MTA2;MTA2;MTA2	0.0987959	0.0009305	0.344638
chr12	cg00493276	Body;TSS1500	RP11-641A6.2;KRT73;RP11-641A6.2	-0.1307495	0.0009317	0.344638
chr2	cg23554286	Body		-0.1288484	0.0009322	0.344638
chr6	cg01299932	5'UTR;5'UTR	ATXN1;ATXN1;ATXN1;ATXN1	0.1203937	0.0009325	0.344638
chr11	cg24777572			-0.1229959	0.0009331	0.344638
chr1	cg13427179	Body;Body	PRDM16;PRDM16	-0.1317345	0.0009337	0.344638
chr2	cg27560494	Body	UBR3;UBR3;UBR3	-0.1347331	0.0009337	0.344638
chr9	cg21614231	Body;TSS1500;Body	BAG1;BAG1;BAG1	-0.1019179	0.0009349	0.344638
chr3	cg19088924			-0.1296896	0.0009349	0.344638
chr13	cg27021512	TSS1500	ESD;ESD	0.1589679	0.0009352	0.344638
chr14	cg21814550			-0.1684683	0.000936	0.344638
chr2	cg03422907			-0.1035302	0.0009369	0.344638
chr6	cg16943816		RP11-428F8.2	-0.1728047	0.0009372	0.344638
chr5	cg04415699	ExonBnd;Body;Body;Body;Body;Body	RP11-2O17.2;RP11-2O17.2;RP11-2O17.2;C5orf64;C5orf64;C5orf64;C5orf64	-0.1364811	0.0009376	0.344638
chr11	cg04334121	5'UTR	SAA4;SAA2-SAA4	-0.1275417	0.0009377	0.344638

chr5	cg07486383	5'UTR	GDNF;GDNF	0.1866206	0.0009378	0.344638
chr17	cg13383842	TSS1500	SECTM1	0.0958273	0.0009381	0.344638
chr1	cg00991578			-0.2063534	0.0009387	0.344638
chr16	cg15195412	Body		0.0965015	0.0009397	0.344638
chr19	cg09935271	Body	YJEFN3;NDUFA13;YJEFN3	0.1590463	0.0009403	0.344638
chr2	cg15031550			-0.1695564	0.0009406	0.344638
chr1	cg05095832		RP11-74C13.4	-0.1427934	0.0009409	0.344638
chr8	cg03306780		RP11-369E15.1	-0.1229467	0.0009418	0.344638
chr1	cg14694639	5'UTR	BCL9	0.0935037	0.0009419	0.344638
chr11	cg15948836	Body	LDLRAD3	0.1928925	0.0009421	0.344638
chr2	cg00454592		AC096559.1	-0.2068078	0.0009424	0.344638
chr12	cg22845525	Body;Body	MYO1A	0.1295255	0.0009431	0.344638
chr5	cg05198507	Body		-0.1376993	0.0009435	0.344638
chr9	cg09782493	Body;Body;Body	C9orf3;C9orf3;C9orf3;C9orf3;C9orf3	-0.1194929	0.0009435	0.344638
chr15	cg12206157			0.0912128	0.0009435	0.344638
chr11	cg17745872			-0.1363185	0.0009441	0.3446611
chr4	cg06589421	3'UTR	ODZ3	-0.1100766	0.0009445	0.3446611
chr2	cg02996903			0.1140674	0.0009454	0.3448077
chr10	cg00706275		EXOC6	0.1393352	0.0009464	0.3449573
chr19	cg11939550	Body;Body;Body;Body;Body;Body		0.1484268	0.0009468	0.3449573
chr19	cg01778750		AC011500.1	-0.143938	0.0009477	0.3451172
chr10	cg22177688		C10orf90;C10orf90	-0.1488111	0.0009484	0.3451575
chr10	cg13490895		RP11-554I8.2	0.1740867	0.0009495	0.3451575
chr9	cg07941680	Body	BNC2	0.1638013	0.0009505	0.3451575
chr18	cg21273905	3'UTR	FHOD3	-0.1211585	0.0009512	0.3451575
chr7	cg21561953			-0.1809742	0.0009512	0.3451575
chr17	cg14352416	Body	CCDC40	-0.1228632	0.0009533	0.3451575
chr19	cg22656115	Body		-0.1230996	0.0009536	0.3451575

chr15	cg06094959	Body;Body;Body;Body;Body	PPCDC;PPCDC;PPCDC	0.0954332	0.0009536	0.3451575
chr22	cg07331806	5'UTR;5'UTR	AIFM3;AIFM3;AIFM3;AIFM3;AIFM3;AIFM3;AIFM3;AIFM3	0.0888852	0.0009537	0.3451575
chr5	cg03889016	Body	ANKH	-0.1266973	0.0009541	0.3451575
chr4	cg06635722			0.1645409	0.0009542	0.3451575
chr10	cg02555952		STK32C	0.1786413	0.0009545	0.3451575
chr11	cg09209543			-0.1378601	0.0009549	0.3451575
chr7	cg09515778	TSS200;TSS200	SLC4A2;SLC4A2;SLC4A2;CDK5;CDK5;CDK5;CDK5;CDK5	0.1292939	0.000956	0.3451575
chr1	cg00700214	5'UTR	FMO2;FMO2;FMO2;FMO2;FMO2;FMO2;FMO2;FMO2;RP1-45C12.1	-0.2137022	0.000956	0.3451575
chr11	cg03742937	5'UTR;Body		0.100706	0.0009564	0.3451575
chr17	cg10713461		CTD-3195I5.5	-0.1725289	0.0009566	0.3451575
chr17	cg25282715	Body	ADAM11	0.102277	0.0009568	0.3451575
chr16	cg22459179		RP11-77K12.5	-0.1727643	0.0009571	0.3451575
chr21	cg10200551	Body;Body	TTC3;TTC3;TTC3;TTC3;TTC3;TTC3;TTC3;TMPRSS3	-0.1174472	0.0009574	0.3451575
chr6	cg06501295		RP11-497D6.4;RP11-497D6.4;RP11-497D6.4	0.2410021	0.0009581	0.3452338
chr6	cg17459557	Body;Body;Body;Body;Body;Body	RP11-73O6.4	-0.1712049	0.0009602	0.3458022
chr6	cg22748272			-0.1363667	0.000961	0.3458022
chr6	cg10918729	Body;Body	MLIP-AS1	-0.1429929	0.0009615	0.3458022
chr8	cg10327067			0.1936644	0.0009616	0.3458022
chr16	cg07383076	5'UTR;5'UTR;TSS200	ABCC11;ABCC11;ABCC11;ABCC11;ABCC11	-0.1192016	0.0009621	0.3458099
chr2	cg25734127	Body	AC010127.3	-0.1412936	0.0009627	0.3458564
chr17	cg11972332			0.0937597	0.000964	0.3458564
chr6	cg03954573	TSS200	FAM50B;FAM50B;RP11-420L9.4	0.1012527	0.0009653	0.3458564

chr18	cg07356006			-0.107201	0.0009654	0.3458564
chr6	cg26845404	Body;Body	ASF1A	-0.1476008	0.0009656	0.3458564
chr8	cg18843853			-0.2303018	0.0009658	0.3458564
chr6	cg02236874	Body;Body;Body	TAPBP;TAPBP	-0.1467471	0.0009658	0.3458564
chr21	cg22684717	Body	SLC37A1	0.1575233	0.0009663	0.3458564
chr8	cg18533313	5'UTR;5'UTR	EBAG9;EBAG9;EBAG9;EBAG9;EBAG9;EBAG9	-0.1491586	0.0009667	0.3458564
chr2	cg10112391	Body	AC008063.2;DPP4;DPP4;DPP4	0.1961397	0.0009673	0.3458564
chr17	cg21787305	TSS1500	CDC42EP4;CDC42EP4	0.1119322	0.0009682	0.3458564
chr4	cg13941682	5'UTR	PCGF3;PCGF3;PCGF3;PCGF3;PCGF3;PCGF3;PCGF3;PCGF3;PCGF3	-0.3473125	0.0009691	0.3458564
chr4	cg15209200	Body	RP11-213G21.1;RP11-83C7.2	0.0771206	0.0009693	0.3458564
chr2	cg05729783	TSS1500;TSS1500;TSS1500;TSS1500;TSS1500	AGFG1;AGFG1;AGFG1;AGFG1;AGFG1	-0.1245238	0.0009697	0.3458564
chr1	cg08116144		DDR2	-0.1264303	0.0009714	0.3458564
chr12	cg15289144	TSS1500	RP11-916O13.1;C12orf12	-0.137902	0.0009714	0.3458564
chr4	cg18593893		RP11-608O21.1	-0.1373322	0.0009717	0.3458564
chr4	cg10702457	TSS1500	MEPE;MEPE;MEPE;MEPE;MEPE;MEPE;MEPE	-0.1421516	0.000972	0.3458564
chr12	cg06446550	Body;Body;Body;Body;Body;Body;Body	TXNRD1	0.0914908	0.0009733	0.3458564
chr1	cg04604962	Body	PCNXL2	-0.1174361	0.0009736	0.3458564
chr21	cg06524507			-0.161297	0.0009745	0.3458564
chr21	cg07557896		AF015262.2;RUNX1	-0.1256677	0.0009761	0.3458564
chr8	cg09365284		CSGALNACT1;CSGALNACT1;CSGALNACT1;CSGALNACT1;CSGALNACT1	-0.1201825	0.0009763	0.3458564
chr3	cg02979978	Body;Body;Body	ITPR1	-0.1794938	0.000977	0.3458564
chr13	cg16579641	TSS1500;Body	STARD13;RP11-81F11.3;STARD13	-0.2471172	0.000977	0.3458564

chr1	cg21124196	TSS1500;TSS1500	PRRX1;PRRX1;PRRX1;PRRX1;PRRX1	-0.2082795	0.0009773	0.3458564
chr1	cg21542223			0.13118	0.0009774	0.3458564
chr8	cg24923430	Body	MFHAS1;MFHAS1;MFHAS1;MFHAS1	-0.6095226	0.0009779	0.3458564
chr8	cg23204701			-0.1137467	0.0009786	0.3458564
chr16	cg21724915	Body	FTO;FTO	-0.2264441	0.000979	0.3458564
chr18	cg18706960	5'UTR;5'UTR	C18orf1	-0.1121244	0.0009802	0.3458564
chr1	cg04247069		RP11-73M7.1	-0.1355988	0.000981	0.3458564
chr4	cg14023398	TSS1500	RBM47;RBM47;RBM47;RBM47;RBM47;RBM47;RBM47;RBM47	0.1065498	0.0009811	0.3458564
chr16	cg15836785			-0.130047	0.0009815	0.3458564
chr5	cg11591082			0.0921704	0.0009815	0.3458564
chr9	cg13633625			-0.1290035	0.0009816	0.3458564
chr12	cg25347264	TSS1500	NTF3;NTF3	0.2156479	0.000982	0.3458564
chr5	cg10945695			-0.1086507	0.0009822	0.3458564
chr11	cg12958961			0.4064336	0.0009828	0.3458564
chr8	cg21698295		RP11-53M11.3;RP11-53M11.3	0.1371611	0.0009829	0.3458564
chr16	cg10716697	5'UTR;5'UTR;5'UTR;1stExon;1stExon;1stExon;Body;Body;Body;Body	RBFOX1;RBFOX1;RBFOX1	-0.1807089	0.000983	0.3458564
chr9	cg06012090	Body		0.1396503	0.0009835	0.3458564
chr1	cg04446579	TSS200	ANKRD45;ANKRD45	0.1069381	0.0009836	0.3458564
chr2	cg16526526	TSS1500;1stExon;1stExon;TSS200;5'UTR;5'UTR	NLRC4;NLRC4;NLRC4;NLRC4;NLRC4	-0.11402	0.0009838	0.3458564
chr19	cg08321827	TSS1500	AC002398.5;AC002398.5;AC002398.5	0.0949744	0.0009839	0.3458564
chr16	cg26532102	Body	VWA3A;VWA3A;VWA3A	-0.1045131	0.000985	0.3460865
chr17	cg11142556	Body	TBCD	-0.1410129	0.0009861	0.3461502
chr20	cg07434077	Body;Body		0.0873512	0.0009861	0.3461502

chr10	cg14366146			-0.1225081	0.0009877	0.346518
chr1	cg26348343	Body;Body		-0.1266378	0.0009898	0.3466571
chr19	cg19298856	Body		0.0862316	0.0009903	0.3466571
chr5	cg02094681			0.6305719	0.0009908	0.3466571
chr7	cg04988103		RP5-978I12.1	-0.1730105	0.0009916	0.3466571
chr6	cg14756006			-0.1660205	0.0009922	0.3466571
chr16	cg13813391	5'UTR;1stExon	CMTM2;CMTM2;CMTM2;CMTM2;CMTM2;CMTM2	0.1192671	0.0009924	0.3466571
chr5	cg00559992	Body		-0.1412782	0.0009951	0.3466571
chr11	cg14868747	TSS1500	ALX4	0.1443225	0.0009951	0.3466571
chr15	cg13151006			-0.1242503	0.0009954	0.3466571
chr7	cg14788130			-0.1115032	0.0009955	0.3466571
chr8	cg27510066	Body;5'UTR	RP11-1105O14.1;RP11-1105O14.1;CSGALNACT1;CSGALNACT1;CSGALNACT1;CSGALNACT1;CSGALNACT1;CSGALNACT1;CSGALNACT1;CSGALNACT1	-0.2105869	0.000996	0.3466571
chr8	cg00550132		RP11-400K9.3	-0.1247522	0.0009961	0.3466571
chr17	cg09413013	Body		0.1826855	0.0009964	0.3466571
chr14	cg22465490	Body		-0.2003954	0.0009964	0.3466571
chr16	cg00121135	5'UTR;Body	CBFA2T3;CBFA2T3;CBFA2T3;CBFA2T3;CBFA2T3;CBFA2T3	0.0862873	0.0009966	0.3466571
chr2	cg20232723			-0.1673263	0.0009969	0.3466571
chr1	cg23722790	3'UTR	SLC35D1	0.1097601	0.0009972	0.3466571
chr1	cg24587119		ST13P19	-0.1533102	0.0009972	0.3466571
chr11	cg24968203	TSS1500;TSS1500;TSS1500;TSS1500;TSS1500	GRIA4;GRIA4;GRIA4;GRIA4;GRIA4;GRIA4;GRIA4;GRIA4;GRIA4;GRIA4;GRIA4	0.1648821	0.0009974	0.3466571
chr5	cg26485878	1stExon;Body;Body;Body;Body	PDE4D;PDE4D;PDE4D;PDE4D	0.110266	0.0009977	0.3466571
chr6	cg07708721	TSS1500;Body	PRRC2A;SNORA38	0.0910444	0.0009982	0.3466757

chr17	cg22805688	0.2913048	0.0009996	0.3468107

Supplementary Table S2. Results for the six FDR-significant DMPs after further adjustment for YMRS and MADRS scores (discovery cohort).

chr	probe	log2FC	p	FDR q	gene	gene distance	gene feature
chr4	cg20244265	-0.32	7.28E-07	0.12	*CXCL8*	-22861	intergenic
chr17	cg25876840	0.28	1.28E-07	0.07	*CD300LG*	-4066	intergenic
chr17	cg11476866	0.33	2.07E-07	0.07	*CD300LG*	-4128	intergenic
chr7	cg15653194	-0.58	3.01E-06	0.15	*LFNG*	0;0	exon+utr5;intron
chr2	cg00306112	0.24	7.95E-07	0.12	*LINC01494*	0	nc_intron
chr7	cg20242392	0.3	3.57E-06	0.15	*DGKI*	0;0	intron;intron+utr5

Supplementary Table S3. DMPs at nominal p < 0.001 in the discovery cohort BD/SA vs. CON EWAS.

chr	Probe ID	Gene feature	Gene annotation	logFC	p	FDR q
chr6	cg20008357	TSS1500;TSS1500;5'UTR	AGPAT1;AGPAT1;RNF5;RNF5;RNF5;AGPAT1;AGPAT1;AGPAT1	0.3868297	8.27E-08	0.060061
chr6	cg00169175	TSS200	HACE1;HACE1;HACE1;HACE1;HACE1	0.3279597	6.54E-07	0.2374105
chr4	cg13430719	1stExon;TSS200	RP11-453E17.1;RP11-453E17.1;RP11-453E17.1;UBA6;UBA6;UBA6;UBA6;RP11-453E17.1;UBA6;UBA6	0.2467868	4.18E-06	0.7221816
chr8	cg07419619	Body	ZNF251;ZNF251	-0.1750089	5.65E-06	0.7221816
chr3	cg05791356	Body;Body;Body	ATG7	-0.189435	8.75E-06	0.7221816
chr2	cg12662214	5'UTR;5'UTR;TSS1500	PIGF;PIGF;PIGF;PIGF;CRIPT;CRIPT;PIGF;CRIPT;PIGF;PIGF	0.288764	8.78E-06	0.7221816
chr5	cg14302113	1stExon;1stExon;5'UTR	ITGA1;PELO;ITGA1;PELO;PELO;ITGA1;PELO;CTD-2288O8.1	0.2128315	9.17E-06	0.7221816
chr4	cg07097005			0.2170542	1.10E-05	0.7221816
chr12	cg02493153	Body;Body;Body;Body;Body;Body	RP11-359J14.2	0.1821271	1.26E-05	0.7221816
chr13	cg11199428	TSS200;TSS200;TSS200	INTS6;INTS6;INTS6;INTS6;INTS6-AS1;INTS6;INTS6;INTS6;INTS6;INTS6;INTS6;INTS6;INTS6	0.2364949	1.74E-05	0.7221816
chr19	cg26202425	Body	PPP1R37;PPP1R37	-0.1867956	1.86E-05	0.7221816
chr1	cg12445832			0.2147276	1.97E-05	0.7221816
chr11	cg14063402	Body;Body;5'UTR;5'UTR;5'UTR;5'UTR;TSS1500	LSP1;LSP1;LSP1;LSP1;LSP1;LSP1;LSP1;LSP1;LSP1	-0.2150931	2.08E-05	0.7221816
chr5	cg06622735	3'UTR;TSS1500	CTD-2228K2.5;CTD-2228K2.5;CTD-2228K2.5;CTD-2228K2.5;CTD-2228K2.5;SLC9A3	0.1603231	2.10E-05	0.7221816

chr1	cg12632487	Body;TSS1500	RP11-242F24.1;RP11-242F24.1;RP11-242F24.1	0.3894865	2.28E-05	0.7221816
chr8	cg27167922	Body;Body;Body;Body;Body;Body;Body;Body;Body;Body;Body;Body	ZNF707;ZNF707;ZNF707	-0.2142433	2.28E-05	0.7221816
chr3	cg13913917	5'UTR;1stExon	RP11-225N10.1;ATP11B;ATP11B;ATP11B;ATP11B	0.1325999	2.35E-05	0.7221816
chr20	cg23159704			0.2629837	2.68E-05	0.7221816
chr16	cg15973550			-0.3115069	2.74E-05	0.7221816
chr5	cg13624988		CTC-229L21.1	0.6260608	2.81E-05	0.7221816
chr19	cg22912577	TSS200	NDUFA3;NDUFA3;NDUFA3;NDUFA3;NDUFA3;NDUFA3;NDUFA3;NDUFA3;OSCAR;NDUFA3;NDUFA3	0.1348713	2.91E-05	0.7221816
chr4	cg08118646	1stExon;TSS200;1stExon	EIF4E;EIF4E;EIF4E;EIF4E;EIF4E;EIF4E;EIF4E;EIF4E;EIF4E;EIF4E	0.1329553	2.99E-05	0.7221816
chr22	cg25703541	TSS200		-0.822994	3.14E-05	0.7221816
chr22	cg17192519	TSS1500	JOSD1;JOSD1;JOSD1;JOSD1;JOSD1;JOSD1;JOSD1;JOSD1;JOSD1	0.3637058	3.22E-05	0.7221816
chr1	cg07975032	5'UTR;1stExon;Body	SLC6A9;SLC6A9;SLC6A9;SLC6A9;SLC6A9;SLC6A9;SLC6A9;SLC6A9;SLC6A9	0.4037661	3.48E-05	0.7221816

chr12	cg26899692	5'UTR;1stExon;1st Exon;5'UTR	DYRK2;DYRK2;DYRK2;DYRK2;DYR K2;DYRK2;DYRK2	0.3128116	3.64E-05	0.72 2181 6
chr5	cg23928910	TSS1500	CETN3;CETN3;CETN3;CETN3	0.1857908	3.70E-05	0.72 2181 6
chr13	cg10354859	Body	VPS36;VPS36	0.140842	3.84E-05	0.72 2181 6
chr15	cg16587794	TSS200;TSS200	KIF23;RP11-253M7.1;KIF23;RP11-253M7.1;KIF23;KIF23;KIF23;KIF23;KI F23;KIF23;KIF23;KIF23;RP11-253M7.1	0.1329108	3.93E-05	0.72 2181 6
chr16	cg26954464	Body	ATP6V0D1;ATP6V0D1;ATP6V0D1;AT P6V0D1;ATP6V0D1;ATP6V0D1;ATP6 V0D1;ATP6V0D1	-0.1992446	3.97E-05	0.72 2181 6
chr17	cg05511978	Body;Body;Body	ANKFY1;ANKFY1;ANKFY1	0.230917	3.98E-05	0.72 2181 6
chr19	cg13711528	TSS200	ACTN4;ACTN4;ACTN4;ACTN4;ACTN 4	0.2355138	4.12E-05	0.72 2181 6
chr5	cg26208419	3'UTR;TSS1500	CTD-2228K2.5;CTD-2228K2.5;CTD-2228K2.5;CTD-2228K2.5;CTD-2228K2.5;SLC9A3	0.2907374	4.15E-05	0.72 2181 6
chr22	cg01963885	TSS200	ARVCF;ARVCF;C22orf25;C22orf25	0.2331861	4.15E-05	0.72 2181 6
chr5	cg22792660		RP11-229C3.2;RP11-229C3.2	0.3347742	4.23E-05	0.722 1816
chr10	cg02360445			-0.1838432	4.42E-05	0.722 1816

chr17	cg24443925	1stExon;5'UTR	NCOR1;NCOR1;NCOR1;NCOR1;NCOR1;NCOR1;NCOR1;NCOR1;NCOR1;NCOR1;NCOR1	0.2554183	4.61E-05	0.721816
chr2	cg01757209	Body;Body	RBMS1;RBMS1;RBMS1;RBMS1;RBMS1;RBMS1;RBMS1	0.2008931	4.76E-05	0.721816
chr3	cg01783386	1stExon	RFT1;RFT1;RFT1	0.1924831	4.84E-05	0.721816
chr4	cg20656602	TSS200	TIFA;TIFA;ALPK1	0.1385673	5.09E-05	0.721816
chr12	cg26953122	TSS1500	ATF1;ATF1;ATF1;ATF1;ATF1	0.2323086	5.12E-05	0.721816
chr13	cg27059840			-0.3195289	5.18E-05	0.7221816
chr4	cg01380244			-0.1615248	5.29E-05	0.7221816
chr5	cg24359706	Body	AC034228.7;AC034228.7;AC034228.4;ACSL6	0.153018	5.35E-05	0.721816
chr10	cg10831391	5'UTR;1stExon	PFKP;PFKP;PFKP;PFKP;PFKP;PFKP	0.2691608	5.42E-05	0.721816
chr14	cg26398024			-0.1167855	5.49E-05	0.7221816
chr3	cg05876340	Body	CELSR3	-0.1746708	5.64E-05	0.721816
chr6	cg16961036			0.3303415	5.69E-05	0.7221816

chr12	cg07354583	Body;Body;Body	CD69;CD69	0.5748962	5.78E-05	0.7221816
chr19	cg05250932	3'UTR;3'UTR;3'UTR	TNNT1;TNNT1	0.3116843	5.78E-05	0.7221816
chr15	cg20749283		RP11-279F6.1;RP11-253M7.4;RP11-279F6.1	0.2019682	5.92E-05	0.7221816
chr3	cg11846236	1stExon	COL7A1;COL7A1	0.3848365	6.04E-05	0.7221816
chr17	cg24612420	1stExon;1stExon;5'UTR;5'UTR	ACLY;ACLY;ACLY;ACLY;ACLY;ACLY	0.152326	6.14E-05	0.7221816
chr5	cg07697561	TSS200;TSS200	DIAPH1;DIAPH1;DIAPH1;DIAPH1;AC008781.7	0.2172133	6.33E-05	0.7221816
chr17	cg15963109	Body	CTC-524C5.5	0.1706058	6.41E-05	0.7221816
chr8	cg11149658	Body	MCPH1;MCPH1	-0.1592459	6.44E-05	0.7221816
chr1	cg04471037	Body	MAST2;MAST2;MAST2	0.4942488	6.54E-05	0.7221816
chr4	cg27472384	Body;Body;Body;Body;Body;Body	RP11-323J4.1;RAP1GDS1;RAP1GDS1;RAP1GDS1	0.1546427	6.60E-05	0.7221816
chr5	cg24528447	TSS1500	NR2F1;RP11-65F13.2	0.243627	6.64E-05	0.7221816

chr5	cg12694122	TSS200;TSS200;TSS200	RFESD;RFESD;RFESD;RFESD;RFESD;RFESD	0.1646919	6.74E-05	0.7221816
chr7	cg21364231	TSS200	RP5-1129J21.3	0.1948986	6.88E-05	0.7221816
chr11	cg19792802	TSS200	CTSW;CTSW;CTSW;CTSW	0.1948959	6.89E-05	0.7221816
chr1	cg25742246		PROX1-AS1	0.3328949	6.93E-05	0.7221816
chr4	cg17935422	Body	FAM193A;FAM193A;FAM193A	-0.1919976	6.96E-05	0.7221816
chr5	cg12882476	1stExon;TSS200	CCDC112;CCDC112;CCDC112;CCDC112;CCDC112;CCDC112;CCDC112	0.1270453	7.06E-05	0.7221816
chr11	cg01596495	3'UTR	SMTNL1;SMTNL1	0.250578	7.40E-05	0.7221816
chr19	cg10750464	Body		0.5083369	7.44E-05	0.7221816
chr2	cg01136571	Body;TSS200	MPHOSPH10;MPHOSPH10;MCEE;MPHOSPH10;MCEE;MCEE	0.1984571	7.94E-05	0.7221816
chr11	cg10950661		RCN1;RCN1	0.2631358	7.96E-05	0.7221816
chr3	cg13337095	TSS1500;TSS1500;TSS1500	LRRC34;LRRC34;LRRC34;LRRC34;LRRC34;LRRC34;LRRC34	0.602104	7.98E-05	0.7221816

chr5	cg09835486	5'UTR;1stExon	REEP5;REEP5;REEP5;REEP5;REEP5;REEP5;REEP5;REEP5;REEP5	0.2681608	8.04E-05	0.721816
chr22	cg19737037	1stExon	TNFRSF13C	0.1924308	8.21E-05	0.721816
chr13	cg18170989	5'UTR;5'UTR;1stExon	CAB39L;CAB39L;CAB39L;CAB39L;CAB39L;CAB39L;CAB39L;CAB39L;CAB39L;CAB39L	0.2798335	8.34E-05	0.721816
chr6	cg12611423	TSS200	FOXF2	0.2145424	8.36E-05	0.721816
chr15	cg23386807	TSS200;TSS200	KIAA0101;KIAA0101;KIAA0101;KIAA0101;KIAA0101	0.1410559	8.49E-05	0.721816
chr19	cg12559208	Body;Body	TMC4;TMC4;TMC4;TMC4;TMC4;TMC4	0.4752392	8.63E-05	0.721816
chr20	cg14002969	5'UTR;5'UTR	PTPRA;PTPRA;PTPRA;PTPRA;PTPRA;PTPRA;PTPRA;PTPRA;PTPRA;PTPRA	0.242281	8.64E-05	0.721816
chr4	cg05295810	TSS200;TSS200	CASP6;CASP6;CASP6;CASP6	0.209682	8.65E-05	0.721816
chr21	cg14757344	1stExon;5'UTR	RRP1;RRP1;RRP1;RRP1;RRP1;RRP1;RRP1;RRP1;RRP1;RRP1;RRP1;RRP1;RRP1	0.1520014	8.96E-05	0.721816
chr17	cg20131685	TSS1500	LLGL1	0.1518584	9.16E-05	0.721816
chr3	cg10610461	TSS1500	PLCL2;PLCL2	-0.172967	9.22E-05	0.721816

chr7	cg10749792	5'UTR;1stExon;TSS200;TSS200	CCT6A;CCT6A;PSPH;PSPH;CCT6A;PSPH;PSPH;PSPH;CCT6A;PSPH;PSPH	0.2257794	9.25E-05	0.721816
chr13	cg20807920	Body;5'UTR;5'UTR;5'UTR	TEX30;TEX30;TEX30;TEX30;TEX30	0.1862487	9.29E-05	0.721816
chr13	cg00282249	TSS1500;TSS1500;TSS1500;TSS1500	CCNA1;CCNA1;CCNA1;CCNA1	0.2127272	9.35E-05	0.721816
chr12	cg14787758	Body		-0.2115884	9.41E-05	0.721816
chr10	cg01017197	TSS1500;TSS200	BLOC1S2;BLOC1S2;BLOC1S2	0.2004071	9.52E-05	0.721816
chr15	ch.15.1614682R		RP11-152L20.3	0.1761104	9.64E-05	0.7221816
chr3	cg12448161	TSS200	COL7A1;COL7A1	0.55151	9.76E-05	0.721816
chr5	cg27381216	Body;TSS1500;5'UTR	FASTKD3;MTRR;MTRR;MTRR;MTRR;MTRR;MTRR;FASTKD3;FASTKD3;FASTKD3;MTRR;FASTKD3;FASTKD3;MTRR;MTRR;MTRR;MTRR	0.2558947	9.77E-05	0.721816
chr11	cg02787737			0.3150117	0.0001004	0.7221816
chr2	cg25809290	Body;Body;Body;Body;Body;Body		-0.1913073	0.0001013	0.721816
chr15	cg26590064	Body	UBAP1L	0.3539493	0.0001039	0.721816

chr3	cg18043455	Body	SEC61A1;SEC61A1;SEC61A1;SEC61A1	0.1795501	0.0001039	0.721816
chr15	cg18374220	TSS200	MESDC1	0.1196583	0.0001054	0.721816
chr11	cg21909343	Body;TSS1500;Body	RP11-856B14.1;SSSCA1;RP11-856B14.1	0.1966725	0.0001066	0.721816
chr16	cg27508904			-0.1757123	0.0001067	0.7221816
chr17	cg07433755	Body;Body;Body;Body;Body;Body;Body;Body;Body;Body;Body	9-Sep	-0.1695896	0.0001069	0.721816
chr1	cg09736653		U6	0.1835041	0.0001074	0.7221816
chr14	cg01849466	Body	ZFYVE21;ZFYVE21;ZFYVE21;Y_RNA;ZFYVE21;ZFYVE21	-0.4209564	0.0001077	0.721816
chr2	cg23817281		AC010980.2;AC010980.2;AC010980.2	0.1900822	0.0001099	0.7221816
chr14	cg16258702	Body		-0.1559823	0.0001102	0.721816
chr16	cg04784519	TSS1500	PRR25	-0.3054146	0.0001111	0.721816
chr1	cg08853050	5'UTR;5'UTR;5'UTR;Body	PSMA5;PSMA5;PSMA5	0.1587776	0.0001119	0.721816

chr15	cg14590214	Body	RP11-505E24.3;LRRK1;LRRK1;LRRK1	-0.1382811	0.0001122	0.72 2181 6
chr7	cg04828509	TSS200	ARPC1A;ARPC1A;ARPC1A	0.1374546	0.0001123	0.72 2181 6
chr14	cg00527781	TSS1500;TSS1500;TSS1500;TSS1500	BCL11B;BCL11B;BCL11B	0.2116534	0.0001144	0.72 2181 6
chr5	cg04587428	Body		-0.2021215	0.0001159	0.72 2181 6
chr7	cg27089483	Body;TSS200	PRKAG2;RP11-796I2.2;PRKAG2;RP11-796I2.2;PRKAG2	0.2184176	0.0001162	0.72 2181 6
chr16	cg00394385			-0.2024797	0.0001172	0.722 1816
chr1	cg00737943	Body;Body		-0.2416197	0.0001186	0.72 2181 6
chr14	cg14288330	TSS200	INSM2	0.1509122	0.0001194	0.72 2181 6
chr19	cg06808897	Body;Body		0.2590266	0.0001201	0.72 2181 6
chr7	cg14597504	Body;Body;Body	MAD1L1	-0.1779169	0.0001207	0.72 2181 6
chr3	cg16357381	TSS1500	COL7A1;COL7A1	0.3326093	0.0001215	0.72 2181 6

chr5	cg01984450	TSS200;TSS200;TSS200;TSS200;TSS200;TSS200;TSS200;TSS200;TSS200;TSS200;TSS200;Body	STARD4;STARD4;STARD4;STARD4;STARD4;STARD4;CTC-426B10.1	0.1698113	0.000122	0.721816
chr6	cg19694566		RPS6KA2	-0.175545	0.0001228	0.7221816
chr1	cg26013553	1stExon	KCNA3	0.2641495	0.000124	0.721816
chr11	cg22312298	5'UTR;5'UTR	SERPINH1;SERPINH1;SERPINH1;SERPINH1;SERPINH1;SERPINH1;SERPINH1;SERPINH1;SERPINH1;SERPINH1;SERPINH1;SERPINH1;SERPINH1	0.1746852	0.0001251	0.721816
chr7	cg21189070	TSS1500	ACTB;ACTB;ACTB;ACTB;ACTB;ACTB;ACTB;ACTB;ACTB;ACTB;ACTB	0.1950594	0.0001254	0.721816
chr1	cg18023674	Body	RP3-395M20.2;PLCH2	-0.2016577	0.000126	0.721816
chr7	cg13381243	Body;Body;Body	C7orf50	-0.1595054	0.0001266	0.721816
chr15	cg17352215			0.2081341	0.0001269	0.7221816
chr16	cg27294163	Body	CBFA2T3;CBFA2T3	-0.2415348	0.0001284	0.721816
chr2	cg24171337	5'UTR;5'UTR	AC012360.4;TGFBRAP1	0.3993531	0.0001289	0.721816

chr18	cg20536512	TSS1500	MYL12A	0.103201	0.0001316	0.7221816
chr13	cg25868998			0.1246496	0.0001322	0.7221816
chr7	cg21408734			-0.2168736	0.0001344	0.7221816
chr5	cg22883995	Body	CXXC5	-0.1498543	0.0001359	0.7221816
chr2	cg22880456	Body	ACTR1B	-0.1389594	0.0001361	0.7221816
chr8	cg22728385			0.1982551	0.0001375	0.7221816
chr1	cg24161057	TSS200;TSS200;TSS200	TSTD1;TSTD1;F11R;TSTD1;TSTD1;F11R;TSTD1;F11R;TSTD1;TSTD1;F11R	0.182348	0.0001382	0.7221816
chr14	cg25989668	5'UTR;1stExon	TRIP11;TRIP11;TRIP11	0.1128696	0.0001391	0.7221816
chr2	cg17786201	Body;Body;Body;Body	AC093732.1;TTC7A;TTC7A;TTC7A;TTC7A;TTC7A;TTC7A;TTC7A;TTC7A	-0.1370715	0.0001409	0.7221816
chr2	cg24343835	TSS200	MIR216A;AC011306.2	-0.2486377	0.0001409	0.7221816
chr9	cg08758850	5'UTR;1stExon;5'UTR;1stExon	NR6A1;NR6A1;NR6A1;NR6A1;NR6A1;NR6A1;NR6A1;NR6A1;RP11-175D17.3	0.2386124	0.0001411	0.7221816
chr16	cg22188029	TSS200	RAB11FIP3;RAB11FIP3;RAB11FIP3	0.1401935	0.0001412	0.7221816

chr5	cg05593411	Body;TSS1500;Body	PDE4D;PDE4D;PDE4D;PDE4D;PDE4D	0.2877383	0.0001418	0.7221816
chr17	cg21303745	Body	TBC1D16	-0.1636589	0.0001436	0.7221816
chr11	cg08360511	Body		0.3234116	0.0001455	0.7221816
chr16	cg05806333	TSS1500	FBXL16;FBXL16;MSLN	0.1719141	0.0001457	0.7221816
chr2	cg05373455	5'UTR	RFX8;RFX8;RFX8	0.1315142	0.0001496	0.7221816
chr13	cg08157336	Body;Body;Body		0.1822963	0.0001497	0.7221816
chr7	cg03844499	5'UTR;1stExon	C7orf41;C7orf41;C7orf41;C7orf41	0.143254	0.0001504	0.7221816
chr1	cg25844211	TSS1500;5'UTR;5'UTR	URB2;TAF5L;TAF5L;TAF5L	0.1417365	0.0001517	0.7221816
chr16	cg14673706			0.2320177	0.0001527	0.7221816
chr3	cg17402072	5'UTR;5'UTR;1stExon;1stExon;1stExon;5'UTR	FXR1;FXR1;FXR1;FXR1;FXR1;FXR1;FXR1;FXR1;FXR1;FXR1;FXR1;FXR1;FXR1;FXR1	0.2318893	0.0001535	0.7221816
chr2	cg08057136	TSS200	AC010731.4;AC010731.4	0.2671756	0.0001565	0.7221816

chr7	cg13941256	Body;Body	SVOPL;SVOPL	-0.1819951	0.000157	0.7221816
chr1	cg19651159		RP11-15I11.2	0.2095153	0.000159	0.7221816
chr7	cg21110052	5'UTR;1stExon	CHN2;CHN2;CHN2;CHN2;CHN2;CHN2;CPVL;CPVL;CPVL;CPVL;CPVL	0.1520721	0.0001591	0.7221816
chr8	cg02679502	Body;TSS200	CTHRC1	0.2419729	0.0001594	0.7221816
chr10	cg08013396			-0.2403591	0.0001599	0.7221816
chr17	cg21448507	TSS200	NXPH3;NXPH3;NXPH3;RP5-1029K10.4	0.1340987	0.0001606	0.7221816
chr6	cg03118206	TSS200	KIAA1244	0.2075714	0.0001609	0.7221816
chr7	cg19420101	Body	PDAP1;PDAP1	-0.1208732	0.0001609	0.7221816
chr1	cg00563824			0.4762241	0.0001612	0.7221816
chr11	cg21144158	TSS1500	MIR612;NEAT1	0.2578973	0.0001627	0.7221816
chr2	cg14014890	Body	PQLC3;PQLC3;PQLC3	0.2585234	0.0001632	0.7221816
chr12	cg17480870			-0.176869	0.0001641	0.7221816

chr9	cg11635143	TSS1500;1stExon;1stExon;5'UTR;5'UTR	C9orf85;C9orf85;C9orf85;C9orf85;C9orf85;C9orf85;FAM108B1;FAM108B1;FAM108B1	0.3381199	0.0001644	0.721816
chr17	cg05269713	Body;Body	TMEM92	-0.1519428	0.0001655	0.721816
chr7	cg11236311	TSS1500	RINT1;RINT1;RINT1;RINT1;RINT1	0.5214259	0.0001665	0.721816
chr17	cg14248106	TSS1500	C1QL1	0.1931235	0.0001693	0.721816
chr8	cg15740790	TSS200;5'UTR;5'UTR;5'UTR	SLC39A14;SLC39A14;SLC39A14;SLC39A14;SLC39A14;SLC39A14	0.1836123	0.0001695	0.721816
chr7	cg25679366	5'UTR;5'UTR;5'UTR	ABCB4;ABCB4;ABCB4;ABCB4;ABCB4;ABCB4	0.1321301	0.0001695	0.721816
chr2	cg16948082	TSS200	PER2;PER2;PER2	0.1968837	0.0001712	0.721816
chr17	cg19354750	TSS200	ACE;ACE;ACE	0.1928601	0.0001714	0.721816
chr12	cg02510471	Body	HOTAIR;HOTAIR	0.2325304	0.0001732	0.721816
chr3	cg15243630	TSS200	GPD1L;GPD1L;GPD1L;GPD1L;GPD1L	0.1596192	0.0001749	0.721816
chr10	cg17529598	Body;Body;Body	RP11-202P11.1;RP11-202P11.1	-0.155785	0.0001751	0.721816

chr2	cg16675700	TSS200;TSS200;TSS200	ANTXR1;ANTXR1;ANTXR1;ANTXR1	0.1470391	0.0001752	0.7221816
chr6	cg11898431			0.2181247	0.0001762	0.7221816
chr17	cg23823134	5'UTR;1stExon	RPL19;RPL19	0.1215822	0.0001762	0.7221816
chr10	cg26685590	TSS200	BNIP3;BNIP3	0.1291596	0.0001765	0.7221816
chr20	cg03733979			-0.1768796	0.0001817	0.7221816
chr1	cg23280418	TSS1500	TMEM167B;TMEM167B;TMEM167B	0.4633346	0.0001842	0.7221816
chr3	cg13560548	TSS1500;TSS1500	C3orf24;C3orf24;C3orf24	0.2138671	0.0001851	0.7221816
chr14	cg09880040	Body	RP11-1077A2.1;RP11-1077A2.1;RP11-1077A2.1;RP11-1077A2.1	0.2474894	0.0001852	0.7221816
chr19	cg11778253	3'UTR	PPP1R12C;PPP1R12C	-0.2071606	0.0001853	0.7221816
chr5	cg09491410		CTD-2532K18.1	0.1849249	0.0001854	0.7221816
chr1	cg25621096	5'UTR;1stExon;5'UTR;TSS200;1stExon	CDC42SE1;CDC42SE1;MLLT11;MLLT11;CDC42SE1;CDC42SE1;RP11-316M1.11;CDC42SE1;CDC42SE1;CDC42SE1;CDC42SE1	0.3193029	0.0001856	0.7221816
chr6	cg13875840			0.1854492	0.0001874	0.7221816

chr2	cg25810927	TSS200	SUCLG1;SUCLG1;SUCLG1;SUCLG1;SUCLG1	0.1324979	0.0001882	0.721816
chr6	cg27123071	Body	TRIM31;TRIM31;TRIM31;TRIM31-AS1;TRIM31	0.406499	0.0001885	0.721816
chr14	cg19611807		C14orf37	0.3533567	0.0001888	0.7221816
chr3	cg18503679	1stExon;5'UTR	ZNF385D;ZNF385D;ZNF385D;ZNF385D;ZNF385D;ZNF385D;ZNF385D	0.4517596	0.0001899	0.721816
chr1	cg07906103	5'UTR	PIGR	-0.1535327	0.00019	0.721816
chr11	cg22015966	TSS200	ARFGAP2;ARFGAP2;ARFGAP2;ARFGAP2;ARFGAP2;ARFGAP2;ARFGAP2;ARFGAP2;ARFGAP2;ARFGAP2;ARFGAP2;ARFGAP2;ARFGAP2;ARFGAP2;ARFGAP2;ARFGAP2;ARFGAP2;ARFGAP2	0.2217575	0.0001911	0.721816
chr16	cg27013478	TSS200;TSS200	FLYWCH1;FLYWCH1;FLYWCH1;FLYWCH1;FLYWCH1;FLYWCH1;FLYWCH1	0.1615098	0.000192	0.721816
chr6	cg13365340	1stExon	B3GALT4	0.1446758	0.0001945	0.721816
chr1	cg16651196	TSS200	BMP8A	0.1423423	0.0001945	0.721816
chr7	cg13379332			-0.1453714	0.0001949	0.7221816

chr7	cg14582691	Body;Body;Body		-0.1698818	0.0001977	0.721816
chr10	cg05132851			-0.2011589	0.000198	0.7221816
chr5	cg06025835	Body;Body;Body;Body;Body	PDE8B	0.2149196	0.0001985	0.721816
chr17	cg26422469	Body		-0.1348482	0.0002031	0.721816
chr1	cg22957381	TSS1500;TSS1500	RP11-88H9.2;RP11-88H9.2;RP11-88H9.1;KCND3	0.144709	0.0002036	0.721816
chr11	cg26821848	TSS1500;TSS1500	DNHD1;DNHD1;DNHD1;DNHD1;DNHD1;FXC1;FXC1	-0.1256386	0.0002041	0.721816
chr11	cg01368777	Body;Body;5'UTR;Body;TSS200	IGF2;INS-IGF2;IGF2;IGF2;IGF2;IGF2	0.1508396	0.0002047	0.721816
chr19	cg05192108	TSS1500;TSS1500;TSS200;TSS200;TSS1500;TSS1500;TSS1500;TSS1500	TSEN34;MBOAT7;MBOAT7;MBOAT7;MBOAT7;MBOAT7;TSEN34;MBOAT7;MBOAT7;TSEN34;MBOAT7;TSEN34;MBOAT7;TSEN34;TSEN34	0.149541	0.0002059	0.721816
chr4	cg06393290	TSS1500;TSS1500;TSS1500	FGFRL1;FGFRL1;FGFRL1;FGFRL1;FGFRL1;FGFRL1;FGFRL1;FGFRL1	0.2068853	0.0002062	0.721816
chr3	cg19474418	5'UTR;1stExon;5'UTR;5'UTR;TSS1500;TSS1500	TADA3;ARPC4;ARPC4;ARPC4;ARPC4;TADA3;TADA3;ARPC4;ARPC4;ARPC4;ARPC4;ARPC4-TTLL3;ARPC4;TADA3;TADA3;TADA3	0.154934	0.0002076	0.721816

chr11	cg02681040	TSS200;Body	C11orf36;C11orf36;C11orf36;MRGPRG	-0.2073752	0.0002084	0.721816
chr17	cg25719092	5'UTR;5'UTR	CRHR1;CRHR1	-0.3220443	0.0002106	0.721816
chr3	cg15141371	1stExon;5'UTR	CSRNP1;CSRNP1;CSRNP1;CSRNP1	0.1960148	0.0002114	0.721816
chr8	cg20980783		TCF24	0.2725754	0.0002116	0.7221816
chr6	cg11439592	Body		0.2898715	0.000213	0.721816
chr4	cg25766271		PPP2R2C	0.2587762	0.0002153	0.7221816
chr7	cg21403089	TSS200	PNPLA8;PNPLA8;PNPLA8;PNPLA8;PNPLA8;PNPLA8;PNPLA8;PNPLA8;PNPLA8;PNPLA8;PNPLA8	0.1331002	0.0002156	0.721816
chr6	cg04271792		XXbac-BPG308K3.6	0.4026094	0.000216	0.7221816
chr4	cg02464357	TSS200;TSS200	HERC3;PIGY;HERC3	0.1406216	0.0002162	0.721816
chr9	cg01541867			0.1198827	0.0002168	0.7221816
chr15	cg24912334	Body	PEAK1	0.2083755	0.000217	0.721816
chr4	cg13213598	5'UTR	FAM53A;Y_RNA;FAM53A;FAM53A;FAM53A;FAM53A	0.3850472	0.0002174	0.721816

chr6	cg25067245			0.3378271	0.0002206	0.722 1816
chr17	cg09596173	TSS200	GALK1;GALK1;GALK1	0.1806726	0.0002212	0.72 2181 6
chr6	cg07914200			0.1764577	0.0002218	0.722 1816
chr2	cg01273987	Body		0.2490842	0.0002243	0.72 2181 6
chr3	cg20559608	TSS200	CPNE4;CPNE4;CPNE4;CPNE4;CPNE4	0.1369015	0.0002244	0.72 2181 6
chr17	cg21506059	TSS1500	HELZ;HELZ	0.1717941	0.0002244	0.72 2181 6
chr5	cg20435267	Body	RP11-281O15.3	0.2272336	0.0002253	0.72 2181 6
chr4	cg05193411	5'UTR;TSS1500;T SS200	LARP7;C4orf21;C4orf21;C4orf21;LARP 7;C4orf21;LARP7;LARP7;LARP7;LAR P7;C4orf21;LARP7;C4orf21;LARP7;LA RP7	0.1966957	0.0002268	0.72 2181 6
chr22	cg17487553	TSS200;TSS200;T SS200	PANX2;PANX2	0.3048271	0.0002277	0.72 2181 6
chr21	cg07511459	Body;Body;Body	BRWD1;BRWD1;BRWD1;BRWD1;TM PRSS3	0.1517795	0.0002291	0.72 2181 6
chr1	cg21449897			- 0.2322685	0.0002296	0.722 1816

chr22	cg22896904	1stExon;5'UTR	ANKRD54;ANKRD54;ANKRD54;ANKRD54;ANKRD54;ANKRD54;ANKRD54;ANKRD54;ANKRD54;ANKRD54	0.1518594	0.0002306	0.721816
chr9	cg00587003	TSS1500;Body;Body;Body;Body;Body;Body;Body;Body;Body;Body;Body;Body;Body	CDKN2A;CDKN2A;CDKN2A;CDKN2A;CDKN2A;CDKN2A;CDKN2B-AS1;MTAP	0.1643566	0.0002325	0.721816
chr16	cg16713168	Body		-0.2552117	0.0002326	0.721816
chr2	cg09724301	5'UTR;5'UTR	GAD1;GAD1;GAD1;AC007405.8;AC007405.8;GAD1;GAD1;GAD1;GAD1;GAD1;AC007405.8;GAD1;GAD1;GAD1	0.1272472	0.0002332	0.721816
chr5	cg17413750			-0.1569059	0.0002336	0.7221816
chr12	cg06203772		WNT5B;WNT5B	-0.1829912	0.0002338	0.7221816
chr14	cg16774604	1stExon	CTD-2555K7.2;OXA1L;OXA1L;CTD-2555K7.2;CTD-2555K7.2;CTD-2555K7.2;CTD-2555K7.2;OXA1L;OXA1L;OXA1L;OXA1L;OXA1L;OXA1L;OXA1L;OXA1L;OXA1L;CTD-2555K7.2;CTD-2555K7.2;CTD-2555K7.2	0.1497433	0.0002342	0.721816
chr7	cg14873985	Body	SRCRB4D	0.1319751	0.0002354	0.721816
chr22	cg26395601	5'UTR;5'UTR;TSS200;TSS200;1stExon;1stExon	THOC5;THOC5;THOC5;THOC5;THOC5;THOC5;THOC5;THOC5;THOC5;THOC5;THOC5	0.18765	0.0002367	0.721816

chr1	cg20160182	5'UTR	DDX59;DDX59;DDX59;DDX59;DDX59;RP11-92G12.3	0.1989676	0.000237	0.7221816
chr7	cg13049357		CAMK2B	-0.2274402	0.0002379	0.7221816
chr20	cg26172013	5'UTR;1stExon	SNTA1;SNTA1	0.1766764	0.0002389	0.7221816
chr11	cg14797402		GLYATL1;GLYATL1	0.3259954	0.0002394	0.7221816
chr10	cg17138769	Body		0.2217256	0.0002402	0.7221816
chr17	cg20361594	Body	PFAS	-0.1588709	0.0002407	0.7221816
chr11	cg17568129	TSS1500;Body;TSS1500	AASDHPPT;AASDHPPT;KBTBD3;KBTBD3;KBTBD3;KBTBD3;KBTBD3;KBTBD3	0.1367822	0.0002421	0.7221816
chr15	cg07956058	Body;5'UTR	CLK3;CLK3;CLK3;CLK3;CLK3;CLK3;CLK3;CLK3;CLK3;CLK3;CLK3	-0.1991434	0.0002424	0.7221816
chr15	cg03043296	Body	AP3B2;RP11-752G15.3	0.2500654	0.0002424	0.7221816
chr1	cg27099896	5'UTR;1stExon;1stExon;5'UTR;TSS200;Body;Body;5'UTR;5'UTR	GPR161;GPR161;GPR161;GPR161;GPR161;GPR161;GPR161	0.164911	0.0002431	0.7221816
chr14	cg01168865	Body;Body;Body	RP11-537P22.1;RP11-537P22.1;SLC25A21	0.2719233	0.0002444	0.7221816

chr9	cg04651914	1stExon	C9orf64;C9orf64;C9orf64	0.2981995	0.0002449	0.72 2181 6
chr4	cg24322144	TSS200	TMEM128;TMEM128;TMEM128	0.1397687	0.0002462	0.72 2181 6
chr4	cg26348902		NKX1-1	0.2639546	0.0002468	0.722 1816
chr7	cg06851224	1stExon	NOM1	0.1496549	0.0002479	0.72 2181 6
chr8	cg07085824	TSS1500	SGK196;SGK196	0.1356155	0.0002516	0.72 2181 6
chr1	cg15988263	TSS200	IPO13;IPO13;IPO13;RP11-7O11.3;RP11-7O11.3;RP11-7O11.3	0.1143326	0.0002528	0.72 2181 6
chr1	cg25379026	1stExon;1stExon;1stExon;5'UTR;5'UTR;5'UTR	DNAH14;DNAH14;DNAH14;DNAH14;DNAH14;DNAH14;DNAH14;DNAH14;DNAH14	0.2158711	0.0002529	0.72 2181 6
chr7	cg09515778	TSS200;TSS200	SLC4A2;SLC4A2;SLC4A2;CDK5;CDK5;CDK5;CDK5;CDK5	0.1552353	0.0002535	0.72 2181 6
chr2	cg00436174	1stExon	ERCC3;ERCC3;ERCC3;ERCC3;ERCC3;ERCC3;ERCC3;ERCC3;ERCC3;ERCC3	0.2730407	0.0002539	0.72 2181 6
chr1	cg10015974			0.1703765	0.000255	0.722 1816
chr3	cg04442328	1stExon;5'UTR	SENP2;SENP2;SENP2;SENP2;SENP2;SENP2;SENP2;SENP2;SENP2;SENP2;SENP2;SENP2	0.2156387	0.0002555	0.72 2181 6

chr3	cg03369965	TSS1500	LRRC34;LRRC34;LRRC34;LRRC34;LRRC34;LRRC34;LRRC34	0.5806291	0.0002563	0.721816
chr20	cg24189721			0.4735162	0.0002575	0.7221816
chr1	cg22237937	Body	AIM1L;AIM1L;AIM1L;AIM1L	-0.1060314	0.0002586	0.721816
chr18	cg02427109	3'UTR;Body;Body	PARD6G	0.4054379	0.0002587	0.721816
chr3	cg14957943	TSS200	COL7A1;COL7A1	0.3266465	0.0002614	0.724858 7
chr8	cg11474778	TSS200	RAD54B;RAD54B;RAD54B;RAD54B	0.4392473	0.0002635	0.7248587
chr15	cg27425655		RP11-1007O24.3	0.2084561	0.0002639	0.7248587
chr6	cg14068796		XXbac-BPG308K3.6	0.2110546	0.0002654	0.7248587
chr10	cg19204631	TSS200;TSS1500	CISD1;CISD1;IPMK	0.1293855	0.0002655	0.7248587
chr22	cg21070172	1stExon	CELSR1	0.1597166	0.0002656	0.7248587
chr3	cg20745538			0.181602	0.0002675	0.7257639
chr3	cg09983683	TSS200;Body	IQSEC1;IQSEC1;IQSEC1	0.1477657	0.0002723	0.7257639

chr4	cg26262152	TSS1500;Body;Body	PIGG;PIGG;PIGG;PIGG;PIGG;PIGG;PIGG;ZNF721;PIGG;ZNF721	-0.0996799	0.0002746	0.725763 9
chr6	cg10459950	TSS200	PERP;PERP	0.2783674	0.0002773	0.725763 9
chr15	cg15421821	1stExon;1stExon;TSS200;5'UTR;5'UTR	BTBD1;BTBD1;BTBD1;RP11-382A20.5;RP11-382A20.6	0.1029812	0.0002773	0.725763 9
chr15	cg01645753	TSS200	CPEB1;CPEB1;CPEB1;RP11-752G15.3;CPEB1;CPEB1;CPEB1;CPEB1;RP11-752G15.3	0.327363	0.0002777	0.725763 9
chr8	cg20135243	5'UTR;5'UTR;5'UTR;5'UTR	PTK2B;PTK2B;PTK2B;PTK2B;PTK2B;PTK2B;PTK2B	0.1281889	0.0002788	0.725763 9
chr4	cg20028233	Body;Body;Body;Body;Body;Body	CAMK2D;CAMK2D	0.2423798	0.0002807	0.725763 9
chr11	cg07348535	Body	POLD4;POLD4;AP003419.11	0.1848951	0.000281	0.725763 9
chr19	cg17122153	5'UTR;5'UTR;1stExon	FAM125A;FAM125A;FAM125A;FAM125A;FAM125A;FAM125A;FAM125A;FAM125A;FAM125A;FAM125A;FAM125A;FAM125A	0.1390659	0.0002841	0.725763 9
chr2	cg09708295	Body		-0.2658135	0.0002849	0.725763 9
chr12	cg06782606			0.2156788	0.0002855	0.725 7639
chr4	cg04360780	1stExon	WHSC2;WHSC2;WHSC2;WHSC2;WHSC2;WHSC2;WHSC2;WHSC2;WHSC2;WHSC2;WHSC2	0.1108177	0.0002863	0.725763 9

chr18	cg20029745	Body;Body		0.221554	0.0002868	0.725763 9
chr10	cg03820205	TSS200	VDAC2;VDAC2;VDAC2;VDAC2;VDAC2;VDAC2;VDAC2;VDAC2;VDAC2;VDAC2;VDAC2;VDAC2;VDAC2	0.2753849	0.0002902	0.725763 9
chr4	cg06532184	TSS1500;TSS1500;Body	RP11-783N5.1;RP11-783N5.1;RP11-783N5.1;TAPT1;TAPT1;TAPT1;TAPT1;TAPT1	0.182461	0.0002902	0.725763 9
chr4	cg26678138			0.515111	0.0002919	0.725 7639
chr4	cg20530223	5'UTR;5'UTR	KLHL5;KLHL5	0.1422506	0.0002923	0.725763 9
chr19	cg09425032		C19orf67;C19orf67;C19orf67;C19orf67;C19orf67	0.2199356	0.0002925	0.725 7639
chr19	cg14558639	Body;Body		0.2928187	0.0002932	0.725763 9
chr15	cg08889009		RP11-30K9.5;RNF111;RP11-30K9.5	0.2696262	0.0002936	0.725 7639
chr7	cg04024839	TSS200	ZKSCAN1;ZKSCAN1;ZKSCAN1;ZKSCAN1;ZKSCAN1	0.1383138	0.0002939	0.725763 9
chr1	cg03151917	1stExon;1stExon;1stExon;1stExon;5'UTR;5'UTR;5'UTR;5'UTR	RBMXL1;RBMXL1;CCBL2;CCBL2;RBMXL1;CCBL2;CCBL2;RBMXL1;RBMXL1;RBMXL1;CCBL2;CCBL2	0.2423398	0.0002943	0.725763 9
chr9	cg03409832	Body	C9orf85;C9orf85	0.1490674	0.0002947	0.725763 9

chr3	cg17237320	TSS1500	AC022007.5;AC022007.5;AC022007.5;AC022007.5;AC022007.5;TMEM111	0.2066783	0.0002949	0.725763 9
chr17	cg13428561	Body		0.3129817	0.0002957	0.725763 9
chr16	cg09492451	Body	CTD-3032H12.2	0.3404571	0.0002961	0.725763 9
chr5	cg26880493	Body	PDZD2;PDZD2	0.1872584	0.0002989	0.725763 9
chr8	cg09440788	TSS1500;TSS1500	RAB11FIP1;RAB11FIP1;RAB11FIP1	0.2094079	0.0003005	0.725763 9
chr1	cg12477165	5'UTR	MTF1;MTF1	0.1330704	0.0003021	0.725763 9
chr5	cg14537507	5'UTR;1stExon;1stExon;1stExon;5'UTR;1stExon;5'UTR;5'UTR;1stExon;1stExon;5'UTR;5'UTR	RAPGEF6;RAPGEF6;RAPGEF6;RAPGEF6;RAPGEF6;RAPGEF6;RAPGEF6;RAPGEF6;RAPGEF6;RAPGEF6;RAPGEF6	0.1488938	0.0003031	0.725763 9
chr12	cg12250883	TSS200	Y_RNA;PPM1H;PPM1H;PPM1H	0.1841861	0.0003046	0.725763 9
chr5	cg00322656	TSS1500;Body;Body;Body	PDE4D;PDE4D;PDE4D;PDE4D;PDE4D	0.2709562	0.0003049	0.725763 9
chr9	cg04778212	TSS1500	RP11-35N6.1	0.287177	0.0003053	0.725763 9

chr15	cg23991388	Body	ADAMTS17	-0.2271564	0.0003072	0.725763 9
chr6	cg22339775	TSS1500	HIST1H3E	0.3154099	0.0003081	0.725763 9
chr14	cg26085966	5'UTR;1stExon;1stExon;5'UTR	SYNE2;SYNE2;SYNE2;SYNE2;SYNE2;SYNE2;SYNE2;SYNE2;SYNE2	0.2787298	0.0003086	0.725763 9
chr19	cg09137630	TSS1500	ZC3H4	0.2010722	0.0003102	0.725763 9
chr14	cg22792862	1stExon;5'UTR;TSS1500	ATP6V1D;ATP6V1D;ATP6V1D;ATP6V1D;EIF2S1;ATP6V1D;ATP6V1D;ATP6V1D;ATP6V1D;EIF2S1;EIF2S1;EIF2S1;EIF2S1;EIF2S1;EIF2S1	0.2359748	0.0003109	0.725763 9
chr17	cg17170741	TSS200;TSS200	C17orf39;ATPAF2;ATPAF2;ATPAF2;ATPAF2;ATPAF2;C17orf39	0.1761396	0.0003141	0.725763 9
chr12	cg26493932	5'UTR;TSS1500	ZNF664;ZNF664;ZNF664;ZNF664;CCDC92;ZNF664;RP11-522N14.1;ZNF664;ZNF664;ZNF664;CCDC92;ZNF664;CCDC92;CCDC92;CCDC92;CCDC92;CCDC92;CCDC92;ZNF664	0.1590685	0.0003147	0.725763 9
chr11	cg25089598	Body;Body;Body	ILK;TAF10;ILK;ILK;RP11-732A19.2;ILK;ILK;ILK;ILK;ILK;ILK;ILK	-0.1589642	0.0003183	0.725763 9
chr14	cg11782714			-0.1408253	0.0003194	0.725 7639
chr22	cg08431931	TSS200	WBP2NL;WBP2NL;WBP2NL;WBP2NL	0.4500416	0.0003195	0.725763 9

chr16	cg05983723	Body	ST3GAL2;RP11-529K1.4	-0.1130516	0.0003199	0.7257639
chr8	cg24546549	1stExon;5'UTR;Body	TRAPPC9;TRAPPC9	0.1798262	0.0003202	0.7257639
chr7	cg12022583	TSS1500	WIPF3	0.2265092	0.0003205	0.7257639
chr17	cg00918917	TSS200;TSS200;TSS200;TSS200;TSS200;TSS200	STRADA	0.1785028	0.0003208	0.7257639
chr20	cg26831081	TSS200	TAF4;TAF4	0.1880148	0.0003226	0.7257639
chr7	cg23785719	TSS200	STK17A;STK17A	0.2291378	0.0003228	0.7257639
chr17	cg14328457	Body		0.2487148	0.0003234	0.7257639
chr22	cg24989447	TSS1500;Body;Body;Body	RP3-400N23.6;RP3-400N23.6	-0.6968354	0.0003252	0.7257639
chr11	cg02184673	TSS1500	CLMP;CTD-2216M2.1	0.1899975	0.000326	0.7257639
chr3	cg03298890	5'UTR;Body	TMCC1;TMCC1;TMCC1;TMCC1	-0.1261578	0.0003264	0.7257639
chr9	cg06254440	Body	LHX2	0.2200745	0.0003281	0.7257639

chr22	cg19194233	1stExon;5'UTR;TSS200	MLC1;MLC1;MLC1;MLC1;MLC1;MLC1;MLC1;MLC1;MLC1;MLC1;MLC1;MLC1	0.1967799	0.0003288	0.725763 9
chr22	cg12419862	Body		-0.9555567	0.0003305	0.725763 9
chr2	cg13718369			-0.1794005	0.0003329	0.7257639
chr2	cg17668949	TSS1500;TSS1500	AC096649.2;MIR933;ATF2;ATF2;ATF2;ATF2;ATF2;ATF2;ATF2	0.1012019	0.0003347	0.725763 9
chr1	cg10846285	1stExon;5'UTR	SRSF4;SRSF4;SRSF4;SRSF4	0.1648155	0.0003429	0.725763 9
chr3	cg04408595	5'UTR;5'UTR;TSS1500	GPR27;EIF4E3;EIF4E3;EIF4E3;EIF4E3	0.1078787	0.000343	0.725763 9
chr11	cg16705665	TSS200	RCOR2	0.1741309	0.0003436	0.725763 9
chr9	cg03528881	TSS200;TSS1500;TSS1500	ALG2;SEC61B;SEC61B;ALG2;ALG2;SEC61B	0.1381529	0.0003447	0.725763 9
chr1	cg13184886	1stExon	ZDHHC18	0.231718	0.0003463	0.725763 9
chr9	cg00045967	TSS200	ARRDC1;ARRDC1;ARRDC1;ARRDC1;ARRDC1;ARRDC1;ARRDC1	0.1752231	0.0003482	0.725763 9
chr2	cg19075129			0.1016206	0.0003486	0.7257639

chr7	cg12508286	TSS1500	EN2;AC008060.8	0.1951088	0.0003492	0.725763 9
chr19	cg11787367	1stExon	PSMD8	0.1098876	0.0003496	0.725763 9
chr18	cg21678445	Body		0.181592	0.0003518	0.725763 9
chr16	cg06330621	TSS200	RP11-482M8.1;ZCCHC14;ZCCHC14;RP11-482M8.1;ZCCHC14;ZCCHC14	0.206464	0.000356	0.725763 9
chr2	cg12163823	TSS1500	D2HGDH;D2HGDH;D2HGDH;D2HGDH;D2HGDH	0.5017511	0.0003562	0.725763 9
chr3	cg02724127	TSS200	PLXND1	0.2926823	0.0003566	0.725763 9
chr10	cg23881119	5'UTR;TSS1500;TSS1500	KIAA1217;KIAA1217;KIAA1217;KIAA1217	0.1744068	0.0003575	0.725763 9
chr6	cg09863441	1stExon;5'UTR	HTR1E;HTR1E;HTR1E	0.2574605	0.0003605	0.725763 9
chr7	cg21435902	TSS200;TSS1500;TSS200;TSS200;TSS1500;TSS1500	KRIT1;ANKIB1;KRIT1;KRIT1;KRIT1;KRIT1;KRIT1;KRIT1;KRIT1;KRIT1;KRIT1;KRIT1;KRIT1;KRIT1;KRIT1;KRIT1	0.1540721	0.0003612	0.725763 9
chr20	cg07019386		RP1-66N13.1	-0.3499309	0.0003619	0.725 7639
chr9	cg21210985	Body;TSS200	GNE;GNE;GNE	0.1498942	0.0003619	0.725763 9

chr16	cg08870933	5'UTR;Body;1stExon	RP11-498D10.4;ATXN1L;ATXN1L;ATXN1L;RP11-498D10.4;RP11-498D10.4;RP11-498D10.4;RP11-498D10.4	0.1831853	0.0003622	0.725763 9
chr11	cg10607599		GRIK4;AP002348.1	0.2539231	0.0003664	0.725 7639
chr17	cg06475633	Body	P2RX1	-0.192286	0.0003704	0.725763 9
chr21	cg04174787			-0.1723119	0.0003726	0.725 7639
chr8	cg01498503	Body;1stExon;5'UTR	HAS2-AS1;HAS2-AS1;HAS2-AS1;HAS2;HAS2;HAS2-AS1;HAS2;HAS2	0.1700828	0.000375	0.725763 9
chr11	cg16974037	5'UTR;5'UTR;1stExon;1stExon	SLC22A11;SLC22A11;SLC22A11;SLC22A11;SLC22A11;SLC22A11	-0.1587526	0.0003751	0.725763 9
chr8	cg27114138	TSS1500	TRIB1	0.1134308	0.000376	0.725763 9
chr6	cg08791234	3'UTR;3'UTR;TSS1500;TSS1500	RGL2;RGL2;RGL2;RGL2;RGL2;RGL2;RGL2;TAPBP	0.1626324	0.0003763	0.725763 9
chr8	cg06824641	TSS200	PPP3CC;PPP3CC;PPP3CC;PPP3CC;PPP3CC;PPP3CC	0.3041971	0.0003768	0.725763 9
chr17	cg05798107	TSS200;5'UTR;TSS200;1stExon	TUBG1;TUBG1;FAM134C;FAM134C	0.1908327	0.0003774	0.725763 9
chr10	cg10680376	Body;Body;Body	ABLIM1;ABLIM1;ABLIM1	0.401095	0.0003801	0.725763 9

chr16	cg21898752	TSS200;TSS1500	ATMIN;ATMIN;RP11-303E16.3;RP11-303E16.3;ATMIN;ATMIN;RP11-303E16.3;RP11-303E16.3	0.188753	0.0003802	0.7257639
chr16	cg01336629			-0.2643427	0.0003818	0.7257639
chr19	cg20655070			0.5676607	0.0003824	0.7257639
chr16	cg08289130			0.499708	0.000383	0.7257639
chr14	cg21231789	5'UTR;1stExon	KCNK13;KCNK13	0.1562807	0.0003832	0.7257639
chr19	cg26300975	TSS1500;5'UTR;1stExon;TSS1500	ZNF576;ZNF576;ZNF576;ZNF576;ZNF576;ZNF576;SRRM5;IRGQ;IRGQ	0.1520811	0.0003849	0.7257639
chr6	cg09910967	TSS200	HECA	0.107493	0.0003851	0.7257639
chr2	cg06103677	TSS200;TSS200;5'UTR;TSS200;5'UTR	SEPT2;SEPT2;SEPT2;SEPT2;SEPT2;SEPT2;SEPT2;SEPT2;HDLBP;HDLBP;SEPT2;SEPT2;HDLBP;SEPT2;HDLBP;SEPT2;SEPT2;SEPT2;SEPT2;HDLBP;SEPT2;SEPT2;SEPT2;SEPT2;HDLBP;SEPT2;SEPT2;SEPT2;SEPT2;SEPT2;SEPT2;SEPT2;SEPT2;SEPT2;HDLBP;HDLBP;SEPT2;SEPT2;HDLBP	0.1337559	0.0003864	0.7257639
chr12	cg07357346	Body	RP11-728G15.1	0.2494352	0.0003874	0.7257639
chr1	cg04310354	TSS1500	IFFO2;IFFO2;IFFO2	0.1580713	0.0003891	0.7257639

chr22	cg15242686	TSS1500		1.390862	0.00039	0.7257639
chr12	cg27174387	TSS200;Body	UNG;UNG;UNG;UNG;UNG;UNG	0.1140638	0.0003906	0.7257639
chr16	cg07917546			-0.1514777	0.0003913	0.7257639
chr17	cg03012468			-0.1581836	0.0003926	0.7257639
chr1	cg06807126	5'UTR;5'UTR	AKR1A1;AKR1A1;AKR1A1;AKR1A1;AKR1A1;AKR1A1;AKR1A1	0.1782422	0.0003929	0.7257639
chr7	cg01248108	ExonBnd;ExonBnd;Body;Body	MDFIC	0.1925859	0.000393	0.7257639
chr11	cg08831996	5'UTR;1stExon;TSS200;1stExon;5'UTR	MRE11A;MRE11A;MRE11A;MRE11A;MRE11A;ANKRD49;ANKRD49;MRE11A;MRE11A;ANKRD49;ANKRD49;MRE11A;MRE11A;MRE11A;MRE11A;ANKRD49;ANKRD49;ANKRD49;MRE11A;MRE11A;MRE11A;ANKRD49;MRE11A;MRE11A	0.1199503	0.000394	0.7257639
chr8	cg02503933	5'UTR;1stExon	RP11-37B2.1;RP11-37B2.1;RP11-37B2.1;RIPK2;RIPK2;RIPK2;RIPK2;RIPK2;RIPK2;RIPK2;RIPK2;RP11-37B2.1	0.2880171	0.0003947	0.7257639
chr11	cg02050903	1stExon;5'UTR;1stExon;5'UTR	EXT2;EXT2;EXT2;EXT2;EXT2;EXT2;EXT2;EXT2;EXT2	0.148076	0.0003954	0.7257639
chr3	cg03240200			-0.1222696	0.0003965	0.7257639

chr3	cg01815150	1stExon;5'UTR	SENP2;SENP2;SENP2;SENP2;SENP2;SENP2;SENP2;SENP2;SENP2;SENP2;SENP2;SENP2	0.2552211	0.0003967	0.725763 9
chr15	cg21348997	5'UTR;1stExon	RP11-210M15.2;ARNT2;ARNT2;ARNT2;ARNT2;ARNT2;ARNT2	0.2168759	0.0003972	0.725763 9
chr2	cg24704336	5'UTR;5'UTR;Body;Body;Body	MOB4;MOB4;HSPD1;MOB4;MOB4	0.1845907	0.0003976	0.725763 9
chr8	cg04293180	TSS1500	TSTA3;TSTA3;TSTA3;TSTA3;TSTA3;TSTA3;TSTA3;TSTA3;TSTA3;TSTA3;TSTA3;TSTA3	0.3181069	0.0003989	0.725763 9
chr4	cg23967249	TSS200	ELMOD2;ELMOD2;ELMOD2;ELMOD2;ELMOD2	0.1485791	0.000401	0.725763 9
chr12	cg13830870	TSS200	CLEC9A;CLEC9A;RP11-133L14.5;RP11-133L14.5	0.4961337	0.0004013	0.725763 9
chr8	cg10677468	TSS1500;TSS1500;TSS1500;TSS1500;TSS1500;TSS1500	LETM2;LETM2;LETM2;LETM2;LETM2;LETM2;LETM2;LETM2	0.1390033	0.0004018	0.725763 9
chr22	cg27424226	1stExon;5'UTR	H1F0;H1F0;H1F0	0.1331194	0.0004018	0.725763 9
chr6	cg02050354	5'UTR	GUCA1A;GUCA1A;GUCA1A;GUCA1A	-0.1484679	0.0004027	0.725763 9
chr15	cg08267442	TSS200	UNC45A;UNC45A;UNC45A	0.284228	0.0004031	0.725763 9

chr2	cg14719675	TSS1500	ADCY3;ADCY3;ADCY3	0.2808906	0.0004049	0.72 5763 9
chr19	cg21926077			0.1551041	0.0004051	0.725 7639
chr1	cg15579696			- 0.3173865	0.0004055	0.725 7639
chr9	cg07566304	Body;Body		0.1993107	0.000406	0.72 5763 9
chr19	cg17389835	TSS200;Body	EML2	0.1480154	0.0004061	0.72 5763 9
chr1	cg26750139	1stExon	ACBD6	0.1624031	0.0004062	0.72 5763 9
chr3	cg00368218			- 0.1315714	0.0004064	0.725 7639
chr9	cg14506032		RP11-473E2.4	- 0.2708512	0.0004065	0.725 7639
chr19	cg03552658	TSS1500;TSS200	CHMP2A	0.1118677	0.0004067	0.72 5763 9
chr7	cg21959181	TSS200;TSS1500; TSS200	SLC4A2;CDK5;CDK5;CDK5;SLC4A2; SLC4A2;CDK5;SLC4A2;SLC4A2;CDK 5	0.3004848	0.0004074	0.72 5763 9
chr20	cg19077271	Body;TSS200;TSS 1500	SNORA71D;RP4-564F22.2;RP4- 564F22.2;RP4-564F22.2;RP4- 564F22.2;RP4-564F22.2;RP4-564F22.2	0.1280198	0.0004087	0.72 5763 9
chr2	cg25685934	Body;TSS200	FER1L5;FER1L5;FER1L5;FER1L5;FER 1L5	- 0.1498067	0.0004098	0.72 5763 9

chr6	cg19497998			0.2200379	0.0004102	0.725 7639
chr4	cg06831790	Body	ADAMTS3	0.1872337	0.0004104	0.72 5763 9
chr14	cg02277999	TSS200;TSS200	SPATA7;SPATA7;SPATA7;SPATA7;SPATA7;SPATA7;SPATA7;SPATA7;SPATA7;SPATA7;SPATA7;SPATA7;SPATA7;SPATA7;SPATA7;SPATA7	0.1116336	0.0004113	0.72 5763 9
chr3	cg07849604	TSS200	TRH;TRH	0.2318999	0.0004131	0.72 5763 9
chr13	cg03072762		RP11-309I15.1	0.1213386	0.0004133	0.725 7639
chr12	cg01805480	Body	FBRSL1;FBRSL1;FBRSL1	0.2049166	0.0004134	0.72 5763 9
chr22	cg04717802	TSS200	WBP2NL;WBP2NL;WBP2NL;WBP2NL	0.5756124	0.0004134	0.72 5763 9
chr12	cg05404701	TSS200	RP11-530C5.1;PAWR;PAWR;PAWR;PAWR;PAWR;PAWR;PAWR	0.1248908	0.0004135	0.72 5763 9
chr6	cg00092682	Body;Body		0.1363732	0.0004147	0.72 5763 9
chr1	cg23512567	Body	GPR137B	0.2293135	0.0004152	0.72 5763 9
chr11	cg27218829	TSS1500;5'UTR;1stExon	SRPR;FOXRED1;SRPR;FOXRED1;FOXRED1;FOXRED1;FOXRED1;FOXRED1;FOXRED1;FOXRED1;FOXRED1;F	0.1341273	0.0004169	0.72 5763 9

			OXRED1;SRPR;SRPR;SRPR;SRPR;SRPR			
chr16	cg16563187			-0.1466426	0.0004174	0.7257639
chr14	cg23894571	TSS200;TSS200	RP11-173D9.3;RP11-173D9.3;RP11-173D9.3;RP11-173D9.3;PPP2R3C;PPP2R3C;PPP2R3C;PPP2R3C;PPP2R3C;PPP2R3C;PPP2R3C;PPP2R3C;PPP2R3C;PPP2R3C;PPP2R3C;RP11-173D9.3;RP11-173D9.3;PPP2R3C;RP11-173D9.3;KIAA0391;RP11-173D9.3;RP11-173D9.3;RP11-173D9.3;RP11-173D9.3;PPP2R3C;PPP2R3C;PPP2R3C;RP11-173D9.3	0.2294559	0.0004176	0.7257639
chr12	cg10192527	Body		-0.1266327	0.0004183	0.7257639
chr1	cg09247534	TSS1500;TSS1500	C1orf9	0.1869236	0.0004203	0.7257639
chr8	cg05637265	Body;Body		-0.1411683	0.000421	0.7257639
chr13	cg00859544	TSS200	GJA3	0.1190247	0.0004212	0.7257639
chr10	cg16654801	TSS200;Body;TSS200	EMX2;EMX2OS;EMX2OS;EMX2;EMX2;EMX2OS;EMX2OS;EMX2OS	0.1908178	0.0004215	0.7257639
chr1	cg05712296			0.2371168	0.0004216	0.7257639

chr8	cg11898212	TSS200;TSS200;TSS200	FBXO25;FBXO25;FBXO25;FBXO25	0.1424437	0.0004231	0.725763 9
chr3	cg05666842	Body	CHCHD6	0.1763854	0.0004236	0.725763 9
chr14	cg15302765	TSS200;5'UTR;5'UTR;5'UTR;1stExon;1stExon;1stExon;1stExon;1stExon;5'UTR;5'UTR;1stExon;5'UTR	JKAMP;C14orf149;C14orf149;JKAMP;JKAMP;JKAMP;JKAMP;JKAMP;JKAMP;C14orf149;C14orf149;JKAMP;JKAMP;JKAMP;JKAMP;JKAMP;JKAMP;JKAMP;RP11-701B16.2;JKAMP	0.1089024	0.0004249	0.725763 9
chr7	cg20994703	Body	ZNF862;ZNF862	-0.1140577	0.0004255	0.725763 9
chr1	cg14985478	TSS200;TSS200	ZNHIT6;ZNHIT6	0.1908667	0.0004256	0.725763 9
chr6	cg05575213			-0.1640229	0.0004261	0.725 7639
chr20	cg18598695		RP1-66N13.1	-0.3619253	0.0004284	0.725 7639
chr8	cg18065361	TSS200	ADHFE1;ADHFE1;ADHFE1;ADHFE1;ADHFE1;ADHFE1;ADHFE1;ADHFE1;ADHFE1;ADHFE1;ADHFE1;ADHFE1;ADHFE1	0.1318982	0.0004287	0.725763 9
chr1	cg00026909	5'UTR	DAB1;DAB1;DAB1	-0.2961862	0.0004288	0.725763 9
chr6	cg17746791	TSS200	PIM1	0.1758991	0.0004291	0.725763 9

chr1	cg02059391	TSS200	FOXJ3;FOXJ3;FOXJ3;FOXJ3	0.1028341	0.0004294	0.7257639
chr16	cg07055694		CDH11	0.2476839	0.0004301	0.7257639
chr12	cg13919694		SP7;SP7	0.1423411	0.0004316	0.7257639
chr7	cg09250959	5'UTR	POM121;POM121	-0.1228439	0.0004319	0.7257639
chr2	cg06745666	Body	SNRNP200;SNRNP200;SNRNP200;SNRNP200;SNRNP200	-0.1499169	0.0004321	0.7257639
chr10	cg04982951	TSS1500;TSS200;TSS200;TSS1500	RP11-379F12.3;GATA3;RP11-379F12.3;RP11-379F12.7;GATA3;GATA3;GATA3	0.2003155	0.0004333	0.7257639
chr20	cg00500770		RP1-66N13.1	-0.2791768	0.0004334	0.7257639
chr6	cg00474500		GRM4	-0.2280188	0.0004337	0.7257639
chr17	cg21487830		TOB1;TOB1;AC091062.2;AC091062.2;AC091062.2;TOB1;TOB1	0.2303225	0.0004339	0.7257639
chr22	cg06696028	Body	MTMR3	-0.1589883	0.0004374	0.7294977
chr15	cg02878040	TSS200;TSS200	SNAP23;SNAP23;SNAP23;SNAP23;SNAP23;SNAP23;SNAP23;SNAP23;SNAP23;SNAP23;SNAP23;SNAP23;SNAP23;SNAP23	0.1272244	0.0004383	0.7294977
chr6	cg13302181			-0.1246979	0.0004391	0.7294977

chr1	cg24936468	TSS1500	C1orf63;C1orf63;TMEM50A;TMEM50A;TMEM50A;TMEM50A;C1orf63;C1orf63;C1orf63;C1orf63;C1orf63;C1orf63	-0.1731233	0.0004408	0.730538
chr20	cg07246325	Body;Body;Body	HM13;HM13;HM13;HM13;HM13;HM13;HM13;HM13;HM13;HM13	-0.1219027	0.000443	0.7306558
chr19	cg07053469	1stExon	EID2B	0.1267892	0.0004445	0.7306558
chr12	cg23295636	Body;TSS1500	Y_RNA;RP11-834C11.3;RP11-834C11.6;RP11-834C11.5	0.1580833	0.0004445	0.7306558
chr11	cg02783889	TSS1500;TSS1500	SCUBE2;SCUBE2;SCUBE2;SCUBE2;SCUBE2	0.1658873	0.0004452	0.7306558
chr20	cg10615414	TSS200	SLC32A1	0.1524	0.0004459	0.7306558
chr5	cg22911074	Body;TSS1500	NSUN2;SRD5A1;SRD5A1;SRD5A1;SRD5A1	0.2237338	0.0004476	0.731764
chr16	cg03063703	Body		-0.1555918	0.0004515	0.7350114
chr5	cg12847007	Body;TSS1500	GALNT10;GALNT10;GALNT10;GALNT10;CTB-157D17.1;CTB-157D17.1;GALNT10	-0.1457504	0.0004516	0.7350114
chr15	cg18384926	TSS200;TSS200	C15orf48;C15orf48;C15orf48;C15orf48	0.2487379	0.0004532	0.7355783
chr19	cg12210543	TSS1500	ZNF844;ZNF844;ZNF844;ZNF844	0.216263	0.0004561	0.7355783

chr1	cg16732616	Body	DMRTA2	0.1962981	0.0004564	0.73 5578 3
chr6	cg15643000			- 0.1499603	0.0004587	0.735 5783
chr15	cg24449371	TSS1500	CIB2;CIB2;CIB2;CIB2;CIB2;CIB2;CIB 2;IDH3A	0.0991006	0.0004599	0.73 5578 3
chr1	cg25494767	TSS200	FBLIM1;FBLIM1;FBLIM1;FBLIM1;FB LIM1;FBLIM1;FBLIM1;FBLIM1;FBLI M1;FBLIM1;FBLIM1;FBLIM1;FBLIM1	0.2730841	0.0004618	0.73 5578 3
chr16	cg05057452	TSS1500	HBM;HBM;HBM	0.4831365	0.0004641	0.73 5578 3
chr7	cg02603686	1stExon;5'UTR;5' UTR	IMMP2L;IMMP2L;IMMP2L;IMMP2L;I MMP2L;IMMP2L;IMMP2L	0.1223162	0.0004657	0.73 5578 3
chr17	cg10667386	5'UTR	AKAP1;AKAP1;AKAP1;AKAP1;AKAP 1;RP11-166P13.3;RP11- 166P13.3;AKAP1;AKAP1;AKAP1	0.1048939	0.0004679	0.73 5578 3
chr14	cg16082877		CTD-2555C10.3	0.1941675	0.0004683	0.735 5783
chr6	cg09083108	Body;Body;Body; Body	PACRG	- 0.1698352	0.0004703	0.73 5578 3
chr16	cg01130978	3'UTR;3'UTR	COG8;PDF;RP11-343C2.8;COG8	0.154623	0.0004708	0.73 5578 3
chr5	cg19875969	Body;3'UTR;3'UT R;3'UTR;TSS200	FAM172A;FAM172A;MIR2277;FAM17 2A;FAM172A	0.1921734	0.0004723	0.73 5578 3

chr1	cg06642677	TSS1500	SNRPE;SNRPE;SNRPE;SNRPE	-0.1196501	0.0004726	0.7355783
chr8	cg20585841	Body;Body;Body;Body;Body;Body;Body;Body		-0.5357942	0.0004727	0.7355783
chr3	cg10935698	Body;Body;Body	RSRC1;RSRC1;RSRC1	0.1894876	0.0004731	0.7355783
chr11	cg06688411	TSS200	FRMD8;FRMD8;FRMD8;FRMD8;FRMD8;FRMD8;FRMD8	0.1855086	0.0004746	0.7355783
chr19	cg08179120	1stExon;Body	AC011484.1	0.2527745	0.0004754	0.7355783
chr6	cg00176718			0.2882567	0.0004758	0.7355783
chr1	cg10177197	3'UTR	DHCR24;DHCR24;RP11-67L3.6	0.1401685	0.0004768	0.7355783
chr4	cg06292277	TSS1500;TSS1500	CLCN3;CLCN3;CLCN3;CLCN3;CLCN3;CLCN3;CLCN3	0.1426992	0.0004787	0.7355783
chr13	cg17782972	TSS200	TM9SF2;TM9SF2;LINC00449	0.1458199	0.0004809	0.7355783
chr3	cg02167021	TSS200	HCLS1;HCLS1;HCLS1;HCLS1;HCLS1;HCLS1;HCLS1;HCLS1;HCLS1	0.2542263	0.0004813	0.7355783
chr16	cg23843797	TSS200	CCNF;CCNF	0.2708054	0.0004815	0.7355783

chr1	cg06216620	5'UTR;TSS200;5'UTR;5'UTR	STMN1;STMN1;STMN1;STMN1;STMN1;STMN1	0.1186456	0.0004827	0.7355783
chr17	cg12565681	5'UTR;5'UTR	RHBDF2	0.1457041	0.000483	0.7355783
chr10	cg08601192			-0.1213316	0.0004833	0.7355783
chr19	cg20556517	Body;Body		0.1305328	0.0004841	0.7355783
chr8	cg20387387		MAFA;MAFA;MAFA	0.171679	0.0004858	0.7355783
chr10	cg04474988			0.2576755	0.0004864	0.7355783
chr10	cg17064520	TSS1500;TSS1500;TSS1500	LCOR;LCOR;LCOR;LCOR	0.2203019	0.0004874	0.7355783
chr3	cg12988424	Body	RP11-204J18.3;RP11-204J18.3	0.2295297	0.0004901	0.7355783
chr3	cg00702638	1stExon;5'UTR;TSS200	KIF15;KIAA1143;KIAA1143;KIF15;KIF15;KIF15;KIF15	0.0901222	0.0004912	0.7355783
chr13	cg00722493	5'UTR;5'UTR;TSS1500;5'UTR;1stExon;1stExon;TSS200;1stExon	CUL4A;CUL4A;CUL4A;CUL4A;PCID2;PCID2;CUL4A;CUL4A;CUL4A;PCID2;PCID2;PCID2;PCID2;PCID2;PCID2;PCID2;PCID2;PCID2;PCID2;PCID2;PCID2;PCID2;PCID2;CUL4A;PCID2;PCID2;PCID2;PCID2;CUL4A	0.1340256	0.0004928	0.7355783
chr2	cg07250315	Body	IL1B;IL1B;IL1B	0.160115	0.0004934	0.7355783

chr5	cg01870619	Body	ZFR;ZFR	0.2076896	0.0004946	0.73 5578 3
chr2	cg11947548	Body	BBS5	0.2337307	0.0004946	0.73 5578 3
chr3	cg18016254	5'UTR;1stExon	PARP15;PARP15;PARP15;PARP15	0.2178356	0.0004957	0.73 5578 3
chr17	cg15920124	TSS200	GAST	- 0.2236555	0.0004981	0.73 5578 3
chr2	cg13437337	Body		0.213929	0.0005006	0.73 5578 3
chr8	cg04046119	TSS200;Body;Bod y	OXR1;OXR1	0.4283511	0.0005015	0.73 5578 3
chr5	cg17047981	TSS200	PCDHB12;PCDHB12;PCDHB12	0.1960502	0.0005024	0.73 5578 3
chr1	cg25359678	Body;1stExon;5'U TR	C1orf35;C1orf35;C1orf35;C1orf35;C1orf 35;C1orf35;C1orf35;C1orf35;C1orf35;C 1orf35;C1orf35;C1orf35	0.105408	0.0005026	0.73 5578 3
chr8	cg08431549			0.1434944	0.0005029	0.735 5783
chr1	cg13375638	5'UTR;5'UTR;Bod y;Body	UBE2J2;UBE2J2;UBE2J2;UBE2J2;UBE 2J2;UBE2J2;UBE2J2;UBE2J2;UBE2J2; UBE2J2	- 0.1402627	0.000503	0.73 5578 3
chr3	cg19188060	Body	PSMD2;PSMD2;PSMD2;PSMD2;PSMD 2;PSMD2;PSMD2;PSMD2;PSMD2;PS MD2;PSMD2	0.24634	0.0005032	0.73 5578 3

chr12	cg06584758	Body	RP11-13G14.4;RP11-13G14.4	0.201522	0.0005073	0.7355783
chr7	cg02562602	Body;Body;Body		-0.1793512	0.0005078	0.7355783
chr3	cg11081799	TSS1500;5'UTR;5'UTR;5'UTR;5'UTR	B3GALNT1;B3GALNT1;B3GALNT1;B3GALNT1;B3GALNT1;B3GALNT1;B3GALNT1;B3GALNT1;B3GALNT1;B3GALNT1;B3GALNT1;B3GALNT1;B3GALNT1;B3GALNT1;B3GALNT1;B3GALNT1;B3GALNT1;NMD3;NMD3	0.153036	0.0005084	0.7355783
chr17	cg14758012	Body		-0.1577265	0.0005086	0.7355783
chr1	cg16814416	Body	TCTEX1D4	-0.2282252	0.0005093	0.7355783
chr12	cg25329573	TSS200;Body;Body;Body;Body	SLC6A12;SLC6A12;SLC6A12;RP11-283I3.2;SLC6A12	1.1739658	0.0005108	0.7355783
chr22	cg02066590		GRAMD4;GRAMD4;GRAMD4	-0.1444147	0.0005149	0.7355783
chr1	cg14656093	Body;Body	RUNX3	-0.1600316	0.000515	0.7355783
chr6	cg03322161			0.3628706	0.0005183	0.7355783
chr16	cg06173319		PRDM7	0.4290532	0.0005191	0.7355783

chr21	cg04497512	1stExon	CLIC6	0.2049846	0.0005209	0.7355783
chr2	cg03147185	Body	NCAPH;NCAPH	-0.1042986	0.0005213	0.7355783
chr3	cg15895819	TSS200;TSS200	MFI2;MFI2;MFI2;MFI2	0.1146297	0.0005232	0.7355783
chr2	cg01909777			-0.1765525	0.000524	0.7355783
chr4	cg15540044	5'UTR;TSS200;1stExon;5'UTR;1stExon;1stExon;5'UTR	THAP6;RCHY1;RCHY1;RCHY1;THAP6;THAP6;THAP6;RCHY1;THAP6;RCHY1;RCHY1;THAP6;THAP6;RCHY1;RCHY1;RCHY1;RCHY1;THAP6;RCHY1;RCHY1;THAP6;RCHY1;RCHY1	0.1074146	0.0005248	0.7355783
chr1	cg10948827	3'UTR	EPHA8	-0.1392134	0.0005253	0.7355783
chr12	cg00655679		RP11-510I5.1	0.2051515	0.0005276	0.7355783
chr19	cg18054632	Body;Body;Body;Body;Body		-0.2420815	0.0005292	0.7355783
chr5	cg08328513	Body;TSS1500	DMGDH;BHMT2;BHMT2;BHMT2;DMGDH;DMGDH;DMGDH;DMGDH	0.3871117	0.0005306	0.7355783
chr15	cg06230236	TSS1500;TSS1500;TSS1500;TSS1500;TSS1500;TSS1500	TLE3;TLE3;TLE3;TLE3;TLE3;TLE3;TLE3;TLE3;TLE3;TLE3;TLE3;TLE3	0.1684428	0.0005307	0.7355783

chr19	cg22068659	Body		-0.2281966	0.0005319	0.7355783
chr4	cg25364273		RP11-440L14.3	-0.13708	0.0005331	0.7355783
chr5	cg16375290			0.2316214	0.0005333	0.7355783
chr19	cg24298440	Body;Body;Body;Body		0.1795924	0.0005342	0.7355783
chr20	cg23410129	5'UTR;5'UTR	SRC;SRC;SRC;SRC;SRC;SRC;SRC	-0.1140755	0.0005353	0.7355783
chr12	cg02339937		RP11-756H6.1;R3HDM2;R3HDM2	0.1692112	0.0005354	0.7355783
chr17	cg06906268	1stExon;5'UTR;1stExon;5'UTR	SEZ6;SEZ6;SEZ6;SEZ6;SEZ6;SEZ6;SEZ6	0.1926263	0.0005367	0.7355783
chr17	cg18962055	Body	C1QBP;C1QBP;C1QBP;C1QBP	0.1041237	0.0005374	0.7355783
chr16	cg07497645	Body	NAGPA;NAGPA;NAGPA;NAGPA;NAGPA;NAGPA;NAGPA;NAGPA;NAGPA;NAGPA;NAGPA	0.1289778	0.0005383	0.7355783
chr16	cg16730559	Body;Body	NPRL3;NPRL3;NPRL3;NPRL3;NPRL3;NPRL3;NPRL3	0.2278404	0.0005389	0.7355783
chr10	cg26892368	TSS200;TSS200	ALDH18A1;ALDH18A1	0.4132837	0.0005403	0.7355783
chr3	cg19143760	Body	GYG1;GYG1;GYG1	0.1207203	0.0005429	0.7355783

chr9	cg16132443	5'UTR;1stExon	NANS;NANS;NANS	0.1964349	0.0005438	0.7355783
chr14	cg08182021	Body	C14orf43;RP5-1021I20.1;RP5-1021I20.1	-0.3633188	0.0005444	0.7355783
chr5	cg16182972			0.1833097	0.0005471	0.7355783
chr6	cg10729419	Body	TRIM27	-0.1806346	0.0005472	0.7355783
chr2	cg10953983	1stExon;TSS200;5'UTR;5'UTR;1stExon	PHOSPHO2;C2orf77;PHOSPHO2;PHOSPHO2;PHOSPHO2;PHOSPHO2;KLHL23;KLHL23;C2orf77;PHOSPHO2;PHOSPHO2;PHOSPHO2;KLHL23;KLHL23	0.1424777	0.000549	0.7355783
chr1	cg04887124		RSG1;C1orf134	-0.1144305	0.0005499	0.7355783
chr11	cg02163039	TSS1500;TSS1500	PHF21A;PHF21A;PHF21A;PHF21A;PHF21A;PHF21A;PHF21A	0.1884447	0.00055	0.7355783
chr22	cg10188823	5'UTR;1stExon	NEFH;NEFH;NEFH	0.2653372	0.0005503	0.7355783
chr8	cg15937832	5'UTR	PNMA2;PNMA2;PNMA2;PNMA2;PNMA2;PNMA2;PNMA2;PNMA2;PNMA2	-0.2034956	0.0005506	0.7355783
chr7	cg14504536			-0.153731	0.0005531	0.7355783
chr7	cg08815583	Body	HEATR2	-0.1764663	0.0005544	0.7355783

chr1	cg21285056	TSS200	PLCH2;PLCH2;PLCH2	-0.1640661	0.0005556	0.7355783
chr9	cg14788802	TSS1500;TSS1500;TSS200	PNPLA7;PNPLA7;MRPL41;MRPL41	0.2059384	0.0005581	0.7355783
chr7	cg04194173	Body;Body;Body	C7orf50	-0.1506619	0.0005584	0.7355783
chr19	cg00487727	5'UTR	NFKBID	0.1131121	0.0005639	0.7355783
chr22	cg23015157	5'UTR;1stExon;5'UTR	FAM118A;FAM118A;FAM118A;FAM118A;FAM118A;FAM118A;FAM118A;FAM118A	0.1096999	0.0005652	0.7355783
chr5	cg16310668	Body	CTC-564N23.3;CTC-564N23.3;CTC-564N23.3;CTC-564N23.3;CTC-564N23.3;CTC-564N23.3;CTC-564N23.3;CTC-564N23.3;CTC-564N23.3;CTC-564N23.3;CTC-564N23.3;CTC-564N23.3	0.1796721	0.0005653	0.7355783
chr3	cg04841337			-0.2004378	0.0005664	0.7355783
chr19	cg01743192	Body		-0.1347995	0.0005673	0.7355783
chr19	cg25745472	TSS200	KPTN;KPTN	0.1198826	0.0005678	0.7355783
chr19	cg22152279	Body;Body	TCF3	-0.21624	0.0005686	0.7355783

chr19	cg03950599			0.8268899	0.0005709	0.7355783
chr13	cg11707796			-0.1766142	0.000574	0.7355783
chr6	cg04612667			0.3895008	0.0005747	0.7355783
chr15	cg22263031		RP11-143C19.1;RP11-143C19.1	-0.1651753	0.0005754	0.7355783
chr14	cg10943842	3'UTR	ANKRD9;ANKRD9	-0.2208908	0.0005762	0.7355783
chr7	cg22176042	Body	NUPL2;NUPL2;NUPL2;NUPL2	0.1457044	0.0005792	0.7355783
chr12	cg11358257	5'UTR	RASAL1;RASAL1;RASAL1;RASAL1;RASAL1;RASAL1;RASAL1;RASAL1;RASAL1;RASAL1;RASAL1	0.1610256	0.0005793	0.7355783
chr1	cg24378253	3'UTR	C1orf159;C1orf159;C1orf159;C1orf159;C1orf159;C1orf159	-0.1621157	0.0005794	0.7355783
chr9	cg13639672	1stExon;1stExon;5'UTR;5'UTR	PHPT1;PHPT1;PHPT1;PHPT1;PHPT1;PHPT1;PHPT1;PHPT1;PHPT1;PHPT1	0.1114256	0.0005797	0.7355783
chr6	cg20438766	TSS1500;TSS1500;TSS1500	CASP8AP2;CASP8AP2;CASP8AP2;CASP8AP2;CASP8AP2	0.2148335	0.0005797	0.7355783
chr11	cg06698742	5'UTR	TMEM126A;TMEM126A;TMEM126A;TMEM126A;TMEM126A	0.136761	0.0005811	0.7355783
chr11	cg23963595	5'UTR;Body;5'UTR;1stExon;1stExon;5'UTR;1stExon	MTMR2;MTMR2;MTMR2;MTMR2;MTMR2;MTMR2;MTMR2;MTMR2;MTMR2;MTMR2;MTMR2;MTMR2;MTMR2;MTMR2;MTMR2;MTMR2	0.1206444	0.0005814	0.7355783

chr10	cg07523098	TSS1500	ARHGAP12;ARHGAP12;ARHGAP12	0.198541	0.0005824	0.7355783
chr15	cg01021488	Body	IGF1R;IGF1R;IGF1R;IGF1R	0.2847054	0.000586	0.7355783
chr3	cg17913386	1stExon	COL7A1;COL7A1	0.3145674	0.0005879	0.7355783
chr7	cg22598458	Body;Body	ZNF92	-0.1180922	0.0005897	0.7355783
chr11	cg02769172	TSS1500;TSS1500	SUV420H1;SUV420H1;SUV420H1;SUV420H1;SUV420H1;SUV420H1;SUV420H1;SUV420H1;SUV420H1;SUV420H1;SUV420H1;SUV420H1;SUV420H1;SUV420H1	0.1861834	0.0005899	0.7355783
chr20	cg21633795	TSS200	PFDN4;PFDN4;PFDN4;PFDN4;PFDN4	0.1287281	0.0005923	0.7355783
chr12	cg15733245			-0.2238525	0.0005931	0.7355783
chr15	cg07501391	TSS200;TSS200;TSS200;TSS200	NUSAP1;OIP5;NUSAP1;NUSAP1;NUSAP1;NUSAP1;OIP5;NUSAP1;NUSAP1;NUSAP1;NUSAP1;NUSAP1;NUSAP1	0.1217071	0.0005936	0.7355783
chr20	cg22232492			0.1186182	0.0005968	0.7355783
chr15	cg08502856	TSS200;TSS1500;TSS1500	PDE8A;PDE8A;PDE8A;PDE8A	0.1537838	0.0005972	0.7355783
chr2	cg19571304	Body	SNRNP200;SNRNP200;SNRNP200	-0.1843771	0.0005979	0.7355783

chr4	cg23069057		LINC00504;LINC00504;LINC00504	0.188643	0.0005995	0.735 5783
chr7	cg05602310		PMS2CL	0.2068046	0.0006003	0.735 5783
chr22	cg04748357	TSS1500;TSS200; TSS1500	LMF2;NCAPH2;NCAPH2;NCAPH2;NC APH2;NCAPH2;NCAPH2;NCAPH2;NC APH2;NCAPH2;NCAPH2;NCAPH2;L MF2;LMF2;LMF2;LMF2;LMF2;LMF2	0.2342796	0.0006004	0.73 5578 3
chr3	ch.3.243862 0R	Body	ITGB5;ITGB5	0.5470469	0.0006004	0.73 5578 3
chr11	cg24932449	Body;Body;Body		-0.220145	0.0006014	0.73 5578 3
chr10	cg12093371			0.1480018	0.0006019	0.735 5783
chr17	cg01749555	TSS200	TBCD;TBCD;TBCD;TBCD	0.1623392	0.0006023	0.73 5578 3
chr19	cg27052113	Body	SLC8A2	0.368808	0.0006043	0.73 5578 3
chr19	cg19429286			0.1032335	0.0006045	0.735 5783
chr5	cg23053741	1stExon;5'UTR	TNPO1;TNPO1;TNPO1;TNPO1;CTD-2631K10.1;TNPO1;TNPO1;TNPO1;TN PO1;TNPO1	0.1604656	0.0006047	0.73 5578 3
chr11	cg12004045	TSS1500;Body;Bo dy;Body;Body;Bo dy	NAV2-AS2	0.1865527	0.0006057	0.73 5578 3
chr1	cg15084499	TSS200;TSS200	CDC42BPA;CDC42BPA;CDC42BPA;C DC42BPA;CDC42BPA;CDC42BPA	0.2515524	0.0006072	0.73 5578 3

chr19	cg03247892	Body;Body;Body	ICAM4	0.249111	0.0006082	0.735578 3
chr11	cg09666841	Body;TSS1500;TSS1500;TSS1500;TSS1500	ARFIP2;FXC1;ARFIP2;ARFIP2;ARFIP2;ARFIP2;ARFIP2;ARFIP2;ARFIP2	-0.2264314	0.0006093	0.735578 3
chr18	cg08914190	Body;Body;Body;Body;Body;Body;Body;Body;Body;Body		-0.2301378	0.00061	0.735578 3
chr2	cg11472573		U6	0.1967223	0.0006115	0.735 5783
chr9	cg08754068	3'UTR;Body;Body;Body;Body;Body;Body	ST6GALNAC6;ST6GALNAC6;RP11-203J24.9;ST6GALNAC6;RP11-203J24.9	-0.2032373	0.000612	0.735578 3
chr14	cg13158272		C14orf43;RP5-1021I20.1;RP5-1021I20.1	-0.3635986	0.0006122	0.735 5783
chr17	cg16560774	TSS200	RP11-1055B8.6;BAHCC1;RP11-1055B8.6	0.1593125	0.0006128	0.735578 3
chr2	cg17986036	TSS200;Body;Body;Body	ACTR3;ACTR3;ACTR3	0.098542	0.0006132	0.735578 3
chr10	cg01320200		RP11-525A16.1	0.1821972	0.0006138	0.735 5783
chr1	cg10210571	TSS200;TSS200;TSS200;TSS200;TSS200;TSS200;TSS200;TSS200	TSNAX;RP11-295G20.2;TSNAX;RP11-295G20.2;RP11-295G20.2;TSNAX;TSNAX;RP11-295G20.2	0.2648443	0.0006149	0.735578 3
chr22	cg00108569		ADORA2A;ADORA2A;ADORA2A;ADORA2A;ADORA2A;ADORA2A;ADORA2A;KB-1896H10.1	0.1080054	0.0006151	0.735 5783

chr1	cg05021743	Body;TSS200		0.1687939	0.0006154	0.73 5578 3
chr17	cg02241363	5'UTR;1stExon	NPTX1;NPTX1;NPTX1;NPTX1	0.2397451	0.0006159	0.73 5578 3
chr8	cg13580084		CTC-756D1.2	0.1451633	0.0006167	0.735 5783
chr17	cg12255698	TSS200	MIR132	0.2604653	0.0006175	0.73 5578 3
chr19	cg05886453	3'UTR	LRFN3	- 0.2164437	0.0006188	0.73 5578 3
chr2	cg17664192			- 0.1369432	0.0006192	0.735 5783
chr13	cg21294654	Body;Body	MCF2L	- 0.1322327	0.0006196	0.73 5578 3
chr1	cg21367040	1stExon;5'UTR	C1orf63;C1orf63;C1orf63;C1orf63;TMEM50A;TMEM50A;C1orf63;C1orf63;TMEM50A;C1orf63;TMEM50A;TMEM50A;TMEM50A;C1orf63;TMEM50A;TMEM50A;TMEM50A	0.1568692	0.00062	0.73 5578 3
chr9	cg24225917	TSS1500;TSS1500;TSS1500;TSS1500;Body	URM1;URM1;URM1	0.1700519	0.0006201	0.73 5578 3
chr1	cg06760305	3'UTR	OBSCN	0.1195899	0.0006206	0.73 5578 3
chr2	cg01118224	TSS200	ADAM17;ADAM17;ADAM17;ADAM17	0.1297787	0.0006207	0.73 5578 3

chr2	cg07800384	TSS1500	FAM150B;FAM150B;FAM150B;AC079779.4	0.1800459	0.0006214	0.735578 3
chr22	cg03373419	TSS200	ADM2;ADM2;ADM2	0.1180122	0.0006239	0.735578 3
chr12	cg06134974	TSS1500	CSRNP2;CSRNP2;CSRNP2;CSRNP2;CSRNP2;CSRNP2;CSRNP2	0.3038521	0.0006246	0.735578 3
chr5	cg16582978	1stExon;1stExon;5'UTR;5'UTR;5'UTR;1stExon	NPM1;NPM1;NPM1;NPM1;NPM1;NPM1;NPM1;NPM1;NPM1;NPM1;NPM1;NPM1;NPM1	0.1280222	0.0006268	0.735578 3
chr8	cg22107011			0.3425405	0.0006272	0.735 5783
chr15	cg01714418	TSS1500;TSS1500;TSS1500	EDC3;EDC3;EDC3;EDC3;EDC3;EDC3;EDC3;EDC3;EDC3;EDC3;EDC3;EDC3;EDC3;EDC3;EDC3	0.1748945	0.0006275	0.735578 3
chr19	cg22806837	5'UTR;1stExon	ZNF773;ZNF773;ZNF773	0.1338543	0.0006277	0.735578 3
chr9	cg03986101	TSS200	RALGPS1;RALGPS1;RALGPS1;RALGPS1;RALGPS1;RALGPS1;RALGPS1;RALGPS1	0.1662326	0.0006282	0.735578 3
chr10	cg02399189	Body	RASSF4;C10orf10;RASSF4;RASSF4;RASSF4	0.095313	0.0006283	0.735578 3
chr2	cg19130836	TSS200;TSS200	MRPL35;MRPL35	0.2870205	0.0006289	0.735578 3
chr1	cg10311240	TSS200	DDX59;DDX59;DDX59;DDX59;DDX59;RP11-92G12.3	0.1442368	0.0006289	0.735578 3

chr3	cg14716208	Body		0.2590162	0.0006294	0.73 5578 3
chr13	cg20524211	Body;1stExon;5'U TR;TSS1500;5'UT R	CARS2;ING1;ING1;ING1;ING1;CARS2 ;CARS2;CARS2;CARS2	0.1136726	0.0006322	0.73 5578 3
chr4	cg21042265	Body;Body		0.2596077	0.0006335	0.73 5578 3
chr13	cg02702515	Body;TSS1500;Bo dy;Body	UBAC2;UBAC2;UBAC2;UBAC2;MIR6 23	-0.140057	0.0006337	0.73 5578 3
chr1	cg04139409			0.2435327	0.0006339	0.735 5783
chr5	cg20924470	TSS200	FBXL21;FBXL21;FBXL21	0.2045682	0.0006339	0.73 5578 3
chr1	cg22796507	1stExon	FOXE3	0.182269	0.000634	0.73 5578 3
chr5	cg18804667	Body;TSS1500;Bo dy	PDE4D;PDE4D;PDE4D;PDE4D;PDE4D	0.2462242	0.0006347	0.73 5578 3
chr12	cg12790145	TSS200;Body;Bod y;Body;Body	SLC6A12;SLC6A12;SLC6A12;RP11-283I3.2;SLC6A12	1.1941794	0.0006368	0.73 5578 3
chr18	cg19057711	Body	FAM69C	- 0.1455966	0.0006371	0.73 5578 3
chr1	cg23847109	TSS200	COL9A2;COL9A2;COL9A2;COL9A2;C OL9A2;COL9A2;COL9A2;COL9A2	0.3251836	0.0006384	0.73 5578 3

chr1	cg20792826	Body;Body;Body	RP1-37C10.3;ATP13A2	-0.1154039	0.0006387	0.7355783
chr7	cg17170568	TSS200;1stExon;5'UTR	RNF32;RNF32;AC073871.2;RNF32;AC073871.2;RNF32;AC073871.2;C7orf13;RNF32;RNF32;RNF32;RNF32	0.13687	0.0006394	0.7355783
chr19	cg05253604	TSS1500	CHERP;CHERP;CHERP;CHERP;CALR3	0.1734221	0.0006395	0.7355783
chr8	cg08650125			0.2876397	0.00064	0.7355783
chr3	cg12324144	TSS1500	LRRC34;LRRC34;LRRC34;LRRC34;LRRC34;LRRC34;LRRC34	0.4131659	0.0006414	0.7355783
chr4	cg09861350	TSS1500;TSS1500;TSS1500;TSS1500;TSS1500;ExonBnd;Body	EPHA5;RP11-807H7.1;RP11-807H7.1;RP11-807H7.1	0.3340731	0.0006424	0.7355783
chr17	cg20289346		PLXDC1	0.1377907	0.0006429	0.7355783
chr3	cg26456937	TSS200;TSS200;5'UTR	BDH1;BDH1;BDH1;BDH1;BDH1;BDH1;BDH1;BDH1;BDH1	0.1384433	0.0006443	0.7355783
chr18	cg20743280	Body	ZNF521	0.1780259	0.0006449	0.7355783
chr19	cg04689371	1stExon;5'UTR;1stExon;5'UTR	U2AF2;U2AF2	0.1616623	0.0006449	0.7355783
chr2	cg11606796	TSS1500;5'UTR;5'UTR;TSS200;Body	BZW1;BZW1;BZW1;BZW1;BZW1;AC007163.6;BZW1;BZW1;BZW1;BZW1;AC007163.6	0.1348948	0.0006509	0.7355783

chr7	cg13760339	5'UTR;TSS200;Body;Body;Body	FAM133B;FAM133B;FAM133B;FAM133B;FAM133B	0.2687601	0.0006512	0.735578 3
chr10	cg00369151	Body	PIP4K2A	0.599461	0.0006515	0.735578 3
chr14	cg13198844	TSS200;TSS200;TSS200;TSS200;TSS200	DCAF4;DCAF4;DCAF4;DCAF4;DCAF4;DCAF4;DCAF4;DCAF4;DCAF4;DCAF4	0.1587226	0.0006516	0.735578 3
chr10	cg27412516	TSS1500;TSS1500	SAMD8;SAMD8;SAMD8;SAMD8	0.1636565	0.0006521	0.735578 3
chr1	cg14965611	Body;Body	SDF4;SDF4	-0.1206307	0.0006552	0.735578 3
chr5	cg26318502			-0.127478	0.000656	0.735 5783
chr3	cg06341749	TSS200	PCOLCE2;PCOLCE2;PCOLCE2;PCOLCE2;PCOLCE2;PCOLCE2	0.1197148	0.0006571	0.735578 3
chr15	cg20103352	Body;Body;Body;Body	TCF12;TCF12	0.1393688	0.0006574	0.735578 3
chr2	cg07834408	Body;Body	DGKD;DGKD;DGKD	-0.1494674	0.0006578	0.735578 3
chr5	cg23975425			-0.1036283	0.0006619	0.735 5783
chr1	cg11857025	TSS200	TMEM167B;TMEM167B;TMEM167B	0.2675702	0.0006645	0.735578 3

chr16	cg05973832	5'UTR;5'UTR;1stExon;1stExon;5'UTR	HMOX2;HMOX2;NMRAL1;NMRAL1;HMOX2;HMOX2;HMOX2;HMOX2;HMOX2;HMOX2;NMRAL1;HMOX2;HMOX2;HMOX2;HMOX2;HMOX2;HMOX2;HMOX2;HMOX2;HMOX2	0.1023119	0.0006654	0.7355783
chr9	cg12703418	TSS200	COL27A1;COL27A1	0.1288958	0.0006671	0.7355783
chr3	cg07608658	TSS1500	TTLL3;TTLL3;TTLL3;TTLL3;TTLL3;TTLL3;TTLL3;TTLL3;TTLL3;TTLL3;TTLL3;TTLL3;TTLL3;TTLL3;TTLL3;TTLL3;ARPC4	0.1770462	0.0006672	0.7355783
chr3	cg20839041	Body	TERC	0.1383611	0.0006674	0.7355783
chr8	cg00687766	Body;5'UTR;TSS1500;Body;TSS1500;Body;TSS1500;TSS1500;1stExon	TM2D2;TM2D2;TM2D2;TM2D2;TM2D2;TM2D2;ADAM9;ADAM9;ADAM9;ADAM9;TM2D2;TM2D2;ADAM9;ADAM9	0.2240007	0.000669	0.7355783
chr6	cg10894807	Body;Body		-0.2178259	0.0006702	0.7355783
chr6	cg18704865	Body;Body;Body;Body	DAXX;DAXX;DAXX;DAXX	-0.1849093	0.0006704	0.7355783
chr14	cg05481811	TSS200;TSS200;TSS200;TSS200	NPAS3;NPAS3;NPAS3;NPAS3	0.1636477	0.0006704	0.7355783
chr2	cg19763809	TSS200	VWA3B;VWA3B;VWA3B;VWA3B;VWA3B;VWA3B;VWA3B	0.2265898	0.0006709	0.7355783

chr9	cg05928239	TSS1500	FOXE1	0.1548533	0.0006717	0.73 5578 3
chr12	cg18262805	TSS1500;TSS1500 ;TSS200	CCT2;CCT2;CCT2;CCT2;CCT2;CCT2; CCT2	0.1293573	0.000672	0.73 5578 3
chr4	cg21463776	Body		0.1726078	0.0006726	0.73 5578 3
chr1	cg12796440	1stExon	YOD1;PFKFB2	0.3044414	0.000674	0.73 5578 3
chr14	cg12892776		RP11-543C4.1	- 0.1579958	0.0006758	0.735 5783
chr10	cg23378937	Body;Body	PAPSS2;PAPSS2;PAPSS2;RP11- 57C13.3	0.121796	0.0006763	0.73 5578 3
chr5	cg18617005	Body;Body;Body; Body;Body;Body; Body;TSS1500;Bo dy;Body;Body;Bo dy;Body;TSS1500; Body;Body	PCDHGB6	0.1425776	0.0006775	0.73 5578 3
chr1	cg13048512	5'UTR	SLC6A17;SLC6A17	0.1233331	0.0006787	0.73 5578 3
chr6	cg06993041	TSS200	TUBB;TUBB;TUBB;TUBB;TUBB	0.1129519	0.0006795	0.73 5578 3
chr19	cg04306971	TSS200	EID2;EID2	0.1097408	0.0006796	0.73 5578 3

chr15	cg07729059			0.316163	0.0006804	0.7355783
chr6	cg00617180			0.1512897	0.0006809	0.7355783
chr1	cg24690722	5'UTR	POGK;POGK;POGK;POGK	0.1319611	0.0006835	0.7355783
chr13	cg12322445	TSS1500;TSS200;TSS200;TSS200;Body	LMO7;RP11-173B14.5;RP11-29G8.3;LMO7	0.3543237	0.0006836	0.7355783
chr14	cg16561543	TSS1500;Body;TSS200	BTBD6;BRF1;BRF1;BTBD6;BRF1;BRF1;BTBD6;BTBD6;BTBD6;BTBD6;BRF1;BRF1;BRF1;BRF1;BRF1	0.1989493	0.0006852	0.7355783
chr2	cg01099876	TSS200;TSS200;TSS200	SEPT10;SEPT10;SEPT10;SEPT10;SEPT10;SEPT10;SEPT10;SOWAHC;SEPT10;SEPT10;SEPT10;SEPT10	0.1381145	0.0006859	0.7355783
chr3	cg08538682	1stExon;5'UTR;Body	LXN;LXN;GFM1	0.1769376	0.0006868	0.7355783
chr5	cg10600010	TSS200	CLK4;CLK4;CLK4;CLK4;CLK4;CLK4;CLK4;CLK4;CLK4;CLK4;CLK4	0.1733424	0.0006885	0.7355783
chr4	cg11265221	Body	CEP44	-0.7352281	0.0006885	0.7355783
chr12	cg21669272	TSS200;TSS200	ASIC1;ASIC1;ASIC1;ASIC1	0.1221618	0.00069	0.7355783
chr1	cg00778544	TSS200	SNX27;SNX27;RP11-404E16.1;RP11-404E16.1;SNX27;SNX27	0.1383025	0.0006923	0.7355783

chr1	cg26015888	Body;Body	RP1-224A6.3;LINC00339;LINC00339;LINC00339;LINC00339;LINC00339;LINC00339	0.187472	0.0006931	0.735578 3
chr12	cg06867016	TSS200;Body;Body		-0.1284992	0.0006932	0.735578 3
chr1	cg18528640	TSS200	MSTO1;MSTO1;MSTO1;RP11-29H23.4;MSTO1;MSTO1;MSTO1;MSTO1;MSTO1;MSTO1;MSTO1;MSTO1;MSTO1;MSTO1;MSTO1;MSTO1	0.1521114	0.0006933	0.735578 3
chr19	cg24098404	TSS200	LSM4	0.1342827	0.0006955	0.735578 3
chr6	cg26958615			0.2699163	0.0006963	0.735 5783
chr8	cg04803226	TSS1500;TSS1500;TSS1500	NKX3-1;NKX3-1	-0.2382859	0.0006998	0.735578 3
chr11	cg09222269			-0.269649	0.0007009	0.735 5783
chr6	cg11601400	5'UTR	RN7SK;GSTA4;GSTA4;GSTA4;GSTA4;GSTA4;GSTA4	0.2440932	0.0007034	0.735578 3
chr20	cg14773603		RP1-148H17.1	-0.1485877	0.0007044	0.735 5783
chr22	cg06943853	Body		-0.2026944	0.0007093	0.735578 3
chr20	cg23459915	TSS1500;TSS1500;TSS1500;TSS200;TSS200;TSS200	TGIF2;RP5-977B1.10;TGIF2;TGIF2;RP5-977B1.7;RP5-977B1.7;TGIF2;RP5-977B1.7;RP5-977B1.7;RP5-977B1.11	0.1028859	0.0007095	0.735578 3

chr19	cg26532042	TSS200	FTL;FTL	0.2514936	0.0007122	0.735578 3
chr5	cg03559817	1stExon;5'UTR;5'UTR;5'UTR;1stExon;1stExon;Body	SMAD5-AS1;SMAD5;SMAD5;SMAD5;SMAD5;SMAD5;SMAD5;SMAD5;SMAD5;SMAD5	0.2557623	0.0007125	0.735578 3
chr4	cg06083330	Body		0.2846936	0.0007127	0.735578 3
chr7	cg09296001	Body	LRRC4;LRRC4;LRRC4;SND1;SND1	0.1583981	0.0007131	0.735578 3
chr19	cg20917477	Body;5'UTR;Body;Body	GDF1	-0.1626129	0.0007131	0.735578 3
chr9	cg13442960	5'UTR;5'UTR;TSS200	ELAVL2;ELAVL2;ELAVL2;ELAVL2;ELAVL2	0.2083724	0.0007132	0.735578 3
chr7	cg27452451	TSS200;TSS200;TSS200	CCT6A;PSPH;CCT6A;PSPH;PSPH;CCT6A;CCT6A;CCT6A;PSPH;PSPH;PSPH	0.1211254	0.0007134	0.735578 3
chr9	cg12564647	TSS1500;Body;Body;Body	MAN1B1;MAN1B1;MAN1B1;MAN1B1	-0.1252203	0.0007146	0.735578 3
chr20	cg08820386			-0.19123	0.0007151	0.735 5783
chr1	cg13864586	Body	RNF220;RNF220;RNF220;RNF220;RNF220;RNF220;RNF220;RNF220	0.1893965	0.0007153	0.735578 3
chr3	cg22824767	1stExon	GRK7	-0.1477594	0.0007177	0.735578 3

chr19	cg09231514	5'UTR;5'UTR;5'UTR;5'UTR;TSS1500;5'UTR;5'UTR	HSD11B1L;HSD11B1L;HSD11B1L;C19orf70;HSD11B1L;HSD11B1L	0.2577117	0.0007179	0.735578 3
chr8	cg22083639	TSS1500	RP11-159H10.3;RP11-159H10.3;SLCO5A1;AC079089.1;SLCO5A1;SLCO5A1;SLCO5A1;SLCO5A1;RP11-159H10.3	0.2038	0.0007193	0.735578 3
chr17	cg25364898			0.191164	0.0007193	0.735 5783
chr17	cg04369100	Body		-0.1553778	0.0007258	0.735578 3
chr17	cg01730976	Body	FN3KRP;FN3KRP;FN3KRP	-0.1159745	0.0007261	0.735578 3
chr17	cg01832036	Body;5'UTR;1stExon	DLX4;DLX4;DLX4;DLX4;DLX4	0.140501	0.0007266	0.735578 3
chr20	cg07547549	Body;Body		0.1420193	0.000727	0.735578 3
chr1	cg05009066	TSS200;TSS200;TSS200;TSS200;TSS200	ABL2;ABL2;ABL2	0.1426707	0.0007273	0.735578 3
chr8	cg10219742			0.1284425	0.0007274	0.735 5783
chr9	cg21161931			-0.1132421	0.0007319	0.735 5783
chr18	cg26938597			0.2015458	0.0007323	0.735 5783
chr8	cg13580096	1stExon;5'UTR	ARMC1;ARMC1;ARMC1;ARMC1;ARMC1;ARMC1;ARMC1;ARMC1;ARMC1;ARMC1;ARMC1	0.1585858	0.0007329	0.735578 3

chr14	cg16522045		C14orf37	0.4113364	0.0007346	0.7355783
chr15	cg15488329	Body;Body	SEMA4B;SEMA4B;SEMA4B;SEMA4B;SEMA4B	-0.4673532	0.0007347	0.7355783
chr17	cg19104443	3'UTR	TMEM105	-0.2627436	0.0007355	0.7355783
chr16	cg16211462	Body	HN1L;HN1L;HN1L;HN1L;LA16c-431H6.6	-0.1268517	0.0007383	0.7355783
chr3	cg27580173	Body;5'UTR;5'UTR;Body	RP11-475O23.2;FAM107A;FAM107A;FAM107A	-0.141355	0.0007383	0.7355783
chr7	cg03009463	TSS200	SNX13;SNX13;SNX13;SNX13;SNX13;SNX13;SNX13;SNX13;SNX13	0.1197477	0.0007386	0.7355783
chr4	cg24862189	Body	GAK;GAK	-0.1475141	0.0007394	0.7355783
chr20	cg25803642	TSS1500;TSS1500;TSS1500	DNMT3B;DNMT3B;DNMT3B;DNMT3B;DNMT3B	0.1287128	0.0007398	0.7355783
chr2	cg13217260	TSS200	HOXD10;HOXD10;HOXD10;HOXD10	-0.3220267	0.0007401	0.7355783
chr11	cg13249008		DOC2GP	0.2137142	0.0007414	0.7355783
chr5	cg14376424	1stExon	FAM71B;ITK	-0.1465651	0.0007435	0.7355783

chr9	cg23322868	TSS1500;TSS1500;TSS1500;TSS1500;TSS1500;TSS1500;TSS1500;TSS1500;TSS1500;TSS1500;TSS1500;TSS1500;TSS1500;Body	CDKN2A;CDKN2B-AS1;MTAP;CDKN2A;CDKN2A	0.1451185	0.0007442	0.735578 3
chr5	cg14475001	TSS200;TSS200;TSS200	PAPD4;PAPD4;PAPD4;PAPD4;PAPD4;PAPD4;PAPD4;PAPD4;PAPD4;PAPD4	0.1340859	0.0007452	0.735578 3
chr5	cg19780254	1stExon;1stExon;1stExon;5'UTR;5'UTR;5'UTR;Body;5'UTR;5'UTR	AC034228.2;ACSL6;ACSL6;ACSL6;ACSL6;ACSL6;ACSL6;ACSL6;ACSL6;ACSL6;ACSL6	0.2793299	0.0007467	0.735578 3
chr13	cg10285162	Body;Body;Body;Body	LINC00422	-0.1480099	0.0007478	0.735578 3
chr7	cg06596689	TSS1500;Body	RP4-659J6.2	0.1399385	0.0007495	0.735578 3
chr5	cg09820378	Body;Body;Body;TSS200;Body;TSS200;Body;Body;TSS200	PCDHA6;PCDHA6	0.2081846	0.0007505	0.735578 3
chr19	cg02580948			-0.4055858	0.0007513	0.735 5783
chr6	cg11498188	TSS1500	HECA	0.1695016	0.000752	0.735578 3
chr5	cg11745506	1stExon;TSS1500;5'UTR	CCDC112;CCDC112;CCDC112;CCDC112;CCDC112;CCDC112;CCDC112	0.1072817	0.0007525	0.735578 3

chr21	cg07872789	TSS200	MORC3;CHAF1B;CHAF1B;MORC3;MORC3	0.4342408	0.0007566	0.7355783
chr17	cg01681009	1stExon;TSS200	CCT6B;CCT6B;CCT6B;ZNF830	0.1128722	0.0007593	0.7355783
chr11	cg24462596	1stExon;5'UTR;TSS200	CWC15;CWC15;CWC15;CWC15;CWC15;KDM4D;KDM4D;KDM4D	0.2309278	0.0007612	0.7355783
chr11	cg17037149			-0.1739904	0.0007618	0.7355783
chr12	cg20402783	Body	HOXC10;HOXC10;HOXC10;HOXC5	0.399011	0.0007622	0.7355783
chr11	cg25327839	5'UTR;1stExon	FAM168A;FAM168A;FAM168A;FAM168A;FAM168A;FAM168A	0.2636431	0.0007631	0.7355783
chr17	cg10421979	TSS200;Body	ACSF2;CHAD;CHAD;ACSF2;ACSF2;ACSF2;ACSF2;ACSF2;CHAD;ACSF2;ACSF2;ACSF2;ACSF2	0.7681467	0.0007647	0.7355783
chr5	cg03295517		CTC-229L21.1	-0.4525891	0.000765	0.7355783
chr3	cg14049380	TSS200	NEK4;NEK4;NEK4;NEK4	0.1276226	0.0007656	0.7355783
chr1	cg16242653		PROX1-AS1	-0.375318	0.0007691	0.7355783
chr17	cg14572758	1stExon;5'UTR	LASP1;LASP1;LASP1;LASP1;LASP1;LASP1	0.0998652	0.0007692	0.7355783
chr22	cg18338288	Body		-0.2892099	0.0007694	0.7355783

chr5	cg10399496	Body;Body;Body	RP11-823P9.1;RP11-823P9.1;RP11-823P9.1;RP11-823P9.1;RP11-823P9.1	0.16195	0.0007709	0.7355783
chr11	cg03103218	Body	MACROD1;MACROD1;MACROD1;RP11-21A7A.2;RP11-21A7A.2	0.2018591	0.000772	0.7355783
chr8	cg26702929	1stExon;5'UTR;1stExon;5'UTR	HR;HR;HR;HR;HR;HR;HR	0.2486795	0.0007742	0.7355783
chr19	cg22336774	Body;Body;Body;1stExon;Body	PNPLA6	0.1026635	0.0007746	0.7355783
chr10	cg27358811	TSS1500	TM9SF3;TM9SF3;TM9SF3	0.1703979	0.0007753	0.7355783
chr13	cg06705004	Body	GJB2	-0.1480282	0.000777	0.7355783
chr5	cg08647699	1stExon;5'UTR	CDKN2AIPNL;CDKN2AIPNL	0.0931526	0.0007776	0.7355783
chr11	cg12958961			0.4330165	0.0007777	0.7355783
chr12	cg14518868	5'UTR;5'UTR	FRS2;FRS2;FRS2;FRS2;FRS2;FRS2;FRS2;FRS2;FRS2	0.158816	0.0007793	0.7355783
chr16	cg22025478	TSS200	RP11-482M8.1;ZCCHC14;RP11-482M8.1;ZCCHC14;ZCCHC14	0.3765865	0.0007797	0.7355783
chr11	cg18908524	5'UTR;1stExon	RP11-783K16.14;VEGFB;VEGFB;RP11-783K16.14;VEGFB;VEGFB	0.1207063	0.0007815	0.7355783

chr12	cg10583414	Body;Body	HDAC7;HDAC7;HDAC7;HDAC7;HDAC7;HDAC7;HDAC7;HDAC7	0.1468655	0.0007827	0.7355783
chr20	cg17027046	TSS200;TSS200	ITPA;ITPA;ITPA;ITPA;ITPA;ITPA;ITPA;ITPA	0.1526659	0.0007828	0.7355783
chr7	cg16362592	Body;Body	TBX20;TBX20	0.2272645	0.0007832	0.7355783
chr6	cg12033528			0.154075	0.0007834	0.7355783
chr4	cg08209777	Body;Body	CTBP1;CTBP1;CTBP1;CTBP1;CTBP1	-0.1353055	0.0007836	0.7355783
chr1	cg06408034	1stExon	ZSWIM5;ZSWIM5	0.212111	0.0007843	0.7355783
chr10	cg05381746	TSS1500	MKX;MKX;MKX;RP11-360I20.2	0.1896385	0.0007847	0.7355783
chr7	cg11464238	TSS200;TSS200	MKRN1;MKRN1;MKRN1;MKRN1;MKRN1;MKRN1;MKRN1;MKRN1;MKRN1;MKRN1;MKRN1;MKRN1;MKRN1;MKRN1	0.1378241	0.0007854	0.7355783
chr17	cg02289168	Body	FASN	-0.1632023	0.0007856	0.7355783
chr2	cg18222526	TSS200	PAPOLG;PAPOLG;PAPOLG;PAPOLG	0.1123337	0.000786	0.7355783
chr16	cg07341290			-0.2940496	0.0007866	0.7355783

chr19	cg00457361	5'UTR	FBXO46	-0.1214652	0.0007877	0.7355783
chr16	cg00843236	TSS200	GSG1L;GSG1L;GSG1L	0.1235423	0.0007882	0.7355783
chr22	cg04242821	Body;Body;Body	SMTN;SMTN;SMTN;MTMR3	-0.1439634	0.0007885	0.7355783
chr7	cg07617636	Body	PRKAG2	-0.1535101	0.0007888	0.7355783
chr1	cg22736787	TSS200	PTGFRN;PTGFRN	0.1494994	0.0007897	0.7355783
chr6	cg00014327	TSS1500;TSS1500;TSS200;TSS200;TSS200;TSS200	RAB23;RAB23;RAB23	0.1373366	0.0007905	0.7355783
chr1	cg17138539	Body;3'UTR	MED8;MED8;RP1-92O14.6	-0.1707854	0.000791	0.7355783
chr5	cg11979452	5'UTR;5'UTR	SKP1;SKP1;SKP1;SKP1;SKP1;SKP1;SKP1;SKP1;SKP1	0.1791645	0.0007912	0.7355783
chr19	cg10775492	Body		-0.1253951	0.0007916	0.7355783
chr22	cg26040261	1stExon;5'UTR	PMM1;PMM1;PMM1;PMM1;PMM1;PMM1;PMM1;PMM1;PMM1;PMM1;PMM1;CSDC2;PMM1;PMM1	0.1232782	0.000792	0.7355783
chr1	cg16231240	TSS200;TSS200;TSS200	RBM34;RBM34;RBM34;RBM34;RBM34;RBM34;RBM34;RBM34;RBM34;RBM34;RBM34;RBM34	0.109321	0.0007923	0.7355783

chr8	cg00189906	TSS1500	FAM91A1;FAM91A1;FAM91A1;FAM91A1	0.1612026	0.000793	0.735578 3
chr12	cg15946163			-0.195407	0.0007939	0.735 5783
chr11	cg13559712	TSS200;Body;Body;Body	MIR1343	-0.146947	0.0007958	0.735578 3
chr19	cg17859835	TSS200	ZNF615;ZNF615	0.2854591	0.000796	0.735578 3
chr5	cg00529697	5'UTR;1stExon	POC5;POC5;POC5;POC5;POC5;POC5;POC5;POC5;POC5;POC5;POC5;POC5;POC5;POC5;POC5;POC5;POC5;POC5;POC5;POC5	0.3504771	0.0007967	0.735578 3
chr19	cg15745619	TSS200;TSS200;TSS200;TSS200;TSS200;TSS200;TSS200	ZNF419;ZNF419;ZNF419;ZNF419;ZNF419;ZNF419;ZNF419;ZNF419;ZNF419;ZNF419;ZNF419	0.2657538	0.0007971	0.735578 3
chr7	cg11835459			-0.2527108	0.0007977	0.735 5783
chr19	cg10465626	TSS200;TSS200	PPP5C;PPP5C;PPP5C;PPP5C	0.1370417	0.000798	0.735578 3
chr21	cg02074274	3'UTR	PDXK;PDXK	0.174281	0.0007983	0.735578 3
chr1	cg20685674	TSS1500;TSS1500;TSS1500;TSS200	STMN1;STMN1;STMN1;STMN1	0.1753957	0.0007989	0.735578 3
chr13	cg25319309	TSS1500;TSS1500;Body	DOCK9;DOCK9;DOCK9;RP11-122A8.4	0.1504233	0.0007994	0.735578 3

chr13	cg24842528		DLEU1;DLEU1	0.175902	0.0008012	0.7355783
chr11	cg08198542	Body	TEAD1	-0.2029775	0.0008012	0.7355783
chr2	cg05156062	TSS200;TSS200;TSS200;TSS200	AC016725.4;CCNT2;CCNT2;CCNT2;CCNT2;CCNT2;AC016725.4;AC016725.4;AC016725.4;CCNT2;CCNT2;CCNT2	0.1881802	0.0008025	0.7355783
chr2	cg12641736			-0.1463785	0.0008046	0.7355783
chr9	cg11255687	Body		0.1730808	0.0008049	0.7355783
chr19	cg14951193	TSS200	PLIN5	0.1983532	0.0008057	0.7355783
chr19	cg02365577	5'UTR	SIPA1L3;SIPA1L3	-0.1162796	0.0008074	0.7355783
chr6	cg10308833			0.290241	0.0008094	0.7355783
chr2	cg06362432			-0.1456911	0.0008102	0.7355783
chr19	cg23467944	Body		-0.165734	0.0008112	0.7355783
chr9	cg16850173	5'UTR;1stExon;1stExon;1stExon;5'UTR;5'UTR	GRIN1;GRIN1;GRIN1;GRIN1;GRIN1	0.2131091	0.0008119	0.7355783
chr17	cg10801102	TSS1500	DUS1L;DUS1L;DUS1L	0.3379525	0.0008122	0.7355783

chr4	cg08750078			0.2427396	0.0008126	0.735 5783
chr7	cg09056595			0.1784963	0.0008131	0.735 5783
chr11	cg21842523	Body	LMO1;LMO1	0.1771883	0.0008136	0.73 5578 3
chr1	cg05642789	Body;Body;Body; Body;Body;Body	MIB2;MIB2;MIB2;MIB2;MIB2;MIB2; MIB2;MIB2;MIB2;MIB2	0.5258313	0.0008136	0.73 5578 3
chr19	cg26955996	TSS1500	CABP5	0.2553672	0.0008153	0.73 5578 3
chr21	cg05531134	TSS1500;TSS1500 ;TSS1500	C21orf91;C21orf91;C21orf91;C21orf91; CHODL	0.2712032	0.0008154	0.73 5578 3
chr1	cg11835068	TSS200;TSS200	AJAP1;AJAP1;AJAP1	0.1792183	0.0008164	0.73 5578 3
chr1	cg11424597	5'UTR;1stExon	ERI3;ERI3;ERI3;ERI3;ERI3;ERI3;ERI3; ERI3;ERI3;ERI3;ERI3	0.20587	0.0008177	0.73 5578 3
chr15	cg25933094	TSS200	ITPKA	0.1555461	0.0008185	0.73 5578 3
chr22	cg00115040		PPIL2	0.1024851	0.0008187	0.735 5783
chr1	cg23807924			- 0.2683296	0.0008215	0.737 1532
chr11	cg05218311	TSS200	ODZ4;ODZ4;ODZ4	0.2322531	0.0008264	0.73 9198 2

chr14	cg04510871	1stExon;5'UTR	RTN1;RTN1;RTN1	0.1466797	0.0008265	0.739198<br>2
chr19	cg26151999			0.3163293	0.0008268	0.739<br>1982
chr11	cg26825614	Body	PTS	0.1950933	0.0008311	0.740282<br>7
chr3	cg14067377	Body;Body	XXYLT1;XXYLT1;XXYLT1;XXYLT1;<br>XXYLT1	0.101048	0.0008313	0.740282<br>7
chr6	cg23667405	Body	PRRC2A;PRRC2A	-<br>0.1953976	0.000832	0.740282<br>7
chr4	cg13332538			0.1467643	0.0008334	0.740<br>2827
chr4	cg14924940			0.1284858	0.0008336	0.740<br>2827
chr9	cg21429410	5'UTR;5'UTR	LINGO2;LINGO2	0.2301996	0.0008342	0.740282<br>7
chr17	cg06420487	Body;Body		0.1478189	0.000836	0.741046<br>1
chr1	cg11132698	1stExon;5'UTR;TS<br>S1500	NOC2L;NOC2L;NOC2L;KLHL17;KLH<br>L17;KLHL17;KLHL17;KLHL17;KLHL<br>17;KLHL17;KLHL17	0.1200715	0.0008398	0.741469<br>9
chr11	cg14933468	Body;Body	ASRGL1;ASRGL1;ASRGL1	0.2996812	0.000841	0.741469<br>9
chr11	cg15788537			-<br>0.1805589	0.0008431	0.741<br>4699

chr1	cg00114008	TSS200	PIGV;PIGV;PIGV;PIGV;PIGV;PIGV;PIGV	0.2014277	0.0008459	0.7414699
chr14	cg07087646	Body;1stExon;TSS1500	MIR433;RTL1;MIR431	-0.1680673	0.0008465	0.7414699
chr6	cg25005633	TSS200;1stExon;1stExon;5'UTR;5'UTR	VARS2;VARS2;VARS2;VARS2;VARS2;VARS2;VARS2;VARS2;VARS2;VARS2;VARS2;VARS2	0.1461661	0.0008478	0.7414699
chr1	cg16718920	TSS1500	HP1BP3;HP1BP3;HP1BP3;HP1BP3;HP1BP3;HP1BP3;HP1BP3;HP1BP3;HP1BP3	0.1225712	0.0008521	0.7414699
chr6	cg02897675	5'UTR	TRIM26;TRIM26;TRIM26;TRIM26;TRIM26	-0.1389279	0.0008536	0.7414699
chr9	cg17927434	Body;Body		0.1552047	0.0008544	0.7414699
chr16	cg01214240			-0.3201837	0.0008553	0.7414699
chr1	cg11706911	Body	DNAJC16	0.2357948	0.0008557	0.7414699
chr12	cg13426510	Body;Body;Body	CPM	0.2910948	0.0008573	0.7414699
chr19	cg11894951	Body		0.1733766	0.0008586	0.7414699
chr19	cg19617213	Body		0.1339153	0.0008597	0.7414699

chr17	cg12999453	Body	TBX2;TBX2	0.1584518	0.0008598	0.7414699
chr7	cg06556068		AC003985.1	-0.1117516	0.0008599	0.7414699
chr2	cg08881168	Body		-0.1209476	0.0008599	0.7414699
chr17	cg03908270	Body;Body;Body;5'UTR;5'UTR;TSS200;TSS200	PCYT2;PCYT2;PCYT2;PCYT2;PCYT2;PCYT2;PCYT2	0.2864221	0.0008601	0.7414699
chr17	cg09730211	TSS200;TSS200	MMP28	0.1679821	0.0008612	0.7414699
chr6	cg19637177	5'UTR	SESN1;CEP57L1	0.1412371	0.0008613	0.7414699
chr12	cg03552327	Body;TSS1500;Body;Body	NCKAP5L;RP11-70F11.6;RP11-70F11.6;RP11-70F11.6;RP11-70F11.6;NCKAP5L;RP11-70F11.6;NCKAP5L	0.1728459	0.0008651	0.7414699
chr17	cg09435821	5'UTR	RBFOX3;RBFOX3	-0.1813644	0.0008654	0.7414699
chr2	cg07791671	5'UTR;1stExon;5'UTR;TSS200;5'UTR;5'UTR;1stExon	SEPT2;SEPT2;SEPT2;SEPT2;SEPT2;SEPT2;SEPT2;SEPT2;HDLBP;HDLBP;SEPT2;HDLBP;SEPT2;SEPT2;SEPT2;SEPT2;SEPT2;SEPT2;SEPT2;SEPT2;SEPT2;HDLBP;HDLBP;SEPT2;SEPT2;SEPT2;HDLBP;SEPT2;HDLBP;SEPT2;SEPT2;	0.1050246	0.000866	0.7414699

			SEPT2;SEPT2;SEPT2;SEPT2;SEPT2;SEPT2;HDLBP;SEPT2;SEPT2;SEPT2;SEPT2;SEPT2;SEPT2;SEPT2;SEPT2;SEPT2;SEPT2;SEPT2;SEPT2;SEPT2;SEPT2;SEPT2;HDLBP			
chr4	cg18005867	TSS200	ZNF876P	-0.1737434	0.0008669	0.741469 9
chr5	cg18389568			-0.156789	0.0008693	0.741 4699
chr2	cg23075101	TSS200	BARD1;BARD1;BARD1;BARD1;BARD1	0.2446593	0.00087	0.741469 9
chr9	cg13754661	TSS1500	COL5A1	0.118242	0.0008705	0.741469 9
chr3	cg07780856	TSS1500;TSS1500;1stExon;TSS1500;1stExon;TSS1500	ALG3;ALG3;ALG3;ALG3;ALG3;ALG3;ALG3;ECE2;ECE2;ALG3;ALG3	0.0967813	0.0008707	0.741469 9
chr12	cg07919476	Body;Body;Body; Body;Body;Body	ERC1	0.1737988	0.0008709	0.741469 9
chr1	cg15121510			0.1818323	0.000871	0.741 4699
chr11	cg18492647	TSS1500;5'UTR;5'UTR	RASGRP2;RASGRP2;RASGRP2;RASGRP2;RASGRP2;RASGRP2;RASGRP2;RASGRP2;RASGRP2;RASGRP2;RASGRP2;RASGRP2;RASGRP2;RASGRP2;RASGRP2;RASGRP2	0.2309816	0.0008743	0.741469 9
chr14	cg12930714	TSS1500	NFATC4;NFATC4;NFATC4;NFATC4	0.2285391	0.0008743	0.741469 9

chr19	cg24027100		AC092296.1;AC092296.1	0.2691401	0.0008761	0.7414699
chr12	cg26410484	TSS1500;TSS1500;TSS1500;TSS1500;TSS1500	CHFR;CHFR;CHFR;CHFR;CHFR;CHFR;CHFR;CHFR;RP11-46H11.12;RP11-46H11.5;CHFR;CHFR;CHFR;CHFR	0.1481086	0.0008762	0.7414699
chr22	cg03044200		IGLV3-32	0.1782029	0.0008762	0.7414699
chr9	cg11509404	TSS200	GABBR2	0.2305838	0.0008764	0.7414699
chr9	cg14339116			0.2066241	0.0008766	0.7414699
chr9	cg12207024	TSS1500;TSS1500;TSS200;TSS200;TSS200	GFI1B;GFI1B;TSC1;TSC1;TSC1;TSC1	0.1312957	0.0008779	0.7414699
chr6	cg14848259	Body	SYNGAP1;SYNGAP1	0.1761789	0.0008789	0.7414699
chr19	cg14032725	Body	DMWD	-0.1787713	0.0008801	0.7414699
chr7	cg14159963	1stExon;1stExon	CAV2;CAV2;AC002066.1;CAV2;AC002066.1;CAV2;CAV2;CAV2;CAV2;CAV2;CAV2;CAV2;CAV2;CAV2;CAV2;CAV2	0.2077413	0.0008803	0.7414699
chr10	cg22832231		RP11-107C16.2	-0.3029173	0.0008806	0.7414699
chr4	cg10335018		RP11-44F21.5;RP11-44F21.5;RP11-44F21.5;RP11-44F21.5	-0.1689121	0.0008813	0.7414699
chr17	cg02745847	Body;Body	IGF2BP1;RP11-501C14.5;IGF2BP1	0.2753825	0.0008815	0.7414699

chr3	cg07279281	TSS200	ALS2CL;ALS2CL;ALS2CL;ALS2CL;ALS2CL	0.1432911	0.0008849	0.742145 2
chr8	cg20027456	Body;Body	TRAPPC9;TRAPPC9;TRAPPC9	-0.2098155	0.0008853	0.742145 2
chr20	cg12600163	Body;Body;TSS200	NAA20;NAA20;NAA20;NAA20;NAA20;NAA20;NAA20	0.1229293	0.0008855	0.742145 2
chr2	cg23700226	TSS1500;TSS1500;TSS1500;1stExon;1stExon;1stExon;1stExon;5'UTR;5'UTR;5'UTR;5'UTR	AC018737.1;AC018737.1;AC018737.1;AC018737.1;AC018737.1;CLASP1;CLASP1;CLASP1;CLASP1	0.0939621	0.0008865	0.742145 2
chr19	cg09911399	TSS200;TSS1500	RPS11;SNORD35B	0.1383549	0.0008906	0.742145 2
chr2	cg01675229		AC073043.2;AC073043.2;AC073043.2	0.1581087	0.0008909	0.742 1452
chr16	cg21964055	TSS200;TSS200;TSS200;TSS200	NUDT7;NUDT7;NUDT7;NUDT7	0.1589057	0.0008911	0.742145 2
chr5	cg09886864	TSS1500;TSS1500	SLC36A3	-0.230855	0.0008914	0.742145 2
chr19	cg04080057	TSS1500;TSS1500;TSS1500	CACNG6;CACNG6	0.1556235	0.0008928	0.742145 2
chr3	cg02343881	5'UTR;5'UTR	PLD1;PLD1;PLD1;PLD1;PLD1;PLD1;PLD1	0.1638569	0.0008931	0.742145 2

chr14	cg20337720	Body;Body;TSS1500;Body;Body;TSS200	NFATC4;NFATC4;NFATC4;NFATC4;NFATC4;NFATC4;NFATC4;NFATC4;NFATC4	0.2085964	0.0008935	0.7421452
chr15	cg06207120		RP11-718O11.1;RP11-718O11.1	0.2479476	0.0008951	0.7426016
chr15	cg20259534	TSS200	BUB1B;BUB1B;BUB1B;BUB1B;BUB1B	0.176738	0.0008976	0.7438283
chr17	cg15472843			-0.1168814	0.0008987	0.7438283
chr1	cg26566407	Body	SKI;SKI	-0.1984509	0.0008996	0.7438283
chr11	cg25274320	TSS1500	ZNHIT2;ZNHIT2	-0.1544121	0.0009013	0.744386
chr17	cg03679521	5'UTR;1stExon	NEUROD2;NEUROD2	0.2410817	0.0009035	0.7453691
chr5	cg19416326	3'UTR	RNF44	-0.1571452	0.0009071	0.7466719
chr6	cg26009773	TSS200;TSS200	RP3-467N11.1;ASCC3;ASCC3;ASCC3;ASCC3;ASCC3	0.147469	0.0009108	0.7466719
chr1	cg04509099	Body		-0.1417347	0.0009129	0.7466719
chr12	cg04774914	Body	CERS5;CERS5;CERS5	0.1586506	0.0009144	0.7466719
chr15	cg20304530	Body;Body;Body		0.4624609	0.0009146	0.7466719

chr19	cg26009803	1stExon;1stExon;5'UTR;5'UTR	LSM14A;LSM14A;LSM14A;LSM14A	0.1358459	0.0009147	0.7466719
chr1	cg10085977	Body		-0.2008128	0.0009149	0.7466719
chr9	cg03559209	Body	RP11-85O21.5;LHX2	0.1793743	0.0009169	0.7466719
chr9	cg00559120	5'UTR;1stExon	COL15A1;COL15A1;COL15A1;COL15A1;COL15A1	0.2076	0.0009185	0.7466719
chr19	cg12952012	Body	ZNF460	0.1847929	0.0009189	0.7466719
chr2	cg06755871			-0.2293454	0.000919	0.7466719
chr11	cg21210690	Body		-0.1346553	0.0009192	0.7466719
chr3	cg06788675	1stExon;5'UTR;TSS200	DZIP3;DZIP3;KIAA1524;KIAA1524;KIAA1524;KIAA1524;DZIP3;DZIP3;DZIP3;DZIP3;DZIP3;DZIP3;DZIP3	0.1262845	0.0009199	0.7466719
chr7	cg06079564	Body;Body		0.2186992	0.0009201	0.7466719
chr2	cg11383961	TSS200;TSS200;TSS200	DTYMK;DTYMK;DTYMK;DTYMK;DTYMK;DTYMK;DTYMK	0.170206	0.0009206	0.7466719
chr16	cg01709119	Body;1stExon;TSS200;TSS200	FAM96B;FAM96B;FAM96B;CES2;FAM96B;FAM96B;CES2;FAM96B;FAM96B;CES2;FAM96B;CES2;FAM96B;CES2;CES2;CES2	0.1603426	0.0009237	0.7481505

chr7	cg16960046			0.1690287	0.0009271	0.7481505
chr16	cg19080964	Body;Body;Body	LMF1;LMF1;LMF1;LMF1	-0.1727575	0.0009283	0.7481505
chr11	cg13833525	5'UTR	PNPLA2;PNPLA2	0.1660161	0.0009291	0.7481505
chr22	cg13120357	Body;Body;Body	CABIN1	0.203178	0.0009292	0.7481505
chr17	cg00393814			-0.1289548	0.0009309	0.7481505
chr3	cg07031680	Body;Body		0.1404793	0.0009318	0.7481505
chr3	cg03446973	TSS200;TSS1500	RFC4;RP11-573D15.3;RFC4;RFC4;RFC4;RFC4;RFC4;RFC4;RFC4	0.1739886	0.0009319	0.7481505
chr17	cg01019746	1stExon;TSS1500	MSI2;MSI2;MSI2	0.1516148	0.0009321	0.7481505
chr19	cg00869285	TSS1500;TSS1500	ZNF837	0.4184452	0.0009327	0.7481505
chr21	cg26861066	TSS200	HMGN1;HMGN1;HMGN1;HMGN1;HMGN1;HMGN1;HMGN1;HMGN1;HMGN1;HMGN1;HMGN1;HMGN1;HMGN1;HMGN1;HMGN1;HMGN1;HMGN1;TMPRSS3	0.2401642	0.0009384	0.7486766
chr16	cg16410373	3'UTR	RP11-140H17.1;CYB5B;CYB5B;CYB5B;CYB5B;CYB5B	0.1345947	0.0009394	0.7486766

chr17	cg03319936	5'UTR;5'UTR;TSS1500;TSS1500;TSS200;TSS200	TADA2A;ACACA;ACACA;TADA2A;TADA2A	0.1364167	0.0009396	0.748676 6
chr8	cg02141358	Body		-0.1368446	0.0009417	0.748676 6
chr12	cg24365028	TSS200;TSS200;TSS200	PXN;PXN;PXN;PXN;PXN;PXN	0.1554619	0.0009418	0.748676 6
chr1	cg20014094	Body	RP11-288I21.1	-0.1445524	0.0009454	0.748676 6
chr11	cg09749064	5'UTR;Body;Body;Body	SYTL2;SYTL2	0.1964412	0.0009456	0.748676 6
chr4	cg17904698			-0.192204	0.0009465	0.748 6766
chr10	cg18676500	TSS1500	EBF3;EBF3	0.2302643	0.0009477	0.748676 6
chr11	cg19002337	Body;TSS1500;Body;5'UTR;TSS1500;TSS1500	IGF2;IGF2-AS1;IGF2-AS1;IGF2-AS1;IGF2;IGF2;INS-IGF2;IGF2;IGF2;IGF2	0.1237375	0.000948	0.748676 6
chr3	cg00404641	TSS200;TSS1500	NUDT16P;NUDT16P;RP11-933H2.4;RP11-933H2.4;RP11-933H2.4	0.3988527	0.0009481	0.748676 6
chr1	cg07593903			-0.1613178	0.0009483	0.748 6766
chr18	cg12317151	TSS1500	LPIN2	0.1324459	0.0009523	0.748676 6

chr10	cg25786525	Body	C10orf11	0.1693828	0.0009539	0.748676 6
chr11	cg24366211	TSS1500;TSS1500	CPT1A;CPT1A;CPT1A	0.1755521	0.0009551	0.748676 6
chr6	cg03806038	TSS200	TAB2;TAB2;TAB2	0.2370401	0.0009552	0.748676 6
chr7	cg08606227	1stExon;5'UTR;5'UTR;1stExon	HILPDA;HILPDA;RP11-155G14.5;METTL2B;HILPDA;HILPDA;HILPDA;HILPDA	0.1000498	0.0009584	0.748676 6
chr8	cg27436283			-0.3414488	0.0009621	0.7486766
chr7	cg03751356	TSS1500;TSS1500	ZNF789;ZNF789;ZNF789;ZNF789;ZNF789;ZNF789;ZNF789	0.110429	0.0009637	0.748676 6
chr10	cg15433798	TSS1500		-0.2796182	0.0009639	0.748676 6
chr18	cg25925582	TSS200;TSS200	VAPA	0.139091	0.0009646	0.748676 6
chr12	cg12890192	TSS200;Body;Body;Body	CDK2AP1;CDK2AP1;CDK2AP1	0.1879786	0.0009704	0.748676 6
chr15	cg22297146	5'UTR;1stExon	MYEF2;MYEF2;MYEF2;MYEF2;MYEF2;MYEF2;MYEF2;MYEF2;MYEF2;MYEF2	0.1592253	0.0009713	0.748676 6
chr14	cg13451153	Body;Body		0.1332576	0.0009715	0.748676 6

chr10	cg23875319	Body;Body	LHPP;LHPP;LHPP	-0.1082699	0.000974	0.748676 6
chr8	cg00678712	TSS200;5'UTR;5'UTR;1stExon;1stExon;Body;Body	FAM92A1;FAM92A1;FAM92A1;FAM92A1;FAM92A1;FAM92A1;FAM92A1;LINC00535;FAM92A1;FAM92A1;FAM92A1;FAM92A1;FAM92A1;FAM92A1;FAM92A1;FAM92A1;FAM92A1;FAM92A1;FAM92A1;FAM92A1	0.1353458	0.0009758	0.748676 6
chr11	cg20512711		RCN1;RCN1	0.1958673	0.000976	0.748 6766
chr1	cg06366937	TSS200;1stExon;5'UTR;TSS200	FPGT-TNNI3K;FPGT-TNNI3K;FPGT-TNNI3K;FPGT-TNNI3K;FPGT;FPGT-TNNI3K;LRRIQ3;LRRIQ3;FPGT;LRRIQ3;LRRIQ3;FPGT;FPGT;FPGT;FPGT;FPGT;FPGT;FPGT;FPGT;FPGT	0.1955027	0.0009763	0.748676 6
chr12	cg23142394	Body;TSS1500;Body	TRHDE;AC087886.1;TRHDE;AC087886.1;AC087886.1;AC087886.1;TRHDE	0.1798033	0.000977	0.748676 6
chr6	cg18596689	TSS1500	CLPS;CLPS	-0.1485348	0.0009771	0.748676 6
chr13	cg22700880			0.330745	0.0009785	0.748 6766
chr21	cg21703068	5'UTR;5'UTR;TSS1500	CRYZL1;CRYZL1;CRYZL1;CRYZL1;CRYZL1;CRYZL1;CRYZL1;CRYZL1;CRYZL1;CRYZL1;CRYZL1;CRYZL1;ITSN1;ITSN1;ITSN1;ITSN1;ITSN1;ITSN1;ITSN1;ITSN1;ITSN1	0.1408553	0.0009793	0.748676 6
chr17	cg15646911	TSS200;TSS200	ALDH3A2;ALDH3A2;ALDH3A2;ALDH3A2;ALDH3A2;ALDH3A2;ALDH3A2	0.2429726	0.00098	0.748676 6

chr8	cg11973777		PRAGMIN	0.2568689	0.0009809	0.7486766
chr12	cg24504927			0.1236374	0.000982	0.7486766
chr7	cg00858973	1stExon;5'UTR	C7orf41;C7orf41;C7orf41;C7orf41	0.1870054	0.0009824	0.7486766
chr9	cg13954127	Body	KCNT1;KCNT1	-0.184439	0.0009835	0.7486766
chr19	cg18437710	5'UTR;5'UTR;1stExon;5'UTR	KLK10;KLK10;KLK10;KLK10	0.1989382	0.0009837	0.7486766
chr8	cg24159559	TSS200;Body	RP11-770E5.3;RP11-567J20.1;RP11-770E5.1;AC026904.1	-0.1650423	0.0009839	0.7486766
chr4	cg00244517	5'UTR;1stExon	ARL9;ARL9	0.1417589	0.0009861	0.7486766
chr16	cg21821173			-0.1817548	0.0009869	0.7486766
chr6	cg11262669	TSS200;TSS200	TMEM30A;RP1-234P15.4;TMEM30A;TMEM30A;TMEM30A	0.1364214	0.0009871	0.7486766
chr20	cg18759081	Body	COL9A3;COL9A3;COL9A3;COL9A3;COL9A3;COL9A3;COL9A3	-0.2984198	0.0009886	0.7486766
chr6	cg02633491	TSS200;TSS200	GCLC;GCLC;GCLC;GCLC;GCLC	0.1695126	0.0009891	0.7486766
chr10	cg03312882			-0.1577646	0.0009892	0.7486766
chr3	cg18010752	Body	WNT5A	0.1970714	0.0009952	0.7486766

chr1	cg05664601	Body;Body;Body;Body	KIF2C;KIF2C;RP11-269F19.2;RP11-269F19.2	-0.1363567	0.0009964	0.7486766
chr19	cg21717879	TSS200;Body;TSS200	BSG;BSG;BSG;BSG;BSG;BSG;BSG	0.1265185	0.0009966	0.7486766
chr17	cg10185667	TSS1500;TSS1500;TSS1500	PRKAR1A;PRKAR1A;PRKAR1A	0.1321843	0.0009993	0.7486766

Supplementary Table S4. DMPs at nominal p < 0.001 in the discovery cohort BD/non-SA vs. CON EWAS.

chr	Probe ID	Gene feature	Gene annotation	logFC	p	FDR q
chr7	cg15653194	200	LFNG;LFNG	0.68872846	2.48E-07	0.09652354
chr3	cg20849127	3'UTR	C61A1	-0.1685978	2.66E-07	0.09652354
chr11	cg10817615	TSS200	CHST1;CHST1	-0.2257433	5.17E-07	0.12517706
chr11	cg25327839	5'UTR;1stExon	FAM168A;FAM168A	0.40689367	9.21E-07	0.15183763
chr3	cg13577407	n;1stExon	FLNB	0.41644172	1.05E-06	0.15183763
chr9	cg09340485	Body;Body;Body;Body	PALM2	0.22770982	1.88E-06	0.18353627
chr20	cg04254347	0	686C3.7;IDH3B;IDH3B;IDH3B;IDH3B;IDH3	0.21387041	2.24E-06	0.18353627
chr6	cg18356679		HEY2;RP11-624M8.1;RP11-624M8.1	0.30504516	2.73E-06	0.18353627
chr18	cg09348353	Body;Body		-0.1478581	3.19E-06	0.18353627
chr3	cg05666842	Body	CHCHD6	0.23477607	3.20E-06	0.18353627
chr3	cg19188060	Body	MD2;PSMD2;PSMD2;PSMD2;PSMD2;PSM	0.36396534	3.96E-06	0.18353627
chr16	cg03893095	Body;Body	TPPP3	-0.2864417	4.36E-06	0.18353627
chr8	cg15873460	Body		0.28188392	4.64E-06	0.18353627
chr7	cg10749792	TSS200	SPH;PSPH;CCT6A;PSPH;PSPH	0.28048865	4.73E-06	0.18353627
chr5	cg05272349	Body	PRDM6;PRDM6	-0.2499885	5.31E-06	0.18353627
chr1	cg25742246		PROX1-AS1	0.41393986	5.37E-06	0.18353627
chr7	cg03094728	Body;Body	AKAP9;AKAP9;AKAP9	0.33079505	5.57E-06	0.18353627
chr8	cg11973777		PRAGMIN	0.390347	5.93E-06	0.18353627
chr6	cg25098876	;5'UTR	BTBD9;BTBD9;BTBD9;BTBD9	-0.6068097	6.49E-06	0.18353627
chr8	cg17638730	TSS1500;TSS1500	219B4.7	0.37863423	6.56E-06	0.18353627
chr1	cg19313373	Body;Body		0.24048844	6.89E-06	0.18353627
chr19	cg22152279	Body;Body	TCF3	-0.2757559	7.07E-06	0.18353627
chr5	cg15327231	TSS1500	RP11-249M12.2	0.22770735	7.29E-06	0.18353627
chr14	cg26085966	;5'UTR	E2;SYNE2;SYNE2;SYNE2	0.34361832	7.42E-06	0.18353627
chr1	cg11831004			0.19269421	7.48E-06	0.18353627
chr14	cg25438338	1500;TSS1500;TSS200;	MPR2;GMPR2;GMPR2;GMPR2;GMPR2;GM	0.40338708	7.69E-06	0.18353627
chr1	cg22189070	xon;1stExon;5'UTR;5'U	FAM54B;FAM54B;FAM54B;FAM54B;FAM	0.2731374	7.86E-06	0.18353627
chr6	cg20478264	1stExon;TSS1500	HIST1H2BM;HIST1H2AJ	0.33305232	8.10E-06	0.18353627
chr4	cg06532184	y	783N5.1;TAPT1;TAPT1;TAPT1;TAPT1;TAP	0.27833017	8.18E-06	0.18353627
chr1	cg24104045	TSS200	D1L;FMO5	0.17888384	8.62E-06	0.18353627

chr10	cg21107950		SNORD60	0.2399971	8.65E-06	0.18353627
chr6	cg26576806	5'UTR;1stExon	POU3F2;POU3F2;POU3F2	0.18524378	9.09E-06	0.18353627
chr1	cg11857025	TSS200	TMEM167B;TMEM167B;TMEM167B	0.37532462	9.18E-06	0.18353627
chr5	cg26618989	Body	53O19.1;RP11-53O19.1;RP11-53O19.1;RP11-	0.26346928	9.64E-06	0.18353627
chr12	cg01805480	Body	FBRSL1;FBRSL1;FBRSL1	0.28674591	9.81E-06	0.18353627
chr6	cg13286323	TSS200	90J20.8;RP1-90J20.8;NQO2;NQO2;NQO2	0.21066228	1.00E-05	0.18353627
chr9	cg12157519	Body		0.25917618	1.04E-05	0.18353627
chr21	cg11758840	TSS1500	IFNAR1;IFNAR1;IFNAR1;IFNAR1	0.23920619	1.07E-05	0.18353627
chr19	cg19489987			0.20829232	1.07E-05	0.18353627
chr4	cg22452009		RP11-1J7.1	0.19679319	1.11E-05	0.18353627
chr13	cg00907661	TSS1500	ZDHHC20;ZDHHC20;ZDHHC20;ZDHHC20	-0.2121735	1.12E-05	0.18353627
chr12	cg26899692	;5'UTR	YRK2;DYRK2	0.36843848	1.14E-05	0.18353627
chr4	cg08928646	TSS1500;Body	453E17.1;UBA6;RP11-453E17.1;RP11-	0.1865956	1.15E-05	0.18353627
chr16	cg07019252	Body	IFT140;IFT140;IFT140;IFT140	-0.285307	1.15E-05	0.18353627
chr9	cg00424066	1500	MPDZ;MPDZ	0.22112313	1.15E-05	0.18353627
chr16	cg26885517			0.24614495	1.16E-05	0.18353627
chr20	cg13024359	5'UTR;1stExon	PARD6B;PARD6B;PARD6B	0.31830002	1.28E-05	0.19699995
chr4	cg14804342	TSS1500	SCRG1;SCRG1	0.22848123	1.35E-05	0.20460227
chr9	cg04258229	Body	COL27A1	0.19630545	1.39E-05	0.20567075
chr15	cg17520942	TSS200;TSS1500	RGMA;RGMA;RGMA	0.18441753	1.45E-05	0.20991944
chr8	cg15593965		ARHGAP39	-0.2507269	1.52E-05	0.21583414
chr1	cg05177851	5'UTR;5'UTR	2B3;EIF2B3	0.25489205	1.58E-05	0.21583414
chr21	cg17373656	5'UTR;1stExon	CSTB;CSTB;CSTB	-0.2011088	1.59E-05	0.21583414
chr21	cg13775054	Body;Body;5'UTR	AS1;MCM3AP-AS1;MCM3AP-	0.31304072	1.67E-05	0.21583414
chr18	cg17617360	S1500	MYL12A;MYL12A	0.19799483	1.71E-05	0.21583414
chr5	cg15765877		CTD-2007H13.3	0.2166752	1.74E-05	0.21583414
chr1	cg11664956	Body		0.20420465	1.76E-05	0.21583414
chr16	cg02218809	TSS200;TSS200	;TMEM219;TMEM219;TMEM219;TMEM21	-0.6370592	1.78E-05	0.21583414
chr19	cg24109215	Body;Body;Body		-0.2094391	1.78E-05	0.21583414
chr9	cg13564621	Body;Body;Body	C9orf3	0.20934523	1.79E-05	0.21583414
chr9	cg14065121	1stExon;5'UTR	9orf41	-0.6432835	1.84E-05	0.21583414
chr1	cg26015888	Body;Body	224A6.3;LINC00339;LINC00339;LINC00339;	0.24406936	1.84E-05	0.21583414

chr15	cg07466576			-0.3080991	1.88E-05	0.2166396
chr3	cg13051263		PLCL2	-0.2796122	1.93E-05	0.21900382
chr12	cg00301864	stExon	35;RAB35;RAB35;RAB35;RAB35;RAB35	0.21551998	1.97E-05	0.21900382
chr3	cg04489803	TSS1500	2021J15.1	0.25286965	2.03E-05	0.21900382
chr22	cg23015157	5'UTR;1stExon;5'UTR	FAM118A;FAM118A;FAM118A;FAM118A	0.17747449	2.10E-05	0.21900382
chr13	cg20293942		DLEU2;DLEU1;DLEU1;DLEU1;DLEU1	0.93299738	2.15E-05	0.21900382
chr1	cg13950447			0.17514607	2.19E-05	0.21900382
chr10	cg26472690	Body	INPP5A	-0.2952759	2.25E-05	0.21900382
chr1	cg12472246	Body;Body	UBE4B	0.23819689	2.26E-05	0.21900382
chr12	cg15830607	TSS200;TSS1500	HP	0.15255583	2.27E-05	0.21900382
chr9	cg04492963	5'UTR;1stExon	HEMGN;HEMGN	-0.2807585	2.27E-05	0.21900382
chr5	cg23866759	Body		-0.4714757	2.29E-05	0.21900382
chr7	cg13324057			0.20917321	2.36E-05	0.21900382
chr19	cg14576286	1stExon;5'UTR	ERF;ERF;ERF	0.3259636	2.36E-05	0.21900382
chr14	cg23138682	TSS200;5'UTR	F5;EIF5;EIF5;EIF5;EIF5;EIF5	0.39136272	2.36E-05	0.21900382
chr22	cg25301142	5'UTR;Body	MGXB4;HMGXB4	0.35199916	2.38E-05	0.21900382
chr13	cg07662945	Body		-0.1763827	2.38E-05	0.21900382
chr4	cg13873620			0.25026195	2.54E-05	0.22604727
chr4	cg20656602	TSS200	TIFA;TIFA;ALPK1	0.1652437	2.61E-05	0.22604727
chr12	cg08836319			0.23494538	2.63E-05	0.22604727
chr2	cg09333522		AC023115.3	0.18143715	2.65E-05	0.22604727
chr3	cg03446973	TSS200;TSS1500	573D15.3;RFC4;RFC4;RFC4;RFC4;RFC4;RF	0.2464038	2.70E-05	0.22604727
chr12	cg15635472	TSS1500;1stExon	TPCN1;TPCN1;TPCN1;TPCN1;TPCN1;TPC	0.52922593	2.72E-05	0.22604727
chr15	cg20473072	Body	RP11-198M11.2;RP11-198M11.2	0.24653335	2.74E-05	0.22604727
chr20	cg27250121	dy	719C8.1;PHACTR3;PHACTR3	0.29938351	2.77E-05	0.22604727
chr1	cg06807126	5'UTR;5'UTR	1A1;AKR1A1;AKR1A1	0.23808805	2.85E-05	0.22604727
chr17	cg22939704	5'UTR;5'UTR	CRHR1;CRHR1	0.1969835	2.85E-05	0.22604727
chr14	cg13692993			0.30300216	2.86E-05	0.22604727
chr1	cg23014221			0.19719544	2.90E-05	0.22604727
chr16	cg07055694		CDH11	0.32195846	2.94E-05	0.22604727
chr22	cg06957130	TSS200	CELSR1	0.35427045	3.03E-05	0.22604727
chr2	cg04959058	ExonBnd;Body	HEATR7B1	0.21456179	3.05E-05	0.22604727

chr2	cg13667965	Body;Body;Body;Body	9948.5;AC009948.5;AC009948.5	0.34060601	3.10E-05	0.22604727
chr2	cg11942603	5'UTR	A4;ANXA4;ANXA4	-0.2615708	3.10E-05	0.22604727
chr5	cg12464387	Body	RP11-61G23.1;RP11-61G23.1;RP11-61G23.1	0.3146919	3.12E-05	0.22604727
chr21	cg17148947	5'UTR;TSS1500	SETD4;SETD4;SETD4;SETD4;SETD4;SETD	0.20045955	3.14E-05	0.22604727
chr3	cg12404216	Body		-0.1312293	3.18E-05	0.22604727
chr18	cg04162656			0.26025815	3.22E-05	0.22604727
chr5	cg27001534	5'UTR;1stExon;TSS200	orf55	0.16435619	3.27E-05	0.22604727
chr1	cg05771459	Body;Body;Body;Body	4DIP;PDE4DIP;PDE4DIP;PDE4DIP;PDE4DI	0.19164295	3.31E-05	0.22604727
chr10	cg05284050			0.23941195	3.33E-05	0.22604727
chr22	cg21694059	Body	MYO18B	0.31513075	3.33E-05	0.22604727
chr3	cg13501912			-0.1993647	3.36E-05	0.22604727
chr7	cg02793750			-0.2821806	3.36E-05	0.22604727
chr4	cg01543582	Body	AF146191.4;FRG1;FRG1;FRG1	0.22944439	3.36E-05	0.22604727
chr6	cg15717840	Body;Body	SLC22A23	0.25080753	3.36E-05	0.22604727
chr3	cg10084513	5'UTR	A5B;SEMA5B;SEMA5B	0.2154014	3.41E-05	0.22655957
chr1	cg13499468	5'UTR;5'UTR	LUZP1;LUZP1;LUZP1;LUZP1	0.35036559	3.43E-05	0.22655957
chr8	cg09734791	1stExon	383H13.1;MSC;MSC;MSC;RP11-	0.13702987	3.56E-05	0.23166896
chr10	cg19562312	5'UTR;5'UTR;5'UTR	ZNF239;ZNF239;ZNF239;ZNF239	0.1543525	3.57E-05	0.23166896
chr5	cg12882476	1stExon;TSS200	CDC112;CCDC112;CCDC112	0.15439678	3.61E-05	0.23166896
chr10	cg12702211			-0.1978287	3.65E-05	0.2320887
chr2	cg08762806	Body		0.28308013	3.70E-05	0.23368095
chr19	cg04689371	;5'UTR	U2AF2;U2AF2	0.19034561	3.75E-05	0.23433692
chr13	cg05201784	TSS1500	KLF12	0.15720387	3.80E-05	0.23558954
chr14	cg23894571	TSS200;TSS200	173D9.3;RP11-	0.30489497	3.92E-05	0.23959593
chr2	cg16948082	TSS200	PER2;PER2;PER2	0.24551935	4.03E-05	0.23959593
chr17	cg19536407	3'UTR	BAHCC1	-0.220588	4.05E-05	0.23959593
chr7	cg16037806	TSS200;TSS200	AKAP9;AKAP9;AKAP9;AKAP9	0.2893209	4.12E-05	0.23959593
chr6	cg08259472	TSS200;Body	MAD2L1BP;MAD2L1BP	0.14034591	4.13E-05	0.23959593
chr17	cg25134567	Body;TSS1500	MIR634	-0.1796058	4.18E-05	0.23959593
chr20	cg11602459	TSS200;TSS200	1J6.2	0.21668875	4.22E-05	0.23959593
chr19	cg20147100	Body;Body	U6	0.15840664	4.25E-05	0.23959593
chr13	cg10910055	5'UTR;1stExon	FGF9	0.22073782	4.25E-05	0.23959593

chr22	cg26395601	SS200;1stExon;1stExon	OC5;THOC5;THOC5;THOC5;THOC5;THOC	0.26910438	4.26E-05	0.23959593
chr3	cg15141371	1stExon;5'UTR	CSRNP1;CSRNP1;CSRNP1;CSRNP1	0.21190397	4.27E-05	0.23959593
chr17	cg25431220	TSS200	1L1;TOM1L1;TOM1L1;TOM1L1;TOM1L1;T	0.16451456	4.27E-05	0.23959593
chr6	cg02043083	1stExon	MT1;PCMT1;PCMT1;PCMT1;PCMT1	-0.2819831	4.29E-05	0.23959593
chr18	cg12356311	Body;TSS1500	GATA6	0.32220677	4.38E-05	0.23985893
chr15	cg20046875	TSS200	1007O24.3;ARIH1;ARIH1;ARIH1;ARIH1;RP	0.2373243	4.40E-05	0.23985893
chr16	cg06485706	SS200	264B17.3;LAT;LAT;LAT;LAT;LAT;LAT;LA	0.25623254	4.44E-05	0.23985893
chr5	cg17413750			-0.1765496	4.48E-05	0.23985893
chr12	cg03209541	n	C1B;POC1B;POC1B;POC1B;POC1B;POC1B-	0.14799984	4.48E-05	0.23985893
chr19	cg19648552	Body;Body;Body		-0.3084359	4.49E-05	0.23985893
chr20	cg27359633	Body	DYNLRB1;DYNLRB1;DYNLRB1	0.16602748	4.58E-05	0.24260978
chr3	cg20173193	Body;Body	DHX30;DHX30	-0.1107732	4.63E-05	0.24367158
chr1	cg17429065	TSS200	ACOT7;ACOT7;ACOT7;ACOT7	0.31288539	4.71E-05	0.24590039
chr5	cg20433262	y;Body;1stExon	PCDHGA4	-0.2349854	4.80E-05	0.24590039
chr14	cg17817199	5'UTR;TSS200;TSS200	GTF2A1;GTF2A1;GTF2A1;GTF2A1	0.1547208	4.80E-05	0.24590039
chr1	cg09247534	TSS1500;TSS1500	C1orf9	0.22774423	4.81E-05	0.24590039
chr16	cg10033612	TSS1500	CRAMP1L;CRAMP1L;CRAMP1L	0.34079277	4.84E-05	0.24590039
chr2	cg18162294	TSS1500	ADCY3;ADCY3;ADCY3	0.21868703	4.95E-05	0.24965542
chr8	cg11775367		89K10.1;RP11-89K10.1	0.2182428	5.01E-05	0.2505342
chr15	cg21772626	TSS200;TSS200	RCN2;RCN2;RCN2;RCN2	0.21154399	5.08E-05	0.2505342
chr3	cg04894169	1500;Body	XPC;XPC;XPC;XPC	-0.4386776	5.10E-05	0.2505342
chr9	cg03890659	1stExon;5'UTR	APBA1;RP11-109D9.4	0.35176913	5.11E-05	0.2505342
chr3	cg10916702	00	AS1;WWTR1-	0.2408748	5.22E-05	0.25132908
chr14	cg04346127		CEBPE	0.25133967	5.24E-05	0.25132908
chr10	cg05226454	Body		0.20051106	5.26E-05	0.25132908
chr1	cg26447320	TSS200;TSS200	TTC22;TTC22	-0.2261195	5.27E-05	0.25132908
chr9	cg11311554	TSS200;TSS200	RP11-509J21.1;RFX3;RFX3	0.16200334	5.32E-05	0.25132908
chr17	cg12631853	Body;Body	SREBF1;SREBF1	0.20128226	5.33E-05	0.25132908
chr3	cg10747115	TSS200	SLC12A8;SLC12A8;SLC12A8;SLC12A8	0.17525692	5.45E-05	0.25388352
chr7	cg19319088	1stExon;5'UTR	FAM220A;FAM220A	0.1614932	5.46E-05	0.25388352
chr19	cg22537343	TSS1500	MIR525	0.18373562	5.61E-05	0.2556694
chr16	cg21094410	TSS200	ABAT;ABAT;ABAT	0.16885304	5.70E-05	0.2556694

chr4	cg06076996	TSS1500	366M4.3	0.29826009	5.79E-05	0.2556694
chr5	cg06025835	Body	PDE8B	0.26830093	5.89E-05	0.2556694
chr1	cg09133562	5'UTR;1stExon	MCO4;TMCO4	0.23280257	5.91E-05	0.2556694
chr20	cg10616337	TSS1500;TSS1500	CRLS1;CRLS1;CRLS1	0.18465662	5.93E-05	0.2556694
chr2	cg23723476			0.23113258	5.99E-05	0.2556694
chr2	cg17458653	Body;TSS200	PLEKHA3;PLEKHA3	0.33721776	5.99E-05	0.2556694
chr4	cg07223164	TSS1500	OSTC;OSTC;OSTC;OSTC	0.1900004	6.00E-05	0.2556694
chr11	cg26825614	Body	PTS	0.2639872	6.09E-05	0.2556694
chr5	cg00559992	Body		0.22668009	6.11E-05	0.2556694
chr4	cg18593893		RP11-608O21.1	0.20910769	6.11E-05	0.2556694
chr8	cg06824641	TSS200	PPP3CC	0.36864286	6.15E-05	0.2556694
chr4	cg10018232			0.24394622	6.24E-05	0.2556694
chr8	cg20027456	Body;Body	TRAPPC9;TRAPPC9;TRAPPC9	-0.2878025	6.26E-05	0.2556694
chr18	cg25010118	TSS1500	DSEL	0.32454916	6.27E-05	0.2556694
chr12	cg02876201	Body	E	0.18762612	6.29E-05	0.2556694
chr1	cg02505554	TSS200;TSS200	AS1;RP11-480I12.10	0.2358245	6.33E-05	0.2556694
chr7	cg04786207	TSS1500	EST;MEST;MEST;MEST;MEST	0.29125325	6.39E-05	0.2556694
chr8	cg00687766	ody;TSS1500;Body;TSS	2D2;ADAM9;ADAM9;ADAM9;ADAM9;TM	0.28312665	6.42E-05	0.2556694
chr5	cg01559436	TSS200;TSS200	RSK;TTC37;ARSK;ARSK	0.14606098	6.44E-05	0.2556694
chr8	cg23651403		392C11.1;CTA-392C11.1	0.31561805	6.45E-05	0.2556694
chr17	cg24289134	Body;Body	TTYH2	0.18550168	6.48E-05	0.2556694
chr1	cg03440588	TSS1500;TSS200	FOXD2;RP11-511I2.5	0.29122701	6.52E-05	0.2556694
chr1	cg22734925	1500;TSS1500;TSS1500	9;CASP9;CASP9;CASP9;CASP9;DNAJC16;	-0.3702071	6.54E-05	0.2556694
chr11	cg14538055	TSS1500	867G23.2;RAB1B	-0.4224402	6.55E-05	0.2556694
chr4	cg06520877			-0.2992211	6.58E-05	0.2556694
chr8	cg25219734	Body;Body		0.15751615	6.59E-05	0.2556694
chr4	cg02557534			0.20953484	6.59E-05	0.2556694
chr6	cg18353713	n;1stExon;1stExon;5'UT	ARD;PPARD	0.29714522	6.62E-05	0.2556694
chr19	cg27057602	TSS1500;TSS1500	CB1;TULP2;NUCB1;NUCB1;NUCB1;NUCB	0.24193067	6.66E-05	0.2556694
chr2	cg27211284			0.29420816	6.79E-05	0.2556694
chr17	cg13092355	TSS1500;TSS200	L;NT5C3L;NT5C3L;NT5C3L;NT5C3L;NT5C	0.25592155	6.82E-05	0.2556694
chr6	cg12322015	1500;TSS1500;TSS200	ECI2;RP3-400B16.1;RP3-400B16.4	0.25804689	6.82E-05	0.2556694

chr17	cg20123891	Body	RP5-1029K10.4	0.18007198	6.83E-05	0.2556694
chr9	cg12015691	5'UTR	AM214B;FAM214B;FAM214B;FAM214B;F	0.26170771	6.86E-05	0.2556694
chr1	cg08610987	Body	KIRREL	0.16991628	6.91E-05	0.2556694
chr16	cg09343327	5'UTR	YRM1;LYRM1;LYRM1;LYRM1;LYRM1;LY	0.2148943	6.94E-05	0.2556694
chr11	cg01865091	5'UTR;TSS1500	FRKB;NFRKB;NFRKB	0.18500902	7.04E-05	0.2556694
chr6	cg07413497	5'UTR;1stExon	C6orf57;C6orf57;C6orf57	0.20806009	7.06E-05	0.2556694
chr1	cg20059467	R;1stExon;1stExon	FIA	0.24095777	7.06E-05	0.2556694
chr8	cg22475582	1500;TSS1500	2L	0.16961499	7.09E-05	0.2556694
chr3	cg12345593	500	3;RRP9	0.24137622	7.13E-05	0.2556694
chr18	cg11348478	Body		0.277191	7.17E-05	0.2556694
chr19	cg17615134			-0.2611849	7.25E-05	0.2556694
chr1	cg24310780	Body		0.25150027	7.28E-05	0.2556694
chr3	cg07692746		SLCO2A1	0.19334718	7.31E-05	0.2556694
chr11	cg26349554	TSS200	SYTL2;SYTL2;SYTL2;SYTL2	0.24740715	7.43E-05	0.2556694
chr20	cg11362259	TR;5'UTR	ALY;RALY	-0.2168888	7.48E-05	0.2556694
chr5	cg04751031	n;5'UTR;5'UTR	AI14;RAI14;RAI14;RAI14	0.44253111	7.53E-05	0.2556694
chr3	cg24572995	5'UTR	L1;MBNL1;MBNL1;MBNL1;MBNL1;MBNL	0.21521023	7.55E-05	0.2556694
chr10	cg22888269	TSS200	SUPV3L1;SUPV3L1;SUPV3L1;SUPV3L1	0.15718671	7.60E-05	0.2556694
chr1	cg23510396	;Body;Body	CRB1;CRB1	0.17979921	7.60E-05	0.2556694
chr17	cg26202378	Body		-0.1766193	7.63E-05	0.2556694
chr2	cg18222526	TSS200	PAPOLG;PAPOLG;PAPOLG;PAPOLG	0.14923768	7.67E-05	0.2556694
chr1	cg26009832	Body;Body	ATP1B1	0.2601844	7.67E-05	0.2556694
chr5	cg23803153	0;TSS200	HINT1;HINT1;HINT1;HINT1	0.18053144	7.80E-05	0.2556694
chr2	cg01648310			0.28088458	7.81E-05	0.2556694
chr4	cg05570415	Body;Body;Body	SLC4A4	0.17479661	7.85E-05	0.2556694
chr10	cg02624067	Body		0.21890409	7.86E-05	0.2556694
chr15	cg03928353			0.20125737	7.89E-05	0.2556694
chr5	cg11394338			0.67499535	7.90E-05	0.2556694
chr6	cg10894807	Body;Body		-0.2588914	7.91E-05	0.2556694
chr17	cg10341935	TSS1500	CLTC;CLTC	0.14112953	7.91E-05	0.2556694
chr8	cg06943005	TSS200;TSS200	SCRIB;SCRIB	0.25800559	7.99E-05	0.2556694
chr5	cg03666316	Body	CTD-2384B11.2	0.15820336	8.03E-05	0.2556694

chr1	cg07600211	TSS200;TSS1500	HR2	0.28940827	8.05E-05	0.2556694
chr14	cg23664186	TSS1500	C6	0.25442785	8.11E-05	0.2556694
chr13	cg03133301	Body		0.22614498	8.13E-05	0.2556694
chr4	cg04662714	Body;TSS200	453E17.1;UBA6;RP11-453E17.1;RP11-	0.20825533	8.15E-05	0.2556694
chr3	cg16340161	5'UTR;Body	MECOM	0.17302344	8.21E-05	0.2556694
chr4	cg17439158	Body	M3;SHROOM3	0.35493675	8.36E-05	0.2556694
chr11	cg02783889	TSS1500;TSS1500	E2	0.18329911	8.37E-05	0.2556694
chr4	cg25766271		PPP2R2C	0.34806687	8.38E-05	0.2556694
chr2	cg18486825	TSS1500	KCNE4;KCNE4	0.17211133	8.38E-05	0.2556694
chr5	cg23928910	TSS1500	CETN3;CETN3;CETN3;CETN3	0.1976671	8.40E-05	0.2556694
chr7	cg16960046			0.23718131	8.45E-05	0.2556694
chr1	cg21773314	TSS200	TCEB3;TCEB3;TCEB3	0.18453125	8.49E-05	0.2556694
chr9	cg11635143	on;5'UTR;5'UTR	9orf85;FAM108B1;FAM108B1;FAM108B1	0.3647647	8.55E-05	0.2556694
chr22	cg17487553	0	PANX2;PANX2	0.32928303	8.59E-05	0.2556694
chr17	cg27327551	dy	EPN2-AS1;EPN2;EPN2;EPN2	0.2293883	8.67E-05	0.2556694
chr6	cg18104606	Body;Body	COL9A1	-0.2269889	8.70E-05	0.2556694
chr2	cg00436174	1stExon	C3;ERCC3;ERCC3;ERCC3;ERCC3	0.34968172	8.77E-05	0.2556694
chr13	cg20140092			0.17466903	8.84E-05	0.2556694
chr2	cg24095320	Body;Body;Body	Y_RNA;CDK15;CDK15	0.22200475	8.86E-05	0.2556694
chr18	cg05355058			0.22335137	8.86E-05	0.2556694
chr19	cg13711528	TSS200	ACTN4;ACTN4;ACTN4;ACTN4;ACTN4	0.25096476	8.94E-05	0.2556694
chr12	cg06406067			0.23386422	8.95E-05	0.2556694
chr2	cg10156861	0;TSS200;TSS200;TSS2	MFF;MFF;MFF;MFF;MFF;MFF;MFF;MFF;	0.21225009	8.96E-05	0.2556694
chr18	cg14064262	TSS200	NAPG;NAPG	0.20553857	8.96E-05	0.2556694
chr16	cg08298673			0.17526621	9.02E-05	0.2556694
chr10	cg11257689			0.2402615	9.02E-05	0.2556694
chr19	cg02709032	3'UTR;3'UTR	LRP3;SLC7A10	-0.3007671	9.05E-05	0.2556694
chr15	cg06022179		CTD-2335A18.1	0.34093489	9.17E-05	0.2556694
chr4	cg17987651		LRAT;LRAT;LRAT;LRAT;LRAT;LRAT	0.31059534	9.19E-05	0.2556694
chr17	cg15092787	Body		0.35527978	9.19E-05	0.2556694
chr19	cg18775767	TSS1500;TSS1500	ZNF837	0.15113828	9.22E-05	0.2556694
chr11	cg02877433	Body	DEAF1;DEAF1	-0.1716372	9.28E-05	0.2556694

chr19	cg05253604	TSS1500	CHERP;CHERP;CHERP;CHERP;CALR3	0.20067661	9.28E-05	0.2556694
chr3	cg27144791	on;5'UTR;5'UTR	L10;OSBPL10;OSBPL10	0.33894831	9.31E-05	0.2556694
chr2	cg17509807	5'UTR;5'UTR	C074117.10;GTF3C2;GTF3C2;GTF3C2;GTF	0.18663368	9.32E-05	0.2556694
chr8	cg20732620			0.20649205	9.37E-05	0.2556694
chr22	cg08825848	R	RD2;TXNRD2;TXNRD2;TXNRD2;TXNRD2	-0.1949132	9.37E-05	0.2556694
chr14	cg13629586			-0.249632	9.39E-05	0.2556694
chr11	cg06886059	TSS1500	DDX10;DDX10;DDX10;DDX10	0.26643273	9.41E-05	0.2556694
chr4	cg05295810	TSS200;TSS200	CASP6;CASP6;CASP6;CASP6	0.21869659	9.43E-05	0.2556694
chr11	cg14790883	5'UTR	ODZ4;ODZ4;ODZ4	0.20366651	9.53E-05	0.2556694
chr8	cg10477468	0;TSS200	4;PRKDC;PRKDC;PRKDC;MCM4;MCM4;P	0.2071358	9.54E-05	0.2556694
chr13	cg13254979		LINC00327	0.12424343	9.55E-05	0.2556694
chr13	cg25398486	UTR;1stExon;TSS200	48A;FAM48A;FAM48A;FAM48A;FAM48A;	0.13577414	9.55E-05	0.2556694
chr1	cg03320441	TSS1500	KIF26B	-0.2048796	9.61E-05	0.2556694
chr14	cg18815626	Body	ACTR10;C14orf37	0.28547035	9.65E-05	0.2556694
chr21	cg10158818		TMPRSS3	0.17952176	9.67E-05	0.2556694
chr6	cg25601320	TSS1500	RSPH3;RSPH3;RSPH3	0.26254269	9.74E-05	0.2556694
chr2	cg13070817			-0.1548357	9.83E-05	0.2556694
chr13	cg24328042	on;1stExon	ING1;ING1;ING1;ING1	0.20363742	9.83E-05	0.2556694
chr6	cg23024959		CMAHP	0.36315991	9.85E-05	0.2556694
chr3	cg14209399	Body;Body	ATP2B2;ATP2B2;ATP2B2	-0.1965073	9.88E-05	0.2556694
chr6	cg18866084	TSS1500;Body	PS6KA2	0.32784912	9.91E-05	0.2556694
chr15	cg00409183	TSS200	ZNF592	0.14848601	0.00010011	0.2556694
chr6	cg25729545	Body	;ABHD16A;ABHD16A;ABHD16A;ABHD16	-0.1253376	0.00010065	0.2556694
chr1	cg15392364	Body		0.1658093	0.00010143	0.2556694
chr14	cg24256211	Body;Body	SAMD4A	0.29547679	0.00010153	0.2556694
chr16	cg03745002	5'UTR;TSS1500	TOX3;TOX3;TOX3;TOX3	0.17230233	0.00010174	0.2556694
chr11	cg24358246		RP11-820L6.1;RP11-820L6.1;RP11-820L6.1	0.22614068	0.00010183	0.2556694
chr17	cg00918917	0;TSS200;TSS200;TSS2	STRADA	0.21277399	0.00010247	0.2556694
chr17	cg05208812	TSS200	ANKRD40;ANKRD40	0.21943678	0.00010254	0.2556694
chr17	cg03908270	;5'UTR;TSS200;TSS200	T2;PCYT2	0.39195118	0.00010342	0.2556694
chr12	cg15118523		AC006559.1;SLCO1A2;SLCO1A2;SLCO1A2	0.22354083	0.00010343	0.2556694
chr16	cg04558112	TSS200	NOB1;NOB1;NOB1;NOB1;NOB1	0.24783166	0.00010344	0.2556694

chr11	cg06425430	Body;TSS1500	PPC4;TRAPPC4;RPS25;RPS25;TRAPPC4;R	0.22153859	0.00010408	0.2556694
chr5	cg26797721	Body	RP11-93O7.5;CTC-369A16.2;CTC-369A16.3	0.20189115	0.00010443	0.2556694
chr5	cg15905124			0.1949014	0.0001045	0.2556694
chr4	cg08229950			0.2049791	0.00010581	0.2556694
chr5	cg06630943	Body;Body;Body;Body	FAM172A;FAM172A	0.21509121	0.00010593	0.2556694
chr2	cg22236276			-0.2144189	0.00010627	0.2556694
chr4	cg07367189	00;1stExon;5'UTR;TSS1	TBCK;TBCK;TBCK;AIMP1;TBCK;TBCK;T	0.26208615	0.00010654	0.2556694
chr17	cg13482628		209D14.2;SPECC1;SPECC1;SPECC1;SPECC	0.16886203	0.00010688	0.2556694
chr4	cg04879097			0.22457316	0.00010774	0.2556694
chr6	cg19177722	Body;Body	290I10.6;RP1-290I10.6;TFAP2A;TFAP2A	0.2642999	0.00010791	0.2556694
chr8	cg26372636	TSS200	SNAT	0.14918916	0.0001085	0.2556694
chr1	cg08853050	dy	PSMA5;PSMA5;PSMA5	0.18937592	0.00010858	0.2556694
chr13	cg23104954		DLEU1;DLEU1;DLEU1;DLEU1	0.6651566	0.00010868	0.2556694
chr19	cg22676763	TSS1500	4;CERS4;CERS4;CERS4;CERS4	0.27789759	0.00010945	0.2556694
chr4	cg04360780	1stExon	WHSC2;WHSC2;WHSC2;WHSC2;WHSC2;	0.13545723	0.00010974	0.2556694
chr17	cg10858828	Body;Body		-0.1677512	0.00011006	0.2556694
chr1	cg07049833	TSS1500	MIA3;MIA3;MIA3;MIA3	0.20163648	0.00011047	0.2556694
chr3	cg22495480	TSS1500	EIF1B;EIF1B;EIF1B;EIF1B	0.20538608	0.00011048	0.2556694
chr4	cg11976233	0	EXOC1;EXOC1;EXOC1	0.27359798	0.0001111	0.2556694
chr4	cg12264049	Body	MED28;AC006160.5	0.17602099	0.00011123	0.2556694
chr17	cg14385325	Body		-0.1276536	0.00011134	0.2556694
chr9	cg15246686	Body;Body	RP11-195E11.2	-0.1892017	0.00011179	0.2556694
chr17	cg22441074	TSS200	MNT;MNT;MNT;MNT;MNT;MNT	0.20209909	0.00011224	0.2556694
chr11	cg16705665	TSS200	RCOR2	0.21033811	0.00011241	0.2556694
chr7	cg03104936	5'UTR	10;GRB10;GRB10;GRB10;GRB10;GRB10;G	0.19852817	0.00011253	0.2556694
chr17	cg25364898			0.20934716	0.00011263	0.2556694
chr12	cg26856031	TSS1500	C12orf34;C12orf34	-0.5181085	0.00011265	0.2556694
chr19	cg03067574	Body	CCDC9	-0.1882184	0.00011325	0.2556694
chr7	cg20862743	Body	MKLN1;MKLN1;MKLN1	0.19583963	0.00011372	0.2556694
chr11	cg17504306	TSS1500	PHLDA2	0.2963573	0.00011406	0.2556694
chr6	cg12263857	Body	RNF217;RNF217	0.17636555	0.00011415	0.2556694
chr13	cg21278181	TSS200	PRHOXNB	0.19642932	0.00011455	0.2556694

chr7	cg20767025	TSS1500	CDC136;CCDC136;CCDC136	-0.3534411	0.00011582	0.2556694
chr7	cg19309562			0.23108299	0.00011638	0.2556694
chr22	cg00279778	;5'UTR;Body	PRR5;PRR5;PRR5;PRR5	-0.1718998	0.00011648	0.2556694
chr12	cg27211402	TSS200	554E23.4;CCDC53;CCDC53;CCDC53;CCDC	0.17425727	0.00011674	0.2556694
chr9	cg13604445	UTR	;RGS3;RP11-168K11.2;RGS3;RGS3;RGS3	0.24778113	0.00011686	0.2556694
chr1	cg07400091	5'UTR	S1PR1;S1PR1;S1PR1;S1PR1	0.17983499	0.00011749	0.2556694
chr7	cg00303745	TSS1500;TSS1500	ZPBP;ZPBP;ZPBP	0.18149756	0.00011769	0.2556694
chr3	cg06339171	Body		0.22036603	0.00011775	0.2556694
chr10	cg19204631	TSS200;TSS1500	CISD1;CISD1;IPMK	0.15185689	0.00011781	0.2556694
chr14	cg19741455	stExon;TSS1500;TSS15	CS4;SOCS4;WDHD1;WDHD1;SOCS4;SOCS	0.12583918	0.00011804	0.2556694
chr17	cg25826457	5'UTR;1stExon	HLF;HLF;HLF;HLF	0.20121164	0.00011829	0.2556694
chr22	cg25371038	5'UTR;1stExon	3B;TOP3B;TOP3B;TOP3B;TOP3B;TOP3B;T	0.28212248	0.00011874	0.2556694
chr6	cg17681549	on;5'UTR;5'UTR	;WDR27;WDR27;WDR27;WDR27	0.1468279	0.00011909	0.2556694
chr20	cg24788194	Body;Body		0.2858902	0.00011923	0.2556694
chr4	cg13082573	R	AT9;AGPAT9	0.12604187	0.00011968	0.2556694
chr5	cg08693705	0;TSS200;TSS200	129O4.1;MAPK9;MAPK9;MAPK9;MAPK9;	0.18550044	0.00011989	0.2556694
chr11	cg09685193	Body;Body;Body	MICAL2;MICAL2;MICAL2	0.21560368	0.0001204	0.2556694
chr8	cg24603408		RP11-459E5.1	0.27237654	0.0001211	0.2556694
chr12	cg01207748	Body;Body;Body;Body	AC068799.1;NOS1	0.23082926	0.00012121	0.2556694
chr1	cg04131943	TSS200;TSS200	4L;RAD54L;RAD54L	0.22539456	0.00012142	0.2556694
chr3	cg17644932	Body;Body	GSK3B	0.16360353	0.00012142	0.2556694
chr9	cg14375849	Body	COL15A1	-0.1721644	0.00012147	0.2556694
chr12	cg07615018	TR;Body	PC5;ANAPC5;ANAPC5;ANAPC5;ANAPC5;	0.17189885	0.00012227	0.2556694
chr3	cg04534726	TSS200;Body	NR1D2;NKIRAS1	0.16251581	0.00012255	0.2556694
chr11	cg14569209		SLC22A6	0.22595854	0.0001226	0.2556694
chr2	cg10626935	Body	RBM45	0.21695413	0.00012299	0.2556694
chr16	cg03669394	Body		0.29034652	0.00012359	0.2556694
chr7	cg23096130	Body	4540.5	0.19874696	0.00012389	0.2556694
chr11	cg15246362		RP11-469N6.1	0.24874441	0.00012433	0.2556694
chr12	cg01353809	TSS200	;ARID2;ARID2	0.14750606	0.00012457	0.2556694
chr1	cg09605828	TSS1500;TSS1500	AU1AP;TRNAU1AP;TRNAU1AP;TRNAU1	-0.1945515	0.00012472	0.2556694
chr3	cg11137483	5'UTR	613B23.1;RP4-613B23.1;ZBTB47;ZBTB47	0.18188872	0.00012558	0.2556694

chr22	cg05453648	TSS200	RBX1;RBX1;XPNPEP3;XPNPEP3	0.21104983	0.00012558	0.2556694
chr15	cg03043296	Body	AP3B2;RP11-752G15.3	0.31401885	0.00012602	0.2556694
chr22	cg17192519	TSS1500	;JOSD1;JOSD1;JOSD1	0.3544787	0.0001264	0.2556694
chr1	cg12401731	5'UTR	CD160;CD160	0.18115865	0.00012654	0.2556694
chr20	cg11287061		RP4-776F14.3	0.27189924	0.00012671	0.2556694
chr8	cg09986977	TSS1500;Body	382J12.1;RP11-	0.15252917	0.00012671	0.2556694
chr20	cg01721538	TSS200;TSS200	SS18L1;PSMA7;PSMA7;SS18L1;PSMA7	0.16149786	0.00012704	0.2556694
chr2	cg02222722	TSS200	TTC32;TTC32;TTC32	0.22158297	0.00012705	0.2556694
chr4	cg05936438	5'UTR;5'UTR	COX3	0.15806516	0.00012755	0.2556694
chr5	cg22909935	Body		0.29991756	0.00012755	0.2556694
chr3	cg23138110		U6;RUVBL1	0.20816118	0.00012861	0.2556694
chr12	cg12020201	TSS200	F40B;PRPF40B;PRPF40B	0.31666027	0.00012868	0.2556694
chr11	cg01449504	ExonBnd;Body	ZP1;ZP1;ZP1	0.23272755	0.00012888	0.2556694
chr5	cg20031198			0.20870736	0.00012922	0.2556694
chr5	cg02711460	dy	MIR449C	0.19536883	0.00012943	0.2556694
chr16	cg11766101	y	2258A20.5;BEAN1;TK2;RP11-	0.23994969	0.00012953	0.2556694
chr8	cg24604091	Body		0.22900402	0.0001297	0.2556694
chr11	cg26304637		AP002856.6	0.20388776	0.00013018	0.2556694
chr22	cg25842101			0.2008236	0.00013026	0.2556694
chr6	cg05512065		RP1-177I10.1	0.26091232	0.00013033	0.2556694
chr2	cg07540722	TSS200	GMCL1;GMCL1	0.14144189	0.00013339	0.25697058
chr22	cg19063066	Body	XPNPEP3	-0.3637682	0.00013356	0.25697058
chr5	cg19472116	TSS200	CAMLG;CAMLG	0.17866591	0.00013364	0.25697058
chr14	cg08886000	TSS200;TSS200	2R5C;PPP2R5C;PPP2R5C	0.1693247	0.00013396	0.25697058
chr11	cg26438284	Body	CD81;CD81;CD81;CD81;CD81;CD81	-0.1646237	0.00013417	0.25697058
chr6	cg06357807	Body;Body;Body;Body		0.2403824	0.00013453	0.25697058
chr5	cg04706530			0.21960229	0.00013461	0.25697058
chr4	cg25583174	1stExon	FGF2	-0.3168666	0.00013479	0.25697058
chr15	cg23448096	5'UTR	MD4;LYSMD4;LYSMD4;LYSMD4;LYSMD	0.14130382	0.00013524	0.25697058
chr7	cg05092396	Body;Body;Body	HIBADH	0.1698958	0.00013563	0.25697058
chr3	cg24522105	1stExon;5'UTR	PIK3R4;PIK3R4	0.17652905	0.00013564	0.25697058
chr6	cg05039080		HEY2;RP11-624M8.1;RP11-624M8.1	0.29510074	0.00013576	0.25697058

chr3	cg23628276			0.20273704	0.00013612	0.25697058
chr10	cg04392284	Body	KNDC1	-0.2852818	0.00013635	0.25697058
chr4	cg18795809	5'UTR	F518B	0.14575599	0.0001368	0.25697058
chr21	cg17722823	TSS200	KRTAP13-4	0.19494867	0.00013683	0.25697058
chr9	cg26232708	TSS1500	UBE2R2	0.52762771	0.00013701	0.25697058
chr8	cg03900851	Body	FE1;ADHFE1;ADHFE1	0.1623629	0.0001376	0.25740904
chr13	cg12225854			0.2317092	0.00013835	0.25805856
chr19	cg23097143			0.22072661	0.00013866	0.25805856
chr12	cg25022342	TSS1500	SCAF11	0.27106961	0.00014015	0.25862378
chr3	cg12118082	1stExon;5'UTR	LCO2A1;SLCO2A1	0.18287646	0.00014032	0.25862378
chr10	cg26685590	TSS200	BNIP3;BNIP3	0.13932359	0.0001408	0.25862378
chr17	cg07733457	5'UTR;1stExon	333E1.1;ZNF594;ZNF594	0.19933695	0.00014146	0.25862378
chr3	cg01819017	Body	MC1	0.21391208	0.00014243	0.25862378
chr2	cg01986232		AC010987.5	0.29170815	0.0001426	0.25862378
chr14	cg21872383	Body	CCDC88C;CCDC88C	-0.2369458	0.00014302	0.25862378
chr8	cg06294637	;5'UTR;5'UTR;5'UTR;1s	CBP;SDCBP;SDCBP;SDCBP;SDCBP;SDCB	0.15688791	0.00014315	0.25862378
chr10	cg01901893			0.20136058	0.00014359	0.25862378
chr1	cg23855566	TSS200	RN5S21;FAM73A;FAM73A;FAM73A	0.19330332	0.00014365	0.25862378
chr7	cg07757108		GPR141;GPR141;EPDR1;GPR141	0.22305683	0.00014383	0.25862378
chr11	cg04357390			0.18277362	0.00014404	0.25862378
chr2	cg24906316		AC010872.2	0.16683562	0.00014415	0.25862378
chr20	cg17030437		EFCAB8	0.1745696	0.00014417	0.25862378
chr2	cg04946715	5'UTR	SSB;SSB;SSB;SSB;SSB;SSB;SSB;SSB	0.15712019	0.00014439	0.25862378
chr10	cg20973769	Body;Body		0.27545734	0.00014517	0.25862378
chr1	cg24444188	Body	RP11-168O16.1;RP11-168O16.1	0.19733441	0.00014554	0.25862378
chr10	cg11028409	TSS200	CUEDC2;CUEDC2;CUEDC2	0.19540669	0.00014568	0.25862378
chr1	cg16085019			0.19354546	0.00014575	0.25862378
chr17	cg25026715	TSS200;TSS1500	SUPT6H;SDF2;SDF2;SDF2;SDF2	0.27598289	0.00014658	0.25862378
chr8	cg07058706		SNORA67	0.20367322	0.00014712	0.25862378
chr14	cg22837406	5'UTR;5'UTR	C14orf118;C14orf118;C14orf118	0.2779518	0.00014803	0.25862378
chr11	cg25320665	Body;5'UTR	725K16.4	0.23464954	0.00014821	0.25862378
chr8	cg16356472	5'UTR;5'UTR;5'UTR	SLC30A8;SLC30A8;SLC30A8;SLC30A8	0.20858236	0.00014871	0.25862378

chr13	cg07615111			0.44273341	0.00014897	0.25862378
chr1	cg09170397	Body	CACNA1E;CACNA1E;CACNA1E	0.17170565	0.00014929	0.25862378
chr2	cg14719675	TSS1500	ADCY3;ADCY3;ADCY3	0.32644435	0.00014945	0.25862378
chr1	cg00072172	Body	U3	0.25805185	0.00014982	0.25862378
chr19	cg08901057	Body;Body;Body;Body		-0.2524689	0.00015032	0.25862378
chr13	cg04779999	TSS200;TSS200	48A;FAM48A;FAM48A;FAM48A;FAM48A;	0.24976519	0.00015052	0.25862378
chr2	cg24752028	;5'UTR;1stExon;1stExo	5;YPEL5;YPEL5	0.16014348	0.00015073	0.25862378
chr5	cg00859327	Body	STK10	0.18653282	0.00015074	0.25862378
chr1	cg14212631	1stExon	ASPM;ASPM	0.26457635	0.00015115	0.25862378
chr4	cg11465971	5'UTR;1stExon	GSX2;GSX2;GSX2;GSX2;GSX2	0.31968952	0.00015117	0.25862378
chr1	cg08541953	Body	RPL22	0.23807114	0.00015169	0.25862378
chr5	cg05576925	R	DKD1;NADKD1;NADKD1	0.1270113	0.000152	0.25862378
chr16	cg23175613	TSS200;TSS200	IFC3;KIFC3;KIFC3;CTD-	0.22017045	0.00015214	0.25862378
chr14	cg21410754	TSS200;Body	HSP90AA1;HSP90AA1;HSP90AA1	0.26765117	0.00015319	0.2591843
chr3	cg02384255	Body	GALNTL2	0.1751555	0.00015358	0.2591843
chr11	cg23719950	TSS200	OD1	0.4717089	0.0001538	0.2591843
chr19	cg06620911	stExon	ANGPTL4;ANGPTL4;ANGPTL4	0.30962179	0.0001539	0.2591843
chr8	cg11249851	TSS1500	OX6C;COX6C;COX6C;COX6C	0.20125789	0.00015482	0.26013242
chr1	cg11219407	TSS1500	UBE2Q1;UBE2Q1;UBE2Q1	0.1187691	0.00015568	0.26048991
chr11	cg03922588	00;Body;Body;Body;Bo	;BSCL2;BSCL2;BSCL2;RP11-831H9.16	0.34761578	0.00015619	0.26048991
chr5	cg21677976			-0.1237037	0.00015624	0.26048991
chr10	cg14034858	TSS200	LRIT1	0.50010959	0.00015647	0.26048991
chr16	cg07721872	Body	10536.1	-0.20833	0.00015709	0.26092953
chr7	cg21189070	TSS1500	CTB;ACTB;ACTB;ACTB;ACTB	0.20925403	0.00015787	0.26161926
chr2	cg05370471		AC009404.2;AC009404.2;AC009404.2	-0.182355	0.00015915	0.26199974
chr8	cg09300749	Body;Body	SCRIB;SCRIB	-0.2362355	0.0001601	0.26199974
chr20	cg03373518	TSS200	FAM210B;FAM210B	0.16890008	0.00016056	0.26199974
chr10	cg25639195	5'UTR;5'UTR	3K8;MAP3K8;MAP3K8	0.31349075	0.00016057	0.26199974
chr13	cg12868449	TSS1500;Body;Body	DOCK9;DOCK9-AS1	0.19789671	0.00016082	0.26199974
chr19	cg15442610	TSS200	PIK3R2;PIK3R2	0.4728432	0.0001613	0.26199974
chr20	cg23054166	TSS200;TSS1500	CRLS1;CRLS1;CRLS1	0.14479803	0.0001617	0.26199974
chr4	cg17252605	TSS200;TSS200	INPP4B;INPP4B;INPP4B;INPP4B;INPP4B	0.21297881	0.00016237	0.26199974

chr12	cg15844144	TSS200	FAM86FP	0.19068852	0.00016278	0.26199974
chr2	cg09566392	TSS200	C3;PQLC3;PQLC3	0.26158487	0.00016299	0.26199974
chr12	cg08160641	TSS1500	NSC1	0.24030953	0.00016327	0.26199974
chr2	cg15781375	5'UTR	NRBP1;NRBP1;NRBP1;NRBP1;NRBP1	0.35592339	0.00016403	0.26199974
chr13	cg18474152	Body	MRPS31;MRPS31	0.14697885	0.00016409	0.26199974
chr10	cg06185924	Body	CBD5;ACBD5;ACBD5;ACBD5	0.40104365	0.00016434	0.26199974
chr2	cg08744769	1stExon	MXD1	0.25078558	0.00016437	0.26199974
chr16	cg27373657	TSS200;TSS200	1;AP1G1;AP1G1;AP1G1;AP1G1;AP1G1;AP1	0.13606172	0.00016514	0.26199974
chr5	cg15101292	500;TSS1500;TSS1500;	NIP1;TNIP1;TNIP1;TNIP1;TNIP1;TNIP1	0.14257241	0.00016552	0.26199974
chr1	cg06825153	TSS200;5'UTR	LRRC8D;LRRC8D;LRRC8D	0.14967633	0.00016578	0.26199974
chr17	cg23371940	Body		0.17923159	0.00016611	0.26199974
chr18	cg20521672	Body		0.18425365	0.00016782	0.26199974
chr10	cg05043757		RP11-556E13.1	0.37233426	0.00016803	0.26199974
chr18	cg17571306			0.2227142	0.00016816	0.26199974
chr1	cg24602411		RP5-1011O1.2	0.20549684	0.00016851	0.26199974
chr16	cg02530705		RP11-715J22.2	0.14819949	0.00016853	0.26199974
chr21	cg12581717	Body;Body;5'UTR	AS1;MCM3AP-AS1;MCM3AP-	0.13651676	0.00016912	0.26199974
chr6	cg05281544	3'UTR;1stExon	SOX4;SOX4	0.17238617	0.00016917	0.26199974
chr10	ch.10.109839658F			0.7057205	0.00016928	0.26199974
chr4	cg12639835	Body	SORCS2	0.23163266	0.00016986	0.26199974
chr21	cg04285033			0.20154673	0.00016987	0.26199974
chr1	cg08918937			0.26868129	0.00016988	0.26199974
chr12	cg04237898	Body;1stExon;5'UTR	ALDH1L2;ALDH1L2	0.34346325	0.00016995	0.26199974
chr3	cg23818738		RP11-389G6.3	0.21309042	0.00017006	0.26199974
chr2	cg09509080	Body;Body;Body	BCL11A;BCL11A;BCL11A;BCL11A	0.21648171	0.00017014	0.26199974
chr12	cg16864198		SSPN;SSPN;SSPN	0.20687479	0.00017072	0.26199974
chr12	cg13096470			0.15348759	0.00017089	0.26199974
chr22	cg19194233	1stExon;5'UTR;TSS200	LC1;MLC1;MLC1;MLC1;MLC1;MLC1	0.19858556	0.00017137	0.26199974
chr2	cg13487054	5'UTR;5'UTR	KCNS3;KCNS3;KCNS3;KCNS3;KCNS3	0.24626219	0.00017145	0.26199974
chr16	cg27554551	TSS1500;Body	VASN;CORO7;CORO7;CORO7	-0.2342015	0.000172	0.26228128
chr17	cg20544410	TSS200;TSS200	TMEM11;AC087294.2	0.29998861	0.00017269	0.26229128
chr1	cg08706837	TSS200	422J8.1	0.31128228	0.00017273	0.26229128

chr6	cg24018550	Body;Body;Body;Body	IPCEF1;OPRM1	0.19115387	0.00017347	0.26263423
chr11	cg04972224	5'UTR;TSS1500	MMP1L;IMMP1L;IMMP1L;IMMP1L;IMMP1	0.1897616	0.00017399	0.26263423
chr6	cg19223274	TSS200	AIG1;AIG1;AIG1;AIG1;AIG1;AIG1;AIG1	0.22829638	0.00017475	0.26263423
chr4	cg03967431	Body;Body;Body	EIF4E;EIF4E;EIF4E;EIF4E	0.16566516	0.00017476	0.26263423
chr14	cg01517370			0.15639432	0.00017476	0.26263423
chr1	cg25192948	1stExon;5'UTR	1;KHDRBS1;KHDRBS1	0.28912982	0.0001761	0.26325497
chr2	cg19850959	TSS200;5'UTR	FAM176A;FAM176A;FAM176A;FAM176A;	0.16058481	0.00017616	0.26325497
chr4	cg23069057		LINC00504;LINC00504;LINC00504	0.25518043	0.00017627	0.26325497
chr12	cg01579535	5'UTR;TSS1500	HOXC4;HOXC4	0.31510432	0.00017723	0.26414434
chr16	cg07710686	3'UTR	RP11-510J16.3	0.21736966	0.00017787	0.26432237
chr6	cg05063680			0.17029194	0.0001782	0.26432237
chr7	cg10606640			0.2083077	0.0001786	0.26432237
chr14	cg01046434	TSS1500	PNP;PNP;PNP;PNP;PNP;PNP	0.20800152	0.0001788	0.26432237
chr5	cg05851148	Body;TSS1500;Body	MGAT4B;MGAT4B;MIR1229;MGAT4B	-0.2194096	0.00018024	0.26541887
chr13	cg11122148	TSS200	TSC22D1;TSC22D1;TSC22D1	0.3927304	0.00018029	0.26541887
chr4	cg08118646	n	4E;EIF4E;EIF4E;EIF4E	0.14500839	0.00018175	0.26541887
chr19	cg18462898	ody	MEF2B;MEF2B;MEF2B	-0.2830233	0.00018211	0.26541887
chr18	cg05812269	1stExon;5'UTR	RIOK3;RIOK3	0.19355985	0.00018214	0.26541887
chr20	cg01101127	3'UTR;3'UTR	RIMS4;RP4-781B1.2;RIMS4	0.16612234	0.00018242	0.26541887
chr13	cg16058571			0.17971423	0.00018296	0.26541887
chr5	cg09420988	1stExon	53O19.1;RP11-53O19.1;RP11-53O19.1;RP11-	0.15891042	0.00018329	0.26541887
chr9	cg14717126	200;TSS200;1stExon;Bo	H1;PTCH1	0.14932692	0.00018342	0.26541887
chr10	cg16119628	TSS1500	77P6.2;WAPAL;WAPAL	0.15578572	0.00018359	0.26541887
chr7	cg13990958	TSS1500;TSS1500	DAGLB;DAGLB;DAGLB	0.27096096	0.00018371	0.26541887
chr12	cg02564061	Body;Body	FAM186B;PRPF40B;FAM186B	-0.2898478	0.00018393	0.26541887
chr14	cg00820637	TSS1500	FRMD6;FRMD6;FRMD6-AS2	0.20433167	0.00018431	0.26543338
chr7	cg26896093		AC004485.3	0.21289937	0.00018499	0.26589152
chr12	cg05775742			0.23412502	0.00018577	0.26648719
chr1	cg26377940		RP11-373J16.1	0.26218629	0.00018665	0.26689927
chr13	cg13108194	TR	NG1	0.18474501	0.00018724	0.26689927
chr5	cg15044825	Body	CTD-2297D10.1;ADAMTS16	0.21030722	0.00018757	0.26689927
chr17	cg09795194	stExon	ULK2;ULK2;ULK2;ULK2	0.1732598	0.00018839	0.26689927

chr13	cg11115582			0.17307646	0.0001884	0.26689927
chr1	cg14594621			0.21209329	0.00018857	0.26689927
chr2	cg00244654	TSS200	B;TAF1B;TAF1B;TAF1B;TAF1B	0.12177708	0.00018885	0.26689927
chr6	cg21565309	Body	RPP21;RPP21;RPP21;RPP21;RPP21;RPP21	0.23638154	0.00018924	0.26689927
chr1	cg09227621	Body		0.32290153	0.00018938	0.26689927
chr17	cg03734677	Body		-0.2154777	0.00018974	0.26689927
chr11	cg09200665	ody;Body	PS3;RPS3;RPS3	0.26286145	0.00019014	0.26695465
chr6	cg00595324			0.18800549	0.00019055	0.26700678
chr20	cg16720605	Body		0.18438342	0.00019162	0.26727966
chr1	cg13458335	TSS1500	RP1-118J21.25;BMP8B;BMP8B	-0.2098487	0.00019193	0.26727966
chr7	cg08563433			0.17068939	0.00019224	0.26727966
chr15	cg20122925			0.26504824	0.00019243	0.26727966
chr10	cg03610228			0.32962208	0.00019259	0.26727966
chr15	cg27341933	1stExon	TPLAD1;PTPLAD1;PTPLAD1;PTPLAD1;PT	-0.5162781	0.00019344	0.2673884
chr1	cg18516252	0;TSS200	98D18.9;TDRKH;TDRKH;TDRKH;TDRKH;	0.17767478	0.00019369	0.2673884
chr7	cg15910880	0;TSS200	MLXIPL;MLXIPL;MLXIPL;MLXIPL	0.22854908	0.00019409	0.2673884
chr11	cg16612699	TSS1500	OR8B8	0.19186323	0.00019432	0.2673884
chr11	cg06371967	Body		0.29363151	0.00019469	0.2673884
chr14	cg23069277		RP11-662J14.2	0.22773734	0.00019487	0.2673884
chr1	cg15988263	TSS200	7O11.3;RP11-7O11.3	0.12116064	0.00019766	0.26844835
chr11	cg06908474	1stExon;5'UTR	CAT;CAT	-0.6988294	0.00019785	0.26844835
chr2	cg07468037		AC108025.2;AC107057.2;AC108025.2	0.15627405	0.00019813	0.26844835
chr12	cg02149508	TSS1500	768F21.1	0.16893651	0.00019879	0.26844835
chr9	cg14970612	TSS1500	GCNT1;GCNT1;GCNT1	0.19243538	0.00019881	0.26844835
chr3	cg04117693			0.26281434	0.00019898	0.26844835
chr1	cg25494767	TSS200	FBLIM1;FBLIM1;FBLIM1;FBLIM1;FBLIM1;	0.32531755	0.00019948	0.26844835
chr8	cg09831796	UTR	BU;SYBU;SYBU;SYBU;SYBU;SYBU	0.26101421	0.00020076	0.26844835
chr7	cg10487891	TSS1500	LRCH4;SAP25	-0.2212228	0.00020099	0.26844835
chr3	cg18031986	TSS1500	OR5H2;RP11-325B23.2	-0.1706989	0.00020115	0.26844835
chr6	cg05586676	Body		-0.5313235	0.00020171	0.26844835
chr11	cg05815958	'UTR;1stExon;1stExon;	MARK2;MARK2;MARK2;MARK2	0.17746459	0.00020228	0.26844835
chr6	cg15971505	Exon;1stExon	C6orf203;C6orf203;C6orf203;C6orf203	0.20682919	0.00020246	0.26844835

chr7	cg17527119			0.21506627	0.00020348	0.26844835
chr12	cg26944011	0;TSS200	YRM5;LYRM5;CASC1;CASC1;CASC1;CAS	0.14626253	0.00020355	0.26844835
chr3	cg09065654	TSS200	VWA5B2;VWA5B2	0.35262105	0.00020367	0.26844835
chr5	cg01799653	TSS1500	ANKRD33B;ANKRD33B	-0.4877384	0.0002039	0.26844835
chr21	cg27312626	TSS1500;TSS1500	TMPRSS3	0.19231031	0.00020392	0.26844835
chr19	cg23291200	3'UTR	APC2;APC2	-0.2164757	0.00020395	0.26844835
chr20	cg25178765	TSS1500	ISM1	0.26321176	0.00020532	0.26844835
chr1	cg00910518			0.34804196	0.00020537	0.26844835
chr21	cg06590956		TMPRSS3	0.17537377	0.00020608	0.26844835
chr12	cg24504927			0.14747626	0.00020615	0.26844835
chr7	cg07922895	Body;Body	OC4	0.14992223	0.00020671	0.26844835
chr16	cg25622387	TSS1500	2288F12.1	0.28360336	0.00020713	0.26844835
chr1	cg07841371	Body;TSS200	AS1;TTLL10;TTLL10-AS1	-0.2214234	0.00020776	0.26844835
chr17	cg05176131	TSS200	ATA20;SPATA20;SPATA20;SPATA20;SPA	0.21334922	0.00020832	0.26844835
chr1	cg08785155	Body;Body	ATP1B1;ATP1B1	-0.2028082	0.00020842	0.26844835
chr10	cg02086858	y;Body;Body;Body	KCNIP2	0.12329764	0.00020846	0.26844835
chr5	ch.5.97862610R			0.43089145	0.00020846	0.26844835
chr17	cg08989921	5'UTR	USP36;USP36	0.15340808	0.00020853	0.26844835
chr6	cg18465030			0.29294672	0.00020876	0.26844835
chr1	cg22083815	5'UTR	CCDC28B	0.18890631	0.00020907	0.26844835
chr14	cg25697181		AL163953.3	0.22810055	0.00020921	0.26844835
chr10	cg05336550	TSS200	M1;PGAM1;PGAM1	0.2351192	0.00020976	0.26844835
chr12	cg12601353	Body;Body		0.1700416	0.00020979	0.26844835
chr4	cg14686113	TSS1500	GSX2;GSX2;GSX2;GSX2	0.16576486	0.00021006	0.26844835
chr18	cg11255230	TSS1500;TSS1500	BCL2;BCL2	0.15328247	0.00021063	0.26844835
chr17	cg06352343	0;TSS200	;TOB1;TOB1	0.12352698	0.00021071	0.26844835
chr3	cg08673716		RP11-95M5.1;RP11-95M5.1	0.17258572	0.00021076	0.26844835
chr3	cg00345443			-0.4493812	0.00021085	0.26844835
chr17	cg10619042			0.26413591	0.00021118	0.26844835
chr15	cg14822850	5'UTR;1stExon	3154N5.1;CLK3;CLK3;CLK3;CLK3;CLK3;C	0.15789603	0.00021243	0.26858161
chr3	cg04446134		Y_RNA	0.21210153	0.0002127	0.26858161
chr8	cg10709532	TSS200;TSS200	VPS28;VPS28;VPS28;VPS28	0.14142966	0.00021318	0.26858161

chr1	cg22695117			-0.2470226	0.0002132	0.26858161
chr13	cg01404873	TSS1500	DLEU2;DLEU1;DLEU1;DLEU1;DLEU1	0.35821787	0.00021358	0.26858161
chr1	cg23702073	Exon;1stExon;1stExon;	7D1;MAP7D1;MAP7D1;MAP7D1;MAP7D1;	0.33905941	0.00021401	0.26858161
chr14	cg16561543	TSS1500;Body;TSS200	BD6;BTBD6;BTBD6;BTBD6;BRF1;BRF1;B	0.27390259	0.00021432	0.26858161
chr11	cg16149508			0.2185965	0.000215	0.26858161
chr3	cg07089698			0.24734202	0.0002153	0.26858161
chr3	cg25852545	TSS200;TSS200	TRTD1;KIAA1407;KIAA1407;QTRTD1;QTR	0.1471156	0.00021539	0.26858161
chr7	cg07464358		RP11-132A1.4	-0.1199803	0.00021575	0.26858161
chr18	cg19104836	1stExon	ZCCHC2	0.12825096	0.00021617	0.26858161
chr11	cg27218829	n	D1;FOXRED1;FOXRED1;FOXRED1;FOXR	0.16491527	0.00021651	0.26858161
chr1	cg16800405	Exon;1stExon;1stExon;1	612B15.3;SH3GLB1;SH3GLB1	0.21697895	0.00021716	0.26858161
chr20	cg10260164	TSS1500		0.1909051	0.00021727	0.26858161
chr3	cg13518792	TSS1500;5'UTR;5'UTR	ARG;PPARG	0.12812688	0.00021776	0.26858161
chr3	cg02592957			0.19409538	0.00021782	0.26858161
chr1	cg18324862	Body;Body;Body;Body	ACOT7;ACOT7	-0.1413309	0.00021795	0.26858161
chr8	cg11898212	0	FBXO25;FBXO25;FBXO25;FBXO25	0.16115779	0.0002192	0.26948205
chr16	cg03586879	TSS1500;TSS1500	420N3.2	-0.1996786	0.00022005	0.26948205
chr11	cg15331284	5'UTR	f75	0.18401893	0.00022057	0.26948205
chr6	cg26005569			0.35440166	0.00022088	0.26948205
chr4	cg15786353	Body	CLNK;CLNK	0.15245384	0.00022115	0.26948205
chr4	cg18827773	5'UTR		0.20099691	0.00022144	0.26948205
chr1	cg12316341		LRRC8C	0.22038613	0.00022161	0.26948205
chr13	cg16932210	Body;Body;Body;Body	ABCC4	0.18712011	0.00022245	0.26948205
chr11	cg17568129	0	;KBTBD3;KBTBD3;KBTBD3;KBTBD3	0.15391302	0.00022294	0.26948205
chr7	cg18971054	5'UTR	MGAM;MGAM;MGAM	0.21288017	0.0002232	0.26948205
chr1	cg20539324	Body	RASAL2	0.20009966	0.00022329	0.26948205
chr1	cg00404394	5'UTR	FGR;FGR;FGR	-0.1248719	0.00022342	0.26948205
chr2	cg25574750			0.15051591	0.0002235	0.26948205
chr1	cg06419258	TSS200	TRNP1;TRNP1	0.16489688	0.00022429	0.26998822
chr3	cg14512813			0.16834342	0.00022598	0.27067809
chr11	cg21842523	Body	LMO1;LMO1	0.21667036	0.00022599	0.27067809
chr22	cg19694818	0;TSS200	OC5;THOC5;THOC5;THOC5	0.21628489	0.00022599	0.27067809

chr5	cg01569428		CTD-2503O16.4	0.25272169	0.00022764	0.27221199
chr13	cg02696428	5'UTR	LCP1;LCP1;LCP1;LCP1;LCP1	0.18389548	0.00022826	0.27250416
chr19	cg10589950	Body		0.15973816	0.00022911	0.27262478
chr6	cg03611990	Body		0.16846644	0.00022919	0.27262478
chr10	cg02818775	Body	SH3PXD2A	-0.3032589	0.0002299	0.27262478
chr14	cg01318702	1500;1stExon	KHNYN;KHNYN;KHNYN;KHNYN;CBLN3	-0.1490229	0.00023009	0.27262478
chr3	cg05855917	Body;Body;Body	ZIC4;ZIC4;ZIC1	0.25921249	0.00023041	0.27262478
chr1	cg13064658	5'UTR;1stExon	552D8.1;RP11-	0.39286406	0.00023083	0.27262478
chr7	cg09418592			0.25069192	0.00023111	0.27262478
chr7	cg12963736		CAMK2B	0.20796118	0.00023223	0.27262478
chr10	cg21960123			0.21102215	0.00023287	0.27262478
chr19	cg19244855	UTR	TLE2;TLE2;TLE2;TLE2;TLE2	0.36133889	0.00023331	0.27262478
chr18	cg16071681			0.21528281	0.00023396	0.27262478
chr3	cg08483723	TSS200	TMEM158	0.29092895	0.00023402	0.27262478
chr13	cg23387183	Body;TSS1500	NEK5	0.17518052	0.00023558	0.27262478
chr9	cg21233189		RNF38	0.17577657	0.00023721	0.27262478
chr10	cg07529353		RP11-69C17.3	0.19220733	0.00023742	0.27262478
chr11	cg12820122	TSS200	111M22.3;C11orf30;C11orf30;C11orf30;C11o	0.28350314	0.000238	0.27262478
chr14	cg10318510	TSS200	PRKD1;PRKD1	0.31187508	0.00023831	0.27262478
chr7	cg10206873	5'UTR	ELFN1;ELFN1;ELFN1	-0.2600211	0.00023846	0.27262478
chr5	cg19314159	5'UTR	SH3RF2;SH3RF2;SH3RF2	0.22961424	0.00023901	0.27262478
chr21	cg12767180		PCBP3;PCBP3	0.22372879	0.00023979	0.27262478
chr2	cg09867869		AC079779.4	0.21353495	0.0002399	0.27262478
chr16	cg16989969			0.15708528	0.00024014	0.27262478
chr2	cg07262519	Body	SLC4A10;SLC4A10;SLC4A10	0.22595737	0.00024034	0.27262478
chr2	cg09726569		snoU13	0.20380376	0.00024036	0.27262478
chr11	cg20338703	1stExon;1stExon	MLL;MLL;MLL;MLL;MLL;MLL;MLL	0.13696984	0.00024049	0.27262478
chr7	cg10864883			0.22430505	0.00024072	0.27262478
chr12	cg23655651			0.29712642	0.00024091	0.27262478
chr1	cg12793305	TSS200	864K19.4;RRAGC;RRAGC	0.20086442	0.00024136	0.27262478
chr5	cg04493143	y;Body;3'UTR	HTR4;HTR4;HTR4	-0.4670824	0.00024145	0.27262478
chr1	cg17864206	0	MED8;MED8;SZT2	0.18547759	0.0002418	0.27262478

chr8	cg14436888	TSS200;TSS200	ZHX1;ZHX1;ZHX1;ZHX1;ZHX1;ZHX1	0.12583816	0.00024327	0.27262478
chr19	cg16992787	Body;Body		-0.1241662	0.00024346	0.27262478
chr11	cg08358813	0	CTD-2523D13.2;PVRL1;PVRL1;PVRL1	0.21003052	0.00024358	0.27262478
chr17	cg22068467	5'UTR;5'UTR	SLC16A5;SLC16A5	-0.1815418	0.00024367	0.27262478
chr9	cg13828227	y;Body;Body	C;SH2D3C	-0.3237791	0.00024369	0.27262478
chr1	cg17452713	R;TSS200	213;ZNF436;ZNF436;ZNF436	0.22099841	0.00024379	0.27262478
chr19	cg02481918	Body		-0.1828819	0.0002439	0.27262478
chr14	cg23357503	200	6;STXBP6	0.19794537	0.0002439	0.27262478
chr2	cg09905191		AC108868.5	0.20379182	0.00024415	0.27262478
chr14	cg14288330	TSS200	INSM2	0.15667342	0.00024418	0.27262478
chr18	cg06225979	TSS200	ZNF396	0.23448736	0.00024487	0.27262478
chr11	cg26276611	Body	DEAF1;RP11-754B17.1;DEAF1;DEAF1	-0.1600331	0.00024499	0.27262478
chr16	cg10226628	TSS200	SETD1A;SETD1A	0.15078819	0.00024538	0.27262478
chr4	cg01182661	ody;Body;5'UTR	GPM6A;GPM6A;GPM6A;GPM6A;GPM6A;	0.41637562	0.00024587	0.27262478
chr5	cg25714323			0.24809538	0.000246	0.27262478
chr4	cg11928122	Body	RP11-10K16.1;RP11-10K16.1;RP11-10K16.1	0.15840398	0.00024625	0.27262478
chr10	cg01109508	TSS200	ECHS1;ECHS1	0.12802964	0.00024686	0.27262478
chr10	cg18676500	TSS1500	EBF3;EBF3	0.26931523	0.00024711	0.27262478
chr20	cg16871527	TSS200	RALGAPA2;RALGAPA2;RALGAPA2	0.18249307	0.00024771	0.27262478
chr8	cg01794245	TSS1500	ARHGAP39	0.22574077	0.00024809	0.27262478
chr20	cg00057603			0.20893139	0.00024907	0.27262478
chr19	cg26572233	Body		-0.1728613	0.00024961	0.27262478
chr20	cg22808989	stExon;Body	G1;RP1-1J6.2	0.34435865	0.00025027	0.27262478
chr2	cg19721797	Body	snoU13	0.1873677	0.00025028	0.27262478
chr1	cg14446588	TSS1500	IQGAP3;IQGAP3	-0.1059879	0.0002504	0.27262478
chr1	cg12819298	Body		-0.1088367	0.00025049	0.27262478
chr14	cg26010412	Body;Body		0.20832144	0.00025104	0.27262478
chr7	cg10276302	TSS200	WDR91;WDR91;WDR91	0.29753118	0.00025149	0.27262478
chr8	cg23051059	Body		0.21869039	0.00025247	0.27262478
chr7	cg00973426	0;TSS200	PTPRN2;PTPRN2;PTPRN2;PTPRN2	0.13792976	0.00025257	0.27262478
chr11	cg04012924	5'UTR	FAR1-IT1;FAR1;FAR1;FAR1	0.13459277	0.00025269	0.27262478
chr4	cg04574592	1stExon;5'UTR	NAJB14;DNAJB14;DNAJB14;DNAJB14	0.3458975	0.00025302	0.27262478

chr1	cg11851382	Body;Body	PPAP2B	0.36002012	0.0002531	0.27262478
chr13	cg20894110	Body	SACS	0.25153862	0.00025341	0.27262478
chr9	cg24178514	Body		0.26742932	0.00025389	0.27262478
chr16	cg19319725		RP11-510J16.5	0.49175187	0.00025485	0.27262478
chr22	cg23055716		RP11-375H17.1;RP1-213J1P__B.1	0.27496006	0.00025494	0.27262478
chr10	cg18903093	TSS1500	C10orf107;C10orf107	0.21755346	0.00025504	0.27262478
chr1	cg23804659	Body;Body	SYT14;SYT14	0.20837587	0.00025505	0.27262478
chr19	cg03828221	Body;Body	ZNF497	0.3581005	0.00025568	0.27262478
chr10	cg13554667	TSS200;1stExon;5'UTR;	K1;CDK1	0.21828453	0.00025589	0.27262478
chr5	cg08099655	;5'UTR;5'UTR	ERAP1;ERAP1;ERAP1;ERAP1;ERAP1	0.16541232	0.0002561	0.27262478
chr11	cg20070597	n;5'UTR;5'UTR;5'UTR;	M;PICALM;PICALM;PICALM;PICALM;PIC	0.19432772	0.00025622	0.27262478
chr8	cg13508543		RP11-383H13.1	0.16628412	0.00025626	0.27262478
chr1	cg08326593	Body;Body;Body;Body	TMCC2;TMCC2;TMCC2	0.21442945	0.00025688	0.27262478
chr8	cg12341757	5'UTR;5'UTR	T3GAL1;ST3GAL1;ST3GAL1;ST3GAL1	0.20427095	0.00025699	0.27262478
chr10	cg06340043	ExonBnd;5'UTR	108L7.7;LZTS2;LZTS2;LZTS2;LZTS2;LZTS	-0.1298308	0.00025728	0.27262478
chr18	cg00464274	Body;5'UTR	TTC39C	0.25962981	0.00025855	0.27268992
chr1	cg10104290		RP3-455J7.4;RP3-455J7.4	0.23011456	0.00025873	0.27268992
chr1	cg17020695	5'UTR;5'UTR;1stExon	31F15.1;RP11-	0.20235647	0.00025924	0.27268992
chr1	cg15084499	TSS200;TSS200	BPA;CDC42BPA;CDC42BPA	0.29014072	0.00025965	0.27268992
chr1	cg24492862	TR;5'UTR	AS1;LGALS8;LGALS8;LGALS8;LGALS8;L	0.16814745	0.00025977	0.27268992
chr2	cg07422110			0.23673849	0.00026019	0.27268992
chr13	cg25012919	TSS200	DCLK1;DCLK1	0.30740883	0.00026027	0.27268992
chr15	cg16731620		RNU5A-1;PARP16	1.20987462	0.00026045	0.27268992
chr14	cg22822094	Body;Body;Body	RAD51B	0.19784168	0.00026073	0.27268992
chr19	cg04273622	Body;Body	AP3D1	-0.193837	0.00026124	0.27283116
chr9	cg03298938	TSS1500	COL5A1	0.17426401	0.00026169	0.27290676
chr16	cg14673706			0.23341162	0.00026294	0.2734092
chr2	cg18847112	5'UTR;5'UTR	Z2B;BAZ2B	0.15224291	0.0002632	0.2734092
chr9	cg14455366	TSS1500	IB	0.15459097	0.00026344	0.2734092
chr13	cg00669363	y	FNDC3A;FNDC3A;FNDC3A	0.43150173	0.00026427	0.2734092
chr2	cg19102858	TSS200;TSS200	PS8;COPS8;COPS8;COPS8	0.1631404	0.00026463	0.2734092
chr12	cg07739483	y	CABP1;CABP1	0.17688904	0.00026561	0.2734092

chr2	cg03506768			0.17861914	0.00026597	0.2734092
chr11	cg09646051			0.17939268	0.00026598	0.2734092
chr18	cg10733251	Body;Body;Body	DSG1	0.17289556	0.00026603	0.2734092
chr1	cg10062141			0.4022171	0.0002665	0.2734092
chr19	cg12336033	Body		-0.1903389	0.00026866	0.2734092
chr3	cg20807374	Body;Body		-0.3403876	0.00026879	0.2734092
chr7	cg18002011	5'UTR;5'UTR	TFEC;TFEC;TFEC	0.19306902	0.00026888	0.2734092
chr6	cg25005633	n;5'UTR;5'UTR	RS2;VARS2;VARS2;VARS2;VARS2;VARS2	0.16643663	0.00026895	0.2734092
chr8	cg17981470	Body		-0.2299553	0.00026908	0.2734092
chr2	cg06771950	Body;Body;Body	ARMC9;ARMC9;ARMC9	0.2274812	0.00026924	0.2734092
chr14	cg02719245	Body;TSS1500	AS1;DICER1;DICER1;DICER1;DICER1-	-0.5102566	0.00026925	0.2734092
chr1	cg17979628	TSS200;TSS200	NENF;NENF;NENF;NENF	-0.3331892	0.00026941	0.2734092
chr12	cg08041019	5'UTR;1stExon	11-686G8.2;ATXN2;ATXN2;ATXN2	0.21982093	0.00026943	0.2734092
chr8	cg12028883	TR;TSS1500;TSS1500	2A2;FBXL6;FBXL6;FBXL6;SLC52A2;SLC5	0.15001429	0.0002697	0.2734092
chr19	cg15123730			0.33500909	0.00027064	0.27364091
chr14	cg09718076		1152H15.1	0.23865701	0.00027176	0.27364091
chr5	cg06923210	Body;5'UTR	FBXL7	0.20000693	0.00027202	0.27364091
chr2	cg10423342		AC092484.1	0.22228129	0.00027218	0.27364091
chr3	cg02255184	1stExon	MANF;MANF;MANF	0.20023543	0.00027262	0.27364091
chr7	cg18416576	TSS200;Body	170O19.21;HOXA10	0.42477575	0.00027267	0.27364091
chr1	cg25989970	5'UTR;1stExon	UFC1;UFC1;UFC1;UFC1;UFC1;UFC1	0.20075423	0.00027289	0.27364091
chr11	cg07635249	Body		0.21459344	0.0002735	0.27364091
chr5	cg14435583	Body;Body	MCC;MCC;MCC	0.1853012	0.00027394	0.27364091
chr19	cg21068576	Body;5'UTR	TRAPPC2P1;ZNF547	0.25547634	0.0002744	0.27364091
chr20	cg02162556			-0.2552064	0.00027446	0.27364091
chr8	cg27541630			0.24490877	0.00027505	0.27364091
chr3	cg08453281	TSS200	TMEM115;TMEM115	0.25624794	0.00027519	0.27364091
chr1	cg11205824	0	EM48;TMEM48	0.33013127	0.00027521	0.27364091
chr15	cg03532957	1stExon;5'UTR	CHSY1;CHSY1	0.15592749	0.00027567	0.27372973
chr3	cg09703620	Body		0.20001808	0.00027651	0.27418458
chr7	cg11671946	TSS1500	KIAA1147;KIAA1147	-0.2280592	0.00027759	0.27477423
chr2	cg02788938			0.41782435	0.00027846	0.27477423

chr2	cg25503044	Body;TSS200	RP11-523H20.2;LBX2;LBX2;LBX2;LBX2	0.19761277	0.00027912	0.27477423
chr12	cg05613195		RP11-397H6.1;RP11-541G9.1	0.21575325	0.00027922	0.27477423
chr13	cg08215918	1500;TSS1500	CCNA1;CCNA1;CCNA1;CCNA1	0.29985381	0.00027991	0.27477423
chr2	cg01757209	Body;Body	BMS1;RBMS1	0.17699481	0.00028008	0.27477423
chr10	cg09960003	TSS1500;TSS1500	CRTAC1	-0.1370875	0.0002805	0.27477423
chr16	cg07709980		AJ003147.9	-0.1676729	0.00028069	0.27477423
chr15	cg05880817	n;TSS1500	D1;DTWD1;DTWD1;DTWD1;DTWD1;DTW	0.19197731	0.00028087	0.27477423
chr2	cg23466916	Body;1stExon;5'UTR	2A;OBFC2A;OBFC2A;OBFC2A;OBFC2A;O	0.14727668	0.00028157	0.27477423
chr3	cg14808132	;5'UTR	;ATP2B2;ATP2B2;ATP2B2;ATP2B2	0.16899777	0.0002816	0.27477423
chr6	cg01321872	5'UTR;5'UTR;Body	RS2;VARS2;VARS2	0.17050361	0.00028165	0.27477423
chr18	cg14729980	Body	SIGLEC15	0.1666963	0.00028242	0.2750532
chr12	cg05191825	0;5'UTR;1stExon	T3;ISCU;ISCU;ISCU;ISCU;ISCU	0.23066631	0.00028269	0.2750532
chr12	cg20090957	0	5;C12orf47;C12orf47;MAPKAPK5;MAPKAP	0.25185303	0.00028423	0.27577531
chr4	cg23780797		AC004069.2	0.2029589	0.00028459	0.27577531
chr13	cg09899665	TSS200	29G8.3;UCHL3;COMMD6;COMMD6;UCHL	0.33961854	0.00028497	0.27577531
chr3	cg04885336	TSS1500	PPP1R2;PPP1R2;PPP1R2;AC091633.3	-0.1021662	0.00028546	0.27577531
chr5	cg25888694	Exon;1stExon;1stExon	394O4.4;PCYOX1L;PCYOX1L;PCYOX1L	0.29102704	0.00028555	0.27577531
chr16	cg08392591	5'UTR	1;ANKRD11;ANKRD11;ANKRD11	0.18879773	0.00028571	0.27577531
chr11	cg16977596	Body;Body;Body;Body	DGKZ;DGKZ;DGKZ;DGKZ	-0.1742168	0.00028732	0.27682587
chr5	cg22792660		RP11-229C3.2;RP11-229C3.2	0.3781778	0.00028756	0.27682587
chr5	cg20656637	1stExon;1stExon	FST;FST	0.13518568	0.00028813	0.2770002
chr12	cg09349614			0.26195822	0.00028971	0.27815926
chr19	cg19375492			0.20199127	0.00029156	0.278893
chr2	cg24935556			0.20301604	0.00029162	0.278893
chr7	cg11018271	Body	GNA12;GNA12	-0.18106	0.00029163	0.278893
chr14	cg20188816	Body	MIS18BP1	0.18644386	0.00029235	0.27921006
chr3	cg25789721	3'UTR;3'UTR	ATP2B2;ATP2B2;ATP2B2;ATP2B2	-0.2330931	0.00029373	0.2793332
chr18	cg00861839	Body;Body	SS18;SS18;SS18;SS18	0.21454708	0.00029377	0.2793332
chr7	cg05372578	'UTR	CA1;ICA1;ICA1;ICA1;ICA1;ICA1;ICA1;ICA	0.13128321	0.00029401	0.2793332
chr7	cg25411333	TSS1500	NCAPG2;NCAPG2;NCAPG2;NCAPG2	0.31545165	0.00029451	0.2793332
chr8	cg00378306			0.24722763	0.00029504	0.2793332
chr1	cg22937163			0.23886821	0.00029532	0.2793332

chr3	cg00803692	TSS200;TSS200	CCR5;CCR5;RP11-24F11.2	0.18120544	0.00029559	0.2793332
chr2	cg19512473			0.17284121	0.00029585	0.2793332
chr2	cg26373379	TSS200	SEMA4C;SEMA4C	0.20825419	0.00029594	0.2793332
chr9	cg08754068	;Body;Body;Body	203J24.9;ST6GALNAC6;RP11-203J24.9	-0.1947823	0.00029641	0.27941691
chr7	cg10479490	y	ELMO1-AS1;ELMO1;ELMO1	-0.2185802	0.00029692	0.27950655
chr8	cg01499546	UTR;5'UTR;5'UTR;5'U	U2;STAU2;STAU2;STAU2;STAU2;STAU2;	0.20982967	0.00029728	0.27950655
chr1	cg15842317	3'UTR;Body	VSIG8;C1orf204;C1orf204	0.24806593	0.00029768	0.27951945
chr14	cg05902783	Body	EVL;EVL	0.16924716	0.00029891	0.28031507
chr7	cg11464238	TSS200;TSS200	MKRN1;MKRN1;MKRN1;MKRN1;MKRN1;	0.14572248	0.00029965	0.28051876
chr12	cg13880786	00	;C12orf52;C12orf52	0.14430002	0.00030018	0.28051876
chr9	cg23075568	TSS1500	CORO2A	0.2072477	0.00030029	0.28051876
chr21	cg12044973	TSS1500	KRTAP11-1	0.19244583	0.00030139	0.28095711
chr8	cg02700454	TSS1500	TRIB1	0.16217535	0.00030209	0.28095711
chr5	cg22554488	Body	ODZ2	0.1763153	0.00030223	0.28095711
chr2	cg11556696	Body	ALS2;ALS2;ALS2	-0.1175427	0.00030269	0.28095711
chr18	cg06509213	5'UTR		0.20083988	0.00030302	0.28095711
chr7	cg22614757	TSS1500	ZNF853	0.12927381	0.00030308	0.28095711
chr6	cg24246360	5'UTR	IBTK;IBTK;IBTK	0.18169669	0.00030479	0.28218187
chr2	cg01137798			0.14014717	0.00030553	0.28250826
chr2	cg08266945	TSS200	2;PHOSPHO2;KLHL23;KLHL23;C2orf77;C2	0.18578851	0.0003061	0.28267592
chr12	cg16277306		C41A2	0.35724228	0.0003084	0.28298752
chr22	cg00493910	5'UTR	ED1;MPPED1;MPPED1;MPPED1;MPPED1;	0.22763454	0.00030849	0.28298752
chr20	cg07177408		TSPY26P	-0.1861115	0.00030871	0.28298752
chr16	cg07670266	Body;Body	SLC9A3R2	-0.2333215	0.00030892	0.28298752
chr4	cg05284727	5'UTR	WHSC1;WHSC1;WHSC1;WHSC1	-0.4670555	0.000309	0.28298752
chr3	cg05481881		166N6.3;RP11-166N6.2	0.20261193	0.00030995	0.28298752
chr4	cg22968767	Body	SORCS2	0.24787747	0.00031017	0.28298752
chr19	cg07636338	3'UTR	FBXO46;FBXO46	0.20171514	0.00031031	0.28298752
chr10	cg22792014			-0.3660407	0.00031051	0.28298752
chr3	cg11041457	00;TSS1500	BRM1;PBRM1;GNL3;GNL3;GNL3;GNL3;G	0.36589761	0.00031069	0.28298752
chr15	cg19613905	1stExon;5'UTR	MNS1;MNS1;MNS1;MNS1	0.28577259	0.00031073	0.28298752
chr22	cg09192333	TSS200;Body;TSS200	TRIOBP	0.29783692	0.00031167	0.28326156

chr21	cg26921254	TSS1500	KRTAP21-1	0.30491842	0.00031184	0.28326156
chr1	cg13671816	3'UTR	RP11-134G8.8	0.20062956	0.000313	0.28326156
chr6	cg22328955		HCG14	0.17702491	0.00031355	0.28326156
chr1	cg16718920	TSS1500	HP1BP3;HP1BP3;HP1BP3;HP1BP3	0.14272994	0.00031387	0.28326156
chr12	cg22132332	UTR;1stExon	A1A;TUBA1A;TUBA1C;TUBA1A;TUBA1A	0.19863381	0.0003142	0.28326156
chr13	cg18992706	0	2	0.32766372	0.00031455	0.28326156
chr6	cg04500437			-0.1575599	0.00031613	0.28326156
chr5	cg01142438	Body		0.15780075	0.00031634	0.28326156
chr11	cg03669907	0;TSS200;5'UTR;5'UTR	AS;WT1;WT1-AS;WT1-AS;WT1;WT1	0.29388061	0.00031689	0.28326156
chr22	cg19299940	TSS1500;Body	SBP11;GUSBP11;GUSBP11;GUSBP11;GUS	0.20630873	0.00031715	0.28326156
chr6	cg13823495			0.18941776	0.0003174	0.28326156
chr13	cg20807920	TR	TEX30;TEX30;TEX30;TEX30;TEX30	0.20979362	0.00031766	0.28326156
chr8	cg04232586	Body	C8orf38;C8orf38;C8orf38;C8orf38;C8orf38	0.24043822	0.00031774	0.28326156
chr2	cg19763809	TSS200	;VWA3B;VWA3B	0.28902215	0.00031851	0.28326156
chr1	cg16685388	5'UTR;5'UTR	HIVEP3;HIVEP3;HIVEP3;HIVEP3	-0.4042011	0.00031915	0.28326156
chr2	cg23700226	1500;1stExon;1stExon;1	8737.1;AC018737.1;CLASP1;CLASP1;CLAS	0.11668432	0.00031922	0.28326156
chr3	cg01449754		MIR720	0.22356573	0.00031967	0.28326156
chr2	cg16236960	5'UTR;1stExon	MGAT5	0.16007747	0.00032002	0.28326156
chr3	cg18083704	TSS200	;TTLL3;TTLL3;TTLL3;TTLL3;TTLL3;TTLL	0.16095018	0.00032012	0.28326156
chr1	cg19375336			0.1516703	0.00032117	0.28326156
chr8	cg27344576	Body	EIF3E;EIF3E;EIF3E;EIF3E	0.23068848	0.00032123	0.28326156
chr16	cg03283048	0	H2	0.14794616	0.00032149	0.28326156
chr11	cg14892886	TSS1500;5'UTR;5'UTR	35C1;SLC35C1	0.16140127	0.00032158	0.28326156
chr20	cg00505393	TSS1500	E2F1	0.15445	0.00032166	0.28326156
chr12	cg11988396		RP11-283G6.3;RP11-283G6.3;SSPN;SSPN	0.19459305	0.00032189	0.28326156
chr5	cg09225720			0.15248519	0.00032245	0.28326156
chr16	cg01366354			-0.2679412	0.00032318	0.28326156
chr3	cg27564016	n;TSS200;5'UTR;TSS20	2;IFT122;IFT122;MBD4;MBD4;MBD4;MBD	0.30906482	0.00032405	0.28326156
chr16	cg06524479	TSS200;Body	CA7;NAE1;RP11-61A14.1	0.27585218	0.00032422	0.28326156
chr1	cg04937080	5'UTR	DAB1;DAB1;DAB1	0.23817559	0.00032491	0.28326156
chr7	cg08177063	TSS200	WASL;WASL;WASL	0.23311537	0.00032507	0.28326156
chr19	cg18054632	Body		-0.2771706	0.00032517	0.28326156

chr12	cg02804341	TSS200	F1;TWF1;TWF1;TWF1	0.16346988	0.00032537	0.28326156
chr7	cg21996614			0.16560394	0.00032679	0.28326156
chr10	ch.10.2609808I	Body;Body;Body;Body;	FGFR2	0.17214141	0.00032696	0.28326156
chr21	cg09613494	5'UTR	SIK1	0.13589812	0.00032721	0.28326156
chr8	cg17852081	1500	2320G14.2;GDAP1;GDAP1;GDAP1;GDAP1;	0.26343745	0.00032725	0.28326156
chr8	cg14084648		3046C4.1	0.17581522	0.00032736	0.28326156
chr21	cg08538258	dy	X322557.10;POFUT2;POFUT2	0.36636041	0.00032765	0.28326156
chr1	cg02057063	1stExon	1073O3.2;RSBN1;RSBN1	0.25655555	0.00032767	0.28326156
chr22	cg13422535			-0.1693857	0.00032793	0.28326156
chr2	cg23667267	;5'UTR;1stExon;1stExo	BIN1;BIN1;BIN1;BIN1;BIN1;BIN1;BIN1	0.3077407	0.00032795	0.28326156
chr22	cg18485569	0	PANX2;PANX2	0.27434254	0.0003282	0.28326156
chr7	cg24934063	1stExon;1stExon	560I19.4;FEZF1;RP11-560I19.4	0.32471068	0.00032903	0.28363715
chr7	cg07617636	Body	PRKAG2	-0.1658153	0.00033004	0.28403947
chr1	cg03808263			0.15921292	0.00033028	0.28403947
chr5	cg17674090	TSS1500;TSS200	ITGA1;PELO;ITGA1;PELO;CTD-2288O8.1	-0.1436979	0.00033202	0.28520434
chr14	cg04297581	Body;Body;Body	AKT1;AKT1;AKT1;AKT1;AKT1	-0.1725114	0.00033247	0.28524676
chr14	cg01533082	Body		0.19321711	0.00033423	0.28599083
chr6	cg11247116	1stExon;5'UTR	VARS;VARS;VARS;VARS;VARS	0.20813389	0.00033509	0.28599083
chr13	cg03738328			-0.2277328	0.00033541	0.28599083
chr2	cg02313965	TSS1500;5'UTR	D1;CENPO;PTRHD1;CENPO;CENPO;CENP	0.18886204	0.00033579	0.28599083
chr7	cg27120649	00;5'UTR;TSS1500	E;PEG10;PEG10;PEG10	0.13779924	0.00033579	0.28599083
chr17	cg19931178	TSS200;TSS200	F2;ATPAF2;ATPAF2;ATPAF2;ATPAF2	0.30059768	0.00033624	0.28599083
chr4	cg16933229		PITX2;PITX2;PITX2	0.26305366	0.00033638	0.28599083
chr6	cg11854797			0.15188255	0.00033696	0.28599083
chr11	cg21915672	stExon	BIRC3	0.20814426	0.00033697	0.28599083
chr16	cg27344162	Body;Body		0.30355928	0.00033727	0.28599083
chr18	cg19186966	Body		0.19670468	0.00033831	0.28631444
chr12	cg07226883	Body	PPP1R12A;PPP1R12A;PPP1R12A	0.20815218	0.00033865	0.28631444
chr8	cg19688250	n;1stExon;5'UTR;5'UTR	ALYL;RALYL;RALYL	0.41671852	0.00033884	0.28631444
chr5	cg02656525	TSS200	551A13.2;WDR36;WDR36;WDR36	0.16967088	0.00034018	0.28654768
chr1	cg25791996	Body	SPAG17;SPAG17	0.12905841	0.00034075	0.28654768
chr17	cg04533189	TSS200	PRKCA	0.21596239	0.00034121	0.28654768

chr3	cg21262651	Body	MRPS22;BPESC1;MRPS22	0.22099204	0.00034126	0.28654768
chr7	cg12510614	Body;Body;Body		-0.3851954	0.00034127	0.28654768
chr16	cg16471167	Body;Body	BANP;BANP	-0.1650784	0.00034183	0.28654768
chr10	cg09169356			0.2289764	0.00034188	0.28654768
chr10	cg26737090			0.15869084	0.00034333	0.28703272
chr15	cg07709485	Body	PIAS1	0.17176401	0.00034341	0.28703272
chr6	cg12280276			0.21694039	0.00034407	0.28703272
chr5	cg14546471	TSS200;TSS200	MAST4;MAST4;MAST4;MAST4	0.16596908	0.00034442	0.28703272
chr1	cg14157073	Body	CAMTA1	0.33022521	0.00034451	0.28703272
chr6	cg19931770			0.21608231	0.00034483	0.28703272
chr4	cg07775007			0.1599161	0.00034582	0.28752666
chr22	cg21862100			-0.1380999	0.00034663	0.2878742
chr20	cg02165799	1stExon;5'UTR;Body	NKAIN4;NKAIN4;NKAIN4;NKAIN4	0.19807405	0.00034706	0.28790206
chr2	cg00824141	stExon;5'UTR;5'UTR;1s	FHL2	0.14908441	0.00034804	0.28837335
chr4	cg04757345	Body	C4orf29;C4orf29	0.21830967	0.00034921	0.28837335
chr2	cg10679103	Body	RN5S93	0.18986198	0.00035003	0.28837335
chr21	cg02533120	TSS200	SOD1;SOD1;SOD1;AP000253.1;SOD1;SOD1	0.13313441	0.00035044	0.28837335
chr3	cg07388899		TPRG1;TPRG1	0.14768254	0.00035045	0.28837335
chr17	cg23117191	1stExon;1stExon	MMP28	0.15915144	0.0003506	0.28837335
chr12	cg13236378	TSS200	WSCD2;WSCD2;WSCD2;WSCD2	0.1854209	0.00035089	0.28837335
chr2	cg19855215	TSS200	CRYGB	0.16393492	0.00035157	0.28837335
chr20	cg27634549	1500	SEC23B;SEC23B;SEC23B;SEC23B;SEC23B	0.14593305	0.00035162	0.28837335
chr4	cg07574458	1stExon	DPR	0.3942172	0.00035203	0.28837335
chr7	cg03119639	0;5'UTR	5;GPR85	0.15071505	0.00035206	0.28837335
chr2	cg07987287			0.18225889	0.00035241	0.28837335
chr4	cg26993729	TSS200	LRAT;LRAT;LRAT;LRAT;LRAT;LRAT	0.18270211	0.00035308	0.28837335
chr19	cg05192108	200;TSS200;TSS1500;T	OAT7;MBOAT7;TSEN34;MBOAT7;MBOAT	0.16512648	0.00035326	0.28837335
chr3	cg08980237		RP11-392A22.2;RP11-392A22.2	0.17749454	0.00035366	0.28837335
chr1	cg06377929	TSS200	ATAD3B;ATAD3B	0.33707281	0.00035399	0.28837335
chr17	cg06380449	Body;Body	GGA3;GGA3	0.35621429	0.00035443	0.28837545
chr6	cg14546778	Body		0.23385633	0.00035515	0.28837545
chr5	cg07548282	TSS200	UBTD2;UBTD2	0.21262027	0.00035518	0.28837545

chr17	cg01066212	Body;Body		0.12470445	0.00035655	0.28896229
chr19	cg15555741	Body;Body	MYO9B	-0.2010057	0.00035724	0.28896229
chr10	cg06706483	Body;Body;Body;Body	RHOBTB1	0.20069187	0.00035904	0.28896229
chr9	cg06622857	1stExon	IZUMO3	0.22384868	0.0003592	0.28896229
chr18	cg16317961	Body;Body;5'UTR;Body	MAPRE2	-0.2048043	0.00035927	0.28896229
chr4	cg07419795			0.20722999	0.00035986	0.28896229
chr3	cg17583264	Body;Body;Body		-0.1748884	0.00036022	0.28896229
chr8	cg25838936	y;Body	PRKDC;MCM4;PRKDC;PRKDC;PRKDC	0.20019212	0.00036099	0.28896229
chr22	cg19198924	Body;5'UTR;5'UTR	PIWIL3;PIWIL3;PIWIL3;PIWIL3;PIWIL3	-0.2510258	0.00036145	0.28896229
chr11	cg23395315	Body	snoU13;OPCML	0.16463333	0.00036177	0.28896229
chr2	cg19746754			0.20093148	0.00036184	0.28896229
chr5	cg04686412	5'UTR	PSD2	0.30628206	0.0003619	0.28896229
chr12	cg07728036	TSS1500	TMPRSS12;TMPRSS12	0.17942335	0.00036205	0.28896229
chr13	cg06125080			0.19438951	0.00036213	0.28896229
chr13	cg12498181	Body;Body	ATP11A	-0.176051	0.00036238	0.28896229
chr1	cg20014094	Body	RP11-288I21.1	-0.1493558	0.00036242	0.28896229
chr12	cg09329151	1500;TSS1500;TSS1500	2;YAF2;YAF2;YAF2;YAF2;YAF2	0.29423614	0.00036312	0.28896229
chr12	cg26659143	3'UTR;3'UTR	BCL7A;AC156455.1;AC156455.1	0.19768666	0.00036344	0.28896229
chr2	cg00494761	UTR;1stExon;5'UTR;1st	1;REEP1;REEP1;REEP1;REEP1;REEP1	0.30179882	0.00036378	0.28896229
chr7	cg02657930	5'UTR;TSS200	33;MRPS33;MRPS33;MRPS33;MRPS33	0.1515668	0.00036387	0.28896229
chr20	cg04928513	Body;TSS1500	PSMA7;PSMA7;SS18L1;SS18L1;PSMA7	0.16025105	0.00036502	0.28955784
chr19	cg27247554	TSS200	IZUMO2;IZUMO2;IZUMO2	-0.2042163	0.00036579	0.28985596
chr6	cg23139609			0.22764668	0.00036652	0.28985597
chr3	cg16831612	Body;Body	ADCY5;ADCY5;ADCY5	0.16444578	0.00036659	0.28985597
chr17	cg23516660	TSS200	GALK1;GALK1;GALK1	0.12844353	0.00036808	0.29057642
chr1	cg22736787	TSS200	PTGFRN;PTGFRN	0.15585593	0.00036864	0.29057642
chr11	cg26574964	TSS200	DT2;SIDT2;SIDT2;SIDT2;SIDT2;SIDT2	0.24765599	0.00036887	0.29057642
chr5	cg17871403	3'UTR	ADCY2;ADCY2;ADCY2;ADCY2;ADCY2	0.16140026	0.00036916	0.29057642
chr20	cg14261863	TSS1500;TSS1500	LMO2;ELMO2;ELMO2;ELMO2;ELMO2	0.22308661	0.00036965	0.29057642
chr9	cg01487461	5'UTR;5'UTR	A2;SMARCA2	0.16722456	0.0003699	0.29057642
chr11	cg12911902		RCN1	0.16720828	0.00037104	0.29115355
chr6	cg18495531	Body	GPR110;GPR110	0.1685198	0.00037269	0.29213653

chr19	cg19659125	1stExon;1stExon	CRTC1	0.4120526	0.0003731	0.29214207
chr3	cg20017382	Body;Body;Body	MAGI1	0.25813452	0.00037409	0.29259763
chr14	cg00295574			0.14684323	0.00037489	0.29290507
chr4	cg02464357	TSS200;TSS200	HERC3;PIGY;HERC3	0.16651918	0.00037545	0.29303457
chr10	cg08997713	Body	298E9.3	-0.2149499	0.00037685	0.2935557
chr12	cg11358257	5'UTR	L1;RASAL1;RASAL1;RASAL1;RASAL1;RA	0.18566809	0.00037693	0.2935557
chr10	cg09944605	ExonBnd;Body;Body		0.29118872	0.00037762	0.29377483
chr2	cg09470142		C2orf88;C2orf88;C2orf88;C2orf88	-0.3268767	0.00037881	0.2941608
chr19	cg09231514	UTR;TSS1500;5'UTR;5'	;HSD11B1L;HSD11B1L	0.24052008	0.00037892	0.2941608
chr15	cg16522107	TSS200	16	0.23995091	0.0003802	0.29483356
chr7	cg03148428	TSS200;1stExon;5'UTR	1.2;RNF32;AC073871.2;C7orf13;RNF32;RNF	0.39233545	0.00038181	0.2951551
chr9	cg26087052	Body;Body	COL5A1	0.20723866	0.00038279	0.2951551
chr6	cg07909292	5'UTR;1stExon	KIAA1919;RP11-428F8.2	0.13063756	0.00038313	0.2951551
chr4	cg02833127	1stExon;5'UTR	SPATA4;SPATA4;SPATA4;SPATA4	-1.3191413	0.00038372	0.2951551
chr11	cg21023284	00;TSS200	K;RRP8;RRP8;ILK;RRP8;RP11-732A19.8	0.11030715	0.00038442	0.2951551
chr6	cg10346782	TSS200	C6orf97;C6orf97	0.29383117	0.00038486	0.2951551
chr10	cg20020488	y;Body	C10orf113	0.22073042	0.00038494	0.2951551
chr2	cg17290000		AC096559.1	0.31886943	0.00038505	0.2951551
chr10	cg26681603			0.31785964	0.00038537	0.2951551
chr20	cg09309570			-0.2855472	0.0003854	0.2951551
chr3	cg14508198	ExonBnd;Body	RP;U2SURP	0.16638729	0.00038627	0.2951551
chr12	cg11803027			0.20307871	0.00038687	0.2951551
chr7	cg21671879			0.2140401	0.00038694	0.2951551
chr22	cg14846750	Body;Body;Body		0.20885935	0.00038715	0.2951551
chr5	cg15890173	TSS200	ANKRD31	0.14814095	0.00038755	0.2951551
chr10	cg05610379	1stExon;1stExon	A;NSMCE4A;NSMCE4A;NSMCE4A;NSMC	0.12875163	0.00038778	0.2951551
chr15	cg22084098	1500;TSS1500;TSS1500	107F6.3;MAPKBP1;MAPKBP1;MAPKBP1;	0.18353817	0.00038832	0.2951551
chr18	cg15139222	Body;TSS1500	KIAA1328;TPGS2;TPGS2;KIAA1328	-0.1543282	0.00038839	0.2951551
chr12	cg25537060	dy;Body;Body;Body;Bo	RP11-406H4.1	0.15765983	0.00038868	0.2951551
chr1	cg07141207	Body;Body	VPS13D	0.2118502	0.0003889	0.2951551
chr7	cg06109608	1stExon;5'UTR	PDIA4;PDIA4	0.25510962	0.00038915	0.2951551
chr18	cg04442497	TSS200;TSS200	PIAS2;PIAS2	0.12161235	0.00038994	0.29544165

chr11	cg01550685		GDPD4;GDPD4;GDPD4	0.20304516	0.00039074	0.29547482
chr11	cg23156348		687M24.3;TMEM218;TMEM218;TMEM218;	0.15208098	0.00039079	0.29547482
chr21	cg21039874	TSS200	CYYR1;CYYR1	0.17392182	0.00039134	0.29552412
chr17	cg24793470	Body;5'UTR;Body	GOSR1;GOSR1;GOSR1;GOSR1	0.15634372	0.00039177	0.29552412
chr17	cg09435821	5'UTR	RBFOX3;RBFOX3	-0.2175198	0.00039208	0.29552412
chr1	cg13990197			-0.1697242	0.00039336	0.29585425
chr3	cg26292910	UTR;Body;5'UTR;5'UT	K2;IP6K2;IP6K2;IP6K2;IP6K2;IP6K2;IP6K2;	-0.1946686	0.00039392	0.29585425
chr17	cg17118948	Body;Body;Body	CACNB1;CACNB1	0.17486252	0.00039405	0.29585425
chr17	cg26200976	TSS200	NUP85;NUP85;NUP85;NUP85	0.17184902	0.0003945	0.29585425
chr4	cg25223851	0;TSS200;TSS200	PA2;PPA2;PPA2;PPA2	0.22697801	0.00039456	0.29585425
chr20	cg13640430			0.17537593	0.00039545	0.29597838
chr13	cg01213557	5'UTR	SLC7A1;SLC7A1	0.15629659	0.00039577	0.29597838
chr6	cg20206224	TSS200;1stExon;5'UTR	5;POLH;POLH;XPO5;XPO5;XPO5;XPO5	0.15393508	0.000396	0.29597838
chr14	cg13082157	TSS1500;TSS200	5;SNHG10;SNHG10;SNHG10;GLRX5	0.13641877	0.00039751	0.29597838
chr9	cg01707425	TSS1500	ZNF618;ZNF618;ZNF618	0.26201567	0.00039757	0.29597838
chr1	cg07855129	TSS200;5'UTR	LRRC8D;LRRC8D;LRRC8D	0.27254874	0.00039771	0.29597838
chr6	cg19109204	Body	TFAP2B	0.2244559	0.00039786	0.29597838
chr7	cg06110297		STEAP1B	0.28335923	0.00039798	0.29597838
chr3	cg13523587	1500;TSS1500	NISCH;NISCH;NISCH;TNNC1	-0.1008889	0.00039934	0.29647814
chr1	cg06388206	TSS200;TSS200	DR63	0.24386153	0.00040028	0.29647814
chr20	cg12017057	Body		-0.1947123	0.00040037	0.29647814
chr16	cg04408576	1stExon;1stExon	CN7;CLCN7;CLCN7;CLCN7;CLCN7;CLCN	0.22297183	0.00040062	0.29647814
chr1	cg03688501			0.1889882	0.0004007	0.29647814
chr1	cg06796611	;5'UTR	IL24;IL24;IL24;IL24;IL24;IL24;IL24	0.16470066	0.00040112	0.29648935
chr18	cg02442222	5'UTR;Body;Body	OSBPL1A	0.20595137	0.00040197	0.29660269
chr11	cg11356794	TSS200	929.5	0.15441668	0.00040209	0.29660269
chr15	cg25441207	Body	GPR176;GPR176	0.17588996	0.00040251	0.29660642
chr10	cg05361325	TSS200	135A24.2;EPC1;EPC1;AL391839.1;EPC1	0.16689981	0.00040343	0.29698654
chr2	cg06522768	Body	FAM82A1	-0.3120881	0.00040402	0.29711896
chr4	cg03416665	TR;5'UTR	;PDLIM5;PDLIM5;PDLIM5;PDLIM5	0.22795341	0.00040486	0.29714282
chr7	cg08945844	Body;Body;Body	RELN;7SK;CTB-107G13.1	0.18705569	0.00040487	0.29714282
chr1	cg26426745	TSS1500;Body		0.43061754	0.00040556	0.297349

chr3	cg03320508	Body	RP11-88I21.2	0.24984449	0.00040674	0.29743312
chr10	cg08848887	Body;Body;Body;Body;	CREM;CREM;CREM;CREM;CREM	0.20915812	0.00040707	0.29743312
chr18	cg24474701	1stExon;5'UTR	NARS;NARS;NARS;NARS;NARS	0.24966227	0.00040726	0.29743312
chr17	cg11089813	Body		0.14576507	0.00040771	0.29743312
chr11	cg20493960	Body		0.13880381	0.00040818	0.29743312
chr18	cg25284252	Body		0.17436935	0.00040821	0.29743312
chr14	cg09184192	Body		0.14882932	0.00040855	0.29743312
chr4	cg11804293			0.20218865	0.00040913	0.29755865
chr13	cg22075222	Body;Body;Body;Body	CCNA1	0.21202317	0.00041033	0.29774074
chr3	cg16953067	TSS200	RAD18;RAD18;RAD18;RAD18;RAD18	0.12090038	0.00041042	0.29774074
chr2	cg08697297	5'UTR	LYG1;LYG1;LYG1;C2orf15	0.18283206	0.00041069	0.29774074
chr1	cg16651196	TSS200	BMP8A	0.1594806	0.00041133	0.29774074
chr12	cg04402351			0.17208204	0.00041156	0.29774074
chr2	cg00900124	TSS200	AC092431.1;ANXA4	0.34522076	0.00041209	0.29774074
chr19	cg23048660	TSS1500	ZNF180;ZNF180	0.25063008	0.0004123	0.29774074
chr18	cg22532061	5'UTR	ELAC1;SMAD4	0.13669	0.00041266	0.29774074
chr8	cg13996619	5'UTR	2336O2.1;CLN8;CLN8	0.21060084	0.00041492	0.2990725
chr1	cg22957381	TSS1500;TSS1500	88H9.1;KCND3	0.14541612	0.000416	0.2993455
chr1	cg11809162			-0.1074613	0.00041612	0.2993455
chr10	cg25793692	UTR;5'UTR	CDK1;CDK1;CDK1;CDK1;CDK1;CDK1	0.19693389	0.00041723	0.29984499
chr1	cg06850597		RP11-134G8.7	-0.1007688	0.00041806	0.30014203
chr6	cg25349391	Body;Body;Body		0.20399803	0.00041878	0.30036483
chr5	cg18114890	Body		-0.2837435	0.00042071	0.3007869
chr7	cg18417464	TSS1500	UC7L2;C7orf55;LUC7L2	0.19003047	0.00042185	0.3007869
chr17	cg11920325	TSS1500	NLK;NLK	0.19539045	0.00042224	0.3007869
chr11	cg23940915	Body	OPCML	0.21954679	0.00042229	0.3007869
chr6	cg16113813			-0.116518	0.00042234	0.3007869
chr21	cg25675393	1500;TSS1500;TSS1500	RB1	0.13858304	0.00042277	0.3007869
chr17	cg23869307	TSS1500;5'UTR	UBTF;UBTF;UBTF;UBTF;UBTF;UBTF	0.37624316	0.00042284	0.3007869
chr1	cg00866484	0;TSS200	9;OSBPL9	0.13717682	0.00042293	0.3007869
chr19	cg17122153	5'UTR;5'UTR;1stExon	FAM125A;FAM125A;FAM125A;FAM125A;	0.15560553	0.0004231	0.3007869
chr2	cg27227250			-0.159555	0.00042422	0.30094311

chr7	cg14644787	TSS200;Body	SRI;SRI;SRI;SRI;SRI;SRI	0.18249921	0.00042544	0.30094311
chr8	cg05353977	ody	OXR1;OXR1;OXR1	0.51299345	0.00042586	0.30094311
chr4	cg24411535	Body;Body	HERC3;HERC3	0.23597915	0.0004259	0.30094311
chr8	cg06898585			0.27041855	0.00042605	0.30094311
chr1	cg22635935			-0.4521421	0.00042637	0.30094311
chr1	cg24557058	1stExon;5'UTR	CR1L;CR1L;CR1L;CR1L	-0.2258753	0.00042713	0.30094311
chr2	cg23538590	1500;TSS1500;TSS1500	3L2	0.17370642	0.0004278	0.30094311
chr7	cg24465041	Body	Y_RNA;AC068610.5	0.18420065	0.00042811	0.30094311
chr6	cg26165406	stExon;1stExon;5'UTR	35B3	0.24725246	0.00042827	0.30094311
chr1	cg12720424		RP11-148B18.1;ARHGAP29	0.19399115	0.00042955	0.30094311
chr14	cg02411284	Body	CLMN;CLMN;CLMN	0.18942158	0.00042978	0.30094311
chr10	cg11132518		RP11-69C17.3	0.16299199	0.00043038	0.30094311
chr5	cg05522148			0.37422197	0.00043072	0.30094311
chr20	cg04834228	3'UTR	NTSR1;NTSR1	-0.1572875	0.00043119	0.30094311
chr1	cg03805558	1500;1stExon;TSS1500;	11B	0.14407363	0.00043229	0.30094311
chr5	cg00593008	Body		0.16753731	0.00043305	0.30094311
chr5	cg27222301	TSS200	DUSP1;RP11-779O18.3	0.15150059	0.00043598	0.30094311
chr8	cg11948688	5'UTR	2336O2.1;CLN8;CLN8	0.12588193	0.00043604	0.30094311
chr13	cg18326313	Body;Body	PSPC1-AS1;PSPC1;PSPC1;PSPC1	0.2155985	0.00043612	0.30094311
chr1	cg04261791	5'UTR;1stExon	STXBP3;STXBP3;STXBP3;STXBP3	0.15903949	0.00043658	0.30094311
chr19	cg02698668			-0.1628792	0.00043704	0.30094311
chr4	cg19502700	0;TSS200	ARP7;LARP7;LARP7;LARP7;LARP7;LARP	0.43416407	0.00043731	0.30094311
chr3	cg07985746	Body		0.16622556	0.00043736	0.30094311
chr7	cg18037107	Body	310H4.1;VOPP1;VOPP1;VOPP1	0.14093743	0.00043771	0.30094311
chr1	cg11266570	5'UTR;1stExon;1stExon	PRUNE;PRUNE;PRUNE;PRUNE;PRUNE;P	0.16906362	0.00043798	0.30094311
chr7	cg03641797	Body		-0.2153206	0.00043888	0.30094311
chr10	cg13336642	Body;Body	C10orf125;C10orf125;C10orf125	0.20010979	0.00043973	0.30094311
chr14	cg08139688	5'UTR;5'UTR	4;RP11-665C16.5	0.19407051	0.00043978	0.30094311
chr7	cg20496478	Body	MOXD2P;MOXD2P	0.17963877	0.00044035	0.30094311
chr2	cg09116068	UTR	DOK1;DOK1;LOXL3;LOXL3;LOXL3	0.24953882	0.00044079	0.30094311
chr7	cg02373627	Body	ZNF783;ZNF783	-0.1856713	0.00044136	0.30094311
chr5	cg23027345	TSS200	PGGT1B;PGGT1B;PGGT1B	0.17089505	0.00044153	0.30094311

chr6	cg01856558	Body;Body	RP5-1077H22.1	0.15518143	0.00044183	0.30094311
chr2	cg05034918	TSS1500	FAM82A1;FAM82A1;FAM82A1	0.1308811	0.00044189	0.30094311
chr4	cg01106248			-0.3190009	0.00044201	0.30094311
chr4	cg04446576			0.21898097	0.00044224	0.30094311
chr3	cg00482389	Body;Body;Body;Body	LZTFL1;LZTFL1;LZTFL1;LZTFL1	0.16728447	0.00044411	0.30094311
chr4	cg05527034	Body;Body	DCHS2	0.15737873	0.00044466	0.30094311
chr10	cg14171448	Body		0.22979858	0.0004447	0.30094311
chr17	cg08512882	TSS200;TSS200	SLC25A39;SLC25A39;SLC25A39	0.13427182	0.00044608	0.30094311
chr17	cg11341775			0.12930168	0.00044615	0.30094311
chr21	cg09659514	1stExon;TSS200;5'UTR	URB1;URB1;C21orf119	0.15970055	0.00044633	0.30094311
chr7	cg13798825	00	CREB5;CREB5;CREB5;CREB5	0.20090627	0.00044637	0.30094311
chr18	cg25212146	TSS1500	DSEL	0.1616847	0.00044668	0.30094311
chr8	cg02498439		RP11-120I21.2	0.17133469	0.00044737	0.30094311
chr5	cg09934003	Body;Body		0.13870728	0.00044746	0.30094311
chr8	cg09667617	TSS200;Body	PSD3;PSD3;PSD3;PSD3;PSD3;PSD3;PSD3	0.17573053	0.00044783	0.30094311
chr9	cg15310270	TSS200	SVEP1;SVEP1;SVEP1	0.15861718	0.00044807	0.30094311
chr1	cg09785555	TSS200	PEAR1;PEAR1;PEAR1;PEAR1	0.18048358	0.00044807	0.30094311
chr10	cg26838449	TSS200	NMT2;NMT2;NMT2;NMT2	0.15997471	0.00044845	0.30094311
chr18	cg12583607	Body	SOGA2;SOGA2	0.18475701	0.00044877	0.30094311
chr21	cg10542708	Body	DSCR8;DSCR8;TMPRSS3	0.17373196	0.00044992	0.30094311
chr5	cg23697400	TSS200;TSS200	CWC27;CWC27;CWC27	0.22287356	0.0004502	0.30094311
chr19	cg06380553			0.21366415	0.00045065	0.30094311
chr22	cg05064352	TSS1500;TSS1500	PHF21B;PHF21B	0.18123394	0.00045072	0.30094311
chr7	cg08132980	Body;Body	IQCE;IQCE;IQCE	-0.2044979	0.00045118	0.30094311
chr2	cg16873443		AC114730.2	0.18514719	0.00045192	0.30094311
chr17	cg01532051	dy;Body		0.2649724	0.00045264	0.30094311
chr7	cg21959181	00	A2;CDK5;SLC4A2;SLC4A2;CDK5	0.32624713	0.00045265	0.30094311
chr6	cg04471409	TSS1500;Body	PS6KA2	0.20793911	0.00045281	0.30094311
chr1	cg05114234	TSS200;Body	RP11-31F15.2;LRIG2;RP11-31F15.2	0.15654672	0.00045304	0.30094311
chr10	cg16000665	Body;Body;Body;Body	13C	0.19383494	0.0004533	0.30094311
chr17	cg24099023	0;Body	C;RAD51C;RAD51C;RAD51C	0.267037	0.00045357	0.30094311
chr15	cg17332963	SS200;TSS200;TSS200;	ARG2;NARG2;RP11-219B17.1;RP11-	0.13082494	0.00045408	0.30094311

chr11	cg26744402			-0.3470421	0.00045454	0.30094311
chr8	cg07068089		PVT1;PVT1	0.16752406	0.00045572	0.30094311
chr6	cg26422792	Body	RP1-104O17.1	0.27803451	0.00045609	0.30094311
chr12	cg05856601		UBE2N	-0.2220596	0.00045638	0.30094311
chr4	cg01810502	TSS1500;TSS200	BC1D14	0.2103455	0.00045647	0.30094311
chr9	cg14344122	Body;Body	DNM1	0.44826495	0.00045683	0.30094311
chr2	cg26818340	Body;Body;Body;Body		0.27041765	0.00045744	0.30094311
chr7	cg15370005	UTR	KIAA1324L;KIAA1324L;KIAA1324L	0.15518847	0.00045817	0.30094311
chr13	cg06016577			-0.2802642	0.00045946	0.30094311
chr2	cg09387749	TSS200	HOXD3;HOXD3;HOXD3;HOXD3	0.23431479	0.00045947	0.30094311
chr11	cg05218311	TSS200	ODZ4;ODZ4;ODZ4	0.27810597	0.00045959	0.30094311
chr3	cg06609282		U6	0.29698098	0.00045995	0.30094311
chr14	cg19326916	Body;Body	SALL2;SALL2;SALL2;SALL2	0.16445059	0.00046002	0.30094311
chr22	cg09390308			0.19853505	0.00046005	0.30094311
chr10	cg26956444	TSS200	HMX3	0.16690698	0.00046014	0.30094311
chr10	cg14460403	TSS200;Body;TSS200	MX2OS;EMX2OS;EMX2OS	0.31835186	0.00046019	0.30094311
chr6	cg03583318	Body	RPS6KA2	0.24997058	0.00046029	0.30094311
chr17	cg05390437	1stExon;5'UTR	R45L;WDR45L;WDR45L;WDR45L;WDR45	0.16696355	0.00046077	0.30094311
chr12	cg13175739	5'UTR	STYK1;STYK1;STYK1;STYK1;STYK1	0.36137086	0.00046099	0.30094311
chr20	cg03737691	TSS200	ZNF512B;TCEA2;SOX18	0.26908996	0.0004613	0.30094311
chr2	cg12270119	Body;Body	DLX1;DLX1;DLX1	0.24580309	0.00046155	0.30094311
chr17	cg26619043	5'UTR;TSS200	SLC16A6;ARSG	0.30391638	0.00046222	0.30094311
chr5	cg03559817	'UTR;1stExon;1stExon;	AS1;SMAD5;SMAD5;SMAD5;SMAD5;SMA	0.31228793	0.00046231	0.30094311
chr10	cg04141377			0.20732099	0.00046265	0.30094311
chr13	cg15671725	Body;Body	PCDH9-AS2	0.18994399	0.00046287	0.30094311
chr9	cg01781202			0.17888839	0.00046318	0.30094311
chr11	cg05262777		ARHGAP32	0.23856997	0.00046344	0.30094311
chr19	cg25755428	TSS1500;TSS1500	MRI1	1.87761309	0.00046385	0.30094311
chr10	cg01906237			0.17114078	0.00046501	0.30094311
chr11	cg00776741	Body	NADSYN1	0.20920869	0.0004652	0.30094311
chr15	cg19136717	5'UTR;1stExon	1007O24.3;ARIH1;ARIH1;ARIH1;RP11-	0.27415802	0.00046535	0.30094311
chr1	cg03352346	Body;Body	PRDM16	-0.1261149	0.00046538	0.30094311

chr15	cg07729059			0.34950638	0.00046546	0.30094311
chr11	cg21194723	TSS200	881M11.1;TMEM109	0.2364381	0.00046551	0.30094311
chr20	cg12108990			0.20922944	0.00046573	0.30094311
chr10	cg24387129	3'UTR	OR13A1	0.21411977	0.00046588	0.30094311
chr20	cg14866117	Body	ANGPT4	-0.1459437	0.00046603	0.30094311
chr3	cg02397640		RP11-217E22.5;RP11-23D24.2	-0.283451	0.00046619	0.30094311
chr12	cg06107469	1stExon;5'UTR	IP	0.12593984	0.00046719	0.30094311
chr5	cg19780254	n;5'UTR;5'UTR;5'UTR;	ACSL6;ACSL6;ACSL6;ACSL6;ACSL6;ACS	0.33394301	0.00046728	0.30094311
chr10	cg13464955	TSS200;Body	RP11-462G8.3;TCERG1L	-0.1355503	0.00046742	0.30094311
chr2	cg20690483	TSS200	STARD7;STARD7;STARD7;AC012307.3	0.1653401	0.00046781	0.30094311
chr12	cg14529170	Body		-0.2399562	0.00046827	0.30094311
chr15	cg25079408			0.20496469	0.00046861	0.30094311
chr17	cg20009499	;TSS1500;5'UTR	PRR11;SKA2;SKA2;SKA2;SKA2	0.16872436	0.00046925	0.30094311
chr16	cg08804013	R;TSS1500;Body	MIR1538;NFAT5;NFAT5;NFAT5	-0.183864	0.00046934	0.30094311
chr7	cg22586324	1stExon	LMOD2;LMOD2;LMOD2;LMOD2	0.16946773	0.00047045	0.3013839
chr9	cg14144038	5'UTR;5'UTR;5'UTR	L2;ELAVL2	0.14713096	0.00047133	0.3013839
chr11	cg22697540	5'UTR;5'UTR;TSS1500	D5;ZBED5;ZBED5;CTD-2003C8.1;CTD-	0.18322763	0.00047203	0.3013839
chr12	cg21746969	;5'UTR;Body;5'UTR;1st	TM2;LRTM2;LRTM2;CACNA2D4;LRTM2;	-0.4113241	0.00047212	0.3013839
chr17	cg17769925	0;TSS200	2;PDK2;PDK2	0.1222864	0.00047249	0.3013839
chr8	cg18288121			0.17299577	0.00047252	0.3013839
chr1	cg20607106	TSS200	ALG14;ALG14	0.28635787	0.00047382	0.30195114
chr1	cg16318112	Body	AGRN;AGRN	0.24248564	0.00047438	0.30204268
chr19	cg08207256	'UTR;1stExon;1stExon;	MIR638;DNM2;DNM2;DNM2;DNM2	0.27478004	0.00047552	0.30249924
chr19	cg26532042	TSS200	FTL;FTL	0.31232339	0.00047615	0.3026359
chr12	cg00327031			0.22056256	0.0004766	0.30265763
chr3	cg17762035	1stExon;5'UTR	ZDHHC23;ZDHHC23	0.28727438	0.00047828	0.30266245
chr13	cg20840706	1500	AS1;INTS6;INTS6;INTS6;INTS6;INTS6;INT	0.36844842	0.00047866	0.30266245
chr19	cg14558639	Body;Body		0.29522702	0.00047903	0.30266245
chr1	cg22772170	5'UTR;5'UTR;5'UTR	FMO1;FMO1;RP1-45C12.1	0.19115004	0.00047912	0.30266245
chr8	cg00189906	TSS1500	FAM91A1;FAM91A1;FAM91A1;FAM91A1	0.17687477	0.00047927	0.30266245
chr7	cg11835459			-0.2901816	0.00047937	0.30266245
chr6	cg17586256	Body		0.16044371	0.00047974	0.30266245

chr1	cg09011950		RP11-372M18.2	0.18350574	0.00047999	0.30266245
chr18	cg21171746	Body		0.27392364	0.00048089	0.30266245
chr2	cg07310661	5'UTR;1stExon	CUL3;CUL3;CUL3;CUL3	0.30228392	0.00048148	0.30266245
chr19	cg27146808	Body	SAMD1	0.14587071	0.00048158	0.30266245
chr3	cg17082662	TSS200	C3orf38;C3orf38;C3orf38;CGGBP1;CGGBP1	0.18976304	0.0004821	0.30266245
chr2	cg12895717	3'UTR	MEIS1;MEIS1;MEIS1;MEIS1;MEIS1;MEIS1	-0.2647076	0.00048278	0.30266245
chr2	cg11274233	TSS200;TSS200	ATP6V1C2	0.14149808	0.000483	0.30266245
chr16	cg01811561	0	GFOD2	0.1690841	0.00048313	0.30266245
chr5	cg09667170	TSS200	NEURL1B	0.18937074	0.00048328	0.30266245
chr19	cg18568930	Body		0.31480364	0.00048383	0.30274481
chr1	cg07159802	1stExon	OR1C1	0.1630835	0.00048477	0.30295206
chr6	cg04893786			0.23164065	0.00048685	0.30295206
chr5	cg19389056	Body;Body		-0.1968668	0.0004869	0.30295206
chr2	cg26381918	TSS1500;5'UTR	UNC50	0.23345484	0.00048719	0.30295206
chr20	cg26046329			0.20982931	0.00048759	0.30295206
chr2	cg00405942		CRYGEP	0.27679036	0.00048822	0.30295206
chr1	cg07318050	TSS1500	PCR	0.22876472	0.00048861	0.30295206
chr1	cg14985478	TSS200;TSS200	ZNHIT6;ZNHIT6	0.19818607	0.00048904	0.30295206
chr14	cg13148496	Body		-0.2098873	0.00048907	0.30295206
chr2	cg15636480			0.20311578	0.00048981	0.30295206
chr17	cg12694472	Body;Body		-0.2336696	0.00048989	0.30295206
chr2	cg20262764	Body;Body	NKMY1	0.19693438	0.00049017	0.30295206
chr11	cg03036422	Body;Body;Body	PVRL1;PVRL1	-0.1685458	0.00049075	0.30295206
chr4	cg18924209	TSS1500;Body	1-15B17.1;RP11-15B17.1;RP11-15B17.1	0.18587133	0.00049151	0.30295206
chr1	cg05682181			0.1754824	0.00049244	0.30295206
chr3	cg20888019	Body;Body		0.3002291	0.0004929	0.30295206
chr11	cg02583999	Body	OPCML-IT1;OPCML	-0.1750952	0.00049293	0.30295206
chr15	cg21348997	5'UTR;1stExon	210M15.2;ARNT2;ARNT2;ARNT2;ARNT2;	0.24860162	0.00049323	0.30295206
chr20	cg24418346	5'UTR	CDH22	-0.183703	0.00049328	0.30295206
chr19	cg21192775			-0.1915313	0.00049356	0.30295206
chr11	cg16821068	TSS200	BARX2	-0.1835878	0.0004937	0.30295206
chr18	cg20581250	TSS200	HDHD2	0.12881607	0.00049449	0.30295206

chr10	cg24880387	Body	TRUB1	0.15062491	0.00049479	0.30295206
chr2	cg11432790		AC079779.4	0.26937531	0.00049512	0.30295206
chr7	cg18494018	TSS1500;TSS1500	EZH2	0.17855377	0.00049515	0.30295206
chr7	cg12656475	;5'UTR	AS1;STEAP2;STEAP2;STEAP2;STEAP2;ST	0.24495805	0.00049561	0.30295206
chr16	cg13663385		RP11-488I20.9;RP11-244B22.6	-0.2986236	0.00049637	0.30295206
chr22	cg14703589	;Body;5'UTR	2;LIMK2;LIMK2;LIMK2	-0.193464	0.00049726	0.30295206
chr4	cg24322144	TSS200	TMEM128;TMEM128;TMEM128	0.1812289	0.00049733	0.30295206
chr19	cg23532138	3'UTR	CYP2S1	0.13666657	0.00049774	0.30295206
chr12	cg05358915			-0.1237788	0.00049787	0.30295206
chr1	cg07798602	5'UTR	JAK1	0.1679616	0.00049835	0.30295206
chr16	cg13663252		RP11-538I12.3;RP11-538I12.2	0.28734253	0.00049905	0.30295206
chr6	cg24738424		RP3-495K2.3	0.23265686	0.00049924	0.30295206
chr2	cg10425361	TSS1500	CYS1;CYS1	0.18534443	0.00050052	0.30295206
chr16	cg07277923			0.21314624	0.00050082	0.30295206
chr1	cg25018357			0.2275324	0.00050133	0.30295206
chr13	cg01063243	1stExon;5'UTR	CBP2;MYCBP2;MYCBP2;MYCBP2	0.35738986	0.00050153	0.30295206
chr10	cg25812309			0.17825298	0.00050154	0.30295206
chr21	cg21436342	Body	AP001422.3;AP001422.3;TMPRSS3	0.15095749	0.00050167	0.30295206
chr3	cg23284139	SS1500;TSS1500	FB5;NDUFB5;NDUFB5;NDUFB5;NDUFB5;	0.12317446	0.00050199	0.30295206
chr11	cg00013006	TSS1500	OR52B4	0.17038257	0.0005022	0.30295206
chr2	cg18977641	TSS200	98A;FAM98A;FAM98A	-0.2056573	0.00050246	0.30295206
chr6	cg00092682	Body;Body		0.13430917	0.00050252	0.30295206
chr17	cg22533099			0.16104535	0.00050345	0.30308012
chr15	cg23364517		RP11-553E24.3	-0.1904686	0.00050357	0.30308012
chr3	cg06703881		AC133680.1	0.20938081	0.00050444	0.303208
chr16	cg20892069	Body;Body;Body	MRAL1;NMRAL1;NMRAL1	0.1491924	0.00050478	0.303208
chr6	cg11753363	Body	MDGA1	-0.1627119	0.00050504	0.303208
chr2	cg19386576	n;1stExon;5'UTR;5'UTR	;HADHB;HADHB;HADHB;HADHA;HADH	0.16120124	0.0005057	0.3033272
chr10	cg01017197	TSS1500;TSS200	BLOC1S2;BLOC1S2;BLOC1S2	0.17639612	0.00050635	0.3033272
chr6	cg10089294	SS200;TSS200	C29A1;SLC29A1	0.13614571	0.00050649	0.3033272
chr11	cg03874344	Body	RP11-23F23.2	0.17334866	0.00050846	0.30380412
chr22	cg15446156	Body	KIAA1671;KIAA1671	-0.1456902	0.00050864	0.30380412

chr17	cg09732851	3'UTR	FAM101B	-0.280473	0.00050914	0.30380412
chr2	cg25820224	5'UTR;1stExon	PS5	0.18195405	0.00050956	0.30380412
chr22	cg01963885	TSS200	ARVCF;ARVCF;C22orf25;C22orf25	0.21168894	0.0005109	0.30380412
chr3	cg04442328	1stExon;5'UTR	2;SENP2;SENP2;SENP2;SENP2;SENP2;SEN	0.23249976	0.00051122	0.30380412
chr2	cg21069019		AC021021.2;AC021021.2	-0.2653281	0.00051158	0.30380412
chr12	cg13588483	5'UTR	444D3.1;RP11-444D3.1;RP11-444D3.1	0.16715364	0.00051176	0.30380412
chr8	cg02503933	5'UTR;1stExon	37B2.1;RIPK2;RIPK2;RIPK2;RIPK2;RIPK2;	0.29370875	0.00051177	0.30380412
chr5	cg00529697	5'UTR;1stExon	;POC5;POC5;POC5;POC5;POC5;POC5;POC	0.3967133	0.00051183	0.30380412
chr16	cg01472317	Body;Body;Body	ACSF3	-0.1028796	0.00051231	0.30380412
chr11	cg10847870	UTR;5'UTR;5'UTR	RNTL;ARNTL;ARNTL;ARNTL;ARNTL;AR	0.19632912	0.00051411	0.30380412
chr1	cg21968580	1stExon;5'UTR	B4;DNAJB4;DNAJB4;DNAJB4;GIPC2	0.21757338	0.00051489	0.30380412
chr2	cg21597595			0.38946585	0.00051511	0.30380412
chr22	cg01400493	Body;Body;Body		0.20163814	0.00051599	0.30380412
chr16	cg07901374			0.14529265	0.00051612	0.30380412
chr8	cg24360069	Body		0.16084792	0.00051621	0.30380412
chr2	cg25138484	TSS1500	ALPP;ALPP	-0.2320549	0.00051623	0.30380412
chr3	cg18452149	5'UTR;TSS200;1stExon	A1524;KIAA1524;KIAA1524;KIAA1524;DZI	0.17523894	0.00051693	0.30380412
chr5	cg23407804			0.23219938	0.00051714	0.30380412
chr3	cg07466493	Body;Body;Body;Body	C26A6;SLC26A6;SLC26A6;SLC26A6	-0.1011562	0.00051783	0.30380412
chr21	cg25866059	TSS1500;TSS1500	G2;UBE2G2;UBE2G2;UBE2G2;UBE2G2;UB	0.17827016	0.00051807	0.30380412
chr2	cg08457213		AC104655.3;AC104655.3;AC104655.3	0.13363273	0.00051809	0.30380412
chr5	cg17008295	Body	MCTP1	0.18324666	0.00051855	0.30380412
chr12	cg01877212	TSS200	C12orf45;C12orf45	0.18644278	0.00051907	0.30380412
chr4	cg01468272	Body	RP11-565A3.1;RP11-565A3.2	-0.1276253	0.00051945	0.30380412
chr19	cg19708659	5'UTR	KANK3	0.29674358	0.00051949	0.30380412
chr7	cg22404042	TSS200	AC018464.3;AC018464.3	0.26788073	0.00051995	0.30380412
chr3	cg09007811	Body		0.19949259	0.00052018	0.30380412
chr2	cg24388610	Body;Body;Body	THADA;THADA;THADA;THADA	0.32503981	0.00052073	0.30380412
chr16	cg01466678	200	DRG4;NDRG4;NDRG4;NDRG4;NDRG4;ND	0.21549518	0.00052143	0.30380412
chr16	cg04044983	Body	SLC9A5;FHOD1;SLC9A5	0.25236174	0.0005215	0.30380412
chr6	cg10200629	UTR;5'UTR;TSS1500	HMGA1;HMGA1;HMGA1;HMGA1;HMGA1	0.13056587	0.00052159	0.30380412
chr10	cg26015743			-0.1593209	0.00052284	0.30380412

chr8	cg27334717	TSS1500;TSS1500	RP13-467H17.1	0.17378054	0.00052291	0.30380412
chr20	cg26172013	5'UTR;1stExon	SNTA1;SNTA1	0.19143484	0.00052329	0.30380412
chr3	cg10451772	TSS200	PODXL2	0.19923383	0.00052384	0.30380412
chr3	cg17402072	stExon;1stExon;5'UTR	;FXR1;FXR1;FXR1;FXR1;FXR1;FXR1;FXR	0.17832116	0.00052397	0.30380412
chr1	cg11424597	5'UTR;1stExon	ERI3;ERI3;ERI3	0.25826268	0.00052465	0.30380412
chr1	cg24584721		PHC2;PHC2;PHC2	0.21287337	0.00052476	0.30380412
chr13	cg00574740	Body	173B14.5;RP11-29G8.3	-0.1253847	0.00052555	0.30380412
chr2	cg19430586	UTR;5'UTR;5'UTR;5'U	TTN;TTN;TTN;TTN;TTN;TTN	0.16733378	0.00052596	0.30380412
chr20	cg24531267	TSS200	1;GDF5;CEP250;CEP250;CEP250;CEP250;C	0.16115869	0.0005265	0.30380412
chr3	cg19458891			-0.1454993	0.00052653	0.30380412
chr7	cg13552468	Body	U2;GS1-179L18.1	0.19891751	0.00052706	0.30380412
chr13	cg19445097			-0.2574704	0.00052732	0.30380412
chr14	cg22699193	TSS200	ZFP36L1;ZFP36L1;ZFP36L1;ZFP36L1	0.19128393	0.00052743	0.30380412
chr8	cg14159108	Body	127H5.1;ZFPM2;ZFPM2	0.17548273	0.00052821	0.30380412
chr18	cg07372879	TSS200	ZCCHC2	0.15348491	0.00052829	0.30380412
chr17	cg17578717	00;TSS200;TSS200	INCA1;INCA1;KIF1C;KIF1C	0.17086582	0.00052907	0.30380412
chr16	cg09325291	TSS200;TSS1500	ME2;NME3;NME3;NME3;NME3;NME3	0.23637099	0.00052971	0.30380412
chr6	cg25835311	TSS1500		0.17333931	0.00053007	0.30380412
chr7	cg22100553			0.18825827	0.00053013	0.30380412
chr4	cg07681935	Body;TSS1500	471J12.1;RP11-471J12.1;RP11-	0.2020966	0.00053162	0.30380412
chr14	cg04436646	TSS1500	KLHDC1;KLHDC1;KLHDC1	0.1458443	0.00053187	0.30380412
chr9	cg13219226	1500	613M10.9	0.18783134	0.00053209	0.30380412
chr16	cg20692900	dy;Body;Body;Body;Bo	K2;RP11-403P17.4	0.15042575	0.00053217	0.30380412
chr7	cg09606588			0.19689754	0.00053271	0.30380412
chr6	cg00380172	TSS200	SASH1	0.22834666	0.0005329	0.30380412
chr11	cg04836530			0.19850677	0.00053304	0.30380412
chr2	cg13187130	TSS200	FAM110C;FAM110C	0.20719538	0.00053311	0.30380412
chr5	cg13327936	UTR;5'UTR;Body	2544H17.1;SNCAIP;SNCAIP;SNCAIP;SNCA	-0.2845613	0.00053408	0.30380412
chr18	cg12964741	S200;Body	NFATC1;NFATC1;NFATC1;NFATC1	0.24570084	0.00053432	0.30380412
chr12	cg17339927			-0.1358805	0.00053484	0.30380412
chr8	cg03869414		PDP1	0.32478425	0.00053507	0.30380412
chr8	cg00157319	Body;Body;Body;Body	STAU2	-0.1533944	0.00053538	0.30380412

chr16	cg10144964	1500;TSS1500	FUS;FUS;FUS;FUS;FUS;FUS	0.28268167	0.00053561	0.30380412
chr9	cg06405299	TSS1500	5;ARPC5L	0.23015036	0.00053575	0.30380412
chr11	cg06491116	Body		0.2087098	0.00053626	0.30385825
chr10	cg16260355	5'UTR;1stExon	SGPL1;SGPL1;SGPL1;SGPL1;RP11-432J9.4	0.17609016	0.00053869	0.30499416
chr5	cg23457619		RP11-124N3.3	0.20636394	0.00054136	0.30521103
chr1	cg24051554	Body;5'UTR	C2CD4D	0.26199102	0.00054143	0.30521103
chr3	cg26385494	Body	MYLK-AS1;MYLK-AS1;PTPLB	0.1459466	0.00054191	0.30521103
chr11	cg26185531	1stExon;5'UTR	2530H12.1;DGAT2;DGAT2;DGAT2;DGAT2;	0.23987574	0.00054213	0.30521103
chr15	cg01145430	Body;TSS1500	M	0.22099701	0.00054235	0.30521103
chr18	cg22352746	Body		0.1655083	0.00054344	0.30521103
chr11	cg12776156	UTR;5'UTR;5'UTR;5'U	4;TSPAN4;TSPAN4;TSPAN4;TSPAN4;TSP	-0.1119757	0.00054405	0.30521103
chr19	cg05678175	Body		-0.2002161	0.00054433	0.30521103
chr10	cg26488828		RP11-135D11.2	0.16307625	0.0005449	0.30521103
chr6	cg12997953	5'UTR	PAQR8;PAQR8;PAQR8	0.24839422	0.00054514	0.30521103
chr6	cg26766272	onBnd;ExonBnd;ExonB	1;SGK1;SGK1;SGK1;SGK1;SGK1;SGK1;SG	0.38649875	0.00054536	0.30521103
chr14	cg10518693	TSS200	OC5;EXOC5;AP5M1;AP5M1;EXOC5;AP5M	0.15848165	0.00054543	0.30521103
chr7	cg13665263	stExon	MTPN;MTPN;MTPN;MTPN	0.34023347	0.0005455	0.30521103
chr17	cg17746833			0.18588506	0.00054656	0.30521103
chr17	cg20840825	Body	SPECC1;SPECC1;SPECC1;SPECC1;SPECC1	0.19589489	0.00054689	0.30521103
chr5	cg26364097	Body	463N11.3;CTC-463N11.3	0.13892569	0.00054756	0.30521103
chr1	cg06746829	Body		0.2349147	0.0005481	0.30521103
chr6	cg12989020	1500	ARID1B;ARID1B;ARID1B	0.26284621	0.00054811	0.30521103
chr2	cg15767103	Body	EML6	0.15823835	0.00054845	0.30521103
chr4	cg23694616	TSS200	NDST3;NDST3;NDST3	0.1870638	0.00054899	0.30521103
chr20	cg03951132	stExon;TSS200	MT6;MCM8;MCM8;MCM8;MCM8	-0.1635033	0.00054903	0.30521103
chr1	cg16308270		RP11-576D8.2	0.20154911	0.00054911	0.30521103
chr1	cg00035237	TSS1500;TSS1500	M1;PUM1;PUM1;PUM1;PUM1	0.15951311	0.00054913	0.30521103
chr19	cg26172812	Body		0.17979391	0.00054962	0.30521103
chr5	cg17036833	Body	175K6.1;EBF1	0.13839741	0.00055023	0.30521103
chr5	cg22656351		RN5S178	0.17954954	0.00055072	0.30521103
chr1	cg12438155			0.19540694	0.00055089	0.30521103
chr19	cg09545123	TSS200;Body	MIR330	-0.2463866	0.00055101	0.30521103

chr20	cg16415710	Body;Body	FAM65C	0.27013539	0.00055168	0.30521103
chr1	cg09769655	0;5'UTR	201;C1orf201	0.15549553	0.00055235	0.30521103
chr1	cg11158057		CHRM3;CHRM3	0.186062	0.00055323	0.30521103
chr12	cg13683516	TSS200	2B;OBFC2B	0.16150472	0.00055329	0.30521103
chr3	cg02940101		PIK3CB;PIK3CB;PIK3CB;PIK3CB	0.1345347	0.00055337	0.30521103
chr11	cg07823293	TSS200;TSS200	RG1;TBRG1;TBRG1	0.14754994	0.00055371	0.30521103
chr13	cg07926066			0.18358296	0.00055379	0.30521103
chr16	cg16758037			0.22023088	0.00055457	0.30528971
chr13	cg07104706	1stExon;5'UTR	SLITRK1;SLITRK1	-0.2807617	0.00055477	0.30528971
chr12	cg15410411	TSS1500	AS1;HOXC5;HOXC6;HOXC9;HOXC6	0.39116045	0.0005554	0.305403
chr5	cg05255341	TSS1500;TSS1500	GM2A	0.16123876	0.00055625	0.3056428
chr22	cg06573331	Body	CTA-125H2.2;MYO18B;MYO18B	0.31572718	0.00055779	0.30587719
chr19	cg18139462	TSS1500;Body	C19orf55;C19orf55;C19orf55;C19orf55;C19or	-0.2632342	0.00055796	0.30587719
chr2	cg16942653		AC106053.1	0.18470267	0.00055807	0.30587719
chr13	cg12288985	TSS1500	EEF1DP3	0.14821111	0.00055837	0.30587719
chr4	cg02770809	Body	DTHD1	0.21296379	0.00055917	0.30608921
chr1	cg02576073	TSS1500	;SHC1;SHC1	0.21179555	0.00055991	0.30609842
chr17	cg15246895	5'UTR	RAI1-AS1;RAI1	-0.1039471	0.00056003	0.30609842
chr1	cg09799432	TSS200	612B15.3;SH3GLB1;SH3GLB1	0.19622142	0.00056205	0.30654146
chr16	cg01450228			-0.2346466	0.00056378	0.30654146
chr7	cg26102801			0.16968578	0.00056452	0.30654146
chr19	cg03305587	1stExon	CLPTM1;CLPTM1;CLPTM1	0.25606522	0.00056455	0.30654146
chr1	cg20048602	TSS200	AIP1;AURKAIP1;AURKAIP1;AURKAIP1;A	0.27245791	0.0005647	0.30654146
chr10	cg20816857	Body;Body;Body		0.18209281	0.00056512	0.30654146
chr1	cg15658487	TSS200	PTCHD2;PTCHD2;PTCHD2	0.18419272	0.00056513	0.30654146
chr1	cg15202770			0.15921936	0.00056543	0.30654146
chr12	cg20228119	Body;Body;Body	S1B;ANKS1B;ANKS1B;ANKS1B;ANKS1B;	0.11781524	0.00056602	0.30654146
chr1	cg14517946			0.21076832	0.00056629	0.30654146
chr13	cg06343869	stExon;5'UTR;1stExon	CHAMP1	0.12308643	0.00056648	0.30654146
chr20	cg18346693	TSS1500;TSS1500	STMN3	-0.1388091	0.00056651	0.30654146
chr5	cg05593349	5'UTR;1stExon	CMBL;CMBL;CMBL	0.13744632	0.00056712	0.30654146
chr11	cg26175933	Body;Body;Body;Body	MS4A6A;MS4A6A;MS4A6A;MS4A6A	0.15871657	0.00056739	0.30654146

chr12	cg01181492	0;TSS200;TSS200	BH2;ALKBH2;ALKBH2;ALKBH2;ALKBH2	0.26377368	0.00056748	0.30654146
chr4	cg23403868	Body		-0.1455529	0.00056761	0.30654146
chr8	cg11149658	Body	MCPH1;MCPH1	-0.1548	0.00056802	0.30654146
chr4	cg20173065	TSS1500	ANXA5	0.35992253	0.00056958	0.30683889
chr12	cg26935304	5'UTR;Body	OSBPL8;OSBPL8;OSBPL8;OSBPL8	0.24612866	0.00056986	0.30683889
chr1	cg04040682			0.18248132	0.0005703	0.30683889
chr11	cg04641330			0.16238302	0.00057059	0.30683889
chr17	cg12039422			0.19078189	0.00057069	0.30683889
chr1	cg16073238	Body	RP11-181B18.1;RP11-181B18.1	0.18055779	0.00057126	0.30685661
chr2	cg16777216		AC012506.4;AC012506.4	0.20393205	0.00057157	0.30685661
chr4	cg25710351	1500	PDS5A;PDS5A	0.17545863	0.00057278	0.30709521
chr16	cg20533393	TSS200;TSS200	SLC9A3R2;SLC9A3R2;SLC9A3R2	0.29404357	0.0005738	0.30709521
chr8	cg08318076		CLVS1	0.29310421	0.00057501	0.30709521
chr13	cg16602799	5'UTR;1stExon	ATP12A;ATP12A;ATP12A;ATP12A	0.12515553	0.00057507	0.30709521
chr3	cg14116399		C3orf58	0.17645452	0.00057556	0.30709521
chr3	cg08785396	Body;Body;Body;Body	ALCAM	0.15256008	0.0005758	0.30709521
chr8	cg11896633	TSS1500	FAM83H;RP11-429J17.6;RP11-429J17.6	0.1697845	0.00057589	0.30709521
chr12	cg09354309	5'UTR;1stExon	A1;ST8SIA1;ST8SIA1;ST8SIA1;ST8SIA1	0.15559578	0.00057696	0.30709521
chr6	cg25473293			-0.1164466	0.00057711	0.30709521
chr11	cg25101976	TSS1500;TSS200	11L1;TCP11L1;TCP11L1	0.12693066	0.00057729	0.30709521
chr6	cg19098017	5'UTR	RING1;RING1	0.24423131	0.00057738	0.30709521
chr5	cg00516449	5'UTR;1stExon	RXFP3;RXFP3	-0.2470132	0.00057776	0.30709521
chr19	cg06781950	TSS200;TSS200	RFX2	0.15149344	0.00057793	0.30709521
chr4	cg06113835		RP11-359D14.3	0.16385068	0.00057794	0.30709521
chr7	cg20496693	5'UTR;5'UTR;5'UTR	C7orf50;C7orf50;C7orf50;C7orf50	0.57974681	0.00057948	0.30747767
chr9	cg14214182	TSS1500	LINC00092	0.19901067	0.00057982	0.30747767
chr2	cg06318624			0.1930228	0.00058041	0.30747767
chr9	cg14631218	Body		-0.240427	0.00058092	0.30747767
chr5	cg00627755		7SK	0.23134427	0.00058099	0.30747767
chr4	cg03893307	5'UTR;1stExon	L;MTHFD2L;MTHFD2L;MTHFD2L;MTHFD	0.1581774	0.00058224	0.30747767
chr11	cg04458776	1500;TSS1500	SLC22A6;SLC22A6	0.17338179	0.00058243	0.30747767
chr1	cg09703732	xon;1stExon;Body;TSS1	ROX;BROX;BROX;AIDA;AIDA;AIDA;AID	0.15384098	0.00058277	0.30747767

chr6	cg19401895			0.51494315	0.00058345	0.30747767
chr6	cg04068042	1stExon;5'UTR	RP11-69I8.3;CTGF;CTGF;RP11-69I8.3	0.34698578	0.00058362	0.30747767
chr10	cg05171195	Body;Body;Body;Body	RHOBTB1;RHOBTB1	0.16561322	0.00058368	0.30747767
chr8	cg18063332			-0.1756696	0.00058374	0.30747767
chr11	cg10114358	5'UTR;Body;Body	RP5-916O11.2;LDLRAD3	0.18569689	0.0005843	0.30754981
chr17	cg17789517	TSS200	;AC091062.2	0.27367571	0.00058504	0.30762615
chr5	cg08691577	Body	FAM105B	0.15663853	0.00058564	0.30762615
chr14	cg11287675	TSS200	RP11-299L17.3	0.17367557	0.00058572	0.30762615
chr3	cg17055322			0.28398255	0.00058625	0.30768598
chr16	cg08823186	Body;Body	AXIN1	-0.2336918	0.00058751	0.30778225
chr8	cg23534983	TSS1500	ZNF7;ZNF7;ZNF7;ZNF7	0.2436934	0.00058767	0.30778225
chr18	cg00706410		U6	-0.1269512	0.00058835	0.30778225
chr20	cg21633795	TSS200	PFDN4;PFDN4;PFDN4;PFDN4;PFDN4	0.1497826	0.00058844	0.30778225
chr7	cg07378472	TSS200;TSS200	PHF14;PHF14;PHF14	0.15666923	0.00058895	0.30778225
chr7	cg18502334			0.11373271	0.00058898	0.30778225
chr3	cg14527942	Body		-0.197973	0.00058948	0.30781994
chr11	cg03959549		RP11-25I9.2	0.21136373	0.0005899	0.30781994
chr6	cg08698655	Body;Body		0.14924373	0.00059111	0.30822994
chr16	cg08562016	ody	GFOD2;GFOD2	0.14673383	0.00059287	0.30856772
chr5	cg05492752	TSS1500;TSS1500	ATG10;ATG10;ATG10;ATG10	0.36181384	0.00059321	0.30856772
chr3	cg18473544			0.15943475	0.00059329	0.30856772
chr16	cg02609850	Body	ZFPM1;ZFPM1;ZFPM1	-0.2460005	0.00059346	0.30856772
chr2	cg17193639	Body		0.23996242	0.0005951	0.30899737
chr6	cg10710974	Body;Body		0.18171748	0.00059521	0.30899737
chr7	cg00546491	TSS200	1C;PDE1C	-0.1520494	0.00059556	0.30899737
chr1	cg18387343	1stExon	NUDC;NUDC	0.2160903	0.00059671	0.30904484
chr9	cg26467952	Body;Body;Body;Body	PRUNE2;PRUNE2;PRUNE2;PRUNE2	0.19431422	0.00059676	0.30904484
chr5	cg06853376	Body	PHAX	0.29537249	0.00059693	0.30904484
chr1	cg16813892	0;TSS200	F2;MTF2	0.14574095	0.00059748	0.30909175
chr7	cg21053748	1stExon;5'UTR	FA4;NDUFA4	0.13286819	0.00059846	0.30909175
chr2	cg07808773	0;TSS200;TSS200;TSS2	MFF;MFF;MFF;MFF;MFF;MFF;MFF;MFF;	0.18170764	0.00059933	0.30909175
chr13	cg27463736	1stExon;TSS200;5'UTR	LIG4;LIG4;ABHD13	0.14144655	0.00060053	0.30909175

chr8	cg20465143	Body		-0.4863929	0.00060114	0.30909175
chr15	cg04865659	Body;Body	SEMA4B;SEMA4B	-0.2401106	0.00060259	0.30909175
chr10	cg00624332	TSS200	LDB1;RP11-302K17.7;LDB1	0.28235005	0.00060398	0.30909175
chr3	cg02786951	Body	RP11-631B21.2	0.15145006	0.00060418	0.30909175
chr17	cg12166802	TSS200	UTS2R	-0.2430665	0.000605	0.30909175
chr19	cg17308464	TSS1500	CALR;CALR	0.20639835	0.00060517	0.30909175
chr3	cg01137401	Body	ZIC4;ZIC4;ZIC4;ZIC4;ZIC4;ZIC4;ZIC4	0.28800535	0.00060523	0.30909175
chr14	cg18403361	TSS200	CLEC14A;CLEC14A	0.22166453	0.00060623	0.30909175
chr14	cg21063480	1stExon	SPTLC2	0.20736194	0.00060659	0.30909175
chr13	cg26348690			0.24327421	0.00060668	0.30909175
chr1	cg07374403	TSS200;TSS200	AS1;ATPAF1;ATPAF1;ATPAF1;ATPAF1;A	0.1868932	0.0006067	0.30909175
chr4	cg02182354	TSS200	RP11-402J6.1;NEUROG2;RP11-402J6.1	-0.1276218	0.00060725	0.30909175
chr13	cg08830225		LINC00464	0.14811039	0.00060736	0.30909175
chr10	cg01071279	TSS1500	SUPV3L1;SUPV3L1;SUPV3L1;SUPV3L1	0.15769806	0.00060835	0.30909175
chr1	cg01584084	Body;Body		0.19115945	0.00060913	0.30909175
chr21	cg26315964	Body	WRB;WRB;WRB;WRB;WRB;TMPRSS3	0.21246407	0.00060967	0.30909175
chr16	cg15027624	Body;Body	CDK10;CDK10;CDK10	-0.1224952	0.0006099	0.30909175
chr5	cg09032310			0.22437358	0.00061029	0.30909175
chr1	cg25399158	TSS200;Body	INSRR;NTRK1;NTRK1	0.28295558	0.00061039	0.30909175
chr10	cg01850856	Body	FRMD4A;FRMD4A	0.12848772	0.00061047	0.30909175
chr9	cg25605163	0;Body	EGFL7;EGFL7;EGFL7;EGFL7;EGFL7	0.31383798	0.00061083	0.30909175
chr18	cg00994763	Body;Body;Body		0.12554247	0.00061138	0.30909175
chr12	cg17847749			0.17502176	0.00061182	0.30909175
chr3	cg01698108	5'UTR;1stExon	AMP3;LAMP3	0.1962106	0.0006121	0.30909175
chr5	cg09693811			-0.2785292	0.00061268	0.30909175
chr20	cg01585717	TSS1500;TSS1500	EYA2;EYA2	0.18074309	0.00061338	0.30909175
chr3	cg09933638	TSS200	RAB7A;RAB7A;RAB7A	0.21093505	0.00061366	0.30909175
chr6	cg11852643	dy;Body;Body;Body	TCP11;TCP11;TCP11;TCP11;TCP11	0.32456008	0.00061401	0.30909175
chr11	cg14371153	1stExon	NF259;ZNF259;ZNF259	0.1912135	0.00061462	0.30909175
chr4	cg06758191	Body;Body		1.4346754	0.0006149	0.30909175
chr10	cg05199552	1500;TSS1500;5'UTR	CCNY	0.27896997	0.00061495	0.30909175
chr6	cg04573538	Body	DST;DST	0.15483559	0.00061552	0.30909175

chr17	cg18308351	stExon	ALDH3A2;ALDH3A2;ALDH3A2;ALDH3A2;	0.21019491	0.00061559	0.30909175
chr8	cg10324643	5'UTR	RP11-706C16.5;RP11-706C16.5;GML;GML	-0.2681792	0.00061619	0.30909175
chr12	cg18927604		LINC00485	0.24753719	0.00061651	0.30909175
chr17	cg06319579	Body	LINC00324;LINC00324	-0.1288131	0.00061683	0.30909175
chr6	cg18555555	TSS1500	FABP7;FABP7	0.14944441	0.00061695	0.30909175
chr1	cg13153377	TSS200;Body	160H22.5;RP11-160H22.5;RP11-	0.18744051	0.00061707	0.30909175
chr11	cg01952234	0;TSS200;TSS200	AS;WT1;WT1;WT1-AS;WT1-AS;WT1-	-0.3324857	0.00061714	0.30909175
chr1	cg00476580	TSS1500;Body;Body	ANP32E	-0.2343868	0.00061742	0.30909175
chr1	cg13075357		LINC00466;LINC00466	0.18573028	0.00061765	0.30909175
chr5	cg23932859	TSS1500	MEGF10;MEGF10;MEGF10;MEGF10	0.24727976	0.00061787	0.30909175
chr3	cg11283640	Body;Body	NDUFB4;NDUFB4;NDUFB4;NDUFB4	0.1577831	0.00061806	0.30909175
chr15	cg13956907	Body		-0.1336256	0.00061849	0.30909175
chr2	cg27390437	Body	AC106870.2	0.28370685	0.00061934	0.30909175
chr1	cg11073926	stExon;TSS200	;MED8	0.34651486	0.00061944	0.30909175
chr5	cg09601692		CTD-2066L21.3	0.17321089	0.00061952	0.30909175
chr3	cg06196742			0.22795083	0.00062033	0.30909175
chr10	cg27426287	TSS1500	SVIL;SVIL;SVIL	0.22407722	0.00062041	0.30909175
chr8	cg18530175		PRAGMIN	0.1278063	0.00062042	0.30909175
chr21	cg20178606		CHODL;CHODL	-0.1995154	0.00062099	0.30909175
chr15	cg09788125		522B15.4;RP11-522B15.4;RP11-522B15.4	-0.2126982	0.00062141	0.30909175
chr20	cg19079994	0;TSS200	NCOA3;NCOA3;NCOA3;NCOA3	0.21484189	0.00062204	0.30909175
chr14	cg17926940			0.14519096	0.00062205	0.30909175
chr15	cg03953246		SEMA6D;SEMA6D;SEMA6D;SEMA6D	0.18341754	0.00062215	0.30909175
chr11	cg17108838	5'UTR	DM;ADM	0.14955639	0.00062313	0.30936948
chr21	cg21357629		PCBP3;PCBP3;PCBP3;PCBP3;PCBP3	0.24542146	0.00062362	0.30940158
chr4	cg22658311	Body	VEGFC	0.17906253	0.00062442	0.30958498
chr2	cg09554202	TSS200	FZD7	0.1573615	0.00062643	0.31036931
chr11	cg08360511	Body		0.32557278	0.00062753	0.31070153
chr3	cg05683016	Body;Body;Body	SLMAP;SLMAP;SLMAP	0.15978537	0.00062853	0.31077715
chr17	cg19917030	TSS200;TSS200	MYO18A;MYO18A;MYO18A	0.14247876	0.00062854	0.31077715
chr6	cg22258412			-0.1695695	0.00062982	0.31094803
chr5	cg20713816	TSS200	CETN3;CETN3;CETN3;CETN3	0.20912922	0.00062985	0.31094803

chr2	cg07590609			0.29037198	0.00063036	0.31094803
chr4	cg18045701	Body;Body;Body;Body	LDB2;LDB2;LDB2	0.17515541	0.0006306	0.31094803
chr3	cg14716208	Body		0.30623122	0.00063104	0.31095385
chr8	cg22042348			0.19760948	0.00063177	0.31100552
chr16	cg06341397			0.12971085	0.000632	0.31100552
chr17	cg05867823	;5'UTR;TSS1500	PHF23;PHF23;PHF23;PHF23;PHF23;PHF23	0.20268355	0.00063337	0.3112121
chr6	cg26556926	5'UTR;5'UTR	R1;DDR1;DDR1;DDR1;DDR1;DDR1;DDR1;	0.13728093	0.0006334	0.3112121
chr1	cg13685026			0.20513779	0.0006337	0.3112121
chr2	cg22220674		AC098872.3	0.17396509	0.00063445	0.31136874
chr19	cg21953769			-0.1141716	0.00063571	0.31177612
chr1	cg15756091	Body	RGSL1;RGSL1;RGSL1	0.19215434	0.0006373	0.31214489
chr17	cg20132735		RP13-516M14.1	0.20527314	0.00063806	0.31214489
chr11	cg16889189	TSS1500	FXYD6;FXYD6;FXYD6	0.17425942	0.00063815	0.31214489
chr6	cg25799032		RP11-146I2.1	0.24508769	0.00063847	0.31214489
chr17	cg16352624	5'UTR	AXIN2	0.17851947	0.00063863	0.31214489
chr3	cg14055195			0.20312239	0.00063904	0.31214489
chr11	cg16153294	Body;TSS200	19;H19;H19;H19;H19;H19;H19;H19;H19;H19	-0.2094489	0.00064029	0.31254461
chr16	cg06323896			-0.1977255	0.00064125	0.31280338
chr2	cg14715961	5'UTR	V17;MPV17;MPV17;MPV17;MPV17;MPV17	0.25983135	0.00064193	0.31292381
chr13	cg19741032	1500	CHAMP1	0.17557943	0.00064312	0.31297264
chr20	cg22107338			0.1949354	0.00064317	0.31297264
chr1	cg14375345			0.20172738	0.00064333	0.31297264
chr5	cg01027498	1stExon;5'UTR	WDR41;WDR41;WDR41;WDR41;WDR41;	0.16214429	0.00064438	0.31327611
chr6	cg04030141	5'UTR;5'UTR	ATXN1;ATXN1;ATXN1;ATXN1	0.19719511	0.00064628	0.31331945
chr5	cg06176418	Body	AC008592.4;C5orf27	0.19487331	0.00064654	0.31331945
chr17	cg24171114	0	GDPD1	0.1418683	0.00064674	0.31331945
chr2	cg04793821	5'UTR;TSS200;1stExon	;KDM3A;KDM3A;KDM3A;KDM3A;KDM3	0.13883313	0.00064815	0.31331945
chr22	cg21040466	Body	CECR1;CECR3	-0.1208765	0.00064822	0.31331945
chr21	cg18543312	nBnd;ExonBnd;ExonBn	MPRSS3	0.15220386	0.00064826	0.31331945
chr22	cg09646737	Body;Body	X2	0.19855176	0.00064827	0.31331945
chr14	cg16274229	5'UTR	LRFN5;LRFN5;LRFN5	0.2190454	0.00064868	0.31331945
chr3	cg04680635	Body;Body;Body	SOX2-OT;SOX2-OT;SOX2-OT;SOX2-OT	0.18576696	0.00064878	0.31331945

chr3	cg00592903	TSS200	MAP6D1;MAP6D1;MAP6D1;MAP6D1	0.23123549	0.00064925	0.31331945
chr19	cg17968956	TSS1500	ZNF844;ZNF844;ZNF844;ZNF844	-0.1517237	0.00064956	0.31331945
chr14	cg16320779	5'UTR	ZFYVE1;ZFYVE1;ZFYVE1	0.22187387	0.00065003	0.31331945
chr11	cg22294845	TSS200;TSS1500	HC24;ZDHHC24;ZDHHC24;ACTN3;ZDHH	0.13475123	0.00065033	0.31331945
chr19	cg21801535		AC005307.1	0.15471499	0.00065051	0.31331945
chr5	cg10224037	5'UTR	281O15.3	0.13278564	0.00065191	0.31340005
chr10	cg01316983	Body	ACBD7;ACBD7	0.14577688	0.0006526	0.31340005
chr18	cg10423421			0.1752645	0.0006526	0.31340005
chr10	cg00039793	TSS200	N4	0.11724609	0.00065297	0.31340005
chr7	cg23572311	Body	AC004160.4;AC004538.3	-0.3043817	0.00065339	0.31340005
chr6	cg17515297			0.18011667	0.00065376	0.31340005
chr1	cg02664390		RP11-533E19.2	0.59658112	0.00065407	0.31340005
chr9	cg09784369	Body		-0.1599115	0.00065494	0.31340005
chr9	cg00015664	Body;Body	EHMT1;EHMT1	0.20429755	0.00065499	0.31340005
chr10	cg15584954	Body		0.23441802	0.000655	0.31340005
chr19	cg14088357	TSS1500;1stExon	HIF3A;HIF3A;HIF3A;HIF3A;HIF3A;HIF3A	0.32555932	0.00065891	0.31482549
chr12	cg11248646	5'UTR;5'UTR	LIMA1;LIMA1;LIMA1;LIMA1;LIMA1	0.17466259	0.00065893	0.31482549
chr9	cg12154506			0.16780276	0.00065928	0.31482549
chr16	cg13595412			0.17348056	0.00066045	0.31517841
chr5	cg26929490	5'UTR;5'UTR	CTD-2653M23.2;NIPBL;NIPBL	0.1366563	0.00066117	0.31518458
chr17	cg20131685	TSS1500	LLGL1	0.15795832	0.00066133	0.31518458
chr19	cg23350838	TSS1500	SMG9	0.22229519	0.00066196	0.31527794
chr6	cg25663770	TSS1500	NCR2;NCR2;NCR2	0.40493663	0.00066326	0.31550602
chr20	cg06239208	500	TOX2;TOX2;TOX2;TOX2	0.27340613	0.00066349	0.31550602
chr10	cg25719594		RP11-441O15.3	0.15234093	0.0006638	0.31550602
chr19	cg24730871	5'UTR	A;GATAD2A;GATAD2A	0.16660018	0.00066488	0.31550602
chr2	cg10403036	00;Body;TSS200	;SLC5A6;SLC5A6;SLC5A6;SLC5A6;C2orf28	0.25872673	0.00066526	0.31550602
chr6	cg17828223	TSS200;TSS1500	RP1-101K10.6;FBXO5;FBXO5	0.16429433	0.00066602	0.31550602
chr18	cg03313849	Body;Body;Body	TCF4;TCF4	0.24887522	0.00066645	0.31550602
chr3	cg23156391	Body;Body;Body		0.21885392	0.00066654	0.31550602
chr14	cg13531234	TSS1500	872J21.3;PAPOLA;PAPOLA;PAPOLA;PAPO	0.133793	0.00066688	0.31550602
chr10	cg07523098	TSS1500	ARHGAP12;ARHGAP12;ARHGAP12	0.22336813	0.00066699	0.31550602

chr16	cg26995992	TSS1500;Body	P1LC3B;FBXO31;FBXO31;MAP1LC3B;MA	0.17278041	0.00066722	0.31550602
chr6	cg23959612	1500;TSS1500;TSS1500	;CD164;RP11-425D10.10	0.15333307	0.0006683	0.31570069
chr11	cg17879219	00;TSS1500;TSS1500;5'	H1;SUV420H1;SUV420H1;SUV420H1;SUV4	0.23701178	0.00066851	0.31570069
chr4	cg07941983	5'UTR	RP11-431M7.3;ARAP2;ARAP2;ARAP2	0.19323961	0.00066944	0.3157582
chr18	cg05559987	Body;Body		0.15374402	0.0006695	0.3157582
chr8	cg10860677	TSS1500	HHLA1;HHLA1	0.16557581	0.00067063	0.31580657
chr2	cg16910097	Body;Body;Body;Body;	AC009948.3;AC009948.3;AC009948.3	0.17486621	0.00067089	0.31580657
chr12	cg27366072	1500	L2L14;BCL2L14	0.19347088	0.0006715	0.31580657
chr7	cg00455526	1stExon	TRIL;AC005013.5;AC005013.5	0.21373682	0.00067211	0.31580657
chr19	cg14047019	5'UTR	SYT5;SYT5;SYT5;SYT5	0.24071506	0.00067291	0.31580657
chr12	cg05363449		RP11-264F23.3;RP11-264F23.4	0.18612537	0.00067299	0.31580657
chr10	cg20718524			-0.1622373	0.00067299	0.31580657
chr19	cg06127662	TSS200	ZNF45	0.16075402	0.00067378	0.31580657
chr1	cg11844079	TSS200	PIGM	0.19980472	0.0006744	0.31580657
chr19	cg08759924			-0.2030368	0.00067477	0.31580657
chr12	cg08925658	TSS200	35;RP11-1060J15.4;RP11-	0.14965595	0.00067526	0.31580657
chr2	cg18169187	TSS200	AC079922.3;SLC20A1;SLC20A1	0.12973259	0.00067531	0.31580657
chr2	cg23895041	Body	FER1L5;FER1L5;FER1L5;FER1L5	-0.1993507	0.00067623	0.31580657
chr7	cg00429706	TSS200	467H10.2	0.24017336	0.0006763	0.31580657
chr14	cg10212537	0	BTBD7;UNC79;BTBD7;BTBD7;BTBD7	0.16375957	0.00067698	0.31580657
chr1	cg27117650	200	DGF;HDGF;HDGF;HDGF;PRCC	0.16173709	0.00067749	0.31580657
chr1	cg16098952	Body;Body	L;HMGCL	0.13895024	0.0006775	0.31580657
chr1	cg13184886	1stExon	ZDHHC18	0.25143755	0.0006783	0.31580657
chr7	cg05917188	UTR;1stExon;5'UTR	CD36;CD36;CD36;CD36;CD36;CD36;CD36;	0.16124326	0.00067912	0.31580657
chr4	cg13216216	UTR	PARCL1;SPARCL1;SPARCL1;SPARCL1;SP	0.20051557	0.00067923	0.31580657
chr9	cg26430805	TSS200	KLHL9;KLHL9	0.14176629	0.00067992	0.31580657
chr7	cg17945195	5'UTR	7;MCM7;AP4M1;AP4M1;AP4M1;AP4M1;A	0.24199927	0.00068003	0.31580657
chr18	cg00215133			-0.2018072	0.00068016	0.31580657
chr8	cg14020957	1500;TSS1500;TSS1500	CHD7;CHCHD7;CHCHD7;CHCHD7;CHCH	0.32360871	0.00068096	0.31580657
chr20	cg22473095	TSS200	RBBP9;RBBP9;RBBP9;RBBP9	0.19598965	0.00068123	0.31580657
chr15	cg19266912			0.18371249	0.00068179	0.31580657
chr20	cg12680326	1stExon	FAM113A;FAM113A;FAM113A;VPS16;VPS	0.15480809	0.00068206	0.31580657

chr17	cg11318268	TSS1500	AP27	-0.1345634	0.00068207	0.31580657
chr9	cg24239321	0;Body	SUSD1;SUSD1;SUSD1;SUSD1	0.25765663	0.00068332	0.31580657
chr17	cg03938507	TSS1500	2;PDK2;PDK2	0.13097485	0.00068368	0.31580657
chr1	cg21813112	TSS1500;TSS1500	TRIM33	0.1792913	0.0006837	0.31580657
chr6	cg15488272	Body;Body	SYNE1;RP3-398G3.5;SYNE1	0.19786954	0.00068375	0.31580657
chr17	cg16059038	5'UTR;5'UTR;5'UTR	TMEM235;AC087645.3	0.30284304	0.00068428	0.31580657
chr5	cg25373100	1500;TSS1500;TSS1500	PDE8B;PDE8B;PDE8B	0.11372732	0.00068454	0.31580657
chr20	cg18598695		RP1-66N13.1	-0.3920813	0.0006849	0.31580657
chr1	cg15225132	TSS200	OPN3;OPN3;OPN3;OPN3;OPN3;OPN3	0.14645489	0.0006854	0.31580657
chr1	cg11202686		RP5-1180C18.1	0.19327637	0.0006858	0.31580657
chr12	cg00577905	Body	DUSP16	0.14949307	0.00068656	0.31580657
chr22	cg21832731	TSS200	C22orf32;C22orf32;C22orf32;C22orf32	0.14199774	0.00068731	0.31580657
chr18	cg02281238	Body	DYM	0.12580571	0.00068733	0.31580657
chr15	cg19563839	Body	RP11-650L12.1;IREB2;IREB2	0.1853385	0.00068754	0.31580657
chr19	cg24117192	Body;Body;Body		-0.2220489	0.00068797	0.31580657
chr7	cg14521995		AC006372.4	-0.2488296	0.00068831	0.31580657
chr3	cg03555651	ExonBnd;Body	ACVR2B;ACVR2B;ACVR2B	0.17054811	0.00068919	0.31590127
chr10	cg17440757	Body;Body	MSMB;MSMB	-0.1767657	0.00068979	0.31590127
chr14	cg01700524	Body	DCY4;ADCY4	0.27987943	0.00069008	0.31590127
chr6	cg03341723	TSS1500;Body	RING1;MIR219-1	0.2432486	0.00069026	0.31590127
chr16	cg12294213	Body	RP11-473I1.9	-0.1617763	0.00069183	0.31635771
chr7	cg24265806	1stExon;5'UTR	FAM126A;FAM126A;FAM126A;FAM126A	0.18315258	0.00069239	0.31635771
chr10	cg06403921	Body		-0.219508	0.00069256	0.31635771
chr19	cg00746693	5'UTR	KIAA0355	0.25040433	0.00069331	0.31649903
chr1	cg09036220	00;TSS1500	;ZBTB8OS;ZBTB8OS;ZBTB8OS;ZBTB8OS;	0.16593485	0.00069556	0.31732728
chr14	cg05196610			0.18114246	0.00069691	0.31734572
chr8	cg07101082	Body	NCOA2	0.18324406	0.00069716	0.31734572
chr5	cg26534871			0.20674174	0.00069751	0.31734572
chr1	cg26601289	;5'UTR	NF669;ZNF669;ZNF669;ZNF669;ZNF669	0.2436688	0.00069753	0.31734572
chr4	cg17637556	TSS1500;TSS1500	RGL;PACRGL;PACRGL;PACRGL;PACRGL	0.23419562	0.00069778	0.31734572
chr3	cg27601014			0.18101679	0.00070013	0.31815896
chr20	cg07098747	Body;Body	BTBD3;BTBD3;BTBD3;BTBD3;BTBD3	0.1697821	0.00070045	0.31815896

chr15	cg08808106			0.18484687	0.00070145	0.31841267
chr10	cg12782605			0.14957186	0.00070264	0.31845566
chr21	cg01148198	1500;TSS1500;TSS1500	9.2	0.1938435	0.000703	0.31845566
chr4	cg15463457	Body	MMRN1	0.28517076	0.00070326	0.31845566
chr12	cg25695610		RP11-644F5.16	0.58572229	0.0007033	0.31845566
chr15	cg07824606			0.16318074	0.00070388	0.31851975
chr3	cg24615528	TSS200	AMP3	0.19956934	0.00070461	0.3185969
chr1	cg05667744			0.18823197	0.00070532	0.3185969
chr14	cg17918285	Body	C14orf182;C14orf182;C14orf182;C14orf182	0.13835011	0.00070536	0.3185969
chr12	cg22875723			0.17730367	0.00070584	0.31861436
chr13	cg02530656			0.17962184	0.00070745	0.31896014
chr11	cg12715569			0.49174643	0.00070973	0.31896014
chr8	cg12044295	n	MEM68;TGS1;TGS1;TMEM68;TMEM68;T	0.15175498	0.00071093	0.31896014
chr8	cg14041100	5'UTR	CHD7;CHD7;CHD7	0.17998682	0.00071112	0.31896014
chr8	cg14855657	Body	CTD-2135J3.3;CTD-2135J3.3	0.26049537	0.00071164	0.31896014
chr19	cg16695634	Body		-0.1378087	0.00071169	0.31896014
chr2	cg05702927	5'UTR;Body;Body;Body	ABI2;ABI2;ABI2	-0.2617205	0.00071202	0.31896014
chr19	cg07950158	TSS200;TSS200	C2	0.2224053	0.00071351	0.31896014
chr8	cg21926091	Body;Body	TRAPPC9;TRAPPC9;TRAPPC9	0.21672807	0.00071412	0.31896014
chr19	cg12522311	Body		-0.1609077	0.00071418	0.31896014
chr2	cg11518826	Body		0.17355412	0.00071486	0.31896014
chr12	cg07847038	TSS200	RDM4	0.13960174	0.00071488	0.31896014
chr19	cg21144398			0.30404982	0.00071519	0.31896014
chr20	cg03404493	Body	CDS2;CDS2;CDS2	0.14904812	0.00071609	0.31896014
chr16	cg00243514			0.16165523	0.00071823	0.31896014
chr17	cg06942197	1stExon;5'UTR	1;SMARCE1;SMARCE1;SMARCE1;SMARC	0.25474142	0.00071824	0.31896014
chr8	cg15646900	1stExon;5'UTR	ATP6V1H;ATP6V1H;ATP6V1H;ATP6V1H;	0.21268278	0.00071906	0.31896014
chr13	cg08866608			0.19048075	0.00071971	0.31896014
chr2	cg18347209			0.2224586	0.00071974	0.31896014
chr1	cg00280564	Body		0.49030584	0.00072003	0.31896014
chr6	cg07279933	1stExon	RSPO3;RSPO3;RSPO3;RP11-73O6.4	0.23789527	0.00072079	0.31896014
chr13	cg11744351	1stExon;5'UTR	UBL3;UBL3	-0.1624681	0.00072141	0.31896014

chr14	cg21804531	Body	FCAB11;RP11-33N16.3	0.14664841	0.00072144	0.31896014
chr17	cg00644007	Body		-0.1283402	0.0007218	0.31896014
chr14	cg01093245		RKCH	0.21153999	0.00072192	0.31896014
chr18	cg06614469	TSS200	MAPK4;MAPK4	0.16138752	0.00072196	0.31896014
chr1	cg07123038	Body	SLC45A1	0.20066802	0.00072245	0.31896014
chr13	cg09511878			0.16275377	0.00072267	0.31896014
chr19	cg18569243			0.17484732	0.00072347	0.31896014
chr21	cg07544832			0.18314704	0.00072371	0.31896014
chr11	cg08683012	Body		-0.2661647	0.00072377	0.31896014
chr2	cg19486482	TSS200	3.1	0.22300402	0.00072451	0.31896014
chr5	cg13222558	0	CCNI2	0.2649504	0.00072452	0.31896014
chr6	cg06993041	TSS200	TUBB;TUBB;TUBB;TUBB;TUBB	0.14114606	0.00072501	0.31896014
chr13	cg11199428	0	AS1;INTS6;INTS6;INTS6;INTS6;INTS6;INT	0.19087409	0.00072576	0.31896014
chr3	cg24494008	5'UTR;TSS1500	C4;SMC4;SMC4;SMC4;SMC4;SMC4;IFT80;	0.21161042	0.00072639	0.31896014
chr1	cg04912542			0.15958496	0.00072646	0.31896014
chr20	cg07300076	TSS1500	TMC2	0.21532852	0.0007269	0.31896014
chr19	cg26952796		NR1H2	0.27358012	0.00072708	0.31896014
chr12	cg04601886			0.22247983	0.00072727	0.31896014
chr15	cg03326689	TSS1500	MYO5C	0.17614968	0.00072731	0.31896014
chr4	cg23195012	1stExon;5'UTR	AIP;CDKN2AIP;CDKN2AIP;CDKN2AIP;CD	0.1046993	0.00072788	0.31896014
chr4	cg18177533	TSS200	KDR;KDR	0.2439678	0.0007284	0.31896014
chr20	cg12600163	Body;Body;TSS200	A20;NAA20	0.13707997	0.0007288	0.31896014
chr15	cg02015934		CHRNB4;RP11-160C18.2	0.17332597	0.00072896	0.31896014
chr9	cg21361433	TSS200	SNX30;RP11-276E15.4	0.16813482	0.00072904	0.31896014
chr12	cg18682447	TSS200	637A17.2;NUP107;NUP107;NUP107;NUP10	0.17278652	0.00072909	0.31896014
chr13	cg19694453			0.17345234	0.00072957	0.31896014
chr15	cg00229508	3'UTR	ODF3L1	0.14117911	0.00072974	0.31896014
chr8	cg25710809	TSS1500;TSS1500	GNRH1;GNRH1	-0.2238316	0.00073018	0.31896014
chr22	cg14577628		IGLV4-3	-0.1186356	0.00073025	0.31896014
chr22	cg10927536	TSS1500;Body	HPS4;HPS4;HPS4;HPS4;HPS4;HPS4;HPS4	0.1825341	0.00073049	0.31896014
chr12	cg09878554	TSS200;TSS200	D1;SMARCD1;SMARCD1;SMARCD1;SMA	0.17183122	0.00073067	0.31896014
chr1	cg16483318	Body;Body	TPM3	0.21125542	0.00073111	0.31896014

chr3	cg07284460	Body	KAT2B	0.18272803	0.00073115	0.31896014
chr2	cg02961397		PTH2R	0.15807297	0.00073255	0.31896014
chr21	cg09906747	00;1stExon;5'UTR;5'UT	ART;GART;GART;GART;GART;SON;SON;	0.1067337	0.00073301	0.31896014
chr6	cg22580625	5'UTR;1stExon	POPDC3;POPDC3;POPDC3	0.13677197	0.00073327	0.31896014
chr1	cg11214739	TSS1500	NEK7;NEK7;NEK7;NEK7	0.14832009	0.00073329	0.31896014
chr21	cg26861066	TSS200	;HMGN1;HMGN1;HMGN1;HMGN1;HMGN	0.25874807	0.00073341	0.31896014
chr11	cg18493947	Body	OPCML	0.17915409	0.00073363	0.31896014
chr3	cg20105734	Body	TMEM212;TMEM212-AS1	0.16161417	0.00073495	0.31896014
chr4	cg23560727	TSS200	PGM2;PGM2;PGM2;PGM2	0.15373842	0.00073585	0.31896014
chr8	cg16423305			0.35036303	0.00073615	0.31896014
chr2	cg01109219	TSS1500	RASGRP3;RASGRP3;RASGRP3;RASGRP3	0.1640323	0.00073657	0.31896014
chr19	cg17519161	TSS200	PBX4;PBX4;PBX4;PBX4;PBX4;PBX4	0.26029586	0.00073817	0.31896014
chr4	cg06499344	5'UTR	431M7.3;ARAP2;ARAP2;ARAP2;ARAP2;A	0.14808863	0.00073832	0.31896014
chr4	cg10627377	TSS200	TKTL2	0.28398466	0.0007397	0.31896014
chr15	cg15738154	Body	RORA;RORA;RORA	-0.2214139	0.00074005	0.31896014
chr16	cg14815767		RP11-152O14.5;RP11-152O14.5	0.15955845	0.00074037	0.31896014
chr5	cg17751746	1stExon;5'UTR	CYSTM1;CYSTM1;CYSTM1;CYSTM1	0.12032025	0.00074045	0.31896014
chr5	cg19971650	5'UTR;5'UTR	RB2;GABRB2;GABRA6	0.15302993	0.0007408	0.31896014
chr17	cg19252218	Body		-0.1499788	0.00074119	0.31896014
chr15	cg25584728	Body		0.1829559	0.00074119	0.31896014
chr11	cg14855425	00;TSS1500	SMD13;PSMD13;PSMD13;PSMD13;PSMD1	0.15751638	0.0007413	0.31896014
chr5	cg01870619	Body	ZFR;ZFR	0.23754318	0.00074165	0.31896014
chr3	cg12782834	0;5'UTR;5'UTR	;PIGX;PIGX;PIGX;PIGX;PIGX;PIGX	0.13481129	0.00074184	0.31896014
chr5	cg26485174			0.13389315	0.000742	0.31896014
chr6	cg06670368	Body		0.1806265	0.00074214	0.31896014
chr15	cg01021488	Body	IGF1R;IGF1R;IGF1R;IGF1R	0.27035643	0.00074253	0.31896014
chr3	cg03959131	Body	85M11.2	0.17059956	0.00074297	0.31896014
chr10	cg24418792	TSS1500;TSS1500	NFKB2;NFKB2	0.15494804	0.00074308	0.31896014
chr8	cg03860720	Body;Body	LRP12;LRP12;LRP12	0.14394074	0.00074402	0.31896014
chr14	cg07580718			-0.2681291	0.00074411	0.31896014
chr20	cg02319485	5'UTR;ExonBnd	;PCK1;PCK1	0.14710576	0.00074483	0.31896014
chr10	cg06466203	5'UTR;TSS200;1stExon	KLLN;PTEN;PTEN;PTEN;PTEN	0.11417145	0.00074505	0.31896014

chr19	cg01885869	Body;Body	ZNF497	0.19608767	0.00074526	0.31896014
chr3	cg25195477	TSS1500	HPS3;HPS3;HPS3;HPS3	0.23667448	0.00074555	0.31896014
chr2	cg24171337	5'UTR;5'UTR	AC012360.4;TGFBRAP1	0.35572864	0.00074572	0.31896014
chr2	cg04221848	Body;Body;Body	U6;NCKAP5	0.28282853	0.00074647	0.319076
chr1	cg13441112			0.21969658	0.00074711	0.319076
chr5	cg25935985	Body;Body	FLT4;FLT4;FLT4;FLT4	0.19046976	0.0007474	0.319076
chr8	cg08117032	UTR;5'UTR;5'UTR;5'U	1;FGFR1;FGFR1;FGFR1;FGFR1;FGFR1;FGF	0.24583861	0.00074775	0.319076
chr12	cg19119284	Body	C12orf51;C12orf51	-0.2162074	0.00074881	0.31934289
chr13	cg04728164	Body	RP11-501G7.1	0.19603433	0.00074926	0.3193459
chr2	cg14856316			0.1609597	0.00075015	0.31953813
chr8	cg10221617	Body;5'UTR	AS1;HAS2;HAS2	0.18295578	0.00075098	0.31963117
chr5	cg22212250	Body	AFF4	0.17343823	0.00075214	0.31963117
chr11	cg24615831	Body	PTS	0.13015609	0.00075224	0.31963117
chr1	cg00067518	Body	CAMTA1	0.20093252	0.00075226	0.31963117
chr6	cg14663792	TSS200;1stExon	6orf48;C6orf48;C6orf48;C6orf48;SNORD48;	0.2616992	0.0007528	0.31963117
chr17	cg03727280	TSS200;TSS200	F2;ATPAF2;ATPAF2;ATPAF2;ATPAF2	0.24173588	0.00075301	0.31963117
chr18	cg01047297			0.37598727	0.00075398	0.31967038
chr16	cg11664969	TSS1500	CTRB2;CTRB2	-0.2101386	0.00075398	0.31967038
chr15	cg01328621		RP11-315D16.4	0.15739283	0.00075519	0.31992057
chr18	cg14071396			0.1963402	0.00075582	0.31992057
chr7	cg03062264	Body;Body;Body		-0.2202516	0.00075632	0.31992057
chr2	cg21563597	0	EPT10;SEPT10;SOWAHC;SEPT10;SEPT10;	0.13517171	0.00075736	0.31992057
chr17	cg03776864	Body;Body	2047H16.4	-0.1864892	0.00075797	0.31992057
chr12	cg21044607	5'UTR;5'UTR;TSS200	C12orf53;C12orf53;C12orf53;C12orf53	0.18831394	0.000758	0.31992057
chr7	cg20518511	Body	AC093620.5;AC092171.2;TNRC18;TNRC18	-0.141651	0.00075833	0.31992057
chr2	cg10908528	3'UTR;Body;Body	GALNT13;AC009227.2;GALNT13	0.20575559	0.00075838	0.31992057
chr7	cg20769356	Body	AC006378.2	0.28015573	0.00075979	0.31992057
chr12	cg13334990	1stExon;5'UTR	O2;ENO2;ENO2;ENO2;ENO2;ENO2;ENO2;	0.23479744	0.00075991	0.31992057
chr3	cg15939396	Body;Body	CD96	0.16249773	0.00076074	0.31992057
chr17	cg25389328	5'UTR;5'UTR	THRA;THRA;THRA	-0.1004575	0.00076133	0.31992057
chr22	cg22896904	1stExon;5'UTR	4;ANKRD54;ANKRD54;ANKRD54;ANKRD	0.15553779	0.00076162	0.31992057
chr19	cg15649193	5'UTR;Body;1stExon	TECR;DNAJB1;MIR639;TECR;TECR	0.15035322	0.00076171	0.31992057

chr18	cg09317221	00;Body;Body	ANKRD12;ANKRD12;ANKRD12	0.17613194	0.00076211	0.31992057
chr22	cg14405528	Body	TBC1D22A;TBC1D22A	-0.2229132	0.00076288	0.31992057
chr16	cg00550616			-0.2165732	0.00076323	0.31992057
chr4	cg10813306	1stExon;5'UTR	RP11-556G22.2;KCNIP4;KCNIP4	0.27396381	0.00076335	0.31992057
chr6	cg18417237	5'UTR	LRFN2	-0.1313582	0.00076345	0.31992057
chr14	cg04408225	0	AS1;DICER1;DICER1;DICER1;DICER1;DIC	0.29246899	0.00076419	0.31992057
chr17	cg08949641	Body;5'UTR	RAI1-AS1;RAI1	-0.1161259	0.00076468	0.31992057
chr8	cg04046119	TSS200;Body;Body	OXR1;OXR1	0.44876477	0.00076572	0.31992057
chr12	cg12048508			0.21041868	0.00076575	0.31992057
chr19	cg13971552	Body	TYK2;TYK2;TYK2;TYK2;TYK2;TYK2	-0.1497103	0.00076594	0.31992057
chr3	cg01929138	Body		0.23469667	0.00076649	0.31992057
chr16	cg10572037	TSS1500	991G20.1;AC004158.2;AC004158.2;RP5-	0.1878194	0.00076656	0.31992057
chr17	cg02397114	TSS200	TMEM102;TMEM102;RP11-104H15.7	0.1463005	0.00076668	0.31992057
chr10	cg03316881		MAPK8;MAPK8;MAPK8;MAPK8;MAPK8	0.29199474	0.00076691	0.31992057
chr13	cg03583571	TSS1500	PCDH20;PCDH20;PCDH20	0.14352763	0.00076875	0.3202522
chr1	cg23260484	1500;TSS1500;TSS1500	B2;EFCAB2	0.17349957	0.00076892	0.3202522
chr17	cg21197711	Body		-0.1208386	0.00076908	0.3202522
chr14	cg07001734	5'UTR;TSS1500	;TECPR2;TECPR2;TECPR2;TECPR2	0.31775134	0.00076952	0.3202522
chr10	cg10537220	0	ZNF365;ZNF365	0.32544185	0.00076992	0.3202522
chr19	cg02546618	TSS1500;TSS200	MAU2;MAU2;SUGP1	0.1968778	0.00077061	0.32035879
chr2	cg03448964	Body;Body;Body;Body	SLC30A6;SLC30A6;U6	-0.2374216	0.00077294	0.32110304
chr2	cg16119667	TSS200	ZNF514;ZNF514	0.41877066	0.00077329	0.32110304
chr12	cg06134974	TSS1500	P2;CSRNP2;CSRNP2	0.30465844	0.00077502	0.32113543
chr12	cg03620561			0.16248258	0.00077524	0.32113543
chr5	cg15997127	TSS1500	CDH6;CDH6;RP11-152K4.2	0.18702569	0.00077558	0.32113543
chr7	cg21088488	TSS1500	DBNL;PGAM2	-0.2408772	0.00077743	0.32113543
chr1	cg27391445	d;ExonBnd;ExonBnd;Ex	DISC1;DISC1;DISC1;DISC1;DISC1	0.15807415	0.00077764	0.32113543
chr6	cg24850577	;5'UTR	BP;SKIV2L;RDBP;RDBP;SKIV2L;RDBP;SK	0.11537025	0.00077786	0.32113543
chr3	cg12741295	Body		0.22768767	0.00077798	0.32113543
chr2	cg04911335			0.17284638	0.00077844	0.32113543
chr2	cg26894435	5'UTR;1stExon	BMPR2;BMPR2;BMPR2	0.24360586	0.00077846	0.32113543
chr7	cg03464573	TSS1500	170O19.21;HOXA9;HOXA9;HOXA9;RP1-	0.23527822	0.00077881	0.32113543

chr15	cg21494626	Body	SMAD3;SMAD3;SMAD3;SMAD3	0.17581354	0.00077918	0.32113543
chr6	cg09472616	n;1stExon;5'UTR;5'UTR	;REPS1;REPS1;REPS1;REPS1;REPS1;REPS	0.35145452	0.00077943	0.32113543
chr16	cg26844180			0.26332902	0.00077956	0.32113543
chr6	cg13995568			0.16311984	0.00077959	0.32113543
chr9	cg01177235	stExon	CHMP5;BAG1;BAG1	0.20506266	0.00078003	0.32113543
chr13	cg01129320	5'UTR	NALCN;NALCN;NALCN;NALCN	0.16754865	0.00078045	0.32113543
chr1	cg18590590	n	LRRC42;LRRC42;LRRC42;HSPB11;HSPB11	0.12808569	0.00078252	0.32139846
chr11	cg08941721	stExon;1stExon	2530H12.1;DGAT2;DGAT2;DGAT2;DGAT2;	0.15490437	0.00078281	0.32139846
chr8	cg05745720			-0.2986187	0.00078487	0.32139846
chr11	cg07110981		AP000619.5;RP11-725K16.4	0.12633344	0.00078591	0.32139846
chr9	cg13482649			-0.2119001	0.00078593	0.32139846
chr11	cg01356919	Body;Body;Body;Body	HTR3A;HTR3A	0.23965993	0.00078597	0.32139846
chr22	cg23762037	TSS200	TUBGCP6;TUBGCP6;TUBGCP6	0.13276074	0.00078625	0.32139846
chr6	cg08126875			-0.2033166	0.0007864	0.32139846
chr7	cg02797569	1stExon;5'UTR	530H6.2;PCOLCE;PCOLCE;PCOLCE;PCOL	-0.1645507	0.00078725	0.32139846
chr20	cg23854747	Body	SAMD10;SAMD10;ZNF512B;ZNF512B	0.31414958	0.00078798	0.32139846
chr2	cg23118151	5'UTR;5'UTR	MFSD2B;C2orf44;C2orf44;C2orf44	0.18264854	0.00078826	0.32139846
chr22	cg12958821			0.28783246	0.00078931	0.32139846
chr2	cg05975120	;5'UTR	BMS1	0.14131775	0.00078944	0.32139846
chr4	cg03573788			0.15457601	0.00078965	0.32139846
chr4	cg16123062	Body	SH3RF1	0.1796044	0.00078999	0.32139846
chr1	cg11485605			-0.2951533	0.00079048	0.32139846
chr3	cg05791779	Body	CHCHD6	-0.1166844	0.00079062	0.32139846
chr16	cg17545418	Body		-0.2403563	0.00079126	0.32139846
chr6	cg09708616			-0.2090156	0.00079136	0.32139846
chr19	cg25932329	Body;5'UTR	DDX39A;DDX39A;DDX39A	-0.0959077	0.00079158	0.32139846
chr13	cg20259674	TSS200	SPRY2;SPRY2;SPRY2	0.31905914	0.00079165	0.32139846
chr21	cg09443102	TSS200	COL18A1;COL18A1	0.24876216	0.00079295	0.32139846
chr1	cg05364361	Body;Body;Body	SSBP3;SSBP3;SSBP3;SSBP3	-0.0873205	0.00079298	0.32139846
chr7	cg16450683	TSS1500	TAS2R4;SSBP1	0.14817134	0.00079311	0.32139846
chr8	cg12847382	Body	RP11-32K4.1;RP11-32K4.1;RP11-32K4.1	0.19687697	0.0007935	0.32139846
chr7	cg10207609	R	CD36;CD36;CD36;CD36	0.19733489	0.00079529	0.32139846

chr18	cg21715088			0.14994002	0.00079549	0.32139846
chr12	cg22867594	TSS200	BRI3BP	0.35197542	0.00079582	0.32139846
chr17	cg24881334			0.17748677	0.00079612	0.32139846
chr21	cg22356087	Body	DXK;PDXK	-0.1545076	0.00079651	0.32139846
chr17	cg15963109	Body	CTC-524C5.5	0.1447861	0.00079669	0.32139846
chr4	cg14702007	Body;Body	SNORA24	0.17993699	0.00079716	0.32139846
chr2	cg09453525			0.15562638	0.0007972	0.32139846
chr2	cg05543972			-0.1253231	0.00079729	0.32139846
chr19	cg14132357	Body		0.24469144	0.00079788	0.32139846
chr10	cg24134845	Body;Body;Body;Body		0.16413443	0.00079833	0.32139846
chr4	cg14311957	Body;Body;Body	U7	0.19004783	0.00079889	0.32139846
chr8	cg24375476	Body	DF3;YTHDF3;YTHDF3;YTHDF3;YTHDF3;	0.15535023	0.00079891	0.32139846
chr22	cg09454509	TSS1500	DNAL4;SUN2;DNAL4;DNAL4;DNAL4	0.19889496	0.00079893	0.32139846
chr12	cg12287194	Body	FOXN4;FOXN4	0.18402548	0.00079895	0.32139846
chr19	cg08278357			0.20934016	0.00079924	0.32139846
chr11	cg10067059			0.17491861	0.00080232	0.32233228
chr1	cg06617136	TSS1500;5'UTR	L	0.25127382	0.00080279	0.32233228
chr2	cg09758039	Body;Body;Body;Body	IQCA1;IQCA1;IQCA1;IQCA1;IQCA1	0.19285362	0.00080289	0.32233228
chr17	cg08729407	Body		0.14504985	0.00080351	0.32235856
chr3	cg17560006	TSS200;TSS200	RYK;RYK;RYK	0.12929089	0.0008045	0.32235856
chr3	cg19394866			0.14968828	0.0008045	0.32235856
chr6	cg00721468	1stExon;1stExon	EF1E1	0.14791789	0.00080486	0.32235856
chr20	cg06309063	TSS1500;TSS200	DPM1;DPM1	0.16627666	0.00080518	0.32235856
chr19	cg06332269	Body	CSNK1G2	-0.1749854	0.00080639	0.32242196
chr2	cg08466051	TSS200	TTC21B;TTC21B;TTC21B	0.26993378	0.00080738	0.32242196
chr8	cg02431866	Body		0.19483204	0.00080756	0.32242196
chr3	cg05209917	5'UTR;1stExon	TN6;CNTN6;CNTN6;CNTN6;CNTN6;CNTN	0.15746208	0.00080767	0.32242196
chr1	cg00677945	0	IL28RA;IL28RA;IL28RA	0.13790628	0.00080782	0.32242196
chr1	cg15063322	Body;1stExon	C1orf204;C1orf204	0.12085421	0.00080883	0.32242196
chr20	cg11659435	TSS200;TSS200	BCL2L1;BCL2L1;BCL2L1;BCL2L1;BCL2L1;	0.1879969	0.00080886	0.32242196
chr14	cg19530576	R;5'UTR;TSS1500;1stE	3;CCNK;CCNK;CCNK;CCNK;CCNK;CCNK	0.13121572	0.00080919	0.32242196
chr12	cg06955856	5'UTR;5'UTR	11L2;TCP11L2;TCP11L2;TCP11L2;CKAP4;	-0.1161206	0.00080934	0.32242196

chr6	cg24579212	TSS200;1stExon;5'UTR	TUBE1;C6orf225;TUBE1;C6orf225	0.17747059	0.00081031	0.3226351
chr18	cg27166860	UTR	TTC39C;TTC39C;TTC39C	0.34233297	0.00081082	0.32265865
chr1	cg17034181	Body;Body	EXTL2	-0.2320298	0.00081173	0.32269167
chr1	cg20848343			-0.1407041	0.00081294	0.32269167
chr10	cg12275661	0	462L8.1;ITGB1;ITGB1;ITGB1;ITGB1;ITGB1	0.12229672	0.00081295	0.32269167
chr1	cg07375700	Body;5'UTR	KCZ;PRKCZ	-0.169712	0.00081329	0.32269167
chr16	cg06186041	3'UTR;3'UTR	473M20.14	-0.2108477	0.00081357	0.32269167
chr17	cg07511128	Body;Body		0.14458947	0.00081357	0.32269167
chr12	cg14879241			-0.1658189	0.00081429	0.32280001
chr22	cg04517696			-0.123265	0.00081565	0.32316207
chr5	cg27628536		2035E11.3;CTD-2035E11.3;CTD-	-0.2175429	0.00081626	0.32322898
chr3	cg21920693	5'UTR	MF1	0.18246694	0.00081678	0.32325998
chr4	cg19918862		MFAP3L	0.15520926	0.0008175	0.32336828
chr1	cg01005080	TSS1500	LHX8	-0.2354543	0.00081811	0.32343179
chr19	cg11205703	Body		-0.1321274	0.0008197	0.32366799
chr2	cg06226973	Body;TSS1500	RKRA;PRKRA;PRKRA;PRKRA;PRKRA;PR	0.15413128	0.00082068	0.32366799
chr5	cg17719133		CTB-57H20.1	0.1563404	0.0008207	0.32366799
chr3	cg18635791	3'UTR;3'UTR	IL5RA;IL5RA;IL5RA;IL5RA	0.15394965	0.00082115	0.32366799
chr10	cg25228510	TSS200;1stExon;5'UTR;	K1	0.17747318	0.00082145	0.32366799
chr14	cg24889553			0.28910893	0.00082188	0.32366799
chr8	cg03298141		RP11-103H7.2	0.17490417	0.00082241	0.32366799
chr16	cg00848693	Body	TERF2;TERF2	-0.2306611	0.00082269	0.32366799
chr1	cg13448816		LRRC7;LRRC7	0.17826445	0.00082272	0.32366799
chr19	cg03345360	1stExon;5'UTR	PGPEP1;PGPEP1;PGPEP1	0.10765956	0.00082478	0.32417508
chr8	cg08431549			0.14693761	0.00082509	0.32417508
chr6	cg18364779	1stExon	HIST1H4C	0.18964322	0.00082535	0.32417508
chr1	cg26343154	Body	MIIP;MIIP;MIIP;MIIP;MIIP	-0.1698448	0.00082597	0.32424367
chr17	cg23465990	TSS1500	64A;FAM64A;FAM64A;FAM64A;FAM64A	0.13258352	0.00082647	0.32426419
chr13	cg21228103			0.12267725	0.00082813	0.32440935
chr4	cg11967108	Body		0.20965687	0.00082833	0.32440935
chr20	cg23380554	n;Body	;ROMO1;NFS1;NFS1;NFS1;NFS1;NFS1;NFS	0.20800211	0.00082844	0.32440935
chr5	cg11715789	Body;Body;Body	RUFY1;RUFY1;RUFY1	-0.2047534	0.00082892	0.32440935

chr18	cg16529095	Body		0.16195058	0.0008294	0.32440935
chr6	cg13456812	Body	RNASET2;RNASET2	0.28078666	0.00082981	0.32440935
chr5	cg03753340	TSS1500	KDM3B	0.14893545	0.00082997	0.32440935
chr2	cg07526221	Body	VR2A	0.15487062	0.0008308	0.32443862
chr5	cg24117063	Body		-0.2432711	0.00083094	0.32443862
chr11	cg23624401	TSS200	UCP2;UCP2;UCP2;UCP2	0.1412556	0.00083254	0.32452351
chr11	cg06618666	TSS1500	MMP3;MMP3	0.21144881	0.00083258	0.32452351
chr4	cg18630337			0.138375	0.00083305	0.32452351
chr3	cg01292094			0.19864102	0.00083389	0.32452351
chr8	cg08819647	5'UTR;5'UTR	P;PHYHIP;PHYHIP	-0.2442647	0.00083509	0.32452351
chr11	cg09494418		MS4A6E	0.19632287	0.00083574	0.32452351
chr10	cg22735222	3'UTR	NKX2-3	0.26980019	0.00083601	0.32452351
chr22	cg19737037	1stExon	TNFRSF13C	0.1473001	0.00083629	0.32452351
chr6	cg24298280	5'UTR	RNF217;RNF217;RNF217;RNF217	0.19507806	0.00083794	0.32452351
chr7	cg10763891		GNGT1	0.15834985	0.00083817	0.32452351
chr13	cg11207455			0.21183561	0.00083894	0.32452351
chr5	cg11059451	TSS200;TSS200	DNAJC21	0.14860023	0.00083895	0.32452351
chr14	cg06830503	TSS200	ARG2;ARG2;ARG2;ARG2	0.2579259	0.00083896	0.32452351
chr22	cg12318501	TSS1500	ADM2;ADM2;ADM2	0.35596013	0.00083923	0.32452351
chr16	cg10189135	TSS1500	D1;ATP6V0D1;ATP6V0D1;ATP6V0D1;ATP	-0.1667311	0.00083961	0.32452351
chr12	cg07935482	Body;Body;Body;Body	2;YAF2;YAF2;YAF2;YAF2	0.20261831	0.00083978	0.32452351
chr12	cg02510471	Body	HOTAIR;HOTAIR	0.23522425	0.00084037	0.32452351
chr8	cg19242560	0	DYHV1;WDYHV1;WDYHV1;WDYHV1;W	0.18720846	0.0008405	0.32452351
chr2	cg01585758	Body;Body;Body	SUMO1;SUMO1	0.21826417	0.00084083	0.32452351
chr14	cg09934925			0.16983272	0.00084253	0.32452351
chr14	cg00215204	Body;Body;Body;Body		-0.1135257	0.00084345	0.32452351
chr13	cg24820672		RP11-315H15.1	-0.7876817	0.0008439	0.32452351
chr16	cg22050642	5'UTR;5'UTR;Body	ZNF778;ZNF778;ZNF778;AC009113.1	0.1664276	0.0008443	0.32452351
chr10	cg07352001	TSS200;TSS1500	ZNF32;ZNF32;ZNF32;ZNF32-AS3	0.1873327	0.0008445	0.32452351
chr1	cg25466090	5'UTR;Body;5'UTR	KCZ;PRKCZ;PRKCZ;PRKCZ;PRKCZ;PRKC	0.16387531	0.00084523	0.32452351
chr6	cg11192767	5'UTR;Body;1stExon	RRT1;PRRT1;PPT2;PPT2;PPT2;PPT2;PPT2;	0.24437241	0.00084553	0.32452351
chr18	cg02832905			0.23681687	0.0008458	0.32452351

chr19	cg06666157	TSS200	FEM1A	0.20096585	0.00084635	0.32452351
chr20	cg18685299			0.20283072	0.00084728	0.32452351
chr5	cg01946760	5'UTR;1stExon	N4BP3;N4BP3	0.27337973	0.0008483	0.32452351
chr6	cg12217308	Body;Body;Body	AL035697.1	0.21137389	0.00084896	0.32452351
chr6	cg05292460	Body		-0.2435559	0.00084922	0.32452351
chr6	cg20211088	TSS1500	RANBP9;RANBP9	0.13419193	0.00084939	0.32452351
chr22	cg20897301	Body	MYH9;MYH9;MYH9	-0.1308363	0.00085067	0.32452351
chr16	cg17681277	TSS200	2A;TNFRSF12A;TNFRSF12A;TNFRSF12A;	0.28833821	0.0008511	0.32452351
chr22	cg08270630			-0.1030426	0.00085136	0.32452351
chr7	cg06183123		AC009365.3	0.22894843	0.00085232	0.32452351
chr7	cg23270617	TSS200	12;CLDN12;CLDN12;CLDN12;CLDN12;CL	0.17696006	0.00085305	0.32452351
chr14	cg18867321	TSS200	ZFYVE1;ZFYVE1;ZFYVE1	0.11943593	0.00085319	0.32452351
chr11	cg14040984	Body;Body;Body	B1;PHLDB1;PHLDB1;PHLDB1;PHLDB1;PH	0.15568507	0.00085338	0.32452351
chr3	cg07704136	TSS1500	AS1;PVRL3;PVRL3-AS1;PVRL3-	0.15770544	0.00085358	0.32452351
chr18	cg22067914	1stExon;5'UTR	TWSG1;TWSG1	0.23528831	0.00085402	0.32452351
chr14	cg22955551		RP11-618G20.1	0.13203163	0.00085436	0.32452351
chr16	cg05806333	TSS1500	FBXL16;FBXL16;MSLN	0.18790536	0.00085477	0.32452351
chr12	cg02652458		PIK3C2G;RERGL	0.18798011	0.00085565	0.32452351
chr18	cg14706363			0.16791776	0.00085587	0.32452351
chr11	cg06890484	TSS200;TSS200	PTPRJ;PTPRJ;PTPRJ;PTPRJ	0.12927449	0.00085587	0.32452351
chr2	cg05655534			-0.3004474	0.00085597	0.32452351
chr7	cg09560365	TSS1500	XRCC2	0.64977607	0.00085623	0.32452351
chr8	cg11685865	TSS200	TCF24	0.18471117	0.00085731	0.32452351
chr2	cg24880821	TSS1500;Body	E2;EIF4E2	0.16182421	0.00085844	0.32452351
chr7	cg13915716	Body;Body	PPP1R17	0.15714171	0.00085845	0.32452351
chr18	cg14423557	0;TSS200	CELF4	0.13351562	0.00085892	0.32452351
chr5	cg26469968	Body	CDH12;CDH12	0.20955962	0.00085931	0.32452351
chr1	cg03151917	n;1stExon;5'UTR;5'UTR	1;CCBL2;CCBL2;RBMXL1;RBMXL1;RBM	0.24847282	0.00086005	0.32452351
chr18	cg11436522	TSS1500	RP11-856M7.1	0.23239441	0.00086017	0.32452351
chr3	cg21849126		RP11-134J21.1	0.16715577	0.00086032	0.32452351
chr11	cg20234170	TSS1500	CAT	-0.3219786	0.00086092	0.32452351
chr8	cg12955975	0;TSS200	6;ANKRD46;ANKRD46;ANKRD46;ANKRD	0.1285509	0.00086113	0.32452351

chr8	cg03103158	500;5'UTR;TSS1500	TS9;INTS9;INTS9;INTS9;INTS9;INTS9;INT	0.21100814	0.00086114	0.32452351
chr9	cg08758850	stExon	A1;NR6A1;NR6A1;RP11-175D17.3	0.19044489	0.00086119	0.32452351
chr20	cg26831081	TSS200	TAF4;TAF4	0.20684458	0.00086192	0.32452351
chr12	cg24860848	UTR;5'UTR	F1;APAF1	0.17451541	0.00086215	0.32452351
chr10	cg11742646			0.13339968	0.00086317	0.32452351
chr16	cg08834429	5'UTR;1stExon;TSS200;	RD6A	0.26154274	0.00086385	0.32452351
chr3	cg22751696	TSS1500	204J18.3;C3orf14;C3orf14;C3orf14;C3orf14;	0.1885071	0.00086387	0.32452351
chr20	cg20080039	5'UTR;TSS1500	RA;PTPRA;PTPRA;PTPRA	0.1746467	0.00086404	0.32452351
chr18	cg14856734	TSS1500	RBFA;RBFA;RP11-795F19.1	0.11254119	0.00086453	0.32452351
chr7	cg04208803	dy	CDK6;AC002454.1;AC002454.1	0.2845534	0.00086464	0.32452351
chr14	cg12685200	stExon	;LGMN;LGMN;LGMN;LGMN;LGMN;LGM	0.10429403	0.000865	0.32452351
chr21	cg21114840	TSS200	S3	0.25415727	0.00086527	0.32452351
chr13	cg19892727	TSS1500	SLC10A2	0.18633566	0.00086534	0.32452351
chr1	cg01876832			0.17698829	0.00086551	0.32452351
chr19	cg19870717	3'UTR	APC2;APC2	-0.1664176	0.00086583	0.32452351
chr18	cg15366423	5'UTR	MAPK4;MAPK4	0.27330373	0.0008663	0.32452351
chr13	cg14754556	Body;Body	EPSTI1;EPSTI1;EPSTI1;EPSTI1;EPSTI1	0.14858436	0.00086645	0.32452351
chr1	cg20771622			0.15658595	0.00086659	0.32452351
chr10	cg25047046	Body	ARHGAP22;ARHGAP22;ARHGAP22	0.18590093	0.00086659	0.32452351
chr9	cg04080597			0.20059091	0.00086718	0.32452351
chr1	cg11911250	TSS200	CLK2;HCN3;HCN3;CLK2	0.14050726	0.0008672	0.32452351
chr3	cg04527876	TSS1500	DVL3;DVL3;DVL3;DVL3;DVL3	0.13141897	0.00086746	0.32452351
chr1	cg17241948	Body;Body;Body;Body	NCF2;SMG7;NCF2	0.17411161	0.00086794	0.32452351
chr5	cg01207010	TSS200	2165H16.4;FAM105B;FAM105B;CTD-	0.18198156	0.0008684	0.32452351
chr1	cg13432945			0.16062977	0.00087064	0.32452351
chr20	cg13642299	Body;Body	ERGIC3	0.17240771	0.00087082	0.32452351
chr12	cg18758601			0.17669301	0.0008712	0.32452351
chr7	cg06790106	Body;Body;Body;Body		0.21307624	0.00087142	0.32452351
chr10	cg23172333	Body		0.178552	0.00087152	0.32452351
chr10	cg22250466			0.17524711	0.00087164	0.32452351
chr12	cg09352124			0.20052163	0.00087299	0.32452351
chr7	cg10529933	UTR;5'UTR;1stExon;TS	CD36;CD36;CD36;CD36;CD36;CD36;CD36;	0.16158346	0.00087329	0.32452351

chr6	cg14777908			0.18813434	0.00087342	0.32452351
chr8	cg25803655		RP11-1049H7.1	0.17585134	0.00087529	0.32452351
chr11	cg19081021	TSS1500;TSS1500	TBCEL;TBCEL;TBCEL;TBCEL;TBCEL	0.15820557	0.00087563	0.32452351
chr6	cg09191810		HCG14	0.22618411	0.0008757	0.32452351
chr1	cg09409484	Body	ZSWIM5	-0.1300865	0.00087585	0.32452351
chr3	cg19890469	1stExon;5'UTR	CCDC71	0.11502274	0.00087593	0.32452351
chr5	cg27545052	Body;Body	SLIT3;SLIT3	0.17971779	0.0008762	0.32452351
chr16	cg04609801	TSS1500;TSS1500	SULT1A2;SULT1A2;SULT1A2;SULT1A2	-0.217809	0.00087638	0.32452351
chr14	cg02657021		TRAV13-2	0.20611101	0.00087655	0.32452351
chr8	cg02844177	Body		0.18768787	0.00087695	0.32452351
chr11	cg18271072			0.21039568	0.00087759	0.32452351
chr6	cg00176718			0.33744573	0.00087762	0.32452351
chr1	cg25841477	TSS1500	ZNF281;ZNF281;ZNF281;ZNF281	0.47129383	0.00087774	0.32452351
chr4	cg27200044		SAP30	0.43592617	0.0008781	0.32452351
chr8	cg16544169	TSS1500	175E9.1	-0.1091976	0.00087921	0.32476894
chr10	cg21315700	Body	94M14.2;RP11-15K3.1	-0.2003347	0.00087991	0.32482607
chr12	cg00845279			0.21661139	0.00088152	0.32482607
chr19	cg22583444	Body;Body		-0.2779833	0.00088163	0.32482607
chr16	cg19218163	Body;5'UTR	MYLK3;MYLK3	0.15073614	0.00088238	0.32482607
chr5	cg11932260			-0.1366614	0.00088262	0.32482607
chr4	cg16581308	Body;Body	LDB2;LDB2	0.13290173	0.00088272	0.32482607
chr14	cg27239392	TSS200	AHNAK2	0.16248143	0.0008829	0.32482607
chr1	cg12387700	1stExon;5'UTR	COLN3;MCOLN3;WDR63;MCOLN3	0.12624975	0.00088331	0.32482607
chr10	cg25891756	Body	snoU13;TTC18;TTC18	0.16958154	0.00088339	0.32482607
chr5	cg04710680	Body	5171.1;AC025171.1;AC025171.1;CTD-	0.16841755	0.00088435	0.32485001
chr12	cg21234793			0.18486661	0.00088504	0.32485001
chr9	cg14263946			0.25152465	0.00088526	0.32485001
chr5	cg20957179	Body	CTD-2127H9.1	0.19546642	0.00088589	0.32485001
chr1	cg05364829	1stExon;5'UTR	ECE1;ECE1;ECE1	0.24596365	0.0008867	0.32485001
chr12	cg01832294	Body		0.18070833	0.00088706	0.32485001
chr11	cg12403546	TSS200;TSS200	NRXN2	0.20582584	0.00088732	0.32485001
chr19	cg23670631	Body	LPPR3;LPPR3;LPPR3	0.25232023	0.00088754	0.32485001

chr1	cg05968270		RP4-535B20.1	0.19676513	0.00088761	0.32485001
chr8	cg16581631			0.17937217	0.00088793	0.32485001
chr17	cg23571857	TSS1500;TSS1500	1;XAF1;XAF1;XAF1;XAF1;XAF1;XAF1;XA	0.13937575	0.00088964	0.3252927
chr6	cg24063411			0.18058009	0.0008907	0.3252927
chr1	cg05198095	Body;Body	TDRD10;TDRD10;TDRD10	0.13461582	0.00089084	0.3252927
chr1	cg00398030	Body;Body;Body	1115A15.1;RERE	-0.1979073	0.00089094	0.3252927
chr11	cg12162751	;TSS200;TSS200;5'UTR	PIH1D2;PIH1D2;PIH1D2;PIH1D2;PIH1D2;C	0.18124573	0.00089196	0.32534669
chr19	cg07248407	Body	MIR638	0.11478905	0.00089205	0.32534669
chr3	cg16931877	TSS200	10	0.22666529	0.00089243	0.32534669
chr6	cg20775755	Body;Body;Body;Body	;ABHD16A;ABHD16A;ABHD16A;LY6G6E;	-0.1057788	0.00089338	0.32546634
chr11	cg26120902	3'UTR;3'UTR	CKAP5;CKAP5;CKAP5;CKAP5	0.14550929	0.00089405	0.32546634
chr1	cg15682388			0.14490998	0.00089412	0.32546634
chr3	cg21815551	xon;5'UTR;Body	EF26;ARHGEF26;ARHGEF26-	-0.2233934	0.00089455	0.32546634
chr6	cg10988349	Body;Body		0.18610736	0.00089538	0.32560697
chr21	cg20108328	Body	FAM207A;FAM207A	-0.128319	0.00089638	0.32580735
chr9	cg01421830	Body	KCNT1	-0.2532684	0.000897	0.32586821
chr5	cg21606957	Body	EPB41L4A	0.37232512	0.00089844	0.32605617
chr15	cg07052083	Body;Body	ER	0.45093329	0.00089877	0.32605617
chr6	cg19014730	UTR	FKBP5;FKBP5;FKBP5;FKBP5;FKBP5	-0.2401401	0.00090006	0.32605617
chr1	cg16601186	1500	PBX1;PBX1;PBX1;PBX1;PBX1	0.18514605	0.00090032	0.32605617
chr13	cg05478838	Body	RP11-501G6.1	0.12290382	0.00090033	0.32605617
chr4	cg00810986	Body	PCGF3;PCGF3;PCGF3	-0.22113	0.00090045	0.32605617
chr1	cg07257234	TSS200	COX20;COX20;COX20;COX20	0.27114801	0.00090067	0.32605617
chr5	cg26377963			0.21412581	0.00090121	0.32605617
chr2	cg02425365	Body	ARID5A;ARID5A;ARID5A	0.11738354	0.00090173	0.32605617
chr16	cg02669544	Body	GALNS;GALNS;GALNS;GALNS	-0.2385698	0.00090225	0.32605617
chr11	cg05524039	Body;5'UTR;Body	CLCF1;AP003419.11;CLCF1	-0.1248826	0.00090285	0.32605617
chr19	cg12209965	1stExon;5'UTR	ERF;ERF;ERF	0.27990279	0.00090312	0.32605617
chr17	cg05894322	Body	10;ABCA10;ABCA10;ABCA10	0.23024159	0.00090336	0.32605617
chr15	cg25151688	TSS200;TSS1500	11-653J6.1;DIS3L;DIS3L;DIS3L;DIS3L	0.22502918	0.00090425	0.32610074
chr11	cg11084035	1stExon;1stExon	;GANAB;GANAB;GANAB;GANAB;GANA	0.20872009	0.00090438	0.32610074
chr17	cg15646911	TSS200;TSS200	ALDH3A2;ALDH3A2;ALDH3A2	0.28411317	0.00090671	0.32643879

chr8	cg05825660			0.18455646	0.00090676	0.32643879
chr19	cg12952012	Body	ZNF460	0.19837605	0.00090853	0.32643879
chr2	cg20791712	TSS1500;5'UTR;5'UTR	HPCAL1;HPCAL1;HPCAL1;HPCAL1	-0.1005805	0.00090879	0.32643879
chr2	cg01934777		RP11-734K21.5	0.16451047	0.00090933	0.32643879
chr1	cg18111733	0	P4-735C1.4;RP4-735C1.4	0.15068395	0.00090962	0.32643879
chr5	ch.5.2533981R	Body;Body;Body		0.28550352	0.00091019	0.32643879
chr20	cg00509772	Body		-0.094039	0.00091021	0.32643879
chr8	cg25173275	Body	AA1;GPAA1;GPAA1;GPAA1	0.18607247	0.00091107	0.32643879
chr6	cg19450145	Body;Body		-0.2062856	0.00091125	0.32643879
chr19	cg19048403			0.14594191	0.00091127	0.32643879
chr2	cg00289125	TSS200	RP11-644K8.1;LAPTM4A	0.14773246	0.00091152	0.32643879
chr12	cg02276860	S1500;TSS1500;1stExo	2;SOCS2;SOCS2;RP11-887P2.1;RP11-	0.28299133	0.00091158	0.32643879
chr3	cg04653887		AC092047.1	0.2677534	0.00091161	0.32643879
chr13	cg14317195	Body;Body;Body	STK24;STK24	-0.2234289	0.00091227	0.3265129
chr10	cg12233833			0.14692785	0.00091312	0.32665661
chr2	cg11572305	TSS1500	5A	0.12350439	0.00091519	0.3268872
chr14	cg26673609	;Body;Body;Body;Body;	TXN3;ATXN3;ATXN3;ATXN3	0.09981347	0.00091524	0.3268872
chr8	cg24990978	TSS1500	CHD7;CHD7;CHD7	0.18634879	0.00091643	0.3268872
chr21	cg12690462	1500	A	-0.6214509	0.0009166	0.3268872
chr3	cg00496427	0;TSS1500;TSS200;5'U	AS1;FGD5-AS1;FGD5-	0.16834617	0.00091741	0.3268872
chr10	cg05916456			0.18745491	0.000919	0.3268872
chr22	cg07224310	Body		-0.1957049	0.00091914	0.3268872
chr1	cg07765403	Body;Body		0.18849351	0.00091976	0.3268872
chr10	cg26317269			0.13543155	0.00092007	0.3268872
chr7	cg04739306	Body	FR2	0.16352558	0.00092043	0.3268872
chr3	cg05380130			0.15673687	0.00092061	0.3268872
chr6	cg00321824	3'UTR	SLC22A3	0.15077353	0.00092086	0.3268872
chr13	cg21577811	stExon	SLC46A3;SLC46A3	0.13427281	0.00092097	0.3268872
chr7	cg24845274	UTR;5'UTR;5'UTR;5'U	M2	-0.1689005	0.000921	0.3268872
chr16	cg09243332	ExonBnd;ExonBnd;Exo	MYT1	-0.1139327	0.00092124	0.3268872
chr5	cg15045943	Body		0.2605191	0.00092138	0.3268872
chr5	cg26337020	TSS200;TSS200	ST8SIA4;ST8SIA4;ST8SIA4	0.14783934	0.00092142	0.3268872

chr7	cg06243400	TSS1500	TWIST1;TWIST1;TWIST1	-0.1701053	0.00092244	0.32708893
chr2	cg02545303		AC010729.3	0.21786947	0.00092429	0.32741777
chr12	cg07216660			0.19300194	0.00092431	0.32741777
chr20	cg01289240			0.17924862	0.00092472	0.32741777
chr17	cg16707139	1500;TSS200;TSS200	NBR1;NBR1;NBR1;BRCA1;NBR1	0.16648877	0.0009259	0.32754176
chr2	cg21117768	stExon;Body	M150A;TMEM150A;TMEM150A;TMEM150	0.12988383	0.00092624	0.32754176
chr10	cg13931663	Body;Body	LIPA	0.17780164	0.00092643	0.32754176
chr15	cg24449371	TSS1500	3A	0.12574882	0.00092704	0.32760063
chr14	cg25088009	Body;Body	C14orf43;C14orf43;C14orf43;C14orf43	-0.0985902	0.00092802	0.32778612
chr10	cg19960880	TSS200	PDLIM1;PDLIM1	0.26655249	0.0009287	0.32778612
chr17	cg01514436	5'UTR;1stExon	NT5C;NT5C	0.18425764	0.00092917	0.32778612
chr16	cg22227603	1500;TSS200	;CMTM3;CMTM3;CMTM3;CMTM3;CMTM	0.37348297	0.00092979	0.32778612
chr3	cg16772140	TSS200	EPHB1;EPHB1;EPHB1;EPHB1	0.13563778	0.00092983	0.32778612
chr17	cg07580832	Body;Body		-0.1942918	0.0009311	0.32802111
chr1	cg07195508	5'UTR;5'UTR;Body;5'U	R1;WDR78;WDR78;WDR78;WDR78;MIER1	0.22469259	0.0009314	0.32802111
chr4	cg07325595		79E3.2;RP11-79E3.3	0.18566667	0.00093313	0.32844584
chr10	cg07903860	stExon;1stExon;1stExon	DCLRE1C;DCLRE1C;DCLRE1C;DCLRE1C;	0.15812531	0.0009341	0.32844584
chr4	cg24507667	Body	Y_RNA;RP11-415C15.2	0.17714229	0.00093464	0.32844584
chr1	cg26886978	TSS1500	TMCO4;TMCO4;TMCO4;TMCO4	0.20582656	0.00093504	0.32844584
chr12	cg03406913	y;Body;Body;5'UTR	C1B;POC1B	0.25018433	0.00093543	0.32844584
chr9	cg22887281	0;Body	SUSD1;SUSD1;SUSD1;SUSD1	0.21521321	0.00093546	0.32844584
chr20	cg19055411		U6	0.33104419	0.000936	0.32844584
chr6	cg23819331	1500	F1;BCLAF1;BCLAF1;BCLAF1	0.16036375	0.00093656	0.32844584
chr5	cg23417379	Body	CEP72	-0.178181	0.00093668	0.32844584
chr21	cg12145552	TSS1500	C2CD2;C2CD2;TMPRSS3	0.25572401	0.00093986	0.32908007
chr10	cg13400443	TSS1500	EXOC6	0.15597477	0.00093987	0.32908007
chr6	cg02128139	5'UTR;1stExon	ZBTB9;ZBTB9;ZBTB9;ZBTB9	0.14065965	0.00094014	0.32908007
chr7	cg06165179	1stExon;1stExon	RP11-467D6.1;CDK13;CDK13	0.27822759	0.00094078	0.32908007
chr2	cg05691168	1stExon;5'UTR	TIC;ATIC;ATIC;ATIC;ATIC	0.19265395	0.00094112	0.32908007
chr3	cg18429097			0.17603207	0.00094121	0.32908007
chr10	cg16676551	TSS1500	OTUD1	0.20468661	0.00094166	0.32908009
chr8	cg01490091	Body;Body;Body	92K15.1;TPD52;TPD52;TPD52	0.16274859	0.00094451	0.32969925

chr17	cg04307297	3'UTR	TBKBP1;TBKBP1	-0.1707629	0.00094534	0.32969925
chr7	cg27000449	TSS200	SNX8;SNX8;SNX8;SNX8	0.21223169	0.00094546	0.32969925
chr11	cg16724696	5'UTR;5'UTR	HINFP	0.13197672	0.00094579	0.32969925
chr6	cg26498782	Body	RP1-292B18.4	-0.1611552	0.00094645	0.32969925
chr5	cg01976641			0.19552561	0.00094663	0.32969925
chr1	cg13064914	Body	;HMGN2;HMGN2;HMGN2	0.13369435	0.00094663	0.32969925
chr1	cg12290861			0.17919986	0.00094706	0.32969925
chr6	cg22045714	0;TSS200;5'UTR;5'UTR	6;SERPINB6	0.37362307	0.00095004	0.33057813
chr1	cg08158076	TSS1500;5'UTR	LRRC8D;LRRC8D;LRRC8D	0.23494984	0.00095125	0.33083176
chr3	cg18241469	Body	EGFEM1P;EGFEM1P	0.19707467	0.00095283	0.33083176
chr8	cg26476169	Body		0.1500134	0.00095341	0.33083176
chr15	cg12384499			0.25485605	0.00095375	0.33083176
chr1	cg03988532	Body	CHD5;CHD5	-0.1408564	0.00095387	0.33083176
chr15	cg01848910	Body		0.24642604	0.00095424	0.33083176
chr7	cg15585372	TSS1500;TSS1500	BRI3;BRI3;BRI3;BRI3;BRI3;BRI3	0.17801864	0.00095443	0.33083176
chr16	cg15650422	TSS200;Body	5B;GPRC5B;GPRC5B	0.17713668	0.00095457	0.33083176
chr14	cg01851809	y	5M1;EXOC5;EXOC5;EXOC5	0.13160596	0.00095506	0.33083176
chr17	cg04467549			0.18308681	0.00095545	0.33083176
chr9	cg14571425			0.13635013	0.00095594	0.33083176
chr4	cg01380244			-0.1618599	0.00095641	0.33083176
chr22	cg06659169	TSS200;TSS200	ZNF74;ZNF74;ZNF74	0.13970926	0.0009567	0.33083176
chr7	cg06612016	Body;Body;Body		-0.275182	0.00095728	0.33086332
chr15	cg16606478	1stExon;1stExon	2;FBXO22;FBXO22;FBXO22;FBXO22	0.15010808	0.0009577	0.33086332
chr19	cg06391536	Body;Body;Body	MEIS3	-0.1880362	0.00095844	0.3309623
chr1	cg01259145	Body;Body	465N4.4;ELF3;ELF3;ELF3;ELF3;RP11-	0.13078478	0.00095907	0.33102018
chr19	cg13995073	Body		-0.1987488	0.00095972	0.3310547
chr11	cg05375277	TSS1500	C43A1;SLC43A1;SLC43A1	0.12331353	0.00096065	0.3310547
chr3	cg17999289			-0.1597323	0.00096094	0.3310547
chr2	cg20147515	Body	MYO7B	-0.0824756	0.00096105	0.3310547
chr5	cg10437652	TSS1500	ZFP62;ZFP62;ZFP62;ZFP62;ZFP62	0.29893024	0.00096167	0.3310547
chr1	cg08128789	TSS1500	LRRC8B;LRRC8B	0.19384808	0.00096213	0.3310547
chr17	cg10304803	Body		-0.2889314	0.0009631	0.3310547

chr2	cg19130836	TSS200;TSS200	MRPL35;MRPL35	0.3033031	0.00096349	0.3310547
chr2	cg00604449	Body	MERTK;MERTK	-0.1636193	0.00096364	0.3310547
chr19	cg12045676	Body;Body		0.16376306	0.00096387	0.3310547
chr1	cg23137562	1stExon;5'UTR	00A10;S100A10	0.12209102	0.0009643	0.3310547
chr16	cg11238564	TSS1500;Body	895L;KIAA0895L;KIAA0895L;EXOC3L1;EX	-0.1381686	0.00096464	0.3310547
chr20	cg10073706	Body	NPEPL1;NPEPL1;NPEPL1	0.22273305	0.00096685	0.33164747
chr22	cg26361276	TSS200	CCDC134;CCDC134	0.17425114	0.00096728	0.33164747
chr16	cg03949642	TSS1500	P1;CLUAP1;CLUAP1;CLUAP1;LA16c-	-0.1683057	0.00096783	0.33167945
chr20	cg04426832	n;1stExon;5'UTR;5'UTR	FP64;ZFP64;ZFP64;ZFP64	0.12451543	0.00096969	0.33187526
chr13	cg07206531	Body		0.17941142	0.00097012	0.33187526
chr6	cg25172519	TSS200	MOXD1;MOXD1	0.21636883	0.00097046	0.33187526
chr7	cg19269292	5'UTR	744I24.2;AGK;AGK;AGK;AGK;AGK	0.12417825	0.00097097	0.33187526
chr2	cg26600215			0.18494281	0.00097142	0.33187526
chr2	cg11942086	TSS1500	CRYGB	0.13219764	0.00097166	0.33187526
chr10	cg23245945	TSS200	O1;GSTO1;GSTO1	0.19199497	0.0009718	0.33187526
chr5	cg01982422			0.23645262	0.00097366	0.33187526
chr6	cg08354681	Body	CDYL;CDYL;CDYL;CDYL;CDYL;CDYL	-0.2348686	0.0009741	0.33187526
chr8	cg18888392	5'UTR	FAM110B;FAM110B;FAM110B	0.16733552	0.0009754	0.33187526
chr16	cg07253630	UTR;5'UTR	A;CIRH1A;CIRH1A;CIRH1A;CIRH1A;CHT	0.22495612	0.00097564	0.33187526
chr5	cg07256113	Body	175K6.1;EBF1	0.14325337	0.00097638	0.33187526
chr2	cg18994033	Body;Body;Body	ITM2C;ITM2C;ITM2C;ITM2C	-0.2745468	0.0009765	0.33187526
chr2	cg02794788		TPO	0.14038455	0.00097812	0.33187526
chr5	cg14360044	TSS200	ISOC1;ISOC1;ISOC1	0.1141116	0.00097946	0.33187526
chr11	cg19320505	00	MOD1;ELMOD1	0.13609622	0.00097958	0.33187526
chr16	cg16236759	00;5'UTR;TSS1500	UBE2I;UBE2I;UBE2I;UBE2I;UBE2I;UBE2I;	0.20234327	0.00098042	0.33187526
chr3	cg02900978	1stExon	KBTBD5;KBTBD5	-0.1403671	0.00098048	0.33187526
chr6	cg03410961	TSS200	IRF4;IRF4;IRF4	0.31240183	0.00098132	0.33187526
chr16	cg09057080	Body;TSS1500	8;CHTF18;CHTF18;CHTF18;RPUSD1	-0.2491838	0.00098153	0.33187526
chr17	cg07862889	TSS1500	C17orf103;C17orf103	0.28687787	0.0009825	0.33187526
chr14	cg21554139			0.27547696	0.00098257	0.33187526
chr22	cg17285325	;5'UTR;TSS200	YMP;TYMP;TYMP;ODF3B;TYMP;TYMP;T	0.26927348	0.00098273	0.33187526
chr1	cg03499659		RP11-275A6.1	-0.1182562	0.00098373	0.33187526

chr16	cg27515922		RP11-883G14.1	-0.1737583	0.00098397	0.33187526
chr10	cg24887381	TSS200	ARID5B;ARID5B	0.91609659	0.00098403	0.33187526
chr4	cg18423456	Body;Body	C4orf45	0.17654989	0.0009843	0.33187526
chr5	cg16087299	Body;Body;Body;Body	VCAN-AS1;VCAN;VCAN	0.14027934	0.00098439	0.33187526
chr13	cg10354859	Body	VPS36;VPS36	0.1247324	0.00098472	0.33187526
chr3	cg00830116			0.17814992	0.00098508	0.33187526
chr17	cg01439340		RP11-1055B8.6	0.13558388	0.00098627	0.33187526
chr1	cg05553634	;5'UTR	ZYG11A;ZYG11A;ZYG11A	0.1244184	0.00098643	0.33187526
chr2	cg02090437	;5'UTR	PP1;INPP1;INPP1;INPP1;INPP1;INPP1;INPP	0.24684324	0.00098645	0.33187526
chr5	cg02368356	Body	TRIO;TRIO;TRIO	-0.2216781	0.0009865	0.33187526
chr16	cg11449994	Body;Body;Body	H1	0.22832293	0.00098661	0.33187526
chr1	cg05764766	0;TSS200	PQLC2;PQLC2;PQLC2;AKR7A2	0.16405952	0.00098661	0.33187526
chr7	cg18493430	Body;Body;Body	MAD1L1	-0.1179463	0.00098685	0.33187526
chr3	cg24590565	UTR	RB;THRB;THRB;THRB;THRB;THRB	0.19165579	0.00098689	0.33187526
chr1	cg01717973	Body;TSS200	98D18.3;MRPL9;MRPL9;MRPL9;MRPL9;M	0.11879623	0.00098758	0.33187526
chr5	cg08339051			0.15348722	0.0009878	0.33187526
chr17	cg25445749	TSS200	C17orf108;C17orf108;C17orf108;C17orf108	0.14587273	0.00098799	0.33187526
chr12	cg12411093	TSS200	CHST11;CHST11;CHST11;CHST11;CHST11	0.13024721	0.00098831	0.33187526
chr12	cg08179795		EPS8	0.16779029	0.0009885	0.33187526
chr15	cg12925764	TSS1500	1	0.13914682	0.00098854	0.33187526
chr15	cg13704548	Body		0.42507003	0.00098932	0.33187526
chr11	cg07624600	TSS200;TSS200	ART5;ART5;ART5	-0.1502281	0.0009897	0.33187526
chr4	cg15613153	5'UTR;5'UTR;Body	IL15;IL15;IL15;IL15	0.20234676	0.00098989	0.33187526
chr11	cg16274762	Body	BRSK2;BRSK2;BRSK2;BRSK2;BRSK2	-0.1045959	0.00099145	0.33221263
chr2	cg21654303	dy;Body;Body	KIAA1715;KIAA1715;KIAA1715;KIAA1715	0.23961326	0.00099181	0.33221263
chr5	cg13811092	Body;Body;Body		0.2015685	0.00099315	0.33228672
chr8	cg14423045	Body	DENND3;DENND3;DENND3;DENND3	-0.1515336	0.00099321	0.33228672
chr1	cg21841814	Body	DL	0.17272444	0.00099341	0.33228672
chr3	cg19143760	Body	GYG1;GYG1;GYG1	0.12980607	0.00099467	0.3323152
chr15	cg08889009		RP11-30K9.5;RNF111;RP11-30K9.5	0.24281885	0.0009947	0.3323152
chr5	cg12345696	TSS200;TSS200	AM193B;FAM193B	0.12650136	0.00099487	0.3323152
chr20	cg16473762	Body		0.32807588	0.00099562	0.33238597

chr6	cg13917060			0.1595718	0.00099776	0.33238597
chr7	cg22336318	UTR;5'UTR;5'UTR;5'U	7orf49;C7orf49;C7orf49;C7orf49;C7orf49;C7	0.21718507	0.00099813	0.33238597
chr10	cg10131095	TSS200;TSS200	RNLS;RNLS;RNLS;RNLS;RNLS;RNLS	0.21958794	0.00099814	0.33238597
chr1	cg11473322	Body	PADI6	0.17350599	0.00099835	0.33238597
chr8	cg12813754			0.14706938	0.00099875	0.33238597
chr13	cg24089171	Body	FGF14-IT1;FGF14-IT1;FGF14-AS1	0.19902021	0.00099899	0.33238597
chr8	cg08956735	Body;Body	2172A10.1;EXTL3;EXTL3;EXTL3;CTD-	0.12607475	0.00099995	0.33238597

Supplementary Table S5. Comparison of log2 fold changes at the FDR-significant sites across the three discovery cohort contrasts.

chr	probe	log2FC - BD/SA vs. CON	log2FC - BD/SA vs. BD/non-SA	log2FC - BD/non-SA vs. CON	gene	gene distance	gene feature
chr4	cg20244265	-0.18	-0.33	0.09	*CXCL8*	-22861	intergenic
chr17	cg25876840	0.08	0.27	-0.1	*CD300LG*	-4066	intergenic
chr17	cg11476866	0.14	0.32	-0.08	*CD300LG*	-4128	intergenic
chr7	cg15653194	0.13	-0.63	0.69	*LFNG*	0;0	exon+utr5;intron
chr2	cg00306112	0.04	0.23	-0.12	*LINC01494*	0	nc_intron
chr7	cg20242392	0.04	0.32	-0.1	*DGKI*	0;0	intron;intron+utr5

Supplementary Table S6. Comparison of log2 fold changes at the DMPs at nominal p < 0.001 across the three discovery cohort contrasts.

Name	logFC_BDSA_BDnonSA	logFC_BDSA_CON	logFC_BDnonSA_CON	Agree_BDSA_BDNSA_BDSA_CON	Agree_BDSA_BDNSA_BDNSA_CON	Agree_BDSA_CON_BDNSA_CON
cg20244265	-0.3302726	-0.1751553	0.09404345	1	0	0
cg25876840	0.26931195	0.07535794	-0.1031051	1	0	0
cg11476866	0.32321764	0.14206867	-0.0781437	1	0	0
cg15653194	-0.6268628	0.13453205	0.68872846	0	0	1
cg00306112	0.23448923	0.04320544	-0.1178257	1	0	0
cg20242392	0.31593882	0.18362249	-0.0982527	1	0	0
cg13065624	-0.285941	-0.1090529	0.17583047	1	0	0
cg05454076	0.20818129	0.11458842	-0.0772974	1	0	0
cg08649048	-0.2603638	-0.0655595	0.09064015	1	0	0
cg27145165	-0.2193758	-0.0440102	0.16604232	1	0	0
cg05077488	-0.2277804	-0.0549043	0.09122253	1	0	0
cg18042859	-0.1650394	-0.0884858	0.06606639	1	0	0
cg23190823	0.15686357	0.05275217	-0.0671308	1	0	0
cg23143093	-0.1840548	-0.0885301	0.09521383	1	0	0
cg07058706	-0.2136145	-0.0460399	0.20367322	1	0	0
cg02088355	0.22850203	0.06200706	-0.1268416	1	0	0
cg27148007	-0.1671539	-0.0370419	0.10869563	1	0	0
cg11366178	0.25548752	0.07580901	-0.0994163	1	0	0
cg11328284	-0.2690633	-0.0468892	0.18215629	1	0	0
cg05186787	0.47572216	0.06896196	-0.2707863	1	0	0
cg08026078	0.25249873	0.11878996	-0.1475299	1	0	0
cg00888507	-0.1760226	-0.0762452	0.07947538	1	0	0
cg23629264	0.17231103	0.1038934	-0.0101733	1	0	0
cg07397939	-0.1858535	-0.0497699	0.14111897	1	0	0
cg01942558	-0.2350465	-0.0680056	0.09039662	1	0	0
cg15282634	0.19883972	0.0837083	-0.020993	1	0	0

cg26986629	-0.201111	-0.0949385	0.11990932	1	0	0
cg26463200	0.26807502	0.06237334	-0.1021602	1	0	0
cg05999592	0.15591784	0.02174839	-0.0672037	1	0	0
cg13590419	-0.1834079	-0.0514829	0.17880369	1	0	0
cg21248089	-0.2447266	-0.0838829	0.0776742	1	0	0
cg00431564	-0.1732368	-0.0419389	0.14390517	1	0	0
cg24777028	0.36285554	0.15354639	-0.1689519	1	0	0
cg23681017	-0.1261616	-0.0612056	0.05952515	1	0	0
cg00984985	0.11120462	-0.0031365	-0.0953782	0	0	1
cg02113493	0.15560876	0.04295912	-0.1075102	1	0	0
cg01673307	-0.1730423	-0.078403	NA	1	NA	NA
cg13954127	-0.2306245	-0.184439	0.02783465	1	0	0
cg12349832	0.11781878	0.01721593	-0.0503968	1	0	0
cg21902874	0.10498636	-0.0095912	-0.0914171	0	0	1
cg02997497	-0.2458791	-0.0939869	0.07433324	1	0	0
cg14302909	-0.2232178	-0.218164	0.00920483	1	0	0
cg17832917	-0.169277	-0.0656881	0.09350051	1	0	0
cg08564349	-0.1812676	-0.0590742	0.11740696	1	0	0
cg03124710	-0.2373383	-0.0619139	0.17244817	1	0	0
cg26150489	0.22438497	0.12395505	-0.0160996	1	0	0
cg13765206	0.14814166	0.01018854	-0.0999217	1	0	0
cg17770458	-0.2503428	-0.1521507	0.0502593	1	0	0
cg20280337	0.12888028	0.05236582	-0.0682349	1	0	0
cg22025478	0.43576193	0.37658655	0.01588799	1	1	1
cg04430871	-0.1815242	-0.0368559	0.12276645	1	0	0
cg14896157	0.25351645	0.17561664	-0.063187	1	0	0
cg09856246	-0.1407546	-0.0720219	0.03395818	1	0	0
cg13054523	0.44387911	-0.0978382	-0.3861854	0	0	1
cg15046793	0.10860334	0.02155094	-0.0601467	1	0	0
cg12404216	0.132344	-0.0127453	-0.1312293	0	0	1
cg14789775	-0.1869881	-0.0465336	0.15610274	1	0	0

cg20755748	0.15948087	0.11898324	-0.0297787	1	0	0
cg03741118	-0.1787635	-0.0521082	0.10823707	1	0	0
cg19560014	0.24946615	0.12869932	-0.1091973	1	0	0
cg15239248	-0.2014681	-0.0359982	0.15166739	1	0	0
cg23504719	0.31662566	0.16271659	-0.1815979	1	0	0
cg17293125	-0.1606111	-0.0310137	0.07395138	1	0	0
cg02954577	-0.180619	-0.0640582	0.06804619	1	0	0
cg23631370	0.15562495	0.0918571	-0.0758011	1	0	0
cg22822599	-0.2195767	-0.1069281	0.05328252	1	0	0
cg14137282	0.14601402	0.07833245	-0.0550502	1	0	0
cg23542477	-0.2137709	-0.1326045	0.08014179	1	0	0
cg25349738	-0.2628617	-0.0546721	0.14172958	1	0	0
cg10680133	0.1120015	0.03981877	-0.0853672	1	0	0
cg18094261	0.10544026	0.0559491	0.01081987	1	1	1
cg00730266	-0.1638288	0.01153756	0.16300192	0	0	1
cg01477959	-0.1586417	-0.0718043	0.06720847	1	0	0
cg23132302	0.15114991	-0.0140004	-0.0923525	0	0	1
cg19405799	-0.1654261	-0.1060775	0.04724015	1	0	0
cg21991092	0.2098959	0.08963691	-0.0513636	1	0	0
cg04935656	0.21751179	0.12931209	-0.0240399	1	0	0
cg20041670	-0.5504991	-0.1733239	0.24538911	1	0	0
cg22657448	-0.1536807	-0.0469003	0.13338448	1	0	0
cg14790883	-0.1639913	-0.0042075	0.20366651	1	0	0
cg10289177	0.13429066	-0.0113763	-0.1067053	0	0	1
cg25241447	0.11336739	-0.0066144	-0.0494309	0	0	1
cg00551617	0.57588924	0.33243823	0.00481103	1	1	1
cg25420477	0.16687343	0.09536543	-0.0375006	1	0	0
cg18536819	-0.1521378	-0.0713484	0.05891383	1	0	0
cg16182479	0.09795328	0.02797242	-0.0471325	1	0	0
cg16788865	-0.2009535	-0.0887003	0.03497556	1	0	0
cg01457573	0.10275934	0.0335229	-0.0535711	1	0	0

cg12729305	-0.1546547	-0.0903507	0.06737239	1	0	0
cg03412153	0.12333666	0.04562297	-0.052674	1	0	0
cg06952529	-0.1749012	-0.0867271	0.09007595	1	0	0
cg00251102	-0.1515774	-0.0491601	0.08043058	1	0	0
cg02459569	0.10503784	0.00859059	-0.0746971	1	0	0
cg24188870	-0.2227512	-0.0663375	0.15956864	1	0	0
cg23118377	-0.3248768	-0.2004286	0.15546637	1	0	0
cg06430695	-0.2825935	-0.0929378	0.1119011	1	0	0
cg22723842	-0.1598849	-0.0126573	0.15197617	1	0	0
cg21660861	-0.1564194	-0.0588849	0.05081918	1	0	0
cg06893273	0.2496519	0.03625714	-0.0849726	1	0	0
cg05016698	0.11366107	0.02074954	-0.0629221	1	0	0
cg11518826	-0.1667247	0.04084598	0.17355412	0	0	1
cg10021935	-0.1506018	-0.0534394	0.05392824	1	0	0
cg27112470	0.14109839	0.02660862	-0.0574372	1	0	0
cg14386691	0.21771934	-0.0138711	-0.0764451	0	0	1
cg19756320	-0.1767822	0.00375	0.15986263	0	0	1
cg09755924	0.13166183	0.0631887	-0.0042117	1	0	0
cg10973967	0.1634775	0.09450059	-0.0207886	1	0	0
cg18759809	0.14073331	0.02800157	-0.0651727	1	0	0
cg06634862	0.23903193	0.11438949	-0.0177067	1	0	0
cg14527639	-0.1501484	-0.0457702	0.0956904	1	0	0
cg14885150	0.13776557	0.07503565	-0.0709347	1	0	0
cg15705079	0.14496518	0.0645231	-0.021837	1	0	0
cg24158019	-0.1648576	-0.0555439	0.07183395	1	0	0
cg04695090	0.1034675	0.04258502	-0.0139256	1	0	0
cg20962215	0.09819384	-0.0072563	-0.0712603	0	0	1
cg18428489	0.12679327	0.07157453	-0.0450457	1	0	0
cg12558410	-0.1788357	-0.0299566	0.15335976	1	0	0
cg04224661	0.15150721	0.04483069	NA	1	NA	NA
cg15351895	-0.1509223	-0.078114	0.03296064	1	0	0

cg02014883	0.09502443	0.04073995	-0.0089773	1	0	0
cg14343283	0.09551881	0.01328412	-0.0451559	1	0	0
cg17243528	-0.1465959	-0.0696726	0.06799602	1	0	0
cg23579306	0.13775257	0.05792082	-0.0370582	1	0	0
cg20263853	0.18918492	-0.0310732	-0.135323	0	0	1
cg16640791	-0.1633468	-0.0702517	0.09056979	1	0	0
cg04898572	0.16319458	0.06914981	-0.0591514	1	0	0
cg09883841	0.09695468	0.00793668	-0.0594989	1	0	0
cg27441580	0.14339894	0.01672064	-0.0611471	1	0	0
cg06559111	-0.1330631	-0.0746952	0.02978144	1	0	0
cg17641710	0.10589881	0.00706749	-0.0789362	1	0	0
cg20636743	0.15084056	0.05685177	-0.0317724	1	0	0
cg08510096	0.15745884	0.05724324	-0.0809871	1	0	0
cg06556650	0.29606796	0.16121722	-0.1063808	1	0	0
cg11007075	0.14296723	0.02271986	-0.0780295	1	0	0
cg14555697	-0.1429491	-0.0441091	0.09289424	1	0	0
cg22566355	0.12004448	0.01880442	-0.0455321	1	0	0
cg08491582	0.16785689	0.06997408	-0.0794781	1	0	0
cg06169270	0.10582554	0.00832125	-0.0730995	1	0	0
cg03894891	-0.213274	-0.1642647	0.02394988	1	0	0
cg11397301	0.13240671	-0.0021117	-0.0887899	0	0	1
cg03723356	0.15146291	-0.0127711	-0.0946623	0	0	1
cg18763727	0.13596185	0.08709539	-0.0226845	1	0	0
cg13333788	0.14045225	0.02475234	-0.0539976	1	0	0
cg11473272	-0.2067107	-0.061709	0.11412142	1	0	0
cg09503853	-0.2030642	-0.0548486	0.09069953	1	0	0
cg02019125	0.16284227	0.12302799	-0.0206574	1	0	0
cg16934894	0.10962331	0.00789198	-0.0535245	1	0	0
cg22578433	0.25717797	0.0531941	-0.1037908	1	0	0
cg16722884	-0.1407572	-0.0201627	0.073103	1	0	0
cg03603211	0.20547071	0.07233308	-0.0323726	1	0	0

cg03935444	-0.168973	-0.0725591	0.0851247	1	0	0
cg12870052	-0.2573995	-0.0674908	0.19702124	1	0	0
cg02233041	-0.2815617	-0.083779	0.17254065	1	0	0
cg02625241	-0.2316465	-0.0771459	0.07676313	1	0	0
cg20215176	-0.2166446	-0.0940986	0.10873034	1	0	0
cg20316542	-0.1432772	-0.1168163	0.0122826	1	0	0
cg26104293	-0.18123	-0.053774	0.05890061	1	0	0
cg05369810	-0.1854433	-0.0871969	0.10568307	1	0	0
cg09859911	0.11121517	0.01254593	-0.0517252	1	0	0
cg27504299	-0.1760711	-0.1452771	-0.0027957	1	1	1
cg10763891	-0.1777126	0.01217004	0.15834985	0	0	1
cg17695512	-0.2727632	-0.2355596	0.06551351	1	0	0
cg16146513	0.14011685	-0.0048856	-0.0773772	0	0	1
cg11919883	-0.2201051	-0.0482081	0.1610457	1	0	0
cg10842530	0.11183489	0.0163041	-0.0767311	1	0	0
cg04660111	0.1469437	0.02190075	-0.0778108	1	0	0
cg09007811	-0.1991592	-0.0438634	0.19949259	1	0	0
cg21320412	-0.1319903	0.00228193	0.08359107	0	0	1
cg10079538	-0.1975943	-0.1256802	0.09933827	1	0	0
cg05539847	0.12593537	NA	NA	NA	NA	NA
cg02726566	-0.1581877	-0.0572865	0.13248308	1	0	0
cg22866825	0.36016601	0.22576267	-0.0213939	1	0	0
cg04721651	0.16431407	0.08955077	-0.0454462	1	0	0
cg16558432	0.70118404	0.42879384	-0.1624851	1	0	0
cg08458745	0.09903482	0.00249332	-0.0431866	1	0	0
cg15209695	0.15895336	0.10779085	-0.025645	1	0	0
cg26845395	-0.1591572	-0.1136361	0.00715814	1	0	0
cg14156592	-0.2227892	-0.0245693	0.13486635	1	0	0
cg04957377	-0.1601038	-0.0841127	0.0650963	1	0	0
cg14765416	0.15195663	0.09931967	0.01528314	1	1	1
cg04751078	-0.1863306	-0.0647089	0.15807096	1	0	0

cg02101203	0.37834262	0.19965925	-0.1959429	1	0	0
cg22957691	-0.2303825	-0.0142104	0.1049715	1	0	0
cg22167396	-0.2096017	-0.1657695	0.03605553	1	0	0
cg06768071	0.14026822	0.06532135	-0.0991748	1	0	0
cg09390308	-0.2002207	-0.0450554	0.19853505	1	0	0
cg23991388	-0.2542202	-0.2271564	0.08038353	1	0	0
cg20197622	-0.1579141	-0.041894	0.08182875	1	0	0
cg01899252	0.10535047	0.05021419	-0.0483522	1	0	0
cg01067137	0.14641734	0.0308091	-0.1078852	1	0	0
cg21214508	0.24521693	0.11534	-0.0725964	1	0	0
cg04324158	-0.1887632	-0.0551375	0.18516643	1	0	0
cg06496197	0.10628876	0.0460668	-0.0142357	1	0	0
cg12219401	-0.1396135	-0.0434268	0.06747572	1	0	0
cg20400045	-0.1948689	-0.0366707	0.1129427	1	0	0
cg13392258	0.26894952	0.11328872	-0.0201398	1	0	0
cg02445229	0.10544845	0.00455675	-0.0546935	1	0	0
cg22147003	-0.1215726	0.00751009	0.12006615	0	0	1
cg25272883	-0.2190525	-0.0715426	0.05871657	1	0	0
cg18356679	-0.2655606	0.0455433	0.30504516	0	0	1
cg23055674	0.10504086	0.0127889	-0.0746756	1	0	0
cg00404394	0.11145743	-0.0050304	-0.1248719	0	0	1
cg02411284	-0.1507542	0.02940797	0.18942158	0	0	1
cg05208605	0.19995568	NA	-0.0064733	NA	0	NA
cg03217204	-0.1916049	0.01593168	0.1688649	0	0	1
cg22303418	0.12016036	0.06612631	-0.0359317	1	0	0
cg10538440	-0.1651974	-0.0178544	0.16954508	1	0	0
cg22023664	0.11707788	0.00587131	-0.071814	1	0	0
cg26377940	-0.1969658	0.10497657	0.26218629	0	0	1
cg26542091	0.11272543	-0.0005516	-0.0706452	0	0	1
cg04906485	-0.1531284	-0.0339226	0.13370372	1	0	0
cg17884069	-0.2478801	-0.0823754	0.14486899	1	0	0

cg09808864	0.21248441	0.13078013	-0.0019618	1	0	0
cg16450683	-0.1642216	-0.0270244	0.14817134	1	0	0
cg05812836	0.23847338	0.05864529	-0.1619942	1	0	0
cg01363264	0.13083345	0.06625882	-0.0034079	1	0	0
cg21977234	0.41927987	0.37394592	0.05447971	1	1	1
cg05977840	0.11364277	0.05078063	-0.0552112	1	0	0
cg14775431	0.10051913	-0.0236598	-0.0919007	0	0	1
cg24164973	0.24787393	0.04757018	-0.2119584	1	0	0
cg17474349	0.10051847	0.02829239	-0.0197424	1	0	0
cg26009832	-0.2041717	0.03961287	0.2601844	0	0	1
cg05352821	0.20080327	0.05185668	-0.0781391	1	0	0
cg21220721	0.56286589	-0.1880672	-0.5555324	0	0	1
cg16715953	0.16609767	0.09621246	0.01269275	1	1	1
cg23342367	-0.2465877	-0.075408	0.116771	1	0	0
cg01610123	-0.1499423	-0.0645025	0.07019768	1	0	0
cg18731460	-0.1650366	-0.0329369	0.12599293	1	0	0
cg03492094	0.14625264	-0.0037775	-0.1020858	0	0	1
cg05482226	0.19253203	0.00077951	-0.1778885	1	0	0
cg15236764	0.26431138	0.0954525	-0.1439882	1	0	0
cg02969500	-0.1419109	-0.1069927	0.02547059	1	0	0
cg17487559	-0.1472439	-0.0714727	0.04082251	1	0	0
cg07175985	0.21781564	0.13943095	-0.0087818	1	0	0
cg12807368	-0.1842219	-0.1335488	0.08372393	1	0	0
cg14791530	-0.1635032	-0.0770326	0.04758684	1	0	0
cg02264459	-0.3026294	-0.1740562	0.0124902	1	0	0
cg22594773	0.14384951	0.01352466	-0.0777758	1	0	0
cg27040968	-0.1466229	-0.1085961	0.02259459	1	0	0
cg24922045	0.12043638	0.05271254	-0.0285345	1	0	0
cg19480933	0.11808901	0.04264583	-0.0549505	1	0	0
cg13603638	-0.1452059	0.00862852	0.13336067	0	0	1
cg11906641	-0.1562661	-0.0253437	0.08649671	1	0	0

cg20981515	-0.2712373	NA	0.17116223	NA	0	NA
cg13569987	-0.1257859	-0.0204305	0.10231875	1	0	0
cg02158486	0.23187337	0.24570649	0.00798779	1	1	1
cg03528037	-0.1607747	-0.0321501	0.09704842	1	0	0
cg13676099	0.11472046	0.04289465	-0.0673164	1	0	0
cg11789185	0.1875541	0.03463117	-0.045136	1	0	0
cg16047053	-0.1770229	-0.0287294	0.10475177	1	0	0
cg10611016	-0.1446558	-0.0445535	0.0506966	1	0	0
cg25910699	-0.1977986	-0.0124645	0.13761738	1	0	0
cg02575035	-0.1602261	-0.0720722	0.09701033	1	0	0
cg00449491	-0.1951875	-0.0548398	0.11588775	1	0	0
cg00143376	0.1226573	0.03236187	-0.0438146	1	0	0
cg07829731	-0.1959841	-0.0667431	0.09267119	1	0	0
cg10688790	-0.1602887	-0.026413	0.09753716	1	0	0
cg20669090	-0.1585733	-0.0588231	0.07840265	1	0	0
cg13588483	-0.1551865	-0.0079761	0.16715364	1	0	0
cg05257429	0.15567958	0.08665599	-0.047177	1	0	0
cg12028998	0.14415752	0.04678522	-0.0490114	1	0	0
cg22853608	0.12379615	0.07503662	-0.0552783	1	0	0
cg26592496	-0.198994	-0.024547	0.19402524	1	0	0
cg07115820	0.12651085	-0.0012207	-0.1065519	0	0	1
cg01344420	-0.1314424	-0.0284624	0.0863321	1	0	0
cg04603413	-0.1564895	-0.0942235	0.0974364	1	0	0
cg19139618	-0.1410335	-0.0541869	0.11265913	1	0	0
cg09742895	-0.138853	-0.053649	0.0862001	1	0	0
cg18571531	0.115282	-0.0038441	-0.1166379	0	0	1
cg26109393	0.0891353	0.01385732	-0.0378024	1	0	0
cg20182202	0.18354143	0.00712312	-0.11497	1	0	0
cg04897804	0.15290269	0.04540998	-0.0829448	1	0	0
cg07667018	-0.1578751	-0.0891851	0.03529484	1	0	0
cg17134838	0.19991183	0.02213344	-0.0574832	1	0	0

cg24474319	0.09320759	-0.0329988	-0.0879548	0	0	1
cg10995649	-0.2024494	-0.121963	0.06792681	1	0	0
cg14162806	0.10156974	-0.0034154	-0.0875857	0	0	1
cg04151444	0.12057134	0.03835509	-0.029406	1	0	0
cg20267408	-0.1642808	-0.0691256	0.13091015	1	0	0
cg01798813	0.3535299	0.10097457	-0.1069846	1	0	0
cg04447623	0.11948292	0.02019721	-0.1103098	1	0	0
cg14782678	0.17567136	0.08666825	-0.0349624	1	0	0
cg05468843	-0.3408507	-0.0611537	0.3504748	1	0	0
cg14093610	-0.1937788	-0.0409882	0.15453035	1	0	0
cg09183284	-0.1425979	-0.041088	0.09027831	1	0	0
cg03574735	0.17279235	0.05535228	-0.037991	1	0	0
cg00633339	-0.156829	-0.0681753	0.06448227	1	0	0
cg00220225	0.22099745	0.06120868	-0.0312673	1	0	0
cg25533100	-0.1398795	-0.0550624	0.05133461	1	0	0
cg18003644	-0.1851393	-0.0633122	0.1150384	1	0	0
cg00292336	0.161808	-0.0057803	-0.1081676	0	0	1
cg17485531	0.0897347	-0.0163677	-0.0907619	0	0	1
cg18417562	-0.178843	-0.0114991	0.13272037	1	0	0
cg22739554	-0.1812786	-0.0551518	0.10778124	1	0	0
cg18015427	-0.1818328	0.05266688	0.20651303	0	0	1
cg17717565	0.2229714	NA	NA	NA	NA	NA
cg19304100	-0.1468903	-0.1092476	0.10237486	1	0	0
cg14773011	-0.1245625	0.01146913	0.09396672	0	0	1
cg10418276	-0.2070833	0.00058404	0.16653116	0	0	1
cg26318502	-0.1427832	-0.127478	0.01419863	1	0	0
cg22364991	-0.1856966	-0.0117345	0.14500865	1	0	0
cg05386577	-0.1305419	-0.0388835	0.09298004	1	0	0
cg15458313	-0.1576972	-0.0115713	0.07053994	1	0	0
cg19247877	-0.1431425	-0.032774	0.04063857	1	0	0
cg05876737	0.14176021	0.04467936	-0.0475786	1	0	0

cg14484628	0.09302645	0.0001775	-0.0609161	1	0	0
cg11793449	0.09396328	-0.0088252	-0.0690975	0	0	1
cg09444160	0.25333215	0.18465578	-0.0155384	1	0	0
cg07490694	-0.1563985	-0.057424	0.11919853	1	0	0
cg07960587	-0.1529407	-0.074823	0.04999225	1	0	0
cg26771258	-0.1853363	-0.0367218	0.12634093	1	0	0
cg24425958	0.38640479	0.21931493	-0.1068929	1	0	0
cg08004835	-0.1815997	-0.0451858	0.12297744	1	0	0
cg16500497	-0.161519	-0.0597036	0.09196193	1	0	0
cg25957306	-0.1290399	-0.0475869	0.04220124	1	0	0
cg23088318	0.33178899	NA	NA	NA	NA	NA
cg11688402	-0.1478792	0.00061629	0.12629497	0	0	1
cg01137798	-0.1320127	-0.0065795	0.14014717	1	0	0
cg20358574	-0.1396599	-0.0265349	0.09411068	1	0	0
cg17591980	-0.2064679	-0.0708642	0.06435478	1	0	0
cg14080050	-0.1377946	-0.0753586	0.05717432	1	0	0
cg19265972	0.1568377	0.02703071	-0.1008027	1	0	0
cg20669366	-0.1341972	-0.0647337	0.02660737	1	0	0
cg07519811	-0.1327451	-0.0360382	0.09290847	1	0	0
cg14869618	-0.1497993	-0.0318488	0.11028112	1	0	0
cg26181433	-0.1506679	-0.0255706	0.10571094	1	0	0
cg26527490	0.15437045	0.10363375	0.01920823	1	1	1
cg09343676	0.12243863	0.01080534	-0.0529908	1	0	0
cg27509634	-0.1297648	-0.0569184	0.04356517	1	0	0
cg27131955	0.16673967	0.02326928	-0.069148	1	0	0
cg18657788	-0.1601078	-0.0469972	0.08384202	1	0	0
cg26055019	0.12796508	0.09093218	-0.0270908	1	0	0
cg24713838	0.15015841	0.119915	-0.0622921	1	0	0
cg10778736	0.13014301	0.04774011	-0.0710418	1	0	0
cg10228677	-0.1667459	-0.018483	0.1199049	1	0	0
cg19237879	0.136261	0.06064077	-0.0454538	1	0	0

cg08288223	0.19617139	0.03816346	-0.1100541	1	0	0
cg02141358	-0.1571632	-0.1368446	0.05591185	1	0	0
cg06891344	-0.1679247	0.01655627	0.16291954	0	0	1
cg25308531	0.13286662	0.00770378	-0.090455	1	0	0
cg21284750	0.10644547	0.03423176	-0.0286025	1	0	0
cg03946834	0.13683157	0.05835744	-0.0426135	1	0	0
cg08291506	0.10829119	0.0049092	-0.0360974	1	0	0
cg20739309	-0.3019735	-0.2268664	0.01088618	1	0	0
cg23013137	0.10280476	0.01969417	-0.0439899	1	0	0
cg26834542	-0.1672454	-0.086296	0.08261518	1	0	0
cg24912565	0.12875435	0.02454671	-0.0647467	1	0	0
cg06483739	-0.1586476	-0.0314704	0.10983472	1	0	0
cg05360373	-0.1631172	-0.0998855	0.01679837	1	0	0
cg01437998	-0.1403007	-0.1175788	0.03702163	1	0	0
cg04313719	-0.1461883	-0.0274465	0.14448305	1	0	0
cg17909977	-0.2330364	-0.1556987	0.0239339	1	0	0
cg17569303	-0.1487387	-0.0654727	0.06261297	1	0	0
cg02086349	0.12984056	0.02151274	-0.097561	1	0	0
cg05054673	-0.1635206	-0.0829381	0.10596886	1	0	0
cg11432540	-0.169103	0.02093524	0.1648915	0	0	1
cg09218258	-0.1513914	-0.0581313	0.10735342	1	0	0
cg08969102	0.17957926	-0.028501	-0.1268603	0	0	1
cg01109729	-0.1320981	-0.0397154	0.09945019	1	0	0
cg00188327	-0.1963198	-0.0215246	0.11735823	1	0	0
cg25485930	0.27459809	0.2295867	0.0163757	1	1	1
cg19565757	0.17565938	0.04210882	-0.0653614	1	0	0
cg14960238	0.14695955	0.04199113	-0.0541159	1	0	0
cg09504658	-0.1447581	-0.0656829	0.07712176	1	0	0
cg12344589	-0.1830612	-0.0469553	0.17430154	1	0	0
cg12508286	0.21142726	0.19510885	-0.0221402	1	0	0
cg16323609	0.11849591	-0.0070471	-0.0673128	0	0	1

cg01715384	0.11236158	0.02534491	-0.0518312	1	0	0
cg13614383	-0.2311797	-0.0414173	0.1275069	1	0	0
cg09480190	0.3417248	-0.070037	-0.2899079	0	0	1
cg11729950	-0.1251594	-0.0910309	0.01805143	1	0	0
cg16219384	-0.147187	-0.0764764	0.06031596	1	0	0
cg04999025	0.11358519	0.0672042	0.01215911	1	1	1
cg25790531	-0.1962326	-0.0379156	0.12034882	1	0	0
cg21783716	-0.1408	-0.031961	0.10776781	1	0	0
cg02326273	-0.1968934	0.0276401	0.19465437	0	0	1
cg01539133	0.20449534	0.1000029	-0.0434924	1	0	0
cg13584406	-0.2087649	-0.0844164	0.05470201	1	0	0
cg19038540	0.34555334	0.06632646	-0.1775089	1	0	0
cg00068571	-0.174991	-0.0889957	0.11137521	1	0	0
cg04436877	0.19730933	0.14808766	-0.0002277	1	0	0
cg13250847	0.13460439	-0.0142422	-0.0958334	0	0	1
cg22797971	0.12412315	0.02264868	-0.0588485	1	0	0
cg04898487	-0.1864806	-0.0291433	0.17123115	1	0	0
cg08230856	-0.1357859	-0.1064618	-0.0060083	1	1	1
cg03668686	-0.1619803	-0.0620574	0.07242904	1	0	0
cg18446861	-0.1249813	-0.0742442	0.02326244	1	0	0
cg11142038	-0.1376933	-0.0925121	0.05225176	1	0	0
cg10335612	-0.1438613	-0.0448794	0.12293172	1	0	0
cg25070692	-0.1626637	-0.0751535	0.09204917	1	0	0
cg04195136	0.27458165	0.07083081	-0.1438572	1	0	0
cg03637218	-0.2055633	-0.0139732	0.14629369	1	0	0
cg05753799	-0.1308637	-0.0517956	0.07662144	1	0	0
cg18068929	-0.1652343	-0.0584058	0.13521614	1	0	0
cg12954619	0.11805998	0.0853118	-0.0405447	1	0	0
cg20393839	0.13511826	0.02707039	-0.0558965	1	0	0
cg05296619	-0.1037809	-0.002144	0.06889927	1	0	0
cg14823860	0.14671433	-0.0180069	-0.13328	0	0	1

cg18022607	-0.1600004	-0.0580895	0.09468074	1	0	0
cg07019697	-0.1284612	-0.0112845	0.10446011	1	0	0
cg06748889	-0.160702	-0.0846036	0.04911766	1	0	0
cg06389955	-0.1373115	-0.0377176	0.06401012	1	0	0
cg01656853	0.12832315	0.03417457	-0.0489041	1	0	0
cg11070531	-0.137246	-0.0196948	0.11010727	1	0	0
cg13645296	0.2162076	-0.0175954	-0.1350035	0	0	1
cg13587621	0.19860527	0.05694902	-0.0729426	1	0	0
cg16760899	0.33009311	0.20230611	0.1041012	1	1	1
cg08989195	-0.1341099	-0.046494	0.09233803	1	0	0
cg16371949	-0.2329192	-0.059164	0.13094604	1	0	0
cg09275205	-0.2075609	-0.0694009	0.15640168	1	0	0
cg23684449	0.09343885	-0.0085999	-0.0640292	0	0	1
cg03060158	0.14626942	-0.0467564	-0.1174581	0	0	1
cg26158288	0.162543	0.07132	-0.0531498	1	0	0
cg04180623	-0.1246297	-0.0613879	0.05400539	1	0	0
cg23332328	0.12847243	0.0065285	-0.1219633	1	0	0
cg14871186	0.08568678	0.003436	-0.0419117	1	0	0
cg02614847	0.0769589	0.03870441	-0.0003352	1	0	0
cg03774275	0.21941521	-0.0093144	-0.116045	0	0	1
cg24527098	0.29267451	0.20518427	-0.0435032	1	0	0
cg03365496	0.16817507	0.160364	0.0152365	1	1	1
cg01319403	0.16061849	0.05288229	-0.0666962	1	0	0
cg06490962	-0.1261221	0.01479631	0.09413144	0	0	1
cg05704183	-0.2064241	-0.0926901	0.065203	1	0	0
cg20810702	0.12681529	0.00853586	-0.1093231	1	0	0
cg25697727	0.30142781	0.18329547	-0.1407007	1	0	0
cg15756507	0.14303432	0.04499835	-0.0388589	1	0	0
cg03023732	-0.2043231	0.02371778	0.11042477	0	0	1
cg18109427	-0.1570952	-0.0551853	0.09624271	1	0	0
cg19043851	-0.1517843	-0.0974253	0.08966952	1	0	0

cg17131606	-0.1326425	-0.0346093	0.06837298	1	0	0
cg24915409	-0.2121445	-0.0766008	0.03430786	1	0	0
cg23457203	-0.1584009	-0.0646448	0.08515224	1	0	0
cg23530133	0.10910043	0.00591042	-0.0751565	1	0	0
cg08709434	0.18475747	0.06404656	-0.027176	1	0	0
cg10018232	-0.1905535	0.08557335	0.24394622	0	0	1
cg07583563	0.10422061	0.00728553	-0.0221662	1	0	0
cg06559756	0.12750509	0.02806542	-0.0656912	1	0	0
cg00933914	-0.1385509	-0.0753934	0.06078449	1	0	0
cg08508763	0.24422495	0.07654968	-0.0830164	1	0	0
cg00593333	0.10107843	0.05304637	-0.0581342	1	0	0
cg16165759	-0.158695	-0.0668224	0.11005148	1	0	0
cg06835483	0.109769	0.06470357	-0.0351505	1	0	0
cg13739348	-0.1855352	-0.0342192	0.1369702	1	0	0
cg17299745	0.13738205	-0.0137778	-0.0999599	0	0	1
cg19412131	-0.2281205	-0.0769177	0.1650108	1	0	0
cg15599182	-0.1407481	-0.019898	0.08753708	1	0	0
cg23956385	0.09433713	0.02913457	-0.0429178	1	0	0
cg14084896	-0.172573	-0.1362938	-0.0160531	1	1	1
cg10214540	-0.1919052	-0.1564049	0.03052036	1	0	0
cg10231978	-0.1773127	-0.0465743	0.09993414	1	0	0
cg23007418	0.12069341	0.00735868	-0.060284	1	0	0
cg04337153	-0.1562248	-0.0134721	0.09965772	1	0	0
cg13367729	-0.1619078	NA	0.05486677	NA	0	NA
cg27377644	-0.1501812	-0.0869054	0.05457309	1	0	0
cg05282030	0.13368859	-0.0097064	-0.1124207	0	0	1
cg09017660	-0.1337639	-0.0273427	0.08801785	1	0	0
cg25215011	-0.1295616	-0.014236	0.14849818	1	0	0
cg06748146	0.15681728	0.09838338	-0.0311579	1	0	0
cg24788999	-0.1447833	-0.0460308	0.05213412	1	0	0
cg13685026	-0.2011961	-0.0473768	0.20513779	1	0	0

cg05525416	-0.142525	-0.039493	0.1410879	1	0	0
cg27402811	-0.1442277	-0.1201276	0.0310396	1	0	0
cg20205586	0.13589395	0.00972822	-0.040991	1	0	0
cg17865604	-0.2195382	-0.1966473	-0.0259734	1	1	1
cg14120215	0.41972948	0.15771547	-0.3241138	1	0	0
cg13689390	-0.129537	-0.0571866	0.04663551	1	0	0
cg07092213	0.42638867	0.19338021	-0.1926175	1	0	0
cg00860225	0.13074473	0.09201085	-0.0294281	1	0	0
cg05685493	0.16729939	0.04129422	-0.0658359	1	0	0
cg04338129	0.15234834	0.05464642	-0.0937022	1	0	0
cg07068187	0.11180532	0.03363124	-0.0012624	1	0	0
cg20382920	-0.1902653	0.01061797	0.14570891	0	0	1
cg09781514	-0.2003781	-0.0157532	0.1022178	1	0	0
cg01347529	0.22426191	0.14433298	-0.0268688	1	0	0
cg07755718	-0.1245414	-0.0609046	0.03776619	1	0	0
cg01467882	0.1916345	0.16316206	0.05937235	1	1	1
cg25084760	-0.1994706	-0.1750576	-0.0377936	1	1	1
cg26109401	-0.1501142	-0.0404849	0.11438531	1	0	0
cg23791076	0.14856117	0.06184314	-0.071158	1	0	0
cg02290349	0.24182997	0.00953296	-0.0544642	1	0	0
cg21137052	0.17840461	0.07473255	-0.0486881	1	0	0
cg16276856	-0.1561282	0.00383518	0.14244644	0	0	1
cg09519327	0.3289578	0.2854635	-0.0617065	1	0	0
cg18995064	0.11345282	0.04045151	-0.0335743	1	0	0
cg02722614	-0.1593763	-0.1203989	-0.0010425	1	1	1
cg16547027	0.12588085	0.07414342	-0.0411225	1	0	0
cg03336997	-0.189533	-0.0472185	0.14169376	1	0	0
cg24474561	0.14291907	0.08436958	-0.0555964	1	0	0
cg20647118	0.24589674	0.08721466	-0.1500509	1	0	0
cg11270237	0.14287494	0.09254156	-0.0732749	1	0	0
cg21625740	-0.1309478	-0.0483266	0.0644357	1	0	0

cg04241623	0.52486506	0.35801083	-0.0659435	1	0	0
cg00429509	-0.1612582	-0.0608029	0.06138662	1	0	0
cg26463289	-0.1162753	-0.0169347	0.11282866	1	0	0
cg00518702	0.14636224	0.03847378	-0.0796118	1	0	0
cg12294213	0.13828056	-0.0462256	-0.1617763	0	0	1
cg16606773	-0.1104888	-0.0029555	0.07126111	1	0	0
cg08840751	-0.1460182	0.00136523	0.12065725	0	0	1
cg06145347	0.11501412	0.02930525	-0.0745679	1	0	0
cg02876191	-0.1689997	0.00842424	0.16625244	0	0	1
cg12178807	0.36565367	0.17693182	-0.092927	1	0	0
cg13213598	0.36849866	0.38504718	0.03013117	1	1	1
cg06379721	0.12249615	0.09805989	-0.0229766	1	0	0
cg17808440	-0.2193141	-0.0324338	0.1042427	1	0	0
cg03094728	-0.179173	0.1152637	0.33079505	0	0	1
cg24716960	-0.1683042	-0.0273506	0.12487047	1	0	0
cg09255716	-0.121625	-0.0546596	0.03980343	1	0	0
cg00319774	0.16028068	0.09169602	-0.0403625	1	0	0
cg22373593	-0.1505625	0.00994461	0.1441886	0	0	1
cg21458828	0.14117278	0.08348335	-0.0556551	1	0	0
cg05646433	0.13695355	-0.0020992	-0.0861129	0	0	1
cg00803692	-0.1559077	-0.0277019	0.18120544	1	0	0
cg23261327	0.23851212	-0.0022928	-0.1292951	0	0	1
cg04303382	0.22458562	0.17172354	-0.0361321	1	0	0
cg22098683	0.13862294	0.01243096	-0.0626917	1	0	0
cg15148156	0.1813633	0.0461939	-0.1012894	1	0	0
cg01838759	0.14231242	0.14799106	0.06428508	1	1	1
cg07851807	0.10747056	0.04443534	-0.0117455	1	0	0
cg18792281	0.1120432	0.06370059	-0.0057335	1	0	0
cg19387137	-0.1527292	0.00352275	0.11739488	0	0	1
cg18702715	-0.1244592	0.01014192	0.1220068	0	0	1
cg26906466	-0.2134743	-0.1242087	0.03760476	1	0	0

cg11214064	-0.1192079	-0.0450359	0.03843428	1	0	0
cg16570051	0.11584278	0.06388244	-0.0490542	1	0	0
cg06489965	0.17925961	0.12089116	-0.0566048	1	0	0
cg22339837	0.15498369	0.02088841	-0.1075204	1	0	0
cg06782865	-0.1521342	-0.0387887	0.15996454	1	0	0
cg16556868	-0.1591063	-0.131959	0.01241528	1	0	0
cg07412002	-0.1517793	-0.0194633	0.07812171	1	0	0
cg08544192	-0.1524598	-0.0467906	0.13477376	1	0	0
cg05558046	0.09072948	-3.53E-05	-0.0799312	0	0	1
cg09865411	0.20789651	0.13831953	-0.0926495	1	0	0
cg00797434	-0.1513717	-0.0257375	0.13276495	1	0	0
cg04505045	0.11703349	0.01287958	-0.0891738	1	0	0
cg10787440	-0.1419393	-0.0077422	0.04815651	1	0	0
cg15811468	0.33559618	0.17206323	-0.104039	1	0	0
cg15613488	0.31093299	0.07360694	-0.1580033	1	0	0
cg19551115	-0.1375832	-0.0471543	0.08902769	1	0	0
cg24466850	0.21562169	0.10205436	-0.0632449	1	0	0
cg26139975	0.23989539	0.02268915	-0.1782442	1	0	0
cg19733736	0.14196023	0.05130583	-0.0622309	1	0	0
cg26318532	-0.3053628	-0.2009441	0.06572185	1	0	0
cg00292334	-0.2042156	0.00919906	0.15908501	0	0	1
cg21968765	0.19659178	0.03817283	-0.1137588	1	0	0
cg15400629	0.11451944	0.05257964	-0.0542429	1	0	0
cg01327313	-0.1377108	-0.0669851	0.03229146	1	0	0
cg01908419	0.11887182	0.02372672	-0.0668959	1	0	0
cg16478616	-0.1436951	-0.0370317	0.06345253	1	0	0
cg26320520	0.13227774	0.02229668	-0.1007497	1	0	0
cg16699198	-0.1497615	-0.0355992	0.06574741	1	0	0
cg08727202	0.08687342	0.02638705	-0.0302071	1	0	0
cg16543024	-0.1329777	-0.0399395	0.07065557	1	0	0
cg14021899	0.0929884	-0.0324769	-0.0882414	0	0	1

cg18518720	0.19711279	0.03364477	-0.198074	1	0	0
cg14180680	-0.1459479	-0.0536782	0.12052957	1	0	0
cg05572410	0.18359077	0.05451603	-0.1197497	1	0	0
cg01945501	-0.1439507	-0.0498155	0.0929487	1	0	0
cg21017385	0.18358474	0.08117839	-0.0913521	1	0	0
cg08140389	-0.1426992	-0.0408551	0.10719837	1	0	0
cg15922651	-0.1380856	-0.0517749	0.10959664	1	0	0
cg18367488	0.21720891	0.00876465	-0.1575025	1	0	0
cg12183789	-0.139247	0.02639954	0.13549582	0	0	1
cg05267294	-0.1701805	-0.0410732	0.11297745	1	0	0
cg20920045	-0.1578657	-0.0683785	0.08094691	1	0	0
cg26162435	-0.1704067	-0.0692168	0.11541226	1	0	0
cg02660895	-0.134412	-0.0444912	0.05715119	1	0	0
cg12445832	0.14821226	0.2147276	0.05281854	1	1	1
cg07832591	-0.2092937	NA	0.16049851	NA	0	NA
cg20815919	0.09565653	-0.0024248	-0.077506	0	0	1
cg10148702	-0.1838219	-0.0564989	0.05135434	1	0	0
cg10633128	0.11288898	-0.0075688	-0.0928483	0	0	1
cg03232933	0.17098353	0.10801518	-0.0022472	1	0	0
cg06856216	0.18069463	0.03081322	-0.0937803	1	0	0
cg07488574	-0.1319087	0.01115136	0.11010702	0	0	1
cg21256257	0.28643209	0.17925306	-0.0546526	1	0	0
cg10250828	-0.1521718	-0.0415625	0.0956344	1	0	0
cg19218072	-0.1741707	-0.0093179	0.19689665	1	0	0
cg17409313	0.15158401	0.06352431	-0.0345477	1	0	0
cg11845417	0.13332703	0.06021795	0.00625347	1	1	1
cg07529715	0.21548253	0.15887774	0.0073564	1	1	1
cg24666085	-0.2059996	-0.1120293	0.07205327	1	0	0
cg04223227	0.1539372	0.01706393	-0.0993316	1	0	0
cg27281093	0.21453039	0.06839322	-0.113231	1	0	0
cg22897419	-0.1565251	-0.0480419	0.08911449	1	0	0

cg02965553	0.10593319	0.02143534	-0.0355896	1	0	0
cg24558747	-0.1796487	-0.075757	0.0429077	1	0	0
cg24014202	0.17039046	0.07863449	-0.0635028	1	0	0
cg02628787	0.11806523	0.01760132	-0.084441	1	0	0
cg05928239	0.17014762	0.15485331	-0.1031912	1	0	0
cg08017142	-0.1727921	-0.1509397	-0.0483896	1	1	1
cg13226907	0.1297507	0.0687203	-0.0296125	1	0	0
cg26924223	0.15829243	0.0334675	-0.1017235	1	0	0
cg10806711	0.14629116	0.10114888	0.01586807	1	1	1
cg03974563	-0.1451649	-0.1221655	0.01848287	1	0	0
cg07765814	-0.1953587	-0.0028482	0.1549248	1	0	0
cg18852096	0.27642372	0.14240923	-0.0923243	1	0	0
cg08223291	0.14783342	0.1171885	-0.0228634	1	0	0
cg05938218	-0.1254245	-0.0843992	0.00109697	1	0	0
cg03771840	0.23074264	0.21193691	0.01160998	1	1	1
cg07176561	-0.1453384	-0.0715422	0.0720834	1	0	0
cg16051727	-0.1421904	0.01450264	0.16189493	0	0	1
cg10555800	0.21375729	0.04483365	-0.1473173	1	0	0
cg20678082	0.1908983	0.05826729	-0.1427843	1	0	0
cg21695020	0.20155011	0.14392341	-0.0063047	1	0	0
cg24517604	0.19884565	0.01945199	-0.1008877	1	0	0
cg25695172	-0.1568201	-0.1129609	0.00287324	1	0	0
cg19585816	-0.1486846	0.01477151	0.16672835	0	0	1
cg16406315	0.0856349	0.02703154	-0.0231391	1	0	0
cg02650908	0.17052593	0.0907886	-0.0385175	1	0	0
cg23702716	-0.1065475	-0.0660898	0.01590209	1	0	0
cg02880508	0.12733382	-0.0500278	-0.1329871	0	0	1
cg20157127	0.14746431	0.06362797	-0.0070668	1	0	0
cg17944801	-0.1573619	-0.0610965	0.12611673	1	0	0
cg04858164	-0.1919262	-0.0566005	0.02782432	1	0	0
cg21222556	-0.1557952	-0.1038776	0.06461546	1	0	0

cg15676040	0.2129053	0.1309305	-0.1098577	1	0	0
cg11942603	0.18483633	0.01335023	-0.2615708	1	0	0
cg01914428	0.12826138	0.03198787	-0.0676961	1	0	0
cg03090145	-0.2288333	-0.0016566	0.13488178	1	0	0
cg07412562	-0.1657506	-0.0880837	0.08641479	1	0	0
cg12829473	0.12481384	0.04982815	-0.0323448	1	0	0
cg26334832	0.21306968	0.10862923	-0.0994359	1	0	0
cg02457145	-0.209868	-0.1144682	0.07502738	1	0	0
cg13767707	0.1469681	0.02508191	-0.0655054	1	0	0
cg03045620	0.14633375	-0.0073551	-0.0552481	0	0	1
cg15266658	0.08706084	0.0088011	-0.0315162	1	0	0
cg03699904	0.15010632	0.12342129	0.01787254	1	1	1
cg12104982	0.3336726	0.00987022	-0.1155416	1	0	0
cg24343835	-0.1948632	-0.2486377	0.02064827	1	0	0
cg22674251	-0.130868	-0.1213473	0.03059245	1	0	0
cg07357891	-0.1548101	-0.058625	0.08693699	1	0	0
cg11970192	0.30389249	0.11935774	-0.0511515	1	0	0
cg10934870	-0.2247429	0.00057021	0.15051318	0	0	1
cg12073779	0.32644201	0.22817611	-0.1404503	1	0	0
cg00565113	-0.1673207	-0.0800994	0.1219369	1	0	0
cg09110314	-0.1343168	-0.0300764	0.14545214	1	0	0
cg06303936	-0.1298416	0.00453943	0.11403759	0	0	1
cg01887689	0.15778278	0.09065507	0.04715938	1	1	1
cg07532405	-0.1160948	-0.027975	0.05588984	1	0	0
cg11206788	-0.1356818	-0.0511849	0.05907367	1	0	0
cg22955595	-0.125007	-0.0498188	0.08308356	1	0	0
cg00885460	0.16986993	0.09249588	0.00753094	1	1	1
cg22695117	0.21413866	-0.047033	-0.2470226	0	0	1
cg15277588	-0.174601	0.02111578	0.15368233	0	0	1
cg10154487	-0.1576609	-0.0840045	0.0547936	1	0	0
cg20137410	0.15891901	0.05468823	-0.0782748	1	0	0

cg18936052	0.12009694	-0.0123452	-0.1229063	0	0	1
cg14478368	-0.1627046	-0.1079771	0.06800883	1	0	0
cg01989456	0.12552114	-0.0114225	-0.0772035	0	0	1
cg08979870	-0.1929246	-0.0535058	0.10642696	1	0	0
cg16410508	0.1087172	0.0108611	-0.0421846	1	0	0
cg17631924	0.1407276	-0.0088402	-0.0976319	0	0	1
cg25555847	-0.1504661	0.01035935	0.12293106	0	0	1
cg01976224	-0.3031049	-0.0125113	0.22143651	1	0	0
cg14158827	0.08750104	0.02517946	-0.0432882	1	0	0
cg15756091	-0.161031	0.07334282	0.19215434	0	0	1
cg03250742	0.16315956	0.14022544	0.02402281	1	1	1
cg24515946	-0.1899695	0.01578467	0.18011216	0	0	1
cg00498529	-0.1338048	-0.0554447	0.11043238	1	0	0
cg26395115	-0.1339976	-0.067457	0.04518464	1	0	0
cg04458670	0.2190552	0.05652864	-0.1474069	1	0	0
cg19850503	-0.1352188	-0.0580577	0.05363176	1	0	0
cg16251647	-0.1409811	-0.0713427	0.09232928	1	0	0
cg04339041	-0.1286915	-0.080261	0.01654998	1	0	0
cg15190788	-0.1291365	0.00357617	0.11900429	0	0	1
cg13859764	0.31260527	0.05274197	-0.0979515	1	0	0
cg23689615	0.11524653	-0.0019994	-0.0508987	0	0	1
cg02187276	-0.1203408	-0.0397866	0.06704147	1	0	0
cg00575501	-0.1345099	-0.0728635	0.04358219	1	0	0
cg13993274	0.15382692	0.07960441	-0.0593727	1	0	0
cg22584127	-0.1182044	-0.034139	0.10086401	1	0	0
cg25918510	-0.424506	-0.2295892	0.1170967	1	0	0
cg24599329	-0.2277327	-0.1252416	0.08118095	1	0	0
cg27625732	0.13118715	-0.0118881	-0.0984304	0	0	1
cg27539046	0.35477102	0.25907725	0.03876001	1	1	1
cg03958364	-0.1321304	-0.0323144	0.0582442	1	0	0
cg05132851	-0.1741361	-0.2011589	0.07956928	1	0	0

cg00517117	-0.1602352	-0.0332237	0.07469544	1	0	0
cg23743252	0.27164514	0.09666216	-0.1681698	1	0	0
cg12463140	-0.2652343	-0.1102949	0.1487075	1	0	0
cg21389750	0.15300049	0.0822211	-0.0365794	1	0	0
cg09137630	0.15897745	0.20107215	0.07707052	1	1	1
cg15777890	-0.1679513	-0.0649375	0.13118559	1	0	0
cg07405121	0.14811137	0.01727213	-0.0787165	1	0	0
cg07945323	-0.1278278	-0.0193167	0.10881438	1	0	0
cg02897707	0.19099978	0.08010047	-0.0699286	1	0	0
cg16921950	0.15355642	0.07055073	-0.0987709	1	0	0
cg24587879	0.22515218	0.10174093	-0.1665695	1	0	0
cg21191743	0.09258793	0.02527525	-0.0584274	1	0	0
cg10475014	0.37888151	-0.0608407	-0.3718554	0	0	1
cg03443943	-0.17627	-0.1263648	0.06718656	1	0	0
cg00492341	-0.1707048	-0.0951213	0.03830656	1	0	0
cg01031101	0.44962772	0.21119048	-0.18657	1	0	0
cg05377527	0.1054946	-0.0137165	-0.0935882	0	0	1
ch.4.12604R	-0.531554	-0.4578519	0.27200079	1	0	0
cg15338967	0.115293	0.0450557	-0.0473225	1	0	0
cg17781418	-0.2113438	-0.0614585	0.09698596	1	0	0
cg12309114	-0.1326912	-0.0823737	0.04905042	1	0	0
cg06621309	0.56969586	0.1117412	-0.3181656	1	0	0
cg12717533	0.0907804	0.03846709	-0.0436893	1	0	0
cg17927434	0.12329335	0.15520475	0.06108147	1	1	1
cg17498476	-0.1594643	-0.0623607	0.16859415	1	0	0
cg27110790	0.20389859	0.14329556	-0.0246486	1	0	0
cg05183211	-0.1544808	-0.0942362	0.05896011	1	0	0
cg05106778	-0.1514583	-0.0759517	0.07336876	1	0	0
cg02281167	0.11205258	0.07566699	-0.0292791	1	0	0
cg06657841	-0.1218991	-0.0206052	0.06760613	1	0	0

cg16263722	0.161145	0.03567163	-0.0672082	1	0	0
cg03060590	0.65149044	0.42547213	-0.3462979	1	0	0
cg10125918	-0.2199804	-0.0938159	0.1053876	1	0	0
cg15767529	0.11083821	0.05198524	-0.0212107	1	0	0
cg12423387	-0.1271967	-0.0958266	0.01472455	1	0	0
cg27426267	0.10113066	-0.0456651	-0.1048326	0	0	1
cg11427329	0.10791044	0.03304253	-0.0086493	1	0	0
cg04165922	-0.2033128	-0.0548945	0.07372983	1	0	0
cg05220510	-0.1446657	-0.0982363	0.0668446	1	0	0
cg27628536	0.18900995	-0.0140068	-0.2175429	0	0	1
cg02081431	0.08378534	0.01164215	-0.0539012	1	0	0
cg17110400	0.13331441	0.03358827	-0.0564131	1	0	0
cg08334830	0.15185542	0.00562312	-0.0852822	1	0	0
cg15755673	-0.1282187	-0.0953018	0.03841099	1	0	0
cg16435459	0.11480623	0.02837141	-0.0367344	1	0	0
cg02315509	-0.1425492	-0.0202077	0.12116566	1	0	0
cg09170311	0.14015544	0.01537398	-0.074942	1	0	0
cg13062960	0.1763919	0.02593716	-0.0510259	1	0	0
cg22794803	0.11668669	0.04785343	-0.0150024	1	0	0
cg04672210	-0.1197999	-0.0341384	0.10049186	1	0	0
cg16479957	0.14013814	0.01522071	-0.0765627	1	0	0
cg26491882	-0.1641312	-0.0062973	0.09306826	1	0	0
cg09126636	0.14411045	0.04266331	0.00523652	1	1	1
cg04346128	0.12985635	-0.005503	-0.1122147	0	0	1
cg25247859	0.19103365	-0.0277604	-0.1450825	0	0	1
cg00726151	-0.1715051	-0.0645377	0.0769922	1	0	0
cg13540225	0.1499863	0.05499089	-0.0636626	1	0	0
cg01650347	-0.1597316	-0.0076433	0.08031783	1	0	0
cg14871588	0.14112413	0.04596605	-0.0160718	1	0	0
cg08554115	0.32162463	0.2416807	0.00125798	1	1	1
cg04715503	0.51930129	0.36045669	-0.1437049	1	0	0

cg08046836	-0.1554695	-0.0101604	0.10916229	1	0	0
cg12784704	0.15556753	0.10948749	0.00509415	1	1	1
cg23494863	0.11919013	0.02179117	-0.0898784	1	0	0
cg18709144	-0.1180993	-0.0475243	0.02916275	1	0	0
cg12992268	-0.1645047	-0.0389384	0.10674089	1	0	0
cg17571306	-0.1422069	0.0224861	0.2227142	0	0	1
cg18655410	0.19841519	0.05688514	-0.0687243	1	0	0
cg04194173	-0.1411998	-0.1506619	0.02506314	1	0	0
cg18054492	0.27898453	0.22817898	-0.0908991	1	0	0
cg17495529	-0.2204457	-0.0163101	0.2039142	1	0	0
cg05533549	-0.1275952	0.01694108	0.13422393	0	0	1
cg03860720	-0.1435188	-0.0356043	0.14394074	1	0	0
cg26393036	0.17110091	0.1230315	-0.0065476	1	0	0
cg04361103	0.10917592	-0.0179158	-0.0659537	0	0	1
cg01192291	-0.2041642	-0.1209787	0.0828645	1	0	0
cg17214263	-0.1556253	-0.0752988	0.07570189	1	0	0
cg22079269	0.17093894	-0.0349271	-0.1250399	0	0	1
cg16170767	0.19298665	0.05020388	-0.1868848	1	0	0
cg04046848	0.19812322	0.06167084	-0.0609942	1	0	0
cg17941143	0.15655397	0.09422736	-0.0139456	1	0	0
cg22805341	-0.1478172	-0.035992	0.09694531	1	0	0
cg07999953	-0.1466227	-0.0657092	0.1198848	1	0	0
cg07332511	-0.1459678	-0.094892	0.07277014	1	0	0
cg17219740	-0.1462978	-0.0199097	0.13729292	1	0	0
cg07429804	-0.1521577	-0.0757834	0.07701433	1	0	0
cg12703652	-0.1749795	0.0015824	0.12508001	0	0	1
cg11788604	-0.1854199	0.02425145	0.146362	0	0	1
cg05670219	-0.1489845	-0.0676887	0.0924566	1	0	0
cg09128059	-0.1348744	-0.0358296	0.09925077	1	0	0
cg06794913	0.17164041	-0.0032252	-0.1005472	0	0	1
cg00084864	0.0878477	0.05416759	-0.0391236	1	0	0

cg06689758	0.12734155	0.05636343	-0.045892	1	0	0
cg01756827	0.17336911	0.05588501	-0.0787194	1	0	0
cg09164611	0.15882784	0.02403782	-0.0532686	1	0	0
cg07042338	-0.1576937	-0.1154808	0.02298393	1	0	0
cg16362027	-0.175506	-0.0788894	0.04045792	1	0	0
cg22681148	-0.1451934	-0.0788056	0.04189322	1	0	0
cg17032689	0.10447733	0.00054839	-0.0315202	1	0	0
cg09006366	-0.1941719	-0.1221077	0.10993769	1	0	0
cg25837909	-0.2148724	-0.1241096	0.08581348	1	0	0
cg15804598	0.07966892	-0.0151608	-0.0521531	0	0	1
cg18466162	-0.1847824	-0.02441	0.12188454	1	0	0
cg23357789	-0.162767	0.02490395	0.12562928	0	0	1
cg18733548	0.17089814	0.08795838	-0.036714	1	0	0
cg02296661	-0.1335522	-0.0087788	0.13811918	1	0	0
cg20632567	0.08056038	0.00644093	-0.0466357	1	0	0
cg27016609	0.11614972	0.02844799	-0.0798139	1	0	0
cg09991298	0.15625462	0.12104979	0.0142578	1	1	1
cg16449581	-0.2024747	-0.0098772	0.1058343	1	0	0
cg26513180	0.62307877	0.3497021	-0.1157766	1	0	0
cg07058998	-0.1460712	-0.0220113	0.08853203	1	0	0
cg03984347	0.27118975	-0.0906164	-0.2194876	0	0	1
cg10640894	-0.1566686	-0.0113764	0.11480199	1	0	0
cg12471376	0.12938699	0.00792399	-0.0697387	1	0	0
cg20122123	0.08209295	0.00458148	-0.0601851	1	0	0
cg03379552	0.36954407	0.22936075	-0.2111203	1	0	0
cg11445554	-0.1418107	-0.0207874	0.14976354	1	0	0
cg10805039	0.26051756	0.17823697	-0.085413	1	0	0
cg23759710	0.10550013	-0.0131716	-0.0764584	0	0	1
cg09255383	0.27999854	0.19459949	0.01459209	1	1	1
cg10577793	-0.1242307	-0.0881624	0.00324219	1	0	0
cg06920962	0.09368816	-0.0063081	-0.0539769	0	0	1

cg03805475	0.21379091	0.11731904	-0.0631018	1	0	0
cg10769507	-0.1303707	-0.0366843	0.05661357	1	0	0
cg26104910	-0.2025742	-0.0306808	0.15589058	1	0	0
cg17034390	0.31065204	0.12783268	-0.1162187	1	0	0
cg18832428	-0.1502963	-0.0505301	0.10968369	1	0	0
cg19773296	-0.1190364	-0.0929913	0.01898441	1	0	0
cg08196561	0.38134605	0.24938967	-0.0056617	1	0	0
cg09131617	-0.1519819	-0.0696532	0.12624761	1	0	0
cg04645556	-0.1498509	-0.0052047	0.06861316	1	0	0
cg25302050	-0.1217436	-0.0349893	0.04946221	1	0	0
cg01259156	0.21165981	0.03282975	-0.173243	1	0	0
cg03691549	0.1005287	0.01011795	-0.041604	1	0	0
cg25293251	0.16302872	0.00571917	-0.1411214	1	0	0
cg17591816	-0.1434655	0.05068864	0.1164453	0	0	1
cg09459451	-0.1603127	-0.0242853	0.08258641	1	0	0
cg17118948	-0.1593837	-0.0029742	0.17486252	1	0	0
cg11260662	-0.1946415	-0.0269241	0.06403021	1	0	0
cg19059495	0.18637111	0.00233486	-0.1162126	1	0	0
cg16241033	0.32045261	0.13158611	-0.1333309	1	0	0
cg11786320	0.25796447	0.13689539	-0.1099706	1	0	0
cg22804222	-0.2133778	-0.0055177	0.13456664	1	0	0
cg06465076	-0.1662812	0.00664655	0.10466425	0	0	1
cg19237150	-0.1949399	-0.0053946	0.16290662	1	0	0
cg00178562	-0.1448867	-0.0772077	0.04210361	1	0	0
cg07703358	0.30468738	0.13484287	-0.1659536	1	0	0
cg19380072	0.12155268	-0.010347	-0.0747101	0	0	1
cg15583642	0.10228978	0.05209208	-0.018317	1	0	0
cg05083507	0.19759547	0.0623815	-0.1394293	1	0	0
cg25900192	-0.2076724	-0.0372018	0.06534395	1	0	0
cg22968401	0.12369647	0.01680612	-0.070796	1	0	0
cg24765602	0.15754123	0.12362958	-0.0718291	1	0	0

cg00446046	-0.22377	-0.0284448	0.1139466	1	0	0
cg14869043	-0.1441998	-0.0885451	0.066145	1	0	0
cg17222682	-0.123498	-0.0519333	0.0941064	1	0	0
cg17710952	0.14362404	0.00862469	-0.0556455	1	0	0
cg01954600	-0.2230894	0.02632678	0.18426443	0	0	1
cg19997384	0.21803157	0.05083668	-0.1606848	1	0	0
cg22960962	-0.1277269	-0.0093125	0.05842199	1	0	0
cg16935794	-0.147269	0.00686932	0.12412685	0	0	1
cg25422895	-0.1354481	-0.0958033	0.00889698	1	0	0
cg01493106	0.07867076	0.01599072	-0.0216191	1	0	0
cg10533057	-0.1612292	-0.0560941	0.11065423	1	0	0
cg00861295	-0.110297	-0.1082675	-0.0179921	1	1	1
cg16734913	-0.3621717	-0.1872278	0.1233951	1	0	0
cg05509894	-0.1572128	-0.0212315	0.1088039	1	0	0
cg19500415	-0.1518348	-0.0261288	0.14810728	1	0	0
cg12629991	-0.1363104	-0.0507879	0.11042557	1	0	0
cg14529070	0.11245867	0.07970684	-0.0148675	1	0	0
cg18055610	0.24152528	0.00682908	-0.1996768	1	0	0
cg19085165	-0.2800765	-0.2041198	0.11844934	1	0	0
cg03028611	0.12807007	0.01351321	-0.0879373	1	0	0
cg26491213	0.22190939	0.09752449	-0.0614287	1	0	0
cg26277248	-0.1783197	-0.062751	0.11383066	1	0	0
cg10207609	-0.1623296	0.0226636	0.19733489	0	0	1
cg00564657	0.10652883	0.01891811	0.00818209	1	1	1
cg20098710	0.19057328	0.11832175	-0.0478418	1	0	0
cg00849335	-0.2023097	-0.0328545	0.1174664	1	0	0
cg19168966	-0.1572058	-0.1294941	0.01286622	1	0	0
cg22012583	0.28535436	0.14744487	-0.0349747	1	0	0
cg08075719	0.14181998	0.04298812	-0.0948252	1	0	0
cg26106807	0.08411973	0.03254796	-0.0437727	1	0	0
cg21235823	0.18801505	0.01549379	-0.1032497	1	0	0

cg14061051	0.11974211	0.05231281	-0.0355438	1	0	0
cg27071234	-0.1511299	0.02406511	0.16485422	0	0	1
cg13862900	-0.1484574	-0.0709578	0.04182851	1	0	0
cg05622862	0.32223309	0.06055277	-0.262704	1	0	0
cg22828022	0.1510972	0.06384019	-0.0856128	1	0	0
cg25785733	-0.1255715	-0.0484352	0.07355814	1	0	0
cg27469152	0.14939638	-0.012374	-0.089466	0	0	1
cg18528196	-0.1338762	-0.074992	0.05628737	1	0	0
cg14929207	0.21938661	0.02000685	-0.1015262	1	0	0
cg18512769	0.15737907	0.08892167	-0.1139718	1	0	0
cg05898280	-0.1225067	-0.0837016	0.04190708	1	0	0
cg12820006	-0.1425884	-0.0565093	0.09562548	1	0	0
cg17164249	-0.1097499	0.04952062	0.10702123	0	0	1
cg06392470	-0.1259905	-0.0547641	0.03044962	1	0	0
cg14475527	-0.1611654	-0.0582257	0.14555803	1	0	0
cg24265217	0.1517163	0.01688551	-0.0840313	1	0	0
cg07347315	-0.2323903	-0.0870433	0.13958222	1	0	0
cg01764822	-0.1389669	-0.0981105	0.07917543	1	0	0
cg18735214	0.16920134	0.10859402	-0.008895	1	0	0
cg07914111	-0.1805167	-0.0608444	0.04842443	1	0	0
cg10567964	0.11592638	0.00899509	-0.1020731	1	0	0
cg18932687	0.11605273	0.07097816	-0.0195665	1	0	0
cg24921262	0.10751218	0.03153303	-0.0757809	1	0	0
cg14002042	-0.144058	-0.0172665	0.14465269	1	0	0
cg18561265	-0.1685548	-0.0390401	0.06305303	1	0	0
cg20518572	0.32531173	0.10656183	-0.1498191	1	0	0
cg20147290	0.10991206	0.03203691	-0.0517217	1	0	0
cg10049251	0.10529993	0.01467758	-0.0627054	1	0	0
cg06206989	-0.1263612	-0.0358586	0.0691968	1	0	0
cg02663352	-0.1625056	-0.0515109	0.118555	1	0	0
cg22040874	-0.1508313	-0.0581945	0.06652072	1	0	0

cg18885766	-0.1465206	-0.1137214	0.11858732	1	0	0
cg15119467	-0.1423914	-0.0388167	0.07731541	1	0	0
cg16898283	-0.155225	-0.0719726	0.03018665	1	0	0
cg24221559	-0.1602418	-0.09638	0.01020789	1	0	0
cg10190863	-0.3904318	-0.1369035	0.1915096	1	0	0
cg11086566	0.14635365	0.01715574	-0.0728401	1	0	0
cg20285546	0.19364806	0.14004696	-0.0778882	1	0	0
cg11892080	-0.1281142	-0.0963232	0.01458873	1	0	0
cg22963015	-0.14804	-0.0166295	0.10513886	1	0	0
cg13657884	-0.1251796	-0.0900019	-0.0102103	1	1	1
cg24595611	0.09651919	0.02196646	-0.0364954	1	0	0
cg05488527	-0.1293194	-0.0475397	0.05213005	1	0	0
cg02907319	-0.1328511	-0.056617	0.10443137	1	0	0
cg11180667	-0.1824543	0.00740307	0.17011393	0	0	1
cg22635041	-0.1499793	-0.118282	0.05755469	1	0	0
cg03780875	0.15965376	-0.0431415	-0.1870898	0	0	1
cg12487205	-0.1423593	-0.0790551	0.07874112	1	0	0
cg11522683	-0.2076595	-0.0452277	0.21794892	1	0	0
cg06004232	0.1041541	0.01443417	-0.0775013	1	0	0
cg14943837	0.13428867	-0.0105951	-0.0930283	0	0	1
cg08409465	0.23502205	0.13878865	-0.0088296	1	0	0
cg26079959	-0.2785296	-0.1660973	0.1639858	1	0	0
cg07589968	0.08537208	0.01003206	-0.0768092	1	0	0
cg03290223	-0.1840252	-0.0431089	0.14661624	1	0	0
cg15881077	0.17384238	0.05382233	-0.0198989	1	0	0
cg16215960	-0.1440714	-0.0125325	0.11793119	1	0	0
cg21644701	0.09910456	0.00571326	-0.0609047	1	0	0
cg02473195	0.19918759	0.09586588	-0.0027933	1	0	0
cg27585268	-0.1283111	-0.0005575	0.06284978	1	0	0
cg22178280	0.28485953	0.17240666	-0.0473755	1	0	0
cg24468939	-0.1301143	-0.0411365	0.08338431	1	0	0

cg27338840	-0.2044889	NA	0.08556686	NA	0	NA
cg22696426	-0.1645402	-0.0781727	0.09991177	1	0	0
cg22411536	-0.1657017	NA	NA	NA	NA	NA
cg24501036	0.10111962	-0.0123326	-0.1143096	0	0	1
cg10422845	0.16969682	0.05701782	-0.1183185	1	0	0
cg00852816	-0.1239419	-0.0751768	0.02918406	1	0	0
cg08462030	-0.1539708	-0.057426	0.10071195	1	0	0
cg11310044	-0.1179442	-0.0324544	0.02674498	1	0	0
cg17042037	0.21720577	0.13020665	-0.06592	1	0	0
cg05481881	-0.1467423	-0.0138494	0.20261193	1	0	0
cg01770296	0.1691688	0.05858476	-0.0679055	1	0	0
cg24358089	-0.5614074	-0.1378235	0.42558097	1	0	0
cg11215870	0.16483566	0.01791891	-0.0676841	1	0	0
cg08600456	-0.1223356	-0.1072741	0.00137377	1	0	0
cg23125737	-0.145729	-0.0536992	0.08216295	1	0	0
cg04439077	-0.1601597	-0.0758112	0.03784525	1	0	0
cg13113121	-0.2080683	-0.0607184	0.08629926	1	0	0
cg00940972	-0.1393595	-0.0482609	0.05919322	1	0	0
cg18586212	-0.1530888	-0.0067279	0.1333271	1	0	0
cg07014126	-0.1684488	-0.061582	0.09648606	1	0	0
cg12358978	-0.1462844	-0.0638262	0.08499969	1	0	0
cg17094269	0.08383456	0.02790705	-0.0188538	1	0	0
cg12245876	0.12764975	0.00883383	-0.1004033	1	0	0
cg02486845	0.14976962	0.1390654	0.0511153	1	1	1
cg21122437	0.16916889	-0.0175035	-0.1398287	0	0	1
cg15246895	0.09782579	-0.0343971	-0.1039471	0	0	1
cg26417468	-0.1504979	-0.0584778	0.09808345	1	0	0
cg04175248	-0.125529	-0.0584068	0.03687939	1	0	0
cg07164659	0.14795415	0.05730896	-0.0499877	1	0	0
cg14593033	0.15557852	0.0603321	-0.0599015	1	0	0
cg13214422	-0.1319177	-0.0776266	0.04413984	1	0	0

cg25159046	0.14023602	0.02166227	-0.0515412	1	0	0
cg14626259	0.20519287	0.11761659	-0.0845174	1	0	0
cg20562823	-0.1609275	-0.0353306	0.10834566	1	0	0
cg18484130	-0.1546376	-0.0778446	0.07712557	1	0	0
cg10650353	0.14486985	0.09127913	-0.033781	1	0	0
cg19423804	-0.1791385	-0.0728135	0.10568627	1	0	0
cg09620131	0.11792633	0.04067741	-0.1032569	1	0	0
cg18844614	0.08387478	0.00541611	-0.0624558	1	0	0
cg09447225	-0.1647536	-0.0548839	0.11110464	1	0	0
cg01173104	-0.2630008	0.01659543	0.22524584	0	0	1
cg26351746	0.33228475	0.14588235	-0.2634706	1	0	0
cg26692263	0.14672269	0.03958451	-0.0389304	1	0	0
cg14703589	0.13435514	-0.034193	-0.193464	0	0	1
cg09722400	-0.1237664	-0.0406498	0.06957879	1	0	0
cg10537627	-0.2011239	-0.0930955	0.1102066	1	0	0
cg17705735	-0.1308777	-0.0449088	0.12421963	1	0	0
cg19705450	-0.1211154	-0.1161056	0.01110632	1	0	0
cg08563201	-0.1495511	-0.0630474	0.11554986	1	0	0
cg26181894	-0.1503292	-0.1121049	0.14577594	1	0	0
cg13629807	-0.1486161	-0.0305986	0.13485173	1	0	0
cg11967110	-0.1669794	-0.0989715	0.08596567	1	0	0
cg15676528	-0.1373701	-0.0792929	0.02250109	1	0	0
cg06743599	0.16659217	0.0445615	-0.0306696	1	0	0
cg07056242	-0.1233131	-0.0485837	0.02017343	1	0	0
cg10360845	-0.1626351	0.00757144	0.15759512	0	0	1
cg27230882	-0.1018734	-0.0266429	0.06933733	1	0	0
cg21178389	-0.2792396	NA	NA	NA	NA	NA
cg12410124	-0.158873	-0.0372133	0.10999922	1	0	0
cg24936411	-0.1425872	-0.0079802	0.17686389	1	0	0
cg10282491	0.20687604	0.03910388	-0.1683242	1	0	0
cg10425861	0.18466532	0.16304529	0.00530172	1	1	1

cg26483332	0.16787471	0.07520777	-0.0112477	1	0	0
cg09036712	0.07945548	0.0402178	0.0085806	1	1	1
cg05152300	0.21721544	0.03883347	-0.1943397	1	0	0
cg15447685	-0.138299	-0.0355536	0.07970995	1	0	0
cg07992935	-0.2173361	-0.1176425	0.03669077	1	0	0
cg18833383	-0.2490331	-0.220213	0.03942482	1	0	0
cg18333061	0.22331574	0.10482221	-0.1137155	1	0	0
cg15178904	-0.1203806	-0.0756604	0.0240749	1	0	0
cg08770998	-0.1010697	-0.0302472	0.04919891	1	0	0
cg26078436	-0.1452184	-0.0171393	0.09490756	1	0	0
cg00343839	0.47228515	0.32085665	-0.0831662	1	0	0
cg21576256	-0.1303347	-0.0453849	0.03978646	1	0	0
cg23170057	-0.1461746	-0.0584349	0.11703665	1	0	0
cg10529804	-0.1463644	-0.0747911	0.05012474	1	0	0
cg25808797	0.13172302	0.04336515	-0.0114026	1	0	0
cg01995281	0.09235372	0.00751873	-0.0572605	1	0	0
cg18476444	-0.1267147	-0.0882748	0.05703951	1	0	0
cg26240231	0.44679135	0.13988384	-0.1982706	1	0	0
cg10334948	-0.1776141	-0.0998289	0.12406538	1	0	0
cg02151399	-0.1623531	-0.0959846	0.10024648	1	0	0
cg25043986	-0.2161436	-0.1420685	NA	1	NA	NA
cg07505964	-0.129456	-0.0408234	0.08323033	1	0	0
cg03672322	-0.1157928	-0.0708171	0.04551855	1	0	0
cg25142847	-0.1483971	-0.0161459	0.07298729	1	0	0
cg03847893	-0.1786922	-0.0023818	0.09112934	1	0	0
cg07470570	-0.1654869	-0.0709385	0.09878687	1	0	0
cg02722997	-0.1498495	-0.0511445	0.04918294	1	0	0
cg01457080	0.14540702	0.04622567	-0.0332879	1	0	0
cg12367906	-0.2046024	-0.1342233	0.04519781	1	0	0
cg10016185	-0.1390972	-0.0997189	0.02720369	1	0	0
cg16539768	-0.1603856	-0.0776403	0.07481248	1	0	0

cg00930515	-0.1183461	-0.074636	0.01257076	1	0	0
cg01132893	0.27723742	0.0548147	-0.1533452	1	0	0
cg10721728	-0.1245832	-0.0992585	0.01993283	1	0	0
cg04800909	-0.1251789	-0.0656309	0.05414355	1	0	0
cg24478125	-0.1471999	0.06451766	0.13585009	0	0	1
cg22917372	0.10947931	0.02532816	-0.0599822	1	0	0
cg16214582	-0.162279	-0.0954197	0.0696677	1	0	0
cg06255299	0.21329933	0.0206656	-0.0973741	1	0	0
cg14263957	0.13581746	0.007999	-0.1045864	1	0	0
cg19722748	0.17510115	0.0413921	-0.1153156	1	0	0
cg20450082	-0.1516334	-0.0296354	0.17867085	1	0	0
cg07362328	0.1492952	0.15438463	-0.036164	1	0	0
cg14856316	-0.1425409	-0.0275226	0.1609597	1	0	0
cg19931348	-0.1845068	-0.0704813	0.1010935	1	0	0
cg12299771	-0.1372646	-0.0777124	0.10730434	1	0	0
cg05848434	-0.1496524	-0.0526726	0.10447184	1	0	0
cg01285676	-0.1291046	-0.0956859	0.02909403	1	0	0
cg19595239	-0.117227	-0.0467936	0.08800351	1	0	0
cg18569296	-0.1234662	-0.0877553	0.01809368	1	0	0
cg00770599	0.08318451	-0.0018893	-0.0761173	0	0	1
cg12117654	0.145361	0.02925196	-0.0508549	1	0	0
cg10189135	0.13598109	-0.0710052	-0.1667311	0	0	1
cg26645700	0.14365692	0.05418594	-0.0720414	1	0	0
cg09243384	-0.2397405	-0.102344	0.1171959	1	0	0
cg06290690	0.25156378	0.26184203	0.02128089	1	1	1
cg23658362	-0.1364588	-0.0416825	0.09644164	1	0	0
cg09535526	0.23111198	0.00495226	-0.1850477	1	0	0
cg10631173	-0.1303403	-0.0547379	0.05943456	1	0	0
cg20876248	-0.1553299	-0.086619	0.14026228	1	0	0
cg01522717	0.10397188	0.06413244	-0.0395763	1	0	0
cg21852429	0.41453182	0.22583736	-0.1894838	1	0	0

cg11616676	-0.2481452	-0.0418982	0.18720589	1	0	0
cg13385981	0.09779555	-0.0087519	-0.0782904	0	0	1
cg14928241	-0.1296513	0.02211011	0.15547202	0	0	1
cg25959826	-0.1391483	-0.0729287	0.07500011	1	0	0
cg20802710	0.10455863	0.03207009	-0.0398567	1	0	0
cg25348888	-0.1992214	NA	0.13767028	NA	0	NA
cg22816626	-0.1215763	-0.0771556	0.04938577	1	0	0
cg20127957	0.1625133	0.05701265	-0.0829147	1	0	0
cg03131054	0.08516915	0.00995929	-0.0724783	1	0	0
cg04964428	-0.1978546	0.05411285	0.16180333	0	0	1
cg21315358	-0.4888564	NA	NA	NA	NA	NA
cg13628444	0.18961892	0.02635285	-0.1363338	1	0	0
cg07836600	-0.1393692	-0.0732742	0.01946194	1	0	0
cg21931506	0.10031674	0.02714118	0.00033609	1	1	1
cg03382601	-0.1187357	-0.0397691	0.05084858	1	0	0
cg09715906	-0.2015592	0.10659153	0.20776828	0	0	1
cg18883887	-0.1744995	-0.0357792	0.14618207	1	0	0
cg18886630	0.10222708	0.01903938	-0.0526553	1	0	0
cg12493255	0.19018157	0.11593902	-0.0486605	1	0	0
cg04549141	-0.1567856	0.01140575	0.15506658	0	0	1
cg00452639	0.07217823	-0.0025576	-0.0428907	0	0	1
cg15649464	0.12760089	0.04538613	-0.0097625	1	0	0
cg12341047	0.19295686	0.09065211	-0.132611	1	0	0
cg23545098	0.11319984	-0.0178296	-0.0671827	0	0	1
cg13632142	-0.1279421	-0.0406134	0.07707479	1	0	0
cg05965700	0.13845643	0.08536691	0.01422312	1	1	1
cg11651209	0.19647399	0.05561529	-0.1050802	1	0	0
cg17344560	0.08873318	0.02719381	-0.0290843	1	0	0
cg20450401	0.21992517	0.04788327	-0.1251278	1	0	0
cg19455323	-0.1446587	-0.0204057	0.12437415	1	0	0
cg08881516	0.18394962	0.06883903	-0.0828716	1	0	0

cg10199250	0.1163422	-0.0091764	-0.1350235	0	0	1
cg00778525	-0.2489466	-0.1224252	0.1148381	1	0	0
cg18161208	0.11655715	-0.0170046	-0.0813615	0	0	1
cg21669940	-0.131475	-0.009326	0.11530679	1	0	0
cg00291573	-0.1554952	0.0047376	0.19352349	0	0	1
cg22851977	0.24685644	0.14427277	-0.0186575	1	0	0
cg23518541	-0.2079538	-0.0204408	0.18094088	1	0	0
cg23189953	0.12089322	0.00578328	-0.0822299	1	0	0
cg18030133	-0.1265029	-0.0851938	0.01765946	1	0	0
cg23879308	0.09405201	0.00487957	-0.0255496	1	0	0
cg18935506	-0.1741554	-0.0396641	0.07266511	1	0	0
cg26405475	0.18843269	0.15023206	-0.0056787	1	0	0
cg07529381	0.09799725	-0.0288621	-0.0923541	0	0	1
cg20543681	0.14380296	0.02178319	-0.0552363	1	0	0
cg21932542	-0.151277	-0.0575286	0.06733313	1	0	0
cg21497935	0.14308759	0.08985301	-0.0855076	1	0	0
cg10202714	-0.1253148	-0.0139342	0.11618838	1	0	0
cg20176212	0.10210543	0.03391598	-0.0891261	1	0	0
cg22467301	0.11576003	0.0205109	-0.0661924	1	0	0
cg01008102	-0.1825533	NA	NA	NA	NA	NA
cg23157190	0.11035818	0.04806331	-0.034125	1	0	0
cg18667130	-0.1302163	-0.0609381	0.02755669	1	0	0
cg02773624	0.15296346	0.08869142	-0.0149969	1	0	0
cg22972893	0.2339581	-0.002635	-0.1443404	0	0	1
cg17517849	-0.1450312	0.00804519	0.07817176	0	0	1
cg01685428	-0.1709032	-0.0053556	0.09792164	1	0	0
cg23721124	-0.1555198	-0.0353	0.08555886	1	0	0
cg23717459	-0.1186088	-0.0057752	0.09741292	1	0	0
cg08112841	-0.1348196	-0.0442532	0.09144943	1	0	0
cg07206256	-0.1308632	-0.0005272	0.1269511	1	0	0
cg12840325	-0.1400073	-0.0787673	0.06973493	1	0	0

cg10970798	-0.1133332	-0.0896576	0.00073536	1	0	0
cg27490143	0.20683878	0.06139451	-0.0650017	1	0	0
cg02711271	0.07521912	-0.0008517	-0.0410707	0	0	1
cg26063655	-0.1213014	-0.0037334	0.11348523	1	0	0
cg05659265	0.12871113	0.08429893	0.01945822	1	1	1
cg11136040	-0.2028477	-0.0820781	0.07208947	1	0	0
cg07620446	0.15863145	0.05128116	-0.0324296	1	0	0
cg02940093	-0.165865	-0.0746425	0.14556484	1	0	0
cg23906060	0.12928274	0.10171059	-0.0362059	1	0	0
cg23475732	0.11970974	0.12663883	0.05569223	1	1	1
cg01495604	0.11723501	0.06352336	0.02880531	1	1	1
cg04666263	0.07848248	-0.0187111	-0.0661417	0	0	1
cg12525449	-0.2104186	0.10712398	0.27067759	0	0	1
cg26957633	-0.206827	-0.1001762	0.02822259	1	0	0
cg11485154	0.17088309	0.00729032	-0.1273981	1	0	0
cg09653610	0.28981068	0.14583581	-0.1476692	1	0	0
cg23574215	0.08712744	0.00479692	-0.0564632	1	0	0
cg07579284	-0.1254085	-0.0220511	0.04110897	1	0	0
cg06079564	0.17890881	0.21869918	0.05087712	1	1	1
cg09677330	-0.1535834	-0.0922738	0.07672504	1	0	0
cg15698851	-0.2946676	-0.2962116	0.05801535	1	0	0
cg21787176	-0.1537802	0.02533689	0.15436147	0	0	1
cg03397616	-0.1644157	-0.0268901	0.13881212	1	0	0
cg08401618	0.07209499	0.00524947	-0.0452151	1	0	0
cg02665999	-0.1232319	-0.0519885	0.07865664	1	0	0
cg08743514	0.12749163	0.02496853	-0.0776702	1	0	0
cg16007541	0.37443961	0.20704687	-0.1769598	1	0	0
cg05511978	0.17190358	0.23091697	0.03933525	1	1	1
cg19272144	-0.1235857	-0.0278091	0.083872	1	0	0
cg16773312	0.10758401	0.03415663	-0.0347118	1	0	0
cg21103269	-0.1499189	-0.050437	0.10830956	1	0	0

cg08279189	-0.2106981	-0.1452587	0.05125499	1	0	0
cg18538954	-0.1433503	-0.0604453	0.08084896	1	0	0
cg08530537	0.19693205	0.09613762	-0.1368397	1	0	0
cg08126665	-0.1554393	-0.0351334	0.09675813	1	0	0
cg09224452	0.28486936	0.25519619	0.04471469	1	1	1
cg21585766	0.12025999	0.04126438	-0.0888191	1	0	0
cg19934294	0.09478878	0.04377022	-0.0199464	1	0	0
cg14311763	0.1554406	0.03964667	-0.0333209	1	0	0
cg14803335	-0.1319347	-0.016947	0.0771475	1	0	0
cg07685357	0.11248222	0.00523596	-0.0601603	1	0	0
cg22652435	-0.261311	-0.0886364	0.21267293	1	0	0
cg00785312	0.10676706	0.01368032	-0.0727808	1	0	0
cg25695450	0.17314668	0.00701858	-0.1553873	1	0	0
cg19866578	-0.1233639	-0.0117389	0.09892793	1	0	0
cg10770335	-0.140163	-0.0168926	0.089119	1	0	0
cg05486616	-0.1165578	0.01484354	0.12011373	0	0	1
cg20143092	0.22576099	0.13039794	-0.0984176	1	0	0
cg19296400	0.15131822	0.01786805	-0.136531	1	0	0
cg09114528	0.18612023	0.0559882	-0.0234061	1	0	0
cg11352024	-0.1228388	-0.015838	0.08580815	1	0	0
cg25975995	0.11743027	0.02058855	-0.0246456	1	0	0
cg09649901	-0.1383774	-0.0785836	0.02548053	1	0	0
cg17612461	0.10670554	0.05095775	-0.0358143	1	0	0
cg13225865	-0.1486358	-0.0563033	0.06734358	1	0	0
cg21193966	-0.1795462	-0.1325168	0.11873119	1	0	0
cg22989941	-0.136057	-0.0235002	0.12128015	1	0	0
cg22915906	-0.1359024	-0.0311251	0.04716791	1	0	0
cg10995397	0.12300935	0.07677003	-0.0092652	1	0	0
cg08135895	-0.1529363	-0.1002647	0.07909992	1	0	0
cg11317199	0.21637671	NA	0.01580825	NA	1	NA
cg22742001	-0.1738172	-0.0287546	0.15814073	1	0	0

cg26828017	-0.2046989	-0.1004401	0.01031656	1	0	0
cg26231071	-0.1560403	-0.0219765	0.08835263	1	0	0
cg17424856	0.15215847	0.06737849	-0.0462034	1	0	0
cg21480319	-0.1383417	-0.0129339	0.07031481	1	0	0
cg07899997	0.21155473	0.0925955	-0.0897461	1	0	0
cg18805734	0.10653102	0.01564048	-0.0512571	1	0	0
cg15224011	0.11161811	0.05774193	-0.034799	1	0	0
cg04333485	-0.1811805	-0.011004	0.16271407	1	0	0
cg15074047	0.09490849	0.01416117	-0.0682293	1	0	0
cg05544828	-0.1275911	-0.0983375	0.05414459	1	0	0
cg10922280	0.09787292	0.05143786	-0.0393082	1	0	0
cg01964152	0.18825524	0.02819895	-0.1397259	1	0	0
cg07882781	-0.1981361	-0.0227071	0.14647966	1	0	0
cg04277040	0.11308134	0.01398646	-0.0823333	1	0	0
cg16736000	-0.1553087	-0.0740311	0.10655723	1	0	0
cg18141902	0.10010415	0.0019603	-0.0614156	1	0	0
cg03624510	0.14024716	0.00187038	-0.0689791	1	0	0
cg09348353	0.09866422	-0.0586494	-0.1478581	0	0	1
cg06193788	-0.1216939	-0.0741048	0.02412532	1	0	0
cg07223180	0.21337941	0.12874944	-0.0706459	1	0	0
cg18005261	-0.1861713	-0.0772546	0.12887643	1	0	0
cg27333706	0.10943766	0.01778798	-0.0465612	1	0	0
cg15033653	0.09500722	-0.0075252	-0.0665044	0	0	1
cg19968134	0.11757809	0.04992441	-0.0188408	1	0	0
cg11888727	-0.152242	-0.0408248	0.12471162	1	0	0
cg22561487	0.14059118	0.08168595	-0.0597472	1	0	0
cg00872631	-0.1432489	-0.0617681	0.08365319	1	0	0
cg25385939	-0.1366802	-0.0087109	0.10283416	1	0	0
cg14635466	0.12220804	0.06292224	0.02264151	1	1	1
cg26921068	-0.1150911	-0.0400018	0.06770688	1	0	0
cg25370448	-0.2973172	0.00023312	0.26770706	0	0	1

cg00646396	0.168469	0.07711333	-0.009674	1	0	0
cg04071487	-0.2380111	-0.1529658	0.06629605	1	0	0
cg00401161	-0.1295796	-0.0471889	0.05878732	1	0	0
cg16672147	-0.0922448	-0.0679968	0.03090752	1	0	0
cg02308280	0.16421775	0.07757247	-0.0711812	1	0	0
cg17044418	-0.2664546	-0.1279408	0.08099595	1	0	0
cg09900139	-0.1468739	-0.0765764	0.08253569	1	0	0
cg24269863	-0.1401573	-0.1329819	0.04502896	1	0	0
cg22076474	-0.1740731	-0.0967849	0.05469252	1	0	0
cg00533891	0.1957964	0.054775	-0.0838068	1	0	0
cg23311800	-0.1343178	-0.1272228	0.03508006	1	0	0
cg13073141	0.19867413	0.02066492	-0.1343979	1	0	0
cg10471548	-0.184521	-0.020693	0.1184823	1	0	0
cg17139369	0.33615137	NA	NA	NA	NA	NA
cg21130103	-0.1763092	-0.0428316	0.11720514	1	0	0
cg19495022	0.10664855	0.02546929	-0.0777063	1	0	0
cg06255037	0.10425447	-0.012677	-0.0964925	0	0	1
cg11570271	0.1310936	0.03493053	-0.0400346	1	0	0
cg05075268	0.09397671	0.015524	-0.0608332	1	0	0
cg07936975	-0.2067252	-0.0101951	0.20112322	1	0	0
cg18326313	-0.1741883	0.07649535	0.2155985	0	0	1
cg13500499	-0.1304206	-0.0584403	0.07582369	1	0	0
cg22033352	-0.122043	-0.0638696	0.0495825	1	0	0
cg18948913	0.16009667	0.04031625	-0.083341	1	0	0
cg21849126	-0.1421859	-0.0407534	0.16715577	1	0	0
cg03537184	-0.1551517	-0.0677609	0.15193173	1	0	0
cg10256708	0.13133682	0.04184936	-0.0313916	1	0	0
cg23636267	0.12551904	0.0681202	-0.0501137	1	0	0
cg18161904	0.22008019	0.16089657	-0.0383704	1	0	0
cg04459751	0.17839582	-0.0100225	-0.1403652	0	0	1
cg20989225	0.10916921	0.06484915	-0.0740134	1	0	0

cg05944343	-0.1966618	0.03702521	0.13179367	0	0	1
cg08338597	-0.1626799	-0.0747568	0.0982245	1	0	0
cg10108296	0.14236708	0.13897151	0.0233314	1	1	1
cg12477985	-0.1221465	-0.0458159	0.04354477	1	0	0
cg10017260	-0.1553437	-0.0811979	0.06470847	1	0	0
cg05958778	-0.1408577	-0.0079246	0.15676893	1	0	0
cg15402265	0.1347263	-0.0007317	-0.0981624	0	0	1
cg01889083	-0.1403333	-0.0640971	0.07828143	1	0	0
cg14374954	0.10475969	0.06453718	-0.0068964	1	0	0
cg01449754	-0.1715415	-0.0109679	0.22356573	1	0	0
cg11102760	-0.1730488	0.00965585	0.17597485	0	0	1
cg10271836	-0.1305432	-0.0576772	0.05951822	1	0	0
cg22934246	0.1337454	0.03018866	-0.0867084	1	0	0
cg11835197	0.14510796	0.03428625	-0.0598214	1	0	0
cg24310780	-0.1687707	0.09006628	0.25150027	0	0	1
cg09459291	0.1700125	0.09559232	-0.0735389	1	0	0
cg14297914	0.12427865	-0.0266473	-0.06633	0	0	1
cg18483766	-0.1234082	-0.0425191	0.09084433	1	0	0
cg23030937	-0.135714	-0.0045333	0.12758064	1	0	0
cg04023465	-0.22075	-0.1507141	0.09879163	1	0	0
cg00759022	-0.1299385	-0.0667022	0.05272906	1	0	0
cg05849615	-0.1348679	-0.0771518	0.10816401	1	0	0
cg24798968	0.13974874	0.09523057	-0.0471136	1	0	0
cg17345081	-0.1747554	-0.0436397	0.1383608	1	0	0
cg03703356	0.11777955	0.10474826	0.0122975	1	1	1
cg04734865	-0.2015977	-0.0264393	0.15317314	1	0	0
cg15321814	0.11377682	-0.0113958	-0.0566089	0	0	1
cg10248878	0.15304043	0.04671107	-0.1240786	1	0	0
cg16748520	-0.1190914	-0.0559885	0.05360737	1	0	0
cg12214913	-0.1468151	-0.0854428	0.06203384	1	0	0
cg16580197	-0.1916597	0.04791122	0.18385913	0	0	1

cg17987306	-0.1890888	-0.0428646	0.10615045	1	0	0
cg04317263	0.19130148	0.03451061	-0.1052391	1	0	0
cg04995615	-0.142505	-0.0379445	0.14581521	1	0	0
cg04854282	-0.1530233	0.01394481	0.13269541	0	0	1
cg11827925	0.12064487	0.06301599	-0.0147448	1	0	0
cg11970289	0.16813775	0.09160674	-0.0099588	1	0	0
cg01664929	-0.1415938	-0.0393658	0.12606963	1	0	0
cg21644740	0.20078123	0.05555648	-0.0254288	1	0	0
cg02254587	0.13796827	0.05492603	-0.0725549	1	0	0
cg23616761	0.37780795	0.22703862	-0.1713121	1	0	0
cg14275933	-0.1569336	0.0069695	0.16859829	0	0	1
cg16862319	-0.1859982	-0.0354003	0.09159566	1	0	0
cg03106207	0.09465621	0.0308008	-0.0330151	1	0	0
cg25084279	-0.1066975	-0.0596028	0.03961149	1	0	0
cg25064530	-0.1079554	-0.0420565	0.05795582	1	0	0
cg27201889	0.12013415	-0.0016526	-0.0729756	0	0	1
cg20165746	-0.1482321	0.04392456	0.1256662	0	0	1
cg06161398	-0.1472176	-0.0390462	0.07171085	1	0	0
cg14882828	-0.1899405	0.01882624	0.17757468	0	0	1
cg12917573	-0.1467598	-0.0548651	0.11454357	1	0	0
cg23732576	0.09052492	0.01705396	-0.0651709	1	0	0
cg02548289	0.21348879	0.0891277	-0.1222167	1	0	0
cg26647111	0.32314474	0.00212333	-0.2735003	1	0	0
cg13802316	0.21259003	0.13751981	-0.097928	1	0	0
cg00424066	-0.1388065	0.09702709	0.22112313	0	0	1
cg14117065	-0.1229373	-0.0230309	0.10811635	1	0	0
cg20777670	0.20461901	0.19959743	0.06670174	1	1	1
cg03006527	0.08753589	-0.0039595	-0.0497185	0	0	1
cg06161550	-0.4849076	-0.3863122	0.03658027	1	0	0
cg00502575	-0.1325964	-0.0811283	0.03339613	1	0	0
cg04707703	-0.129535	-0.033757	0.09843585	1	0	0

cg12361705	0.10550481	0.04344843	-0.0299897	1	0	0
cg26508681	-0.2157062	-0.0738528	0.2074046	1	0	0
cg04931126	-0.2177531	-0.1115904	0.08699924	1	0	0
cg25990696	-0.1693291	-0.0504815	0.11045531	1	0	0
cg00823843	0.1980287	0.0309328	-0.0907343	1	0	0
cg06168021	-0.1304438	-0.0637575	0.08902326	1	0	0
cg10716909	-0.1476688	-0.0490051	0.05808039	1	0	0
cg17055821	0.26110747	0.0663777	-0.1445449	1	0	0
cg09606588	-0.1429602	0.07610402	0.19689754	0	0	1
cg12700055	-0.1242603	-0.0370009	0.06397373	1	0	0
cg27523087	0.15076748	0.06806943	-0.0248597	1	0	0
cg15358079	0.14730736	0.10966542	0.03929722	1	1	1
cg00018635	-0.1135198	-0.0173355	0.08285402	1	0	0
cg02008651	-0.1757686	-0.0399838	0.04323744	1	0	0
cg01491932	-0.1635636	-0.1010528	0.07779614	1	0	0
cg16094352	0.12314223	0.00867575	-0.0648569	1	0	0
cg19530281	0.16691034	0.03278231	-0.0903823	1	0	0
cg01671341	0.19161799	-0.0136301	-0.1127422	0	0	1
cg05398335	-0.1774091	-0.0669253	0.09465701	1	0	0
cg06117341	0.18743936	0.08278536	-0.0801697	1	0	0
cg09891302	-0.1381974	-0.0287616	0.09268314	1	0	0
cg19713057	-0.2526334	0.13744247	0.31823001	0	0	1
cg07123118	0.12474118	0.00172029	-0.097113	1	0	0
cg05462339	0.10082464	0.00913676	-0.049085	1	0	0
cg10585661	0.23105909	0.04501046	-0.1064023	1	0	0
cg05815196	0.12005938	0.02233498	-0.0341864	1	0	0
cg08354007	-0.1383791	-0.0091457	0.08970128	1	0	0
cg23230929	0.1010872	-0.000515	-0.0635627	0	0	1
cg20980494	-0.1250505	-0.027451	0.08190903	1	0	0
cg19046189	0.08332171	0.01268526	-0.0585525	1	0	0
cg21479898	-0.2245917	-0.0335536	0.17087163	1	0	0

cg14480531	0.18097724	0.02432559	-0.0567875	1	0	0
cg24189904	0.1737868	0.09980037	-0.068799	1	0	0
cg18577232	-0.1266324	-0.0040535	0.10946741	1	0	0
cg00274203	0.12650526	-0.0296323	-0.090556	0	0	1
cg08114120	-0.1744423	-0.0810336	0.11120518	1	0	0
cg12793044	-0.1207646	NA	0.07050402	NA	0	NA
cg25110204	-0.1206824	-0.0773881	0.06668429	1	0	0
cg11881599	0.16833092	0.00562466	-0.0891274	1	0	0
cg25021613	-0.2846557	0.10614091	0.31643908	0	0	1
cg14164099	-0.2176703	-0.1010368	0.06644358	1	0	0
cg21987970	-0.1553919	-0.0002831	0.14379007	1	0	0
cg12432168	-0.1178824	-0.0144173	0.05431066	1	0	0
cg01058983	-0.3541402	-0.1129949	0.09942632	1	0	0
cg12718502	0.09827746	-0.0050739	-0.0876465	0	0	1
cg16562724	0.1468703	0.08916168	-0.039901	1	0	0
cg13272526	-0.1130733	-0.0420237	0.03423444	1	0	0
cg08967503	-0.1760696	0.03447283	0.17753688	0	0	1
cg01172965	0.17641318	0.06455619	-0.0844966	1	0	0
cg06966092	-0.141362	-0.0552746	0.10892488	1	0	0
cg09755932	0.15648039	0.11041245	-0.0789308	1	0	0
cg12911648	-0.1187402	NA	NA	NA	NA	NA
cg14880747	-0.1629414	-0.0155488	0.10769506	1	0	0
cg27009812	0.23647443	0.15957141	-0.0690425	1	0	0
cg00728973	0.0979351	0.02265225	-0.09468	1	0	0
cg08310058	0.1173218	0.02043966	-0.0550151	1	0	0
cg23301821	-0.1186571	-0.0393974	0.04493437	1	0	0
cg09346823	-0.1353612	-0.0278629	0.08412734	1	0	0
cg17731524	-0.1548131	-0.0336973	0.09457181	1	0	0
cg23664833	-0.1535447	-0.0039432	0.13868841	1	0	0
cg22372849	0.14491491	-0.0542347	-0.1018459	0	0	1
cg24742092	-0.1015346	-0.0555785	0.04086521	1	0	0

cg15961904	-0.1852158	-0.1543632	0.01297039	1	0	0
cg07261071	0.09118558	0.04006915	-0.0318692	1	0	0
cg11881963	-0.1530761	-0.0001464	0.14448114	1	0	0
cg13048201	-0.0983767	-0.02394	0.04142561	1	0	0
cg11832000	-0.1717749	-0.0402086	0.07560693	1	0	0
cg07101594	0.13293775	0.08798023	-0.0267625	1	0	0
cg18477104	-0.1374549	-0.0511769	0.07978137	1	0	0
cg05425893	-0.1476426	0.00103981	0.10590135	0	0	1
cg03310874	-0.1173179	0.0056543	0.09854446	0	0	1
cg23518655	-0.2171956	-0.1613248	-0.0318771	1	1	1
cg06743426	0.17520679	0.10331181	-0.0316199	1	0	0
cg08514145	-0.136513	-0.0715691	0.07381493	1	0	0
cg15199016	-0.1717672	-0.0864401	0.05598136	1	0	0
cg17308852	-0.1466698	-0.0464518	0.14691479	1	0	0
cg09212407	0.08987298	-0.0187869	-0.0919302	0	0	1
cg14767069	-0.1054557	0.01652428	0.11153078	0	0	1
cg06697744	0.0909135	0.02697663	-0.0283132	1	0	0
cg13153834	0.12143635	0.02658798	-0.0461201	1	0	0
cg04492166	0.19570605	0.07929376	-0.0733807	1	0	0
cg27544384	-0.1519566	-0.1153697	0.0654102	1	0	0
cg16797474	0.11900829	0.02746266	-0.0650901	1	0	0
cg22763136	-0.1274242	-0.0731394	0.00966006	1	0	0
cg18947261	0.06782304	-0.0058638	-0.0620515	0	0	1
cg26636590	-0.1322514	-0.1033675	0.06201822	1	0	0
cg24038033	-0.1300931	-0.0788345	0.09765894	1	0	0
cg09439920	0.16125593	0.12205116	0.00116735	1	1	1
cg20969199	-0.1319472	-0.1221151	-0.0375507	1	1	1
cg02946465	-0.1399915	0.02168125	0.14133399	0	0	1
cg07236324	0.07788079	0.02645813	-0.0292133	1	0	0
cg10485816	-0.130167	0.00560022	0.13391872	0	0	1
cg06900004	0.11282923	0.01797896	-0.0359084	1	0	0

cg07879334	0.07578647	-0.0158925	-0.0699443	0	0	1
cg06002052	-0.1337004	-0.0977001	0.03249215	1	0	0
cg14202477	0.09957239	0.06748187	-0.0240516	1	0	0
cg00749382	-0.1345274	-0.0521109	0.04629009	1	0	0
cg02616710	0.16145757	0.05506963	-0.0698233	1	0	0
cg10827962	0.39284755	0.11197945	-0.1253736	1	0	0
cg08749132	-0.1157365	0.01183257	0.13068696	0	0	1
cg14649827	0.11370109	-0.0049781	-0.1178298	0	0	1
cg18284616	-0.128198	0.01089399	0.12194128	0	0	1
cg12692727	-0.1394859	-0.0725171	0.04577969	1	0	0
cg17850049	0.12772103	0.05065413	-0.0830004	1	0	0
cg04809274	0.1810422	0.03567354	-0.1452327	1	0	0
cg08668140	-0.1206551	-0.0615589	0.06289048	1	0	0
cg27348779	-0.1321423	-0.0893046	0.02713846	1	0	0
cg05584597	0.19385442	0.06329452	-0.0992778	1	0	0
cg24540678	0.17293556	0.09284134	-0.0791645	1	0	0
cg12883739	-0.1235294	-0.0226444	0.08500542	1	0	0
cg14280658	0.17702998	0.08305282	-0.0814494	1	0	0
cg25173129	0.16701941	-0.0110278	-0.0676027	0	0	1
cg12559906	0.1843878	0.09212287	-0.0237391	1	0	0
cg13995230	-0.1451532	-0.0523222	0.06901313	1	0	0
cg16832302	-0.1108183	0.00277477	0.09972168	0	0	1
cg24859236	0.21124881	-0.0300418	-0.2148161	0	0	1
cg14887344	-0.1610332	-0.055046	0.09529333	1	0	0
cg07935562	-0.1365836	-0.0248248	0.14009449	1	0	0
cg07792332	-0.135626	0.03157191	0.15104185	0	0	1
cg17928607	-0.1366075	-0.0276576	0.12281549	1	0	0
cg12080566	-0.1350415	-0.0045248	0.15228653	1	0	0
cg12261117	0.13924212	0.14162675	0.02263778	1	1	1
cg20487632	0.17626619	0.14878738	0.03471423	1	1	1
cg03081549	-0.1558922	-0.0384963	0.13214483	1	0	0

cg05180206	-0.1056599	-0.0031282	0.07358631	1	0	0
cg24974047	-0.1205642	-0.0617403	0.06329187	1	0	0
cg06973172	0.09219745	0.00287984	-0.0727877	1	0	0
cg12123924	-0.1748447	-0.0400216	0.11436025	1	0	0
cg17119394	0.10955912	0.01021438	-0.0453237	1	0	0
cg05981034	0.14818883	0.08815355	-0.002657	1	0	0
cg25977913	0.11203482	0.02692517	-0.0383369	1	0	0
cg14452528	0.17667057	0.06296109	-0.1130477	1	0	0
cg27452471	-0.1411283	0.01449498	0.14209383	0	0	1
cg13234901	0.09582428	0.00768325	-0.097638	1	0	0
cg22779542	0.22858442	0.14764705	-0.1191646	1	0	0
cg05374483	-0.1054461	-0.0509983	0.04894362	1	0	0
cg10148841	0.0781954	0.0351412	-0.0441073	1	0	0
cg11388006	-0.1054414	-0.0379534	0.03367605	1	0	0
cg06330621	0.17455762	0.20646403	0.05949408	1	1	1
cg02848389	-0.1177035	-0.0592959	0.05295168	1	0	0
cg19851005	-0.138282	-0.0093823	0.11126477	1	0	0
cg25589055	-0.1093393	-0.0883341	0.02615044	1	0	0
cg13995568	-0.1614544	-0.0062769	0.16311984	1	0	0
cg21269738	-0.1243746	-0.0460943	0.0507655	1	0	0
cg24301690	0.13014529	0.06212367	-0.0136599	1	0	0
cg03515346	-0.1292577	-0.0460962	0.08859911	1	0	0
cg09151120	-0.1321916	0.01647043	0.05124729	0	0	1
cg19365328	0.17631589	0.05328017	-0.1451292	1	0	0
cg20134151	0.23244107	0.0517476	-0.2384663	1	0	0
cg21070941	0.08303748	-0.0045713	-0.0560824	0	0	1
cg10473171	-0.1255191	-0.0213841	0.12681854	1	0	0
cg11047257	0.10540464	0.02779047	-0.0700965	1	0	0
cg06214925	0.13408342	0.07542791	-0.0708991	1	0	0
cg23951683	-0.1452374	-0.0366384	0.15665863	1	0	0
cg09696535	0.22402238	0.07538413	-0.0669532	1	0	0

cg18765270	-0.147924	-0.0378694	0.08491807	1	0	0
cg00258409	0.13586526	0.03330915	-0.0826456	1	0	0
cg16514544	-0.1644409	-0.0265508	0.13540045	1	0	0
cg18637476	0.16835674	0.04138901	-0.1235561	1	0	0
cg20852226	0.50028384	0.24240753	0.08926033	1	1	1
cg15835620	0.21357935	0.048643	-0.1382331	1	0	0
cg16021126	0.09286491	-0.0201453	-0.0682153	0	0	1
cg06299447	0.11978692	0.06286067	-0.0238711	1	0	0
cg00915918	0.13179925	0.04807513	-0.082125	1	0	0
cg07281419	-0.1191804	-0.012222	0.06718349	1	0	0
cg19836999	0.11695458	0.02128791	-0.0478147	1	0	0
cg25976755	-0.1690926	-0.0572657	0.07992201	1	0	0
cg05178561	0.19941089	0.00048441	-0.084243	1	0	0
cg26099778	-0.1131992	-0.1034307	0.02150159	1	0	0
cg22411165	-0.1376764	0.00697231	0.12119935	0	0	1
cg13053441	-0.1510599	-0.0247628	0.1311938	1	0	0
cg05772206	0.13954007	0.10143674	-0.0171457	1	0	0
cg16071691	-0.1534205	-0.0412233	0.09275621	1	0	0
cg18326616	-0.3157344	-0.255461	0.04948686	1	0	0
cg05825703	0.18449162	0.12095751	-0.0348274	1	0	0
cg27376984	0.22301638	0.09405812	-0.0397546	1	0	0
cg00405086	0.23146184	-0.0294551	-0.1526478	0	0	1
cg19038804	-0.1237779	-0.1007457	0.01073208	1	0	0
cg16214148	-0.2547947	0.08524968	0.32732079	0	0	1
cg14500637	-0.1487639	-0.0531916	0.13432806	1	0	0
cg16336556	-0.1553069	-0.1051046	0.08777835	1	0	0
cg05381578	0.14335762	0.06487814	-0.0406262	1	0	0
cg18635631	-0.1342689	0.00860408	0.08457941	0	0	1
cg25661986	-0.1882317	-0.1264007	0.0279598	1	0	0
cg25427880	0.13618193	-0.0304407	-0.1729925	0	0	1
cg20521672	-0.1250855	-0.0062867	0.18425365	1	0	0

cg00026554	0.11828107	-0.0341031	-0.0888167	0	0	1
cg06809604	0.17385225	0.07623755	-0.0957478	1	0	0
cg15515311	-0.1596122	-0.0671896	0.11828907	1	0	0
cg24826151	-0.13572	-0.0986721	0.09035157	1	0	0
cg16160712	-0.1845352	-0.0367095	0.17383041	1	0	0
cg18416734	-0.2569556	-0.0013643	0.15261502	1	0	0
cg08316097	0.13286069	0.00908839	-0.0750864	1	0	0
cg06695534	-0.1385764	-0.0541052	0.13115086	1	0	0
cg21385983	-0.1615549	-0.110102	0.03892767	1	0	0
cg04196458	-0.1306865	-0.00908	0.13116782	1	0	0
cg07260003	0.0979243	0.03859163	0.00718489	1	1	1
cg00407231	0.12955488	0.1104704	0.01734047	1	1	1
cg02729593	-0.1353312	-0.1153081	0.04130498	1	0	0
cg11949606	0.15740713	0.07127877	-0.0438769	1	0	0
cg07891507	0.18068996	0.04166139	-0.1148921	1	0	0
cg08451988	-0.1403602	0.01259002	0.10286896	0	0	1
cg16114629	-0.1168346	-0.0752483	0.0061223	1	0	0
cg13051245	-0.2234082	0.03544986	0.08777409	0	0	1
cg24399872	-0.1204822	-0.0556125	0.06888618	1	0	0
cg01360258	-0.1319476	-0.012107	0.07966331	1	0	0
cg18077061	-0.1212978	-0.0459195	0.03205033	1	0	0
cg12829286	0.13930629	-0.019106	-0.1090954	0	0	1
cg09786634	0.41806959	0.24752342	-0.0938872	1	0	0
cg16034742	-0.1152492	-0.0144671	0.06380155	1	0	0
cg09915950	-0.1357572	-0.0589182	0.04325129	1	0	0
cg17414215	0.13386212	-0.0250391	-0.0990088	0	0	1
cg05098880	-0.1279384	-0.0758007	0.03098462	1	0	0
cg06275417	-0.1307983	-0.0406292	0.08500757	1	0	0
cg25492319	-0.1429372	-0.0442591	0.09743156	1	0	0
cg27176043	-0.1237595	-0.0743669	0.04136531	1	0	0
cg23585420	0.14362765	0.04029875	-0.0552684	1	0	0

cg23136457	0.09390467	0.08108027	0.02097869	1	1	1
cg10701636	-0.2725831	0.05710961	0.34806195	0	0	1
cg06669700	0.11923607	0.15032149	0.02052243	1	1	1
cg10058913	-0.2018854	-0.065937	0.13057282	1	0	0
cg01166384	0.11525201	0.03153487	-0.0464518	1	0	0
cg18893685	-0.2009426	-0.0635049	0.06335234	1	0	0
cg10424417	0.19139377	0.14353539	-0.0157548	1	0	0
cg20825664	-0.1332024	-0.0445172	0.04848111	1	0	0
cg26848157	-0.1369028	-0.0347581	0.08663736	1	0	0
cg24168413	-0.0680131	-0.006342	0.0501574	1	0	0
cg21468274	0.26557796	0.201042	0.03245999	1	1	1
cg00852924	-0.0897277	0.01230963	0.06186523	0	0	1
cg23864881	0.11634136	0.01062595	-0.0687182	1	0	0
cg26768406	0.1197605	0.04072409	-0.0525953	1	0	0
cg00418695	-0.1469106	-0.0729439	0.02010683	1	0	0
cg05249343	-0.1177465	-0.0602	0.05350087	1	0	0
cg24530225	-0.1445001	-0.0510936	0.11652893	1	0	0
cg01420388	0.10214852	-0.0415913	-0.1079508	0	0	1
cg04526584	0.24864787	0.06085826	-0.2193197	1	0	0
cg13476950	0.2012535	0.0934892	-0.1006983	1	0	0
cg14099479	-0.1283102	-0.0840255	0.06422564	1	0	0
cg02023469	0.12790659	0.09501611	-0.0553619	1	0	0
cg03632717	0.11456106	0.01863961	-0.0451274	1	0	0
cg01571916	-0.1334256	-0.0564234	0.09308228	1	0	0
cg25028289	0.19077732	0.13734636	0.01250218	1	1	1
cg10690887	0.20472657	0.03581522	-0.1061221	1	0	0
cg13171192	-0.1267801	-0.040628	0.07642122	1	0	0
cg21344159	-0.1468306	-0.0510273	0.14197645	1	0	0
cg00610991	0.26332591	0.20444851	0.0144042	1	1	1
cg11551940	-0.1447335	-0.0555292	0.04908769	1	0	0
cg00660817	0.10079378	0.09376288	0.01275619	1	1	1

cg19781462	0.38803631	0.29211097	-0.2247964	1	0	0
cg17365440	0.17152437	-0.010324	-0.1085861	0	0	1
cg11641916	-0.1407388	-0.0269217	0.05763748	1	0	0
cg16404106	0.19258631	0.02782896	-0.200176	1	0	0
cg12113819	0.09316596	0.00012759	-0.0717033	1	0	0
cg14898111	-0.149529	0.00499531	0.07723321	0	0	1
cg03871753	-0.1357057	-0.0834074	0.04774165	1	0	0
cg16055406	0.08970246	0.01411567	-0.0796981	1	0	0
cg02906470	-0.2028503	-0.1022931	0.11141115	1	0	0
cg16678001	0.12854482	0.10299212	0.01853567	1	1	1
cg07252482	-0.1561415	-0.126645	0.04064073	1	0	0
cg05264101	0.0771499	-0.0047357	-0.0694741	0	0	1
cg15765497	-0.1089103	-0.0190849	0.05393463	1	0	0
cg24602369	-0.1077228	-0.0329457	0.08249479	1	0	0
cg10843046	-0.1551673	-0.1294316	0.00796492	1	0	0
cg07422110	-0.1722907	0.0193607	0.23673849	0	0	1
cg12669065	0.11014523	0.0330377	-0.0651064	1	0	0
cg19477689	-0.1493165	-0.0991923	0.08210974	1	0	0
cg26890354	0.17565292	0.07326787	-0.1150016	1	0	0
cg19693110	0.08579747	0.01114187	-0.0538216	1	0	0
cg19186966	-0.1627667	0.05047885	0.19670468	0	0	1
cg00632863	-0.2028707	-0.1069057	0.11320354	1	0	0
cg14011077	0.2867252	0.07103723	NA	1	NA	NA
cg02833471	-0.1322242	-0.0614299	0.04134381	1	0	0
cg13502414	0.18096312	0.03419007	-0.1514534	1	0	0
cg09521093	-0.203761	-0.0362722	0.15713179	1	0	0
cg25667337	0.09823191	-0.0019955	-0.0538702	0	0	1
cg25225310	0.10106849	0.02297403	-0.0524442	1	0	0
cg14017489	-0.1247556	-0.0941635	0.021217	1	0	0
cg11251215	0.1146028	0.03723344	-0.0900402	1	0	0
cg16509736	-0.1273506	-0.0546062	0.03603419	1	0	0

cg17527673	0.26210827	0.10427987	-0.1234399	1	0	0
cg01017212	-0.1393776	-0.0545535	0.06112776	1	0	0
cg14762528	-0.1613237	-0.0119295	0.14981125	1	0	0
cg24529722	0.10124702	0.00385752	-0.056866	1	0	0
cg26117521	0.13447442	-0.000926	-0.0782813	0	0	1
cg13987674	0.15629181	0.10315261	-0.0234359	1	0	0
cg04928338	-0.1474158	-0.0239511	0.12066968	1	0	0
cg27452217	0.16247491	0.03866793	-0.058435	1	0	0
cg01911585	0.12646415	0.03849616	-0.0463847	1	0	0
cg12216419	-0.1637662	-0.0455377	0.1298667	1	0	0
cg26446602	-0.1458226	-0.0266524	0.13823858	1	0	0
cg03656583	0.10651901	0.00955843	-0.0493672	1	0	0
cg13552468	-0.1405008	0.02463137	0.19891751	0	0	1
cg06017028	-0.3979314	-0.124575	0.28123252	1	0	0
cg04518155	0.19561393	0.06085902	-0.0600404	1	0	0
cg05726450	0.10638103	0.01422639	-0.0286771	1	0	0
cg03826976	0.1791866	0.07411467	-0.1208416	1	0	0
cg08860346	-0.1657233	0.05794239	0.16759069	0	0	1
cg09102573	-0.1050755	-0.0979606	0.02102919	1	0	0
cg14373277	-0.1317577	-0.0732541	0.08101719	1	0	0
cg14106638	-0.1273592	-0.0123608	0.06807424	1	0	0
cg26781482	0.10932023	0.04818348	-0.0091348	1	0	0
cg15805330	-0.1333999	-0.0833409	0.05024618	1	0	0
cg17220734	-0.123863	-0.0128065	0.09613951	1	0	0
cg05083423	-0.1054886	-0.0639807	0.00711717	1	0	0
cg05620451	-0.1273805	-0.0706839	0.11306556	1	0	0
cg22287738	0.09385769	0.0412934	-0.0161706	1	0	0
cg13740078	-0.1177752	0.00850134	0.10661485	0	0	1
cg18553232	0.1233308	0.034874	-0.0669582	1	0	0
cg15645685	0.2208873	0.03943405	-0.0704112	1	0	0
cg26200980	-0.1811631	-0.1199207	0.00171087	1	0	0

cg06981391	0.3374343	0.09145911	-0.1392459	1	0	0
cg01942646	0.22302389	-0.0146268	-0.1165587	0	0	1
cg01084270	-0.1314276	-0.0536096	0.03636057	1	0	0
cg20959189	-0.1232017	-0.0341255	0.11953046	1	0	0
cg18836146	0.10508307	0.0757275	0.00684249	1	1	1
cg26759568	-0.1169094	-0.0267901	0.07465071	1	0	0
cg18747276	-0.1248234	-0.0587033	0.03942884	1	0	0
cg16248584	-0.1636358	-0.0366933	0.14428847	1	0	0
cg11401101	-0.1188761	-0.0255004	0.10659867	1	0	0
cg07199534	0.19934473	-0.0118103	-0.0989258	0	0	1
cg04188430	-0.1376342	-0.0940773	0.08216964	1	0	0
cg09449447	0.27941652	0.20067905	0.06443839	1	1	1
cg07349477	-0.2230777	-0.0382287	0.08155322	1	0	0
cg06299718	-0.1220527	0.00633243	0.12125365	0	0	1
cg00095772	-0.1202929	0.00970812	0.11231223	0	0	1
cg26744460	-0.2203925	-0.1248192	0.01902275	1	0	0
cg11970682	0.15203355	0.0456154	-0.0546353	1	0	0
cg24424833	-0.1751607	-0.0990148	0.04579658	1	0	0
cg03416592	-0.1365967	-0.0052049	0.11862225	1	0	0
cg26015743	0.13521418	-0.0409933	-0.1593209	0	0	1
cg03211711	-0.1410409	-0.0059038	0.06578287	1	0	0
cg12800962	0.1556924	0.04178634	-0.0652958	1	0	0
cg11544434	-0.1833049	-0.0734984	0.04909354	1	0	0
cg27306101	-0.1418003	-0.0473178	0.12648738	1	0	0
cg24808271	0.09171135	-0.00727	-0.0661255	0	0	1
cg13802686	-0.1245253	-0.0252852	0.08029692	1	0	0
cg20039814	0.28179362	0.16940592	-0.0213159	1	0	0
cg20788584	0.13383475	0.09706392	0.0389158	1	1	1
cg03462634	-0.1364185	-0.0486621	0.08091777	1	0	0
cg13905238	0.24203796	0.1409714	0.02103766	1	1	1
cg24011830	-0.147979	-0.0408524	0.12407783	1	0	0

cg19898108	0.13892981	0.15487623	0.00359306	1	1	1
cg05371135	0.1005904	0.02812915	-0.0424912	1	0	0
cg23354714	-0.1158185	-0.0180299	0.10252396	1	0	0
cg03554134	-0.118155	-0.0082789	0.06863125	1	0	0
cg00105570	0.07885399	0.02986082	-0.0045303	1	0	0
cg09653626	0.25429394	-0.0168457	-0.1213556	0	0	1
cg05694908	0.09809347	0.02866995	-0.0418739	1	0	0
cg08970112	-0.1715218	-0.074135	0.05233894	1	0	0
cg08215366	-0.2058204	-0.0217868	0.15585549	1	0	0
cg15497006	0.22866285	0.10956635	0.0005042	1	1	1
cg21439214	-0.1333361	-0.098103	0.02004024	1	0	0
cg16685388	0.31413238	-0.0228655	-0.4042011	0	0	1
cg09012269	0.16846336	0.08210553	-0.0333141	1	0	0
cg03839155	-0.1449122	-0.0825111	0.01786039	1	0	0
cg20779473	-0.1271434	-0.0785898	0.07816638	1	0	0
cg26764980	0.21423359	0.12644384	0.00515607	1	1	1
cg02562540	-0.175937	-0.0259496	0.13395181	1	0	0
cg03688771	0.1225409	0.0108532	-0.0811733	1	0	0
cg09352738	-0.1619526	-0.0610967	0.10967384	1	0	0
cg02622288	0.11942917	0.06403419	-0.0030638	1	0	0
cg03221702	0.09669129	0.02703736	-0.0401588	1	0	0
cg12792931	0.1124916	0.10748584	0.04557775	1	1	1
cg01822965	-0.1074699	-0.0371089	0.07245932	1	0	0
cg11122136	-0.102389	-0.0643707	0.02591224	1	0	0
cg16576811	0.10322028	0.03076666	-0.0116494	1	0	0
cg25360898	-0.1118915	-0.0703035	0.04435118	1	0	0
cg09035930	-0.8241988	-0.4806439	0.3340943	1	0	0
cg12220237	-0.1849187	-0.0923223	0.07147393	1	0	0
cg06004033	0.18932561	0.0984966	-0.0259185	1	0	0
cg02725313	0.12216162	0.07242858	-0.0502564	1	0	0
cg08859379	-0.2147191	-0.0749456	0.1076082	1	0	0

cg09908050	0.20382862	0.03738754	-0.0438062	1	0	0
cg11842502	-0.1764619	-0.0168444	0.11137064	1	0	0
cg12016107	0.28433913	-0.0492754	-0.2666142	0	0	1
cg22609474	-0.1629276	-0.0653124	0.14723615	1	0	0
cg18654697	-0.0947064	-0.0775562	-0.0169744	1	1	1
cg14825462	0.11016374	0.00972655	-0.0955726	1	0	0
cg04875128	0.55055599	0.39483068	-0.1570101	1	0	0
cg19498960	0.12633815	0.01658115	-0.0872612	1	0	0
cg17372806	0.15113937	-0.0117524	-0.0839145	0	0	1
cg04653025	-0.1297287	-0.0870138	0.02317134	1	0	0
cg25985734	0.12890049	0.06937622	-0.0457125	1	0	0
cg11980897	0.10933836	0.06644877	-0.0149998	1	0	0
cg04580170	-0.1111293	-0.0679373	0.05874888	1	0	0
cg25206709	-0.1208569	-0.0282131	0.06909959	1	0	0
cg11928963	-0.1269649	-0.0635773	0.05270833	1	0	0
cg19819280	-0.1393581	-0.0379911	0.11057812	1	0	0
cg04107129	-0.1540829	0.01365462	0.15494338	0	0	1
cg07337434	-0.1384239	-0.0012857	0.11677243	1	0	0
cg19676181	0.22737613	0.17276213	-0.041671	1	0	0
cg04317477	-0.1281492	-0.0283147	0.10731479	1	0	0
cg12767180	-0.15447	0.03338266	0.22372879	0	0	1
cg21430218	-0.1169061	-0.0504465	0.06134414	1	0	0
cg11841529	0.2671989	0.03985797	-0.1993646	1	0	0
cg04544898	-0.1252911	0.00281819	0.11114744	0	0	1
cg01178601	-0.1121925	-0.0767802	0.00682777	1	0	0
cg18618355	0.23182729	-0.0559878	-0.2606354	0	0	1
cg19139330	-0.1272728	-0.0932304	0.04370814	1	0	0
cg05897316	0.07125938	0.00028821	-0.0397602	1	0	0
cg22281866	0.16868649	0.03749055	-0.111458	1	0	0
cg00272709	0.15353877	0.0439811	-0.0761742	1	0	0
cg13256716	-0.1979974	-0.0266509	0.09677942	1	0	0

cg09895527	0.31634024	0.11148427	-0.1116254	1	0	0
cg05215884	-0.1421443	-0.0711819	0.12121675	1	0	0
cg26924247	-0.1671935	-0.0159046	0.1504392	1	0	0
cg03508170	-0.1179867	-0.0702843	0.03313894	1	0	0
cg04123409	0.20276926	0.06289663	-0.152667	1	0	0
cg15517335	0.08876764	-0.0124761	-0.0651708	0	0	1
cg22338369	-0.3391938	0.0234804	0.24305947	0	0	1
cg21640732	-0.113556	-0.0698357	0.07691342	1	0	0
cg08774410	0.17804778	0.10618812	-0.0347253	1	0	0
cg05409540	0.3143065	0.13011262	-0.0993439	1	0	0
cg06484622	0.18662093	0.0151837	-0.131596	1	0	0
cg15168825	-0.1020252	-0.0758358	0.02402561	1	0	0
cg02150060	-0.144835	-0.0196153	0.090412	1	0	0
cg05408680	-0.1368443	-0.0808521	0.0732885	1	0	0
cg02932098	0.12543291	0.09527032	-0.0201854	1	0	0
cg20562630	-0.1298456	-0.1192155	-0.0149402	1	1	1
cg00845279	-0.2140671	-0.0104889	0.21661139	1	0	0
cg16120196	-0.1191067	-0.1241086	0.04218504	1	0	0
cg09601692	-0.1550343	0.0168693	0.17321089	0	0	1
cg03172058	0.16092107	0.04910291	-0.0434651	1	0	0
cg18301717	-0.1085871	-0.0234155	0.11636304	1	0	0
cg00851361	0.11002558	0.06600913	-0.0177648	1	0	0
cg08955035	0.1739533	0.09564205	-0.0657414	1	0	0
cg01999641	0.10826446	0.00853019	-0.0631868	1	0	0
cg16090476	-0.1305039	-0.0028314	0.13427148	1	0	0
cg21199629	-0.2030029	-0.0775965	0.03884679	1	0	0
cg03406313	-0.1463618	-0.0489986	0.09781625	1	0	0
cg01454215	0.1497278	0.07156391	-0.0535779	1	0	0
cg16821558	-0.1746975	-0.0554788	0.04728524	1	0	0
cg11715789	0.16905209	-0.0267623	-0.2047534	0	0	1
cg20984904	-0.1322894	-0.1304039	0.03760697	1	0	0

cg16992787	0.09051392	-0.0430023	-0.1241662	0	0	1
cg07207813	0.13659391	-0.0351648	-0.1584587	0	0	1
cg05123019	-0.1431715	0.00103364	0.12844229	0	0	1
cg14086828	0.13433244	0.02659017	-0.0759745	1	0	0
cg22974428	-0.1420431	-0.0337117	0.17069101	1	0	0
cg04616680	-0.171	0.0398259	0.1729948	0	0	1
cg00841845	0.24376144	0.10991877	-0.1144434	1	0	0
cg05813955	-0.1159866	-0.0742897	0.03511812	1	0	0
cg15012214	-0.124612	0.00881479	0.12438022	0	0	1
cg08875089	-0.1774077	-0.0644033	0.13284192	1	0	0
cg16842449	0.11440592	0.04219399	-0.0639001	1	0	0
cg13356734	0.09062206	-0.0163003	-0.0846063	0	0	1
cg18389639	0.12346155	0.0947289	0.00900647	1	1	1
cg26774971	0.19721915	0.01104381	NA	1	NA	NA
cg12778716	0.32179677	0.15132012	0.04419538	1	1	1
cg22154992	-0.1188313	-0.002397	0.11951388	1	0	0
cg05415146	0.15078667	-0.02915	-0.1492688	0	0	1
cg03540731	0.17904452	0.04220562	-0.0764187	1	0	0
cg18462898	0.17018885	-0.1767375	-0.2830233	0	0	1
cg25768108	-0.1198866	-0.0251408	0.10156816	1	0	0
cg18062490	0.14071443	-0.0093265	-0.082173	0	0	1
cg05213189	-0.1727494	-0.1263803	0.03348265	1	0	0
cg26667500	0.08001388	0.00838962	-0.0696234	1	0	0
cg24575128	0.10720062	0.095326	-0.0238699	1	0	0
cg26371327	0.25823173	0.18698234	-0.0318746	1	0	0
cg08993331	0.33175133	0.26221789	-0.0158798	1	0	0
cg08259755	-0.1289372	-0.0175332	0.0965924	1	0	0
cg21304569	0.11745776	0.04830896	-0.0467862	1	0	0
cg07578986	-0.1191153	-0.0308669	0.06052567	1	0	0
cg18016128	0.09590063	-0.0079098	-0.0798635	0	0	1
cg09940349	0.13380123	-0.0050538	-0.1095281	0	0	1

cg04010123	0.17808125	0.11083663	-0.0229418	1	0	0
cg10433059	-0.1375195	0.0206441	0.182874	0	0	1
cg14878151	-0.1288698	-0.0226263	0.05324573	1	0	0
cg23938419	0.18953341	0.08317237	-0.0987082	1	0	0
cg12903948	-0.2088808	-0.0249099	0.1234215	1	0	0
cg03506768	-0.1394272	0.04801735	0.17861914	0	0	1
cg03617420	0.09183942	0.04722912	-0.0541225	1	0	0
cg12010413	-0.1549162	-0.0445818	0.07820598	1	0	0
cg19511929	-0.2056993	-0.0732536	0.09933242	1	0	0
cg26508841	0.10264066	0.03193233	-0.0423777	1	0	0
cg02483931	0.12282756	0.02872243	-0.0645469	1	0	0
cg04880487	-0.132063	-0.0770043	0.05201761	1	0	0
cg03755115	0.12537453	0.01140132	-0.0280373	1	0	0
cg05405150	-0.1211671	-0.0487098	0.05696053	1	0	0
cg14082441	-0.1313118	-0.0448775	0.10611075	1	0	0
cg25667676	-0.1419615	-0.0097196	0.10403265	1	0	0
cg01987034	-0.1223542	-0.0675257	0.05791328	1	0	0
cg01267415	-0.1310908	-0.0426429	0.04896996	1	0	0
cg19811609	-0.1404959	-0.0504761	0.08593088	1	0	0
cg07172178	-0.1402405	-0.0404494	0.08246396	1	0	0
cg14434111	-0.1380996	-0.0710599	0.09252459	1	0	0
cg04954726	0.29655474	0.12394282	-0.0993126	1	0	0
cg20345275	0.13828461	-0.0444252	-0.1117498	0	0	1
cg07969622	0.11175108	0.05972522	-0.0248509	1	0	0
cg11508935	0.11798802	0.06292638	-0.0119784	1	0	0
cg14615449	-0.15433	-0.0494829	0.1442919	1	0	0
cg25653759	-0.1288095	-0.0369619	0.07588903	1	0	0
cg01541867	0.10076966	0.11988275	-0.0006436	1	0	0
cg15359788	-0.1285328	0.00224097	0.07989953	0	0	1
cg01051024	-0.1162951	-0.0754579	0.07050746	1	0	0
cg13357998	-0.1412897	-0.1142426	0.02156182	1	0	0

cg21695476	-0.1488362	-0.0506912	0.10345607	1	0	0
cg08634012	0.10451091	0.02549922	-0.0470718	1	0	0
cg18468235	-0.1458129	-0.0164297	0.09893799	1	0	0
cg01140719	0.07504248	0.0196469	-0.0380503	1	0	0
cg14975122	0.19349977	0.02726284	-0.0728603	1	0	0
cg16072296	-0.1290166	-0.0068188	0.10236846	1	0	0
cg04482760	-0.1177459	-0.0714386	0.03194424	1	0	0
cg06127160	0.26515842	0.05251921	-0.0896561	1	0	0
cg17098441	-0.1125776	-0.0719974	0.01186969	1	0	0
cg09090006	0.11001761	-0.0206505	-0.0985184	0	0	1
cg01616313	0.09417544	-0.0143439	-0.0716574	0	0	1
cg06759780	0.11345663	0.07892701	-0.0566748	1	0	0
cg07066019	0.09281247	-0.0035108	-0.067994	0	0	1
cg11399136	0.115232	-0.0015466	-0.0551452	0	0	1
cg05867210	0.14511617	0.07838994	-0.0651482	1	0	0
cg11487037	0.22095753	0.25199918	0.05284121	1	1	1
cg08787837	0.33450506	0.13751046	-0.0410031	1	0	0
cg05014732	-0.2046139	-0.0587485	0.1063862	1	0	0
cg02492280	0.07820039	0.01499397	-0.0526629	1	0	0
cg00592995	0.10725249	0.06077308	-0.0365672	1	0	0
cg00974681	-0.1114583	-0.0331493	0.09760766	1	0	0
cg26532208	-0.1364679	-0.0432461	0.08951342	1	0	0
cg07738234	0.25068142	0.0005457	-0.2147047	1	0	0
cg26478216	0.09203667	-0.0097768	-0.0568585	0	0	1
cg02805410	-0.1408351	-0.075588	0.00439357	1	0	0
cg20538707	0.08860057	-0.0352334	-0.0805777	0	0	1
cg11316100	0.11617147	0.03193001	-0.0358426	1	0	0
cg21136371	0.13126385	0.00489356	-0.034577	1	0	0
cg01226543	-0.1301468	0.01929291	0.16927164	0	0	1
cg06161010	0.4014472	0.06659411	-0.3143792	1	0	0
cg01876832	-0.1382332	0.00086925	0.17698829	0	0	1

cg07301795	0.13819506	0.02037882	-0.0849367	1	0	0
cg15600598	-0.1337131	0.03037206	0.14333627	0	0	1
cg22490405	-0.2319894	-0.01026	0.16284532	1	0	0
cg02186450	-0.1761467	-0.0794758	0.12938704	1	0	0
cg27093835	0.09271746	0.00953331	-0.0600077	1	0	0
cg02603284	-0.151988	-0.1252384	0.09057473	1	0	0
cg19649900	-0.0805837	0.01687679	0.05219391	0	0	1
cg02107801	0.14652463	0.02738491	-0.0630952	1	0	0
cg20816857	-0.1495099	0.02977259	0.18209281	0	0	1
cg08926287	0.08905882	0.02894255	-0.048392	1	0	0
cg08079888	-0.1294064	-0.0290891	0.10087995	1	0	0
cg19362478	0.10316375	0.04205591	-0.040377	1	0	0
cg15613153	-0.1646518	0.0578698	0.20234676	0	0	1
cg07209338	0.13834741	0.00088143	-0.0767334	1	0	0
cg15501329	-0.1506179	-0.0067858	0.15366649	1	0	0
cg13687924	0.18605304	0.09741709	-0.0410778	1	0	0
cg13969089	-0.1238124	-0.0886848	0.08523093	1	0	0
cg13569053	0.10014309	0.02392877	-0.0345231	1	0	0
cg07343039	-0.1518709	-0.0482735	0.11516062	1	0	0
cg00674569	-0.144931	0.03502934	0.16560428	0	0	1
cg23083936	0.08145102	-0.0098197	-0.0671217	0	0	1
cg17638070	0.1046029	0.01323664	-0.0806687	1	0	0
cg11469627	-0.1306016	-0.0220869	0.0583932	1	0	0
cg14426030	-0.1303345	-0.0479093	0.05065383	1	0	0
cg06478649	0.17712294	0.01631996	-0.1417458	1	0	0
cg01701207	-0.2136179	-0.0433159	0.1319231	1	0	0
cg19262963	0.12528802	0.08203155	0.03259363	1	1	1
cg21605772	-0.2066866	-0.042644	0.13836803	1	0	0
cg22039638	-0.2520646	-0.1200241	0.05649444	1	0	0
cg10247328	0.13907425	0.01783487	-0.0484217	1	0	0
cg16699632	-0.1959984	0.03415548	0.21265786	0	0	1

cg03278639	0.13830072	0.03456925	-0.0240554	1	0	0
cg02737861	-0.1298035	-0.0537794	0.08052599	1	0	0
cg05763294	-0.1242969	-0.0866124	0.0008274	1	0	0
cg10026995	0.107732	-0.0247083	-0.1028665	0	0	1
cg27427318	0.15834483	-0.0299894	-0.1039687	0	0	1
cg07120993	0.60092509	0.19460231	-0.150112	1	0	0
cg08461765	-0.2091189	NA	0.12791555	NA	0	NA
cg10132858	-0.1139146	-0.0913607	0.0515643	1	0	0
cg27531590	-0.1263336	-0.0294612	0.07986597	1	0	0
cg10718608	0.13270394	0.11353328	-0.021206	1	0	0
cg27134495	-0.1422643	0.05809665	0.18259004	0	0	1
cg11046544	0.08442095	0.00221637	-0.0577393	1	0	0
cg16111073	-0.1864075	-0.1483453	0.04391769	1	0	0
cg00180787	-0.1182439	-0.0762928	0.04970973	1	0	0
cg26062439	-0.1104816	-0.0499958	0.03822049	1	0	0
cg23069718	0.12180777	0.01248764	-0.0623268	1	0	0
cg14620221	0.08983254	0.06619067	0.01042232	1	1	1
cg00851518	0.25427399	0.21114333	-0.0177441	1	0	0
cg04923758	0.10148298	0.04924695	-0.0264814	1	0	0
cg01650399	0.0774701	0.00568784	-0.0444802	1	0	0
cg11755913	-0.111341	-0.0416801	0.06853526	1	0	0
cg02952849	-0.145813	-0.1160958	0.01676719	1	0	0
cg00159951	-0.1275906	-0.0857832	0.0442531	1	0	0
cg03665255	-0.1391091	-0.036069	0.06435923	1	0	0
cg26708596	0.19538946	0.18401184	0.03537553	1	1	1
cg01058360	-0.1616685	0.07735206	0.18951187	0	0	1
cg26137432	-0.1156356	-0.0741007	0.04900601	1	0	0
cg15189415	-0.1969043	-0.1396876	0.04990854	1	0	0
cg06540283	-0.1264483	-0.0106763	0.07596986	1	0	0
cg13761842	-0.127272	-0.0291965	0.08183816	1	0	0
cg07391141	0.08943529	0.05116767	-0.010987	1	0	0

cg17850597	-0.1016919	-0.0263316	0.04269448	1	0	0
cg17768039	-0.125574	-0.0127995	0.0926209	1	0	0
cg04650889	0.14402604	0.12539153	0.01027796	1	1	1
cg06548369	-0.1277445	-0.0620828	0.04923883	1	0	0
cg08035195	-0.1402545	-0.053458	0.09911649	1	0	0
cg15523907	-0.1628753	-0.0531319	0.13831642	1	0	0
cg11121623	-0.2597359	-0.0806594	0.09163701	1	0	0
cg22035625	-0.1134188	-0.0666314	0.02915608	1	0	0
cg04767060	0.1768535	0.13831573	0.0226541	1	1	1
cg04701420	0.25357755	0.21144367	0.07943251	1	1	1
cg00239420	-0.1160046	-0.0709566	0.0172657	1	0	0
cg22982930	0.10349866	0.04192843	-0.0249791	1	0	0
cg19525758	0.0815285	0.0147918	-0.035898	1	0	0
cg17762936	0.08264485	0.01467273	-0.0298774	1	0	0
cg06254804	-0.1060714	-0.0736565	0.02383492	1	0	0
cg21780171	0.22637836	0.0763245	-0.0904321	1	0	0
cg09896909	0.14283645	0.05550322	-0.0516502	1	0	0
cg22975913	0.16964205	0.11775512	0.03696758	1	1	1
cg15838394	0.07928136	-0.0158206	-0.0616812	0	0	1
cg24642169	0.11217873	0.01563495	-0.0960131	1	0	0
cg17337307	-0.114194	0.00115447	0.08737356	0	0	1
cg21226227	-0.1382497	-0.0385617	0.07349922	1	0	0
cg01832935	-0.1478926	-0.1015524	-0.0166471	1	1	1
cg07004313	-0.1309578	-0.065024	0.07328512	1	0	0
cg06979587	-0.1288102	-0.1331557	-0.0070355	1	1	1
cg13721397	0.1201727	NA	-0.097416	NA	0	NA
cg02495604	0.11203179	0.06315931	-0.036345	1	0	0
cg24568187	-0.1458703	-0.1000154	0.07043157	1	0	0
cg12902625	0.07326036	0.0109866	-0.0508116	1	0	0
cg23430652	0.19475723	0.11072776	-0.0682813	1	0	0
cg10516317	-0.1250411	-0.1121206	0.00055551	1	0	0

cg08990402	-0.1479758	-0.0578863	0.0426522	1	0	0
cg20986135	0.19235036	0.08546199	-0.0520571	1	0	0
cg08787988	0.24180014	-0.0032271	-0.2086647	0	0	1
cg15562189	0.1201389	0.09814295	0.04713112	1	1	1
cg24344841	0.07512547	0.01977794	-0.027218	1	0	0
cg18060685	0.19329342	0.09911473	-0.0146924	1	0	0
cg03422350	-0.1190683	-0.0280829	0.10664741	1	0	0
cg07018107	0.21429588	0.17586872	-0.0015439	1	0	0
cg16597425	-0.13076	0.0052185	0.15265184	0	0	1
cg25633717	0.06883052	-0.0117799	-0.0516682	0	0	1
cg16947270	-0.1363196	-0.0880414	0.04210476	1	0	0
cg07848657	-0.1383494	-0.0066342	0.16550474	1	0	0
cg22332666	-0.1275824	-0.0119135	0.11732164	1	0	0
cg09409956	-0.1180544	-0.0361047	0.1112939	1	0	0
cg22320795	0.14589974	0.09781103	-0.0427342	1	0	0
cg24189734	-0.1537543	-0.0784722	0.04145966	1	0	0
cg01469823	0.14651706	0.10695978	-0.0135328	1	0	0
cg02840199	0.13551687	0.05008246	-0.0810926	1	0	0
cg10801473	-0.1417339	0.05260998	0.18618065	0	0	1
cg25516542	-0.2034208	0.00408581	0.13968868	0	0	1
cg04351156	0.08591257	0.02049129	-0.0373006	1	0	0
cg09342997	-0.2016781	-0.0800147	-0.0273409	1	1	1
cg02049700	-0.1184316	-0.0349041	0.06439329	1	0	0
cg13207340	0.09879594	-0.0037754	-0.0316667	0	0	1
cg00493276	-0.1307495	-0.0012671	0.13055708	1	0	0
cg23554286	-0.1288484	-0.0419666	0.08211902	1	0	0
cg01299932	0.12039366	0.04129385	-0.0696224	1	0	0
cg24777572	-0.1229959	-0.009389	0.11709131	1	0	0
cg13427179	-0.1317345	-0.0671249	0.02265409	1	0	0
cg27560494	-0.1347331	NA	0.04078139	NA	0	NA
cg21614231	-0.1019179	-0.0662899	0.02651514	1	0	0

cg19088924	-0.1296896	-0.1119208	0.02030717	1	0	0
cg27021512	0.15896788	-0.0023326	-0.1046573	0	0	1
cg21814550	-0.1684683	0.04972986	0.12655015	0	0	1
cg03422907	-0.1035302	-0.0952177	-0.0360617	1	1	1
cg16943816	-0.1728047	-0.0191077	0.13538708	1	0	0
cg04415699	-0.1364811	-0.0966384	0.0226335	1	0	0
cg04334121	-0.1275417	-0.0553814	0.03826721	1	0	0
cg07486383	0.18662065	0.10178612	-0.0177527	1	0	0
cg13383842	0.09582731	-0.0371898	-0.0738572	0	0	1
cg00991578	-0.2063534	-0.0154116	0.09637111	1	0	0
cg15195412	0.0965015	0.00338996	-0.0453315	1	0	0
cg09935271	0.15904628	0.08473913	0.05011882	1	1	1
cg15031550	-0.1695564	0.0091826	0.19473035	0	0	1
cg05095832	-0.1427934	-0.0258108	0.12901085	1	0	0
cg03306780	-0.1229467	-0.0445091	0.02338223	1	0	0
cg14694639	0.09350374	0.02792373	-0.0385812	1	0	0
cg15948836	0.19289251	0.10409404	-0.038643	1	0	0
cg00454592	-0.2068078	-0.0774554	0.08358555	1	0	0
cg22845525	0.12952546	0.05988641	-0.0653022	1	0	0
cg05198507	-0.1376993	-0.0737219	0.07807474	1	0	0
cg09782493	-0.1194929	-0.0562185	0.06424547	1	0	0
cg12206157	0.09121281	0.00283408	-0.0157131	1	0	0
cg17745872	-0.1363185	-0.0589678	0.07501216	1	0	0
cg06589421	-0.1100766	-0.0553471	0.03352357	1	0	0
cg02996903	0.11406736	0.05219121	-0.0625113	1	0	0
cg00706275	0.1393352	0.07700934	-0.0280231	1	0	0
cg11939550	0.14842679	0.05032155	-0.0815348	1	0	0
cg01778750	-0.143938	-0.0347103	0.06105126	1	0	0
cg22177688	-0.1488111	-0.0630434	0.07226275	1	0	0
cg13490895	0.17408674	0.13670369	0.02976974	1	1	1
cg07941680	0.1638013	0.0429157	-0.0676784	1	0	0

cg21273905	-0.1211585	-0.054121	0.05429615	1	0	0
cg21561953	-0.1809742	-0.0199859	0.1732252	1	0	0
cg14352416	-0.1228632	-0.0177217	0.10679329	1	0	0
cg22656115	-0.1230996	-0.0913412	-0.00151	1	1	1
cg06094959	0.0954332	0.01431672	-0.0180042	1	0	0
cg07331806	0.08888523	-0.0014728	-0.0501241	0	0	1
cg03889016	-0.1266973	-0.0304859	0.08986441	1	0	0
cg06635722	0.16454089	0.09005262	-0.0750708	1	0	0
cg02555952	0.17864125	0.05044362	-0.1021699	1	0	0
cg09209543	-0.1378601	-0.0312118	0.10313982	1	0	0
cg09515778	0.12929394	0.15523532	0.03874898	1	1	1
cg00700214	-0.2137022	-0.007632	0.14761221	1	0	0
cg03742937	0.10070596	0.0029364	-0.0462033	1	0	0
cg10713461	-0.1725289	-0.0389294	0.07386538	1	0	0
cg25282715	0.10227701	0.04964052	-0.0448009	1	0	0
cg22459179	-0.1727643	-0.0433609	0.12803241	1	0	0
cg10200551	-0.1174472	-0.0176822	0.07485647	1	0	0
cg06501295	0.2410021	0.27101567	-0.0166765	1	0	0
cg17459557	-0.1712049	-0.087246	0.08855605	1	0	0
cg22748272	-0.1363667	-0.1415079	0.03357029	1	0	0
cg10918729	-0.1429929	-0.0584347	0.0847403	1	0	0
cg10327067	0.19366445	0.00540568	-0.1126151	1	0	0
cg07383076	-0.1192016	-0.0671855	0.01604339	1	0	0
cg25734127	-0.1412936	-0.0550251	0.17195393	1	0	0
cg11972332	0.09375972	0.07539147	0.01868403	1	1	1
cg03954573	0.10125271	0.0455855	-0.0496591	1	0	0
cg07356006	-0.107201	0.01287019	0.11193425	0	0	1
cg26845404	-0.1476008	-0.0318224	0.11069315	1	0	0
cg18843853	-0.2303018	-0.1136767	0.15117094	1	0	0
cg02236874	-0.1467471	-0.0939499	0.0341945	1	0	0
cg22684717	0.15752333	0.10958078	-0.0524231	1	0	0

cg18533313	-0.1491586	-0.0522342	0.09387557	1	0	0
cg10112391	0.1961397	0.14733601	-0.0337927	1	0	0
cg21787305	0.11193218	0.02149449	-0.0275355	1	0	0
cg13941682	-0.3473125	-0.2854951	0.1133499	1	0	0
cg15209200	0.0771206	0.01200514	-0.0532169	1	0	0
cg05729783	-0.1245238	-0.0424677	0.09683817	1	0	0
cg08116144	-0.1264303	0.00089336	0.12485824	0	0	1
cg15289144	-0.137902	-0.0285627	0.0961937	1	0	0
cg18593893	-0.1373322	0.00904973	0.2091077	0	0	1
cg10702457	-0.1421516	-0.0573955	0.12005361	1	0	0
cg06446550	0.09149085	-0.0259972	-0.1169123	0	0	1
cg04604962	-0.1174361	-0.0175273	0.08894701	1	0	0
cg06524507	-0.161297	-0.0829193	0.09271354	1	0	0
cg07557896	-0.1256677	-0.09389	0.01450332	1	0	0
cg09365284	-0.1201825	-0.0315285	0.08165125	1	0	0
cg02979978	-0.1794938	0.08617837	0.1131614	0	0	1
cg16579641	-0.2471172	-0.1729276	0.06552662	1	0	0
cg21124196	-0.2082795	-0.0910102	0.07347332	1	0	0
cg21542223	0.13118005	0.06275351	-0.0416182	1	0	0
cg24923430	-0.6095226	-0.2755184	0.2308464	1	0	0
cg23204701	-0.1137467	-0.0558496	0.05671142	1	0	0
cg21724915	-0.2264441	-0.0096747	0.23720726	1	0	0
cg18706960	-0.1121245	-0.030789	0.0554569	1	0	0
cg04247069	-0.1355988	0.00589086	0.0936627	0	0	1
cg14023398	0.10654976	0.0493526	0.01057427	1	1	1
cg15836785	-0.130047	-0.0860104	0.05280037	1	0	0
cg11591082	0.09217036	0.03362673	-0.0318798	1	0	0
cg13633625	-0.1290035	-0.0698716	0.03347125	1	0	0
cg25347264	0.21564786	0.18185065	-0.1125641	1	0	0
cg10945695	-0.1086507	-0.0936089	0.0257162	1	0	0
cg12958961	0.40643363	0.43301645	0.05192105	1	1	1

cg21698295	0.13716108	0.07146335	0.00381575	1	1	1
cg10716697	-0.1807089	0.00086481	0.05519865	0	0	1
cg06012090	0.13965029	0.03446365	-0.0423397	1	0	0
cg04446579	0.1069381	0.06953385	-0.030001	1	0	0
cg16526526	-0.11402	-0.0451651	0.01795458	1	0	0
cg08321827	0.09497439	-0.0256219	-0.1018763	0	0	1
cg26532102	-0.1045131	-0.0375563	0.0911454	1	0	0
cg11142556	-0.1410129	-0.0700325	0.0653358	1	0	0
cg07434077	0.08735123	-0.0131929	-0.0678952	0	0	1
cg14366146	-0.1225081	-0.0392263	0.08212431	1	0	0
cg26348343	-0.1266378	-0.0609965	0.05039623	1	0	0
cg19298856	0.08623156	0.03003087	-0.0114912	1	0	0
cg02094681	0.63057191	0.04834704	-0.3686731	1	0	0
cg04988103	-0.1730105	-0.0291931	0.11251192	1	0	0
cg14756006	-0.1660205	-0.1112854	0.02255271	1	0	0
cg13813391	0.11926712	-0.0022024	-0.1108516	0	0	1
cg00559992	-0.1412782	0.05697141	0.22668009	0	0	1
cg14868747	0.14432251	0.12510775	-0.0109241	1	0	0
cg13151006	-0.1242503	-0.0697652	0.03901035	1	0	0
cg14788130	-0.1115032	-0.0366434	0.0573403	1	0	0
cg27510066	-0.2105869	-0.0678486	0.06094049	1	0	0
cg00550132	-0.1247522	-0.0729555	0.02814573	1	0	0
cg09413013	0.18268551	0.12855083	-0.0849772	1	0	0
cg22465490	-0.2003954	-0.0812562	0.04770372	1	0	0
cg00121135	0.08628729	-0.0243707	-0.1135257	0	0	1
cg20232723	-0.1673263	-0.0718609	0.09425339	1	0	0
cg23722790	0.10976009	0.08813185	0.00608498	1	1	1
cg24587119	-0.1533102	0.00435914	0.15866383	0	0	1
cg24968203	0.16488211	-0.0429359	-0.1590691	0	0	1
cg26485878	0.11026598	0.07185038	0.0003007	1	1	1
cg07708721	0.09104437	-0.0193321	-0.078395	0	0	1

cg22805688	0.29130484	0.1382849	-0.0933644	1	0	0

Supplementary Table S7. List of overlapping DMPs at $p < 0.001$ across the three contrasts.

Contrasts	CpGs
BDSA vs. BD/non-SA, BD/SA vs. CON	cg13954127
	cg22025478
	cg23991388
	cg26318502
	cg02141358
	cg12508286
	cg13213598
	cg12445832
	cg05928239
	cg24343835
	cg05132851
	cg09137630
	cg17927434
	cg04194173
	cg06079564
	cg05511978
	cg06330621
	cg01541867
	cg09515778
	cg12958961
**Contrasts**	**CpGs**
BD/SA vs. BD/non-SA, BD/non-SA vs. CON	cg15653194
	cg07058706
	cg12404216
	cg14790883
	cg11518826
	cg10763891
	cg09007811
	cg09390308
	cg18356679
	cg00404394
	cg02411284
	cg26377940
	cg16450683
	cg26009832
	cg13588483
	cg01137798
	cg10018232
	cg13685026

	cg12294213
	cg03094728
	cg00803692
	cg11942603
	cg22695117
	cg15756091
	cg27628536
	cg17571306
	cg03860720
	cg17118948
	cg10207609
	cg05481881
	cg15246895
	cg14703589
	cg14856316
	cg10189135
	cg09348353
	cg18326313
	cg21849126
	cg01449754
	cg24310780
	cg00424066
	cg09606588
	cg13995568
	cg20521672
	cg07422110
	cg19186966
	cg13552468
	cg26015743
	cg16685388
	cg12767180
	cg00845279
	cg09601692
	cg11715789
	cg16992787
	cg18462898
	cg03506768
	cg01876832
	cg20816857
	cg15613153
	cg18593893
	cg00559992
**Contrasts**	**CpGs**

BD/SA vs. CON, BD/non-SA vs. CON	cg11199428
	cg08118646
	cg17192519
	cg26899692
	cg23928910
	cg10354859
	cg13711528
	cg01963885
	cg22792660
	cg01757209
	cg20656602
	cg01380244
	cg15963109
	cg11149658
	cg25742246
	cg12882476
	cg19737037
	cg05295810
	cg20131685
	cg10749792
	cg20807920
	cg01017197
	cg08853050
	cg14288330
	cg21189070
	cg24171337
	cg08758850
	cg08360511
	cg05806333
	cg14673706
	cg17402072
	cg11635143
	cg16948082
	cg02510471
	cg26685590
	cg16651196
	cg06025835
	cg22957381
	cg05192108
	cg15141371
	cg25766271
	cg02464357
	cg17487553

cg22896904
cg17413750
cg26395601
cg26172013
cg17568129
cg03043296
cg24322144
cg15988263
cg00436174
cg04442328
cg19204631
cg17122153
cg04360780
cg06532184
cg14558639
cg08889009
cg03151917
cg26085966
cg00918917
cg26831081
cg19194233
cg16705665
cg13184886
cg06824641
cg06807126
cg02503933
cg21348997
cg14719675
cg21959181
cg01805480
cg00092682
cg27218829
cg23894571
cg09247534
cg11898212
cg05666842
cg14985478
cg18598695
cg07055694
cg02783889
cg24449371
cg25494767
cg00176718

cg01870619
cg04046119
cg08431549
cg19188060
cg18054632
cg19143760
cg23015157
cg22152279
cg11358257
cg07523098
cg01021488
cg21633795
cg23069057
cg15084499
cg08754068
cg06134974
cg19130836
cg14716208
cg05253604
cg04689371
cg11857025
cg00687766
cg10894807
cg19763809
cg06993041
cg07729059
cg16561543
cg26015888
cg26532042
cg03559817
cg09231514
cg25364898
cg19780254
cg25327839
cg11464238
cg18222526
cg07617636
cg22736787
cg00189906
cg00529697
cg11835459
cg21842523
cg11424597

cg05218311
cg26825614
cg25005633
cg16718920
cg03908270
cg09435821
cg20027456
cg12600163
cg23700226
cg12952012
cg16960046
cg03446973
cg26861066
cg20014094
cg18676500
cg15646911
cg11973777
cg24504927

Supplementary Table S8. Blood-brain correlations for the six FDR-significant DMPs from the BD/SA vs. BD/non-SA EWAS.

chr	probe	rho	gene	gene distance	gene feature
chr4	cg20244265	-0.01	*CXCL8*	-22861	intergenic
chr17	cg25876840	0.21	*CD300LG*	-4066	intergenic
chr17	cg11476866	0.37	*CD300LG*	-4128	intergenic
chr7	cg15653194	-0.49	*LFNG*	0;0	exon+utr5;intron
chr2	cg00306112	0.3	*LINC01494*	0	nc_intron
chr7	cg20242392	0.37	*DGKI*	0;0	intron;intron+utr5

Supplementary Table S9. Genomic location enrichment results for the discovery cohort EWAS.

**BD/SA vs. BD/non-SA**	**location**	**OR**	**p**	**input**	**background**
	Island	0.371	2.02E-42	0.075	0.18
	OpenSea	1.531	1.14E-20	0.664	0.563
	TSS200	0.529	1.92E-12	0.052	0.094
	Intergenic	1.32	5.43E-09	0.327	0.269
	1stExon	0.525	6.58E-09	0.034	0.062
	S_Shelf	0.786	0.04228965	0.035	0.044
	TSS1500	0.913	0.15248961	0.143	0.154
	S_Shore	1.113	0.16214594	0.091	0.083
	N_Shore	1.07	0.36649436	0.091	0.086
	Body	0.993	0.89446676	0.447	0.449
	N_Shelf	0.981	0.91479497	0.043	0.044
	5'UTR	0	1	0	0
	3'UTR	0	1	0	0
**BD/SA vs. CON**	**location**	**OR**	**p**	**input**	**background**
	Island	5.044	1.38E-127	0.524	0.18
	OpenSea	0.252	1.08E-88	0.245	0.563
	TSS200	3.279	1.80E-46	0.254	0.094
	1stExon	3.125	1.26E-31	0.172	0.062
	Intergenic	0.526	6.73E-15	0.162	0.269
	Body	0.743	7.90E-06	0.377	0.449
	TSS1500	1.372	0.00016041	0.2	0.154
	N_Shelf	0.677	0.03995147	0.03	0.044
	S_Shelf	0.723	0.08278636	0.032	0.044
	S_Shore	1.001	0.95315457	0.083	0.083
	5'UTR	0	1	0	0
	3'UTR	0	1	0	0
	N_Shore	0.989	1	0.085	0.086
**BD/non-SA vs. CON**	**location**	**OR**	**p**	**input**	**background**
	Island	3.156	2.27E-136	0.408	0.18
	OpenSea	0.463	1.84E-70	0.374	0.563
	TSS200	2.448	9.52E-53	0.202	0.094
	1stExon	2.497	9.43E-41	0.142	0.062
	Body	0.717	4.05E-14	0.369	0.449
	Intergenic	0.759	5.25E-08	0.219	0.269
	TSS1500	1.285	8.33E-06	0.19	0.154
	S_Shelf	0.63	0.00016987	0.028	0.044
	N_Shelf	0.737	0.00908905	0.033	0.044
	N_Shore	0.878	0.10792906	0.076	0.086

S_Shore	0.973	0.78522912	0.081	0.083
5'UTR	0	1	0	0
3'UTR	0	1	0	0

Supplementary Table S10. Trait enrichment results for the discovery cohort EWAS.

BD/SA vs. BD/non-SA	trait	p	OR	DE	background	CpGs	background
	fractional exhaled nitric oxide	3.36E-260	12.348	199	8372	199	8372
	allergic sensitization	6.67E-45	10.962	34	1501	34	1501
	allergic asthma	1.27E-43	12.166	31	1235	31	1235
	aging	1.05E-30	2.048	149	31184	149	31184
	childhood asthma	1.38E-20	13.771	13	456	13	456
	ancestry	7.29E-20	2.614	62	10618	62	10618
	systemic lupus erythematosus (SLE)	2.51E-19	2.954	49	7848	49	7848
	atopy	7.17E-18	3.129	41	6198	41	6198
	myalgic encephalomyelitis/chronic fatigue syndrome	1.12E-17	3.056	42	6500	42	6500
	Claes-Jensen syndrome	3.87E-17	7.262	16	1050	16	1050
	alcohol consumption	1.72E-13	4.695	18	1816	18	1816
	Werner syndrome	1.93E-13	6.11	14	1088	14	1088
	air pollution (NO2)	4.85E-13	2.912	32	5185	32	5185
	down syndrome	5.41E-13	1.92	63	14649	63	14649
	perinatally-acquired HIV	1.39E-11	5.066	14	1309	14	1309
	Parkinson's disease (PD)	4.40E-11	12.087	7	278	7	278
	epithelial ovarian cancer risk	8.14E-09	21.988	4	89	4	89
	multiple sclerosis	2.65E-08	2.086	36	8093	36	8093
	Crohn's disease (CD)	5.47E-08	2.996	17	2672	17	2672
	oral squamous cell carcinoma (OSCC)	3.73E-07	1.63	48	14095	48	14095
	Coffin‚ÄìSiris syndrome (CSS)	3.90E-07	8.888	5	268	5	268
	neurodevelopmental presentations and congenital anomalies (ND/CAs)	1.66E-06	4.412	8	856	8	856
	circadian rhythm	3.27E-06	6.954	5	341	5	341
	prostate cancer	5.34E-06	1.927	29	7047	29	7047

	preterm birth	1.23E-05	1.682	41	10662	41	10662
	maternal smoking	3.00E-05	1.902	25	6153	25	6153
	leukoaraiosis (LA)	0.00013189	4.442	5	531	5	531
	SETD1B-related syndrome	0.0002045	2.242	13	2721	13	2721
	prenatal perfluoroalkyl substance exposure	0.00034393	3.926	5	600	5	600
	B Acute Lymphoblastic Leukemia with t(12;21)(p13.2;q22.1); ETV6-RUNX1	0.00034469	1.866	19	4768	19	4768
	cognitive function	0.00035254	4.828	4	391	4	391
	follicular thyroid carcinoma	0.00054413	1.762	21	5575	21	5575
	absolute fat free mass (FFM, kg)	0.00084822	5.673	3	250	3	250
	attention-deficit/hyperactivity disorder (ADHD)	0.00160258	38.936	1	13	1	13
	Kabuki syndrome (KS)	0.00183641	2.233	9	1891	9	1891
	polybrominated biphenyl exposure	0.00391383	2.182	8	1719	8	1719
	alcohol treatment	0.00397695	23.355	1	21	1	21
	Huntington's disease (HD)	0.00397695	23.355	1	21	1	21
	diastolic blood pressure	0.00472489	21.217	1	23	1	23
	systolic blood pressure	0.00472489	21.217	1	23	1	23
	autoimmune diseases	0.00593658	3.796	3	372	3	372
	sperm viability	0.00629553	2.614	5	898	5	898
	colorectal cancer overall survival	0.00639836	17.955	1	27	1	27
	acute myelocytic leukemia (AML)	0.00685296	17.288	1	28	1	28
	maternal hypertensive disorders in pregnancy	0.00749158	2.546	5	922	5	922
	osteonecrosis of the femoral head (ONFH)	0.00830157	15.561	1	31	1	31
	1,6-hexamethylene diisocyanate exposure	0.0088123	15.06	1	32	1	32
**BD/SA vs. CON**	**trait**	**p**	**OR**	**DE**	**background**	**CpGs**	**background**
	Behcet's disease	2.28E-50	9.975	41	4364	41	4364
	metabolic syndrome (MetS)	4.63E-14	15.417	8	539	8	539
	colorectal laterally spreading tumor	1.85E-12	3.247	26	8245	26	8245
	prostate cancer	1.14E-10	3.209	22	7047	22	7047

	colorectal cancer	1.50E-10	3.004	24	8207	24	8207
	childhood stress	2.03E-09	11.262	6	550	6	550
	B Acute Lymphoblastic Leukemia with t(1;19)(q23;p13.3); E2A-PBX1 (TCF3-PBX1)	2.51E-08	1.859	48	21099	48	21099
	insulin resistance	1.13E-07	7.732	6	798	6	798
	maternal circadian disruption	2.09E-07	13.873	4	298	4	298
	Helicobacter pylori infection	4.69E-06	4.054	8	2021	8	2021
	maternal eating disorders	3.85E-05	335.843	1	4	1	4
	hepatocellular carcinoma (HCC)	4.35E-05	2.134	18	8598	18	8598
	hepatocellular carcinoma overall survival	5.77E-05	254.596	1	5	1	5
	serum IL8 levels	5.77E-05	254.596	1	5	1	5
	serum dioxins and dioxin-like compounds level	0.00095137	48.367	1	22	1	22
	nasopharyngeal carcinoma	0.0015081	3.827	4	1068	4	1068
	prenatal socioeconomic status	0.00162089	36.314	1	29	1	29
	schizophrenia	0.00181042	2.78	6	2202	6	2202
	child abuse	0.00259275	2.441	7	2924	7	2924
	autism spectrum disorders (ASD)	0.00465	3.99	3	768	3	768
	serum IL-10 levels	0.00480148	20.33	1	51	1	51
	interstitial cystitis/bladder pain syndrome (IC/BPS)	0.00552832	5.829	2	351	2	351
	adrenocortical carcinoma	0.00646876	2.992	4	1364	4	1364
	kidney disease	0.00669506	3.69	3	830	3	830
	meditation practice	0.00675908	16.952	1	61	1	61
**BD/non-SA vs. CON**	**trait**	**p**	**OR**	**DE**	**background**	**CpGs**	**background**
	Behcet's disease	2.13E-105	9.648	90	4364	90	4364
	metabolic syndrome (MetS)	2.68E-14	9.272	11	539	11	539

polycystic ovary syndrome (PCOS)	1.30E-09	16.817	5	137	5	137
kidney disease	9.56E-08	4.87	9	830	9	830
treated with assisted reproductive technology	2.03E-05	11.575	3	118	3	118
insulin resistance	2.51E-05	3.928	7	798	7	798
child abuse	5.02E-05	2.287	15	2924	15	2924
B Acute Lymphoblastic Leukemia with t(1;19)(q23;p13.3); E2A-PBX1 (TCF3-PBX1)	7.67E-05	1.369	76	21099	76	21099
major depression disorder	0.00014177	4.403	5	509	5	509
prenatal arsenic exposure	0.00035684	1.83	20	4860	20	4860
colorectal laterally spreading tumor	0.00041798	1.613	30	8245	30	8245
perimembranous ventricular septal defect	0.00071172	63.353	1	8	1	8
maternal pre-pregnancy central obesity	0.000887	55.391	1	9	1	9
plasma fasting insulin levels	0.00152374	40.301	1	12	1	12
nonobstructive azoospermia (NOA)	0.00400568	23.351	1	20	1	20
alcohol treatment	0.00439316	22.16	1	21	1	21
maternal alcohol consumption	0.00565403	1.854	11	2640	11	2640
prenatal bisphenol A (BPA) exposure	0.00645353	2.996	4	596	4	596
prenatal socioeconomic status	0.0080785	15.835	1	29	1	29
osteonecrosis of the femoral head (ONFH)	0.00915687	14.781	1	31	1	31
response to dietary stilbenoids	0.00971879	14.306	1	32	1	32

Supplementary Table S11. Gene ontology enrichment results for the discovery cohort EWAS.

BD/SA vs. BD/non-SA	GO	term	type	CpGs	background	p	FDR
	GO:0051924	regulation of calcium ion transport	BP	36	2.53E+02	5.35E-05	1
	GO:0055117	regulation of cardiac muscle contraction	BP	17	8.20E+01	0.0001231	1
	GO:0006816	calcium ion transport	BP	55	4.31E+02	0.0001526	1
	GO:0006942	regulation of striated muscle contraction	BP	18	9.60E+01	0.0002315	1
	GO:1903169	regulation of calcium ion transmembrane transport	BP	24	1.50E+02	0.0003002	1
	GO:0098839	postsynaptic density membrane	CC	17	7.40E+01	0.000331	1
	GO:0099061	integral component of postsynaptic density membrane	CC	13	5.00E+01	0.0003763	1
	GO:0070296	sarcoplasmic reticulum calcium ion transport	BP	10	4.20E+01	0.0004223	1
	GO:0070838	divalent metal ion transport	BP	57	4.80E+02	0.0004439	1
	GO:0010959	regulation of metal ion transport	BP	47	3.91E+02	0.0004775	1
	GO:0099146	intrinsic component of postsynaptic density membrane	CC	13	5.30E+01	0.0005362	1
	GO:0072511	divalent inorganic cation transport	BP	57	4.86E+02	0.0005654	1
	GO:0014808	release of sequestered calcium ion into cytosol by sarcoplasmic reticulum	BP	9	3.60E+01	0.000609	1
	GO:1903514	release of sequestered calcium ion into cytosol by endoplasmic reticulum	BP	9	3.70E+01	0.0006672	1
	GO:1901841	regulation of high voltage-gated calcium channel activity	BP	6	1.50E+01	0.0009966	1
	GO:1901019	regulation of calcium ion transmembrane transporter activity	BP	16	8.80E+01	0.0011509	1
	GO:0015747	urate transport	BP	3	5.00E+00	0.0012988	1
	GO:0010880	regulation of release of sequestered calcium ion into cytosol by sarcoplasmic reticulum	BP	8	3.20E+01	0.0013694	1
	GO:0005246	calcium channel regulator activity	MF	10	4.80E+01	0.0014892	1
	GO:0035303	regulation of dephosphorylation	BP	29	2.08E+02	0.0017109	1

GO:0030326	embryonic limb morphogenesis	BP	23	125	0.0017611	1
GO:0035113	embryonic appendage morphogenesis	BP	23	125	0.0017611	1
GO:0001501	skeletal system development	BP	60	514	0.0018192	1
GO:0070588	calcium ion transmembrane transport	BP	41	312	0.0020103	1
GO:0009790	embryo development	BP	105	1015	0.002047	1
GO:0032279	asymmetric synapse	CC	47	326	0.0020502	1
GO:1904062	regulation of cation transmembrane transport	BP	41	338	0.0020887	1
GO:1900127	positive regulation of hyaluronan biosynthetic process	BP	3	4	0.0021991	1
GO:0086064	cell communication by electrical coupling involved in cardiac conduction	BP	7	24	0.0022173	1
GO:0042733	embryonic digit morphogenesis	BP	13	61	0.002316	1
GO:0099634	postsynaptic specialization membrane	CC	19	101	0.0023571	1
GO:0032412	regulation of ion transmembrane transporter activity	BP	33	256	0.0026133	1
GO:0014069	postsynaptic density	CC	46	322	0.0027739	1
GO:0005942	phosphatidylinositol 3-kinase complex	CC	7	27	0.0028773	1
GO:0099060	integral component of postsynaptic specialization membrane	CC	15	74	0.0029056	1
GO:0030546	receptor activator activity	MF	5	11	0.0030564	1
GO:1900125	regulation of hyaluronan biosynthetic process	BP	4	8	0.0032008	1
GO:0046854	phosphatidylinositol phosphorylation	BP	10	50	0.0032961	1
GO:0035107	appendage morphogenesis	BP	25	148	0.003345	1
GO:0035108	limb morphogenesis	BP	25	148	0.003345	1
GO:0004031	aldehyde oxidase activity	MF	2	2	0.0033516	1
GO:0016623	oxidoreductase activity, acting on the aldehyde or oxo group of donors, oxygen as acceptor	MF	2	2	0.0033516	1
GO:0048736	appendage development	BP	28	178	0.003377	1
GO:0060173	limb development	BP	28	178	0.003377	1
GO:0010882	regulation of cardiac muscle contraction by calcium ion signaling	BP	7	29	0.0034718	1

GO:0098948	intrinsic component of postsynaptic specialization membrane	CC	15	77	0.0036506	1
GO:0004439	phosphatidylinositol-4,5-bisphosphate 5-phosphatase activity	MF	4	9	0.0038362	1
GO:0022898	regulation of transmembrane transporter activity	BP	33	264	0.0039966	1
GO:0046834	lipid phosphorylation	BP	12	64	0.0040013	1
GO:0006172	ADP biosynthetic process	BP	3	5	0.004052	1
GO:0043269	regulation of ion transport	BP	71	694	0.0040883	1
GO:1901385	regulation of voltage-gated calcium channel activity	BP	8	35	0.0043832	1
GO:0032409	regulation of transporter activity	BP	34	279	0.0044122	1
GO:0099572	postsynaptic specialization	CC	47	346	0.0046093	1
GO:0035304	regulation of protein dephosphorylation	BP	20	138	0.0046587	1
GO:0010921	regulation of phosphatase activity	BP	24	174	0.0048798	1
GO:0070782	phosphatidylserine exposure on apoptotic cell surface	BP	3	5	0.0050142	1
GO:0046899	nucleoside triphosphate adenylate kinase activity	MF	2	2	0.005151	1
GO:0042056	chemoattractant activity	MF	6	38	0.005251	1
GO:1904026	regulation of collagen fibril organization	BP	3	7	0.0053191	1
GO:0099604	ligand-gated calcium channel activity	MF	8	27	0.0055374	1
GO:0048568	embryonic organ development	BP	50	428	0.0055427	1
GO:0045123	cellular extravasation	BP	10	60	0.0056677	1
GO:0046488	phosphatidylinositol metabolic process	BP	23	174	0.0057086	1
GO:0048562	embryonic organ morphogenesis	BP	37	288	0.0058377	1
GO:0048598	embryonic morphogenesis	BP	64	585	0.0059817	1
GO:0002720	positive regulation of cytokine production involved in immune response	BP	9	53	0.0060256	1
GO:0090075	relaxation of muscle	BP	8	31	0.0063627	1
GO:0045211	postsynaptic membrane	CC	43	322	0.006407	1
GO:0030157	pancreatic juice secretion	BP	4	12	0.006591	1

GO:1903979	negative regulation of microglial cell activation	BP	3	7	0.0066081	1
GO:0010644	cell communication by electrical coupling	BP	7	29	0.0066385	1
GO:0032819	positive regulation of natural killer cell proliferation	BP	3	7	0.006681	1
GO:0048280	vesicle fusion with Golgi apparatus	BP	3	7	0.0070524	1
GO:0030001	metal ion transport	BP	85	876	0.0070543	1
GO:0098984	neuron to neuron synapse	CC	47	347	0.007063	1
GO:0007611	learning or memory	BP	34	256	0.0072951	1
GO:0015114	phosphate ion transmembrane transporter activity	MF	4	11	0.0075001	1
GO:0006022	aminoglycan metabolic process	BP	23	169	0.0075259	1
GO:0051209	release of sequestered calcium ion into cytosol	BP	18	124	0.0076818	1
GO:0005801	cis-Golgi network	CC	9	65	0.0077344	1
GO:0071149	TEAD-2-YAP complex	CC	2	2	0.0078544	1
GO:0004620	phospholipase activity	MF	15	102	0.0080916	1
GO:1990403	embryonic brain development	BP	4	13	0.0081731	1
GO:1904888	cranial skeletal system development	BP	12	66	0.0081918	1
GO:0099106	ion channel regulator activity	MF	16	116	0.0082454	1
GO:0097553	calcium ion transmembrane import into cytosol	BP	20	140	0.0085254	1
GO:0061448	connective tissue development	BP	34	271	0.0085457	1
GO:0072672	neutrophil extravasation	BP	4	12	0.0088904	1
GO:0009136	purine nucleoside diphosphate biosynthetic process	BP	3	6	0.0089169	1
GO:0009180	purine ribonucleoside diphosphate biosynthetic process	BP	3	6	0.0089169	1
GO:0009188	ribonucleoside diphosphate biosynthetic process	BP	3	6	0.0089169	1
GO:0051283	negative regulation of sequestering of calcium ion	BP	18	126	0.009176	1
GO:0006811	ion transport	BP	144	1677	0.0092131	1
GO:0006812	cation transport	BP	104	1142	0.0094919	1
GO:0062023	collagen-containing extracellular matrix	CC	42	406	0.0095737	1
GO:0098633	collagen fibril binding	MF	2	2	0.0098202	1

	GO:0051282	regulation of sequestering of calcium ion	BP	18	128	0.009925	1
**BD/SA vs. CON**	**GO**	**term**	**type**	**CpGs**	**background**	**p**	**FDR**
	GO:0017014	protein nitrosylation	BP	5	15	0.0005343	1
	GO:0018119	peptidyl-cysteine S-nitrosylation	BP	5	15	0.0005343	1
	GO:0070164	negative regulation of adiponectin secretion	BP	3	4	0.0005878	1
	GO:2000169	regulation of peptidyl-cysteine S-nitrosylation	BP	4	9	0.0007701	1
	GO:0070162	adiponectin secretion	BP	3	6	0.0015035	1
	GO:0070163	regulation of adiponectin secretion	BP	3	6	0.0015035	1
	GO:0015816	glycine transport	BP	4	12	0.0019542	1
	GO:0046823	negative regulation of nucleocytoplasmic transport	BP	5	20	0.0023917	1
	GO:1902186	regulation of viral release from host cell	BP	6	31	0.0027428	1
	GO:0090038	negative regulation of protein kinase C signaling	BP	2	2	0.0035364	1
	GO:1902083	negative regulation of peptidyl-cysteine S-nitrosylation	BP	3	7	0.0038408	1
	GO:0060465	pharynx development	BP	2	3	0.0044356	1
	GO:0008355	olfactory learning	BP	2	2	0.004445	1
	GO:0015187	glycine transmembrane transporter activity	MF	3	8	0.0048227	1
	GO:0019076	viral release from host cell	BP	6	35	0.005132	1
	GO:0035890	exit from host	BP	6	35	0.005132	1
	GO:0035891	exit from host cell	BP	6	35	0.005132	1
	GO:0052126	movement in host environment	BP	6	35	0.005132	1
	GO:0052192	movement in environment of other organism involved in symbiotic interaction	BP	6	35	0.005132	1
	GO:1902033	regulation of hematopoietic stem cell proliferation	BP	3	10	0.0055409	1
	GO:0099571	postsynaptic cytoskeleton	CC	4	8	0.0061016	1
	GO:0030145	manganese ion binding	MF	9	62	0.0066375	1
	GO:0097177	mitochondrial ribosome binding	MF	2	3	0.006814	1

	GO	term	type	CpGs	background	p	FDR
	GO:0044752	response to human chorionic gonadotropin	BP	2	3	0.007221	1
	GO:0022829	wide pore channel activity	MF	4	21	0.0073145	1
	GO:2000297	negative regulation of synapse maturation	BP	2	3	0.0084605	1
	GO:0034389	lipid droplet organization	BP	4	21	0.0086102	1
	GO:0101006	protein histidine phosphatase activity	MF	2	3	0.0087255	1
	GO:0090317	negative regulation of intracellular protein transport	BP	7	41	0.0088853	1
	GO:0010610	regulation of mRNA stability involved in response to stress	BP	2	3	0.0092939	1
	GO:0042249	establishment of planar polarity of embryonic epithelium	BP	4	17	0.0096051	1
	GO:2000249	regulation of actin cytoskeleton reorganization	BP	6	37	0.0096358	1
	GO:0007172	signal complex assembly	BP	3	8	0.0096767	1
**BD/non-SA vs. CON**	**GO**	**term**	**type**	**CpGs**	**background**	**p**	**FDR**
	GO:0004082	bisphosphoglycerate mutase activity	MF	3	4	0.0006255	1
	GO:0004619	phosphoglycerate mutase activity	MF	3	4	0.0006255	1
	GO:0060712	spongiotrophoblast layer development	BP	6	13	0.0008952	1
	GO:0003713	transcription coactivator activity	MF	49	318	0.0014133	1
	GO:1903508	positive regulation of nucleic acid-templated transcription	BP	190	1608	0.001753	1
	GO:1902680	positive regulation of RNA biosynthetic process	BP	190	1609	0.0017932	1
	GO:0045893	positive regulation of transcription, DNA-templated	BP	180	1523	0.0024104	1
	GO:0051254	positive regulation of RNA metabolic process	BP	198	1693	0.0026147	1
	GO:1903525	regulation of membrane tubulation	BP	4	6	0.0026627	1
	GO:0051238	sequestering of metal ion	BP	5	15	0.0026773	1
	GO:0043009	chordate embryonic development	BP	85	625	0.002919	1
	GO:0045935	positive regulation of nucleobase-containing compound metabolic process	BP	215	1883	0.0035072	1
	GO:0035748	myelin sheath abaxonal region	CC	4	6	0.0039656	1

GO:0071568	UFM1 transferase activity	MF	2	2	0.004025	1
GO:0003183	mitral valve morphogenesis	BP	5	10	0.0040882	1
GO:0010557	positive regulation of macromolecule biosynthetic process	BP	210	1866	0.0050167	1
GO:2000793	cell proliferation involved in heart valve development	BP	3	4	0.0055711	1
GO:0009792	embryo development ending in birth or egg hatching	BP	85	642	0.0056817	1
GO:0003174	mitral valve development	BP	5	11	0.0059325	1
GO:0016868	intramolecular transferase activity, phosphotransferases	MF	4	11	0.0059328	1
GO:0048239	negative regulation of DNA recombination at telomere	BP	2	2	0.0066729	1
GO:0072695	regulation of DNA recombination at telomere	BP	2	2	0.0066729	1
GO:0031848	protection from non-homologous end joining at telomere	BP	5	10	0.0067266	1
GO:0005667	transcription factor complex	CC	51	361	0.0067646	1
GO:0005026	transforming growth factor beta receptor activity, type II	MF	3	5	0.0078693	1
GO:0043565	sequence-specific DNA binding	MF	133	1120	0.008439	1
GO:0043461	proton-transporting ATP synthase complex assembly	BP	3	7	0.0085993	1
GO:0009891	positive regulation of biosynthetic process	BP	219	2009	0.0091736	1
GO:0030332	cyclin binding	MF	9	30	0.0096946	1
GO:0003828	alpha-N-acetylneuraminate alpha-2,8-sialyltransferase activity	MF	3	5	0.0098909	1
GO:0001701	in utero embryonic development	BP	52	372	0.0098959	1

Supplementary Table S12. Results from the GLM relating WGCNA eigengene expression to SA history among individuals with BD.

**Module**	**Module size (probes)**	**Beta**	**SE**	***P value***	***P FDR***
MEbrown	4539	77.70067	15.99112	1.18E-06	7.08E-06
MEblue	4428	-24.27863	5.71424	2.15E-05	6.45E-05
MEturquoise	7237	-19.76762	4.82451	4.18E-05	8.36E-05
MEpink	188	11.175809	3.233436	0.000548	6.58E-04
MEblack	354	8.98236	2.58119	0.000502	6.58E-04
MEgreen	939	8.75756	2.68679	0.00112	1.12E-03

Supplementary Table S13. Comprehensive information for the probes included in the WGCNA analysis.

Probe ID	Gene annotation	module	MM.turquoise	MM.blue	MM.green	MM.pink	MM.brown	MM.black
cg15653194	LFNG;LFNG	black	0.07262858	0.06328434	-0.4043719	-0.2273921	-0.0459947	-0.4386732
cg02277821	SORD;SORD;SORD;SORD	black	0.08376307	-0.0510605	0.42438269	0.47439195	0.12709878	0.609876
cg27016609	STON1-GTF2A1L;STON1-	black	0.25125777	-0.0169478	0.31314333	0.59474701	0.42681854	0.57173378
cg01916685	SERHL;SERHL;SERHL;SER	black	-0.0976621	-0.3488055	0.34647953	0.18702826	0.36829806	0.590126
cg05396503	MAPT;MAPT;MAPT;MAPT	black	0.39650598	0.39307532	0.16265982	0.01132345	-0.2081194	-0.2861962
cg15312298	FAM84B;RP11-	black	0.19539627	0.10612119	-0.1570377	-0.0311095	0.02432995	-0.348219
cg04969808	WNT7A	black	0.05826386	0.04190353	-0.3695981	-0.0462251	-0.0239526	-0.3705411
cg07898796	RPPH1;PARP2;RPPH1;PAR	black	0.05273083	0.15589508	-0.3696347	-0.0924519	-0.0930735	-0.5320596
cg25654697	RPA3;RPA3;RPA3;AC00646	black	0.01896716	0.09372607	-0.2144744	-0.1410568	-0.093695	-0.4269475
cg02697074	TUBG1;FAM134C;FAM134	black	-0.2015696	-0.0494099	-0.3824543	-0.3060126	-0.1597339	-0.5094761
cg01261044	SRP54;SRP54;SRP54;SRP54	black	-0.1079423	0.06839016	-0.4470829	-0.1403242	-0.1253834	-0.5999998
cg01132893	HOXB7;RP11-	black	0.14775995	-0.1384312	0.08602267	0.3318434	0.2352837	0.45634508
cg12315590	C3orf37;C3orf37;C3orf37;C3	black	-0.0364812	0.02175809	-0.3221429	-0.1900852	-0.0321368	-0.3552355
cg18652941	CD44;CD44;CD44;CD44;CD	black	-0.0759917	0.07486294	-0.3332562	-0.2412017	-0.1777643	-0.5167188
cg04527840	GAR1;GAR1;GAR1;GAR1;	black	-0.1040913	0.06284248	-0.3088626	-0.0520657	-0.0684886	-0.4533629
cg16646384	UBA5;UBA5;UBA5;UBA5;	black	0.051307	0.19932711	-0.2593238	-0.087789	-0.1616056	-0.3836074
cg15319641	ZNF331;ZNF331;ZNF331;Z	black	-0.2301775	-0.1856386	0.29927848	0.00558475	0.08125355	0.35238241
cg21337074	DOK4;DOK4;DOK4;DOK4;	black	0.07337647	0.01812731	0.32294227	0.18480632	0.01684401	0.51937322
cg20255711	PSENEN;U2AF1L4;U2AF1L	black	0.07674914	0.09093165	-0.2121245	-0.0828952	-0.0669288	-0.3168897
cg15743657	TAS1R2;RP13-279N23.2	black	0.1777387	-0.1836137	0.19508203	0.29310529	0.45482722	0.72725525
cg26894435	BMPR2;BMPR2;BMPR2	black	-0.0625494	-0.027077	-0.246432	-0.1810725	-0.0098213	-0.3779999
cg17482927	RGS9;RGS9	black	0.06822562	0.09355294	-0.1329519	-0.1585525	-0.0946844	-0.3569051
cg04291668	FAM219B;FAM219B;FAM2	black	0.01067096	0.06791775	-0.2493951	-0.0912002	-0.0204375	-0.4608411
cg01140202	RP11-219E7.3;RP11-219E7.3	black	0.22030974	-0.0866709	0.51348966	0.459579	0.39957717	0.56372894
cg03086376	HAGH;HAGH;HAGH;HAG	black	0.08046499	0.12106185	-0.3615475	-0.1231178	-0.0457775	-0.4966632
cg08667777	MANF;MANF;MANF;MAN	black	-0.0726162	0.11303657	-0.3540688	-0.1577847	-0.1401138	-0.4454069
cg10475014	SRRT;SRRT;SRRT;SRRT;S	black	0.32011453	-0.1153395	0.1577462	0.44117067	0.39320829	0.73077844
cg02141602	MEIS3;MEIS3;MEIS3;MEIS	black	-0.0103763	-0.0803905	0.3962149	0.15044178	0.11414734	0.59106409
cg26519745	ABCG1;RN5S492;ABCG1;T	black	0.02178766	0.06731808	-0.3771901	-0.1976316	-0.077295	-0.494279
cg15057251	MAPRE2;MAPRE2;MAPRE	black	-0.211942	-0.0508859	-0.2881146	-0.1884377	-0.0287721	-0.5784236
cg17408923	RANBP2;RANBP2;RANBP	black	-0.1646134	-0.0189391	-0.2708705	-0.1911613	-0.0790518	-0.5799718
cg11355319	LYSMD3;LYSMD3;AC0273	black	0.03817332	0.14693771	-0.370362	-0.0912465	-0.0543578	-0.501428
cg17004104	NNMT;NNMT;RP11-	black	-0.0670352	-0.3096375	0.44383744	0.09175808	0.31621894	0.56907088

cg15812759	RNF14;RNF14;RNF14;RNF	black	-0.1173606	-0.2338094	0.22424554	0.17245895	0.15642437	0.45345452
cg03672602	GMIP;GMIP	black	-0.1110123	0.03872732	-0.4697875	-0.1467856	-0.0715144	-0.6236863
cg24736201	HRH1;HRH1	black	-0.0688892	-0.0793745	0.27844352	0.16969381	0.09554026	0.47425374
cg14294096	MIR670;HSD17B12	black	-0.1179776	-0.0606558	0.26036516	0.22258841	0.09188176	0.37661835
cg22060492	TINF2;TINF2;TINF2;TINF2;	black	-0.1425317	0.02733803	-0.2213276	-0.1488203	-0.0507011	-0.4349012
cg05305434	LSP1	black	0.03412675	0.07130584	-0.217747	-0.1516043	-0.0492676	-0.452498
cg25115812	HEATR2;HEATR2	black	-0.0911151	-0.0437123	0.13454897	0.03575893	-0.0202337	0.56278558
cg07567260	GPR108;GPR108;GPR108;G	black	-0.0221329	-0.0576328	0.09895654	0.14065078	-0.0214507	0.52955046
cg18607308	CES2;FAM96B;CES2;CES2;	black	-0.1285199	-0.367611	0.29864329	0.18321895	0.31002467	0.56462398
cg01543582	AF146191.4;FRG1;FRG1;FR	black	0.16898403	0.21849307	0.0168344	0.0352012	0.01367354	-0.4675402
cg19662870	GALNT1	black	0.11257832	-0.2684686	0.21221211	0.31519747	0.47872692	0.69366293
cg26530630	GPR161	black	0.19959272	0.0427939	0.48481208	0.28567605	0.0746942	0.6020595
cg19517509	RP11-	black	-0.1794973	-0.0099255	-0.4204209	-0.1966172	-0.0672165	-0.6217434
cg15235185	TROVE2;TROVE2;UCHL5;	black	-0.175431	-0.0491839	-0.3731071	-0.17985	-0.0168087	-0.4231889
cg16204618	ZNF320	black	0.16063226	-0.0784301	0.40129156	0.41459786	0.30049549	0.76399279
cg26001336	RAB3A;RAB3A;RAB3A;AC	black	-0.3125	-0.08414	-0.1552325	-0.2886246	-0.1337481	-0.5799855
cg12666107	SDC3	black	-0.1182105	-0.1674855	0.27205399	0.01228526	0.09001026	0.4448855
cg26649469	RP11-508M1.5;RP11-	black	-0.1425404	-0.1176627	0.0180883	-0.0152957	0.00156188	0.38626163
cg17790400	ZCCHC4;ZCCHC4;ZCCHC4	black	-0.1693869	-0.0849936	-0.2572138	-0.1517342	0.00520738	-0.3346213
cg12784848	RP11-1149O23.3;RP11-	black	-0.1427777	-0.0082065	-0.1769426	-0.1609526	-0.0613867	-0.4700354
cg27506609	RP4-665N4.4;TEKT2;TEKT2	black	0.12950498	0.09788368	-0.0672221	0.11275738	0.08034162	-0.4610231
cg24993501	RABGAP1;RABGAP1;RAB	black	0.2884998	-0.2442857	0.36819474	0.49228246	0.55487147	0.7465997
cg12821546	L3MBTL2;L3MBTL2;L3MB	black	-0.2205476	-0.0166921	-0.367433	-0.1303703	-0.0559568	-0.5909109
cg05725489	AC147651.5	black	0.18056919	0.01202972	0.42691865	0.2745011	0.14455157	0.65381122
cg20237447	DEM1;DEM1;DEM1;DEM1;	black	-0.1371217	-0.0418143	-0.2742399	-0.1750218	-0.0468332	-0.5184636
cg03859306	SET;SET;SET;SET;SET;SET;	black	-0.2432432	-0.0352694	-0.335828	-0.2420568	-0.0228217	-0.5720614
cg19699211	SDK2	black	-0.0443885	-0.0564964	-0.3657763	-0.1190634	0.02444879	-0.5086963
cg06586915	GS1-124K5.4;GS1-	black	-0.0613994	0.09449899	-0.2654266	-0.1555544	-0.0818939	-0.4219378
cg13830730	MLL4	black	-0.0813405	0.00518483	-0.2389319	-0.1969175	-0.0847088	-0.3535176
cg24283921	AMPD3;AMPD3;AMPD3;A	black	-0.1658611	-0.2578795	0.19497053	0.02550042	0.06619989	0.57263422
cg14460735	CNST;CNST;CNST;CNST	black	0.04935938	0.21184731	-0.0428903	0.05553073	-0.143863	-0.3809048
cg03102494	Y_RNA;RP11-495P10.3	black	0.04196719	-0.2145986	0.20003851	0.30931757	0.28944102	0.4572019
cg27438307	AFTPH;RP11-	black	-0.2032235	-0.0201996	-0.3097453	-0.262802	-0.0547085	-0.5588665
cg13412395	NR4A3;NR4A3	black	-0.0648684	0.07294737	-0.3776365	-0.1395421	-0.0778547	-0.4824452
cg18133957	APC2;APC2	black	0.17493581	0.01099487	0.25697898	0.22956844	0.09674408	0.46725858

cg08665853	RNF14;RNF14;RNF14;RNF	black	-0.0673068	-0.1950143	0.10788972	0.23632142	0.18042842	0.35887401
cg23446514	RPL4;RPL4;RPL4;RPL4;RPL	black	-0.1778204	0.1380527	-0.3060147	-0.1471393	-0.17196	-0.8481643
cg25184507	CYB5D1;CYB5D1;CYB5D1	black	-0.0855382	-0.07141	-0.1352752	-0.0941758	0.09318283	-0.520241
cg15829969	Y_RNA;RP11-495P10.3	black	-0.027141	-0.1474551	-0.0064657	0.20301015	0.24279263	0.03969597
cg03940662	UBE2I;LA16c-	black	0.14104761	-0.1315659	0.47703393	0.33226863	0.36479022	0.5639095
cg01947862	ST3GAL5;ST3GAL5;ST3GA	black	-0.1035016	0.01210575	-0.1754156	-0.1591611	-0.0520069	-0.4594995
cg21815882	HNRNPA2B1;HNRNPA2B1	black	-0.2815543	-0.0654535	-0.2273544	-0.308573	-0.1358605	-0.5023449
cg18030105	ZNF195;ZNF195;ZNF195;Z	black	0.20758121	-0.0375569	0.52415322	0.25131642	0.13589087	0.85413418
cg06391107	ZNF546	black	-0.2514811	-0.149028	-0.4195009	-0.2337155	0.05146729	-0.4880837
cg25618682	RP11-108M9.4;RP11-	black	0.32325722	-0.0366548	0.40369703	0.41834473	0.26053869	0.73936713
cg07390791	AC092619.1	black	0.23161818	-0.0559133	0.34433397	0.26627627	0.2501591	0.72811638
cg06184463	TAMM41;TAMM41;TAMM	black	-0.1857601	-0.0403996	-0.1336128	-0.1601813	-0.0177033	-0.3656147
cg07454192	TMEM70;TMEM70;TMEM7	black	-0.1766792	-0.0828643	-0.1678166	-0.1245889	0.00854425	-0.4344389
cg04949219	C11orf2;C11orf2;C11orf2;C1	black	-0.2057895	0.04038013	-0.445312	-0.277473	-0.1658509	-0.5697747
cg07039469	ZNF219;ZNF219;ZNF219;Z	black	-0.3117948	-0.1355498	-0.2024785	-0.2522689	-0.0461182	-0.5640348
cg03875157	SLC4A1;SLC4A1	black	0.27192872	-0.1855082	0.18520202	0.46026906	0.48024825	0.70149454
cg10713137	ARFGAP1;ARFGAP1;ARF	black	0.3498847	0.05040399	0.52922022	0.49263358	0.15082859	0.74008964
cg13802355	SLC11A1;SLC11A1;SLC11	black	0.08161462	0.20597336	-0.102743	0.01137026	-0.0725574	-0.6083387
cg07080049	FAM120AOS;FAM120AOS;	black	-0.1732726	-0.0246752	-0.2355236	-0.2312429	-0.1005789	-0.4502307
cg17301506	EIF2S2	black	-0.2064287	0.01663998	-0.394537	-0.1980246	-0.0691057	-0.4188621
cg13341668	HYAL2;HYAL2;HYAL2;H	black	0.39379345	-0.1229297	0.1759602	0.44161173	0.5151324	0.71573003
cg09075678	NCOA7;NCOA7	black	-0.1728111	-0.202474	0.22623122	0.05004671	0.10220142	0.37975175
cg04651240	RP11-113D6.10;RP11-	black	0.13372538	0.09803455	0.35319331	0.31370668	0.11975638	0.48257116
cg03653573	AC116366.5;AC116366.5;C5	black	-0.1815783	0.02383951	-0.3721816	-0.1584297	-0.0089047	-0.6730248
cg00981170	ZFP90;ZFP90;ZFP90	black	0.12746986	0.02559616	0.34805561	0.33206055	0.11769958	0.58398595
cg01423368	RP11-	black	-0.1541053	0.03206367	-0.3538122	-0.1714841	-0.0262937	-0.5847704
cg25532061	RPS7;RPS7;RPS7;RPS7;RPS	black	-0.1218011	-0.030889	0.06979593	-0.1201374	-0.0668634	-0.5447018
cg18201630	ATP2A3;ATP2A3;ATP2A3	black	0.41347594	-0.0648495	0.10365442	0.47455021	0.47313081	0.74801426
cg23744212	DNAJC28;DNAJC28;DNAJ	black	-0.3079746	-0.0972739	-0.3701318	-0.2517303	0.01297504	-0.6172855
cg18952654	INO80;INO80;INO80	black	-0.2830061	-0.1607575	-0.3815509	-0.2114349	-0.0171286	-0.4651384
cg12624087	C22orf24;YWHAH;YWHAH	black	-0.2247834	-0.079202	-0.0528528	-0.1412864	0.01801498	-0.4049932
cg12024887	GPR108;GPR108;GPR108;G	black	-0.053498	-0.0720915	0.0275084	0.06618132	0.00151641	0.439502
cg06791024	IGDCC3;IGDCC3	black	0.16690726	0.2212515	0.06901511	-0.0438071	-0.0614321	-0.5039768
cg16875032	SNTB1;SNTB1	black	-0.0502621	0.0162221	0.09759918	-0.0255357	-0.0348924	-0.223736
cg06250128	CCDC151;CCDC151;PRKC	black	-0.1917795	-0.0053763	-0.2667132	-0.1772818	-0.0738042	-0.5201392

cg15508020	CSNK2A2;CSNK2A2;RP11-	black	-0.1637329	-0.0533742	-0.1370441	-0.1081978	-0.0048833	-0.4859516
cg24626744	B9D1;MAPK7;MAPK7;MA	black	-0.1307883	0.04657391	-0.3263123	-0.1758625	-0.0892452	-0.5948933
cg10243681	NPTN;NPTN;NPTN;NPTN;	black	-0.1490477	-0.0292883	-0.1031316	-0.1659328	-0.0592193	-0.3840436
cg27442505	SLC2A14;SLC2A14;SLC2A	black	0.18111541	-0.0303989	0.33127921	0.15825255	0.11848239	0.72544368
cg12667999	KAT7;KAT7;KAT7;KAT7;K	black	0.16970747	-0.048715	0.50692782	0.19065009	0.10045678	0.74593148
cg14868726	METTL4;METTL4;METTL4;	black	-0.2801094	-0.04645	-0.4700793	-0.2142043	-0.0604138	-0.5863378
cg05657581	PKN2;PKN2;PKN2;RP11-	black	0.02359894	-0.069613	-0.1555806	-0.1093898	0.04692441	0.2911576
cg22862407	TCERG1;TCERG1	black	0.12194527	-0.0195862	0.20813646	0.06403137	0.10931544	0.48626701
cg18549547	SULT2B1	black	0.16105002	-0.1369768	0.53230481	0.25343511	0.24063962	0.80518699
cg13827970	GCH1;GCH1;GCH1;GCH1;	black	-0.192709	0.01966984	-0.2590872	-0.2450246	-0.1891	-0.5117656
cg25405172	RN5S109	black	0.16674101	-0.0760854	0.12004649	0.17749999	0.19513439	0.82893752
cg08989903	Y_RNA	black	0.27214737	0.02758537	0.40731749	0.17254973	0.09690159	0.76415691
cg13465831	BRD2;BRD2;BRD2;BRD2;B	black	-0.4114706	-0.1298072	-0.3053616	-0.2422798	-0.1127206	-0.6817065
cg10478799	U6	black	-0.0047882	0.17149279	0.30055492	0.11425918	-0.1794504	0.65778809
cg24114491	MSL2;MSL2;MSL2;MSL2	black	-0.160986	0.07095641	-0.4108496	-0.223067	-0.1035149	-0.520313
cg13861802	OR4N2;OR4N2	black	0.28727206	0.1591057	0.32721847	0.26628638	0.0204624	0.57579313
cg07131210	CYP2W1;CYP2W1;CYP2W	black	0.29400376	0.16464105	0.23295366	0.26679996	0.12854673	0.50828424
cg03462348	SNORD115-29;SNHG14	black	0.23740267	0.08020604	0.33123016	0.26037094	0.15929772	0.45765514
cg20630812	CNBD1;CNBD1	black	0.05074971	-0.1026269	0.38238389	0.14295175	0.09609255	0.81127522
cg06631310	AC025165.8;AC025165.8;SL	black	-0.1799837	-0.1392989	0.08426739	0.00957043	0.13820759	-0.3491947
cg22540282	HCG14	black	-0.2426084	-0.1944408	0.23660438	0.05532307	-0.0010323	0.28854504
cg00369126	MGRN1;MGRN1	black	-0.3711323	-0.1580276	-0.1646731	-0.2585987	-0.1002143	-0.5576774
cg04874031	CALD1;CALD1;CALD1;CA	black	0.37900518	0.06090301	0.54808245	0.32531998	0.26974045	0.60543915
cg25580656	ZFYVE21;ZFYVE21;ZFYV	black	0.34864005	-0.0958202	0.16977752	0.63140289	0.46457034	0.63091123
cg25970230	POU4F2	black	-0.346076	-0.1346762	-0.2355602	-0.3042893	-0.0380397	-0.7744184
cg09428623	PPP3R1;PPP3R1;PPP3R1	black	-0.2679645	-0.1055649	-0.297528	-0.1424484	-0.0543558	-0.4346598
cg21613905	TBC1D19;TBC1D19;TBC1D	black	-0.2674136	-0.0705847	-0.074186	-0.2230203	-0.0852661	-0.4670627
cg08163160	C12orf57;C12orf57;RNU7-	black	0.19881779	0.03536476	0.41437599	0.20009548	0.01279723	0.72071224
cg16956876	VARS;Y_RNA	black	0.29772184	0.04148845	0.22202098	0.29436054	0.12907112	0.5287992
cg20973743	GALK2;COPS2;COPS2;CO	black	-0.399161	-0.1016429	-0.2828757	-0.2335598	-0.117081	-0.7252311
cg00147976	PCBP2;PCBP2;PCBP2;PCB	black	-0.4557255	-0.1977351	-0.4195053	-0.3088906	-0.0584123	-0.8374782
cg11849703	FGFR4;FGFR4	black	0.0915123	0.20355419	0.16357157	0.38357017	0.0199487	0.44138377
cg09287359	LGALS7B	black	0.35012556	0.06237644	0.51404437	0.33589689	0.23562462	0.71054623
cg04869838	Y_RNA;RP11-	black	-0.100919	-0.0444684	0.04097692	0.11940866	0.02763146	-0.0876104
cg09932345	NADK;NADK;NADK;NAD	black	-0.2975363	-0.0538829	-0.404198	-0.2223094	-0.0612212	-0.5960265

cg17833341	CKS1B;CKS1B;SHC1;SHC1	black	-0.2360403	-0.01981	-0.4421778	-0.2305932	-0.0435135	-0.6421101
cg11347316	HPS5;GTF2H1;GTF2H1;GT	black	-0.32111	-0.112938	-0.3035007	-0.174718	-0.0088616	-0.8295209
cg02459090	RP11-80F22.2;RP11-80F22.2	black	0.23316704	-0.0328877	0.28258859	0.22796682	0.15946919	0.57264538
cg19776032	PSMB9;PSMB9;PSMB9;TA	black	-0.29376	-0.0455948	-0.4394627	-0.2110963	-0.0918843	-0.6576269
cg04830773	snoU13;NCALD;NCALD;N	black	0.21540117	-0.0309241	0.15348402	0.18255351	0.19936845	0.60807575
cg14546714	SUMO2	black	-0.36552	-0.1580391	-0.1769651	-0.1570123	-0.0377415	-0.6707859
cg07007092	SIGLEC9	black	-0.1297423	0.01734379	0.41392697	0.04001315	-0.2045741	0.55685757
cg17509612	C9orf78;C9orf78;C9orf78;C9	black	-0.3525567	-0.1397524	-0.2131263	-0.2690327	-0.1131572	-0.5107298
cg13108255	NCF2;NCF2;NCF2;SMG7	black	0.17837	0.04205733	0.30296564	0.25391287	0.16182321	0.61680597
cg10554702	EPHA5;EPHA5;EPHA5;RP1	black	0.04820051	-0.112531	0.17219376	0.07539578	0.08514581	0.66475286
cg00866151	CTB-134H23.3;CTB-	black	0.16796111	-0.0276656	0.39520831	0.22020241	0.19089599	0.75829352
cg04674497	SUPT3H;SUPT3H;SUPT3H	black	-0.3737073	-0.1174071	-0.3432676	-0.2862674	-0.0747608	-0.6155856
cg24334803	ZNF672;ZNF672;ZNF672;Z	black	-0.5063291	-0.1641373	-0.4279373	-0.3421999	-0.151035	-0.7139961
cg10578556	ATG9A;AC068946.1;ATG9	black	0.22832184	0.22796539	0.12072286	0.18788119	0.05609347	0.4252947
cg12886731	HK1;HK1;HK1;HK1;HK1;H	black	0.37317808	-0.0113141	0.18668792	0.50214156	0.35426585	0.50266721
cg14459319	RP11-364L4.3	black	0.35459582	-0.0735948	0.60482789	0.4380799	0.43201039	0.68834483
cg23075366	ANKRD33;ANKRD33;ANK	black	0.29577893	-0.0573261	0.35259556	0.32264285	0.35015941	0.48433629
cg09736426	TIAM1;TIAM1;AP000251.2	black	0.25446099	-0.0017906	0.29664502	0.26040504	0.18739179	0.58691601
cg03602746	AC007319.1;AC007319.1	black	0.12673024	-0.0637885	0.10618341	0.21245986	0.19800205	0.59030631
cg15645685	PBX4;PBX4;PBX4;PBX4;P	black	0.33765225	-0.0234409	0.13947146	0.30724161	0.30441061	0.59552337
cg16840884	PPP1CA;PPP1CA;CARNS1;	black	0.44437918	0.10492759	0.38875592	0.37686692	0.28091067	0.64965303
cg27655494	RGS7	black	0.13111813	0.02947675	0.22952594	0.14992142	0.05051811	0.85030587
cg03499659	RP11-275A6.1	black	-0.0530115	0.33563033	0.23640725	0.07102699	-0.3457337	0.4370342
cg00670641	SUMO1;SUMO1;SUMO1;S	black	-0.4619526	-0.1655002	-0.2642537	-0.2999429	-0.1299711	-0.6532267
cg25710809	GNRH1;GNRH1	black	0.2770038	0.12632216	0.27034925	0.27672016	0.06842284	0.46565364
cg21257831	ADCY6;ADCY6;ADCY6;A	black	0.23074623	0.10473236	0.16020299	0.46878307	0.26308824	0.58383028
cg17091770	STYX;STYX;STYX;STYX	black	0.05714458	-0.1035318	0.19064265	0.112945	0.11747516	0.52885477
cg05064106	MOV10L1;MOV10L1;MOV	black	0.20718404	-0.1218792	0.08212112	0.12800734	0.206248	0.45176211
cg02796621	MIR1180	black	0.20075129	0.24208364	0.29838361	0.18347158	-0.0974116	0.54911249
cg18885895	ASGR1;ASGR1;ASGR1;AS	black	-0.2871598	-0.0762156	-0.1393921	-0.2701513	-0.0665417	-0.5483936
cg18709313	CTD-2506J14.1;CTD-	black	0.23487505	0.02494987	-0.0296825	0.19670231	0.19204586	0.38100708
cg17717357	WLS;WLS	black	0.20824398	-0.0101445	0.41804266	0.14280527	0.11923618	0.81962574
cg12865939	ST5;ST5;ST5;ST5;ST5;ST5;S	black	0.40421815	0.15787166	0.04723484	0.23645239	0.2259068	0.57601926
cg16659470	RBM12B;RBM12B;RP11-	black	0.36629223	0.02181073	0.73272364	0.37632019	0.17396653	0.79157455
cg19869610	PPM1N;RTN2	black	-0.2916408	-0.1468628	-0.1924256	-0.220051	0.01748195	-0.7237313

cg07155478	BDH1;BDH1;BDH1;BDH1;	black	-0.3585637	-0.3397768	-0.2685266	-0.191144	0.15945696	-0.6170018
cg25241573	CTC-459F4.2	black	0.20683563	0.09955246	0.01978119	0.04765248	-0.0281356	0.5376012
cg10947003	KIAA0895L;KIAA0895L;KI	black	-0.3050561	-0.0728436	-0.3148534	-0.2502076	-0.0796769	-0.658336
cg16690859	AC009365.3;PLXNA4	black	0.24617808	-0.0057374	0.61693868	0.21518782	0.1100064	0.83382701
cg09539943	RP5-	black	0.07553417	-0.0480646	0.07000819	0.02047263	0.05131246	0.39090914
cg23621779	NRM;NRM;NRM;NRM;NR	black	-0.4330467	-0.1736138	-0.1317017	-0.2428282	-0.0755593	-0.7047576
cg03951833	Y_RNA;RP11-495P10.3	black	0.24142073	-0.0564102	0.22156918	0.28877278	0.22224086	0.61048435
cg12996417	PRSS48	black	-0.1803928	0.15553467	-0.1796464	-0.1318562	-0.1787855	-0.7723116
cg04751031	RAI14;RAI14;RAI14;RAI14;	black	-0.2895148	-0.0878572	-0.0252144	-0.1840045	-0.0060418	-0.7199271
cg22055860	AC009133.12;AC009133.12;	black	-0.4055122	-0.151652	-0.2644415	-0.203267	-0.0507845	-0.8033055
cg15828235	PCP2	black	0.18952805	0.25712048	0.35362987	0.17907446	-0.1244186	0.65863962
cg17638730	E2F5;E2F5;E2F5;E2F5;RP11-	black	-0.3019637	-0.0590159	-0.2314952	-0.1805264	-0.0628028	-0.7743642
cg12031757	ARAP1-	black	0.54044065	0.05151808	0.31369035	0.52578733	0.4930874	0.62104441
cg12410343	AFG3L2;AFG3L2	black	-0.3018722	-0.0612177	-0.4219694	-0.2766819	-0.0995859	-0.5460314
cg08382009	EDC4;EDC4;EDC4;CTC-	black	0.40091957	0.09635466	0.23129906	0.41321941	0.21148577	0.8355225
cg23526147	NFIX;NFIX;NFIX	black	-0.0925005	-0.11362	-0.2973543	-0.0993972	0.18714166	-0.3916152
cg04180781	MYO18A;MYO18A;MYO1	black	0.21969171	0.0424661	0.22612062	0.19522932	0.10851708	0.43958578
cg05646213	RP11-58A18.1	black	0.3720136	0.16730092	0.35180407	0.31807267	0.11834817	0.70262077
cg05881776	SH3BP4;SH3BP4;SH3BP4;S	black	-0.2934336	-0.095471	-0.0722144	-0.2003576	-0.0324889	-0.7735149
cg04421483	CHCHD3;CHCHD3;CHCH	black	0.35883168	0.06425708	0.27653594	0.29682852	0.16391353	0.81790186
cg02397640	RP11-217E22.5;RP11-	black	0.29170761	0.09107244	0.29837984	0.21397932	0.10320245	0.87078205
cg07744432	ZNF826P	black	0.32203015	0.09017526	0.28069313	0.1311862	0.04071166	0.54024638
cg11592082	MAP3K6;MAP3K6;MAP3K	black	0.29214776	0.17198046	0.34963163	0.24263385	0.08733854	0.45514852
cg13922978	NXN;NXN;NXN;NXN;NX	black	0.38801173	0.20268389	0.18338674	0.28735841	0.03855322	0.64536914
cg21261121	RP4-781B1.2	black	0.21310404	-0.0098703	0.38240627	0.18934827	0.08269219	0.89064797
cg09960003	CRTAC1	black	0.21709078	-0.0372791	0.50619902	0.23504855	0.21421996	0.64157363
cg00306426	RP4-	black	-0.3324765	0.00429518	-0.1739889	-0.1632559	-0.1286329	-0.8152844
cg25417551	TADA2A;ACACA;ACACA;	black	-0.4073027	-0.1824091	-0.1744059	-0.1840887	-0.0226916	-0.7139639
cg13977158	MDP1;MDP1;AL136419.6;A	black	0.06026761	-0.0246505	0.01088951	0.02451416	-0.01119	0.5875231
cg09605828	TRNAU1AP;TRNAU1AP;T	black	0.48817038	0.07856785	0.17174676	0.41343029	0.32579079	0.70402669
cg20824717	IFT81;IFT81;IFT81	black	0.29117095	-0.0615152	0.47560876	0.19240664	0.16204435	0.83029828
cg21215811	VPS25	black	-0.4532153	-0.1718369	-0.1541928	-0.2690339	-0.1283258	-0.7520963
cg10180165	TUBG2	black	0.46690027	0.12831528	0.1922852	0.57347045	0.23002907	0.68894617
cg00588262	LYPD4;DMRTC2;LYPD4	black	0.35838128	0.07609407	0.16976134	0.14546612	0.16910003	0.55140102
cg27210919	SDK2;SDK2	black	0.26590534	-0.0545553	0.21797234	0.17510171	0.1856965	0.6817975

cg16213842	THADA;THADA;THADA	black	0.43709342	0.11385083	0.34062665	0.54598021	0.32172757	0.5758376
cg21316470	TPM1;TPM1;TPM1;TPM1;T	black	-0.4634876	-0.1318688	-0.241468	-0.224203	-0.1517084	-0.6448788
cg03837292	ADRBK1;ADRBK1;ADRB	black	0.56462383	-0.0098701	0.18810902	0.50744343	0.56039522	0.61927777
cg12916575	MINK1;MINK1;MINK1;MI	black	0.27783517	0.23058875	0.30426213	0.28042628	0.04770009	0.42485873
cg17036469	AMPD2;AMPD2;AMPD2;A	black	0.2912426	-0.1248912	0.12038839	0.30651904	0.35514983	0.59176914
cg16283010	RG9MTD1;RG9MTD1	black	-0.2506236	-0.1789009	-0.0369221	-0.1215926	0.12195882	-0.4319867
cg14785368	FBXO7;FBXO7;FBXO7;FB	black	0.35504143	0.03900885	0.3316793	0.57607128	0.15015043	0.72316508
cg05109774	C17orf62;C17orf62;C17orf62	black	0.43797246	0.09035906	0.36880034	0.41423743	0.23103565	0.72587312
cg12615843	MIIP;MIIP;MIIP;MIIP;MIIP	black	0.36953989	0.07553869	0.32267399	0.27711436	0.21272481	0.71992788
cg09230763	MAP3K6;MAP3K6;MAP3K	black	0.31145728	0.25671235	0.18776314	0.26951539	0.07546039	0.31964248
cg02765496	FAM122A;FAM122A;PIP5	black	0.28963611	-0.0299472	0.18781159	0.40426904	0.18558259	0.81134135
cg12240404	RUNX1T1;RUNX1T1;RUN	black	-0.0730439	0.1408186	0.45274362	0.12723368	-0.2052164	0.50186283
cg02593905	ASPDH	black	0.30517911	0.06451786	0.47282214	0.25076658	0.08093055	0.84127094
cg15203271	SPON2;SPON2;SPON2;SPO	black	0.40416843	0.08720231	0.14108505	0.1853854	0.16890018	0.64641368
cg00033773	WDR83;WDR83;WDR83;W	black	0.27173057	0.20500292	0.35007549	0.19810564	-0.0375882	0.45892241
cg00202496	CLEC16A;CLEC16A	black	0.41245519	0.11383671	0.15109706	0.28432617	0.17349191	0.76398341
cg08379212	CCNJ;RP11-	black	-0.4362406	-0.2106372	-0.3169247	-0.2214369	0.00785928	-0.7677171
cg15824284	RP11-	black	0.35052294	0.04808323	0.13766861	0.10806628	0.17477367	0.50095711
cg05054006	NSRP1;NSRP1;NSRP1;NSR	black	-0.1755021	-0.0587902	-0.0414719	-0.0917815	0.01475196	-0.3815291
cg17151420	AC074117.10;GTF3C2;GTF3	black	-0.396712	-0.1667535	-0.2691823	-0.1954376	0.01355323	-0.6014677
cg14623216	ASB13;ASB13;ASB13	black	0.18747385	0.09838617	0.28354862	0.29171115	0.05632829	0.45491254
cg24228123	PRMT1;PRMT1;PRMT1;PR	black	-0.0384379	-0.0783454	0.08986084	-0.1515403	-0.0275597	0.31468788
cg17329304	PLEKHG5;PLEKHG5;PLEK	black	-0.4031298	-0.272625	-0.4348867	-0.3449464	0.0712476	-0.6129892
cg00225126	NXN;NXN;NXN;NXN;NX	black	0.23367184	0.11935002	0.23799669	0.22006898	0.01840281	0.47656266
cg20180562	RP11-53M11.3;RP11-	black	0.33039357	0.22818003	0.40794046	0.19485669	-0.1042746	0.85390242
cg15993762	PPIG;PPIG;PPIG;PPIG;PPI	black	0.10545551	-0.0168509	0.1005831	0.15858976	0.02335284	0.3984415
cg12268102	MIPEP	black	0.36107235	0.1128086	0.21373439	0.22085747	0.10900793	0.83271116
cg24192559	PPAP2C;PPAP2C	black	0.42392351	0.1042767	0.31547876	0.22647181	0.10238739	0.76677248
cg17175624	TXNIP;TXNIP;TXNIP;TXNI	black	-0.4708569	-0.2144445	-0.0096783	-0.2815792	-0.1094291	-0.5408582
cg22416769	PRDM15;PRDM15;PRDM1	black	0.34946826	0.23246242	0.37781918	0.32546988	-0.0101898	0.54660379
cg17052964	SLC5A7;SLC5A7;SLC5A7	black	0.30500198	-0.0256787	0.39550173	0.26009672	0.19245255	0.73487425
cg19450029	CYFIP2;CYFIP2;CYFIP2;C	black	0.47232911	0.24493661	0.22813248	0.30693851	0.07964106	0.59283262
cg07934732	AP001053.11	black	0.37589022	0.16971237	0.61312298	0.29965922	0.04678281	0.7536862
cg27288240	RP11-503N18.4;RNF4	black	0.43709536	0.01660298	0.48586128	0.36968387	0.24215549	0.75511021
cg04084600	AC006037.2	black	0.31567605	0.03348234	0.6377763	0.1912188	0.10534275	0.73369201

cg09225658	FGD3	black	0.16379442	-0.072154	0.1267724	0.14494428	0.17294395	0.41744029
cg00739661	ZNF791;ZNF791;ZNF791;Z	black	-0.3486117	-0.1188817	-0.3746405	-0.1484356	0.0124718	-0.6269473
cg20936152	USP5;USP5	black	0.46269082	0.20580815	0.27391401	0.33776059	0.11335632	0.61729574
cg05353868	KCNK7;KCNK7	black	0.4768049	0.18596243	0.17585415	0.33014663	0.14984497	0.69951528
cg22809920	MATN4;MATN4;MATN4;R	black	0.34125598	0.00351533	0.1332637	0.29275334	0.27241858	0.61468567
cg26038649	MAP3K6;MAP3K6;MAP3K	black	0.3749357	0.20869779	0.16815632	0.30962793	0.09999146	0.65463549
cg10368507	GHDC;GHDC;GHDC	black	0.40950931	0.04956612	0.46403382	0.34978475	0.21231571	0.69969701
cg10675453	PRICKLE4;PRICKLE4;TOM	black	-0.1340131	-0.1871213	-0.0355813	-0.2119367	0.03695757	0.27513147
cg06814977	CTD-2277K2.1;CTD-	black	0.28891974	-0.005648	0.41428781	0.22715393	0.13227684	0.86102076
cg14039343	RGL2;RGL2;RGL2;RGL2;R	black	-0.561245	-0.2517212	-0.2085141	-0.3185446	-0.0809056	-0.7465546
cg20489467	TTC21B;TTC21B;TTC21B	black	-0.3162024	-0.0097899	-0.3529796	-0.2337869	-0.100324	-0.8583027
cg21091985	CLIC1;CLIC1	black	-0.264374	-0.2125177	0.09053493	-0.1265036	0.18258137	-0.4119142
cg01044770	C3orf19;C3orf19;C3orf19	black	-0.4906897	-0.1471283	-0.3277778	-0.2921625	-0.0833735	-0.6939978
cg17828999	FOXN3;FOXN3;FOXN3;FO	black	0.39913958	0.1254379	0.2185586	0.18077978	0.05897532	0.83382268
cg10266418	CMTM3;CMTM3	black	0.24651141	0.00290856	0.42506958	0.22661882	0.07523567	0.64610308
cg08006046	ERVFRD-1;ERVFRD-1	black	0.30867125	0.09191056	0.29998726	0.14782734	0.07531013	0.86000453
cg17660010	MAN1B1;MAN1B1;MAN1	black	0.46650093	0.18319802	0.14574455	0.22378926	0.05096373	0.7229532
cg11229497	TRIM31;TRIM31;TRIM31;T	black	0.36145207	0.11992741	0.41562367	0.17342792	0.11307731	0.51496436
cg03776627	RP11-333E1.2;RP11-333E1.1	black	0.2333546	0.24600225	0.16683611	0.04072532	-0.2947389	0.61256741
cg05639188	GTF2I;AC083884.8;AC0838	black	0.30255478	0.15016247	0.23717905	0.05781174	-0.108723	0.55447845
cg22985216	AC010967.2	black	0.31083819	0.04469833	0.61540745	0.26637651	0.11628715	0.80230249
cg13255190	GALNT8;RP11-	black	0.32460147	0.14259391	0.34343367	0.19617871	-0.01742	0.88353543
cg01941093	PKM2;PKM2;PKM2;PKM2;	black	0.45093777	0.13810515	0.35256268	0.2572877	0.10909696	0.77066462
cg12575136	ZNF397;ZNF397	black	-0.3199435	-0.1697628	-0.1544805	-0.128068	0.03222544	-0.7929779
cg26535023	RP11-544I20.2;RP11-	black	-0.203499	-0.0424005	-0.3205826	-0.0746027	0.1642743	-0.5392472
cg24518943	FAM135A;FAM135A;FAM	black	-0.2810173	-0.0152154	-0.4636344	-0.2252337	-0.1015986	-0.7302938
cg14494313	NAT2;NAT2	black	0.34093129	0.16577202	0.34316	0.1716538	0.04135226	0.80458482
cg09305629	OR4Q3;OR4N2	black	0.15535599	0.17797754	0.2434555	0.23879807	-0.0605827	0.41610689
cg10157951	MORN3;MORN3;MORN3	black	0.38004882	-0.050941	0.46724682	0.28593574	0.28582254	0.82441357
cg21114840	WRB;WRB;WRB;WRB;WR	black	-0.4090207	-0.0461146	-0.2869397	-0.2315749	-0.0936373	-0.7847338
cg17897852	APOBEC3C;APOBEC3C	black	0.34617883	0.0998662	0.34781812	0.27303617	0.06095335	0.85124695
cg19483962	MDC1;MDC1	black	0.49744394	0.13698125	0.33265506	0.37377291	0.10077139	0.74791686
cg07836431	WDFY3-AS2;WDFY3-	black	-0.4164614	-0.4147433	-0.1112041	-0.2469317	0.1273161	-0.6838095
cg07674649	KCNE1;KCNE1;KCNE1;KC	black	0.1752198	0.02396626	0.16390679	0.22856957	0.14625905	0.46853649
cg14770570	ACOX1;TEN1;TEN1;ACOX	black	-0.5067485	-0.362325	0.02004939	-0.1414341	0.09514683	-0.5895133

cg20706599	C15orf58;C15orf58;C15orf58	black	-0.3543908	-0.0843042	-0.1372205	-0.2090291	-0.0673153	-0.8582866
cg00942918	HIST1H3A;HIST1H4A	black	0.15869407	-0.0113589	0.24711422	0.09589597	-0.047481	0.50167388
cg06510438	PIEZO2	black	-0.321779	-0.4323094	-0.1155053	-0.1826421	0.21417964	-0.7076062
cg06034096	FPGT;FPGT;FPGT;FPGT;FP	black	-0.392718	-0.2103946	-0.0418253	-0.323272	-0.0587474	-0.4787227
cg11705208	MAP3K6;MAP3K6;MAP3K	black	0.53634942	0.36784184	0.29250987	0.33781652	0.01118295	0.58321944
cg21193729	MAP3K11;MAP3K11;MAP	black	0.43543218	0.16211079	0.1960002	0.21982814	0.04583811	0.62102142
cg07281370	MIR125B1;RP11-	black	0.4777832	0.12929427	0.28873693	0.30986604	0.08296068	0.68494771
cg21969990	KIAA1755	black	0.40481479	0.0226149	0.38078621	0.26523058	0.29009724	0.70185634
cg00060374	RP4-758J18.7;ANKRD65	black	0.21150413	-0.0582857	0.23741177	0.20300167	0.2267127	0.43192146
cg19707735	IFI16	black	0.41127975	0.06733322	0.28988741	0.23337609	0.14729688	0.87245242
cg13149902	SIDT1;SIDT1	black	0.41676548	0.07655335	0.53454531	0.27035547	0.12104247	0.79863257
cg07936430	ZNF768;ZNF768;ZNF768;Z	black	-0.4765788	-0.1475527	-0.2165821	-0.2374362	-0.0791095	-0.8985188
cg17687970	KIAA1967;KIAA1967;KIA	black	-0.4115601	-0.2181424	-0.1317013	-0.1785533	0.07627231	-0.7775677
cg13649383	MATR3;MATR3;MATR3;M	black	0.25265933	0.02826763	0.50706877	0.27974253	0.10156861	0.56269364
cg08808367	RASAL2;RASAL2;RP11-	black	-0.2311101	-0.1578004	-0.0181539	-0.0880963	0.10807495	-0.638245
cg23677243	MEIS2;MEIS2;MEIS2;MEIS	black	-0.3955412	-0.3117327	-0.2387425	-0.2531218	0.11361122	-0.7819417
cg21872822	IGF2BP1;IGF2BP1;IGF2BP	black	-0.4589561	-0.2002322	-0.2474771	-0.2663629	-0.0415762	-0.6844935
cg05239811	SAPCD1-	black	0.42621279	0.09044648	0.27645371	0.24703605	0.0814196	0.66703364
cg12530481	ILF2;ILF2;ILF2	black	-0.3505196	-0.0983852	-0.3658565	-0.1758371	-0.0472412	-0.6344422
cg11367048	HMBS;HMBS;HMBS;HMB	black	0.35419424	0.13902348	0.26861824	0.36051235	0.1178889	0.51943382
cg10046893	SNORD114-30	black	0.43965525	0.13552217	0.63129652	0.31763892	0.07412621	0.76512225
cg26308814	RP11-108M9.1;RP11-	black	0.2674355	0.08955007	0.24213897	0.08444623	0.02164032	0.49814018
cg27062116	SNORD115-41;SNHG14	black	0.33274202	0.03915575	0.33224009	0.24191333	0.1237192	0.79699008
cg22832407	RP11-	black	-0.1962825	0.0184946	-0.2946567	-0.1518094	-0.0273469	-0.4112902
cg11604912	RAD9A;RAD9A;RAD9A;R	black	0.5136591	0.1282689	0.24827368	0.37475216	0.1839161	0.88136349
cg16658737	ZNF529;AC092295.7	black	-0.5437365	-0.1898371	-0.1694983	-0.2475074	-0.0783808	-0.7219985
cg18693345	C5orf38;IRX2;C5orf38;C5orf	black	-0.3865546	-0.1251189	-0.4163398	-0.2482254	-0.0510087	-0.614995
cg19551704	NXN;NXN;NXN;NXN;NX	black	0.35476674	0.31865145	0.08275619	0.13387245	-0.1581153	0.50076609
cg08086264	DNAJB5;DNAJB5;DNAJB5	black	0.30110687	0.14804424	0.39587341	0.17170666	-0.0863165	0.8381168
cg03448964	SLC30A6;SLC30A6;U6	black	0.39841314	0.12383382	0.32711555	0.24795644	0.10883801	0.82928608
cg14577211	RP11-	black	-0.4820879	-0.1289444	-0.2687424	-0.2840015	-0.1032075	-0.8159306
cg07925064	AC133919.6;AC133919.6;A	black	-0.1173748	0.05139159	-0.1188442	-0.0243302	-0.0406102	-0.3886876
cg07855250	RP11-93I21.3	black	0.46233351	0.15268535	0.5976372	0.27874312	0.01637309	0.77251198
cg06440474	TTLL10;TTLL10	black	0.3846349	0.15040097	0.24140712	0.24300736	0.00399064	0.74201321
cg00969680	SLC16A1	black	0.32966219	-0.0321578	0.35958061	0.21967174	0.22848084	0.77843236

cg08456130	C6orf211;C6orf211;C6orf211	black	-0.4277252	-0.1582233	-0.2563306	-0.2777229	-0.0341324	-0.8095542
cg27308895	SATB2;SATB2;SATB2;SAT	black	-0.4484776	-0.1523354	-0.5806679	-0.2649489	-0.0002681	-0.8309206
cg08187779	ZNF706;ZNF706;ZNF706;Z	black	-0.388407	-0.1759647	-0.1484481	-0.1319583	0.04319723	-0.7959276
cg13120258	NXN;NXN;NXN;NXN;NX	black	0.39641421	0.35518714	0.05152198	0.19107407	-0.1268481	0.5513089
cg18024258	REPIN1;REPIN1;RP4-	black	0.2971456	0.07305337	0.07009725	0.31157051	0.10091361	0.45949369
cg04587220	HMHA1;HMHA1	black	0.42377539	0.14777949	0.11000928	0.284223	0.08559653	0.83122571
cg06538998	CLN3;CLN3;CLN3;CLN3;C	black	-0.4732376	-0.1946163	-0.2822407	-0.2669647	-0.0210426	-0.894664
cg26663686	LY6H;LY6H;LY6H;LY6H	black	-0.3184857	-0.0707136	-0.1301108	-0.157085	-0.0327764	-0.7900682
cg23826287	MYBL1;MYBL1;MYBL1;M	black	-0.5269769	-0.2146897	-0.2403622	-0.2173716	-0.034227	-0.8282921
cg06524757	DACH1;DACH1	black	-0.4396238	-0.1567282	-0.4850548	-0.2869731	-0.0028382	-0.8432
cg26067108	ZFPM1;ZFPM1;ZFPM1;ZFP	black	-0.489642	-0.1750514	-0.154319	-0.2535592	-0.0236684	-0.8045366
cg24109894	PSMB2	black	0.28548158	0.02058667	0.25228815	0.3632325	0.19887966	0.40672865
cg08498597	RAD9A;RAD9A;RAD9A;R	black	0.5871525	0.19953847	0.28013177	0.46443505	0.19903567	0.86685254
cg24145357	FAM133B;FAM133B;FAM1	black	-0.4665065	-0.1368181	-0.2741504	-0.2576944	-0.0543608	-0.9287894
cg25674709	C12orf75;C12orf75;C12orf75	black	-0.4347454	-0.1704266	0.01842655	-0.1138028	-0.0267018	-0.6184789
cg18334849	MMRN1;MMRN1;MMRN1	black	-0.4070453	-0.2839249	-0.5045351	-0.2455259	0.14330575	-0.6990924
cg19304107	COL22A1;COL22A1	black	-0.5492077	-0.2069883	-0.330013	-0.2736839	-0.0401786	-0.8330677
cg24607409	RP11-379I19.1	black	0.447436	0.21011088	0.57869803	0.26024861	-0.0177077	0.72964541
cg20634738	ANO5	black	-0.6477744	-0.274871	-0.3404186	-0.3028674	-0.0522893	-0.8459871
cg07227728	AC018642.1;AC018642.1;A	black	-0.5161671	-0.1584905	-0.267545	-0.269263	-0.0997134	-0.9213055
cg10919889	NRXN1	black	0.47663909	0.43594055	0.27948363	0.27779187	-0.109356	0.6758486
cg07256113	RP11-175K6.1;RP11-	black	-0.5259407	-0.2133881	-0.3453831	-0.2410212	-0.0216012	-0.6963381
cg17860800	C1orf172;C1orf172	black	0.34095828	0.27383064	0.1986218	0.22912767	-0.0262336	0.46734368
cg05131957	CRLS1;CRLS1;CRLS1;CRL	black	-0.6451687	-0.3422925	-0.1700598	-0.3232676	-0.0411625	-0.7263869
cg22596557	TRIOBP;TRIOBP;TRIOBP;T	black	-0.6117146	-0.3301419	-0.194381	-0.2581854	0.01526249	-0.8319046
cg23087653	NLRP3;NLRP3;NLRP3;NLR	black	0.51688665	0.18835869	0.3293085	0.34007672	0.09741346	0.68481945
cg27629776	FAM83C	black	0.50551979	0.32384615	0.23523997	0.26659903	-0.0052947	0.6706115
cg24640065	TARBP1	black	0.48642848	0.14126749	0.14933433	0.2486576	0.08451791	0.73270035
cg09660145	KREMEN2;KREMEN2;PAQ	black	0.18348858	-0.0293489	-0.0744325	0.27305471	0.18946782	0.354852
cg04877802	NOP58;NOP58;NOP58;NOP	black	-0.5889308	-0.1877439	-0.2726666	-0.3204904	-0.0994649	-0.8869119
cg23300851	TAF1D;TAF1D;TAF1D;TAF	black	0.42372426	0.41885059	0.40038565	0.19152368	-0.2540269	0.79009608
cg05420668	RP11-22P6.3;RP11-	black	-0.6661468	-0.3653457	-0.1284377	-0.318978	0.05085369	-0.6803274
cg17241999	FNDC3B;FNDC3B;FNDC3	black	-0.6248691	-0.2372629	-0.1920819	-0.310697	-0.0639608	-0.8513676
cg06747254	SNORD115-31;SNHG14	black	0.5891757	0.1927061	0.29958397	0.25986463	0.17463469	0.6746578
cg08284713	TERF2;TERF2;TERF2;TERF	black	-0.6294301	-0.2792926	-0.3422002	-0.2873399	0.00398764	-0.8204045

cg21130913	VIT;VIT;VIT;VIT;VIT;VIT;	black	0.36448303	0.43018701	0.24645188	0.23204249	-0.2624774	0.7175999
cg11818821	FKBP6;FKBP6;TRIM50;FK	black	0.43276681	0.18799547	0.24328891	0.2704313	0.00826818	0.59925583
cg16578267	HES4	black	-0.5850911	-0.464673	-0.1668108	-0.2592141	0.15432636	-0.7474483
cg22538495	RP11-550H2.2	black	0.59847448	0.39138249	0.53851412	0.34176634	-0.1035658	0.66520262
cg13910484	CEP78;CEP78;CEP78;CEP78	black	-0.7289324	-0.3558649	-0.2302317	-0.3483287	-0.0167035	-0.7874561
cg18452449	TLR9	black	0.36627372	0.26115547	0.06711257	0.08051677	-0.2245607	0.57056125
cg27145165	GLRA3	blue	0.48885819	0.79103813	-0.1544677	0.02689522	-0.4880106	-0.0243018
cg07058706	SNORA67	blue	0.59662243	0.65510752	-0.0838027	0.03806336	-0.2838081	0.13759221
cg03124710	RP6-	blue	0.43398042	0.74421732	-0.2766242	-0.056788	-0.4650531	-0.1087049
cg02954577	RN5S89	blue	0.48468376	0.68814544	-0.2640255	0.06609596	-0.3305951	-0.0561774
cg05386577	BBS7;BBS7	blue	0.69913273	0.67478635	-0.0262658	-0.0166808	-0.3992645	0.24100621
cg14791530	CTBS;CTBS;CTBS;CTBS	blue	0.59278435	0.71836264	0.00781214	0.09619089	-0.409784	0.17983969
cg07505964	MIR301A	blue	0.56685451	0.72664824	-0.0502589	-0.0251931	-0.6336427	0.11680321
cg23301821	SDCBP2;SDCBP2;SDCBP2;	blue	0.63062629	0.73727633	-0.0700516	-0.0177645	-0.4735189	0.17710901
cg11641916	RP11-439C15.2;RP11-	blue	0.536906	0.60186133	0.03361146	0.10254658	-0.4877904	0.11551167
cg23717459	U6	blue	0.72962912	0.77167995	-0.1868886	0.11145641	-0.3771819	0.12723331
cg14887344	AC008410.1	blue	0.37451366	0.65247908	-0.2790688	0.01976047	-0.4482083	-0.0795634
cg17944801	RN5S196;CTB-99A3.1	blue	0.58470613	0.78127231	-0.1315932	0.01814815	-0.5190696	0.14288155
cg03860720	LRP12;LRP12;LRP12	blue	0.76250869	0.73885051	0.0876267	0.14991612	-0.430251	0.3252987
cg24178740	FEV;FEV	blue	-0.5023108	-0.6342799	0.33500697	0.06707054	0.40531209	0.13275265
cg15835620	ALDOC;ALDOC;ALDOC;A	blue	-0.4766214	-0.5656738	-0.0102427	0.0597434	0.50467822	-0.110642
cg26417468	MIR27B;MIR24-	blue	0.56593117	0.59733451	-0.0539399	0.06615352	-0.4047157	0.12230967
cg13641786	ATXN7L1;ATXN7L1;ATXN	blue	0.46807571	0.52495204	-0.2914865	0.04801486	-0.3295773	0.09165403
cg16251647	SLC17A1;SLC17A1;SLC17	blue	0.66203496	0.74493092	-0.2226396	0.02473959	-0.3955569	0.10780362
cg18667130	PSAP	blue	0.54197176	0.77842668	-0.0730967	0.00339413	-0.6180372	0.13269201
cg23530133	SLC17A7	blue	-0.3613313	-0.4046138	0.28858817	0.08870832	0.21106127	0.14311322
cg22989941	TDRD10	blue	0.71927869	0.75869324	-0.0071503	0.10177642	-0.361567	0.12939731
cg24011830	RP11-187E13.2	blue	0.50336251	0.73915031	-0.2410856	0.01977909	-0.5070078	-0.0173514
cg18446861	RP11-	blue	0.71272663	0.74918837	0.12529741	0.16551231	-0.3885694	0.26717669
cg09183284	NPR3	blue	0.66541262	0.75304588	0.05820433	0.20513249	-0.2801207	0.22451373
cg19304100	DCST1;DCST2;DCST1;DCS	blue	0.47165833	0.6374569	-0.212515	6.14E-05	-0.3319469	0.08307805
cg24343835	MIR216A;AC011306.2	blue	0.5621463	0.80559992	-0.0807042	0.04463474	-0.5149112	0.06539011
cg26508681	EPHA10	blue	0.2414276	0.61184251	-0.1121826	-0.0850963	-0.5388125	-0.1450374
cg06979587	RP11-	blue	0.44983903	0.58589721	0.0628122	0.02848288	-0.5139778	0.06268234
cg01105969	UXS1	blue	0.60432184	0.67991401	-0.133615	0.11057676	-0.422188	0.18572942

cg18284616	NFKB1;NFKB1;NFKB1	blue	0.64828453	0.7787313	-0.2641817	0.03947102	-0.4332194	0.17765429
cg02151399	CTBS;CTBS;CTBS;CTBS	blue	0.48007222	0.63148665	0.08137604	0.05674433	-0.4259329	0.07930151
cg02980499	BLNK;BLNK;BLNK;BLNK	blue	0.38887126	0.63809612	-0.321552	-0.1215209	-0.5532763	-0.0689761
cg11919883	RN5S176;RP11-122F24.1	blue	0.27700761	0.67593939	-0.1726926	-0.0978087	-0.5337963	-0.1476742
cg07829731	U6	blue	0.46189042	0.71786569	-0.1390611	0.05755381	-0.4235134	0.03778798
cg10248878	GSX2;GSX2;GSX2	blue	-0.553442	-0.6972833	0.24874831	0.0236791	0.58789299	-0.1200621
cg23457203	RP11-1016B18.1;RP11-	blue	0.611525	0.6295187	0.07902078	0.15093627	-0.2697237	0.17332941
cg07818219	LRRC31;LRRC31;LRRC31;L	blue	0.67426933	0.82830858	-0.2003593	0.10341856	-0.4437423	0.12515459
cg07337434	RP11-	blue	0.50450342	0.73540126	-0.0715366	0.01947331	-0.3851208	0.00627872
cg21368143	C12orf77;C12orf77;C12orf77	blue	0.73106418	0.71920564	0.13767854	0.08820804	-0.4889195	0.23590869
cg18577232	LINC00486;LINC00486	blue	0.66656943	0.78125479	-0.0667237	0.07565778	-0.4133622	0.14057879
cg24272313	CIITA;CIITA	blue	0.73140373	0.72162897	-0.0427053	0.17755121	-0.3535779	0.10820684
cg12883739	U6;EGF	blue	0.4459805	0.80687779	-0.0864312	-0.049565	-0.6412971	-0.0194454
cg16727987	FBN2	blue	0.62245211	0.72462396	-0.3189662	0.12092546	-0.3911916	0.0364185
cg19392109	C15orf54;C15orf54;RP11-	blue	0.52513594	0.62372454	-0.1789613	0.06883383	-0.3989854	-0.0076712
cg25084279	RP11-44I10.6;SIAH1	blue	0.68008816	0.75764124	0.19135286	0.09943387	-0.4360915	0.1120787
cg07945323	KRTAP6-2;KRTAP22-1	blue	0.5918141	0.77930951	0.07336071	0.10497228	-0.3891896	0.06536865
cg00373061	RP11-700H6.1;RP11-	blue	0.53964553	0.68447029	-0.0117586	0.13473919	-0.5145691	0.12181887
cg09459291	GFRA2;GFRA2;GFRA2;GF	blue	-0.5544632	-0.5906844	0.29068324	-0.0065826	0.34671017	0.00571296
cg12117654	ELFN2;ELFN2;RP1-	blue	-0.339231	-0.4982551	0.22762282	-0.0272501	0.32863376	0.05795129
cg26692263	ANKDD1A;ANKDD1A;AN	blue	-0.5548601	-0.7015974	0.10549619	0.00233764	0.56276773	-0.0616978
cg21614161	HGD	blue	0.47087671	0.53942422	-0.3790452	-0.0494134	-0.3073084	-0.037175
cg02411284	CLMN;CLMN;CLMN	blue	0.58595127	0.82053288	-0.138765	0.08751714	-0.4184484	0.13194674
cg00621084	TMEM194B;TMEM194B;A	blue	0.43513183	0.6288968	-0.2343777	-0.0645972	-0.3742522	-0.0873601
cg04276894	GRXCR2	blue	0.68687871	0.7389979	-0.1938722	0.08722621	-0.3725592	0.17816731
cg13935460	FAM174B;FAM174B;FAM1	blue	0.54393583	0.71501232	-0.2824892	-0.0004144	-0.3838288	-0.0096707
cg10228677	AC096753.1	blue	0.40200657	0.77919781	-0.2235402	-0.0236726	-0.5552619	-0.1109345
cg09128059	PER2	blue	0.48575882	0.71760531	0.0939293	0.06273222	-0.5814153	0.0973488
cg23956385	VSTM4	blue	-0.4082949	-0.4344365	0.35570313	0.00206363	0.2198462	-0.0094988
cg01173104	U6	blue	0.28938078	0.44085622	-0.2083594	0.03486144	-0.2749094	-0.1228394
cg11827925	AQP1;AQP1;AQP1;AQP1;A	blue	-0.4100301	-0.5691884	0.25131081	0.01741022	0.49778068	-0.0867365
cg25959826	RP11-	blue	0.73741809	0.79460984	0.05897879	0.13093161	-0.4118295	0.38037483
cg07519811	RP11-217E13.1	blue	0.67841627	0.68016617	0.24978212	0.20034301	-0.2417198	0.2367581
cg06099459	C12orf77;C12orf77;C12orf77	blue	0.63825941	0.68565705	0.03076181	0.06588966	-0.5301078	0.23339909
cg04672210	FAM169B	blue	0.59938594	0.80758667	0.13492168	0.16531915	-0.4383723	0.17017267

cg22373593	GALNT13;GALNT13	blue	0.58745166	0.8044827	-0.034256	0.06543154	-0.4048037	-0.0373619
cg25074817	MED20;MED20;Y_RNA;ME	blue	0.58390003	0.67604966	0.29206692	0.10353306	-0.4718555	0.21570215
cg08116144	DDR2	blue	0.60265922	0.81272635	-0.2205084	0.08021679	-0.4895202	0.08396102
cg20987267	U6	blue	0.7145463	0.75409874	-0.0365565	0.09406962	-0.3993475	0.2164395
cg07794775	TRAPPC10;TRAPPC10	blue	0.55009149	0.61235568	-0.2742572	0.10960467	-0.2775152	-0.0255
cg12080566	FAM176A;FAM176A	blue	0.63833947	0.71282089	0.26318354	0.06815732	-0.4234479	0.18998951
cg05917188	CD36;CD36;CD36;CD36;CD	blue	0.40960923	0.64773426	-0.1658994	0.03721693	-0.5556587	0.02002844
cg19365328	CYP2J2;CYP2J2;CYP2J2	blue	-0.4184256	-0.6050398	0.03363776	0.10925099	0.49637396	-0.0532002
cg12272235	DCP2;DCP2;DCP2;DCP2;D	blue	0.61774556	0.76680438	0.1381986	0.13415598	-0.5233435	0.2361994
cg26592496	RP1-167F1.2	blue	0.44602034	0.79877802	-0.2615587	-0.002638	-0.4464288	-0.097336
cg00861868	RP11-	blue	0.70345412	0.75038079	-0.0265315	0.18882354	-0.384436	0.21919739
cg23075090	NCAM1;NCAM1;NCAM1;	blue	0.64386332	0.72324421	0.00716181	0.1938379	-0.3196671	0.18210397
cg02158486	MAPK4;MAPK4	blue	-0.4278301	-0.5807792	0.10771469	-0.0178838	0.33262306	0.02763251
cg07042338	SDF2;SUPT6H;SDF2;SDF2;	blue	0.42470717	0.63249224	0.20090362	0.02245093	-0.4806462	0.14886463
cg07857113	C8A	blue	0.51661676	0.65880897	-0.2258184	-0.0330453	-0.3490292	0.02850982
cg02755566	TRAJ50	blue	0.49942476	0.80241172	-0.2934388	-0.0694249	-0.5946634	-0.0033191
cg05408680	RP5-955M13.4;RP5-	blue	0.55179349	0.72282682	0.02495125	0.09819215	-0.3749571	0.1462184
cg07593049	RP5-984P4.4	blue	0.63146907	0.62708518	0.04726689	0.11308667	-0.3379403	0.14652479
cg01245301	RP11-53M11.3;RP11-	blue	-0.441499	-0.6466145	0.23945783	0.03840305	0.46397712	-0.0268156
cg27175395	TMEM176B;TMEM176B;T	blue	0.66919407	0.63254725	0.21991962	0.09170606	-0.3520086	0.16850073
cg21849126	RP11-134J21.1	blue	0.61339427	0.70041894	-0.1984772	0.10072356	-0.2870594	0.10535592
cg14730112	THBS4;CTD-2201I18.1;CTD-	blue	0.55087471	0.6418002	-0.1610245	0.02315243	-0.3294784	0.04972197
cg12669065	NUMBL	blue	-0.3550619	-0.44423	0.21330204	0.11376548	0.32840499	0.12556079
cg07815287	ASAP2	blue	-0.3960398	-0.4277118	0.16127885	0.14216653	0.36305666	-0.1449735
cg10127739	SFPQ;SFPQ	blue	0.59491004	0.74387948	-0.0888308	0.1262634	-0.3808882	0.16494997
cg24014202	DLX6-	blue	-0.4910093	-0.81572	0.21806869	0.05323055	0.60748563	0.03211318
cg15676040	RUSC1-	blue	-0.3998275	-0.6289103	0.10225892	0.16150055	0.51825275	0.02707374
cg24137599	RP11-540D4.3	blue	0.55757157	0.60516317	0.10371234	0.10724541	-0.3291037	0.35717779
cg16898283	RP11-956J14.1;C8orf84	blue	0.66092516	0.72233191	-0.1801414	0.22017788	-0.2648153	0.17332824
cg27628536	CTD-2201E18.3;CTD-	blue	-0.40158	-0.4593552	0.28928314	0.05505966	0.33648067	-0.0049739
cg18528196	U6	blue	0.66662686	0.6790298	0.00537769	0.16965571	-0.2862566	0.18256706
cg26773243	CDS2;CDS2;CDS2	blue	0.66645205	0.71590629	0.02845693	0.06531599	-0.5123371	0.17840013
cg22452009	RP11-1J7.1	blue	0.63697381	0.67871594	-0.1678705	0.2042181	-0.2161943	0.0577095
cg04323816	RP5-	blue	-0.468824	-0.5963302	0.18932512	0.021725	0.38620169	0.03118652
cg19842279	KRT31	blue	0.46029883	0.5536583	-0.2973202	0.07503445	-0.1759416	0.05844437

cg06004178	MYOM2;MYOM2;MYOM2	blue	0.3881524	0.77659242	-0.1894282	-0.1171167	-0.7019156	-0.0164527
cg21804531	EFCAB11;EFCAB11;EFCA	blue	0.69544135	0.84957663	-0.1591567	0.20730243	-0.4400157	0.09136856
cg26659143	BCL7A;AC156455.1;AC156	blue	0.63748178	0.70709629	-0.1744793	0.0719896	-0.3984321	0.21894555
cg21496955	EMCN-IT3;EMCN	blue	0.41612749	0.78934224	-0.3819231	-0.0599623	-0.6661584	-0.0819276
cg25769301	CD8B	blue	0.48047772	0.71462534	-0.3303354	0.0086466	-0.5714741	0.0581999
cg02562540	FNDC3A;FNDC3A;FNDC3	blue	0.39296294	0.5515683	0.09297356	0.097619	-0.3448904	-0.0445732
cg14992808	RP11-319G6.3;RP11-	blue	0.63736476	0.74010566	-0.2671041	0.06585061	-0.3581506	0.05650904
cg16484526	C12orf77;C12orf77;C12orf77	blue	0.56149932	0.70778297	-0.1396583	-0.0070034	-0.5727926	0.06491648
cg27482594	RP11-	blue	0.48208721	0.5509359	-0.0705482	-0.085455	-0.4586832	0.10407816
cg24474561	CDIPT;CDIPT;CDIPT;CDIP	blue	-0.5070628	-0.6367804	-0.0484013	-0.0159182	0.3792732	-0.0706569
cg17159843	ERBB2;ERBB2;ERBB2	blue	-0.5328292	-0.7369231	0.30758638	0.03576533	0.55534645	-0.004183
cg02876297	KIF22;KIF22;KIF22;KIF22;	blue	0.68292748	0.67667789	0.19223146	0.12020142	-0.3904408	0.22488956
cg03829538	CYTIP;CYTIP;CYTIP;CYTI	blue	0.51174548	0.70059063	-0.1769936	-0.0240111	-0.4864916	-0.0533411
cg26870194	NAV1;NAV1;IPO9-	blue	0.34957473	0.4787269	-0.1218081	0.11256811	-0.2551817	0.1222833
cg05812836	C4orf33;SCLT1;SCLT1;SCL	blue	-0.3042834	-0.4309831	0.41169384	0.15718244	0.29254386	0.12000102
cg09461846	RP11-36B15.1	blue	0.63182636	0.66427349	-0.076677	0.1771474	-0.2971159	0.20225568
cg25153824	CCDC19;CCDC19;CCDC19	blue	-0.1888016	-0.3833319	0.43483559	0.21792085	0.33086654	0.24828711
cg22177688	C10orf90;C10orf90	blue	0.42306448	0.69090067	-0.0620031	0.05990064	-0.3687508	-0.0643588
cg09669192	MYH8;MYH8	blue	0.42445743	0.83346032	-0.2687156	-0.0488488	-0.6571919	-0.0561339
cg05123019	ALDH18A1;ALDH18A1	blue	0.75289879	0.74452779	-0.1122547	0.10000981	-0.3048918	0.27806074
cg24139120	FAM196A;FAM196A	blue	0.67618796	0.74757772	-0.1146072	0.14306075	-0.333074	0.10520013
cg24120111	AC016738.3;AC016738.3;A	blue	0.61230994	0.77134688	-0.1955777	0.10696437	-0.4229584	0.13762157
cg01726606	PMPCA	blue	0.47363228	0.6824834	-0.08791	0.15406921	-0.4342472	0.05309746
cg02345787	C12orf49	blue	0.67888624	0.84067653	0.03026691	0.06245435	-0.558509	0.26066137
cg08168997	PHF20L1;PHF20L1;PHF20L	blue	0.53590435	0.86089734	-0.1369429	0.11321767	-0.6163172	0.03499011
cg22974428	U6	blue	0.53332714	0.88273643	-0.1018805	0.04944712	-0.5530087	0.07273959
cg13087069	CPXM2;CPXM2;CPXM2	blue	0.58741439	0.86061622	0.12395224	0.09017997	-0.5457888	0.17922947
cg05075015	AC012462.2	blue	0.56622948	0.6739517	-0.2238008	0.02885647	-0.3015256	0.07410663
cg11881963	EPHX4	blue	0.52048738	0.83862333	-0.1814706	-0.0010072	-0.5553543	-0.0106732
cg18201277	BTBD10;BTBD10;BTBD10;	blue	0.50627135	0.65660553	-0.3474216	0.08869481	-0.3428272	-0.0819433
cg13590419	PKIG;PKIG;PKIG;PKIG;PK	blue	0.25209382	0.81969361	-0.099491	-0.1285364	-0.8242467	-0.1115187
cg13915716	PPP1R17	blue	0.73070268	0.75174386	0.01543893	0.11524411	-0.3314942	0.24062571
cg00281430	RP11-656C2.1	blue	0.6861808	0.75825633	-0.1326093	0.16518957	-0.3348841	0.14023538
cg10621213	ZNF335	blue	0.47934973	0.78407222	-0.1183054	-0.0698777	-0.7155751	0.07599745
cg16929960	ALYREF;ALYREF;ANAPC	blue	0.7049937	0.66633841	0.05133162	0.10986265	-0.3741177	0.16879605

cg07935562	KANSL3;KANSL3;KANSL	blue	0.59557776	0.78988674	-0.2132858	0.10013111	-0.3726905	0.15611164
cg06845268	GFPT1;GFPT1	blue	0.53192048	0.78628907	-0.2388233	0.11842279	-0.474998	0.03835198
cg12495829	Y_RNA;RP11-	blue	0.69044447	0.76822046	-0.1870873	0.12822027	-0.4286729	0.1181757
cg04423744	RHBDL2;RHBDL2;RHBDL	blue	0.53196866	0.77156889	0.07528004	0.06266127	-0.5849585	0.18970589
cg02064336	BFSP2;RP11-91K8.1	blue	0.56921634	0.64769896	-0.1740966	0.0366102	-0.4861847	0.15057604
cg05124356	COL6A4P1;COL6A4P1	blue	0.71401845	0.75326515	-0.0158154	0.13095832	-0.3720823	0.23064227
cg15039806	AQP1;AQP1;AQP1	blue	-0.5585071	-0.7780914	-0.0001014	-0.0248176	0.63150917	-0.1039599
cg22975913	WT1-AS;WT1;WT1-	blue	-0.3809999	-0.5172295	0.04449375	-0.0143995	0.37041296	-0.0049469
cg11302751	CST1;CST1	blue	0.57524195	0.70493661	-0.2745905	0.22381582	-0.3122614	0.06100268
cg20278790	CTSZ;CTSZ;CTSZ	blue	0.49083996	0.64075148	-0.2184971	-0.0369628	-0.5057116	0.07907305
cg26153664	RP11-34F13.3;RP11-34F13.3	blue	-0.5721769	-0.6944533	0.179147	-0.002557	0.56015839	0.07250384
cg19898108	HOXC10;HOXC6;HOXC10;	blue	-0.4550639	-0.5504424	-0.1284604	-0.026488	0.44701255	-0.0693759
cg08403171	ALDH1L1;ALDH1L1;ALDH	blue	0.47919002	0.67820185	-0.1480595	0.00910004	-0.3090066	-0.0805781
cg13692344	RP11-703H8.9	blue	0.49037	0.82426463	-0.2556988	0.00673742	-0.5995775	-0.0044416
cg15032098	RP11-	blue	-0.4380563	-0.5765647	0.28511123	0.01740493	0.40955031	0.01777558
cg23137518	ZNF85;ZNF85	blue	0.63327172	0.7672918	-0.0092	-0.0203508	-0.5545975	0.12060418
cg02249228	RP11-252C15.1;KIAA1456	blue	0.55137842	0.61035989	-0.1914489	0.12102803	-0.2486576	0.12137395
cg16597425	ABLIM3;ABLIM3;ABLIM3;	blue	0.59311241	0.86279117	-0.0053488	0.15720367	-0.4998236	0.08987136
cg13618141	RP4-640H8.2	blue	0.33543382	0.66991126	-0.1895332	-0.0878429	-0.4851115	-0.1075618
cg14527683	SPINK4;SPINK4	blue	0.52156427	0.53910931	0.02181477	0.01539098	-0.4028365	0.19050562
cg21262651	MRPS22;BPESC1;MRPS22	blue	0.4694032	0.58288598	-0.1259191	0.10934104	-0.3685611	0.09219926
cg02847472	EIF2B5-AS1	blue	0.48716264	0.72855197	-0.003582	0.06403431	-0.4701822	0.20784784
cg14146538	RP4-724E13.2	blue	0.52851868	0.83295839	-0.0651539	0.01911691	-0.5674665	0.01499952
cg22658316	AC063977.1;AC063977.1	blue	0.63484037	0.80982373	0.05140716	0.06587148	-0.5158635	0.20662584
cg26246101	MYRIP;MYRIP;MYRIP	blue	0.52207589	0.6407617	-0.1177464	0.08590965	-0.3216246	0.0198442
cg05225934	C1orf129	blue	0.69418109	0.79316635	0.09788637	0.1788072	-0.3916975	0.25930956
cg21130103	RP11-315A16.1	blue	0.36162289	0.73796364	-0.1811012	-0.0391954	-0.469103	-0.0690012
cg18577326	RP11-	blue	-0.5197019	-0.6529574	0.18766471	0.0061233	0.56677629	-0.1256917
cg25868978	RP5-1031J8.1	blue	0.36754222	0.72491845	-0.2131306	0.00997536	-0.5084282	0.01636669
cg03406313	AL163953.3;AL163953.3;AL	blue	0.58187448	0.81747453	-0.0482675	0.10051715	-0.4410689	0.09418491
cg02641676	C8orf40;SLC20A2;C8orf40;C	blue	0.43486578	0.51985025	-0.0424276	0.12457156	-0.2854059	0.09252885
cg10871744	C19orf77	blue	0.40147936	0.46112013	-0.2941861	-0.17336	-0.3499489	-0.0505602
cg15154666	RP11-359N11.1	blue	0.75178445	0.78908904	0.07187205	0.19583046	-0.4274898	0.31036131
cg00046432	ARGFX	blue	0.73052473	0.81845313	0.00513471	0.20843971	-0.3913147	0.17015068
cg08928966	IQCF3;IQCF3	blue	0.63068361	0.69410812	0.14403706	0.11831386	-0.3225032	0.25155165

cg25361968	PIK3AP1;PIK3AP1	blue	0.5694524	0.74618774	0.01070555	-0.0060959	-0.5923323	0.14619428
cg16341979	IFI27;IFI27;IFI27;IFI27;IFI2	blue	0.51526232	0.72647638	-0.0636766	0.08754462	-0.4253778	0.15569222
cg18844614	USP49;USP49;USP49	blue	-0.3960654	-0.7177504	0.32019466	0.07175191	0.55326463	0.01334658
cg20323072	LARGE;LARGE;LARGE;LA	blue	0.58529224	0.70030647	-0.1051438	0.16850917	-0.3015744	0.09747873
cg14868747	ALX4	blue	-0.5068526	-0.5764849	0.16825925	-0.0718479	0.38276129	-0.0928159
cg14082441	U6	blue	0.58619234	0.71415928	0.06130029	0.15070673	-0.3367569	0.16318775
cg15649077	C16orf45;RP11-	blue	0.5767214	0.84749223	-0.1815448	0.05107217	-0.4858975	0.07009863
cg12413566	XIRP1;XIRP1;XIRP1	blue	0.47420114	0.64092553	-0.1621822	0.03403245	-0.4070916	0.1155658
cg02161900	MKX;MKX;MKX;RP11-	blue	-0.5510753	-0.6325284	0.24803733	-0.018299	0.41127365	-0.0413161
cg22047350	RP11-528L24.3;RP11-	blue	-0.569593	-0.6303838	0.19296853	-0.0680932	0.37544877	-0.0973761
cg15300488	FAM82A1;AC016689.1;FA	blue	0.6640437	0.84812694	-0.1462262	0.07644348	-0.4853778	0.09996657
cg21417178	RP11-375D13.4;RP11-	blue	0.59346899	0.71102563	0.02502348	0.17579286	-0.2678104	0.14961173
cg01449754	MIR720	blue	0.42378708	0.76248154	-0.1896806	0.01772639	-0.46357	-0.0859531
cg15997127	CDH6;CDH6;RP11-152K4.2	blue	0.5432765	0.63579231	-0.3113067	0.09016635	-0.2245289	0.06873047
cg09275205	GPM6A;GPM6A;GPM6A;G	blue	0.40513143	0.6870515	-0.0269529	0.02062249	-0.3830347	-0.0774018
cg07332511	TMEM130	blue	0.57517032	0.84558718	0.03553303	0.03189673	-0.5169159	0.13944922
cg23938419	ZNF365;ZNF365	blue	-0.4687906	-0.6112266	0.09487333	0.06696138	0.46395523	-0.0159032
cg07754811	RP11-265M18.2;USP3;RP11-	blue	0.56985076	0.76985878	0.07150715	-0.0087086	-0.6247826	0.11343455
cg18454630	POC1B;POC1B;POC1B;PO	blue	0.54959034	0.85872399	-0.1863323	0.07079107	-0.5310213	0.04314903
cg13335048	SLC25A13;SLC25A13;SLC2	blue	0.62370321	0.70629509	-0.0134722	0.00652447	-0.4923478	0.24685496
cg18765270	RP5-952N6.1	blue	0.32041347	0.82015158	-0.1250623	-0.0758526	-0.627296	-0.1915221
cg13586231	RAB30;RAB30;RAB30;RAB	blue	0.67981712	0.72539114	-0.1409028	0.18175443	-0.3332327	0.15435028
cg06398473	MYO3B;MYO3B;MYO3B	blue	0.39055803	0.62418099	-0.2950109	-0.0386254	-0.4707146	0.02686448
cg12984514	NFATC4;NFATC4;NFATC4	blue	-0.4643271	-0.7493544	-0.1129697	0.06579641	0.6646439	-0.0672218
cg26454414	RN5S181	blue	0.6840879	0.82901327	-0.0485361	0.10461716	-0.5124369	0.22666557
cg19837460	RP11-753D20.3	blue	0.57755636	0.838852	-0.1694369	0.05939592	-0.5230695	0.05538551
cg02531509	BMPER	blue	0.45455785	0.80523998	-0.0651725	0.05137491	-0.5445288	-0.0198926
cg15269459	SAMD12;SAMD12;AC0235	blue	0.55244805	0.82114792	0.20128848	0.0181279	-0.6526638	0.14890089
cg25952663	RP11-834C11.3	blue	0.63729633	0.75290193	-0.0581145	0.24888175	-0.3070917	0.11132844
cg16139227	ABCB9;ABCB9;ABCB9;AB	blue	-0.5023218	-0.638885	0.15411339	0.00412405	0.52207145	-0.1540024
cg11005503	RP11-103J17.2	blue	0.34953969	0.81261817	-0.2482545	-0.0093279	-0.6116332	-0.0418141
cg18104606	COL9A1	blue	-0.4730918	-0.4533891	0.14253182	-0.0338488	0.26198611	-0.092108
cg25646679	RP11-709D24.6	blue	0.53902252	0.62364252	-0.0857671	0.09309453	-0.325794	0.10398766
cg03647068	FMNL2;FMNL2	blue	0.60527094	0.82855344	-0.0953098	0.06313054	-0.4790787	0.1289653
cg02154252	ELTD1	blue	-0.4182781	-0.3976483	0.17867433	0.00310841	0.18407552	-0.082409

cg12341047	FGF5;FGF5	blue	-0.5088474	-0.5618932	0.09562198	-0.0225174	0.33226379	-0.1292217
cg14749702	IGF2BP3;SNORD65;IGF2B	blue	0.65605219	0.73384821	0.19871949	0.12386987	-0.497026	0.26856496
cg21226227	RP4-771M4.3	blue	0.63346572	0.78419168	-0.1450914	0.1807445	-0.3283291	0.07028133
cg15331751	TMEM50A;TMEM50A;TME	blue	0.54882949	0.70076231	-0.2414036	-0.0709526	-0.6074286	0.04189845
cg00538417	RP11-439L18.3	blue	0.73537164	0.71892399	-0.0104374	0.24549448	-0.2689853	0.20019576
cg21585766	ZIC2;ZIC2;ZIC2;ZIC2	blue	-0.5144666	-0.7264284	0.19945909	0.07759357	0.52392253	-0.110469
cg20357228	TAP1;PSMB9;PSMB9	blue	0.69392188	0.75735949	-0.0357998	0.12174318	-0.4536255	0.20571034
cg24406277	CTD-2308B18.3	blue	0.74541852	0.74614716	-0.0459893	0.1770666	-0.2802062	0.25079563
cg18085517	TRPM1;TRPM1;TRPM1;TR	blue	0.57009745	0.72347479	-0.1460663	0.13641085	-0.3580102	0.03815812
cg06299718	RP3-523C21.1;RP3-	blue	0.52128481	0.86292281	-0.0934188	0.058669	-0.544779	0.0617627
cg13226907	CDC14B	blue	-0.169855	-0.3572012	0.22238516	0.23233851	0.30824346	0.05500082
cg12074247	SNORA25	blue	0.66060378	0.73943278	-0.0995498	0.13656886	-0.2537464	0.13932878
cg10035115	RP11-324P9.1	blue	0.59898024	0.80874741	-0.1314301	0.0820569	-0.5648717	0.19765134
cg03703356	CKB;CKB;CKB;CKB;CKB;	blue	-0.4826746	-0.6269231	0.14478799	-0.0839992	0.37080123	-0.104111
cg11841529	CD40;CD40;CD40;CD40;CD	blue	-0.33681	-0.5448791	0.0454145	0.03821098	0.42329075	-0.0013344
cg02732130	BNIP1;BNIP1;BNIP1;BNIP1	blue	0.48496277	0.68540189	-0.0650378	0.03691676	-0.458964	-0.0057602
cg03509250	LCT	blue	0.64193745	0.70167482	0.04588083	0.16461626	-0.2519377	0.19101654
cg15661709	RP11-76I14.1;RP11-	blue	0.30535376	0.43417284	-0.0791012	-0.046917	-0.2964013	-0.0395107
cg08636272	RP11-91K9.1	blue	0.59182899	0.70957136	-0.2503765	0.04988541	-0.3910968	0.07612757
cg14370653	RP11-447M12.2	blue	0.73136931	0.72411787	0.2059787	0.18606241	-0.3412587	0.2232583
cg09311266	LEPREL1;LEPREL1-	blue	0.54565435	0.81536682	-0.0342349	0.08549016	-0.4550688	0.05489578
cg23168459	RP11-155I9.1	blue	0.55024396	0.72200142	-0.1179381	0.09733421	-0.3251653	0.03117036
cg09414107	RP11-219F10.1	blue	0.68095378	0.71634244	-0.0976801	0.10084015	-0.3091639	0.20266195
cg07171274	AC108463.1	blue	0.69809275	0.7172799	-0.0603661	0.10477613	-0.419152	0.25515235
cg02399313	DYTN;DYTN	blue	0.55787148	0.74163347	-0.3413038	0.07948926	-0.3670887	-0.0705153
cg14497465	IL1RN;IL1RN;IL1RN;IL1RN	blue	0.40834718	0.73769615	0.03199678	0.02811424	-0.634569	0.03339348
cg27010694	AC091697.1;HDAC9;HDAC	blue	0.68661771	0.65512005	0.13249012	0.13887515	-0.4200604	0.1971425
cg09622651	LRRC37A5P;LRRC37A5P	blue	0.7571479	0.79749602	0.00919991	0.19025345	-0.3621036	0.24116971
cg15731556	PPP1R1A;PPP1R1A;PPP1R	blue	-0.5185401	-0.5876842	0.2160095	-0.0219197	0.44618525	-0.0661958
cg25073137	CASP8;CASP8;CASP8;CAS	blue	0.5496336	0.83687851	0.07752619	0.04333888	-0.6068294	0.09020469
cg00492341	MMEL1	blue	0.40253565	0.5353985	-0.1328136	0.0002747	-0.2840996	0.0464201
cg25347264	NTF3;NTF3	blue	-0.438415	-0.6262126	-0.0354124	-0.1125977	0.43460609	-0.0651037
cg22227965	LMO7;RP11-173B14.5;RP11-	blue	0.65812678	0.77998921	-0.057042	0.17720681	-0.3318675	0.15441271
cg15274964	OSBPL10-AS1;OSBPL10-	blue	0.67677027	0.7524336	-0.1071085	0.19640662	-0.325503	0.12268956
cg26781575	FAM171B;FAM171B;AC01	blue	-0.5401548	-0.7822419	0.03528184	-0.1150901	0.5788372	-0.0989877

cg01051024	TRBV7-1	blue	0.57109057	0.8626506	-0.0914009	0.07194054	-0.5142329	0.07724313
cg10199250	LVRN;LVRN;LVRN	blue	-0.4061197	-0.552615	0.03889615	0.11164816	0.3388437	-0.0395677
cg15718690	SNORA70	blue	0.65144287	0.76096348	0.14373987	0.16376336	-0.40695	0.16308088
cg04268240	AC009365.4	blue	0.6433768	0.7930852	-0.1093869	0.17168407	-0.3624811	0.09358488
cg15642035	FAM135B;FAM135B	blue	0.6220744	0.81230137	-0.0152709	0.10946553	-0.4605145	0.1298841
cg05481881	RP11-373I8.1;CLRN1-	blue	0.39107679	0.78642045	0.0759487	0.12569288	-0.5218403	-0.0007989
cg01282509	RN5S321	blue	0.58348693	0.73458046	-0.1251166	0.11388784	-0.3707353	0.03200325
cg07464845	U6;ANKRD18DP;ANKRD1	blue	0.6213181	0.70320883	-0.0769248	0.11315808	-0.3719284	0.22021266
cg17858865	ZNRF3;ZNRF3;ZNRF3	blue	0.70948504	0.79449408	0.20420745	0.22403179	-0.4122838	0.36099602
cg21925299	AC006460.2;AC006460.2	blue	0.44579903	0.61286266	-0.2803432	-0.0638761	-0.4417233	-0.038812
cg24896687	ODF1	blue	0.54670458	0.6421827	-0.3198965	-0.0316007	-0.4342567	0.04862653
cg16353127	7SK	blue	-0.4031849	-0.4521174	0.42466576	0.12034178	0.29202071	-0.0059911
cg06521280	PLAU;C10orf55;C10orf55;P	blue	-0.4140067	-0.7492016	-0.0058483	0.05736142	0.66501643	-0.0820255
cg09812790	AL132709.5;AL132709.5;AL	blue	0.54247604	0.75655944	-0.1758581	0.09683874	-0.3816502	0.0166286
cg00577905	DUSP16	blue	0.35405486	0.67963174	-0.3984937	-0.1135927	-0.598413	-0.0611705
cg23573227	KB-173C10.2	blue	0.54926798	0.72028588	-0.234562	0.0054883	-0.4144566	0.05098138
cg21809447	OLFM3;OLFM3	blue	-0.5350046	-0.732988	0.28375351	0.01254946	0.50394689	-0.0352977
cg11178281	SFTA3;SFTA3;SFTA3;SFT	blue	-0.5406303	-0.7600771	0.1456396	-0.0046676	0.52191104	-0.0990818
cg15488272	SYNE1;RP3-	blue	0.3824016	0.74201905	-0.1912756	-0.1332299	-0.6727143	-0.0359905
cg15184393	RN5S339;BDNF-AS1;BDNF-	blue	0.58804121	0.72916877	-0.1012513	0.18984543	-0.3389124	0.10144632
cg26426885	U6;LRRC4C;LRRC4C;LRRC	blue	0.37375489	0.76166156	-0.2922213	0.05590271	-0.516888	-0.0568649
cg24701602	SS18L1;SS18L1;GTPBP5;G	blue	0.45919111	0.70142656	-0.1196654	0.06752835	-0.4452857	-0.0183607
cg05683781	AC008440.10;MYADM;MY	blue	-0.5159259	-0.6162655	0.08371782	0.0191876	0.50858535	-0.1676448
cg20058318	CRISP3;CRISP3	blue	0.58973393	0.80499586	0.00421056	0.12341578	-0.5475067	0.13887658
cg12854895	Y_RNA;RP11-	blue	0.70185891	0.70867682	-0.1467948	0.28580203	-0.2671551	0.09571642
cg03552040	U6	blue	0.74482208	0.76525486	0.00424596	0.12684733	-0.3163561	0.22734697
cg00581909	RP11-429H9.4;RP11-	blue	0.58603913	0.65173161	0.1044042	0.17765014	-0.2363515	0.20720467
cg21351647	PSORS1C1;PSORS1C1;PSO	blue	0.59351087	0.83765715	-0.0214584	0.10969287	-0.4558543	0.06444995
cg16615337	STX8;STX8;STX8;STX8	blue	0.55977129	0.60084172	-0.0906372	0.04448759	-0.247616	0.12795088
cg08544192	RP11-71N10.1;RP11-	blue	0.27535526	0.78746823	-0.0549734	-0.1067003	-0.6532553	-0.0744719
cg16582957	RP11-480O10.1	blue	0.42653717	0.83774994	-0.2129131	0.02254198	-0.5993634	-0.0795851
cg19345925	CTC-498J12.1	blue	0.64633886	0.75708885	0.27344945	0.13229456	-0.52709	0.22363583
cg16324934	FLRT2;FLRT2	blue	0.57878981	0.745185	-0.0157523	0.03990097	-0.4037221	0.03341861
cg18994497	NREP;NREP-AS1;NREP-	blue	0.29884308	0.71491015	-0.2281484	-0.1848441	-0.6047798	-0.1141019
cg23338381	CTD-	blue	0.44469679	0.75752297	-0.1366265	-0.0190406	-0.6741268	-0.021933

cg12466731	APBA2;APBA2;APBA2;AP	blue	0.60549078	0.79689285	-0.1629558	0.05787942	-0.4715619	0.13612028
cg23496098	LINC00578;LINC00578	blue	0.69466696	0.70564092	0.18532863	0.17547717	-0.3220366	0.23973576
cg18185662	CTD-2572N17.1	blue	0.43200197	0.81488267	-0.2430041	0.06768108	-0.4563193	-0.1925769
cg08887146	ZNF331;ZNF331;ZNF331;Z	blue	0.46140603	0.51284228	-0.1782508	0.06355516	-0.344879	0.31152214
cg19694282	RP11-496N12.6;RP11-	blue	0.67081266	0.65379139	-0.0487305	0.09931054	-0.3292539	0.23096856
cg10206380	GPR56;GPR56;GPR56;GPR	blue	0.67638778	0.74343022	-0.0840612	0.15769401	-0.4549454	0.20047633
cg06076153	NLRP13	blue	0.63117168	0.65174712	0.09303876	0.17891318	-0.242193	0.14934451
cg25053566	SNORD81	blue	0.64170563	0.74008265	0.05167901	0.17602032	-0.4067705	0.19700771
cg16765886	C12orf12;C12orf12	blue	0.73235992	0.76607413	0.02533822	0.14420001	-0.3175689	0.2485996
cg03422350	MOCS1;MOCS1;MOCS1;M	blue	0.53680345	0.66554717	-0.2508953	0.1842094	-0.2292673	0.06464644
cg17875635	KIAA1199;RP11-351M8.2	blue	0.61821379	0.77418139	-0.055404	0.0258538	-0.5193177	0.066241
cg18294824	KRT2	blue	0.73892302	0.73304397	-0.0475621	0.17717741	-0.3225952	0.27914536
cg08439574	CHRM3-	blue	0.69320348	0.76707777	-0.086343	0.25273877	-0.3500691	0.23534995
cg24827781	ART3;ART3;ART3	blue	0.6395602	0.71873103	0.23450118	0.17461216	-0.3814025	0.26898717
cg25168653	RP11-157E21.1	blue	-0.3902518	-0.3774043	0.28838944	0.06188179	0.29047482	0.05259266
cg21477068	MIR887	blue	0.46949868	0.82824165	-0.2108398	-0.0241664	-0.5602457	-0.0557211
cg04305031	LDB2;LDB2;LDB2;LDB2	blue	0.55149679	0.77225502	-0.3276298	0.16311033	-0.3478924	-0.0857575
cg00001583	NR5A2;NR5A2;NR5A2;NR5	blue	-0.6933983	-0.7466942	0.10451226	-0.037028	0.42949025	-0.3274727
cg16736000	GATA4	blue	0.40132659	0.80220881	-0.1814417	0.10196862	-0.4457813	-0.0476612
cg12512564	ST6GAL1;ST6GAL1;ST6GA	blue	0.42596206	0.43458831	-0.3614197	0.05763238	-0.3073842	-0.0703165
cg10724894	LIFR;LIFR-AS1;LIFR-AS1	blue	0.59262354	0.85376254	0.04908455	0.07559521	-0.4563261	0.09162371
cg24361391	KRT15;KRT15;KRT15;KRT	blue	0.54568006	0.7575017	0.30120713	0.10036081	-0.5005463	0.04918382
cg04460557	SLC25A18;SLC25A18;SLC2	blue	0.3221387	0.75057783	-0.2108215	-0.0814976	-0.5059893	-0.0117681
cg20432475	CTRC;CTRC;CTRC	blue	0.77240214	0.80553689	0.04340188	0.21462331	-0.3898432	0.32346476
cg15289144	RP11-916O13.1;C12orf12	blue	0.34688806	0.77315138	0.02529473	-0.0200475	-0.6297043	0.0060626
cg11263985	FYB;FYB	blue	0.41514093	0.72452488	0.00304143	0.06939555	-0.4754417	-0.086443
cg21625740	RP11-319E16.2;RP11-	blue	0.68353776	0.76732458	0.17192455	0.18650428	-0.3241039	0.27882423
cg06418867	C10orf90	blue	0.40589119	0.76118445	-0.1722577	0.06175128	-0.492705	-0.032007
cg27097106	SIRPB2;SIRPB2;SIRPB2;SI	blue	0.45554638	0.75586889	-0.263602	-0.0257678	-0.5373877	-0.0478088
cg05551937	REST;REST;REST;REST;RE	blue	0.52636746	0.55768123	-0.1663711	-0.0326047	-0.4468507	0.02665156
cg19083458	AFAP1L1	blue	0.66041807	0.86522059	-0.0396849	0.07854428	-0.5463523	0.17626495
cg05420994	TAP2;TAP2;XXbac-	blue	0.61439053	0.59605104	0.05589933	0.25844875	-0.1907185	0.13928084
cg19855215	CRYGB	blue	0.55443729	0.84908863	0.05788481	0.08402377	-0.5862492	0.14740396
cg15762055	ZNF385B;ZNF385B;ZNF385	blue	0.55843358	0.68074911	-0.323678	0.03199982	-0.2717309	-0.0099153
cg24738606	MBD4;MBD4	blue	0.5506645	0.59575032	-0.0140347	0.12033977	-0.3499135	0.08647878

cg21585733	SLC35F2	blue	-0.6595452	-0.6564111	-0.2093964	-0.1157284	0.37021129	-0.2697558
cg12073479	WNT16;WNT16	blue	0.50344495	0.63452957	-0.3120059	0.07959902	-0.197276	0.02737859
cg09097335	BACE2;BACE2;BACE2;TM	blue	0.50508962	0.80903391	-0.0388254	0.02269968	-0.6818636	0.09431556
cg00787981	AL671972.1	blue	0.58895051	0.68604002	-0.1178111	0.064981	-0.4234418	0.11509468
cg12487647	Y_RNA;ZNF565;ZNF565	blue	0.64537846	0.67830633	-0.1653577	0.17519988	-0.3569833	0.16304692
cg27592107	AC012451.1	blue	0.68484092	0.74329225	-0.2392666	0.07339936	-0.4110256	0.07802636
cg14106660	CALCA;CALCA;CALCA;C	blue	-0.3740275	-0.5783912	0.14978984	0.14055517	0.41099695	0.1057452
cg22328742	WDR45L;WDR45L;WDR45	blue	0.70035251	0.70266785	-0.0748613	0.07015785	-0.3369563	0.12525435
cg23574215	XKR7	blue	-0.3863392	-0.3804746	0.29326146	0.09851092	0.24372424	0.08235499
cg02596045	RP11-769N22.1	blue	0.35566805	0.71079289	-0.0569892	0.05804965	-0.4382227	-7.38E-05
cg07224173	RP11-268P4.5	blue	0.58607985	0.83558587	-0.3680998	0.07932664	-0.481898	-0.0448391
cg02696428	LCP1;LCP1;LCP1;LCP1;LCP	blue	0.26584025	0.72028275	-0.0385479	-0.0322435	-0.7586692	-0.0817193
cg06749278	RP11-49O14.2;C9orf3	blue	0.46121447	0.77994459	-0.1837056	-0.0765756	-0.6528492	-0.0231401
cg01222324	VENTX	blue	-0.4572082	-0.6867398	0.08712114	0.00716963	0.5643355	-0.2166727
cg12581592	SPON1;SPON1;SPON1	blue	-0.4802761	-0.6229828	0.16288997	0.0028248	0.4459064	-0.0713488
cg10997284	RP11-890B15.3	blue	0.45686227	0.70795305	-0.2339435	-0.0326926	-0.5560741	0.0110232
cg14202477	GRHL2;GRHL2;GRHL2;GR	blue	-0.3336702	-0.535685	0.15186942	0.15254891	0.43602746	-0.005802
cg12581832	RP11-	blue	0.72470191	0.72415765	0.06179317	0.19890964	-0.2776518	0.31649765
cg19741838	RP11-849I19.1;RP11-	blue	-0.5360686	-0.8071531	0.15262368	-0.0305887	0.54913908	-0.0357428
cg18224686	CCDC167;CCDC167	blue	0.61755692	0.77115569	-0.0358227	0.21468751	-0.4804975	0.14433823
cg18765463	ZNF701;ZNF701;ZNF701;Z	blue	-0.2260701	-0.3901846	0.46845746	0.04503348	0.22096651	0.29552066
cg18642179	MGMT;MGMT;MGMT	blue	0.6395809	0.75197492	-0.2115992	0.13059715	-0.3370645	0.11948314
cg10239638	EDDM3A	blue	0.67796608	0.86709731	0.05142298	0.14041044	-0.510016	0.26277145
cg17740722	PEG3;PEG3;ZIM2;ZIM2;PE	blue	0.45744861	0.70826767	-0.1241996	0.11689703	-0.4598219	0.05283623
cg18888403	HMGCL;HMGCL;HMGCL;	blue	0.38327123	0.61688956	-0.2166738	0.01909109	-0.3823019	-0.1281962
cg01853665	RP11-14J7.6;RP11-	blue	0.63155157	0.86924563	0.06809345	0.10674039	-0.5433264	0.15537492
cg09808864	KIAA1244	blue	-0.2533169	-0.6336745	0.22471236	0.11990264	0.60647171	0.23190852
cg08814228	RP11-720D4.3	blue	0.6122081	0.81859469	0.09371234	0.1319099	-0.418988	0.12389465
cg25009435	PHLDB1;PHLDB1;PHLDB1	blue	0.63959103	0.78585227	-0.0774539	0.14220236	-0.430887	0.23014178
cg09354782	RP4-663N10.1	blue	0.37200876	0.61880417	-0.078167	0.05386622	-0.4110671	0.04173561
cg21151633	AC018866.1;AC018866.1;A	blue	0.41394924	0.63979043	-0.0654483	0.03300719	-0.3472434	-0.042747
cg22773522	DAOA;DAOA;DAOA;DAO	blue	0.51387629	0.77555351	0.14977618	0.11970172	-0.5033462	0.12731595
cg25199591	MIR2113	blue	0.35935447	0.7120485	-0.2446864	0.01870901	-0.4640816	-0.0844346
cg24639098	AC010880.1	blue	0.4462501	0.82438061	-0.2677688	0.0011376	-0.4903146	-0.0699857
cg15623866	C1orf61;C1orf61;C1orf61;C1	blue	0.40352808	0.48424744	-0.2198896	0.00160233	-0.1394592	0.08176506

cg06299093	GZMK	blue	0.32515543	0.67442314	-0.1546226	0.02867016	-0.5822692	-0.1377014
cg27183188	ALDOC;ALDOC;ALDOC;A	blue	-0.4886912	-0.6017767	-0.0902845	0.01597759	0.48102145	-0.0968158
cg13451637	C11orf40	blue	0.53069217	0.89145795	-0.0773188	0.04819042	-0.6039909	0.07907911
cg05928239	FOXE1	blue	-0.3327503	-0.7209998	0.26960326	0.16355298	0.64166908	0.07472963
cg16459890	CARD16;CARD16;CARD16	blue	0.46606765	0.68052437	0.07907282	0.08695316	-0.418255	0.0382211
cg08157569	COL1A2;COL1A2;COL1A2;	blue	0.3296785	0.77679945	-0.0428295	-0.0645573	-0.6155252	0.04364173
cg26130898	SYNE1;RP3-	blue	0.56353214	0.730088	-0.2766655	-0.000234	-0.5403636	0.13868684
cg15863314	BCL2L11;BCL2L11;BCL2L1	blue	-0.3550945	-0.781684	0.18648133	0.15506935	0.71668488	0.12952918
cg15154411	GABBR1;GABBR1;GABBR	blue	0.56897147	0.60563707	-0.0419985	0.16605703	-0.2524635	0.06558538
cg16007541	IGSF22;RP11-	blue	-0.3415959	-0.4004103	0.25209432	0.16225603	0.26913437	-0.036237
cg19181016	PTPN5;PTPN5;PTPN5	blue	0.50292721	0.72552597	0.01046124	0.04562982	-0.3974982	0.1568383
cg05054673	RP11-317M11.1;LNX1;RP11-	blue	0.47166025	0.8331238	-0.1053171	0.08516858	-0.4941541	0.0320927
cg11485188	ADAMTSL1;ADAMTSL1;A	blue	0.26890824	0.78319969	-0.05721	-0.009726	-0.704871	-0.1003649
cg18201034	CYB5R2	blue	0.5773171	0.72972447	-0.048649	0.08310013	-0.408329	0.21129489
cg16433357	SH3BGRL2	blue	-0.4850112	-0.4799134	0.05881039	-0.0214571	0.39472342	-0.1468673
cg23906060	ANKRD34C;RP11-	blue	-0.2788129	-0.480567	0.2623552	0.05968669	0.34242861	0.09098072
cg24906316	AC010872.2	blue	0.66199883	0.64195261	0.11364616	0.26991274	-0.2482289	0.19798768
cg23829024	HIST1H1A	blue	-0.4286366	-0.5170255	0.18516176	-0.128005	0.24559962	-0.1612494
cg05525416	PAG1;PAG1	blue	0.32798737	0.78665423	-0.0331845	-0.1123768	-0.8330656	0.04915882
cg11076544	RP11-709D24.6	blue	0.41375403	0.7096557	-0.2696375	-0.0211525	-0.5708749	-0.0036402
cg14909595	RP11-794P6.2;POU2AF1	blue	-0.4787781	-0.5996339	0.15203342	0.01760389	0.48437186	-0.1182854
cg02940093	EGF;EGF	blue	0.46929512	0.90411475	-0.1238852	0.02165731	-0.6264855	-0.0240159
cg01121211	HRH3;HRH3	blue	-0.5228965	-0.6667698	-0.0734772	-0.0358986	0.44226941	-0.1249623
cg01474253	U6	blue	0.46522127	0.78163341	-0.2175109	0.05203425	-0.5518923	0.06928947
cg09726316	IGHA2	blue	0.58282053	0.67263462	0.13591008	0.03005565	-0.4128804	0.19051176
cg06147496	AP000402.3;AP000235.2	blue	0.64166995	0.63656211	0.03569491	0.15084343	-0.2511123	0.1541267
cg04745278	OSTN-AS1	blue	0.47734651	0.78811603	-0.2550487	0.01802911	-0.5582817	0.05754769
cg16964478	RP11-626I20.3;RP11-	blue	0.62701117	0.67897458	-0.1348967	0.16296641	-0.2360655	0.10004101
cg25357163	RRAGC	blue	0.50492578	0.88607389	-0.0590715	-0.0441891	-0.7178405	0.104182
cg22051124	ZNF287;ZNF287;ZNF287	blue	-0.357766	-0.4671293	0.3011829	-0.0003351	0.35459351	-0.0966478
cg13552468	U2;GS1-179L18.1	blue	0.49314615	0.7746916	-0.113259	0.09957617	-0.3828607	-0.0006679
cg06939923	KB-173C10.2	blue	0.53036841	0.60729488	-0.3320944	0.09677008	-0.2901544	0.08563051
cg00476906	STAT3;STAT3	blue	0.68803598	0.78309197	0.10092572	0.20831587	-0.4490761	0.28854453
cg11658046	PSD3;PSD3;PSD3;PSD3;PS	blue	0.36439947	0.83358292	-0.0811799	0.00957502	-0.6517845	-0.0516346
cg21129117	CTC-479C5.12;CTC-	blue	0.58848273	0.66298284	0.05058292	0.01096737	-0.3717594	0.12584773

cg00715811	OR2W5	blue	0.65463525	0.76622797	-0.1651483	0.16404694	-0.3412733	0.17515243
cg04027961	AC021021.2	blue	0.59752587	0.66930622	0.07176347	0.10615806	-0.352678	0.13287612
cg10805440	KRTAP27-1	blue	0.34356248	0.77909487	-0.1687976	-0.0292911	-0.5842523	-0.0327729
cg05446446	U6	blue	0.53521625	0.87705394	0.01082691	0.10406358	-0.6274794	0.15072583
cg11059285	7SK;CNTNAP4;CNTNAP4	blue	0.35182069	0.59009725	-0.0412346	0.00609275	-0.3137278	-0.0020062
cg20211044	PHC1;PHC1;PHC1;PHC1;P	blue	0.57823912	0.81508477	-0.1827878	0.05707928	-0.3862511	0.0738552
cg14643648	7SK;COG7	blue	0.56942007	0.77978834	-0.0037413	0.01489892	-0.4659753	0.0660119
cg00077877	ASAP1;ASAP1;ASAP1;AS	blue	0.59424242	0.80338024	0.01074571	0.06532584	-0.5338932	0.14792251
cg04965200	RP11-202G11.2;RP11-	blue	0.38282171	0.73640987	0.0645158	-0.0799106	-0.7314066	0.0222672
cg03438613	NBL1	blue	0.62178108	0.71048755	-0.0284989	0.14707048	-0.3591349	0.21526763
cg16377872	SLC1A6;SLC1A6;SLC1A6	blue	0.71280705	0.73316312	-0.1302012	0.11885739	-0.3022375	0.23934239
cg20719850	RERG;RERG;PTPRO	blue	0.51527193	0.75123297	-0.2541873	0.03034565	-0.4092612	0.01583
cg19915564	FXYD4;FXYD4	blue	0.7189515	0.72443366	0.18855458	0.18512191	-0.3162472	0.15173982
cg04645211	LOR;LOR	blue	-0.3495784	-0.5690104	0.16646921	0.00230201	0.46609847	0.13343639
cg01910527	TTYH2;TTYH2;TTYH2;TTY	blue	0.61564249	0.70043047	0.0576691	0.12101059	-0.4149616	0.27129125
cg06373787	7SK;ASTN2	blue	0.62402241	0.79857765	-0.0339043	0.13797717	-0.338324	0.12065081
cg18204274	SVIL;SVIL;SVIL;SVIL	blue	0.68372371	0.82996253	0.01356539	0.18857781	-0.4360362	0.21088264
cg03483464	CPOX;CPOX;CPOX	blue	-0.456947	-0.5132544	0.12942839	0.0856225	0.4172573	-0.0252455
cg06204665	NIPAL3;NIPAL3;NIPAL3;N	blue	0.40900739	0.57861341	0.09282335	0.04686838	-0.4881209	0.05632772
cg20584961	AC114808.3	blue	0.71232016	0.69860064	0.11688581	0.19844997	-0.2583739	0.24952029
cg00152095	EFR3B	blue	0.62870149	0.84076803	-0.086744	0.10877808	-0.51383	0.17897247
cg12691372	Y_RNA	blue	0.62352525	0.74116726	-0.3597915	0.05881043	-0.3966441	-0.0137155
cg05386594	TMEM56;TMEM56;TMEM5	blue	-0.5384367	-0.6244918	-0.0628659	-0.0565804	0.37161736	-0.1652274
cg09637885	DDX4;DDX4;DDX4;DDX4;	blue	0.36403061	0.82102447	-0.1730522	-0.028933	-0.6223302	-0.0015496
cg26371224	SPINK13;SPINK13	blue	0.76783898	0.77366251	-0.0747503	0.16652817	-0.3473112	0.19657724
cg25419339	U6	blue	0.50060304	0.69586022	0.1883173	-0.0154884	-0.6151416	0.20027924
cg05139138	7SK	blue	0.49661638	0.81978668	-0.0182325	0.00156439	-0.7205299	0.02429557
cg02023255	RP11-841O20.2;ERO1L	blue	0.36632799	0.82046219	-0.1188297	-0.0234334	-0.6585143	-0.0597254
cg11196428	FGD3	blue	-0.5392923	-0.7189567	0.03289526	0.00052721	0.57492037	-0.1342896
cg23627908	SPINK1;SPINK1	blue	0.61976483	0.87756866	-0.0512434	0.10758305	-0.5483476	0.17993574
cg15715507	HARS;DND1;HARS;HARS;	blue	0.60725659	0.86833547	-0.0748769	0.03434385	-0.5518155	0.15967408
cg17876645	DNAJC6	blue	0.75629352	0.76510552	0.11078663	0.20849615	-0.3796803	0.33750787
cg12145580	U6;WDR72	blue	0.59605094	0.78272333	-0.0624451	0.14578605	-0.4216524	0.18746614
cg16035840	RHOBTB3;RHOBTB3;GLR	blue	0.52909134	0.69291656	-0.2084792	0.0633839	-0.419791	0.10421358
cg05675705	ST14;ST14;ST14;ST14	blue	0.65760315	0.71286245	0.09071111	0.07607657	-0.5139007	0.19976401

cg16377007	SKP1P1;GS1-124K5.2	blue	0.70204981	0.79660735	0.08075564	0.18213687	-0.4076899	0.26765273
cg13671732	RIN2;RIN2;RIN2;RIN2	blue	0.53873772	0.80619617	-0.0868892	0.16920708	-0.4183605	0.11988551
cg01706445	ATF7IP;ATF7IP;ATF7IP;AT	blue	0.58915912	0.83605397	-0.0427298	0.06948916	-0.5910317	0.13609341
cg04292080	MIB2;MIB2;MIB2;MIB2;MI	blue	0.37004549	0.62829876	-0.2279201	-0.0297597	-0.3921771	0.05009348
cg09131617	TLN2;RP11-	blue	0.42240137	0.89361351	-0.061385	-0.0248411	-0.7054143	0.02251187
cg25249448	MIR216A;AC011306.2	blue	0.55300369	0.64836375	-0.1031774	0.12790793	-0.2691603	0.14783376
cg24020842	DFNB31;DFNB31	blue	0.74685677	0.73248216	0.0277171	0.2102416	-0.2863683	0.29682553
cg08326410	KIR2DL4;KIR2DL4;KIR2D	blue	0.50478984	0.70915589	0.04052782	0.00958306	-0.4907907	0.14936121
cg16740299	FREM2	blue	0.50734227	0.81604085	0.06005503	0.08327812	-0.4838729	0.06475502
cg02792333	GPR137;GPR137;GPR137;G	blue	0.58311057	0.71345116	0.14851688	0.05468665	-0.3675467	0.21224341
cg11273037	AC023115.2;AC023115.3	blue	0.56704623	0.82859616	-0.0306584	0.16293483	-0.382947	0.06649686
cg02406543	AL023913.1;RP5-	blue	0.53366052	0.78297018	0.03389282	0.05577844	-0.4739812	-0.0140652
cg13302163	FAM92A1;LINC00535	blue	0.61692474	0.66958401	-0.2184081	0.11928024	-0.3510566	0.00903209
cg16564049	AC012501.2;AC012501.2;SN	blue	0.43014457	0.79414634	0.09774193	0.08836439	-0.5505311	-0.0246339
cg04290158	HOXB-AS4;RP11-	blue	0.47045225	0.81343458	-0.1493948	-0.0830727	-0.6204458	0.04038711
cg14839934	AC103563.9	blue	0.56553532	0.66472059	-0.1769732	0.07738356	-0.4061668	0.09797837
cg15303959	RNU7-54P	blue	0.59285469	0.8285452	-0.0517573	0.02110303	-0.6337941	0.16698858
cg01103387	CYP1B1;CYP1B1;CYP1B1	blue	-0.4099307	-0.5221867	0.16269741	0.02101283	0.27191709	-0.0608054
cg27205798	TMTC1;RP11-	blue	0.55519694	0.84799341	-0.0381229	0.06808947	-0.4933233	0.03467312
cg26753302	SERPINB5;SERPINB5;SER	blue	0.41443051	0.45176891	-0.1269903	0.07931403	-0.2362069	-0.0578186
cg22178396	CEP68;CEP68;CEP68	blue	0.70636053	0.66753453	0.08650284	0.16839659	-0.3813243	0.18208245
cg11277230	C4orf36;C4orf36;C4orf36;C4	blue	-0.3363962	-0.6675469	0.14106938	0.08217189	0.59431003	-0.0621975
cg09771880	PRRX1;PRRX1	blue	0.44990567	0.76354858	-0.1973	0.07416818	-0.4046554	0.05579705
cg16009352	U6;RP11-73O6.4	blue	0.70576909	0.68844409	0.11536774	0.10587619	-0.3076187	0.27586519
cg19513335	RP11-84D1.1;RUNX3	blue	0.66693601	0.68941054	0.08263267	0.07326762	-0.4285412	0.26829187
cg16294013	PDE4B;PDE4B	blue	-0.3904463	-0.6278137	0.17802355	-0.0279885	0.43519901	0.01215071
cg20804721	RYR2	blue	0.37357704	0.83065953	-0.1420615	-0.0504421	-0.6963905	-0.0510059
cg01101733	U8	blue	0.44094803	0.88317615	-0.0862558	0.0059333	-0.6788482	-0.0567706
cg14970612	GCNT1;GCNT1;GCNT1	blue	0.49636876	0.72392721	-0.2059643	0.04146332	-0.4563564	0.11530126
cg13668339	RP11-81N13.1;RP11-	blue	0.24951985	0.49724122	-0.104667	0.06490216	-0.3688827	-0.0990874
cg01865091	NFRKB;NFRKB;NFRKB;N	blue	0.60211164	0.65711063	-0.0646507	0.16011047	-0.3132568	0.16341321
cg10711190	SMAD3;SMAD3;SMAD3;S	blue	0.44883281	0.72924303	-0.1578241	-0.0424044	-0.555127	0.0962035
cg00097237	AC091736.1	blue	0.46739779	0.71989902	-0.0802505	0.00092814	-0.4935534	0.07155413
cg00803692	CCR5;CCR5;RP11-24F11.2	blue	0.3068075	0.84035416	0.01115597	-0.0963787	-0.8401185	-0.0330219
cg17192115	GAS7;GAS7;GAS7	blue	-0.6183906	-0.8139542	0.08860359	-0.0722294	0.54139636	-0.2102495

cg02976475	ROBO4;ROBO4;RP11-	blue	0.72651013	0.80420111	-0.0813416	0.16339311	-0.376629	0.15032206
cg16864758	FREM1;FREM1;FREM1	blue	0.70630451	0.69417059	-0.0522767	0.21549454	-0.216864	0.23324576
cg01942023	COL3A1;COL3A1;COL3A1	blue	0.68748141	0.65328261	0.27122392	0.19509237	-0.2809849	0.28471522
cg12263857	RNF217;RNF217	blue	0.65523986	0.83156954	-0.1224589	0.12789607	-0.4514693	0.19766256
cg09549827	HES2;ESPN;HES2	blue	-0.5929725	-0.6432066	0.12556226	0.01117369	0.38153096	-0.0635142
cg24009331	AC023347.1;AC023347.1	blue	0.4283355	0.8082238	-0.203812	0.09113884	-0.5183271	-0.0651145
cg07105757	AC096669.2;AC096669.3;A	blue	0.62021038	0.77330492	-0.1895619	0.03484583	-0.3615421	0.1146683
cg00925766	EXD2;RP11-	blue	0.65461918	0.73350331	-0.1671747	0.12955542	-0.3712339	0.20386894
cg14264219	RP11-881M11.8	blue	0.66674232	0.71877087	-0.1113753	0.14786165	-0.3872214	0.21351504
cg09933273	AC004041.2;RAD50;AC004	blue	0.62929487	0.8405114	0.18788314	0.12000537	-0.5783248	0.26846679
cg24086845	MTSS1;MTSS1;MTSS1	blue	0.54011591	0.6715564	-0.0868026	-0.0342817	-0.5536543	0.06329297
cg04557125	RP11-457P14.4;RP11-	blue	-0.4765789	-0.6768292	0.14492326	-0.0440483	0.40592973	-0.0724426
cg24196622	AC011897.2	blue	0.46529894	0.68220946	-0.1775082	-0.015127	-0.3624898	-0.0307218
cg22402753	OR9G4	blue	0.60591936	0.79477182	-0.1194549	0.10830801	-0.3813384	0.14921572
cg23257564	SYCP1;SYCP1;SYCP1	blue	0.71605955	0.73468804	-0.0996273	0.10505712	-0.3013355	0.16746302
cg08859379	RBFOX1	blue	0.35183707	0.39006071	0.04042727	0.05895633	-0.0663467	0.02218861
cg10993783	RP11-53M11.3;RP11-	blue	-0.4232571	-0.525244	0.0322046	-0.0279124	0.28793408	-0.0881622
cg20494779	MC2R	blue	0.60881253	0.82568882	0.03765967	0.16845819	-0.4426246	0.19509178
cg13563959	U6;WDR72	blue	0.65384113	0.80244806	-0.1085358	0.13655963	-0.3555665	0.19658631
cg24587119	ST13P19	blue	0.33841815	0.70611838	-0.2837167	0.03787867	-0.339896	-0.1709424
cg19457556	ANK3;ANK3;RP11-	blue	0.65675443	0.78823231	-0.0483629	0.07603226	-0.5129557	0.19482149
cg14842816	PITRM1;RP11-298E9.3	blue	0.70936239	0.81964737	0.07185145	0.06597755	-0.5066937	0.27321158
cg08049537	RP11-332J15.2	blue	0.54245219	0.70253438	-0.2465329	0.02436693	-0.3814337	-0.0795479
cg19452094	U2;GS1-179L18.1	blue	0.50923585	0.85945743	-0.1318348	0.04433529	-0.5390195	-0.0034409
cg04252275	SRRM3	blue	0.66146248	0.78518304	-0.1175208	0.15105742	-0.4054578	0.08979587
cg03085618	BTBD1;BTBD1;RP11-	blue	-0.5573778	-0.5247815	0.22498787	0.08161725	0.43267796	-0.1842891
cg03937517	U6;TFP1;TFP1;TFP1	blue	0.32577183	0.64979304	-0.1883798	0.07775491	-0.3977635	-0.1506802
cg02842704	GLRA1;GLRA1;GLRA1	blue	0.39233063	0.67702316	0.07429728	0.08942939	-0.4613072	0.00889922
cg10492152	IL17A	blue	0.72154997	0.67120647	0.25460144	0.15219624	-0.3673204	0.24865486
cg06022300	CTD-2021H9.2	blue	0.53465633	0.86408215	-0.0913081	0.06008473	-0.5355175	0.03148464
cg18803429	AC006378.3;AC006378.2;A	blue	0.67093057	0.8143584	0.02224847	0.20182957	-0.376549	0.21491457
cg07546334	RP11-38P22.2	blue	0.34411606	0.84631225	-0.1220724	-0.0782953	-0.7627474	-0.0848413
cg16544169	NKX2-6;NKX2-6;RP11-	blue	-0.3773155	-0.7963455	0.28724928	0.0857406	0.71801552	0.10500147
cg08009622	COL12A1;COL12A1;COL12	blue	-0.3565185	-0.5402149	0.35832768	0.02882343	0.30478404	-0.0248354
cg19894527	RP11-572C15.5	blue	0.67871963	0.74611549	-0.2586037	0.1087346	-0.3859682	0.13192042

cg09448652	SNHG1;SNHG1;SNHG1;SN	blue	0.28561433	0.74007755	-0.0558888	-0.0137013	-0.7276464	-0.0341717
cg11110406	RNF113B;FARP1;FARP1;F	blue	0.60810362	0.69769764	-0.0516794	0.20912123	-0.2451585	0.11667075
cg02463253	PIPOX;PIPOX;PIPOX	blue	-0.4094211	-0.4035934	0.27388976	0.13060077	0.24544416	-0.0341208
cg24782228	AC006460.2;AC006460.2	blue	0.65248289	0.71719607	0.02935248	0.1214528	-0.4790438	0.1992542
cg03798688	CTXN3;CTXN3;CTXN3;CT	blue	0.69411804	0.7401015	-0.3022386	0.1025145	-0.3401138	0.1172728
cg07093966	U6atac	blue	0.55343798	0.76253113	0.25475997	0.12957453	-0.46779	0.15268196
cg04296842	MPPED1;MPPED1;MPPED	blue	0.60227506	0.84646844	-0.1097843	0.13392387	-0.4158598	0.16460936
cg15215681	SPRED3;SPRED3;GGN	blue	-0.4784254	-0.6119283	0.26137884	0.16361787	0.50690593	-0.0170815
cg23250795	ST14;ST14;ST14;ST14	blue	0.63863678	0.75226267	-0.0935567	0.06543772	-0.4186387	0.16673872
cg06768955	RP11-22A3.2	blue	0.68253189	0.7347884	0.02847517	0.17039388	-0.2907994	0.23613452
cg05503019	ITGAM	blue	0.60227972	0.78879001	0.07417643	0.05868914	-0.5456266	0.16478235
cg19721797	snoU13	blue	0.56764433	0.68531107	-0.0541956	0.04543885	-0.3309351	0.06824259
cg05106778	SDC2;SDC2;SDC2;SDC2	blue	0.35674447	0.85624776	0.07948026	-0.0687089	-0.7760009	0.0236603
cg11167905	CNTRL;CNTRL;CNTRL	blue	0.60184649	0.59663077	0.30252197	0.14763272	-0.3307635	0.23138428
cg04906607	NTRK3;NTRK3;NTRK3;NT	blue	0.36886691	0.81219645	0.03237837	-0.0302269	-0.6727666	0.03383585
cg07472627	TSHB	blue	0.56748786	0.80926028	-0.1711913	0.10234419	-0.4783448	0.1217402
cg10724297	RMI2;RMI2	blue	0.71682462	0.72894243	0.02077189	0.15028972	-0.4570195	0.31680484
cg07944811	GCK;GCK;GCK;GCK	blue	0.57465751	0.70360226	0.0573746	0.11417255	-0.363267	0.04512361
cg03430502	GAP43;GAP43;GAP43	blue	-0.696506	-0.6865265	0.09420064	-0.0222157	0.29707452	-0.1196984
cg07018739	DNAH10OS;DNAH10;CCD	blue	0.65325929	0.75796538	-0.0447079	0.14964482	-0.3064117	0.16164206
cg22717080	RPS6KA2;RPS6KA2;RPS6	blue	0.66482256	0.79543461	0.06103856	0.18531674	-0.4631939	0.19223305
cg05600352	AP2B1;AP2B1;AP2B1	blue	-0.4112729	-0.4679092	0.20909296	0.00079776	0.37331428	-0.0656407
cg24094087	C8orf12;FAM167A;FAM167	blue	0.6952044	0.74256565	-0.0785677	0.20249919	-0.2408316	0.26221322
cg07466193	HBM;HBM;HBM	blue	-0.5235767	-0.6013644	0.02861347	-0.0666178	0.32812255	-0.2067361
cg11184333	KLHL2;KLHL2;KLHL2;TM	blue	0.42636097	0.77299682	-0.2093474	-0.0041509	-0.6311428	0.05423705
cg11108051	ATP13A5-	blue	0.51612864	0.83607754	-0.013406	0.02623549	-0.5036416	0.12477911
cg21860300	CTD-2382E5.2	blue	0.6012469	0.84550352	0.09711749	0.06674023	-0.588249	0.17500007
cg21817737	NAALAD2;NAALAD2;NA	blue	-0.433349	-0.5797102	0.15091369	-0.0563781	0.39305063	-0.0218772
cg04717258	RNU5B-4P	blue	0.46195763	0.88698403	0.05146625	0.00062916	-0.7273647	0.1040262
cg15660094	SLC27A2;SLC27A2;ATP8B	blue	-0.5663073	-0.6046247	0.04037576	0.02661023	0.4828015	-0.1473169
cg06509035	U66059.58	blue	0.59007029	0.91005803	0.06006877	0.06850408	-0.618927	0.12995559
cg09135441	GRIP1;GRIP1;GRIP1;GRIP1	blue	0.52925141	0.70155813	-0.2561191	0.08038806	-0.4232048	0.04244696
cg05126887	COL21A1;COL21A1;COL21	blue	-0.4148067	-0.6492763	0.13858411	0.05962672	0.52241473	0.05439829
cg00621646	OR10S1	blue	0.41508202	0.86896015	0.01204028	0.00984335	-0.701999	0.02370552
cg25527871	SNORA48;RP11-	blue	0.24861813	0.62733093	-0.1711708	-0.1103882	-0.5458547	-0.2884916

cg07715497	RP11-21L1.1	blue	0.58286011	0.69851626	-0.1111027	0.20977487	-0.1955232	-0.03271
cg13769792	AC022692.1;LYSMD4	blue	0.40639431	0.78910398	-0.1903208	-0.0506212	-0.6201896	-0.0178672
cg08945844	RELN;7SK;CTB-107G13.1	blue	0.51158351	0.83108181	-0.2385759	0.03288325	-0.4797346	-0.0240079
cg12238440	RP11-91I8.1	blue	0.49487746	0.81295442	-0.2931784	0.04057365	-0.4438098	-0.0346113
cg25508633	PPAN;SNORD105;PPAN-	blue	0.29008767	0.83070958	-0.2264219	-0.0987129	-0.7217838	-0.0877592
cg17140352	AC104417.1	blue	0.59334185	0.73603808	-0.0177615	0.12626466	-0.2950832	0.14058205
cg01197156	FBXO24;RP13-	blue	0.59554801	0.68028846	0.10006141	0.15132335	-0.3099125	0.02332818
cg18224077	RP11-166D19.1;RP11-	blue	-0.4915787	-0.5496104	0.10929166	0.00466231	0.40629477	-0.085319
cg14586941	LAMB3;LAMB3	blue	0.50098926	0.87623544	-0.1501215	0.0220551	-0.610645	0.0543246
cg25593128	C16orf89	blue	0.39919366	0.80049894	-0.0029715	-0.0398516	-0.7072535	0.0052854
cg11320271	CCDC36;CCDC36;CCDC36;	blue	0.53236237	0.78656117	0.17677521	0.10524056	-0.4847288	0.14079643
cg17866732	SEPT10;SEPT10;SEPT10;SE	blue	-0.455048	-0.6154011	0.30923816	0.04124085	0.40661904	-0.0139781
cg15994599	RN5S229;BBS9;BBS9	blue	0.55450309	0.79529119	-0.2891299	-0.0587537	-0.4838346	0.02278796
cg05968904	FAM20C;FAM20C;AC0936	blue	0.54876298	0.62049479	0.02443177	0.15639703	-0.3148888	0.20022771
cg17457241	ABAT;ABAT;ABAT;ABAT;	blue	-0.4706577	-0.579729	0.20156882	0.04302446	0.41279045	-0.1074965
cg10843046	POLG;RP11-217B1.2	blue	0.31760149	0.63932979	0.11440049	-0.1176353	-0.4553267	-0.0972352
cg16131616	RNU7-65P	blue	0.36393629	0.7596453	0.05326091	0.04430749	-0.5636447	0.00735374
cg02584094	PITPNM3;PITPNM3	blue	0.40293463	0.81550715	0.01561103	-0.016786	-0.614602	0.04493467
cg21478437	CTSE;CTSE;CTSE	blue	0.70482354	0.76796558	0.11185	0.22852255	-0.2868357	0.28151597
cg08703540	ISLR2;ISLR2;ISLR2;ISLR2;	blue	-0.4985275	-0.5983642	0.12841395	-0.051314	0.3907743	-0.0199949
cg08320359	C16orf45;RP11-	blue	0.75089574	0.72884447	0.03871506	0.18176512	-0.3701604	0.28517963
cg16321048	CTD-2223O18.1;KIF26A	blue	-0.5458947	-0.7917556	-0.0291308	-0.0248869	0.57341956	-0.1618402
cg05210769	TCF20;TCF20;TCF20	blue	0.75223079	0.76712972	0.14149078	0.15494705	-0.3324445	0.28763588
cg17411055	VGLL3;VGLL3	blue	0.32702004	0.83680346	-0.2156448	-0.0365043	-0.7055147	-0.1067453
cg22833175	WBP11;WBP11;WBP11;WB	blue	0.52327356	0.717048	-0.1754925	0.11517245	-0.362868	0.0089538
cg12315723	FUBP1;FUBP1;FUBP1;FUB	blue	0.37828088	0.79132393	0.11605443	-0.1017721	-0.7557937	0.09293276
cg10074641	CSMD3;CSMD3;CSMD3;C	blue	-0.3773227	-0.5789972	0.03494896	0.05046605	0.46977847	-0.0245675
cg04458670	KIAA0947;KIAA0947;KIA	blue	-0.3781895	-0.3976841	0.22845666	-0.0192065	0.20301874	0.01764836
cg21489458	RP1-	blue	0.68087266	0.73168189	-0.1887076	0.21861559	-0.3171442	0.1062217
cg00258409	CPE;CPE;CPE;CPE	blue	-0.4653494	-0.6509433	0.20976237	0.05816737	0.49647404	-0.0187228
cg18278573	LRCH3;LRCH3	blue	0.53244694	0.82568344	-0.2143483	0.03211268	-0.5861467	-0.0155151
cg24432832	CDH10;CDH10	blue	-0.3654841	-0.6152517	0.14408971	0.05464331	0.50017106	0.08184409
cg09728659	PRODH;PRODH;PRODH;P	blue	0.75051294	0.73154969	-0.0461459	0.23069116	-0.2665649	0.26101503
cg00333310	U6;RP11-154D17.1	blue	0.49856701	0.83589635	-0.1331009	0.06413841	-0.4934967	-0.0335896
cg25672669	U6;SLC44A5	blue	0.37300372	0.71219735	-0.1426085	0.07551014	-0.4122869	-0.144952

cg22535267	ARHGEF12	blue	-0.415712	-0.6144782	0.16049416	0.02074446	0.5079439	-0.0333696
cg20746551	RP11-94H18.1;RP11-	blue	0.59385912	0.8563084	-0.1542332	0.02863052	-0.5408835	0.09013262
cg24015237	RP4-781L3.1	blue	0.54895187	0.690459	-0.08461	-0.0028978	-0.5661984	0.10353942
cg12974637	CCDC68;CCDC68;CCDC68	blue	-0.3727354	-0.3716932	0.10508335	-0.0516525	0.24919415	-0.1545189
cg12610449	RP11-152O14.1;RP11-	blue	0.42689258	0.8182193	-0.0338113	0.07529246	-0.5543688	0.00527732
cg03268007	LINGO1;LINGO1;LINGO1;	blue	-0.5900377	-0.7743295	0.05463232	-0.1121104	0.49690844	-0.1078184
cg01599028	MIR31HG	blue	-0.5476625	-0.6562445	0.22272081	0.04400161	0.43293225	-0.0414361
cg22559712	IQCA1;IQCA1;IQCA1	blue	0.62271285	0.7941788	-0.2411578	0.07295885	-0.3821226	0.04887824
cg16845363	ARPP21;ARPP21;ARPP21;	blue	0.72422701	0.73092759	0.02606992	0.25212836	-0.157355	0.20320912
cg27431545	RAPGEF4;RAPGEF4;RAPG	blue	0.61049317	0.79943802	-0.0282281	0.14448566	-0.4423784	0.05593043
cg26930627	RP6-91H8.1;RP1-292L20.1	blue	0.41870851	0.82219075	-0.1078839	-0.0924212	-0.7048196	0.05158247
cg16259591	PNLIP;PNLIP	blue	0.47450983	0.65660305	-0.2258368	0.11284202	-0.305657	-0.0170912
cg22652552	AP000343.2	blue	0.4225769	0.88612596	-0.0292979	-0.0578585	-0.7459795	0.06339495
cg08006727	RNF183;RNF183;RNF183;R	blue	0.37140063	0.8543305	0.01841465	-0.0392354	-0.7637929	0.02017125
cg06712065	AC006305.1	blue	0.50832615	0.80149941	0.18359331	0.13499234	-0.4652949	0.03839173
cg01339648	LY6G6C;C6orf25;C6orf25;C	blue	-0.3762269	-0.7462225	0.14823033	0.02846868	0.6772606	0.05583581
cg13031003	RP11-519G16.3;RP11-	blue	0.63521965	0.72987063	-0.165565	0.14573572	-0.4139562	0.163255
cg03735065	COL13A1;COL13A1;COL13	blue	0.55172353	0.79517716	-0.1333302	0.07229863	-0.4813806	0.10109363
cg14909341	RP11-323H21.3	blue	-0.5554201	-0.6464861	0.15856507	0.00875255	0.5327946	-0.0571406
cg19574906	RP11-227H15.4	blue	0.65427067	0.71540262	-0.0902029	0.12846296	-0.4331068	0.21673418
cg09716731	IL1R2;IL1R2	blue	0.50616807	0.85296206	-0.0802177	0.09820137	-0.5362171	0.03977996
cg19406923	AC062020.1	blue	0.39787253	0.84271684	-0.1485209	-0.0898325	-0.6934377	-0.0437322
cg23831890	RHCG;RHCG;RHCG;RHCG	blue	0.62409917	0.61414521	0.10116411	0.27221617	-0.2756283	0.13707966
cg08896397	RP11-35J10.4	blue	0.57972797	0.71402497	-0.3008798	0.11296656	-0.2704986	0.075199
cg23610455	PPP1R32;RP11-	blue	0.53630678	0.87431315	-0.1265648	0.02623628	-0.6146305	0.06399294
cg03069148	RP11-1028N23.2;RP11-	blue	0.38704468	0.54180794	-0.3265688	0.07434383	-0.2712043	0.01163514
cg15049626	GABRB3	blue	0.29048919	0.65973483	-0.0163797	-0.0171665	-0.4414055	-0.106806
cg10394922	ZBTB38;ZBTB38;ZBTB38;Z	blue	0.62824603	0.67127634	0.28193828	0.0667275	-0.4048558	0.25837843
cg21572731	C3orf52;C3orf52;C3orf52;C3	blue	-0.4695938	-0.54253	0.15439792	0.0297316	0.31632194	-0.1380644
cg19903430	RP11-388K2.1;MACROD2	blue	0.56606362	0.76263444	-0.1706064	0.09900971	-0.3230357	-0.0022701
cg16351766	RP11-284A20.2	blue	0.59395703	0.67109934	-0.1697824	0.13155097	-0.2981642	0.12773939
cg19850080	SLC19A3;SLC19A3;SLC19	blue	-0.4954196	-0.757504	0.18577257	0.04592233	0.5097496	-0.0386094
cg24020826	KCNK9;KCNK9;KCNK9	blue	0.4915618	0.87599875	-0.1420968	0.06186075	-0.6373132	-0.0044701
cg04083226	ARID1B;ARID1B;ARID1B	blue	0.64142972	0.68834055	-0.2585374	0.11201476	-0.3287949	0.06324346
cg15230573	TMEM237;TMEM237;TME	blue	0.56967057	0.67352374	-0.2437166	0.15329114	-0.2430483	0.02036762

cg19098611	UBA2;UBA2;UBA2	blue	0.65341305	0.69829403	0.15516234	0.12313795	-0.4555253	0.37174758
cg17500817	TMEM117;TMEM117	blue	0.47258114	0.74233579	-0.1416842	0.03872736	-0.3712108	-0.0703166
cg09648371	LYG2;LYG2;LYG2;LYG2;L	blue	0.68740446	0.79552798	-0.0556585	0.18163895	-0.4358111	0.19136396
cg20574490	GRHL2;GRHL2;GRHL2;GR	blue	-0.5197072	-0.7214566	0.08167906	0.01119655	0.474349	-0.1632613
cg19831197	NEIL1;NEIL1;NEIL1;NEIL1;	blue	-0.3704323	-0.6544227	0.19866917	0.04364207	0.59072829	0.02710934
cg09767885	CYP8B1;CYP8B1;CCBP2;C	blue	0.46937917	0.63447297	-0.152758	0.02163989	-0.3304839	-0.0766754
cg20986996	TCF21;TCF21	blue	-0.4380752	-0.5920645	0.01100104	0.02888372	0.45655299	-0.0332781
cg07247925	CYP4F22	blue	0.74962056	0.78989173	0.14539528	0.15784789	-0.4115764	0.3171885
cg26606740	NFIB	blue	0.46468859	0.78311718	0.0025547	-0.0131226	-0.584855	-0.0292372
cg08461765	RP11-525K10.2;RP11-	blue	0.40828722	0.69063961	-0.0781817	0.04746033	-0.3115823	-0.0341999
cg04446134	Y_RNA	blue	0.55431177	0.85205733	-0.1251241	0.03876426	-0.5455202	0.05485888
cg05137777	PPFIBP2;PPFIBP2;PPFIBP2	blue	0.65076494	0.72180989	-0.1513268	0.11404665	-0.3019396	0.12092594
cg26534696	LPXN;LPXN;LPXN;LPXN	blue	0.39429624	0.71179407	-0.1395098	-0.018537	-0.5639453	-0.0802767
cg05054096	RP11-351J23.1	blue	0.63399923	0.81854527	-0.0838371	0.06202309	-0.4466062	0.18132455
cg13520547	DNM3-IT1	blue	0.47946168	0.87196943	-0.1056726	-0.0052181	-0.6346488	0.0369574
cg11442052	ARPP21;ARPP21;ARPP21;	blue	0.58536026	0.58138189	0.05455327	-0.0067197	-0.4717686	0.15053689
cg27522801	RP11-	blue	0.6403038	0.65581897	0.20503724	0.10437636	-0.5065855	0.33046128
cg06993050	FNDC9;FNDC9;CYFIP2;CY	blue	0.69231475	0.76143783	0.24022227	0.11398227	-0.4994377	0.27289308
cg15782465	TMEM57	blue	0.68372795	0.76432692	0.28746291	0.15605739	-0.4911087	0.35713342
cg11290922	RP11-	blue	-0.3018958	-0.6242703	0.13524764	0.06191968	0.50614465	0.06784057
cg01451118	RASSF5;EIF2D	blue	0.57692723	0.8090558	0.16480945	-0.0069745	-0.6604138	0.25348215
cg21205358	HSPB8	blue	0.58463121	0.77273657	0.00371684	0.15348086	-0.4262407	0.10749365
cg11248254	ASIC4;ASIC4;AC053503.11	blue	-0.6067411	-0.6566877	0.04182186	-0.0279758	0.54549197	-0.2311155
cg19892727	SLC10A2	blue	0.56040066	0.78656791	-0.0934807	0.04288701	-0.5489761	0.12723023
cg20100292	CYTH1;CYTH1	blue	0.57160438	0.65549144	0.20909983	0.09610892	-0.5565393	0.22096428
cg07273127	MPPED1;MPPED1;MPPED	blue	0.36852629	0.52020104	-0.5647555	0.05788279	-0.2570186	-0.0551244
cg22777932	TRDJ1;AE000661.37	blue	0.5563131	0.60227655	0.02148072	0.08628117	-0.4359747	0.26819797
cg25380126	RP11-174M13.2	blue	0.43218856	0.63532557	0.19218056	0.03478488	-0.386463	0.11960805
cg02495604	HPCAL4;PPIE;PPIE	blue	-0.4690094	-0.7411378	0.10992986	-0.0995083	0.50616369	-0.0740334
cg14595681	RP5-827C21.4	blue	0.62926839	0.62326136	0.31719217	0.13337706	-0.5014936	0.18633279
cg07679173	snoU13	blue	0.6358011	0.69784889	-0.0635613	0.19242404	-0.2984582	0.25103988
cg15464689	CTD-2561F5.1	blue	0.48806343	0.81213846	-0.286015	0.01850155	-0.5763286	0.03744447
cg13461137	RP11-626H12.3;RP11-	blue	0.39673553	0.47262407	0.01504209	-0.0410641	-0.2868146	0.00606239
cg06192890	RP11-215A21.2	blue	0.50930375	0.7633918	-0.1122584	-0.0614868	-0.5017944	0.05239775
cg20340655	BDNF;BDNF;BDNF;BDNF;	blue	-0.5631774	-0.6083604	0.08000916	0.00110279	0.36475596	-0.2903192

cg24713770	RP4-	blue	0.36174125	0.55298087	-0.1880536	0.08408993	-0.3321826	-0.0448738
cg23659056	RP11-511I2.5;FOXD2	blue	0.4251627	0.63795246	-0.062484	0.00659424	-0.3910428	0.09318028
cg17495529	KRT85	blue	0.24784735	0.63799634	0.1348707	0.04179377	-0.4580637	-0.1329086
cg12191789	COL12A1	blue	0.38700705	0.81242014	0.02333041	-0.0111109	-0.6095857	-0.0047543
cg17380131	STAT4;STAT4;STAT4;STA	blue	0.48335192	0.61443038	-0.2434938	0.10673285	-0.2346091	-0.0023631
cg22079098	WWC2	blue	0.5510486	0.83795218	-0.0898141	0.04141333	-0.4923778	0.07726058
cg09087009	AC087069.1;AOAH	blue	0.50361082	0.64248816	-0.282894	-0.0014144	-0.3837254	0.10882973
cg05857597	EDNRA;EDNRA;EDNRA;E	blue	0.60971517	0.71724809	-0.1448924	0.21838778	-0.2619287	0.06550434
cg14845793	RP11-410E4.1	blue	0.72565359	0.6989802	-0.0986634	0.16755012	-0.2017307	0.21837398
cg01832325	HBBP1	blue	0.40751316	0.79107614	0.01342812	0.04992631	-0.5457131	-0.0434281
cg18336281	SLC7A10	blue	-0.5275873	-0.6291908	0.11078476	-0.009094	0.50592871	-0.09408
cg22113063	AP000282.3;AP000282.2	blue	0.69796413	0.69673267	0.12691037	0.21205619	-0.3512326	0.30387075
cg27627026	C4BPB;C4BPB	blue	0.31083115	0.82556954	-0.1409602	-0.1116731	-0.8094318	-0.0463021
cg16450838	ITGA6;ITGA6	blue	0.59334143	0.65897493	-0.1558729	0.09744968	-0.4081044	0.11137491
cg12492301	B3GALNT1;B3GALNT1;B3	blue	0.41305585	0.80649694	-0.2230835	0.02288719	-0.5666275	0.00519701
cg16877681	MAB21L1;NBEA;NBEA;NB	blue	-0.4311417	-0.7355081	0.154943	0.0730022	0.60702435	-0.0547123
cg01150729	ARHGAP28	blue	0.46638201	0.77405612	-0.1337776	0.07136466	-0.5076516	0.00814328
cg25873615	DIO1;YIPF1;YIPF1;YIPF1;	blue	0.36185414	0.61805049	-0.289803	0.04356071	-0.4784033	0.00818328
cg04808896	MYRIP;MYRIP;MYRIP	blue	0.76845372	0.80140347	0.16329186	0.21824295	-0.3299417	0.33051995
cg13549101	IL23R;IL23R	blue	0.30561434	0.72249225	0.12746231	0.08315024	-0.6246584	-0.0475633
cg06879777	ARIH2;ARIH2;ARIH2;ARI	blue	0.6141906	0.59267908	0.3994189	0.15372425	-0.3798044	0.2084695
cg26620745	SCN2B	blue	0.68551164	0.81913009	0.19311828	0.10910849	-0.4615516	0.15344012
cg13173924	Y_RNA;AC010127.4;AC010	blue	0.31975878	0.81500909	-0.2357871	-0.0100489	-0.6552266	-0.1116492
cg14301405	AC091078.1;RP11-	blue	0.50984396	0.85130423	-0.0316202	0.11609032	-0.5385716	0.05645518
cg16989163	SLC39A8;SLC39A8	blue	0.65376827	0.69064317	0.03639355	0.28403739	-0.317276	0.23925183
cg04956653	U7	blue	0.49436146	0.55825134	0.37681164	0.17209006	-0.2386893	0.01029756
cg27598979	SMAD9;SMAD9;SMAD9	blue	0.72159451	0.77771356	-0.0261442	0.14947701	-0.3756002	0.22011768
cg02930200	DNALI1;DNALI1;DNALI1;	blue	-0.4166783	-0.8022622	0.11775844	0.04123957	0.67535513	0.01110616
cg06914270	SS18L1;SS18L1;GTPBP5;G	blue	0.46936351	0.72079856	0.00062467	-0.018653	-0.6046563	0.01714752
cg25053332	RP11-125P18.1	blue	0.3519152	0.58170104	-0.1163126	-0.0006161	-0.4312453	0.00018779
cg26223054	AVIL;AVIL;AVIL;AVIL;TS	blue	0.68597388	0.79863192	0.01441101	0.12698936	-0.4132287	0.09749651
cg07575518	FBLN5;FBLN5	blue	-0.3423156	-0.4230586	0.13583767	0.10324012	0.29516048	0.00804024
cg01135834	RYR3	blue	0.65873986	0.68954909	-0.1202664	0.12633199	-0.2523152	0.15541179
cg24189734	CTD-2195M18.1;CTD-	blue	0.41377489	0.6147938	-0.1995962	0.09998712	-0.2120812	0.02376728
cg23387597	ITPRIP;ITPRIP	blue	0.59316083	0.60915035	-0.0624876	0.0652719	-0.4912338	0.18188244

cg16968681	RN5S462	blue	0.38186101	0.77833438	0.04210936	-0.060117	-0.7041954	-0.0170364
cg21922303	TCEB2P2	blue	0.54952073	0.84517701	0.11081352	0.06756192	-0.5716144	0.10046036
cg17793608	LCP1	blue	0.45097872	0.80700722	-0.1999389	-0.0297015	-0.6957397	0.00439696
cg16482631	C3orf17;C3orf17;C3orf17;C3	blue	0.76141589	0.80020622	0.00208979	0.23444624	-0.4008434	0.26106034
cg14808135	RP11-520D19.1	blue	0.53615039	0.84907302	-0.1023146	0.11045491	-0.5155137	0.07213233
cg00919407	LGI1;LGI1;LGI1;LGI1	blue	0.46428985	0.6879229	0.18369261	0.02532228	-0.4511528	0.13595363
cg27037944	SNORA26	blue	0.68798998	0.72759505	-0.1262668	0.10916736	-0.3217346	0.15647599
cg15937977	RP11-35L17.3	blue	0.32923057	0.75637454	-0.2180403	-0.0281346	-0.6221126	-0.0498264
cg18483704	C4orf3	blue	-0.5533207	-0.6189432	0.08089506	-0.097757	0.42658581	-0.1516633
cg14823876	TEAD1	blue	0.73200875	0.70092406	-0.0516172	0.16809477	-0.267724	0.21589074
cg19336937	RP11-	blue	0.440071	0.879258	-0.059778	0.03402384	-0.6634836	0.05834591
cg03998317	AC006159.3;AC006159.3	blue	0.61311316	0.78544403	0.23296442	0.1864612	-0.4765537	0.21384178
cg15230179	ST3GAL6;ST3GAL6;ST3GA	blue	0.47380641	0.83092264	0.13016936	0.03497348	-0.6215579	0.05352934
cg05439047	NKD2;NKD2;NKD2	blue	0.3951579	0.58110331	-0.0086998	0.09008333	-0.3514976	0.05005418
cg18022224	HOXD12;HOXD12	blue	-0.5333311	-0.6557	0.19297903	0.00561462	0.40404427	-0.0226022
cg14521149	SNORD63	blue	0.49837817	0.86530326	0.00352842	0.05806804	-0.5697752	0.08454891
cg01656641	U6;KIAA1211;KIAA1211;K	blue	0.57708918	0.71237894	0.29915214	0.04938938	-0.5966962	0.21468542
cg18839750	PCDHGA3	blue	-0.2875006	-0.457435	0.0632864	0.08736752	0.38572085	0.12660879
cg02650507	RP11-	blue	0.30666171	0.70911779	-0.378515	-0.080471	-0.5617975	-0.1883062
cg25986722	BDKRB1;RP11-404P21.3	blue	0.62181552	0.79752734	-0.0578834	0.13784392	-0.4221786	0.15924148
cg24509300	PPT2;PPT2;PRRT1;PRRT1;P	blue	-0.4668654	-0.6384426	0.16020494	0.12791703	0.52835873	-0.1121655
cg00516067	COL9A3;COL9A3;COL9A3	blue	-0.4521825	-0.5363261	0.14002316	0.01208482	0.3554838	-0.086736
cg00565348	NRN1	blue	-0.6347446	-0.744108	0.08316061	-0.0384765	0.49059049	-0.2201166
cg23207527	RBM24;RBM24	blue	0.69791152	0.84440998	-0.0285218	0.12555191	-0.4119344	0.16175865
cg26487753	RTBDN;RTBDN	blue	-0.538769	-0.7115317	0.15928559	0.024413	0.54080309	-0.098559
cg04211253	Y_RNA	blue	0.5809487	0.76994225	0.07823886	0.13082106	-0.3806913	0.11397274
cg05218696	SNORD113-	blue	0.5258183	0.89892628	-0.0816335	0.07146971	-0.5948116	-0.0158802
cg09473613	HMGCL;HMGCL;HMGCL;	blue	0.29437053	0.79891545	-0.0861927	-0.0960518	-0.7337751	-0.0487929
cg19921293	WNT7B;WNT7B	blue	-0.4525986	-0.6090633	0.10652328	-0.0333974	0.35700377	0.00754862
cg27441721	Y_RNA	blue	0.57344466	0.6941322	-0.0458718	0.17471865	-0.3910413	0.23289891
cg19580219	RECQL;RECQL;RECQL;RE	blue	0.4910781	0.66634784	-0.1237588	0.02313505	-0.4119744	0.07990304
cg06334363	RP11-	blue	0.36818954	0.45853945	-0.1040448	-0.0115846	-0.2946531	-0.0081953
cg01032298	RP11-490N5.2	blue	0.39676873	0.50747209	-0.3305171	0.06252613	-0.1418642	-0.0557585
cg12593210	RSPO4;RSPO4	blue	-0.5516272	-0.7442035	0.16895116	-0.0128428	0.56934494	-0.0528758
cg26659665	IQCJ-SCHIP1;IQCJ-	blue	0.60451197	0.78998246	-0.069221	0.11405454	-0.420064	0.100185

cg02023469	TMEM132A;TMEM132A;T	blue	-0.4450784	-0.7957773	0.06048044	0.00657638	0.60476589	-0.0010142
cg11904056	NR2F1;RP11-65F13.2	blue	-0.3897191	-0.5956946	0.35747289	0.06346854	0.44634191	-0.0398755
cg08223291	RP11-10N16.3;RP11-	blue	-0.2123144	-0.5301527	0.09191522	0.03227882	0.52286057	0.14993167
cg12796285	CLRN1;CLRN1-	blue	0.44066598	0.87847144	-0.0695208	-0.0012713	-0.6891655	0.01121851
cg17214902	ADAM8;ADAM8;ADAM8;	blue	0.35363231	0.60709425	-0.0720082	0.00509538	-0.4077805	0.06785038
cg23774356	GSC2	blue	-0.6290955	-0.76909	0.09179801	-0.0685666	0.4747302	-0.0920309
cg00598301	MCCC1;MCCC1;MCCC1;M	blue	0.4534879	0.8297896	0.01977972	-0.0097082	-0.7785296	0.12455838
cg21983151	MPLKIP	blue	-0.4945502	-0.5594617	0.072604	0.12150129	0.4576451	-0.0827354
cg14296729	AC011500.1	blue	0.74124758	0.71811334	-0.0117599	0.2524247	-0.23999	0.28979819
cg11362681	snoU109;snoU109	blue	0.6788144	0.8074054	0.18317445	0.18708352	-0.4315521	0.25717728
cg08193363	FRY;FRY;FRY-AS1	blue	-0.6431024	-0.7338988	0.04546865	-0.0859986	0.40181325	-0.1485472
cg17039220	OSBP2;OSBP2;OSBP2;OSB	blue	0.72582501	0.72606613	0.07496673	0.09847535	-0.4716231	0.28743526
cg03539825	AC005284.1	blue	0.57747882	0.63215509	-0.0694616	0.19118741	-0.2044469	0.16082151
cg23710218	MSC;MSC;MSC;RP11-	blue	-0.4586936	-0.6175859	0.068809	0.02448366	0.51590049	-0.0814429
cg00504086	FGF14-AS1;FGF14-	blue	0.6575561	0.80980674	0.00377837	0.20647526	-0.3803487	0.18497402
cg04176072	RFX4;RFX4;RFX4	blue	-0.5947067	-0.7358438	0.09204737	-0.0345371	0.50476982	-0.1447621
cg00095688	PRKCZ;PRKCZ;PRKCZ	blue	0.44395378	0.82770165	-0.0963589	-0.0725073	-0.7789629	0.02782554
cg02124498	HHLA2;HHLA2;HHLA2;H	blue	0.70264066	0.75326928	0.13245524	0.08248162	-0.4048541	0.25129211
cg11415021	EGF	blue	0.62714375	0.77507674	0.0662493	0.18011906	-0.4182067	0.13297314
cg21517629	U6	blue	0.62277939	0.85628175	0.02647893	0.16768004	-0.4779397	0.1187628
cg04259408	AC005703.3	blue	0.62493091	0.81018187	0.15140466	0.06665049	-0.5226369	0.1411644
cg24165509	CTD-2023N9.1	blue	0.70702687	0.70845915	0.14501465	0.13594015	-0.2905365	0.24640572
cg17515753	AC002456.2;AC002456.2;A	blue	-0.4560053	-0.7473845	0.24878222	0.03366189	0.59820118	-0.0155122
cg19974097	RP11-305P14.1;RP11-	blue	0.51337523	0.73283375	-0.3292817	0.13485833	-0.3605816	0.02273115
cg03760101	MASP1;MASP1;MASP1;M	blue	0.47351748	0.66904198	-0.2232576	0.11681916	-0.3345882	-0.0206594
cg09787166	TMEM229A;RP5-921G16.1	blue	-0.4609561	-0.6455561	0.12952286	0.05040991	0.44979343	-0.0209983
cg11227141	PRKCZ;PRKCZ	blue	0.52033804	0.70158114	-0.2636008	0.12738906	-0.3593058	0.08996165
cg13257390	AGBL2;AGBL2	blue	0.50048526	0.92161624	-0.0476845	-0.0198728	-0.6309488	0.0374449
cg20810198	PCK1;PCK1;PCK1;PCK1;P	blue	0.41023465	0.46224709	-0.1914259	0.04970913	-0.1879591	0.11412308
cg23384863	LRPPRC	blue	0.42328959	0.79817046	0.22198773	-0.026713	-0.7615153	0.13787085
cg15865637	PABPC1L;PABPC1L;PABP	blue	0.49569246	0.86249168	-0.0001202	0.11815082	-0.5376113	0.07499956
cg11979793	AL132709.8;AL132709.8;AL	blue	0.41406814	0.84158507	-0.1771394	0.01742013	-0.5901529	-0.1022868
cg16262392	CRTC2;CRTC2;CRTC2;CRT	blue	0.62022109	0.72607213	-0.082013	0.14016569	-0.311712	0.18430089
cg11790211	SHANK2	blue	0.56473998	0.80986399	0.09751546	0.14737414	-0.5230872	0.07522202
cg02616710	ACER2;ACER2	blue	-0.4048963	-0.5010678	0.06002967	0.10853654	0.41476951	-0.0867605

cg06330431	RHPN1;RHPN1	blue	0.34310361	0.76692699	0.05412458	-0.0297378	-0.7563279	0.08856498
cg23726358	AC068535.4;AC013402.2;A	blue	0.51778808	0.81974061	-0.1272189	0.08865411	-0.4955307	-0.0400438
cg19561129	Y_RNA;CYTH3	blue	0.65886314	0.87648991	0.07633673	0.16381804	-0.5113825	0.22731645
cg13298963	ASIC2	blue	-0.5277236	-0.6624323	0.07496817	-0.0294014	0.40452142	-0.2493549
cg14561361	ASAP1;ASAP1	blue	0.44686873	0.87275771	-0.061949	0.01541787	-0.7174569	0.08846051
cg09575601	RP1-302D9.3	blue	0.47091242	0.54227461	0.06189242	0.17799936	-0.2267389	0.17140396
cg08131119	GLB1L2;GLB1L2;GLB1L2	blue	0.50638489	0.67982944	-0.289799	0.15529805	-0.3117723	0.03023296
cg05346878	TACSTD2	blue	-0.5022275	-0.6702228	-0.01902	0.06291118	0.55063517	-0.0885199
cg03547218	HOXA-AS3;HOXA6;HOXA-	blue	-0.550566	-0.6548146	0.15348334	-0.0184068	0.37925151	-0.2128531
cg06819792	U91319.1;U91319.1	blue	0.72677745	0.82508512	-0.080139	0.1276542	-0.3717429	0.14639932
cg09293488	AL132709.5;AL132709.5;AL	blue	0.58611858	0.75670311	-0.2547905	0.10020238	-0.3258415	0.02161895
cg06811361	MIRLET7A2	blue	0.69633594	0.74029493	0.12409102	0.13810566	-0.3252978	0.23408169
cg15268510	CYHR1;CYHR1;CYHR1;CY	blue	0.60949022	0.75631802	0.04311747	0.17618453	-0.3724356	0.14136477
cg04316575	PPP2R2C	blue	0.66638108	0.79378095	-0.0269559	0.14432542	-0.4111535	0.15303307
cg07098762	AC093585.6	blue	0.55367699	0.62944774	0.01928447	0.05477226	-0.3881703	0.15320219
cg15008519	RP11-452D21.2;RP11-	blue	0.44982838	0.83854436	-0.0946887	0.06959777	-0.5532634	0.03315842
cg09099500	MIR942;TTF2	blue	0.45058194	0.74684793	-0.1900471	-0.0999545	-0.6651954	0.06037814
cg26840884	TGIF1;TGIF1;TGIF1;TGIF1	blue	0.32803412	0.82883387	-0.1901801	-0.1510907	-0.7771024	-0.0991259
cg05093169	PHOX2A;PHOX2A	blue	-0.4662433	-0.8175801	0.24028907	0.02008545	0.58527665	-0.0070035
cg08436588	UACA;UACA;UACA	blue	0.59429474	0.87008342	0.04677935	0.10528803	-0.5528908	0.12361258
cg09084461	FLT1	blue	0.65766631	0.84402998	0.10913712	0.15865735	-0.4372884	0.22147809
cg18279486	SSPO;SSPO;SSPO	blue	0.57305616	0.79666858	0.17177432	0.13937618	-0.4589673	0.19258457
cg02466728	RP11-555G19.1;RP11-	blue	0.49411297	0.80631037	-0.0802274	0.05772872	-0.4858406	0.04557771
cg10379084	AP001623.1;TMPRSS3	blue	0.65115798	0.78947694	-0.0789809	0.2087921	-0.3295068	0.15211388
cg12366423	EGFLAM;EGFLAM;EGFLA	blue	0.60889847	0.80230118	-0.2116246	0.08728636	-0.4393528	0.12065086
cg13995230	MIR654;MIR376C	blue	0.26200054	0.81936652	-0.0863738	-0.1301473	-0.7507746	-0.0792116
cg24747960	SLC22A2;SLC22A2;SLC22	blue	0.48707631	0.84611469	-0.1865602	0.04045132	-0.6027655	0.0476811
cg27569575	RP11-542K23.10;RP11-	blue	0.41647367	0.66175431	-0.2111942	0.00512276	-0.4787141	0.06003413
cg08979543	DLGAP1;DLGAP1;DLGAP	blue	0.59261775	0.79408301	0.01577805	0.15787143	-0.5175538	0.16980664
cg23582561	U3	blue	0.7334915	0.69222412	0.06661994	0.1913238	-0.2317247	0.30050633
cg12217308	AL035697.1	blue	0.270242	0.59820604	-0.279209	0.00346181	-0.3920002	-0.1824859
cg26259171	FUT1;FUT1	blue	-0.4770471	-0.5880617	0.11479107	-0.0173012	0.46474362	-0.1291481
cg24282887	RP11-734K21.3	blue	0.62869863	0.86171746	-0.0646046	0.11239505	-0.5704851	0.19959625
cg01649465	HTR4-	blue	0.5352639	0.82926761	-0.0126472	-0.005205	-0.7264351	0.14960299
cg23997436	RP11-563P16.1	blue	0.64564562	0.80338229	0.0384138	0.15022147	-0.4652874	0.27396258

cg23536555	MYBPC1;MYBPC1;MYBP	blue	0.78179645	0.81323134	0.18094183	0.16315543	-0.3727671	0.2976982
cg02949244	AC012175.1;RP11-420N3.2	blue	0.50163225	0.70261597	-0.3252883	0.16173151	-0.3404156	0.02464019
cg03460355	RP11-	blue	0.44452639	0.84457402	-0.0882662	0.00034571	-0.7821745	0.07062981
cg01534464	GPR39	blue	0.69691929	0.67873946	0.02255124	0.19950889	-0.1886664	0.25076809
cg05787537	RP11-	blue	0.46051069	0.64394157	0.00531774	0.05812283	-0.332907	0.10955243
cg17188046	T;T;T;T	blue	-0.4397229	-0.7596377	0.14762627	0.00666108	0.55093497	0.00756822
cg19138043	NCS1	blue	0.62844787	0.8593258	-0.0211976	0.13188782	-0.4794123	0.10843783
cg00499248	LY6K;LY6K;LY6K;LY6K	blue	0.38832163	0.8005562	-0.2326613	-0.0140275	-0.5524624	-0.086422
cg10193817	CADM1;CADM1;CADM1;C	blue	-0.5583638	-0.6674208	0.114684	-0.0724063	0.44303397	-0.1462085
cg21796269	CTD-2331D11.4	blue	0.33351179	0.668364	-0.3598003	-0.059751	-0.4297668	-0.2018573
cg23374807	RN5S135	blue	0.52983007	0.62479972	0.31540075	0.10194332	-0.3449519	0.0386059
cg03309648	PPM1K;PPM1K;PPM1K;PP	blue	0.76606827	0.73732933	-0.0975788	0.17327509	-0.3532162	0.21908349
cg21434457	RP11-63N8.3	blue	0.73835097	0.8102208	0.11023815	0.19887205	-0.438815	0.28184828
cg05836659	RP11-	blue	0.42067579	0.58514998	-0.0484492	-0.0612356	-0.4303723	0.06636104
cg10249997	ADAMTS15	blue	-0.3048368	-0.4459618	0.0557438	0.16821189	0.39045369	0.02612287
cg24304483	LINC00536	blue	0.7503164	0.79825174	0.1169958	0.23453252	-0.3171713	0.26936018
cg05177629	SEMG1	blue	0.24546207	0.73882511	-0.2627992	-0.111829	-0.6673755	-0.1473622
cg14846609	RP11-386D6.1;CNGB3	blue	0.32718943	0.73651745	0.07377076	-0.024726	-0.5232212	-0.064652
cg01688263	RP13-379L11.2	blue	0.57571774	0.68344567	-0.0361762	0.16210079	-0.2866333	0.28502176
cg01716593	RP11-115J16.2;RP11-	blue	0.5854965	0.78441557	-0.0002649	0.16941875	-0.34273	0.07938825
cg13520770	OSBPL5;OSBPL5;OSBPL5	blue	0.65170409	0.69763836	0.13096995	0.11166701	-0.3964694	0.16514807
cg02500533	COL12A1;COL12A1	blue	0.55664756	0.73112049	-0.2613102	0.19370009	-0.2474878	0.01820229
cg18164425	NR1H4;NR1H4	blue	0.55776045	0.86971397	0.02495673	0.0792427	-0.570575	0.09779472
cg24202274	METTL13;METTL13;METT	blue	0.41464049	0.46441451	0.03559582	0.03366122	-0.2654767	0.20618796
cg08655877	RP11-71H17.7	blue	0.58087966	0.78129383	0.01237942	0.12401006	-0.4027409	0.16606934
cg06391536	MEIS3	blue	-0.378079	-0.4039577	0.04616903	-0.1369656	0.32456924	-0.1949647
cg02839019	CPEB4	blue	0.49979282	0.58249244	-0.3426593	0.04188578	-0.1971119	0.00144776
cg05914242	PRSS35;PRSS35	blue	-0.5356154	-0.6818898	-0.0345173	-0.038343	0.44990028	-0.0934932
cg22865831	LUZP1;LUZP1;LUZP1;LUZ	blue	-0.5751043	-0.6603834	0.05720729	-0.1265361	0.31393601	-0.0870811
cg21873448	FZD3	blue	0.6429023	0.80293169	0.08583618	0.1417337	-0.4704277	0.15507414
cg07999953	GJB3;GJB3;GJB3;RP1-	blue	0.38409635	0.84007324	0.06430216	-0.057597	-0.7386847	0.16678152
cg05374451	CTD-2561B21.9;CTD-	blue	-0.5682371	-0.5502154	0.15526046	-0.0082817	0.32278762	-0.0633504
cg09962842	DEFB124;REM1	blue	-0.3933506	-0.7236759	0.1561082	0.10760555	0.6602786	0.02258228
cg27162122	U7;WWC2	blue	0.50372298	0.66766502	-0.4591649	0.08117393	-0.3097634	-0.0367498
cg11638798	AC074112.1;AC074112.1	blue	0.21481343	0.39775628	-0.1225026	-0.0308702	-0.2711633	-0.134576

cg21557493	RP11-13A1.1	blue	0.50738055	0.87325114	-0.181274	0.04905159	-0.5337508	0.01371822
cg00281852	NLRP1	blue	0.65723341	0.82923954	-0.0090332	0.16841902	-0.4540871	0.18587118
cg06693222	RP11-345L23.1	blue	0.66855892	0.69221361	-0.0284771	0.15293864	-0.2607235	0.17240806
cg04579885	RP11-490E15.2	blue	0.48226176	0.85894445	0.05153105	-0.0129806	-0.7882395	0.10031473
cg25840094	CFHR5;CFHR5	blue	0.33818876	0.76507236	-0.0193823	-0.0972648	-0.5765104	-0.0255691
cg12803397	RP4-777D9.2;RP4-	blue	0.53767004	0.808828	-0.2138634	0.08059899	-0.4365387	0.04044548
cg23759926	CSRP3;RP11-428C19.4	blue	0.73493461	0.736871	0.14620291	0.21965872	-0.3215389	0.2612815
cg05368724	FAM178B;FAM178B;FAM1	blue	0.44243394	0.60173003	0.28078554	0.11727317	-0.464247	0.06435717
cg23347160	ARPP21-	blue	0.46693941	0.8462271	-0.1716642	-0.008794	-0.5580658	0.03336544
cg04872373	GTF2A1L;GTF2A1L;GTF2A	blue	0.72860182	0.71553159	0.10887828	0.17150479	-0.2774415	0.24164898
cg14311957	U7	blue	0.44399341	0.70174692	-0.2335831	0.0851063	-0.3850898	0.04091267
cg08967215	AC022098.2	blue	0.60058131	0.8277744	-0.1519574	0.04727541	-0.5172646	0.14532799
cg27298817	RP11-293F5.1;RP11-293F5.1	blue	0.56207347	0.88583376	-0.0227489	0.06456067	-0.6037494	0.27593369
cg09486367	CA10;CA10;CA10	blue	0.61828737	0.66684018	-0.2789569	0.1430207	-0.2201872	0.22272482
cg10889138	FAM110C;FAM110C	blue	0.60049416	0.72157938	-0.0712929	0.14432669	-0.3820239	0.03832448
cg27139805	DMKN;DMKN;DMKN;DM	blue	0.42263111	0.55711469	0.29225579	-0.0408127	-0.4114481	0.13021018
cg01542565	RNF144A;RNF144A;RNF14	blue	0.60015367	0.70264848	-0.0513742	-0.0036197	-0.3915667	0.26468121
cg25248319	GOLPH3;GOLPH3;GOLPH	blue	0.30599597	0.73818957	0.02267767	-0.0878021	-0.8004831	-0.0510482
cg21748003	RP11-89N17.1	blue	0.67868498	0.76772316	-0.0849958	0.21632707	-0.3817754	0.20084763
cg16432061	MPZ;MPZ;MPZ;MPZ;MPZ	blue	-0.4564315	-0.4827647	0.1658182	-0.0550338	0.34399416	-0.1830885
cg11842783	C3orf32;C3orf32;C3orf32;C3	blue	0.38588604	0.65932447	-0.1527692	-0.0247336	-0.5107604	-0.0546787
cg00653987	RP11-298O21.3;RP11-	blue	0.63565219	0.74638829	-0.0543229	0.1600653	-0.3617427	0.09113665
cg22896309	NOVA1;NOVA1;NOVA1;N	blue	-0.2855578	-0.4361861	0.29727349	0.18842166	0.35953623	0.10499311
cg19216791	PLAC2	blue	-0.2691199	-0.4185307	0.33232537	0.1804592	0.35695777	0.03178993
cg11423580	RP11-49O14.2;C9orf3	blue	0.5904224	0.81333443	-0.0151272	0.12587336	-0.5341617	0.13511588
cg17047109	RP1-17K7.3;RCN1	blue	0.48758644	0.82328297	-0.2426647	-0.0134207	-0.572169	0.09532127
cg10621706	AC006305.1	blue	0.35986509	0.84698517	-0.2100812	0.01675331	-0.6029695	-0.0515242
cg14585353	ZPBP2	blue	0.39891008	0.83887441	-0.098297	-0.0565354	-0.7589973	0.0621677
cg15847272	C2;C2;C2;CFB;CFB;CFB;CF	blue	0.59905493	0.65136631	0.11942826	0.0809238	-0.2710971	0.11353878
cg22419649	EIF2B5-AS1	blue	0.73965461	0.79957664	0.00527201	0.18721179	-0.3349778	0.21577417
cg18095041	NEU4;NEU4;NEU4;NEU4;A	blue	0.41328258	0.37794322	-0.2551713	0.23984595	-0.0023145	-2.04E-05
cg12081233	BAIAP2;BAIAP2;BAIAP2	blue	0.33024307	0.8078113	-0.054968	-0.0513382	-0.6172661	-0.0427863
cg13202877	ZNF323;ZNF323;ZNF323;Z	blue	-0.4356255	-0.5549101	0.32649811	0.06591982	0.44345555	-0.0631401
cg10114358	RP5-916O11.2;LDLRAD3	blue	0.3970323	0.89916773	-0.0503722	0.00082827	-0.705804	0.00211278
cg23750365	HLA-DPB1;HLA-	blue	0.54179759	0.80886846	-0.1281558	0.04153272	-0.5648146	0.0577182

cg24323597	KALRN;KALRN;KALRN;K	blue	0.61593407	0.81507963	-0.0683572	0.13226737	-0.4697872	0.07393121
cg23682556	RP11-285B24.1	blue	0.40683649	0.79213617	0.14202774	-0.0297257	-0.7555352	0.10720435
cg12179826	BARX1	blue	-0.4145207	-0.4726824	0.13811239	-0.0552368	0.32568021	-0.0391196
cg10773526	INPP4B;INPP4B;INPP4B	blue	0.5781482	0.79557623	0.202787	0.07934316	-0.5594693	0.18408304
cg06912413	MIR595	blue	0.66919124	0.70783068	-0.0398361	0.2367975	-0.2307601	0.1411111
cg09141093	CTD-2377D24.8	blue	0.63467804	0.84698253	0.16822675	0.12442067	-0.5277947	0.22789293
cg01394116	RBM22;RBM22;RBM22;RB	blue	0.2823643	0.74310922	-0.0228482	-0.0787818	-0.7381316	-0.0481231
cg02249355	Y_RNA	blue	0.58274735	0.66896281	-0.1648874	0.03957942	-0.3883698	0.09273843
cg05624226	BTNL8;BTNL8;BTNL8;BTN	blue	0.49769661	0.7754169	-0.1858759	0.00899317	-0.4660379	0.01419835
cg18587783	TIMP4;SYN2;SYN2;SYN2	blue	0.64388089	0.69673454	-0.1654605	0.15765968	-0.2690286	0.16287042
cg20568433	7SK	blue	0.38737206	0.80426681	-0.2507535	-0.1171294	-0.5902172	-0.0659722
cg14257335	KLHL22-	blue	0.68843738	0.78264176	-0.0493476	0.18283838	-0.3764403	0.22167612
cg20382995	RP11-438E5.1;RP11-	blue	0.48466083	0.87223981	0.07557199	0.08126576	-0.5942245	0.10284008
cg07902369	OR6K6	blue	0.57080449	0.82481454	-0.0525771	0.07090384	-0.5447761	0.0861259
cg09905191	AC108868.5	blue	0.60308239	0.85371485	0.03456814	0.10182902	-0.4694093	0.11922805
cg22719826	KIAA1324L	blue	0.48057849	0.85614867	-0.1188244	0.08317061	-0.6567539	0.01273866
cg08151204	TAP1;PSMB9;PSMB9	blue	0.61762333	0.68723735	0.00419573	0.16832657	-0.3822355	0.25993983
cg12525656	PHYHIP	blue	0.6390644	0.68420845	-0.1261847	0.10864187	-0.275583	0.09661822
cg04287484	VSTM5	blue	0.71737875	0.68499947	-0.1379135	0.16124419	-0.2058744	0.31620338
cg26138257	AC069394.1	blue	0.32927752	0.82332222	-0.1739442	-0.0047237	-0.6704274	-0.0911296
cg17500664	SH3YL1;SH3YL1;SH3YL1;	blue	0.47439135	0.61840448	-0.0122436	0.00666617	-0.4711688	0.08054038
cg09608131	OR1B1;RP11-64P14.7;RP11-	blue	0.52754374	0.84139092	0.01187379	0.05663235	-0.5012254	0.04921955
cg19219624	TAC1	blue	-0.5277503	-0.6996495	-0.0207027	-0.0224751	0.44997202	-0.0558857
cg01167924	OR13A1;OR13A1;OR13A1;	blue	0.6840971	0.69058738	0.17301674	0.17197409	-0.3282108	0.21903996
cg10696780	BBOX1;BBOX1;BBOX1;BB	blue	0.4784011	0.80533722	0.03284911	0.05168226	-0.5070084	0.03229925
cg04262640	TAS2R16	blue	0.39643854	0.8275582	-0.2196326	0.07039346	-0.5846724	-0.1485854
cg06714088	DUSP27	blue	0.5392981	0.89182077	0.01824729	0.07398662	-0.6080574	0.07310435
cg01895239	LINC00570;LINC00570;LIN	blue	0.57851558	0.88638499	-0.0794627	0.03516438	-0.5392273	0.12757161
cg21768342	AC104623.2;AC104623.2	blue	0.4094004	0.88442953	-0.1282363	-0.0387145	-0.6779273	-0.0716749
cg23413085	PTPN21	blue	0.32108286	0.38581023	0.05804655	0.16158103	-0.1257554	-0.0241825
cg14193459	AC005152.3;AC005152.3	blue	0.41304395	0.62707127	0.30563714	0.03833973	-0.4369693	-0.0315854
cg05670354	U1	blue	0.4675845	0.85450395	-0.1409247	0.08073141	-0.567639	0.07924711
cg24123518	ISLR2;RP11-	blue	-0.3629117	-0.3936937	0.07196256	0.05987984	0.31918346	-0.1062405
cg08259083	PLK3;PLK3;PLK3;PLK3;PL	blue	0.53118368	0.6740471	0.0308216	0.09847154	-0.4631135	0.0462854
cg09024606	RER1;MORN1;MORN1;RE	blue	0.46333275	0.64577268	-0.2356106	0.11399146	-0.4342342	-0.0485911

cg11069071	MIR154;MIR496	blue	0.36733979	0.78847987	0.21038945	0.0072076	-0.6679214	0.04165268
cg21993243	U6;CHL1;CHL1;CHL1;CHL	blue	0.53581719	0.80876962	0.04850244	0.08520468	-0.4499607	0.0227846
cg14097889	DMXL1;CTB-	blue	0.69850629	0.82305673	0.00483951	0.21217499	-0.3611506	0.1680881
cg11414268	RP11-141E13.1	blue	0.31861542	0.8119795	-0.199558	0.03971544	-0.5859413	-0.0624603
cg20015240	GRID1	blue	0.4099606	0.51433002	0.13719229	0.00553029	-0.2317714	0.0925229
cg10054857	AC100848.1;LINC00305;LIN	blue	0.68428296	0.736006	0.19770613	0.25163187	-0.2873426	0.14212697
cg25369211	RPL13AP17;RPL13AP17;RP	blue	0.75159169	0.71922656	-0.0146555	0.19394839	-0.2243556	0.2151802
cg15979674	AC013472.3;MAPRE3;MAP	blue	0.7557286	0.73567008	-0.0751469	0.27840195	-0.2771142	0.31698976
cg26739532	RP11-	blue	-0.6437724	-0.8175302	0.15498236	-0.125062	0.51858839	-0.1816742
cg01121711	ITGB5;ITGB5;ITGB5	blue	0.44161496	0.80639866	-0.1392105	-0.0204322	-0.5446142	0.06609338
cg25468450	CIRH1A;CHTF8;CHTF8;CH	blue	0.3083095	0.81734467	-0.0251814	0.00184934	-0.7536755	-0.0540909
cg07930874	SLAIN1;SLAIN1;SLAIN1;S	blue	0.39400053	0.77062967	-0.2083731	0.01647751	-0.6024087	-0.0618469
cg03003052	AC017104.2	blue	0.54400166	0.80751052	-0.0740135	0.06426558	-0.534182	0.09400503
cg02139186	TMEM40	blue	0.58682827	0.79327591	0.16581307	0.11704877	-0.5117938	0.25763951
cg14358411	RN5S31	blue	0.70367827	0.73293212	-0.1540653	0.15646574	-0.2320941	0.11809282
cg20605413	PCK1;PCK1;PCK1;PCK1;P	blue	0.56105817	0.73154229	-0.0791529	0.16093964	-0.332998	0.13002673
cg02203055	SPOCK1;SPOCK1;SPOCK1	blue	0.59930065	0.81015425	-0.1600239	0.15818666	-0.3543669	0.07953007
cg19025234	THBS4;THBS4;CTD-	blue	-0.3524797	-0.6344219	0.14429349	0.15693403	0.59683239	0.03403218
cg02948067	IZUMO2;IZUMO2;IZUMO2	blue	0.5715167	0.71509104	0.04357783	0.20100922	-0.3274193	0.13942229
cg10965779	RP11-249C24.10	blue	0.4175515	0.64705026	0.08286386	0.0357157	-0.4879235	0.1611338
cg21902039	DNASE2B	blue	0.58691508	0.75641971	0.23585415	0.15595652	-0.3721998	0.1436384
cg07285403	BCL2L14;BCL2L14;BCL2L1	blue	0.61095751	0.81755256	-0.1237858	0.11939887	-0.4391031	0.03733202
cg06138526	RP11-1018N14.2;RP11-	blue	0.62819827	0.72670232	0.04898818	0.11553509	-0.3401512	0.19972788
cg02125910	PCDHB3	blue	-0.3848479	-0.5636597	0.21690742	-0.0527268	0.28310213	0.01505285
cg19849728	RP11-380D23.2;RP11-	blue	-0.4488337	-0.4564133	0.20686349	0.06178501	0.31919177	-0.1152721
cg24410811	NMBR	blue	0.28594276	0.79179785	0.01180937	-0.1018547	-0.8222664	-0.0224928
cg25039753	F13B	blue	0.64637363	0.76447071	-0.0006656	0.11920249	-0.4423639	0.18509185
cg02345584	ITGA10	blue	0.65907041	0.71449066	-0.1520846	0.13480472	-0.301057	0.15050151
cg12280113	NLRP9	blue	0.42634216	0.7057307	-0.0364389	0.07581679	-0.4313202	0.08544326
cg02325544	RN5S205	blue	0.61360839	0.73526786	0.03316323	0.08659067	-0.266842	0.05065364
cg21523928	U1;RP11-227F19.1	blue	0.44471541	0.76561112	-0.1468108	0.0494216	-0.4619938	-0.0244303
cg26795073	AC011648.1	blue	0.59829493	0.71310719	-0.0952593	0.04939597	-0.4609165	0.13236572
cg05148947	IGSF22;IGSF22;RP11-	blue	-0.3038204	-0.6646792	0.11845136	0.05136155	0.50347773	0.06014865
cg05804641	ZNF541	blue	0.56735132	0.66514878	0.33701194	0.09613972	-0.4009397	0.17098039
cg10954182	NFYB;NFYB;NFYB;NFYB	blue	-0.5633068	-0.7114701	0.06379772	0.00873423	0.51653718	-0.204631

cg05609436	CLVS1	blue	0.62742384	0.78928407	-0.0369252	0.19795571	-0.3733475	0.10744063
cg15892117	KCTD14;KCTD14	blue	0.58647405	0.89010246	-0.0580903	0.0755074	-0.5612536	0.15375589
cg23860422	C4orf49;NDUFC1	blue	-0.4133153	-0.6386424	0.33062325	0.11042291	0.50770719	-0.0181669
cg01774630	C20orf187;C20orf187	blue	0.59542337	0.80698871	0.23471805	0.09630292	-0.5512597	0.29325582
cg08784129	PAX6;PAX6;PAX6;PAX6;P	blue	-0.482212	-0.6229792	0.14928279	0.02618577	0.39839276	-0.0493991
cg07296661	SCN9A;SCN9A	blue	0.67919945	0.73744833	0.235197	0.25804719	-0.3621507	0.31101997
cg04574135	NIPAL1;NIPAL1;NFXL1	blue	-0.4266662	-0.3869719	0.11026174	0.13087951	0.34056582	0.00472797
cg22084165	RP11-587P21.3	blue	0.67744347	0.67423607	0.12713938	0.09897061	-0.452894	0.40219553
cg23478549	TEAD1	blue	0.59290793	0.68574632	-0.1578404	0.11073643	-0.207903	0.18372562
cg06037770	TEKT3;TEKT3	blue	0.513474	0.89099639	0.0143997	-0.0196898	-0.6527987	0.09969391
cg21353232	SEZ6L;SEZ6L;SEZ6L;SEZ6L	blue	-0.3945301	-0.3895537	0.03500312	-0.0054535	0.26979068	-0.13954
cg10990080	NT5E	blue	0.71260144	0.69619684	0.04705534	0.26954887	-0.3000065	0.25459337
cg12683111	RP11-12M5.1	blue	0.70827156	0.80581116	-0.0507053	0.15973841	-0.4360759	0.20388615
cg19532892	CNTN1;CNTN1;CNTN1;CN	blue	0.46483267	0.82885832	-0.032499	0.00657866	-0.6150901	0.06934529
cg02579241	AC053545.3	blue	0.48057495	0.86620159	0.0144606	-0.0219736	-0.6895519	0.05004422
cg11448055	UBE2F;UBE2F;UBE2F;UBE	blue	0.62263615	0.62493193	0.00631045	0.06276791	-0.4982261	0.27044565
cg15221568	RNY3P9	blue	0.52475951	0.72960502	-0.0480529	0.1660857	-0.4036058	0.09669544
cg04666705	U6;APBB2;APBB2;APBB2;	blue	0.63618081	0.80592595	0.2271916	0.1634724	-0.4359598	0.18359035
cg11841722	LIFR-	blue	-0.598712	-0.6966509	-0.0056118	-0.1018208	0.36410604	-0.2309158
cg22524508	BTBD10;BTBD10;BTBD10;	blue	0.44301919	0.66951968	-0.1066578	0.03763581	-0.5339758	0.04764197
cg00442924	SERPINB7;SERPINB7	blue	0.65640418	0.70049298	0.115592	0.12748414	-0.3232907	0.29044578
cg01214376	AC007278.2	blue	0.51738002	0.77173995	0.02383631	-0.0040592	-0.6423345	0.11473326
cg18975764	BMP4;BMP4;BMP4;BMP4	blue	-0.5106451	-0.5841385	0.20516789	0.00951099	0.4868472	-0.1003331
cg06155641	GLT6D1	blue	0.31030061	0.68908706	-0.2446232	0.01490816	-0.4978263	-0.0329316
cg18319790	SLC25A47;SLC25A47	blue	0.38359013	0.67937768	-0.1442287	0.02278071	-0.4348415	0.10082552
cg21746537	ARHGAP17;ARHGAP17;A	blue	0.42050571	0.73374022	0.16129557	-0.0908876	-0.6301588	0.10987318
cg05772125	AHSP;AHSP;AHSP	blue	0.28824198	0.80597369	-0.0905297	-0.0860358	-0.7028901	-0.0813297
cg07974859	INSC	blue	0.4134311	0.89607771	0.02466369	0.01398887	-0.717697	0.01802825
cg02697979	ABTB2	blue	0.47902216	0.88064645	-0.0169235	-0.015996	-0.7192475	0.10394972
cg00159805	SCGB1D1	blue	0.43418163	0.85816448	-0.1109564	0.01866287	-0.5689779	-0.0367444
cg19931793	RP11-171N4.3;RP11-	blue	0.43609084	0.88992863	-0.1431642	-0.0254402	-0.6818919	0.02531639
cg07782778	GPM6A;GPM6A;GPM6A;R	blue	0.19417574	0.51346068	-0.0782815	-0.1158727	-0.3561868	-0.1799074
cg13868520	U6;TNFRSF8	blue	0.25371235	0.78068999	-0.0430949	-0.1828483	-0.777736	-0.1151384
cg12466324	OPRM1;OPRM1;OPRM1	blue	0.53424824	0.85519557	0.03439308	0.03279877	-0.6061473	0.14310633
cg07393857	AC069154.2;AC069154.2	blue	-0.4928654	-0.6007359	0.26130464	-0.0422675	0.37995517	-0.0654047

cg01940139	KLRD1;KLRD1;KLRD1;KL	blue	0.41895512	0.73545434	0.31513463	-0.0206579	-0.6676807	0.14224655
cg24544716	RP11-1152H15.1;GALC	blue	0.36128856	0.83154935	-0.0333309	-0.1268324	-0.7322589	-0.0375033
cg27062927	USH1C	blue	0.63280549	0.77166962	-0.0383149	0.1616112	-0.3788384	0.22520293
cg25562068	IGLV1-44	blue	0.61174327	0.77645641	-0.0595961	0.08520771	-0.4333062	0.21051102
cg19038454	DMKN;DMKN;DMKN;DM	blue	-0.430487	-0.635879	0.27191168	0.06536669	0.53699306	-0.0438383
cg23250700	RAD23A;RAD23A	blue	0.4774886	0.63896376	-0.1035893	0.1149346	-0.3013584	0.08087314
cg19380293	EIF2AK4;EIF2AK4;EIF2AK	blue	0.59188368	0.68619986	-0.1617972	0.1072979	-0.2831996	0.11643775
cg00362604	SAYSD1;SAYSD1	blue	-0.4534057	-0.5239511	0.13565253	0.01850106	0.27761793	-0.1154092
cg15024178	RP11-167H9.6;RP11-	blue	0.67653257	0.84578334	-0.1246495	0.1022153	-0.4444776	0.16437988
cg26267097	MINPP1	blue	0.39312678	0.69864569	0.25427979	0.00439233	-0.6645391	0.1542297
cg10480300	KLF6;KLF6;KLF6;KLF6	blue	0.42613948	0.84854744	0.11250209	-0.0243669	-0.746237	0.04778973
cg15109596	LRRK1;RP11-	blue	0.61891654	0.7696115	-0.0758805	0.13379686	-0.4391215	0.12153912
cg25027801	RP11-273B19.1;RP11-	blue	0.72369417	0.7507425	0.16505094	0.22364746	-0.3357678	0.26381614
cg16064478	RPP25;SCAMP5;RPP25;SC	blue	-0.5611678	-0.6467847	-0.023178	-0.0998572	0.41671556	-0.2302567
cg08552233	AMOTL2;AMOTL2;AMOT	blue	0.59181079	0.6747385	0.20656596	0.21689892	-0.2812383	0.16340652
cg17470778	TCF4;TCF4;TCF4;TCF4;TCF	blue	0.53742432	0.79346049	0.20069972	0.00891284	-0.5851286	0.13859012
cg10055566	NKAPL;ZKSCAN4	blue	-0.2948185	-0.5757066	0.06511508	0.05140895	0.4891627	0.01623997
cg21307456	RP11-243M5.1;GPR133	blue	0.71340671	0.68799578	0.00057198	0.25859172	-0.1968523	0.24563599
cg11622428	MED12L;P2RY14;P2RY14;P	blue	0.3861	0.88913546	-0.1341946	-0.0490156	-0.710413	0.01203088
cg10727259	DNMBP-AS1;DNMBP-	blue	0.5684977	0.7423054	0.05443656	0.07646431	-0.4879924	0.21785294
cg22776856	THOC6;THOC6;THOC6;TH	blue	0.40524935	0.76249413	0.07292467	-0.0324347	-0.6804636	0.15064322
cg21446930	TANC1;TANC1	blue	-0.4871422	-0.6606507	0.07943777	0.11185295	0.59206456	-0.0799747
cg00163394	AL049647.1	blue	0.42330417	0.83307218	0.01315627	-0.0034789	-0.6821679	0.09882523
cg07580247	RP11-490D19.6;RP11-	blue	0.65192087	0.76022029	-0.194688	0.2058909	-0.2999914	0.05624621
cg15076259	TNNI1	blue	0.43861753	0.80150715	-0.050625	-0.0196383	-0.5791397	0.00252424
cg19969391	AC090206.1;KCTD1	blue	0.31942993	0.78637084	-0.0055871	-0.0804747	-0.6778691	-0.1230173
cg23627040	TDRD5;RP11-	blue	0.28735919	0.46619146	-0.1661416	-0.095578	-0.3598369	0.01644852
cg01267270	PHAX;PHAX;PHAX	blue	0.61471087	0.85586824	0.08025651	0.08213353	-0.567687	0.19670234
cg03106550	RP11-1038A11.3;RP11-	blue	0.55283387	0.74876071	-0.1176568	0.0904257	-0.4306028	0.10793929
cg02890365	MIR554;TUFT1;TUFT1;RP1	blue	0.41315566	0.82873913	-0.1690315	-0.0225409	-0.5144029	-0.0955302
cg09401639	TRAJ28	blue	0.3894707	0.81066423	-0.1054408	-0.0855803	-0.7933558	-0.0406764
cg18987903	AC011748.1;AC073218.1	blue	0.51705721	0.67221499	-0.3367111	0.09938	-0.2584709	-0.0911056
cg23672095	ATP2B2-IT1;ATP2B2	blue	0.40496306	0.74147968	-0.0530605	-0.0361795	-0.5347011	0.0106522
cg03659484	ADAM18;ADAM18;ADAM	blue	0.61836466	0.73815933	0.03718748	0.12069599	-0.4320782	0.18083366
cg10995281	POPDC3;POPDC3;POPDC3	blue	0.42660005	0.67070709	0.10688394	0.03502505	-0.4097315	-0.0317168

cg07859728	GRIN2C	blue	-0.5698854	-0.744425	0.07783227	0.03276425	0.64157508	-0.1761105
cg04187121	FOXF2	blue	-0.5732793	-0.70362	0.20360765	-0.0410936	0.51026772	-0.094307
cg01449504	ZP1;ZP1;ZP1	blue	0.44293841	0.88337027	-0.1543725	0.04796388	-0.5910068	0.10183403
cg10709021	PURG;PURG;PURG;WRN	blue	-0.4872452	-0.6674371	0.17983361	0.08645747	0.54777999	-0.0880658
cg02697867	KSR2	blue	0.55096725	0.62438064	-0.1931623	0.07269945	-0.3323944	0.07085088
cg08535217	CTD-3046C4.1;CTD-	blue	0.38192666	0.69817493	-0.3059922	-0.0473959	-0.4031267	-0.1691593
cg02205957	GBP5;GBP5;GBP5;GBP5	blue	0.36024781	0.59283088	-0.0495254	-0.0525581	-0.4351379	-0.0546949
cg19237879	SCUBE2;SCUBE2;SCUBE2;	blue	-0.3141015	-0.5819712	0.18410254	0.14864814	0.53136675	0.07590356
cg14639919	RP11-	blue	0.67341507	0.69556795	-0.0261303	0.16742221	-0.3070023	0.29828416
cg02159381	BSX	blue	-0.5874817	-0.7629247	-0.0345652	-0.1212918	0.49156338	-0.1104276
cg06218372	DLX3	blue	-0.5188042	-0.7303749	0.09608043	0.0286178	0.58479011	-0.1321283
cg13442786	RP11-298H24.1;RP11-	blue	0.40222329	0.6317746	-0.4161428	-0.0733877	-0.2811142	-0.140535
cg10613336	MLC1;MLC1	blue	0.64684166	0.74845332	0.10999289	0.22547193	-0.4230265	0.18140807
cg23642651	SLC13A3;SLC13A3;SLC13	blue	0.70189727	0.84991423	0.00657171	0.19831955	-0.4868098	0.20823652
cg07862031	LURAP1;POMGNT1;POMG	blue	-0.6246615	-0.7968158	0.09221194	-0.063224	0.48660997	-0.2264358
cg16441044	LPO	blue	0.67495455	0.64502078	0.10041918	0.2225923	-0.2246639	0.25474753
cg15981958	PDZD9;PDZD9;PDZD9	blue	0.36031405	0.785527	-0.0189741	-0.0813412	-0.7791729	0.0512926
cg18173509	SEC11C;SEC11C	blue	0.49061009	0.56890795	-0.2158544	0.18942551	-0.1452558	0.06232772
cg16691844	ATE1;ATE1;ATE1	blue	0.4932367	0.80548074	0.21218172	-0.0107828	-0.712291	0.202061
cg18991998	RP11-99H8.1;RP5-952N6.1	blue	0.39448176	0.83048197	-0.1967032	-0.1163164	-0.5742745	-0.0829594
cg05613195	RP11-397H6.1;RP11-	blue	0.48151562	0.83584684	-0.2365069	0.01557995	-0.4886142	-0.0370672
cg12113251	METTL21B;TSFM;TSFM;TS	blue	0.55401859	0.79892004	0.01947476	0.07102766	-0.5003612	0.09651205
cg11659277	AC007950.1	blue	0.44483214	0.86950706	-0.0012337	-0.006022	-0.7530218	0.06802738
cg14221865	SPAM1;SPAM1	blue	0.5828246	0.78898659	-0.069834	0.18733015	-0.3102267	0.13520517
cg07350718	CYR61;CYR61;CYR61	blue	-0.537507	-0.7162156	0.15360786	-0.0309424	0.5452333	-0.1394717
cg23248351	ANGPT1;ANGPT1	blue	0.58200749	0.8887365	0.01604608	0.08680821	-0.5737312	0.20502837
cg04954926	AC105398.3;CLIP4;CLIP4;C	blue	0.71380012	0.75174803	0.00121643	0.1693786	-0.3670774	0.24398977
cg25380207	FRMPD2;RP11-	blue	0.58083295	0.69797438	-0.1816794	0.12697598	-0.312958	0.04318447
cg14376467	ARHGAP24;ARHGAP24;A	blue	-0.2877579	-0.4440057	0.16647431	0.07915301	0.40769635	-0.0654818
cg02313013	TMCC3;TMCC3;TMCC3	blue	0.5011458	0.60766263	0.03782778	0.1095965	-0.2935421	0.20543127
cg15180253	PRINS;KIAA1217;KIAA121	blue	0.37102406	0.85556981	-0.2238997	0.01059191	-0.6243515	-0.1359145
cg17840775	RP11-554I8.1	blue	0.60089485	0.81816387	0.18327264	0.04077319	-0.6674845	0.18495099
cg06835281	SPEG;SPEG;SPEG;SPEG;SP	blue	0.5421253	0.6047602	-0.0361521	0.2028099	-0.2217885	0.07772208
cg00190034	TACC1;TACC1;TACC1;TA	blue	0.5926004	0.76389118	-0.0429225	0.13077755	-0.3565463	0.13810374
cg22908277	SPINK5;SPINK5;SPINK5;S	blue	0.55724333	0.66721843	-0.3271635	0.08879248	-0.2448973	-0.01924

cg12020736	MECOM;MECOM;MECOM	blue	0.44797714	0.80135161	0.02359248	0.12212705	-0.5407507	0.01655071
cg16442820	TEAD2;TEAD2;TEAD2	blue	-0.4209834	-0.4929292	-0.1995448	0.00433295	0.39906165	-0.2627889
cg10625233	THY1;THY1;RP11-	blue	0.73845344	0.70811914	-0.0029242	0.24133797	-0.2221302	0.31370097
cg10214339	AC009475.2;NOSTRIN	blue	0.42271093	0.67268565	-0.3858418	-0.0343958	-0.3864782	-0.0920882
cg06313775	DSN1;DSN1;DSN1;DSN1;D	blue	0.7180315	0.67973402	0.07209049	0.13186995	-0.4072105	0.25423945
cg14325002	RP11-307C12.11	blue	0.63867922	0.82029022	0.00720455	0.20041851	-0.3967425	0.16397756
cg16592326	GLB1L3;GLB1L3;GLB1L3	blue	0.46171152	0.4925595	-0.342529	0.12514169	-0.1378546	-0.0406705
cg02361878	LMX1A;LMX1A;LMX1A	blue	-0.4276035	-0.5772159	0.21170221	0.09234589	0.30911479	-0.1299127
cg03462510	SNORD95	blue	0.49296768	0.57370908	-0.2672386	0.17527679	-0.1905417	-0.0270681
cg07476673	FAM120B	blue	-0.436559	-0.651912	0.05722414	-0.0242175	0.56242092	-0.0873271
cg10464163	OR2T4	blue	0.28476224	0.65928051	-0.0774946	-0.1255315	-0.5357776	-0.1034523
cg25485734	RP11-14C22.4	blue	0.45406185	0.71075163	-0.1244128	-0.0017273	-0.4316122	0.06012862
cg18986767	RP11-275I4.2;RP11-275I4.2	blue	0.35439248	0.81207234	0.07397626	-0.1023307	-0.8365984	0.07100744
cg24665667	AC017048.3;AC017048.3;A	blue	-0.5175148	-0.8234115	0.00667452	-0.0941258	0.56339569	-0.102756
cg27266972	WHSC2;WHSC2;WHSC2;W	blue	0.62980006	0.69053882	0.04473448	0.20810817	-0.3059061	0.25992167
cg12571541	PLCZ1;PLCZ1;PLCZ1;PLCZ	blue	0.42564827	0.82529012	-0.1818646	-0.0233983	-0.6710147	0.07533612
cg04369211	RNU5B-4P	blue	0.29704003	0.85723284	-0.1250596	-0.088238	-0.7842888	-0.037808
cg07529087	FURIN	blue	0.62852267	0.87713128	-0.0364054	0.08596334	-0.4837166	0.10769685
cg15905865	RN5S298	blue	0.55658836	0.6897858	-0.1635291	-0.0361096	-0.4432191	0.03621408
cg16562724	ESRP2;ESRP2;ESRP2;ESRP	blue	-0.4170674	-0.7660725	0.14172512	0.10843401	0.65196796	-0.1179711
cg27118072	FGFR1;FGFR1;FGFR1;FGF	blue	0.69210794	0.79908678	-0.0068191	0.2522639	-0.369982	0.22270068
cg13800745	CLDN18	blue	0.62239618	0.77344069	0.08743991	0.16119269	-0.3779494	0.21008751
cg05818824	SCN4A	blue	0.66227639	0.83295527	-0.0291236	0.19893936	-0.3759666	0.17760801
cg09584097	RP11-1084E5.1;RP11-	blue	0.67185328	0.82813917	-0.0859521	0.17538143	-0.4107423	0.10925863
cg20346534	GPR116	blue	0.65783985	0.74161212	-0.2738528	0.18283127	-0.2886304	0.11096996
cg27512252	REG1A;REG1A;REG1A;RE	blue	0.66759194	0.71226279	0.06382253	0.30664269	-0.1828006	0.23235103
cg12598089	KCNAB1;KCNAB1;KCNA	blue	-0.4115484	-0.4869005	0.03565158	0.06848335	0.43934223	-0.1055656
cg24568533	SNRPD3;SNRPD3;SNRPD3	blue	0.20387054	0.74775718	-0.2076779	-0.0718412	-0.7092039	-0.1135154
cg15683025	POMT2;POMT2;POMT2;PO	blue	0.67706599	0.61398171	-0.0920733	0.16029666	-0.1878165	0.21481584
cg08952929	RP11-697B24.1	blue	0.43860457	0.7899799	-0.2792738	-0.0126858	-0.5620412	0.00803552
cg02986171	U6	blue	0.56020758	0.86833876	-0.1776042	0.08000637	-0.4976724	0.07626552
cg05412137	MIR196A2;HOXC5;HOXC6	blue	-0.3776856	-0.4020119	0.20975166	0.07271518	0.23515469	-0.0151385
cg26776703	C8orf46;U6;C8orf46;C8orf46	blue	0.68552031	0.80925942	0.09571896	0.19316582	-0.3554222	0.13072376
cg17497599	RP11-451G4.2;RP11-	blue	0.37731276	0.81417873	-0.137643	0.02249655	-0.5652786	-0.0308965
cg12681745	GPRIN3	blue	0.39723437	0.75766699	-0.0452903	-0.0605717	-0.6408493	-0.0296253

cg15666789	TTR;TTR	blue	0.51090273	0.89113206	-0.0955774	0.03538301	-0.6056882	0.03947886
cg17652408	ZMIZ1;ZMIZ1	blue	0.44087156	0.8348511	-0.1069796	0.07227121	-0.5364572	0.04449812
cg01885503	IRX3;IRX3	blue	-0.5074957	-0.6136784	0.23326041	-0.0172342	0.38753403	-0.077075
cg16882903	VPS13D;VPS13D;VPS13D	blue	0.65232532	0.69765865	-0.0477746	0.03119556	-0.4118719	0.20790605
cg16523044	ITGB3	blue	0.54776823	0.79601605	-0.1734697	0.09645559	-0.4559497	0.14077961
cg17572695	RP11-13L2.4	blue	0.44462799	0.75961203	0.01554973	0.09456088	-0.4500316	0.13470096
cg21543394	RP4-782G3.1	blue	0.68586308	0.75059762	-0.0269636	0.13927047	-0.3217984	0.24041927
cg23987565	RP11-382A20.4;RP11-	blue	0.44362312	0.8074629	-0.0737793	0.054301	-0.5591083	0.00099529
cg20004451	GUCA1B;GUCA1B	blue	0.52431848	0.62379977	-0.0181704	0.16753981	-0.2697522	0.06318797
cg14580859	TRAF1;TRAF1	blue	0.33178323	0.75449283	0.02395687	0.04332754	-0.5765309	-0.1013085
cg03982250	RP11-65D17.1	blue	0.49958046	0.84842479	-0.1240539	0.03079464	-0.5927095	-0.0333133
cg17777683	CFLAR;CFLAR;CFLAR;CF	blue	0.33556046	0.5840866	0.12132626	0.16752271	-0.273235	-0.0555096
cg21534994	IRX4	blue	-0.4667714	-0.6612364	-0.0380107	-0.0613198	0.42879699	-0.0513513
cg02026449	MARVELD3;SNORA70D	blue	-0.4999093	-0.8285347	0.07174193	0.00144503	0.63863863	-0.065338
cg23970675	RP11-	blue	0.57163367	0.66668267	-0.3206964	0.06514537	-0.3150335	0.07741348
cg12438903	RP11-372M18.2	blue	0.76569007	0.80509871	0.10320899	0.24951424	-0.3173199	0.26840877
cg24365969	ACTN2	blue	0.58402628	0.69312059	-0.1576495	0.22009676	-0.2300447	0.10783393
cg08081865	PCCA-AS1;PCCA-	blue	0.35868088	0.83518448	-0.0485364	-0.0298851	-0.7288355	-0.0459443
cg25075662	FGD3;FGD3;FGD3;FGD3;F	blue	0.48255875	0.79360026	-0.0142975	0.00919072	-0.6204116	0.20405192
cg15586439	NPY5R;NPY5R;NPY1R	blue	-0.5323078	-0.6703255	0.20540404	0.11036085	0.4296257	-0.023245
cg24445674	CTAGE1	blue	0.63461425	0.7574773	-0.1303722	0.17602487	-0.3690195	0.13094424
cg22496928	CTD-2384A14.1;CTD-	blue	0.30844574	0.77057422	-0.039761	-0.0682522	-0.7231525	-0.0736777
cg11276986	CAP2;CAP2;CAP2;CAP2;C	blue	-0.4873264	-0.590955	0.17752467	-0.0079588	0.46607176	-0.1039554
cg11401996	WIPF1;WIPF1;WIPF1;WIPF	blue	0.31280841	0.72255289	-0.2272747	0.04477064	-0.4557308	-0.1622568
cg03832321	DSG4	blue	0.2614974	0.84107697	-0.1621515	-0.0919956	-0.7829164	-0.1737769
cg22432010	RP11-	blue	0.67334616	0.82530683	0.06750636	0.07626559	-0.5052438	0.24160331
cg00909259	RP11-107I14.4;RP11-	blue	0.53194619	0.69717114	-0.1537919	0.12956816	-0.2977927	0.08093934
cg05505745	ISLR2;ISLR2;ISLR2;ISLR2;	blue	-0.3895558	-0.5694008	0.11008144	-0.0486313	0.36882255	-0.0581219
cg25378502	AC007317.1	blue	0.48674884	0.86792082	-0.0945979	0.04746589	-0.6248271	0.06779565
cg17123655	TNFRSF9	blue	0.60598309	0.86643033	-0.055442	0.05998517	-0.6128063	0.17717522
cg02021789	CASP14;CASP14;AC004699	blue	0.40964402	0.57069741	-0.0732834	0.03485103	-0.3552381	0.02606195
cg09094797	RORB	blue	0.42734211	0.68187946	0.00400112	0.01703291	-0.4376625	-0.0480117
cg04166439	WDR49;WDR49	blue	0.41555773	0.90003969	-0.0708422	0.07111159	-0.6869998	-0.0153482
cg25499911	GRHL1	blue	0.47820996	0.84736265	-0.1880735	-0.0050132	-0.5612902	0.02694714
cg02254587	DDAH1;DDAH1;CYR61;C	blue	-0.3031435	-0.4601873	0.12690703	0.07627177	0.40173249	-0.0561745

cg14706131	RP11-	blue	0.46961745	0.89732739	-0.0442148	0.02066629	-0.678761	0.05228813
cg18200219	RP11-744J10.3;RP11-	blue	0.60536636	0.82181161	-0.091134	0.10469724	-0.4003739	0.1098553
cg08776627	SOGA2;SOGA2;SOGA2;SO	blue	0.42712365	0.86254619	-0.1065029	0.00337878	-0.630544	0.0335578
cg09712066	PART1;PART1;PART1;PAR	blue	0.64191712	0.86105468	-0.1092145	0.10451161	-0.5254964	0.23439931
cg24972091	snoMBII-202	blue	0.49551563	0.80080963	-0.0520171	0.03482591	-0.4512794	0.06450236
cg27336379	SLC44A5;SLC44A5;SLC44	blue	0.4973732	0.79130274	-0.2430482	0.04643306	-0.460296	-0.0422566
cg12992620	EXOC6B	blue	0.47881701	0.80563798	-0.3246466	0.04755184	-0.4326735	0.01237673
cg16892022	snoU13;AC109315.1	blue	0.4514857	0.81057747	-0.1133618	0.05988275	-0.5162131	-0.0523708
cg10374564	FRYL;FRYL;FRYL;FRYL;F	blue	0.51800314	0.81726407	0.14509358	0.15785855	-0.6514367	0.20438468
cg05061249	HDGFL1;HDGFL1	blue	0.41349536	0.70199164	-0.2270742	0.12354146	-0.3587153	-0.1041106
cg08703718	CORO2B;CORO2B	blue	-0.4935044	-0.5863613	0.15868899	-0.1185637	0.28980037	-0.0447382
cg04819714	TMEM133;ARHGAP42	blue	0.37290906	0.70572199	0.05529071	0.00925669	-0.4994251	0.16203756
cg16725262	DCLK1	blue	0.42074299	0.84681294	-0.0957505	0.00482728	-0.6195126	-0.0282513
cg18173821	RP11-789C1.1	blue	0.52683022	0.79782758	-0.1461805	0.19461873	-0.367812	-0.0318906
cg27452471	snoU13	blue	0.33897084	0.76145368	-0.2481893	0.03945358	-0.4402866	-0.1200445
cg18174654	CD8A;CD8A	blue	0.53177544	0.7189174	0.07768105	0.09820378	-0.5768603	0.10857584
cg25872852	RPLP2;RPLP2;RPLP2;RPLP	blue	0.52865417	0.69736235	0.32687728	0.08638848	-0.4291905	0.05026888
cg04358103	FBXO16;FBXO16;FBXO16;	blue	0.63571549	0.88006431	0.06374438	0.17979336	-0.4902646	0.19547161
cg01229868	AC074129.1;CTD-	blue	0.52389115	0.7888221	0.01302882	0.07231487	-0.4236397	0.07773197
cg04652611	C1orf114;RP1-206D15.3;RP1-	blue	-0.2073864	-0.6140991	0.17151137	0.23437425	0.60200574	0.16623452
cg13008005	LRRC7;LRRC7	blue	0.38787953	0.81689632	0.13603089	0.00749853	-0.6275764	0.04664396
cg08914740	SPINK5;SPINK5;SPINK5;S	blue	0.5299242	0.86665322	0.0797992	0.08404123	-0.5541411	0.09989851
cg02589673	AC091559.1;TUSC3	blue	0.54785687	0.77319306	-0.1436277	0.1158801	-0.4386757	0.00331314
cg02934347	CAP2;CAP2;CAP2;CAP2;C	blue	-0.5399146	-0.6320778	0.13598359	-0.0033923	0.45573327	-0.0418024
cg21616089	IGF2BP1;IGF2BP1;RP11-	blue	0.51667096	0.72223811	-0.0851135	0.09234168	-0.3866679	0.02434722
cg09121994	RP11-380B22.1	blue	0.4481072	0.5971505	0.12435923	0.04201298	-0.3316458	0.03445645
cg23879721	RTDR1;RTDR1	blue	0.48932896	0.74459025	-0.0013626	0.08458703	-0.4328474	0.08099705
cg00570821	RP11-342C23.4	blue	0.51013772	0.81403491	-0.0379652	0.09516223	-0.5580221	0.15463949
cg15695213	MYF5	blue	-0.4013707	-0.4579628	0.12109774	0.04650993	0.38387488	0.00921041
cg01403057	7SK;MUC17	blue	0.36657842	0.54913123	-0.0977708	0.07143201	-0.2548925	-0.0507624
cg23709011	GPNMB;GPNMB;GPNMB;	blue	0.5932146	0.88052716	-0.0022041	0.10830074	-0.5828638	0.06871027
cg02545303	AC010729.3	blue	0.38457799	0.52898878	-0.115971	0.12208683	-0.1741331	0.00026013
cg16602957	RP11-244F12.2	blue	0.59728497	0.74564373	-0.2172233	0.13426171	-0.350971	0.00345316
cg10334385	AC062028.1;AC062028.1;A	blue	-0.5702343	-0.5919391	0.07041549	0.02973457	0.46402091	-0.1541388
cg19646165	CTD-	blue	0.39545293	0.42152953	-0.1774509	0.13718547	-0.1251839	-0.0338176

cg03388789	ZIC4;ZIC1	blue	-0.379239	-0.7940638	0.22788022	0.07063618	0.64201047	0.02030058
cg26170496	SLC34A2;SLC34A2	blue	0.72647231	0.70768509	0.24222674	0.15890744	-0.3347705	0.3085365
cg16746471	KIAA1324L;KIAA1324L;KI	blue	-0.44747	-0.5483311	0.22952455	-0.0080933	0.38308738	0.03180362
cg13529626	2-Mar	blue	0.54614298	0.89858362	-0.1014307	-0.0319349	-0.6533853	0.04229117
cg11674695	RP1-251I12.1	blue	0.38035158	0.73974002	-0.2622734	-0.0126092	-0.4960086	0.00303637
cg17001758	RN5S388	blue	0.57602095	0.8227518	-0.0618207	0.16672601	-0.4053147	0.13270624
cg15659537	CAPS2;CAPS2;CAPS2;CAP	blue	0.46678227	0.82230634	-0.2309541	0.06751871	-0.4964388	-0.1090146
cg02691523	RP1-144F13.4;PPIE	blue	0.24296654	0.3950986	0.04335683	-0.0053614	-0.1958903	-0.0386313
cg18332471	RSF1;RSF1	blue	0.31468925	0.85895193	-0.1357164	-0.0877307	-0.8049071	-0.0440394
cg11040636	RP11-268F1.3	blue	0.38203324	0.87240113	-0.1813881	-0.0308045	-0.6807651	-0.0765238
cg11268628	EFR3A;EFR3A;EFR3A	blue	-0.4307735	-0.5521342	0.17267473	0.07904256	0.47368778	0.00231015
cg19476053	CCDC140;CCDC140	blue	-0.3450876	-0.5165201	0.25171233	-0.0229543	0.32413445	0.14527542
cg27090808	RP11-673F18.1	blue	0.57943925	0.81317793	-0.0637037	0.1407989	-0.3829569	0.04900183
cg02377948	RFX4;RFX4;RFX4;RFX4	blue	-0.4083564	-0.573456	0.16780153	0.03594031	0.37308453	0.02397442
cg06448508	AC009410.1	blue	0.5361322	0.72639921	0.08941441	0.03007179	-0.3881074	0.13010976
cg13669740	TIGIT;TIGIT;TIGIT;TIGIT;T	blue	0.34384245	0.81695375	-0.0831497	-0.1013518	-0.7977393	-0.0102264
cg21332070	AC006159.5;CAV1	blue	0.50452779	0.70952923	-0.3717988	0.05006674	-0.2907092	-0.0080079
cg09158313	RP3-417O22.3	blue	0.60362851	0.75543602	-0.117228	0.23134166	-0.3243623	0.06618086
cg06298751	BCAR3;BCAR3	blue	0.30992622	0.83080098	0.00788956	-0.0737999	-0.8290024	-0.0296553
cg03586879	RBFOX1;RBFOX1;RBFOX	blue	-0.4210124	-0.5337797	0.12660999	0.00463028	0.32138923	-0.0123546
cg11400151	NGFR;NGFR	blue	-0.4743971	-0.7888787	0.01632388	-0.0144572	0.56284618	-0.0135891
cg08591438	STK33;STK33;STK33;STK3	blue	0.70370348	0.65949961	-0.0190093	0.22082215	-0.2329004	0.2424792
cg06805557	APBB1;APBB1;APBB1;AP	blue	-0.3639763	-0.5498177	0.1914509	0.03450303	0.47199525	0.00133905
cg27507681	VSTM2L;VSTM2L;VSTM2	blue	-0.2823554	-0.472314	0.22772536	0.03907611	0.40375403	-0.0465595
cg05017001	PLEKHA1;PLEKHA1;PLEK	blue	0.4854984	0.86082032	-0.1709136	-0.0215059	-0.5542462	-0.0591317
cg17423568	ZNF503-AS1	blue	0.72241875	0.69560378	0.14932989	0.10931057	-0.3127858	0.23727528
cg23441478	AC123767.1	blue	0.62992248	0.6145321	0.00217366	0.22230602	-0.1554871	0.31853904
cg01625621	CHRM5;CHRM5	blue	0.28755002	0.54225594	-0.3396405	0.00095444	-0.359741	-0.1007824
cg04640842	RP11-22C11.2;ESRP1	blue	-0.4750425	-0.7664852	0.04851806	-0.0086186	0.56761295	-0.1295314
cg13335775	RP11-1016B18.1;RP11-	blue	0.26384131	0.69414628	-0.2325748	-0.1052668	-0.4952169	-0.3309766
cg07271510	RP11-305A4.3	blue	0.68780316	0.83245836	0.03072972	0.16935748	-0.4060569	0.1810517
cg23817969	RP11-981G7.2;RP11-	blue	0.40023783	0.85612232	-0.1458187	-0.0739503	-0.7191716	0.02590906
cg19085165	RP11-148I19.1;RP11-	blue	0.14442217	0.46398598	-0.1584392	-0.1048092	-0.3766862	-0.1144891
cg26116651	AC008834.1	blue	0.29232479	0.55156002	-0.1287254	0.0573059	-0.2328596	-0.0408908
cg02686048	NEB;NEB;NEB;RIF1	blue	0.72820256	0.78154258	0.00909484	0.17741076	-0.330356	0.28104705

cg02841716	C9orf100;C9orf100;CA9	blue	-0.3883203	-0.7025358	0.16896005	0.11065263	0.59879365	0.04841909
cg22305495	AC090324.1	blue	0.66318828	0.68824688	0.25367442	0.14720814	-0.3631135	0.26543155
cg10702457	MEPE;MEPE;MEPE;MEPE;	blue	0.37724668	0.82220755	-0.0638746	-0.0180988	-0.5733282	-0.0634813
cg03466765	AC069286.1;SDK1	blue	0.27913656	0.79939663	-0.0231251	-0.114871	-0.8402639	-0.0240423
cg16612562	RASL10A;RASL10A	blue	-0.1942206	-0.4796909	0.33600152	0.11416022	0.39987452	0.14714064
cg24036639	CTC-264O10.1	blue	0.51640355	0.79815234	-0.1536245	0.08717726	-0.4591823	0.15376058
cg12776605	ARPP21;ARPP21;ARPP21;	blue	0.30491323	0.75472585	-0.1334772	-0.0024354	-0.491482	-0.0716755
cg06283304	FBXO21;FBXO21	blue	0.58375515	0.78631537	-0.0687024	0.10713276	-0.4536407	0.058276
cg10495096	RP1-159G19.1	blue	0.24456994	0.78616162	-0.2247415	-0.0964437	-0.6777947	-0.1672612
cg08008573	RP11-565N2.1	blue	0.47564853	0.77811937	-0.0807506	0.05533068	-0.4434894	0.04235747
cg21142398	GLIS1	blue	0.66351976	0.82808323	0.16036279	0.19768889	-0.4324354	0.22936723
cg20811659	RP11-	blue	-0.5737042	-0.7768939	0.03654086	-0.0498346	0.57861192	-0.1535037
cg25052562	TUBA8;TUBA8;TUBA8	blue	-0.4917831	-0.581627	0.09077315	-0.0078536	0.40712926	-0.245262
cg25176357	RP11-372H2.1	blue	0.51307472	0.77033217	0.06904082	0.07426894	-0.528769	0.22313664
cg07795407	MEIS2;MEIS2;MEIS2;RP11-	blue	-0.5230834	-0.6673121	0.08788384	-0.0755573	0.39857697	-0.0526413
cg26675994	EBF2	blue	0.65783429	0.71872332	0.02115125	0.20160986	-0.2732691	0.18559746
cg10641011	RP11-22D3.2	blue	0.3328448	0.74120678	-0.0996602	0.00533081	-0.5328593	-0.0447467
cg24554839	MIR921;RP11-9L18.3	blue	0.53469646	0.84479264	0.1299498	0.09417123	-0.5328735	0.12507747
cg07392048	LNX1;LNX1;LNX1;LNX1;L	blue	0.59145748	0.70696509	-0.0961675	0.09161327	-0.2755201	0.16806031
cg03708544	RP5-945I17.2	blue	0.49168085	0.70796112	-0.1328151	0.01017674	-0.431329	-0.0340849
cg01456057	7SK	blue	0.48969941	0.76662001	0.18745406	0.06280417	-0.5178789	0.20424682
cg15234527	TCF7L2;TCF7L2;TCF7L2	blue	0.57118839	0.8683042	0.18254138	0.11113334	-0.6274257	0.17141733
cg06805498	ALCAM;ALCAM	blue	-0.3047819	-0.6920448	0.23232281	0.06117401	0.63730059	0.05238209
cg26319563	AC005154.6;AC005154.6;A	blue	0.40163341	0.72924848	0.02115612	-0.119018	-0.6649173	0.06537441
cg00512998	AC092765.1	blue	0.3684559	0.78284053	-0.1701303	0.00986436	-0.5429788	-0.0800749
cg02014117	HMGCLL1;HMGCLL1	blue	0.33799338	0.73561592	0.0663892	0.06175061	-0.5089825	-0.0729928
cg14787179	CYB5RL;CYB5RL;CYB5RL	blue	0.6472069	0.6936839	-0.1357837	0.16608013	-0.2600283	0.16812719
cg26101086	ARHGEF10;ARHGEF10;AR	blue	0.65021785	0.64808997	-0.0228111	0.16977539	-0.2665143	0.17407749
cg16228415	DGKI;DGKI;DGKI;DGKI;D	blue	0.68264122	0.74179205	-0.0362346	0.19556996	-0.2768483	0.16879281
cg27068678	ALK;ALK	blue	-0.4338519	-0.8126883	0.14546532	-0.0056739	0.68467559	-0.116161
cg07135941	NRG1-IT3	blue	0.16890046	0.65386257	-0.2043919	-0.0832481	-0.5857137	-0.1314376
cg11679151	RP11-760H22.2;RP11-	blue	0.57608976	0.89684011	-0.0607624	0.09569729	-0.5754552	0.09450381
cg06119400	CRNDE;CRNDE;CRNDE;C	blue	0.39549434	0.62460717	0.37129811	0.03884746	-0.492	0.19490947
cg23381265	C11orf92;C11orf92;C11orf93	blue	-0.6570898	-0.6631628	0.21167846	-0.0197779	0.43371284	-0.1545186
cg05772272	SLC22A6;SLC22A6;SLC22	blue	0.68320655	0.74027796	0.06147773	0.20802675	-0.30537	0.11190853

cg06493439	CLVS1	blue	0.57407918	0.8184732	-0.0005841	0.0971904	-0.4244161	0.05615335
cg11104313	TAGLN3;TAGLN3;TAGLN	blue	-0.4152186	-0.6786658	0.02673444	0.0939107	0.58445334	-0.1143073
cg24197763	CLRN2	blue	0.71756873	0.73770929	0.1414798	0.3041106	-0.3208893	0.26337868
cg25783047	KLHL33	blue	0.59054736	0.70101018	0.02726798	0.21536137	-0.3083787	0.09736627
cg19444422	RP11-	blue	0.57196867	0.8764718	0.15248399	0.00829609	-0.6052277	0.1745064
cg00959375	A1CF;A1CF	blue	0.49931315	0.75037458	-0.1742063	0.01911268	-0.4633316	-0.0130758
cg09306252	RP11-454C18.1	blue	0.55950426	0.86425041	-0.1631174	0.07537739	-0.5415622	0.05050027
cg15109791	C7orf44;C7orf44	blue	0.40768593	0.57055635	-0.1115789	0.01034373	-0.3745731	0.10353018
cg20433382	RP11-	blue	0.42417208	0.61774489	-0.1217316	-0.0574937	-0.3201182	0.03390998
cg18827221	HLA-DPB1;HLA-	blue	0.55477064	0.77829431	-0.0410917	0.07970988	-0.4912173	0.17176533
cg10729196	RP11-167H9.3;snoU13;RP11-	blue	0.58629951	0.82731198	0.05452784	0.10043004	-0.4681116	0.12144139
cg21483818	U6	blue	0.52259945	0.64783187	0.2185057	0.17088417	-0.253792	0.11977078
cg13210580	MPZ;MPZ;MPZ;MPZ	blue	-0.5593825	-0.6731126	0.04204859	-0.0158942	0.47045323	-0.0307889
cg07012128	DLGAP2;DLGAP2	blue	0.45417405	0.80895363	-0.1290852	0.10087514	-0.5202668	0.05265051
cg26764445	TRAJ27	blue	0.46283732	0.76554053	0.22969037	-0.0555281	-0.6673842	0.12667665
cg23659216	CDH1;RP11-	blue	0.66462118	0.74460688	-0.1354729	0.11282523	-0.3687072	0.20053524
cg03993263	IGSF21	blue	-0.5597602	-0.7853082	0.17201599	-0.0553404	0.53256553	-0.1049786
cg23837299	ACACB;ACACB	blue	-0.4908692	-0.6194251	0.13930746	-0.001904	0.52790446	-0.1232271
cg05280750	PLAC2	blue	-0.2829782	-0.425058	0.16104601	0.02872655	0.28101819	0.08530969
cg03826976	CYB5R2;CYB5R2;CYB5R2;	blue	-0.2710565	-0.5293899	0.04558946	0.05520522	0.38898341	0.10408396
cg14804342	SCRG1;SCRG1	blue	0.51625757	0.82835322	-0.1151658	0.18688713	-0.5080287	0.10476948
cg19981951	AC100848.1;LINC00305;LIN	blue	0.75499634	0.76250192	0.29129039	0.28726716	-0.3495935	0.35284217
cg20142535	ADORA1;ADORA1;ADOR	blue	-0.4641143	-0.4565698	0.1910273	0.04714001	0.28779555	-0.0491417
cg00820637	FRMD6;FRMD6;FRMD6-	blue	0.34951041	0.85354475	-0.1613591	-0.0710865	-0.7185794	-0.0375284
cg00182393	LINC00343;LINC00343;LIN	blue	0.45851952	0.84166508	-0.2475217	-0.0114612	-0.4972351	-0.035155
cg22809516	SPIC	blue	0.34477784	0.45163762	0.07494595	0.10131292	-0.2719905	0.05787008
cg14411221	WDR89;CTD-2302E22.2	blue	0.27425669	0.83280377	-0.065516	-0.1241564	-0.8492499	-0.081729
cg26129675	RN5S134;SYNPR;SYNPR;S	blue	0.52939677	0.77393834	0.17410104	0.09130502	-0.4578775	0.09038066
cg16832407	TRPM3;TRPM3	blue	-0.221851	-0.6272858	0.23826588	0.14488705	0.54254984	0.11740796
cg14326161	PLCB1-IT1;PLCB1-IT1	blue	0.47015175	0.82039062	0.00404228	0.01222378	-0.5997523	0.06132699
cg07901571	GPR115;GPR111;GPR111	blue	0.41212791	0.87249802	-0.2403683	-0.0567998	-0.7003152	-0.0022131
cg16962933	TRPM3;TRPM3;TRPM3;TR	blue	0.63447762	0.74915763	0.06105945	0.08789615	-0.4172771	0.24684166
cg16088761	PARVA;PARVA;PARVA	blue	0.3020471	0.40383646	-0.1931242	0.09231638	-0.1521804	0.03475082
cg04547576	BCL7A;AC156455.1	blue	0.75291334	0.74933749	0.14371451	0.20205504	-0.3107923	0.33762888
cg27469932	OSBPL10;OSBPL10-	blue	0.71273364	0.80324621	0.05690456	0.16632122	-0.3572473	0.24112975

cg15357821	HK3;HK3;HK3	blue	0.38113772	0.85645031	0.12464617	0.03234259	-0.6994495	0.06865717
cg07266364	RP11-520D19.2;RP11-	blue	0.42934534	0.79624546	0.02563912	0.06997516	-0.5609094	0.10319595
cg05303639	TRPV1;TRPV1;TRPV1;RP1	blue	0.7016973	0.79279068	0.32712308	0.15965482	-0.4603401	0.31429169
cg03632704	LINC00461;LINC00461;LIN	blue	-0.4147291	-0.6382328	0.09451265	0.05417142	0.42704434	-0.0414502
cg15112355	NMU;NMU;NMU;NMU;N	blue	-0.3874531	-0.5865563	0.28928736	0.091136	0.53880428	-0.1032035
cg02775263	SNORD112	blue	0.4747588	0.61739607	-0.3809601	-0.0725242	-0.3786693	-0.0025254
cg03948727	TMC3;TMC3	blue	0.56071684	0.86188131	0.14008353	0.08250221	-0.6042787	0.17297225
cg01826714	BTNL8;BTNL8;BTNL8;BTN	blue	0.66063484	0.74764452	0.07197478	0.156608	-0.3763489	0.23811353
cg14354851	RP11-503C24.2	blue	0.61971035	0.77299376	0.03908217	0.09854789	-0.406281	0.2342251
cg04098051	RN5S107	blue	0.57385435	0.85644152	0.03497896	0.06832045	-0.5918101	0.11037391
cg17006442	RP11-69C17.2;RP11-	blue	0.42552391	0.76522661	-0.0280981	0.02930988	-0.4896997	0.01715076
cg03737188	RP11-572M11.4;RP11-	blue	0.41167318	0.88954657	-0.0526238	0.00263478	-0.6599132	-0.0561598
cg25682080	APOA5;APOA5;APOA5;AP	blue	0.50476917	0.76598047	-0.066671	0.09086614	-0.4027483	0.00536689
cg25052625	U7;PTPRD;PTPRD;PTPRD	blue	0.38974571	0.81998513	0.02734703	-0.0275588	-0.612309	-0.0510791
cg24026435	PSTPIP2;PSTPIP2;PSTPIP2	blue	-0.5698936	-0.6566153	-0.0188747	-0.1564747	0.44508396	-0.2346506
cg05425374	GREM1;GREM1;GREM1	blue	0.49929547	0.8545262	0.10237658	0.11317563	-0.6232262	0.08617907
cg18724755	AC007362.3;NRP2;NRP2;N	blue	0.71381732	0.82401829	0.05886955	0.19357757	-0.3859625	0.2043922
cg27322705	RP11-356O9.2;RP11-	blue	0.27609398	0.8075075	-0.1503278	-0.0494447	-0.6754431	-0.1376182
cg06618666	MMP3;MMP3	blue	0.4228853	0.56303838	0.06004444	0.0039637	-0.3150212	0.1160611
cg01767862	SNORD114-28	blue	0.55325029	0.87798374	-0.1122101	0.06159627	-0.5150269	0.02231788
cg15509705	SERPINA5;SERPINA5;SER	blue	-0.4415579	-0.5593127	-0.0533029	0.0026477	0.40696317	-0.1579413
cg03099025	TRBV2	blue	0.65218034	0.76411311	0.18468313	0.09621886	-0.5937045	0.21026552
cg27584713	PHACTR1	blue	0.50895237	0.839813	-0.071564	0.07553741	-0.4710266	0.03586818
cg20360285	PKD1L1;HUS1	blue	0.37101169	0.8482247	-0.0666344	0.03610289	-0.6300453	-0.0220651
cg07650439	RP11-443N24.4	blue	0.62620486	0.67682168	-0.0764808	0.21946289	-0.2002378	0.15190107
cg17922851	DZIP1;DZIP1;DZIP1	blue	-0.2388725	-0.4526592	0.27681713	0.17501488	0.42113743	0.03497832
cg21871080	KCNJ16;KCNJ16;AC002539	blue	0.6499566	0.67673431	-0.0977678	0.1389839	-0.2696843	0.16558718
cg26855208	AVEN;CHRM5;CHRM5;CH	blue	-0.4803018	-0.5896013	0.12554367	-0.0123373	0.37699073	-0.1891613
cg15990724	NR1H4;NR1H4;NR1H4	blue	0.57622664	0.77652315	0.11567998	0.13977596	-0.4172885	0.11015917
cg15386044	AC007563.1;AC007563.5	blue	0.49885474	0.74721853	0.13193681	0.22049229	-0.3295842	0.11064622
cg21995038	AC007743.1;AC007743.1;CC	blue	0.68335961	0.83733009	0.09000969	0.19332358	-0.4093883	0.24713874
cg12945572	RP3-333A15.1;PTGER3	blue	0.53959856	0.89202865	-0.1087287	0.06360483	-0.5732853	0.12186593
cg16921950	GBAS;GBAS;GBAS;GBAS;	blue	-0.201152	-0.3493416	0.13941258	0.16976565	0.35688459	-0.0791607
cg07617039	RSPO3;RSPO3;RP11-	blue	0.37900543	0.41555934	-0.0943219	0.0967956	-0.1269202	0.05889519
cg09724210	U6	blue	0.73451288	0.79020351	0.19654837	0.21108175	-0.4390048	0.33859693

cg06156376	SHOX2;SHOX2;SHOX2;SH	blue	-0.5989559	-0.6022582	0.10149251	-0.104149	0.30768838	-0.1469872
cg16932304	WFDC6;WFDC6	blue	0.3363383	0.51652265	0.06386388	-0.0850112	-0.4220115	0.05568217
cg00068217	RP4-564O4.1	blue	0.50605016	0.79268933	-0.1260181	0.0597814	-0.4922	0.06258878
cg00639176	EPB41L3;EPB41L3;EPB41L	blue	0.57737514	0.79666502	0.17654564	0.06648618	-0.5169509	0.28881288
cg09726569	snoU13	blue	0.43498373	0.85618907	0.01650312	0.0312746	-0.6495895	0.00100644
cg11731257	ADAMTS12;ADAMTS12;A	blue	-0.4455819	-0.5942631	0.18208939	0.01598719	0.43121699	-0.0730169
cg01129459	RP11-	blue	-0.3546622	-0.7767184	0.2036299	0.09316702	0.59876067	0.02812676
cg15057062	FASTKD2;FASTKD2;FAST	blue	0.46402091	0.66852432	-0.1157656	0.11470009	-0.2616902	0.02709226
cg00864867	PAWR;RP11-	blue	-0.423502	-0.7248472	0.14229867	-0.0198478	0.49243709	-0.0521162
cg23772533	RPL13;RPL13;RPL13;RPL13	blue	0.33639819	0.81425876	-0.0494783	-0.0149371	-0.6765917	-0.0030341
cg03089468	CTD-2125J1.1	blue	0.68467604	0.69559258	0.19041442	0.21440735	-0.3051939	0.26146144
cg11774275	SPAG9;SPAG9;SPAG9;SPA	blue	0.36664109	0.82990028	-0.0099426	-0.0303015	-0.7103491	-0.0774247
cg16412222	LL22NC03-86D4.1	blue	0.41505265	0.64459957	-0.1218589	0.03082675	-0.368683	-0.0268954
cg14341654	AP000345.1;AP000345.1	blue	0.63325775	0.83605911	0.06046154	0.10875693	-0.4681447	0.21484578
cg16136973	CTD-2139B15.4	blue	0.61737666	0.80630024	0.28171263	0.16530819	-0.4994185	0.26957239
cg20587394	HOXC13;HOXC-AS5	blue	-0.4711066	-0.7790472	0.11048261	0.02806454	0.59474999	-0.0167269
cg13320585	MIR1252;RP1-78O14.1;RP1-	blue	0.3560517	0.85728888	-0.0612485	0.02358083	-0.6914494	0.0092906
cg21379968	ARSG	blue	0.52422555	0.78749189	0.04482111	0.13671453	-0.4831527	0.12281268
cg15757430	TMEM219;TMEM219;TME	blue	-0.517059	-0.6222585	0.09831129	0.07010528	0.46479233	-0.0478772
cg05619021	LAMA1;LAMA1;LAMA1;L	blue	0.67454273	0.78814837	-0.1534535	0.09191433	-0.3700716	0.20696488
cg23498494	CLDN12;CLDN12;CDK14;C	blue	0.38445953	0.82354099	0.13356632	-0.0120755	-0.7155262	0.11945203
cg02577049	TMEM52;TMEM52;TMEM5	blue	-0.5392107	-0.6225321	-0.0335368	-0.0245925	0.49786493	-0.2120383
cg04939673	WWC2-AS2;WWC2-	blue	-0.5200838	-0.7531024	0.06354315	-0.0877646	0.49011199	-0.0948058
cg10406690	RP11-497E19.1;RP11-	blue	-0.3325718	-0.3938132	0.17664094	0.03898345	0.30740922	-0.066452
cg20321998	NME5;NME5;NME5	blue	0.47857016	0.74406295	-0.3012813	0.10686896	-0.3566898	0.02172953
cg25824816	PTH1R;MYL3	blue	0.52308842	0.81842482	-0.0851636	0.1087656	-0.470277	0.1413169
cg15766962	SLC44A3;SLC44A3;RP11-	blue	0.5059542	0.83132254	-0.1603324	0.13154555	-0.458779	0.00982122
cg07913976	SEMA6A;SEMA6A;SEMA6	blue	0.59632289	0.66112725	0.02418805	0.10456371	-0.3457211	0.18954371
cg14134364	OR2L8;OR2L13	blue	0.67678048	0.77009353	-0.051876	0.16481692	-0.2994767	0.14619759
cg06999661	RP5-842K16.2	blue	0.42236443	0.80298213	0.10110625	0.06428964	-0.6601748	0.02620215
cg19063688	AC016722.2;AC016722.3;A	blue	0.42771045	0.80564636	-0.0930599	0.00613498	-0.5582061	0.07816933
cg17257731	FGF12;FGF12;FGF12;FGF1	blue	0.31983613	0.61342478	-0.1886907	0.03938456	-0.3381069	-0.0610229
cg16513286	IQUB;IQUB;IQUB;IQUB;IQ	blue	-0.4549231	-0.5029535	0.28496243	0.01686707	0.35680767	-0.0895987
cg24831168	RP11-98J9.2	blue	0.62080961	0.77819737	-0.1388008	0.1647939	-0.3174095	0.07520287
cg09830542	AC069082.1	blue	0.46754614	0.75770527	0.20282654	0.05431597	-0.5111319	0.11798879

cg00136547	DST;DST;DST;DST;DST;DS	blue	0.57330746	0.83479283	0.18614574	0.17138674	-0.472484	0.12128839
cg00016439	ST8SIA3	blue	0.71580471	0.71233529	0.28542153	0.21327969	-0.355562	0.34334154
cg18162838	HK1;HK1;HK1;HK1;HK1;H	blue	0.61961812	0.7210268	0.26521627	0.14400315	-0.4401375	0.08893802
cg09439489	ZFYVE28	blue	0.50746658	0.76067097	-0.1590403	0.10235022	-0.4202975	0.08449198
cg14834881	AC004054.1;FAM190A	blue	0.51093198	0.61888406	-0.1363897	0.11715318	-0.2097182	0.01190333
cg23167606	TRAV8-1	blue	0.24869837	0.43414591	-0.1000345	-0.1073109	-0.3973555	-0.0138808
cg20266436	Y_RNA	blue	0.65162744	0.70690046	-0.0387155	0.19109643	-0.2488082	0.10452315
cg07524855	RP11-315I14.2	blue	0.32832485	0.693362	-0.0451839	0.10429195	-0.3962536	-0.0141084
cg27099692	CLDN10	blue	0.65511615	0.80824961	-0.0280649	0.1727122	-0.4404032	0.20218884
cg24238929	RP11-506G7.1;RP11-	blue	0.6749224	0.851723	0.00706035	0.15862843	-0.4256264	0.20420304
cg13545696	ALDH1A2;ALDH1A2;ALD	blue	0.68063707	0.6500732	0.39538117	0.19303211	-0.3386048	0.36220515
cg24407589	HBM;HBM;HBM	blue	-0.401718	-0.5094821	0.12328338	-6.33E-05	0.37733381	-0.0370802
cg20281815	PSCA;PSCA;JRK;PSCA;PS	blue	0.43923217	0.65478966	0.0036988	0.14716511	-0.36094	0.05197017
cg05996204	RP11-669M16.2;RP11-	blue	0.50008519	0.79330321	-0.1392871	0.11718211	-0.4135098	-0.0456757
cg03871460	CCNA1;CCNA1	blue	-0.3176911	-0.4326878	0.38255344	0.04255461	0.3177454	0.03259599
cg24591615	AC006076.1	blue	0.39534753	0.7916297	0.07733474	-0.0106405	-0.6162515	0.04208843
cg09556715	U6;HAPLN1;HAPLN1	blue	0.56335138	0.80286994	-0.2294003	0.11994851	-0.3739677	-0.0090459
cg20220755	TRBV4-1	blue	0.54712036	0.82958206	-0.046697	0.08662309	-0.5031738	0.05533077
cg25276457	KIAA0146;KIAA0146;KIA	blue	0.54061484	0.57381296	-0.2308254	0.09508531	-0.2114267	0.13536252
cg07490719	AC007246.3;AC007246.3;A	blue	0.45389288	0.89805751	-0.0837735	0.03909743	-0.6660016	0.00628093
cg02287817	EGR4;EGR4	blue	0.38222947	0.5410551	-0.0247558	0.06761136	-0.3459752	0.09848969
cg22072332	MUC15;MUC15;MUC15;M	blue	0.3073195	0.64624204	-0.038159	-0.0421283	-0.5183597	-0.05653
cg23338195	SLC30A8;SLC30A8;SLC30	blue	0.40534157	0.75666449	0.01780518	0.0696439	-0.5132071	0.01137733
cg23970989	RP11-1197K16.2;RP11-	blue	0.60754446	0.60870674	0.20881722	-0.0127244	-0.4058912	0.21682658
cg05202424	SNORD114-13;AL132709.8	blue	0.56280971	0.78899483	-0.0309299	0.09401471	-0.4396173	0.18948063
cg12811284	OR6K3;OR6K3	blue	0.33877516	0.80913308	-0.1552359	-0.0371101	-0.6930815	-0.1001518
cg16073862	RP11-89N17.3	blue	0.39344448	0.86486069	-0.1719203	-0.0299336	-0.6754708	-0.0712779
cg06483266	U3	blue	0.47542971	0.90049096	-0.129501	0.00644739	-0.6545156	0.02676875
cg20723459	RP11-686F15.2	blue	0.4748579	0.53071131	0.26546635	-0.019455	-0.4583967	0.0897996
cg11814836	CIB4	blue	0.39158803	0.62662871	-0.1668441	0.07590761	-0.3041066	0.04909303
cg10582773	CALN1;CALN1;CALN1;CA	blue	0.55649361	0.79340513	-0.2985915	0.08501952	-0.4683393	0.01011674
cg17897843	RP11-6N13.1;RP11-6N13.1	blue	0.56051409	0.66507079	-0.398621	0.11173123	-0.2079165	0.02872948
cg15429002	AC007099.1;FAM176A;FA	blue	0.67545985	0.8536769	-0.0541776	0.1492431	-0.4491997	0.16640322
cg01080915	PROC;PROC;PROC	blue	0.48788083	0.58672014	-0.1148889	-0.0107794	-0.2774326	0.01854469
cg00865173	RP11-116D17.2	blue	0.5027409	0.79108698	-0.0969246	0.08042932	-0.4066005	0.08693713

cg04069811	IMMT	blue	0.56087645	0.71904585	0.07509628	0.17083796	-0.4717719	0.23460496
cg11457267	PLOD1;PLOD1;PLOD1;PLO	blue	-0.4531812	-0.7267872	-0.0475621	0.05273949	0.64099733	-0.1877072
cg02400506	RP11-490G2.2	blue	0.5849252	0.82234261	-0.125862	0.19365093	-0.3604591	0.069883
cg03019224	ACAA2	blue	0.64128103	0.65241607	-0.0562508	0.16553174	-0.331737	0.23848139
cg13105228	CADM1;CADM1;CADM1;C	blue	0.37022453	0.84264487	-0.1313222	-0.046933	-0.6514078	-0.0372086
cg02429476	CTC-293G12.1	blue	0.5115152	0.80175609	-0.3371232	0.10591426	-0.4305343	-0.0575976
cg23180235	LMCD1;LMCD1;LMCD1;A	blue	0.62830077	0.8073375	0.02700295	0.15250079	-0.4328037	0.15462494
cg03783060	RP1-251M9.2	blue	0.64319648	0.7953357	0.02440067	0.10209813	-0.462899	0.22613772
cg13937257	KRT18P32	blue	0.51770213	0.83088107	-0.038034	0.19192544	-0.4565045	0.0564977
cg03827102	FAM3B;FAM3B;FAM3B;T	blue	0.37326556	0.79706587	0.02026057	-0.0442573	-0.7219013	0.04721655
cg18503031	MITF;MITF;MITF;MITF;MI	blue	0.62787864	0.84971457	0.09253608	0.1349147	-0.5277321	0.1845018
cg13001127	RP11-677I18.4	blue	0.51782649	0.82140384	-0.2128707	0.08548459	-0.4493336	-0.0420431
cg21485303	ABCC9;ABCC9	blue	-0.4107562	-0.6191617	0.31490747	0.06288176	0.53526165	-0.0146628
cg09132846	TMEM139;AC073342.12	blue	0.5227719	0.76052641	0.26595529	0.08304631	-0.6007064	0.11261128
cg07542184	TTC29;TTC29;TTC29	blue	0.36175836	0.75262481	-0.3158339	0.02553718	-0.4900727	-0.1527914
cg12118465	EFHD1;EFHD1;EFHD1	blue	-0.3885104	-0.7107417	0.22638727	0.02158583	0.55124125	0.11940667
cg15787623	OR2L13;OR2L13	blue	0.65870961	0.82478328	0.00584826	0.13500752	-0.3996428	0.25309829
cg27544223	FAXC;FAXC	blue	-0.352961	-0.5213107	0.31763401	-0.0262684	0.3011507	0.13968143
cg11780785	PMFBP1;PMFBP1;PMFBP1	blue	0.29760422	0.80572929	-0.1201314	-0.1525533	-0.8045954	-0.05061
cg18330398	ZC3H12C	blue	0.50902118	0.86371312	0.08311437	-0.0023191	-0.6575019	0.0976463
cg04897080	EXOC3L1;EXOC3L1	blue	0.60072201	0.70248471	-0.1374841	0.1424839	-0.325277	0.24070994
cg17257934	RP11-638F5.1;RP11-638F5.1	blue	0.31804805	0.79276302	-0.1919642	0.01640232	-0.5729295	-0.1082986
cg22971191	SLC10A2	blue	0.3626449	0.50001586	-0.106875	-0.1226931	-0.376427	0.05028024
cg00037188	RP11-	blue	0.62829404	0.7238189	0.23809533	0.10618241	-0.3725959	0.17052831
cg27205487	MIR125B2;LINC00478;LIN	blue	0.61714024	0.78733846	0.16345226	0.18358861	-0.4422811	0.21588269
cg12794758	ADORA1;ADORA1;RP11-	blue	-0.3020957	-0.3601748	-0.0441183	0.08047446	0.31060385	0.02656659
cg19658834	RP11-486F17.1;SATB2	blue	0.48978278	0.86618372	-0.1937676	0.08633567	-0.5224845	0.01678742
cg10060625	U6	blue	0.49709037	0.85921986	0.05599326	0.09279032	-0.637218	0.06125427
cg00729133	C7orf62	blue	0.35751673	0.69367832	-0.3014307	-0.0537593	-0.5173275	-0.1820082
cg26119470	AZGP1;AZGP1;AZGP1	blue	0.51460038	0.77892337	-0.0245628	0.10931457	-0.4325227	0.17740741
cg04879561	ADCYAP1R1	blue	0.69232384	0.6849292	-0.1206229	0.15537521	-0.2423832	0.22297721
cg03785728	BANF2	blue	0.59928138	0.78775625	-0.0684586	0.13591305	-0.4058497	0.16962141
cg09228646	RP11-174I12.2	blue	0.43490673	0.57642526	0.10699162	0.01607787	-0.2687489	0.13581734
cg21949712	DLX6-	blue	-0.407505	-0.7379801	0.21415615	0.02739378	0.5902181	0.01312916
cg21392202	RP11-364B6.1	blue	0.50249121	0.82319839	-0.0858287	0.11124986	-0.4147578	0.03517671

cg23260221	AFAP1L2;AFAP1L2;AFAP1	blue	0.51718686	0.78466196	-0.0923731	0.12591499	-0.3474562	-0.0460033
cg11888886	RP11-213G6.2;RP11-	blue	0.7529521	0.78210187	0.15060659	0.19876092	-0.3320206	0.2539206
cg23154806	RP11-373J16.1	blue	0.41451805	0.86320481	-0.1937647	-0.0496135	-0.6397063	-0.0463951
cg00322656	PDE4D;PDE4D;PDE4D;PDE	blue	-0.2786201	-0.374426	0.1905508	0.08892155	0.24499546	0.09411997
cg04438377	GPR161;GPR161;GPR161;G	blue	0.45759592	0.76459896	-0.1084115	0.12670438	-0.5454045	0.04582337
cg15802323	GCC1;ARF5;ARF5;ARF5;A	blue	-0.4233357	-0.6392327	0.06564639	0.03848643	0.54536902	-0.0348392
cg26545268	PKHD1L1	blue	-0.2749169	-0.5487798	0.37052763	0.12458811	0.38150362	0.18373754
cg06600911	C11orf92;C11orf92;C11orf93	blue	0.69854404	0.72088954	0.28958947	0.11223822	-0.4316532	0.33463269
cg07010146	CALHM3	blue	0.52241691	0.66143731	-0.1022412	0.12893163	-0.3703109	0.21541388
cg23321703	NDST1;NDST1;NDST1;ND	blue	0.4675047	0.75043539	0.03227533	0.03485632	-0.5195657	0.22639267
cg01918492	AC022816.2	blue	0.51224542	0.78085366	-0.2214765	-0.019075	-0.452198	0.02282264
cg15446373	KRT35	blue	0.32541773	0.81655916	-0.1776099	-0.0886459	-0.6654927	-0.0192258
cg00195402	MYOF	blue	0.31578316	0.86082037	-0.1227788	-0.0108794	-0.74507	-0.0335462
cg17124700	RGS8;RGS8;RGS8;RGS8;R	blue	0.72602379	0.68838113	0.17867967	0.19873157	-0.3151066	0.29686309
cg23325423	PDGFD;DDI1	blue	0.5344218	0.6301543	-0.0227274	-0.0214203	-0.3959588	0.16719289
cg21163972	ARL8A	blue	0.48473394	0.78424145	-0.1390563	-0.0032964	-0.5324735	0.03044513
cg21727129	HLA-G;HLA-G	blue	0.52357402	0.8553838	-0.006392	0.01281856	-0.5709782	0.08860284
cg10973400	ARHGEF19-AS1	blue	0.64700528	0.8342962	-0.0502366	0.15627887	-0.4701209	0.19052869
cg18508824	THAP6;THAP6;RCHY1;TH	blue	0.31727563	0.78879528	0.11193496	-0.0422588	-0.757171	0.09441917
cg04078369	RP11-76I14.1;RP11-	blue	0.51251969	0.76499537	-0.1234621	0.08021514	-0.3868762	0.01820476
cg16361253	GPR111;GPR111;GPR111;G	blue	0.51918274	0.64667495	0.05749778	0.18149494	-0.2606542	0.20416491
cg13506158	UTS2D;UTS2D;UTS2D;UTS	blue	0.25446568	0.65829192	-0.1116586	-0.1466807	-0.6317564	-0.0180208
cg06977538	SVEP1	blue	0.63361277	0.79862792	0.05961503	0.19607489	-0.4348222	0.21034743
cg26693693	AC105760.3	blue	0.76368038	0.79785246	-0.0029954	0.19773237	-0.4256755	0.28125557
cg11131752	PLA2G6;PLA2G6;PLA2G6;	blue	0.37509507	0.67162748	-0.1970496	0.05074759	-0.5233663	0.0046704
cg17415790	PTPN3;PTPN3;PTPN3	blue	-0.4046613	-0.6860817	0.2224331	0.02299923	0.48717073	0.02997843
cg08967204	TTC7B;TTC7B;TTC7B	blue	0.58630241	0.65236255	-0.1433787	0.12244684	-0.3294423	0.20993404
cg18947008	GPR39	blue	0.70248668	0.79187479	-0.1368228	0.14316669	-0.294061	0.09225732
cg25971170	RP11-59J5.1;RP11-59J5.1	blue	0.28314228	0.74935652	-0.3649879	-0.0104923	-0.4942777	-0.1615441
cg17066785	ELMO2;RP5-981L23.1	blue	0.45807142	0.85969247	-0.0505421	0.0299547	-0.6099767	0.0478445
cg14068796	XXbac-BPG308K3.6	blue	-0.5512279	-0.5982228	0.11982417	0.00192243	0.37463221	-0.3039346
cg11244151	TMEM108;TMEM108;TME	blue	0.48555346	0.76815776	-0.1336118	0.1119369	-0.3700552	-0.0416441
cg02996939	TRIM29;TRIM29;TRIM29;T	blue	0.55099308	0.75194885	-0.0816983	0.05394872	-0.4512576	0.1794855
cg25135333	SEC16B;SEC16B;SEC16B;S	blue	0.64697761	0.66121171	0.08743405	0.1401193	-0.2837782	0.19995289
cg02289040	SEC16B;SEC16B;SEC16B;S	blue	0.54919951	0.78740258	-0.047572	0.05397423	-0.5136688	0.1880036

cg20873570	NKX2-6;NKX2-6;RP11-	blue	-0.4713765	-0.7464281	0.27083293	0.00821576	0.58099133	-0.0282315
cg09146813	ZNF354B;ZNF354B;RP11-	blue	0.38713802	0.58455744	-0.0965961	-0.0073086	-0.446085	-0.0093756
cg20527089	HS3ST1;HS3ST1;HS3ST1	blue	-0.4142258	-0.5059435	0.02135388	0.02469065	0.37669158	-0.0288753
cg25315819	ZSWIM5	blue	0.34816857	0.81937292	-0.0817502	-0.1303578	-0.7902842	-0.0152431
cg05349042	BCL9	blue	-0.5192642	-0.6954122	0.02710046	-0.1373691	0.46106484	-0.2428593
cg21605540	PEMT;PEMT;PEMT	blue	0.51832053	0.74889527	0.03600898	0.08535786	-0.4664376	0.26769057
cg12842552	IGSF10	blue	0.52286471	0.87339173	-0.1142167	0.05929408	-0.574078	0.00538475
cg14326909	RP1-60O19.1;RP1-	blue	0.51554149	0.87196972	-0.020897	0.03193047	-0.6719771	0.11391385
cg25099853	STX7;STX7;STX7;STX7	blue	0.58153308	0.65866047	-0.2828064	0.08451355	-0.3653028	0.00374304
cg15802639	FHL5;FHL5	blue	0.52659753	0.6491331	-0.2316809	0.14260991	-0.240903	-0.0176793
cg07725825	CEP152	blue	0.4309249	0.6921475	-0.0770556	-0.0122728	-0.5458042	0.0745322
cg22741651	7SK;PID1	blue	0.42075236	0.78019139	-0.2494268	0.03847046	-0.4536199	-0.0135799
cg11320725	OASL;OASL;OASL	blue	0.43017159	0.78381406	-0.0352017	0.02812831	-0.5930012	0.06840317
cg00385839	C20orf78	blue	0.29727112	0.7344911	-0.3130044	0.03341357	-0.5281515	-0.1529465
cg15377585	TMEM196;TMEM196;TME	blue	-0.5886618	-0.7587541	0.21083254	-0.0931188	0.44577666	-0.0743611
cg06031301	PLCG2;PLCG2;PLCG2;PLC	blue	0.48610454	0.69991427	-0.0809094	0.06935636	-0.5149203	0.12167271
cg06730285	SLC35A4;APBB3;SLC35A4;	blue	0.27623292	0.73123852	-0.1690682	0.05982805	-0.5806743	-0.1310508
cg02210887	GUCY1A3;GUCY1A3;GUC	blue	0.44406457	0.69176847	-0.2887934	0.11542824	-0.2732203	-0.0431439
cg02203103	LAG3;LAG3	blue	0.52775436	0.82892054	0.02785062	0.06480138	-0.5934533	0.12477873
cg03928182	PTPRR	blue	-0.3684856	-0.6579938	0.33494571	-0.0323077	0.45812291	0.14832008
cg09583780	NMUR2	blue	0.3105947	0.62493943	0.25321247	0.0881182	-0.4523715	0.02474489
cg02648389	SLC30A5;SLC30A5;SLC30	blue	0.64520111	0.85115859	-0.005362	0.11594015	-0.5108855	0.19885182
cg27510065	RP11-84E24.3;RP11-	blue	0.2958988	0.75704875	-0.1450237	-0.0297335	-0.6235293	-0.0220479
cg09868882	GRM8;GRM8;GRM8;GRM8	blue	0.6774563	0.76903592	-0.0412307	0.22201516	-0.2763261	0.16297572
cg18110200	TIAM2;TIAM2	blue	0.32839634	0.85313963	-0.0761494	0.01212948	-0.6833891	-0.0837855
cg07728036	TMPRSS12;TMPRSS12	blue	0.44479481	0.83682847	0.02421581	0.04190065	-0.6586148	0.07585634
cg02779215	U6	blue	0.62952504	0.76591105	-0.1664482	0.15202504	-0.2701489	0.21662037
cg20313628	RAD51AP2	blue	0.35950099	0.4785037	-0.2095705	0.01070041	-0.1974946	-0.0468843
cg03028148	CTD-2647E9.3;CTD-	blue	0.70078645	0.77458519	-0.0128472	0.26158243	-0.2934596	0.2115099
cg27439413	AP001271.3	blue	0.65680244	0.75176689	0.17006891	0.1341952	-0.4164716	0.16072788
cg17838626	ACER3;ACER3;ACER3;AC	blue	-0.4363234	-0.6057557	0.18954955	0.04327305	0.41760595	-0.0012004
cg10082500	PCBP2;PCBP2;PCBP2	blue	0.64419473	0.70694703	-0.1333112	0.16867116	-0.3316268	0.08271227
cg00087055	RPTN	blue	0.50311208	0.70766984	-0.2538402	0.06500826	-0.2822377	-0.0450004
cg10177030	ZNFX1-AS1;ZNFX1-	blue	0.25786395	0.80401195	-0.1046306	-0.0673442	-0.7962684	-0.0084706
cg08426081	ZNF691;ZNF691;ZNF691;Z	blue	0.38364326	0.85425104	0.03000954	-0.0854776	-0.7929475	-0.0118932

cg03950873	C14orf38	blue	0.32548844	0.85965519	-0.0947493	-0.0940252	-0.7754642	-0.0227395
cg08040962	FCRL1;FCRL1;FCRL1;FCR	blue	0.43167244	0.75705185	-0.113277	0.11697119	-0.5148615	0.08175551
cg04098176	TSPYL6;ACYP2	blue	0.64875081	0.76690462	-0.0546235	0.14863303	-0.3674314	0.2197968
cg11417528	RP11-543G18.1	blue	0.35643263	0.6446038	-0.0005791	0.0341799	-0.5481086	0.09579775
cg25115154	TSPAN8	blue	0.21621882	0.4572746	0.05807829	-0.064815	-0.3678447	-0.013785
cg10876776	RP11-298E9.5	blue	0.66463597	0.75426025	0.0484338	0.22209301	-0.3200398	0.13175162
cg02046532	DEFB129	blue	0.26824991	0.80491938	-0.0840426	-0.1699314	-0.8027956	-0.0870399
cg19914336	AC024028.1	blue	0.44749537	0.77379491	-0.2555535	0.04838157	-0.4699725	-0.0982728
cg00713825	CD163L1;CD163	blue	0.40769666	0.82375298	-0.1690648	0.04059871	-0.5539199	-0.0377657
cg02729601	PON3;PON3;PON3;PON3	blue	0.30251938	0.8003151	-0.1627764	0.04347943	-0.5915247	-0.0254746
cg24354664	RP11-120K18.2;ITGAD	blue	0.68491385	0.65169507	0.09717911	0.14012885	-0.3034554	0.27640303
cg23934295	RP11-497E19.1;RP11-	blue	-0.2746799	-0.3755766	0.16380538	0.09440056	0.23567819	0.05733531
cg05643453	CASQ1;CASQ1;CASQ1	blue	0.37884387	0.8810039	-0.1413163	-0.0591421	-0.735174	-0.0455244
cg18492996	RP11-116N8.4	blue	0.38487491	0.68930993	-0.2301923	0.08000969	-0.415661	-0.0498347
cg23459573	RP11-	blue	0.23589537	0.79293866	-0.0850775	-0.0803849	-0.750842	-0.06902
cg07777964	FRMPD1;FRMPD1;RP11-	blue	0.48677857	0.87473426	-0.0501598	0.09877653	-0.5481376	0.09912057
cg03680422	MAP1A;MAP1A;MAP1A	blue	0.58627982	0.69708904	-0.2522672	0.10367724	-0.3639618	0.13137799
cg10923956	RP11-295D22.1	blue	0.52967156	0.76582018	-0.2027717	0.06169018	-0.380106	0.00903583
cg17114950	STARD13-AS1	blue	0.6817694	0.72318319	0.21730822	0.18086725	-0.3024573	0.22637671
cg12501586	RP1-40E16.9;RP1-40E16.9	blue	0.58043662	0.82141368	0.15800234	0.05577275	-0.5985427	0.20972143
cg19101823	RP4-614C15.2	blue	0.70074372	0.75001216	-0.0345874	0.16212189	-0.3330757	0.26353169
cg25003014	RBFOX1;RBFOX1	blue	0.40554792	0.58511955	-0.2479337	0.0928396	-0.2953662	-0.0717632
cg08861115	IL36G;IL36G;IL36G	blue	0.3955383	0.82206179	0.12472139	-0.0296943	-0.7428949	0.08846077
cg08064085	4-Sep	blue	0.45795335	0.79680559	-0.2302146	-0.0387005	-0.5505226	0.00157336
cg26427115	C2orf70;C2orf70	blue	-0.4828214	-0.6212521	0.25350327	-0.0294167	0.51075411	-0.0385668
cg00378730	CHAT;CHAT;CHAT;CHAT;	blue	-0.5103436	-0.6119139	0.13166872	0.12693935	0.4370553	-0.1417069
cg24706930	U2	blue	0.68100354	0.69963163	-0.1472022	0.11385918	-0.3047002	0.22639751
cg18954869	TFAP2B	blue	-0.48068	-0.6256917	0.33624207	0.00611122	0.42283859	-0.0319288
cg17917176	AC037487.1	blue	0.46375844	0.72508873	-0.3165108	0.07849885	-0.3666262	-0.0076754
cg10399946	RPS18;RPS18;RPS18;RPS18	blue	0.29994031	0.7943872	-0.0639245	-0.0213053	-0.6559245	-0.1016221
cg20461912	RP11-	blue	-0.4815124	-0.7401302	0.14997894	-0.043901	0.53040796	-0.0403958
cg17754914	SCN3A;SCN3A;SCN3A;SC	blue	0.41596421	0.8076293	-0.0687606	0.0438431	-0.5213278	0.0607679
cg02900223	KCNAB1;KCNAB1;KCNA	blue	-0.4124856	-0.5823661	0.03981825	0.0451016	0.42669545	-0.1803269
cg02709812	RP11-165I9.8;RP11-165I9.4	blue	0.41195772	0.54866855	0.17043529	0.12571243	-0.2670636	0.13496471
cg06015599	AKAP12;AKAP12	blue	0.60356297	0.57708247	0.26669466	0.15392776	-0.2653157	0.17459328

cg14264566	SLC6A13;SLC6A13;SLC6A	blue	0.53005095	0.60927025	0.06970672	0.10211197	-0.3502221	0.31359176
cg12091975	U66059.29	blue	0.3453608	0.87557619	-0.1244297	-0.0093861	-0.6923101	-0.0673472
cg01879161	KIAA1211;KIAA1211;KIA	blue	0.45319542	0.79180026	-0.097041	0.08180626	-0.4694834	0.09457264
cg05389237	OR1N2	blue	0.43022183	0.70758962	0.06999463	0.00177606	-0.4762095	0.09717587
cg18624059	GALNT14;GALNT14;GALN	blue	0.69514223	0.81094578	0.05685581	0.19189279	-0.4328747	0.27879027
cg00600604	RP11-889D3.1	blue	0.41993955	0.63387327	-0.3127688	-0.0501032	-0.3738506	-0.0230623
cg14841408	CTD-2024D23.1;CTD-	blue	0.42509926	0.86883054	-0.1967242	0.00483949	-0.6111205	-0.0929178
cg04902327	TRIM58	blue	0.63596453	0.73421414	-0.1061436	0.1502497	-0.2739417	0.17377837
cg14839085	PHYHIP	blue	0.66756883	0.65387973	0.07338907	0.16404801	-0.2143341	0.23540143
cg02027791	TRAV9-1	blue	0.36852531	0.7801452	0.14465804	-0.0508739	-0.6923896	0.03568314
cg12820291	Y_RNA	blue	0.44148447	0.78506583	-0.066757	0.07705072	-0.5485655	0.09930141
cg22510442	TMEM167B;TMEM167B;T	blue	-0.5608672	-0.6519585	-0.0645097	-0.0772604	0.40529022	-0.1423598
cg13632064	PAOX;MTG1;MTG1;PAOX;	blue	0.40153276	0.80336214	-0.0549557	-0.0610256	-0.7110658	0.11634154
cg02951514	SLA;SLA;SLA;SLA;SLA;SL	blue	0.53248758	0.6189659	-0.0738582	0.03129061	-0.2890291	0.16194064
cg06256838	WLS;WLS;WLS;RP11-	blue	0.44922274	0.89701781	0.07339896	0.00759534	-0.7158832	0.08420949
cg01062300	AC107070.1;AC107070.1;A	blue	0.3352806	0.80411548	-0.2926704	-0.0138773	-0.5797788	-0.0801916
cg05296643	HOXC5;HOXC6;HOXC4;H	blue	-0.4998305	-0.6414633	0.17302159	0.00851666	0.44105292	-0.0245752
cg25987102	APOA1;APOA1;APOA1;AP	blue	0.61305004	0.81986539	-0.0361236	0.10380383	-0.4602459	0.10868921
cg03312205	COPS7A;COPS7A;COPS7A	blue	0.31851323	0.4629556	-0.0480466	-0.0921768	-0.3640328	-0.0162162
cg23346801	CFB;CFB;CFB;CFB;CFB;CF	blue	0.50629728	0.65726282	0.01768285	0.02474849	-0.4637126	0.1808997
cg23769296	CELF6;CELF6;CELF6;RP11-	blue	0.51453533	0.83704056	-0.061189	0.09726633	-0.5376005	0.1176114
cg27480064	ADORA1;ADORA1;RP11-	blue	-0.5470364	-0.5902453	-0.004699	-0.1303144	0.36649249	-0.1587957
cg25946758	PHYHIPL;PHYHIPL;PHYH	blue	-0.5526918	-0.7844247	0.10488348	-0.0536673	0.48036799	-0.0294739
cg11975608	LPA	blue	0.46642594	0.59392637	0.04114639	0.11403711	-0.1899169	-0.0260022
cg00846136	MIR1302-	blue	0.26998646	0.80303705	-0.1831899	-0.110972	-0.7257631	-0.0455843
cg02968741	GABRB1;GABRB1;GABRB	blue	-0.4353389	-0.7724597	0.27456144	-0.0510762	0.56463808	-0.0083924
cg09578509	SLC19A3;SLC19A3;SLC19	blue	0.43529565	0.83840391	-0.095017	0.02185514	-0.5509706	-0.072487
cg23366074	ARHGAP22-	blue	0.73392135	0.75313184	-0.0633194	0.19312846	-0.3770445	0.28689042
cg11380134	DROSHA;DROSHA;DROS	blue	0.30525679	0.71535672	-0.1713651	0.04635536	-0.5956439	-0.0015113
cg25583982	RP11-419C23.1	blue	0.52243038	0.82532815	-0.2033612	0.07133306	-0.5300593	0.09049872
cg15084437	RP4-650F12.2;RP4-	blue	0.42227839	0.6165227	-0.3114818	0.14604202	-0.2237963	-0.061812
cg04672418	KCNA5	blue	0.62910177	0.81713659	-0.0497833	0.17776296	-0.394464	0.13934257
cg11055449	IFIT1B	blue	0.52196046	0.89779578	0.12510438	0.083398	-0.6223427	0.11840964
cg19725044	SAP130;SAP130;SAP130;S	blue	-0.5357426	-0.6623636	-0.0231788	-0.0700404	0.45441882	-0.1283093
cg18217706	TFF3;TMPRSS3	blue	0.38236768	0.62403014	-0.2123958	0.01112484	-0.3720791	-0.1047305

cg17030437	EFCAB8	blue	0.72291828	0.71045387	-0.0409735	0.16872659	-0.2632474	0.24146202
cg17303068	SMAD3;SMAD3;SMAD3;S	blue	0.46959315	0.53914811	-0.1674926	0.03208824	-0.3380808	0.1487881
cg02554564	NTF3;NTF3;NTF3	blue	0.35146899	0.48413666	-0.2302131	0.03910899	-0.2211212	-0.0714085
cg12300178	RP11-231I13.2	blue	0.34725935	0.81401083	-0.2024569	0.03132007	-0.5771193	-0.1515626
cg19415383	DSG1	blue	0.51747181	0.82086049	-0.0646799	0.14054715	-0.4390659	0.04176124
cg16565579	AC013448.1	blue	0.51396144	0.7206476	-0.12088	0.09100995	-0.3686862	0.06112546
cg26120226	RP11-	blue	0.48966743	0.89724226	-0.0536714	0.08822888	-0.6000325	0.04210719
cg19254312	STON1;STON1-	blue	0.56177	0.6970132	-0.1155456	0.16536367	-0.3129778	0.10049186
cg26502429	MEIS3;MEIS3;MEIS3;MEIS	blue	-0.497235	-0.7181424	0.21581355	-0.0636019	0.4921971	-0.0554776
cg26184695	KLF15;KLF15	blue	0.56690668	0.70325211	-0.1870817	0.13966135	-0.2647565	0.12221967
cg12555975	AC092786.1	blue	0.3877031	0.83552931	-0.0957472	0.03141261	-0.6292964	0.01244308
cg16273469	RP4-669B10.3	blue	0.62849726	0.81071404	0.10412946	0.14227195	-0.4196298	0.09525362
cg21278181	PRHOXNB	blue	0.49616373	0.57055838	-0.1365651	0.04490364	-0.1628066	-0.0171308
cg21322563	S1PR5;S1PR5;S1PR5	blue	-0.4982629	-0.6313966	0.05826322	-0.0098164	0.52525454	-0.1362405
cg08747931	RP11-191N8.2	blue	0.43926667	0.54325465	-0.3177334	0.01483029	-0.2406901	-0.03362
cg15329866	PLD1;PLD1	blue	0.49707641	0.68224709	0.41493674	0.13087493	-0.4746756	0.15610114
cg25233516	LECT1;LECT1	blue	0.71700845	0.80376228	-0.0350795	0.20068152	-0.3553383	0.19457316
cg19895786	SLC30A2;SLC30A2;SLC30	blue	-0.4977937	-0.690501	0.17342677	-0.0648179	0.47627087	0.02197113
cg24069701	U6atac	blue	0.50644889	0.68809275	-0.0931536	0.04777263	-0.4385103	0.11195133
cg14472888	AC104777.1;AC104777.4	blue	0.58485708	0.87436094	-0.0901278	0.1316435	-0.4815451	0.04602411
cg10690427	RP11-416N4.1;RP11-	blue	0.69822055	0.73353693	0.25389407	0.18562974	-0.3414803	0.30288691
cg02650170	AC093304.1;ODZ2;ODZ2	blue	0.32854545	0.67638352	0.03173024	-0.0683925	-0.5197497	-0.0995914
cg19530295	F11R;F11R;F11R	blue	0.73279424	0.72255963	-0.08044	0.20301772	-0.2768765	0.30831911
cg15211598	NFASC;NFASC;NFASC;NF	blue	0.72766618	0.84642907	0.0547642	0.21792557	-0.3943167	0.23958036
cg18884670	Y_RNA;MED20;MED20;ME	blue	0.26761337	0.60664894	0.06749132	-0.0651706	-0.4127905	-0.0378939
cg02077084	OR2T6	blue	0.6157809	0.76358657	-0.0626614	0.12218518	-0.3909806	0.13463251
cg20939319	TEX15;TEX15	blue	0.32471114	0.84502601	-0.0021028	-0.0420547	-0.7286132	-0.0058078
cg14104072	FAM45A;FAM45A;FAM45	blue	0.73716182	0.71681374	0.10671236	0.29177108	-0.279787	0.27326291
cg13169939	SNRNP200;SNRNP200;SNR	blue	0.52127877	0.60554643	-0.2161586	0.14528672	-0.2488429	0.06870016
cg20662786	AP000355.2;UPB1	blue	0.61410306	0.81880083	0.00673863	0.0793496	-0.4927392	0.2857501
cg12705392	RP11-125M16.1	blue	0.31030693	0.85052533	0.01755734	-0.0723702	-0.7746446	-0.0609294
cg17070923	CTD-2589M5.4	blue	0.63513017	0.70793135	0.32834376	0.18442419	-0.3545567	0.25536474
cg04143750	EFHD1;EFHD1;EFHD1	blue	-0.342605	-0.603295	0.31097398	0.10822862	0.46334779	0.03437338
cg09266113	DIO1;YIPF1;YIPF1;YIPF1;	blue	0.23335976	0.42454441	-0.2481911	0.11815155	-0.2940362	-0.0325048
cg16919501	RP11-16L9.4;RP11-16L9.3	blue	0.4533561	0.73112148	-0.3143035	0.00668282	-0.4680547	-0.0829086

cg00526628	TMEM132B	blue	0.35122877	0.81956969	-0.0789724	-0.1123181	-0.7221302	-0.032805
cg20299313	SLC13A3;SLC13A3	blue	0.65595388	0.83938852	0.02178646	0.21526365	-0.4081558	0.24831985
cg14420691	TRHDE	blue	0.51500221	0.81634978	-0.281809	-0.0077125	-0.4522103	0.04055862
cg01360231	AC130710.1;AC130710.1	blue	0.60416894	0.82426778	-0.0713373	0.09342371	-0.4911848	0.12196828
cg00651401	TNK2;TNK2;TNK2;TNK2;T	blue	-0.4769858	-0.5744079	0.18611288	-0.0558486	0.45252947	-0.1151213
cg04075793	AC113607.2;AC113607.2	blue	0.72849131	0.76286151	-0.0738695	0.24766685	-0.2630339	0.22647702
cg18139245	AC005592.3	blue	0.53645256	0.85911491	0.13500974	0.0837397	-0.6229458	0.16826076
cg09493943	WFDC5;WFDC5	blue	0.53757868	0.83309303	-0.2367335	0.13748191	-0.4938165	0.04377932
cg17565478	TRIML2;TRIML2;TRIML2;T	blue	0.63011302	0.70585459	-0.1142835	0.15176137	-0.2497545	0.17507413
cg21970438	TTLL2;TTLL2;TTLL2;TTLL2	blue	0.47569542	0.76924995	-0.008607	0.11299677	-0.4651771	-0.0352968
cg01674623	NAMPT	blue	0.25544519	0.82192007	0.00513758	-0.1665526	-0.8467938	-0.0739668
cg15241779	KLRD1;KLRD1;KLRD1;KL	blue	0.5223738	0.809881	0.03780795	0.0499929	-0.5860622	0.09113745
cg02101571	DEFB134;DEFB134	blue	0.49026615	0.86359518	-0.1089525	0.03571615	-0.5553332	-0.0398174
cg15897579	SERPINA10;SERPINA10;SE	blue	0.44836747	0.73768703	-0.050274	0.03391254	-0.4889362	0.00508538
cg04263387	RP11-205K6.2	blue	0.61744201	0.77575572	-0.0460557	0.22375865	-0.3331624	0.19877271
cg04966997	SHOX2;SHOX2;SHOX2;SH	blue	-0.5173094	-0.7511782	0.14688366	0.02783236	0.6095267	-0.1185102
cg20461533	RIMS1;RIMS1;RIMS1;RIM	blue	0.45713363	0.85259512	-0.2638312	0.05225219	-0.6041514	-0.0239836
cg02629657	MACROD2-	blue	0.57852079	0.81942679	-0.1625537	0.1594737	-0.4280312	0.04530961
cg07364180	CHRM3-	blue	0.41755014	0.89684588	-0.1248566	0.0165522	-0.6902906	0.01915636
cg03215160	OR2H1;OR2H1;OR2H1;OR1	blue	0.52794804	0.6768117	0.3070955	0.18772571	-0.3561146	0.2458953
cg04663564	AKR1B1;AKR1B1;AKR1B1	blue	0.57387524	0.79112533	0.11093693	0.08917632	-0.5729001	0.21105582
cg05040725	CLDN22	blue	0.43162412	0.80900147	0.19578974	0.07079201	-0.6983302	0.09353544
cg18316672	GPR98;GPR98	blue	0.55752409	0.85141954	0.21441432	0.15991787	-0.5788028	0.1473881
cg22028200	AC093865.1;IKZF2;IKZF2;I	blue	0.28767835	0.80084301	-0.1267631	-0.1885324	-0.8770478	-0.0432448
cg00812578	C6orf47-AS1;APOM	blue	0.46147326	0.8022554	-0.0683899	0.07351078	-0.5348274	0.16936642
cg03719988	RP11-263K19.4;RP11-	blue	0.72074041	0.71374171	-0.0508885	0.1702562	-0.3131624	0.27718147
cg15703422	LRCH4;LRCH4;LRCH4;LRC	blue	0.7023549	0.70982905	0.0720152	0.13767958	-0.3383233	0.31902284
cg16117113	RP11-410C4.4	blue	0.43333717	0.77542943	-0.116987	0.06488781	-0.4926593	0.01767759
cg05133329	SLIT1;ARHGAP19	blue	0.44326511	0.58071233	0.1567984	0.04712194	-0.3653424	0.31490161
cg26640467	GRM8;GRM8;GRM8;GRM8	blue	-0.5051824	-0.72404	0.15213659	-0.0824226	0.4110514	0.0404494
cg11694405	FAM217B;PPP1R3D;FAM2	blue	-0.3493595	-0.6557938	0.27107759	0.17323528	0.59676009	0.10011391
cg21177673	DAB1;DAB1;DAB1	blue	-0.5477562	-0.6677337	0.08262589	-0.1263682	0.43178953	-0.1272508
cg14247287	NEURL3;NEURL3	blue	0.40190069	0.79661013	0.02228227	0.00402332	-0.5723054	0.05204748
cg04372796	RP11-1007G5.2	blue	0.36083234	0.792179	0.09958737	-0.0472005	-0.801083	0.03045615
cg19927457	FSHR;FSHR;FSHR;FSHR	blue	0.53380065	0.57894013	-0.0402684	0.14610534	-0.1978218	0.12579275

cg13030331	ANKMY1;ANKMY1;ANK	blue	-0.2446712	-0.5575468	0.37501435	0.08845371	0.51889076	0.10332826
cg13122532	MIR520C	blue	0.54491386	0.62800992	0.10100341	0.11706603	-0.3347381	0.37583564
cg00564305	GTDC1;GTDC1;GTDC1;GT	blue	0.45041371	0.87056519	-0.0740221	-0.0007069	-0.7016492	0.07102341
cg09372525	CYB561D1;CYB561D1;CY	blue	0.45152031	0.62865039	-0.2085764	-0.0189785	-0.2587161	0.06877435
cg21242613	FHAD1;RP3-	blue	-0.3057807	-0.3550487	0.25785894	0.06802561	0.3053804	-0.0100067
cg03069731	ST7;ST7;ST7;ST7;ST7;ST7;S	blue	0.48852617	0.88103631	0.0422468	0.10073495	-0.626358	0.09635034
cg08099797	LIPH;LIPH;LIPH;LIPH;LIP	blue	0.3147866	0.69669165	-0.2645591	-0.0710049	-0.6057903	-0.0131463
cg10859605	RBPMS;RBPMS;RBPMS;R	blue	0.31619198	0.85040823	0.00477499	-0.0098459	-0.7495095	0.00478207
cg03278431	RP11-540E16.2	blue	0.37282523	0.83153963	-0.0784776	0.05890947	-0.5702558	-0.0766651
cg20548148	PGF;PGF;PGF;PGF;PGF	blue	0.71571526	0.70538117	0.18357477	0.25107803	-0.2469635	0.25919144
cg07174608	MED21;MED21	blue	0.7187814	0.80177841	0.07260352	0.19547806	-0.4011776	0.34590647
cg07550417	AC108865.1	blue	0.46577639	0.6940779	-0.2690206	0.12724714	-0.2105041	-0.0283493
cg20578731	OSBPL6;OSBPL6	blue	0.33521568	0.85782803	-0.1307791	-0.0520229	-0.6865823	-0.0822813
cg19310430	C11orf45;C11orf45;C11orf45	blue	-0.4464271	-0.7477865	-0.0865686	-0.0281903	0.64838168	-0.0356674
cg22950487	RP11-572M11.4;RP11-	blue	0.71305672	0.82989961	0.03160839	0.17370453	-0.4306372	0.27212989
cg21080533	PGK2	blue	0.28058219	0.84142739	-0.0812881	-0.0751453	-0.8129644	-0.0427533
cg26349048	OR5W2	blue	0.5053033	0.67786523	-0.0436862	0.16409015	-0.2158088	0.07888993
cg05473131	RP5-1119O21.2	blue	0.68118834	0.74278679	-0.0201256	0.24450566	-0.2719541	0.23881184
cg22097777	NCKAP5-IT1	blue	0.41173636	0.78851989	-0.267339	0.08114116	-0.4431567	-0.0770827
cg12050518	STK33;STK33;STK33;STK3	blue	0.52489852	0.67839937	-0.1477576	0.2062066	-0.2547765	0.0100615
cg04413674	STRA6;STRA6;STRA6;STR	blue	0.6546691	0.67869386	0.36764007	0.21424042	-0.2954775	0.20329026
cg16098952	HMGCL;HMGCL;HMGCL;	blue	0.67870596	0.77337087	-0.1657008	0.14926551	-0.3721193	0.18252143
cg20133313	CYB5R1;CYB5R1;CYB5R1;	blue	-0.4891738	-0.6826063	0.11357294	0.00793612	0.53737372	-0.1137139
cg02509576	AC010987.6	blue	0.44430945	0.62074378	-0.0348224	0.17127802	-0.269628	0.04394626
cg25380707	RP11-553E24.2;RP11-	blue	0.36067913	0.73977103	0.19191076	0.07778812	-0.5320824	0.01849787
cg08018572	LMF1;LMF1;LMF1;LMF1;L	blue	0.35955573	0.76046131	0.19208944	-0.0775803	-0.7635148	0.09817886
cg20665689	HMGB1P4	blue	0.68645144	0.80462202	0.17107142	0.1564622	-0.4226221	0.2395088
cg13589942	7SK	blue	0.56414814	0.84374769	-0.0065185	0.16694695	-0.4923994	0.12528628
cg23694268	GALNTL4;GALNTL4	blue	-0.4862995	-0.5356327	0.13301507	0.03501948	0.40404412	-0.1226032
cg05094706	ZNF648	blue	0.73977834	0.79329659	-0.015862	0.19842239	-0.3121097	0.28965914
cg27318216	SNORD95	blue	0.59795299	0.82105743	0.09034425	0.20213982	-0.3762749	0.16623286
cg15281042	RP11-142G1.2;RP11-	blue	0.37554852	0.80646283	-0.2803236	-0.0318654	-0.593511	-0.0625311
cg04380409	RDH12;RDH12	blue	0.63393398	0.86130933	0.04095984	0.08123846	-0.5256613	0.21072174
cg21570702	snoU13;LAMTOR1;LAMTO	blue	-0.4116621	-0.5238771	0.18263178	0.0638231	0.45166401	-0.0068864
cg01357846	REG3G;REG3G;REG3G;RE	blue	0.53440169	0.85735814	-0.0243404	0.08357108	-0.5473152	0.161621

cg06218726	MIR203	blue	-0.4665723	-0.8414802	0.15485843	-0.052655	0.58836867	0.01777327
cg22448084	RP11-77H9.8;USP7	blue	0.53519452	0.84855697	0.19077898	0.07464268	-0.6141894	0.12212165
cg09751042	RP11-148M9.1	blue	0.2581354	0.7904669	-0.2366857	-0.0760425	-0.6198663	-0.0500534
cg23109507	AP000704.5;HLCS;HLCS;H	blue	0.26879529	0.43440517	-0.1578968	0.03608914	-0.1559158	0.05862831
cg20441539	CST9L	blue	0.57502461	0.66673126	-0.0728918	0.18522617	-0.2547422	0.1229361
cg21385792	RP11-63N8.3	blue	0.74069451	0.76501315	0.25818457	0.19501211	-0.383364	0.30477484
cg06713935	BMPER	blue	0.51576007	0.75410638	-0.1691078	0.08282591	-0.4557165	0.0935429
cg04149804	SLC36A2;SLC36A2;SLC36	blue	0.66810618	0.82413592	0.08380787	0.10629949	-0.4971573	0.21586041
cg12034494	BLNK;BLNK;BLNK;BLNK;	blue	0.51632995	0.77255885	-0.0250258	0.22492228	-0.3479566	-0.0001232
cg03278467	IL23R;IL23R	blue	0.39255041	0.85994388	-0.0344043	-0.0698166	-0.7470745	-0.0393173
cg05592633	CPS1	blue	0.45891779	0.69310446	0.43368364	0.09650117	-0.4683095	0.19272189
cg16755041	RP11-474J1.1	blue	0.44179017	0.6157276	0.34193068	0.12577268	-0.3682877	0.0883584
cg08114979	LDHB;LDHB;RP11-59N23.3	blue	0.48274897	0.7612451	-0.1126076	-0.0159233	-0.538823	0.00913445
cg09274046	SMOX;SMOX	blue	0.36328499	0.59040346	-0.310877	0.08683413	-0.3414497	-0.0839528
cg01353586	HS3ST4	blue	-0.4099531	-0.4457962	0.23541645	0.02262975	0.22041002	-0.0718679
cg20138077	RP11-214L13.1	blue	0.61562287	0.68258948	-0.2318041	0.23586641	-0.169015	0.13347303
cg06885616	C12orf42;C12orf42;C12orf42	blue	0.37944208	0.58806858	-0.194338	0.15577599	-0.177753	-0.185675
cg07412570	CHD5;CHD5;CHD5	blue	0.41240528	0.85052694	0.00688744	0.02429695	-0.6508399	-0.0104532
cg05178734	FAM81A;FAM81A;FAM81	blue	-0.5704287	-0.7160753	0.03261092	-0.0685621	0.44544687	-0.1634183
cg24661104	RAPGEF2;RAPGEF2;RAPG	blue	0.64386025	0.859393	0.01138568	0.17110687	-0.4432042	0.11378136
cg15993118	RP11-297L17.2;RP11-	blue	0.38355137	0.84385845	0.0127074	0.03749209	-0.6371095	-0.0228108
cg07181450	RP11-226P1.2;RP11-	blue	0.74695061	0.74420381	0.1573501	0.24341688	-0.3122112	0.32171676
cg15547513	RNF150;RNF150	blue	0.30204464	0.84184799	-0.2437045	-0.0832222	-0.6764142	-0.1788321
cg15738696	OTOF;OTOF	blue	0.52101297	0.88914393	-0.0220813	0.13685229	-0.5491364	0.08277226
cg27158952	RP11-328K2.1;TOX	blue	0.4882333	0.61727706	0.38492296	0.10901631	-0.501244	0.20496856
cg16636878	NAALADL2;NAALADL2;N	blue	0.63935319	0.73948274	0.28933838	0.14722226	-0.384902	0.3142916
cg25660297	C15orf32;RP11-	blue	0.25095753	0.73049852	-0.0249073	-0.1101508	-0.6864711	0.02896682
cg06433970	7SK	blue	0.71367302	0.71065599	0.04519275	0.20839059	-0.2518497	0.21273151
cg06568890	U6atac;RP11-766N7.3;RP11-	blue	0.66264158	0.80534109	0.12958158	0.18816574	-0.3778468	0.21598451
cg19727360	EYA2;EYA2	blue	0.66266405	0.84379096	-0.0681429	0.10934645	-0.4301255	0.18060829
cg02282350	CCDC63;CCDC63;CCDC63	blue	-0.4998164	-0.5391369	0.16670158	0.10539935	0.41008386	-0.0484947
cg16313158	RP11-543D5.3;RP11-	blue	0.4344262	0.71934052	-0.1158072	0.12467695	-0.4681965	0.02794305
cg15285815	ISPD	blue	0.44626992	0.61668589	0.2284799	0.09466072	-0.4005942	0.00313293
cg15026112	AC007126.1;RP11-341G5.1	blue	0.25067011	0.81459212	-0.168717	-0.1239894	-0.7210305	-0.0488822
cg05604079	QRFPR;QRFPR;QRFPR	blue	-0.4447056	-0.5020804	0.19088718	0.03299038	0.31641299	-0.0258422

cg01223673	INSC	blue	0.34309504	0.80870609	0.0681569	-0.0305191	-0.7199302	0.06647519
cg02952984	LIPC;LIPC;LIPC;ALDH1A2;	blue	0.5953743	0.67500586	0.46135395	0.17713853	-0.37139	0.19333437
cg24082174	EIF4A2;EIF4A2;EIF4A2;EIF	blue	0.36761578	0.86265222	-0.0095655	-0.0511538	-0.7840769	0.02421925
cg11878254	C1QL4	blue	-0.4785199	-0.6040262	0.22354165	0.00101405	0.3815695	-0.0682969
cg16906497	ARHGAP28;ARHGAP28;A	blue	0.19894942	0.74557378	-0.2802851	-0.1480711	-0.6796769	-0.1996941
cg01374281	RP11-415A20.1	blue	0.44502013	0.86435116	-0.187614	0.05727001	-0.5754001	-0.0238114
cg13590413	ATAT1;ATAT1;ATAT1;AT	blue	-0.3422941	-0.6277253	0.14518401	0.00687952	0.43933117	-0.0009192
cg01805732	PXDN;PXDN;PXDN;PXDN	blue	-0.3776419	-0.3985289	0.17709795	-0.0084476	0.24052113	-0.0450582
cg07426979	AP006748.1;TMPRSS3	blue	0.42007158	0.67181116	-0.0701346	0.05811787	-0.4301103	-0.0671674
cg04469167	MOB3B	blue	-0.5088446	-0.6249367	0.17795138	-0.0152937	0.50817565	-0.0941092
cg23330281	NAV2	blue	0.41231328	0.6705953	0.36809857	0.00692678	-0.5371928	0.12704884
cg24718827	ANKRD33B;ANKRD33B	blue	-0.430105	-0.451126	0.17599508	-0.0084039	0.30557417	-0.0865447
cg12871937	RP11-536I6.1	blue	0.62089385	0.84213163	0.01856697	0.11390705	-0.4862772	0.25697163
cg04578029	CCDC36;CCDC36;RP11-	blue	0.44078731	0.59669471	-0.2564866	0.08512981	-0.2149433	0.01886319
cg01055208	U4	blue	0.69394689	0.64630053	-0.0206018	0.27185874	-0.1758377	0.2313966
cg23776302	GOLGA4	blue	0.45740105	0.87146255	-0.1075221	-0.0407616	-0.671523	0.05216031
cg19596509	EIF2B5-AS1	blue	0.69431363	0.83379699	0.01474686	0.19378518	-0.3592631	0.17716177
cg26614943	SGK1;SGK1;SGK1;SGK1;S	blue	-0.6086314	-0.6831208	0.11992322	-0.0975612	0.311583	-0.1916374
cg13329685	KCND3;KCND3	blue	0.66048809	0.70207465	0.04401851	0.13441854	-0.2536784	0.2597755
cg07784217	RP11-955H22.1	blue	0.31068009	0.69938671	-0.163197	-0.0238566	-0.5735757	-0.1169139
cg04119593	C21orf88;C21orf88;B3GALT	blue	-0.4142831	-0.5928734	0.12007066	-0.0751104	0.37579516	-0.0306313
cg00887049	ZNF546	blue	0.69995191	0.68302875	0.17292711	0.20643516	-0.4101242	0.26130707
cg22538063	AC091096.1;RP11-587H10.2	blue	0.47067655	0.78550828	-0.2025848	0.0592248	-0.4608559	-0.0123872
cg04476400	RP11-259F4.1	blue	0.22331705	0.7357199	-0.1426063	-0.1263926	-0.6225021	-0.1205017
cg10262037	ESRRG;ESRRG;ESRRG;ES	blue	-0.4130984	-0.724315	0.32493735	-0.1049061	0.43012933	0.09000934
cg17707805	AC078842.3	blue	0.58769058	0.69364229	-0.2557751	0.09764036	-0.2346139	0.07720038
cg03461885	AC025165.8;AC025165.8;A	blue	-0.4029959	-0.672762	0.04911286	0.05065563	0.60807684	-0.1222794
cg25615878	AC091633.3;AC091633.3;A	blue	0.33105126	0.77855355	-0.1152616	-0.136075	-0.6831498	0.01723765
cg04198778	GABRP;GABRP;GABRP;G	blue	0.42319515	0.81909627	-0.0475656	0.10103891	-0.597217	0.07063787
cg11973286	ANKS1B;ANKS1B;ANKS1	blue	0.53896793	0.76434553	-0.0617075	0.14610481	-0.3770872	0.07721794
cg09057327	AL132709.8;SNORD114-	blue	0.23092111	0.38535739	-0.4498482	-0.0341337	-0.1544334	-0.1959864
cg08205065	RP11-168L7.1	blue	0.42754187	0.78529814	-0.1318232	0.01340021	-0.4991988	-0.0103789
cg00772587	AC016738.3;AC016738.3;NP	blue	0.59948712	0.64696615	-0.0356554	0.05047295	-0.3797136	0.23809696
cg16696296	IPCEF1;OPRM1;IPCEF1	blue	0.5651995	0.6856807	-0.0596054	0.15387756	-0.3412021	0.21002878
cg13890019	ZNF32-AS3	blue	0.56045144	0.68779734	-0.3699884	0.15623891	-0.3114551	0.07462507

cg02827278	ORAI1	blue	0.27068804	0.77690684	-0.0730929	-0.1001443	-0.7018717	-0.1022907
cg02757295	PPP3R2;PPP3R2	blue	0.55598349	0.86981899	-0.1639591	0.10373847	-0.4979749	0.05855793
cg22424108	SLC44A3;SLC44A3;SLC44	blue	-0.490499	-0.487674	0.16604528	-0.0188987	0.42273076	-0.1858102
cg16590189	CPLX2;CPLX2;CPLX2;CPL	blue	-0.5008594	-0.6972276	0.12222081	-0.0918051	0.45325493	-0.0942878
cg10718885	BTD;BTD;BTD;BTD;BTD;B	blue	0.47429467	0.85632235	-0.0463482	0.03357323	-0.5442364	0.07131575
cg03998348	TMCO5A;TMCO5A;TMCO	blue	0.51326691	0.83710671	-0.1063494	0.07366499	-0.4943755	-0.0001579
cg12184157	CST9L	blue	0.69193169	0.86655749	-0.0077078	0.12266396	-0.5101399	0.23922785
cg26406074	NBEA;NBEA	blue	-0.2548191	-0.657645	0.12658911	0.12909149	0.5372518	-0.0255137
cg05016953	SLC6A4;SLC6A4;SLC6A4;S	blue	-0.4590346	-0.6554249	0.10179303	-0.0092629	0.37082518	-0.0718514
cg21159015	AC115115.2	blue	0.48256738	0.60165199	-0.019434	-0.0111971	-0.4116668	0.1107796
cg06757810	CNGB3;CNGB3;RP11-	blue	0.58220471	0.67621881	0.21124328	0.16344841	-0.2979722	0.26596959
cg17319998	U6;GINS3;GINS3;GINS3;GI	blue	0.38840671	0.87098487	0.03022597	-0.0118296	-0.7117694	-0.044337
cg02246190	PCDH20;PCDH20;PCDH20	blue	-0.4811177	-0.5771371	0.15686986	0.02873265	0.3848553	-0.0352526
cg04537729	RP11-382A20.3;RP11-	blue	-0.575566	-0.7512429	-0.0083563	-0.0955294	0.54549792	-0.1769119
cg20960490	GFRAL	blue	0.64991553	0.69438853	0.13857302	0.20662999	-0.2928716	0.26876027
cg04215516	RNU1-17P	blue	0.33579046	0.45356337	-0.2134481	-0.1065395	-0.3736125	0.03207231
cg04706353	VSNL1;VSNL1	blue	-0.421684	-0.4922682	0.30139232	0.01061174	0.23345187	0.09383423
cg20089532	FRMPD2;FRMPD2;FRMPD	blue	0.45834682	0.52582036	0.1298357	0.16476817	-0.1995796	0.16191781
cg23180713	FAM178B;FAM178B;FAM1	blue	0.47872434	0.63602887	-0.1436652	0.10592647	-0.400904	0.08415235
cg03525049	GDAP1;GDAP1	blue	-0.4448088	-0.8294468	0.11419797	0.07670617	0.66096714	-0.0408766
cg17523822	RP11-395P13.4	blue	0.40943389	0.54934317	-0.129258	0.16549804	-0.2171427	-0.0473943
cg26884705	PTP4A2;PTP4A2;AL136115.	blue	0.31540128	0.82483378	-0.0053125	-0.0767714	-0.7744169	0.03030394
cg03562978	SNORD113-3;MEG8	blue	0.36745031	0.87222077	-0.115799	-0.0402847	-0.670523	-0.0550362
cg15323564	AC074389.5	blue	0.68187388	0.78197551	0.00463139	0.14962264	-0.3555674	0.22445963
cg12316290	UBL3	blue	0.62893657	0.87477031	0.00404688	-0.0011827	-0.6235862	0.25020261
cg15444562	RP11-624J12.1	blue	0.50969093	0.77954311	-0.2042372	0.12916172	-0.4147615	0.00414576
cg17668846	RP11-492L8.2	blue	0.22818873	0.68815218	0.02810579	0.07248446	-0.4848762	-0.1187065
cg25700996	RP11-753D20.1;RP11-	blue	0.35923536	0.78952423	-0.1583957	-0.0524646	-0.5493783	-0.0485601
cg18486698	ALX4	blue	-0.5244531	-0.7094422	0.154416	-0.0481018	0.52894282	-0.1476246
cg00833487	U6	blue	0.52169233	0.75680678	-0.0511771	0.05697309	-0.4788193	0.12412053
cg00763594	KAZN;KAZN	blue	0.60502641	0.62762505	0.33045255	0.20014577	-0.3476084	0.19884137
cg06825518	KCNMB2-IT1;KCNMB2-	blue	0.40172058	0.83305302	-0.2269129	-0.0257453	-0.5630186	-0.1043125
cg10014563	C14orf149;GPR135;GPR135;	blue	-0.4602483	-0.7431397	0.04405284	-0.0051707	0.64561117	-0.0878123
cg13923094	DSP;DSP;RP3-512B11.3	blue	-0.6303358	-0.6838083	0.10910539	-0.0148434	0.42182962	-0.1407871
cg00721480	RP11-417L19.2	blue	0.67999415	0.70641898	-0.0406343	0.24547247	-0.2053805	0.19784862

cg03434423	ZNF839;ZNF839;ZNF839;Z	blue	-0.5138362	-0.7116199	0.17956834	-0.0032985	0.58860919	-0.1710783
cg01354581	C15orf59;C15orf59;C15orf59	blue	-0.3812193	-0.4264736	0.13778726	-0.046447	0.29657233	-0.0859534
cg11673092	ARMC3;ARMC3;ARMC3;A	blue	-0.4665711	-0.6740046	0.08617317	-0.0172453	0.58438405	-0.0414777
cg15532149	TMEM5;TMEM5;TMEM5;T	blue	-0.3646389	-0.6082423	-0.0037347	0.09385278	0.52621785	0.00649128
cg00481216	AC068196.1;AC104820.2	blue	0.54696126	0.83266083	-0.1389678	0.10881468	-0.4767924	0.08673613
cg04501273	RNY1P2	blue	0.50392133	0.81103753	-0.0650287	0.10228561	-0.4360801	0.05097145
cg23621729	C1orf222;C1orf222	blue	-0.5095296	-0.7553616	0.00567692	-0.0644539	0.5630765	-0.135603
cg23588609	KIF26B	blue	0.57693178	0.7692996	0.17836593	0.05371422	-0.5694649	0.25986486
cg18947801	RP4-788L13.1;RP4-	blue	-0.4106493	-0.7165294	0.19170857	0.07680855	0.59377959	0.01291592
cg11500257	PNRC2;CNR2	blue	0.29703609	0.37671173	-0.3115966	0.07810642	-0.2516547	-0.0191387
cg26752321	CCNJL;CCNJL;CCNJL;CCN	blue	0.53447028	0.78276595	0.33706992	0.07774067	-0.6074811	0.23537969
cg18201198	LY86-AS1;LY86-AS1	blue	0.67190267	0.80714406	0.10757941	0.09045668	-0.4845683	0.31461232
cg01371072	PITX2	blue	-0.6124954	-0.7271507	0.06419061	-0.0265116	0.4332762	-0.2527185
cg11840152	GMEB2;GMEB2	blue	0.48657184	0.59314398	-0.1166833	0.03606206	-0.3921091	0.09536068
cg02426836	TRPM5;TRPM5;TRPM5	blue	0.68981732	0.71168395	0.02451795	0.28416011	-0.2666798	0.25099714
cg07692746	SLCO2A1	blue	0.63884251	0.8551809	-0.0338546	0.16168159	-0.444606	0.21823277
cg24539375	RP11-503C24.4	blue	0.43667301	0.76849807	-0.1062426	-0.0571299	-0.4897537	0.1480069
cg05729836	ACTL6B;ACTL6B;ACTL6B;	blue	0.34684761	0.51672919	-0.0515208	0.01352691	-0.3713473	0.08781211
cg02097396	KCNJ15;DSCR8;TMPRSS3	blue	0.4234826	0.85545625	0.01928585	-0.0585889	-0.7213047	0.0102088
cg01539573	RN5S486	blue	0.77238218	0.78607749	0.09710603	0.1919533	-0.2919822	0.29576788
cg19995038	Y_RNA	blue	0.66964932	0.8160498	0.13583198	0.20264209	-0.4605175	0.27877635
cg09954052	AC108142.1;AC108142.1;A	blue	-0.5216469	-0.72351	0.21774986	-0.0938016	0.44000677	-0.0367964
cg17063929	NOX4;NOX4;NOX4;NOX4;	blue	-0.371566	-0.5139295	0.22439265	-0.0757457	0.30194957	0.05497208
cg07109762	EPB41L3;EPB41L3;EPB41L	blue	0.61945804	0.87871006	-0.0581934	0.06440348	-0.5857128	0.23060202
cg05674944	SLC30A2;SLC30A2	blue	-0.4348662	-0.7186323	-0.0877317	-0.0556076	0.52244617	-0.0352644
cg06314761	SYNPR;SYNPR;SYNPR;SY	blue	-0.5038646	-0.7777935	0.20261432	0.01108491	0.56189611	-0.0960249
cg08713760	U6;MUC19;MUC19	blue	0.60328099	0.77618608	-0.1790252	0.15331889	-0.3063387	0.03569467
cg10849866	RP11-325N19.3;RP11-	blue	-0.591919	-0.7019749	0.00430105	0.0152617	0.40627086	-0.2000305
cg12370583	RN5S87;AC068490.2	blue	0.20301043	0.63536906	-0.0490549	-0.0438515	-0.5154957	-0.1574711
cg10679103	RN5S93	blue	0.27052399	0.82027865	-0.1507811	-0.0486866	-0.6559953	-0.0917422
cg02230171	CTD-2520I13.1	blue	0.46086487	0.64018225	-0.1970995	0.14888926	-0.1966167	0.07352748
cg01687040	DCD	blue	0.39768295	0.72826194	-0.0711445	-0.0382219	-0.5531839	-0.0412866
cg02414840	RP11-196O2.1;RP11-	blue	0.5778085	0.70206437	0.32254712	0.15406463	-0.3248522	0.11263303
cg06157602	PCDHGB6	blue	-0.3889056	-0.7002775	0.19406723	-0.0311045	0.53510565	0.09363311
cg04085032	SNORA68	blue	0.34331119	0.7332825	-0.0514163	0.03705274	-0.4827601	0.00104051

cg09498788	SNORA26	blue	0.4376001	0.82671278	-0.0737973	-0.002151	-0.6159349	0.06195642
cg23232093	TPST2	blue	0.64096868	0.77092841	-0.0841876	0.08069813	-0.4848084	0.2327683
cg23655227	SIGIRR;SIGIRR;SIGIRR;SI	blue	0.55076613	0.72205182	0.20774042	0.1748801	-0.4633501	0.23393053
cg08483340	RP1-90L14.1;RP1-	blue	0.54033294	0.76149374	-0.014594	0.216775	-0.3511362	0.09286736
cg08786644	ABCA1	blue	0.39708705	0.84430097	-0.2966201	-0.0093822	-0.5517202	-0.1023803
cg09009318	snoU13	blue	0.4736373	0.84090071	-0.0654372	-0.0522902	-0.6519955	0.08401038
cg24754066	NRSN1;NRSN1;NRSN1;NR	blue	-0.4844985	-0.7920242	0.13487665	0.03219411	0.54418489	-0.063437
cg27047075	HAND2;HAND2;HAND2;H	blue	-0.3074693	-0.754769	0.15633432	0.06861663	0.63679232	-0.059318
cg25070253	COX7A2L;AC006038.4	blue	0.57096674	0.76752114	-0.0187518	0.14011318	-0.4151975	0.08721746
cg15941845	TMEM78	blue	0.52273018	0.84568914	-0.0915892	-0.0155327	-0.6154437	0.12872245
cg06595693	DISP2	blue	-0.3320938	-0.6064239	0.02245146	0.10871094	0.48637401	0.05624701
cg12006271	RPL37;RPL37;SNORD72;RP	blue	0.58702146	0.68521813	0.34337601	0.20189723	-0.3973939	0.14238051
cg22837767	PAX1;PAX1;PAX1	blue	-0.4235954	-0.6192737	0.33336403	0.04393378	0.41408649	-0.0277839
cg07675966	U6;RALYL	blue	0.35318844	0.71689759	0.11953299	0.09717204	-0.4228503	0.01485492
cg22879111	snoU13	blue	0.71817749	0.76187932	0.05986151	0.25743816	-0.3117823	0.24013898
cg06192142	AC011998.1	blue	0.64748555	0.77792686	0.08715652	0.1899802	-0.3296366	0.2139341
cg10687991	ADAMTS18	blue	0.27090954	0.6847391	-0.2744363	0.05103955	-0.3912113	-0.1446566
cg09444186	PPM1N;PPM1N;PPM1N;PP	blue	-0.5972097	-0.6588156	0.08042163	0.09046801	0.52048838	-0.1858944
cg13794571	MYOM2;MYOM2;MYOM2	blue	0.30612545	0.61483188	-0.036708	-0.1081658	-0.5449651	-0.0324494
cg00666105	SOX2;SOX2;SOX2-	blue	-0.5615985	-0.6858371	0.13754448	0.00529878	0.46602614	-0.3087577
cg25444084	C20orf61	blue	0.40257538	0.87390714	-0.0741758	-0.0408696	-0.695156	-0.0159624
cg19809645	CAPN13;CAPN13;CAPN13;	blue	0.41758776	0.8993984	-0.1192036	-0.0188357	-0.7255894	0.02886741
cg27618318	TPM1;TPM1;TPM1;TPM1;T	blue	0.51813012	0.72318203	-0.116724	-0.056708	-0.6216601	0.13518219
cg09685763	C20orf160;C20orf160	blue	0.4602679	0.83335245	0.08087305	-0.0203567	-0.6734555	0.10732681
cg22176779	RP5-1097P24.1	blue	0.45434862	0.82352043	-0.1709087	0.04760328	-0.5602922	0.01043387
cg14941029	PLCZ1;PLCZ1;PLCZ1;PLCZ	blue	0.38352431	0.77555969	-0.2948546	0.03406405	-0.5045957	-0.149415
cg04299266	IL33;IL33	blue	0.61986222	0.75942247	0.1277449	0.21294118	-0.2957995	0.08155329
cg14314156	PTPN5;PTPN5	blue	0.56111764	0.69053283	-0.0318857	0.20100446	-0.2905867	0.19757103
cg19979827	DNHD1;DNHD1	blue	0.41257207	0.44972869	-0.1211441	0.15230093	-0.1330389	-0.0769592
cg05982423	CTNND2;CTNND2;CTNND	blue	0.46402188	0.90545679	-0.0423669	0.08857517	-0.605569	0.04263999
cg01916907	IRGC	blue	0.58832129	0.80656565	-0.0421906	0.06152584	-0.4608709	0.14485128
cg16362222	AC019055.1	blue	0.41619851	0.72228866	-0.0419325	0.05692044	-0.4165141	-0.055211
cg00743664	DENND2A;DENND2A;DE	blue	0.57135493	0.8033532	-0.0326717	0.04392096	-0.4771757	0.11249114
cg20750943	APLP1	blue	-0.6123877	-0.6749328	0.12244218	-0.0287072	0.44965516	-0.2474015
cg07688490	TMEM45B;TMEM45B;TME	blue	-0.3786054	-0.6158533	-0.0083463	0.04002753	0.45005134	-0.1620731

cg25378279	AL133232.1	blue	0.38568775	0.83245295	-0.0046471	0.0527907	-0.614434	0.01268969
cg11531198	ALCAM;ALCAM	blue	-0.3753754	-0.6996595	0.1837378	0.04184238	0.58042841	0.02161978
cg18489923	LRRC8C;LRRC8C;LRRC8C;	blue	-0.4287921	-0.4883854	0.20324144	0.03319249	0.38055352	0.0483408
cg09605636	AC017096.1;AC017096.1;A	blue	-0.49559	-0.6617061	0.20349194	0.06904535	0.49423203	-0.048941
cg15490107	AC013722.1;TBC1D8	blue	0.46725477	0.84037777	-0.0648751	-0.047947	-0.6135531	0.10371471
cg21990556	OSBPL5;OSBPL5;OSBPL5;	blue	-0.5215732	-0.75736	-0.0075094	-0.0337362	0.64538953	-0.2451371
cg21847062	Y_RNA;AC019221.4	blue	0.46872929	0.85136781	-0.0826318	0.00378351	-0.6819692	0.09065625
cg09987466	AC132807.1;TBC1D5	blue	0.54662361	0.87411931	0.09582492	0.06784327	-0.5931353	0.10253512
cg00656150	RP11-20L19.1	blue	0.26542042	0.66582029	-0.2846808	-0.005731	-0.4304035	-0.1678685
cg06073417	PLA1A;PLA1A;PLA1A;PLA	blue	0.48850507	0.84711954	-0.1285146	0.06670529	-0.5373752	0.08949707
cg06836772	PRKAA2;PPAP2B;PPAP2B	blue	-0.4201601	-0.5652531	0.17089659	0.08812528	0.45983044	0.03172737
cg22119768	ASS1;ASS1	blue	0.37628028	0.83021931	-0.0698365	0.03036421	-0.6333067	0.00751963
cg17692403	R3HDML	blue	-0.4304345	-0.5514578	0.14179335	0.01538916	0.35809343	-0.1503484
cg26636379	TP53AIP1;TP53AIP1;TP53A	blue	0.57997072	0.78500061	0.11075396	0.13234394	-0.4694582	0.20261754
cg03475172	USP6NL;USP6NL	blue	-0.4607795	-0.6844929	0.1380604	0.03969539	0.53211685	-0.0437093
cg19246197	USP2;USP2;USP2	blue	0.52992227	0.58511118	0.45152782	0.1151584	-0.3409602	0.20355717
cg21059475	EML1;EML1	blue	-0.5418797	-0.7497244	-0.0307356	-0.0982411	0.51373111	-0.1716867
cg20720876	ERG;ERG;TMPRSS3	blue	0.47847204	0.71983007	-0.306371	0.08084381	-0.3672326	0.05930049
cg21578542	NOVA2	blue	-0.3996085	-0.5022866	0.28004834	0.02718326	0.38082282	0.02321744
cg03450491	PAK1;PAK1;PAK1;PAK1;P	blue	-0.2893004	-0.4774878	0.3501404	0.04486479	0.33522086	0.07789028
cg20691539	RP11-883G14.2	blue	0.33894283	0.797715	0.0743302	-0.0588524	-0.6995952	-0.0381761
cg15618473	INPPL1;INPPL1;INPPL1;IN	blue	0.46447322	0.88070865	-0.0626041	0.00084206	-0.6618896	0.11255073
cg27512716	IRGC	blue	0.49641504	0.86680933	0.09433257	0.03733424	-0.6813192	0.14318765
cg17279394	RP11-54A22.2	blue	0.6740333	0.62738737	0.05942915	0.15012268	-0.2076442	0.31449377
cg27384253	DAQB-12N14.5;DAQB-	blue	0.65526509	0.69589721	-0.0126189	0.23644422	-0.284806	0.25224681
cg16764236	CCL11	blue	0.68799026	0.80734017	0.13591021	0.26957513	-0.3338956	0.22424911
cg26558036	SLC22A7;SLC22A7	blue	0.31999408	0.80654622	-0.0402199	0.03966808	-0.631696	-0.0306164
cg20372535	Y_RNA	blue	0.48633126	0.9065008	0.02773852	0.0472662	-0.6115148	0.08099215
cg02558539	RHOH;RHOH;RHOH;RHO	blue	-0.1899152	-0.5685788	0.09705264	0.06667172	0.50161294	0.16619517
cg09210931	FCHSD1;FCHSD1;FCHSD1	blue	0.48936951	0.76152357	-0.0129722	0.11899378	-0.4430114	0.07918617
cg19680048	AC010729.3	blue	0.67304405	0.72324126	0.29439155	0.20061274	-0.3006614	0.34292229
cg26575637	LMO3;LMO3;LMO3;LMO3;	blue	-0.3059102	-0.425063	0.28528932	0.03417371	0.33155685	0.08513984
cg07800384	FAM150B;FAM150B;FAM1	blue	-0.5313863	-0.6084154	0.18798207	-0.0598011	0.30813412	-0.0200735
cg02249292	MKX;MKX;MKX;RP11-	blue	-0.4466195	-0.6597865	0.23774729	0.08094849	0.54255205	0.03153889
cg09031882	RP4-601P9.1;RP4-601P9.2	blue	0.67829896	0.76382219	0.04344874	0.12937193	-0.3102433	0.17673813

cg20256134	RP1-90L6.3;RP1-90L6.3	blue	-0.5088663	-0.7378655	-0.0503207	0.05568643	0.58544536	-0.153819
cg11594811	CADM1;CADM1;CADM1;C	blue	0.33053146	0.82933674	-0.0870834	-0.0238252	-0.6382762	-0.0263387
cg01368041	AC008158.1	blue	0.45179765	0.73975535	-0.0912237	0.08787744	-0.4168424	0.03610391
cg02807558	AC009227.2	blue	0.39566749	0.77696353	-0.2713269	0.03369448	-0.510107	-0.0880217
cg15304800	NLRP11;NLRP4	blue	0.3825003	0.7582791	-0.1743021	0.03072521	-0.4509067	-0.0713341
cg09807565	NMU;NMU;NMU;NMU;N	blue	-0.5112411	-0.6767851	0.18401486	-0.0313781	0.39973908	0.10543398
cg16084872	TMPRSS2;TMPRSS2;TMPR	blue	-0.5420391	-0.727944	0.16288462	0.00611482	0.50574032	-0.0093603
cg23863066	RP11-84E24.3;RP11-	blue	0.68269431	0.71657165	0.2512122	0.22313089	-0.3253027	0.21931303
cg14491761	GLP2R;GLP2R;RP11-	blue	0.33335929	0.73838456	-0.0302503	-0.0583879	-0.6169066	0.02869778
cg20708842	FBN2;FBN2;FBN2;SLC27A	blue	-0.44189	-0.5904514	0.31888969	0.04507247	0.39274394	0.07157073
cg08153098	CLRN1;CLRN1;CLRN1;CLR	blue	0.42981633	0.90738238	-0.0093806	-0.02604	-0.7343383	0.09565883
cg11305401	RNU4-7P	blue	0.69133796	0.80886972	0.1149593	0.22079599	-0.3668818	0.13944473
cg23499947	AC003664.1	blue	0.4364865	0.73565297	-0.2107144	0.07948328	-0.3884729	-0.1060556
cg09855693	RP11-397H6.1;RP11-	blue	0.44448793	0.78753329	0.17227501	0.00202273	-0.6028248	0.11190762
cg11932225	AFAP1	blue	0.38412495	0.53430311	-0.1232886	0.05431995	-0.2630404	0.06197011
cg26298612	RIMS1;RIMS1	blue	0.42309163	0.83614579	-0.2540801	-0.0481505	-0.5548299	-0.0811601
cg01141506	KIAA1467;KIAA1467	blue	0.64083807	0.76628509	-0.1898247	0.17129623	-0.3739543	0.17356843
cg12454073	U6;RP11-631F7.1	blue	0.49060969	0.74346293	0.29708827	0.09591404	-0.5368555	0.14383164
cg24089171	FGF14-IT1;FGF14-	blue	0.32803986	0.83005577	-0.2637771	-0.0164998	-0.5977977	-0.1684667
cg06179465	U7	blue	0.41755623	0.85773397	-0.1130928	0.00863808	-0.6429035	0.02903307
cg03892256	RP11-1C1.7	blue	0.42929034	0.76250329	-0.1220198	0.02624628	-0.5367253	0.04462988
cg16781855	RP11-699L21.1;RP11-	blue	0.63044918	0.83701199	0.07818017	0.19782738	-0.4237341	0.18326734
cg15208851	TNKS1BP1;TNKS1BP1;TN	blue	0.55527362	0.62298577	0.18183034	0.16055185	-0.2747011	0.24510735
cg05026785	KCNIP3;KCNIP3;KCNIP3;	blue	0.46376596	0.54318229	-0.1336021	0.14674728	-0.1759714	0.1146665
cg05524039	CLCF1;AP003419.11;CLCF1	blue	-0.3752663	-0.720397	0.15135795	0.06341149	0.62130169	0.10851721
cg09807275	MMP2	blue	0.57893789	0.89772171	0.00898978	0.10240489	-0.6052413	0.1533169
cg07102632	NAT2;NAT2	blue	0.46645865	0.85598709	-0.0687322	0.12160393	-0.5165783	0.039279
cg00422312	SLCO2A1	blue	0.37180866	0.7277519	0.08779102	0.03841795	-0.4411567	0.12526859
cg26896093	AC004485.3	blue	0.31611574	0.49273102	-0.1319664	-0.0083813	-0.2677109	-0.1479803
cg07283423	KRTAP19-8	blue	0.60364026	0.78785331	-0.0850089	0.13469171	-0.3507438	0.1305042
cg12264949	PITX3;RP11-242B12.5	blue	-0.5008537	-0.8171798	0.15986502	0.06950469	0.64042553	-0.0265691
cg03939903	ZNF385B;ZNF385B;ZNF385	blue	0.36582909	0.82894206	-0.0162052	-0.0263045	-0.6051047	-0.0192365
cg05273887	U6;KREMEN1	blue	0.74025183	0.72347047	0.02328131	0.24043686	-0.3553499	0.33048978
cg03742705	ATAD3B;ATAD3B	blue	-0.4039514	-0.5372848	0.13873288	0.01820245	0.37630485	-0.0061195
cg01032200	RUSC1-AS1;RUSC1-	blue	-0.2586327	-0.5328986	0.15810793	0.04119343	0.43449808	-0.0512439

cg05335315	JAG2;RP11-44N21.4	blue	-0.5276701	-0.4939036	0.18757555	0.0266891	0.35373209	-0.0897439
cg14888636	MAN1C1	blue	-0.2426424	-0.6230414	0.17739893	0.09528203	0.57851788	0.18230761
cg03946012	GINS3;GINS3;GINS3;PRSS	blue	0.61216523	0.80894143	-0.1325164	0.10325102	-0.4077773	0.10859177
cg04597711	U6;NR5A2	blue	0.40860621	0.87464541	-0.1975053	-0.0016131	-0.6395962	-0.088427
cg05919685	APCS	blue	0.27973138	0.85410327	-0.0751947	-0.1133202	-0.7927337	-0.0856779
cg18102767	AC021066.1;AC021066.1;K	blue	0.42776097	0.75097003	-0.06684	0.04125468	-0.4774589	-0.1280098
cg19741727	RP11-6B4.1	blue	0.55368413	0.71041865	-0.0218598	0.14409738	-0.3657702	0.07474398
cg13271155	ERBB3;ERBB3;ERBB3;ERB	blue	0.51677157	0.74676377	-0.2332085	0.12882643	-0.363915	0.02057555
cg15882537	KIF1A;KIF1A;KIF1A;KIF1	blue	0.4451269	0.72414162	0.00663394	0.00298848	-0.4133873	0.04309447
cg12995528	U3;WWC2	blue	0.47656225	0.87206352	-0.0441238	-0.040798	-0.7223721	0.11597981
cg04874751	WNK2;WNK2;WNK2;WNK	blue	0.65597827	0.6957105	0.13665356	0.23714819	-0.2621072	0.27579551
cg14399569	C9orf11;C9orf11;C9orf11	blue	0.70434867	0.7974447	-0.1221612	0.17088504	-0.3442531	0.20008984
cg26452654	SEC24C;SEC24C;SEC24C;S	blue	-0.4329898	-0.6097329	0.1320935	0.05133074	0.48102886	-0.0544266
cg01110347	ST7;ST7;ST7;ST7	blue	0.61634961	0.79213977	0.13472127	0.2904629	-0.3514824	0.18481239
cg03910962	ANGEL2	blue	0.40627972	0.82038466	0.1610509	-0.0220826	-0.7582217	0.12116464
cg02396520	AC010983.1	blue	0.37141106	0.78183964	-0.1996864	0.03347266	-0.4815823	-0.0740462
cg20576597	TSPEAR;TSPEAR	blue	-0.5844278	-0.63397	0.03194968	0.05158888	0.39943758	-0.210019
cg13857519	TPH1;TPH1;TPH1;TPH1	blue	0.48793722	0.84774816	-0.1935067	0.02388456	-0.5194402	0.00922489
cg02179652	BSX	blue	-0.3748516	-0.6231675	0.17747847	0.08253691	0.4487113	-0.1120878
cg23564879	NAV2-AS1	blue	0.69731674	0.76168692	-0.1104276	0.20321285	-0.3486484	0.18771253
cg23172400	TP53INP1;TP53INP1;RP11-	blue	-0.1850202	-0.4999269	0.22410273	-0.0061911	0.39273265	0.16361559
cg22043745	GATA4;GATA4;GATA4;G	blue	-0.4893412	-0.5354331	0.23788926	-0.0896444	0.23668356	-0.0354473
cg10778441	TBX20;TBX20;TBX20	blue	-0.5563167	-0.6999949	0.05486557	-0.02899	0.35239579	-0.2106651
cg19358493	EMX2;EMX2;EMX2OS;EM	blue	-0.36048	-0.7766064	0.24566167	-0.012593	0.58674467	0.07456281
cg13673239	SDK1;SDK1	blue	0.68860478	0.7689568	0.03239976	0.19515043	-0.3669757	0.33449091
cg12177643	MAP7;MAP7	blue	0.60507622	0.79137369	-0.1912821	0.02501653	-0.4658638	0.15185437
cg18200549	Y_RNA	blue	0.55869425	0.71550047	-0.2901125	0.06406238	-0.3740567	-0.0220478
cg08774410	RP11-10N16.3;RP11-	blue	-0.2535628	-0.5221311	0.1986071	0.01699947	0.45813956	0.15185502
cg21010701	DTHD1	blue	0.37515954	0.80328952	-0.1078208	-0.0645764	-0.7561517	0.03537991
cg11923198	RP11-34F13.3;RP11-34F13.3	blue	-0.4059217	-0.7941898	0.25989929	-0.0198088	0.64067011	0.03756296
cg04100201	WT1-AS;WT1-AS;WT1-	blue	-0.5152314	-0.5860024	0.15309447	0.04461621	0.30813353	-0.0929967
cg25421053	RP1-105O18.1	blue	0.40643651	0.80410789	-0.1408762	-0.0343282	-0.5483038	-0.0516264
cg13575601	ARHGAP25;ARHGAP25;A	blue	-0.3557849	-0.4343188	0.10048695	-0.0888328	0.30086678	-0.0637715
cg20670882	RP11-552E20.1	blue	0.60848004	0.82274996	0.04406307	0.14899209	-0.4199639	0.11068411
cg14067066	Y_RNA;HLA-F-AS1;HLA-F-	blue	-0.3409347	-0.3726541	0.02124901	0.160794	0.3386676	-0.1103764

cg04135977	RP11-79N23.1	blue	0.40633505	0.56121113	-0.1952344	0.01036876	-0.3498365	0.10934161
cg14644855	RP11-770E5.1	blue	0.71122361	0.75404467	0.16925084	0.22298118	-0.2629672	0.28124581
cg12612406	TRIM40;TRIM40;TRIM40;T	blue	0.65353562	0.83322612	0.024035	0.16699655	-0.4999363	0.2485074
cg15339720	SDC1;SDC1;SDC1;SDC1	blue	-0.521399	-0.6959339	0.09538542	-0.0325731	0.43951362	-0.0517627
cg27176536	RAB26;RAB26;RAB26;RAB	blue	-0.6363505	-0.6841905	-0.0373342	-0.060758	0.46342068	-0.358257
cg15852130	ANKRD62	blue	0.35050772	0.86970871	-0.1274443	-0.1268551	-0.8025308	-0.041792
cg13353375	CAPS2;CAPS2;CAPS2;CAP	blue	0.46243571	0.50277143	0.46076751	0.15066607	-0.1985984	0.26475772
cg24738424	RP3-495K2.3	blue	0.4043854	0.82226977	-0.0521659	0.08070738	-0.5623925	-0.0037582
cg08898349	TRUB1;TRUB1	blue	0.48564447	0.54150404	0.0415615	0.15482227	-0.148118	-0.0471836
cg06805227	RIMBP2;RIMBP2;RIMBP2;	blue	0.37663494	0.72746882	-0.2411163	-0.0191715	-0.4565044	0.04153728
cg12074857	CDH11;CDH11;CDH11;CD	blue	0.62647848	0.82804841	-0.1195524	0.15878156	-0.3873884	0.10642586
cg09574618	C1QTNF2	blue	-0.4534497	-0.5175042	0.38858318	0.05105254	0.20080377	0.02373231
cg14651645	RP11-90P13.1	blue	0.68485383	0.8188087	-0.0448558	0.16053832	-0.3885462	0.1377811
cg04458776	SLC22A6;SLC22A6	blue	0.29121245	0.78991677	-0.0583735	-0.0021516	-0.6820562	0.02157794
cg08137495	MCC;MCC	blue	-0.4176296	-0.6230357	0.35958298	-0.1180063	0.43300635	-0.003716
cg07146435	LOXL4	blue	-0.5340828	-0.7223492	0.19375405	-0.0368373	0.52126123	-0.1834624
cg23869663	AC113167.1	blue	0.52180594	0.76704316	0.1323906	0.12116376	-0.477555	0.10264629
cg14404008	SLC38A9;SLC38A9;SLC38	blue	0.30921378	0.82496391	-0.122474	-0.0956085	-0.8348233	-0.0281511
cg06643528	RP11-298O21.5;RP11-	blue	0.63908988	0.81514027	0.19040832	0.2006329	-0.4310252	0.25443958
cg19050418	TMTC2;TMTC2	blue	0.5962126	0.83671227	-0.0687702	0.17946885	-0.4361199	0.10616677
cg21784917	MAGI2;MAGI2;MAGI2;MA	blue	-0.324862	-0.7714372	0.12196878	0.0980371	0.70908383	-0.1172157
cg11876705	RASGRP4	blue	0.29554747	0.78533719	0.12428385	-0.0646277	-0.7609527	0.0265483
cg16989018	ZNF713;ZNF713;ZNF713;R	blue	0.56747614	0.74207606	-0.2608046	0.0432964	-0.3547526	0.1089233
cg18082332	U6;RP11-2O17.2;RP11-	blue	0.55825284	0.74108445	-0.0478372	0.1840973	-0.2906781	0.25355685
cg01593385	FGG;FGG;FGG;FGG;FGG;	blue	0.40937689	0.87799055	-0.0528785	0.0201653	-0.6520192	-0.0193774
cg07027073	KRT15;KRT15;KRT15;KRT	blue	0.55762009	0.7918484	0.06525248	0.1175554	-0.4074199	0.16973196
cg22504696	KCNK18	blue	0.56155028	0.69236369	-0.0512763	0.15427126	-0.3410451	0.22997459
cg13482327	CYP2A13	blue	0.42736601	0.81206729	0.03235716	-0.0008391	-0.6132523	0.00671131
cg01837269	MIR125B1;RP11-	blue	-0.306053	-0.5777678	0.2317125	0.10597778	0.45055443	0.05077049
cg24784310	CAMK2B;CAMK2B;CAMK	blue	-0.352058	-0.5234351	0.21512581	0.10512327	0.46511883	-0.0287646
cg06368932	CYP24A1;CYP24A1;CYP24	blue	-0.3697542	-0.6361472	0.2116202	0.13297697	0.47173358	0.12619492
cg19119016	C3orf30;C3orf30;RP11-	blue	0.49688768	0.84736401	-0.082356	0.0576227	-0.5350571	0.0157933
cg26233951	MIR381;MIR487B;RP11-	blue	0.46364358	0.85793349	-0.0667971	0.04706139	-0.5713611	0.14116664
cg04872399	TNNT2;TNNT2;TNNT2;TN	blue	0.70625341	0.75589956	-0.0145285	0.20409876	-0.3481952	0.29587785
cg10518969	RP11-540H22.2;RP11-	blue	0.33902377	0.79937145	-0.1180643	-0.006625	-0.6241512	-0.0416592

cg02797577	GPR78;GPR78;GPR78;GPR	blue	-0.3979581	-0.7661777	0.12787039	0.00082285	0.64910096	-0.0064306
cg24456981	RP13-16H11.1;RP13-	blue	0.47283628	0.8461608	-0.0897422	0.01373874	-0.6036698	0.0148641
cg25047768	RP11-219J21.2	blue	0.35293304	0.73476973	-0.0603863	0.0200627	-0.4461372	-0.0956349
cg20179726	FRMD4A	blue	0.57416415	0.76292416	0.03053292	0.15366305	-0.3621623	0.1183939
cg18237551	ZNF300;ZNF300;ZNF300	blue	-0.3620158	-0.7381643	0.08876229	0.06171342	0.61892676	0.0101435
cg23676215	RN5S226	blue	0.4860565	0.83923302	-0.2666592	0.0009187	-0.5756834	-0.0466885
cg05894322	ABCA10;ABCA10;ABCA10	blue	0.333735	0.76480609	-0.4143356	-0.0222555	-0.5252029	-0.1789446
cg02065676	AC093911.1	blue	0.47143149	0.68404346	-0.2619383	0.1437257	-0.2539228	-0.1547209
cg11522125	RP13-539F13.3	blue	0.43147422	0.68617741	-0.3393236	0.10362391	-0.2633633	-0.0556243
cg13735822	RP11-356O9.2;RP11-	blue	0.36986721	0.43718957	-0.3903038	-0.0635694	-0.1828109	-0.1157412
cg24735941	GRM7-AS1;GRM7;GRM7	blue	0.72733935	0.69152472	0.05425256	0.26225036	-0.1607684	0.2423384
cg25131620	KRBOX1-	blue	0.34726794	0.68354502	-0.1150139	0.00498774	-0.3841529	-0.0182191
cg16389610	SERPINA1;SERPINA1;SER	blue	0.32351104	0.7548688	0.09253688	-0.0511217	-0.6302317	-0.0237141
cg23990624	MTUS2-	blue	0.51535411	0.85162953	-0.1934547	0.05105438	-0.5031256	-0.007955
cg26746540	CTC-276P9.1;CTC-	blue	-0.2824828	-0.4222352	0.14800523	-0.0088397	0.27154627	0.11005435
cg15196903	PGGT1B;PGGT1B	blue	0.61380209	0.85120177	-0.0970311	0.07515299	-0.5433909	0.13074548
cg04164184	AC018730.1;AC018730.1;A	blue	-0.5711478	-0.6381431	0.05717518	-0.0748303	0.38081074	-0.1608406
cg01129454	RN5S199	blue	0.47867118	0.62768241	-0.328747	0.08854183	-0.2556124	-0.0219889
cg18847127	RP11-699L21.2;RP11-	blue	0.53513277	0.81503272	-0.2287514	0.08678573	-0.3905433	0.03449676
cg09850101	RTN1	blue	0.39402758	0.85557633	0.04900446	0.00524541	-0.6929438	-0.0059937
cg08505133	LEMD1;LEMD1;LEMD1;LE	blue	0.3781404	0.86983303	-0.1012984	0.03524969	-0.6619349	-0.0121587
cg23691287	U6;RP11-451B8.1	blue	0.64688576	0.77496474	0.06572187	0.18031902	-0.3678554	0.14457422
cg12003463	ANK1;ANK1;ANK1;ANK1;	blue	0.37199132	0.75832156	-0.0624041	-0.0256839	-0.4284268	-0.0315006
cg09130308	XXyac-YM21GA2.4	blue	0.49362177	0.84595714	-0.1160246	0.04668278	-0.5290564	-0.0194892
cg05297088	RP11-1152H15.1;GALC	blue	0.33426229	0.83384892	-0.2208262	-0.0205478	-0.6495968	-0.0066218
cg13289231	CTC-420A11.2	blue	0.52582247	0.85364741	-0.1390455	0.12246253	-0.492128	0.02334049
cg24143287	C10orf90;C10orf90;C10orf90	blue	0.57144674	0.71983897	0.19180854	0.06213461	-0.477395	0.22295595
cg10120683	MAVS;MAVS	blue	-0.471131	-0.7236505	0.05896462	0.03047565	0.52560415	-0.059194
cg10242763	CACNA2D1;CACNA2D1;C	blue	-0.4400803	-0.5057353	0.21573582	0.08838107	0.34947943	-0.1123354
cg09363604	MIR575	blue	0.36110782	0.78042655	-0.1280616	0.06621906	-0.5173182	0.0490905
cg10868658	NCKAP1;NCKAP1	blue	-0.4074791	-0.77253	0.14627647	0.00915044	0.54500297	-0.0771607
cg12841020	PGF;PGF;PGF;PGF;PGF	blue	-0.4796187	-0.6214551	0.14418756	0.03085388	0.48071529	-0.1390311
cg05191222	RP11-34C15.1;RP11-	blue	0.48999009	0.82882409	-0.3181043	0.07726107	-0.4335165	-0.0141346
cg00306571	CTC-340A15.2;CTC-	blue	0.51020718	0.80841518	0.17681348	0.11713919	-0.5320087	0.08195296
cg15698244	FILIP1;RP11-415D17.3	blue	0.35376208	0.83990767	-0.1289215	-0.0747313	-0.7213057	-0.0187202

cg19152529	GNG4;GNG4;GNG4	blue	0.39937405	0.85501005	-0.0599034	0.05886014	-0.6801548	0.01242757
cg21319053	GDNF;GDNF;GDNF-AS1	blue	-0.436987	-0.7398948	0.23570955	-0.0300824	0.54315423	-0.0776745
cg00959249	PCSK1;PCSK1;PCSK1;CTD-	blue	-0.4054997	-0.6210992	0.09682252	0.07271163	0.45880029	0.00828237
cg02770479	RP1-149A16.12	blue	0.34530708	0.74598629	-0.1413171	-0.1072263	-0.679672	-0.0797231
cg03216518	RP11-889D3.2	blue	0.54848586	0.79829523	-0.0844202	0.05903638	-0.4742234	0.084313
cg27553708	PRKRIP1;PRKRIP1;PRKRI	blue	-0.4149726	-0.669198	0.03195479	0.02717899	0.56345561	-0.0168964
cg21475771	AC106875.1;AC106875.1	blue	0.58802884	0.87434117	0.09088942	0.16659298	-0.5168157	0.1762864
cg13034682	SRRM4	blue	-0.4907443	-0.8356979	0.13214216	0.03272686	0.59593512	-0.0079142
cg09533926	ATP13A4-AS1;ATP13A4-	blue	0.65605746	0.72926434	0.00849593	0.14729507	-0.3399684	0.22069548
cg02924053	AC005281.2;SCIN;SCIN;SCI	blue	-0.4302676	-0.521064	0.13292294	0.03845374	0.3537166	0.07591398
cg14449051	SLC6A15;SLC6A15;SLC6A	blue	-0.4176753	-0.6529476	0.35952325	0.02310642	0.41607164	0.13295796
cg16912478	SLC34A2;SLC34A2;SLC34	blue	-0.5391146	-0.6310253	0.14720428	-0.04991	0.40746996	-0.2173066
cg04986668	RP11-710A11.2	blue	0.60369811	0.71409783	0.14009957	0.21086839	-0.2829729	0.15639515
cg04665818	FAM13A;FAM13A;FAM13	blue	-0.461738	-0.812344	0.07675615	-0.0026787	0.59334492	-0.0524864
cg15671158	CAMLG;CAMLG	blue	-0.4339592	-0.5063576	0.2106424	-0.0190866	0.32345374	-0.0581455
cg19859957	GP5;GP5	blue	-0.403817	-0.5830969	0.13221755	0.08509312	0.52356455	-0.0400127
cg23405243	PROK1;PROK1	blue	0.34351203	0.73386186	-0.0979474	-0.0222156	-0.5136808	-0.0761672
cg17127956	SACS	blue	-0.452925	-0.4077522	0.08830186	0.06623895	0.27212817	-0.1603546
cg06480998	RP11-478K15.1	blue	0.69986931	0.71476712	0.24523338	0.21912915	-0.302445	0.28819672
cg16416014	U6atac	blue	0.35904337	0.67746463	0.14339081	0.09141198	-0.3827525	-0.0153151
cg18203604	AC073257.1	blue	0.52813669	0.63203074	0.07899642	0.21043955	-0.2701143	0.26846496
cg15179825	GDF10;GDF10	blue	-0.5084331	-0.6884201	0.13607005	0.04497415	0.52493847	-0.1196829
cg16080852	ANO4	blue	-0.4532663	-0.5027986	-0.0826297	0.01140798	0.30453511	-0.1104002
cg03178748	OSBPL1A;OSBPL1A	blue	-0.5158479	-0.6967437	0.08798439	-0.1265255	0.51637292	-0.0739218
cg03455845	RP5-898J17.1	blue	0.45523468	0.8601094	-0.2127092	0.10064889	-0.4962025	-0.0186599
cg01779273	ADAMTSL1;ADAMTSL1	blue	0.62608417	0.82289162	0.04735771	0.21581864	-0.3515583	0.16888842
cg25388528	KRTAP20-1	blue	0.34992927	0.71535449	-0.2089501	0.06597256	-0.3959421	-0.1206442
cg07029759	ANK2;ANK2	blue	0.46112345	0.77088343	-0.2861206	0.06940287	-0.414227	-0.1039714
cg07454091	PTPRN;PTPRN;PTPRN;PTP	blue	0.38908293	0.58345365	-0.1130109	-0.0512066	-0.3915211	0.05273597
cg05489904	SCGB3A2;SCGB3A2;SCGB	blue	0.44849991	0.89588225	-0.0809512	0.04570008	-0.6728799	0.02675457
cg11995363	7SK	blue	0.20105226	0.53183652	-0.1589269	0.01300009	-0.3362236	-0.0759968
cg07687349	TBR1;TBR1;TBR1;TBR1;TB	blue	-0.546246	-0.651423	0.0228994	-0.0310165	0.48397539	-0.2000318
cg20826165	RP11-196H14.2	blue	0.59225811	0.8830191	-0.0046778	0.20387985	-0.5191165	0.18091822
cg17767960	LINC00159	blue	0.61749902	0.79074119	-0.2058557	0.13569736	-0.3354052	0.12265123
cg17027233	MEIS3;MEIS3;MEIS3;MEIS	blue	-0.4427011	-0.7851735	0.23675165	0.12074187	0.56851657	0.01039698

cg03757262	CTB-99A3.1	blue	0.55509096	0.70378989	0.32466149	0.14633097	-0.4148313	0.12654484
cg00689835	G6PC2;G6PC2;G6PC2;G6P	blue	0.46375829	0.73710419	-0.231899	0.08853761	-0.3332219	0.08542432
cg17054006	PDE1A;PDE1A;PDE1A;PDE	blue	0.6882557	0.72984733	-0.0388276	0.24752298	-0.2182107	0.09605317
cg19119995	NCOA6;NCOA6;NCOA6	blue	0.6942861	0.66690401	0.00046077	0.28599783	-0.2355444	0.17434923
cg09220783	RP11-116D17.1	blue	0.59610069	0.74816861	-0.1734766	0.23335874	-0.2203059	0.03918292
cg25131420	U4;GRIA4;GRIA4	blue	0.46192654	0.7587546	-0.3569562	-0.01425	-0.3734288	-0.0670103
cg22476744	AC004458.1	blue	0.76018869	0.75738324	0.15086169	0.28734882	-0.2916612	0.373913
cg16680125	EIF2D;EIF2D;EIF2D	blue	0.29596619	0.8171244	-0.0319216	-0.1205162	-0.802648	0.03051976
cg15927086	CTB-11I22.2;CTB-11I22.1	blue	0.33746673	0.78940936	0.02750073	-0.0124853	-0.6943424	0.06814284
cg22648994	RP11-382N13.2	blue	0.56497839	0.61688749	0.3342735	0.15979118	-0.2792858	0.12975247
cg04725937	ACTG2;ACTG2;ACTG2;AC	blue	0.43869991	0.79459535	-0.0841382	0.0113414	-0.4697001	-0.0412174
cg20449404	C3	blue	0.57534016	0.77571527	-0.1011552	0.07523217	-0.436676	0.00166427
cg22063844	A2ML1;A2ML1;A2ML1;A2	blue	0.36597697	0.49711783	-0.0517225	0.0392134	-0.2667504	0.09486412
cg15794034	AMICA1;AMICA1;AMICA	blue	-0.4259048	-0.6306121	0.02500269	0.03400973	0.47544458	-0.1824225
cg01244651	TMEM125;TMEM125;TME	blue	0.57057061	0.84089224	-0.051021	0.20759143	-0.4558086	0.1903652
cg26704844	RASGEF1C;RASGEF1C;RA	blue	-0.4209832	-0.6191226	-0.0269097	-0.0108715	0.50034348	-0.1616237
cg15191052	RP11-383H13.1	blue	0.47100954	0.74541763	-0.1679547	0.09979714	-0.464264	0.03107532
cg03653672	UGGT1;UGGT1	blue	0.61575071	0.72580591	-0.017707	0.17397405	-0.3947194	0.05503134
cg14527078	AC100808.7	blue	0.42020993	0.64707488	0.13906395	0.02076254	-0.4458657	0.00808774
cg01410500	ZNF365;ZNF365	blue	-0.3907462	-0.5293599	0.12767766	0.16445776	0.43333171	0.0270723
cg03923934	OPCML;OPCML;OPCML;O	blue	-0.582476	-0.7589567	0.20680331	-0.0332049	0.47939313	-0.12812
cg00755980	C10orf107;C10orf107	blue	-0.4218207	-0.7139399	0.17421319	0.00786948	0.5528963	-0.1412159
cg11468522	CKB;CKB;CKB;CKB;CKB;	blue	-0.4881677	-0.5704478	0.08334571	-0.0862383	0.3149881	-0.2418293
cg21240441	RP11-	blue	-0.4126123	-0.3964489	0.06243292	-0.0519357	0.24411971	-0.0649084
cg13061249	ARHGEF4;ARHGEF4;ARH	blue	-0.3937666	-0.646428	0.2102554	-0.0199348	0.52705876	0.02242926
cg11192416	TRIM67	blue	-0.5182103	-0.7839344	0.10793976	-0.0282255	0.58195817	-0.1741159
cg18144849	RP11-844P9.2;RP11-	blue	0.48155335	0.85810096	0.04898746	0.03314533	-0.5969191	0.08436494
cg15399517	RP11-571O6.1	blue	0.4799646	0.73530994	-0.2198912	0.08302572	-0.327734	0.04581793
cg21434492	ATP2B2-IT1;ATP2B2	blue	0.59584508	0.84890687	0.04462837	0.15927827	-0.454532	0.14886346
cg24406668	MMP9	blue	-0.542418	-0.6581509	-0.0678359	-0.1279201	0.49092921	-0.2388694
cg00804303	NRCAM;NRCAM;NRCAM	blue	0.43844049	0.89732441	-0.0407159	0.01049041	-0.6766968	0.02170902
cg12206501	RP11-490O24.2	blue	0.61421658	0.69259964	0.10418376	0.14124542	-0.301878	0.18001285
cg24813790	PIK3R1;PIK3R1;PIK3R1;PI	blue	0.36120784	0.8541635	-0.2226342	-0.043215	-0.6742477	-0.0646733
cg09447219	RP11-766F14.2	blue	0.42210066	0.77605021	-0.1024106	0.09771389	-0.4981975	0.02985334
cg05478348	CAPRIN2;CAPRIN2;CAPRI	blue	-0.4995503	-0.5449672	0.07394865	0.09006783	0.37877977	-0.1998371

cg05928873	NEGR1-IT1;NEGR1	blue	0.36895153	0.85169885	-0.0406905	-0.0135242	-0.6989413	0.02999691
cg02642017	AC005082.12	blue	-0.2852904	-0.611809	0.11601472	0.05578688	0.53655998	0.0191928
cg13938376	OLR1;C12orf59;OLR1;C12or	blue	0.52240303	0.90102224	-0.0939135	0.0756519	-0.6071566	0.09340119
cg19476241	RP11-767C1.1	blue	0.65039139	0.69287492	-0.1590879	0.16971726	-0.2211317	0.10597485
cg26667956	C10orf31	blue	0.62283661	0.88183888	0.14437903	0.13596932	-0.5864286	0.2180353
cg05574244	RN5S405;HS3ST4	blue	0.40081191	0.8487931	-0.1997012	0.00738113	-0.5756562	-0.0042929
cg25852422	CHL1-AS2;CHL1-	blue	-0.4572838	-0.5786276	0.24543544	-0.0508619	0.31913903	-0.0311906
cg06779123	OSBPL3;OSBPL3;OSBPL3	blue	-0.4499874	-0.5027626	0.04101092	0.03676294	0.42017114	-0.0800256
cg12244133	WDR17;WDR17;WDR17;W	blue	0.58576326	0.76147777	-0.0782613	0.30564922	-0.2684874	0.05121467
cg25915410	HSP90AA1;HSP90AA1;HS	blue	0.39566825	0.81849338	0.16518388	-0.0479674	-0.6951275	0.08059523
cg01630060	KB-1083B1.1;KB-1083B1.1	blue	0.35612363	0.86141906	-0.088728	0.0522423	-0.6450655	-0.0477336
cg02472264	KRT13;KRT13	blue	0.69828544	0.79981535	0.10109471	0.13900549	-0.4183044	0.17549408
cg10448630	OTOA;OTOA	blue	0.75274832	0.72902385	0.12906376	0.14739057	-0.3169942	0.2833705
cg01464617	RP11-446J8.1;RP11-446J8.1	blue	0.57545285	0.78575289	-0.1765208	0.17965228	-0.3360919	0.0637652
cg18882786	CPS1	blue	0.26473336	0.51616471	-0.2405359	-0.0062222	-0.2922038	-0.1599298
cg25489405	AC096750.1	blue	0.47339295	0.67236912	-0.2682279	0.10099226	-0.2041144	0.03957761
cg02799370	LL22NC03-86D4.1	blue	0.64928169	0.76215965	0.3014373	0.19752399	-0.362585	0.36159195
cg08129471	DNAH7	blue	0.57047014	0.85057915	0.09105743	0.18411879	-0.5050531	0.10013353
cg23785940	CYR61;CYR61;CYR61	blue	-0.6322974	-0.721017	0.10398695	-0.1183324	0.3641594	-0.219196
cg13652888	RP11-661I21.2;ATF4P4	blue	0.6432595	0.65080874	0.25481273	0.17872489	-0.2868473	0.28251155
cg11367948	DAPK1;DAPK1;DAPK1	blue	0.3935348	0.87463098	-0.1014198	-0.0540314	-0.7559376	-0.0245788
cg11971724	AAGAB;AAGAB;AAGAB;I	blue	-0.5125223	-0.5239168	0.19479694	-0.0012298	0.40621704	-0.1788242
cg11905108	RN5S299	blue	0.6169595	0.7740533	0.09205554	0.10184786	-0.3750759	0.19278276
cg03041038	RP11-753N8.1	blue	0.39066428	0.835755	-0.2280489	-0.056058	-0.6640597	-0.0508923
cg12929809	RP11-1016B18.1;RP11-	blue	0.53862407	0.70252509	-0.0815912	0.0552533	-0.357058	0.114136
cg03685439	RP11-379I19.3;RP11-	blue	0.51668739	0.69750905	0.30390259	0.15824272	-0.4160524	0.16648561
cg12356261	NES	blue	-0.618482	-0.7007485	-0.0457838	-0.0438133	0.47292846	-0.2562174
cg26083141	MYH6	blue	0.37560097	0.53853532	0.24160793	0.03372519	-0.4636409	0.22545547
cg12168076	SBK2	blue	0.6919038	0.66697132	0.06059927	0.19797009	-0.2782142	0.26660939
cg17582637	AHR;AHR;AC003075.4;AC0	blue	-0.3252994	-0.6946245	0.1055666	-0.0518937	0.61570979	0.02712886
cg19733792	snoU13;BACH1	blue	0.51271132	0.74084362	0.3243213	0.08938691	-0.5071914	0.18796013
cg04535746	MITF;MITF;MITF;MITF;MI	blue	0.43457632	0.88767025	-0.0462086	-0.0146675	-0.6977371	0.00760014
cg15982625	RP4-718D20.3	blue	0.5060474	0.83058263	0.27069449	0.05265515	-0.5927903	0.09842053
cg22223941	MAPK10;MAPK10;MAPK1	blue	0.49155133	0.83624963	0.03599559	0.17573964	-0.5025198	0.10512469
cg26016712	STX12;STX12;STX12	blue	-0.2392549	-0.469684	0.14196275	0.0866746	0.42048232	0.12861181

cg09009538	RP11-753H16.3;RP11-	blue	-0.4269977	-0.4597019	0.14955133	-0.0084744	0.25424723	-0.0781338
cg06921345	RP11-379I19.1	blue	0.43356084	0.81529438	-0.3024072	0.07563149	-0.4962502	-0.1530331
cg19697981	OSTM1;OSTM1;NR2E1;NR	blue	-0.5421485	-0.6283606	0.02100264	-0.0352894	0.38682985	-0.1281472
cg27539692	RBFOX1;RBFOX1	blue	-0.2793928	-0.346651	0.22126771	0.11710565	0.2745117	0.06840337
cg14662229	RNU6-63	blue	0.43414973	0.77639295	-0.011923	0.00779579	-0.5066788	0.00574371
cg00403532	GPR77	blue	0.67471555	0.8101316	0.16026788	0.15584693	-0.422476	0.11474203
cg04010123	GAPVD1;GAPVD1;GAPV	blue	-0.3375801	-0.6511948	0.0006962	0.08464137	0.59173634	-0.0380986
cg07595845	MEG8;MEG8;MEG8	blue	0.50171993	0.89818853	-0.1618833	0.08161233	-0.567563	0.00850745
cg25968219	SERPINA1;SERPINA1;SER	blue	0.66520241	0.81200417	0.09332581	0.13153867	-0.4645619	0.23823132
cg26119028	RP11-1096D5.1	blue	0.7157655	0.77695724	-0.1638783	0.20937644	-0.28075	0.15252274
cg00512287	PHLDB1;PHLDB1;PHLDB1	blue	0.40113888	0.71491572	0.17493929	0.08302392	-0.5170077	0.23006742
cg17999652	GLDN;RP11-	blue	0.3958046	0.67778628	0.07842415	0.02549855	-0.3835811	-0.0186751
cg00340102	ERGIC3;ERGIC3;ERGIC3;E	blue	-0.4107041	-0.4604022	0.29423375	-0.0313808	0.34482496	-0.0102285
cg20998134	U6	blue	0.254054	0.59450606	-0.0673997	0.02472056	-0.4673579	-0.1437398
cg26276014	CUBN;CUBN	blue	0.64267067	0.84729015	0.04913113	0.19976711	-0.4091947	0.20633213
cg04802618	IGKJ5	blue	0.74155276	0.71281066	0.21444477	0.20601008	-0.2520952	0.25539032
cg02854917	RP11-550H2.1	blue	0.50634483	0.87085743	-0.1664752	0.09607875	-0.5622413	0.00374255
cg02778244	CTC-441N14.4;CTC-	blue	0.58925863	0.86567517	0.05549384	0.15636709	-0.4661951	0.17725953
cg25726518	RP11-412P11.1	blue	0.54795531	0.87901589	-0.0698549	0.10177828	-0.5286126	0.02126205
cg04227467	OR7A10	blue	0.24887062	0.64893052	0.35327928	-0.0631333	-0.6051185	0.0077061
cg05169077	MEF2C	blue	0.55949536	0.83324733	0.20820519	0.09397777	-0.5265303	0.15090824
cg09408098	PRRX1;PRRX1	blue	-0.4394138	-0.7843706	0.26944436	0.00071795	0.53240121	-0.0010326
cg01031999	DOM3Z;DOM3Z;DOM3Z;S	blue	0.26544826	0.79448786	-0.0386083	-0.0505257	-0.8024939	0.04849481
cg05786809	CKB;CKB;CKB;CKB;CKB;	blue	-0.4698922	-0.7563282	0.09288893	-0.0181952	0.50183976	-0.1442995
cg01420166	UNC13C	blue	0.44337792	0.86043775	0.09834721	0.05821738	-0.6404042	0.10386418
cg17254386	MCCC2;MCCC2;MCCC2	blue	0.27598023	0.79775905	-0.0035527	-0.1419529	-0.876506	0.0072774
cg07718478	CHGB;CHGB	blue	0.26704366	0.58743761	-0.2308987	-0.0651069	-0.437616	-0.100758
cg15104875	RP11-57P19.1	blue	0.40996416	0.69297689	0.23038722	0.12110555	-0.5010645	0.05189955
cg08989917	Vault;AC074391.1	blue	0.67191199	0.776815	0.07713057	0.22947701	-0.3888884	0.14045217
cg16501892	LOXHD1;LOXHD1;LOXH	blue	0.48746217	0.89713684	-0.0777984	0.11946329	-0.5864719	0.02873803
cg00341504	ARHGAP28;ARHGAP28	blue	0.40749241	0.7910746	-0.0224827	0.0320464	-0.5171938	0.04067401
cg01841050	KRT34	blue	0.34507893	0.78630605	-0.0668228	0.04018956	-0.5964157	-0.1060459
cg04917867	SDS;SDS;SDS	blue	0.54571086	0.59380999	-0.0781899	0.1925268	-0.2804145	0.14820177
cg27265170	DMRTA2	blue	-0.3908289	-0.6316293	0.03913632	-0.0091488	0.50918019	-0.1915247
cg05570980	C3orf52;C3orf52;C3orf52;C3	blue	-0.4934209	-0.6139655	0.17483053	-0.025315	0.35035208	-0.1159323

cg14267151	MUTED;MUTED;MUTED	blue	0.29760618	0.82694171	-0.0278469	-0.0927554	-0.8279724	0.00184848
cg21865293	RP1-230L10.1	blue	0.28894206	0.79215558	0.02344996	-0.1445565	-0.8326874	-0.0591593
cg26082478	RGS6;RGS6;RGS6;RGS6;R	blue	-0.3394389	-0.4657119	0.32666983	-0.0350272	0.37705273	-0.132285
cg22120345	C7orf46;C7orf46;C7orf46;C7	blue	-0.3229126	-0.5049645	0.43060252	0.01119263	0.31155575	0.13975113
cg19398812	SSTR1	blue	-0.2755585	-0.4496074	0.17843981	0.10564224	0.41213503	0.10284539
cg07208057	FUNDC2P2	blue	0.60686419	0.77697191	0.15722378	0.1759141	-0.386239	0.24351073
cg16178907	FAM101A;RP11-522N14.1	blue	0.47878694	0.86726928	0.01347017	0.11646444	-0.554326	0.04337929
cg09761747	GRB10;GRB10;GRB10;GRB	blue	0.52227619	0.78112478	-0.0597809	0.13631514	-0.5399623	0.10625615
cg21228547	RP11-806O11.1;RP11-	blue	0.71551861	0.71727051	0.12974609	0.21679098	-0.276677	0.30577485
cg26424967	MATN1;RP5-	blue	0.6200817	0.64847358	-0.0084242	0.13307922	-0.2781976	0.18752444
cg04709357	HOXA11-AS;HOXA11-AS	blue	-0.4698235	-0.6612905	0.05138735	-0.0939765	0.4069457	-0.0624424
cg12300779	SEMA4G;SEMA4G;SEMA4	blue	0.49025739	0.83455532	-0.1289563	0.17063738	-0.4335436	0.1078251
cg27607412	CREB3L1;CREB3L1	blue	0.72505128	0.85760185	-0.0034176	0.15610611	-0.4288419	0.24480073
cg01547581	PCSK6;PCSK6;PCSK6;PCS	blue	0.14329384	0.63865624	-0.2139699	-0.0749653	-0.6100615	-0.0897612
cg20127420	UBE3B	blue	0.59983505	0.67381698	-0.0297827	0.2158711	-0.2200486	0.21085824
cg10657259	FRMD1;FRMD1;FRMD1;F	blue	0.50998508	0.76706037	-0.2775294	0.14943379	-0.3660237	0.07581258
cg02644686	PDGFRA;PDGFRA;PDGFR	blue	-0.4613365	-0.5621466	0.18422756	0.04426866	0.44056971	-0.165453
cg11114020	RP11-69E9.1;EPHB2	blue	0.40172672	0.8356685	0.02853212	-0.036166	-0.6157351	0.12488636
cg13582793	NLGN1;NLGN1;NLGN1;NL	blue	-0.4694839	-0.5001586	0.32056501	0.03759966	0.34244942	0.07527363
cg01224221	RP11-267A15.1;RP11-	blue	0.49137477	0.63377938	-0.1868105	0.0623727	-0.2512	0.05699867
cg20134151	PPP1R14C	blue	-0.2243001	-0.5898827	0.13085726	0.15808199	0.57742937	0.11279746
cg25541621	FMO6P;FMO6P	blue	0.39752747	0.54058279	-0.0707792	-0.0611874	-0.2754585	0.10370692
cg21081700	HEATR8;HEATR8;HEATR8	blue	0.74844074	0.79796166	0.05241544	0.20236393	-0.3789514	0.29809217
cg27464342	RP11-315A16.1	blue	0.54135252	0.6539313	-0.2202092	0.22699777	-0.2267409	-0.029007
cg03088525	TMC5;TMC5;TMC5;TMC5;	blue	-0.5714021	-0.7396119	-0.0883048	-0.0121207	0.49503322	-0.1922067
cg25889797	SLC25A10;SLC25A10;SLC2	blue	-0.4106191	-0.7592438	0.06400198	0.07057075	0.67870908	-0.0315897
cg01589972	AC010336.1	blue	-0.313468	-0.5124907	0.35420297	0.14644944	0.45541603	-0.0111944
cg25306023	RP11-349O10.1	blue	0.42157178	0.75918219	-0.156322	0.0615963	-0.5031419	-0.0468013
cg21324045	RP11-321C1.1	blue	0.42061406	0.79194841	-0.1675312	0.09132643	-0.4553246	-0.0794921
cg05480055	SLC2A13;SLC2A13	blue	-0.4959799	-0.7338213	0.1035836	0.00417975	0.53622074	-0.088491
cg19061781	GLE1;GLE1;GLE1	blue	-0.4494996	-0.4943612	0.11323912	0.0621444	0.36963163	-0.1263297
cg16264004	MCF2L;MCF2L;MCF2L;MC	blue	0.54437655	0.72556891	-0.0997485	0.09326179	-0.3970882	0.00030811
cg14343986	RP11-6B19.2;EPS8	blue	0.52155593	0.80175051	0.19499134	0.08297965	-0.4678118	0.063269
cg12749540	IGKV1-6	blue	0.54054	0.67757891	-0.1099628	0.18121111	-0.2119688	0.04878851
cg00855466	ALDH3A1;ALDH3A1;ALD	blue	-0.018671	-0.092027	0.17733807	0.05014385	0.2645804	0.15839772

cg07072463	FLT4;FLT4;FLT4;FLT4	blue	0.69213416	0.69171659	0.15719103	0.12406768	-0.314302	0.42067636
cg19712262	L3MBTL4	blue	0.37757893	0.83492273	-0.0586583	-0.0064433	-0.6720392	-0.0107004
cg17857510	TMEM119;TMEM119;TME	blue	0.54340983	0.822392	0.00564269	0.11992129	-0.4531097	0.12272796
cg16185125	AC005027.4;AC005027.4;A	blue	-0.4470878	-0.7996443	0.01118867	0.0197394	0.68600082	-0.0644857
cg05067889	LNX1;LNX1-AS1;LNX1-	blue	0.68584417	0.75157678	0.07881241	0.20877588	-0.3613707	0.20478234
cg16681516	DMWD;DMWD	blue	-0.3233532	-0.3964366	0.31128903	0.05640782	0.26369372	0.19626306
cg19633953	AC104782.3;AC104782.3	blue	0.66371451	0.74026629	-0.0480458	0.13202322	-0.3297464	0.21534248
cg18571585	MYO1A;MYO1A;MYO1A;	blue	0.69574914	0.69516274	-0.0360171	0.21398106	-0.20269	0.13123548
cg19105462	KIRREL3;KIRREL3;KIRRE	blue	-0.4037631	-0.5586875	0.15203766	0.03131921	0.43848426	-0.1373585
cg24846725	ANO1-AS2;ANO1-AS2	blue	0.46595814	0.71077207	0.04872419	0.12725978	-0.3919785	0.07164353
cg11195002	ICAM5	blue	-0.3960026	-0.5886385	0.15032381	0.11409818	0.44444336	0.14828507
cg03154197	CLMN;CLMN;CLMN;CLM	blue	0.62236948	0.67815636	-0.0489274	0.16226676	-0.3165377	0.12759679
cg22852840	OPN5;OPN5;OPN5	blue	0.39622838	0.74613423	-0.308837	0.04088913	-0.3816528	-0.0548738
cg25684760	CERS3;CERS3	blue	0.42520175	0.68324982	0.15631921	0.09782018	-0.4190293	0.05358304
cg09049701	PGCP1	blue	0.66046501	0.67785356	-0.0715452	0.22483418	-0.1694227	0.23652206
cg02735829	OR2Y1	blue	0.36779333	0.67144429	-0.1081325	0.00655422	-0.4559726	0.04271722
cg06381693	RP11-	blue	0.65311916	0.84118032	0.00617799	0.13945531	-0.493454	0.20408484
cg18492445	SLC11A2;SLC11A2	blue	0.45479104	0.7054258	-0.1154064	0.09483059	-0.3746204	0.07648821
cg09851465	C1orf87;C1orf87	blue	-0.4082053	-0.4271765	0.29641977	0.01125282	0.1615705	0.03034341
cg19067303	DHRS7C	blue	0.35439766	0.68381418	-0.2070605	0.09383112	-0.4537252	0.07920854
cg18007463	TLL1	blue	-0.5322847	-0.8195807	0.21453467	-0.0196008	0.52252623	-0.0342112
cg24892217	MMP17;MMP17;MMP17;M	blue	0.55270904	0.60106022	0.14895719	0.03841306	-0.285983	0.1195774
cg24974365	RP11-471J12.1;RP11-	blue	-0.5806049	-0.6520665	0.10918297	-0.1242159	0.33187081	-0.2640368
cg16699645	RP11-18A15.1	blue	0.58050329	0.8367731	0.14681983	0.15488055	-0.5192524	0.16028822
cg02774439	HAND2;HAND2;RP11-	blue	-0.4504961	-0.6937326	0.20038153	0.0184239	0.4169605	-0.0685533
cg14715640	RP11-767I20.1;RP11-	blue	0.43108307	0.79157762	-0.2608826	-0.0012443	-0.5394316	-0.1248449
cg26734132	RP11-298O21.5;RP11-	blue	0.4759021	0.81339157	0.18677936	0.06166501	-0.5933844	0.1566119
cg24780234	SPOCK1;SPOCK1;SPOCK1	blue	0.43261125	0.89562993	-0.0177446	0.00312567	-0.7208075	0.04508975
cg03866607	TBX4;TBX4	blue	-0.5088871	-0.8216407	0.06294689	-0.0060862	0.5799283	0.02796692
cg22498784	KRTDAP;KRTDAP;KRTDA	blue	0.43440295	0.6796357	0.34616257	0.07351639	-0.4695141	0.09687865
cg08991927	PPP2R2B;PPP2R2B;PPP2R2	blue	0.58032468	0.85684254	0.01419501	0.13103359	-0.5184884	0.1987007
cg13243685	GLIS3;GLIS3;GLIS3;GLIS3;	blue	0.41672012	0.78523931	-0.2107875	0.03442683	-0.4761848	-0.0273614
cg11129008	SOX2;SOX2;SOX2-	blue	-0.5214136	-0.7242871	0.14956746	0.01739307	0.4829632	-0.1297507
cg13037602	TOM1L1;TOM1L1;TOM1L1	blue	0.59283992	0.85591533	0.03675988	0.17023347	-0.4526375	0.19446918
cg12642725	RERG;RERG;RERG;RERG;	blue	0.62867663	0.80569645	-0.2000823	0.07007818	-0.4027097	0.05334095

cg11909553	RP11-	blue	0.43785895	0.78289916	-0.0078782	0.04255029	-0.6051353	0.0754179
cg12567991	TRPM3;TRPM3;TRPM3;TR	blue	0.45379842	0.65694021	-0.2570855	0.07931309	-0.2887829	0.04085722
cg02871707	MRPL23-	blue	-0.4111456	-0.8517585	0.00376973	-0.0057631	0.76298097	-0.0655857
cg12429482	RP11-35O7.1	blue	0.38018377	0.61713348	-0.2091488	0.09242	-0.260048	-0.0800631
cg02546590	MEG3;MEG3;MEG3;MEG3;	blue	0.35961178	0.87872955	-0.0814789	-0.0432609	-0.7181023	0.0023947
cg01410476	RP11-626P14.2;RP11-	blue	0.17023325	0.60947001	0.09146868	0.03539195	-0.4590655	-0.1322125
cg08473766	AC096643.1	blue	0.45777613	0.78301948	-0.110763	0.09338718	-0.4671401	-0.0226344
cg06512849	MCC;MCC	blue	-0.3654366	-0.6063887	0.3079445	-0.0742936	0.46241964	-0.0055372
cg25320788	RP11-297H3.4;RP11-	blue	0.41993833	0.6991302	0.28618257	0.1206128	-0.4611937	0.12463559
cg15081500	AC147651.4;PRKAR1B;PR	blue	0.42778295	0.62374216	0.23215146	0.00734088	-0.4257113	0.16502875
cg08511578	RP11-398B16.2	blue	0.41391277	0.78497174	-0.2186013	0.04402523	-0.5329283	0.04048031
cg01595397	MED20;BYSL;BYSL;BYSL;	blue	0.27513255	0.53077397	-0.1066885	0.03326045	-0.3125844	0.01100189
cg01799681	RP1-62D2.3	blue	-0.2875192	-0.3687546	0.38806546	0.16047714	0.24328254	0.17488282
cg18442753	RP11-651L5.2;CKAP4	blue	-0.5271961	-0.7261967	-0.0125088	-6.08E-05	0.54718377	-0.1661033
cg09191232	PAPSS1;PAPSS1;PAPSS1;P	blue	-0.4275965	-0.7434239	0.20962171	0.11889331	0.65170933	-0.0204786
cg04744597	GABRB2;GABRB2;GABRB	blue	-0.5558246	-0.6410278	-0.0017592	-0.0849356	0.34009423	-0.2237236
cg26072428	CHODL;CHODL;CHODL	blue	0.37386057	0.67901898	-0.0439078	0.08855608	-0.3927854	-0.1221184
cg16525281	KCNK6	blue	-0.4393802	-0.7326131	0.12124389	0.13257565	0.66090473	-0.1117527
cg18738344	LINC00159;LINC00159	blue	0.35620302	0.80194273	0.11484324	-0.0176769	-0.6373065	0.04085097
cg16932094	PRAME;PRAME;PRAME;P	blue	0.5114157	0.59337618	0.02816056	0.08683742	-0.3128926	0.09953307
cg09151754	ZNF232;ZNF232;USP6	blue	-0.3623697	-0.6073964	0.17262732	0.08784683	0.47423985	-0.0027099
cg00770964	SGK3;SGK3;SGK3;SGK3;S	blue	-0.503873	-0.5970071	0.18628538	0.00559384	0.34975373	0.09028215
cg05795367	RYR2;RYR2	blue	0.44852338	0.74687777	-0.1528535	0.01612184	-0.4663037	0.08727941
cg24815273	RP13-497K6.1;PPARGC1A	blue	0.34662463	0.82985428	-0.1485181	0.01080433	-0.6246151	-0.0216781
cg01026660	RP1-37J18.1	blue	0.46415839	0.5648493	-0.2829285	0.17398847	-0.2029458	0.05925168
cg19686983	PHACTR1;PHACTR1;PHA	blue	0.52594161	0.90170962	0.04032481	0.08156337	-0.6225422	0.10330573
cg16184803	PITX2;PITX2	blue	-0.4197636	-0.784815	0.13696289	0.04027717	0.62572715	0.02840312
cg21048688	Y_RNA	blue	0.50995439	0.74259208	-0.0999185	0.08223212	-0.3565175	-0.0267334
cg09910207	KERA	blue	0.64820659	0.62926458	0.42101666	0.22678471	-0.278341	0.33530115
cg04697218	POU6F2;POU6F2	blue	0.60345887	0.79518687	-0.2639984	0.08996125	-0.3696027	0.01524158
cg11207886	GRIK4;GRIK4;GRIK4;GRI	blue	0.37788038	0.80790735	-0.129081	-0.0665652	-0.7070291	-0.0437693
cg08057361	PTPN3;PTPN3;PTPN3;RP11-	blue	0.56349173	0.69978686	-0.2012946	0.14932403	-0.3165096	0.29099286
cg25838936	PRKDC;MCM4;PRKDC;PR	blue	0.27512784	0.80741318	-0.0421823	-0.0103159	-0.7152108	-0.0353132
cg18540157	NAALAD2;NAALAD2;NA	blue	-0.425371	-0.6793786	0.0775934	-0.0978601	0.4756958	-0.0901982
cg27063497	RP11-575B7.3	blue	0.37022725	0.7427502	0.22117534	-0.0087699	-0.6323847	0.02604708

cg19712746	KDR;KDR	blue	-0.5272913	-0.6127574	0.1371202	-0.0710778	0.43006133	-0.2126219
cg05774900	SYT10;SYT10	blue	0.32369515	0.54522808	0.05789818	-0.056146	-0.3895069	0.00021791
cg13701273	EPHA5;EPHA5;EPHA5;RP1	blue	-0.530319	-0.7410505	0.13775632	-0.056309	0.5114969	-0.0568579
cg18411429	ANGEL1;ANGEL1	blue	0.60350463	0.72093123	-0.1964661	0.24125718	-0.2173239	0.08066382
cg10097072	RAB19;RAB19	blue	0.44396286	0.65013143	-0.2133747	0.01641311	-0.3558184	0.04682093
cg19005707	GABRA5;GABRA5;GABR	blue	-0.2432067	-0.5473289	0.15328383	0.04085959	0.43764685	0.17541972
cg23701033	SLC44A4;SLC44A4	blue	0.39107536	0.51371084	-0.0615285	0.06275738	-0.2394254	0.07526215
cg09667617	PSD3;PSD3;PSD3;PSD3;PS	blue	0.42345744	0.71056779	-0.2186925	0.08934855	-0.4220001	0.02419921
cg17791346	RP11-354H21.1	blue	0.50323893	0.75713448	-0.2882635	0.15121149	-0.2436737	-0.0999815
cg20437604	ANXA9;ANXA9;ANXA9	blue	0.58147146	0.74933425	0.12984927	0.12008107	-0.4271624	0.24563066
cg26770074	MTPAP;AL161651.1;MTPA	blue	0.55989885	0.78415505	0.13272566	0.02927401	-0.4939388	0.06988709
cg14447523	RP11-680F20.9;RP11-	blue	0.41114917	0.58126671	-0.0747495	0.14584901	-0.3066117	0.21375767
cg18707141	RP11-1143G9.4	blue	0.32883816	0.52052613	0.08100208	-0.0718186	-0.4017097	-0.0299755
cg13302842	PTCHD4;PTCHD4;PTCHD4	blue	-0.3871922	-0.5319146	0.03918896	0.0250747	0.45444275	-0.0175703
cg08354137	KRT13;KRT13	blue	0.37054709	0.7188126	-0.091484	-0.0185576	-0.4610386	-0.0500819
cg02825243	STK17B;STK17B	blue	0.27511706	0.82099509	-0.0726251	-0.1192538	-0.8510411	-0.0558632
cg25065906	CEACAM16	blue	0.54393171	0.83794497	-0.0970561	0.10454982	-0.4508395	0.07068245
cg15504958	RP11-25I9.2	blue	0.46604612	0.79958686	-0.2438795	0.03892463	-0.4234932	-0.017312
cg11443523	RP11-	blue	-0.540179	-0.6436063	0.06154027	-0.1106023	0.37582135	-0.1443141
cg02746232	MAP2K4;MAP2K4	blue	0.50160325	0.74672581	-0.0281354	0.18750369	-0.3491259	0.02907807
cg20527168	BCKDK	blue	-0.6187287	-0.7052726	0.00431248	-0.1014631	0.46193794	-0.2054263
cg14487577	RP11-771D21.2;MIR133B	blue	0.70795262	0.77681234	0.14579905	0.23087261	-0.2618693	0.16557519
cg15890210	CYP19A1;CYP19A1;CYP19	blue	0.76002332	0.78701736	0.0322351	0.27585199	-0.2778534	0.31799822
cg18122767	DLK1;DLK1;DLK1;DLK1;D	blue	0.33008996	0.83924814	-0.1484994	-0.0384345	-0.6854667	-0.1565106
cg00222174	VOPP1;VOPP1;VOPP1;VO	blue	0.34861658	0.79919353	0.20324553	-0.0462007	-0.7639889	0.09447482
cg15352065	AC009262.2	blue	0.55171878	0.72678261	-0.0123405	0.0979788	-0.3728352	0.17245177
cg10235253	AL390119.1	blue	0.57260117	0.78000155	-0.2479723	0.05077021	-0.3923472	0.03250121
cg00698204	SIM2;SIM2;SIM2	blue	-0.548921	-0.7898478	0.15312898	-0.0458601	0.48189733	-0.0757994
cg04034958	GTF2A1L;GTF2A1L;GTF2A	blue	0.49542079	0.62254354	0.38775073	0.13064551	-0.3879395	0.19481498
cg15859435	RND3;RND3;RND3;RND3;	blue	0.35500654	0.79619948	-0.1783999	-0.0887931	-0.6698994	0.07535828
cg12513763	SLAMF9;SLAMF9;SLAMF	blue	0.52005068	0.56606829	0.56028257	0.18381334	-0.2758042	0.25628217
cg24324984	NANOS2	blue	0.43644761	0.78831625	-0.0148533	0.11519276	-0.4710933	-0.0339653
cg25852941	DNAJC21	blue	-0.2153953	-0.3703158	0.08051293	0.0174761	0.3311124	-0.0186781
cg05248581	CTD-3148I10.1	blue	-0.5461602	-0.6966028	0.18653684	-0.080128	0.44012743	-0.097843
cg08583841	FCRL3;FCRL3;FCRL3;RP11-	blue	0.54526735	0.81883346	-0.0507718	0.12457851	-0.4997098	0.10492622

cg20924302	TTC7B;TTC7B	blue	0.49813391	0.87888621	0.11880573	0.11659945	-0.5937956	0.11880126
cg07172476	AE000658.1	blue	0.49656976	0.8256449	-0.1138792	0.23455491	-0.470644	0.01296157
cg06610978	U6;AC017074.2	blue	0.34645791	0.81824657	-0.0046936	-0.0387348	-0.7466247	0.00207533
cg06371135	RP11-	blue	0.2760433	0.82047062	-0.09602	-0.0865198	-0.8348631	-0.054546
cg19720265	MARK1;MARK1;MARK1;	blue	-0.4777258	-0.7340361	0.16409027	0.05713337	0.52342154	-0.0913368
cg27113868	CTD-2003C8.2;CTD-	blue	0.19768637	0.43875457	-0.1366992	-0.1625209	-0.2833631	-0.1689119
cg10079022	AC009142.1;CDH13;CDH13	blue	0.40961998	0.76335476	-0.1730037	0.01426892	-0.4648173	-0.0403758
cg16714427	CDH10;CDH10	blue	-0.2566147	-0.4885768	0.12656638	0.02727104	0.38250493	0.06296911
cg16298087	KIF13A;KIF13A	blue	0.36434354	0.36998263	-0.0533585	0.07702995	-0.1383523	-0.2286475
cg13755349	RP11-143E21.7;RP11-	blue	-0.3954218	-0.699166	0.14719394	-0.0022737	0.52618187	-0.0010201
cg04154354	FOXA1;TTC6	blue	-0.5147755	-0.7934402	0.14204155	-0.0247416	0.52824626	-0.0165592
cg13852730	TLN2;TLN2	blue	0.76019179	0.78906086	0.13530509	0.23199289	-0.3311295	0.35056307
cg00950004	TPM3;TPM3;TPM3;TPM3	blue	0.37609687	0.83089433	-0.0105357	0.03483723	-0.623371	0.06626311
cg23203890	FGF23	blue	0.59979168	0.73993009	-0.187042	0.11624482	-0.3420914	0.08743626
cg21578720	UTS2D;UTS2D;UTS2D	blue	0.43335591	0.65180482	-0.1384963	-0.0145091	-0.4820443	0.05337896
cg10630111	RP4-734C18.1	blue	0.56749199	0.71770131	0.32291005	0.11745717	-0.4689163	0.25191023
cg15722151	RTBDN;RTBDN;RTBDN	blue	-0.3385328	-0.7262946	0.20136682	0.05016433	0.55668027	0.02915176
cg15794372	RP11-138H10.2	blue	0.64294942	0.68479828	0.1013531	0.22810413	-0.227163	0.15921991
cg09105123	AC005009.1	blue	0.47074448	0.86924497	-0.189573	0.06333688	-0.5314501	-0.0565718
cg00839597	KAZALD1;KAZALD1;KAZ	blue	0.70913831	0.75292113	0.01304228	0.25469345	-0.271491	0.1604772
cg10583651	AC053545.3	blue	0.3339271	0.81338527	-0.1460533	-0.1280032	-0.7209034	-0.1017713
cg12155356	RP11-497E19.1;RP11-	blue	-0.5020332	-0.5475869	0.09744261	-0.0114093	0.31336419	-0.1897226
cg25941151	TYR;TYR;TYR	blue	0.32746822	0.71987011	-0.2048389	-0.0076803	-0.5050989	-0.1094295
cg15181928	AC133633.1	blue	0.56141108	0.51882752	0.32529368	0.24674719	-0.1461326	0.21989477
cg05703230	RP11-341G5.3;RP11-	blue	0.29719343	0.79250069	-0.0964048	-0.0779778	-0.6779962	-0.0098579
cg25891756	snoU13;TTC18;TTC18	blue	0.53960446	0.82233527	-0.1634947	0.15513764	-0.4176077	0.06628442
cg09052978	RESP18;RESP18	blue	0.30744536	0.67540898	-0.2930786	0.02230788	-0.4617586	-0.1583453
cg04549489	PTPRO;PTPRO;PTPRO;PTP	blue	0.56703499	0.64808553	-0.1824856	0.19017554	-0.2308126	0.17776156
cg19995899	FN1	blue	0.42284676	0.85253884	-0.1228307	0.04548175	-0.5945025	-0.0040468
cg18333061	TAF4;TAF4	blue	-0.1918251	-0.7002148	0.13932414	0.17144905	0.66501254	0.0774566
cg18501112	C14orf43;C14orf43;C14orf43	blue	0.32385694	0.55550541	0.0204182	0.06066072	-0.3957331	0.13200367
cg08243849	PUM1;PUM1;PUM1;PUM1;	blue	-0.5906723	-0.708147	0.16391623	-0.0105284	0.48988851	-0.1035157
cg26214074	C2orf16	blue	0.53733868	0.82396685	-0.0109085	0.09598732	-0.5524516	0.13947116
cg02396944	RP11-956E11.1	blue	0.5259002	0.81144827	0.14736609	0.12272709	-0.5303525	0.12165567
cg26723590	RP11-372M18.2;RP11-	blue	0.71158072	0.77660238	-0.0266896	0.2200194	-0.3174538	0.25076559

cg09816756	FTLP10	blue	0.318365	0.85992572	-0.1000042	-0.0416032	-0.7907984	-0.0081174
cg24441621	NAT2;NAT2	blue	0.46812806	0.82475693	0.00365854	0.17300497	-0.4993163	0.02878241
cg00433866	MOG;MOG;MOG;MOG;M	blue	0.49169841	0.56904823	-0.0149474	0.10815954	-0.2610208	0.20315852
cg10327400	CTD-2298J14.2;LRFN5	blue	-0.4779191	-0.8078134	0.27055961	-0.0396667	0.54517235	-0.0144297
cg09290707	SNORA70	blue	-0.2909894	-0.4658873	0.30344395	0.10643224	0.39330106	0.00232594
cg03331208	KRT20;KRT20	blue	0.58582665	0.80238444	0.05487244	0.11305512	-0.4109979	0.16849752
cg10392572	RP11-466L17.1	blue	0.27202935	0.73870584	-0.2061543	0.05031854	-0.4624232	-0.1483111
cg12550951	RP11-259P6.1	blue	0.42261495	0.82816138	-0.2000414	-0.0021633	-0.4816979	-0.0289431
cg16709385	COL9A3;COL9A3;COL9A3	blue	-0.5293046	-0.5820214	0.16003168	-0.070625	0.31954051	-0.1027213
cg01490163	KRT36	blue	0.43239976	0.8797954	-0.1604665	0.0294221	-0.6159552	-0.0203757
cg15097463	U6	blue	0.40845575	0.85036091	-0.1337923	0.04773206	-0.6227698	0.07860284
cg06339090	IQCF3;IQCF3	blue	0.49429079	0.71762483	-0.0751749	0.18026763	-0.3951282	0.08378916
cg08528984	PRDM16;PRDM16;PRDM1	blue	-0.4491357	-0.7204847	0.06764501	-0.0540595	0.5327263	-0.0363818
cg25838533	PTPRF;PTPRF	blue	0.64307464	0.6962874	0.08938174	0.2092951	-0.2863747	0.19431324
cg19937056	KCNMB2;KCNMB2;KCNM	blue	0.39402781	0.81560698	0.09175594	0.05139867	-0.5485096	-0.0385931
cg20914508	GAP43;GAP43;GAP43	blue	-0.7401024	-0.7473228	-0.030698	-0.0728921	0.35711595	-0.2858596
cg13984803	CCBP2;RP11-	blue	0.59320339	0.87571482	-0.0610968	0.12528567	-0.4706744	0.11396175
cg22697365	PROK1	blue	0.41747229	0.88094139	0.02371102	0.04978041	-0.6820396	0.0731525
cg26040366	snoU13	blue	0.39429487	0.80907502	-0.1745143	-0.0058439	-0.5562643	-0.0122458
cg25730790	U6	blue	0.50737078	0.54011902	-0.0953891	0.19615719	-0.1393595	0.04683286
cg06477179	SLC12A6;SLC12A6	blue	0.37351309	0.73737902	-0.0118017	0.01341068	-0.4999889	-0.0048982
cg21708916	RP11-4N23.1;RP11-4N23.1	blue	0.47411537	0.87415045	-0.0076813	0.10848311	-0.5728752	0.11062568
cg03309964	AL137024.1;PAPPA	blue	0.41764096	0.39905886	-0.2679698	0.18438315	0.00769768	0.05828335
cg06699519	CD81;CD81;CD81;AC12992	blue	-0.4183539	-0.4639881	0.15108297	-0.0393974	0.2356114	-0.105602
cg26928585	AC091705.1	blue	0.46347344	0.88386082	0.04722349	0.05758841	-0.5961237	0.00922957
cg23243617	CAMK1;CAMK1;CAMK1	blue	-0.5356572	-0.5735657	0.1931141	0.03595617	0.36489199	-0.0270613
cg16146322	AC140481.8;AC140481.8;FA	blue	-0.3711106	-0.5638971	0.29928916	0.03085551	0.38487142	0.05978544
cg07647691	UTS2D;UTS2D;UTS2D;UTS	blue	0.30015846	0.65287504	-0.1485707	-0.0447031	-0.5573453	0.02620287
cg23892856	CASQ2;CASQ2;CASQ2	blue	0.74635738	0.75175914	-0.0840428	0.26180986	-0.1904153	0.15531802
cg00937742	LYPD1	blue	0.36689855	0.45710364	0.15787401	0.1379811	-0.2399203	0.06255947
cg16961648	AL662890.1	blue	-0.3826858	-0.7434269	0.17188555	0.0769804	0.65980987	-0.0852806
cg06261620	SERPINA5;SERPINA5;SER	blue	-0.453465	-0.4719614	0.06641592	-0.0318727	0.24268013	-0.1294001
cg17921439	C10orf90;C10orf90;C10orf90	blue	0.59529812	0.80656856	0.11831751	0.18229439	-0.4348586	0.13722367
cg13029887	ARHGEF4	blue	0.40643462	0.63531592	0.04388755	0.06962819	-0.3695562	0.06432134
cg06373029	RP11-558A11.2	blue	0.67792631	0.84257112	0.052613	0.13231198	-0.4506432	0.22760307

cg26495109	GNAO1;RP11-	blue	-0.5372569	-0.5735179	0.0977795	0.03658508	0.39866122	-0.139834
cg19968403	COL8A1;COL8A1;COL8A1;	blue	-0.4810182	-0.65453	0.12650855	-0.0190292	0.39921189	-0.2580231
cg07025782	AC002480.2	blue	0.39451444	0.82432581	-0.2038081	-0.0404151	-0.6024559	-0.0272433
cg18581663	WIPF3;WIPF3	blue	0.46115857	0.87378149	0.09886898	0.07679908	-0.6657343	0.07405623
cg12883769	U7	blue	0.30673842	0.79352761	-0.1646753	-0.0844997	-0.5866049	-0.0220078
cg09247623	PHGDH	blue	0.29374614	0.81670792	-0.1120298	-0.0165076	-0.6840258	0.10593767
cg03942051	TBX15	blue	-0.3784411	-0.6750682	0.07125614	0.12699472	0.56981953	-0.1207773
cg10066095	OTOGL	blue	0.51359934	0.89772638	-0.057512	0.02813867	-0.6026469	0.07728945
cg09965258	NFIB;NFIB;NFIB;NFIB;NFI	blue	-0.5389644	-0.6992383	0.17388134	-0.0550667	0.45075104	-0.1376087
cg22877930	RP11-454P21.1	blue	0.47785183	0.87652885	0.05213219	0.0862507	-0.6155931	0.07101909
cg02975060	MIR1185-2;RP11-909M7.2	blue	0.51220932	0.82195518	-0.0608022	0.11272657	-0.4869772	0.18895694
cg06621126	HSF4;HSF4;HSF4;HSF4;HS	blue	-0.5422784	-0.8073061	-0.1031713	-0.089053	0.60076311	-0.1122982
cg16049864	TP53INP1;TP53INP1;RP11-	blue	-0.1697806	-0.4024405	0.036544	0.00582902	0.34062996	0.07415878
cg03060228	BAIAP2;BAIAP2;BAIAP2;B	blue	0.49923312	0.67435337	-0.172899	0.04968015	-0.34373	0.07507277
cg21075140	RP11-3J1.1;RP11-608O21.1	blue	0.23043853	0.63789105	0.03983736	-0.0145587	-0.4371075	-0.0754325
cg00287589	RP4-753D10.5;RP4-	blue	0.4197027	0.86038639	-0.2035377	0.00811514	-0.64322	0.01227792
cg13309429	PBOV1	blue	0.66160753	0.78990417	-0.0531904	0.19312184	-0.3467864	0.22287347
cg09205611	CYP19A1;CYP19A1;CYP19	blue	0.5087577	0.90572939	0.02263368	0.09525452	-0.6512981	0.12665087
cg06542216	TP53INP1;TP53INP1;RP11-	blue	-0.1645739	-0.4140547	0.13646545	0.04634246	0.34361849	0.16004036
cg15343849	AXDND1;AXDND1;AXDN	blue	0.33590828	0.74905776	-0.1629691	0.0186411	-0.5739779	0.02427049
cg10520840	LINC00518;LINC00518;LIN	blue	0.63192851	0.58859711	0.42300884	0.18764039	-0.2236658	0.35693214
cg07935568	MLNR	blue	-0.3748738	-0.7586479	0.15988852	0.07020789	0.67183184	-0.0234571
cg11898209	FER1L4;FER1L4;FER1L4	blue	-0.3681648	-0.734653	0.0758992	0.13301781	0.6435862	-0.043541
cg26921638	WNT3A;WNT3A	blue	-0.5421215	-0.6652735	-0.067539	-0.0841259	0.41781391	-0.2591465
cg12018403	DNAAF3;DNAAF3;DNAA	blue	-0.4750826	-0.7754367	0.05621426	-0.0081164	0.56217441	-0.0194815
cg20428144	APOLD1;RP11-	blue	0.4308517	0.65365545	0.18641102	0.09106406	-0.410256	-0.0309535
cg11104634	HSPH1;HSPH1;HSPH1;HSP	blue	-0.3478065	-0.4994174	0.13277835	0.04488783	0.31624238	0.05248561
cg02029970	SGCD	blue	0.40094288	0.74662666	0.05695593	0.05321354	-0.4944631	0.0321494
cg00731526	UBE2F;UBE2F;UBE2F;UBE	blue	-0.3502474	-0.6783673	-0.0451711	0.00794016	0.57721147	-0.0005606
cg09224845	CNTLN;CNTLN;CNTLN	blue	-0.3320379	-0.7210841	0.25547673	0.03505554	0.6538913	0.08976313
cg27638441	RP11-442O18.1	blue	0.42666848	0.84072032	-0.16435	0.05806412	-0.585889	-0.0437632
cg19307750	RGS7	blue	0.38123977	0.65417861	-0.0944713	0.16002877	-0.2896431	-0.0352683
cg22563825	ANO2;ANO2;ANO2	blue	0.37731382	0.85324742	-0.0549414	-0.0510328	-0.7309613	0.05235251
cg04744713	AC100803.1	blue	0.44111091	0.77214225	0.02791697	-0.0122636	-0.4582137	-0.0197496
cg09742177	RP11-	blue	0.50263163	0.60410235	-0.0068536	0.13963937	-0.2525336	0.15339079

cg21568131	SLC47A1;SLC47A1;SLC47	blue	0.32482114	0.8323451	-0.2078059	-0.031801	-0.7051099	-0.1092261
cg16294566	WT1-AS;WT1-AS;WT1;WT1-	blue	-0.4557194	-0.5765749	0.04057181	-0.0508809	0.35901791	-0.0115413
cg08926287	SLC6A6;SLC6A6;SLC6A6;S	blue	-0.2975468	-0.3981665	0.24909214	0.1621845	0.30528782	-0.052228
cg25264351	RP11-507P23.3;RP11-	blue	0.48578881	0.70758675	-0.132192	0.11385036	-0.3567846	0.08076927
cg07321742	PLCB1;PLCB1	blue	0.39040426	0.89792543	-0.0709076	0.0194951	-0.696679	-0.0555469
cg23440636	RP11-79P5.6;RP11-79P5.2	blue	-0.5000546	-0.5465588	0.42317585	0.03168726	0.22729609	-0.0709196
cg12788535	TACR2	blue	0.46312351	0.51488493	-0.2008224	0.04222713	-0.2116976	0.11091893
cg15883606	PAK1;PAK1;PAK1	blue	0.47263945	0.84941491	0.0709917	0.01235268	-0.6600793	0.09661521
cg26259363	DES	blue	0.52485245	0.81992463	-0.034921	0.08972333	-0.5066423	0.12883244
cg21678760	TSEN15;TSEN15;TSEN15;T	blue	-0.4132379	-0.6055908	0.15106854	0.05967187	0.53564363	-0.0817214
cg17345930	CTSF;CTSF;CTSF;CTSF	blue	0.40479629	0.79718023	-0.0322844	0.01817212	-0.5438174	0.03020277
cg27234090	USP2;RP11-305N23.1;RP11-	blue	-0.4148636	-0.7501976	0.03204585	-0.0927451	0.58213746	-0.0306339
cg14231749	CA10;CA10;CA10;CA10;CA	blue	-0.445073	-0.524032	-0.0270042	-0.0154791	0.26550289	-0.1535209
cg24606069	C13orf44-IT1;C13orf44-AS1	blue	0.41161106	0.86481589	-0.0790924	0.01227229	-0.6717195	0.0619091
cg21306761	RP3-523C21.1	blue	0.40928811	0.59257115	-0.1958983	0.10106265	-0.2819813	0.08098101
cg24465041	Y_RNA;AC068610.5	blue	0.36437076	0.64497359	-0.3748579	0.01487529	-0.2112513	-0.1222924
cg04339790	EMP2;EMP2	blue	0.35747899	0.81796275	-0.0673581	0.03101252	-0.5808192	0.03042555
cg11850392	PDIA6	blue	0.34346484	0.37511752	-0.3025717	0.08717343	-0.07785	-0.1173287
cg06180758	RP11-326E22.1;RP11-	blue	0.49868173	0.63748886	-0.1491446	0.16440642	-0.1838931	0.10867292
cg22866838	AC104837.1	blue	0.40428867	0.83617689	-0.0789823	0.0921066	-0.5633851	-0.0385729
cg20308528	RP11-22M7.2;RP11-	blue	0.57070449	0.83141564	-0.0125903	0.09838145	-0.4547869	0.06928224
cg25706808	AKR1C3;AKR1C3	blue	0.39940726	0.84456518	-0.1876666	-0.0118874	-0.5652538	0.00616199
cg10291288	SHROOM3;SHROOM3;SH	blue	-0.4715604	-0.7852974	0.04735599	-0.0321273	0.50276359	-0.0135551
cg08782920	PAX6;PAX6;PAX6;PAX6;P	blue	-0.6169244	-0.7097579	0.15369387	-0.0287216	0.43127402	-0.2173405
cg22536259	RP11-96A1.5	blue	0.35872264	0.75372308	-0.3224637	0.04492085	-0.4879855	-0.1069047
cg07524505	TSPAN18;TSPAN18;TSPA	blue	0.52114596	0.57348481	-0.1474042	0.16353551	-0.1650235	0.18564953
cg01193064	CBS;CBS;CBS;CBS;CBS;CB	blue	-0.4771605	-0.5971914	0.03617745	-0.0006548	0.36968708	-0.1212823
cg02540498	LYAR	blue	0.40441436	0.5711603	0.15714155	0.05020292	-0.3105802	0.15830188
cg18836206	RP11-401P9.1	blue	0.42180989	0.84256803	-0.1678034	-0.023488	-0.5652676	-0.031034
cg24249770	AC118754.4	blue	-0.4370896	-0.644296	-0.1695757	-0.0064383	0.55258602	-0.116678
cg09002886	RP11-418I22.2	blue	0.34137753	0.54245104	-0.3884034	0.04080718	-0.2187796	-0.1122184
cg11920005	CCL14;CCL14;CCL14;CCL1	blue	0.43294107	0.86051225	-0.197668	0.00753483	-0.6203551	-0.0050502
cg16429505	UFSP1;ACHE;ACHE;ACHE	blue	-0.4193573	-0.7996689	0.11678934	0.16516555	0.73838157	-0.0399325
cg04435534	AL049647.1	blue	0.42315572	0.8958083	-0.0645732	-0.0230959	-0.7634933	0.05222435
cg24718516	CDH17;CDH17;CDH17	blue	0.49409222	0.82358212	-0.1762657	0.10800046	-0.4086817	-0.0001805

cg25374714	SVOP;SVOP;USP30	blue	0.55216106	0.58717944	0.02940645	0.19984659	-0.0944719	0.04693548
cg13549428	AC009229.6;CYP1B1-AS1	blue	0.73957293	0.78466123	0.15696366	0.23222635	-0.3343998	0.29131567
cg20758834	ANK2;ANK2;ANK2	blue	0.36084363	0.82145029	0.09010599	-0.0024033	-0.6750332	-0.005278
cg09228961	AC013402.2	blue	0.46461671	0.84389188	-0.2340963	-0.0200134	-0.6270601	0.05843624
cg09365529	C1orf173;RP11-17E13.3	blue	0.27448172	0.63614966	-0.1819317	-0.0703536	-0.5313457	-0.017517
cg16266517	RP11-476H24.1	blue	0.31595694	0.78900857	-0.2048447	-0.028499	-0.5655798	-0.1165174
cg18100580	7SK	blue	0.36733658	0.80119655	0.08523316	0.10178423	-0.559425	0.01389461
cg16030006	FGFR2;FGFR2;FGFR2;FGF	blue	-0.5112642	-0.772778	-0.0393026	-0.0844093	0.51029688	-0.152323
cg24404973	NPM1;NPM1;NPM1;NPM1;	blue	-0.3296358	-0.4965571	0.09319556	0.08648906	0.41076193	0.01388937
cg21556213	EXD3;EXD3;EXD3;EXD3;E	blue	0.38797396	0.64068792	-0.1602322	-0.0009648	-0.3953346	-0.0643126
cg02403144	SVOPL;SVOPL	blue	0.47359648	0.79501601	-0.0999118	0.05823999	-0.4495439	-0.0786891
cg19304003	CAPN13;CAPN13	blue	0.38277412	0.9044974	-0.0921816	-0.0591878	-0.7354845	-0.0042454
cg18012232	RP11-189E14.3;RP11-	blue	0.62290167	0.67358539	-0.1197915	0.15513286	-0.2172995	0.12966536
cg00360531	C13orf44;C13orf44-AS1	blue	0.39119283	0.88541415	-0.0682902	-0.0041103	-0.7653923	0.04707156
cg25757752	CTD-2247C11.3;CTD-	blue	0.67689138	0.60155368	-0.0448924	0.2463947	-0.0061622	0.21903515
cg12921805	snoU13	blue	0.36905333	0.43128809	-0.382997	-0.0111527	-0.1887397	0.01934656
cg14746807	RN5S326	blue	0.71261643	0.73599019	0.01221351	0.19247662	-0.1945933	0.22508551
cg12391630	SPATA25	blue	0.63549447	0.68495552	-0.1279846	0.22414871	-0.1977507	0.24171766
cg05329976	PHACTR3;PHACTR3;PHA	blue	0.30628305	0.8267933	-0.1032897	-0.0432349	-0.6921745	-0.004087
cg20426571	TBX5;TBX5;TBX5;TBX5	blue	-0.4982627	-0.597393	-0.0903586	-0.0419739	0.39602074	-0.1365537
cg02548252	RN5S460	blue	0.45023308	0.86054065	-0.2699112	0.02018823	-0.5597646	-0.1146494
cg13471209	LPHN2;LPHN2;LPHN2;LPH	blue	0.54052712	0.77112655	-0.0593466	0.18082806	-0.3317324	-0.0106697
cg01054027	RP11-314A5.3;RP11-	blue	0.64511893	0.74817456	0.10017677	0.21427136	-0.3523363	0.2267795
cg01504616	AC091736.1	blue	0.36555421	0.52494803	-0.3839939	-0.0731316	-0.2720309	-0.0341327
cg26785896	SLC26A10;SLC26A10;SLC2	blue	-0.4608563	-0.6338884	0.01772715	0.00602969	0.49382348	-0.1919031
cg15536490	TFAP2C;TFAP2C	blue	-0.5582574	-0.6698669	0.14522553	-0.0235813	0.40987331	-0.1842563
cg05798318	HRAS;HRAS;HRAS;HRAS;	blue	-0.2846678	-0.3917627	0.10462502	0.03479093	0.33051118	-0.0137007
cg20417500	RP11-428C6.1	blue	-0.4361814	-0.5975267	0.33710563	0.00024691	0.3865652	-0.0122303
cg25309891	RP11-	blue	0.53723901	0.80106236	-0.0352788	0.02146019	-0.5180917	0.19047826
cg26265774	Y_RNA;DYTN	blue	0.57107952	0.89483034	0.12779687	0.12651202	-0.5720422	0.16873705
cg04895295	RP11-59D5__B.2;EYS	blue	0.55448055	0.71352239	-0.2656402	0.05648085	-0.243761	-0.0074163
cg14674139	LAMB4;LAMB4;LAMB4	blue	0.44781861	0.85920153	0.06323615	0.05256125	-0.6630554	0.08419894
cg25132979	7SK	blue	0.46319501	0.8818015	-0.0364866	0.05438344	-0.5947508	0.08273279
cg14001281	RGS6	blue	0.33077928	0.83441772	-0.1531578	-0.0138422	-0.5954854	-0.0622017
cg18068583	RP11-458K10.1	blue	0.38575498	0.84014226	-0.1756124	-0.0235245	-0.642749	0.00295611

cg18428707	SLC9A5;SLC9A5;SLC9A5;S	blue	-0.6259293	-0.7109966	0.03776383	0.05742414	0.51769425	-0.2296879
cg04876474	KCNQ1DN	blue	-0.5256705	-0.7322786	0.03256421	-0.0195666	0.54052202	-0.1649718
cg23771866	CNTN4;CNTN4;CNTN4;CN	blue	0.38800961	0.77101801	-0.3771483	-0.0322313	-0.4476053	-0.1793215
cg02325664	CYP19A1;CYP19A1;CYP19	blue	0.22894822	0.80811442	-0.0787876	-0.0826662	-0.7725209	-0.0960824
cg20036732	DEGS2;DEGS2;DEGS2	blue	-0.5156858	-0.7452145	0.20218888	0.00271266	0.52035263	-0.0134462
cg08703755	AC055758.1	blue	0.49575321	0.76493726	-0.2372674	0.1424984	-0.3577199	-0.0030152
cg12604192	KCNA10	blue	0.52884256	0.88323949	-0.0506649	0.09050353	-0.6136781	0.14013936
cg18158670	IGF1R;IGF1R;IGF1R;IGF1R	blue	0.67139556	0.73168842	0.37821611	0.16084731	-0.3814376	0.30226077
cg05321929	AC007392.4;AC007392.4;A	blue	0.32165775	0.82683368	-0.1290346	0.02618131	-0.6136534	-0.0651565
cg24965791	CLDN10	blue	0.53263516	0.83318131	0.00521695	0.19524848	-0.4830142	0.11221926
cg08605993	U66059.58	blue	0.43418488	0.89548394	0.05347038	0.02903041	-0.7009458	0.0763742
cg19541865	ACPP;ACPP;ACPP;ACPP;A	blue	0.62584262	0.81407423	0.23461794	0.20661057	-0.4373534	0.3032628
cg13262467	LRRIQ4	blue	0.38947185	0.90350229	-0.1032417	0.00168555	-0.7167662	0.02371834
cg07823535	LINC00446	blue	0.4006435	0.8256712	-0.1719925	-0.0153589	-0.6532382	-0.0153326
cg06491249	IGSF11;IGSF11;IGSF11;IGS	blue	-0.3590753	-0.4279467	0.14929839	0.09820104	0.3807011	-0.062732
cg04938317	TNXB	blue	0.71569572	0.71480044	0.0662635	0.25568277	-0.2463836	0.20372219
cg18502227	RBPMS;RBPMS;RBPMS;R	blue	0.47419005	0.83701471	-0.1629553	0.06987448	-0.48855	-0.0323236
cg21804802	TMEM40;TMEM40	blue	0.59285944	0.79346063	-0.0540931	0.14419224	-0.3980612	0.14829222
cg13703605	RN5S306	blue	0.33963857	0.84501294	-0.1693206	-0.0424312	-0.6472228	-0.1001799
cg06975035	RP11-317O24.2	blue	0.30383856	0.80040869	-0.1091706	-0.0454507	-0.6222393	-0.0832089
cg10090792	EPHA10;EPHA10;EPHA10;	blue	0.21286531	0.51649628	-0.0473228	-0.0151916	-0.3802788	-0.0159459
cg15194163	PRRT1;PRRT1;PRRT1;PRR	blue	-0.3344411	-0.5168677	0.28410491	0.09221667	0.37075927	0.07434454
cg04128358	SRGAP3;SRGAP3;SRGAP3	blue	0.60333824	0.72115572	-0.1341316	0.16021312	-0.2946974	0.04869196
cg23553442	TRPM3;TRPM3	blue	-0.4304662	-0.7151153	0.13579106	0.00076687	0.49859731	0.01507979
cg20475322	MIR125B1;RP11-	blue	-0.2975908	-0.6492205	0.20984577	0.03558937	0.50663039	0.00059232
cg02472818	COPG2;COPG2	blue	0.44007516	0.8641167	-0.0457547	-0.0329733	-0.6815903	0.08462084
cg00025526	CTD-2588J1.1	blue	0.40746516	0.80182169	-0.0918499	-0.0428722	-0.6747295	0.05106999
cg17744110	CALM3;CALM3	blue	0.56273865	0.56854559	0.21579515	0.2509815	-0.2573459	0.10562905
cg17175889	RP11-483B5.3	blue	0.47719879	0.70207262	-0.2046899	0.17358341	-0.2724239	0.07118788
cg07508910	RP11-379F12.4;RP11-	blue	-0.5660573	-0.8011838	-0.0551164	-0.0389439	0.55687474	-0.0703889
cg05619785	TNXB;TNXB;ATF6B;ATF6	blue	0.49421264	0.64655463	-0.0590797	0.15567756	-0.3208795	0.15110013
cg27289181	CPXM2;CPXM2;CPXM2	blue	0.20170539	0.71168388	-0.1610078	-0.0292842	-0.5447783	-0.1374834
cg10887021	PCDHB2	blue	-0.3789249	-0.6172818	0.27180808	0.06210467	0.53193249	0.04752729
cg17450733	RP11-478B9.1	blue	-0.2945477	-0.5040052	0.06087526	0.14751748	0.45096061	0.09242729
cg05079191	C7orf62	blue	0.42271525	0.50580259	0.09521234	0.05201814	-0.269221	0.09460863

cg20582941	ARPP21;ARPP21;ARPP21;	blue	0.58600618	0.87175694	-0.0536838	0.19199653	-0.5292223	0.05368721
cg16840156	ITGA11;ITGA11;ITGA11	blue	0.34901345	0.4050148	0.25635559	-0.0399195	-0.1695137	0.07813506
cg03606594	RP11-263K4.3	blue	0.50747002	0.80657426	-0.2264671	0.11208842	-0.3834699	-0.0331018
cg08433293	FANCI;FANCI;FANCI;FAN	blue	-0.2361506	-0.3371466	0.38681879	0.07403575	0.27604867	0.19715501
cg23273758	WBSCR28;WBSCR28	blue	0.37399292	0.76463875	-0.1142846	0.08404772	-0.518454	-0.0807339
cg27381412	SYPL2	blue	0.45829205	0.81418594	-0.0882259	0.10479866	-0.4830488	0.01113399
cg18856632	HDAC9;HDAC9	blue	-0.4775176	-0.8338376	0.0820573	-0.0331845	0.64046059	-0.0673522
cg18642719	COL4A2;COL4A2-AS2	blue	0.41006588	0.86089608	-0.1441181	0.02508715	-0.6076482	0.00065312
cg02495670	SCN1B	blue	-0.4311989	-0.459098	0.14680639	-0.0848974	0.29952039	-0.0686357
cg22381011	GHRHR;GHRHR	blue	0.33749389	0.70626827	-0.1739127	0.04842521	-0.4865115	0.02547084
cg19512268	GRM1;GRM1;GRM1;GRM1	blue	-0.1912784	-0.5081552	0.20088199	0.13437189	0.45179633	0.13008114
cg06722383	RP1-293L8.2;RP11-	blue	0.37295962	0.81123787	-0.1489787	0.04730477	-0.4779738	-0.0419204
cg12568898	PCDH8;PCDH8	blue	-0.4382816	-0.7480071	0.15804415	0.08715135	0.61113506	-0.0229829
cg08212784	C6orf165;C6orf165;C6orf165	blue	0.41271024	0.6682798	0.15216265	0.03951459	-0.4634164	-0.020256
cg02596153	NR2E1	blue	-0.4206853	-0.6481012	0.20611199	-0.0354802	0.43818412	0.00574316
cg05836145	SP8	blue	-0.3620975	-0.6837409	0.22374533	0.07400711	0.47236676	0.05242292
cg04836176	RP11-	blue	0.36607158	0.75159276	-0.0923561	0.13292656	-0.3930115	-0.1039488
cg04863713	ADAMTSL4;AL356356.1;A	blue	-0.4716639	-0.645741	0.14213699	0.03570954	0.51625022	-0.1454174
cg22775873	RP11-711C17.2	blue	0.4281861	0.77303578	0.00806757	0.06958599	-0.5199704	-0.064448
cg03178543	RP11-385J1.2;RP11-	blue	0.54734692	0.8181345	0.05413454	0.17641249	-0.4073074	0.08479945
cg19283459	NCAM1;NCAM1;NCAM1;	blue	0.63547603	0.75144593	-0.1754861	0.18236635	-0.2658662	0.09720176
cg05426918	BCAS1;BCAS1	blue	0.42074845	0.89422053	-0.1162297	-0.0414404	-0.7154609	0.04643397
cg04694437	CTC-454M9.1;CTC-	blue	-0.4275275	-0.8661364	0.12590427	0.0715686	0.66333815	-0.0116129
cg02228789	ST6GAL2-IT1	blue	0.39503108	0.78772039	-0.3290917	0.07248548	-0.5038512	-0.0794645
cg10817441	PSMB9;PSMB9;PSMB9;PS	blue	0.19568644	0.76820347	-0.1344795	-0.0851215	-0.7667137	-0.0623769
cg25077778	TRIM9;TRIM9;TRIM9;TRI	blue	-0.3845887	-0.8131906	0.0510466	0.0013783	0.67324017	-0.007822
cg07387591	PCSK2	blue	-0.3698013	-0.6509498	0.06206889	-0.001534	0.51169267	0.05888159
cg04669336	AC097532.2	blue	0.32886249	0.80522958	-0.0271601	0.04151546	-0.6091623	0.05989205
cg26695632	AC091321.1	blue	0.66754846	0.6129847	0.18764588	0.17090826	-0.1991647	0.30923296
cg19134874	RP11-204E9.1	blue	0.4618133	0.4850261	0.01119012	0.04008194	-0.2783277	0.1721264
cg23224120	SOCS2;SOCS2;SOCS2;SOC	blue	-0.6231503	-0.7003937	-0.0208556	-0.0914562	0.47765696	-0.2006805
cg08712303	RP5-1030M6.2	blue	0.19403249	0.34283095	-0.0549818	-0.0343125	-0.2197654	0.00134701
cg17073196	7SK	blue	0.57562868	0.67453594	0.02471877	0.16808339	-0.2905409	0.23132867
cg06240320	WDFY4;WDFY4	blue	0.29682388	0.79294618	-0.1042491	-0.04582	-0.689348	-0.1193627
cg16450893	RP11-268G13.1	blue	-0.3502198	-0.601643	0.13067796	0.08496545	0.5235146	0.1026374

cg14326956	FEZ1;FEZ1;FEZ1;FEZ1;FEZ	blue	0.66085405	0.70247763	0.42489008	0.16270867	-0.3851873	0.30172552
cg08564831	RIPK4;RIPK4;RIPK4;TMPR	blue	-0.4043568	-0.7295591	0.09153836	0.01820452	0.52139531	-0.0244292
cg18901644	GPR78;GPR78;GPR78;GPR	blue	-0.1607811	-0.4978731	0.25578732	0.06616664	0.42773495	0.25519393
cg12518166	CPEB1;CPEB1;CPEB1;RP11-	blue	-0.3990352	-0.4569136	0.28893858	0.02184427	0.30861643	-0.0026548
cg23542968	SFTA3;SFTA3;SFTA3;SFT	blue	-0.5762519	-0.7456706	0.03616761	-0.0077109	0.5008094	-0.1601343
cg11279021	ETV1;ETV1;ETV1;ETV1;ET	blue	-0.4622523	-0.747384	0.1681172	-0.044664	0.49736288	-0.0111344
cg26353448	OR2T4	blue	0.31722288	0.63128031	0.10930272	-0.0299855	-0.5286898	0.06534705
cg12459117	KRT36	blue	-0.3629278	-0.350248	0.19911282	0.16090936	0.29110191	-0.0978054
cg08288602	SLCO1C1;SLCO1C1;SLCO1	blue	0.69330053	0.69159911	0.28847101	0.20328253	-0.2115995	0.28427901
cg25992258	PRICKLE2;PRICKLE2	blue	-0.3100564	-0.4262487	0.0364296	0.01987614	0.3451644	-0.1053662
cg01517431	ABCA9;ABCA9	blue	0.47292068	0.87511295	0.04259653	0.03448738	-0.6221026	0.06431531
cg11725768	RP3-495K2.3	blue	0.41534959	0.83656701	-0.1878568	-0.0278362	-0.5576215	0.00844251
cg06285439	NRP2	blue	0.25567894	0.81248866	-0.0783226	-0.1028663	-0.7727275	-0.0284139
cg23353920	CTNNA2;CTNNA2;CTNNA	blue	0.40109206	0.80496392	-0.1307267	0.05975497	-0.5110475	-0.0078759
cg01329687	SNN	blue	-0.3345878	-0.602216	0.18036059	0.04105947	0.50219875	0.13142544
cg12333300	FAM5B;FAM5B	blue	-0.4996987	-0.8450996	0.17107474	-0.0747179	0.55987306	-0.055528
cg26679335	7SK	blue	0.44932848	0.7771313	-0.176041	0.11810069	-0.3945455	0.02204762
cg05638188	TRIM44	blue	-0.5446734	-0.7504973	-0.0325781	-0.0701332	0.50734769	-0.147469
cg26206396	RP11-966I7.1;RP11-	blue	-0.5162015	-0.75693	0.26141444	-0.0444406	0.42186688	-0.0539227
cg03375049	RP11-298D21.2;RP11-	blue	0.48026254	0.87091393	0.01332481	0.02348575	-0.6338147	0.08014726
cg20046330	STMN2;STMN2;STMN2;ST	blue	-0.4375066	-0.4441941	0.26596481	-0.0342082	0.19127863	-0.1577537
cg25424205	CPM;CPM;CPM;CPM	blue	0.40660718	0.86170403	-0.101098	-0.0291582	-0.6810921	0.0121673
cg27245980	DSCAM-IT1;TMPRSS3	blue	0.46502147	0.84624501	-0.0520495	0.04256272	-0.5690042	0.06988701
cg09083622	RP5-843L14.1	blue	0.30716486	0.53692347	-0.1654551	-0.0933877	-0.3371512	0.02730435
cg03870002	RP4-	blue	-0.4142269	-0.5601908	0.15490016	0.06970756	0.32815965	0.00419945
cg24445687	RP11-199O14.1	blue	0.54641769	0.77082169	-0.1407567	0.12268575	-0.4203672	0.12644473
cg21754296	RP11-10A14.7;RP11-	blue	0.39140112	0.86755738	-0.0877999	0.02803195	-0.6020883	-0.0376439
cg13628514	TRPV4;TRPV4;TRPV4	blue	-0.355715	-0.4489735	0.24565488	0.02574958	0.29539068	-0.07221
cg06550984	PTF1A	blue	-0.3323763	-0.6512786	0.27618597	0.01824144	0.54583468	0.13435638
cg19925399	snoU13	blue	0.51635596	0.69454136	0.13989034	0.16579724	-0.3577165	0.06321854
cg07721275	CTB-43E15.4;CTB-	blue	0.43301976	0.68576051	0.00284677	0.01027489	-0.4268603	0.04339367
cg03942839	RNU5B-4P	blue	0.26812088	0.77369244	0.18753447	-0.0878786	-0.8208194	-0.0153439
cg15598154	DDIT4L;DDIT4L;DDIT4L;R	blue	-0.4415946	-0.6842299	0.20776986	-0.0581763	0.41722503	0.01680371
cg27263998	IGF2;IGF2;INS-IGF2;INS-	blue	0.20803344	0.61831951	-0.0301873	-0.0506801	-0.4942356	0.03561688
cg09262269	LOX;LOX;LOX	blue	-0.3619102	-0.4588707	0.07197844	-0.0759941	0.31195081	-0.0616026

cg00931336	RP4-591L5.2	blue	0.47435547	0.78851381	0.13441692	0.0452509	-0.5945561	0.12372144
cg20910637	GNG8	blue	-0.4736306	-0.6641455	0.02260816	-0.0164697	0.47299554	-0.0875318
cg13959426	RP11-408H20.1	blue	0.2477256	0.81246438	-0.1442667	-0.1057052	-0.7242011	-0.0775827
cg02914379	MDFI;MDFI;MDFI;MDFI;	blue	-0.5788571	-0.6985999	0.06908924	-0.0544235	0.4491606	-0.1257102
cg21974766	CASD1;CASD1;CASD1;CA	blue	-0.4811081	-0.677672	0.09809222	0.06259476	0.55405797	-0.0317266
cg22090271	BOC;BOC;BOC	blue	0.4396958	0.8301719	0.11597577	0.06024644	-0.5753316	0.0562582
cg11298735	TBR1;TBR1	blue	-0.3915514	-0.4451099	0.29683462	0.09548021	0.31829059	-0.0339802
cg27350411	U6	blue	0.43048374	0.58404537	-0.1258123	0.10863735	-0.2706423	0.16304333
cg14550667	RP11-148B18.1;ARHGAP29	blue	-0.4398214	-0.5593577	0.22180429	0.03959496	0.43558407	-0.0450422
cg16767687	AC107057.1	blue	0.41800286	0.65415195	-0.1927072	0.03722472	-0.3408907	0.03008347
cg27190843	APBB1;APBB1;APBB1;AP	blue	-0.4289265	-0.5685092	0.1973939	0.05197399	0.4380335	-0.0200903
cg24699871	TRIM10	blue	0.57508302	0.84512463	-0.0932172	0.04069964	-0.494203	0.13209446
cg02780870	AC034205.1	blue	0.46910416	0.86160385	-0.1840886	0.07359556	-0.5466846	0.07178976
cg16408856	VWA3B;VWA3B;VWA3B;	blue	0.29485397	0.8150596	0.0339868	-0.0301663	-0.6849128	-0.091817
cg17775003	MYH7B	blue	0.45262107	0.87268904	-0.0918944	0.03493128	-0.653945	0.07843089
cg16405472	TMC1;TMC1	blue	0.34492629	0.45564735	-0.1664197	0.08687038	-0.1036451	-0.0488599
cg19717150	RP5-881L22.5	blue	0.28322113	0.62156248	-0.1290107	-0.0894299	-0.401426	-0.0163192
cg04711608	RP11-378I13.1	blue	0.5530444	0.77296161	3.62E-05	0.17787074	-0.3590313	0.20361128
cg03620729	FICD;FICD;FICD	blue	0.44573208	0.54837825	-0.0450924	0.17694092	-0.1856885	0.08890245
cg01089702	CHRNB3;CHRNB3	blue	0.72161532	0.70800236	0.17597897	0.22375139	-0.2689654	0.26159952
cg05524191	TMPRSS11E	blue	0.56968661	0.74113002	-0.2182068	0.16221718	-0.2745074	-0.0126884
cg03097013	TAF4;TAF4	blue	-0.3343557	-0.8046779	0.11470686	0.12080645	0.72093965	0.0051017
cg07614286	CTA-796E4.3	blue	0.45606691	0.86836416	-0.0926896	-0.002926	-0.565777	0.06402725
cg23444678	RP11-53B5.1	blue	0.37263033	0.81031212	0.25690099	0.0125339	-0.7228409	0.09754382
cg09369863	LINGO1;LINGO1;LINGO1;	blue	-0.7185863	-0.798816	-0.0724522	-0.1889574	0.42152731	-0.3485336
cg17277529	FGF3	blue	-0.306857	-0.6888549	0.17058227	-0.0137951	0.53926727	0.10328114
cg23075212	UMODL1;UMODL1;UMOD	blue	0.64947121	0.82686383	0.01840884	0.19158343	-0.3845403	0.32246067
cg24091995	RP11-77I22.3;RP11-	blue	-0.5444228	-0.6549272	0.06935709	-0.0585746	0.37361044	-0.2652537
cg24350628	GADL1;GADL1	blue	-0.4514192	-0.4972133	0.145831	-0.0390587	0.41234275	-0.1089881
cg19355078	MAST4-IT1	blue	0.6279784	0.8073009	-0.0358232	0.17868301	-0.4164395	0.24068025
cg13523557	ADCY1;ADCY1;ADCY1	blue	-0.3755576	-0.4226073	0.2705741	-0.0461954	0.26839234	-0.0579744
cg21104946	WNT7B;WNT7B	blue	-0.3906141	-0.3748431	0.24450201	0.03498345	0.05493098	0.01494716
cg07697234	RP11-342D11.3	blue	0.33014882	0.72228166	-0.1191628	-0.0143097	-0.5484484	0.00146361
cg13638371	snoU13	blue	0.21124103	0.51154461	0.03335959	-0.0714163	-0.310852	0.00137147
cg07386878	AP000593.5	blue	0.49354402	0.64980151	-0.2450429	0.04107383	-0.3840518	0.05826758

cg25728469	U4	blue	0.55022718	0.87436213	0.01232453	0.06823915	-0.5447302	0.13947864
cg03816680	AP001257.1	blue	0.27740746	0.75137605	0.14051001	-0.0906088	-0.7307517	-0.0307248
cg17200460	PTCD2;PTCD2;PTCD2;PTC	blue	0.30494995	0.77923462	-0.1355728	0.11539892	-0.5231175	-0.0823028
cg16827090	U4	blue	0.681914	0.72270574	-0.1448298	0.11289754	-0.3192659	0.25997848
cg08771861	SLC22A7;SLC22A7;SLC22	blue	0.42195347	0.85825059	0.07621917	0.00292348	-0.6786942	0.10904338
cg12285491	RP11-133F8.2;CTC-	blue	0.57944528	0.84566184	0.05898039	0.18175792	-0.4288734	0.12943668
cg09749643	PP2D1;PP2D1;PP2D1	blue	0.71188877	0.70661787	0.12938491	0.15359114	-0.2640364	0.21222824
cg18679830	U6	blue	0.64514322	0.80014756	0.09663384	0.25107668	-0.340663	0.22828696
cg16384849	AC115286.1;C1orf94;C1orf9	blue	-0.2962805	-0.5332822	0.19172272	0.03055141	0.4018266	0.09677986
cg01467397	MPDZ;MPDZ;MPDZ;MPDZ	blue	0.60949459	0.68878454	-0.2110792	0.11595675	-0.1816176	0.03068964
cg11737871	PRAMEF12	blue	0.34151899	0.70423338	0.04451236	-0.038721	-0.4703848	-0.0072826
cg03865600	PPFIBP2;PPFIBP2;PPFIBP2	blue	0.24915564	0.64221097	-0.1301958	-0.1067528	-0.5576221	-0.0169728
cg22975726	BX322559.3;BX322559.3	blue	0.48924748	0.61193702	0.06161406	0.14075212	-0.2694584	0.08549964
cg06330932	U6	blue	0.54761854	0.81301037	0.07902065	0.07222504	-0.5311224	0.30952106
cg14649874	SYNPR	blue	0.47830365	0.5354695	0.269386	0.17116956	-0.2551888	0.14546035
cg26061177	ADAMTS9-AS2;ADAMTS9-	blue	-0.4491433	-0.7644934	0.32438488	0.04346125	0.5266171	0.08111886
cg05482463	RTBDN;RTBDN	blue	-0.4290012	-0.5926192	0.08906983	0.07562297	0.49728185	-0.0985262
cg18389630	DNAJC16;DNAJC16;DNAJ	blue	0.51326022	0.78344687	0.25812175	0.04339288	-0.5840618	0.22500316
cg05009737	CDH18;CDH18;CDH18;CD	blue	0.63600659	0.73437958	0.00010095	0.23200699	-0.3110367	0.12815039
cg07633435	GRIA1;GRIA1;GRIA1;GRI	blue	0.39595469	0.88326416	-0.1533168	-0.0740299	-0.6687593	-0.043123
cg18794321	POU6F2-AS1;POU6F2	blue	0.53219281	0.73478697	0.02935556	0.0786167	-0.4179198	0.29865052
cg24983135	RP11-39H13.1	blue	0.25472597	0.82194569	-0.2049198	-0.0778226	-0.692963	-0.2087898
cg06246510	KB-1615E4.3;KB-1615E4.2	blue	0.20512002	0.78045358	-0.06866	-0.0876008	-0.7946763	-0.0488563
cg11727008	RP11-242P2.1;NRXN3	blue	0.56537114	0.76941913	-0.2230971	0.14022346	-0.282745	0.02552471
cg01296160	PDZRN3;PDZRN3;PDZRN3	blue	0.37638842	0.87251814	-0.1925651	0.00094577	-0.6624513	-0.0166263
cg13042319	U2	blue	0.40356409	0.78252384	-0.3159565	0.07052052	-0.4265304	-0.039268
cg23890086	RP11-704C2.1	blue	0.46559162	0.56766398	-0.2807353	0.08901689	-0.165335	0.04593362
cg24631409	TRIM9;TRIM9;TRIM9	blue	-0.4110371	-0.793046	0.03484564	0.06164695	0.7026195	-0.117046
cg04478095	TRNAI6	blue	-0.3520697	-0.4785992	0.26663086	-0.0364617	0.26480186	0.0086435
cg00533409	RNF26	blue	-0.3820542	-0.7291473	0.19516979	0.09989586	0.63056552	-0.017462
cg21084516	HSD17B12;HSD17B12;HSD	blue	-0.4572109	-0.6682975	0.17020584	-0.032308	0.51532405	-0.066557
cg08815081	MKX;MKX;MKX;RP11-	blue	-0.5206033	-0.5821758	0.2565103	-0.0859285	0.30057584	-0.1192423
cg20544848	ELAVL3	blue	-0.2307431	-0.4948819	0.39986968	0.12740172	0.3328251	0.16939363
cg05824026	RP13-612N21.1	blue	0.28310634	0.66371566	0.26377363	0.0056618	-0.5329939	0.07560931
cg08004742	PCDH1;PCDH1;PCDH1	blue	0.54982313	0.81374575	0.19182072	0.13437759	-0.5197226	0.24870939

cg08321129	EDNRB;EDNRB;EDNRB;E	blue	-0.3458308	-0.5216065	0.11214348	0.0011991	0.35873258	0.11419559
cg25499537	IGSF10	blue	0.52830677	0.82965354	0.08886721	0.1478604	-0.47888	0.19617211
cg18818210	C5orf46;C5orf46;C5orf46	blue	0.39757755	0.70217984	0.40045996	0.03966413	-0.6042483	0.19637183
cg04333495	PRPSAP2;PRPSAP2;PRPSA	blue	0.25675355	0.78087775	-0.0047552	-0.1721769	-0.836268	-0.0022667
cg08626831	AC090774.1;AC090774.1;A	blue	0.38463609	0.8753853	-0.0217519	0.01277701	-0.6787534	-0.0764931
cg16356224	USP2;USP2	blue	-0.3907169	-0.6239084	0.11196164	0.0113223	0.48476295	-0.1789251
cg05355157	C4BPB	blue	0.38129954	0.78512874	0.06535572	-0.0378278	-0.6849082	0.07535954
cg11966450	TNFRSF11A;TNFRSF11A	blue	-0.4591599	-0.5861446	0.06080623	0.01127254	0.37999326	-0.3226921
cg25583593	CDH2	blue	0.33630922	0.6225187	-0.3097628	0.07267217	-0.332098	-0.0939231
cg24457647	UBE2U;UBE2U	blue	0.29764106	0.8372615	-0.1401947	-0.0447943	-0.7354288	-0.033636
cg09444657	PDE4B;PDE4B;PDE4B	blue	-0.2042579	-0.6909246	0.18082556	0.04863372	0.63204422	0.17890426
cg01330548	SECISBP2;SECISBP2;SECI	blue	0.15051109	0.39738735	-0.2830849	-0.013855	-0.1763023	-0.2052964
cg13246242	ZNF707;ZNF707	blue	-0.1982559	-0.3815003	0.22280836	0.15851258	0.29433092	0.1177074
cg05221629	RNU6ATAC2P	blue	0.34605908	0.84809037	-0.1107428	0.02303598	-0.6165994	-0.0654457
cg03308494	AC025165.8;AC025165.8;SL	blue	-0.3708766	-0.6409676	0.11523292	0.12451139	0.59767409	-0.1102303
cg20682222	GRXCR1	blue	0.57338172	0.86854992	-0.0674161	0.18023102	-0.5271913	0.11437345
cg24201224	C19orf45;C19orf45	blue	0.43950098	0.53994337	-0.001884	0.05166702	-0.2814845	0.0950706
cg07924892	RP11-49I11.1;MOCOS;RP11-	blue	-0.5137949	-0.7777626	-0.0247349	-0.0251675	0.55151619	-0.1584741
cg07732827	RP11-110H10.2;RP11-	blue	0.44650423	0.8766155	-0.007626	0.00130795	-0.6816071	0.07221465
cg27284718	NXN;NXN;NXN;NXN	blue	0.39926329	0.78401936	-0.1504611	-0.1032656	-0.6947736	0.03650635
cg23000020	RP11-486A14.1	blue	0.24777365	0.8110518	-0.2520559	-0.0807017	-0.6863733	-0.1391924
cg09985192	AKAP6;AKAP6;AKAP6;A	blue	0.40579007	0.75525433	-0.2895449	0.09835241	-0.4457519	-0.0371994
cg11507438	RP11-374A22.1;NKAIN2	blue	0.54396932	0.87952901	-0.0029659	0.138186	-0.5666918	0.12122276
cg23986404	RP11-437L7.1	blue	0.39289468	0.74259672	0.21958575	0.05944984	-0.521669	0.07795607
cg07800110	RGS3	blue	0.42627341	0.88324017	-0.0489969	0.069308	-0.6365582	-0.0365752
cg26482862	ZNF396	blue	-0.3232927	-0.3719756	0.12536073	0.09262582	0.33892037	-0.0274984
cg25406755	CTA-445C9.14;CTA-	blue	-0.3108433	-0.4351246	0.23026886	-0.0355122	0.26823066	0.14176756
cg07251892	CCDC162P;snoU13	blue	0.36615838	0.86183869	-0.1908118	-0.0242685	-0.6773083	-0.0991258
cg24105314	NAGS;PYY	blue	-0.4439898	-0.4933357	0.01438199	-0.0241036	0.29558139	-0.0627434
cg23797553	RP11-397G17.1	blue	0.47100383	0.86101457	-0.1041044	0.02214861	-0.5518981	0.0046054
cg18508525	CD36;CD36;CD36;CD36	blue	0.36964629	0.79085563	-0.3005794	0.10646946	-0.507145	-0.0905026
cg05565022	DYSF;DYSF;DYSF;DYSF;	blue	0.48829183	0.87577156	-0.1618008	0.07987259	-0.5814248	0.07112568
cg20540746	VWCE;VWCE;VWCE	blue	0.52383344	0.70089677	0.16820504	0.11079324	-0.323559	0.14996546
cg25733268	AC073325.1	blue	0.26072707	0.67873847	-0.0328985	-0.0314036	-0.5618223	-0.0249752
cg18570872	RP11-71E19.2	blue	0.5102323	0.70077901	-0.0089957	0.16321862	-0.3173893	0.11030656

cg08934077	CDK14	blue	0.3802393	0.84590922	-0.0397666	-0.0213405	-0.6974913	0.01289445
cg12006072	FSIP2;FSIP2	blue	0.55957756	0.76716387	-0.233131	0.07304981	-0.3520338	-0.0426198
cg04194678	ACSS1;ACSS1	blue	0.36898112	0.55477434	0.14200761	0.05782239	-0.2441645	-0.0041861
cg18140293	SDC2;SDC2	blue	0.30325556	0.85684093	-0.18761	-0.063621	-0.7013151	-0.0716886
cg01661742	ZNF330;ZNF330;ZNF330;Z	blue	0.68198803	0.74091031	-0.0174194	0.20342523	-0.2502018	0.13906243
cg21826978	PSMB9;PSMB9;PSMB9;PS	blue	0.30416479	0.73909067	-0.1655129	-0.0414803	-0.6198735	-0.0713057
cg23608935	C1orf87;C1orf87	blue	0.28494315	0.4014269	0.25009569	0.035169	-0.2280011	0.05623629
cg17498811	U6;RHBDL2;RHBDL2;RHB	blue	0.67381605	0.76632247	0.12750972	0.27260586	-0.2772403	0.22882035
cg17165841	AC010731.4;AC010731.4	blue	-0.3622801	-0.7714868	0.19780726	-0.0053985	0.5742638	-0.0110457
cg08510985	RP11-	blue	0.49770001	0.67976415	-0.1879536	0.03538647	-0.3284973	0.04208854
cg03513986	AC108137.1	blue	0.19561289	0.75374496	-0.1659173	-0.1093276	-0.6433041	-0.1648241
cg15809488	RARB;RARB;RARB;RARB;	blue	-0.3865337	-0.7660516	0.07705858	0.04152905	0.68069917	-0.0908527
cg15321154	C12orf59;C12orf59;C12orf59	blue	0.64372709	0.68100413	-0.0663627	0.20606116	-0.1911271	0.19793969
cg17061244	ACSS3;LIN7A;LIN7A;LIN7	blue	-0.5216688	-0.8611592	-0.0430072	-0.046682	0.61504462	-0.1388505
cg18308783	Y_RNA;SH3PXD2A	blue	0.63418855	0.72387404	0.07302652	0.13487368	-0.2721264	0.16378698
cg08450404	ZNF552	blue	-0.4429503	-0.7178335	0.10319542	0.06583074	0.53235599	0.031675
cg25688502	C12orf55;C12orf55;C12orf55	blue	0.60830592	0.8195677	0.13963622	0.17099099	-0.4251432	0.1718343
cg04423214	RP11-203P23.1;RP11-	blue	0.65140873	0.72278494	-0.0111834	0.20921284	-0.2960254	0.23288955
cg25579806	NLGN1;NLGN1;NLGN1;NL	blue	0.39159034	0.7973998	-0.1671619	0.05365039	-0.5253068	-0.0069308
cg13508543	RP11-383H13.1	blue	0.33046276	0.79411646	-0.0947385	0.0229095	-0.5676826	-0.0390218
cg18433343	ELOVL3;RP11-242B12.5	blue	0.20498399	0.7732894	-0.1176847	-0.0255234	-0.7163203	-0.1357532
cg14340070	GPATCH1	blue	-0.4932897	-0.6263316	0.15677018	-0.0538675	0.47641017	-0.0707889
cg08980503	C11orf44	blue	0.40374059	0.85204029	-0.1421523	0.02182642	-0.5670971	-0.0175828
cg21997465	MYOG	blue	0.63334734	0.77463932	0.05330782	0.20162115	-0.3854884	0.26542553
cg20052865	SNORD114-	blue	0.3085352	0.66346044	0.02865066	0.01348273	-0.3979119	-0.0911001
cg26659636	C1orf137	blue	0.65679159	0.77209541	0.05672621	0.20541136	-0.3801333	0.23235929
cg02004851	PCDHA6;PCDHA6	blue	-0.2387837	-0.4166292	0.22613842	0.05939775	0.35106205	0.15419208
cg16522557	COL4A2-	blue	0.47159139	0.86652175	0.05703631	0.08058639	-0.5680147	0.06134758
cg16375880	RP11-307P5.2;RP11-307P5.1	blue	0.71058959	0.65572529	0.18040248	0.27796272	-0.1831612	0.30611778
cg08813679	ITGA11;ITGA11;ITGA11	blue	0.49231397	0.9132061	-0.052931	0.05296792	-0.6433854	0.01852091
cg04893119	PI15;PI15;RP11-	blue	0.53589359	0.69454699	0.00551228	0.20067936	-0.263507	0.00272229
cg03126817	AC005255.1	blue	0.44785015	0.5573565	-0.0904127	0.0341488	-0.2576014	0.01264385
cg23457386	SASH1	blue	0.58654141	0.80357722	-0.0150804	0.14008105	-0.4161406	0.06806867
cg06485774	FAM129A	blue	0.36475417	0.86884621	0.00029725	-0.0041362	-0.7567619	0.02423487
cg03099421	KCNIP1;KCNIP1;KCNIP1;	blue	-0.3692652	-0.4279465	0.24761951	0.12325489	0.35443616	0.02652008

cg26334672	VAX2	blue	-0.5155736	-0.7522833	0.08040535	-0.0361717	0.50934016	-0.1758719
cg13082666	CSN1S2AP	blue	0.55543265	0.68156404	0.08882027	0.19969406	-0.2518918	0.13891013
cg26171870	CTC-441N14.1;CTC-	blue	0.42818617	0.88508787	-0.1397742	-0.0052	-0.6032109	-0.1040053
cg19120580	ADCY6;ADCY6	blue	0.55820009	0.60211842	0.10944744	0.1217568	-0.2094615	0.21509569
cg21851187	U6;TANC1;TANC1	blue	0.53441701	0.58481132	-0.1280784	0.22780211	-0.2681799	0.1026731
cg16272007	FCRL6;FCRL6;FCRL6;FCR	blue	0.62486553	0.80969408	0.04012661	0.11119359	-0.4326797	0.20173562
cg07245160	FAM92A3	blue	0.71942289	0.79797769	0.03969177	0.24507913	-0.3067996	0.28269893
cg14158558	NRP2	blue	0.53641295	0.79967851	-0.1098519	0.11293807	-0.4722977	0.12418286
cg10257565	AP001615.9;TMPRSS3	blue	0.36846758	0.83578122	-0.0837787	0.02198849	-0.6883978	0.09493869
cg01394052	MAP3K6;MAP3K6;MAP3K	blue	0.6545222	0.70692889	0.02390602	0.13514866	-0.400337	0.25644506
cg17882660	ZIC2;ZIC2;ZIC2;ZIC2;ZIC2	blue	-0.4386641	-0.8079542	0.02197145	-0.0084864	0.57444404	-0.1049459
cg08902710	ZG16;AC009133.21	blue	0.52283168	0.81376286	-0.0946511	0.12870056	-0.5044416	0.09675832
cg05511503	AC104472.1;PLCH1	blue	-0.4148972	-0.7142673	0.04018105	0.007068	0.52961792	-0.0149806
cg01352108	KCNK4;RP11-	blue	-0.3479574	-0.8332077	0.16433047	0.13734828	0.73397441	0.13673273
cg11781976	RP11-361M10.5;RP11-	blue	-0.3296911	-0.4667147	0.2889916	0.05994029	0.38492751	-0.0080879
cg13268663	TBRG4;TBRG4;TBRG4;TBR	blue	-0.4382912	-0.5770186	-0.0027699	0.02235408	0.46741589	-0.2110649
cg03609427	SUN3;SUN3;SUN3	blue	0.37903076	0.8473488	-0.1303026	-0.0152185	-0.6529749	-0.0355759
cg16355465	RP5-1051D14.1	blue	0.47560505	0.79073609	0.10712055	0.06368684	-0.5386673	0.02982484
cg17813225	FETUB;FETUB	blue	0.54309397	0.771038	0.0169975	0.11163101	-0.4232779	0.12720195
cg01493237	RP11-	blue	0.53131735	0.74603899	-0.1150135	0.12785655	-0.4003691	0.14532866
cg25627044	PDGFA;PDGFA;PDGFA;A	blue	-0.5173324	-0.6755913	-0.1514045	-0.0601934	0.43925215	-0.1165633
cg11953311	ZBTB12;C2;C2;C2	blue	-0.2537839	-0.3632137	0.12705324	0.15131825	0.2987215	-0.0079016
cg13949073	RP11-70F11.8;RP11-	blue	0.35850235	0.8672664	-0.0714801	0.01004041	-0.6982583	0.06202151
cg17101595	EGLN3;EGLN3;EGLN3;EGL	blue	0.46026518	0.77994716	-0.0015833	0.06103996	-0.4812516	0.02978844
cg17726211	CTC-459M5.2;CTC-	blue	0.37973373	0.6858035	-0.3125013	0.05648993	-0.3018893	-0.0656295
cg21298174	ZNF709;ZNF709	blue	-0.2467928	-0.5765602	0.27597073	0.06618969	0.52570785	0.12038771
cg19217964	RP11-2K6.2;RP11-368L12.1	blue	0.37875868	0.81672281	-0.2636153	0.01963162	-0.5164695	-0.0560465
cg17492830	RP11-157E14.3;SPTLC3	blue	0.51902624	0.79619891	-0.2885579	0.04355577	-0.384803	-0.0499537
cg24504442	MEIS2;MEIS2;MEIS2;MEIS	blue	-0.2667106	-0.655578	0.3083608	0.06114625	0.4442691	0.16825614
cg00786408	SNTG2;SNTG2	blue	0.5467251	0.65398523	-0.209709	0.1553134	-0.173312	0.13407524
cg03099208	NXPH1;NXPH1;NXPH1;N	blue	-0.4843862	-0.770358	0.09163487	0.04689027	0.60167552	-0.0608462
cg21105204	AC007464.1;LINC00299;LIN	blue	0.63941129	0.65307399	0.20942574	0.15723192	-0.3518321	0.20527533
cg18142295	RAP1GAP;RAP1GAP	blue	0.3850036	0.84597557	0.02884351	-0.041054	-0.7460577	0.1524595
cg18236477	ATP8A2;ATP8A2;ATP8A2;	blue	-0.5257095	-0.7433013	0.2046602	-0.1181635	0.40544375	-0.0563156
cg06036912	CLDN6;CLDN6;CLDN6;TN	blue	-0.4210928	-0.7792263	0.01326796	0.04570316	0.69592906	-0.1900447

cg07037583	RP11-	blue	0.41222438	0.67947535	0.31647272	0.15099292	-0.464012	0.18379174
cg27545286	RP11-654D12.2	blue	0.30896968	0.8376461	-0.2103709	-0.0985415	-0.723379	-0.1378194
cg22223530	ST8SIA2;ST8SIA2	blue	0.27352042	0.72557661	0.06353339	0.01124327	-0.550458	0.00962426
cg08623787	RXRG;RXRG	blue	-0.3565773	-0.6930694	0.24612602	0.08794605	0.55182268	-0.0463533
cg03132556	HOXC11;HOXC11;HOTAIR	blue	-0.1290051	-0.3407501	-0.1147411	0.12700491	0.29958463	0.15151318
cg06960579	ADIG;ADIG;ADIG	blue	0.3183693	0.69481492	-0.091435	0.03467552	-0.4353481	-0.0248948
cg10441934	HPSE2;HPSE2	blue	-0.3961302	-0.4794946	0.21887914	0.07830774	0.28183427	0.02319541
cg18479981	RP11-386G21.2	blue	0.30716665	0.75174561	-0.1434851	-0.0110617	-0.4995411	-0.130807
cg08210688	OTX1;AC009501.4;AC00950	blue	-0.5263855	-0.5999225	0.25133801	-0.0708073	0.30524875	-0.0571899
cg20256220	LRRK1;RP11-	blue	0.7432073	0.80277161	0.16326782	0.24451031	-0.3328728	0.35302294
cg01196531	ONECUT1;ONECUT1;ONE	blue	-0.3409068	-0.4727371	0.37326925	0.03312647	0.31006828	0.08678143
cg22347748	AC093390.1;MYT1L	blue	0.64351624	0.73221108	0.14518808	0.21077253	-0.230372	0.26729989
cg00346557	RP5-1028L10.1;RP5-	blue	0.4096741	0.78621967	-0.0337497	0.02353724	-0.4310904	0.04870295
cg20210051	PDZRN4;PDZRN4;RP11-	blue	0.34601351	0.78957664	0.19476021	0.01155365	-0.6669725	0.05238818
cg10373728	U6	blue	0.47435137	0.76680971	0.20712212	0.02517226	-0.5523199	0.06968081
cg08729279	AC122136.1;AC122136.1;A	blue	0.63603161	0.6774956	-0.2248817	0.1851414	-0.2456146	0.24881904
cg12515774	RP11-143O10.1;RP11-	blue	0.47573637	0.81955382	-0.0670832	0.06473287	-0.4511996	-0.0435702
cg27123859	MIR587;RP1-60O19.1;RP1-	blue	-0.3104058	-0.4311925	0.24378151	0.10800671	0.36797748	0.04692246
cg07369590	HSPH1;HSPH1;HSPH1	blue	0.4603209	0.86797791	0.15861201	0.02580222	-0.7026812	0.11696416
cg24002167	CTD-2269F5.1	blue	-0.3697043	-0.5490583	0.17253944	0.09178865	0.42611013	0.05590161
cg26709720	B3GALT5;B3GALT5;AF064	blue	0.55731256	0.87038461	0.07014439	0.17186537	-0.5205006	0.17418746
cg07211875	ADCYAP1;ADCYAP1	blue	-0.401274	-0.4681648	0.06300578	-0.1263827	0.26469424	-0.0849257
cg00910067	SLC7A10	blue	-0.4821401	-0.7029812	0.16403347	0.00911667	0.53461944	-0.0072422
cg00485194	ZNF385D;ZNF385D;ZNF38	blue	-0.4455352	-0.4862018	0.20050307	0.01523383	0.24649205	-0.1374947
cg14518607	CTB-174D11.2;SLIT3	blue	0.47501117	0.8587504	-0.0344847	0.08619202	-0.5381699	0.00124282
cg23346658	KCNT1;KCNT1	blue	0.34327104	0.54202461	-0.046972	0.01159573	-0.346489	0.1734187
cg05344372	Y_RNA	blue	0.29859497	0.81032021	-0.0830781	0.00028829	-0.6279327	-0.0327966
cg20760085	BARX2	blue	0.55262574	0.80993836	-0.1578233	0.10100795	-0.4470785	0.05385689
cg14581803	FTCD	blue	0.49581892	0.82987162	0.03096539	0.04656616	-0.5214081	0.07317377
cg02492873	DNAJC27;DNAJC27;SNOR	blue	0.40289954	0.45722642	-0.1944606	0.06782223	-0.13635	0.00809212
cg19018248	AC024239.1	blue	0.42849692	0.69280524	-0.124355	0.06851004	-0.3671253	-0.0188483
cg11129772	GS1-179L18.1	blue	0.5045617	0.67059352	0.03457968	0.17186339	-0.2815824	0.17184123
cg22170183	GRHL1	blue	0.54604157	0.74002964	-0.0852128	0.17752927	-0.3579133	0.05412458
cg25995289	C19orf12;C19orf12;C19orf12	blue	0.51392929	0.61850959	0.05302435	0.28905184	-0.18165	0.09441972
cg27590407	OSBPL10;OSBPL10;OSBPL	blue	-0.2970888	-0.4980273	0.26781373	0.13395545	0.42036373	0.06008747

cg06969206	HHIPL1;HHIPL1	blue	-0.3573362	-0.4786471	0.02540871	-0.0081069	0.31459648	-0.0199424
cg18241351	SCGB1D4	blue	0.30537331	0.60629295	-0.0052517	0.05921334	-0.4017865	-0.1156537
cg14055835	FCAR;FCAR;FCAR;FCAR;	blue	0.22340529	0.64492031	0.13858118	-0.0296301	-0.5404099	-0.0491217
cg21914702	CTB-33O18.1	blue	0.5337198	0.53887228	0.25883598	0.15275803	-0.3038946	0.19863324
cg26680995	RP11-76I14.1;RP11-	blue	0.38334231	0.75779389	-0.3268957	-0.017477	-0.4720908	-0.0928701
cg04831870	C4orf32	blue	0.38232308	0.75199693	-0.060106	-0.0227689	-0.5990452	0.03753107
cg07688988	ACOXL;ACOXL;ACOXL;A	blue	-0.5378569	-0.6733692	0.13143982	-0.0529879	0.32203474	-0.1684468
cg14832023	C17orf105;DUSP3;DUSP3	blue	-0.3165158	-0.7146763	0.00201203	0.03348186	0.64536068	-0.0032623
cg09140851	SH2D4A;SH2D4A;SH2D4A	blue	0.57155574	0.64578739	-0.1629542	0.28234085	-0.2470202	0.1848348
cg14825736	FGF1;FGF1;FGF1	blue	0.3667889	0.81436615	0.14199679	0.04973477	-0.6417523	-0.0110984
cg14977480	CDHR3;CDHR3;CDHR3;CD	blue	0.47540109	0.73366827	-0.1050672	0.09135066	-0.3554477	0.10177663
cg07450275	RP11-254K3.1	blue	0.2472399	0.71529549	-0.2368085	-0.0705907	-0.6010005	0.00542936
cg15050865	CDH18;CDH18;CDH18;CD	blue	0.38305478	0.64507496	0.05662511	0.13210506	-0.3103051	0.04489263
cg16747973	FGG;FGG;FGG;FGG;FGG;	blue	0.73929165	0.7753564	-0.034718	0.19994908	-0.3209554	0.2274784
cg20800391	NRM;NRM;NRM;NRM;NR	blue	0.58758677	0.67720557	-0.1042833	0.04261633	-0.3746229	0.09726714
cg06503144	KDM4DL	blue	0.5589227	0.69050204	-0.031508	0.05853155	-0.3646618	0.00808451
cg16302310	TLR9	blue	-0.2429804	-0.4998277	0.34146206	0.11747748	0.45867175	0.19355142
cg26445167	AC068490.2	blue	0.39506293	0.85456311	-0.0828288	0.07948038	-0.5962508	-0.0276837
cg14812265	ANKRD7;ANKRD7;ANKR	blue	0.56952079	0.71926637	0.05788994	0.14910348	-0.2996975	0.10712359
cg21277447	AC118653.2;AC118653.2	blue	0.34841797	0.62338921	-0.0395655	0.0850633	-0.366103	-0.0089394
cg03519907	LARP1	blue	0.59523712	0.78166569	0.11595161	0.09423469	-0.4089893	0.18479932
cg23046682	TCTE1;TCTE1	blue	-0.3317642	-0.500535	-0.023623	0.06222387	0.42775519	-0.1248215
cg14941396	GPRIN2;GPRIN2	blue	0.56999434	0.66835504	0.00834317	0.19799793	-0.2490289	0.18525513
cg27562431	GCC1;ARF5;ARF5;ARF5;A	blue	-0.4072158	-0.6347193	0.32644251	0.0105383	0.42952778	0.01809493
cg01098474	ZNF716	blue	0.40467276	0.49679347	0.15171575	0.14591777	-0.1739425	0.12828555
cg26268085	OPALIN;OPALIN	blue	0.50778841	0.82084786	-0.0389354	0.1670792	-0.4027321	0.02992621
cg26381611	RP11-292D4.1	blue	0.35123875	0.79931267	0.1964484	0.02827488	-0.6085604	0.09474412
cg24484339	CA13;CA13;CA13;RP11-	blue	-0.3778437	-0.6159847	0.17915208	0.08827792	0.53661041	-0.0665648
cg02101523	OSR1;OSR1;OSR1	blue	-0.4255178	-0.6763833	0.2528235	-0.0409747	0.48419822	-0.085934
cg07865326	GNB2L1;GNB2L1;GNB2L1;	blue	0.43207037	0.49019597	-0.1984396	0.10625978	-0.2313191	-0.071718
cg21068873	RP11-561E1.1;RP11-	blue	0.5564459	0.71403852	-0.0795756	0.12525032	-0.2670072	-0.0415195
cg06041677	ISLR2;ISLR2;ISLR2;ISLR2;	blue	-0.4045266	-0.4796056	0.09418035	0.03285883	0.2954736	-0.1833307
cg20172287	AC016710.1	blue	0.31452039	0.70705055	-0.2944418	0.04566412	-0.4293096	-0.1214611
cg20640433	LAMA2;LAMA2;LAMA2;R	blue	-0.4350583	-0.7028544	0.2455151	0.03246043	0.56886676	-0.0231931
cg23879460	AC018495.2;AC018495.2	blue	0.64130863	0.72225324	0.20732962	0.15403369	-0.3636236	0.2862998

cg04405075	TMEM239;TMEM239	blue	0.63323739	0.75176457	0.03419838	0.2387308	-0.3668375	0.23232629
cg09275060	U4atac	blue	0.32524649	0.54193576	0.16985971	0.18452629	-0.2270365	0.08693952
cg16471974	AC060834.3	blue	0.51379544	0.84148955	0.051143	0.11711571	-0.5103321	0.04997516
cg14362105	RP5-827O9.1	blue	0.32676378	0.84391963	-0.0668185	-0.005695	-0.6796844	-0.0727688
cg14235731	RYR3;RYR3	blue	0.71153283	0.71413445	0.04599883	0.29839961	-0.2029102	0.23925144
cg13984563	SERPINA5;SERPINA5;SER	blue	-0.4569868	-0.4479507	0.08759485	0.00089903	0.17512992	-0.0301063
cg01656651	PRRX1;PRRX1;PRRX1;PRR	blue	0.37012121	0.65451387	-0.2844567	0.09627006	-0.2865706	-0.1062603
cg11781963	PIWIL2	blue	0.25641541	0.77812277	0.01570577	0.00468085	-0.7313486	-0.0897235
cg26145959	SPATS1;SPATS1;SPATS1;T	blue	-0.5050172	-0.654448	0.10926668	-0.0506846	0.4655505	-0.074778
cg03660710	TRABD;TRABD;TRABD;T	blue	-0.3834489	-0.7807105	0.08055888	0.06541499	0.64504686	0.04128179
cg13278496	ZIC4;ZIC4;ZIC4;ZIC4;ZIC4;	blue	-0.3849687	-0.581404	0.22697756	-0.0232998	0.30785116	0.08981673
cg00762081	STAU2	blue	0.62432516	0.62347751	0.10208372	0.21822116	-0.2011461	0.19742641
cg14545239	AC068286.1	blue	0.38896276	0.66274271	-0.1830668	0.07841497	-0.4075086	-0.0039801
cg06375409	C1orf159;C1orf159;C1orf159	blue	0.50315708	0.62645818	-0.0185767	0.22867989	-0.2032381	0.06969293
cg17968523	CPT1C;CPT1C;C19orf76;CP	blue	-0.22028	-0.4608947	0.21105281	0.07679162	0.39951157	0.16276021
cg07519926	IGLV3-16	blue	0.42758818	0.7091211	0.05793609	0.2686105	-0.3927367	0.11535243
cg22183537	RP11-129K20.2;RP11-	blue	0.41894291	0.74716343	-0.0004329	0.11065772	-0.417291	0.01323363
cg22418152	PLA2G5;PLA2G5;PLA2G5;	blue	0.40064404	0.51690446	-0.2900377	0.14617416	-0.1727716	-0.0485953
cg22407768	RP11-	blue	0.44605893	0.8918777	-0.0705337	0.02094644	-0.6474543	-0.0031333
cg00904576	RP11-380B22.1	blue	0.24799604	0.60293492	0.11295386	-0.0134833	-0.4401212	0.03043453
cg05707463	C1orf141;C1orf141;C1orf141	blue	0.53674159	0.63543391	-0.1233773	0.12744086	-0.1909174	0.13978305
cg01112778	PPP2R2B;PPP2R2B;PPP2R2	blue	0.40023364	0.58882539	-0.1534829	0.02477508	-0.3847421	-0.0074859
cg08810007	MEIS3;MEIS3;MEIS3;MEIS	blue	-0.4564519	-0.5137358	0.261355	0.0349711	0.31934213	0.08195238
cg13993576	RP13-379L11.2	blue	0.62406217	0.82249591	0.16563507	0.18004382	-0.414851	0.23299288
cg16191389	OBFC2A;OBFC2A;OBFC2	blue	0.26312484	0.50361151	-0.2270273	0.14095225	-0.3049398	-0.0916273
cg10500220	KIF5C;KIF5C;KIF5C;KIF5C	blue	0.61071397	0.72608041	0.05206881	0.09365704	-0.3501597	0.17324291
cg24426405	AGR2;AGR2;AGR2;AGR2;	blue	0.54060857	0.78419167	0.17028138	0.15900527	-0.4054786	0.12477189
cg07403294	ADAMTSL1;ADAMTSL1;A	blue	-0.4714709	-0.7090058	0.24516744	0.01853768	0.54459397	-0.0147217
cg24622988	LINC00359	blue	0.63317318	0.77731659	-0.103901	0.21261935	-0.3297935	0.15513592
cg06387829	OTX2;OTX2;OTX2;OTX2;O	blue	-0.345335	-0.6667027	0.29007068	-0.0013004	0.50424895	0.07578343
cg19099213	SPP2;SPP2;SPP2;SPP2	blue	0.62616745	0.69005697	-0.0370505	0.09425652	-0.3233115	0.14492518
cg15473755	IGLV4-3	blue	0.43962173	0.75773437	-0.2508101	0.1098932	-0.3711082	-0.0329695
cg18023666	CTB-43E15.3;CTB-	blue	0.55932426	0.77112384	0.15310627	0.16646538	-0.438239	0.23787575
cg15036529	PYHIN1;PYHIN1;PYHIN1;	blue	0.29579871	0.78905029	0.13737508	-0.098563	-0.8077014	0.05945319
cg27524731	SLC6A12;SLC6A12;SLC6A	blue	0.41320943	0.53223647	0.08247014	0.17705638	-0.1732636	-0.0700366

cg25313930	FAM124B;FAM124B;FAM1	blue	-0.2973877	-0.7677835	0.10719417	0.0594931	0.68430225	-0.0298454
cg27079502	U6;GRIP2;GRIP2;GRIP2;GR	blue	0.45751903	0.87400866	-0.1768521	0.04544932	-0.6171054	0.05691424
cg16073238	RP11-181B18.1;RP11-	blue	0.36325034	0.84057434	-0.0895961	-0.0337636	-0.685652	-0.0590121
cg16325502	CCDC140;CCDC140	blue	-0.509323	-0.8347986	0.03014123	0.00252808	0.65031794	-0.0855333
cg00110688	NUDT4;NUDT4;NUDT4;N	blue	-0.3268882	-0.4635733	0.23163125	0.11713065	0.37991345	0.08115243
cg12978308	NEIL1;NEIL1;NEIL1;NEIL1;	blue	-0.3113311	-0.8087283	0.12310075	0.08520322	0.73655974	0.02303055
cg19190068	ERBB3;ERBB3;ERBB3;ERB	blue	0.37392099	0.84379093	-0.1619637	0.02137872	-0.6157308	-0.0683061
cg24089340	U6;RUVBL1	blue	0.53984364	0.84753905	0.023884	0.14397944	-0.5380596	0.1152355
cg17378017	CRYBB2	blue	0.53618495	0.77216454	0.00629455	0.16464878	-0.4403899	0.2197778
cg03464804	PCDH15;PCDH15;PCDH15;	blue	0.31748818	0.50562032	0.24246139	-0.0101326	-0.34308	0.13409102
cg10329351	HSH2D;HSH2D;HSH2D	blue	-0.5043321	-0.5589127	0.11829637	-0.0675508	0.33569559	-0.1582236
cg06212823	VWA3B;VWA3B;VWA3B;	blue	-0.4570262	-0.7283182	0.08519425	-0.0206303	0.54960116	-0.1685476
cg08521381	SCN3A	blue	0.41955116	0.78864179	0.09110644	0.08487981	-0.4560165	0.04212202
cg12887021	CTC-276P9.1;CTC-	blue	-0.4630479	-0.5404562	0.15316923	0.02697577	0.36927779	0.04155478
cg12637437	AC113610.1;AC016682.1	blue	0.55273922	0.63159695	-0.0804128	0.30296987	-0.1682023	0.07253468
cg18806630	CHRD;CHRD;CHRD;CHRD	blue	0.32743102	0.81934784	0.08559933	-0.021056	-0.7450113	0.07115898
cg05685587	FOXQ1	blue	-0.5595665	-0.7181324	0.05239484	-0.0628076	0.46400186	-0.1140145
cg02524946	TBX20;TBX20	blue	-0.4875501	-0.7251176	0.23899196	0.01315665	0.49760706	-0.0677872
cg03478497	C18orf34;C18orf34;C18orf34	blue	-0.3539916	-0.4843862	0.26845623	-0.0402536	0.35633567	0.11208593
cg18085435	ATP8B1;ATP8B1	blue	0.4424139	0.83946794	-0.1728599	0.07626117	-0.5009088	-0.0167389
cg20992114	PITX2;PITX2	blue	-0.4970866	-0.8147034	0.10310696	-0.0616951	0.52864872	-0.0891979
cg25697152	TEAD4;TEAD4;TEAD4;TEA	blue	-0.4143309	-0.5635064	0.24235675	0.06265877	0.48432548	-0.0297178
cg13342710	MYLK-	blue	0.48028834	0.79042924	0.26434474	0.10726455	-0.5349258	0.17833502
cg15020109	RP11-17L5.4;RP11-	blue	-0.4719408	-0.7338595	0.11890409	-0.0661042	0.45422832	-0.0338305
cg02649818	ALDH4A1;ALDH4A1;ALD	blue	-0.4601332	-0.8006778	-0.0558923	-0.0546495	0.68871291	-0.1068349
cg10721503	LRRK1;LRRK1;LRRK1;LRR	blue	0.3314954	0.52961735	-0.0244656	0.0614599	-0.2593103	0.08207762
cg23572741	PHLDB1;PHLDB1;PHLDB1	blue	0.43216102	0.7711262	-0.0398041	0.09464204	-0.4438169	0.02747139
cg03925149	PDHA2	blue	0.5455514	0.7194324	-0.0392557	0.15586155	-0.3334036	0.1551493
cg23545906	GOT1L1;GOT1L1	blue	0.56446202	0.6121555	-0.0224001	0.09497309	-0.197786	0.09637999
cg00723842	SULT1C2P1	blue	0.61151959	0.85697174	-0.0054992	0.10000289	-0.5198841	0.2102005
cg07664173	REEP4;REEP4;REEP4;REEP	blue	-0.4409802	-0.7049732	0.31147924	-0.0280605	0.52931944	-0.0410075
cg23155043	PPME1;P4HA3;PPME1;PP	blue	0.47722766	0.60770026	-0.3991306	0.16908846	-0.1885872	0.10667694
cg07121250	CTB-164N12.1;CTB-	blue	0.64149583	0.81106367	-0.035907	0.18986234	-0.3527103	0.20109552
cg17894008	AC002994.1;NACA2	blue	0.58241943	0.69055105	-0.0195162	0.14866118	-0.3006351	0.09576631
cg09601830	7SK;7SK	blue	0.51672886	0.78042354	0.00420894	0.0544632	-0.454201	0.06297466

cg00866483	POU2AF1;POU2AF1;POU2	blue	0.5718222	0.8680891	-0.0386436	0.11687754	-0.475658	0.15625193
cg13723492	HAPLN2;HAPLN2;HAPLN	blue	-0.398634	-0.4403055	0.21907877	-0.0250055	0.33786236	-0.1535902
cg07696099	HPYR1	blue	0.52634916	0.6893826	0.33046945	0.17789972	-0.3373681	0.15046637
cg14182841	RP11-	blue	-0.5024807	-0.530083	-0.0670652	-0.0161765	0.3972568	-0.2748394
cg26623455	PPP1R42;PPP1R42;PPP1R4	blue	-0.4943271	-0.7134208	0.12266553	-0.0033776	0.57248723	-0.1517408
cg01909656	KRT73;KRT73	blue	-0.4451263	-0.5505591	0.06287576	-0.0796504	0.45051404	-0.245231
cg16082705	ANKRD44;ANKRD44;ANK	blue	-0.4765168	-0.5249218	0.12662366	-0.0586679	0.4059742	-0.0648003
cg21195468	LHX2;RP11-	blue	-0.4456832	-0.7322475	0.101917	8.18E-05	0.53715611	-0.100728
cg23387183	NEK5	blue	0.72372857	0.74426821	0.14786022	0.23268049	-0.2976759	0.25254616
cg21676242	SLC46A3;SLC46A3	blue	-0.4648727	-0.7147753	0.12102332	0.10947994	0.62498174	-0.1447562
cg00532610	CTD-2066L21.3;CTD-	blue	0.36200181	0.80693385	-0.187146	-0.0304326	-0.5677011	0.04514361
cg05570558	AC026202.5	blue	0.63029468	0.82876298	-0.1278842	0.1004276	-0.3989173	0.1572031
cg23927725	AP000688.14	blue	0.55540964	0.65734719	0.01535201	0.17559488	-0.255837	0.08178719
cg10432859	UGT1A7;UGT1A7	blue	0.61013752	0.77810494	0.32383795	0.16038599	-0.4078665	0.24511716
cg25723645	U4	blue	0.44242504	0.80751438	-0.1458101	0.00130387	-0.5204313	0.02248628
cg05070620	RP11-174G17	blue	0.56691202	0.84451923	-0.0193631	0.13817172	-0.4361988	0.1762406
cg22187726	RP11-3M1.1;ABCC11	blue	0.40990218	0.85828717	0.11117246	-0.0179146	-0.7266871	0.11368883
cg08452334	Y_RNA	blue	0.46680665	0.74707419	0.28532107	0.15520428	-0.3939716	0.13793942
cg17109514	STEAP4;AC003991.3;AC003	blue	0.31810415	0.86506767	-0.2127995	-0.0619973	-0.7227815	-0.0808604
cg20047612	C7orf10	blue	0.56653864	0.89713645	-0.0076306	0.08083438	-0.5801712	0.10206833
cg23641267	LPXN;LPXN;LPXN;LPXN	blue	0.47177441	0.63601944	-0.3016687	0.28415292	-0.2262489	-0.0837137
cg14698880	CTD-2515H24.3	blue	0.52949167	0.86160934	-0.0819469	0.16063321	-0.5104913	0.12054947
cg15081344	DLGAP3	blue	-0.3529234	-0.4234824	0.24435668	0.07393738	0.28208154	0.00260297
cg20707889	AC005042.4;AC005042.4;A	blue	0.42053783	0.90415914	-0.0891788	0.04105953	-0.7114005	0.02897454
cg18972972	SNORA26	blue	0.46858678	0.69113532	-0.06737	0.0079267	-0.3562132	-0.0146689
cg08529333	SERPINE2;SERPINE2;SERP	blue	-0.3678998	-0.7417183	0.12037788	0.07560891	0.66179238	-0.0046624
cg06893549	AL133247.2;SRD5A2;SRD5	blue	0.38745026	0.84965437	0.05957137	-0.0123693	-0.6013555	-0.0838169
cg13457222	SLC12A1;SLC12A1;SLC12	blue	0.48236133	0.78534291	-0.1885201	0.03023068	-0.4483686	0.04315089
cg27456468	AC017096.1;AC017096.1;A	blue	-0.4264334	-0.8205512	0.15009499	0.05810365	0.65459728	-0.0776244
cg18654305	ZNF284	blue	-0.3628097	-0.7101327	0.19014301	0.15043133	0.64107045	0.01743593
cg15008865	AC074019.1	blue	0.2710317	0.55927221	-0.1239192	-0.0028778	-0.3372634	-0.0090415
cg05170183	VOPP1;VOPP1;VOPP1;VO	blue	0.28828103	0.84394634	-0.0506269	-0.1410599	-0.8503213	-0.0378253
cg04951476	FAM50B;FAM50B;RP11-	blue	0.32838946	0.86771239	-0.1160702	-0.0877093	-0.7628491	0.00406755
cg27181554	MSRB1	blue	-0.5694655	-0.6275001	0.10037599	0.0032886	0.39828391	-0.2376448
cg10457539	NKX6-1	blue	-0.5109197	-0.7932515	0.12624703	-0.0114524	0.50160378	-0.0686215

cg25303653	CTB-49A3.5;CTB-	blue	0.41319939	0.70469946	0.06353652	0.03142027	-0.3852126	0.17525799
cg06553442	MAPK10;MAPK10;MAPK1	blue	0.58357563	0.8470138	0.13781442	0.19102151	-0.4825977	0.26131423
cg23515460	DPF3;DPF3	blue	-0.4302177	-0.7920619	0.03295611	-0.0305951	0.55476873	-0.0774894
cg02186769	GTF2H4;GTF2H4;VARS2;V	blue	-0.294834	-0.4755298	0.26605775	0.04803918	0.41456021	0.09128883
cg05569167	TRPM3;TRPM3;TRPM3;TR	blue	0.39349314	0.56337749	-0.2279342	0.19034626	-0.2637475	0.06458041
cg18132090	CELF3;CELF3;CELF3;CELF	blue	-0.49119	-0.7101522	-0.0143141	-0.0355449	0.47317483	-0.1504224
cg26905572	ANK3;ANK3;ANK3;ANK3;	blue	-0.3100618	-0.5931774	0.22760726	0.01379792	0.51001998	0.05135086
cg13558491	BCL9	blue	-0.5363227	-0.6034738	0.09748895	-0.0840141	0.38630696	-0.2248155
cg09883798	MSRA;MSRA;MSRA;MSR	blue	0.5451415	0.60325837	0.05183232	0.10971624	-0.2769158	0.219088
cg12821154	Y_RNA	blue	0.20339356	0.75352193	-0.1992909	-0.0832427	-0.582714	-0.3136054
cg22358580	MIR615;HOXC5;HOXC5;H	blue	-0.364417	-0.7429257	0.26364242	-0.0269668	0.50099058	0.10639009
cg12361295	TADA3;ARPC4;ARPC4;AR	blue	0.49922458	0.66547958	-0.1309303	0.08822639	-0.3637056	0.13816832
cg23563684	FAM5C;FAM5C;FAM5C;F	blue	-0.3729393	-0.7731753	0.25624888	0.0320337	0.57520574	0.05015651
cg13858909	FAM189A1	blue	0.4660096	0.84525922	-0.1443032	0.11649536	-0.5149063	-0.0183799
cg03664097	SNORA5;MYLK;MYLK;M	blue	0.29054896	0.67165239	-0.0745745	0.08186923	-0.3604641	-0.0471005
cg11445139	SLC5A1	blue	0.48684814	0.76625866	-0.0426948	0.07027307	-0.382374	0.14170738
cg24302416	SAMD12;SAMD12;SAMD1	blue	-0.4705612	-0.6710765	0.31066567	-0.0021118	0.44709501	-0.0306052
cg11365116	AC078941.1;AC078941.1;A	blue	0.565483	0.72437853	0.39886577	0.15216132	-0.3866634	0.21136811
cg23389023	F10;F10-AS1;F10	blue	-0.2342123	-0.3634634	0.13354287	0.09455034	0.310278	0.07692305
cg00366778	RP11-90J7.4;RP11-	blue	0.4572763	0.55156531	-0.1597268	0.16619238	-0.1901589	0.22050871
cg08330778	TBX3;TBX3;TBX3	blue	-0.4144292	-0.6432064	0.00583774	-0.0333591	0.43429561	-0.1307698
cg05034761	RP11-92C4.3	blue	0.51569639	0.88289898	-0.0508317	0.04773444	-0.5368235	0.11262821
cg18811269	RP11-460N16.1;RP11-	blue	0.47543356	0.81887846	-0.1082839	0.07048366	-0.506377	0.01510999
cg06704518	GATA4;GATA4;GATA4;G	blue	-0.4734323	-0.6689078	0.11102432	-0.0835091	0.39584937	-0.0586359
cg01304062	CTA-481E9.4;CTA-481E9.4	blue	0.32348931	0.72893903	0.03585123	0.09761518	-0.4342621	-0.0911615
cg00170295	RP11-268F1.2	blue	0.38038827	0.85874058	0.00220219	0.05315803	-0.6921304	0.01627102
cg13773631	BARX1	blue	-0.4865661	-0.5666174	0.2329065	0.00378834	0.34650155	-0.0238466
cg26467952	PRUNE2;PRUNE2;PRUNE2	blue	0.32206875	0.81075829	-0.0091435	0.05634207	-0.6198563	-0.0224867
cg03764381	GRIN2A;GRIN2A;GRIN2A;	blue	-0.5130033	-0.660107	-0.1269536	-0.0191132	0.33864652	-0.0938014
cg20124188	FAM64A;FAM64A;FAM64	blue	-0.3806588	-0.4041983	0.1910754	-0.0201205	0.33041081	-0.0136055
cg14645095	CTD-2507G9.1;ANO3	blue	0.33587751	0.67954392	0.03569244	0.13569888	-0.4145955	-0.074864
cg19558029	KATNAL2	blue	-0.3025556	-0.5954003	0.10232095	-0.0071978	0.43144759	0.04269427
cg27560864	SCN4B;SCN4B	blue	-0.3827838	-0.6355654	0.13469001	-0.0688379	0.48923957	-0.0673481
cg01296982	RP11-	blue	-0.3298382	-0.4466063	0.12480734	-0.0093188	0.33548144	-0.0123824
cg13740815	SHANK1;SYT3	blue	-0.321712	-0.5819094	0.24145322	0.14267933	0.53023268	0.05996472

cg24064300	KB-1930G5.3	blue	0.4837164	0.8764125	-0.09573	0.05637006	-0.5899765	0.09906975
cg02387561	RN5S71;SHCBP1L;SHCBP1	blue	0.19998183	0.62647409	0.23078514	0.00311741	-0.5021363	-0.0419971
cg19891156	SREBF1;SREBF1;SREBF1;S	blue	0.5040825	0.64593004	-0.0656048	0.1718254	-0.2532727	0.15568622
cg15110396	Y_RNA	blue	0.48476922	0.83999742	-0.0150775	0.04543538	-0.5362801	0.21048037
cg00471000	BPIFB6	blue	0.37889486	0.7192414	0.04416752	0.09919315	-0.393175	0.00366434
cg26658341	SATB2;SATB2;SATB2;AC0	blue	-0.3954475	-0.730109	0.2083254	0.0406328	0.53438821	-0.0173949
cg21161682	ATP10B;CTC-	blue	0.44165938	0.90070294	-0.0822112	0.05226517	-0.6414094	0.02977693
cg04707408	RP1-170O19.14	blue	-0.3557901	-0.7356071	0.141144	0.07446346	0.58709328	-0.0063045
cg00129389	AC005493.1;AAA1;AAA1;A	blue	0.35689788	0.63636982	-0.2176342	0.1003729	-0.3188977	-0.073549
cg19697351	INTS1	blue	-0.4385112	-0.7711	0.09937567	-0.0375194	0.53616388	-0.0695542
cg12592276	RP5-1063M23.2;RP5-	blue	0.28951794	0.6875928	-0.2026222	-0.0180552	-0.5755092	-0.0470381
cg20827489	Y_RNA	blue	0.59725897	0.7159156	-0.1179238	0.10090309	-0.2753244	0.1197483
cg18157838	MBL1P;MBL1P	blue	0.41537562	0.69522891	-0.1142103	0.04049686	-0.4931734	0.05090661
cg04684481	OFCC1;OFCC1;OFCC1;OF	blue	0.58948371	0.73415994	0.25483872	0.25083828	-0.3751404	0.16948551
cg05271406	RP4-591L5.2	blue	0.44421311	0.55115239	0.04604358	0.23203664	-0.1867585	0.28893301
cg19430586	TTN;TTN;TTN;TTN;TTN;TT	blue	0.75783261	0.75061904	-0.0251535	0.22648824	-0.1982975	0.31330893
cg15364874	TSPEAR-AS1	blue	0.70518403	0.7814284	-0.0266756	0.20813525	-0.3116545	0.21969075
cg18839255	SDC3;SDC3	blue	0.37763165	0.42619237	0.03349199	-0.0059061	-0.1826903	0.14221931
cg00450003	CTD-3046C4.1;CTD-	blue	0.691602	0.74510234	0.05100673	0.26519671	-0.2472947	0.26435912
cg25453381	RNF152	blue	-0.4736832	-0.8221977	0.0976694	-0.0711208	0.59991192	-0.0634136
cg24115032	RP5-	blue	-0.4393504	-0.7442677	0.25322751	-0.0044328	0.55690903	0.01353331
cg00717494	RN5S103	blue	0.22528848	0.49708461	-0.2444492	0.04094893	-0.2621605	-0.0082746
cg12825324	MYLK;MYLK;MYLK;MYL	blue	0.34501167	0.85055405	0.04699401	0.00106715	-0.6288931	0.0130551
cg21811994	RP11-557J10.5	blue	0.39360947	0.83389594	0.11901098	0.01146256	-0.655157	0.06897555
cg17880049	RP1-74B13.2	blue	0.49808106	0.83656955	0.20256672	0.05552354	-0.5508472	0.15943039
cg21285383	SPIRE2;SPIRE2;SPIRE2;SPI	blue	-0.2984285	-0.6687558	-0.0138121	0.06881959	0.5688719	-0.019363
cg02163033	RCAN2;RCAN2;RCAN2	blue	0.57456748	0.66325106	0.34149148	0.18635713	-0.3437941	0.12462691
cg16002100	PID1;PID1;PID1;PID1	blue	0.50279303	0.58222156	0.01397998	0.27564822	-0.1678195	0.18101559
cg11365072	KIF4B	blue	0.34609154	0.71448995	0.03399546	0.0471881	-0.4884605	-0.0397893
cg02810927	RP1-212P9.2	blue	0.33535171	0.7862644	0.05071338	0.06651123	-0.5683988	-0.0639852
cg09876253	Y_RNA;RP11-260E18.1	blue	0.36355573	0.58319812	-0.0327254	0.00842649	-0.3208519	0.05276871
cg07734442	RP11-575I8.1	blue	0.53338357	0.78622382	-0.0625039	0.19899234	-0.3781415	0.11214958
cg03388670	SNORA40;SVOPL;SVOPL;	blue	-0.2063736	-0.776003	0.10721537	0.15378623	0.81833293	0.14611355
cg16780480	GSTM3;GSTM3;RP4-	blue	0.3517458	0.46682482	-0.2006309	0.1210582	-0.1802354	0.00895538
cg15353612	SGCB;SGCB;SGCB	blue	-0.4442994	-0.5509698	0.10534934	0.1226505	0.39671761	-0.0375562

cg03515999	RP11-27I1.2;PSMD5;RP11-	blue	0.15950856	0.40147241	-0.0946293	-0.0859586	-0.3508378	0.06965011
cg14750274	RP4-586O15.1	blue	0.28939001	0.79720261	-0.1711886	-0.0487214	-0.6823802	-0.0771812
cg26510009	HSPA12A	blue	0.36211904	0.54714667	-0.1681426	-0.0259703	-0.3691057	0.08687017
cg07231563	STXBP2;STXBP2;STXBP2	blue	-0.4738971	-0.6041193	0.0651211	-0.0398567	0.51494158	-0.1075497
cg11318790	NRXN3;NRXN3;NRXN3	blue	0.21834715	0.68253141	-0.2053857	0.10822052	-0.4627512	-0.1232159
cg02153930	AC004870.4;AC004870.4;A	blue	0.41567126	0.78064218	-0.1917056	0.09922297	-0.4296384	0.01001413
cg08954634	U2	blue	0.36237959	0.62936915	0.47613206	0.01224757	-0.5234179	0.1784552
cg23029159	MIR154	blue	0.49953573	0.85709046	-0.0953497	0.05150293	-0.6010262	0.04197879
cg05863174	GRK4	blue	0.24453771	0.82009392	-0.0055623	-0.1015208	-0.8117454	-0.0064045
cg18641151	ARHGEF10L	blue	-0.4581843	-0.7544572	0.05780736	-0.0590005	0.51051691	0.00292563
cg13199589	RIMKLA;RIMKLA	blue	-0.4386881	-0.7146899	-0.0879022	0.05663614	0.63284389	-0.0665815
cg01455828	RP11-503N18.2;RP11-	blue	0.33542283	0.46503194	0.14064624	0.12691968	-0.194969	0.07099087
cg23936463	FAM19A5;FAM19A5	blue	-0.4675998	-0.5209349	0.22330037	0.0047124	0.32488838	-0.0789188
cg22661942	TRPV6;TRPV6;TRPV6;TRP	blue	0.43874032	0.84295936	-0.0793633	0.08447349	-0.4854585	0.0003043
cg14905657	LMO3;LMO3;LMO3;LMO3;	blue	-0.3444963	-0.411364	0.18871183	-0.0394842	0.28317597	0.00505166
cg18829674	C21orf88;C21orf88;C21orf88	blue	-0.4312978	-0.825841	0.10136032	-0.0296784	0.64545573	-0.0768958
cg10095577	LHX8	blue	-0.4752874	-0.6520749	0.1865839	-0.1153851	0.40319521	-0.1517804
cg12067468	ITGA3;ITGA3;ITGA3;ITGA	blue	0.44948607	0.69258498	-0.0776074	0.06959909	-0.3632437	-0.0158283
cg17214860	LRRC32;LRRC32;LRRC32	blue	0.38833179	0.37410121	0.06740341	0.07995219	-0.1678455	0.12514704
cg12502158	AC003988.1	blue	0.64187897	0.70733384	-0.0760728	0.23269726	-0.1966671	0.19513602
cg17196961	AC006322.1	blue	0.34437748	0.47071422	-0.1314462	-0.0071327	-0.2365784	-0.0426461
cg12611801	Y_RNA	blue	0.35246402	0.83506162	-0.2528251	-0.0355562	-0.610588	-0.1113172
cg03079497	MYO1C;MYO1C;MYO1C;	blue	-0.3425196	-0.6791029	-0.0175791	0.06410438	0.57880013	-0.0439018
cg05881163	TPO	blue	0.49843039	0.63604251	-0.2426931	0.04095309	-0.2692518	0.02486783
cg26599969	AL356214.1	blue	0.32228109	0.78824666	-0.20563	0.06528834	-0.5351905	-0.1222749
cg11801107	EPHA8;EPHA8	blue	-0.4860881	-0.6042008	0.15069838	-0.0333861	0.32967659	-0.1024257
cg23038791	PTHLH;PTHLH;PTHLH;PT	blue	0.41078711	0.64605823	-0.3125809	0.01562493	-0.3521994	-0.0529401
cg17389519	PTF1A	blue	-0.388034	-0.5244197	0.24320612	0.0622546	0.39089242	-0.0156169
cg15301904	SLN;SLN	blue	0.51297998	0.79828387	-0.0881537	0.09085461	-0.4366248	0.09682264
cg26182628	AP006748.1;TMPRSS3	blue	0.2470519	0.47135197	0.07447851	0.08504795	-0.2885085	0.04434278
cg15705086	MLPH;MLPH;MLPH;MLPH	blue	0.51104965	0.82779329	-0.0142208	0.1383403	-0.4404719	-0.0428393
cg13614060	RP11-451G4.1;RP11-	blue	0.44173717	0.76326643	-0.0982769	0.07222452	-0.3914816	0.02319481
cg04767897	BEGAIN;CTD-2644I21.1	blue	-0.1075818	-0.3462417	0.19669725	0.08944118	0.3434114	0.11075045
cg14174204	OTX1;AC009501.4;AC00950	blue	-0.5614962	-0.6436086	0.28296233	-0.0205821	0.32485346	-0.1150438
cg19501024	ACSM2B;ACSM2B;ACSM2	blue	0.40638604	0.65850752	-0.0530532	0.08008542	-0.3901501	0.04112145

cg17023292	CTD-2029E14.1	blue	0.47683991	0.52124927	0.23544255	0.11943369	-0.243072	0.09567398
cg23234607	TSHR;TSHR;TSHR;TSHR;T	blue	-0.3819849	-0.455029	0.19600388	0.05136495	0.31858464	0.03626393
cg14217584	KCNIP3;KCNIP3;KCNIP3;	blue	-0.5477839	-0.6698231	-0.0973554	-0.0611091	0.50051596	-0.2532637
cg02232320	RP11-345M22.2;RP11-	blue	-0.5616157	-0.7411395	-0.0215704	-0.1359557	0.39862593	-0.1679135
cg18207384	U6;CGNL1;CGNL1;CGNL1	blue	0.41743489	0.85749934	-0.192628	0.04492293	-0.5903061	-0.0780473
cg11227817	SPATA19;SPATA19	blue	0.57791665	0.67898669	-0.1588236	0.18692087	-0.1819767	0.00322146
cg20620498	U1	blue	0.5635816	0.75545336	-0.0585436	0.16030522	-0.3618981	0.15948941
cg11183001	TTC23L;TTC23L;TTC23L;TT	blue	-0.459745	-0.5183595	0.08841011	0.00934101	0.37976462	-0.1337785
cg20648672	FRMPD2;FRMPD2	blue	0.22479913	0.7952587	0.06735932	-0.0227926	-0.6957003	-0.0091715
cg22942804	HIST1H2BE	blue	-0.4227681	-0.4220981	0.20364731	-0.1429899	0.16087544	-0.0626344
cg10146203	GABRA5;GABRA5;GABR	blue	-0.2979896	-0.480616	0.27936614	0.02917263	0.32677639	0.15144876
cg11579681	CTC-459M5.2;CTC-	blue	0.31806332	0.78664844	-0.2606917	0.00405838	-0.564127	-0.052407
cg20724184	EFHA1;EFHA1;EFHA1;EFH	blue	0.43738806	0.61048472	0.13728688	0.05091305	-0.470521	0.18781353
cg20516845	UBASH3A;UBASH3A;UBA	blue	0.26057729	0.77599581	-0.0022459	-0.075457	-0.8152253	-0.001984
cg18447815	RP11-1060J15.7;RP11-	blue	0.47864139	0.65723537	-0.0915759	0.16561608	-0.2979936	-0.0068014
cg02344539	RP11-	blue	-0.4861063	-0.7293219	0.00682018	0.00500408	0.51861442	-0.0303545
cg00457367	U1;FILIP1;FILIP1	blue	0.31788366	0.5573566	-0.1998272	0.03055805	-0.3887798	0.01058719
cg19829787	RP11-449J1.1	blue	0.59777325	0.82188438	-0.0223198	0.17531912	-0.356138	0.12935301
cg12080253	CTD-2298J14.2	blue	-0.434481	-0.6756018	0.34192905	0.00418715	0.46660452	0.09697988
cg00260012	CAPN13;CAPN13;CAPN13;	blue	0.42071381	0.77646297	-0.1426375	0.10076018	-0.4881216	0.03476502
cg09609418	RP11-867G23.13	blue	-0.418677	-0.7329078	0.16366935	0.08399665	0.53198346	0.02605026
cg02839123	FAM83F;FAM83F	blue	-0.3955192	-0.6751247	0.2482201	0.04573942	0.44409344	-0.0579796
cg10074775	ZFHX4;RP11-65D13.1;RP11-	blue	-0.3402898	-0.6276992	0.15485408	0.00264021	0.42029166	0.06080875
cg05064673	PTPRU;PTPRU;PTPRU;PTP	blue	-0.4239148	-0.4061406	0.12840871	-0.0885861	0.23268243	-0.1673726
cg12934461	TTLL13;RP11-697E2.6	blue	-0.4471145	-0.509536	0.07461531	-0.1133873	0.22540562	-0.2022533
cg05083647	NFASC;NFASC;NFASC;NF	blue	0.25001817	0.39735586	-0.2604285	-0.0001333	-0.2163071	-0.0477879
cg19411060	RP11-3P22.2;RP11-3P22.2	blue	0.17552224	0.61223625	-0.2086911	-0.0812412	-0.4831429	-0.102017
cg13974394	IRX4;IRX4	blue	-0.3656649	-0.5025565	0.12077429	-0.0653312	0.28708434	0.00074066
cg09548995	RP11-91P17.1	blue	0.64376636	0.64172252	0.26163333	0.21006431	-0.2203006	0.28918119
cg01829277	ANO4;ANO4;ANO4;ANO4;	blue	-0.3041556	-0.6000551	0.30515663	0.0539821	0.50111653	0.09440356
cg16478236	MAP1B;MAP1B;MAP1B	blue	-0.5801893	-0.68625	0.04734487	-0.1716847	0.43038919	-0.2791433
cg15584788	RNF207;RNF207;RNF207;R	blue	-0.4596992	-0.53967	-0.0594549	0.02525839	0.36499271	-0.1517499
cg03934905	TBR1;TBR1;TBR1;TBR1;TB	blue	-0.4498411	-0.6856251	0.38121539	0.00436096	0.41670457	0.03619633
cg12731397	PCDH18;PCDH18;PCDH18;	blue	0.37961392	0.58689008	-0.0258379	0.02402958	-0.2908591	0.02562194
cg14455494	CGN;SNORA44	blue	0.46746471	0.66786176	0.44291986	0.13138502	-0.4454142	0.19966695

cg18142906	WDR49;WDR49	blue	0.44162312	0.87485427	0.05159721	0.03717259	-0.638807	0.02954579
cg14988425	FKBP1A;FKBP1A;FKBP1A	blue	0.55785208	0.75161683	-0.079565	-0.0041531	-0.4754238	0.15545122
cg07131336	ECE2;ECE2;ECE2;ECE2	blue	0.53116402	0.65073615	0.25271642	0.20046276	-0.2282199	0.13391644
cg03513978	CDH2;CDH2;CDH2	blue	0.29547861	0.57269257	0.07504097	0.07948398	-0.4100059	0.09164778
cg17454318	ADRA1A;ADRA1A;ADRA	blue	0.14335345	0.32518748	0.01625714	-0.123691	-0.2762724	-0.0495901
cg27626102	STEAP2-	blue	-0.4304465	-0.5975678	0.17750685	0.07030519	0.39103363	0.02058542
cg00930628	FBLN7;FBLN7;FBLN7	blue	-0.3098969	-0.7532289	0.0611621	0.02861383	0.65481577	0.00678226
cg11606412	MYO5B;MYO5B	blue	0.40253264	0.69318118	-0.1973958	0.13208964	-0.2979362	-0.0297021
cg11134999	WFIKKN2;WFIKKN2	blue	0.4186348	0.54987105	-0.0588008	0.03138644	-0.2008269	0.02505218
cg10558494	BDNF;BDNF;BDNF;BDNF;	blue	-0.487908	-0.681584	0.23218783	-0.0690205	0.38117839	-0.1281617
cg17219432	COL1A1	blue	-0.449941	-0.5025984	0.23330182	0.1347893	0.37821127	-0.0646156
cg03388193	HPSE2;HPSE2	blue	-0.4090321	-0.6281005	0.19360882	0.02860281	0.44209505	-0.0181217
cg00284152	RP5-1033H22.2;RP5-	blue	-0.3171551	-0.8026499	-0.0071965	0.06922645	0.81885151	-0.0374629
cg11575789	GBA3;GBA3	blue	0.56817904	0.72277016	-0.1864317	0.1875055	-0.2443919	0.00033534
cg10224090	PYROXD2	blue	0.46472537	0.59456348	-0.0560359	0.06829245	-0.291563	0.15917399
cg22415472	SLC5A7;SLC5A7;SLC5A7	blue	0.56388927	0.6942634	-0.1252196	0.16071442	-0.2176889	0.08201369
cg15909056	TSPAN8;LGR5	blue	0.32360046	0.7364884	0.35689282	0.02053602	-0.6438786	0.14138515
cg19447020	AC114808.3	blue	0.2673963	0.51925221	0.15253736	0.01948905	-0.3066302	-0.0267072
cg02158755	BTNL2;BTNL2;BTNL2	blue	0.28083099	0.50006849	-0.0835376	0.1006668	-0.2625788	-0.2322853
cg16884979	7SK	blue	0.61834729	0.82346998	0.03003462	0.11532916	-0.4061224	0.15717222
cg14885137	AC091969.1;AC091969.1;A	blue	0.51238456	0.67161235	0.12445842	0.16885632	-0.2725673	0.07671566
cg27153146	MYCBPAP;MYCBPAP;MY	blue	0.63402192	0.71381475	0.07551786	0.0881988	-0.3790079	0.33121347
cg01951477	SLA;TG;TG;TG	blue	0.44633782	0.74265801	0.10442749	0.10409921	-0.4696388	0.06796641
cg13853159	TBR1;TBR1;TBR1;TBR1;TB	blue	-0.3640963	-0.4468993	0.27169631	-0.0174723	0.24694972	0.04836317
cg19758087	CTD-	blue	-0.426767	-0.6348681	0.09754813	-0.0270321	0.37108403	-0.0050904
cg17166123	RP11-79P5.7	blue	-0.3891318	-0.8569101	0.19654113	0.08954315	0.7455302	0.02338471
cg13939792	SIX6;C14orf39	blue	-0.5063228	-0.7231639	0.07585713	-0.0856315	0.39521097	-0.083844
cg05859477	BACE2;BACE2;BACE2;BA	blue	0.37406426	0.88547651	-0.1594578	-0.0433415	-0.6613819	-0.023774
cg18100045	AC034187.2;AC087859.1	blue	0.53076423	0.70108128	-0.1396386	0.13273648	-0.2501235	0.02250119
cg20326908	RP11-445P17.8	blue	0.36406104	0.80699372	-0.1673342	-0.0146564	-0.5713486	0.00411684
cg01471648	U7;ZBTB7C	blue	0.36447183	0.85750009	-0.13013	0.00738692	-0.6577983	0.0320427
cg26691307	KCNN2;KCNN2	blue	-0.5194463	-0.7167973	0.15307772	-0.151675	0.38099493	-0.0105249
cg04432341	RPS20P32	blue	0.39050531	0.65233448	-0.2199005	0.07999802	-0.357075	-0.0487831
cg12545905	RP11-330M19.1;RP11-	blue	0.41002547	0.43141395	-0.0372339	0.20462695	-0.1244838	0.00666082
cg19314159	SH3RF2;SH3RF2;SH3RF2	blue	0.26335038	0.84229243	-0.0717784	-0.1120037	-0.8484897	-0.0649438

cg10008501	RP11-403C10.2	blue	-0.5245538	-0.5940946	0.08429606	-0.1277342	0.28853139	-0.1293098
cg24508976	GRIP2	blue	0.50004856	0.5554897	0.06763852	0.19878188	-0.2853931	0.20573635
cg04658405	AC078941.1;AC078941.1;A	blue	0.67369296	0.70162052	0.35637146	0.21980674	-0.2836086	0.43129074
cg23305172	OR51F2;MMP26;MMP26	blue	0.41684399	0.75200088	0.11902582	0.11978087	-0.4500452	0.06949771
cg01036731	RP11-594C13.2	blue	0.56496305	0.73439819	-0.2062533	0.16344987	-0.2393023	0.01310087
cg27650171	GTF2H4;GTF2H4;VARS2;V	blue	-0.2895508	-0.4322846	0.19926866	0.08150659	0.39871755	-0.0283994
cg00477203	DUSP27	blue	0.35436456	0.72168401	-0.098613	-0.0014143	-0.4350021	0.03349075
cg06638433	IGF2BP1;IGF2BP1;IGF2BP	blue	-0.504929	-0.740434	0.28138786	-0.0224493	0.47469306	0.0038674
cg14627810	RP11-558N14.1;LEF1;LEF1	blue	-0.3549025	-0.5974436	-0.0257989	-0.1000382	0.45093879	-0.0839703
cg26924043	RP11-469N6.2	blue	0.47013596	0.64004875	-0.2131745	0.0751912	-0.2437839	-0.0029889
cg24119454	RPS6KL1;RPS6KL1;RPS6K	blue	0.54586876	0.8254825	0.02239454	0.13031787	-0.5050056	0.1880985
cg09386284	SPRY4;SPRY4;AC005592.2;	blue	-0.4879602	-0.5215704	0.05968897	-0.0106229	0.31764286	-0.1750431
cg17435897	PKHD1;PKHD1	blue	0.3528113	0.8229829	-0.0857369	0.11048541	-0.515676	0.00934398
cg04631160	RP1-151B14.6;IL2RB;IL2RB	blue	0.42680428	0.75465007	-0.0940886	0.06322586	-0.5007033	0.06558221
cg14432797	WFDC9	blue	0.6069725	0.63533032	-0.0922051	0.17456093	-0.2340996	0.15671056
cg07445258	ALDH1L1;ALDH1L1;ALDH	blue	0.34621784	0.76860256	-0.1065781	-0.0583394	-0.5268643	0.04010359
cg24159264	AC005232.1;AC005232.1;CP	blue	0.19721207	0.80662516	-0.1585583	-0.1067557	-0.7955001	-0.0884017
cg08937058	EPB41L1;EPB41L1;EPB41L	blue	0.39875467	0.83574887	0.03019061	0.09631179	-0.5594749	0.06266175
cg04822719	RP11-521E5.1	blue	0.37177761	0.61269622	-0.3797636	0.0886371	-0.249032	-0.1885706
cg12091542	RP11-148G20.1;CELSR3	blue	-0.4078915	-0.673287	-0.0015951	-0.001237	0.49440576	0.03415026
cg25755575	ESRRG;ESRRG	blue	-0.4959237	-0.7915162	0.13219902	-0.0390784	0.52912176	-0.0944335
cg01618385	C1orf167	blue	0.34189989	0.69250685	0.03072976	0.09342754	-0.3909831	-0.0526528
cg15927196	PON3;PON1;PON3;PON3	blue	-0.4457055	-0.7207222	0.09056846	-0.0749625	0.4929152	-0.0399506
cg10590465	CARM1	blue	-0.3888902	-0.5747037	0.15358158	-0.0914799	0.45203169	-0.0641279
cg04258099	AC090660.1	blue	0.5073369	0.59437285	0.20160703	0.19560583	-0.1569147	0.21714372
cg16267976	POU4F1;POU4F1-	blue	-0.3546856	-0.4330033	0.32971301	-0.0298973	0.33166304	0.0049391
cg08948808	SPRR4	blue	0.40865136	0.78576222	-0.1920722	0.10509034	-0.4934922	-0.0433248
cg12687597	ACOX2;ACOX2	blue	0.4929275	0.78573432	-0.1008439	0.19887878	-0.3817087	0.10018841
cg01041256	GABRA5;GABRA5;GABR	blue	0.73119946	0.68928029	0.02311638	0.29186106	-0.1866578	0.28690048
cg14887581	U6	blue	0.44154791	0.72988267	-0.3026057	0.08620365	-0.3301083	0.11641212
cg26184028	C6orf97;C6orf97	blue	-0.4599495	-0.6122595	0.01853833	-0.0208969	0.4548351	-0.1267276
cg20892257	U1	blue	0.25311142	0.45993062	-0.2316453	0.10220453	-0.1412778	-0.166226
cg00966255	ATF7IP2;ATF7IP2;ATF7IP2	blue	0.1477344	0.37953477	-0.1439973	-0.0143285	-0.2794821	0.00687228
cg16762439	AC006004.1	blue	0.44974039	0.81084294	-0.3209073	0.0147131	-0.4854612	-0.1471764
cg07707586	RAB27A;RAB27A;RAB27A	blue	0.18235013	0.74014535	-0.1500058	-0.0838459	-0.7972022	-0.1056596

cg17280346	ZIC1;ZIC1	blue	-0.2827235	-0.6586655	0.15016834	0.0400276	0.49177692	0.16585572
cg09349530	TBX20	blue	-0.2911162	-0.5471685	0.24082498	0.03098029	0.35607202	0.07945878
cg10301990	UBXN8;UBXN8;UBXN8;U	blue	-0.4754904	-0.5769473	0.13435231	0.07358228	0.49268973	-0.1042051
cg10675010	LOXL2;LOXL2;LOXL2;LO	blue	0.48726675	0.84061019	-0.160459	0.1131753	-0.4641669	0.06922139
cg25190534	CHRNA9	blue	0.52243084	0.8197362	-0.027602	0.15327102	-0.4292129	0.08778219
cg00397306	7SK	blue	0.36731788	0.77256542	-0.1147966	0.10228718	-0.4894506	-0.0464021
cg06908862	NKX2-2	blue	-0.4890019	-0.5193304	0.27861552	-0.0476471	0.25568268	-0.0594116
cg18431640	ZIC2;ZIC2;ZIC2;ZIC2;ZIC2;	blue	-0.4785122	-0.7801044	0.13966259	-0.0261281	0.49724864	-0.1112917
cg03004484	TBC1D14	blue	-0.2517253	-0.5478645	0.16271661	0.10432274	0.48024751	0.11107768
cg14426421	ACVR2A;ACVR2A;ACVR2	blue	-0.3281606	-0.7889182	0.05354754	0.08750321	0.67619737	-0.026736
cg13092589	SYCE1;SYCE1;SYCE1;CYP	blue	0.49192014	0.74145762	0.01608644	0.05983765	-0.4869198	0.15981085
cg10533304	MS4A18	blue	0.56074709	0.76342914	-0.0363436	0.20831817	-0.3591814	0.1858092
cg05118916	PCSK9;PCSK9;PCSK9	blue	-0.3239798	-0.5661747	0.29137187	0.11335058	0.4780677	0.00927297
cg23726512	RPL41P1	blue	0.5346884	0.56990551	0.10895826	0.12019301	-0.2008569	0.14170257
cg03356900	QRFPR;QRFPR;QRFPR	blue	-0.4638152	-0.5772216	0.20271481	0.04270799	0.41191972	0.01928748
cg02479198	DLG2	blue	0.4570191	0.8423733	0.0838723	0.02369714	-0.6268599	0.08065646
cg01936287	SGK2;SGK2;SGK2;SGK2;S	blue	0.35763437	0.81979529	0.02246071	0.06766981	-0.6045856	-0.0073995
cg08885556	SPEG;SPEG;SPEG;SPEG;SP	blue	0.62611634	0.8172974	-0.0589362	0.14023239	-0.381172	0.20901755
cg03545227	PTPRN;PTPRN;PTPRN;PTP	blue	-0.4709582	-0.7768574	0.07893492	-0.0725251	0.57309879	-0.1037003
cg05481474	ODZ4;ODZ4;ODZ4	blue	-0.3580628	-0.58297	0.33096774	0.05673419	0.41020777	0.02661009
cg18827787	AC100848.1;LINC00305;LIN	blue	0.27024706	0.790748	-0.1827386	-0.0509754	-0.6413731	-0.1254157
cg12802329	RIOK3	blue	0.31150177	0.69858325	0.14043125	0.08872265	-0.5087726	0.08909913
cg16589598	GPRC5B;GPRC5B;GPRC5B	blue	0.45613674	0.64386065	0.2295206	0.06139834	-0.3448134	0.26361441
cg23989204	RP11-594C13.2	blue	0.31249655	0.69536267	-0.0352322	0.02150248	-0.4979223	0.13770937
cg19943783	RP11-203H2.1	blue	0.34474477	0.78873159	-0.0670593	0.05225101	-0.5557332	-0.0511732
cg23470914	MBP;MBP;MBP;MBP;MBP	blue	0.39983451	0.43861648	-0.1413575	0.04951725	-0.2435768	0.08660822
cg13953090	RP11-20G13.2	blue	0.67317942	0.74326944	0.14388248	0.15440381	-0.3268244	0.20293681
cg11821086	ANKMY1;ANKMY1;ANK	blue	-0.2125677	-0.5142599	0.22696132	0.03957747	0.44716519	0.06029373
cg20991203	CCDC129	blue	0.41044332	0.61969248	-0.0448861	0.06834766	-0.273192	0.04706021
cg17408498	RP11-14O19.2;RP11-	blue	0.527347	0.57685453	0.35602787	0.11425981	-0.3004167	0.23219929
cg18987288	CECR3	blue	0.66911074	0.71931495	-0.0374924	0.16929752	-0.3329875	0.22570531
cg23217707	CTSE	blue	0.33277881	0.76485116	0.0055006	0.06074709	-0.514537	-0.0899586
cg05404256	RP11-70F11.8;RP11-70F11.8	blue	0.46011494	0.69268328	-0.0094218	0.13973977	-0.4201918	0.1377936
cg05020189	RP4-594L9.2	blue	0.317387	0.8227416	-0.0974215	0.00581134	-0.6683363	0.01737477
cg03516994	GS1-122H1.1;GS1-122H1.2	blue	0.27180577	0.72555497	0.10392952	-0.0459548	-0.595017	-0.0168982

cg15563382	RNF141;MRVI1-	blue	-0.4571846	-0.513149	0.07712674	-0.0687461	0.39329319	-0.0335243
cg23747244	LRRIQ4	blue	0.50192016	0.90058421	-0.0527226	0.0965966	-0.617456	0.03734269
cg24863814	RP11-35O7.1	blue	0.57884483	0.76629379	-0.0857173	0.1683384	-0.2992327	0.12143339
cg08936063	AC062028.1;AC062028.1;A	blue	-0.3595477	-0.3741172	0.04440238	0.07868018	0.31028398	-0.1919999
cg07790887	PRKACB;PRKACB;PRKAC	blue	0.49832389	0.82903621	0.00380779	0.07957008	-0.5124516	0.07419929
cg08260861	CNTNAP2	blue	0.46598883	0.65217048	-0.3708852	0.07262966	-0.2103854	-0.025308
cg25950936	RP11-14I17.2	blue	0.5989783	0.7039648	-0.1130667	0.13645898	-0.3157883	0.1273226
cg18911683	CRHR2;INMT;INMT	blue	0.43517195	0.84759579	-0.0634593	0.05974362	-0.5508055	-0.0010138
cg06073141	CLDN16;CLDN16;CLDN16;	blue	0.58867655	0.55071243	0.41061848	0.23257473	-0.2165985	0.21386455
cg19746031	SLC27A1	blue	0.47602489	0.58526059	-0.0189284	0.15845659	-0.0816312	-0.0621369
cg15527308	AC100775.1	blue	0.62625406	0.76908061	-0.1687057	0.23710084	-0.3166062	0.18449252
cg16992794	RP11-349O10.1	blue	0.56703576	0.76422058	-0.1277353	0.24436657	-0.2953959	0.13729893
cg22930031	ACER2;ACER2	blue	-0.2843048	-0.5307312	0.11672097	0.06834483	0.46458808	0.02259028
cg03086663	NR2F1;RP11-65F13.2	blue	-0.3604769	-0.4873112	0.51269509	0.05425453	0.30678644	0.04657246
cg14977785	ROBO2	blue	0.50168588	0.66419748	-0.0899688	0.15327476	-0.2049005	-0.0172452
cg22480178	CACNA2D3;CACNA2D3	blue	-0.4081825	-0.3887782	0.00915046	-0.1093894	0.18787009	-0.1650608
cg23976464	ASIC2	blue	-0.4574412	-0.5883186	0.13633492	-0.0133234	0.33594999	-0.0506799
cg24428040	C17orf28;C17orf28;C17orf28	blue	-0.401749	-0.5514275	0.13669081	0.07405841	0.4267808	-0.0099229
cg05501177	C7orf57;C7orf57;C7orf57	blue	0.48854709	0.52278855	0.15307837	0.25843324	-0.2071063	0.22197056
cg05297121	NPNT;NPNT;NPNT;NPNT;	blue	-0.1306367	-0.3552921	0.2830365	0.10417328	0.2779083	0.17926001
cg21709241	RP11-203H2.1	blue	0.19955886	0.68163276	-0.1115065	-0.1856793	-0.6412315	-0.0929839
cg00016913	PIPOX;PIPOX;PIPOX	blue	-0.3034921	-0.4244936	0.18851749	0.10597492	0.36015243	-0.0599369
cg20703957	ALDH1L1;ALDH1L1;ALDH	blue	0.37375695	0.7866037	-0.0619829	0.02544463	-0.5141446	0.06537445
cg10726517	B4GALNT4;B4GALNT4	blue	-0.3979826	-0.4763887	0.13821921	0.05489766	0.36420724	-0.0720005
cg13272108	NOP56;NOP56;NOP56;NOP	blue	0.05953524	0.49836294	0.1102327	-0.0125555	-0.4617105	-0.0422823
cg02289038	CCDC149;CCDC149;CCDC	blue	0.3274163	0.77754691	0.01661786	0.06511146	-0.6015346	0.02662432
cg11001844	ALDH1A2;CTD-	blue	-0.5092171	-0.6457462	0.18755009	-0.0890549	0.32340767	-0.1027608
cg11709870	USH2A	blue	0.66238958	0.68874098	0.20650474	0.30207891	-0.1744472	0.2325234
cg25977493	SHOX2;SHOX2;SHOX2;SH	blue	-0.3383543	-0.5613712	-0.0033713	-0.0229255	0.38126541	0.04018467
cg18542972	GPM6A;GPM6A;GPM6A;G	blue	0.51427016	0.77966161	-0.1459239	0.15269289	-0.3620443	0.06919826
cg00580617	CTD-2184D3.4;CTD-	blue	0.26001783	0.71690426	0.15450557	-0.0826258	-0.6556804	0.09211622
cg05341878	RIMS2;RIMS2;RIMS2;RIM	blue	0.22091641	0.6934481	-0.066383	0.01870652	-0.5716395	-0.0692051
cg17086672	ACSBG1;ACSBG1;ACSBG	blue	0.32750504	0.86963323	-0.0513057	-0.0513026	-0.7457444	0.00982265
cg12578955	HTRA1	blue	0.39240455	0.70677318	-0.180539	0.07324638	-0.407451	0.01899574
cg13808083	BARX1;RP11-231K24.2	blue	-0.4174972	-0.6118518	0.27236396	0.03104518	0.37074336	-0.0561073

cg17388248	EHF;EHF;EHF;EHF;EHF;EH	blue	0.39602532	0.86253299	0.12492217	-0.0375314	-0.7375719	0.08701153
cg04264549	INTU	blue	-0.5207474	-0.5444047	0.11617159	-0.0312618	0.38562936	-0.2056695
cg12002807	ATP12A;ATP12A	blue	-0.4886792	-0.5939342	0.1568206	0.03091275	0.43860431	-0.0735561
cg01303442	U6;ADGB;ADGB;ADGB	blue	0.31621528	0.8580512	-0.0776573	-0.0924032	-0.7597686	-0.0797613
cg26765815	NPTX1	blue	-0.5046912	-0.7456596	-0.0545706	-0.0587389	0.53841469	-0.2411066
cg25762442	CHD3;CHD3	blue	0.30749554	0.64517776	-0.0225791	-0.0062379	-0.4314409	-0.0598144
cg25838080	NR2E1;NR2E1	blue	-0.3623719	-0.7945036	0.00790335	0.06353459	0.62466355	0.06063411
cg00264597	AC012363.13	blue	0.33947412	0.37840053	-0.0550304	-0.0034033	-0.2094579	-0.2181822
cg10774309	RP11-436D23.1	blue	0.43301223	0.71489984	-0.2911537	0.05928325	-0.3304822	-0.0836671
cg11983051	UBA52	blue	-0.4445082	-0.5251688	0.20928847	-0.018023	0.39820597	-0.1057574
cg01632425	RP11-143J24.1	blue	-0.3520106	-0.64501	0.0884347	0.10114315	0.58990049	0.11548296
cg25285440	TSPAN8;LGR5	blue	0.64086945	0.79194013	0.1897267	0.27165229	-0.3465744	0.24638201
cg24438644	ADRB3;ADRB3;ADRB3	blue	-0.2772318	-0.5329989	0.19421148	0.01087372	0.38535101	0.02441239
cg18306943	ENTPD3;ENTPD3;ENTPD3;	blue	-0.5437821	-0.5851012	-0.0342043	-0.0600409	0.33473156	-0.1958658
cg10634619	TP73-AS1;TP73-AS1;TP73-	blue	-0.1961926	-0.5784279	0.23772907	0.03210491	0.54449077	0.11425309
cg13900978	AC005863.2	blue	0.53564945	0.87138608	0.12610699	0.14165477	-0.5364682	0.07293128
cg02089480	CYP2J2;CYP2J2;CYP2J2	blue	0.39651596	0.7866563	-0.0666678	0.12115534	-0.4409673	0.08439753
cg14384532	NTRK3;NTRK3;NTRK3;NT	blue	-0.6027822	-0.6830858	-0.1923351	-0.0920815	0.34322105	-0.1519472
cg19024632	KCNS3;KCNS3;KCNS3;KC	blue	-0.4240328	-0.6835379	0.03281048	0.01246673	0.4758099	-0.0762299
cg08397344	FNDC1	blue	-0.3409368	-0.471447	0.13563479	0.04444469	0.30950622	0.00194331
cg00178749	GREB1;GREB1;GREB1;GR	blue	0.52183318	0.69896252	-0.1373831	0.07074718	-0.2817804	0.00338343
cg02933002	AC092786.1	blue	0.42529634	0.76028911	-0.1405888	0.08075586	-0.4134141	0.00216724
cg06754312	ADARB2	blue	0.45169223	0.55495753	-0.0070188	0.06837886	-0.2354075	0.06839766
cg10957151	HOXD9;HOXD9;HOXD-	blue	-0.3383508	-0.4126277	0.21847891	0.07062332	0.28296026	0.0684853
cg09628454	TFAP2A;TFAP2A;TFAP2A;	blue	-0.5853263	-0.754708	0.21098241	-0.0460294	0.43777455	-0.119067
cg15489003	RP13-455A7.1	blue	0.37038793	0.57188692	-0.0980424	0.13932513	-0.2944199	-0.0469878
cg11652085	ARPP21;ARPP21;ARPP21;	blue	0.25479368	0.83669301	-0.1071645	-0.0915102	-0.6984687	-0.0463316
cg24383498	MMP21	blue	0.5530613	0.6479838	-0.0249893	0.12476465	-0.2690996	0.05228378
cg21631156	RP11-292D4.3	blue	0.13825534	0.50413836	0.00776588	-0.0532821	-0.3462313	-0.0279649
cg05678758	SHANK2;SHANK2	blue	0.44958623	0.84636885	-0.037778	0.05754899	-0.4928771	0.06462971
cg19939130	IFI16;IFI16;IFI16;IFI16;IFI1	blue	0.27541433	0.73231978	-0.2091606	-0.1258853	-0.6022571	-0.0567388
cg10736528	RP3-416H24.4	blue	0.61743812	0.70735248	-0.0333829	0.28502056	-0.2281497	0.13973255
cg08763292	ZSWIM5;ZSWIM5	blue	-0.3637229	-0.389517	0.21340141	0.09190001	0.25766228	-0.0734747
cg03724612	AP000697.6;SIM2;SIM2	blue	-0.438481	-0.5362485	0.10062342	-0.0498981	0.29614531	-0.0679675
cg16944574	MIR497HG;MIR497HG;MI	blue	0.5960173	0.70668052	-0.1045223	0.15428344	-0.2324139	0.08875564

cg05836521	AC007403.1;AC007403.2;A	blue	0.5254919	0.70069996	0.29146859	0.10410265	-0.3772692	0.1940998
cg25885282	INTS1	blue	-0.3691298	-0.7879688	0.07141217	0.07217911	0.67518365	-0.0673527
cg14473057	C1QL2	blue	0.58858991	0.71295068	-0.1089433	0.21961535	-0.2557373	0.0885844
cg04840871	RP11-344F13.1	blue	0.4774575	0.82859965	0.08751048	0.10569986	-0.4819745	0.02367987
cg08952776	OLFML2B	blue	0.54673941	0.68911285	0.03546215	0.12409682	-0.3697211	0.19466693
cg13984173	C15orf23;C15orf23;C15orf23	blue	0.51115752	0.6743035	-0.002212	0.18969844	-0.2721204	0.10451288
cg21525554	CCDC129;CCDC129;CCDC	blue	0.17995099	0.42860256	0.06235309	0.05965187	-0.3368014	-0.0558412
cg01244514	MIR1193;MIR494	blue	0.60243288	0.72834505	-0.2566878	0.24143483	-0.2481371	0.04871106
cg13920232	ERBB4;ERBB4;ERBB4;ERB	blue	-0.5039618	-0.7042596	0.06411673	-0.0921855	0.36684764	-0.0938011
cg23131353	RP11-651L5.2;CKAP4	blue	-0.5147771	-0.7990882	-0.1034142	-0.0972052	0.57118105	-0.1604205
cg12768383	H2AFX;H2AFX;DPAGT1;D	blue	0.47410967	0.57430222	0.03861766	0.11179892	-0.3164155	0.17963651
cg14411285	MCF2L	blue	0.43396515	0.76034998	0.05807062	0.04617619	-0.4880781	0.09584931
cg24500683	PTP4A1;PTP4A1;PTP4A1;P	blue	-0.2356142	-0.5556682	0.16893891	0.06079072	0.4825063	0.10053043
cg13864473	WNT3A;WNT3A	blue	-0.4271884	-0.3913955	0.1395627	-0.019637	0.11851987	-0.0911029
cg00023024	HTR1D;HTR1D	blue	0.43670209	0.6727555	-0.1169824	0.12944515	-0.327338	0.01006201
cg15196028	DEFB124;REM1	blue	-0.3542261	-0.5660864	0.20311569	0.15206839	0.51285835	-0.0494554
cg13357393	HOXC5;HOXC6;HOXC9;H	blue	-0.5946987	-0.6816441	0.00037074	-0.1182705	0.35676758	-0.2393741
cg01945641	AC006445.8	blue	-0.2152379	-0.3953506	0.13242988	0.0883141	0.25521515	0.18722063
cg17792108	CACNG5;CACNG5	blue	0.28936067	0.36649773	0.2082036	0.05604262	-0.1844445	-0.0138542
cg15809959	SRCIN1	blue	-0.4026409	-0.5709994	0.19829825	0.09823451	0.48667541	-0.0205208
cg03554283	HNF4G;HNF4G;HNF4G	blue	-0.4504525	-0.8116948	0.05603373	-0.0606475	0.56224107	-0.0102122
cg17363074	AC008869.1	blue	0.32933541	0.84479796	-0.090598	0.01901055	-0.6046503	-0.0020252
cg10148764	RPL31;RPL31;RPL31;RPL31	blue	-0.3580095	-0.7820936	0.16160469	0.05189891	0.56898532	0.0376655
cg08267047	SUMO1;SUMO1;SUMO1;S	blue	-0.3253235	-0.4142347	0.22337961	-0.0625813	0.24118342	-0.0423479
cg12108730	HAS3;HAS3;HAS3;HAS3	blue	-0.4196096	-0.6701022	-0.0077921	-0.0001375	0.54464141	-0.1568886
cg22045888	ADAM29;ADAM29;GLRA3	blue	-0.4981159	-0.7310718	0.21011992	0.04747395	0.51708876	-0.0271262
cg13580419	PSD3;PSD3;PSD3;PSD3	blue	0.41674673	0.78427599	-0.0815452	0.06308329	-0.4268671	0.02161432
cg19747232	ASIC2	blue	-0.4290117	-0.4562365	-0.0790309	0.10333566	0.32491066	-0.086707
cg26848105	AC002511.2	blue	0.36328914	0.89276339	-0.1113595	0.00340897	-0.6954845	-0.0241327
cg25922009	AC009236.1;AC009236.1	blue	0.43749474	0.78017362	-0.1053514	0.11801226	-0.438706	0.0816624
cg23143090	XXyac-YX65C7_A.3	blue	0.26364822	0.37955708	0.01096927	-0.0162794	-0.1506844	0.04250201
cg26662703	CFHR4;CFHR4;CFHR4	blue	0.28622562	0.69904295	-0.0979342	-0.0721548	-0.5346535	-0.0976782
cg05429386	S1PR5;S1PR5;S1PR5	blue	-0.3195795	-0.5641296	0.05445024	0.01160513	0.49607563	-0.0064167
cg09442828	ADRB3;ADRB3	blue	-0.407665	-0.6358617	-0.0413996	-0.0551235	0.45917825	-0.037118
cg15202552	FCRL3;FCRL3;FCRL3;FCR	blue	0.53060688	0.68753968	-0.0650926	0.24282577	-0.1526759	-0.0326402

cg18295183	GABRG2;GABRG2;GABR	blue	-0.3645611	-0.7767457	0.25195449	0.02594451	0.61174685	0.0640265
cg15150970	DNMT3A;DNMT3A;DNMT	blue	-0.3836695	-0.6145644	0.19469223	-0.094509	0.2213534	-0.0019133
cg05087928	ANKDD1B;ANKDD1B	blue	0.30014863	0.6972479	-0.1770843	-0.0028899	-0.4773434	0.00434164
cg05166820	HMGCLL1;HMGCLL1;HM	blue	-0.2374213	-0.407119	0.02459849	0.03718439	0.17544227	0.13349154
cg08510658	RP11-	blue	-0.468586	-0.66824	0.08483896	-0.065344	0.45053247	-0.0818999
cg13897416	TRIOBP;TRIOBP;TRIOBP;	blue	-0.2872941	-0.5484786	0.01150246	0.02405827	0.4234012	-0.0374435
cg17167253	EN1	blue	-0.3381198	-0.7147824	0.28011508	0.05025001	0.53187648	0.06794025
cg05194972	NKX2-4;RP11-	blue	-0.3856856	-0.6605777	0.30887916	-0.0192701	0.39129932	0.04074425
cg02595809	RP4-792G4.2;RP4-	blue	-0.4354864	-0.6785237	0.07742134	-0.033	0.51774918	-0.0790166
cg01177476	RP11-445O3.3	blue	0.3937365	0.51169608	-0.2068895	0.10666781	-0.1120675	0.00719996
cg01345505	RGS22;RGS22;RGS22;RGS	blue	0.35483828	0.80503334	-0.3610437	-0.0242329	-0.5329949	-0.0564152
cg01137772	AL354933.1	blue	0.52663008	0.61983558	-0.2263453	0.16535851	-0.2143494	0.01905689
cg18185665	CENPV;CENPV	blue	-0.5952383	-0.7095984	0.01968998	-0.0528924	0.41369896	-0.1908104
cg10298187	TPPP3;TPPP3;TPPP3;TPPP3	blue	-0.4110396	-0.5482033	0.2379691	0.0389998	0.38913548	-0.0290927
cg20718183	LRRC49;LRRC49;LRRC49;L	blue	0.46709025	0.5988638	-0.407093	0.05379643	-0.1939558	-0.0038099
cg09718076	RP11-804L24.2;RP11-	blue	0.49989231	0.70507554	-0.226265	0.14730709	-0.2980057	0.12855092
cg09873328	MIR370;MEG8;MEG8	blue	0.63672095	0.80123609	0.18563851	0.21974378	-0.3582688	0.31045207
cg05569203	PLCXD3;PLCXD3	blue	-0.4720978	-0.5719729	0.11390964	-0.1066595	0.31620162	-0.1024357
cg22021394	MITF;MITF;MITF;MITF;MI	blue	0.38667381	0.79248573	-0.1529053	0.29847986	-0.4643532	-0.0106814
cg15439515	SNORD112;DKK2	blue	0.37242424	0.7438701	-0.1168688	0.06300798	-0.4510058	0.12488351
cg00460235	ADRA1A;ADRA1A;ADRA	blue	-0.5579097	-0.7359718	-0.0313109	-0.1263182	0.50628942	-0.2584458
cg16428857	WDR46;WDR46;WDR46;W	blue	0.52789367	0.595797	0.03826791	0.27373965	-0.1670545	0.0158592
cg19544372	ZNF560	blue	-0.3396944	-0.696594	0.22477381	0.00159087	0.58210724	0.04717011
cg14018690	C3orf52;C3orf52;C3orf52;C3	blue	-0.2780555	-0.2230712	0.25759819	0.06071593	0.00908092	-0.0802264
cg01331430	RP11-474B16.1;C12orf75	blue	0.41705689	0.76872119	-0.2816414	0.07840114	-0.3878622	-0.0911468
cg04486846	TTC28;TTC28-AS1;TTC28-	blue	0.28672068	0.66790404	-0.0284027	0.04593496	-0.4304055	-0.0296343
cg26490299	CTB-43E15.3;CTB-	blue	0.27628428	0.8506969	-0.048771	-0.0665411	-0.7905	-0.0225521
cg01223086	RP11-522B15.3;RP11-	blue	-0.3126843	-0.4696683	0.03964657	0.03741586	0.28805119	-0.053082
cg10007431	RP11-33N16.2	blue	-0.2839236	-0.3578373	0.2377275	-0.1034608	0.19736119	-0.0164089
cg23748098	IL31;LRRC43	blue	0.28710171	0.77698928	0.02847981	-0.0147137	-0.662333	-0.0031202
cg07994661	FHDC1	blue	-0.3265432	-0.7199533	0.21679201	0.10099288	0.61173422	0.09779622
cg23225103	CNTN2;CNTN2	blue	0.25682882	0.78326646	-0.015843	-0.0597423	-0.6209504	-0.081053
cg05800321	LY6D;RP11-	blue	0.26226755	0.49107405	0.03354557	-0.0059658	-0.2756565	0.10473959
cg24385652	RP11-507P23.3;RP11-	blue	0.27114405	0.80800914	0.08375876	-0.0039957	-0.7287169	0.08482331
cg11453712	PHPT1;PHPT1	blue	-0.2859959	-0.7050973	0.26503014	0.0791657	0.59463055	0.03623907

cg00592995	ZBP1;ZBP1;ZBP1;ZBP1;ZBP	blue	-0.0382548	-0.4643008	0.07962606	0.05488764	0.43459665	0.18057584
cg01232479	MIR543	blue	0.3817043	0.55040019	0.02684147	-0.0297526	-0.275314	0.02396616
cg06295928	PAPOLB	blue	0.37047335	0.61533515	0.03061598	0.0761978	-0.3197383	0.10667417
cg05617886	RP11-	blue	0.4864832	0.58955528	-0.0435794	-0.0316975	-0.3033444	0.09264189
cg00913949	ESRRG;ESRRG	blue	-0.3395858	-0.709679	0.13768925	-0.0128209	0.58409592	-0.0005529
cg16682135	RP11-390E23.3;RP11-	blue	0.4391184	0.68097154	0.17777224	0.18071853	-0.3193695	0.12964853
cg03619815	AC096559.2	blue	0.53686441	0.55249362	0.23176446	0.15047074	-0.130152	0.18168086
cg04566296	RP11-438B23.2	blue	0.51355355	0.70142304	-0.0793119	0.26226381	-0.1930515	0.02366844
cg07621046	C10orf82;C10orf82;RP11-	blue	-0.3943192	-0.7300358	0.16334926	-0.0366284	0.478783	0.08667169
cg27459307	CERK;CERK	blue	0.61625264	0.73272137	0.01851896	0.11940838	-0.3486895	0.25588907
cg07072594	PCDH7;PCDH7	blue	0.50538179	0.72030274	0.12876843	0.16968526	-0.2800652	0.07997936
cg02719508	AHNAK2	blue	-0.4847338	-0.6348608	0.08554335	0.1003186	0.51119918	-0.1546743
cg17149655	RORA;RORA;RORA;RORA	blue	-0.3456844	-0.5162115	0.2667529	0.00041436	0.34816643	-0.1109274
cg09887687	CLEC2B;RP11-	blue	-0.2639063	-0.3986723	0.00881774	0.06483658	0.39035714	-0.1587286
cg04026701	BTF3L4P1	blue	0.63392216	0.75467872	0.12056009	0.2127774	-0.2597474	0.27876521
cg02990368	ETNK2	blue	-0.4195975	-0.5648742	0.03716748	0.1333539	0.50301993	-0.13093
cg06970461	ADRA2B	blue	-0.5065861	-0.663608	0.06388092	0.00597089	0.4413856	-0.0260718
cg10144868	RP3-438O4.4;MTMR3	blue	0.37219312	0.73342446	-0.0610472	0.03517228	-0.4969956	0.10749272
cg08037744	SCAND3	blue	-0.4475195	-0.6041761	0.04885629	0.06218245	0.51965025	-0.1594185
cg25761626	PPYR1;XXyac-YR14BB7.1	blue	-0.2218073	-0.3710235	0.13675568	0.08902532	0.33819935	-0.2223714
cg12325507	GDA;GDA;GDA;GDA;GD	blue	-0.4116519	-0.3910494	0.10766072	-0.0023508	0.20762929	0.02165636
cg02322365	RP11-256I9.1;RP11-256I9.1	blue	0.59308941	0.79782957	-0.1124097	0.17917	-0.3148529	0.12864977
cg14588606	KRTAP1-5;KRTAP1-5	blue	0.40761632	0.63940947	-0.1784013	0.13003973	-0.3295315	-0.1008786
cg01648751	OSMR;OSMR;OSMR	blue	0.38849908	0.69927872	0.31293576	0.01730014	-0.4647902	0.07367331
cg15796496	DDX18P6;LINC00502	blue	0.42921754	0.58822839	0.18202754	0.13463224	-0.2090336	0.03114606
cg27331316	CSMD2	blue	0.35920025	0.69872577	-0.0184113	0.08525755	-0.4134196	0.05686795
cg08267489	RP11-	blue	-0.5460754	-0.6197283	0.06445751	-0.0061399	0.36297984	-0.1078892
cg21357291	ATP8B1	blue	-0.2778484	-0.792656	0.23781959	0.12924878	0.73145977	0.06158214
cg03223765	CHKA	blue	0.47803589	0.56998787	-0.1866929	0.21337019	-0.1395104	0.21399983
cg12060123	HBG2	blue	0.37872216	0.69259166	-0.1543481	0.08724555	-0.3624879	-0.0606172
cg11585912	SORBS2;RP11-301L8.2	blue	-0.4177523	-0.5156897	0.24113543	0.03099376	0.33956105	0.09768684
cg12665742	CALCR;CALCR;CALCR	blue	-0.5074759	-0.6145739	0.26942739	-0.0996088	0.28078228	0.02514966
cg02601061	PODXL;PODXL	blue	0.36186331	0.6023166	0.00803895	0.08746941	-0.3959208	0.10463535
cg09518518	AADACL4	blue	0.41416873	0.41382028	-0.0334982	-0.0251431	-0.2842217	0.10817558
cg20122786	GTF2A1;GTF2A1;GTF2A1;	blue	-0.3714348	-0.5705199	0.14358935	0.02587672	0.32063907	0.1074105

cg26730571	RP11-317O24.1	blue	0.50678833	0.57899085	-0.0807464	0.09634297	-0.2641503	0.22172285
cg02773945	NR5A2;NR5A2;NR5A2;NR5	blue	-0.6678751	-0.7773909	0.12429313	-0.1260043	0.30344454	-0.1613546
cg01914045	CLDN8;CLDN8	blue	0.55609206	0.74133874	-0.0756139	0.0765268	-0.26961	0.04280488
cg05412882	RCAN2	blue	0.20005201	0.70224075	-0.1749392	-0.0611144	-0.5860143	0.02916182
cg21306006	LINC00473;LINC00473	blue	-0.4666344	-0.7275827	0.03577181	-0.0315338	0.46681122	-0.0257508
cg25998062	RP11-554K11.2	blue	0.48270727	0.58875607	-0.0095407	0.13534684	-0.2509466	0.16740536
cg26648579	LA16c-366D1.3	blue	0.39101979	0.54722159	0.08546393	0.11501096	-0.2321403	0.10440722
cg05862393	SLC5A12;SLC5A12;SLC5A	blue	0.68409019	0.66522095	0.03863687	0.29659093	-0.0921504	0.1562923
cg23333513	ZNF607;ZNF607	blue	-0.5296863	-0.6085088	0.11548015	-0.1330121	0.26308969	-0.1358411
cg14971895	RP11-80K21.1	blue	-0.2622746	-0.7513021	0.21960044	0.10367104	0.66148159	0.15544133
cg22461835	ADRA1A;ADRA1A;ADRA	blue	-0.2792605	-0.5710895	0.14286992	0.10173997	0.507208	0.0201124
cg23644005	AC093702.1	blue	-0.4327126	-0.6539301	0.02164711	-0.0330574	0.47434263	-0.1266732
cg03575666	SFRP1;SFRP1	blue	-0.2398542	-0.4133964	0.31831841	0.12523389	0.36374096	0.12416872
cg23930923	ZNF665	blue	-0.322662	-0.4290129	0.27110504	0.06309723	0.20794791	0.0255481
cg20068620	MAPK4;MAPK4	blue	-0.5010921	-0.6747566	0.1291791	-0.0635004	0.41527167	-0.1097543
cg11926119	GPRC5B;GPRC5B;GPRC5B	blue	-0.2170283	-0.4454511	0.17265803	0.00404908	0.38237621	0.07896996
cg00382230	SNORD112	blue	0.56712008	0.78570504	-0.109018	0.16287665	-0.3515914	0.10654984
cg27556051	NR5A2;NR5A2;NR5A2;NR5	blue	-0.5828229	-0.6340344	0.10253232	-0.1067492	0.26802955	-0.306058
cg24144259	RP11-116A1.1	blue	0.37682791	0.77190254	-0.1766531	0.16544255	-0.4155441	-0.0522482
cg19130511	RP6-109B7.3;C22orf26;RP6-	blue	0.50284635	0.75906688	0.0281724	0.15663712	-0.4455277	0.23826539
cg04080057	CACNG6;CACNG6	blue	-0.3490014	-0.4777703	0.17961028	0.01461431	0.27130595	-0.0110102
cg25286285	DOC2A;DOC2A;DOC2A;D	blue	-0.4084584	-0.3905943	0.10648532	0.02718381	0.30615209	-0.0593133
cg19108881	CASR;CASR	blue	-0.4359743	-0.6673915	-0.0102664	0.01135345	0.52074279	-0.1272044
cg20134781	S1PR5;S1PR5;S1PR5	blue	-0.424538	-0.5843008	0.15909435	-0.0185372	0.44638214	-0.0890361
cg25901248	NBEA;NBEA	blue	-0.3723898	-0.5175902	0.15416771	0.01390122	0.42294512	-0.0424007
cg02637512	NME5;NME5;NME5	blue	0.32202062	0.86467041	-0.0861023	-0.0404105	-0.7042865	-0.0493666
cg15647538	GDA;GDA;GDA;GDA	blue	0.69046393	0.74644904	0.32229813	0.25050478	-0.3098727	0.32817435
cg15745401	P2RY6;P2RY6;P2RY6;P2R	blue	0.46040209	0.56283882	-0.0614803	0.02212927	-0.0799134	0.06219212
cg23039728	CPS1	blue	0.22523579	0.54715001	-0.2376143	-0.0527123	-0.3153626	-0.1807102
cg16681647	RNU1-23P	blue	0.41963265	0.5309372	-0.0620885	0.11893114	-0.2562654	0.00648968
cg20102245	RP11-536D16.2	blue	0.52103039	0.59099165	0.12730578	0.27091429	-0.2032324	0.34943531
cg07492757	TFAP2A;TFAP2A;TFAP2A;	blue	-0.3432943	-0.6801421	0.20413369	0.00992826	0.54349852	0.07046176
cg17821375	DBX1;DBX1	blue	-0.4034285	-0.4303721	0.33878888	0.0359528	0.22871072	0.07848963
cg09229893	BMP4;BMP4;BMP4	blue	-0.3409059	-0.631707	0.22783093	-0.0139536	0.41729268	0.13252724
cg14317513	AP000697.6;SIM2;SIM2	blue	-0.2954085	-0.5977237	0.22911455	0.15722216	0.46571778	0.10354814

cg11081206	DEFB103A	blue	0.30716174	0.76083998	0.12596091	-0.0649495	-0.6236963	0.06972689
cg15213116	RIBC2;RIBC2	blue	0.54236511	0.71119987	0.01341563	0.11572156	-0.4570328	0.07499901
cg23779153	RP11-713P17.5	blue	0.36509018	0.3617924	-0.2845412	0.23069845	-0.0612034	-0.0605846
cg03568518	PLXNB1;PLXNB1;PLXNB1	blue	0.49037244	0.76585381	0.27258443	0.18986875	-0.4525739	0.24116549
cg23483316	C10orf54	blue	-0.2950871	-0.5561263	0.23127262	0.09786088	0.49585609	-0.0420939
cg25995980	GPRIN2	blue	-0.5287603	-0.765754	0.08519424	-0.0302893	0.5780666	-0.1961596
cg12770741	NXN;NXN;NXN	blue	-0.0833471	-0.3174495	0.2601197	0.06870679	0.272842	0.21238188
cg26266099	AL031602.1	blue	-0.2601484	-0.6991445	0.13275145	0.12380891	0.64347134	-0.0600975
cg21638259	RP11-683L23.4;RP11-	blue	0.27813687	0.42116417	-0.0935347	0.09196046	-0.2015767	-0.0353117
cg09209931	RP11-692C24.2	blue	0.45896039	0.84473435	0.03171957	0.06271466	-0.596992	0.06726656
cg09924165	IGFBP5;IGFBP5;AC007563.	blue	-0.4972601	-0.6125463	0.00182292	-0.0732587	0.37338741	-0.1126261
cg03659565	DIRC3;DIRC3	blue	-0.4149201	-0.776351	0.04026779	0.04991704	0.61843737	-0.1272461
cg13606025	KCNJ16;AC002539.1	blue	0.53639956	0.73703076	-0.163637	0.15284698	-0.3090982	0.0632759
cg20673481	KCNS3;KCNS3;KCNS3;KC	blue	-0.4723935	-0.67674	0.01834601	-0.0699258	0.41690411	-0.0535848
cg03192867	C20orf118	blue	-0.3000924	-0.4281871	0.11796139	-0.0442029	0.32231615	-0.0322717
cg24697582	RP11-164C12.2;RP11-	blue	0.47591966	0.65906724	0.01840556	0.04464585	-0.3245483	-0.0015869
cg27304042	CA12;CA12;CA12;CA12	blue	-0.4046762	-0.4708628	0.08242587	0.07397152	0.40115565	-0.074393
cg09659734	GNAO1;CTD-	blue	-0.2550943	-0.7745663	0.13485059	0.03500776	0.66270829	0.06248123
cg16456919	LRRN2;LRRN2;LRRN2	blue	0.56196987	0.61810898	0.05951968	0.28679366	-0.1594513	0.18758975
cg00891278	CCDC37;CCDC37;RP11-	blue	-0.5413494	-0.8038397	-0.0016216	-0.0326929	0.52161436	-0.0994492
cg17078629	RP11-211G3.3;RTP2;RP11-	blue	0.21099259	0.64850173	0.04156908	-0.0003277	-0.5564895	0.09445813
cg24875029	POU3F3	blue	-0.2640121	-0.3816797	0.27667164	0.06351747	0.34933322	0.06272021
cg01930686	GANC;GANC	blue	0.24536361	0.66613155	-0.1833212	0.00905453	-0.5449571	-0.0748162
cg22633722	CBS;CBS;CBS;CBS;CBS;CB	blue	-0.3813107	-0.5508192	0.23313382	0.01920987	0.40029535	0.14865164
cg02694099	CT62;CT62;CT62;THSD4	blue	-0.3662035	-0.5805491	0.18450891	-0.1286214	0.36799477	0.02466665
cg15811883	TSSK1B;CTD-	blue	0.42737492	0.57516502	-0.3175144	0.04683921	-0.2415259	0.03293428
cg23119960	TCF12;RP11-	blue	-0.4363711	-0.6778456	8.18E-05	-0.0748062	0.52465475	-0.2015161
cg09257824	WT1-AS;WT1-AS	blue	-0.4759384	-0.6285461	0.24075553	0.04023666	0.43572745	-0.1188579
cg06929230	AATF	blue	-0.4538096	-0.7240256	-0.0569911	0.01367953	0.47317468	-0.1124727
cg20183094	FEZF1	blue	-0.3942089	-0.7920349	0.13828727	-0.056684	0.58107644	-0.0914373
cg22083798	IGFBP3;IGFBP3;IGFBP3;IG	blue	-0.3902045	-0.5368181	0.19049562	0.02519525	0.29926324	-0.0368874
cg25493727	HAND2;HAND2;HAND2;H	blue	-0.2799967	-0.7444209	0.24817678	0.04037791	0.66436604	0.09208077
cg03321152	DNASE1;DNASE1;DNASE	blue	0.38109958	0.37365307	-0.3431942	0.04324173	0.03275341	0.01576554
cg20892840	CCNL1;CCNL1;CCNL1;CC	blue	0.25175059	0.33647891	-0.0895252	0.04003246	-0.0595531	-0.0431528
cg10552807	PDE4B;PDE4B	blue	-0.2782173	-0.7032707	0.15164737	0.00866418	0.5936715	0.0588647

cg11095897	TFAP2A;TFAP2A;TFAP2A;	blue	-0.533416	-0.5968431	0.02057719	0.02557357	0.36940742	-0.2486797
cg00708598	LECT1;LECT1;LECT1;LECT	blue	-0.2901992	-0.5847816	0.13596568	-0.0484981	0.4330888	-0.0395788
cg24911146	CAMLG;CAMLG	blue	-0.3266739	-0.5723607	0.13615942	0.08844042	0.45615014	-0.0409424
cg00286621	CD164L2;CD164L2;CD164L	blue	-0.4214623	-0.6878388	0.12014399	0.03776973	0.54580156	-0.0837833
cg17507317	FGD5;FGD5	blue	0.48526916	0.59175006	-0.1738919	0.11231424	-0.2380978	0.25260573
cg17974477	ASAP2	blue	0.26395669	0.83147072	-0.1102717	-0.00395	-0.7475988	-0.0359848
cg14414646	RP1-149A16.3	blue	0.24993302	0.80127787	0.07991608	-0.0180483	-0.6580223	-0.0681204
cg05641351	EFNA1;EFNA1;EFNA1	blue	-0.4310695	-0.467085	0.05839862	-0.021322	0.28968269	-0.1291145
cg23712683	U6	blue	0.29042513	0.78635521	-0.2141721	0.01366015	-0.5626962	-0.1934912
cg23912509	RMST;MIR135A2;RMST	blue	0.3597876	0.76261691	-0.176305	-0.0120837	-0.4592476	-0.1407688
cg13734329	AC093381.2;AC093381.2;A	blue	0.42805353	0.77695462	-0.2429113	0.03369924	-0.3631999	0.02289943
cg18552861	GDF7	blue	-0.5603989	-0.6440337	0.01101256	-0.137837	0.2757968	-0.251182
cg07644335	RP5-1050E16.1;RP5-	blue	0.24384605	0.71207531	-0.1971858	0.02581106	-0.5199266	-0.1168081
cg17465616	ANO7L1;ANO7L1	blue	-0.3230045	-0.745856	-0.0454025	0.0169844	0.65740873	0.12588028
cg04461867	SERPINA11;RP11-349I1.2	blue	0.35275224	0.38297152	0.10086582	0.1933526	-0.1015402	-0.0001148
cg24091470	RP5-1010E17.2;RP5-	blue	0.2274741	0.48575357	-0.2789192	-0.0070779	-0.3237931	-0.0647617
cg10406295	SFRP1;SFRP1	blue	-0.2757702	-0.6848094	0.23716555	0.01603196	0.56242987	0.13458446
cg23410129	SRC;SRC;SRC;SRC;SRC;SR	blue	0.2314225	0.43935583	-0.1479391	-0.0600919	-0.3436291	0.09456579
cg09591866	ALDH1L1;ALDH1L1-	blue	-0.4974982	-0.786905	0.19030379	-0.0172936	0.50531721	-0.0411126
cg18830442	SAMD5;SAMD5	blue	-0.5299027	-0.6692012	0.01580982	-0.0687609	0.38078379	-0.1234234
cg00942432	CDH4	blue	-0.2335666	-0.3754123	0.09985579	0.11691531	0.32574127	0.00680433
cg13459303	TMEM176A;TMEM176A;T	blue	0.61221966	0.64468618	0.07301645	0.22731779	-0.2306431	0.24959914
cg25853940	EHBP1;EHBP1	blue	0.48134249	0.67626084	-0.1447243	0.19753502	-0.3013505	0.13158895
cg22922249	AJAP1	blue	-0.5029345	-0.6912213	0.00166939	0.00122227	0.44743776	-0.2190242
cg05238069	CENPV;CENPV	blue	-0.44738	-0.6097531	0.06503293	5.11E-05	0.34145838	-0.043785
cg07492518	TBC1D20;TBC1D20;TBC1D	blue	-0.3354141	-0.5243081	0.06160589	0.16663218	0.4365542	0.0063379
cg11026799	AC073479.1;AC073479.1;A	blue	0.46354288	0.53168508	-0.0103175	0.18932349	-0.1208944	-0.0041467
cg18835865	FRMD6;FRMD6;FRMD6;F	blue	0.32038149	0.70804063	0.3470457	-0.0083671	-0.6187066	0.09571378
cg03217897	AGBL4;AGBL4	blue	-0.4470099	-0.7802811	0.00565936	-0.010759	0.56586688	-0.0929766
cg01404615	DKK2;DKK2	blue	-0.4026405	-0.4822867	0.2542847	-0.0085452	0.28552161	-0.0736185
cg02791331	ECE2;ECE2;ECE2;ECE2;ECE	blue	-0.2281324	-0.5007864	0.19335725	0.01793482	0.40066804	0.05046934
cg06444390	AHNAK2;AHNAK2;AHNA	blue	0.31756536	0.86757832	0.01288847	0.01107205	-0.6948002	-0.0312727
cg15050627	RP11-547I7.2;RP11-	blue	-0.2136299	-0.6165807	0.35071691	0.19124467	0.52382755	0.16036372
cg14738170	PCDHGA5	blue	-0.3266667	-0.4911443	0.2245506	-0.1481826	0.28480219	-0.0234614
cg00076543	HLA-L;HLA-L;HCG17	blue	-0.403879	-0.4012958	0.12597119	0.06880828	0.27910506	-0.0973184

cg10538146	KLHDC5;KLHDC5;KLHDC	blue	-0.2363278	-0.4529017	0.3994867	0.0988988	0.40658179	0.11078861
cg16471444	NEK10;NEK10	blue	0.40006721	0.77109536	-0.2144358	0.11606815	-0.3309126	-0.0441325
cg24744455	ADAMTS12;ADAMTS12;A	blue	-0.2945732	-0.8305667	0.16224537	0.08947916	0.71372805	0.03096849
cg25807280	GS1-	blue	0.21057613	0.60072684	-0.0318641	0.00751827	-0.451442	-0.1551844
cg05432996	RASIP1	blue	-0.1822985	-0.4451736	0.24369443	0.19560831	0.40281405	0.0734527
cg18887327	CHD7	blue	0.28634801	0.84408512	-0.1387457	-0.1218126	-0.7689645	-0.0317901
cg03520683	LINC00391	blue	-0.440958	-0.6035255	0.20386378	-0.0243644	0.4021831	-0.0856591
cg04243723	Y_RNA	blue	0.39396402	0.57294049	-0.0835731	-0.0128739	-0.3157957	0.02994283
cg01789829	HTR4	blue	0.442512	0.75174	-0.0395461	0.03259902	-0.4580348	-0.0001641
cg12683944	ITGB3;ITGB3;ITGB3;ITGB3	blue	-0.4106117	-0.4230081	0.2317366	0.04272965	0.28281088	-0.0952116
cg02540504	ANAPC16;ASCC1;ASCC1;	blue	-0.3477236	-0.4615431	-0.0074518	-0.0224373	0.39100777	-0.0234513
cg08383551	AP005138.1	blue	0.43414648	0.84970085	-0.0034283	0.10895645	-0.4979396	0.01825613
cg23791803	RP11-481G8.2;RP11-	blue	0.49883306	0.66546582	-0.2389037	0.16198929	-0.1919975	0.20254488
cg07900886	CHRNA7;CHRNA7	blue	0.51512031	0.75099471	-0.1368624	0.08360401	-0.3778026	0.12000122
cg03860562	CTC-504A5.1;CTC-504A5.1	blue	-0.4193145	-0.4492587	-0.0056594	-0.0213597	0.27675636	-0.1716707
cg10307545	MED12L;MED12L	blue	-0.302214	-0.7953338	0.08017141	0.07425355	0.72336623	0.03904982
cg11326613	CCDC107;CCDC107;CCDC	blue	-0.3999596	-0.6452055	0.13215687	0.02359695	0.57094814	-0.0534286
cg07553626	LPAR3	blue	-0.3758434	-0.5453942	0.1851707	0.03984918	0.35604226	-0.0018536
cg14724471	RP5-	blue	-0.4465119	-0.7032857	0.11414345	-0.0953936	0.45879855	-0.0598226
cg23799375	PACRG;PACRG;PACRG;P	blue	-0.2667796	-0.428422	0.29798614	0.20212076	0.31850483	0.08872652
cg14369643	CTC-209H22.3	blue	-0.4536986	-0.456519	0.22835888	0.0193954	0.2927758	-0.0922179
cg15001747	RP11-445F12.2;RP11-	blue	-0.4784475	-0.8092647	0.14088508	-0.13469	0.49206689	-0.0477514
cg11515378	RP11-315F22.1	blue	0.30638437	0.44905215	0.17961373	-0.0284339	-0.2495253	-0.1284006
cg06059987	ETNK2;ETNK2;ETNK2;RP1	blue	0.46160516	0.76037263	-0.0698335	0.16392726	-0.3715463	0.09154803
cg21056723	RP11-345F18.1	blue	-0.4517704	-0.493813	-0.0271463	0.00763179	0.33471066	-0.1708732
cg10009157	MAGI1;MAGI1	blue	-0.4903135	-0.6326078	0.0771376	0.0046114	0.50674624	-0.088364
cg21043312	SASH1	blue	0.45307115	0.70849783	0.19959188	0.16039703	-0.3651915	0.09460287
cg06147474	AC011741.1;CTNNA2;CTN	blue	0.40357278	0.66621529	-0.3210799	0.12716385	-0.2682036	0.01043184
cg12670477	IGSF22;IGSF22;RP11-	blue	-0.3651066	-0.5242357	0.17212719	-0.0005025	0.30243608	-0.0119307
cg13896687	PELI3;PELI3;PELI3;PELI3;P	blue	-0.3427806	-0.4295211	0.06073684	0.12420214	0.3570859	-0.0220589
cg17237022	SIGLEC11;U3;SIGLEC11	blue	0.40127314	0.82856514	-0.0094349	0.0316128	-0.5335738	-0.0349954
cg11137145	KPRP	blue	0.25246431	0.44120898	0.1596521	-0.0904089	-0.2205461	-0.0607154
cg06418993	RP11-183I6.2	blue	0.43406042	0.78685587	-0.2014536	0.03649994	-0.400848	0.01309656
cg15381304	GPR6;GPR6	blue	-0.2663339	-0.5477997	0.16524734	0.05093117	0.39208154	0.05348709
cg18403792	MIR9-	blue	-0.3595791	-0.7527402	0.14873504	0.01226538	0.55888706	-0.0055403

cg20567537	AP1M2	blue	-0.3595181	-0.6465481	0.12324315	-0.0049376	0.46326544	0.07453847
cg13373589	TFAP2A;TFAP2A;TFAP2A;	blue	-0.3881409	-0.6964225	0.22477819	0.00435279	0.52491991	-0.0616892
cg14317648	DSG1	blue	0.41842803	0.86520585	-0.209942	0.03790248	-0.5591915	0.00299554
cg24419198	IGSF22;IGSF22;RP11-	blue	-0.2755727	-0.648242	-0.0350004	0.03832623	0.57913169	-0.0091765
cg18960565	NAGS;NAGS;PYY;PYY	blue	-0.3316278	-0.4631911	0.10340465	0.02303795	0.3157195	-0.091714
cg27156259	PXN;PXN;PXN;PXN;PXN;	blue	0.24831205	0.60697543	-0.1025314	0.10351767	-0.5271122	-0.0678824
cg02910208	VSTM2A;VSTM2A;VSTM2	blue	-0.4941514	-0.5639979	0.0110026	-0.1154786	0.2602741	-0.2677795
cg00482489	PIGW;MYO19;PIGW;MYO	blue	0.2750996	0.82476225	-0.1044593	-0.0032561	-0.6670397	-0.0267492
cg07523809	CARD10;CARD10	blue	0.66698317	0.67836612	-0.04167	0.26909244	-0.1558724	0.22727719
cg01286655	DKK3;DKK3;DKK3;DKK3;	blue	-0.3637284	-0.5139563	0.17721214	-0.0341647	0.27287703	-0.0134774
cg18808506	FGF1;FGF1;FGF1	blue	0.20195678	0.68298684	-0.0388544	-0.0441357	-0.5444422	-0.0632151
cg01577414	NAALAD2;NAALAD2;NA	blue	-0.2860958	-0.6300008	0.13997133	-0.0062295	0.56304559	-0.0435971
cg14995214	CHD5;CHD5	blue	-0.6017147	-0.6371377	0.0798195	-0.1006834	0.37321821	-0.163317
cg20472707	KIAA1755;BPI	blue	-0.443653	-0.5855886	0.17666187	-0.0233961	0.44920928	0.01465304
cg09249486	C3orf20	blue	0.17078261	0.75947546	0.03682197	-0.0645782	-0.7106367	0.00295778
cg15969123	RP11-883G14.2;RP11-	blue	0.2292885	0.33380966	-0.3560557	0.08728135	-0.138674	-0.0117466
cg23097897	MYCNOS;MYCN;MYCNO	blue	-0.534171	-0.8074271	-0.0621781	-0.1301559	0.52425702	-0.21272
cg10176692	OR4B1	blue	0.30376899	0.3789902	-0.0332061	0.02767026	-0.1833952	0.12954704
cg05762769	BTG3;BTG3;BTG3;BTG3	blue	-0.4325052	-0.6689824	0.17233292	-0.0232272	0.57535511	-0.0620625
cg25484990	FOXA2;FOXA2;FOXA2	blue	-0.4934683	-0.6405322	0.02444326	-0.0492474	0.43173445	-0.0953059
cg11738098	RP11-468N14.09	blue	0.5002491	0.66015265	0.02990448	0.2139805	-0.147907	0.15246888
cg23776282	RBX1;RBX1;XPNPEP3;XP	blue	-0.4557459	-0.4660811	0.09365589	0.05472519	0.22704995	-0.0730151
cg05986505	STK3;STK3;OSR2;OSR2;OS	blue	-0.1704629	-0.4993507	0.12576559	0.02947403	0.41539362	0.17863516
cg04284122	ZNF485;ZNF485;ZNF485	blue	-0.347855	-0.5374738	0.16361118	-0.0763137	0.29977001	-0.0426839
cg21381065	DCC	blue	-0.3502559	-0.7406539	0.20969163	0.01120618	0.54479102	0.1183524
cg12034118	IRF6;IRF6	blue	-0.290136	-0.5134802	0.06772226	-0.0466695	0.29813662	0.10916375
cg00541854	GPR142	blue	0.36191742	0.87353781	0.02780308	-0.0009749	-0.6779469	-0.0658764
cg16287507	RP11-69C17.3	blue	0.24479872	0.74828785	-0.1194341	-0.0644874	-0.6431837	0.02737267
cg15808426	AC007228.1;AC007228.1	blue	-0.3757713	-0.6790725	0.19739132	0.06548991	0.59733105	-0.0246928
cg16427752	MSH4	blue	-0.1104106	0.29009149	0.21453027	0.30386888	-0.2042649	0.0463419
cg07933230	LIN7B;LIN7B;LIN7B;LIN7B	blue	-0.3720472	-0.7450412	-0.0591641	0.01721378	0.62528496	-0.0604559
cg00266009	RALYL;RALYL;RALYL;RA	blue	-0.5302582	-0.6620099	0.0240801	-0.1091741	0.3047814	-0.109543
cg08860374	RP11-508M8.1	blue	0.50610616	0.72813097	0.14766166	0.24277463	-0.3125056	0.15411333
cg06715390	LOXL2;LOXL2;LOXL2;LO	blue	0.43274319	0.52415451	0.0522205	0.18149547	-0.0934404	0.23196261
cg13563284	LINC00237;LINC00237	blue	-0.4422596	-0.6146716	-0.0603524	-0.1003355	0.39786294	-0.1742753

cg02062744	ZFAND4;ZFAND4;ZFAND	blue	-0.1798491	-0.3697713	0.23036569	0.14022496	0.34051463	0.02540647
cg12595281	RGMA;RGMA;RGMA	blue	-0.3685054	-0.3910205	0.15411914	0.02034927	0.19168324	-0.0111819
cg19545580	RP11-725M22.1;RP11-	blue	0.41164101	0.67332984	0.30737389	0.21476617	-0.3505617	0.08056731
cg05583681	HSDL1;DNAAF1;DNAAF1;	blue	-0.4148477	-0.7988034	0.08907856	0.05992012	0.65286816	-0.0405312
cg09816507	PTCHD4;PTCHD4;PTCHD4	blue	-0.3326321	-0.7277072	0.10371096	0.01589874	0.58442008	0.00423279
cg13647382	COL12A1;COL12A1;COL12	blue	-0.4487852	-0.5733633	0.17209316	-0.0185131	0.34723387	-0.0959899
cg18538110	EBF4;EBF4;AL035460.2;EB	blue	0.30260605	0.61040474	0.11001523	0.08852835	-0.3531436	0.06443783
cg22174486	HRAS;HRAS;HRAS;HRAS;	blue	-0.4801426	-0.5929146	0.03213891	-0.0715416	0.39034703	-0.2244528
cg11076994	TCF15	blue	-0.3905385	-0.7005282	0.13374437	-0.0447747	0.47855553	-0.0348251
cg13371954	PRDM14;PRDM14	blue	-0.5139104	-0.7152076	-0.1000609	-0.1297475	0.42426065	-0.2615905
cg00048697	PDCL2	blue	0.61225997	0.66723694	-0.0828205	0.25002358	-0.1297943	0.07285426
cg23571811	ANO1;ANO1	blue	0.28922451	0.38821653	-0.0496577	0.00892131	-0.1770523	-0.1458105
cg24641352	ZFP41;ZFP41;ZFP41;ZFP41	blue	-0.1312683	-0.3979515	0.06549563	0.10144288	0.37284872	-0.0628556
cg20416037	PIP4K2C;PIP4K2C;PIP4K2	blue	-0.4616091	-0.5828033	0.02942968	0.09840526	0.47673501	-0.1203666
cg10918087	CCDC141;TTN;CCDC141	blue	0.50738646	0.71616811	-0.0513835	-0.0305441	-0.3558478	0.26098946
cg05596294	RP4-788L13.1;RP4-	blue	-0.3508835	-0.5111098	0.29529436	0.06561869	0.38646718	0.01768914
cg09344986	RP11-744I24.1;RP11-	blue	-0.5817198	-0.6424374	-0.0013185	-0.1046923	0.36376368	-0.2106819
cg03272370	RP5-839B4.7;RP5-839B4.7	blue	0.45595854	0.68640102	0.32935147	0.19385174	-0.3990557	0.1404575
cg27418256	RP11-483E7.1	blue	0.4205964	0.84542326	-0.0930107	0.09813498	-0.536943	0.02890061
cg04678392	SKOR1	blue	-0.4137363	-0.7745468	0.03524926	0.01759091	0.57025862	0.02852833
cg17218045	DHDDS;DHDDS;DHDDS;	blue	-0.5763517	-0.6741741	0.02508816	-0.1266833	0.34235955	-0.1548159
cg14440295	SNAP25;SNAP25;SNAP25;	blue	-0.3339372	-0.669417	0.17229957	0.11790995	0.56874593	0.09051138
cg21093033	TRAPPC9;TRAPPC9	blue	-0.3693421	-0.6262424	0.1427932	0.02098235	0.350868	0.0422438
cg14940173	RP11-398M15.1	blue	0.57881088	0.53922323	0.18957033	0.19335821	-0.1306769	0.25137268
cg25236230	RP11-325N19.3;RP11-	blue	-0.5832313	-0.7129001	0.15761233	-0.1094684	0.37867624	-0.2669702
cg04850479	PROZ	blue	0.24932812	0.7735264	0.034148	-0.0384125	-0.6415843	-0.0621994
cg06002660	MIB2;MIB2;MIB2;MIB2;MI	blue	0.31613455	0.55701715	0.03632831	0.0966892	-0.3530818	-0.1028908
cg02636137	CCDC13-AS1;CCDC13-	blue	0.39612255	0.85462864	-0.063101	0.03318724	-0.6334107	0.02188664
cg04991447	SEMA6A;SEMA6A;SEMA6	blue	-0.4750354	-0.6320272	0.0617336	-0.1785992	0.28468674	-0.0199776
cg18996636	AC073257.2	blue	0.39533164	0.67669884	0.00677718	0.1341284	-0.2695564	0.03406474
cg23519626	RP11-	blue	-0.1754697	-0.596113	0.12294836	0.10494033	0.55688334	0.11917557
cg26206039	RP11-1152H15.1;RP11-	blue	0.44483042	0.54906865	0.02219875	0.10526826	-0.1036395	0.01986926
cg16264616	HOXC9;HOXC-	blue	-0.3165368	-0.6005537	0.19860079	0.14268768	0.2957661	0.07917214
cg22271921	ZMAT4;ZMAT4;ZMAT4;Z	blue	-0.3963982	-0.6928237	0.15510265	-0.0868234	0.45548215	0.01883905
cg05527430	HPGD;HPGD;HPGD;HPGD	blue	-0.3444783	-0.4725989	0.11239467	-0.094304	0.24233699	-0.0204179

cg27046233	AC005307.1	blue	0.41303934	0.72497964	-0.103234	0.06335518	-0.3763505	-0.0836875
cg11178920	CA12;CA12;CA12;CA12	blue	-0.3892367	-0.485256	0.02061554	0.03050715	0.41846677	-0.0590812
cg19685627	DDIT4L;DDIT4L;DDIT4L;R	blue	-0.3420304	-0.7434513	0.11261691	0.08968368	0.59052915	0.00175745
cg09240474	EIF2B5;EIF2B5;EIF2B5;EIF	blue	0.41725137	0.44044626	-0.1219624	0.10030136	-0.1745848	0.12467634
cg25441287	SLC37A2;SLC37A2	blue	0.12918259	0.55463384	0.13047543	6.01E-05	-0.3777249	-0.0009211
cg12043062	PCDHGB1	blue	-0.2881227	-0.3717747	0.23763774	-0.0453859	0.21358519	0.06844531
cg21358537	CDH20;CDH20	blue	-0.2641161	-0.3637639	0.24171203	0.07763779	0.27145087	0.00728871
cg11101926	ASIC2	blue	-0.3073845	-0.4853889	0.06126735	0.08224711	0.34725229	-0.0034416
cg19612717	U6	blue	0.15318279	0.32358269	-0.4632631	-0.0694356	-0.1790002	-0.2251182
cg20547759	RP11-628E19.2	blue	0.29832194	0.72525127	-0.2143627	0.10658328	-0.3969327	-0.0642372
cg27283943	BAIAP3;BAIAP3;BAIAP3;B	blue	-0.4393768	-0.5166231	0.25382556	0.05311056	0.34639985	-0.040682
cg25992330	SPOCK2;SPOCK2;SPOCK2	blue	-0.1480726	-0.4114705	0.22827586	0.05256144	0.31682223	0.10121261
cg06972911	FAM59A;FAM59A	blue	-0.3896495	-0.5702408	0.20470031	0.09785506	0.40386029	-0.0444153
cg23734973	GRIA2;GRIA2;GRIA2;GRI	blue	-0.4300043	-0.704858	0.05388826	0.035191	0.50953938	-0.0293841
cg27488279	ANKRD7;ANKRD7;ANKR	blue	0.45166532	0.7791458	-0.1172179	0.08712221	-0.4309529	0.12910197
cg19092508	RP11-699L21.2;RP11-	blue	0.33631831	0.78542289	-0.0259413	0.03661355	-0.5306711	0.02249406
cg20858149	NKAIN1	blue	-0.3846784	-0.6423452	0.16516807	0.01005042	0.48185777	-0.0380035
cg01883643	C5orf47	blue	-0.4559974	-0.6753067	0.0447267	0.06676493	0.54741926	-0.0504039
cg00393837	AC018730.1	blue	-0.4573614	-0.6865368	0.12965838	-0.0287399	0.40255635	-0.0672624
cg03220633	MIR2277;FAM172A	blue	-0.3374219	-0.8035765	0.01638789	0.04410427	0.67430258	-0.0638266
cg23394610	VWA3B;VWA3B;VWA3B;	blue	-0.5748814	-0.700278	0.0242261	-0.1284319	0.34885518	-0.2172999
cg17120792	OR5I1	blue	0.29706098	0.61069041	0.02443077	0.18922854	-0.3281963	-0.1275268
cg04212589	ZNF765;ZNF765;ZNF765;Z	blue	-0.5466812	-0.649057	0.00961994	-0.0255346	0.4219217	-0.1583147
cg08533783	C11orf88;C11orf88;RP11-	blue	-0.4042609	-0.6468958	0.15327571	-0.0422621	0.39055676	0.01983292
cg19596468	MSX1	blue	-0.2381901	-0.4275417	0.12320345	0.07225669	0.33338695	0.13018368
cg15766075	ANKMY1;ANKMY1;ANK	blue	-0.2283818	-0.4681395	0.18060805	0.05507829	0.41532314	-0.0049488
cg13695726	UMODL1;TMPRSS3	blue	-0.401872	-0.4021012	0.13720628	0.07593639	0.26674089	-0.0767439
cg08941298	PGC;PGC;PGC	blue	0.34452696	0.70632582	0.01832009	0.07326015	-0.4226267	-0.0368067
cg22591525	FEZF2;FEZF2;FEZF2	blue	-0.2947516	-0.6829594	0.28205798	0.10177081	0.57991296	0.1000512
cg16857868	GPT2;GPT2;GPT2;GPT2;GP	blue	-0.4094469	-0.6635932	-0.1136318	-0.1401773	0.4339805	-0.0607957
cg06571387	HOXD12;HOXD12	blue	-0.3853432	-0.8032713	0.22402849	0.0315499	0.65812563	0.04052568
cg05279291	RP11-266O8.1;RP11-	blue	0.42663956	0.6129084	-0.2430606	0.09896468	-0.2519586	-0.0405246
cg00795825	KRTAP17-1	blue	0.20918737	0.74753568	-0.0091816	0.02170242	-0.5969219	-0.127501
cg16379826	ALOX5;ALOX5;ALOX5	blue	0.45445776	0.53996392	0.17061708	0.0528235	-0.2827166	0.29276433
cg16370491	KCNC2;KCNC2;KCNC2;KC	blue	-0.4587103	-0.6226703	0.10378965	-0.0144008	0.38445537	-0.045754

cg25562130	FAM65A;FAM65A;FAM65	blue	-0.488962	-0.7101871	0.03140744	0.04151439	0.597721	-0.1579633
cg05181354	SLC38A11;SLC38A11;SLC3	blue	-0.2970247	-0.4192032	0.23786962	0.13527006	0.32183495	-0.0064847
cg26635219	CFTR;CFTR;CFTR;CFTR	blue	-0.3633399	-0.559942	0.24466907	-0.0197539	0.31644665	0.11635138
cg13029847	SEZ6;SEZ6;SEZ6;SEZ6	blue	-0.4299358	-0.6483616	-0.004526	-0.094067	0.34049457	-0.123099
cg11762904	ETV5;ETV5;ETV5;DGKG	blue	-0.2950442	-0.6876738	0.13250609	0.05888193	0.56132415	0.02277828
cg24407204	C10orf96;C10orf96;C10orf96	blue	0.48933677	0.56523465	-0.0871765	0.13780903	-0.2195145	0.18365623
cg18339591	SNORD22	blue	0.32873555	0.62844263	0.1108871	0.11631814	-0.349716	0.04831026
cg12280622	INSR	blue	-0.3338208	-0.5388156	0.24093584	0.04526288	0.4000358	0.03840526
cg02380833	PTPRO;PTPRO;PTPRO;PTP	blue	-0.4508838	-0.5428347	0.17872499	-0.0211845	0.33628705	-0.0826215
cg09321972	C6orf97	blue	0.413598	0.69635889	-0.4154346	0.11587806	-0.3282082	-0.0093419
cg15033135	VAT1L	blue	0.2989117	0.5999748	-0.0786148	-0.0709168	-0.4139276	0.04886675
cg14250931	ZRANB2-	blue	-0.3956006	-0.3909349	-0.0580204	-0.0255036	0.21746668	-0.0761841
cg05304797	RP11-142G1.1	blue	0.40251771	0.59135999	0.09431739	0.14244262	-0.3214729	0.14112256
cg12358602	RP11-71G7.1;RP11-71G7.1	blue	0.60060748	0.7218036	0.21597088	0.27471829	-0.2731231	0.17042362
cg09045100	NKX2-1-AS1;NKX2-1;RP11-	blue	-0.4409429	-0.6667546	0.0366993	-0.0332544	0.54557761	-0.0569273
cg13105327	RP11-328K4.1;RP11-	blue	0.31457947	0.41030149	-0.1152247	-0.0178767	-0.1820596	0.07759774
cg02620731	AC122136.1;AC122136.1	blue	0.35204617	0.81552914	-0.0475169	0.0710638	-0.5212659	0.07808534
cg13498519	CACNA1A;CACNA1A;CA	blue	-0.3445545	-0.6072479	0.32735364	0.10300387	0.48370068	0.00159346
cg20891125	RP11-523O18.1	blue	0.34081779	0.87084088	-0.0668385	0.06007859	-0.6696123	0.03092071
cg13433250	AC009501.4;OTX1;AC00950	blue	-0.4216168	-0.7038451	0.23322485	-0.0504158	0.49142969	-0.0349402
cg15384060	NRXN1;NRXN1;NRXN1;N	blue	-0.4693739	-0.6036686	-0.0086768	-0.1324255	0.28152717	-0.1323966
cg10683965	AC097468.7	blue	-0.4830773	-0.719907	-0.052186	-0.0736553	0.46482347	-0.0341167
cg00569532	AC016670.1	blue	0.58131033	0.72927062	-0.1630967	0.19764685	-0.2696371	0.08423346
cg27112470	ACVRL1;ACVRL1	blue	0.14623043	0.40609698	0.12935344	0.49611507	-0.1069758	0.15183036
cg10921709	TRIOBP;TRIOBP;NOL12	blue	-0.1238236	-0.4660193	0.11197176	0.13855431	0.46237985	0.09855887
cg24722099	CYP27B1;RP11-	blue	-0.2551284	-0.7469943	0.19962967	0.09139963	0.70176958	0.13459408
cg25363151	ST5;ST5;ST5;ST5;ST5;ST5;S	blue	-0.4125535	-0.383218	-0.0605555	-0.0084451	0.1857234	-0.2919756
cg06371836	LINC00261	blue	-0.3837492	-0.5377403	0.15949204	0.04603515	0.43437203	-0.0707469
cg16801277	U7	blue	0.30947881	0.66884492	-0.0999827	0.03127443	-0.4668568	-0.015642
cg14836960	DPPA4;DPPA4;DPPA4;DPP	blue	-0.3159317	-0.4920088	0.19973427	0.055015	0.41344046	0.00250219
cg19606028	U6	blue	0.5410093	0.73532275	-0.1315862	0.17211052	-0.2655623	0.10253332
cg12031211	FNDC5;FNDC5	blue	-0.6074409	-0.6602139	0.11676895	-0.1373242	0.35611466	-0.207596
cg19718360	CD84;CD84;CD84	blue	0.48258126	0.6670009	0.15079518	0.1557488	-0.268162	0.06344417
cg25947945	LAD1;LAD1	blue	-0.3460729	-0.572412	0.04493375	0.01924463	0.38996899	-0.2492912
cg00637340	GRAMD2;GRAMD2	blue	0.26817429	0.71309478	0.03208933	-0.0160847	-0.4985784	-0.0278028

cg04172237	LINC00391	blue	-0.3537654	-0.4276547	0.26326954	-0.0090884	0.22059643	-0.0658191
cg23683020	RP11-521M14.2	blue	0.36055572	0.50575851	0.17292985	0.16754162	-0.2112414	0.00249455
cg15368775	GCK;GCK;GCK	blue	0.66473096	0.66634539	0.37051562	0.27445801	-0.2510335	0.36432105
cg01893414	SATB2;SATB2;SATB2;AC0	blue	-0.4661857	-0.7702104	0.00163492	-0.0226662	0.52125879	-0.1597787
cg12838385	TP53INP1;TP53INP1;RP11-	blue	-0.1979907	-0.5166362	0.06316878	-0.0243187	0.39907666	0.13583928
cg08246857	RP13-497K6.1;PPARGC1A	blue	0.26418946	0.50300015	-0.5099427	-0.0116298	-0.2096296	-0.130243
cg08390696	SLC15A1	blue	-0.2568517	-0.6095903	0.22682807	0.13606525	0.56280483	0.16913121
cg09682185	TMEM130;TMEM130;TME	blue	-0.4629985	-0.5209458	0.13291754	-0.0835487	0.21986125	-0.1195486
cg22928750	AC011484.1	blue	-0.2535908	-0.55212	-0.027595	0.08956859	0.54517576	-0.0264786
cg00281551	RP11-457P14.4;RP11-	blue	-0.2577816	-0.4241556	0.12736825	0.12454402	0.38006955	0.13768114
cg02109484	C2orf65;C2orf65;C2orf65;C2	blue	-0.3435986	-0.6585713	0.03681526	-0.0442919	0.54035812	-0.1438781
cg15892617	CACNA1S;CACNA1S;CAC	blue	0.20546575	0.34457942	-0.1518872	0.03542495	-0.1284023	0.1108865
cg12625731	U4	blue	0.41355014	0.535265	-0.0653684	0.06232935	-0.2309693	0.13435307
cg16668728	RP11-	blue	-0.4088648	-0.6295804	0.14352792	-0.0881794	0.3851991	-0.0585946
cg02009311	MAFG;MAFG	blue	-0.3568983	-0.500251	0.1030481	0.06159522	0.41173587	-0.0794884
cg26486663	SYNGR3;AC005606.14	blue	-0.606894	-0.6817096	0.0331969	-0.0762802	0.42206184	-0.1636009
cg25133016	PIP4K2C;PIP4K2C;PIP4K2	blue	-0.3629975	-0.4601925	0.18301473	0.10747085	0.317783	-0.0098159
cg08537215	CRISPLD2;CRISPLD2;CRIS	blue	-0.2511142	-0.3988885	0.0581028	0.10879557	0.28456342	0.0538675
cg24826102	U1	blue	0.14138078	0.592018	-0.2362947	0.01735922	-0.4192421	-0.1369075
cg14750066	PDZRN4	blue	-0.375802	-0.537751	0.03975441	-0.0148887	0.40954234	-0.0495276
cg03700837	RP11-161M6.4;RP11-	blue	-0.2970736	-0.404047	0.13950705	-0.1215849	0.20157697	0.01068733
cg00080325	RN5S255	blue	0.29316408	0.4076911	0.08100871	0.15705679	-0.0995454	0.06037809
cg05483509	ATP6V0C;ATP6V0C;RP11-	blue	-0.3877273	-0.4341653	0.21606574	-0.1446104	0.12091126	-0.0068681
cg22334352	AC017096.1;AC017096.1;A	blue	-0.4215217	-0.7928644	0.17193593	-0.0214658	0.56253805	-0.0475084
cg23236018	MGLL;MGLL	blue	0.36104794	0.82524467	0.04299657	0.00493592	-0.6313681	0.04485851
cg11679014	TRIOBP;TRIOBP;NOL12	blue	-0.2294376	-0.6248506	0.05037055	0.04037931	0.53231485	0.04468868
cg09473576	CAMK2N1	blue	-0.4163582	-0.6408948	0.09670334	-0.088183	0.39561484	0.01400811
cg21308754	KCNH1-IT1	blue	-0.4166236	-0.7769369	-0.030769	-0.017087	0.51312576	-0.0792076
cg26977737	LINC00320;LINC00320;LIN	blue	0.51586729	0.64986088	-0.1217841	0.21864387	-0.1224805	0.02485543
cg00739593	AFAP1L2;AFAP1L2;AFAP1	blue	-0.3328534	-0.5931583	0.1596397	0.00217305	0.4097152	-0.0166708
cg06582394	CASR;CASR;CASR	blue	-0.3518192	-0.6607166	0.19878092	0.03867653	0.53276798	0.05784099
cg06448090	C8orf48;RP11-145O15.3	blue	-0.4468971	-0.5880155	-0.1149495	-0.1814141	0.31165332	-0.2079761
cg14230397	HOXB-AS5;HOXB-	blue	-0.6270543	-0.7399494	0.0447104	-0.1516805	0.35436126	-0.2358107
cg12928668	SEMA6A;SEMA6A;SEMA6	blue	-0.5460729	-0.7158167	0.12002004	-0.1389138	0.4183045	-0.1262783
cg05063448	ROBO3	blue	-0.5192981	-0.6084761	0.14706282	-0.0790097	0.38009216	-0.1947821

cg22943329	TMEM179;TMEM179;RP11-	blue	-0.3964524	-0.516222	0.11041541	-0.0050419	0.27127523	-0.1128685
cg26998537	OTX2;OTX2;OTX2	blue	-0.3528581	-0.5394188	0.16576986	-0.1115512	0.3140518	0.03337023
cg06840323	ADAMTS17;CTD-	blue	0.4167748	0.72449443	-0.172582	0.19100187	-0.3481625	0.1027333
cg03949978	LRRC14;LRRC14;LRRC14	blue	0.22124755	0.51530021	0.06669463	0.02005532	-0.347733	-0.0488068
cg25374161	RPH3AL;RPH3AL;RPH3AL	blue	0.33241486	0.80681765	0.01064012	0.03842841	-0.6403531	0.05090377
cg19171393	RP4-809F18.1;RP4-809F18.2	blue	0.43957635	0.6334374	0.00200907	0.09459199	-0.2812752	0.07669337
cg11321178	AL450423.1	blue	0.18716895	0.51999818	0.05701106	-0.0230521	-0.2873373	-0.1049542
cg23380552	AC093662.5	blue	0.27891487	0.45448714	-0.1440386	0.04547504	-0.3239595	-0.043638
cg04349727	KLK5	blue	0.29534537	0.62791497	0.00482958	-0.0243479	-0.4935828	-0.0096402
cg02121060	RP11-171A24.3	blue	-0.4843295	-0.58704	0.11072184	0.00244487	0.35066913	-0.0348501
cg06329965	CTC-479C5.6;DUS2L;CTC-	blue	0.48016582	0.60176342	0.16711378	0.24910069	-0.2682165	0.21233062
cg20727114	CPT1C;CPT1C;CPT1C;CPT1	blue	-0.2708499	-0.3605811	0.16192782	0.00063153	0.19277963	-0.0010406
cg20488281	CCDC60;CCDC60;CCDC60;	blue	-0.3250369	-0.5731348	0.10801653	-0.0136065	0.40434956	0.08137547
cg18567703	C6orf70;TCTE3	blue	-0.4060018	-0.4043982	0.12144977	0.05023226	0.05906943	0.02572417
cg09035732	RP11-325D8.1;RP11-	blue	0.28897627	0.73256655	-0.209497	0.01411557	-0.4443108	-0.0548556
cg00697440	CD86;CD86;CD86;CD86;CD	blue	0.2879336	0.83977742	-0.0116118	-0.037032	-0.7010475	-0.0060226
cg18371700	CLIC6	blue	-0.4226362	-0.5608949	0.00074635	-0.0277154	0.34317007	-0.1368702
cg22598841	LRIG2;RP11-31F15.2	blue	-0.4125404	-0.4618711	0.30249026	0.00477695	0.15751143	0.06008569
cg22485693	BVES-	blue	-0.1974268	-0.5761026	0.19177444	0.07709508	0.47223234	0.18658666
cg05283629	LTK;LTK;LTK;LTK;LTK	blue	-0.1675492	-0.6318112	0.22044052	0.09575914	0.59540857	0.15813692
cg04380297	TBPL2;TBPL2	blue	-0.3773969	-0.391579	0.04584009	0.00743448	0.25675138	-0.0168826
cg14658867	TBC1D20;TBC1D20;TBC1D	blue	-0.3379445	-0.4295543	0.03797616	0.17677036	0.32450177	-0.0408844
cg21402071	CHRNB4	blue	-0.3844986	-0.4869446	0.11350572	0.018717	0.41408837	-0.170386
cg12274030	CTD-3135A9.3	blue	0.27168267	0.3983171	0.03819966	0.0535865	-0.1574263	0.01541154
cg13912545	EYA4;EYA4;EYA4;EYA4;E	blue	-0.2807324	-0.6630865	0.16142604	0.01822677	0.55985037	0.06942212
cg12875538	FAM13C;FAM13C;FAM13	blue	-0.3178611	-0.441145	0.16232673	0.06373973	0.35013657	-0.0434393
cg27586487	DLG5;DLG5;RP11-	blue	-0.3624995	-0.5189336	0.08048095	0.02010616	0.33686899	-0.1011384
cg22976884	ZIC5;ZIC5	blue	-0.5216597	-0.6801678	0.02918828	-0.0794518	0.4070884	-0.159618
cg18334518	RP11-	blue	0.31689541	0.67640531	0.2463259	0.07718821	-0.4231432	0.05328809
cg18981979	MIR187	blue	0.36310802	0.41059702	0.19002786	0.06683441	-0.212332	0.14834393
cg24500711	KRTAP13-3;KRTAP13-3	blue	0.44116735	0.63096467	-0.1794616	0.12766113	-0.2396265	0.02410753
cg08114317	C1orf222;C1orf222	blue	-0.3901987	-0.5809513	0.25542262	0.1173889	0.48976077	-0.0571947
cg12788982	IGSF22;RP11-	blue	-0.3239531	-0.5884729	0.2096182	0.02557302	0.42704968	-0.0545344
cg25083792	RP11-10N16.3;RP11-	blue	-0.2648494	-0.5698645	0.14529111	0.02601932	0.57409442	-0.0055976
cg21543102	SPRR3;SPRR3;SPRR3;SPR	blue	0.37048908	0.54226267	-0.0414739	0.04878586	-0.3241903	0.0168722

cg13044077	MAP6;MAP6;MAP6;MAP6	blue	-0.3302477	-0.4286748	0.09958933	0.02332643	0.22540914	-0.0751866
cg07181374	PLEKHG5;PLEKHG5;PLEK	blue	-0.3686744	-0.4736081	0.1376549	0.09134573	0.44778558	-0.0687641
cg11413039	DDX25;PUS3;DDX25;DDX	blue	-0.4713595	-0.8297308	-0.0238351	-0.0609054	0.55704109	-0.1457767
cg18158391	VSNL1;VSNL1;VSNL1;VS	blue	-0.4206178	-0.7342683	0.17490773	-0.0142862	0.51605352	-0.0285559
cg04636876	PRND	blue	0.36432044	0.4104998	-0.3097847	0.27664415	0.03312586	-0.0196603
cg08415964	U6;DSG1	blue	0.41585272	0.50957832	-0.2093507	0.13799257	-0.0331863	0.03758084
cg19993819	NKX6-3;NKX6-3;NKX6-3	blue	0.40533438	0.85683738	0.01007389	0.00079644	-0.6018751	0.01700135
cg05541765	PPP2R3A;PPP2R3A	blue	-0.2917369	-0.562176	0.17282551	0.002869	0.40807685	0.12926829
cg14057562	7SK	blue	0.25408275	0.75670896	-0.2707451	-0.0431128	-0.5541912	-0.1040631
cg11491961	ASZ1;ASZ1;ASZ1;ASZ1;AS	blue	0.55517657	0.61348254	0.15280343	0.26020089	-0.151254	0.25312802
cg04827692	RP11-	blue	-0.3846077	-0.3920128	0.12693187	0.06163139	0.16022091	-0.0431819
cg22334059	RUNX1T1;RUNX1T1;RUN	blue	0.68883342	0.69039984	0.23470308	0.27499904	-0.2019813	0.28854595
cg03476195	ANK2;ANK2;ANK2;ANK2;	blue	-0.3290041	-0.693771	0.24216567	0.07295254	0.50172022	0.09796734
cg18691434	STAG3;STAG3;GPC2;GPC2	blue	-0.302354	-0.5112229	0.26858164	-0.0098733	0.2624202	0.13198122
cg14631333	AP5Z1;AP5Z1;AP5Z1	blue	0.31946126	0.39124184	0.0384625	0.03836537	-0.1368515	0.21275084
cg20909145	GPR39	blue	0.43149757	0.71865478	-0.0021638	0.0748261	-0.3150206	-0.0046032
cg19571412	RP11-	blue	-0.3227829	-0.5459988	0.16505648	0.05280681	0.37313475	-0.0245231
cg23847109	COL9A2;COL9A2;COL9A2;	blue	-0.284179	-0.6106608	0.06586839	-0.0609328	0.36206784	-0.0221984
cg17588800	GLI3;GLI3;GLI3	blue	-0.3255303	-0.5047725	0.11747686	0.09899939	0.36622642	0.01067666
cg19902824	U6	blue	0.39875414	0.61038717	0.1940966	0.12039542	-0.3139022	0.05062518
cg20365170	MYH7;MYH7	blue	0.23207462	0.74387746	0.15567514	-0.0367342	-0.5762168	-0.063012
cg03822704	FBXL7	blue	-0.3821117	-0.6404518	0.26062425	-0.0030981	0.50084686	0.03070175
cg21392341	TBX15;TBX15;TBX15	blue	-0.480148	-0.7049123	0.07694636	-0.0745571	0.44593669	-0.1896056
cg23727072	CYP1A1;CYP1A1;CYP1A1;	blue	-0.3973572	-0.5634601	0.0542601	0.0952114	0.41567717	-0.0458491
cg17344694	LURAP1;POMGNT1;POMG	blue	-0.3651363	-0.6323786	0.24671634	0.09968895	0.54230929	-0.0054757
cg27320127	KCNK12;KCNK12	blue	-0.4342635	-0.7660571	0.16965372	-0.0397378	0.47839635	-0.0243142
cg02285673	RP11-198M11.2;RP11-	blue	0.44567845	0.7311376	-0.1246507	0.10153678	-0.3274063	0.1341563
cg03755993	CES4A;CES4A;CES4A;CES	blue	-0.3654939	-0.5674727	0.17017862	-0.0013211	0.5006667	-0.0993213
cg08159945	HDAC10;HDAC10;HDAC1	blue	-0.0272402	0.29172765	0.39795918	0.19615877	-0.2226749	0.12304208
cg18268547	TSPAN10;NPLOC4;TSPAN	blue	-0.1376851	-0.5888349	0.36621923	0.14022975	0.55980301	0.23034119
cg11662340	NOTCH4;NOTCH4	blue	0.3004652	0.59164751	0.23644189	-0.0655334	-0.3915906	0.13722069
cg01386275	POU4F3;RBM27;CTC-	blue	-0.4239143	-0.4771929	0.10264006	-0.1081539	0.25603344	-0.1526971
cg09470625	RP11-519M16.1;RP11-	blue	0.36455922	0.57630926	-0.1526691	0.18427579	-0.1172385	-0.1214006
cg11752380	snoU13	blue	0.20681173	0.76484613	-0.0970091	-0.0321072	-0.6295464	-0.1291038
cg19107595	SGCE;PEG10;SGCE;SGCE;	blue	-0.1715828	-0.3359702	0.3976555	0.0594098	0.1699645	0.21335594

cg08319756	SNTG2;SNTG2	blue	0.14691235	0.35100224	-0.1034291	-0.0280076	-0.2139104	-0.0170284
cg19820372	LAYN;LAYN;LAYN;LAYN	blue	-0.3432483	-0.7068526	0.15952788	0.01637005	0.63432589	-0.0089996
cg07586017	CTC-338M12.6;CTC-	blue	0.21551113	0.38061536	-0.1469741	0.07513544	-0.2094284	-0.0038508
cg06234182	AP1B1P1	blue	0.27047832	0.74687898	-0.0749612	0.05814768	-0.4984614	-0.189397
cg10109215	C20orf160	blue	-0.3082499	-0.4327716	0.13473506	0.04110991	0.32951843	0.00033307
cg20317866	BVES-	blue	-0.4340099	-0.6241672	0.21012806	0.04754732	0.44792125	-0.0858491
cg14666150	RP11-30G8.1	blue	0.48562778	0.74587311	-0.1738894	0.22574355	-0.261305	0.028449
cg05720939	RP11-669N7.3	blue	0.57130751	0.65051057	0.16551501	0.3069102	-0.1420429	0.19993504
cg24902920	TFAP2A;TFAP2A;TFAP2A;	blue	-0.2670259	-0.6001623	0.29450672	0.10152124	0.50304503	0.08604466
cg24516953	RP11-143O10.1	blue	0.25763285	0.64124674	0.24197766	0.07139247	-0.4440214	-0.0889155
cg15006404	HOTTIP	blue	-0.3375297	-0.5451141	0.15008845	0.01725351	0.38297869	0.01455994
cg09585725	RP11-14D22.2;RP11-	blue	-0.3947034	-0.8104281	-0.0194111	-0.0829621	0.57795649	-0.076355
cg21604325	MTUS2-AS1;MTUS2	blue	0.27559385	0.48574551	0.12964037	0.00555517	-0.3095599	-0.0483846
cg20309121	RUNX1T1;RUNX1T1;RUN	blue	-0.35928	-0.5541583	0.25659093	0.02457115	0.3624031	-0.0173852
cg05966923	RP11-	blue	0.38852815	0.40191317	0.09423336	0.16595515	-0.0300401	0.06712656
cg01285501	RP1-170O19.21;RP1-	blue	-0.3054595	-0.729228	0.10376016	0.01640152	0.64536658	0.07957565
cg21296676	EYA4;EYA4;EYA4;EYA4;E	blue	-0.3204959	-0.5809065	0.22378551	-0.0205929	0.45820451	0.00075928
cg16213655	PPL	blue	-0.6083739	-0.6955339	-0.0185976	-0.1107159	0.39958076	-0.2016678
cg21922319	OSGIN1;RP11-	blue	0.46621858	0.63537194	0.20047541	0.12364791	-0.323451	0.27172939
cg09946496	ZNF771;ZNF771;ZNF771;Z	blue	-0.3061702	-0.4123249	-0.0463737	0.08200218	0.28896038	-0.0416324
cg22287738	HSD17B1P1	blue	0.17541009	0.32072649	0.1931826	0.25310902	-0.0547472	0.41043904
cg11204099	ETNK1;ETNK1;ETNK1;ETN	blue	-0.4408083	-0.7386182	0.20320741	0.02605701	0.59668283	-0.0180856
cg00348858	TUFM;TUFM;SH2B1;SH2B	blue	-0.497186	-0.5214732	0.12362018	0.01300898	0.33787359	-0.1316097
cg05492442	MAPK4;MAPK4	blue	-0.4642755	-0.7547987	0.02782985	-0.0708203	0.56889117	-0.0798368
cg04735310	HOXB7;RP11-	blue	-0.4693383	-0.5281429	0.16351598	-0.0494995	0.22293573	-0.1008077
cg05824757	SGPL1;SGPL1	blue	0.39495707	0.41132041	-0.0041555	0.19836595	-0.0588222	0.28679214
cg14780183	CLUL1;CLUL1	blue	0.38609111	0.39293809	-0.1919342	0.16317352	-0.065214	0.04383493
cg21165089	C11orf85;C11orf85;C11orf85	blue	-0.2891836	-0.6685866	0.1099231	0.0704985	0.55164308	0.10616972
cg16158289	RP11-502H18.2	blue	0.24927433	0.61681943	0.08712662	-0.0374348	-0.4846125	0.02406678
cg21231944	PPFIA2;PPFIA2;PPFIA2;PP	blue	-0.4170617	-0.5479612	0.11420792	0.05457562	0.40655901	0.00613184
cg02274336	OR2H1;OR2H1;OR2H1;OR2	blue	0.44096524	0.72205896	-0.1119971	0.17375554	-0.3041623	-0.0532728
cg24915511	IER5;IER5	blue	-0.4782672	-0.7739916	0.06310511	0.01218874	0.56358793	-0.1176034
cg24203315	CCND1;CCND1;CCND1;CC	blue	-0.4060094	-0.5013948	0.24480414	-0.0324695	0.29836964	-0.089571
cg25306915	AC012215.1;UNC5D;UNC5	blue	0.15573839	0.5904646	-0.130405	-0.0281944	-0.399495	-0.0563561
cg03637976	RGL2;RGL2;RGL2;RGL2;R	blue	-0.3340937	-0.4721798	0.20703935	0.03124862	0.33052172	0.03761423

cg13614181	RGCC	blue	-0.4103754	-0.5218232	0.18207475	0.08629503	0.41001985	-0.1006701
cg25215292	FBXL21;FBXL21;FBXL21	blue	-0.3548017	-0.4125588	-0.0583005	0.05340946	0.25678022	-0.0296594
cg23222898	GUCA2B	blue	0.51869264	0.56416195	-0.0432615	0.17859246	-0.0901482	0.07394186
cg07394719	C10orf90	blue	-0.1643224	-0.6956792	0.03939236	0.09550658	0.62311349	0.17670281
cg22373112	RAB5C;RAB5C;RAB5C;RA	blue	-0.229493	-0.4426365	0.22611546	0.07843001	0.40313909	0.02101969
cg22909050	RP11-366M4.3;RP11-	blue	0.32199608	0.75675085	-0.1477797	0.07780109	-0.4746568	-0.0872417
cg13920367	CDH1;CDH1;CDH1;CDH1;	blue	-0.2739198	-0.6135658	0.20758015	0.03595823	0.48251184	0.12124905
cg11610350	PRICKLE2	blue	-0.3787961	-0.6814337	0.10328125	-0.0637888	0.47291251	-0.1275038
cg16409367	MEIS2;MEIS2;MEIS2	blue	-0.5227653	-0.7619154	0.11983932	-0.0845063	0.45233271	-0.1081988
cg11490276	ALDH3B2;ALDH3B2;ALD	blue	0.39173483	0.72913548	0.17749572	0.04559114	-0.4828104	0.14154482
cg18877284	HPCAL4;PPIE	blue	-0.2132642	-0.5627237	0.12169727	0.10641745	0.50457197	0.06167236
cg24197374	PCDHGA11;PCDHGA11	blue	-0.2003726	-0.4106713	0.11998565	0.06243192	0.3330546	0.12828596
cg20650602	RP11-356N1.2;RP11-	blue	0.34920646	0.68294652	-0.203673	0.16705724	-0.2954872	-0.1468413
cg11400613	TYK2;TYK2;TYK2;TYK2;T	blue	-0.4312948	-0.5897311	0.06572213	-0.1558848	0.33900372	-0.0369323
cg07392884	LAMA4	blue	0.36779885	0.3969795	-0.3285999	0.12238413	0.0213379	0.06647317
cg05074554	CTD-2089N3.1	blue	-0.3654338	-0.4839765	0.13273586	-0.0403045	0.19359983	0.07077873
cg15211996	ACTRT2;ACTRT2	blue	0.16435416	0.69038669	0.14838092	0.02762541	-0.6241837	-0.0166266
cg07555832	LINC00545	blue	0.18417011	0.7112376	-0.136507	-0.0803302	-0.5914066	-0.0592412
cg26734175	RP11-62I21.1	blue	0.15406582	0.33776401	0.15896088	0.04065041	-0.1725109	-0.0395879
cg27102941	TTC23;TTC23;TTC23;RP11-	blue	0.2270441	0.79806011	-0.0466916	-0.0403358	-0.6584496	-0.0693158
cg09969806	KIF1A;KIF1A;KIF1A;KIF1	blue	-0.453585	-0.6550028	0.10541582	-0.0471378	0.49187718	-0.05089
cg00744739	PRR21	blue	0.32737403	0.42083237	0.03371832	0.11356115	-0.1901743	0.18533145
cg16667279	ELAVL3	blue	-0.3443078	-0.633589	0.1251434	0.01110081	0.48379244	0.00422469
cg14218343	ZNF32;ZNF32;ZNF32;ZNF3	blue	-0.3507211	-0.806716	0.13088407	0.02150362	0.68043936	-0.0461087
cg16933388	BSN-AS2;BSN-AS2	blue	-0.444731	-0.7872014	-0.0546744	0.00927022	0.59122106	-0.044843
cg24340786	LHX9;LHX9	blue	-0.3439084	-0.437675	0.16345356	-0.0181442	0.23497142	-0.0998857
cg06814612	OSBPL9;OSBPL9;OSBPL9;	blue	0.34198872	0.35886426	-0.3646207	0.03961337	-0.1005071	0.04631117
cg24291500	REST;REST	blue	-0.2565375	-0.439396	0.04726736	-0.0072103	0.33765531	-0.0913139
cg24495344	COL11A1;COL11A1;COL11	blue	-0.5430828	-0.6854292	0.05805867	-0.1544093	0.40694851	-0.160764
cg14678188	RP11-	blue	0.4319664	0.74033107	0.03269661	0.05435334	-0.3639586	0.06927352
cg10945180	RP11-	blue	0.49802139	0.75482887	-0.0991903	0.22229647	-0.2616986	0.02788128
cg12946225	HMG20B;HMG20B;HMG20	blue	-0.5461876	-0.6296904	0.07479745	-0.1232909	0.31760564	-0.1365003
cg03433260	BID;BID;BID;BID;BID;BID;	blue	-0.4560273	-0.6852347	-0.0667009	-0.0043943	0.54121511	-0.2335113
cg26552743	RP11-142C4.6;RP11-	blue	0.41490846	0.49751569	-0.083214	0.03495571	-0.3684492	0.14646971
cg24405702	AC011497.1	blue	-0.4400081	-0.6691529	0.15018827	-0.0620358	0.47290712	-0.0342819

cg18802946	SNORD115;SNHG14;SNHG	blue	0.26819963	0.45465181	0.22203102	-0.008032	-0.278672	0.05472204
cg05778559	DAZL	blue	0.3975199	0.40554258	-0.1797792	0.04302912	-0.1484434	0.07426309
cg23824762	NEURL3;NEURL3	blue	0.29568435	0.64509525	0.0439541	0.00118971	-0.3051075	0.08910684
cg22289810	NF1;NF1	blue	-0.3481484	-0.4714828	0.24514634	-0.0181268	0.20240293	-0.0194872
cg07126690	HOXB-AS4;HOXB-	blue	-0.3260605	-0.6090952	0.14950688	0.01688962	0.4061465	-0.0330483
cg15716277	TAF6L;TAF6L;TAF6L;TAF6	blue	-0.2223368	-0.3643574	0.24368914	0.14955746	0.3089022	0.04308198
cg13688573	FAM82A1	blue	0.35792067	0.49409404	-0.3316305	0.0106258	-0.2064221	0.03470611
cg17704189	FEZF1;FEZF1;FEZF1;RP11-	blue	-0.2094035	-0.6766242	0.19750378	0.0965408	0.63435146	0.14514653
cg12559208	TMC4;TMC4;TMC4;TMC4;	blue	-0.3168619	-0.3960909	0.09814195	-0.1440323	0.24228252	0.00422265
cg06550344	AC005370.1;FGF1;FGF1;FG	blue	0.24484648	0.39149156	-0.1410641	0.04291592	-0.1816732	-0.14198
cg19928195	KALRN;KALRN;KALRN;K	blue	0.26675395	0.83064324	0.12892112	-0.0266978	-0.7256503	-0.0140904
cg02743650	IGSF22;IGSF22;IGSF22;RP	blue	-0.2130195	-0.2965374	0.10720126	-0.0178237	0.15955499	0.19612145
cg06188744	PFKP;PFKP;PFKP	blue	-0.2370188	-0.6162882	0.18925004	0.1078386	0.54243736	0.15488781
cg02351971	FSTL1;FSTL1;FSTL1	blue	0.43911113	0.6376866	-0.0651387	0.14462057	-0.2413058	0.13250889
cg15028756	PEX5;RP11-273B20.3;RP11-	blue	-0.2904578	-0.5603696	0.18753205	0.1784555	0.52111578	0.00120418
cg06263843	CPEB1;CPEB1;CPEB1;CPE	blue	-0.3150841	-0.4649539	0.07525783	0.02288004	0.39595593	-0.0143256
cg13060334	LYSMD4;LYSMD4;LYSM	blue	-0.3182867	-0.375968	0.06357367	-0.0196097	0.25433375	-0.0150793
cg15823859	MIR9-	blue	-0.5123313	-0.7372989	0.15390222	-0.0820713	0.38124699	-0.1544131
cg24035447	PTCHD4;PTCHD4;PTCHD4	blue	-0.3655041	-0.7121731	0.13365296	0.02946524	0.46673735	0.05676108
cg19123339	ABCG5;ABCG5;ABCG5;AB	blue	0.21828722	0.56994409	-0.0883742	-0.1185612	-0.4730969	-0.0401015
cg07556744	RP11-1102P16.1;RP11-	blue	-0.3475586	-0.6680385	0.23954397	0.1076196	0.54697104	0.07457216
cg13062913	NCAM2;NCAM2	blue	-0.3542341	-0.6932862	0.15796881	0.13886533	0.56775256	0.04322661
cg17237086	MKL1;MKL1	blue	-0.2552532	-0.418259	0.418136	0.19512816	0.1502963	0.01746678
cg19227170	WFIKKN1;WFIKKN1	blue	-0.4660083	-0.7059478	0.03661983	-0.0348446	0.47790002	-0.1299636
cg23959605	SYT2	blue	-0.4373687	-0.4471545	0.13834802	-0.1858938	0.25089865	-0.2179056
cg12239218	LRP1	blue	0.63235473	0.57705887	0.31394824	0.23421021	-0.1597352	0.27812544
cg06109275	CRCT1	blue	0.35104811	0.34829666	0.1715599	0.18326234	-0.1363998	-0.0578294
cg06360543	LRRC71;LRRC71	blue	-0.3234337	-0.4817986	0.06701537	0.08881888	0.36364518	-0.1144582
cg01942530	CYP11B1;CYP11B1;CYP11	blue	0.26730033	0.57786315	-0.0123502	0.04919616	-0.3458631	0.0036364
cg19736608	CORIN;CORIN;CORIN;CO	blue	-0.3253714	-0.7556152	0.28137983	-0.0016169	0.57926525	0.0823049
cg24688348	OR5B21	blue	0.53347319	0.77246846	0.00837	0.18349479	-0.2753634	0.1414946
cg02248826	TJP1;TJP1;RP11-	blue	-0.3690637	-0.7391921	0.18597623	0.01667156	0.52718876	0.03009337
cg04920044	RP11-375D13.4;RP11-	blue	0.48871653	0.70171967	-0.0969302	0.1260537	-0.2020748	0.04999159
cg20615848	C18orf34;C18orf34;C18orf34	blue	-0.2957932	-0.6104938	0.19471051	0.01675711	0.47835117	0.21188723
cg18056600	ZMYND15;ZMYND15;CXC	blue	-0.3483274	-0.5357303	0.18262854	0.06146103	0.25473365	-0.0382316

cg06558069	SSTR1	blue	-0.3706195	-0.5367707	0.07301016	-0.0398039	0.31550529	-0.0341887
cg24214068	NEFM;NEFM;NEFM;NEFM	blue	-0.4225856	-0.6819445	0.09012549	-0.1097091	0.39157791	0.03664164
cg23399662	CTA-796E4.4	blue	0.34315859	0.44837864	-0.1248035	0.22822299	0.02256085	0.13917173
cg05932139	WFIKKN1;WFIKKN1	blue	-0.3888293	-0.3960578	0.11732465	-0.0429126	0.24122192	-0.1609803
cg06259248	C1orf51;C1orf51;C1orf51;C1	blue	-0.3195615	-0.6583642	0.20563798	0.04160928	0.53809385	-0.0597374
cg19296641	TFAP2A;TFAP2A;TFAP2A;	blue	-0.3996231	-0.7663055	0.12271285	0.00594662	0.54838164	0.01443751
cg01644927	CNR1	blue	-0.466508	-0.6310799	0.07572425	-0.1022313	0.31030151	-0.1254961
cg04840290	NODAL	blue	-0.29451	-0.4779141	0.03234542	0.09440942	0.35216247	0.09726984
cg18218371	RIMS2;RIMS2;RIMS2;RP11-	blue	-0.3881372	-0.796775	0.06838345	-0.024862	0.58409147	-0.0004112
cg19014036	CECR5	blue	0.05310566	0.29193936	0.27550844	0.16830759	-0.0214134	0.15560641
cg05438461	BMF;BMF;BMF;BMF;BMF	blue	-0.4649202	-0.4229268	0.22365675	-0.0786817	0.0995127	-0.1330765
cg14023149	RPL38;RPL38;RPL38;RPL38	blue	-0.3036449	-0.5327522	0.25485735	0.07291339	0.44647507	0.1091899
cg08949164	BAIAP3;BAIAP3;BAIAP3;B	blue	-0.2930867	-0.6133922	-0.0008956	-0.011891	0.46245853	0.02283493
cg24077102	C11orf85;C11orf85;C11orf85	blue	-0.3727226	-0.7293176	0.15813003	0.0333023	0.548597	-0.011469
cg02126051	ZIC4;ZIC4;ZIC1	blue	-0.3510486	-0.6515988	0.26210298	-0.0033355	0.41279854	0.03271134
cg07665387	RP11-9G1.3;PCDH10;RP11-	blue	-0.3164447	-0.7851075	0.0847989	0.0089185	0.65710386	-0.026554
cg15594205	AC018730.1;POU3F3	blue	-0.4416948	-0.4701815	0.15717729	0.00557235	0.11692628	-0.0733201
cg15480367	CHGA;CHGA;CHGA;CHG	blue	-0.3945552	-0.7510334	0.14542962	-0.0205326	0.48744428	0.02076643
cg04598121	RP11-	blue	-0.3465041	-0.8049442	0.11045012	-0.0154135	0.60339575	0.06811398
cg08781526	RP11-	blue	-0.3677349	-0.3717011	0.23808025	-0.0023664	0.29948887	-0.0464723
cg12273483	RP11-217C7.1	blue	0.19782228	0.66670996	-0.0333758	-0.0792046	-0.5506599	-0.0002579
cg13361779	AC008806.2;ZNF570;ZNF56	blue	-0.3385093	-0.4804994	0.07332658	0.07103885	0.36334397	-0.3146383
cg02254574	ASIC2	blue	-0.2648498	-0.483595	0.00708323	0.15576866	0.41347512	0.00206219
cg17287462	PPA2;PPA2;PPA2;PPA2;PP	blue	-0.1739215	-0.37631	0.02599947	-0.0051578	0.31807967	0.09753652
cg18902090	PCDHAC1;PCDHAC1	blue	-0.4599371	-0.7759127	-0.0763095	-0.1887083	0.46878401	-0.1140484
cg11800620	ESRRG;ESRRG	blue	-0.2431637	-0.614399	0.02435026	-0.0320122	0.49706054	0.07065618
cg14894216	CATSPER1	blue	0.22122589	0.35782662	-0.0781036	0.18259703	-0.0872329	-0.0838483
cg13057857	LRRC7;LRRC7	blue	-0.37798	-0.5034464	0.19959393	0.02089097	0.21850819	0.01922354
cg03077453	ZNF528;ZNF528;ZNF528;Z	blue	-0.2721815	-0.5789615	0.14799285	-0.0639232	0.40123508	0.06588081
cg02257405	AC034228.2;ACSL6;ACSL6;	blue	-0.0962119	-0.3774854	0.42623021	0.20247446	0.33446559	0.21886153
cg02611848	C2orf65;C2orf65;C2orf65;C2	blue	-0.2722163	-0.5737775	-0.0309648	-0.0716301	0.4656747	-0.1204532
cg21784383	ESRRG;ESRRG	blue	-0.3115868	-0.7934604	0.11946778	0.02995787	0.67421669	0.01226796
cg21390624	EYA4;EYA4;EYA4;EYA4;E	blue	-0.3728535	-0.6905776	0.17712696	-0.0723099	0.52018365	-0.1102883
cg03722243	CALCR;CALCR	blue	-0.2568616	-0.4707809	0.2702437	0.01243429	0.3186784	0.1482766
cg16735881	NBEA;NBEA	blue	-0.4132531	-0.7751574	0.1265725	-0.0227914	0.51294737	0.0197728

cg25112312	ZNF518B;ZNF518B;ZNF518	blue	-0.4670632	-0.5763011	0.13182769	-0.0871468	0.29826177	-0.1202629
cg14057221	RP5-1100I6.2	blue	0.43876136	0.49040566	-0.1346459	0.26386809	-0.0371092	0.18320998
cg02268171	C1QTNF9B	blue	-0.3924591	-0.6976196	0.06141899	0.04165051	0.53065017	-0.049997
cg18240400	ZFAND4;ZFAND4;ZFAND	blue	-0.1503315	-0.4811637	0.11428916	0.08797321	0.40583449	0.08161629
cg06953874	HOXD9;HOXD9;HOXD-	blue	-0.4688548	-0.6976495	0.0784087	-0.0554013	0.42276471	-0.1105327
cg19519747	MIR10B;HOXD3;HOXD4;H	blue	-0.3569792	-0.3778679	0.10140521	-0.0337412	0.14320671	-0.0559895
cg19613722	DCC	blue	-0.1853674	-0.7083959	0.17902021	0.14369566	0.62419093	0.19549766
cg12087695	SERPINA10;SERPINA10;SE	blue	-0.3488153	-0.5621504	0.22230624	0.096268	0.51139532	-0.04116
cg03330058	ABTB1;ABTB1;ABTB1;AB	blue	-0.2293442	-0.3809254	0.15541131	0.02388493	0.353858	0.03959162
cg05250467	RP11-32K4.2	blue	-0.2766724	-0.5061102	0.20194976	0.02356353	0.3168107	0.00568869
cg05250458	ZNF177;ZNF177;ZNF177	blue	-0.3024031	-0.4371454	0.34261899	-0.0298138	0.30307354	-0.0306029
cg05652569	DQX1;DQX1;DQX1	blue	0.04881806	0.46309524	-0.0548665	-0.0067407	-0.3294114	-0.1522251
cg27642744	MMEL1;MMEL1;MMEL1;M	blue	0.3549574	0.53107806	0.2002008	0.16578028	-0.1932012	0.13191998
cg24225668	SLC22A2;SLC22A2;SLC22	blue	0.24985515	0.49845429	0.09320188	0.04797292	-0.2997292	0.0159469
cg10160276	TOX3	blue	-0.385725	-0.5831171	0.19679097	-0.0739092	0.37343861	-0.0213645
cg02298570	ZNF555	blue	-0.2371258	-0.35537	0.1954597	0.09451508	0.33757838	-0.0129398
cg10241132	RP11-116G8.5;RP11-	blue	0.09911037	0.31777987	-0.1591818	-0.0652351	-0.2798631	-0.1144213
cg02086431	RBP3	blue	0.4805078	0.61188478	-0.1826308	0.1451219	-0.1038887	-0.0366386
cg25004163	AC055873.1	blue	0.21377958	0.60601517	0.03349301	0.12875991	-0.3248824	-0.089891
cg14643892	RPS11P6;RPS11P6;RPS11P	blue	-0.3101173	-0.4466952	-0.0452477	0.04502751	0.40278222	-0.2252325
cg06752633	PLD4;PLD4;PLD4;PLD4	blue	0.3343965	0.77859831	0.13457084	0.04138875	-0.589273	0.10994465
cg12479313	RP11-537P22.2;SLC25A21	blue	0.38492261	0.70707478	-0.2161471	0.10771037	-0.3861213	-0.0217498
cg25357666	ADAMTS17;CTD-	blue	0.33289932	0.38759359	0.22384927	0.17187144	-0.1831698	0.38634244
cg12242077	PABPN1L;PABPN1L;PABP	blue	0.34073761	0.6921494	0.14077083	-0.0034857	-0.4956406	0.27217115
cg08799997	TTLL2;TTLL2;TTLL2;TTLL2	blue	0.41378249	0.55418475	-0.0805343	0.26030606	-0.1755895	0.07159505
cg03813688	TLX2;TLX2;TLX2;TLX2	blue	-0.2666046	-0.4171529	0.25753594	0.10075409	0.27420587	0.06846749
cg14918958	ANK2;ANK2;ANK2	blue	0.45903206	0.50412361	0.05925834	0.22293476	-0.1073474	0.08722037
cg04937184	BMP4;BMP4;BMP4	blue	-0.3273685	-0.6477677	0.0200353	-0.0264978	0.51549755	-0.2362526
cg14800603	GRM6	blue	0.31080416	0.4785329	-0.0498765	0.06076242	-0.1821973	0.13230452
cg08118241	NKAIN3;NKAIN3;NKAIN3	blue	-0.3822232	-0.4433651	0.12913817	-0.0375864	0.24501926	-0.0794442
cg00181125	ABCC9;ABCC9	blue	-0.4073689	-0.6877958	0.15576931	0.03845438	0.48835032	0.00139618
cg18074297	CLIC6	blue	-0.4284029	-0.6500777	-0.0980537	-0.1056913	0.41549435	-0.0950589
cg11583762	ADAM5P;ADAM5P;ADAM	blue	-0.1681914	-0.3568166	0.23003523	0.03286687	0.34117602	0.10458734
cg10768150	CTC-461F20.1;RP11-	blue	-0.4155824	-0.3888898	0.35901882	-0.0633131	0.04145781	-0.0615873
cg14053123	AC096559.1	blue	0.38134544	0.4175685	0.00356368	0.16441813	-0.0456146	0.07192954

cg14514569	RP11-120J1.1;RP11-120J1.1	blue	-0.3328374	-0.630508	0.15750266	0.05950986	0.47154994	-0.0827005
cg25249144	RN5S185	blue	0.57376335	0.62853672	-0.1721082	0.28535667	-0.158347	0.19246281
cg20518045	SPOCK1;SPOCK1	blue	0.17767858	0.41052754	-0.2105628	-0.0168852	-0.1796657	-0.0239115
cg15140117	USP36;USP36;USP36	blue	0.13564672	0.36151723	0.2272361	0.23119618	-0.0835713	0.11603808
cg01288114	KCNK10;KCNK10	blue	-0.3239055	-0.4621757	0.00198578	0.02269264	0.34870668	-0.0080598
cg03360992	TRIOBP;TRIOBP;NOL12	blue	-0.0350727	-0.224926	0.17642475	0.04682108	0.17920126	0.19938253
cg15225201	NLGN1;NLGN1;NLGN1;NL	blue	-0.3581958	-0.690766	0.21015698	-0.0716014	0.47510459	0.06229281
cg06300254	CHAT;CHAT;CHAT;CHAT;	blue	-0.392138	-0.4622025	0.04689033	0.04689635	0.25731607	-0.0878635
cg07808023	LINGO1;LINGO1;LINGO1;	blue	-0.3361016	-0.4002385	0.20640189	0.03209262	0.27500899	-0.0268996
cg10508025	MAP7;MAP7	blue	-0.3055136	-0.6064648	0.03221432	0.07567134	0.48217539	-0.0694668
cg22472306	ATP6V1B2;ATP6V1B2;ATP	blue	-0.3390281	-0.5024809	0.15672894	-0.0260738	0.35855925	-0.0949359
cg23197087	RP1-	blue	-0.3907004	-0.7513696	0.18261734	0.0772521	0.53245374	-0.0010457
cg24021113	PXDC1;PXDC1;PXDC1	blue	-0.2965791	-0.4859984	0.07662687	0.0869312	0.43235289	-0.0927605
cg14979826	ALDH1L1;ALDH1L1;ALDH	blue	0.13057611	0.32907423	0.2771286	0.12274578	-0.1701763	-0.0422159
cg04536296	TNFSF11;TNFSF11;TNFSF	blue	-0.268536	-0.6170805	0.22267061	0.0598283	0.46761395	0.02080005
cg11313136	RALBP1;RALBP1	blue	-0.3904197	-0.7367239	0.17819487	0.01849304	0.49989272	-0.0543139
cg13603171	MOXD1;MOXD1	blue	-0.3090323	-0.5618435	0.17349533	-0.0087392	0.44898114	-0.0610859
cg17344384	GGA1;GGA1;GGA1;GGA1;	blue	-0.4152017	-0.4090665	0.27142682	-0.0023868	0.18799967	-0.118499
cg14612428	GNAL;GNAL;GNAL;GNAL	blue	-0.3301107	-0.790494	0.11446281	0.02234589	0.62275934	0.04083328
cg00069242	RP11-	blue	-0.366267	-0.6763682	0.14615625	-0.0513477	0.41715191	-0.1030316
cg04354589	NPM1;NPM1;NPM1;NPM1;	blue	-0.242756	-0.3438835	0.14677519	0.07647181	0.30314748	0.08161329
cg03299487	DBX1;DBX1	blue	-0.3230117	-0.5368678	0.12992668	0.04423103	0.3362811	-0.0098379
cg19428336	FAT1;FAT1;FAT1	blue	-0.3411968	-0.7141712	0.11408609	0.00628423	0.52303532	-0.0741197
cg09595479	PRPH;PRPH;PRPH;PRPH;P	blue	-0.3727232	-0.68576	-0.0483815	-0.0982399	0.45964014	-0.003846
cg08872493	HTR1D;HTR1D	blue	0.19853589	0.40269506	-0.180262	0.01563479	-0.1755573	-0.1060012
cg02732509	CTDSP2;CTDSP2;CTDSP2	blue	-0.2986245	-0.4741095	0.27466793	0.03842712	0.24210224	0.06180375
cg20051292	RGS22;RGS22;RGS22;RGS	blue	-0.372321	-0.7195512	0.0244073	-0.0729693	0.59509446	-0.0881811
cg01368777	IGF2;INS-	blue	-0.4296404	-0.4794339	0.12115767	-0.0266783	0.19756963	-0.0544458
cg08114617	TRPV4;TRPV4;TRPV4	blue	-0.2568834	-0.4007167	0.31028492	-0.0320018	0.24169324	0.07114565
cg09330898	VWA3B;VWA3B;VWA3B;	blue	-0.361117	-0.6579296	-0.079377	-0.0477879	0.45732303	-0.2120914
cg23618440	PTCHD4;PTCHD4;PTCHD4	blue	-0.325253	-0.6608496	0.20523346	0.02951137	0.46006462	-0.0675936
cg04655510	RP11-227D2.3;RP11-	blue	-0.2842197	-0.6016732	0.2383524	0.05409268	0.38771202	0.07383214
cg02729866	RP5-1121H13.4	blue	0.25109352	0.45142817	-0.0436945	0.1757012	-0.2403321	0.11486665
cg09071155	EVX1;EVX1;RP1-	blue	-0.3098196	-0.4953331	0.14966905	0.04429681	0.3544708	0.11869994
cg07427606	MMP17;MMP17;MMP17	blue	0.13980446	0.66991058	0.10482107	-0.0057074	-0.5645013	-0.0995813

cg09242192	OR6B2	blue	0.3038757	0.43011167	-0.1148805	0.08082162	-0.0928743	-0.0583827
cg15275259	RP11-450H5.2	blue	0.35497702	0.69316013	-0.0287195	0.09233623	-0.3604989	-0.009022
cg18547659	SRRM3;SRRM3	blue	-0.3770585	-0.4346694	-0.0443174	-0.0315348	0.32806077	-0.4811325
cg15368771	ASIC2	blue	-0.4056534	-0.4126347	0.04694251	-0.0709478	0.22629193	-0.1175381
cg20554785	AC018678.1	blue	0.6473418	0.60099564	0.30902172	0.25855012	-0.0902137	0.32222648
cg18603228	FBLN2;FBLN2	blue	-0.2947549	-0.7577736	0.02133524	0.01638826	0.63016936	-0.0806471
cg23600146	SRRM3;SRRM3	blue	-0.2585292	-0.5448606	0.11745146	0.01394397	0.49993251	-0.0299332
cg08023500	ZNF385D;ZNF385D;ZNF38	blue	-0.2336726	-0.4458701	0.19491775	0.10331486	0.31433964	0.05377526
cg11039476	CYP27C1	blue	-0.2667051	-0.6176786	0.07645993	0.22273788	0.57993611	0.0315119
cg13511154	CBS;CBS;CBS;CBS;CBS;CB	blue	-0.3512285	-0.490225	0.29484455	0.02155802	0.3417366	0.00149295
cg12725810	RP3-380B8.3	blue	0.41593409	0.38645135	-0.2809199	0.1778077	0.14036995	0.01757499
cg00556112	DRD4	blue	-0.4778429	-0.5515492	-0.1115329	-0.0716488	0.30135132	-0.0997997
cg05181846	ASB10;ASB10;ASB10;ASB	blue	0.25549938	0.44854982	0.15541325	0.22491167	-0.1133384	0.02529504
cg05103231	GTF2H4;GTF2H4;VARS2;V	blue	-0.3328013	-0.3869538	0.08857245	-0.0312776	0.23497076	-0.0460344
cg08639586	CHST8;CHST8	blue	0.26579228	0.38022761	0.07449046	0.01221275	-0.1389923	0.08317607
cg11355135	SLC5A7;SLC5A7;SLC5A7	blue	-0.2651694	-0.5305248	0.29573307	0.02941483	0.46265146	0.06426806
cg15204861	CRYAB;CRYAB;CRYAB;C	blue	-0.1366457	-0.4207119	0.23150213	0.21904408	0.40590929	0.18624562
cg25775014	FRMD5;FRMD5;FRMD5;F	blue	-0.3992543	-0.4909965	0.15945183	-0.0876585	0.23949217	-0.0465675
cg14129775	DKK2;DKK2;DKK2	blue	-0.3841203	-0.4265754	0.08685445	0.04285547	0.23354697	-0.1087846
cg22329711	PAX8;PAX8;PAX8;PAX8;P	blue	-0.1771954	-0.5323083	0.29897922	0.14586476	0.49674245	0.09528004
cg08799727	SEMA3C;SEMA3C;SEMA3	blue	-0.3088141	-0.3719033	0.14976445	0.04117117	0.27947575	-0.097931
cg24267699	ABO;ABO	blue	-0.286097	-0.5278071	-0.1474252	-0.0093972	0.37961068	-0.0608477
cg05483448	CTD-3065J16.6;CTD-	blue	-0.4799978	-0.4811756	0.12694589	-0.1570086	0.18324167	-0.0387623
cg16606320	RP11-345F18.1	blue	-0.2400648	-0.6049379	0.27048834	0.12296948	0.46287195	0.07602869
cg15850520	FAM110C;FAM110C	blue	-0.2284179	-0.4268152	0.13606785	0.10465996	0.36310606	0.03378329
cg26855107	CPAMD8	blue	-0.3143868	-0.6356696	0.17634066	0.04659664	0.52262525	-0.0528668
cg25996119	NKX2-1-AS1;NKX2-	blue	-0.2330893	-0.3724186	-0.0239901	0.01906569	0.21559267	-0.0116205
cg08203127	ARHGDIG;ARHGDIG;ARH	blue	0.39004065	0.39573498	0.03736016	0.18727603	-0.0853516	0.12875771
cg05211662	RP11-	blue	-0.4777864	-0.7810364	0.0636365	-0.0495523	0.5398943	-0.1024167
cg26249046	AGBL4;AGBL4	blue	-0.4214313	-0.7905465	0.1846161	-0.0054537	0.52514387	-0.050084
cg24768561	AGAP1;AGAP1	blue	-0.1342763	-0.5444417	0.24470144	0.16777665	0.44976704	0.18036158
cg10655144	TAAR3	blue	-0.1888888	-0.6782006	0.13541903	0.15423568	0.63414625	0.21353021
cg17102034	RP5-1099E6.3	blue	0.23385878	0.42198351	-0.233115	0.03388591	-0.1780436	-0.1279558
cg26794160	ARHGEF7;ARHGEF7;ARH	blue	-0.1355609	-0.4744918	0.29292827	0.03013238	0.41169904	0.06233829
cg06073094	RP11-867G2.8;RP11-	blue	-0.2577248	-0.4529235	-0.237735	-0.2962387	0.18568045	-0.3691065

cg11201710	FGF12-AS2;FGF12	blue	-0.2667109	-0.7930928	0.09454325	0.02146954	0.70363773	0.03252918
cg24311743	UBE2E2;UBE2E2;UBE2E2;	blue	-0.4140418	-0.5319097	0.01450302	-0.0466592	0.40179951	-0.183796
cg16928173	CDH2;CDH2;CDH2	blue	0.32359019	0.43261222	-0.2822276	0.13807344	-0.0762635	-0.0855788
cg21338558	ZNF426	blue	-0.2428894	-0.487421	0.17058743	0.15111296	0.39266876	0.03687737
cg01565949	AC131384.1;CTA-	blue	0.34064358	0.75057371	-0.009466	0.11801459	-0.4075526	0.02550454
cg10492511	RP11-286E11.1;RP11-	blue	-0.4995391	-0.611657	0.17122876	-0.1945498	0.22400411	-0.0087751
cg16864658	CCK;CCK;CCK;CCK	blue	-0.3216611	-0.4254081	0.16656063	-0.0637629	0.20989213	0.01073103
cg24056567	PSD3;PSD3;PSD3	blue	-0.3821234	-0.6079731	0.17711673	-0.0954197	0.34755835	-0.0671288
cg04398070	SHISA6;SHISA6	blue	-0.2807877	-0.3818935	-0.0085111	0.05444506	0.29099957	-0.0041714
cg08889687	PVRL3-	blue	-0.1029249	-0.4103147	0.16809929	0.12164229	0.40935155	0.12137586
cg11083325	RASIP1	blue	-0.0978611	-0.3756923	0.09748708	0.05854697	0.29255002	0.21185418
cg08179120	AC011484.1	blue	-0.2077029	-0.5085882	0.03071906	0.05477325	0.43045129	-0.0560509
cg27519330	SYT15;SYT15;SYT15;SYT1	blue	-0.3625152	-0.4703184	0.17929129	-0.0288265	0.26849611	-0.1169898
cg18456761	ADAMTS7;ADAMTS7;AD	blue	-0.3802485	-0.4162536	-0.043419	-0.0737172	0.28111644	-0.1950518
cg20219381	RGS22;RGS22;RGS22;RGS	blue	-0.2213477	-0.709653	0.18093335	0.02314483	0.60591423	0.02746267
cg18767073	RP11-1084I9.2;RP11-	blue	0.29371568	0.54438117	0.02043369	0.18303412	-0.137449	0.14793874
cg23191950	ALDH1A3;ALDH1A3;ALD	blue	-0.1030994	-0.3211235	0.09440846	0.01080256	0.25051083	-0.0605274
cg15001687	NINL	blue	-0.4968904	-0.5803797	-0.000146	-0.1778419	0.25882735	-0.1384984
cg00590036	TMEM181;TMEM181	blue	-0.4542754	-0.5748817	-0.0899151	-0.1438191	0.26535386	-0.1184471
cg13119611	CDK20;CDK20;CDK20;CD	blue	-0.3758367	-0.4654532	0.29844801	-0.027719	0.21283769	-0.0285478
cg14987769	RESP18	blue	-0.1769378	-0.4356199	0.01670732	0.06694963	0.38341296	0.08860978
cg11257595	RGMA;RGMA;RGMA	blue	-0.253815	-0.4302975	0.01454264	0.08867553	0.28209827	0.01717481
cg24144244	SNORA40	blue	0.2432821	0.40454104	0.40946888	0.23964442	-0.1329121	0.18128336
cg06333719	CTD-	blue	-0.3038265	-0.617738	0.01805758	0.06156607	0.57475261	-0.1266749
cg26615440	TMEM108;TMEM108;TME	blue	0.01607896	0.33408565	0.08928897	0.16975509	-0.1293148	0.01202428
cg24000860	ZCCHC24	blue	-0.4544834	-0.6404173	0.05497986	0.04945395	0.48503394	-0.0861378
cg13557939	PTPN3;PTPN3;PTPN3	blue	-0.3213399	-0.4845576	0.19696805	-0.1067171	0.21331968	0.08481169
cg12065138	AJAP1	blue	-0.3032043	-0.7721212	0.10742571	0.0356364	0.55981622	0.10782766
cg23013958	AK4;AK4;AK4;AK4;AK4	blue	-0.2551806	-0.399699	0.10498026	0.18160166	0.36925071	0.00979986
cg00345862	LECT1;LECT1;LECT1;LECT	blue	-0.1765678	-0.3643788	0.15706879	-0.0076476	0.30045186	0.10656272
cg09045262	AKR7L;AKR7L;AKR7L	blue	-0.143595	-0.3959915	0.12385124	0.0566396	0.3509289	0.05433826
cg23998405	MGMT;MGMT;MGMT	blue	-0.4342504	-0.4887146	-0.0052489	-0.0360798	0.20982719	-0.0721024
cg06444226	C1orf159;C1orf159;C1orf159	blue	0.10687034	0.34212313	-0.1725086	-0.0880682	-0.1760876	-0.0670362
cg16065899	ALX4	blue	-0.1960717	-0.4205253	0.07162731	0.08814949	0.34484962	-0.0501506
cg18945420	LAMP5;LAMP5;RP5-	blue	-0.2800155	-0.5085569	0.28164966	0.07740273	0.36872138	0.03721493

cg24866418	LHFPL4	blue	-0.3004912	-0.7582832	0.1750891	-0.0668099	0.52604492	0.12953638
cg04514249	RP13-578N3.3;FREM3	blue	-0.0435404	-0.499152	-0.1914484	-0.0757796	0.41287508	-0.0958972
cg00476032	SLC22A23;SLC22A23;SLC2	blue	0.23874034	0.44470333	0.18620188	0.13139903	-0.2064319	0.1623581
cg13022388	MYEF2;MYEF2;MYEF2;M	blue	-0.3100637	-0.5482715	0.05104665	-0.0616637	0.34673776	-0.0898818
cg15174905	MEF2B;MEF2B;MEF2B;ME	blue	-0.2733179	-0.6167672	0.03814618	0.08463572	0.51855644	0.02956864
cg08685096	GRIK1;GRIK1;GRIK1;GRI	blue	-0.2833119	-0.4042029	0.14504312	-0.0331505	0.24856585	0.10864742
cg01231735	AC006445.8;AC006445.8	blue	-0.3959155	-0.4154983	0.26396111	-0.0259731	0.12964717	-0.0198916
cg25693132	GRM2;GRM2;GRM2;GRM2	blue	-0.1317965	-0.3795646	0.23685856	0.00612866	0.21903232	0.04866372
cg13062960	RP11-507P23.3;RP11-	blue	0.20423651	0.36852641	-0.1713247	0.50707209	-0.0843451	0.10180491
cg24754313	RP11-78A18.2	blue	-0.1646829	-0.4872776	0.2494989	0.12200864	0.47564905	0.09328637
cg00720747	GLIS2	blue	-0.2372761	-0.3563201	0.02745821	0.00822765	0.30320381	-0.2412272
cg03372470	DEFB121;DEFB121	blue	0.17268277	0.33608344	-0.4188183	0.07359607	-0.1610479	-0.2107655
cg02002551	NEFM;NEFM;NEFM;NEFM	blue	-0.0900131	-0.3784034	0.06740314	0.06460128	0.37137904	0.22500503
cg00419702	BAIAP2L2	blue	-0.2350596	-0.3807928	0.15083625	-0.0015611	0.24069137	0.01501287
cg03260781	CLCNKA;HSPB7;HSPB7;C	blue	-0.0798223	0.35811921	0.33172804	0.08777076	-0.2540082	-0.0063054
cg15246619	FGF17;FGF17;EPB49;EPB4	blue	-0.2253301	-0.5775499	0.17311248	0.08874098	0.44635823	0.10454842
cg20855344	NKIRAS1;NR1D2;NR1D2;N	blue	-0.3110804	-0.3667275	0.35073604	0.12434668	0.25212315	0.045357
cg13154724	MARVELD3;SNORA70D	blue	-0.2270181	-0.379677	0.19478494	0.0148534	0.24335252	0.04445746
cg17270101	C1orf168	blue	0.0145211	0.30535511	-0.0301682	0.1227107	-0.157404	-0.0631819
cg00158333	IGF2BP1;RP11-	blue	-0.342085	-0.6988492	0.11133907	0.04557976	0.51352297	-0.144546
cg24132694	SNAPC2;CTD-3193O13.1	blue	-0.2815572	-0.3846907	0.08604333	0.01166246	0.24747183	-0.1321267
cg23605991	PDE6B;PDE6B	blue	-0.1859323	-0.3783699	0.2729623	0.1989017	0.34763312	0.01194588
cg03671597	SEZ6;SEZ6;SEZ6;SEZ6	blue	-0.3601677	-0.6933751	0.10561827	-0.0327321	0.45081791	-0.0715861
cg05844895	CBFA2T3;CBFA2T3;CBFA2	blue	0.09291993	0.50425221	0.19500727	-0.0692078	-0.4082216	-0.0845691
cg09050331	PRDM14;PRDM14	blue	-0.316954	-0.4252489	0.12124592	-0.0376108	0.21833205	-0.0697846
cg11622008	SPSB4	blue	-0.3822525	-0.3969582	-0.0021401	-0.0478144	0.16394614	-0.2210241
cg10285365	NDUFB9;NDUFB9;NDUFB	blue	0.07891525	0.28212099	0.35016824	0.36124318	-0.0937211	0.14808664
cg05512327	SHOX2;SHOX2;SHOX2;SH	blue	-0.120665	-0.5314529	0.09315169	0.16514413	0.45681034	0.05860655
cg21604042	SCUBE3;SCUBE3	blue	-0.2707771	-0.626911	0.21815162	-0.0548241	0.43566504	0.10946413
cg17502037	RP11-64J4.2;RP11-64J4.2	blue	-0.0277996	-0.4170246	0.11915949	0.11716409	0.41880967	0.08634366
cg15986413	ECEL1;ECEL1;ECEL1	blue	-0.320427	-0.4255329	0.10973542	-0.1525379	0.16610785	-0.0555858
cg25056460	PPAPDC1A;PPAPDC1A;PP	blue	-0.2605587	-0.5244831	0.0792479	0.07349866	0.39069156	-0.0321548
cg11920122	GNAL;GNAL;GNAL;GNAL	blue	-0.2989031	-0.6588454	0.21698425	0.01503083	0.4908002	0.07233845
cg02022733	PCDHGC4	blue	-0.1901913	-0.3636675	0.23914724	-0.1095278	0.15475996	-0.0479339
cg04379508	MAGI2;MAGI2;MAGI2	blue	-0.1611078	-0.4739293	-0.2184799	0.03730195	0.39547501	0.06693616

cg23172480	DCBLD1;DCBLD1;DCBLD	blue	-0.2306454	-0.4351739	0.15922716	0.07574115	0.22723282	0.07669458
cg01739251	TAL1;TAL1;TAL1	blue	-0.1609232	-0.362338	-0.1729741	-0.084657	0.25372423	-0.3462584
cg22543665	RP11-350F16.1	blue	0.3055506	0.64078143	0.05479139	0.10472433	-0.3510862	0.04135082
cg26533154	AC114302.1	blue	-0.1896693	-0.4841122	0.13733467	0.00755111	0.41790692	0.01684734
cg14095959	RP11-359D14.3	blue	-0.2441651	-0.4780163	-0.0547537	0.05425869	0.39760223	0.0204017
cg27382744	RP11-64J4.2;RP11-64J4.2	blue	-0.0719882	-0.4511666	0.1214433	0.08121429	0.41375648	0.06683491
cg02056062	WWTR1-	blue	-0.3326435	-0.5837925	0.33133376	-0.0448491	0.29696377	0.03958642
cg00172849	COL11A1;COL11A1;COL11	blue	-0.1923243	-0.4059061	0.11548212	-0.0558937	0.31357463	0.18093592
cg25384513	GFPT2;GFPT2;GFPT2;GFP	blue	0.07445803	0.34310077	0.21042592	0.56549558	-0.0498046	0.09956845
cg23926743	TAC1;TAC1;TAC1	blue	-0.26573	-0.4260709	0.19032612	-0.0478915	0.22229794	0.05113907
cg04142864	PHLDB1;PHLDB1;PHLDB1	blue	-0.1706071	-0.4539543	0.08787676	0.02618603	0.37809873	0.05943177
cg01593851	AC011467.2	blue	0.38282465	0.48088604	0.01207082	0.17029885	-0.0180652	-0.0322238
cg07739179	NAGS;NAGS;PYY;PYY	blue	-0.1783955	-0.3656078	-0.0631993	-0.0167938	0.25360174	-0.0395069
cg25410668	RPA2;RPA2;RPA2;RPA2	blue	-0.1675362	-0.526	0.01490313	-0.0732773	0.36080348	0.20987183
cg20927649	RP1-	blue	-0.0049989	0.32882168	0.31527045	0.15703486	-0.1162814	0.18417462
cg27272363	RP11-1082L8.2;RP11-	blue	0.1029391	0.30819342	0.14875208	0.26280052	-0.1471658	0.29509115
cg20294304	HMGA1;HMGA1	blue	-0.2591672	-0.4646209	0.06045649	-0.0265005	0.40145023	-0.1486631
cg04486617	BARX1	blue	-0.4172298	-0.4557694	0.02712763	-0.0960501	0.20104593	-0.1684233
cg22935456	ZNF470;ZNF470	blue	-0.2461257	-0.4146583	-0.2177814	-0.1450559	0.23943856	-0.4632375
cg07917478	RP11-12M5.3;RP11-12M5.3	blue	0.25675163	0.44479798	0.08397469	0.19886382	-0.0406093	0.19658025
cg22624285	IGFL3	blue	0.28055554	0.41826201	0.24104117	0.2002964	-0.0868527	0.09698827
cg17921672	RUNX1T1;RUNX1T1;RUN	blue	-0.207243	-0.4840764	0.19932244	0.13462215	0.40401063	0.04516427
cg07643096	DCBLD1;DCBLD1;DCBLD	blue	-0.0544046	-0.4594709	0.18276123	0.18675569	0.39265513	0.21874512
cg21843015	PGLYRP3	blue	0.15105392	0.47865757	0.02105645	0.18149971	-0.2446949	0.14597255
cg22697825	CAPN2;CAPN2	blue	-0.026177	0.27000502	0.31854851	0.18643503	-0.0264509	0.08664706
cg13134509	PDE4D;PDE4D;PDE4D;PDE	blue	-0.3376419	-0.4023013	0.23140158	-0.0087516	0.15138686	-0.0193343
cg24032888	RP11-120J1.1	blue	-0.0962775	-0.542793	0.22746727	0.16791146	0.52752951	0.2059746
cg24725576	FOXG1;RP11-966I7.1;RP11-	blue	-0.1968444	-0.4506035	-0.1189039	-0.0680595	0.2840545	0.09202244
cg22890950	PPAPDC1A;PPAPDC1A;PP	blue	-0.2598722	-0.3678416	-0.2451921	-0.2277767	0.19462473	-0.3581474
cg10598104	MTMR7;MTMR7;MTMR7	blue	0.24392682	0.4374004	0.26641519	0.27301603	-0.1246329	0.33084741
cg09381003	SHKBP1;SHKBP1;SPTBN4;	blue	-0.2110322	-0.4272809	0.18096059	0.03254196	0.26545111	0.14353397
cg24657523	MYEF2;MYEF2;MYEF2;M	blue	-0.0780862	-0.3419927	0.03638642	0.10750841	0.34238283	0.0408038
cg15141434	RALBP1;RALBP1	blue	-0.3184386	-0.658837	0.16126311	0.01738677	0.43717946	-0.0361131
cg19909996	PTPN14;PTPN14	blue	0.3332152	0.45266441	0.18939962	0.13032442	-0.1907098	0.07008692
cg21683284	DNAJC6;DNAJC6;DNAJC6	blue	-0.2181275	-0.4591245	0.20154892	-0.0334417	0.2996541	0.08258843

cg02015909	CES4A;CES4A;CES4A;CES	blue	-0.2742152	-0.4627814	0.04004284	0.06511375	0.3546555	-0.2160938
cg02797569	RP13-	blue	0.36163948	0.37447727	0.1447555	0.38835906	-0.1697345	0.41294449
cg03309176	ARHGEF25;ARHGEF25	blue	-0.0341256	-0.3440718	-0.088181	0.16717308	0.35569408	0.11840584
cg27363247	RP11-162G9.1;RP11-	blue	-0.3235894	-0.3625865	0.08446534	0.00136908	0.24300888	-0.0973027
cg25762125	RP11-69H7.2	blue	0.22075761	0.31077942	0.47663576	0.26674008	-0.1260213	0.29696235
cg16902385	ST8SIA3	blue	-0.1211233	-0.3765369	-0.0319685	-0.0836127	0.26972517	0.01077041
cg23100668	TBX21	blue	0.06112676	0.31346452	0.22975819	0.17848479	-0.1788959	0.12459836
cg20474425	RP11-753H16.3	blue	-0.1485149	-0.3912655	-0.0924457	0.03643787	0.32044558	-0.0120876
cg04118462	RNF13;RNF13;RNF13;RNF	blue	0.19342722	0.35658872	0.21974788	0.49830068	-0.1411157	0.18820384
cg14104301	SSTR1	blue	-0.098127	-0.4246464	0.09212489	0.12236065	0.40114893	0.09429128
cg09880359	SGSM2;SGSM2;SGSM2	blue	-0.1408112	0.34800861	0.35441273	0.13768	-0.2533828	-0.0460346
cg17124509	C17orf57;C17orf57;C17orf57	blue	-0.1836956	-0.3343125	-0.0871758	0.0876266	0.25663779	-0.0168566
cg26400267	WDR66	blue	0.11786679	0.40100137	0.14963096	0.30586218	-0.0377903	0.09673458
cg03459312	WWC2-AS2;WWC2	blue	-0.1942308	-0.5094766	0.20076809	-0.0840715	0.34283132	0.02036798
cg04695516	RP13-614K11.1;RP13-	blue	0.03140191	0.27027297	0.39201403	0.0982917	-0.145724	0.04770878
cg17508941	AC003986.5	blue	0.0257166	-0.3293479	0.16546459	0.14240517	0.32080583	0.08956433
cg03394122	MFAP2;MFAP2;MFAP2;M	blue	0.27925255	0.37290078	0.38732644	0.31165616	-0.0392798	0.38683653
cg16568248	RP5-881L22.4	blue	0.14217399	0.31767329	0.22331094	0.39649067	-0.0993195	0.11906772
cg15297841	RP4-705F19.1	blue	0.11398361	0.41746842	0.4923484	0.13937234	-0.304796	0.30417956
cg21899500	GRM2;GRM2;GRM2;GRM2	blue	0.07852494	-0.3462758	0.2942235	0.09927804	0.33112116	0.27519113
cg00048759	STAG3;STAG3;GPC2;GPC2	blue	-0.1683813	-0.4756507	0.0706696	-0.0513584	0.29919716	0.07092529
cg23451424	CACNA2D1;CACNA2D1;C	blue	-0.1692329	-0.5009245	-0.180041	-0.1734847	0.36239971	-0.3112942
cg20284000	DBNL;PGAM2	blue	0.31443005	0.41939725	0.34504798	0.3229294	-0.219052	0.25344431
cg04352272	RP11-64J4.2;RP11-64J4.2	blue	0.07883753	-0.340714	0.21201475	0.14169521	0.38745909	0.259492
cg26254792	HOOK1;HOOK1;HOOK1	blue	-0.193325	-0.3863743	-0.126366	-0.1441961	0.28030773	-0.1749192
cg27547835	ABCD4;ABCD4;ABCD4;AB	blue	-0.0630068	0.30977133	0.33292899	0.08629929	-0.2541613	0.20136624
cg07914457	NR0B2	blue	0.01406309	0.41543028	0.46330515	0.43689038	-0.301695	0.220892
cg15456821	BEST1;BEST1;BEST1;BEST	blue	0.17311726	0.37540966	0.36824083	0.54962363	-0.0687557	0.2566561
cg20570841	POLR2F;POLR2F;POLR2F;	blue	0.21694026	0.39687323	-0.133907	0.25352533	-0.1130693	0.10559742
cg21368354	CARKD;CARKD;CARKD;C	blue	-0.3768235	-0.3910114	-0.0104115	-0.0986831	0.15511759	-0.1596913
cg08365760	GPS2;GPS2;GPS2;RP11-	blue	0.11912577	0.36971771	0.38249629	0.22362736	-0.1953517	0.24931006
cg23784313	EPS8;EPS8;EPS8;EPS8;EPS	blue	-0.3119786	-0.3988912	-0.3300818	-0.2122999	0.24676851	-0.4945977
cg02643054	RP1-170O19.21;RP1-	blue	-0.0498595	-0.3570599	-0.0488823	-0.0161566	0.27836092	0.17650738
cg21155316	EIF2D;EIF2D;EIF2D;EIF2D	blue	-0.0677855	-0.3563163	0.21801363	0.05116138	0.29725344	0.08457154
cg06872437	CCM2;CCM2;CCM2;CCM2;	blue	0.2883387	0.40818682	0.24340929	0.27180456	-0.1665585	0.27112662

cg21957925	LUM;LUM;LUM;LUM	blue	0.09912226	0.32827633	-0.0975146	0.26063861	-0.1195451	0.00343341
cg22720632	CTD-	blue	0.30463845	0.48182016	0.38449566	0.61144467	-0.1773252	0.28252069
cg08551870	GAL3ST1;GAL3ST1;GAL3S	blue	0.22238506	0.37053331	0.47255152	0.22068279	-0.1470565	0.29510731
cg13392029	ARTN;ARTN;ARTN;ARTN;	blue	0.07214892	0.41909193	0.27707576	0.26668607	-0.1272658	0.11025794
cg01472101	CLEC10A;CLEC10A;CLEC1	blue	0.03849543	0.30529221	0.07321064	0.13778335	-0.0822342	0.11485453
cg19339993	RP1-167G20.2;RP1-	blue	0.36621293	0.39857288	0.11281647	0.33044485	-0.1027495	0.2379656
cg12896519	AL136373.1	blue	0.02444065	0.29466308	0.41786357	0.16368489	-0.1928475	0.11539576
cg14713333	RP1-32I10.10	blue	-0.0364927	0.30737018	0.23917372	0.11522596	-0.201708	0.0163235
cg05996615	PSAPL1;SORCS2	blue	0.08833881	0.50254707	0.34726972	0.21389792	-0.3028104	0.16224282
cg14352983	L3MBTL4	blue	-0.2057969	-0.5645782	-0.0324371	-0.1400592	0.31289345	-0.0056002
cg02119837	STARD10-	blue	0.24426509	0.45374561	0.22826508	0.26230115	-0.2610997	0.26466009
cg26371327	EPHA2	blue	0.25884931	0.41811721	0.18134066	0.16083523	-0.3013369	0.19565888
cg05864261	ABCG5;ABCG5;ABCG8;AB	blue	0.00641107	0.46521373	0.19298688	0.30957137	-0.3712528	0.11552421
cg26419941	RP11-579D7.4;RP11-	blue	-0.1388321	-0.473349	-0.2325554	-0.0644989	0.35862008	-0.2750342
cg00419963	FEZF1;FEZF1;FEZF1;RP11-	blue	-0.1198409	-0.3726875	-0.0088994	-0.1560641	0.28010742	-0.0202183
cg12363807	SPG20;SPG20;SPG20;SPG2	blue	-0.4030614	-0.47428	-0.1908956	-0.1850935	0.19979943	-0.4098855
cg11641809	FBLN1;FBLN1;FBLN1	blue	-0.042061	0.46078181	0.19631505	0.10563233	-0.3604854	0.10373938
cg10172783	PYY;NAGS;NAGS;NAGS	blue	-0.2767862	-0.4095281	-0.1084894	-0.1063599	0.19571677	-0.3037059
cg09591286	ZNF804B	blue	-0.3390754	-0.5001209	0.01711555	-0.2731115	0.28539724	-0.1235572
cg20768314	DOK7;DOK7	blue	0.31779529	0.41707903	0.4843944	0.40411617	-0.0865493	0.41702504
cg17010349	LAG3;LAG3	blue	0.15060986	0.31198733	0.01737421	0.08344497	-0.2104942	0.25384173
cg04779161	DMPK;DMPK;DMPK;DMP	blue	0.29773954	0.41347132	0.09554837	0.13186877	-0.194038	0.33795065
cg10908528	GALNT13;AC009227.2;GAL	blue	-0.3061257	-0.3985573	-0.4201375	-0.2014248	0.20645531	-0.2652715
cg13132007	RP1-102E24.8	blue	0.36452278	0.39539541	0.32008129	0.18840061	-0.0746886	0.58599683
cg13370209	MIR95;ABLIM2	blue	-0.0680347	0.24864171	0.12274025	0.13778203	-0.1983558	0.15943662
cg20829569	KIAA1644	blue	0.17505714	0.41343444	0.46441653	0.24913295	-0.2094001	0.26203377
cg17755964	HES5	blue	-0.3877258	-0.5021575	-0.1351458	-0.178468	0.27858989	-0.3519846
cg25145360	ALDH3A1;ALDH3A1;ALD	blue	-0.0227055	0.37774871	-0.0614796	-0.1185844	-0.3078376	-0.070809
cg05649487	LRRFIP2;LRRFIP2;LRRFIP	blue	0.16323669	0.3532356	-0.0143897	0.42252429	-0.1168947	0.22454253
cg25742136	GPAA1;GPAA1;GPAA1;GP	blue	0.15737058	0.31903833	0.17363984	0.20522543	-0.1141676	0.19943898
cg17153568	SP9	blue	-0.3216812	-0.3719867	0.00619549	-0.2684374	0.19826781	-0.1352726
cg25829666	AC012087.2;AC012087.2;TH	blue	-0.4417378	-0.6240835	-0.0452877	-0.2338568	0.250691	-0.2381319
cg25948592	L3MBTL1;L3MBTL1;L3MB	blue	0.23657675	0.38250154	-0.0653976	0.26025651	-0.2016168	0.28361638
cg03916430	LINC00243	blue	0.2113325	0.3245729	0.12825109	0.1030247	-0.1585738	0.24694481
cg10878205	AGXT;AGXT;AGXT	blue	0.24696872	0.42142148	0.31934401	0.50641802	-0.047922	0.11077703

cg15980954	PSMG4;PSMG4;PSMG4;PS	blue	-0.3889662	-0.4593551	0.09188047	-0.1971749	0.17776613	-0.2689419
cg19000048	Y_RNA	blue	0.16150155	0.48169329	0.19552397	0.45309275	-0.1878192	0.30454104
cg19133199	TMEM91;B9D2;TMEM91	blue	0.01184055	0.29961888	0.10027791	0.19808364	-0.218423	0.22199017
cg06387459	ARPP21;ARPP21;ARPP21;	blue	-0.3989168	-0.6040121	-0.0870612	-0.2512362	0.32292503	-0.3676566
cg14841103	E4F1;DNASE1L2;E4F1;E4F	blue	0.22672805	0.35705989	0.46179081	0.22797921	-0.2449961	0.18761
cg22476252	CCDC57;CCDC57	blue	0.37724845	0.43012765	0.16165881	0.37416461	0.02089992	0.44446015
cg17961359	RP11-14K3.7	blue	0.04846682	0.35053114	0.06495347	0.21860065	-0.2053682	0.00574443
cg01845664	CTA-992D9.7	blue	0.05547499	0.49212181	0.20156605	0.24692295	-0.2746419	0.10244941
cg19478228	DFNB31;DFNB31	blue	0.13963375	0.43124848	0.1793686	0.19291551	-0.1412629	0.24745956
cg15159060	FSHB	blue	-0.0200067	0.44353604	0.21223826	-0.0158415	-0.3337566	-0.0160267
cg21092930	CTC-	blue	0.11022542	0.44337688	0.15962682	0.61002554	-0.208726	0.09732723
cg08917230	AMPH;AMPH	blue	0.33740367	0.42088113	-0.0186988	0.29168675	-0.1383066	0.26735123
cg03099014	RP11-10J21.6	blue	0.43424836	0.46713365	0.46250695	0.37522877	-0.0415139	0.54675177
cg23371278	CNNM1;CNNM1;CNNM1	blue	-0.2547085	-0.4626872	-0.0844827	-0.1946822	0.24090604	-0.2410919
cg01770598	COL20A1;COL20A1;COL20	blue	0.21581628	0.38874802	0.28204304	0.29187995	-0.1578598	0.29843107
cg01522083	GNPTG;GNPTG;GNPTG;G	blue	0.18684732	0.44470811	0.42720195	0.23500197	-0.2315035	0.31184317
cg07191311	RN5S275	blue	0.16903518	0.43451542	0.02080576	0.36172573	-0.1765873	0.09886686
cg03326689	MYO5C;MYO5C;MYO5C;	blue	-0.3348567	-0.3720766	-0.3339039	-0.1336227	0.21581998	-0.3492828
cg02522973	CDC42BPG;CDC42BPG;CD	blue	0.27184541	0.34881526	0.09353854	0.23718189	0.01630781	0.2168638
cg04692506	SPI1;SPI1;SPI1;SPI1	blue	0.17024784	0.48584642	0.47989689	0.50068097	-0.3562647	0.3047402
cg15831581	PLA2G4C;PLA2G4C	blue	0.16604529	0.37685427	0.22603487	0.05333087	-0.2559206	0.18082956
cg26077378	ZNF385D;ZNF385D;ZNF38	blue	-0.1217118	-0.1618425	0.23009165	0.03543437	0.02371736	0.08672845
cg20625165	DAAM2;DAAM2	blue	-0.1326353	-0.5064666	-0.1636897	-0.1038174	0.40099163	-0.2744407
cg03590031	RP4-697K14.7;RP4-	blue	0.19181426	0.3982841	0.49508376	0.15181107	-0.2394618	0.32180801
cg22752602	TMEM143;TMEM143;SYN	blue	0.31681396	0.44494185	0.56852593	0.29254801	-0.2672559	0.29249445
cg02530321	TTPAL;TTPAL;TTPAL;TTP	blue	-0.0457483	0.39681841	0.15452954	0.15304745	-0.23821	-0.0148608
cg25448699	TRAF3IP3;TRAF3IP3;TRAF	blue	0.24192112	0.76275053	-0.0380184	0.00829079	-0.744964	0.00337562
cg01771061	FHL2;FHL2	blue	0.08871836	0.54582903	0.19656364	0.25674271	-0.3720544	0.10634034
cg27105645	TP53;TP53;TP53;TP53;TP53;	blue	0.29058043	0.43946666	0.25561193	0.21740744	-0.2126804	0.28899173
cg24146773	SH3BGR;SH3BGR;SH3BG	blue	0.17658689	0.43112007	0.14950693	0.22582164	-0.3000923	0.09278498
cg19255656	SH3BP2;SH3BP2;SH3BP2;S	blue	0.27704515	0.75607395	0.10916436	-0.0100654	-0.7263154	0.05578409
cg25064552	SLC39A1;SLC39A1;CREB3	blue	0.243457	0.7413992	0.05505283	0.07192748	-0.7437584	0.09390089
cg15900011	PSENEN;U2AF1L4	blue	0.08333385	0.30799386	0.32917136	0.32696523	-0.210456	0.21544194
cg16620673	RP11-385E5.7	blue	0.15859117	0.31575557	0.43459657	0.18603775	-0.2480191	0.26988936
cg09045196	AC062028.1;AC062028.1;A	blue	0.13152643	0.46485052	0.30441277	0.14754839	-0.2499972	0.1799514

cg08482694	PTGS2;PTGS2;PTGS2;PTG	blue	-0.4108761	-0.3963632	-0.2266475	-0.204722	0.13204274	-0.1982217
cg26369382	TFR2;TFR2;TFR2;TFR2;TFR	blue	0.23029044	0.4799708	0.00907344	0.54108682	-0.2943513	0.08859721
cg08120071	SLC9A3R2;SLC9A3R2;SLC	blue	0.23899327	0.49226191	0.18619327	0.12553225	-0.289249	0.19909874
cg27309294	H2AFY2;AIFM2	blue	0.34471358	0.7701637	-0.0444648	-0.0304005	-0.7003227	0.07684391
cg26322248	ISM1	blue	-0.3773198	-0.6745128	0.00525732	-0.1677593	0.36877758	-0.2097671
cg19669531	CTD-2555A7.3;ACSF3	blue	-0.2202989	-0.4175885	-0.5626012	-0.1592915	0.31111803	-0.30567
cg26988916	TMEM91	blue	0.21711575	0.76758346	-0.0217577	-0.0015543	-0.7396865	0.00488416
cg01836353	AQP7;AQP7;AQP7;AQP7;A	blue	0.42938682	0.47270339	0.40758087	0.21032656	-0.1470503	0.62806262
cg17822373	TMEM130;TMEM130;TME	blue	0.34695726	0.47854156	0.324812	0.57654508	-0.1993045	0.21278005
cg11755372	ECE2;ECE2;ECE2;ECE2;ECE	blue	0.39073823	0.3942927	0.10255716	0.35022755	-0.0752664	0.23318286
cg17695891	SLC9C2;SLC9C2;SLC9C2;S	blue	-0.0752593	0.38333083	0.10337454	0.06395348	-0.2215902	-0.1369056
cg20253238	ALDH3A1;ALDH3A1;ALD	blue	0.12881867	0.51430245	0.07946014	0.0084368	-0.4222538	0.01608465
cg03433986	BSCL2;BSCL2;BSCL2;BSC	blue	0.04966209	0.37035814	0.28491108	0.25060359	-0.2580997	0.13818745
cg20710730	HOXB-AS4;HOXB-	blue	0.34642968	0.34360304	0.32379506	0.30010421	-0.0359645	0.34468596
cg14748210	RP11-181G12.4;AL590822.1	blue	0.28209016	0.47133805	0.25710962	0.35694581	-0.1417073	0.212619
cg25574111	IGFL4	blue	0.21047009	0.4599776	0.05295167	0.0812693	-0.2440262	0.24447959
cg21485704	PLEKHD1;PLEKHD1	blue	-0.2737506	-0.597869	-0.0547335	-0.082061	0.41125422	-0.0593654
cg13368756	CTNND2;CTNND2;CTNND	blue	-0.3563686	-0.546414	0.04301186	-0.1943527	0.18527967	-0.2371109
cg04929006	HDAC10;HDAC10;HDAC1	blue	0.28789012	0.56021106	0.36617458	0.33393719	-0.2615149	0.23173482
cg09142020	AGAP1;AGAP1	blue	-0.3456262	-0.5437057	-0.0166528	-0.141365	0.32790642	-0.2200384
cg12850675	SEMA3C;SEMA3C;SEMA3	blue	-0.2570344	-0.6566424	0.08021188	-0.1399621	0.37568594	-0.046374
cg26601295	TIAM2;TIAM2;TIAM2;TIA	blue	0.0904938	0.39083168	0.02853903	0.09422333	-0.1862734	0.08824027
cg08817148	UROS;UROS;UROS;UROS;	blue	0.24787104	0.47256814	0.42600585	0.15366777	-0.3569251	0.27748498
cg27605489	CRHR2;CRHR2;CRHR2	blue	0.21993983	0.47856633	0.36833544	0.25778726	-0.1932103	0.19658904
cg12388583	PTPRB;PTPRB;PTPRB;PTP	blue	-0.3363857	-0.6384981	-0.0084308	-0.1948302	0.39864585	-0.3015987
cg05639405	CHST13;CHST13	blue	-0.1873697	-0.6077627	-0.1259444	-0.1094062	0.47774919	-0.1232995
cg00109375	DNAH3;DNAH3	blue	0.29271617	0.46058742	0.07560101	0.13738154	-0.2223721	0.14864908
cg16985652	NR0B2	blue	0.11631398	0.58858432	0.14293523	0.33086508	-0.4680522	0.14344764
cg13245883	ELAVL4;ELAVL4;RP11-	blue	0.15029178	0.51365808	0.11916467	0.19843564	-0.3700542	0.09089589
cg11284316	UNC80;UNC80	blue	-0.4179527	-0.5752859	-0.0173095	-0.1791645	0.31872884	-0.5389148
cg22022010	TRPM2;TRPM2;TRPM2;TR	blue	0.18465839	0.53467166	0.34697881	0.05938451	-0.4737297	0.34465037
cg03761216	EPS15;EPS15;EPS15;EPS15;	blue	0.36746096	0.64590007	0.26552321	0.5474923	-0.4212174	0.32429762
cg10766475	RAB32	blue	-0.3291077	-0.5306379	-0.0479115	-0.1201614	0.32091495	-0.2657471
cg11724516	SLC46A2;SLC46A2	blue	-0.4048301	-0.5392319	-0.1391582	-0.1746438	0.281327	-0.320696
cg10189763	MUC7;MUC7;MUC7;MUC7	blue	0.31510096	0.34918064	-0.2025646	0.13326678	-0.092107	0.10563686

cg21101356	EGLN3;EGLN3;EGLN3;EGL	blue	-0.2605613	-0.6067082	-0.0660442	-0.0786485	0.41933387	-0.2005378
cg02899083	NELL1	blue	0.27790268	0.35515869	0.28766877	0.25770746	-0.0865565	0.30537684
cg26146027	AC012087.2;AC012087.2;TH	blue	-0.342288	-0.6209206	-0.0804764	-0.1555294	0.2865017	-0.168643
cg20176349	SPOCK3;SPOCK3;SPOCK3	blue	-0.3599605	-0.6076754	0.04530881	-0.1527442	0.28676878	-0.0211719
cg08855568	PTPRG;PTPRG;PTPRG;PTP	blue	-0.5008228	-0.5294872	-0.1532915	-0.1916808	0.2286604	-0.5313108
cg21887193	AGAP3;AGAP3;AGAP3;A	blue	-0.1513301	-0.7836242	0.05042937	0.04930807	0.80803629	0.05931463
cg02707277	BRD2;BRD2;BRD2;BRD2;B	blue	0.43146412	0.45569355	0.03588358	0.462286	-0.1029744	0.26165598
cg14310418	NUCB1-	blue	0.41259687	0.58895832	0.24490344	0.27400259	-0.3186912	0.37780268
cg05764376	LRRC49;LRRC49;LRRC49;L	blue	-0.1945365	-0.6832096	0.03085758	-0.0995913	0.50623613	0.03266088
cg07863854	SPOCD1;SPOCD1;SPOCD1	blue	0.16714771	0.48397421	0.30778058	0.24071049	-0.3679485	0.15191332
cg00712342	TPM3;TPM3;TPM3;TPM3;T	blue	0.28752127	0.55014537	0.08886398	0.47752185	-0.2830402	0.17482682
cg00501931	RP11-363D24.1	blue	0.13153808	0.59154954	0.31648935	0.26699632	-0.3594608	0.08782094
cg18908780	PDE4DIP;PDE4DIP;PDE4DI	blue	0.34810531	0.48569606	0.29586912	0.1282061	-0.2568847	0.27208265
cg04190870	UGT8;UGT8	blue	-0.4647614	-0.6159217	-0.1267089	-0.3019455	0.26088157	-0.4000213
cg18911972	CPSF4L;CPSF4L	blue	0.18714881	0.5683652	0.33425998	0.30055667	-0.431964	0.25634226
cg22301987	VPS16	blue	0.30403933	0.58520471	0.20035073	0.16822633	-0.2980809	0.30595518
cg15680620	ANKRD45;ANKRD45	blue	-0.2051664	-0.3998613	-0.342145	-0.0272454	0.25889337	-0.2514984
cg01693026	DIO3;DIO3;DIO3OS;DIO3O	blue	-0.1682088	-0.6525668	0.04618004	-0.0619059	0.51940402	-0.1030937
cg08027385	IGFBP3;IGFBP3;IGFBP3;IG	blue	-0.2534879	-0.5403213	-0.1395084	-0.1727837	0.35379035	-0.140149
cg03201811	CTC-	blue	0.31166508	0.45602138	0.15063542	0.13132726	-0.2058616	0.3350697
cg07707227	MIR449C;CDC20B;CDC20B	blue	-0.2607849	-0.6290962	-0.1826603	-0.0940874	0.47681851	-0.2707973
cg24514412	HMCN1	blue	-0.3529104	-0.5230494	-0.020397	-0.1695411	0.2997418	-0.1142953
cg10407200	ROBO1	blue	-0.2809502	-0.4375381	-0.4113535	-0.0767994	0.33521014	-0.4210956
cg18131689	ANO5	blue	-0.3206638	-0.4419403	-0.2641862	-0.2767306	0.2574827	-0.3536508
cg17268585	P4HA3;P4HA3;P4HA3	blue	0.2106245	0.59515758	0.16362358	0.19682208	-0.3677103	0.22644109
cg23915349	IGDCC4	blue	0.00982225	0.40871403	0.2350771	0.25965677	-0.3308929	0.0455541
cg14073722	CA10;CA10;CA10;CA10;CA	blue	-0.3952169	-0.5955236	-0.0860185	-0.1620362	0.35132718	-0.5265718
cg13796952	INSR	blue	0.27048779	0.50528532	0.07846748	0.31232175	-0.1268325	0.189763
cg11113760	MIR1258;ZNF385B	blue	-0.5046201	-0.5432653	-0.075547	-0.2420727	0.19830568	-0.3289078
cg04817496	CLSTN1	blue	0.26794639	0.37405341	-0.1255277	0.10013291	-0.2288472	0.2783115
cg21975540	ADK;ADK;ADK	blue	-0.4312012	-0.5328175	-0.1070951	-0.0506884	0.31819367	-0.2590893
cg13486675	ABCG5;ABCG8;ABCG5;AB	blue	0.1435607	0.55249075	0.37636049	0.12657003	-0.3954584	0.06923709
cg15775102	AC011995.1	blue	0.37142027	0.39063292	0.38886954	0.29714251	-0.0804727	0.28859576
cg18149485	TMEM155;TMEM155;AC07	blue	-0.2964572	-0.4207069	0.07744228	-0.0501822	0.25758387	-0.140015
cg22369787	RP11-122D10.1	blue	-0.2185307	-0.5518518	-0.0269728	-0.0775347	0.45012868	-0.1726812

cg13920886	NCAM2;NCAM2	blue	-0.2534209	-0.5898896	-0.035225	-0.0747377	0.37887624	-0.060764
cg21309109	H2AFY2;AIFM2	blue	0.21862127	0.4004758	0.01258943	0.08567917	-0.2824896	0.03023148
cg23414876	HSF4;HSF4;HSF4;HSF4;HS	blue	-0.52078	-0.572022	-0.2390641	-0.2257703	0.25928412	-0.4721927
cg16870215	C4orf26;C4orf26;C4orf26;C4	blue	0.2305947	0.73043511	-0.0937047	-0.0330098	-0.7164716	0.0793752
cg06571075	MACROD2;MACROD2;SE	blue	-0.2031604	-0.6448985	0.06439345	-0.0783028	0.47976566	-0.0295707
cg09213922	CCDC87;CCS;CCS;CCS;CC	blue	0.3307733	0.46832102	0.23073536	0.39416307	-0.2505958	0.27848816
cg24547885	CCDC141;CCDC141;CCDC	blue	-0.22288	-0.5598578	-0.3111047	-0.0524238	0.45911581	-0.1657399
cg15979017	PTH1R	blue	-0.4702834	-0.5743698	-0.1349377	-0.2432653	0.27001102	-0.3105989
cg12353428	ZFP2;ZFP2;ZFP2;RP11-	blue	-0.2768176	-0.6045631	-0.1514921	-0.1270219	0.4967693	-0.3024551
cg16229508	LOXHD1	blue	0.02184002	0.31728287	0.03062157	0.01864595	-0.2252315	-0.0310284
cg14792755	RP11-35N6.1	blue	-0.2431091	-0.6843751	-0.1054673	-0.114143	0.50756758	-0.2043571
cg06555230	ARHGEF38;ARHGEF38	blue	0.20003958	0.34310675	0.37950043	0.12931812	-0.2899693	0.23565825
cg09506954	CCDC130	blue	0.2496965	0.55140875	0.3484999	0.18651541	-0.2732452	0.214413
cg12427404	GPR113;GPR113;GPR113;G	blue	0.12420506	0.34962419	0.19044863	0.19625707	-0.2620191	0.29080005
cg06561835	ZFP112;ZFP112;ZFP112	blue	-0.2755708	-0.6350573	-0.1671949	-0.127842	0.38384342	-0.0356899
cg23505196	FBXO21	blue	0.36124997	0.37738063	-0.0753971	0.3787902	-0.060428	0.30480154
cg01310029	IL5RA;IL5RA;IL5RA;IL5RA	blue	0.32675578	0.54715514	0.08696649	0.68526174	-0.2395764	0.1351067
cg15321231	TPM3;TPM3;TPM3;TPM3;T	blue	0.09561852	0.38352734	0.15305621	0.44844796	-0.1668447	0.09069592
cg09294077	GPHN;RP11-72M17.1	blue	-0.6199149	-0.6087727	-0.1411044	-0.198942	0.17641626	-0.4130528
cg21319323	HOXD-	blue	-0.5894107	-0.6855508	-0.2094256	-0.213106	0.3244716	-0.4302673
cg15345896	HBM;HBZ	blue	0.21847068	0.49342855	0.13342923	0.510666	-0.2468706	0.16656537
cg14275207	STRA6;STRA6;STRA6;STR	blue	0.30912656	0.32745003	0.23657173	0.16146258	-0.2270852	0.45115051
cg03010638	RP11-65J3.3	blue	0.18210227	0.51371531	0.51964024	0.20738149	-0.3024892	0.20500173
cg00916884	MT1M;MT1M;MT1M	blue	-0.1126589	-0.606038	-0.0343964	-0.0329746	0.47140832	-0.0332907
cg22814287	BAI2;BAI2;BAI2;BAI2	blue	0.23839132	0.67607365	0.15699399	-0.0099617	-0.7502052	0.14922654
cg14852384	ACTA1;ACTA1;ACTA1	blue	-0.1539324	-0.6085612	0.10845994	-0.0034886	0.51495442	-0.1155751
cg24825722	DLG4;DLG4;DLG4;DLG4;A	blue	0.33378567	0.566179	0.03303614	0.22739286	-0.2502504	0.32106561
cg07528692	DMRT1;DMRT1	blue	-0.1285661	-0.5936492	0.05654897	-0.097131	0.52168615	-0.1487829
cg00007076	RRS1;RP11-	blue	-0.0883946	0.35576838	0.11026476	0.16419751	-0.2775881	-0.0153401
cg02650215	RP11-181G12.2;RP11-	blue	0.32494507	0.42396673	0.33418374	0.23946561	-0.2043088	0.30911118
cg06316642	BTBD3;BTBD3;BTBD3;BTB	blue	-0.3500019	-0.6145409	-0.0463829	-0.0820238	0.36940291	-0.3491385
cg26650480	RASSF2;RASSF2;RASSF2	blue	0.13897812	0.35478928	0.37836192	0.32161833	-0.2217042	0.09382158
cg13206794	SNED1;SNED1	blue	0.3893957	0.57281669	0.25894855	0.18354369	-0.3829065	0.47498564
cg02517134	EPHA3;EPHA3;EPHA3;EPH	blue	-0.4573358	-0.7310251	-0.1209281	-0.1960393	0.36150639	-0.207323
cg00392242	ARRDC4	blue	-0.3723543	-0.6319148	-0.0004852	-0.2134573	0.27374993	-0.1295876

cg26510017	RP11-	blue	-0.2399721	-0.6016117	0.07834414	0.02799083	0.47010423	-0.109695
cg00942507	C14orf159;C14orf159;C14orf	blue	0.22903058	0.35674226	-0.0043678	0.06791309	-0.227792	0.24672985
cg09221267	AP002380.1;C11orf9	blue	0.36135502	0.50814032	0.24952537	0.15612519	-0.3429392	0.44591195
cg17863326	RP11-44I10.6;SIAH1	blue	0.24076066	0.50833204	0.25549396	0.10254356	-0.338066	0.13056449
cg13632816	SVEP1;SVEP1;SVEP1;SVE	blue	-0.1375082	-0.6988031	0.05570436	-0.0597308	0.5546967	-0.0039897
cg24390233	LAPTM4B;LAPTM4B;LAPT	blue	-0.2903154	-0.4846379	0.0028118	-0.2237207	0.2793843	-0.1816121
cg19083779	PRKAA2;PPAP2B;PPAP2B	blue	-0.5308331	-0.5643095	-0.2523391	-0.2901217	0.14407523	-0.4591521
cg14378889	NPC1L1;NPC1L1	blue	-0.0049172	0.50939404	0.17609246	0.00765876	-0.4670943	0.00779064
cg21285198	TMEM88B;RP4-758J18.8	blue	0.12797614	0.34030834	0.06575652	0.08740366	-0.2419222	0.08608159
cg17926821	MITF;MITF;MITF;MITF	blue	-0.3841723	-0.5624452	-0.319735	-0.1123225	0.37621304	-0.3014671
cg06536988	RP11-768B22.2	blue	0.36234989	0.51250217	-0.0890231	0.428921	-0.2928283	0.03589224
cg21015966	ZNF704	blue	0.07567694	0.51785371	-0.1312147	0.09691571	-0.3972804	-0.0960143
cg05085230	EYA4;EYA4;EYA4;EYA4;E	blue	-0.4989418	-0.5690422	-0.0014924	-0.2193741	0.22547836	-0.3797348
cg06370542	CD4;CD4;CD4;CD4;CD4;C	blue	0.27406975	0.58541334	0.03963765	0.44765387	-0.2560483	0.04828358
cg05355416	ZFP37;ZFP37;ZFP37	blue	-0.3757209	-0.5788058	-0.1047408	-0.1987916	0.29624074	-0.1987623
cg07346739	RP5-1100I6.1	blue	0.28103274	0.39626679	-0.0424389	0.4792951	-0.1748641	0.17732742
cg16625844	GCC1;GCC1	blue	0.32892451	0.41199335	0.03344078	0.17681228	-0.1807996	0.45440237
cg13084574	RP3-414A15.2	blue	0.22980821	0.47140896	0.24592546	0.23213203	-0.2152087	0.09423587
cg00552255	CTD-3049M7.1;CTD-	blue	0.29928597	0.59399767	-0.0877441	0.39089736	-0.2514173	0.13659782
cg19440242	MYO16	blue	0.1999002	0.44395415	-0.0807811	0.13843039	-0.3342177	0.01772884
cg08771084	AL355512.1;RP11-525A16.1	blue	0.24221232	0.42928285	0.13380274	0.00663952	-0.3007422	0.12543008
cg18031307	LBX2;AC005041.17;LBX2	blue	-0.1419176	-0.6523553	0.03718729	-0.0170745	0.5413414	-0.1217015
cg00075967	STRA6;STRA6;STRA6;STR	blue	0.45115665	0.6118232	0.16029566	0.06457961	-0.4936976	0.37525762
cg15084801	ZNF707;ZNF707;ZNF707;Z	blue	0.25633942	0.51615542	0.01187262	0.19545035	-0.3033151	-0.0424651
cg25577099	RP11-342M3.5	blue	0.48980728	0.56658735	0.28555321	0.25110152	-0.2785479	0.46374985
cg13711385	AC010136.2;TNS1	blue	0.27858027	0.46428851	0.07127101	0.16615562	-0.305462	0.2153711
cg18432099	TACR1	blue	-0.2806646	-0.7087821	0.00893211	-0.1029955	0.51842876	-0.1875279
cg08224779	EYA2	blue	0.31436869	0.58336037	-0.0537695	0.06131834	-0.4444317	0.13569944
cg07942598	ADAMTS5	blue	-0.4221119	-0.5495713	0.07026408	-0.0877435	0.28954462	-0.1945632
cg22507304	TMEM63C	blue	-0.3105083	-0.5860684	-0.1443477	-0.1358859	0.44540671	-0.3161827
cg04731710	BNC2;BNC2;BNC2;BNC2;B	blue	-0.2503972	-0.6502099	-0.0831814	-0.1522908	0.43990888	-0.0854271
cg23850212	ZFP28	blue	-0.3659531	-0.696817	-0.0620501	-0.1307317	0.40268111	-0.1255486
cg21632079	RP11-625H11.1;RP11-	blue	0.29782505	0.41505129	0.08525204	0.19637061	-0.2883819	0.56722633
cg21003581	PCLO;PCLO;PCLO	blue	-0.2115139	-0.647301	0.10634943	-0.0165848	0.48284971	-0.0507734
cg05609656	DAAM2;DAAM2;DAAM2	blue	0.38161891	0.50990302	0.27772721	0.19714178	-0.2747284	0.12327736

cg21255171	CDC6	blue	0.23536945	0.55156118	0.34426463	0.22435137	-0.4129282	0.23295577
cg26408426	RALYL;RALYL;RALYL;RA	blue	-0.2083169	-0.6693976	0.20799315	0.00996745	0.50300833	-0.1877232
cg04895904	ENO4;RP11-539I5.2;RP11-	blue	-0.5097453	-0.6832059	-0.1869211	-0.1848316	0.37150192	-0.4764431
cg17246140	HAAO;HAAO;HAAO;HAA	blue	-0.1583098	-0.6993636	0.00292962	-0.0678317	0.5525798	-0.0313741
cg02824202	ARHGAP28	blue	-0.1408681	-0.6131741	0.0853663	-0.006385	0.52262737	-0.000945
cg04928005	AC012087.2;AC012087.2;TH	blue	-0.6290247	-0.6885939	-0.1142951	-0.2434906	0.240058	-0.4058089
cg26595208	MT3;MT3	blue	0.36571942	0.38778089	0.07609058	0.29947621	-0.1429375	0.13468411
cg15194110	LRRN1;SUMF1;LRRN1;LR	blue	-0.4599488	-0.6258513	-0.1635804	-0.1589906	0.3400636	-0.3005008
cg03767970	ZNF362;ZNF362;ZNF362	blue	-0.2626141	-0.6766395	-0.1222817	-0.1276146	0.53760666	-0.2255783
cg18160402	SHISA6;SHISA6;SHISA6	blue	-0.1101731	-0.649955	0.04461138	-0.093801	0.51895043	-0.0515576
cg19777853	CD52;CD52;UBXN11;UBX	blue	0.32405772	0.69087467	-0.0984274	0.01860409	-0.5904205	0.05474369
cg04432319	ISM1	blue	-0.1254777	-0.6789564	0.08978404	-0.0842694	0.53435354	-0.0010538
cg25110782	SHMT2;SHMT2;SHMT2;SH	blue	0.21791085	0.54237233	0.5582246	0.21880204	-0.4420842	0.24102764
cg23988892	C19orf12;C19orf12;C19orf12	blue	0.02093564	0.46809911	0.09579119	0.16100988	-0.3787876	-0.0342469
cg23475294	ADAR;ADAR;ADAR;ADA	blue	0.30651182	0.54554884	0.00110654	0.58637684	-0.285886	0.08069044
cg14060111	ISM1	blue	-0.4452917	-0.7659292	-0.00833	-0.2041456	0.40873132	-0.1311721
cg06610795	AC011995.1	blue	0.14911523	0.6248539	0.32716581	0.18695189	-0.4080454	0.10957429
cg24851539	MDGA2;MDGA2	blue	-0.3936663	-0.6634548	-0.0898175	-0.1441803	0.38155899	-0.2996147
cg14088357	HIF3A;HIF3A;HIF3A;HIF3	blue	-0.3463741	-0.7255311	0.04642477	-0.1520345	0.4639552	-0.163615
cg06473675	ASB18;ASB18;ASB18	blue	0.08934227	0.5696423	-0.064183	0.02291107	-0.5103835	0.03499956
cg00858855	SCAND3;SCAND3;SCAND	blue	-0.4436804	-0.6517209	0.05779323	-0.2038644	0.38856949	-0.1383508
cg17603206	PLXDC2;PLXDC2	blue	-0.2821975	-0.7035514	-0.1801007	-0.0786953	0.55719963	-0.1806858
cg27573017	HILS1	blue	0.23825231	0.60631276	-0.112528	0.37744885	-0.3296959	0.16157602
cg10711857	FAM47E;FAM47E	blue	-0.1778598	-0.4824339	-0.0645818	-0.0320418	0.35290488	-0.0914744
cg00865356	FAM190A;FAM190A	blue	-0.4903696	-0.6177442	-0.2253849	-0.1943204	0.28950682	-0.4264377
cg11398511	KCNK2;KCNK2;KCNK2;K	blue	-0.3609506	-0.5637067	-0.1853197	-0.0838463	0.38110217	-0.1677233
cg24424545	CNNM1;CNNM1	blue	-0.442497	-0.5923292	-0.241983	-0.2075387	0.33278971	-0.3374557
cg27294460	RBMS3;RBMS3	blue	0.2233026	0.42401493	0.52852897	0.16827059	-0.3506023	0.10824504
cg08844849	SIGLEC10;SIGLEC10;SIGL	blue	0.12606709	0.39295777	-0.1372558	0.42957766	-0.2714015	-0.0550387
cg03481509	NEURL1B	blue	0.34507401	0.648897	-0.1525326	0.23952586	-0.3051493	0.10546934
cg04290274	AC005037.2	blue	0.26561828	0.63223583	0.38057587	0.15604776	-0.4817315	0.34016406
cg15492738	SLC6A12;SLC6A12;SLC6A	blue	0.44202055	0.48278846	0.05288627	0.20717389	-0.199555	0.55049941
cg00592779	FXR2	blue	0.36686801	0.58286982	-0.0259318	0.12762602	-0.2645287	0.21635809
cg14151861	RIMS1;RIMS1;RIMS1	blue	-0.5105943	-0.5801416	-0.1189912	-0.2448821	0.21717452	-0.4920087
cg14348756	CTNNA2;CTNNA2;CTNNA	blue	-0.4528699	-0.6404671	0.07674481	-0.1800352	0.31496264	-0.2060832

cg23463356	U1;TEX14	blue	0.12045782	0.33657628	0.12456246	0.18285763	-0.1877982	0.09593844
cg07369881	BRSK2;BRSK2	blue	0.16242529	0.55689383	0.04651192	0.1272166	-0.4304352	0.04238072
cg00592593	MOV10L1;MOV10L1	blue	0.37415593	0.56700707	0.30980844	0.17024953	-0.3655011	0.4586239
cg06802658	MIPOL1;MIPOL1;MIPOL1;	blue	-0.4530306	-0.5210132	-0.2659709	-0.227344	0.28848088	-0.5248203
cg13760683	LINC00494;LINC00494;RP1-	blue	0.47771047	0.67153123	0.15884892	0.14725117	-0.4798165	0.47024421
cg13056552	AC002511.1;AC002511.4	blue	0.23531245	0.65335395	0.10505489	0.44950514	-0.4774025	0.15746168
cg09203880	NAGPA;NAGPA;NAGPA;	blue	0.4497582	0.74014495	0.17180334	0.16615187	-0.469006	0.27167519
cg11219426	RP11-554D14.1	blue	-0.3118106	-0.5487601	0.02285759	-0.091249	0.43252549	-0.143437
cg03745002	TOX3;TOX3;TOX3;TOX3	blue	-0.4977018	-0.5563375	-0.3270973	-0.2789674	0.31236683	-0.4113487
cg17754362	PUM2;PUM2	blue	0.26471064	0.42753095	0.06550836	0.29856367	-0.301669	0.00133153
cg15858994	STRA6;STRA6;STRA6;STR	blue	0.38065729	0.40739196	0.45260994	0.22824191	-0.2584888	0.41930812
cg24775076	OR13A1;OR13A1	blue	0.2141143	0.67643265	0.05201256	0.20911852	-0.4733197	0.02475259
cg05298790	CTD-2216M2.1	blue	0.20977324	0.5206988	-0.3043103	0.13076575	-0.3059658	0.00694809
cg21742876	ITIH1	blue	0.33074411	0.75212829	0.03705896	0.12991091	-0.5750866	0.07858459
cg19688250	RALYL;RALYL;RALYL;RA	blue	-0.2809925	-0.6999734	-0.0281848	-0.0028311	0.5250038	-0.1725266
cg10150421	OCIAD2;OCIAD2;OCIAD2;	blue	0.39152735	0.5384401	0.12191202	0.21095094	-0.36048	0.60643955
cg06091013	FBXO39	blue	-0.1837022	-0.7043611	0.10593258	-0.0366595	0.60824643	-0.090275
cg20000016	JMJD5;CTD-	blue	0.2956616	0.51109154	0.35813604	0.1365458	-0.3559386	0.1758214
cg03225817	GRIA4;GRIA4;GRIA4;GRI	blue	-0.5901596	-0.6517519	-0.118002	-0.2850469	0.2441944	-0.2988551
cg24634420	PDGFC;PDGFC;PDGFC;PD	blue	-0.2598888	-0.6629877	0.0240311	-0.0869606	0.5056522	-0.0831078
cg20311863	BARHL2	blue	-0.2791965	-0.5834027	0.0172086	-0.1231828	0.46586268	-0.4323456
cg03363863	PRSS8;PRSS8;PRSS8;PRSS	blue	0.40412358	0.54831733	0.18193719	0.27824074	-0.3282353	0.63426392
cg24382100	LEMD1;LEMD1	blue	0.36549689	0.55687815	-0.1439473	0.15750959	-0.2611317	0.12845308
cg16972830	NISCH;NISCH	blue	0.37371889	0.48380291	0.14793424	0.60185998	-0.2560237	0.31617272
cg20170777	COX10;COX10;COX10-AS1	blue	0.15413848	0.5510316	0.12792391	0.14983232	-0.4132858	0.18957601
cg12085570	ADAMTS10;ADAMTS10	blue	-0.4238642	-0.784012	-0.1528337	-0.0891289	0.55542937	-0.2047401
cg17282301	RARA;RARA	blue	-0.1120341	-0.5449369	-0.0180189	-0.0618555	0.4455812	-0.0944471
cg20926035	SIX2	blue	-0.1642228	-0.6452331	0.10584252	-0.0505758	0.5173503	-0.1537386
cg09159050	MIB2;MIB2;MIB2;MIB2;MI	blue	0.409858	0.47542601	0.11753508	0.12668954	-0.3379146	0.45303386
cg12911122	RP11-328M4.2;RP11-	blue	-0.5586129	-0.5861738	-0.1001073	-0.1964978	0.20887976	-0.4611239
cg15272034	BIRC3;BIRC3;BIRC3;BIRC3	blue	0.32563362	0.59562938	0.51765485	0.27895595	-0.4773125	0.25469003
cg03026462	FOXA1;TTC6;FOXA1;FOX	blue	-0.2818586	-0.7319532	0.00683206	-0.0563961	0.58889558	-0.0802565
cg20157315	KCNJ14	blue	0.26867722	0.60055863	0.1086046	0.16236096	-0.386696	0.12751172
cg03602280	PIEZO2	blue	-0.3765943	-0.6492748	-0.1250596	-0.1704469	0.41089983	-0.5570751
cg00293757	TMEM55B;TMEM55B;TME	blue	0.21125735	0.52070955	-0.143437	0.12282459	-0.4129763	0.16586039

cg23582054	IL5RA;IL5RA;IL5RA;IL5RA	blue	0.28973231	0.70468426	-0.0105041	0.47086787	-0.4783923	0.05243303
cg06128198	DOCK1	blue	-0.5282	-0.7827353	0.00338806	-0.1596044	0.43429691	-0.1966567
cg07549059	TTK;TTK;TTK;TTK;TTK;TT	blue	0.23934291	0.37232434	-0.0422639	0.3226689	-0.1759705	0.02218185
cg10290814	TNK1;TNK1;TNK1;TNK1;T	blue	-0.1910685	-0.7025839	-0.0485516	-0.0601533	0.62813754	-0.1558171
cg05451884	GPSM1;GPSM1;GPSM1	blue	0.55120025	0.57195315	0.29889375	0.27887478	-0.2343806	0.62405246
cg01060218	NLE1;NLE1;NLE1;NLE1	blue	0.34289161	0.52443973	0.40738453	0.24065887	-0.3988579	0.23171254
cg25218793	RGS12;RGS12;RGS12;RGS	blue	-0.388278	-0.5913122	-0.2148119	-0.0798202	0.46666365	-0.5085564
cg05702747	RP11-830F9.6;RP11-	blue	-0.429802	-0.5383644	0.00392883	-0.3105821	0.35569234	-0.1691929
cg14785542	CTD-2184C24.2;CTD-	blue	0.26329078	0.70639536	-0.0777896	0.22590562	-0.5598787	0.07493201
cg14038650	TRAF3IP3;TRAF3IP3;TRAF	blue	0.411265	0.47851703	0.12545926	0.2907108	-0.2770139	0.30719491
cg22558303	UGGT2;UGGT2;UGGT2;U	blue	-0.4707107	-0.764913	0.0083683	-0.1888068	0.40150828	-0.2182903
cg26803289	NPL;NPL;NPL;NPL;NPL;NP	blue	0.19880767	0.63040869	-0.0800442	0.35073287	-0.4999288	0.01025719
cg08843809	FBXO39	blue	-0.4464704	-0.8132763	0.05630518	-0.1278497	0.5102886	-0.1616114
cg05522252	C5orf50;C5orf50	blue	0.67758834	0.68820051	0.31378553	0.297302	-0.26856	0.44932574
cg20687242	CBX5;CBX5;CBX5;CBX5;C	blue	-0.1739741	-0.6482948	-0.3246054	-0.0587641	0.57821054	0.0329893
cg12750151	OR5D18	blue	0.1151703	0.45800035	-0.2189302	0.01528388	-0.3335362	-0.0783735
cg02244718	RIPPLY2;RIPPLY2	blue	-0.5492215	-0.6330848	-0.0383948	-0.2536296	0.23590795	-0.4831185
cg24290640	LUZP2;LUZP2;LUZP2;LUZ	blue	-0.4401583	-0.6214616	-0.082271	-0.1439264	0.39660843	-0.6097305
cg24252492	ERBB3;ERBB3;ERBB3	blue	0.49278553	0.53899422	-0.0347522	0.24224243	-0.085401	0.22977281
cg26286530	RP11-275F13.1;RP11-	blue	-0.3719458	-0.612453	0.03696143	-0.1319964	0.38861478	-0.4103275
cg16828484	GUCY2D	blue	-0.4133801	-0.4669046	-0.3131641	-0.0567329	0.3657188	-0.2268256
cg08920610	PCSK9;PCSK9;PCSK9;PCS	blue	-0.4383227	-0.673247	0.04035997	-0.0782138	0.46168103	-0.3363616
cg21561970	ISM1	blue	-0.6480778	-0.670628	-0.0988044	-0.2646665	0.30868658	-0.549108
cg19521646	OR1I1	blue	0.22501814	0.69961763	-0.1093701	-0.0079911	-0.5619411	0.05736108
cg19547144	C3orf25;C3orf25	blue	-0.1934315	-0.5589802	-0.0753939	-0.0869747	0.4813192	-0.110696
cg04108875	TIAL1;TIAL1;TIAL1;TIAL1;	blue	0.23732868	0.52672836	-0.0276736	0.22580655	-0.3241376	0.13713909
cg07757163	RP4-	blue	-0.4830297	-0.6544565	-0.0219764	-0.2052857	0.33022541	-0.3442575
cg07300451	RP11-	blue	-0.3433722	-0.688203	-0.1402134	-0.1174219	0.53866137	-0.1979544
cg19266910	ELMOD1;ELMOD1;ELMO	blue	-0.5487353	-0.6512252	-0.1372731	-0.2779002	0.29596359	-0.432124
cg26089577	USP35;USP35;USP35;USP3	blue	0.52210833	0.67015474	0.02861058	0.21473417	-0.3654435	0.32858531
cg25296173	RP11-561P12.5	blue	0.26072224	0.74326445	0.20420837	0.11328445	-0.6213442	0.0847082
cg11893763	GPR26	blue	-0.2671651	-0.6769914	0.18061223	-0.0039657	0.59969148	0.00327927
cg19616230	SLC34A2;SLC34A2;SLC34	blue	-0.2842538	-0.6811895	-0.0485178	-0.1461472	0.51654849	-0.1999972
cg21173239	ELK3;ELK3;ELK3;ELK3	blue	0.29674731	0.48172968	-0.1100461	-0.0167413	-0.3974383	0.20316865
cg20707222	ST6GALNAC3;ST6GALNA	blue	-0.4109284	-0.6736181	0.01741	-0.1404815	0.5385173	-0.3200315

cg20670160	GULP1;GULP1;GULP1;GU	blue	-0.4593649	-0.8103455	0.05112356	-0.1222584	0.53030281	-0.157319
cg18556788	L3MBTL4	blue	-0.6466535	-0.6948805	-0.0385108	-0.2286774	0.24454688	-0.2217875
cg09212118	GUCA2A	blue	0.13868104	0.62715249	0.20851755	0.42070907	-0.4661388	0.04516176
cg09718869	KIAA1984;KIAA1984;KIA	blue	0.31508483	0.45095971	0.1912742	0.04945232	-0.2611471	0.17665134
cg24790628	CHST8;CHST8	blue	-0.3918646	-0.4201999	0.0939497	-0.0468877	0.28425497	-0.1278609
cg15262952	FAM57B;FAM57B;FAM57	blue	0.24255758	0.44693579	0.02393935	0.22123513	-0.2650663	0.05784437
cg11387131	KCNJ5;KCNJ5;KCNJ5	blue	-0.4685396	-0.542564	-0.096242	-0.0973258	0.31400289	-0.0849736
cg23838190	AC004041.2	blue	0.26134366	0.67860422	0.31638946	0.3186611	-0.4991065	0.25094889
cg18320737	CDK18;CDK18;CDK18;CD	blue	0.21016676	0.68347491	-0.0243963	0.02420929	-0.5277963	0.03340791
cg19107642	RP11-279O17.3	blue	0.14898793	0.66575772	-0.0716353	0.04089657	-0.557555	0.02025482
cg11855705	TLR9	blue	0.36045926	0.5724653	0.07606484	0.09257722	-0.4197719	0.17717198
cg16824405	RP11-973D8.3;PMEL	blue	0.35176958	0.52809631	0.35699055	0.27928511	-0.3596568	0.40773501
cg08383262	KALRN	blue	0.3157835	0.71477783	0.04101426	0.19596367	-0.4404838	0.21653471
cg10300440	RP11-1029J19.2	blue	0.26994293	0.7027496	0.2775227	0.11378112	-0.5239036	0.22154866
cg09850980	UGT3A2;UGT3A2;UGT3A2	blue	-0.3554187	-0.7281358	-0.0561859	-0.1475851	0.57682428	-0.1843177
cg15160624	ZNF385D;ZNF385D;ZNF38	blue	0.10236531	0.32664615	-0.0096663	0.08585225	-0.2583514	-0.0336323
cg17435617	RP1-13P20.6	blue	0.02404836	0.43547933	-0.1116537	-0.031746	-0.4039456	-0.1597246
cg09573359	UBE2E1;UBE2E1;UBE2E1;	blue	-0.1609616	-0.7074165	0.00381368	0.02210802	0.62861247	-0.0398313
cg15700022	GPC1;GPC1;GPC1;AC11061	blue	0.65224944	0.66067363	0.07630837	0.33004874	-0.2348841	0.48346222
cg20291548	FBXL8;HSF4;HSF4;HSF4;H	blue	-0.5025261	-0.6340035	-0.0457534	-0.2347831	0.23546272	-0.3976993
cg08736072	C16orf91;C16orf91	blue	0.2962811	0.38634583	0.1417533	0.27329199	-0.0834314	0.05099891
cg11553544	FRMD3	blue	0.4641813	0.54749347	0.17307832	0.28550243	-0.2360277	0.39564071
cg24284973	NTM;NTM;NTM;NTM;NT	blue	-0.3873732	-0.6141949	-0.0893819	-0.1984216	0.43722057	-0.2149919
cg00924388	EMID1;EMID1;EMID1;EMI	blue	0.34974313	0.6892045	0.29213574	0.24081056	-0.4359333	0.24892756
cg10016380	EDNRB;EDNRB;EDNRB;E	blue	-0.4653645	-0.6228188	-0.0495457	-0.1621997	0.35819687	-0.1122339
cg04564030	CDH7;CDH7;CDH7	blue	-0.37631	-0.612228	-0.14291	-0.0675153	0.48468139	-0.105188
cg25055251	CSK;CSK;CSK;CSK;CSK;C	blue	0.37655955	0.55024773	0.14687657	0.24452032	-0.3908239	0.47249638
cg10397440	PENK;PENK;PENK;PENK;	blue	-0.4370949	-0.6483646	0.01903754	-0.1732565	0.3439715	-0.3278507
cg24827032	AC008063.2;DPP4;DPP4;DP	blue	0.28924371	0.55499549	-0.1079411	0.18239965	-0.4296048	0.00028315
cg23564028	CDH11;CDH11;CDH11;CD	blue	-0.5753861	-0.6945945	-0.0976981	-0.2658594	0.31230375	-0.400779
cg02236673	RP11-	blue	0.25561078	0.67271568	0.31423249	0.09662984	-0.5547101	0.22989431
cg01220564	DDC;DDC;DDC	blue	0.17916875	0.55512333	0.21685586	0.11267098	-0.44978	0.18518197
cg09983746	NDUFA4;NDUFA4;NDUF	blue	0.32754473	0.46257508	0.1399912	0.42766559	-0.3288565	0.21603748
cg23473561	AP003064.1;AHNAK	blue	0.30149614	0.64048158	-0.1132175	0.42810497	-0.4657651	0.01273816
cg12708634	U1;KITLG;KITLG;KITLG;K	blue	-0.2932553	-0.752843	-0.0025575	-0.0884113	0.63383342	-0.2194651

cg00720629	TBX1	blue	0.19718446	0.63229878	0.06801541	0.15704652	-0.4482536	-0.0161591
cg17754095	RP11-525A16.1	blue	0.40064725	0.57623319	0.02672816	0.10512401	-0.4241461	0.17940406
cg08105352	SYT6;SYT6;SYT6;SYT6	blue	-0.4894129	-0.674926	0.07766864	-0.197158	0.40321848	-0.3585102
cg09987740	GULP1;GULP1;GULP1;GU	blue	-0.2709502	-0.6838642	0.08712366	-0.0848208	0.49156429	0.01483308
cg06385087	CTSZ;CTSZ;CTSZ	blue	-0.3445422	-0.7018738	-0.0127959	-0.0868836	0.53434056	-0.1056867
cg27300125	AC008074.4	blue	0.17561401	0.70488009	0.15011237	0.36201099	-0.5418842	0.04218856
cg23865698	WFDC1;WFDC1	blue	-0.2524282	-0.6535989	0.1142874	-0.027226	0.56650914	-0.0912291
cg00741836	DOK5;DOK5;DOK5;DOK5;	blue	-0.5713541	-0.6272006	-0.0550506	-0.2045804	0.25015404	-0.4135664
cg09491991	ISM1	blue	-0.6174084	-0.7653213	-0.0149816	-0.2228481	0.37189186	-0.2831497
cg09942364	SYT6;SYT6;SYT6;SYT6	blue	-0.4461286	-0.7107778	-0.0454462	-0.2192515	0.48752708	-0.3578831
cg13779162	ANO1-AS2;ANO1	blue	0.20955865	0.65992848	0.21448451	0.11378107	-0.5737309	0.24267751
cg27217253	CCDC42	blue	0.41754426	0.60600095	0.10442273	0.19251309	-0.4198631	0.16117913
cg26593267	F7;F7;F7	blue	-0.2905178	-0.4858782	0.04535668	-0.0712903	0.3548431	-0.240607
cg10154779	CLU;CLU	blue	0.30283483	0.68380839	-0.0302765	0.40688891	-0.5321469	0.05404305
cg08831034	ANO7	blue	0.46681205	0.64520341	0.22773173	0.19118284	-0.4034568	0.57122716
cg22442197	RPS2;RPS2;RPS2;RPS2;RPS	blue	0.07698915	0.63506175	0.14837111	0.0902124	-0.572464	0.00327378
cg11764900	DNAH8;DNAH8	blue	-0.2319374	-0.7134722	-0.0220245	-0.1126148	0.5735922	-0.1258717
cg14673618	AC012354.5	blue	-0.3308531	-0.7475043	-0.0590287	-0.0707778	0.59447997	-0.3232947
cg04476152	MLPH;MLPH;MLPH;MLPH	blue	-0.4366776	-0.7257871	0.00994878	-0.1608056	0.42802527	-0.331437
cg11081049	HNMT;HNMT;HNMT;HNM	blue	0.17696155	0.54276366	-0.1064083	0.34761415	-0.2931108	0.02630477
cg27602153	LRAT;LRAT;LRAT;LRAT;L	blue	-0.5361832	-0.6819103	-0.0152696	-0.1518542	0.36519746	-0.4105512
cg12664209	ISM1	blue	-0.4850769	-0.7820309	-0.0111217	-0.179818	0.43166991	-0.1671058
cg05083839	RP3-454B23.1	blue	0.18588021	0.66635875	0.26088051	0.01875416	-0.5370599	0.13544301
cg02499494	RP11-	blue	-0.3149805	-0.7525891	0.08840073	-0.0623806	0.57017983	0.01840438
cg12349832	C17orf28;C17orf28	brown	-0.2986387	-0.3870717	0.10194687	0.3528404	0.54593849	0.0297674
cg14162806	PDGFB	brown	-0.3468763	-0.5505526	0.24588661	0.13631353	0.59711371	0.07185487
cg09503853	TSPYL5	brown	0.498591	0.52413187	-0.1205388	-0.1248194	-0.5571111	0.05784571
cg14871186	USP7;USP7;USP7;USP7;US	brown	-0.2306777	-0.2869423	0.28533341	0.5421147	0.42374415	0.07952398
cg17485531	USP49;USP49;USP49;USP4	brown	-0.3775343	-0.395869	0.34197707	0.21652338	0.368265	-0.0037686
cg00984985	BCKDHA;CTC-	brown	-0.0064943	-0.4413579	0.34674017	0.40996942	0.75886625	0.35097584
cg05377527	NDUFS3;PTPMT1;PTPMT1;	brown	-0.3748417	-0.3196204	0.30134014	0.13895211	0.33373884	-0.0072883
cg05977840	LIMK2;LIMK2;LIMK2;LIM	brown	-0.1823345	-0.4756707	0.40814723	0.26872703	0.59559956	0.18414244
cg18571531	SHANK2-AS1	brown	0.08260475	-0.2358822	0.38730786	0.32012882	0.42895236	0.39971093
cg24922045	GAS2L2	brown	-0.3623118	-0.6011902	0.12905298	0.14265904	0.61187473	0.17102976
cg20815919	MGLL;MGLL;MGLL;MGLL	brown	-0.3179164	-0.3363468	0.27089127	0.34286483	0.54338876	0.06750654

cg07532405	TRAJ56;AE000661.37;AE00	brown	0.4982156	0.70560645	0.02655779	-0.0172142	-0.6867638	0.08961534
cg18935506	UTP6	brown	0.1753822	0.60553862	-0.2421037	-0.2322945	-0.6411135	-0.2003326
cg06169270	TTLL6;TTLL6;TTLL6;TTLL6	brown	-0.0862997	-0.3753432	0.28658811	0.5914989	0.70326883	0.15166222
cg05539847	HSD3B7;HSD3B7;HSD3B7;	brown	0.01432752	-0.4208178	0.37832297	0.54841736	0.72160453	0.42528938
cg07589968	KCNS1;KCNS1	brown	-0.3511756	-0.5662064	0.24820615	0.21432479	0.6836859	0.02526427
cg12902625	PYGM;PYGM;PYGM	brown	-0.491985	-0.5043429	0.29766425	0.12467615	0.46925449	-0.0270183
cg15804598	HEXIM1	brown	-0.3529236	-0.6147576	0.26443548	0.3285947	0.78767231	0.030987
cg17056853	BLM;BLM	brown	0.33215372	0.61177875	-0.0884125	-0.156716	-0.6923953	-0.0318008
cg07175985	SACS	brown	-0.385237	-0.542795	0.26467728	0.12643456	0.48882827	0.00624557
cg09212407	NANOS3;NANOS3	brown	-0.3246059	-0.4744385	0.26853691	0.18669061	0.57325337	0.12598294
cg08727202	MPST;TST;MPST;MPST;M	brown	-0.1072278	-0.4548296	0.38132198	0.26660686	0.66319997	0.26424856
cg03723356	CDK5RAP3;CDK5RAP3	brown	-0.0421836	-0.2082847	0.56995895	0.55112788	0.43862321	0.16254498
cg17487559	CATSPERB;CATSPERB;CA	brown	0.46269773	0.77184426	-0.0410064	-0.0906021	-0.7548337	0.0853969
cg26426448	CREB5	brown	0.43141186	0.58046653	0.02412011	-0.1798941	-0.601692	0.03461414
cg14484628	SEPT1;SEPT1;ZNF48;ZNF4	brown	-0.2936563	-0.6795797	0.20645073	0.25451708	0.71141966	0.18060835
cg00940972	TRAJ52	brown	0.47602755	0.71174133	-0.1141816	-0.1225395	-0.6740637	0.11767351
cg03537184	CLASP2;CLASP2;CLASP2;	brown	0.37797203	0.74798266	-0.1040808	-0.1167786	-0.7722378	-0.0423772
cg04721651	MGLL;MGLL;MGLL	brown	-0.0133015	-0.3025389	0.29008468	0.73041605	0.5644733	0.23056319
cg00961640	SLC16A4;SLC16A4;SLC16	brown	0.52758763	0.66859009	-0.1903781	-0.0289971	-0.6400086	0.00568124
cg15033653	CCDC42B	brown	-0.2122135	-0.4461276	0.1753097	0.23881753	0.71076629	-0.0030878
cg19380072	BCKDHA;BCKDHA	brown	-0.0958706	-0.2074017	0.31802877	0.38134481	0.4789057	0.25819323
cg14869043	CTD-2113L7.1	brown	0.4822375	0.69541905	-0.131781	-0.1028948	-0.6426793	0.11819864
cg16935794	MRPS27;MRPS27;MRPS27	brown	0.36160722	0.60556718	-0.0462408	-0.1062069	-0.7857034	0.0622578
cg16570051	AEBP1	brown	-0.2794319	-0.5628388	0.22704215	0.29107864	0.70755907	0.14349483
cg06496197	SNORA70	brown	-0.1158431	-0.2985249	0.24415145	0.45844915	0.47332424	0.13927578
cg14446588	IQGAP3;IQGAP3	brown	-0.3149855	-0.5986609	0.13573591	0.19467051	0.67459315	0.07293886
cg16021126	SERP2;SERP2;SERP2	brown	-0.3501958	-0.5612679	0.27566316	0.18952066	0.64984301	-0.0907427
cg23157190	HK2;HK2;HK2	brown	-0.1816166	-0.2636485	0.347277	0.19117504	0.41917557	0.19817538
cg16440069	CELF1;CELF1;CELF1;CELF	brown	0.40594401	0.63963668	-0.0935897	-0.0182714	-0.6703526	0.1106889
cg18968475	MLIP-IT1	brown	-0.3787938	-0.3619997	0.28948731	0.2806546	0.52850312	-0.0137313
cg08640361	SGK1;SGK1;SGK1;SGK1;S	brown	0.44142087	0.64520158	0.02616395	-0.0836673	-0.6080149	0.05602368
cg04351156	PDE4A;PDE4A	brown	-0.3543953	-0.4879372	0.4278124	0.20460905	0.49827472	0.11589886
cg01656853	FUT2;FUT2;FUT2	brown	-0.3073439	-0.4789791	0.23297904	0.16048327	0.49330615	0.13001615
cg21199629	TRAJ38	brown	0.38335729	0.6774168	-0.0827349	-0.0616194	-0.6755673	-0.0414098
cg02389631	SHANK2;SHANK2	brown	-0.3908125	-0.5987277	0.12635494	0.2388532	0.63945607	0.00924212

cg23732576	RP11-672A2.5	brown	-0.1910005	-0.5213874	0.25290242	0.19388038	0.64383378	0.05229769
cg07529381	CDK5RAP3;CDK5RAP3	brown	-0.1149202	-0.2117103	0.52248838	0.4919213	0.3980661	0.1501124
cg08087541	CD151;CD151;CD151;CD15	brown	-0.1332958	-0.2901366	0.43678586	0.57316363	0.54215957	0.21585967
cg08869118	MLXIP;MLXIP;MLXIP;ML	brown	-0.3870395	-0.6753065	0.19248389	0.12804689	0.79977531	0.04301444
cg02086349	MTMR11;MTMR11;MTMR	brown	-0.1941745	-0.3062265	0.19845534	0.16968813	0.3264471	0.06378155
cg19578864	STX18;STX18;STX18;STX1	brown	0.4627148	0.66258115	0.02958534	-0.1016869	-0.7482773	0.15299038
cg16787643	PPAPDC3;PPAPDC3	brown	-0.2111272	-0.0973	0.41412548	0.22721737	0.22420572	0.08054672
cg06697744	MACF1;MACF1;MACF1	brown	-0.1479495	-0.4954314	0.29726848	0.53938129	0.78877498	0.23514303
cg13333788	ZDHHC18;ZDHHC18	brown	0.24479067	-0.0849848	0.29724727	0.72999982	0.49806527	0.46230551
cg11637005	ENTPD1;ENTPD1;ENTPD1;	brown	-0.4923426	-0.547512	0.2063738	0.04314431	0.48696072	-0.0613643
cg03624510	KCTD6;KCTD6;KCTD6	brown	-0.388869	-0.2666457	0.19599505	0.1337886	0.30674118	-0.0921941
cg06295590	RP4-697K14.7	brown	-0.5296561	-0.6271786	0.07713124	0.06674403	0.63228149	-0.194641
cg20393839	CEBPB	brown	0.09001967	-0.3709748	0.27159344	0.6610409	0.70799706	0.36113448
cg14103343	MAMSTR;MAMSTR	brown	-0.3605196	-0.5381334	0.36630544	0.19738097	0.59480404	0.000168
cg23864881	GAS2L2	brown	-0.4058698	-0.5239296	0.03788697	0.07542795	0.48009792	0.06506329
cg07066019	C17orf107;CHRNE;CHRNE;	brown	-0.254663	-0.2134954	0.32351096	0.44401191	0.3813661	0.12264809
cg23898792	NDUFB9;NDUFB9;NDUFB	brown	-0.1886112	-0.4616628	0.18138328	0.46993819	0.74792896	0.1598863
cg17935233	RHBG;RHBG;CCT3;CCT3;C	brown	-0.3896025	-0.5922625	0.1944369	0.2106876	0.58545973	0.10009118
cg25317774	ZNF574	brown	-0.2507465	-0.4815219	0.55791733	0.28757775	0.6243143	0.21739752
cg09755932	ANKRD33;ANKRD33;ANK	brown	-0.2495539	-0.3768695	0.09671971	0.11940208	0.36104526	0.03611888
cg12234455	MAMSTR;MAMSTR	brown	-0.3283792	-0.4581608	0.35134712	0.2701063	0.56136121	0.08923936
cg03691549	TENC1;TENC1;TENC1;TEN	brown	-0.0611109	-0.442545	0.15066231	0.24327157	0.76892738	0.18066911
cg04660111	FUT2;FUT2;FUT2;FUT2;FU	brown	-0.2493121	-0.3566626	0.2614455	0.06539827	0.24409845	0.13400672
cg11397301	SCG5;SCG5;SCG5	brown	0.17298013	-0.1261217	0.44280678	0.52499371	0.49293849	0.31929359
cg17424856	ANKRD33;ANKRD33;ANK	brown	-0.174972	-0.3796827	0.18890534	0.2242266	0.4199498	0.16921888
cg23190823	RUSC1-	brown	-0.0496695	-0.5626121	0.21005026	0.38404921	0.89563117	0.13278552
cg24059786	SEMA4A;SEMA4A;SEMA4	brown	-0.2901967	-0.6121596	0.24425628	0.37593179	0.81152608	0.05931447
cg21219443	MED13L;MED13L	brown	-0.3374642	-0.4264172	0.22873456	0.21852401	0.5148591	0.01704926
cg09570614	AC068491.1;AC068491.1;A	brown	-0.1405156	-0.5988746	0.22690319	0.20804337	0.77467314	0.12778357
cg10148841	ROBO4;ROBO4;ROBO4;RO	brown	-0.2666665	-0.620539	0.07864081	0.13340136	0.83784794	-0.045517
cg09005171	PLEKHG5;PLEKHG5;PLEK	brown	-0.2064307	-0.4064044	0.29656219	0.23155788	0.61030709	0.07303775
cg02685435	IQCE;IQCE;IQCE;IQCE;IQC	brown	-0.2125472	-0.5479675	0.31521792	0.36465082	0.74012622	0.11198426
cg02319392	RP11-	brown	-0.3515977	-0.4278026	0.23619202	0.25347399	0.51682865	0.08555684
cg11061975	SIRPG;SIRPG;SIRPG	brown	0.37487692	0.66544589	-0.1444958	-0.0964809	-0.7601352	0.07035896
cg09170311	C11orf88;RP11-794P6.6	brown	-0.0930385	-0.3119021	0.21814447	0.21078254	0.37488838	0.17856338

cg15705079	SPG7;RP11-104N10.1	brown	-0.0338939	-0.2537909	0.10155385	0.45257021	0.71792882	0.1583536
cg07879334	SEMA7A;SEMA7A;SEMA7	brown	-0.2135947	-0.1802061	0.53496545	0.24854003	0.27999915	0.14126326
cg04980777	TRAJ57;TRAJ58;AE000661.	brown	0.51959864	0.62579485	-0.063461	0.05087133	-0.5531994	0.13485948
cg16934894	BTG3;BTG3;BTG3;BTG3	brown	-0.2259885	-0.6977619	0.21174792	0.26004138	0.87726242	0.09889578
cg03131054	IQCE;IQCE;IQCE;IQCE;IQC	brown	-0.1527307	-0.5812693	0.2887709	0.3580802	0.85462392	0.13597659
cg06017697	TCF25;TCF25;TCF25;TCF25	brown	-0.2039276	-0.5331573	0.23861803	0.26587079	0.74505225	0.16758441
cg15506264	RP11-736N17.8	brown	-0.5451544	-0.6599385	-0.0332474	-0.0120436	0.59909571	-0.1633005
cg24354799	RAB6A;RAB6A;RAB6A;RA	brown	-0.4604179	-0.604178	0.10477369	0.11509636	0.5772051	-0.0612359
cg08157684	SYTL1;SYTL1;SYTL1	brown	-0.2614264	-0.3689904	0.23486815	0.00060974	0.42164379	0.03969343
cg00759456	RHBDD1;RHBDD1;RHBD	brown	0.43675064	0.5046095	-0.1085191	-0.1109865	-0.6096057	0.04458666
cg16323609	PRKAR2A-AS1;PRKAR2A-	brown	-0.2158557	-0.1258657	0.15629817	0.27423381	0.28845861	-0.006677
cg00885460	MMP15	brown	-0.2653863	-0.4700307	0.23746033	0.25989142	0.55128654	-0.0582682
cg12610801	GLYCTK-AS1;GLYCTK-	brown	-0.4498238	-0.6762938	0.2982731	0.18427741	0.72239336	0.05507721
cg13356734	LPAR5;ACRBP	brown	-0.120662	-0.2878853	0.27349746	0.58029264	0.61601082	0.09535693
cg01950449	MTUS2;MTUS2;MTUS2	brown	-0.5189809	-0.5231425	0.03189818	0.10499678	0.52948413	-0.2376204
cg20422157	C7orf43;C7orf43;C7orf43;C7	brown	-0.3067883	-0.6267262	0.29201772	0.14726247	0.64545913	0.03441145
cg06639488	EFNA1;EFNA1;EFNA1	brown	-0.3649825	-0.6718956	0.188063	0.14480832	0.78819644	0.07027738
cg25702859	RP11-736N17.8	brown	-0.4630401	-0.4925992	-0.0386115	-0.0020704	0.47729635	-0.0922901
cg15937073	HIVEP3;HIVEP3;HIVEP3;H	brown	-0.1107913	-0.3111311	0.18911067	0.15579615	0.45133536	-0.0112323
cg23452957	ADAM8;ADAM8;ADAM8;	brown	-0.175058	-0.3865477	0.39911199	0.45842501	0.54326638	0.20686527
cg10763288	DHCR7;DHCR7;DHCR7;D	brown	-0.2503021	-0.5572013	0.3982748	0.23738528	0.68404396	0.24412139
cg06900004	NKIRAS1;NR1D2;NR1D2;N	brown	-0.1859386	-0.4478927	0.04116694	0.41450302	0.68662137	0.06042053
cg18066055	WBSCR22;STX1A;STX1A;S	brown	-0.2559318	-0.5706025	0.26226014	0.19140648	0.65379964	0.2719063
cg08235798	RAC2;RAC2;RAC2;RAC2;R	brown	-0.2852749	-0.4822343	0.05404184	0.17048893	0.56622125	-0.013372
cg03045620	SLC44A4;SLC44A4;SLC44	brown	-0.1183713	-0.4753065	0.3383428	0.37414841	0.64913657	0.20522444
cg01709977	MST1R;MST1R;CTD-	brown	-0.4406817	-0.6351945	0.21445708	0.06099416	0.58442264	0.00061329
cg05965700	RP11-303E16.6;RP11-	brown	-0.191455	-0.4221747	0.33638862	0.27471951	0.49117136	0.13947498
cg02020911	MYADM;AC008440.10;MY	brown	-0.4169794	-0.6567515	0.12387601	0.15410352	0.72734725	-0.1207381
cg20267005	TYROBP	brown	-0.2284103	-0.5705551	0.25758577	0.14774658	0.66494622	0.14923364
cg00785312	RP11-174G6.1	brown	0.02242746	-0.3840677	0.34008414	0.50456792	0.71185284	0.30310675
cg06697074	PRKAR2B	brown	0.32341868	0.61557143	-0.2143108	-0.0599382	-0.6606809	-0.0926457
cg01522717	RP5-1022P6.4	brown	0.00618662	-0.3337733	0.32082687	0.48462122	0.63680884	0.3034418
cg13455960	RGMA;RGMA;RGMA;RG	brown	-0.33954	-0.6102789	0.23343023	0.09418205	0.59096661	0.07296432
cg00774999	OPLAH;OPLAH;OPLAH	brown	-0.4665276	-0.7069245	0.13041087	0.09625843	0.68837675	-0.0715755
cg08510096	RP11-387D10.3;RP11-	brown	0.0793944	-0.358157	0.30797964	0.712325	0.66634695	0.28280697

cg18687845	HNRNPH3;PBLD;PBLD;PB	brown	0.46382327	0.59332014	-0.0350419	-0.0801502	-0.6678851	0.08175812
cg21714980	BLM;BLM	brown	0.29797143	0.69166151	-0.2569918	-0.0917987	-0.6973592	-0.1076853
cg20580088	HIF1A;RP11-	brown	-0.3554512	-0.640801	0.35125513	0.11974446	0.58792048	0.05422853
cg13668490	ITGA2B	brown	-0.2700327	-0.2729372	0.25314858	0.29466468	0.36453554	-0.0132932
cg04446579	ANKRD45;ANKRD45	brown	-0.3912112	-0.8023983	0.18395689	0.10711586	0.8148707	0.01563285
cg07685357	MIR125B1;RP11-	brown	-0.0984716	-0.1686174	0.21634373	0.3828299	0.30971894	0.15332063
cg14234442	ARHGAP18;RP11-73O6.4	brown	-0.4199471	-0.5385142	0.09464818	0.19043259	0.51897948	0.03318619
cg00770599	SLC26A8;SLC26A8;SLC26	brown	-0.1635793	-0.6013728	0.1497603	0.23313114	0.88167341	0.17763193
cg05748295	TRAF5;TRAF5;TRAF5;TRA	brown	0.29571709	0.54469902	-0.3168826	-0.1972303	-0.6270765	-0.1089265
cg03846113	RP11-	brown	0.3616184	0.63064673	-0.0484316	0.01324102	-0.6194541	0.01279688
cg13592485	EPHX4	brown	-0.4897173	-0.4873462	0.20760113	0.03858312	0.42709863	-0.0779022
cg06913600	HAAO;HAAO;HAAO;HAA	brown	-0.1219221	-0.3454554	0.35557764	0.14546869	0.40293471	0.27654489
cg11712386	AL844908.5;ITGB2;ITGB2;I	brown	0.34012129	0.66954817	0.01936349	-0.0978204	-0.6943896	0.06910372
cg06835483	ALOX12B	brown	-0.1768924	-0.4170985	0.12485564	0.34261999	0.66331479	0.09545791
cg19534551	BMP4;BMP4;BMP4	brown	-0.3814821	-0.5452303	0.12862332	0.15230775	0.55921607	-0.0736548
cg24902269	RP11-251M1.1;EGFL7	brown	-0.4203803	-0.7193812	0.08077275	0.09402158	0.66696256	-0.0659377
cg13537328	PTPN18;PTPN18;PTPN18;P	brown	-0.3552577	-0.3748545	0.18248375	0.23921167	0.46264534	0.00966496
cg02484491	Y_RNA;RREB1;RREB1	brown	0.12438262	0.46783368	-0.3475608	-0.2134233	-0.5181603	-0.2366779
cg26079959	SLC15A4;SLC15A4;SLC15	brown	0.18626207	0.62031231	-0.1316474	-0.1287848	-0.6516545	-0.0844675
cg12235672	USP7;USP7	brown	-0.1075454	-0.1109558	0.29508996	0.50042063	0.33829665	0.13264085
cg17560311	C2;C2;C2	brown	-0.3741427	-0.3571911	0.33863255	0.2075478	0.39947917	0.1184904
cg08900098	KCNH2;KCNH2;KCNH2;K	brown	-0.031757	-0.305205	0.51615484	0.65977251	0.52242571	0.30603716
cg21833837	KAZN;KAZN	brown	-0.4588563	-0.4036187	0.17003298	0.10495094	0.45363326	-0.1637598
cg06436903	ELFN2;ELFN2;RP1-	brown	-0.356483	-0.7095093	0.07567053	0.09801511	0.80140061	0.02287566
cg16404106	CCDC81;CCDC81;CCDC81	brown	-0.2745464	-0.5003517	0.21372326	0.1837016	0.47680483	0.05743118
cg24912565	GBAP1;GBAP1;GBAP1;GB	brown	0.02171271	-0.4227721	0.08284029	0.43777511	0.80529646	0.16591146
cg03236948	DNAJC4;DNAJC4;DNAJC4	brown	-0.2748438	-0.4713425	0.23349686	0.20721183	0.5217258	0.07135219
cg08198540	TRAJ43;TRAJ44	brown	0.37212622	0.76142573	-0.029579	-0.1468266	-0.8543086	0.00147119
cg24130673	CAPN11;CAPN11	brown	-0.1984978	-0.5570506	0.11694068	0.17247265	0.76538986	0.08922632
cg19184414	DNMT3B;DNMT3B;DNMT	brown	-0.494074	-0.6213918	0.23590272	0.14149688	0.663356	-0.1397407
cg06998507	KCNG4;KCNG4	brown	-0.3458462	-0.4580774	0.16136783	0.26458402	0.50004905	0.06514563
cg01495604	SH2D4A;SH2D4A;SH2D4A	brown	-0.3865058	-0.3508643	0.0945526	0.09127078	0.34370619	-0.1589274
cg12777992	ZNF300;ZNF300;ZNF300	brown	-0.2616228	-0.6843458	0.23041943	0.11604786	0.67347814	0.16248822
cg06008378	RP11-	brown	-0.2389304	-0.5659629	0.30230855	0.26866718	0.69601613	0.10393948
cg26845395	CIRH1A;RP11-70O5.1	brown	0.24687652	0.71821722	-0.1260765	-0.1647539	-0.8874029	-0.0594544

cg00751072	SSBP4	brown	-0.4172228	-0.6057768	0.12387609	0.15897057	0.70212695	-0.0598819
cg23029292	TRAJ29	brown	0.52768103	0.60661725	0.259168	0.00595724	-0.6079808	0.14897297
cg22968401	PIPOX;PIPOX;PIPOX;PIPO	brown	-0.1912665	-0.4718148	0.26960596	0.21306971	0.54945263	0.13892454
cg20694863	RP11-860B13.3;KLHDC5	brown	0.53989254	0.76416068	-0.1598543	-0.0275192	-0.6784927	0.09240763
cg08899203	RASGRP3;RASGRP3	brown	0.49854873	0.54151473	-0.1776389	-0.0322341	-0.4587849	0.07247728
cg07891507	PLCH1	brown	-0.2630243	-0.5612264	0.2213147	0.19159921	0.53192186	-0.006194
cg24644201	CREB3L1;CREB3L1	brown	-0.3602261	-0.5879835	0.04513554	0.07282308	0.65136079	0.01731248
cg23055674	PILRB;PILRB;PILRB;PILRB	brown	-0.0721002	-0.6079854	0.27760496	0.39537294	0.90265286	0.27830108
cg23909147	ERCC6;RP11-123B3.6;RP11-	brown	-0.2795222	-0.5935241	0.16833631	0.21407815	0.64139915	0.04255024
cg11855332	TKT;TKT;TKT;TKT	brown	0.00762879	-0.33204	0.28617526	0.58190799	0.63288278	0.21072039
cg19080638	TRAJ29	brown	0.55913539	0.80272151	0.08689157	-0.031744	-0.7653594	0.16476605
cg09210323	AP3B1;AP3B1	brown	0.35648809	0.42071657	0.24591366	-0.0670645	-0.4314987	0.17557053
cg15209695	TMED10;TMED10;TMED10	brown	0.13765135	-0.2484759	0.08455156	0.45269286	0.65584307	0.2298614
cg19560014	METTL20	brown	-0.0337043	-0.319489	0.06256751	0.17776499	0.48926326	0.12281984
cg17618111	PPP1R26	brown	-0.4670827	-0.5109324	0.0927909	0.17848757	0.51810649	-0.2161293
cg05364361	SSBP3;SSBP3;SSBP3;SSBP	brown	-0.405777	-0.7297223	0.11065813	0.23613264	0.85217034	-0.0324379
cg03239580	CYP21A1P	brown	-0.455782	-0.5474781	0.20118578	0.08260907	0.58768938	-0.0926816
cg08325568	PPP1R12B;PPP1R12B;PPP1	brown	0.27399593	0.60323319	-0.2826781	-0.2695369	-0.8017544	-0.0622607
cg20728292	CEP85L;CEP85L	brown	0.48069938	0.67504273	0.04883753	-0.0392413	-0.6640055	0.17653209
cg09114528	RP11-503N18.1	brown	-0.2996788	-0.3123061	0.23854311	0.28349369	0.4489866	0.0704915
cg14494745	PDE2A;PDE2A;PDE2A	brown	-0.2683428	-0.4447312	0.35231583	0.31646224	0.64955958	0.14274753
cg23338977	C17orf61;NLGN2;NLGN2	brown	-0.3595701	-0.6354654	0.09370836	0.146594	0.65105999	0.03102496
cg14124980	RBM4;RBM14-	brown	0.00832852	-0.2596345	0.44404264	0.44353385	0.61995432	0.24763165
cg25215011	PLCL2-AS1	brown	0.33119786	0.78330006	-0.0284316	-0.1343395	-0.8812341	0.03651119
cg10539274	RASSF6;RASSF6;RASSF6;	brown	-0.313511	-0.4844487	0.15827635	0.19312708	0.54595073	0.04444352
cg12630354	CIITA;CIITA;CIITA;CIITA;	brown	-0.4038867	-0.2600321	0.22062974	0.02658203	0.29431667	-0.025493
cg06972695	PRR5;PRR5;PRR5;PRR5-	brown	-0.437319	-0.6164247	0.08614401	0.11872845	0.66388632	-0.0973335
cg24477583	FTSJD2;FTSJD2;FTSJD2	brown	0.46289323	0.59005377	0.0117507	-0.0532879	-0.6659563	0.04261239
cg03172991	NFIX	brown	-0.2226342	-0.4332336	0.40304852	0.35869828	0.64088186	0.12494111
cg18567504	VTI1B;VTI1B;VTI1B;RP11-	brown	-0.1909122	-0.3797602	0.39243992	0.14687536	0.31961406	0.50340403
cg06412996	RP11-25C19.1	brown	0.41166405	0.60678269	-0.0307565	-0.0118279	-0.6426598	0.07020412
cg07103510	SEC14L6;MTMR3	brown	-0.1061955	-0.4538303	0.26358824	0.30318885	0.81908281	0.13263808
cg08709434	ACER2;ACER2	brown	-0.3099314	-0.3645326	0.07261579	0.20754225	0.40153248	-0.0447376
cg27625732	TBC1D13;TBC1D13;TBC1D	brown	0.00802564	-0.378354	0.50087287	0.41368391	0.5529206	0.31741197
cg25933401	RP11-	brown	-0.479188	-0.5157562	0.1543868	0.18529204	0.58616416	-0.1343252

cg24392274	C9orf16;C9orf16;C9orf16;C9	brown	-0.3403823	-0.4832398	0.19692289	0.10243753	0.52652795	-0.0286502
cg22708914	SH2D4A;SH2D4A;SH2D4A	brown	-0.5183533	-0.6111227	0.02392714	0.05082909	0.55757114	-0.1717936
cg19083497	ZNF474;ZNF474;ZNF474;Z	brown	-0.2660908	-0.4447679	0.1352118	0.06298481	0.56352246	0.07437233
cg23791076	TOP1MT;TOP1MT;U6;TOP	brown	-0.0554664	-0.2562601	0.34383108	0.54484162	0.34371698	0.2801279
cg18835815	ATP6V1H;ATP6V1H;ATP6	brown	0.44170465	0.75326701	-0.1041938	-0.1107591	-0.7109219	0.0709652
cg02114747	TRDV2;AE000661.37;AE000	brown	-0.3014278	-0.5691434	0.15363517	0.31049501	0.71750616	-0.0600848
cg04002806	GRASP	brown	-0.4644993	-0.5217182	0.20726957	0.10000571	0.45824824	-0.0116309
cg07719604	E2F4;ELMO3;ELMO3;ELM	brown	-0.1812831	-0.5961097	0.20874846	0.29433143	0.81055036	0.12574372
cg06605897	RP11-407B7.1;RP11-	brown	-0.2345946	-0.5479017	0.31583983	0.17896921	0.67152346	0.10737269
cg09452432	DEAF1;DEAF1	brown	0.37092278	0.64746193	-0.1403298	-0.0852918	-0.6756823	0.05735516
cg05256971	MYT1L	brown	-0.1702326	-0.3809429	0.1723283	0.23583271	0.57752043	0.12683466
cg20112500	HCG22;HCG22;HCG22;HC	brown	-0.3310614	-0.6570595	0.08098216	0.13199852	0.70025688	0.05638433
cg22437284	SRC	brown	0.35923773	0.4491777	-0.3002916	-0.0630792	-0.5705482	-0.0024744
cg08321827	AC002398.5;AC002398.5;A	brown	-0.0445004	-0.1634011	0.55321522	0.43832822	0.3121834	0.25682987
cg18995064	MINK1;MINK1;MINK1;MI	brown	-0.177139	-0.6006605	0.16448525	0.21474423	0.79393255	0.21617205
cg22467301	GNAI2	brown	-0.3585301	-0.4609148	0.33537107	0.21160363	0.46731239	0.03894569
cg20143092	CD44;CD44;CD44;CD44;CD	brown	-0.4177641	-0.4924721	0.08741898	0.05912988	0.53493438	-0.1812093
cg23454061	TAF6;TAF6;TAF6	brown	-0.2184212	-0.4650994	0.16619983	0.30435697	0.69398947	0.12046062
cg04944545	LY6G6D;LY6G6E;LY6G6E;	brown	0.37602894	0.70727522	0.02787529	-0.0942557	-0.845405	0.10516155
cg06171633	LTK;LTK;LTK;LTK;LTK	brown	-0.5814188	-0.63986	0.13721973	-0.0095415	0.56733282	-0.151581
cg02315509	ZBED2	brown	0.26413474	0.76755619	-0.1131518	-0.1972351	-0.8926774	-0.0306927
cg16252872	PDE4DIP;PDE4DIP;PDE4DI	brown	-0.4598648	-0.7184727	0.04100414	0.05817899	0.71168687	-0.2168991
cg03214715	MARS;ARHGAP9;ARHGA	brown	-0.2894287	-0.408889	0.37787603	0.30834996	0.6004147	0.04245686
cg16638238	TINAGL1;TINAGL1;RP11-	brown	-0.3079972	-0.6660174	0.20459888	0.14641705	0.71325258	0.0812376
cg26824780	RP11-830F9.7;RP11-	brown	-0.2754998	-0.4804008	0.43387119	0.2505459	0.47941081	0.10711657
cg13096434	SYN3;SYN3;SYN3	brown	-0.0176479	-0.2590778	0.32090286	0.53041857	0.69312011	0.16900862
cg16435459	CRK	brown	-0.0692751	-0.280008	0.34962186	0.29814556	0.63942165	0.20612862
cg10498429	GPR137;GPR137;GPR137;G	brown	-0.4292093	-0.5086671	0.13172026	0.08827578	0.57154706	-0.1149615
cg09343458	MNT;MNT;MNT;RP1-	brown	-0.1032018	-0.2296946	0.37481077	0.36404088	0.38543	0.16192817
cg01803808	KCNAB1;KCNAB1;KCNA	brown	-0.2073979	-0.4109578	0.29685425	0.20561126	0.43389264	0.17846334
cg00291142	FAM117B;FAM117B;FAM1	brown	0.39216724	0.62562845	-0.0339595	-0.1157101	-0.7780049	0.0121465
cg09348347	ERBB3;ERBB3;ERBB3;ERB	brown	-0.2456419	-0.4071333	0.19212915	0.23096216	0.73535365	0.07185
cg11104252	TEX33;TEX33;TEX33	brown	-0.2733331	-0.4610344	0.24401551	0.2657146	0.52790743	0.09879037
cg05242244	CSAD;RP11-	brown	-0.3686313	-0.2680093	0.14678529	0.13576909	0.39339769	-0.09146
cg07915862	IL3	brown	-0.0387256	-0.3131193	0.11309159	0.20447565	0.5129321	0.19152809

cg10729062	PODNL1;PODNL1;PODNL	brown	-0.2609514	-0.5439052	0.21957676	0.20765258	0.80537528	0.07294003
cg23352191	DCAF7;DCAF7;DCAF7	brown	-0.262789	-0.6179082	0.29266697	0.13888288	0.73001485	0.10160643
cg08401618	RP11-96C21.1	brown	-0.128429	-0.5446287	0.06341041	0.21037351	0.78729508	0.16091376
cg17445812	TRANK1	brown	-0.2574374	-0.5506851	0.00033865	0.18597778	0.6240496	0.03607092
cg25311764	GCNT3;GCNT3;GCNT3;GC	brown	-0.2536302	-0.2880964	0.35827249	0.21018006	0.38330043	0.08391485
cg07820882	RMND5A;RMND5A	brown	0.44196554	0.69180522	0.0124969	-0.0319999	-0.6491129	0.01567659
cg17055821	C17orf75	brown	-0.0897434	-0.5505142	0.34337488	0.29466058	0.64501479	0.47175327
cg03754743	C22orf13;C22orf13;C22orf13	brown	-0.2011082	-0.5642866	0.19683601	0.25165391	0.80201813	0.14800182
cg13203811	TSPAN31;TSPAN31;TSPA	brown	-0.2654422	-0.2171502	0.33865322	0.19331531	0.41890085	0.09665439
cg01300277	INPP5B;INPP5B;INPP5B;IN	brown	-0.3011253	-0.423188	0.32834851	0.09208148	0.60031598	0.1632263
cg23461714	TTC12;TTC12;TTC12;TTC12	brown	-0.3469024	-0.3578346	0.2420455	0.17521708	0.37742208	-0.0161053
cg11367633	CHD6;CHD6;CHD6	brown	-0.1840847	-0.4200835	0.06778488	0.37262547	0.69933567	0.00462469
cg11441124	ZMIZ2;ZMIZ2;ZMIZ2;ZMIZ	brown	-0.1658493	-0.5739585	0.33460725	0.286688	0.83171949	0.23076473
cg23545098	CREBBP;CREBBP	brown	-0.1604756	-0.4214723	0.28531068	0.29323153	0.58141659	0.30873459
cg17617360	MYL12A;MYL12A	brown	0.4493448	0.73931421	0.05749909	-0.0102677	-0.7169778	0.07229889
cg13993274	AK3;AK3;AK3	brown	-0.2516095	-0.6836489	0.08373174	0.13861301	0.69604356	0.04785311
cg15806304	SLC18A2;RP11-501J20.5	brown	0.09116284	-0.2389502	0.33140306	0.35104169	0.56848402	0.30337971
cg08618604	PPAPDC3;PPAPDC3;PPAP	brown	-0.2204786	-0.143102	0.27665187	0.10708574	0.19097307	0.05212141
cg18039296	AC024575.1	brown	-0.0491294	-0.3541667	0.4165274	0.36753597	0.68545384	0.24920138
cg27201889	SYN3;SYN3	brown	0.03591672	-0.2848948	0.0912757	0.5079004	0.7398313	0.1400377
cg10203610	LRAT;LRAT;LRAT;LRAT;L	brown	-0.3597111	-0.5349328	0.21543678	0.14311562	0.52142201	0.05235599
cg19255886	NDRG1;NDRG1;NDRG1;N	brown	-0.0861247	-0.3734947	0.25226582	0.36667823	0.70401918	0.10911066
cg06754224	SESN2	brown	-0.3487908	-0.718082	0.19896417	0.21733758	0.83025847	0.03467809
cg11736228	ENTPD7	brown	-0.0453195	-0.4236596	0.40779703	0.52568023	0.72996937	0.15782422
cg20556503	AL844908.5;ITGB2;ITGB2;I	brown	0.3852943	0.65890569	-0.0456732	-0.0769432	-0.6950734	0.11045813
cg04369874	RP11-616K22.3;RP11-	brown	-0.4019536	-0.6986673	0.14813051	0.07020908	0.65181917	-0.0671291
cg16144222	PRKACA;PRKACA	brown	-0.3931893	-0.2164097	0.29891373	-0.024212	0.28719062	-0.007988
cg26106353	WDFY2	brown	0.33629937	0.68302271	0.02092939	-0.1034181	-0.7941453	0.04479561
cg18936052	FAM76A;FAM76A;FAM76	brown	-0.1349102	-0.3803376	0.2583322	0.27430393	0.48464479	0.22862086
cg04419315	PROX1;PROX1;PROX1;PR	brown	-0.3335004	-0.2852347	0.32111455	0.14307989	0.29887624	-0.0154747
cg09331334	VOPP1;VOPP1;VOPP1;VO	brown	-0.1680722	-0.585819	0.2465565	0.37131789	0.88565971	0.13604681
cg13780065	UBTF;UBTF;UBTF	brown	-0.3017541	-0.2885203	0.44591368	0.1981526	0.36754916	0.01497266
cg03016927	NCR2;NCR2;NCR2	brown	0.48583968	0.47273838	-0.0405708	0.01923339	-0.3871569	0.12498816
cg26236109	ENPP2;ENPP2;ENPP2	brown	0.50273745	0.61859828	0.17386901	-0.0217878	-0.6067493	0.18313799
cg00921574	INTS1;INTS1;INTS1	brown	-0.3436746	-0.4327605	0.12869082	0.106367	0.53840758	-0.040932

cg06359968	ZFPM1;ZFPM1;ZFPM1;ZFP	brown	-0.2950826	-0.6428634	0.25188842	0.26520966	0.69715164	0.042436
cg03496350	ZBTB48;ZBTB48	brown	-0.3299789	-0.3831056	0.21113392	0.27774368	0.61789183	-0.0387448
cg17395207	BCL9;BCL9;BCL9	brown	0.44146063	0.75676161	-0.1074122	-0.0456178	-0.7112557	0.00481674
cg10201668	DUOX1;DUOX1;DUOX1;D	brown	-0.5295422	-0.6554901	0.12892526	0.07495337	0.59051175	-0.0763123
cg25824754	TRBV23-1	brown	-0.2283793	-0.6305505	0.14209202	0.19256868	0.85241611	0.01671412
cg14315198	UNC119;UNC119;UNC119;	brown	-0.2321768	-0.5790824	0.31484287	0.15881291	0.61111512	0.11565435
cg12133952	SLC6A9;SLC6A9;SLC6A9;S	brown	-0.341731	-0.6587244	0.23615084	0.21301513	0.80025234	0.0114479
cg21028604	DIPAH3-AS2	brown	0.52497836	0.60848115	0.08852672	-0.0193123	-0.663475	0.19863179
cg17239103	CCDC19;CCDC19;CCDC19	brown	-0.081967	-0.3318094	0.47180323	0.27263426	0.3931633	0.3692362
cg20976581	GAS2L2	brown	-0.1852232	-0.2352984	0.31700525	0.20943507	0.40763149	0.20157635
cg21757368	C6orf170;C6orf170;C6orf170	brown	-0.1808816	-0.4046222	0.19254649	0.22830729	0.50558533	0.15957513
cg03138957	PLVAP	brown	-0.0758967	-0.2420199	0.38680761	0.36986837	0.56630919	0.17385926
cg18016128	FAM211A;FAM211A	brown	-0.1046073	-0.6073202	0.23398234	0.29872654	0.8766223	0.16928059
cg01234195	FAM65B	brown	0.32983822	0.63306479	-0.1333228	-0.0248104	-0.6339592	-0.0132233
cg07732172	TBCD;TBCD;TBCD;TBCD;	brown	-0.2715544	-0.3155144	0.18260269	0.21105627	0.28225976	0.20950629
cg15959238	CALD1;CALD1;CALD1;CA	brown	-0.3473268	-0.621239	0.122715	0.08660851	0.59864617	0.0641992
cg19767538	C8orf38;C8orf38;C8orf38;C8	brown	-0.180747	-0.5686934	0.2319959	0.30686259	0.83397393	0.06411382
cg20755748	METTL20	brown	-0.0262229	-0.4653466	0.01715493	0.29170856	0.74565669	0.15755384
cg01031101	NKAPL;ZKSCAN4	brown	-0.1735579	-0.4367288	0.12645313	0.08797308	0.42460918	0.1150968
cg04267691	GAS7;GAS7;GAS7;GAS7	brown	-0.3221164	-0.5795318	0.26238635	0.12687468	0.69102949	0.03686727
cg02773624	SMTNL1;SMTNL1	brown	0.01234001	-0.3611488	0.1406221	0.3112303	0.64989988	0.20941396
cg17166434	ARNT;ARNT;ARNT;ARNT;	brown	-0.001339	-0.2300415	0.07697454	0.33706865	0.55415953	0.14344894
cg17536532	PPP1R26	brown	-0.4393097	-0.6444257	0.12026769	0.17328501	0.64146677	-0.0875615
cg17034109	CYB561D1;CYB561D1;CY	brown	-0.3713461	-0.3005614	0.17980011	0.17032563	0.42733602	-0.0439117
cg23126152	SSH1;SSH1;SSH1;SSH1	brown	-0.0226674	-0.4492898	0.20934592	0.38903258	0.79207843	0.23228125
cg22870483	ZFAT;ZFAT;ZFAT	brown	0.43882855	0.63917122	-0.2590559	-0.0670102	-0.7136698	0.01992315
cg07755196	RP11-107I14.1;RP11-	brown	-0.5910028	-0.5001332	0.19814823	-0.013368	0.39659813	-0.1728924
cg21279955	SLC27A3;SLC27A3;SLC27	brown	-0.3462023	-0.5519148	0.04042331	0.14191854	0.73151389	-0.0362802
cg01012339	GNAI2;GNAI2;GNAI2	brown	-0.3550424	-0.6762982	0.18403638	0.1902115	0.76335212	0.01989381
cg25933511	VPS39;VPS39	brown	0.46034386	0.62859956	-0.0127186	-0.05307	-0.7490389	0.09294148
cg06763375	CBFA2T3;CBFA2T3	brown	-0.1638351	-0.588048	0.33875818	0.30715789	0.82332617	0.19600524
cg18655410	GSN;GSN;GSN;GSN;GSN;	brown	0.05394448	-0.3019557	0.01831395	0.67902568	0.64319301	0.21715386
cg05726450	PIK3R6	brown	0.07100653	-0.2862922	0.10597214	0.56222746	0.74348843	0.10997643
cg13252968	KCNIP2;KCNIP2;KCNIP2;	brown	-0.2034997	-0.390595	0.15067622	0.33050944	0.52765628	0.04732822
cg09625962	KDM2A;KDM2A;KDM2A;	brown	-0.4156158	-0.7002181	0.13448936	0.14144062	0.77577311	0.00585008

cg14393462	LRFN4;LRFN4;LRFN4;LRF	brown	-0.5252179	-0.5598094	0.07914734	0.0454802	0.48080949	-0.2378836
cg04644869	ROBO4;ROBO4;ROBO4;RO	brown	-0.3791767	-0.3731107	0.19463019	0.13257208	0.40909157	-0.0451396
cg09145932	KANK2	brown	-0.0031412	-0.3443392	0.19317818	0.33069853	0.67030432	0.2745271
cg16451402	ITGB4;ITGB4	brown	-0.3233938	-0.5699281	0.20178011	0.0837026	0.75147799	0.02870625
cg22749623	MYEOV;MYEOV;MYEOV;	brown	-0.0364792	-0.2934604	0.25564531	0.32286417	0.63683749	0.15353135
cg17372758	ASCC1;ANAPC16;ASCC1;	brown	0.2837091	0.65277047	-0.1851974	-0.0846527	-0.694771	-0.0258566
cg25300094	LOXL2;LOXL2;LOXL2;ENT	brown	0.43767749	0.72333998	-0.112136	-0.0447012	-0.6539532	0.0800454
cg10114725	FAM124B;FAM124B;FAM1	brown	-0.3189045	-0.6504331	-0.0319222	0.09432262	0.7326532	-0.0724135
cg21820357	CTD-2012K14.1;CTD-	brown	-0.2564831	-0.6271969	0.32509105	0.26908175	0.76303144	0.06610696
cg12417466	ARPP21;ARPP21;ARPP21;	brown	0.31709502	0.67698556	0.00678533	-0.1510398	-0.8118436	-0.0201415
cg26066917	TAGLN;TAGLN;TAGLN;T	brown	-0.0816505	-0.3886437	0.37747111	0.15945746	0.43834252	0.24400471
cg17932056	CD9;CD9;CD9;CD9;CD9;C	brown	-0.0957565	-0.4106199	0.22280713	0.28409697	0.76456857	0.18951534
cg06537333	KIAA0182	brown	-0.1487114	-0.5947913	0.24669101	0.29540134	0.88871616	0.22249794
cg06378491	MAP4K2;MAP4K2;MAP4K	brown	-0.3268597	-0.7104968	0.2746343	0.14338073	0.76672688	0.03987918
cg26624796	SEPT1;SEPT1;ZNF48;ZNF4	brown	-0.4785929	-0.7669405	0.08709705	0.13236943	0.73075593	-0.0414661
cg02092616	INF2;INF2;INF2	brown	-0.1881644	-0.4851792	0.21090911	0.19299711	0.6984741	0.17743262
cg12979844	SIAH3;SIAH3	brown	-0.1359218	-0.4003796	0.06499179	0.27700577	0.57962432	0.08171758
cg08580836	ZNF300;ZNF300;ZNF300;Z	brown	-0.3016062	-0.5950779	0.15066833	0.02886298	0.52983681	0.09320845
cg02404400	TRAJ30	brown	0.39249008	0.76878993	0.07586262	-0.1146085	-0.860112	0.05972878
cg02780269	SECTM1	brown	-0.0493794	-0.3233759	0.27476679	0.2403427	0.4687742	0.14352101
cg06531671	C17orf64;C17orf64;C17orf64	brown	-0.0938513	-0.2559862	0.17667852	0.25960248	0.53348805	0.10023612
cg07730193	R3HCC1;R3HCC1;R3HCC1;	brown	-0.2885829	-0.4663884	-0.0707061	0.0291162	0.5942434	-0.018464
cg08518475	RCAN3;RCAN3	brown	0.43136945	0.61366017	-0.1085262	-0.1062618	-0.7435165	0.16405003
cg22977317	SERPINB1;SERPINB1;SER	brown	-0.168251	-0.4716656	0.19664468	0.33525553	0.77298092	0.11720538
cg22341471	ABCB9;ABCB9;ABCB9;AB	brown	-0.1503519	-0.303906	0.25265032	0.30022518	0.58795679	0.1256579
cg04695090	MAFF;MAFF;MAFF;MAFF	brown	0.01614293	-0.4791598	0.16759878	0.46067596	0.840192	0.261425
cg21031875	DNMBP;DNMBP;DNMBP-	brown	0.35614082	0.60272025	-0.0923575	-0.0972344	-0.7885946	0.00496124
cg13975750	TRAJ40	brown	0.29571156	0.66305584	-0.2102965	-0.0939519	-0.6662749	-0.1107385
cg01520681	CNOT10;CNOT10;CNOT10;	brown	0.00046041	-0.3322559	0.04985107	0.44006898	0.63153675	0.2697289
cg16136400	CEP85L;CEP85L	brown	0.46487223	0.673124	0.10090607	-0.0073403	-0.7270733	0.15099247
cg09769749	RP4-694B14.5;RP4-	brown	-0.4257376	-0.591583	0.14867057	0.11392514	0.57015733	0.03410982
cg06446719	CGREF1;CGREF1;CGREF1;	brown	-0.199071	-0.4259067	0.50688946	0.32128531	0.42546093	0.24238849
cg10345494	C1orf162;C1orf162	brown	-0.1009086	-0.398093	0.30918244	0.40390717	0.73100832	0.08799142
cg17210263	CHIT1;CHIT1	brown	-0.3386735	-0.6492229	0.28367095	0.19939583	0.60243183	0.00744925
cg23960868	HM13-	brown	-0.3176833	-0.6176858	0.11950029	0.18566384	0.72285403	-0.0442704

cg02710230	RP11-158M2.4	brown	0.06140819	-0.4164961	0.14762749	0.34069568	0.73918333	0.30774428
cg02959609	RIIAD1;RIIAD1;RIIAD1;AL	brown	-0.3579889	-0.4716713	0.1270875	0.06960272	0.45368588	-0.0359087
cg24815728	SH3TC2;SH3TC2;SH3TC2	brown	0.28718093	0.52530327	-0.0853361	0.01117184	-0.4577953	-0.0242151
cg14073716	MIR3654;EEF1G;EEF1G;EE	brown	-0.0217712	-0.3603174	0.34060122	0.57061536	0.64956294	0.17663757
cg15487687	MLST8;MLST8;MLST8;ML	brown	-0.103794	-0.2733801	0.56529851	0.49138024	0.50334798	0.27304819
cg07405121	SLC9A3R2;SLC9A3R2;SLC	brown	-0.0369695	-0.4973323	0.19137896	0.23344454	0.72443129	0.23708476
cg07013258	BCL7C;BCL7C;BCL7C;BCL	brown	-0.3163165	-0.7177391	0.19306724	0.13512572	0.68980139	0.07082495
cg14910195	ST6GALNAC6;ST6GALNA	brown	-0.0203881	-0.2053297	0.56789705	0.418178	0.50004565	0.16840147
cg19479086	RNF145	brown	0.30631456	0.68851301	-0.1633777	0.05950465	-0.7205061	-0.0351092
cg04975272	RP11-	brown	0.03775432	-0.3484291	0.25242235	0.49102583	0.69980582	0.33427492
cg24770845	VKORC1;RP11-	brown	-0.3854394	-0.5129347	0.35529979	0.20856649	0.59037037	0.02900864
cg01420388	FBXO44;FBXO2;FBXO2;F	brown	-0.2220284	-0.4762851	0.27884566	0.18625625	0.50763474	0.09536779
cg19716816	GMCL1;GMCL1	brown	-0.3738218	-0.5817675	0.06075512	0.18769516	0.61367712	-0.1377421
cg21193966	CTD-2503O16.4	brown	0.12944576	0.64350909	0.030806	-0.1775776	-0.8346136	-0.0806298
cg01189603	TM6SF1;RP11-	brown	0.43424337	0.77249752	0.04933998	-0.1114978	-0.7312388	0.10165129
cg11834473	GABBR1;GABBR1;GABBR	brown	-0.049614	-0.4002092	0.09491462	0.4374839	0.76330499	0.13308381
cg08836984	SERTAD4;SERTAD4;SERT	brown	-0.508311	-0.5461325	0.11802563	0.15043561	0.55899762	-0.1398951
cg26478216	TUBA8;TUBA8;TUBA8	brown	-0.0081668	-0.2358511	0.24291479	0.2936679	0.5289576	0.21651272
cg02784809	ZNRF3-AS1;ZNRF3	brown	-0.3402662	-0.3026782	0.16444747	0.06293686	0.40196025	-0.0241894
cg22361181	NKIRAS2;NKIRAS2;NKIR	brown	-0.4245399	-0.3297736	0.19083948	0.1206688	0.44705221	-0.0650526
cg26129664	RP11-432J24.2	brown	-0.1341442	-0.5536717	0.1187643	0.32888866	0.83558957	0.06680419
cg24103563	TRIM34;TRIM34;HBG2;AC	brown	0.43927258	0.67065447	-0.1278597	0.04519629	-0.6368341	0.00701158
cg18981338	DHRS4;DHRS4;DHRS4;DH	brown	-0.1752388	-0.5176474	0.33519717	0.36604266	0.65803032	0.31040305
cg18524319	CDK13	brown	0.47560971	0.62172423	-0.0899038	-0.0300822	-0.7174938	0.08420533
cg07056606	SIGLEC6;SIGLEC6;SIGLEC	brown	-0.3513858	-0.6117173	0.18667901	0.26222358	0.72447263	0.23630095
cg02481256	AC024575.1	brown	-0.4185997	-0.6413558	0.10679941	0.10423385	0.67477658	-0.0489761
cg05465666	C1orf54;C1orf54;C1orf54	brown	-0.2956164	-0.5070354	0.04718906	0.07043093	0.55912478	-0.0632903
cg26576597	AC104655.2	brown	-0.3574434	-0.2888495	0.37225758	0.19462727	0.29186444	0.07148426
cg05168764	TTC7B;TTC7B	brown	-0.2608462	-0.3326531	0.22816527	0.22731778	0.34630358	0.13886674
cg18462898	MEF2B;MEF2B;MEF2B;ME	brown	-0.0177021	-0.5276729	0.14467328	0.3381446	0.86981656	0.17716427
cg18814142	TRAJ43	brown	0.38764364	0.7320638	-0.0847201	-0.1554882	-0.8145653	0.04158788
cg23551917	RNU5B-	brown	-0.0648586	-0.3003055	0.25995071	0.19488836	0.40135165	0.19243332
cg27560367	DCUN1D2;DCUN1D2;TMC	brown	-0.2615086	-0.4608368	0.30310343	0.29247445	0.64433611	0.05996215
cg26132647	U73166.2;U73166.2;U73166.	brown	-0.1693947	-0.6388444	0.25878623	0.25994448	0.87717033	0.17716819
cg20254120	RP11-157N3.1;RP11-	brown	0.37820368	0.64471379	-0.029122	-0.1015613	-0.7907698	0.01274204

cg18041140	ACTRT2;ACTRT2;ACTRT2	brown	-0.2796081	-0.6455317	0.13112945	0.12812071	0.84511403	0.03816929
cg04032578	ZNF300;ZNF300;ZNF300	brown	-0.2970128	-0.4943303	0.29612396	-0.0169886	0.36113362	0.17403221
cg13853953	THAP4;THAP4	brown	0.44063837	0.61430331	-0.0432493	0.00466326	-0.6073492	0.13155456
cg22930187	ARTN;ARTN;ARTN;ARTN;	brown	-0.3776155	-0.7023414	0.16687723	0.21433542	0.72775274	0.00619587
cg18358621	SCG5;SCG5;SCG5	brown	-0.3504836	-0.5044755	0.37988532	0.21658587	0.43529795	0.09744265
cg08496737	NXF1;NXF1;NXF1;NXF1;N	brown	-0.2436052	-0.2627812	0.30214103	0.40488665	0.34126678	0.11399935
cg23631370	LAMA5	brown	-0.4048279	-0.0780161	0.26073897	0.05857115	-0.1894054	-0.097175
cg27294796	C15orf38;C15orf38;C15orf38-	brown	-0.3360818	-0.6687251	0.11545467	0.21160953	0.80351108	0.05269647
cg14076195	STMN1;STMN1;STMN1;ST	brown	-0.5593864	-0.570712	0.06282461	-0.0045694	0.44293343	-0.1138341
cg21855244	CES4A;CES4A;CES4A;CES	brown	-0.1659108	-0.5261091	0.40448647	0.40547563	0.75461734	0.13452495
cg07687673	HAVCR2;HAVCR2;CTB-	brown	0.23111617	0.56294305	-0.1118919	-0.1495569	-0.7141197	-0.0226338
cg10247328	KCNT2;KCNT2;KCNT2	brown	-0.1998802	-0.4031152	0.09989007	0.17950319	0.46093594	0.08147605
cg00433188	XPO5;XPO5;XPO5;POLH;P	brown	0.27655694	0.69725687	-0.2050154	-0.2035393	-0.8525022	-0.0404456
cg27353919	IP6K2;IP6K2;IP6K2;IP6K2;I	brown	-0.395638	-0.7055613	0.00037127	0.08819463	0.68254759	-0.0395571
cg13522588	XPO6;XPO6;XPO6;XPO6	brown	0.49305136	0.59137904	-0.0935067	-0.0705527	-0.5682932	0.08011264
cg04852826	CAMKK1;CAMKK1;CAM	brown	0.00091863	-0.2902834	0.67002337	0.44303686	0.49573345	0.36969339
cg19525758	ARHGEF3;ARHGEF3;ARH	brown	-0.0528198	-0.5692625	0.22976822	0.36379117	0.90639783	0.24612213
cg04284196	TRAJ35;TRAJ34	brown	0.40975069	0.75163325	0.05535046	-0.0777279	-0.8259953	0.11093297
cg22828022	DPEP3;DPEP3;DPEP3	brown	-0.1952995	-0.3508499	0.00211962	0.1534824	0.40164345	0.13229647
cg26114100	ENTPD7	brown	-0.3035547	-0.5497824	0.40082598	0.215893	0.58636796	-0.0083519
cg14798877	THBS4;THBS4;CTD-	brown	0.43443104	0.71536116	-0.0892374	-0.0843707	-0.6764407	0.07734665
cg14380517	BTG3;BTG3;BTG3;BTG3	brown	-0.3773294	-0.7718533	0.11089049	0.10944255	0.77636219	0.03562523
cg26323242	USP19	brown	0.11702584	-0.2683724	0.43391628	0.71469466	0.57830686	0.43940803
cg13806740	LRRC48;LRRC48;LRRC48;L	brown	-0.3611002	-0.6277133	0.16690494	0.24085853	0.72283799	-0.0079071
cg22680827	RP11-27P7.1	brown	0.32446436	0.72241576	-0.1752473	-0.1765767	-0.7936171	-0.0534427
cg24057710	KIRREL3;KIRREL3;KIRRE	brown	-0.0969034	-0.3256616	0.10685247	0.34698767	0.7117433	0.14371405
cg24878059	CNTD2;CNTD2	brown	-0.2126556	-0.4357479	0.25139876	0.28458301	0.5248258	0.15114725
cg11044476	SLC29A1;SLC29A1;SLC29	brown	-0.0348067	-0.5110484	0.30142784	0.55190568	0.75281488	0.2651868
cg07619953	TNFRSF11A;TNFRSF11A	brown	-0.1937246	-0.6337401	0.12159123	0.3168472	0.81927341	0.02754352
cg06710212	CERCAM;CERCAM;CERC	brown	-0.323735	-0.3772064	0.2472044	0.01565408	0.31444543	0.04201673
cg10973967	RP11-387D10.3;RP11-	brown	0.17101797	-0.2457765	0.16429127	0.67067319	0.56567482	0.36340358
cg06721393	TRAV36DV7	brown	-0.2905467	-0.4673241	0.06457598	0.20194253	0.59114616	-0.0724843
cg25151359	HCK;HCK;HCK;HCK;HCK	brown	-0.294809	-0.6707348	0.20414265	0.08302317	0.79478952	0.02010773
cg06796611	IL24;IL24;IL24;IL24;IL24;IL	brown	0.30782141	0.79458826	-0.0305224	-0.1258952	-0.874556	-0.0468389
cg10650353	EIF6;RP4-614O4.11	brown	0.04182536	-0.436358	0.20545498	0.54784679	0.74281712	0.27429307

cg17300723	SLC3A2;SLC3A2;SLC3A2;S	brown	-0.2563634	-0.6310127	0.10745693	0.26640653	0.85048748	0.05614878
cg23632849	CTD-2514K5.2;CTD-	brown	-0.0875797	-0.3616269	0.43484698	0.26716985	0.57398142	0.39468689
cg24265217	RP11-363J20.1;RP11-	brown	-0.1582907	-0.3815594	0.31663491	0.27636848	0.43441372	0.21714554
cg08005849	HGF;HGF;HGF;HGF;HGF;	brown	-0.207084	-0.5987032	0.20908517	0.15119195	0.77826924	0.15053427
cg13767707	SLC9A3R2;SLC9A3R2;SLC	brown	0.04939349	-0.3219751	0.24022743	0.27713747	0.58492718	0.27794262
cg18716390	DENND3;DENND3	brown	-0.2709948	-0.6168118	0.15003901	0.02054438	0.62947359	0.1089427
cg26731131	RP11-744N12.3;FLI1;RP11-	brown	-0.1544951	-0.5938536	0.08867102	0.29047848	0.9042657	0.14825753
cg03051392	HYAL2;HYAL2;HYAL2;H	brown	-0.2196076	-0.63231	0.26800267	0.27219678	0.86915569	0.08085087
cg14389518	RP11-195M16.1	brown	-0.158014	-0.5119784	0.23948674	0.38496818	0.8353757	0.14248465
cg00721951	SEMA3F;SEMA3F;SEMA3	brown	-0.1654456	-0.161428	0.3291326	0.31166302	0.23455015	0.23169852
cg09137630	ZC3H4	brown	-0.1434967	-0.5780881	0.06160624	0.20811115	0.79937226	0.12867591
cg14329350	NXF1;NXF1;NXF1;NXF1;N	brown	-0.292911	-0.3188343	0.18216161	0.27449735	0.3481938	0.00343293
cg02737621	SESN2	brown	-0.3477476	-0.6174032	0.1144408	0.18955992	0.76542009	0.01585832
cg06330621	RP11-	brown	-0.0897	-0.2458719	0.20385469	0.20896248	0.34027521	-0.0839167
cg17579753	GAS2L2	brown	-0.2154118	-0.4403741	0.00623475	0.1720191	0.55479438	0.06227123
cg09916783	POMC;POMC;POMC;POM	brown	-0.1713269	-0.43293	0.1174442	0.20289551	0.69273145	0.03671008
cg23362108	SMTNL1;SMTNL1	brown	-0.0908077	-0.4895958	0.08559668	0.26045145	0.8197746	0.09062176
cg04651042	SFRP4;SFRP4;EPDR1	brown	-0.4130808	-0.5171414	0.17763163	0.02475721	0.55162349	-0.0286041
cg09358400	ISG20;ISG20	brown	0.3833149	0.687895	0.08771698	-0.0904849	-0.8062256	0.09064812
cg17815177	PTGER1	brown	-0.510179	-0.5760898	0.13972503	0.06030572	0.65261429	-0.1220788
cg17078317	NYAP1;NYAP1	brown	0.06833524	-0.2517681	0.26765696	0.41725628	0.63249117	0.38674805
cg11496559	TMEM108;TMEM108;TME	brown	-0.1992401	-0.5954835	0.17641712	0.16731347	0.74157916	0.1402467
cg18107181	RP11-61O1.1	brown	0.33299312	0.4704297	-0.1067429	-0.0921686	-0.5558395	-0.0139821
cg23385718	HDAC9;HDAC9	brown	-0.3703764	-0.4168257	0.22150704	0.04885834	0.43781773	-0.157914
cg15972301	TTYH1;TTYH1;TTYH1;TTY	brown	-0.3387288	-0.5029535	0.33952428	0.20274703	0.4484413	0.14844046
cg24529722	SLC44A4;SLC44A4;SLC44	brown	-0.0820806	-0.5580166	0.21017301	0.35137669	0.85883418	0.13356453
cg12791849	RP1-300I2.2	brown	0.01971418	-0.2539178	0.10084693	0.41125177	0.56648329	0.21205568
cg17318863	FER1L5;FER1L5;FER1L5;K	brown	-0.5072188	-0.586261	0.16986144	0.04350917	0.55587429	-0.0832042
cg11888977	AC018890.4	brown	0.34301597	0.75998701	-0.2305727	-0.1158071	-0.7269421	0.01642146
cg25748108	LPIN3;LPIN3	brown	-0.281179	-0.4131999	0.22593169	0.26018247	0.40678366	-0.0017008
cg06467473	NEK6;NEK6;NEK6;NEK6;N	brown	-0.401656	-0.686834	0.0715474	0.07443928	0.75855974	0.00227357
cg15903529	C7orf50;C7orf50;AC073957.	brown	-0.3971349	-0.5831114	0.1786797	0.15767821	0.62909439	-0.0168182
cg02727678	RP11-804A23.1	brown	-0.1420852	-0.5296281	0.25661259	0.39747709	0.84009868	0.09210984
cg00570594	RP11-432J24.2	brown	-0.139641	-0.6033084	0.14391881	0.30805263	0.88756672	0.09595743
cg14692944	RP1-153P14.5;RP1-153P14.3	brown	-0.3988449	-0.6555144	0.10549832	0.08011827	0.67559487	-0.1028694

cg23964386	PTGR1;PTGR1;PTGR1;PTG	brown	-0.2053034	-0.4007837	0.09880106	0.00268773	0.39327735	0.09520762
cg07708721	PRRC2A;SNORA38	brown	0.08529709	-0.4312457	0.21320463	0.54346721	0.79969059	0.32342642
cg01874152	TESPA1;TESPA1;TESPA1;T	brown	0.22638779	0.60274145	0.14861915	-0.1723582	-0.8438205	0.03048839
cg06849059	RP5-1187M17.10	brown	-0.1868033	-0.6169987	0.23142025	0.1707657	0.69137624	0.09746298
cg08980570	LINC00211;LINC00211	brown	-0.0153373	-0.3065446	-0.0465659	0.14530078	0.63848878	0.11684245
cg20534570	TEF;TEF	brown	-0.3633544	-0.6284428	0.16355952	0.12731256	0.60492398	0.0288275
cg12779520	CD6;CD6;CD6;CD6;CD6	brown	-0.454323	-0.5429614	0.3343819	0.13723773	0.45573752	-0.0006558
cg03086022	PPM1F;PPM1F;LL22NC03-	brown	-0.1796294	-0.6491386	0.12221295	0.20382937	0.9082558	0.11689484
cg02459569	RP11-	brown	0.00913965	-0.5199962	0.19276943	0.46589671	0.92566849	0.21276457
cg16576811	NEDD4L;NEDD4L;NEDD4	brown	0.03656656	-0.2875597	0.08875112	0.62593893	0.73706797	0.15679899
cg26357885	HSPA2;HSPA2;HSPA2;RP1	brown	-0.3796538	-0.5104782	0.1354029	0.1327193	0.5234379	-0.0274818
cg10108874	C19orf33	brown	0.01330383	-0.4131502	0.010953	0.32074588	0.79676288	0.15330704
cg26564895	RP11-712B9.2;RP11-	brown	-0.0257563	-0.386795	-0.0162585	0.35671657	0.75165687	0.05263142
cg13408605	SIRPG;SIRPG;SIRPG	brown	0.2360049	0.73360377	0.06258066	-0.1846422	-0.8476698	-0.0653445
cg21497935	GIPC2;GIPC2	brown	-0.4627062	-0.6071164	0.09325801	-0.0280358	0.39963492	-0.1187716
cg18515250	MIR330;EML2	brown	-0.3683938	-0.3194903	0.2466028	0.21858091	0.28739422	-0.1109426
cg02572739	TBC1D19;TBC1D19;TBC1D	brown	0.33844104	0.75089802	-0.0555742	-0.1324649	-0.8851682	0.00600858
cg23933044	C5orf42	brown	-0.2130891	-0.6207852	0.15751919	0.23189899	0.82289974	0.09946786
cg06372718	TRABD;TRABD;TRABD	brown	-0.1405552	-0.5751645	0.28805586	0.33472863	0.83167402	0.22663473
cg16576285	NINJ2;NINJ2;NINJ2;NINJ2	brown	-0.5039652	-0.5627901	0.17994175	-0.0055866	0.53827989	-0.1673709
cg05973881	GPR56;GPR56;GPR56;GPR	brown	-0.1869725	-0.5519875	0.28898723	0.31845808	0.81709807	0.17844093
cg10181798	SYNE2;SYNE2;SYNE2;SY	brown	0.43687418	0.57214366	0.20682432	-0.0089201	-0.7024876	0.14469432
cg00793181	KRT72;KRT72;KRT72;KRT	brown	0.19914181	-0.1630821	0.25207186	0.65835748	0.59666853	0.33694966
cg19285358	EDC3;CLK3;EDC3	brown	-0.1273589	-0.6222671	0.18351489	0.29941772	0.89143836	0.1257762
cg22336648	SATB1;SATB1;SATB1;SAT	brown	0.35917163	0.47330475	-0.0107556	-0.1308968	-0.7075663	0.0841132
cg25062513	RP11-142L1.1	brown	0.35252811	0.66731226	0.05361248	-0.0981774	-0.7411855	0.01008434
cg24615878	TRAJ3	brown	0.39739037	0.71893009	-0.0005913	-0.0554357	-0.8147946	0.06514133
cg23083936	BCKDHA;BCKDHA	brown	-0.0876085	-0.5729392	0.24207599	0.37688443	0.9038385	0.2123414
cg19575804	LINC00261	brown	-0.3556127	-0.5149791	0.31255589	0.20483743	0.45699768	-0.0270172
cg14093936	SEMA7A;SEMA7A;SEMA7	brown	-0.2389519	-0.4615742	0.26886077	0.24030619	0.68848393	0.07836663
cg23260901	ZNF438;ZNF438;ZNF438;Z	brown	0.00663496	-0.4271461	0.17405564	0.46063466	0.73346065	0.22151996
cg13729146	PTMS;PTMS;PTMS;PTMS;	brown	-0.2164464	-0.6221871	0.17703561	0.18308266	0.76907921	0.05444836
cg07777609	IDH3A;IDH3A;IDH3A;IDH	brown	0.51395537	0.53483087	0.00305597	0.04885218	-0.5130944	0.26066565
cg06082750	MGAT3;MGAT3	brown	-0.2884305	-0.6807311	0.16438187	0.24504635	0.90370511	0.00741926
cg05876737	PDE4D;PDE4D;PDE4D;PDE	brown	-0.0221404	-0.5582629	0.06326094	0.36753208	0.90288083	0.1642126

cg24959264	PLD4;PLD4;PLD4;PLD4	brown	-0.1163191	-0.5443136	0.22314458	0.39920677	0.8172132	0.18188197
cg00878766	RP5-1119A7.10	brown	-0.2492114	-0.622975	0.10778723	0.18314496	0.86944159	0.03933384
cg17036007	MEF2D;MEF2D;MEF2D;M	brown	-0.3071857	-0.6196172	0.20825302	0.18570749	0.83833161	0.01293674
cg07253829	DPPA4;DPPA4;DPPA4;DPP	brown	-0.2257172	-0.6412032	0.16879191	0.21068555	0.76331969	-0.0158382
cg19310895	CD5L	brown	-0.2947705	-0.5009772	-0.0079702	0.19575112	0.6645451	-0.0513932
cg04539476	REM2;REM2	brown	-0.2859752	-0.7014048	0.01111708	0.2114428	0.84400832	0.00699634
cg02343823	ZNF300;ZNF300;ZNF300;Z	brown	-0.1867723	-0.6880104	0.21057193	0.13388415	0.69098273	0.13220225
cg05108737	RP11-218D6.4;ARMC4	brown	-0.3347668	-0.3436374	0.02468587	0.2402026	0.39081759	-0.1613664
cg16719404	CD2;CD2	brown	0.09706167	0.51399793	-0.2037664	-0.2705281	-0.6491117	-0.1368203
cg04599149	TMC4;TMC4;TMC4	brown	-0.3660995	-0.6090981	0.13555512	0.15663908	0.61917277	0.03474925
cg07888884	KSR1;KSR1	brown	-0.1262649	-0.5957389	0.29458391	0.25224389	0.84231293	0.12805561
cg13261938	MRM1;DHRS11	brown	-0.0020271	-0.1095361	0.44032385	0.45267975	0.38280747	0.2774024
cg01811187	RP11-	brown	-0.3624261	-0.5990083	0.05779463	0.12722344	0.66114867	0.00329009
cg15219685	KIAA1919;RP11-428F8.2	brown	-0.3231205	-0.2771323	0.1959008	0.30632588	0.42272088	-0.0731899
cg09166900	PLVAP	brown	-0.0784213	-0.4613061	0.16002887	0.38657373	0.87125606	0.1412841
cg24811265	LEF1;LEF1;LEF1;LEF1;LEF	brown	0.30562018	0.66089088	0.09294349	-0.1587269	-0.8431638	0.07858673
cg13423770	RHBG;RHBG;RHBG;CCT3;	brown	-0.1287293	-0.6346133	0.30187651	0.31586819	0.82725886	0.22376355
cg07368507	REEP2;REEP2;REEP2;REEP	brown	-0.2649096	-0.6076128	-0.0992925	0.1331708	0.60548106	0.03068132
cg00478846	SF3B2;SF3B2;SF3B2;SF3B2	brown	-0.0879074	-0.5290522	0.15682053	0.30235127	0.76585848	0.20938083
cg13944870	CSF3R;CSF3R;CSF3R;CSF3	brown	-0.1136963	-0.4281175	0.12303472	0.16463071	0.67537274	0.11978796
cg21251475	LINC00173;LINC00173;LIN	brown	-0.2085465	-0.5075358	0.20238898	0.0860906	0.54966514	0.1469411
cg17086626	YPEL4;YPEL4;AP000662.4;	brown	-0.3935734	-0.7139178	0.13019144	0.11552356	0.79622269	-0.0437929
cg13202678	CTC-786C10.1	brown	-0.0134312	-0.2937618	0.27325006	0.36953156	0.58972322	0.2033084
cg06264633	SLC39A13;SLC39A13	brown	-0.3228044	-0.7096904	0.07589768	0.23028437	0.87535343	0.0079711
cg06426831	RP5-	brown	-0.441715	-0.6839218	-0.0024559	0.05015941	0.73343795	-0.1219296
cg06112415	CGREF1;CGREF1;CGREF1;	brown	-0.2845396	-0.4388653	0.33453786	0.28242666	0.40089519	0.22124632
cg17110400	WNK4	brown	0.0089313	-0.3778152	0.25506401	0.37246874	0.59413477	0.34119907
cg03004985	ALS2CL;ALS2CL;ALS2CL;	brown	-0.1706477	-0.4615932	0.35355029	0.16153141	0.54524041	0.13347251
cg01521436	IL1A	brown	-0.275837	-0.6673305	0.10610227	0.18789988	0.81730539	-0.0212098
cg07391088	FUT10;FUT10;FUT10;FUT1	brown	-0.4948114	-0.618356	0.04005102	-0.0846923	0.57433663	-0.103725
cg02327719	RP1-125I3.2	brown	-0.1005649	-0.5214603	0.21863995	0.31427751	0.8587473	0.15542027
cg10446745	ADAM30;ADAM30	brown	-0.3241507	-0.3279671	0.04519764	0.08325764	0.31368531	-0.094755
cg11835197	RP4-728D4.2;RP4-	brown	0.10496012	-0.2270025	0.33506124	0.15298232	0.38575924	0.25625859
cg19765834	FGD2;FGD2;FGD2;FGD2;F	brown	0.54479391	0.58037878	-0.0025468	-0.025836	-0.5152032	0.15076254
cg24902842	UBA7;UBA7;UBA7;UBA7	brown	-0.1999078	-0.4891279	0.36378367	0.34163386	0.66780349	0.24096519

cg02359160	RP4-	brown	-0.1865037	-0.5594471	0.25646116	0.25660516	0.7242464	0.20246779
cg06417752	RP11-	brown	-0.1880543	-0.372815	0.24059774	0.33337215	0.59200068	0.11688368
cg16229875	CEBPE	brown	-0.0937968	-0.4696471	0.18170987	0.34130583	0.60313695	0.17602645
cg25308531	NCOA4;NCOA4;NCOA4;N	brown	-0.0508213	-0.5884017	0.08104633	0.33001418	0.93138443	0.1714091
cg13909826	RP11-475E11.5;RP11-	brown	0.41013617	0.73871713	0.0259664	-0.0653309	-0.8298815	0.07793836
cg17294065	RP11-18F14.4	brown	-0.2180693	-0.640533	0.15118219	0.10300183	0.80482228	0.1101833
cg12113819	MED13L;MED13L	brown	-0.1796756	-0.6407324	0.08995814	0.22939595	0.85637629	0.07819782
cg22585988	PVRL4;PVRL4;PVRL4	brown	0.09496912	-0.2645392	0.33282919	0.35500965	0.52793232	0.28172672
cg02883958	LRRC25	brown	-0.151856	-0.4639359	0.18369618	0.23059049	0.66248111	0.10992986
cg17152981	GPR6;GPR6;GPR6	brown	-0.2516837	-0.6635666	0.2006868	0.15483467	0.80542104	0.0772915
cg00265022	LPAR5;ACRBP	brown	0.06199054	-0.3468864	0.39211417	0.49333482	0.78519236	0.38616859
cg16383991	FRMD4A;FRMD4A	brown	-0.2898654	-0.5329556	0.21402787	0.12476755	0.69955281	-0.0391372
cg09213929	C11orf92;C11orf92;C11orf93	brown	-0.4974151	-0.6838423	0.17579268	0.11525453	0.63460599	-0.0923447
cg25555772	P2RY6;P2RY6;P2RY6;P2R	brown	-0.216545	-0.5451864	0.26481211	0.20852933	0.73112568	0.05148014
cg25922205	PBX1;PBX1;PBX1;PBX1;P	brown	-0.1538712	-0.5572732	0.15392316	0.24338782	0.76254949	0.15367275
cg25062539	IL7;IL7;IL7;IL7;IL7	brown	-0.3102922	-0.7514417	0.19094883	0.12857359	0.81512553	0.0479603
cg20454002	HLX;HLA-AS1;HLX	brown	-0.1177126	-0.6270915	0.0418094	0.16205701	0.7738594	0.16738662
cg13381521	ST6GALNAC4;ST6GALNA	brown	-0.1491475	-0.5945121	0.20322047	0.27158937	0.85365603	0.14034219
cg27260887	NR4A1;NR4A1;NR4A1;NR4	brown	-0.4638665	-0.5402533	0.19039449	0.09709725	0.56182336	-0.0718161
cg21239627	EVI5L;EVI5L	brown	-0.0509355	-0.3811601	0.28265682	0.33316731	0.74754419	0.15678387
cg15313204	ARHGAP30;ARHGAP30;A	brown	0.32331834	0.4189663	-0.1935539	-0.1760235	-0.3846326	-0.0702142
cg03130056	CTSA;CTSA;CTSA	brown	-0.0845243	-0.3823352	0.31160719	0.47202664	0.66205266	0.14457265
cg25356935	PDZD3;PDZD3;PDZD3;PDZ	brown	-0.125036	-0.3418826	0.43812194	0.20716543	0.41807227	0.21582433
cg09364688	RP4-728D4.2;RP4-	brown	-0.2045597	-0.5822449	0.12760922	0.03235936	0.57442012	0.07148936
cg15104158	FGF12;FGF12;FGF12;FGF1	brown	-0.3215414	-0.8070348	0.17602031	0.1315072	0.75372631	0.10138311
cg09253762	METTL1;METTL21B;METT	brown	-0.4285555	-0.6052784	0.10334837	0.06990949	0.69307141	-0.0232284
cg19523667	ZMIZ2;ZMIZ2;ZMIZ2;ZMIZ	brown	0.05746721	-0.4055462	0.32023067	0.43661062	0.74934357	0.3490091
cg15079885	TINAGL1;TINAGL1;TINAG	brown	-0.1984273	-0.6457706	0.16841865	0.22276746	0.91589707	0.0604015
cg01445659	ABHD8;ABHD8;MRPL34	brown	-0.0289887	-0.4252714	0.03829134	0.32346845	0.79404034	0.06125996
cg24808271	ZNF17	brown	-0.1201095	-0.4130635	0.22857837	0.33173444	0.53488285	0.18159019
cg21787305	CDC42EP4;CDC42EP4	brown	-0.2480104	-0.6954781	0.18283553	0.23930589	0.87674173	0.05807496
cg07362213	C6orf52;C6orf52;C6orf52;C6	brown	-0.3538609	-0.6736908	0.17987579	0.16325952	0.6661136	-0.0078631
cg25740200	ATOX1;G3BP1;G3BP1;G3B	brown	-0.4751106	-0.490673	0.27778116	0.05424856	0.51120584	-0.0783662
cg14378321	GPR108;GPR108;GPR108;G	brown	-0.4152745	-0.5533054	0.16992664	0.11322714	0.52145343	-0.051332
cg18108087	FGGY;FGGY;FGGY;FGGY	brown	-0.3044192	-0.6053813	0.01130571	0.04593735	0.63391385	-0.0471741

cg05142461	KAT5;RNASEH2C	brown	-0.1335036	-0.4423239	0.21569507	0.38427224	0.70271253	0.08053111
cg14844130	VPREB1;VPREB1	brown	-0.5185876	-0.5982105	0.03453218	0.09055504	0.53820264	-0.1568489
cg21235823	GOLGA5;GOLGA5;GOLG	brown	-0.265878	-0.621309	0.02705322	0.17785368	0.70449964	0.11060064
cg15146567	ZNF662;ZNF662;ZNF662;Z	brown	-0.0141386	-0.4045847	0.44205819	0.63476078	0.68629461	0.23106834
cg13745346	CBFA2T3;CBFA2T3;CBFA2	brown	-0.2498611	-0.6103025	0.15281445	0.30755411	0.86246864	0.0470715
cg27474900	EIF4E3;EIF4E3;EIF4E3;EIF4	brown	0.23242324	0.63316127	0.03856852	-0.08525	-0.749023	0.00428488
cg13736131	ST5;ST5;ST5;ST5;ST5;ST5;S	brown	-0.0327342	-0.3536824	0.09381698	0.19508647	0.65290169	0.1370573
cg26372927	ANK3;ANK3;ANK3;ANK3;	brown	-0.225155	-0.6129034	0.21140097	0.08110595	0.58977038	0.14974967
cg21054776	MON1A;MON1A;MON1A;	brown	-0.435737	-0.5662297	0.20091615	0.24195738	0.67427992	-0.1162225
cg09479314	CTA-398F10.2	brown	0.45877122	0.64159242	-0.0572458	-0.0811404	-0.7288208	0.16601312
cg18763727	BCKDHA;BCKDHA	brown	-0.0523624	-0.5565222	0.05942112	0.31830093	0.93110177	0.14436267
cg14511702	TRAJ9	brown	0.35850866	0.72437001	0.19384415	-0.080852	-0.8145779	0.096688
cg19758746	TRAJ39;TRAJ38	brown	0.28099298	0.70147895	-0.1956279	-0.2011697	-0.8176422	-0.1197938
cg06945974	RP11-	brown	0.44178112	0.55583314	0.02151087	-0.0074714	-0.5419072	0.18661042
cg00921350	RP11-401P9.5;RP11-	brown	-0.0739414	-0.1038216	0.20607467	0.36904058	0.34900379	0.13317005
cg03101763	LTBP3;LTBP3;LTBP3;LTBP	brown	-0.1859715	-0.4670164	0.28516613	0.23676583	0.71648825	0.14581569
cg05439914	ADAR;ADAR;ADAR	brown	0.09726714	-0.3999373	0.20861556	0.59778668	0.78751879	0.34274554
cg14171928	RP5-1187M17.7	brown	-0.0291167	-0.4193088	0.22723574	0.34174866	0.70250834	0.13512401
cg26191576	RPS6KL1;RPS6KL1;RPS6K	brown	-0.3549416	-0.5690082	0.19857616	0.12672931	0.60898248	-0.0553405
cg24729228	APOC3;APOC3;APOC3;AP	brown	-0.2049689	-0.6065048	0.10969884	0.21615762	0.82451125	0.09148985
cg22739025	GRM2;GRM2;GRM2;GRM2	brown	-0.3040387	-0.4577791	0.14317441	0.04784829	0.39925324	-0.0129193
cg05990607	RC3H1;RP11-160H22.3	brown	-0.4214419	-0.6600205	0.07216321	0.06174803	0.70144241	-0.1024132
cg03074946	PXN;PXN;PXN;PXN;PXN;	brown	0.07813679	-0.3902273	0.07587533	0.53143064	0.7737823	0.22379355
cg02671819	AC103810.2	brown	-0.2859523	-0.6682631	0.14906391	0.16368267	0.83216591	0.05143834
cg07693844	TMOD4;TMOD4;TMOD4;T	brown	-0.027559	-0.4983741	0.25512312	0.30724048	0.70360767	0.26782786
cg26821540	PRDM11	brown	-0.1888542	-0.490773	0.19242913	0.20527688	0.67953417	0.07655355
cg11401486	ARAP3;ARAP3	brown	-0.1259695	-0.1353109	0.37546781	0.32209026	0.31332616	0.19374583
cg18402325	SLC15A4;SLC15A4;SLC15	brown	0.23252315	0.68656619	-0.1531516	-0.1138428	-0.716357	-0.0230623
cg08536094	C9orf96;C9orf96;C9orf96	brown	-0.1348121	-0.6099509	0.12368733	0.30693073	0.93471498	0.09895432
cg17332198	AVIL;AVIL;AVIL;AVIL;AV	brown	-0.1185223	-0.5838676	0.13665485	0.30657909	0.93263828	0.12755494
cg05664072	PER2;PER2;PER2	brown	-0.4256876	-0.598197	0.29300211	0.15118408	0.56091712	-0.0893775
cg02672144	SPNS2;SPNS2;SPNS2;SPN	brown	-0.1778802	-0.2280622	0.34193921	0.23771459	0.43309388	0.12527556
cg19388312	SRSF10;SRSF10;SRSF10;S	brown	0.19147603	0.69771921	-0.0146591	-0.1679954	-0.8596541	-0.1076868
cg05683165	PRODH;PRODH;PRODH;P	brown	0.21861631	0.65149208	0.10591144	-0.1311624	-0.7449871	-0.0500169
cg25602159	ADCK4;ADCK4;ITPKC	brown	-0.5234847	-0.5082665	0.07147299	0.10242792	0.5270085	-0.3047603

cg12278431	DDAH1;CYR61;CYR61;CY	brown	-0.3052146	-0.659104	0.12291072	0.1262873	0.75317547	-0.0164816
cg23657639	PIEZO1	brown	-0.3220516	-0.4704729	0.38170709	0.16408186	0.40314603	0.07469344
cg02555241	C20orf96;C20orf96;C20orf96	brown	-0.4762339	-0.4584933	0.05368005	0.04612639	0.43446936	-0.2022859
cg05083507	CCDC81;CCDC81;CCDC81	brown	-0.041704	-0.3767978	0.16370542	0.21722619	0.48117331	0.14811574
cg12178807	ZNF292	brown	0.02030774	-0.1763297	-0.06275	0.36341451	0.3486396	0.14628986
cg05148829	KCNK1	brown	-0.3092104	-0.7572192	0.14798121	0.08266529	0.71084517	0.09841468
cg14803663	POC1B;POC1B;POC1B;PO	brown	-0.3777336	-0.5409576	0.23289367	0.13755304	0.59490009	-0.1245723
cg06973172	CMIP;CMIP	brown	0.19579373	-0.2541496	0.23720556	0.4627774	0.74364189	0.3029815
cg27073068	REST;REST	brown	0.22491603	0.6158737	-0.1027223	-0.1219672	-0.7673925	-0.0567444
cg23990557	IGF1R;IGF1R	brown	0.06494292	-0.4066355	0.10371357	0.58755466	0.76854004	0.19079905
cg20822990	ATP13A2;ATP13A2;ATP13	brown	-0.313719	-0.339104	0.26122789	0.28650754	0.50903406	-0.0931874
cg01577604	CCDC88B;CCDC88B;CCDC	brown	-0.2138935	-0.5405193	0.36138037	0.21617741	0.69336946	0.10913371
cg20749059	SPATA12;ARHGEF3;ARH	brown	-0.4233703	-0.5967344	0.04278394	0.12686699	0.68008837	-0.0456809
cg18431127	EPB42;EPB42;EPB42;EPB42	brown	-0.0558338	-0.3638874	0.11462419	0.48693631	0.79512096	0.14977848
cg06217745	PPIL1;C6orf89;PI16	brown	-0.3144883	-0.63388	0.06881728	0.15223799	0.74098194	0.0895833
cg05826823	CIZ1	brown	-0.0142872	-0.4468076	0.28266011	0.42823544	0.83958692	0.2983926
cg04514683	ABP1;ABP1;ABP1;ABP1;A	brown	-0.1905972	-0.5991559	0.15152766	0.24761963	0.85179574	0.14325393
cg06680529	NOTUM	brown	-0.0307974	-0.359001	0.39116598	0.38850268	0.63623199	0.29380754
cg12624792	CSNK1E;CSNK1E;CSNK1E	brown	-0.2796669	-0.6153228	0.27587291	0.12195802	0.66580077	0.0105574
cg11033495	TRAJ45;TRAJ44	brown	0.40764447	0.7512574	0.10191555	-0.0979651	-0.8097299	0.06440882
cg25293251	GOLGA5;GOLGA5;GOLG	brown	-0.2293116	-0.5542885	0.05586963	0.20740834	0.65858695	0.12866867
cg20537629	MAGI2-	brown	-0.3909759	-0.8092034	0.13024463	0.04292046	0.79545114	0.02001093
cg03508063	DLG4;DLG4;DLG4;ACADV	brown	-0.1002385	-0.3462623	0.23562242	0.4157261	0.67087319	0.12013082
cg12073779	CRYGD	brown	-0.1088551	-0.535635	0.15976657	0.22772625	0.68306076	0.09314922
cg00563932	PTGDS;PTGDS;PTGDS;PT	brown	-0.0116062	-0.3669336	0.17923078	0.34445752	0.78015355	0.23731613
cg22476084	FXYD6;FXYD6;FXYD6;RP	brown	-0.4056425	-0.6721631	0.08146239	0.04249118	0.68662806	-0.0790045
cg20249500	YPEL4;YPEL4;AP000662.4;	brown	-0.4267289	-0.7577021	0.16013171	0.10439	0.70523157	-0.094717
cg11998049	RP1-180E22.3	brown	-0.1485492	-0.4784922	0.25402944	0.2356542	0.61091856	0.18995067
cg07881013	MRPL33;MRPL33;MRPL33;	brown	-0.069663	-0.4316156	0.13699239	0.24858657	0.63763766	0.24219824
cg15313891	AVP	brown	-0.0528393	-0.4168971	0.35790024	0.17378613	0.49698828	0.24652156
cg05545167	AL109767.1	brown	-0.3677744	-0.4673205	0.30258681	0.03813634	0.44786876	-0.0763259
cg09404862	TEX33;TEX33;TEX33	brown	-0.2914886	-0.4739627	0.19629331	0.27267159	0.55688812	0.03639315
cg22978287	GPD1;GPD1;GPD1;GPD1;G	brown	-0.0098955	-0.2181559	0.36463474	0.2840938	0.41766282	0.25377857
cg17691657	SLC4A4;SLC4A4	brown	-0.445651	-0.5232053	0.22021413	0.03669067	0.49400517	-0.0089994
cg15906503	DIDO1;DIDO1;DIDO1;DID	brown	0.33770031	0.55631958	0.06681536	0.00795331	-0.5544174	0.19584546

cg03579665	CDHR5;CDHR5;CDHR5;CD	brown	-0.4305667	-0.7163356	0.02599708	0.07105841	0.71918289	-0.0810005
cg17423763	SLC27A3;SLC27A3;SLC27	brown	0.03274865	-0.4428579	0.32903538	0.41555772	0.74605327	0.31452652
cg00176496	PITPNM3;PITPNM3	brown	-0.4154743	-0.4716335	0.20573866	0.09797787	0.43283812	-0.0632836
cg15194019	STK40;STK40;STK40;STK4	brown	-0.3499667	-0.1852037	0.37821087	0.21877078	0.27533661	0.04142301
cg09308553	NUP93;NUP93;NUP93;NUP	brown	-0.3168792	-0.4272279	0.31688634	0.16439822	0.46437661	-0.0092796
cg25058654	MBTPS1;MBTPS1;MBTPS1	brown	-0.1571011	-0.5251423	0.25855433	0.35625773	0.86176543	0.1496654
cg06115314	PARP12;PARP12;PARP12	brown	-0.123869	-0.3680262	0.12715903	0.36362559	0.51493291	0.08524232
cg02025412	NR4A1	brown	-0.3832477	-0.5567436	0.13041486	0.1482765	0.65507024	0.00535208
cg10585278	RP11-718B12.2	brown	-0.3242953	-0.6616361	0.24219513	0.10479711	0.65803051	0.04448473
cg21433125	HDAC9;HDAC9;HDAC9;H	brown	-0.4092408	-0.7820899	0.08709022	0.0354017	0.72546761	-0.0287211
cg15446391	WT1	brown	-0.0236872	-0.3227222	0.26506848	0.2509178	0.47191565	0.24089986
cg14408428	ZBTB43;ZBTB43;ZBTB43;Z	brown	0.3894937	0.60232019	0.31491971	-0.0308855	-0.7135814	0.0935195
cg11218699	CLK3;CLK3;CLK3;CTD-	brown	-0.1753019	-0.4130557	0.11685333	0.29834338	0.65031392	-0.0018571
cg24727203	PPP1R3B;PPP1R3B;RP11-	brown	-0.2429518	-0.5976953	0.10717916	0.19743809	0.76802608	0.01443811
cg14681958	CDHR5	brown	-0.2305496	-0.5043003	0.15254127	0.23040199	0.74104944	0.02976481
cg09547880	IL1A	brown	-0.3382942	-0.6443834	0.14988306	0.1506776	0.76821306	-0.0023667
cg10969211	YPEL4;YPEL4;AP000662.4;	brown	-0.1568468	-0.5786342	0.28564449	0.22904516	0.75027594	0.10324041
cg18151608	RP11-996F15.2	brown	-0.3006734	-0.4772733	0.06896294	0.18746792	0.58373366	-0.0025092
cg05615150	ARPP21;ARPP21;ARPP21;	brown	0.32398246	0.6746671	-0.1929934	-0.1982018	-0.8093872	-0.0243912
cg05820491	MYL9;MYL9;RP5-	brown	-0.2258909	-0.4670964	-0.0017134	0.16983541	0.65399529	-0.0292292
cg24404329	TES;TES;TES;TES;TES	brown	0.34257841	0.41166087	-0.2598708	-0.1568596	-0.5838928	0.04611087
cg17384889	NKAPL;ZKSCAN4	brown	-0.3069822	-0.5903419	0.15897091	0.00463447	0.50855543	0.03360765
cg15345285	MPPED2;MPPED2;MPPED	brown	-0.186438	-0.5965441	0.09060029	0.10936294	0.6853345	0.08101629
cg17913566	AP000695.6	brown	0.01045965	-0.4859083	0.20439917	0.28395873	0.80035464	0.29153001
cg26287978	RP11-	brown	-0.2243425	-0.5652696	0.2526982	0.28389785	0.73168885	0.16434141
cg08574671	NAT6;HYAL3;NAT6;NAT6;	brown	-0.0681801	-0.565429	0.3047555	0.49492352	0.85309455	0.2311339
cg12486287	VASN;CORO7;CORO7;CO	brown	-0.2985654	-0.8055192	0.12602376	0.111545	0.83879202	0.04270277
cg06327842	SLC7A6;SLC7A6;SLC7A6;S	brown	-0.0219446	-0.4022833	0.4143351	0.32722954	0.7085731	0.17109522
cg10798764	ATP6V1G2;NFKBIL1;NFK	brown	-0.3775494	-0.661617	0.12855216	0.0946715	0.72714946	-0.0915319
cg09041527	RP11-	brown	-0.4809406	-0.5000424	0.27037378	0.12041748	0.461991	0.00451801
cg08855908	TRAJ46;TRAJ45;TRAJ47	brown	0.39347034	0.70560799	0.10782936	-0.0614741	-0.7923285	0.12628571
cg09504658	CLEC2D;CLEC2D;CLEC2D;	brown	0.18506522	0.70548988	-0.0600256	-0.2129852	-0.93689	-0.0838323
cg20203041	DUSP3;DUSP3;C17orf105	brown	-0.3742479	-0.786515	0.03380535	0.07374624	0.81791037	-0.0758747
cg17205250	SQRDL;SQRDL;RP11-	brown	-0.2701324	-0.5411867	0.25871148	0.09274956	0.66853421	0.04612271
cg09778980	ZNF219;ZNF219;ZNF219;Z	brown	-0.1154761	-0.5386564	0.14976438	0.31837844	0.82027851	0.1644875

cg18123385	ACTRT2;ACTRT2;ACTRT2	brown	-0.2339533	-0.4722275	0.28930046	0.00505132	0.4970776	0.06876829
cg21292067	RP11-459C13.1	brown	-0.1942581	-0.615239	0.1831475	0.22634454	0.80786763	0.15436802
cg17165759	RP5-1068B5.3	brown	0.24999623	0.68482456	0.13701733	-0.0504484	-0.7848057	0.02142969
cg06016199	DPEP2;DPEP2;DPEP2;DPE	brown	-0.2489066	-0.4913454	0.07716297	0.25279229	0.56749117	0.08405234
cg11812928	ADCK3;ADCK3;ADCK3;A	brown	-0.1113181	-0.5798552	0.21971297	0.30776153	0.87366368	0.16493501
cg06759845	MEF2D;MEF2D;MEF2D;M	brown	-0.3679802	-0.631634	0.01166173	0.07388182	0.76722958	-0.1028358
cg26804336	PIK3R1;PIK3R1;PIK3R1;PI	brown	0.1870067	-0.165118	0.53044795	0.73187366	0.49175346	0.48781222
cg05490878	ST6GALNAC6;ST6GALNA	brown	0.31168391	0.58085609	-0.1705233	-0.0371109	-0.5011701	-0.1544554
cg21891499	CLPTM1L	brown	0.45056921	0.56136715	-0.1759093	0.07325932	-0.49405	0.13194802
cg03741482	MRPL41	brown	-0.2402367	-0.5929592	0.27245634	0.14878542	0.63798071	0.10100708
cg10178628	PLVAP	brown	-0.3235522	-0.6989919	0.23043258	0.17799674	0.72841092	0.06481338
cg27370812	MIR647;UCKL1;UCKL1	brown	-0.1562641	-0.4324366	0.22181454	0.3790549	0.76724004	0.1010429
cg01184315	SPNS3;SPNS3;RP13-	brown	-0.3918267	-0.4964875	0.14725013	0.06643981	0.58354497	-0.2089195
cg02053425	SETD8;SETD8;SETD8	brown	-0.0543618	-0.5621904	0.24389398	0.44489254	0.90560543	0.19963179
cg21414636	NRBP2	brown	-0.1886413	-0.4749281	0.1359327	0.15699315	0.64898386	0.0803518
cg00810989	ZCCHC7;ZCCHC7;ZCCHC7	brown	0.44519267	0.4924274	-0.0454089	-0.041065	-0.4512289	0.06367413
cg08865926	ZCCHC6;ZCCHC6;ZCCHC6	brown	0.25266446	0.66902946	0.25288865	-0.1603633	-0.7969639	0.09305314
cg17246938	MPST;TST;TST;MPST;TST;	brown	-0.3133983	-0.354926	0.15322405	0.13340337	0.38100301	0.04322029
cg06143012	RDM1;RDM1;RDM1;RDM1	brown	-0.1000702	-0.3120371	0.38144332	0.22467991	0.4879143	0.38182254
cg15516759	APOL3;APOL3;APOL3;AP	brown	0.10443616	0.60458982	-0.0606654	-0.3168962	-0.8798648	-0.1540444
cg01752257	SLC12A6;SLC12A6;SLC12	brown	0.22671733	0.77581791	-0.042713	-0.1671553	-0.8864332	-0.0702602
cg11852794	CATSPERB;CATSPERB;CA	brown	0.30928174	0.68133485	0.08060993	-0.1577343	-0.8455876	0.03559128
cg03819286	MGRN1;MGRN1	brown	0.04616605	-0.4854224	0.22652849	0.43849401	0.84706589	0.24092468
cg25907322	SLC26A8;SLC26A8;SLC26	brown	-0.3548065	-0.6408738	0.17695563	0.05108824	0.6960968	-0.0204824
cg02265748	DNAJC18;DNAJC18;DNAJ	brown	0.41062039	0.68779944	-0.135652	-0.0410158	-0.63628	0.11163783
cg16436872	SH2D6;SH2D6;SH2D6;SH2	brown	-0.1633267	-0.614028	0.17358146	0.24160275	0.82348242	0.12684388
cg07957619	GTPBP2;GTPBP2;GTPBP2;	brown	-0.1784842	-0.6255269	0.06965909	0.29187456	0.88417042	0.11384719
cg26886599	SUOX;SUOX;SUOX;SUOX	brown	-0.0565693	-0.3225292	0.10971895	0.24477279	0.53416853	0.18078679
cg22513955	SERPINF1;SERPINF1;SERP	brown	-0.1175662	-0.5242945	0.21528254	0.28352055	0.84925205	0.09571645
cg20483374	C1QTNF5;C1QTNF5;C1QT	brown	-0.2360373	-0.7215034	0.14263241	0.19637766	0.8208167	0.00261897
cg01198591	SNHG12;SNHG12;SNORA1	brown	-0.1812326	-0.2982234	0.04847661	0.21647511	0.5218869	0.09492192
cg24375490	RP11-117D22.2	brown	-0.1365728	-0.5839213	0.13191813	0.28595271	0.89350019	0.10935966
cg25744957	RP11-367J11.3;RP11-	brown	-0.0371721	-0.266994	0.44208951	0.38267502	0.49444811	0.33922088
cg18152276	RP1-63G5.5;ELFN2	brown	-0.1002739	-0.289676	0.07929976	0.30043991	0.4629575	0.18551594
cg05544793	PLCXD2	brown	-0.2642193	-0.2276135	0.29768692	0.27279921	0.38973787	0.14687514

cg19296400	UBXN11;UBXN11;UBXN1	brown	0.25983284	-0.2428298	0.06878674	0.67590521	0.70687635	0.30497053
cg06734854	SNORD38;FZR1	brown	0.15541881	-0.2155538	0.34975882	0.72554409	0.48059241	0.59916108
cg10137821	GNAI2;GNAI2;GNAI2	brown	-0.1870598	-0.6188805	0.22027122	0.35794311	0.86777069	0.17640797
cg09608718	FAM149A;FAM149A;FAM	brown	-0.3586588	-0.3818345	0.34542422	0.19447308	0.33783993	-0.0180963
cg11366363	GUCY1B2;GUCY1B2;GUC	brown	-0.2081858	-0.6352431	0.15826904	0.24544797	0.86601613	0.06573397
cg04954726	ZNF292	brown	-0.1269409	-0.2887466	-0.0416548	0.27871223	0.4246732	0.02499887
cg12469778	B3GNT5;B3GNT5;B3GNT5;	brown	-0.1675665	-0.6184365	0.22917517	0.21880298	0.87531872	0.18252152
cg26924223	ZNF563;ZNF563	brown	0.19356956	-0.0206535	0.13545342	0.51871279	0.39669241	0.30783525
cg14950298	SLC11A2;SLC11A2;SLC11	brown	-0.2386737	-0.586063	0.22199315	0.25260221	0.77004537	0.06419417
cg06371582	TMEM154;TMEM154	brown	-0.1651199	-0.3391992	0.19710955	0.31257629	0.46060756	0.09528334
cg10142237	CSNK1E;CSNK1E;CSNK1E	brown	-0.1313826	-0.6020038	0.2143036	0.31449288	0.90537449	0.13837985
cg18651300	GALNT10;GALNT10;GALN	brown	-0.2489711	-0.4751326	0.34014361	0.26732718	0.56792489	0.08659303
cg20555282	RP11-34F13.3;RP11-34F13.3	brown	-0.3405976	-0.6331341	0.09425334	0.10459771	0.6331028	-0.0287036
cg19324606	C17orf62;C17orf62;C17orf62	brown	-0.0067579	-0.2403102	0.12221466	0.48671659	0.57070764	0.16533494
cg13119976	SIL1;SIL1;SIL1;SIL1;SIL1;SI	brown	-0.0543351	-0.3780496	0.00551402	0.18132107	0.63717018	0.10808809
cg14539366	COL17A1;COL17A1	brown	-0.0566101	-0.5676865	0.2260761	0.36222819	0.89667526	0.21886019
cg01710179	HM13-	brown	-0.2101814	-0.6131847	0.19940569	0.25219354	0.8122919	0.03430585
cg01717615	PLEKHG6;PLEKHG6;PLEK	brown	-0.284221	-0.5686289	0.22381833	0.20031273	0.66858555	0.18683224
cg04347447	CACNA2D2;CACNA2D2;C	brown	-0.3630128	-0.664699	0.1388525	-0.0004777	0.6857114	0.04269557
cg20234170	CAT	brown	-0.4136225	-0.5655595	0.06562656	0.02599367	0.57041208	-0.1037314
cg05873389	C10orf32;AS3MT	brown	-0.1751426	-0.3946755	0.15773631	0.20176996	0.49522903	-0.0302277
cg14563558	TOLLIP;TOLLIP;TOLLIP;T	brown	-0.1681259	-0.5348828	0.33867465	0.2329909	0.71684052	0.09705698
cg04338129	EFEMP2;EFEMP2;EFEMP2;	brown	0.02268513	-0.5249981	0.04218838	0.2830734	0.737874	0.25339388
cg07527259	CCDC168	brown	-0.2371624	-0.5848279	0.07760101	0.16000566	0.803611	-0.0227014
cg17134838	RP11-804A23.1	brown	0.26264238	-0.0889887	0.09972989	0.71045302	0.52763415	0.319966
cg09926463	ANO10;ANO10;ANO10;AN	brown	-0.1329533	-0.4231609	0.20704308	0.22155141	0.74029917	0.06787993
cg13921605	FIBCD1;FIBCD1;RP11-	brown	-0.3388303	-0.5732102	0.07257907	0.13016818	0.65021418	-0.0803508
cg13997680	ZKSCAN4;NKAPL;ZKSCA	brown	-0.2187544	-0.4625975	0.08078791	0.08055983	0.46399494	0.06399452
cg08407263	CRIP3;CRIP3;CRIP3;CRIP3;	brown	-0.0155635	-0.5551931	0.28887292	0.39286961	0.91675504	0.20520015
cg00792107	RP13-554M15.6;RP13-	brown	-0.2516056	-0.2565009	0.23629328	0.1435867	0.30024342	0.04040751
cg26467422	ADAM33;ADAM33;ADAM	brown	-0.3331384	-0.3509083	0.17318658	0.12675981	0.34732202	-0.1057956
cg05305497	TRDV2;AE000661.37;AE000	brown	-0.0796815	-0.5099384	0.20415539	0.32741328	0.82859038	0.07749031
cg12273502	C18orf1	brown	-0.125238	-0.5544786	0.03946579	0.17150881	0.81689131	0.11620444
cg15741887	FOXN3;FOXN3;RP11-	brown	-0.3098922	-0.6447346	0.24304242	0.15566971	0.80029528	0.03665605
cg08825848	TXNRD2;TXNRD2;TXNRD	brown	-0.1690786	-0.6607789	0.10873487	0.17659591	0.9245759	0.10238361

cg22416867	ALDH1A2;ALDH1A2;ALD	brown	-0.3953636	-0.4220047	0.25248231	0.18051981	0.44955632	-0.0327193
cg25821245	LRRC2;LRRC2;LRRC2;LRR	brown	-0.2417318	-0.5542137	0.20643066	0.29675562	0.72989422	0.08455432
cg05409540	ZNF292	brown	-0.0485689	-0.3054519	-0.0476495	0.31536232	0.47342273	0.11323958
cg06991495	CSNK1E;CSNK1E;CSNK1E	brown	-0.1736463	-0.6106835	0.20088865	0.30140945	0.87822661	0.16995451
cg04035392	DHX30;DHX30;DHX30;DH	brown	-0.3704244	-0.1924577	0.0808549	0.1568923	0.28953709	-0.2169766
cg15613488	PLCH1	brown	-0.1106572	-0.5109946	0.23839217	0.19594682	0.58291547	0.12800288
cg17561417	FBLN2;FBLN2	brown	-0.3582033	-0.7596376	0.0450704	0.03772729	0.70912613	0.02446379
cg15682091	RP11-473J6.1;RP11-473J6.1	brown	-0.1768206	-0.1837762	0.18496866	0.20201823	0.41384896	-0.0076469
cg26272069	GABBR1;GABBR1;GABBR	brown	0.05589236	-0.3788903	0.08337823	0.46145012	0.78716556	0.19231855
cg06629493	TRPM2	brown	-0.1650992	-0.5850625	0.31480413	0.24711664	0.75631693	0.24800425
cg02600983	SYTL1;SYTL1;SYTL1	brown	-0.3230204	-0.3303593	0.33061603	-0.098375	0.18517213	-0.0305705
cg11717907	BCL2L2;BCL2L2;BCL2L2-	brown	-0.0453171	-0.5358153	0.23044689	0.39566024	0.89612302	0.25626699
cg19968134	CCDC136;CCDC136;CCDC	brown	0.0596389	-0.4397465	0.16986245	0.49293216	0.86555346	0.2146529
cg03264209	CREBBP	brown	-0.159229	-0.3281933	0.29713197	0.23662671	0.3939044	0.29393295
cg24778896	UBAP1L	brown	-0.2376909	-0.3483104	0.20893113	0.17188884	0.42748534	0.11571424
cg15290726	PRAGMIN	brown	-0.3447388	-0.6909347	0.05073021	0.16754217	0.73152084	-0.0158758
cg05242561	HOMER2	brown	-0.2318733	-0.3898911	0.09775043	0.1913243	0.39935459	-0.0441906
cg09457812	PPAPDC3;PPAPDC3;PPAP	brown	-0.2104483	-0.44951	0.25081451	0.16521798	0.46600141	0.06281517
cg22509164	LRRC41;LRRC41;LRRC41;L	brown	-0.1892016	-0.6403082	0.27906562	0.26647897	0.87293074	0.14361343
cg14678203	ADAMTSL5;ADAMTSL5	brown	-0.2608067	-0.5139945	0.29253207	0.13370923	0.55104073	0.05254834
cg04856022	PPT2;PPT2;PRRT1;PRRT1;P	brown	-0.0493602	-0.389193	0.40405717	0.40840373	0.52390831	0.3166298
cg13102294	PRRT1;PRRT1;PPT2;PPT2;P	brown	-0.2477134	-0.538937	0.12033938	0.1692468	0.64141698	0.03964734
cg01171329	CCL19	brown	-0.1520226	-0.4963739	0.24417901	0.28295613	0.80283061	0.12474535
cg21586296	LINC00338;LINC00338;LIN	brown	-0.4004815	-0.321947	0.22757501	0.2327022	0.32195112	-0.0947806
cg03645419	AIFM2;AIFM2	brown	-0.052869	-0.4595507	0.08525447	0.22984425	0.68139511	0.12861878
cg19426048	STOML1;STOML1	brown	-0.3448423	-0.7207484	0.09466651	0.19623043	0.87414142	-0.0115268
cg19014419	ZNF300;ZNF300;ZNF300;Z	brown	-0.2877014	-0.7609804	0.12933717	0.0639848	0.74326081	0.15718079
cg24251035	ZC3HAV1L;ZC3HAV1L	brown	-0.3221514	-0.465187	0.10128739	0.18886221	0.62648712	-0.0975382
cg10329874	PC	brown	-0.0512001	-0.3652149	0.45053027	0.22118739	0.62271143	0.22731494
cg21372755	LINC00322	brown	-0.205043	-0.6239278	0.09936449	0.23718778	0.85414387	-0.0392718
cg27635372	SH3BP2;SH3BP2;SH3BP2;S	brown	-0.1737034	-0.4490666	0.27844485	0.11004719	0.64568922	0.11667947
cg12069668	RP11-638I2.8	brown	-0.0899518	-0.5356111	0.25578692	0.37749407	0.85007272	0.17800659
cg23220372	RP11-1079K10.3;RP11-	brown	-0.3101262	-0.4317378	0.07147665	0.17500821	0.53122523	-0.0930543
cg13345380	RP11-48B14.2;TMEM93	brown	-0.15852	-0.6411663	0.21273398	0.34010934	0.81488511	0.1996015
cg14849597	SRD5A3	brown	-0.2564964	-0.6171842	0.08816729	0.12385514	0.84059049	0.09054822

cg05579613	ZNF32;ZNF32;ZNF32;ZNF3	brown	-0.36016	-0.6003651	0.18390343	0.05640596	0.57521901	-0.0806833
cg07880854	FBLN7;FBLN7;FBLN7	brown	-0.0639311	-0.4676868	0.03237148	0.20669272	0.63434805	0.2728573
cg02502358	C17orf108;C17orf108	brown	-0.3052946	-0.5191244	-0.2988807	0.1382205	0.61902154	-0.0522553
cg10537193	PISD;PISD;PISD;PISD;PIS	brown	-0.3896657	-0.3340813	-0.0238037	0.09121648	0.43950826	-0.1702262
cg23322868	CDKN2A;CDKN2B-	brown	-0.1941758	-0.6180551	0.11246527	0.24929133	0.85145674	0.0334459
cg20945221	RP5-1053E7.1	brown	-0.0631335	-0.4974681	0.07135816	0.40065264	0.91025292	0.12284521
cg11184485	CTC-558O2.2	brown	-0.4105627	-0.6024981	0.17700007	0.04473886	0.59911165	-0.0455339
cg11228717	DERA;DERA;DERA;DERA;	brown	-0.2761775	-0.4096332	0.0873042	0.15485749	0.37370065	0.08098909
cg06379531	TSEN15;TSEN15;TSEN15;T	brown	-0.3458196	-0.718374	0.23446642	0.08466767	0.71584873	0.07235237
cg03680238	MIR3654;EEF1G;EEF1G;EE	brown	-0.1882997	-0.4325474	0.03189224	0.22512935	0.60401479	0.08874815
cg23146882	CMTM5;CMTM5;CMTM5	brown	-0.1046752	-0.5360626	0.20146313	0.33364649	0.86765874	0.19934112
cg19083777	RP11-386G11.3	brown	-0.0988408	-0.6261918	0.1734195	0.34923724	0.94940506	0.12960931
cg08187425	EFHD1	brown	-0.3064378	-0.6740522	0.17943988	0.18169095	0.77324021	0.089744
cg17531025	RP11-264B17.5	brown	0.24425097	0.72535329	-0.0516267	-0.1730057	-0.9279117	-0.0669103
cg08613370	RP3-454B23.1	brown	5.34E-05	-0.3327166	0.16000123	0.2983022	0.77236528	0.16464951
cg15314696	PCDHGA3	brown	-0.2767145	-0.5190785	0.15656814	0.04874475	0.50330113	0.00876239
cg10897447	CPNE7	brown	0.27751187	0.73573674	-0.0186843	-0.1603149	-0.8922342	0.00893926
cg13072940	MON1A;MON1A;MON1A;	brown	-0.3152288	-0.3451029	0.19113863	0.24352229	0.52999903	-0.1034943
cg13454258	RCN1;RCN1;RCN1;RCN1;R	brown	-0.3087863	-0.7493468	0.02897258	0.11176534	0.78267786	0.03654648
cg03554335	C17orf72	brown	-0.0701917	-0.5857937	0.18503167	0.22981741	0.87879499	0.16183516
cg02014762	RP11-539G18.2;RP11-	brown	-0.1003846	-0.4304547	0.17349873	0.46712048	0.77966659	0.17244081
cg03562826	MFSD2A;MFSD2A;MFSD2	brown	-0.1464095	-0.4806118	0.34714854	0.3229216	0.77385459	0.16876989
cg23169111	AVP	brown	-0.1945714	-0.4894838	0.38398732	0.11372301	0.45852989	0.16162644
cg02446225	U7;MIR1238;ATG4D	brown	-0.1631274	-0.552193	0.22127865	0.23638996	0.77883445	0.0873021
cg13063704	PRKAR2A-AS1;PRKAR2A-	brown	-0.3714946	-0.4097008	0.01229484	0.07369795	0.52320696	-0.1002898
cg20199255	CATSPER4;CATSPER4	brown	-0.2734663	-0.4445838	0.14791259	0.1518862	0.73562354	-0.0202007
cg12205591	CRYAA;CRYAA;CRYAA	brown	-0.243103	-0.2214561	0.20647712	0.13622419	0.45744618	0.0630397
cg25241757	PSRC1;PSRC1;PSRC1;PSR	brown	-0.1990318	-0.6084392	0.09871339	0.18983251	0.88766423	0.0093607
cg24706115	TRPV4;TRPV4;TRPV4;TRP	brown	-0.3795957	-0.349502	0.26297287	0.13713555	0.40846129	-0.0419337
cg05086084	CHIT1;CHIT1	brown	-0.1789623	-0.5552368	0.32748667	0.24676455	0.58529471	0.10447354
cg16140568	C1QTNF5;C1QTNF5;C1QT	brown	-0.307192	-0.51304	0.12051651	0.2801453	0.71007872	-0.0248414
cg20884967	RNF224	brown	-0.1108175	-0.4219986	0.36058013	0.40429899	0.64700645	0.25662909
cg08020619	ZNRF3-AS1;ZNRF3	brown	-0.1743551	-0.497427	0.18741423	0.13378707	0.66674914	0.08019373
cg13408712	PRDM10;PRDM10;PRDM1	brown	-0.1105602	-0.4484182	0.30527211	0.33667902	0.63663862	0.30306706
cg17183905	TRPV4;TRPV4;TRPV4;TRP	brown	-0.3699469	-0.3866153	0.1799816	0.13036481	0.51707917	-0.1165529

cg08146609	HDAC9;HDAC9;HDAC9;H	brown	-0.4526545	-0.7136163	0.14068499	-0.0209655	0.63501645	-0.0335011
cg20365618	INPP5F;INPP5F;INPP5F	brown	0.02573581	-0.4272457	0.27340412	0.2450974	0.56184241	0.26540761
cg21232625	RXRB;COL11A2;COL11A2;	brown	-0.2498728	-0.618783	0.10054337	0.19512145	0.75868796	0.05994404
cg01810875	NCKAP1L;NCKAP1L;NCK	brown	-0.1517948	-0.463303	0.08460771	0.291511	0.72748942	0.14347342
cg03585755	COL9A3;COL9A3;COL9A3;	brown	-0.1544852	-0.4150977	0.05502696	0.18014906	0.58886376	0.04752159
cg00274203	COQ10A;COQ10A;COQ10	brown	0.19630858	-0.254798	0.24185607	0.59956411	0.69004738	0.30326279
cg08289839	CARS2;CARS2;CARS2;CA	brown	0.02070022	-0.5039497	0.25908619	0.45305113	0.78879309	0.39904356
cg16232674	CSF2RB;CSF2RB	brown	-0.1683231	-0.4707864	0.10724027	0.22088623	0.80606095	0.03807096
cg21226516	NR4A2;NR4A2;NR4A2;NR4	brown	-0.1646681	-0.6847723	0.20459346	0.19071904	0.82325474	0.10096498
cg23823732	USP39;C2orf68;USP39;C2or	brown	-0.3434955	-0.6675715	0.28589785	0.10798392	0.76003807	0.02907093
cg05906777	MIF4GD;MIF4GD	brown	-0.3501836	-0.5179873	0.16273221	0.09753163	0.716321	-0.0908665
cg00501774	PTGS1;PTGS1;PTGS1	brown	0.01502666	-0.4355386	0.25058458	0.37763543	0.79577101	0.19354976
cg17638833	CTD-2227I18.1	brown	0.23498534	0.66337645	-0.0112251	-0.2015779	-0.8990376	0.00464351
cg11046544	LPAR5;ACRBP	brown	-0.0086496	0.15937812	0.16653808	0.43076553	0.20143877	0.15808127
cg18340020	CIRH1A;RP11-70O5.1	brown	0.29343439	0.72868978	-0.0673266	-0.1619773	-0.8613173	0.05298788
cg00242449	FGF11;TMEM102;RP11-	brown	0.01420961	-0.3451801	0.37218011	0.37587832	0.61480497	0.31303559
cg01255315	PLD3;PLD3;PLD3;PLD3;PL	brown	-0.1012003	-0.4999788	0.24284502	0.20083453	0.78465161	0.21605178
cg27060381	TBC1D10A;TBC1D10A;TB	brown	-0.416767	-0.7136918	0.13719752	0.14447266	0.68269016	-0.0341011
cg25635581	DPYSL3;DPYSL3	brown	-0.2745994	-0.2930584	0.31906377	0.17537336	0.38997356	0.03334929
cg07702888	RHBG;RHBG;RHBG;CCT3;	brown	0.1701211	-0.3717984	0.18675148	0.43190418	0.72520653	0.43947114
cg04883329	KIAA1984;KIAA1984;KIA	brown	-0.0529728	-0.5580403	0.21858888	0.45051716	0.88926546	0.18723276
cg15582417	VOPP1;VOPP1;VOPP1;VO	brown	-0.1628484	-0.6140108	0.16207265	0.3411436	0.90803876	0.11025595
cg26955759	THRA;THRA;THRA;THRA	brown	-0.1122243	-0.2729825	0.12764682	0.40362575	0.4721602	0.04823753
cg09187087	PRDM11;PRDM11;PRDM1	brown	-0.0190805	-0.4040166	0.26734556	0.26331932	0.63222828	0.19286385
cg03393889	NTHL1;NTHL1;NTHL1;NT	brown	-0.0961374	-0.5461883	0.27057007	0.29771925	0.8278661	0.23842256
cg08513617	SNHG3;SNHG3;SNORA73	brown	-0.4259811	-0.3596286	0.08568637	0.09987889	0.37579725	-0.0970868
cg17498611	ARC	brown	-0.333331	-0.5242826	0.10147766	0.19297278	0.70580545	-0.0589715
cg27584762	CCNH;CCNH;CCNH;CCNH	brown	-0.3908297	-0.5016554	0.19683244	0.18658108	0.43733035	0.01691435
cg00640033	TRAJ26;TRAJ25	brown	0.28973737	0.71366546	0.02454006	-0.1819111	-0.8881711	0.01322357
cg22801433	TMEM184A;TMEM184A;T	brown	-0.3137719	-0.314175	0.19817658	0.02621902	0.29265599	-0.0299047
cg21115391	CCDC67;CCDC67	brown	0.23946066	0.58466411	0.18637769	-0.0955683	-0.5678416	-0.1765409
cg11157816	OLFML3;OLFML3;OLFML	brown	-0.3216133	-0.6294666	0.20048392	0.16335241	0.76885323	0.01796148
cg05219896	AC011648.1	brown	0.08309919	-0.2991316	0.1731881	0.56914454	0.71341835	0.13968544
cg26935168	RPL22;RPL22;RPL22;RPL22	brown	-0.3577187	-0.6768168	0.19544756	0.02618706	0.70915535	-0.0010284
cg05862431	BUD13;BUD13;AP006216.1	brown	-0.3814887	-0.6220143	0.09147175	0.03625121	0.57718032	0.07043815

cg25103338	AC078953.1;PLD1;PLD1;PL	brown	-0.3847119	-0.6881591	0.07720927	0.09306166	0.69998757	-0.0637776
cg19971833	LA16c-321D4.2	brown	-0.3616202	-0.5398687	0.15793773	0.17728407	0.60189384	-0.0297014
cg13253439	ATAT1;ATAT1;C6orf136;C6	brown	-0.1353104	-0.3286634	0.42803553	0.52645585	0.5221822	0.17956737
cg08010531	RN7SKP9;UBAC2;UBAC2	brown	0.19077147	0.62073459	-0.0619291	-0.2658552	-0.9126319	-0.056466
cg05745223	RP11-	brown	-0.3975465	-0.6046126	0.08581047	0.06943943	0.63099759	-0.0255552
cg11650891	LRRC2;LRRC2;LRRC2;LRR	brown	-0.1261707	-0.4820044	0.16278894	0.32065706	0.73818949	0.10335792
cg02079181	C1orf186;RP11-	brown	0.18492378	0.61370321	-0.1910297	-0.2295962	-0.8812557	-0.1215378
cg26957471	AC137932.5;AC137932.5;A	brown	-0.0720338	-0.516869	0.27952693	0.24096625	0.7931822	0.13056946
cg06070311	TMEM132A;TMEM132A;T	brown	-0.5150457	-0.7513573	0.07321265	0.04093145	0.70754136	-0.1557487
cg16944093	LIMS2;LIMS2;LIMS2;LIMS	brown	-0.2375123	-0.6327473	0.33140359	0.08137349	0.64388925	0.09632924
cg20061371	SHISA5;SHISA5;SHISA5;S	brown	-0.1927531	-0.6516816	0.12603011	0.20473464	0.92013711	0.06018928
cg23440095	SWAP70;SWAP70;SWAP70	brown	-0.3085741	-0.4372233	0.13838381	0.22556498	0.60818881	0.04229002
cg05584597	PLCH1	brown	-0.1942852	-0.5252811	0.23901125	0.17208555	0.5494244	-0.0448999
cg20233436	KCNN3;KCNN3	brown	-0.3194749	-0.7504686	0.04545354	0.17442821	0.78572816	-0.0184402
cg26761618	P2RY2;P2RY2;P2RY2	brown	-0.0169579	-0.5398628	0.21902226	0.39396185	0.92924283	0.14654425
cg05371135	FGR;FGR;FGR	brown	0.02072267	-0.4555005	0.25507217	0.31206531	0.8229183	0.15202614
cg05187055	TCF12;TCF12;TCF12;TCF12	brown	-0.050227	-0.5218907	0.36256378	0.22042661	0.75517441	0.54477594
cg27305772	MUS81;MUS81;CFL1;MUS	brown	-0.0437268	-0.5522744	0.28124419	0.29824163	0.8572388	0.17748371
cg18548246	GCFC1-AS1;GCFC1-	brown	-0.1740267	-0.6241163	0.09633377	0.2048281	0.70429036	0.03877958
cg20091959	L3MBTL1;L3MBTL1;L3MB	brown	-0.1469332	-0.2765174	0.46065108	0.19370315	0.33759582	0.22099736
cg15781073	TRAJ26;TRAJ25	brown	0.20179419	0.72226906	-0.1336265	-0.2002153	-0.9195965	-0.1101842
cg10133284	DOK4;DOK4;DOK4;DOK4;	brown	0.16623925	-0.2298057	-0.0082726	0.35965308	0.52419219	0.31448134
cg06277957	RP11-	brown	0.10078763	-0.3141283	0.22795206	0.64078633	0.76011306	0.24812808
cg03711457	USP28;USP28;USP28;USP2	brown	-0.2319684	-0.5860771	0.24497945	0.16034333	0.73379244	0.10821698
cg26950668	SPNS3;SPNS3;RP13-	brown	-0.3682414	-0.5005788	0.11827386	0.04185515	0.55063267	-0.1398076
cg03802998	TP53I11;TP53I11;TP53I11;T	brown	-0.3876954	-0.3578214	0.1136628	0.05133753	0.37123807	-0.1093266
cg12160258	TMEM26;TMEM26;TMEM2	brown	-0.2188649	-0.6795949	0.15220365	0.13796863	0.78597217	0.03592566
cg19146124	KLF15	brown	-0.3927084	-0.7310623	0.13212209	0.07517919	0.67546783	0.00115571
cg19461093	DIP2A-IT1	brown	0.26212309	0.70383358	-0.1610953	-0.105827	-0.7564761	0.0088051
cg05427901	TRIM11;TRIM11;TRIM11;R	brown	0.11435702	0.0082767	0.42378793	0.29059085	0.26338351	0.29867529
cg24382698	GAS7;GAS7	brown	-0.0603621	-0.6126862	0.15333465	0.27293437	0.90153478	0.23988567
cg11125901	SPICE1;SPICE1	brown	-0.2324398	-0.5067809	-0.0473192	0.18375815	0.53366462	0.01455684
cg10582648	IFFO2;IFFO2	brown	-0.3648243	-0.5430484	0.10301311	0.05574816	0.50169204	-0.0720663
cg07106447	IPO13;IPO13	brown	-0.0027816	-0.4387054	0.36593103	0.26521609	0.70173775	0.23353633
cg20804821	GPR62	brown	-0.138475	-0.5881348	0.11489062	0.30276248	0.83448794	0.15723694

cg06927323	TBX21	brown	-0.2956042	-0.6507663	0.16156841	0.10677901	0.72562388	0.06862729
cg11256132	C1orf100;C1orf100	brown	0.4331292	0.56779237	-0.1739215	-0.1059622	-0.5701323	0.10587598
cg17638564	LCN12;LCN12;LCN12;LCN	brown	0.00976043	-0.0387564	0.36026283	0.38184808	0.32483685	0.20536109
cg05902884	MAP6D1;MAP6D1;MAP6D	brown	-0.1752049	-0.5046823	0.099158	0.18888161	0.62519056	0.05263433
cg15589354	URB2;TAF5L;TAF5L;TAF5	brown	-0.1954478	-0.4295989	0.37455283	0.30368276	0.70008557	0.16046482
cg16633058	ZNF608;ZNF608;ZNF608;Z	brown	-0.3215936	-0.3404963	0.14353257	0.01222606	0.40311703	0.01740664
cg26184741	SRGAP3;SRGAP3;SRGAP3	brown	-0.1911948	-0.5417083	0.21291269	0.24295096	0.71820366	0.04792247
cg11990892	HDLBP;HDLBP;HDLBP;H	brown	-0.0804591	-0.6043417	0.15966205	0.27301098	0.91271438	0.19710325
cg12570309	FARSA;FARSA	brown	-0.2287722	-0.5129453	0.18429234	0.26351509	0.67284731	0.0323741
cg23977964	TCF7;TCF7;TCF7;TCF7;TCF	brown	-0.2312702	-0.4871257	0.34286599	0.24337482	0.46131913	0.17796991
cg21256649	PRKAR1A;PRKAR1A;PRK	brown	-0.2889045	-0.6865734	0.15299278	0.17759696	0.72503266	-0.0326837
cg10384244	ANKRD33;ANKRD33;ANK	brown	0.06434372	-0.2989818	0.20838875	0.28281242	0.48068443	0.25482125
cg26872330	TMEM9	brown	0.10709597	0.45300636	-0.0093957	-0.1061681	-0.4561782	-0.0805559
cg07046101	RP4-781B1.2;KCNK15	brown	0.13348422	0.42294054	0.10042687	-0.0766829	-0.4551574	0.02759393
cg00369640	DNAH7;DNAH7	brown	-0.3705571	-0.3411524	0.15167562	0.07621097	0.34089918	-0.1345917
cg06320150	RP5-983L19.2	brown	-0.0148751	-0.344493	0.33029607	0.23825638	0.6199408	0.22663344
cg15100982	TMEM26;TMEM26;TMEM2	brown	-0.3676238	-0.727376	0.11046487	0.11444465	0.70714518	0.02583796
cg26056946	RALGAPB;RALGAPB;RAL	brown	-0.0916677	-0.4019291	0.04877158	0.34040061	0.75130516	0.12430027
cg21089270	INPP5B;INPP5B;INPP5B;IN	brown	-0.0650067	-0.2899547	0.15945891	0.20630691	0.62999788	0.05263949
cg06451566	U6;RP1-60O19.1;RP1-	brown	0.44721076	0.73388008	-0.123637	-0.1362584	-0.6906521	0.03816913
cg04232816	AC012307.3;AC012307.3	brown	0.0870752	-0.4087304	0.15648109	0.46614848	0.85512192	0.19726731
cg17608497	MTUS2;MTUS2	brown	-0.1124713	-0.4637087	0.19704605	0.24383054	0.6875446	0.01610628
cg03852570	PYROXD2;PYROXD2;PYR	brown	-0.1890891	-0.644896	0.16469444	0.18107725	0.86083515	0.10746875
cg22855405	TINAGL1;TINAGL1;RP11-	brown	-0.2435413	-0.4802367	0.2278962	0.17161018	0.48219981	0.13903162
cg00481600	CILP	brown	-0.3858371	-0.7247274	0.09412855	0.11846207	0.83031324	-0.0053434
cg15528109	SAT2;SAT2;SAT2;SAT2;SA	brown	-0.2938169	-0.7871273	0.0972539	0.17305108	0.87939015	0.06692869
cg08209473	SIAH1;SIAH1;SIAH1;SIAH	brown	-0.3669834	-0.509869	0.1983642	0.07017677	0.52456721	-0.0804207
cg08415592	APOL1;APOL1;APOL1;AP	brown	-0.0543207	-0.257437	0.30388107	0.16422549	0.49867897	0.42151692
cg10439209	EIF3L;EIF3L;EIF3L;EIF3L;E	brown	-0.2132123	-0.3941492	0.0416335	0.29388125	0.59296194	-0.0403934
cg07749047	AC005075.5;AC003991.3	brown	0.37991437	0.8130752	-0.2091471	-0.1028415	-0.7960257	-0.0151681
cg04018315	NOS3;NOS3;NOS3;NOS3;N	brown	0.02061986	-0.4725626	0.28711567	0.24531868	0.67698633	0.29822537
cg14592794	VTN;CTB-96E2.3;SARM1	brown	-0.2925437	-0.5654539	0.24856623	0.14971149	0.55170785	0.05788075
cg26345203	TRAJ7	brown	0.32721624	0.79281302	0.07728354	-0.1001157	-0.8698682	0.0448908
cg25768456	RPA1	brown	0.24364382	0.53012823	-0.0088503	-0.110795	-0.8080712	-0.00057
cg22783327	FXYD7;FXYD7;LGI4;LGI4	brown	-0.2551727	-0.7350914	0.1236302	0.22185488	0.84710222	0.03184907

cg09440270	RP11-	brown	-0.216043	-0.5543797	0.32373861	0.12257243	0.58341683	-0.0182015
cg16068823	SLC9A3R2	brown	0.03422706	-0.263566	0.16940175	0.23733777	0.57847358	0.16939677
cg26451234	MPV17;MPV17;MPV17;MP	brown	-0.0597061	-0.5395103	0.08036912	0.43083504	0.9108455	0.10477366
cg10208550	C9orf50;METTL11A	brown	0.01841675	-0.287572	0.14566311	0.29878649	0.48789125	0.28386433
cg13234901	OSTBETA	brown	0.0681738	-0.3698078	0.21134299	0.37769912	0.8210109	0.216442
cg10343787	PLLP;PLLP	brown	-0.4133324	-0.724794	0.17826569	0.11790473	0.68451912	-0.0296383
cg09319070	NEIL1;NEIL1;NEIL1;NEIL1;	brown	-0.2991857	-0.5982215	0.01561128	0.07765834	0.62963208	0.03672167
cg05319638	CYB5D2;CYB5D2;CYB5D2	brown	-0.0181608	-0.4444891	0.296872	0.41122876	0.78201863	0.22040404
cg06055763	HGF;HGF;HGF;HGF;HGF;	brown	-0.1195968	-0.3960747	0.10177832	0.11488674	0.615998	0.17319506
cg01838523	WISP2;WISP2;WISP2;WISP	brown	-0.0964299	-0.3625575	0.28629611	0.35694115	0.68156509	0.19320582
cg03656583	SGK1;SGK1;SGK1;SGK1;S	brown	-0.0007484	-0.4049754	0.16469206	0.43346249	0.83898847	0.14279529
cg09305680	EIF3H;EIF3H;UTP23;UTP23	brown	-0.0785394	-0.3719857	0.15217026	0.4352969	0.76225767	0.15485166
cg09404380	EVL;EVL;EVL;EVL;EVL;EV	brown	0.30040157	0.77666262	0.00265778	-0.0840296	-0.8569284	0.00705937
cg10821341	SIL1	brown	0.05607229	0.57723892	0.08868405	-0.1312352	-0.7306177	-0.1351175
cg24065807	TRAJ31	brown	0.23599139	0.73182189	-0.097187	-0.2106891	-0.9299331	-0.0546418
cg11683511	GPD1;GPD1;GPD1;GPD1	brown	-0.0743029	-0.3307942	0.4166321	0.3271637	0.52614916	0.25012923
cg13709351	CCDC82;CCDC82;CCDC82;	brown	-0.2551009	-0.5827014	0.27918671	0.30229176	0.72801363	0.20669983
cg00112465	CCDC48;RP11-	brown	-0.1136421	-0.4840387	0.15584906	0.19723531	0.57238434	0.4536734
cg26735135	AC002456.2;AC002456.2;A	brown	-0.0855498	-0.5906309	0.24457322	0.28970733	0.88507046	0.13374459
cg26531901	GAS2L2	brown	-0.2933	-0.5504299	-0.0565151	0.11670411	0.6367305	-0.0529324
cg04582732	RTN3;RTN3;RTN3;RTN3;R	brown	-0.1793511	-0.248051	0.14015315	0.33228912	0.58090964	0.00452523
cg05085782	UNC80;UNC80	brown	-0.2362261	-0.3727363	0.20982081	0.09613464	0.35830253	0.00213612
cg06247806	RP11-525A16.1;DUSP5	brown	-0.2982845	-0.4144116	0.15967421	-0.0058406	0.4312354	-0.0886264
cg18168663	REC8;REC8;REC8	brown	0.02621244	-0.4196439	0.08901517	0.40417862	0.87147831	0.17490266
cg08462274	P4HB;P4HB;P4HB;P4HB;P4	brown	-0.1322432	-0.5798734	0.21239903	0.39583151	0.87095233	0.11352086
cg04714233	RP11-996F15.2	brown	-0.1117402	-0.206795	0.24078942	0.2790898	0.31557228	0.10510078
cg18031455	LST1;LST1;LST1;LST1;LST	brown	-0.2246663	-0.726454	0.10391667	0.17671573	0.89147291	0.05437322
cg11898695	PTCRA;PTCRA	brown	0.22816201	0.51578107	-0.1185132	-0.2360133	-0.7427009	0.0490561
cg26396492	RIN2;RIN2;RIN2;RIN2	brown	-0.3051371	-0.4836728	0.15122388	0.14483551	0.59430901	-0.0392956
cg04027111	LOXL4	brown	-0.2547188	-0.3825502	0.16324483	0.28680471	0.58517426	0.02441847
cg02379533	C5orf4;C5orf4;C5orf4;C5orf4	brown	0.03977129	-0.4236129	0.24341537	0.42460612	0.843086	0.22190699
cg27663476	FAH;FAH;FAH;FAH;FAH;	brown	0.00100684	-0.0968934	0.02845861	0.40390375	0.33774072	0.21200308
cg26952381	PILRB;PILRB;PILRB;PILRB	brown	0.14645191	-0.2816497	0.22443887	0.71837212	0.72145253	0.21939558
cg14374954	CA12	brown	0.04404853	-0.1168687	0.06610553	0.35386689	0.43765279	0.09343964
cg15515788	SPNS3;SPNS3;RP13-	brown	0.02935399	-0.3711519	0.3390347	0.23547372	0.6721679	0.26166618

cg11318268	ARHGAP27;ARHGAP27;A	brown	0.01388944	-0.4771781	0.33624987	0.25405981	0.71818706	0.54108296
cg01617750	RP11-384L8.1;CMTM8	brown	-0.2684826	-0.731057	0.1353671	0.13300562	0.80334087	0.09490671
cg20540862	RP4-781K5.5;RP4-	brown	0.49768547	0.22679684	-0.2568148	0.08466746	0.17113245	-0.0302196
cg22850451	MAP1S	brown	0.04565917	-0.4562596	-0.0790373	0.36244632	0.84046131	0.15392497
cg08095443	DGUOK-AS1;DGUOK-	brown	-0.1257391	-0.5764807	0.16270758	0.24718677	0.78516213	0.20180143
cg08789064	ERCC6;RP11-123B3.6;RP11-	brown	0.04262793	-0.2980691	0.27791053	0.41515583	0.58276559	0.15493759
cg00322767	CLEC2D;CLEC2D;CLEC2D;	brown	0.23847192	0.69882541	0.08232521	-0.1729582	-0.8798839	-0.0609728
cg22953017	AC080164.1	brown	-0.225569	-0.746178	0.21391828	0.22468853	0.83829966	0.11066552
cg20331595	NRSN2;RP5-	brown	-0.378835	-0.6138996	-0.0735328	0.1023938	0.63162561	-0.1427955
cg10741776	RP11-661G16.1	brown	-0.2076555	-0.4681736	-0.0275959	0.24836525	0.66447014	0.11471764
cg06940127	SERPINF1;SERPINF1;SERP	brown	-0.3070996	-0.465617	0.35099324	0.2177792	0.62038546	0.07413922
cg15810171	C1orf122;YRDC	brown	-0.1493222	-0.4491322	0.30108539	0.31988629	0.67374911	0.14526713
cg19327252	GNAI2;GNAI2;GNAI2	brown	-0.2457485	-0.6566264	0.29157933	0.24871492	0.82868074	0.13973633
cg08067438	NDFIP2;NDFIP2;NDFIP2;N	brown	-0.3817906	-0.5261479	0.11296804	0.06547113	0.49416641	-0.049718
cg17463145	MAGI2;MAGI2;MAGI2;MA	brown	-0.1822578	-0.6223989	0.13656053	0.18236815	0.81471367	0.1029486
cg17553260	RP11-783K16.14;RP11-	brown	-0.4479807	-0.5427351	0.12301254	0.11144744	0.50617372	-0.1482933
cg22580353	PVR;PVR;PVR;PVR	brown	-0.3124591	-0.5449412	0.27062392	0.20246249	0.65414065	0.04638781
cg09940349	TGFA-IT1;TGFA	brown	0.12956719	-0.2836229	0.0226671	0.52260518	0.71829521	0.21453595
cg02504384	TSC2;TSC2;TSC2;TSC2	brown	0.05865463	-0.2588983	0.50769968	0.22641636	0.44973826	0.3950492
cg09452728	RP11-713P17.3;RP11-	brown	-0.0406308	-0.2537408	0.19138883	0.44764664	0.51023682	0.2211299
cg13122917	PDE4C	brown	0.04629291	-0.3742637	0.43430653	0.35150331	0.72654096	0.35120297
cg11661054	ZNF503;ZNF503-	brown	-0.3304421	-0.7913007	0.10213275	0.09154065	0.78175175	0.02360488
cg20544516	SREBF1;SREBF1;SREBF1;	brown	0.08105605	-0.3396897	0.24474993	0.38406933	0.69249654	0.44799352
cg01461862	LRRC37A6P;RP11-748L13.1	brown	-0.0551782	-0.6193663	0.31283215	0.25489142	0.69750207	0.39838275
cg26373518	INPP5J;INPP5J;INPP5J;INP	brown	-0.1549972	-0.3050305	0.22517646	0.21333849	0.57002985	0.02510316
cg10837806	PTGDR;PTGDR	brown	-0.4316352	-0.6542069	0.04404011	0.10627656	0.66692778	-0.1501412
cg10989288	TMEM91;TMEM91	brown	-0.1697986	-0.4606904	0.20334522	0.18050815	0.69143005	0.03081993
cg16142668	RP1-130H16.18;RP1-	brown	-0.03603	-0.4545869	0.16038113	0.33591188	0.67034454	0.22679952
cg18080903	RNF130;RNF130;RNF130;R	brown	-0.4834032	-0.6778646	-0.0478055	-0.0771755	0.61327749	-0.1938297
cg25003924	ARPP19;ARPP19;ARPP19;	brown	0.03496883	-0.46511	0.19831207	0.50074872	0.86509505	0.21994792
cg10683668	TNFAIP3;TNFAIP3;TNFAI	brown	0.30272065	0.79521307	0.03219938	-0.1112974	-0.873548	0.02798526
cg06170176	COL18A1;COL18A1	brown	-0.3000834	-0.3997561	0.42217925	0.13049738	0.39883485	0.05275646
cg20119383	C20orf111;RP5-	brown	-0.3208437	-0.6289873	0.05142034	0.1086667	0.62258783	-0.052966
cg16706840	MUC12;MUC12	brown	-0.1079926	-0.5810955	0.07723543	0.185348	0.81784702	0.12695216
cg17119394	GFOD1	brown	2.28E-05	-0.5531838	0.1096132	0.38449188	0.92918325	0.18652382

cg04206417	EMID1;EMID1;EMID1;EMI	brown	-0.2362807	-0.6535869	0.15465183	0.23387151	0.84396319	0.05400627
cg10561067	MGAT4B;MGAT4B;MGAT	brown	-0.3813391	-0.4362647	0.1110456	-0.0600807	0.44038423	-0.0562725
cg18956603	JUP;JUP;JUP;JUP;JUP;JUP;	brown	-0.0148612	-0.2257032	0.30345448	0.13221682	0.41617486	0.25249852
cg08899667	VARS;VARS;VARS	brown	-0.0285523	-0.5455142	0.22487602	0.28220311	0.81311918	0.1880495
cg17478313	SLC7A8;SLC7A8;SLC7A8;S	brown	-0.2337106	-0.6307389	0.14030897	0.14099879	0.72876666	0.0336533
cg04420141	RGS9;RGS9	brown	-0.34482	-0.3636797	0.10872624	-0.042398	0.34280502	0.11304057
cg03079104	CHRM1;CHRM1	brown	-0.0779989	-0.2048893	0.27750989	0.3317844	0.42665358	0.09755573
cg18054492	METTL20	brown	0.02285176	-0.157582	-0.0240425	0.12812187	0.30611223	0.21040933
cg14567671	ARHGEF16;ARHGEF16;AR	brown	-0.1116292	-0.3419093	0.26186927	0.26138199	0.57716291	0.20375824
cg20908828	EPHA2;EPHA2;RP11-	brown	-0.2556632	-0.7634545	0.09739871	0.14430358	0.89942527	0.04604805
cg07665060	C19orf33;YIF1B;YIF1B	brown	-0.1922045	-0.4128729	0.1913637	0.06150508	0.4350665	0.04040229
cg25633717	LARP4B	brown	-0.1594325	-0.655295	0.10016859	0.24431307	0.94969887	0.1137669
cg07368443	PLK3;PLK3;PLK3	brown	-0.1907662	-0.1939622	0.25446716	0.32307513	0.43191228	-0.0078426
cg11685223	GZF1;GZF1;GZF1;GZF1;GZ	brown	0.04552483	-0.31034	0.3131876	0.53470818	0.60208207	0.34363841
cg19374004	CLCF1;AP003419.11;CLCF1	brown	-0.1781689	-0.3207665	0.35823557	0.20465941	0.42307113	0.15894154
cg14182621	RYR1	brown	-0.4741118	-0.5686252	0.08332619	0.06145838	0.52044637	-0.206637
cg00391637	ZNF564;ZNF564;ZNF564;C	brown	-0.2767694	-0.5495989	0.13373146	0.19570807	0.59285552	-0.0554819
cg16574310	MSLN;MSLN;MSLN;MSLN	brown	-0.3157928	-0.3055377	0.24998743	0.12143963	0.38815532	0.20345475
cg26427777	ZHX2;ZHX2	brown	0.23619944	0.4714967	-0.0377437	-0.1585537	-0.7340558	0.15980624
cg20817941	CTSK;CTSK;CTSK;CTSK	brown	0.04644749	-0.4038123	0.09570131	0.54589307	0.74869468	0.20615917
cg09427574	CRADD;RP11-	brown	-0.2467686	-0.695738	0.09357029	0.20781355	0.93476364	-0.0056129
cg01046511	KDM6B;KDM6B;KDM6B	brown	0.00307013	-0.5190912	0.20618426	0.40316636	0.87404452	0.24658099
cg14398464	NOL12;NOL12;NOL12;NOL	brown	-0.2120989	-0.5122179	0.13565495	0.30339599	0.77412372	0.1252831
cg01414857	GPR146;GPR146;C7orf50;G	brown	-0.0258441	-0.3554914	0.37357299	0.15430006	0.48759545	0.29439354
cg12076102	ICAM2;ICAM2	brown	0.02445054	-0.4414001	0.15250255	0.39638798	0.84645987	0.14670258
cg16936060	HOPX;HOPX;HOPX;HOPX	brown	-0.1034498	-0.3643151	0.05879976	0.22306178	0.64704449	0.08617501
cg16310513	ATP8B1	brown	-0.2561959	-0.5316754	0.25919244	0.15784308	0.51841004	0.01601444
cg25661026	CEBPD;CEBPD	brown	-0.2756413	-0.5358807	-0.0317934	0.14541186	0.62472477	-0.0535115
cg14451446	KIAA0182;KIAA0182;KIA	brown	0.03303735	-0.3865727	0.13720377	0.45431291	0.83008251	0.21755641
cg11102484	RP11-150O12.2;RP11-	brown	-0.2018254	-0.362798	0.20295928	0.3789587	0.61929097	0.00386695
cg18248375	EFNB3	brown	-0.0842514	-0.52484	0.19469754	0.26381673	0.76587807	0.26043333
cg17737967	MAN2B1;MAN2B1;MAN2	brown	-0.1543524	-0.3835703	0.22802918	0.08185331	0.43286535	0.09841256
cg17045389	KATNAL1;KATNAL1;KAT	brown	-0.4092175	-0.7794986	0.140493	0.08624594	0.75125743	-0.0228555
cg01996032	AC135050.1	brown	0.14718376	-0.4001637	0.31289935	0.32751023	0.77641401	0.36944144
cg01861509	SPOCK2;SPOCK2;SPOCK2	brown	0.25775576	0.64503476	-0.0115878	-0.1259391	-0.8634185	0.04737653

cg14550985	RP11-	brown	-0.3210703	-0.4304732	0.26891577	0.15103436	0.56974281	0.0463769
cg07056567	GNB2;GNB2;GNB2;GNB2;	brown	-0.3824586	-0.3168988	0.07191785	-0.046495	0.30205093	-0.1281219
cg03868882	CTD-2006C1.2;CTD-	brown	-0.2843209	-0.5982611	0.15665304	-0.0020764	0.61756274	0.02333338
cg18891141	ABCC3;ABCC3;ABCC3;AB	brown	-0.369452	-0.6886582	0.17617676	0.09271968	0.77872938	-0.0618449
cg14630313	PTK7;PTK7;PTK7;PTK7;PT	brown	-0.0016841	-0.3555391	0.42847939	0.3911136	0.63986079	0.26123178
cg02975107	9-Sep	brown	-0.3340406	-0.5336102	0.11016355	0.12996668	0.52331669	-0.0897993
cg19483242	RP11-	brown	-0.0293443	-0.2755432	0.29073252	0.39674035	0.6516109	0.21456576
cg15183092	NOS3;NOS3;NOS3;NOS3;N	brown	-0.1840379	-0.5052623	0.337804	0.0702825	0.48487576	0.09757323
cg02930951	CEBPB	brown	0.10964355	-0.2893064	0.26328907	0.68859314	0.61168714	0.39197666
cg02851944	RBL2;RBL2;RBL2;RBL2	brown	-0.3292023	-0.5359304	0.15714694	0.22352901	0.6871203	-0.0372728
cg01734062	TMEM92;TMEM92;TMEM9	brown	-0.2730433	-0.6498996	0.06030225	0.12708066	0.70184873	0.10012971
cg21646437	TRAJ39;TRAJ38	brown	0.35678628	0.71564341	0.18499325	-0.1202457	-0.8077826	-0.0042901
cg20457147	PRKCH;RP11-	brown	-0.1477705	-0.4625645	0.11999257	0.30271265	0.66460638	0.20151019
cg05924780	LPAR5;LPAR5;LPAR5;LPA	brown	-0.4601486	-0.581387	0.10772596	-0.0139449	0.57844242	-0.088402
cg14061051	FXR2;MPDU1	brown	0.20518509	-0.220886	0.0764992	0.52920657	0.62047015	0.23687109
cg08375139	CERS2;SETDB1;SETDB1;S	brown	-0.0748839	-0.4328359	0.19164187	0.26960473	0.76218516	0.15165356
cg18845312	FGFBP2;PROM1;FGFBP2	brown	-0.083368	-0.375885	0.12631961	0.29531351	0.69590158	0.14434579
cg23205858	PI4KA;PI4KA;PI4KA;PI4K	brown	-0.050651	-0.5284936	0.05463126	0.23116278	0.7090978	0.13819406
cg03705396	MAP6D1;MAP6D1;MAP6D	brown	-0.349386	-0.6863939	0.04826691	0.08419934	0.65769689	0.039092
cg11709544	STMN1;STMN1;STMN1;ST	brown	-0.1904517	-0.4247602	0.22582859	0.19231313	0.50090812	0.09418808
cg23388881	KCNIP1;KCNIP1	brown	-0.1327355	-0.5039533	0.1361401	0.22759178	0.78384845	0.11184922
cg13423389	NRM;NRM;NRM;NRM;NR	brown	0.23099296	0.47092512	-0.0024947	-0.1343186	-0.5580641	-0.0811318
cg15122985	TCEA2;RP13-	brown	-0.2539625	-0.3078561	0.21990683	0.00129666	0.27386097	0.05918893
cg13620770	GPR137;GPR137;GPR137;G	brown	-0.4564324	-0.5974359	0.13468808	0.04863881	0.54733241	-0.0299614
cg11679268	LY6G5C;LY6G5C;LY6G5C;	brown	-0.0422354	-0.3921509	0.05744692	0.32297021	0.62574893	0.2065453
cg00288844	RP11-490O6.2;TXNDC11	brown	-0.0301617	-0.4698373	0.10798528	0.29340666	0.84629559	0.16665892
cg18856359	ARSB;ARSB;ARSB	brown	-0.1865804	-0.217682	0.17480655	0.08872079	0.34310242	0.06322183
cg21650861	TPRA1;TPRA1;TPRA1;TPR	brown	-0.1285308	-0.2434915	0.45543903	0.14735363	0.38972872	0.33743052
cg01657926	CCDC81;CCDC81;CCDC81;	brown	-0.3099608	-0.6144982	0.26213886	0.0965152	0.54514226	0.03670166
cg09450085	AC009404.2;AC009404.2;A	brown	-0.2453132	-0.4231731	0.21471432	0.14688041	0.42481907	-0.0438445
cg04731714	ADRBK1;ADRBK1;ADRB	brown	-0.0142154	-0.4485132	0.37845764	0.31268218	0.77117263	0.12133996
cg13558810	RP4-728D4.2;RP4-	brown	-0.1548736	-0.3580298	0.22021647	-0.0272184	0.3034454	0.04315845
cg15702185	RP4-	brown	-0.1153793	-0.1145808	0.2595214	0.10395691	0.35409262	0.2951297
cg15396220	RP11-497D6.3;RP11-	brown	-0.2671431	-0.1821921	0.20309316	0.11832118	0.31803401	-0.0125427
cg21406003	NEK6;NEK6;NEK6;NEK6;N	brown	-0.1613104	-0.7035562	0.24318935	0.21457413	0.85624093	0.10054779

cg12257439	FER1L5;FER1L5;FER1L5;FE	brown	-0.1700243	-0.6567547	0.21656763	0.25949104	0.89017684	0.10731009
cg24331384	TCHP;TCHP;TCHP;TCHP;T	brown	-0.3083524	-0.6024853	0.28146848	0.20627919	0.67547582	0.09310297
cg10143146	RXRB;COL11A2;COL11A2;	brown	-0.2004583	-0.5458249	0.03877696	0.193336	0.70206234	0.08072039
cg14023398	RBM47;RBM47;RBM47;RB	brown	-0.0725651	-0.4990086	0.00907184	0.13469632	0.68388251	0.14797822
cg07351267	LRP3;LRP3	brown	-0.0989513	-0.5225208	0.30137732	0.27978664	0.83133489	0.14692556
cg27288233	EVI5L;EVI5L	brown	0.21695029	-0.200999	0.37501071	0.62357577	0.66631788	0.36481951
cg05263215	KCNAB2;KCNAB2;KCNA	brown	-0.0439815	-0.5422979	0.23206809	0.46916774	0.86689297	0.20024503
cg00705295	ZNF554	brown	-0.2890829	-0.6088067	-0.0135103	0.1910951	0.75668232	0.02004331
cg09968549	MEF2D;MEF2D;MEF2D;M	brown	0.15926979	-0.2638907	0.25110944	0.6070622	0.67232972	0.32812805
cg24967701	GRB7;GRB7;GRB7;GRB7;G	brown	-0.2109861	-0.6507365	0.10768221	0.04596835	0.7554794	-0.0056404
cg16493584	SAMD3;SAMD3;SAMD3;S	brown	0.22540586	0.6402169	0.18309148	-0.111979	-0.8413225	0.07819624
cg18356935	DGCR2;DGCR2	brown	0.04829887	-0.3790525	0.17370741	0.35670624	0.82500138	0.1923082
cg17710952	ATP2A1;ATP2A1	brown	0.03836139	-0.3808642	0.10103101	0.32180459	0.632147	0.22506524
cg10431989	HHLA2;HHLA2;HHLA2;H	brown	0.31264824	0.77292022	0.12702407	-0.0832485	-0.8365107	0.05766414
cg13468215	RN5S257;LZTS1;LZTS1-	brown	-0.0428476	-0.3603914	0.14636387	0.18866342	0.61355799	0.13298219
cg04105091	PRRT1;PRRT1;PRRT1;PPT2	brown	-0.3808269	-0.5582869	0.01101437	0.05851033	0.48866575	-0.0571124
cg08249435	RPL7;RDH10;RDH10;RPL7;	brown	-0.3564926	-0.8403515	0.12873797	0.02454911	0.77232765	0.05100168
cg03471436	SOX13;SOX13;SOX13;SOX	brown	0.2812039	0.67278253	0.02253404	-0.0372312	-0.7103824	0.06368727
cg16049494	HCK;HCK;HCK;HCK;HCK	brown	-0.2801994	-0.658633	0.15501041	0.08204463	0.83304336	-0.0392717
cg20870362	CCIN	brown	0.01341552	-0.3006189	0.26792786	0.4286007	0.72781395	0.20611764
cg10118912	TRANK1;TRANK1;TRANK	brown	-0.3195358	-0.4389092	0.18497626	0.35952381	0.64265774	-0.1080236
cg20303028	DCTN1;DCTN1;DCTN1;DC	brown	-0.3436505	-0.421859	0.19692113	0.00802802	0.39483464	0.01148045
cg11778548	MTX1;MTX1;MTX1;MTX1;	brown	-0.1812958	-0.6834457	0.15026003	0.23317183	0.82662546	0.10673783
cg25202280	C17orf70;C17orf70;C17orf70	brown	-0.33199	-0.7998175	0.04509228	0.09894245	0.77600514	-0.004016
cg07536453	NUMA1;NUMA1;NUMA1;	brown	-0.0964056	-0.6250386	0.16193389	0.34535658	0.91118845	0.17935118
cg01973394	MEX3B;MEX3B;AC026956.	brown	0.04611406	-0.3871725	0.13830765	0.2254769	0.62641354	0.27471283
cg00994601	C6orf47;C6orf47	brown	-0.3504428	-0.4410966	0.11462916	-0.0226075	0.4904914	0.06981655
cg25168925	RP13-25N22.1;DUSP8	brown	-0.1385448	-0.3989382	0.32678403	0.22691776	0.59252047	0.24269209
cg03835824	SEMA4B;SEMA4B;SEMA4	brown	-0.1256603	-0.6061656	0.30014639	0.2010124	0.79615911	0.23962711
cg18986090	LRFN4;LRFN4;LRFN4;LRF	brown	-0.2703434	-0.460148	0.21202865	0.11507428	0.61563169	0.06102043
cg16235995	AKAP6;AKAP6;AKAP6;A	brown	0.29347698	0.70248408	-0.0364168	-0.1034669	-0.8253568	0.01623587
cg20998511	IL19	brown	0.23971952	0.73600424	-0.0428785	-0.1310185	-0.8182402	-0.045245
cg17333042	KAZALD1;KAZALD1;KAZ	brown	-0.3071107	-0.3242312	0.28766743	0.20100993	0.3349298	-0.0101697
cg15297650	TMEM163	brown	-0.0737939	-0.4660469	0.25504928	0.2956778	0.76153329	0.10831233
cg13639341	C20orf96;C20orf96;C20orf96	brown	-0.304531	-0.6786661	0.1035428	0.11892023	0.75968316	0.0214222

cg19761273	CSNK1D;CSNK1D;CSNK1	brown	-0.3554356	-0.3113625	0.22833701	0.22572658	0.40330397	-0.0829909
cg14327711	GAPT;GAPT;GAPT;GAPT;	brown	-0.0445094	-0.4095965	0.13931616	0.26750376	0.72068405	0.139566
cg06394229	LGALS4	brown	-0.0557031	-0.5184601	0.14898573	0.44123989	0.90213153	0.19838151
cg15480358	SMPD4P1	brown	0.03943386	-0.3325674	0.1634566	0.38805449	0.78968078	0.14644378
cg20409587	SMAP2;SMAP2	brown	-0.2224202	-0.3480077	0.21889029	0.25677595	0.50456682	-0.0286385
cg11321922	NR1I3;NR1I3;NR1I3;NR1I3;	brown	-0.0973021	-0.414698	0.10721037	0.3347726	0.8266267	0.08588074
cg09107315	GIPC2;GIPC2	brown	-0.4137797	-0.5725701	0.00909362	0.02354027	0.48050356	-0.1588854
cg16504781	KLF5;KLF5;KLF5;KLF5	brown	-0.3680049	-0.5611374	0.24143753	0.03602077	0.52656763	-0.0550749
cg04730047	TBC1D23;TBC1D23;TBC1D	brown	-0.19705	-0.6704967	0.05181275	0.21515062	0.89878069	0.0479469
cg13403069	LINC00475;LINC00475;LIN	brown	-0.00867	-0.0819339	0.26898917	0.2188817	0.28306158	0.4078397
cg17761996	EIF5;EIF5;EIF5;EIF5;EIF5	brown	-0.1591939	-0.447509	0.00868069	0.2521837	0.80964987	0.03644183
cg15746620	EMR3;EMR3	brown	-0.2151387	-0.535258	0.08177312	0.18904454	0.7305996	0.05671646
cg09906145	CTD-2012K14.1;CTD-	brown	-0.1788143	-0.4746479	0.32001855	0.2981612	0.64198245	0.10139129
cg09429111	PTPDC1	brown	-0.3342641	-0.4316484	0.21667642	0.06713017	0.41338199	0.12598548
cg04168609	DLEC1;AP006309.4	brown	-0.0678808	-0.5833667	0.24666422	0.30456407	0.90477606	0.18215662
cg11317199	TRIM14;TRIM14	brown	0.07802848	-0.3229174	0.34769417	0.18071144	0.5356283	0.21544073
cg25697727	PPAP2C;PPAP2C	brown	0.13206639	-0.2338607	0.2884103	0.24244081	0.37025536	0.25775279
cg10952190	PTPN14;PTPN14;PTPN14	brown	-0.3054327	-0.741863	0.11689028	0.10522051	0.8152172	0.00288662
cg27068669	PTPRD;PTPRD;PTPRD;PTP	brown	-0.161865	-0.4512623	0.00242099	0.2336079	0.59636308	0.1023533
cg13062436	PDE9A;PDE9A;PDE9A;PDE	brown	0.10060898	-0.1549067	-0.0507771	0.32747171	0.47050784	0.19396185
cg21178711	PRRC2B;PRRC2B	brown	0.09595994	-0.0536332	0.18934926	0.2969369	0.29207847	0.36921563
cg23857904	KLHL6;KLHL6	brown	-0.269109	-0.3903239	0.25916014	0.29656554	0.52066847	0.01305278
cg00226815	NBEAL2;NBEAL2	brown	-0.1595544	-0.4894071	0.22506973	0.27792265	0.65496177	0.04492929
cg08536191	RANGAP1;ZC3H7B;ZC3H7	brown	-0.3744228	-0.429554	0.20571813	0.11309462	0.50346401	-0.0545352
cg04595171	DCTN2;DCTN2;DCTN2;DC	brown	0.01143175	-0.4107717	0.0237834	0.42052479	0.85703836	0.1192372
cg02935298	ATP5L2;CYB5R3;CYB5R3;	brown	-0.1098452	-0.5196797	0.07637255	0.25424169	0.79948714	0.0916649
cg07049940	MPP6;MPP6	brown	-0.0662057	-0.2263906	0.14729899	0.24221131	0.4799628	0.11083752
cg05377120	KLRD1;KLRD1;KLRD1	brown	0.25517474	0.78377509	-0.0541139	-0.1348454	-0.9135995	-0.029574
cg17885791	ZC3H12D;ZC3H12D	brown	-0.0705649	-0.4954518	0.37619835	0.28482926	0.65178598	0.15641073
cg16764342	FGFR1;FGFR1;FGFR1;FGF	brown	-0.0018232	-0.3782073	0.19845586	0.1624074	0.62897907	0.28066215
cg01102854	MARK2;MARK2;MARK2;	brown	-0.0804725	-0.4381059	0.19617414	0.41500099	0.84043227	0.09785
cg05743278	KLHDC10;KLHDC10;KLH	brown	-0.2122467	-0.5841804	0.14386236	0.13718827	0.71940796	0.04803439
cg20685674	STMN1;STMN1;STMN1;ST	brown	-0.3238451	-0.3797764	0.17356303	0.13708568	0.35642049	-0.0049383
cg09175009	FAM18A;FAM18A;FAM18	brown	-0.1048459	-0.4468215	0.40685029	0.29276495	0.62673466	0.14788322
cg12738718	PRRT1;PRRT1;PRRT1;PRR	brown	-0.3021116	-0.5401235	0.01344708	0.09842311	0.54882813	0.01969357

cg19577324	AC098820.3;AC098820.3;RP	brown	-0.229279	-0.4807392	0.24641185	0.28504166	0.65885243	0.05988917
cg17850049	FHAD1;RP3-	brown	0.12852489	-0.1447004	0.08121206	0.38354756	0.40537256	0.29817939
cg20497091	GDPD3;GDPD3;GDPD3	brown	-0.1470703	-0.5921985	0.12797762	0.3217402	0.90547051	0.09707474
cg05751442	AP001187.9	brown	0.05023878	-0.4849419	0.26660819	0.44393221	0.85959908	0.41585718
cg24351013	BMPR1A;BMPR1A	brown	-0.4483546	-0.5186942	0.15691489	0.13774033	0.48044929	-0.0984369
cg07198360	CLEC2D;CLEC2D;CLEC2D;	brown	0.24073386	0.68383361	0.05326015	-0.1638631	-0.9091423	0.07798427
cg02687322	AC087491.2	brown	-0.0967153	-0.6232848	0.09251089	0.29844214	0.9600738	0.10854028
cg05971061	SGIP1;SGIP1;SGIP1;SGIP1;	brown	-0.2428565	-0.4077219	0.24749304	0.10657797	0.45358298	0.20968769
cg14995562	CSNK1E;CSNK1E;CSNK1E	brown	-0.3295727	-0.738506	0.02651876	0.11052317	0.83400079	-0.0182562
cg17382048	CXCR5	brown	0.23287189	0.67805518	-0.0787336	-0.1656034	-0.8651755	-0.0006784
cg21155203	C21orf58;C21orf58;C21orf58	brown	-0.0818191	-0.6066492	0.19718259	0.34947007	0.93153107	0.14878241
cg15029032	RP11-158M2.4;RP11-	brown	-0.1381616	-0.4449496	0.1161936	0.10642518	0.49253758	0.08242131
cg03537644	PLD3;PLD3;PLD3	brown	-0.0793394	-0.6197482	0.18564026	0.34435378	0.92489748	0.1842632
cg21863307	CNNM4;CNNM4;CNNM4	brown	0.01827547	-0.2906288	0.37111337	0.22694179	0.59276743	0.25194319
cg18470593	CC2D2A;CC2D2A;CC2D2A	brown	-0.2556453	-0.672943	0.12934816	0.14761007	0.71092076	0.09520117
cg04222933	LY9;LY9;LY9;LY9;LY9;LY	brown	-0.223803	-0.2598355	0.15716608	0.35016796	0.52241601	0.08474861
cg01186535	RN5S40	brown	-0.0583797	-0.5474329	0.12261302	0.31029187	0.82582479	0.24777116
cg08189186	C19orf33	brown	0.02555301	-0.2834045	0.25024813	0.30153337	0.52055452	0.26857431
cg01298987	HIRIP3;HIRIP3;INO80E;IN	brown	-0.1482428	-0.6080046	0.15282878	0.24175429	0.92201975	0.11970544
cg14269973	SIAH3;SIAH3	brown	-0.2875689	-0.61218	0.15874173	0.20693038	0.60490025	-0.0059792
cg05168468	PRR16;PRR16	brown	-0.3632135	-0.5496293	0.20995716	0.23013984	0.64532989	-0.0666332
cg23500850	AC015969.3;PLEK	brown	-0.270554	-0.3585761	0.09249124	0.30989187	0.59713447	-0.0469861
cg24119869	KIR3DX1;KIR3DX1;KIR3D	brown	0.1727418	0.6611441	-0.0274814	-0.2308221	-0.8031455	-0.0281024
cg07550662	PSMA6;PSMA6;PSMA6;PS	brown	-0.0868607	-0.5213783	-0.0006664	0.2879016	0.89690831	0.14015895
cg05451210	U4	brown	-0.2790648	-0.5988605	0.07670398	0.18635111	0.74435588	-0.0225284
cg10699171	FAM65B	brown	0.11174006	0.64093654	-0.0558481	-0.2736538	-0.9338004	-0.0823012
cg07395354	AC005550.4;MEOX2	brown	-0.2595703	-0.6460429	0.25714157	0.07700135	0.61404631	0.04918532
cg00407468	DENND1A;DENND1A;DE	brown	-0.4549354	-0.566639	0.07707754	0.08528227	0.60798344	-0.1655046
cg09903452	SEPT9;SEPT9;SEPT9	brown	-0.1131757	-0.557516	0.15759319	0.18332398	0.87053999	0.11723126
cg22516643	NTNG1;NTNG1;NTNG1;NT	brown	-0.3035637	-0.591583	0.11524624	0.08403782	0.59306407	0.03654755
cg08262530	GSTO1;GSTO1;GSTO1;GST	brown	-0.197023	-0.7472039	0.06627685	0.19106365	0.91867616	0.05739516
cg25353281	TMBIM1;TMBIM1;PNKD;T	brown	-0.3102656	-0.5399058	0.08041376	0.06159067	0.56304546	-0.0089935
cg06399735	SNORA62;PSD3	brown	-0.3005063	-0.518787	0.19983127	0.13482781	0.71192416	0.09508887
cg03533141	RPS8;RPS8;SNORD38B;RP	brown	-0.2703488	-0.3557164	0.34109004	0.36475238	0.61476189	0.09424322
cg04125972	C7;C7	brown	0.19811535	0.70750451	-0.071913	-0.1555418	-0.7334447	-0.0725182

cg01982602	CNTLN;CNTLN;CNTLN	brown	-0.1841571	-0.613205	0.14252897	0.19364464	0.84390413	0.07689826
cg13357383	ACAP1;ACAP1;ACAP1;AC	brown	-0.1567915	-0.7035909	0.02762652	0.2433848	0.90238923	0.10423475
cg21469373	BCL7C;BCL7C;BCL7C;BCL	brown	-0.1508678	-0.6147122	0.33106197	0.18152895	0.7071676	0.18471694
cg21243455	RP11-6F2.6;RP11-6F2.4	brown	-0.190867	-0.3110329	0.24024341	0.14360959	0.5406637	-0.0343012
cg09168066	TP53;TP53;TP53;TP53;TP53;	brown	-0.0266545	-0.2104626	0.0186432	0.35522604	0.57725647	0.14068886
cg14974772	FBLN5;FBLN5;FBLN5;FBL	brown	-0.1897735	-0.6684209	0.05900822	0.19184131	0.88159465	0.05212905
cg11426013	MMRN2;MMRN2;SNCG;S	brown	-0.2346709	-0.4139245	-0.0059001	0.26190826	0.57184753	-0.0633792
cg24601404	AC020571.3;AC020571.3;A	brown	0.0377039	0.35166542	-0.097554	-0.1407832	-0.5799401	-0.2426043
cg00225858	ADAD2;ADAD2;ADAD2;A	brown	-0.0106475	-0.5373689	0.10060222	0.33309275	0.89875973	0.17746992
cg12651065	ZNF442;ZNF442;ZNF442;Z	brown	-0.4219813	-0.6002554	0.18452729	0.19136283	0.57508896	-0.0951164
cg27325404	CAMK2G;CAMK2G;CAM	brown	-0.062839	-0.6061978	0.11921207	0.33488581	0.95386244	0.14097727
cg07842386	IL23R;IL23R	brown	0.17952218	0.64941838	0.30969346	-0.1356424	-0.770952	0.01543791
cg16169828	SMAD3;SMAD3;SMAD3;S	brown	0.00695732	-0.3713261	0.15942552	0.26775937	0.71190659	0.23865518
cg22166739	UCP2;UCP2;UCP2	brown	-0.3642407	-0.3539782	0.07734734	0.10615485	0.31602397	-0.0773833
cg22433190	TMEM91;TMEM91	brown	-0.2229326	-0.7556635	0.07148681	0.13518081	0.86902186	0.09434653
cg15826398	ITGAE	brown	-0.2181108	-0.5037542	0.34398478	0.2264562	0.58344874	0.10193424
cg21160357	TNS3;TNS3;TNS3;TNS3	brown	0.12275218	-0.2144046	0.12156394	0.45647694	0.55558341	0.19296663
cg22486928	SNRPF;SNRPF;SNRPF;SN	brown	-0.1594092	-0.679024	0.10559415	0.28363023	0.83248825	0.1198448
cg17311122	CTC-340I23.2	brown	0.25751744	0.73132518	-0.0519849	-0.0871328	-0.6954349	0.00447877
cg06113298	CTD-2547E10.2;CTD-	brown	0.09490863	-0.4264625	0.22877163	0.33182623	0.80636724	0.35519031
cg00197612	TIAM1;TIAM1;TIAM1;TIA	brown	-0.3099619	-0.6510205	0.11648112	0.06170187	0.76007721	-0.0991854
cg08225889	FOXA2;FOXA2;FOXA2	brown	-0.520407	-0.6304224	0.05382861	-0.0197956	0.54953164	-0.2165379
cg23332989	C7orf49;C7orf49;C7orf49;C7	brown	-0.1083307	-0.4146139	0.19712246	0.33227467	0.79930486	0.13159552
cg05902684	FOXP1;FOXP1;FOXP1;FO	brown	-0.2911962	-0.6740666	-0.089371	0.09461219	0.67971672	0.10535647
cg15028548	ABI3BP;ABI3BP;ABI3BP;A	brown	-0.0910081	-0.1656465	0.0376681	0.29681655	0.3732439	0.08224193
cg15726326	ALDH3A1;ALDH3A1;ALD	brown	-0.1981644	-0.4757151	-0.0507935	0.1175688	0.60327438	0.00255367
cg18194227	PTOV1;PTOV1	brown	-0.1801124	-0.2007769	0.13845698	0.29091257	0.33338491	0.02456913
cg08313271	WNK1;WNK1;WNK1	brown	0.06217284	-0.3562124	-0.0241875	0.59491838	0.75808824	0.17181277
cg18940774	TLE2;TLE2;TLE2;TLE2	brown	-0.3418958	-0.6221353	0.18012843	0.16998296	0.73607629	0.01810217
cg15767067	ODZ2;CTB-	brown	-0.3188888	-0.708367	0.06927652	0.0217015	0.67574993	0.04578995
cg20237752	ZNF608;ZNF608;ZNF608;Z	brown	-0.133909	-0.3324556	0.25798134	0.11056785	0.39781611	0.12207751
cg01092582	PVT1;PVT1;PVT1;PVT1;PV	brown	0.26832873	0.74529998	-0.0951042	-0.1525046	-0.8966187	0.08743364
cg22600584	RP11-84O12.4	brown	-0.2895402	-0.6154936	0.19984435	0.18165941	0.73881577	0.05412821
cg04064028	BBS5;BBS5;BBS5;BBS5;BB	brown	-0.2659016	-0.3739447	0.32283559	0.14905051	0.40070729	0.08435172
cg19494100	RNF144B;RNF144B	brown	-0.1488569	-0.3747781	0.38405241	0.27520528	0.58290315	0.06797769

cg21835868	AC114798.1	brown	-0.2690623	-0.7226823	0.14249769	0.09470349	0.87365704	-0.0282618
cg17877071	NICN1-	brown	-0.1356633	-0.6516432	0.16184497	0.30724147	0.90704626	0.07507859
cg18206515	NT5DC4;NT5DC4	brown	-0.0515575	-0.5614106	0.13546992	0.2305571	0.89082276	0.14793418
cg12598270	ZNF396	brown	-0.2432164	-0.3667606	0.10464501	0.15653379	0.43666527	0.04185011
cg24516497	RP11-810M2.2	brown	0.09479501	-0.353164	0.03824833	0.48329295	0.75272893	0.24847341
cg09462826	FADS1;FADS1;FADS1;FA	brown	-0.4396011	-0.6383	0.06778249	-0.0419215	0.59074398	-0.0308819
cg10555171	GPR97;GPR97	brown	-0.1750157	-0.6154665	0.16639018	0.1935346	0.87341426	0.08667941
cg07850154	RNF180;RNF180;RNF180	brown	-0.173554	-0.3600704	0.15943546	0.24530143	0.5272152	0.01893182
cg03049863	YIF1B;YIF1B;C19orf33	brown	-0.1858638	-0.4706706	0.163428	0.09025465	0.50143548	0.04859633
cg03370117	DCAF5;DCAF5;DCAF5;DC	brown	-0.2692903	-0.7771108	0.24024287	0.16825669	0.84035982	0.11048602
cg22239727	E2F8;E2F8;E2F8;RP11-	brown	-0.1426799	-0.6554683	0.13115518	0.22779212	0.93871884	0.05754464
cg07330123	LA16c-321D4.2	brown	0.01895462	-0.437493	0.27571512	0.3184743	0.74747718	0.25183026
cg18537183	XRCC3;XRCC3;XRCC3;XR	brown	-0.0702013	-0.5665905	0.14727218	0.22159858	0.77411319	0.16663371
cg17930099	MCF2L;MCF2L;MCF2L;MC	brown	-0.3949883	-0.7280768	-0.0190254	0.0103382	0.71940464	-0.1060927
cg25832100	SLC35E1;SLC35E1;CALR3	brown	0.09861436	-0.2708562	0.31167011	0.37739539	0.37234127	0.16418089
cg13566336	CD2BP2;RP11-	brown	-0.075764	-0.4231785	0.30390814	0.38207349	0.72163721	0.18696064
cg16039157	B2M;B2M;B2M;B2M;B2M;	brown	0.26330604	0.70877773	0.0349657	-0.1613494	-0.9087087	0.02520754
cg24235904	P2RY2;P2RY2;P2RY2	brown	-0.3010542	-0.6915524	0.06493985	0.12125927	0.70695497	0.05840875
cg12591090	PILRB;PILRB;PILRB;PILRB	brown	0.08621915	-0.4743254	0.1481501	0.4546616	0.88615896	0.20462514
cg03613649	FGF11;FGF11;RP11-	brown	0.1157693	-0.3464904	0.11310822	0.41800849	0.77473548	0.29939684
cg12673638	NUP214;NUP214;NUP214;	brown	-0.0064921	-0.4551314	0.20315565	0.41890186	0.86729661	0.21843707
cg03621001	RAB8A	brown	-0.1629168	-0.2741435	0.1499765	0.20136671	0.32844887	0.05566395
cg10348013	KIAA1143;KIAA1143;KIF1	brown	-0.1536546	-0.2852211	-0.106315	0.12252091	0.45512154	0.0817139
cg10053912	TRIM29;TRIM29;TRIM29;T	brown	0.22186652	-0.2914222	0.02897706	0.55584009	0.78800436	0.28697883
cg16956345	MARS;MARS;MARS;MAR	brown	-0.107983	-0.5539014	0.06626265	0.26286981	0.88779923	0.16635291
cg03902505	RP11-309I15.1	brown	-0.1512998	-0.6266853	0.1347928	0.20520597	0.82816217	0.04568809
cg08615650	ADRBK1;ADRBK1;ADRB	brown	-0.0305242	-0.5419578	0.29858652	0.39886974	0.88208621	0.1948704
cg21446892	ARPC1B;ARPC1B;ARPC1B	brown	-0.4111814	-0.3944999	0.06084523	0.10713271	0.44224993	-0.1921644
cg13284470	ARHGAP25;ARHGAP25;A	brown	0.00940311	-0.4082365	0.22735953	0.33086387	0.75191498	0.13645391
cg23494863	STAR;STAR;STAR;RP11-	brown	0.18076232	-0.2926514	0.06399159	0.59285663	0.74736542	0.18087118
cg06896909	TMEM196;TMEM196;TME	brown	-0.3773716	-0.7048629	0.1326009	0.12714273	0.65264377	0.0020814
cg03645851	AC004471.9;DGCR2;DGCR	brown	-0.2927834	-0.763263	0.04277579	0.10425194	0.77250242	0.05816238
cg23476065	RP4-555L14.5;RP4-	brown	-0.1394424	-0.240439	0.27599705	0.16431222	0.31015989	0.20211056
cg00628816	CTD-2555A7.1	brown	-0.1853345	-0.1943241	0.24142002	0.18143696	0.31617265	0.06214555
cg24721963	GFI1B;GFI1B;GFI1B;GFI1B	brown	-0.1486256	-0.6258214	0.13623739	0.26591532	0.94802543	0.10761318

cg12298872	C1orf148;IBA57;C1orf148;C	brown	0.07418752	-0.4693145	0.28244942	0.44132235	0.72155225	0.30324606
cg26605447	PELO;ITGA1;PELO;CTD-	brown	-0.2578194	-0.4588552	0.09457802	0.10695135	0.53806894	-0.1135073
cg25423145	ARHGAP27	brown	0.38989022	0.80391594	-0.0378489	-0.0732523	-0.7706494	0.12196692
cg24375627	S100A6;S100A6;S100A6;S1	brown	-0.1198761	-0.5563851	0.08618229	0.13366232	0.83850758	0.10086624
cg09889479	SLC9A3R2;SLC9A3R2;SLC	brown	-0.286177	-0.2131868	0.17893031	0.09879682	0.39083914	-0.0073957
cg11323506	CKMT2;CKMT2;CKMT2;C	brown	-0.0956998	-0.6608736	0.07216548	0.24123352	0.86038283	0.06693978
cg24689379	RP3-403L10.3;SNX9	brown	-0.147137	-0.3020712	0.23777901	0.45775569	0.41557174	-0.0082306
cg05131483	ERN2;ERN2;ERN2	brown	-0.3645928	-0.6423074	0.11288009	0.07479614	0.62868185	-0.0966528
cg05337019	SLC25A34;RP11-288I21.1	brown	-0.2482883	-0.6727427	0.03695272	0.22231116	0.90793436	0.03365628
cg17890764	ITIH4;MUSTN1;ITIH4;ITIH	brown	0.0590465	-0.2326582	0.01454244	0.52004191	0.67917318	0.15699454
cg15097669	DNAJC14;DNAJC14;DNAJ	brown	-0.1973872	-0.6809441	0.07429276	0.1739071	0.80825942	0.08145105
cg13738609	C17orf74;C17orf74;RP11-	brown	-0.0754577	-0.6007182	0.10606336	0.3513242	0.94500135	0.13619401
cg11970682	DTNB;DTNB;DTNB;DTNB;	brown	0.0508692	-0.4459746	0.01802749	0.42589386	0.87131874	0.13655498
cg06834875	HHIPL1;HHIPL1	brown	-0.2157005	-0.5735343	0.40680034	0.16950952	0.57587696	0.1857992
cg01798114	RP11-396J6.1;CPA6	brown	-0.1128965	-0.5863775	0.01662311	0.26003244	0.83410031	0.09879817
cg19180828	RNF207;RNF207;RNF207;R	brown	-0.0729993	-0.3275898	0.0272638	0.259974	0.37417769	0.20184644
cg26072906	BATF3	brown	-0.404019	-0.5805935	0.16151406	0.04210055	0.55523847	-0.0806562
cg10599948	RASIP1;IZUMO1	brown	-0.0884367	-0.330851	0.09344304	0.28967325	0.41182097	0.20017755
cg24794709	C14orf93;C14orf93;C14orf93	brown	-0.2245469	-0.3946243	0.14553143	0.215613	0.46994181	0.0087727
cg04476874	MIER2	brown	-0.0391988	-0.5300141	0.20270905	0.20764303	0.89098082	0.11076605
cg10943955	SLC9A3R2	brown	0.04935165	-0.2052719	0.13515036	0.22606732	0.49388491	0.16789678
cg20267828	THEMIS;THEMIS;THEMIS;	brown	0.19794786	0.6936805	-0.0075177	-0.1920722	-0.9304869	-0.0386606
cg21097733	HOXB-AS1;HOXB-	brown	-0.0760985	-0.5013806	0.1477485	0.276976	0.80537485	0.19012315
cg16419533	LPGAT1;LPGAT1;LPGAT1;	brown	-0.0711451	-0.5780385	0.21697537	0.3601246	0.84838192	0.16138076
cg24525223	REPIN1;REPIN1;REPIN1;R	brown	-0.2991747	-0.7106508	0.25867327	0.1652333	0.70876005	-0.0047541
cg16049898	ZP2;ZP2;ZP2;ZP2	brown	-0.1264318	-0.5620301	0.15849752	0.18714721	0.82438516	0.07795265
cg25182165	AKAP1;AKAP1	brown	0.0504606	-0.3293887	0.28745138	0.50878435	0.76943369	0.25423919
cg06808585	KCNJ9	brown	-0.0650047	-0.320309	0.2398739	0.3188353	0.55952313	0.21740294
cg22795426	SLC43A3;SLC43A3;SLC43	brown	-0.2363029	-0.1413434	0.31038005	0.25936842	0.37279379	-0.0676172
cg09433908	C1orf105	brown	0.00376054	-0.5451	0.1251475	0.41890059	0.94340324	0.16340495
cg10502175	FAM65A;FAM65A;FAM65	brown	-0.0054674	-0.1959608	0.34630078	0.28327695	0.43208955	0.1481157
cg22818215	CTA-109P11.4	brown	-0.014106	-0.4907869	0.1239512	0.41686699	0.91829561	0.18239761
cg07187268	C22orf15;C22orf15;C22orf15	brown	-0.1173215	-0.6147372	0.15245596	0.24189004	0.88495472	0.0997566
cg02187522	AVP	brown	0.02141279	-0.3606895	0.32374603	0.27416086	0.57134262	0.22505194
cg08292207	CNN2;ABCA7;ABCA7;ABC	brown	-0.2121632	-0.4249939	0.28058289	0.2143006	0.41113776	-0.0031549

cg05010967	SPATS1;SPATS1;SPATS1;S	brown	-0.2749681	-0.5510885	0.35523649	0.06580509	0.5526781	0.23840764
cg25073237	ST5;ST5;ST5;ST5;ST5;ST5;S	brown	-0.3297344	-0.750668	0.14518112	0.0628048	0.78658334	0.02214484
cg27173717	MFSD2A;MFSD2A;MFSD2	brown	-0.0996846	-0.4642147	0.17118057	0.25391607	0.65413246	0.13731572
cg14886255	MGAT4B;MGAT4B;MGAT	brown	-0.120065	-0.6345587	0.19057161	0.32351197	0.9392562	0.15862049
cg25811375	TINAGL1;TINAGL1;TINAG	brown	0.09538468	-0.3988371	0.46022727	0.51104937	0.80907386	0.33213787
cg21180008	TRABD;TRABD;TRABD	brown	0.00487414	-0.3563125	0.48296963	0.35429946	0.57140609	0.43697046
cg03285215	DDX39B;DDX39B;DDX39	brown	-0.011523	-0.4429541	0.22723573	0.39660436	0.82830857	0.20938698
cg10866623	TTLL6;TTLL6;TTLL6;TTLL6	brown	0.23632106	-0.2402081	0.20326444	0.72599259	0.66537031	0.38746254
cg26400750	TRAJ21;TRAJ20	brown	0.22874528	0.52841676	0.2842119	-0.1212397	-0.5920911	0.06554714
cg14787477	NAGLU;NAGLU	brown	0.05673916	-0.3847724	0.2127541	0.42483048	0.71087479	0.31442197
cg07805981	METTL7B	brown	-0.1629509	-0.48358	0.21891446	0.17785082	0.65053059	0.13311903
cg13740929	LY6G5C;LY6G5C	brown	-0.1165032	-0.2887398	0.03105435	0.15101264	0.3994826	0.13357232
cg19098743	ZMYND11;ZMYND11;ZM	brown	-0.3233646	-0.6586118	0.22418315	0.06075842	0.66282089	0.04948443
cg04407135	RP13-580F15.2;SPNS3;RP13-	brown	0.00971716	-0.5053653	0.13776726	0.28175242	0.87883192	0.20287943
cg13351141	MED12L;MED12L	brown	-0.0203581	-0.3039718	-0.0257786	0.29682822	0.39640766	0.23267415
cg06032302	AC116366.5;AC116366.5;C5	brown	-0.3703739	-0.2119051	0.19312255	0.10779357	0.32401938	-0.1339642
cg03635685	CABIN1;CABIN1	brown	-0.1389089	-0.6083734	0.06861523	0.2424541	0.89212764	0.07337353
cg08939481	NDRG2;NDRG2;NDRG2;N	brown	-0.1264431	-0.6228775	0.06834458	0.16410992	0.70820403	0.14347368
cg21094405	MCF2L;MCF2L;MCF2L;MC	brown	-0.2110187	-0.4812659	0.15192993	0.13966627	0.6053917	-0.0102234
cg22403266	IGFBP3;IGFBP3;IGFBP3;IG	brown	-0.4011098	-0.7638064	0.10559104	0.04468699	0.7040191	0.02467248
cg22214659	MAN2A2;MAN2A2;MAN2	brown	0.07597346	-0.4710808	0.26897022	0.44256737	0.77968795	0.21334873
cg15314957	RP11-513H8.1	brown	0.1568423	0.67706129	-0.065873	-0.210527	-0.9052439	-0.1231268
cg13985445	CTSL1;CTSL1;CTSL1;CTSL	brown	-0.2209184	-0.6050533	0.23563015	0.19182846	0.76116902	0.06002797
cg02816049	RP11-401P9.6	brown	-0.1432151	-0.5184812	0.19797009	0.17995126	0.72787822	0.17601724
cg19815863	EMILIN1;EMILIN1	brown	-0.2474596	-0.6553405	0.18836464	0.10473709	0.74595601	0.07009553
cg13583774	PHLDB1;PHLDB1;PHLDB1	brown	-0.0149369	-0.4354958	0.21167507	0.27348213	0.79383922	0.16154024
cg01307229	ULK1	brown	-0.2194252	-0.629704	0.15231699	0.21461197	0.73483449	0.10341469
cg19517940	RP11-543D5.1	brown	-0.1190633	-0.1513854	0.2055108	0.27569548	0.31560446	-0.0340535
cg01811796	STMN1;STMN1;STMN1;ST	brown	-0.1694295	-0.4457963	0.20690548	0.19480597	0.52547161	0.12434383
cg25483706	YIPF3;YIPF3;YIPF3;YIPF3;	brown	0.05144831	-0.46261	0.09079036	0.42082995	0.89355876	0.22035991
cg13371740	RP11-338C15.3	brown	-0.2939187	-0.625129	0.02191041	0.21436788	0.81280748	-0.0527723
cg06711090	OAF;OAF	brown	-0.3093564	-0.6014737	0.1557259	0.13536439	0.69211854	0.03326576
cg07234388	TMEM79;TMEM79;C1orf85;	brown	-0.0533167	-0.1999016	0.21005247	0.31144929	0.50719283	0.22406287
cg24425191	IL32;IL32;IL32;IL32	brown	0.23865469	0.67974653	0.03357909	-0.1693199	-0.9007105	0.07834039
cg13265003	SLC37A1;SLC37A1;SLC37	brown	0.00075275	-0.5188952	0.09356558	0.38508357	0.89112331	0.17990593

cg07713896	FAM92A1;FAM92A1;FAM	brown	-0.3655177	-0.7116646	0.11520317	0.07060085	0.68017674	0.01018352
cg24425171	POMC;POMC;POMC;POM	brown	-0.2505863	-0.6922328	0.13018758	0.11953133	0.68491339	0.11876643
cg07441888	TOM1;TOM1;TOM1;TOM1;	brown	0.19432366	0.74819925	-0.0875128	-0.1580669	-0.9203126	-0.0977251
cg21686808	CALHM2;CALHM2;CALH	brown	-0.2894711	-0.3335362	0.15874464	0.14212951	0.35552693	-0.0546985
cg04677037	DHRS7;DHRS7;DHRS7;DH	brown	-0.4002209	-0.4170125	-0.0103932	0.08519135	0.52898038	-0.2198785
cg17285458	CLEC10A;CLEC10A;CLEC1	brown	-0.2684886	-0.7352597	0.23531119	0.10524061	0.80135934	0.0970734
cg26066180	COL20A1;COL20A1	brown	0.08243226	-0.4391049	0.31087871	0.42941654	0.86393799	0.30064735
cg07328962	LGALS8;LGALS8;LGALS8;	brown	0.23791955	0.72026769	0.05804156	-0.1130088	-0.8646304	0.01276303
cg19371795	INSR	brown	-0.1910755	-0.4706073	0.2420552	0.17685402	0.5036035	0.13417673
cg06645921	SLC2A14;SLC2A14;SLC2A	brown	-0.0538421	-0.3593452	0.1589927	0.10235184	0.45842402	0.07387925
cg26314868	STMN1;STMN1;STMN1;ST	brown	-0.4849543	-0.4477212	0.1154471	0.03192107	0.36954311	-0.1145399
cg13150534	SLC25A10;SLC25A10;SLC2	brown	0.08603093	-0.3035543	0.39436659	0.50454989	0.5649716	0.29614653
cg06752482	C17orf64;C17orf64;C17orf64	brown	-0.0706263	-0.5611606	0.2340614	0.26084981	0.85593324	0.1207885
cg09195408	RP11-447M4.1	brown	0.03038055	-0.2378132	0.564776	0.31257742	0.48313839	0.44062297
cg20006825	PTMS;PTMS;PTMS;PTMS;	brown	-0.2164875	-0.7060626	0.19306353	0.18392501	0.77091642	0.06717998
cg25173739	PDLIM7;PDLIM7;PDLIM7;	brown	-0.0541885	-0.5435822	0.34276465	0.38531585	0.84979182	0.21361471
cg01603095	SLC2A13;SLC2A13	brown	-0.1522709	-0.413335	0.39065525	0.23734768	0.53322741	0.13760172
cg02420582	RP11-410N8.3;C20orf112	brown	-0.1957644	-0.6667886	0.23923629	0.20078125	0.79631904	0.21348421
cg07845392	SLC25A10;SLC25A10;SLC2	brown	-0.2405465	-0.6382851	0.23133873	0.18788085	0.68523226	0.06898133
cg20147290	NUP210L;NUP210L	brown	0.0057391	-0.5112468	0.00442157	0.36990853	0.907594	0.10809327
cg05195823	PVT1;PVT1;PVT1;PVT1;PV	brown	-0.0070356	-0.4188084	0.25764619	0.3346393	0.69505388	0.27077995
cg17904575	PPP2R5C;PPP2R5C;PPP2R5	brown	0.1370802	0.61303757	-0.0265981	-0.1249435	-0.837682	-0.1216943
cg19815228	RP11-432J24.2	brown	-0.0453807	-0.3076549	0.07539884	0.31142987	0.55184773	0.08181964
cg03409668	SLC26A8;SLC26A8;SLC26	brown	0.04173301	-0.477646	0.18745422	0.32856052	0.88932863	0.27735846
cg09961785	HIP1R;HIP1R;HIP1R	brown	-0.030315	0.01507051	0.32008306	0.38478886	0.26692685	0.25479251
cg09671955	RP1-206D15.3;RP1-	brown	-0.0208188	-0.5321656	0.17038225	0.35496613	0.77165206	0.26771567
cg07496388	ANK3;ANK3;ANK3;ANK3;	brown	-0.013944	-0.4593496	0.12112483	0.16449751	0.588512	0.22374861
cg19271626	SLC26A8;SLC26A8;SLC26	brown	-0.0637219	-0.4394242	0.24006127	0.23945623	0.78885637	0.07765817
cg11063364	GSTM2;GSTM2;GSTM2;GS	brown	-0.0763145	-0.5581356	0.07043425	0.2359451	0.8328592	0.17360016
cg23780797	AC004069.2	brown	0.24373672	0.72502248	0.067369	-0.1300063	-0.9001652	0.01513226
cg08893895	POLE2;POLE2;POLE2;POL	brown	-0.171212	-0.6667283	0.33514601	0.12964509	0.7754493	0.13629237
cg00662002	PPP1R13B;PPP1R13B;PPP1	brown	-0.375986	-0.5105124	0.32247917	0.06502055	0.45535194	-0.1115129
cg20251878	DCTN1;DCTN1;DCTN1;DC	brown	-0.096866	-0.6496116	0.18073107	0.30214212	0.94162243	0.24183739
cg11020853	FAM198A;FAM198A;FAM	brown	-0.3403288	-0.6234753	0.29897031	0.14567198	0.62036708	0.07387191
cg13931228	MPP6;MPP6;MPP6;MPP6;	brown	-0.2321831	-0.3055741	0.13604996	0.20809356	0.50406709	-0.0125541

cg20996682	MAGI2;MAGI2;MAGI2;MA	brown	-0.3543992	-0.7769195	0.04272248	0.0761431	0.72051416	0.00914685
cg23021168	PAM;PAM;PAM;PAM;PA	brown	-0.2848892	-0.5890982	0.06147054	0.16769828	0.76491457	-0.0209967
cg18341263	POLR2M;POLR2M;POLR2	brown	-0.4123167	-0.3620078	0.20834096	0.16315792	0.38702106	-0.0789098
cg07177860	FAM59B	brown	-0.407158	-0.8002628	0.07644187	0.05425923	0.7531788	-0.0882763
cg03742771	NFATC1;NFATC1;NFATC1	brown	-0.346747	-0.5722338	0.10372687	0.10881918	0.55366502	-0.0117292
cg21640749	CD300LF;CD300LF	brown	0.21177959	-0.2340348	0.17943396	0.71224287	0.70334688	0.32928176
cg00089550	SLC43A1;SLC43A1;SLC43	brown	-0.033449	-0.3846796	0.2367514	0.26678453	0.59555428	0.1230418
cg01809941	FAM105A	brown	-0.1150653	-0.4713381	-0.0011003	0.20051766	0.56302876	0.16080665
cg13826081	PIP4K2B	brown	-0.221007	-0.4494223	0.19939958	0.24580453	0.53319855	0.09213853
cg09258150	ICAM4	brown	-0.2044146	-0.4726313	-0.0318541	0.05436305	0.47642641	0.0976874
cg13993688	GPC2;GPC2;GPC2;STAG3;	brown	0.02054452	-0.5195981	0.13177434	0.3871965	0.83890149	0.22878766
cg06930745	MEF2D;MEF2D;MEF2D;M	brown	-0.1890167	-0.4443464	0.12929046	0.21160999	0.54955294	0.07036376
cg07538190	C8orf58;C8orf58;C8orf58;C8	brown	0.03480422	-0.3911334	0.21961765	0.32006818	0.70492123	0.28126875
cg07478210	PQLC3;PQLC3;PQLC3;PQL	brown	-0.1052273	-0.2623758	0.01127453	0.30084871	0.445545	0.16311781
cg10122916	HOXB-AS1;HOXB-	brown	-0.0502081	-0.4040412	0.28062199	0.32030707	0.75196887	0.14237976
cg26104910	PTH1R	brown	-0.1331105	-0.3473901	-0.2462317	-0.1351617	0.31953096	-0.3567626
cg02064408	PLK1;PLK1;PLK1;PLK1;PL	brown	-0.3983499	-0.3545021	0.07041141	-0.0235122	0.35578201	-0.1457652
cg00704554	RP11-713P17.3;RP11-	brown	0.0696655	-0.3790089	0.36141286	0.48794517	0.70202577	0.23864316
cg10785537	DCTN1;DCTN1	brown	-0.1512699	-0.644287	0.21496013	0.26401255	0.82886216	0.13258647
cg20641955	SLC38A11;SLC38A11;SLC3	brown	-0.2430522	-0.3785474	0.19177284	0.21451674	0.41882936	0.10580205
cg06048358	PTPDC1	brown	-0.1810335	-0.269259	0.31727048	0.10517476	0.3877815	0.11093046
cg08904394	CCDC88B;CCDC88B;CCDC	brown	0.03936456	-0.4887751	0.2299825	0.30378129	0.86550723	0.20907208
cg22833375	PTGR2;PTGR2;PTGR2;PTG	brown	-0.3327189	-0.6440279	0.17993487	0.18603689	0.68463653	-0.0576932
cg02256105	AC009404.2;AC009404.2	brown	0.05156921	-0.3509816	0.08623607	0.45305309	0.80152548	0.13770506
cg09372007	RP11-	brown	0.16921227	0.66121116	0.13816066	-0.1694132	-0.8768691	-0.0510337
cg20345446	OSBPL5;OSBPL5;OSBPL5;	brown	-0.2517002	-0.3240505	0.10566866	0.16180185	0.38446269	-0.0116697
cg12243822	DNASE1L3;DNASE1L3;DN	brown	-0.2143934	-0.405432	0.08026607	0.09141005	0.42849686	0.03890841
cg20574493	SLC24A6;SLC24A6;SLC24	brown	-0.0362128	-0.5784471	0.16676281	0.37413684	0.86644895	0.20428319
cg23253654	MAP2;MAP2;MAP2;MAP2;	brown	-0.3683356	-0.4466138	0.20885949	0.06328782	0.40409131	-0.0460244
cg03589001	MORF4L1;MORF4L1;MOR	brown	-0.1929474	-0.4411212	0.14340376	0.10643002	0.46071461	0.05468337
cg13868190	MFAP4;MFAP4;MFAP4	brown	0.04185082	-0.1894467	0.39717837	0.3830094	0.46413854	0.28598262
cg03152092	TMEM91;B9D2;TMEM91	brown	-0.2659077	-0.2249506	0.24707746	0.20980451	0.37778339	-0.0335744
cg25976340	UEVLD;UEVLD;UEVLD;U	brown	-0.1691353	-0.5211204	0.34314537	0.32610233	0.65663637	0.20065326
cg18637698	SNCAIP;SNCAIP;SNCAIP;	brown	-0.2910377	-0.4513968	0.2395363	0.16014986	0.44819242	0.0467229
cg08655016	UAP1L1;UAP1L1;UAP1L1	brown	-0.0634908	-0.2813625	0.35772858	0.38790874	0.50647655	0.25527464

cg06708720	ERC1;ERC1;ERC1;ERC1;ER	brown	-0.0980468	-0.5772692	0.09490392	0.26514299	0.91175445	0.08798006
cg22766888	OSBPL5	brown	-0.0233076	-0.3395004	0.12234533	0.12253784	0.48726816	0.18456819
cg13171103	LPAR5;LPAR5;LPAR5	brown	-0.159853	-0.4508779	0.27270913	0.34551624	0.59625263	0.05338757
cg07819065	4-Sep	brown	0.06001151	0.10677551	0.37141336	0.4986783	0.28585823	0.165096
cg08206308	NCOR1;PIGL;PIGL;PIGL;N	brown	-0.3964424	-0.4794115	0.18134996	0.09526381	0.45493486	-0.0950212
cg12210036	GOLT1A	brown	-0.2081714	-0.5785032	0.13387554	0.14459295	0.67456387	0.03077903
cg01185921	ABLIM1	brown	0.14377	-0.2696289	0.11997384	0.37987139	0.7292985	0.28268976
cg19127552	MLF2;MLF2;MLF2;MLF2	brown	-0.0262534	-0.4625994	0.2525901	0.28118363	0.71407767	0.36465349
cg01391427	PTK2;PTK2;PTK2;PTK2;PT	brown	-0.300328	-0.4479527	0.20236806	0.25225191	0.50193916	-0.016108
cg11218383	Y_RNA;DNAJC27-	brown	-0.0350248	-0.2542585	-0.0008484	0.11303075	0.54792998	0.0365673
cg07313319	CSNK1E;CSNK1E;CSNK1E	brown	-0.0426103	-0.5465183	0.14674412	0.2835902	0.88855841	0.19943363
cg21022868	SLAMF9;SLAMF9	brown	0.2406243	0.74093821	-0.2035641	-0.1014838	-0.7059507	-0.1146585
cg02893407	ZC2HC1C;ZC2HC1C;ZC2H	brown	-0.102624	-0.3784086	0.03530831	0.37584784	0.74680308	-0.0074175
cg27510566	KDM6B;KDM6B;KDM6B	brown	-0.0500855	-0.4903487	0.10933494	0.38953737	0.85214206	0.11337211
cg16733993	SEPT11;SEPT11;SEPT11;SE	brown	-0.0275614	-0.3339942	0.28905523	0.26972261	0.55740102	0.10117942
cg19264957	USP36	brown	0.04324288	-0.3871981	0.06786023	0.19312577	0.75088156	0.12880723
cg10960375	ZBTB47;RP4-613B23.1;RP4-	brown	-0.140814	-0.640479	0.10885073	0.2337178	0.94391291	0.09395783
cg10323688	CELF2;CELF2;CELF2	brown	-0.0976553	-0.4358641	0.09458467	0.31247239	0.72423379	0.10248812
cg13571904	PUM1;PUM1;PUM1;PUM1;	brown	0.09821515	-0.4376042	0.07203021	0.31156273	0.80993457	0.21705845
cg25472135	C14orf49	brown	0.0017866	-0.5386839	0.2179966	0.33460703	0.84304298	0.20287786
cg26313173	C2orf40;C2orf40;C2orf40;C2	brown	0.13668519	-0.277719	-0.0232666	0.26839971	0.67195875	0.22526057
cg26712714	RP11-566J3.2	brown	0.02755929	-0.3826783	0.27631059	0.29354039	0.69093625	0.25922686
cg06542808	KRT36	brown	-0.3473512	-0.3618171	0.11814185	0.04836801	0.52954921	-0.0173693
cg12446246	PLXNA2;PLXNA2	brown	-0.3172583	-0.4057314	0.07433004	0.16183996	0.56880579	-0.0796795
cg24824581	CABIN1;CABIN1;CABIN1	brown	-0.0897167	-0.6621539	0.14818529	0.3242738	0.89549403	0.17353514
cg04391048	U6;NT5C1B;NT5C1B	brown	-0.1213969	-0.3355277	0.194489	0.0460627	0.35833885	0.14387575
cg08736088	TTC29;RP11-	brown	-0.0667747	-0.3455578	-0.0289777	0.16442401	0.5491286	0.02052832
cg07985720	MAGI2;MAGI2;MAGI2;MA	brown	-0.3280871	-0.524223	0.18377564	0.07366156	0.54783358	0.03390649
cg03422895	KIAA0664;KIAA0664;KIA	brown	0.29517981	-0.2707165	0.14476125	0.39350221	0.7289384	0.40686492
cg03989792	YPEL4;YPEL4;AP000662.4;	brown	-0.2273046	-0.7353092	0.14901203	0.16392198	0.85478795	0.07058269
cg20969684	TOX2;TOX2	brown	-0.052808	-0.2933636	0.07459305	0.20711834	0.36688005	0.21777316
cg19414706	HNF4G;HNF4G;HNF4G	brown	-0.359147	-0.642042	0.09469512	-0.0104574	0.56248777	-0.0092614
cg11708454	YIF1B;YIF1B;C19orf33	brown	-0.1841903	-0.4660886	0.11497656	0.06835817	0.4731569	0.05782269
cg26346621	PTMS;MLF2	brown	-0.0440247	-0.500142	0.06782831	0.29060843	0.85472175	0.12303314
cg15927328	ANGPTL4;ANGPTL4	brown	-0.1449608	-0.3656678	0.24416872	0.12272996	0.34425215	0.10626108

cg06707515	WDR5	brown	-0.0024425	-0.4088531	0.44803168	0.39116534	0.63351322	0.31555041
cg17633073	UBA7;UBA7;UBA7;UBA7;	brown	0.14325843	-0.281866	0.29138773	0.56800834	0.68441374	0.37208229
cg03555522	PISD;PISD;PISD;PISD;PIS	brown	-0.1833233	-0.6625245	0.08601775	0.25683492	0.89752598	-0.0133389
cg06320982	SLC38A7;SLC38A7;SLC38	brown	-0.4222718	-0.3944243	0.05263222	0.04489602	0.37910753	-0.2303619
cg04754236	DDB1;DAK;DAK;DAK;DA	brown	0.06107029	-0.2705336	0.49202309	0.27361615	0.57013945	0.24377356
cg15469849	RP11-1070N10.3	brown	0.1306715	-0.3767316	0.16167908	0.22293677	0.81520849	0.26604911
cg02712669	C9orf135;C9orf135;C9orf135	brown	0.08105324	-0.2916199	0.09386563	0.23394108	0.49466839	0.1398991
cg27040005	ARAP3;ARAP3	brown	0.00621327	-0.3943307	0.36779111	0.47383084	0.71999204	0.18853485
cg04989970	LMNB2;LMNB2;LMNB2;L	brown	0.05377104	-0.4634446	0.17868459	0.32532911	0.86503679	0.15702939
cg14321269	XAF1;XAF1;XAF1;XAF1;X	brown	0.02652994	-0.3007131	-0.0337978	0.3764473	0.57831507	0.25820971
cg26458867	CCDC162P;snoU13	brown	-0.0035791	-0.2061429	0.26778315	0.381684	0.51309499	0.11815805
cg03107987	C19orf77	brown	0.09389793	-0.2171152	0.33469255	0.41347099	0.56206562	0.34415173
cg00065048	FGR;FGR;FGR	brown	-0.1673914	-0.597082	0.07640999	0.23835734	0.87991196	0.00422503
cg06131547	C22orf24;C22orf24;YWHAH	brown	-0.0740594	-0.4310849	0.40189481	0.35882834	0.74120087	0.23706712
cg07427130	UBR4;UBR4;UBR4	brown	-0.0617532	-0.6049803	0.19531	0.3412271	0.94871776	0.15361282
cg08885947	UBE2Q1;UBE2Q1;UBE2Q1;	brown	0.09732196	-0.3830436	0.12083641	0.25302122	0.81649803	0.24647217
cg17300750	RP11-304L19.12	brown	-0.1090713	-0.53358	0.21936324	0.21366195	0.71684676	0.21280379
cg21049809	TTC7B;TTC7B	brown	0.0388487	-0.2161914	0.34875784	0.43323113	0.64160833	0.12933214
cg22911571	RP11-117D22.2	brown	0.22798314	-0.1154777	0.20624528	0.53665032	0.63634846	0.41174253
cg09439920	TBC1D23;TBC1D23;TBC1D	brown	-0.0896876	-0.6189491	0.0655886	0.27348423	0.84428306	0.15805184
cg25772143	AMPD2;AMPD2;AMPD2;A	brown	0.07124737	-0.2989831	0.07172509	0.31625449	0.70359559	0.25814532
cg10840312	SENP8;SENP8;SENP8;MYO	brown	-0.213244	-0.6791881	0.12354304	0.24481107	0.86347824	0.18604024
cg18587247	MAP2K3;MAP2K3;MAP2K	brown	0.20015313	-0.1068079	0.08541977	0.36874098	0.49983987	0.34636377
cg03777083	WNK4	brown	-0.0247428	-0.2277574	0.21019218	0.40131486	0.5157036	0.20736996
cg15735523	RP11-462G12.4;ADCY9	brown	-0.1684858	-0.4030846	0.10843163	0.05656536	0.41988062	0.15049855
cg00143643	RP11-399K21.10;C10orf11	brown	-0.3873078	-0.7975869	-0.0409497	0.04038968	0.80347975	-0.0875795
cg00673191	DOPEY2;DOPEY2;DOPEY	brown	0.00571794	-0.5144252	0.0902057	0.4552505	0.90430564	0.13722569
cg21841085	BAHCC1;BAHCC1	brown	0.05555798	-0.3059699	0.23230951	0.18017473	0.61119493	0.20011317
cg02606535	TSC2;TSC2;TSC2;TSC2;TSC	brown	-0.1301321	-0.4031608	0.29146121	0.04972876	0.38554919	0.27027311
cg11749142	AC007036.5	brown	0.10116021	0.30241565	-0.1968047	-0.2415137	-0.5702026	-0.0553188
cg04934778	AMBRA1;AMBRA1;AMBR	brown	-0.3262235	-0.3230533	0.31936282	0.28555742	0.32080009	0.04578789
cg00983520	CHKB-	brown	-0.2150849	-0.3547336	0.13133078	0.0132301	0.47032569	-0.0130891
cg22116999	SLC9A3R2;SLC9A3R2;SLC	brown	-0.1110358	-0.5807903	0.12373406	0.15730618	0.85216952	0.09846593
cg05920675	RARA	brown	0.08622478	-0.4179143	0.44819045	0.38194445	0.71604803	0.39053916
cg26271776	TRAJ22	brown	0.16990647	0.63290639	-0.0463941	-0.2178115	-0.8166395	-0.0853686

cg01499579	C16orf93;PHKG2;RNF40;C1	brown	-0.029716	-0.4989527	0.11520117	0.43061197	0.87572359	0.16341422
cg20914572	PRRT1;PRRT1;PPT2;PRRT1	brown	-0.3279499	-0.565099	0.0774799	0.22615488	0.64224701	-0.0035302
cg11029830	COTL1;COTL1;COTL1;COT	brown	-0.0245435	-0.5874708	0.16753918	0.29472997	0.90281886	0.20481083
cg10298205	NAPRT1;NAPRT1;NAPRT1	brown	0.10032388	-0.0503905	-0.0837648	0.36209541	0.33036275	0.28485849
cg25225310	PPM1F;LL22NC03-	brown	0.07962847	-0.4829679	0.14082462	0.35431132	0.90276721	0.21394271
cg21515751	FBXO44;FBXO2;FBXO2;F	brown	-0.2984729	-0.5991965	0.29159282	0.10281674	0.51895554	0.06394456
cg27545784	SRCRB4D	brown	-0.2006838	-0.5700858	0.11798313	0.23786166	0.78047884	0.04640905
cg00858400	SLC7A5	brown	-0.1290798	-0.6418835	0.10595404	0.29695287	0.94002852	0.07089647
cg25570328	SULT1C2;SULT1C2;SULT1	brown	-0.0083794	-0.5848929	0.14409848	0.26066543	0.91530401	0.16409057
cg00840370	ANKMY1;ANKMY1;ANK	brown	-0.0430605	-0.3644775	0.31778964	0.29023862	0.58032785	0.22972506
cg09867953	ERN2;ERN2	brown	0.02503857	-0.5422897	0.2424913	0.28318255	0.68884769	0.40101327
cg16642721	AC135048.13;FBXL19;FBX	brown	0.01357379	-0.5387384	0.22019424	0.33461505	0.85421816	0.24858013
cg15250396	PAQR5;PAQR5;PAQR5	brown	0.09642201	-0.4296657	0.3061677	0.38267146	0.83395065	0.32153348
cg21432842	CSF3;CSF3;CSF3;CSF3;CSF	brown	-0.2521149	-0.4961997	0.10588996	0.19787	0.69764988	0.00524913
cg23533927	ABP1;ABP1;ABP1;ABP1;A	brown	0.04025232	-0.385983	0.19332651	0.28347061	0.62171187	0.22535934
cg27601554	C2orf82;C2orf82;C2orf82;C2	brown	-0.0470714	-0.5583565	0.13978082	0.19158006	0.86914854	0.161851
cg24850585	AQP2;AQP2;RP11-	brown	-0.2521468	-0.5527615	0.2459029	0.19706205	0.60530325	0.1473487
cg23532679	RP11-902B17.1;RP11-	brown	0.21458897	0.71282051	-0.0942443	-0.1716981	-0.9201335	-0.0611825
cg12901133	TPM1;TPM1;RP11-	brown	-0.0847116	-0.2571871	0.18644898	0.24601323	0.41745661	0.06601106
cg06177555	SPN;SPN;RP11-368N21.4	brown	-0.0578097	-0.6323795	0.15270191	0.32978901	0.92056013	0.20321206
cg21235950	CD97	brown	-0.3703554	-0.3702803	0.2385764	0.08426247	0.5296462	-0.1123825
cg00762662	OTUD1	brown	-0.317996	-0.509452	0.1884201	0.10857942	0.55535447	-0.0515248
cg01838971	SLC35C1;SLC35C1;SLC35C	brown	0.04794265	-0.5577861	0.17540097	0.399018	0.860781	0.26222928
cg14437986	LY6G6C;C6orf25;C6orf25;C	brown	-0.1565341	-0.4421661	0.21084052	0.12425147	0.46082474	0.22421606
cg19946734	9-Sep	brown	-0.0297428	-0.1346879	0.37962984	0.35198368	0.38622649	0.13831397
cg12513899	DNAH8;RP3-503A6.2	brown	0.02586504	-0.4855796	-0.0123903	0.26998813	0.78841032	0.1441313
cg07590929	ESRP2;ESRP2;ESRP2;ESRP	brown	-0.1809823	-0.6753208	0.17066424	0.26994454	0.7612685	0.0441606
cg06616081	RP11-797A18.4;RP11-	brown	0.02584833	-0.5012714	0.10998596	0.24328679	0.85747919	0.18057147
cg12315466	INPP5D;INPP5D;INPP5D	brown	-0.0134674	-0.5130627	0.26682235	0.346813	0.8493502	0.15711233
cg27239280	CTF1;CTF1;BCL7C;AC1067	brown	-0.1596639	-0.6544937	0.13145576	0.26980673	0.84743664	0.09575577
cg12480180	AC008122.1;C2orf88;C2orf8	brown	-0.0399037	-0.212387	0.02205731	0.2367491	0.37927414	-0.005674
cg26091557	GRHL3;GRHL3;GRHL3;GR	brown	-0.0891946	-0.5327456	0.06609823	0.38981763	0.8692658	0.08007172
cg09874472	RP11-88I18.2;PGM5	brown	-0.0423092	-0.4258069	0.13803266	0.19145953	0.72859801	0.07360987
cg12150100	AMZ1;AMZ1;AMZ1	brown	0.03215741	-0.3776054	0.26989239	0.27739725	0.72209127	0.21841512
cg22065526	INHA;OBSL1;OBSL1;OBSL	brown	-0.0401988	-0.4275338	0.16370488	0.30142233	0.65835141	0.14427481

cg18545771	RP11-	brown	-0.1564949	-0.3715692	0.11297067	0.22579747	0.43678249	-0.0104616
cg16975863	HOPX;HOPX;HOPX;HOPX	brown	-0.022469	-0.1367538	-0.1143629	0.09970505	0.43155842	0.01648382
cg13382769	MAGI2;MAGI2;MAGI2;MA	brown	-0.2573354	-0.7268652	0.10057517	0.10651314	0.79567128	0.09806615
cg15902112	RP1-67M12.2	brown	0.25526194	0.64956177	0.07850754	-0.0917541	-0.8229424	-0.0110994
cg11510892	HSPBP1;HSPBP1	brown	-0.2369462	-0.650164	0.12250304	0.21870734	0.83844472	0.04963106
cg12233679	C17orf61-	brown	-0.2116983	-0.528057	0.32737308	0.17739013	0.56940543	0.08132695
cg18161748	RP5-	brown	-0.2204519	-0.3017629	0.17169282	0.22800561	0.46190617	-0.0264087
cg19807446	MYO1C;MYO1C;MYO1C;	brown	-0.368001	-0.6197443	-0.159332	0.01223563	0.57809251	-0.1554547
cg14449575	HAPLN4	brown	-0.2368624	-0.4622208	0.33345764	0.09230705	0.40083902	0.07764426
cg18244423	C16orf71;ANKS3;ANKS3;C	brown	-0.2209601	-0.7399784	0.12019383	0.22408574	0.88739215	0.05480526
cg06667640	IGLJ5	brown	-0.3319645	-0.4896488	0.17823569	0.04145243	0.63971768	-0.0732109
cg10772169	AGL;AGL;AGL	brown	0.06078621	-0.0981158	0.09939114	0.47751648	0.50321488	0.0961253
cg18383890	ZIC2;ZIC2;ZIC2;ZIC2	brown	-0.2492816	-0.5492793	0.23915543	0.0638411	0.55559488	0.10106912
cg20468003	TRPM2;TRPM2;TRPM2;AP	brown	-0.0461243	-0.3565539	0.27073926	0.26925531	0.66148001	0.23841371
cg02246692	SLC27A3;SLC27A3;SLC27	brown	0.05270517	-0.4019507	0.25460102	0.33632445	0.76642971	0.14106895
cg08917718	PCBP4;PCBP4;PCBP4;PCB	brown	-0.2167366	-0.6878499	0.11087331	0.19837507	0.82509947	0.08574169
cg14694191	AIF1;AIF1;AIF1;AIF1;AIF1;	brown	-0.2348009	-0.7061296	0.08997946	0.21534767	0.90884364	0.05683928
cg04955232	LTC4S;LTC4S;LTC4S;LTC4	brown	0.03123681	-0.5154818	0.37613649	0.36523457	0.79882879	0.28507457
cg06035250	ALPL;ALPL;ALPL	brown	-0.0685391	-0.3408616	0.19017688	0.20420224	0.46527101	0.22198721
cg16425428	TRAF3IP2;TRAF3IP2-	brown	0.03430348	-0.4850763	-0.0090532	0.43045361	0.87815343	0.21143374
cg18581916	RP11-145M4.2;RP11-	brown	-0.0701486	-0.5067287	-0.0263745	0.40930597	0.84560473	0.10441713
cg15371169	PDE4DIP;PDE4DIP;PDE4DI	brown	-0.0880069	-0.5725599	0.04355844	0.20249206	0.78206364	0.17334569
cg05831191	HSF4;HSF4;HSF4;HSF4;HS	brown	-0.0845272	-0.3231862	0.11484084	0.1975204	0.51641057	0.12781414
cg17179427	DOHH;DOHH	brown	-0.1783544	-0.1247593	0.14915739	0.21593834	0.36159845	-0.0751002
cg16167052	AC011484.1	brown	-0.3704629	-0.7836682	0.03131542	0.09451567	0.75017146	-0.0510287
cg16880773	RARRES2;RARRES2;RARR	brown	-0.1609295	-0.3736313	0.0903441	0.19892943	0.47862654	-0.0764064
cg10835266	CTD-	brown	-0.3815547	-0.6091209	0.13325195	0.22782826	0.67409229	-0.124785
cg18804206	FLII;FLII;FLII;FLII;FLII	brown	-0.3960231	-0.314788	0.3268228	0.1771176	0.34930262	-0.0536865
cg10621012	ARAP3;ARAP3	brown	-0.329665	-0.700322	0.05340472	0.18633101	0.80343814	-0.0238785
cg02052217	PILRA;PILRA;PILRA;PILR	brown	-0.0025872	-0.5418172	0.17574634	0.3059344	0.90381663	0.1552375
cg13891189	ALAD;ALAD;ALAD;ALAD	brown	-0.3311105	-0.4446498	0.00145538	0.10612636	0.44003088	-0.0308933
cg19714985	ZNF503;ZNF503-	brown	-0.3040611	-0.669036	0.03018011	0.1335258	0.71594109	0.06291372
cg10256708	PIK3R2	brown	0.23150603	-0.2140768	0.13673562	0.49313129	0.66076944	0.32738572
cg19948393	ANKRD33;ANKRD33;ANK	brown	0.12112122	-0.2663921	0.1450957	0.30230761	0.44745906	0.28688633
cg08031306	PRSS23;PRSS23;PRSS23;P	brown	-0.423719	-0.7043515	0.14081542	0.11693834	0.672433	-0.060446

cg17736772	CLK2;HCN3;HCN3;CLK2	brown	0.0650359	-0.4298741	0.09792712	0.52292236	0.77636539	0.25924916
cg04180462	ACAT1;RP11-	brown	-0.014814	-0.541863	0.10383949	0.35286802	0.92485416	0.12773988
cg08401483	RP11-661G16.2;TTC7B	brown	-0.1329235	-0.1682798	0.17750341	0.37186066	0.4133467	0.04336092
cg22271905	GDPD5;GDPD5;GDPD5;G	brown	-0.3135092	-0.6462337	0.01768496	0.15944807	0.70434844	-0.0097899
cg01529076	FOS;FOS;FOS;FOS;FOS;FO	brown	-0.2040106	-0.5783664	0.1264754	0.13147951	0.56007952	2.07E-05
cg06416491	KIF21A;KIF21A;KIF21A;A	brown	-0.0627149	-0.5680837	0.20404179	0.32825278	0.84693787	0.14054795
cg16457059	CRYAA;CRYAA;CRYAA	brown	0.00688215	-0.5497938	0.24253744	0.28503763	0.90067138	0.26907981
cg16376155	SLC38A11;SLC38A11;SLC3	brown	-0.1501718	-0.3379626	0.27649979	0.26009388	0.4869443	0.2540236
cg08119325	TNK2;TNK2;TNK2;TNK2;T	brown	0.06390418	-0.3580605	0.51329848	0.28968244	0.6423487	0.38318674
cg27523577	TRABD;TRABD;TRABD;T	brown	-0.0855423	-0.3650889	0.08760167	0.16037613	0.51589578	0.19985196
cg26282236	RAD52;RAD52;RAD52;RA	brown	0.06613118	-0.2886286	0.37030525	0.21812006	0.40401307	0.35593102
cg20276367	RP11-800A3.2	brown	-0.0551136	-0.5304289	0.13100819	0.35031902	0.94219923	0.11709446
cg18365622	IQSEC1	brown	-0.1081961	-0.0695489	0.29179679	0.41672091	0.38281097	0.09070493
cg03123666	ATP8B3;ATP8B3;ATP8B3	brown	-0.2477811	-0.5560965	0.12239595	0.13331268	0.62570461	0.0339902
cg09662329	SLC25A34;RP11-288I21.1	brown	0.0887163	-0.4451935	0.21477827	0.44315836	0.80684373	0.34903497
cg12827224	TEC	brown	-0.1774665	-0.6317424	0.02631462	0.17505164	0.85090277	0.08190358
cg11961813	PSME3;AOC2	brown	-0.1701132	-0.5774084	0.08714351	0.19915204	0.78908867	0.04487395
cg14633721	PIK3R5	brown	0.18678028	-0.3560901	0.39678669	0.46888368	0.75264237	0.35213826
cg02198878	GTF3C2;GTF3C2;MPV17	brown	-0.0568009	-0.5456115	-0.0559386	0.33324855	0.89309424	0.09942409
cg19428149	FBXL8;FBXL8;FBXL8;FBX	brown	-0.2931751	-0.7278921	0.2012642	0.13519238	0.73543505	0.07024782
cg03827739	MYOZ1	brown	-0.3312411	-0.6603151	0.00918262	0.11064158	0.75837941	-0.0887846
cg16597728	APCDD1L	brown	0.08909715	0.21746739	-0.136961	-0.1867657	-0.2870228	-0.1172655
cg08659674	LINC00094;LINC00094;LIN	brown	0.01187165	-0.5377648	0.03256164	0.4200624	0.91487653	0.16965091
cg06031796	RP5-881L22.4	brown	-0.0806401	-0.385861	0.07801869	0.48674501	0.70199445	0.14508891
cg14755852	SLC25A37;SLC25A37;SLC2	brown	-0.3271431	-0.3122402	0.36020544	0.14440099	0.40730889	0.03597492
cg08907118	GTF3C1;GTF3C1;GTF3C1	brown	0.00316772	-0.5040374	0.23655221	0.26353874	0.85424318	0.16967648
cg21141410	USP36	brown	0.01656195	-0.5077084	0.06202231	0.18907044	0.86875954	0.23135267
cg13796737	SFI1;SFI1;SFI1;SFI1;SFI1	brown	-0.0444899	-0.3487265	-0.1259004	0.41762011	0.69557806	0.10865602
cg06571407	CD40;CD40;CD40;CD40;CD	brown	-0.2469675	-0.7216632	0.1474411	0.15567995	0.73919944	0.06092738
cg26224280	STAB1;STAB1	brown	0.26463616	-0.1251143	0.45996405	0.46565255	0.55622816	0.4136748
cg26102423	PTK2;PTK2;PTK2;PTK2;PT	brown	-0.2326205	-0.7374503	0.04554737	0.19330783	0.91633054	0.01992048
cg13622642	PPP1R42;PPP1R42;PPP1R4	brown	-0.0268693	-0.3032011	0.24974277	0.2960808	0.56367235	0.03600351
cg12935715	RP11-	brown	-0.0567585	-0.5388734	0.25066172	0.16967507	0.7489528	0.21140302
cg05963597	PTGDS;PTGDS;PTGDS;PT	brown	0.11853946	-0.4067784	0.1690771	0.39621852	0.82927314	0.35808334
cg10648763	TGFBI;TGFBI;TGFBI;TGFB	brown	-0.2699473	-0.6569352	0.18203619	0.19813246	0.85187821	0.00909638

cg20494841	CTA-384D8.31	brown	0.12224788	-0.2330946	0.41259926	0.66047085	0.56713091	0.30689639
cg18477820	H2AFY;H2AFY;CTC-	brown	-0.0398973	-0.5433414	0.12272428	0.34331364	0.89872367	0.09452158
cg06687305	CCDC17;CCDC17;CCDC17;	brown	-0.0925748	-0.1664324	0.13562521	0.15846099	0.31056309	-0.0366264
cg11970192	ST20;AC015871.2;RP11-	brown	-0.3078443	-0.0346247	0.29613773	-0.0179742	-0.242177	0.05978333
cg16338317	AC008537.2	brown	-0.0085069	-0.3751152	0.06546984	0.39233388	0.82567807	0.11010428
cg14682846	FAM200A;FAM200A	brown	-0.3159557	-0.4489714	0.07216073	0.09452017	0.44372573	-0.0425089
cg15352829	PLD4;PLD4;PLD4;PLD4	brown	-0.0852992	-0.6952811	0.10003189	0.16514791	0.86774225	0.22504833
cg07838048	MTSS1L;MTSS1L	brown	-0.0326664	-0.3319005	0.42319794	0.20527259	0.44945484	0.28954162
cg26478990	IRX5;CTD-3032H12.2	brown	-0.281368	-0.7101651	0.1890348	0.15142308	0.66752623	0.0139874
cg04660234	DCAF10;DCAF10;EXOSC3;	brown	-0.3270498	-0.285023	0.11480695	0.27681332	0.30748902	-0.1223947
cg27306451	RBM28	brown	-0.1404405	-0.514662	0.23412414	0.13186909	0.77487061	0.06006103
cg08644381	SSPO;SSPO;SSPO;SSPO;SS	brown	0.03379455	-0.422638	0.23647065	0.19246162	0.7558016	0.27635154
cg04403423	RP4-	brown	0.23830153	0.71226988	-0.0044728	-0.1610462	-0.9331065	-0.0268983
cg20432206	ESRRA;ESRRA;ESRRA	brown	-0.0130745	-0.5127095	-0.0088435	0.32243588	0.88234083	0.14563505
cg05915859	AC013461.1;AC013461.1;A	brown	-0.0170988	-0.4638029	0.2660402	0.33379934	0.82428945	0.08904046
cg23407781	POLR2B;POLR2B;POLR2B	brown	0.09999632	-0.4391228	0.03136669	0.43705593	0.86466404	0.14852376
cg26098517	DUT	brown	-0.1478964	-0.311407	-0.0075095	0.37740447	0.68892783	0.09548517
cg25727366	EPB41;EPB41;EPB41;EPB41	brown	-0.2084863	-0.6214267	0.16244166	0.16200643	0.7249254	0.09670056
cg21707620	RP11-428C6.1	brown	-0.1710882	-0.3166221	0.29509203	0.15471098	0.38895076	0.16064556
cg01211097	USP10;USP10;USP10;USP1	brown	-0.1582697	-0.4991326	0.09015458	0.23090154	0.730798	0.09781317
cg17621438	RNF180;RNF180;RNF180	brown	-0.2061608	-0.3105008	0.16943048	0.1899429	0.44905638	-0.0288535
cg10681065	TFR2;TFR2;TFR2;TFR2;TFR	brown	-0.1109686	-0.5949403	0.12656011	0.2933785	0.85147081	0.11471998
cg14541209	DTX1;DTX1	brown	-0.1289948	-0.5754304	-0.0601968	0.21042264	0.80322601	0.1207585
cg11365623	DNALI1;DNALI1;DNALI1;	brown	-0.206882	-0.4772805	0.2269727	0.04227145	0.46083215	0.10295477
cg14821468	BAI2;BAI2;BAI2;BAI2;BAI	brown	-0.1129219	-0.656537	0.1470217	0.33826333	0.89194299	0.07198955
cg16564156	C1orf106;C1orf106;C1orf106	brown	-0.0191813	-0.5142503	0.12754044	0.3310629	0.88663526	0.12796199
cg11686923	C11orf95	brown	-0.313062	-0.7863441	0.00953938	0.08172696	0.77397631	0.00359062
cg22690327	ICAM2;ICAM2	brown	-0.0907503	-0.6289675	0.13269593	0.30582934	0.9437056	0.15152661
cg22057050	KANSL3;KANSL3;KANSL	brown	-0.2582274	-0.5755159	0.14166904	0.20586983	0.75111271	0.00355433
cg08992189	HK1;HK1;HK1;HK1;HK1;H	brown	0.0797742	-0.3836873	0.15564435	0.52248089	0.78760106	0.21027227
cg06415153	PITPNM2;PITPNM2;PITPN	brown	-0.1708388	-0.6365256	0.12370836	0.17630028	0.87082751	0.02611921
cg02008229	ANPEP;ANPEP	brown	-0.0018523	-0.212147	0.22302769	0.10489905	0.28465334	0.20530714
cg04423314	TFR2;TFR2;TFR2;TFR2	brown	-0.1311669	-0.6138669	0.15200771	0.25204221	0.85177504	0.1309033
cg14572744	RP11-286H14.8	brown	-0.0868187	-0.5669144	0.12088619	0.3000753	0.94361798	0.16611854
cg25374813	SLC23A1;SLC23A1;SLC23	brown	-0.0737184	-0.5069049	0.05773213	0.39746038	0.85979782	0.20705007

cg07906805	ATP6V0B;ATP6V0B;B4GA	brown	0.01417725	-0.5453959	0.18379465	0.32079523	0.90579056	0.21328272
cg08146029	RAPGEF6;RAPGEF6;RAPG	brown	0.13339025	0.29698733	0.4471058	-0.1266002	-0.5217681	0.11763863
cg10732950	MAST2;MAST2	brown	-0.2040601	-0.4205777	0.12890259	0.17956466	0.49395011	0.11298094
cg21088488	DBNL;PGAM2	brown	-0.225624	-0.2679151	0.19822937	0.16230057	0.3379064	0.02716415
cg04067066	WNT10A	brown	-0.2117932	-0.537471	0.07117145	0.13367822	0.64572431	0.05332418
cg01877606	LNX1;LNX1-AS2;LNX1-	brown	-0.1385583	-0.3465994	0.05681562	0.07774232	0.40411828	-0.0695068
cg05046996	SH3BP5;SH3BP5	brown	-0.0282031	-0.3238786	0.27587757	0.34095834	0.69996973	0.18405016
cg00138126	PMEPA1;PMEPA1;PMEPA	brown	0.10058355	-0.3921538	0.14145771	0.42043422	0.83165101	0.22475926
cg24721049	PDE4DIP;U2	brown	0.00295805	-0.5078129	0.01496006	0.30769555	0.89915548	0.16799905
cg22982930	DIS3L2;AC105461.1;DIS3L2	brown	0.06869717	-0.4762844	0.05358894	0.41503195	0.91031499	0.18004937
cg10160612	RP11-2C24.4;SRCAP	brown	-0.2011208	-0.2857984	0.23799994	0.23863873	0.6451744	0.05598228
cg09368360	SEMA4B;SEMA4B;SEMA4	brown	0.0189172	-0.2966481	0.1415216	0.30523813	0.55851251	0.11051653
cg03289591	SH2D6;SH2D6;SH2D6;SH2	brown	-0.1551226	-0.6756383	0.08470777	0.18974611	0.94981746	0.05837856
cg00611469	PISD;PISD;PISD;PISD;PIS	brown	0.2500107	-0.196287	0.26697308	0.38490356	0.54546129	0.2644013
cg04260224	RGS12;RGS12;RGS12;RGS	brown	-0.0137511	-0.3930304	0.04853491	0.24098672	0.6888702	0.1248279
cg00934848	FAM82A2	brown	0.0865648	-0.4203872	-0.0223451	0.41412995	0.83173439	0.16295641
cg14230378	NUDT16P;NUDT16P;RP11-	brown	-0.2400207	-0.4777425	0.07203416	0.04594538	0.48231314	-0.0067823
cg25133988	LITAF	brown	0.2684494	-0.2412548	0.36446296	0.47647412	0.68583928	0.35654249
cg24743838	ARC	brown	-0.3113351	-0.450257	0.0418138	0.17843035	0.60205407	-0.0713715
cg04242898	CYB5R3;CYB5R3;CYB5R3;	brown	0.04573761	-0.218726	0.35488309	0.40707391	0.4462168	0.25543378
cg06983607	CTD-2561F5.1	brown	-0.0830051	-0.053446	0.20829132	0.41650584	0.30298152	0.07337823
cg04801767	SH2B2;SH2B2	brown	0.07552281	-0.2190833	0.00231704	0.15010086	0.60652739	0.18469582
cg10183069	RP11-960L18.1;RP11-	brown	-0.1543987	-0.6963547	0.11812185	0.23160507	0.92797433	0.07630807
cg19461320	DHX37;DHX37	brown	-0.1931296	-0.2732841	0.22813037	0.26130117	0.42744763	0.0291466
cg17406456	FNDC3B;FNDC3B;FNDC3	brown	-0.0904786	-0.5858985	0.09566786	0.3568455	0.94145587	0.14958859
cg00018606	CRYGD	brown	-0.1147785	-0.5694969	0.11763116	0.21272608	0.69424609	0.08418871
cg10502231	DEGS1	brown	0.02934287	-0.5457894	0.38338911	0.28492884	0.68234165	0.51038637
cg04835284	SEC31B;SEC31B;SEC31B;S	brown	-0.1597458	-0.6465133	0.28245765	0.11412402	0.67728749	0.16651012
cg05311589	SIAH1;SIAH1;SIAH1;SIAH	brown	-0.3676228	-0.7179948	0.1010325	0.04964365	0.69485338	-0.045682
cg07363637	SLC44A4;SLC44A4;SLC44	brown	0.11357933	-0.250731	0.31594566	0.4960052	0.49240503	0.30356802
cg11845417	C11orf52;HSPB2-	brown	0.27632498	-0.1370165	0.06825671	0.29066619	0.42369499	0.33843502
cg08587504	RP4-550H1.6	brown	0.04621858	-0.5455023	0.18813753	0.39737178	0.86450924	0.31820625
cg18223939	NAGLU;NAGLU	brown	-0.0808596	-0.5228728	0.1523373	0.30852986	0.85864032	0.12137511
cg12776156	TSPAN4;TSPAN4;TSPAN4;	brown	-0.5011672	0.02006944	0.19935796	-0.0244652	-0.2390459	-0.1991202
cg20777437	CDCP2;RP11-	brown	0.03722581	-0.4793156	0.18504218	0.24469143	0.86421935	0.22260638

cg12423658	CARS2;CARS2;CARS2;CA	brown	0.02306157	-0.5631982	0.22200702	0.37935379	0.92818102	0.24966717
cg11339849	SLMAP;SLMAP;SLMAP;SL	brown	-0.0714128	-0.2748998	0.21363815	0.19931936	0.3178797	0.2803537
cg05428298	ZC2HC1C;ZC2HC1C;ZC2H	brown	-0.3652129	-0.3709421	0.06920598	0.12336035	0.43873289	-0.1591296
cg08739715	CELF6;CELF6;RP11-	brown	0.03177954	-0.3082712	0.39732695	0.28442603	0.5809668	0.21950354
cg11398517	GTSF1L;GTSF1L;GTSF1L	brown	0.18894159	-0.1332325	0.33985075	0.71717709	0.44679369	0.40131584
cg21921474	C1orf172;C1orf172	brown	0.01720964	-0.1391243	0.38464917	0.22635811	0.33176602	0.1706249
cg23008153	RNF180;RNF180;RNF180	brown	-0.1642353	-0.3049462	0.36917989	0.09804932	0.33733269	0.05271056
cg10513437	NPM1;NPM1;NPM1;NPM1;	brown	-0.3707926	-0.5879726	0.19773768	0.07116538	0.60622924	-0.0205246
cg26076905	PIK3R1;PIK3R1;PIK3R1;PI	brown	0.0115384	-0.5617044	0.13846547	0.33793469	0.93706063	0.22278525
cg21206949	IFI27L2;IFI27L2;IFI27L2;IFI	brown	-0.4071761	-0.5494162	0.12368091	0.03669664	0.5089894	-0.1479299
cg06207201	RP11-401P9.5;RP11-	brown	-0.1028507	-0.2741565	0.27253508	0.23817488	0.38964918	0.17310142
cg02405476	UBE2C;UBE2C;UBE2C;UB	brown	-0.2194567	-0.0156791	0.37461619	0.10436146	0.04294579	0.04371583
cg14445076	DHH	brown	-0.2985755	-0.5636171	0.31618828	0.12045637	0.54614053	0.08858012
cg03529003	PLOD1;PLOD1;PLOD1;PLO	brown	-0.2593753	-0.7385874	0.10163059	0.04546256	0.77851361	0.00571974
cg05822017	GPR97;GPR97;GPR97;GPR	brown	0.25670705	-0.2474279	0.36167376	0.45836995	0.75043417	0.28400254
cg04953218	RP1-206D15.3;RP1-	brown	-0.2052632	-0.6333749	0.07191232	0.20210226	0.66322773	0.15069357
cg10860819	LRRK2;LRRK2	brown	-0.1562085	-0.4610198	0.11014944	0.26662199	0.70599632	0.04402846
cg00339657	CBWD2;CBWD2;CBWD2;C	brown	-0.1250977	-0.4154768	-0.0247992	0.24423339	0.59246827	0.21152362
cg22334188	ENOX1	brown	-0.3361631	-0.5753351	0.13447912	0.19229048	0.61725395	-0.1250724
cg00357971	COL20A1;COL20A1	brown	0.04254342	-0.4693612	0.18763745	0.44235518	0.89221811	0.24369428
cg22706883	RP11-	brown	-0.0710317	-0.5489676	0.09373841	0.27901736	0.89266952	0.1849283
cg23389013	SLC11A2;SLC11A2	brown	0.05660109	-0.1406228	0.01080711	0.43829304	0.39771544	0.04636845
cg22057874	OSBPL6;OSBPL6	brown	-0.0313777	-0.5761483	0.09585575	0.34612297	0.84868596	0.18328279
cg25441645	TIGD6;TIGD6;HMGXB3;TI	brown	-0.3467164	-0.3440573	0.17535797	0.03427168	0.28639731	0.05774483
cg01808739	FNBP1L;FNBP1L;FNBP1L	brown	-0.2311835	-0.6674117	-0.0342946	0.15421126	0.78572871	0.00976219
cg19731777	RP11-613D13.4;RP11-	brown	-0.1386228	-0.32276	0.157428	0.28611134	0.75989507	0.05566884
cg02749265	ZNF503;ZNF503-	brown	-0.0646123	-0.5373023	0.06597561	0.25461276	0.85239951	0.19466155
cg26539301	RP11-	brown	0.10949378	-0.4786735	0.22647107	0.41689568	0.88859561	0.24674606
cg24645513	RP11-610O8.1;RP11-	brown	-0.2171249	-0.2422624	0.10327771	0.29696643	0.45248914	-0.0832234
cg08220966	SNCG;SNCG;SNCG;MMR	brown	-0.0758809	-0.4731284	0.23481065	0.23515476	0.77228168	0.25063218
cg18105842	FGF11;RP11-	brown	-0.1483321	-0.7479053	0.1406599	0.21961466	0.89065089	0.17285619
cg23839110	AIF1L;AIF1L;AIF1L	brown	0.14511157	-0.1846003	0.31777229	0.39220707	0.65041913	0.29171151
cg03446675	TDRD7;TDRD7	brown	-0.0414477	-0.1637184	0.42696594	0.3325444	0.28098275	0.31241053
cg21971539	CTAGE5;CTAGE5;CTAGE5	brown	-0.2341537	-0.7258843	0.13440965	0.14354406	0.80678417	0.01500984
cg23666831	XKR7	brown	-0.3297365	-0.4830143	0.13351399	0.2508152	0.50741632	0.05685446

cg13445592	GTF2A1;GTF2A1;GTF2A1;	brown	-0.2253816	-0.6744527	0.18763273	0.10878896	0.65645403	0.08859597
cg08170787	RUSC1-AS1;RUSC1-	brown	-0.2837576	-0.7814216	0.09377301	0.06542461	0.72472401	0.01873991
cg03446165	RP11-473M20.7;RP11-	brown	0.25426266	-0.2280951	0.42867153	0.45109274	0.6425852	0.34340526
cg19644371	RP11-507B12.1;RP11-	brown	-0.3674418	-0.5179592	0.16854225	0.0275191	0.56455925	-0.0499661
cg01226614	SERP2;SERP2;SERP2	brown	-0.0034641	-0.272373	0.43416508	0.29574458	0.51097442	0.13938556
cg25725541	SPIRE1;SPIRE1	brown	-0.0381595	-0.5443761	0.06584032	0.39807685	0.90513894	0.15317817
cg07990412	NLRC5;NLRC5;RP11-	brown	-0.0002469	-0.4682305	0.22940687	0.2560139	0.86212515	0.18888852
cg20802392	CTSK;CTSK;CTSK	brown	0.21689743	-0.2543031	0.1431948	0.61057619	0.74353727	0.26906026
cg04972099	AC087491.2	brown	0.08180334	-0.3543371	0.10125539	0.44929596	0.81380909	0.18212729
cg05098339	EML3;ROM1;ROM1;EML3;	brown	0.02006543	-0.5019013	0.08338977	0.36803528	0.86568167	0.19870414
cg03198372	KREMEN1;KREMEN1;KRE	brown	-0.426376	-0.6389041	0.16179863	0.08561769	0.59804655	-0.0359756
cg12007977	AC009133.20;AC009133.12;	brown	-0.0100302	-0.5401989	0.12574018	0.2207455	0.91170264	0.1659815
cg13923903	ZNRF3;ZNRF3	brown	-0.1320294	-0.5514412	0.1047591	0.28168503	0.77692126	-0.0196671
cg15070180	C9orf117;C9orf117;C9orf117	brown	0.010065	-0.5627777	0.20109209	0.3357742	0.89930721	0.23241787
cg11210138	MIR1203	brown	0.04629659	-0.479865	0.14389978	0.20950576	0.80198997	0.26278943
cg11350680	CST8;CST8	brown	0.11084412	0.22825477	-0.2003926	-0.2245369	-0.3162038	-0.0771096
cg01838759	NISCH	brown	0.03947195	-0.5455672	0.07237866	0.35771985	0.93311984	0.17491723
cg13603551	ABP1;ABP1;ABP1;ABP1;A	brown	-0.0257901	-0.491069	0.21337638	0.34707797	0.78291223	0.15387692
cg23108821	ZDHHC18;ZDHHC18	brown	-0.0232019	-0.5692019	0.12366453	0.36647652	0.94196044	0.16650808
cg18520119	BBS1;BBS1;BBS1;BBS1;BB	brown	-0.1172459	-0.5252322	0.14334059	0.3263847	0.86748735	0.133145
cg21183502	FAM217B;PPP1R3D;FAM2	brown	-0.1333333	-0.5264181	0.25385371	0.18929095	0.64238264	0.12736012
cg16503753	SYNE2;SYNE2;SYNE2;SY	brown	-0.2245702	-0.7181029	0.04000975	0.13770798	0.8231714	-0.0829672
cg10894072	EPS15;EPS15;EPS15;EPS15;	brown	-0.3815312	-0.2418442	0.17961105	-0.0190159	0.2749418	-0.1092339
cg24334037	Y_RNA;PI16	brown	-0.0498359	-0.1745404	0.14678716	0.15547861	0.38076369	0.09269777
cg08787988	IFT172;IFT172;IFT172;IFT1	brown	0.0185942	-0.1356511	0.20611667	0.3826745	0.31278757	0.21390428
cg01887468	TOR1AIP2;TOR1AIP2;TOR	brown	-0.1808991	-0.1157235	0.07199927	0.15788097	0.36487191	-0.0947606
cg01126560	C9orf142;C9orf142;C9orf142	brown	-0.1542966	-0.2908277	0.17197133	0.24964335	0.35121889	0.24460253
cg27462081	Z95152.1	brown	0.26712715	-0.1297324	0.27772669	0.72757275	0.55882689	0.40810672
cg01637548	MIR647	brown	0.13869306	-0.2484051	0.24149399	0.41373007	0.62533761	0.33845981
cg05380077	LGALS12;LGALS12	brown	-0.0560974	-0.4746192	0.21337728	0.48443635	0.76691975	0.11424838
cg12845808	PCDH12;PCDH12;PCDH12;	brown	-0.0794639	-0.5546767	0.10882739	0.21254456	0.891288	0.14595252
cg21966631	RNF123;RNF123;RNF123;R	brown	0.02855014	-0.4142994	0.16117785	0.14689805	0.64960604	0.2314731
cg25460716	RP11-34F13.3;RP11-34F13.3	brown	-0.1840196	-0.4707749	-0.1296271	0.14707234	0.61380488	0.03473487
cg27232284	TRAJ21;TRAJ20	brown	0.1764916	0.43757975	0.33976567	-0.0836632	-0.5061426	-0.0378018
cg01058983	TBC1D5;TBC1D5;TBC1D5;	brown	0.09163052	-0.0676788	-0.4587966	-0.0345763	0.19281215	-0.1665242

cg27518976	ID3;ID3;ID3	brown	-0.0037629	-0.5606696	-0.060549	0.2216275	0.87751442	0.1531867
cg16734549	ARHGAP27;ARHGAP27;A	brown	0.02161942	-0.5537933	-0.0158454	0.34005012	0.88484629	0.14164115
cg03461243	CLCC1;CLCC1	brown	0.02885791	-0.5125357	0.10648281	0.41440617	0.91672873	0.20323254
cg13973063	TMEM91;TMEM91;TMEM9	brown	-0.2552189	-0.5937691	0.27988923	0.16806244	0.62114545	0.06454137
cg03266686	AC138655.6;MSH2	brown	-0.2025924	-0.6385356	0.06928608	0.10910315	0.74424068	0.0398324
cg00949930	CCDC141;CCDC141;CCDC	brown	0.09060181	-0.4704665	0.15743409	0.45844767	0.87471574	0.26218778
cg14131691	CCM2	brown	0.11994183	-0.1886447	-0.0316302	0.65424262	0.56116874	0.28354535
cg06264679	PRRT1;PRRT1;PPT2;PPT2;P	brown	-0.155502	-0.5637572	-0.0162765	0.2149584	0.78930911	0.07763757
cg01524697	SPARC;SPARC;SPARC;SP	brown	-0.218798	-0.5216879	0.0928978	0.13977553	0.52503592	0.04115559
cg17241404	PELP1;PELP1;PELP1;AC091	brown	0.04469518	-0.3709592	0.00630449	0.51713102	0.70691012	0.10617977
cg13287932	HSF1;HSF1;HSF1;HSF1;HS	brown	0.03765408	-0.0243689	0.30767538	0.29447152	0.27772361	0.07948853
cg19358359	MPP2;MPP2;MPP2;MPP2;	brown	-0.2589807	-0.3003616	0.20573593	0.14604577	0.35174554	0.03968984
cg08695652	PIK3R1;PIK3R1;PIK3R1;PI	brown	0.04209381	-0.5322305	0.00148104	0.35822379	0.91499488	0.17321435
cg17427615	C19orf71;MFSD12	brown	0.03334332	-0.3848899	0.31807533	0.52690645	0.72767849	0.19928907
cg11072113	VWCE;VWCE;VWCE;VWC	brown	-0.2227348	-0.7000566	0.23162216	0.18364839	0.78653446	0.0828435
cg06556337	POR;POR;POR;POR	brown	-0.0677378	-0.3863108	0.34081215	0.42305767	0.61720831	0.2628386
cg14629755	VMO1;VMO1;VMO1;VMO	brown	-0.121605	-0.496148	0.09141966	0.14275305	0.53740297	0.0945941
cg04606779	C4orf49;NDUFC1	brown	-0.002799	-0.4423765	0.09619761	0.34360009	0.70515801	0.18721128
cg16163419	ABI3BP;ABI3BP;ABI3BP;A	brown	-0.0873462	-0.2181208	0.00869235	0.28654463	0.39948058	0.09284522
cg00125205	DTHD1	brown	0.05032044	-0.2032386	0.01090771	0.18918067	0.44768777	0.15046465
cg06721796	TP53TG5;TP53TG5;SYS1-	brown	-0.3068349	-0.7822161	0.1420198	0.06548264	0.75500977	0.02343819
cg08372346	RNASE1;RNASE1;RNASE1	brown	0.10528785	0.49184827	-0.1019823	-0.2046597	-0.6030905	-0.1954341
cg26601073	TLCD2	brown	-0.1942894	-0.7431755	0.09439669	0.13794536	0.79296953	0.08923271
cg16654676	RP11-	brown	-0.1626756	-0.146636	0.07983326	0.37909242	0.41174372	-0.0381474
cg25487976	ZC2HC1C;ZC2HC1C;ZC2H	brown	-0.4089776	-0.4375325	0.0180552	0.13138177	0.472166	-0.2252353
cg14153737	FAM78B;FAM78B;FAM78	brown	-0.3457028	-0.6774981	0.17568318	0.09762487	0.67463898	-0.0068238
cg04675542	ZNF300;ZNF300;ZNF300;Z	brown	-0.1525929	-0.6443746	0.18160562	0.10067647	0.66571099	0.0429665
cg12866859	HEXIM1	brown	0.11512407	-0.2191362	0.40380006	0.60557522	0.56385292	0.32450385
cg27211582	C1orf53;C1orf53	brown	-0.2960499	-0.4348312	0.06942011	0.08896905	0.46586208	0.09263935
cg14801864	BFSP1;BFSP1;BFSP1;BFSP	brown	-0.036284	-0.5579749	0.08769126	0.31245003	0.94632003	0.11002717
cg03464655	SLC38A11;SLC38A11;SLC3	brown	-0.288232	-0.3944129	0.22781152	0.21875093	0.39467212	0.00626353
cg25478266	CARD10	brown	0.03022788	-0.3894981	0.07962065	0.27097912	0.80614411	0.21230177
cg14002385	RP11-804A23.1	brown	-0.2133314	-0.4488342	0.19861507	0.22868899	0.4471575	0.16891018
cg12321810	PDZD2;PDZD2;PDZD2;PDZ	brown	0.05595238	-0.3541369	0.07943923	0.33539111	0.68123166	0.23555104
cg04999025	ABCC1	brown	0.28587444	-0.2327959	0.21586634	0.48006295	0.61003558	0.30926981

cg15243856	MATN4;MATN4;MATN4;R	brown	-0.2279625	-0.3526666	0.01487251	0.13864022	0.3681793	-0.0683333
cg08088161	HSPBP1;HSPBP1	brown	-0.2257847	-0.7035061	0.21435788	0.16965243	0.77246347	0.1523669
cg15302379	KAZALD1;KAZALD1;KAZ	brown	0.13000617	-0.2090624	0.37913129	0.34715534	0.476071	0.27897781
cg12214913	ADH7;ADH7;ADH7;ADH7	brown	0.48160981	0.07247535	-0.1694345	0.26948637	0.428203	0.04134279
cg05818394	HMGB1;HMGB1;HMGB1;	brown	-0.3672336	-0.3840127	0.12264267	0.15084128	0.49707368	-0.0441115
cg18836745	C1QTNF5;C1QTNF5;C1QT	brown	-0.3073978	-0.5160676	0.11917961	0.06305316	0.57023213	-0.1095972
cg00927035	SLC35C1;SLC35C1;SLC35C	brown	-0.097561	-0.675854	0.18616655	0.30338746	0.86830984	0.11612538
cg12291409	CTD-2199O4.3	brown	0.02854566	0.46755433	-0.1788461	-0.1475802	-0.4777208	-0.163114
cg06178315	CEP104;CEP104;CEP104;CE	brown	-0.1079925	-0.3998534	0.3319596	0.27588767	0.50227549	0.28229599
cg17925084	KLC1;KLC1;KLC1;KLC1;K	brown	0.07839188	-0.415533	0.12303203	0.3815119	0.76413937	0.19699436
cg00552704	PRRT1;PRRT1;PPT2;PPT2;P	brown	-0.2516946	-0.6494274	-0.0163716	0.14502315	0.69732895	0.01527309
cg23677648	RP11-	brown	-0.0779146	-0.5950138	0.01870842	0.25236884	0.95230033	0.09471655
cg16865151	ZCWPW1;ZCWPW1	brown	0.05423586	-0.4017681	-0.1302192	0.29254113	0.76157551	0.18872326
cg14765834	YBX2;YBX2;YBX2;YBX2;	brown	0.18668449	-0.036626	0.24027723	0.25536613	0.33121067	0.39044028
cg01554474	SLC50A1;SLC50A1;SLC50	brown	-0.2738581	-0.1948695	0.14957171	0.17338037	0.43476424	0.00109314
cg12151339	AC004448.2	brown	-0.1125533	-0.583654	0.06125856	0.25620377	0.9048228	0.06719289
cg14159011	RP1-	brown	-0.1349448	-0.4704876	0.23265365	0.199498	0.57063499	0.18615777
cg27177335	BLM;BLM;BLM;BLM	brown	0.00161242	-0.530267	0.10513292	0.32177618	0.90745935	0.16615448
cg13247329	ZMYND8	brown	0.02455579	-0.5081237	0.15426597	0.3060849	0.90297027	0.14730014
cg00156427	THY1;THY1;THY1;THY1;T	brown	-0.1708886	-0.2530763	0.21346446	0.13852554	0.36908599	-0.0136038
cg22120682	CA13;CA13;RP11-	brown	0.09786457	-0.3519741	0.09026629	0.39677435	0.82716813	0.23938073
cg10250238	RP11-	brown	-0.2495393	-0.6974626	0.20193848	0.14669547	0.74282008	0.14062908
cg02909372	CUEDC2;CUEDC2;CUEDC	brown	0.00194522	-0.392755	0.05958675	0.26984757	0.59676004	0.18049717
cg16536918	AVP	brown	-0.1041676	-0.4728737	0.24419214	0.22598423	0.61736039	0.21631529
cg17349289	PNPLA1;PNPLA1	brown	0.22621574	-0.0429678	0.19094167	0.70493277	0.51380867	0.2601842
cg13269555	KAZALD1;KAZALD1;KAZ	brown	-0.0706398	-0.5369257	0.18595678	0.28099754	0.73816517	0.10221965
cg21716735	ITGB1;RP11-462L8.1;RP11-	brown	-0.1642444	-0.4404889	0.27849216	0.1527747	0.42393832	0.27938874
cg22741960	RP11-557C18.3	brown	-0.0372202	-0.431814	0.08584684	0.25880228	0.8380028	0.07023986
cg01235057	NACC2	brown	-0.2740281	-0.310911	0.11945014	0.25591075	0.5134383	-0.0453344
cg14844551	CTC-	brown	0.09619576	-0.0798726	0.22264044	0.27411283	0.32957877	0.18702532
cg23582644	SERPINB2;SERPINB2;SER	brown	-0.025147	-0.5234452	0.14603135	0.38404777	0.8793313	0.17902598
cg05511978	ANKFY1;ANKFY1;ANKF	brown	0.09564729	-0.4244326	-0.0245177	0.37697271	0.87752157	0.11609336
cg12124543	PTPRJ;PTPRJ;PTPRJ;PTPRJ	brown	-0.1653771	-0.5466897	0.2431	0.22536039	0.83384016	0.06791645
cg24380653	ALOX5AP	brown	0.26216534	-0.1784718	0.10977132	0.70843067	0.59384303	0.3693302
cg00950497	ABLIM1	brown	0.08404905	-0.2207467	-0.0375819	0.26069543	0.54674076	0.07534654

cg02743878	DHRS9;DHRS9;DHRS9;DH	brown	-0.2295977	-0.7322768	0.00504986	0.12829971	0.86915562	0.02046645
cg11836829	NENF;NENF;NENF;NENF	brown	-0.2822327	-0.2211237	0.22119512	0.32174547	0.35944941	-0.0052199
cg26645700	MTMR11;MTMR11;MTMR	brown	0.03269965	-0.3249212	0.00737697	0.14912059	0.45536945	0.10951102
cg22804631	RP11-65M17.3	brown	0.06074804	-0.3176215	0.24132506	0.17931108	0.63185288	0.25927418
cg01802062	AC009133.20;AC009133.12;	brown	0.14570824	0.4847554	-0.2058803	-0.1267058	-0.4611613	-0.188912
cg18511205	EME1;EME1;EME1;EME1;E	brown	0.14979978	-0.0518685	-0.1319525	0.56987979	0.5644352	0.21054333
cg12133554	ZNF732	brown	-0.1227493	-0.5835416	0.31908209	0.09849675	0.60726873	0.29757335
cg11783264	SCAMP4;SCAMP4;SCAMP	brown	-0.4006361	-0.353928	0.10675094	0.1634067	0.35725588	-0.1457179
cg24779941	CDC42BPB	brown	-0.1820115	-0.4802473	-0.214066	0.20602218	0.560182	0.0385703
cg14558573	CAMK1;CAMK1;CAMK1	brown	-0.3864007	-0.389102	0.0997148	0.13862278	0.36305533	-0.033293
cg17461972	MADD;MADD;NR1H3;NR1	brown	0.00128811	-0.4896957	0.19967095	0.38545709	0.86774247	0.22449233
cg20227495	NEXN-	brown	-0.2202937	-0.7301174	0.11926138	0.10929116	0.82997516	0.02219153
cg00807990	GNB5;GNB5;RP11-	brown	0.13022285	-0.4236824	0.01693418	0.34770731	0.83451434	0.24599761
cg23512275	AP001053.11	brown	-0.2410879	-0.3459121	0.048581	0.22030015	0.54545655	0.01054201
cg02420128	ST5;ST5;ST5;ST5;ST5;ST5;S	brown	-0.1907477	-0.3467793	0.09898614	0.10749891	0.4177001	0.09600656
cg27186528	LAX1	brown	-0.1432763	-0.2033406	0.40464265	0.34437802	0.39696073	-0.0199526
cg19055411	U6	brown	0.02688181	0.27850189	-0.048511	-0.2244843	-0.3484116	-0.0955081
cg09993210	PDE2A;PDE2A;PDE2A;PDE	brown	0.00091005	0.0017986	0.20948179	0.36525381	0.36397518	0.29837048
cg12080306	YPEL2	brown	0.06935693	-0.4508329	0.10184486	0.36511078	0.88351966	0.21050152
cg12350036	CREBBP;CREBBP	brown	0.08096185	-0.3931409	-0.1075474	0.32778358	0.79662218	0.19718356
cg25183896	ADAMTSL5;ADAMTSL5	brown	-0.1883027	-0.2642807	0.27706475	0.1169637	0.29326864	0.06812335
cg01995099	SEPT3;SEPT3;WBP2NL;WB	brown	0.21249386	-0.3185607	0.37039892	0.48194459	0.71035217	0.33888127
cg06743312	IFFO1;IFFO1;IFFO1;IFFO1	brown	0.00067912	-0.3701189	0.25498397	0.29963808	0.70280189	0.21538119
cg16251783	CELSR1	brown	0.05661295	0.10878605	0.517658	0.29415927	0.23990334	0.274814
cg03547797	GAS2;GAS2;GAS2;GAS2;G	brown	-0.1823384	-0.4111593	0.17765668	0.28915287	0.66824436	0.12014801
cg24394172	GABRB1;GABRA4	brown	-0.2830607	-0.7684579	0.1811264	0.14834815	0.80725735	0.08120547
cg27108109	HOPX;HOPX;HOPX;HOPX	brown	0.05638998	-0.3446459	-0.061631	0.30879916	0.70163383	0.11504899
cg17085171	RP11-	brown	0.02197105	-0.2724427	0.21062458	0.52565589	0.66245002	0.09093602
cg18950215	ABI2;ABI2;ABI2;ABI2;ABI	brown	-0.0901734	-0.5566178	0.04633235	0.24400551	0.79517922	0.01432922
cg23441018	LPCAT4;LPCAT4;LPCAT4;	brown	0.210188	0.58975588	0.13463399	-0.0220877	-0.6279512	0.04195009
cg27408085	BAI2;BAI2;BAI2;BAI2;BAI	brown	-0.1055239	-0.5366922	0.05882376	0.31683602	0.83424929	0.1057486
cg18693704	CMTM4	brown	-0.2715395	-0.3946501	0.30620224	0.17430876	0.4574637	0.12401107
cg04702647	CLSTN3;CLSTN3	brown	0.03303561	-0.4126942	-0.0903651	0.47956363	0.73959492	0.22094084
cg07134608	DHDH;DHDH;DHDH;DHD	brown	-0.26266	-0.332593	0.34274077	0.1362506	0.31057176	0.16872748
cg20855448	FHAD1;RP3-	brown	0.10365535	-0.3975865	0.24844112	0.41242754	0.79537697	0.34691829

cg02212301	RP11-557C18.3	brown	0.01833194	-0.3690174	0.0551303	0.28185247	0.76648979	0.08328513
cg14587050	CCDC162P;Y_RNA	brown	-0.224321	-0.7172828	0.09729114	0.12999306	0.86704085	0.08063864
cg26465469	GET4;GET4	brown	0.00705408	-0.5161871	0.18204705	0.41907835	0.86784134	0.19752204
cg12902206	DNAH1;DNAH1;DNAH1	brown	-0.1317994	-0.2840864	0.13950652	0.11062295	0.42330174	-0.0256235
cg04728402	ASRGL1;ASRGL1;ASRGL1	brown	0.06497356	-0.426604	0.14027305	0.42744474	0.80844287	0.20032552
cg05267118	CDKN1C;CDKN1C;CDKN1	brown	-0.1061089	-0.3044192	0.3781602	0.20838905	0.51681901	0.25480001
cg03208691	RP11-	brown	-0.3414421	-0.3334141	0.10340625	0.1441435	0.47095161	-0.0721078
cg15249796	U1;TEX14	brown	-0.2017572	-0.3223738	0.167304	0.25551176	0.39313649	0.0253415
cg08527669	PTGDR2;RP11-804A23.4	brown	0.17529984	-0.1532534	0.05374077	0.72662996	0.6415495	0.27467648
cg12718884	RP11-	brown	-0.0130371	-0.4022686	-0.0355411	0.17754554	0.69306211	0.23419785
cg07658036	PDIA5;PDIA5;PDIA5	brown	0.07933674	-0.4369204	0.17929488	0.35001396	0.84009314	0.20681014
cg10204868	AC140481.8;AC140481.8;FA	brown	-0.2686407	-0.671877	0.17234635	0.08509659	0.66155647	0.07686466
cg13176762	RAB7A;RAB7A;RAB7A;RA	brown	-0.0300736	-0.2020169	0.18250329	0.37850612	0.57369528	0.11023223
cg04494051	CLCF1;CLCF1	brown	0.02061614	-0.4520822	0.29226201	0.38582193	0.85775627	0.19412816
cg27295118	AE000661.37;AE000661.37;	brown	-0.0895865	-0.4169655	-0.0955912	0.11148779	0.59126923	0.07311967
cg13419761	RP11-124D2.6;RP11-	brown	0.03612694	-0.4379598	-0.0200077	0.37886678	0.79433899	0.05991659
cg06159340	CTF1;CTF1;BCL7C;AC1067	brown	0.0369479	-0.3943664	0.09114244	0.3699542	0.79917335	0.16071137
cg11245200	DOC2B;DOC2B	brown	-0.1856506	-0.5623084	0.0627091	0.09890837	0.71079991	0.08983386
cg21644701	FAM65A;FAM65A;FAM65	brown	-0.2807488	-0.0123144	0.22639125	0.0928906	-0.1543148	-0.0798937
cg06882351	CRTAP;CRTAP;CRTAP;CR	brown	0.04049698	-0.5356	0.20887078	0.35394395	0.90153719	0.26963328
cg11874719	LITAF	brown	0.02536049	-0.6262749	0.17013524	0.18846652	0.87262703	0.33406604
cg11235451	RP11-700H6.4	brown	0.06263341	-0.5070877	0.15083823	0.30327185	0.90572915	0.21509365
cg02719051	CRTAM	brown	-0.0250011	-0.478703	-0.0696771	0.25308061	0.75446864	0.10687798
cg16317421	PRAGMIN	brown	-0.1472104	-0.604432	0.1221813	0.22071671	0.79069454	0.02007818
cg08005460	RARA;RARA;RARA	brown	0.01937859	-0.5616283	0.23503548	0.28067601	0.8282967	0.22179332
cg19628958	RP5-965F6.2	brown	-0.0607615	-0.2220899	0.1410753	0.14572665	0.51208745	0.10436457
cg22211173	RP11-	brown	-0.0712084	-0.2778433	0.19813538	0.12230717	0.35421995	0.22795592
cg11154067	RP11-521C20.3	brown	-0.1316748	-0.3942554	0.07019385	0.15771949	0.55300876	0.13832001
cg15827779	CDC42BPB	brown	-0.103295	-0.4707827	0.15779171	0.30621684	0.60369498	0.12301902
cg16899036	HOMER3;HOMER3	brown	-0.2775326	-0.2287899	0.2198552	0.09098873	0.35027519	0.15315955
cg15763565	KAT8;RP11-	brown	0.1132153	-0.3553429	0.1234177	0.59238575	0.79928193	0.18091001
cg22721488	CDC42EP4;CDC42EP4	brown	0.03508467	-0.3811048	0.36481431	0.29986375	0.64728141	0.28917513
cg21122199	C17orf64;C17orf64;C17orf64	brown	0.06126293	-0.4638734	0.19970086	0.31264457	0.82455276	0.15039916
cg12639065	FGFR1;FGFR1;FGFR1;FGF	brown	0.0183595	-0.3761346	0.2185719	0.16421484	0.67225813	0.2132979
cg11970275	COL28A1	brown	0.03897114	-0.0805559	0.10693494	0.21849671	0.31660655	0.22777756

cg16890935	PRDM5;PRDM5;PRDM5;P	brown	0.049513	-0.4424393	-0.0317289	0.31969498	0.76648221	0.1136964
cg04471507	STMN1;STMN1;STMN1;ST	brown	-0.250949	-0.3449852	0.22526869	0.10064832	0.33128516	0.10220347
cg01819281	DALRD3;DALRD3;DALRD	brown	-0.0070576	-0.3490696	0.4086666	0.43928325	0.65040347	0.2654545
cg20661138	AF186192.2;AF186192.2;AF	brown	-0.2734481	-0.7336078	0.07748989	0.07598842	0.67695431	0.04409576
cg10426163	ADAP1;ADAP1;ADAP1	brown	-0.0612781	-0.3910757	-0.0271412	0.09335968	0.51845678	0.03938608
cg10519417	RP11-214O1.1	brown	0.04819496	-0.3916051	0.07885368	0.25180756	0.59976141	0.14828401
cg25923214	MGAT5B;MGAT5B;MGAT	brown	-0.1061235	-0.5411347	0.04513876	0.24918283	0.65077709	0.20093203
cg16892975	IFI27;IFI27;IFI27;IFI27;IFI2	brown	0.09442668	-0.4436155	0.11997202	0.50050144	0.85043083	0.21601017
cg26925253	ZNRF1;ZNRF1;ZNRF1;ZNR	brown	0.18693283	-0.161223	-0.1132665	0.47171471	0.55836396	0.31938136
cg25658983	SIAH3;SIAH3	brown	-0.12131	-0.3730438	0.0014141	0.25169081	0.54707726	0.000429
cg11915001	ZNF844;ZNF844;ZNF844;Z	brown	0.06296621	-0.3035239	-0.0247894	0.38023165	0.69954137	0.0709647
cg08574629	RUSC1-AS1;RUSC1-	brown	-0.2714942	-0.7809192	0.06076146	0.1094064	0.72755059	0.02400013
cg07118372	NEK3;NEK3;NEK3;NEK3;N	brown	0.06863249	-0.4656584	0.05013898	0.37989855	0.86313182	0.2069724
cg11578532	TSTD1;TSTD1;TSTD1;F11R	brown	-0.0054537	-0.1781045	0.1086302	0.27817429	0.40776382	-0.0153852
cg17552742	RP11-461A8.1	brown	0.11513443	-0.3176964	0.35420089	0.33098302	0.62375864	0.35655841
cg00173063	TOP1MT;TOP1MT;TOP1M	brown	0.03485928	-0.1616214	0.40108393	0.46603371	0.26482309	0.19141312
cg01679017	RP11-15M15.2	brown	0.10556119	0.70603049	0.00594463	-0.153617	-0.8386637	-0.0792999
cg25412167	MCM3AP;AP001469.9;MC	brown	0.05958789	-0.3063005	0.58747051	0.37372289	0.50375776	0.40148438
cg23642897	ZDHHC7;ZDHHC7;ZDHHC	brown	-0.0570428	-0.456707	0.10757999	0.32528523	0.69736512	0.17731026
cg08862580	C9orf86;C9orf86	brown	0.14879183	-0.2941706	0.13358953	0.38864084	0.6094442	0.33381082
cg27485334	SPNS3;SPNS3;SPNS3;SPN	brown	0.21371371	-0.3102636	0.08444197	0.50899789	0.80031753	0.21838047
cg15676719	RP3-455J7.4;RP3-	brown	-0.1174988	-0.1500266	0.27591267	0.16683072	0.28864807	0.11038719
cg11523799	LRRC73;YIPF3;YIPF3;YIPF	brown	0.17137882	-0.3422358	0.06601785	0.48785698	0.76670477	0.34523636
cg04948284	LTBP3;LTBP3;LTBP3;LTBP	brown	-0.1946227	-0.5893966	0.23960473	0.06161471	0.63791483	0.09280938
cg07933854	MYO5B	brown	-0.0032759	0.63034551	-0.045983	-0.1818634	-0.6639557	-0.1792459
cg22722028	SV2B;SV2B;SV2B;SV2B;S	brown	-0.1345108	-0.2748649	-0.1013682	0.11036431	0.38403251	0.2136263
cg26622211	OSBPL3	brown	0.01793283	-0.3144314	0.08436262	0.20189023	0.48844523	0.13443059
cg26750913	SGSM3;SGSM3;SGSM3;SG	brown	0.16337566	-0.2600772	0.22541808	0.42108826	0.5788642	0.30128649
cg14271454	VPS37B;VPS37B	brown	-0.0145753	-0.24807	0.09729801	0.16429352	0.4588589	0.28797668
cg02996630	PDLIM7;PDLIM7;PDLIM7;	brown	0.10743335	-0.3231273	0.2859126	0.69623123	0.69576714	0.1889146
cg27481555	LRRC17;LRRC17;LRRC17;F	brown	-0.0416645	-0.3086614	0.16509825	0.18859424	0.39371479	0.11319537
cg03480346	MFSD2B;MFSD2B	brown	0.21044801	-0.2874977	0.13762006	0.54922852	0.69034371	0.24149524
cg17080211	DCPS;DCPS	brown	-0.0920676	-0.6202431	0.30239714	0.20766541	0.66827523	0.32741335
cg01520924	HSPA2;HSPA2;HSPA2;RP1	brown	-0.2450109	-0.5072964	0.21091497	0.17887435	0.58547987	0.04724819
cg07468316	RP11-123B3.2	brown	0.08024614	-0.4107596	0.02941327	0.39697086	0.76950305	0.20033657

cg07204969	ZBTB48;ZBTB48	brown	0.09681753	-0.2834591	0.06885107	0.41114332	0.65097754	0.25712596
cg14180777	GFOD1	brown	0.03450034	-0.5506835	0.12702408	0.37201391	0.94018332	0.18083146
cg20728881	ARID5B	brown	0.08193925	0.67459361	-0.0996211	-0.2591285	-0.9358317	-0.1016332
cg14359787	ANGPTL4;ANGPTL4	brown	-0.055042	-0.3403868	0.23618103	0.22664526	0.41188196	0.22121476
cg20113012	CDC42EP4;CDC42EP4	brown	-0.3334685	-0.6572108	0.08505285	0.16846947	0.73558227	-0.2429871
cg17356255	ZMYND8	brown	0.03680977	-0.5299474	0.14732228	0.37618939	0.952306	0.20156119
cg08055948	FNDC3B;FNDC3B;FNDC3	brown	0.17659954	-0.3143081	0.1166768	0.52408861	0.75965385	0.26637476
cg15896766	HECA	brown	-0.233076	-0.7224292	-0.0030009	0.14528969	0.84119278	0.05215501
cg06146452	ATG7;ATG7;ATG7;ATG7;A	brown	0.06592673	-0.0701224	0.22402346	0.58935601	0.51572152	0.10819269
cg16837662	LINC00205	brown	0.12584064	-0.18993	0.31380006	0.40912505	0.45527164	0.39395802
cg08594100	ZNF821;ZNF821;ZNF821;Z	brown	-0.0654009	-0.5098788	-0.0028016	0.26130081	0.86513517	0.12958575
cg06477069	SOGA1	brown	0.07149596	-0.3740853	0.05031723	0.35069578	0.76072713	0.17245349
cg24603028	SOAT2;SOAT2;SOAT2	brown	0.14976838	-0.1570822	-0.0388521	0.34840885	0.41295835	0.28764627
cg11555438	CARS2;CARS2;CARS2;CA	brown	-0.0551827	-0.2893473	0.18598241	0.19615874	0.36087453	0.12216128
cg04132146	ZNF292	brown	-0.2688904	-0.4416158	-0.0358743	0.14318814	0.48955049	-0.0220212
cg18107425	MESP2;MESP2	brown	-0.1547128	-0.410893	0.40176161	0.21835954	0.45083013	0.13080037
cg11150614	CTD-2337A12.1;CTD-	brown	-0.4451378	-0.5158322	0.11228067	-0.0144015	0.49619909	-0.0623702
cg24201793	MBOAT2;MBOAT2;MBOA	brown	0.08294721	-0.3865009	0.32604193	0.35171591	0.58784437	0.10955128
cg21919809	ETV2;ETV2;ETV2;ETV2;ET	brown	-0.3800491	-0.4190822	0.15089309	0.17885203	0.43131564	-0.0923731
cg09436502	PDK4;AC002451.3;PDK4;P	brown	-0.2311536	-0.7067756	0.02945918	0.14997711	0.78710971	0.07396683
cg15767529	ITGA7;ITGA7;ITGA7;ITGA	brown	-0.0789416	-0.3003936	0.11650302	0.30192792	0.55623183	0.0921692
cg02095589	EPHX4	brown	-0.18533	-0.6603231	0.18958189	0.08812124	0.72964834	0.09109276
cg26597242	GABRA1;GABRA1;GABR	brown	-0.1141792	-0.4517496	0.18243567	0.18413651	0.47125199	0.19678405
cg11172565	PODNL1	brown	-0.0041481	-0.1208516	0.19832846	0.42468969	0.48068321	0.16277577
cg09696536	FCGBP	brown	0.21119522	-0.1853876	-0.06216	0.36494755	0.63263632	0.28114935
cg19889321	VEZT;VEZT;VEZT;VEZT;V	brown	0.10456975	-0.3390832	0.6382442	0.44725627	0.59038564	0.26623593
cg00240229	BTN3A3;BTN3A3;BTN3A3;	brown	0.09589387	-0.4262936	0.06314183	0.41588696	0.7908918	0.18248404
cg21718826	NUDT16L1;NUDT16L1	brown	0.08779737	-0.3762519	0.35927873	0.35535182	0.71358163	0.40086396
cg18291000	GOLGA7;GOLGA7;GOLG	brown	-0.0188175	-0.3119621	-0.0700063	0.33695047	0.47264675	0.11921757
cg05006384	C14orf49	brown	0.07229859	-0.4861451	0.20600022	0.41487645	0.89395378	0.21245661
cg01045458	LATS2;LATS2	brown	-0.1897592	-0.6452754	0.06334223	0.23789693	0.89455692	0.08806914
cg03401519	HOXD-AS1;HOXD-	brown	-0.1525742	-0.4614035	0.11607437	0.04948931	0.42651776	0.18296535
cg06291107	BLCAP;BLCAP;BLCAP	brown	-0.0045436	-0.4778302	0.35373916	0.39889388	0.78273454	0.29797219
cg01095848	ERGIC3;ERGIC3;ERGIC3;E	brown	0.22823451	-0.0289417	0.26390734	0.45285593	0.51265554	0.31487765
cg18654983	RP11-231C14.2;SNX29P2	brown	0.05216824	-0.3571021	0.0573341	0.37997263	0.72451263	0.49291602

cg06290690	TRIM14;TRIM14	brown	0.15371416	-0.29804	0.2205838	0.18434637	0.54821346	0.20350368
cg11972062	MAP2K3;MAP2K3;MAP2K	brown	0.0910026	-0.4197937	0.16860811	0.28841787	0.69721092	0.33725481
cg06004816	HIP1R;HIP1R;HIP1R	brown	0.07953587	-0.443249	0.21017465	0.35348903	0.81006288	0.22829366
cg26534425	IL18;IL18;IL18;IL18;IL18;AP	brown	-0.1795603	-0.6184812	0.06700876	0.17514383	0.70573335	0.02409214
cg04929355	IL18;IL18;IL18;IL18;IL18;AP	brown	0.01581261	-0.3770615	-0.0127383	0.27774552	0.69059527	0.04176665
cg27211576	CSK;CSK;CSK;CSK;CSK;C	brown	-0.0288162	-0.1373895	0.22733484	0.34595451	0.37055331	0.25584789
cg02582500	RP11-65J21.3;RP11-	brown	0.14776208	-0.183182	0.24571482	0.38321414	0.67883598	0.23669637
cg25877847	COL7A1;COL7A1	brown	0.21942222	-0.2487911	0.18030107	0.44393451	0.68512524	0.41177032
cg26271045	LRRFIP1;LRRFIP1	brown	-0.1152239	-0.3983393	0.21991964	0.21171773	0.57569728	0.03996651
cg00969950	FAH;FAH;FAH;FAH	brown	-0.0231897	-0.4475566	0.15258728	0.40221981	0.86691056	0.08866686
cg26363542	KDM6B;KDM6B;KDM6B	brown	0.05882039	-0.5013589	0.09154861	0.38555696	0.88855634	0.1936426
cg04773602	RP11-	brown	-0.2348114	-0.5376467	-0.0772444	0.06397894	0.62424065	-0.0027758
cg20257878	MAP3K14	brown	0.03170484	-0.1359156	0.11094036	0.32093138	0.53895901	0.22998469
cg07514271	ST5;ST5;ST5;ST5;ST5;ST5;S	brown	-0.1234966	-0.5265014	0.10936449	0.27568341	0.8412513	0.04572568
cg05899750	GMCL1;GMCL1	brown	-0.1414925	-0.2188094	0.08163297	0.17300921	0.33463403	0.01234664
cg24679427	ADORA3;ADORA3;ADOR	brown	-0.2194257	-0.719732	0.02245072	0.18328565	0.84728986	0.02317526
cg17703744	PPP1R27;PPP1R27	brown	-0.0370677	-0.0093036	-0.0684056	0.43815513	0.36824091	0.13337315
cg20257143	CTSL1;CTSL1;CTSL1;CTSL	brown	-0.1088544	-0.5746074	0.2785416	0.25093828	0.70180064	0.1086711
cg04638014	ARL9	brown	0.01345569	-0.49956	-0.0239533	0.27182205	0.86187731	0.1430193
cg26355357	ALS2CR11;ALS2CR11;ALS	brown	-0.2168504	-0.442119	0.2216484	0.19218928	0.47691028	0.03574303
cg19219392	IVD;IVD	brown	0.13205608	-0.4465001	0.35483594	0.40311541	0.82135626	0.35597155
cg24349832	TMEM51;TMEM51;TMEM5	brown	-0.1916709	-0.4146603	0.18999925	0.29225639	0.6556814	-0.0087102
cg04126810	RP5-862P8.2	brown	-0.1875612	-0.4842379	0.32990825	0.08525771	0.47121599	0.10283091
cg14647131	ANK3;ANK3;ANK3;ANK3;	brown	-0.1137281	-0.477265	0.01484218	0.08371514	0.49468012	0.15840366
cg13807903	MIS18A-AS1	brown	0.02737711	-0.3850885	-0.0428645	0.24262711	0.74435507	0.08664216
cg07780074	GTDC1;GTDC1	brown	-0.0493607	-0.5407772	-0.0466664	0.26480342	0.91195239	0.07733107
cg06528601	COTL1;COTL1;COTL1;COT	brown	-0.1853982	-0.6744044	0.05428478	0.1363795	0.87739611	0.04917087
cg01359757	CD180	brown	-0.1967104	-0.2847992	-0.1397346	-0.0580327	0.31513402	-0.1032561
cg23947376	LRRC37A6P;RP11-748L13.1	brown	-0.1544229	-0.6433726	0.18254618	0.14548934	0.68445774	0.1078756
cg24776722	RUNX1T1;RUNX1T1;RUN	brown	0.03919771	-0.216464	-0.0512221	0.31490921	0.45060874	0.18406493
cg12550055	UPB1;UPB1;UPB1;C22orf45	brown	-0.2453754	-0.6915606	0.05536814	0.05106378	0.66196563	0.06404245
cg09907758	C11orf95	brown	-0.1447617	-0.6635366	0.13395066	0.22155929	0.80266002	0.07975536
cg19488093	CALD1;CALD1;CALD1;CA	brown	-0.2923115	-0.68191	0.07797999	0.01494137	0.6422251	0.00792298
cg05107803	ZNF625;ZNF625;ZNF625	brown	-0.0967312	-0.4160934	-0.1369676	0.2654185	0.70356449	-0.006003
cg12488919	GATAD2A;GATAD2A;GA	brown	0.0575705	-0.1367244	-0.0609151	0.39452926	0.41367315	0.09177871

cg11316386	C1orf229	brown	0.0862538	-0.3066099	0.25027583	0.35390746	0.63011517	0.33649275
cg04058378	RP11-195F19.5;RP11-	brown	-0.0685427	-0.3402742	0.30938514	0.31326001	0.57313578	0.13968292
cg21800196	SLC26A6;SLC26A6;SLC26	brown	-0.0341744	-0.5694089	0.12899648	0.27849859	0.89576821	0.1668717
cg27490387	U6;HCG11	brown	-0.1177004	-0.307127	0.31105925	0.23057847	0.49710371	0.10214282
cg07769015	SLC45A4;SLC45A4;SLC45	brown	-0.0669732	-0.5835074	0.00403688	0.31936403	0.95113994	0.10259758
cg27037565	DNAJC6;DNAJC6;DNAJC6	brown	-0.0278622	-0.4883741	-0.0195081	0.21790611	0.79304233	0.08747117
cg21439725	C12orf76;C12orf76;C12orf76	brown	-0.1233359	-0.4486903	0.13226022	0.08423375	0.56165537	0.20939664
cg23213217	DEGS1;DEGS1;DEGS1	brown	-0.0214678	-0.2570092	0.32790064	0.32110133	0.45685909	0.20749732
cg03250742	SPON2;SPON2;SPON2;SPO	brown	-0.2923781	-0.0343667	0.07078193	-0.0703356	-0.2037894	0.00615807
cg14221149	WNT5B;WNT5B;WNT5B;W	brown	0.01290588	-0.4122093	0.29497764	0.30203841	0.65128071	0.16593001
cg23637791	ODF3L1	brown	-0.3622406	-0.2436344	0.16645933	0.12809079	0.32061655	-0.0922836
cg24316108	NAALADL1;NAALADL1;N	brown	-0.1914748	-0.5238937	-0.0952799	0.18689368	0.58804451	-0.007642
cg01141721	SRC;SRC;SRC;SRC;SRC;SR	brown	-0.0329637	-0.5884486	0.01966551	0.28975123	0.95463708	0.17263025
cg04140974	RNF144B;RNF144B	brown	-0.282659	-0.6081027	0.26112923	0.14480588	0.61628919	0.01761714
cg23075364	SLC43A1;SLC43A1;SLC43	brown	0.03491121	-0.4607335	0.14360975	0.32040052	0.80505323	0.18052468
cg01128480	DCPS	brown	-0.3028407	-0.3841199	-0.026688	0.04568727	0.36215887	-0.1167856
cg27441872	PSME1;PSME1;PSME1;PS	brown	-0.2022795	-0.411019	0.09987092	0.24130168	0.55645923	0.0433511
cg18505241	STK3;STK3;STK3;STK3	brown	-0.3153472	-0.7374389	0.06237973	-0.0016761	0.6959736	0.02285365
cg09887220	LINC00391	brown	-0.2012068	-0.6574545	0.25828585	0.06596804	0.64927847	0.08135606
cg05544807	DNMT3A;DNMT3A	brown	0.05956432	-0.458391	0.21117199	0.29229444	0.78505973	0.13034715
cg01534416	C19orf48;C19orf48	brown	-0.1771852	-0.2746809	-0.0731311	0.03484831	0.36509566	-0.0201763
cg08624648	LY6G5C;LY6G5C;LY6G5C	brown	-0.2014855	-0.4044103	0.14354738	0.02061629	0.40887954	0.13077171
cg06192618	LIMS2;LIMS2;LIMS2;LIMS	brown	-0.3646229	0.11931767	0.23159065	0.00524809	-0.2250262	-0.0996641
cg02283643	SULF1;SULF1;SULF1	brown	-0.1783375	-0.3855288	0.14245408	0.10657432	0.39296285	0.15218258
cg06846310	STOX2;STOX2	brown	-0.2183793	-0.5048274	0.20201872	0.16066831	0.58947695	0.11310817
cg12806353	PLA1A;PLA1A;PLA1A	brown	-0.0500246	-0.5572195	0.09258402	0.35111226	0.8981527	0.16710593
cg17213154	ST5;ST5;ST5;ST5;ST5;ST5;S	brown	0.09590076	-0.2171412	-0.0173951	0.24832727	0.58592723	0.20153019
cg09921385	TBC1D14;TBC1D14	brown	-0.0778476	-0.5373231	0.07884583	0.14408345	0.86748778	0.08305472
cg04133879	SMAD3;SMAD3	brown	-0.2068546	-0.3855502	0.26828975	0.07637464	0.55602392	0.0842047
cg06068801	PPP1R1B;PPP1R1B;PPP1R1	brown	0.16255826	-0.4003566	0.27452244	0.51069879	0.82701097	0.26902599
cg06493166	PLAGL1;PLAGL1	brown	-0.2053358	-0.275229	0.38803239	0.1165751	0.39126984	0.15834433
cg03085797	C19orf33;YIF1B;YIF1B	brown	-0.0007128	-0.4954471	0.17903356	0.23205041	0.65251018	0.28792399
cg14900031	S100P;S100P;S100P	brown	0.03406697	-0.4759406	0.16000786	0.26954751	0.77249322	0.2362194
cg21941344	CDNF;HSPA14;HSPA14;HS	brown	-0.0671638	-0.5966697	0.05975148	0.35365213	0.82615027	0.12469594
cg05562794	TCL6;TCL6;TCL6;TCL6	brown	-0.1651168	-0.5231928	0.10570943	0.24868737	0.60806994	0.15677442

cg08897150	CCDC81;CCDC81;CCDC81	brown	0.05138059	-0.3728749	0.13906128	0.2858726	0.55905271	0.18156126
cg26335752	WAC;WAC;WAC;WAC	brown	0.00148565	-0.5425133	-0.0315438	0.31532357	0.93667033	0.15747075
cg09388868	KIAA1549	brown	-0.0701822	-0.6119758	0.13472485	0.23550872	0.92125328	0.11760843
cg21012874	MMRN2;MMRN2;SNCG;S	brown	-0.1952863	-0.3412057	0.03255885	0.21589264	0.38711068	-0.0741425
cg24580398	SEPT5;SEPT5;SEPT5;GP1B	brown	-0.3613876	-0.6091907	0.10304091	0.17068508	0.66119423	-0.1046051
cg15056128	SMARCE1;KRT222	brown	-0.019842	-0.4116914	-0.0764089	0.29211674	0.5755499	0.18514503
cg18203859	CYB5R1;CYB5R1;CYB5R1;	brown	0.06914219	-0.410691	0.1720608	0.34915928	0.83704397	0.12976675
cg02237172	ZMYND10;ZMYND10-	brown	0.02970761	-0.3008963	0.24372058	0.27331416	0.64550231	0.31314478
cg14841694	SEMA3F;SEMA3F;SEMA3	brown	-0.2093515	-0.1321612	0.32609188	0.30314542	0.34477623	0.1136823
cg18790187	AE000661.50;AE000661.37;	brown	0.08502871	-0.2079294	-0.0018515	0.46615141	0.61703519	-0.0339086
cg02442623	BACH1;BACH1;BACH1;BA	brown	-0.253167	-0.3871252	0.33023963	0.27007796	0.59724693	0.01522498
cg04289385	RP1-	brown	-0.2184249	-0.5353876	0.13481741	0.08928448	0.53447329	0.06495934
cg07211044	RP11-25K19.1;TOX;RP11-	brown	0.06643383	-0.2140723	0.31153079	0.15476651	0.37544453	0.11982636
cg26845363	FAM159A;FAM159A;FAM	brown	-0.0660038	-0.2092836	0.16820809	0.37061216	0.33079282	0.09165013
cg15591645	EML2	brown	-0.1733878	-0.5546074	0.06956684	0.23180623	0.57449299	0.04628325
cg27383956	CNTD2;CNTD2	brown	0.00390739	-0.2134257	0.37331191	0.2925107	0.28975535	0.23550054
cg00264873	ZADH2	brown	-0.0550642	-0.3067737	0.13315537	0.33237939	0.4404359	0.11355754
cg05550861	C4orf3	brown	-0.3812164	-0.5557704	-0.0018343	-0.0438402	0.50950578	-0.2302516
cg08691338	USP10;USP10;USP10;USP1	brown	0.06773722	-0.3958035	0.164433	0.374002	0.74178047	0.20976406
cg03232933	BTG3;BTG3;BTG3;BTG3	brown	0.16010501	-0.2443292	0.28341903	0.3238167	0.48568196	0.34720834
cg08742424	KLHDC5;KLHDC5;KLHDC	brown	-0.2093166	-0.5125986	0.22122303	0.17290982	0.61417837	-0.0028915
cg01333011	PTHLH;PTHLH;PTHLH;PT	brown	-0.1853238	-0.4533712	0.28592108	0.01262681	0.45169726	-0.0052998
cg22881105	RP11-122M14.1;RP11-	brown	0.16925779	-0.4342364	0.21858324	0.43390466	0.85991431	0.30077873
cg08551218	LRRC73;POLR1C;LRRC73	brown	-0.308687	-0.6814122	0.17373536	0.0959044	0.73391424	0.00404421
cg04942710	MGAT4B;MGAT4B;MGAT	brown	0.13900794	-0.2581598	0.35821306	0.57900029	0.67639986	0.31216524
cg11923320	LINC00466;LINC00466	brown	-0.2453712	-0.6272006	0.31405271	0.16646259	0.60147328	0.00683879
cg18394235	LTBP1;LTBP1;LTBP1;LTBP	brown	0.11683872	-0.3362694	0.03351446	0.56013457	0.76405559	0.19605759
cg07054641	WDR52;WDR52;WDR52;W	brown	-0.1665021	-0.3871628	0.15862244	0.04588034	0.46847	0.01146525
cg02544453	MYLK3;MYLK3;MYLK3	brown	-0.1098775	-0.6159104	0.16223216	0.21439467	0.79972821	0.21251788
cg26601496	NFASC;NFASC;NFASC;NF	brown	0.17309838	-0.3531635	0.17745674	0.36795828	0.76172919	0.40176277
cg24713838	WDR35;WDR35;WDR35;AC	brown	0.15921168	-0.3206859	-0.1665541	0.25527426	0.57396581	0.21786056
cg16495735	AC008074.4	brown	0.12580146	-0.3951155	-0.0042618	0.41879484	0.86226438	0.2164451
cg13310554	ARAP3;ARAP3	brown	0.08648022	-0.3263397	0.41042883	0.47028053	0.70077535	0.33697279
cg21806273	NLRP3	brown	-0.3198651	-0.577289	0.19250311	0.08285094	0.63935516	0.03029678
cg14130039	PRRT1;PRRT1;PRRT1;PRR	brown	-0.2450429	-0.6600048	-0.0510093	0.13281023	0.72520836	-0.0021026

cg07965650	U6	brown	-0.0146208	-0.5359881	0.13740986	0.38136559	0.92048884	0.11396543
cg13219142	TMEM221	brown	0.00725446	-0.3243269	0.04504417	0.18699934	0.71068248	0.13628943
cg02985381	SMAD9;SMAD9;SMAD9	brown	-0.1835446	-0.517949	0.10698693	0.11154471	0.50374885	0.20384901
cg04412116	SLC30A3;SLC30A3;SLC30	brown	0.04967434	-0.3396459	0.46203743	0.39760304	0.553405	0.27526735
cg14348540	SLC39A14;SLC39A14;SLC3	brown	-0.1021676	-0.4212633	0.15221164	0.25024908	0.55115923	0.10058416
cg00404641	NUDT16P;NUDT16P;RP11-	brown	-0.143274	-0.484925	0.08475626	0.10231993	0.57891517	0.02812222
cg09471374	AC007663.1;XXbac-	brown	-0.0031454	-0.4895723	0.21449806	0.3071742	0.85846895	0.19163404
cg10377216	RP11-573D15.8	brown	-0.2200734	-0.7503728	0.07135442	0.13047483	0.87633275	0.03292335
cg09325164	TMEM39B;TMEM39B;TME	brown	-0.3367762	-0.4203752	-0.0073295	0.20400626	0.49020042	-0.092647
cg03830899	ST5;ST5	brown	0.04685751	-0.5097149	0.13828268	0.23068819	0.89735337	0.2041245
cg05292478	SMURF1;SMURF1	brown	-0.3010368	-0.5861281	0.21508785	0.20667722	0.68125484	0.04863528
cg21403066	7SK	brown	-0.1906614	-0.6289385	-0.0211314	0.03978956	0.6110123	-0.0357247
cg08930759	RP11-	brown	-0.0205596	-0.2478175	0.33086261	0.44028323	0.5942695	0.14114078
cg01874808	RBM22	brown	0.04810186	-0.4858502	-0.0073198	0.36180511	0.85313396	0.17659597
cg04221500	CTD-2012K14.1;CTD-	brown	0.26136547	-0.1904729	0.57243506	0.50422202	0.59565067	0.35564132
cg08922698	TGFB3;TGFB3;TGFB3;TGF	brown	0.19451353	-0.2110327	0.12211999	0.74600316	0.63468606	0.22487014
cg14953762	PRSS12	brown	-0.1809599	-0.4098115	0.15166497	0.05613416	0.41881711	0.18162716
cg15616361	PTGDS;PTGDS;PTGDS;PT	brown	0.12842689	-0.2535612	0.13815251	0.36054471	0.70694053	0.33408149
cg12389336	KDM3A;KDM3A;KDM3A;	brown	0.25271997	-0.1518459	0.40564844	0.44992291	0.472879	0.54354649
cg04981000	SYCE1;SYCE1;SYCE1;SYC	brown	0.00700702	-0.1965337	0.19843587	0.08431295	0.30232799	0.22022446
cg24305232	ZDHHC24;ZDHHC24;ZDH	brown	-0.2767194	-0.4328926	0.06008724	0.15667779	0.44588672	-0.0794397
cg01114900	CTNNA1;CTNNA1;CTNNA	brown	-0.285546	-0.6253855	0.20000387	0.17730069	0.65467515	-0.0513448
cg24480540	Y_RNA	brown	-0.2683103	0.14950835	-0.289168	-0.6169208	-0.4377371	-0.2816984
cg13385210	IGFBP4;IGFBP4	brown	-0.0477609	-0.5524922	0.21039181	0.23496254	0.80944665	0.21887867
cg01412886	AHNAK2	brown	-0.406289	0.05554902	0.20338755	0.08004478	-0.1588953	-0.1249129
cg04235361	GRIK4;GRIK4;GRIK4	brown	0.25494614	0.3600112	0.04591115	-0.11436	-0.3694587	0.00767904
cg16920250	TBC1D23;TBC1D23;TBC1D	brown	-0.0502891	-0.5725717	0.07596366	0.28593977	0.86418168	0.15176555
cg12347471	PADI2	brown	0.11638414	-0.3211934	-0.0813208	0.25341392	0.75689064	0.13338027
cg03870394	ITGAV;ITGAV;ITGAV	brown	-0.1142827	-0.5598772	0.14761296	0.21972474	0.78983362	0.14829733
cg10468070	RP11-231P20.2;RP11-	brown	-0.3264859	-0.4740453	0.22698064	0.13995348	0.4535776	-0.0207533
cg23710031	DCTN2;DCTN2;DCTN2;DC	brown	0.19800555	-0.234009	0.01960221	0.44873557	0.66255401	0.27982846
cg11183227	MAN2A2;MAN2A2;MAN2	brown	0.26702832	-0.3262348	0.20073831	0.59980798	0.70249152	0.5052734
cg04751968	CEP72;CEP72;RP11-310P5.1	brown	-0.1439826	-0.3346181	0.41025903	0.23264085	0.36260653	0.11681513
cg14273485	SPECC1;SPECC1;SPECC1	brown	-0.1108154	-0.659473	0.09677347	0.16372675	0.90497699	0.05264965
cg17616192	GPS1;GPS1;GPS1	brown	-0.006415	-0.4457855	0.18908389	0.28044421	0.76988875	0.24629971

cg02115865	FAM19A2;FAM19A2;FAM	brown	0.02159049	-0.2596758	0.14730429	0.23437753	0.42930108	0.10669981
cg05261349	ST3GAL2;ST3GAL2;ST3GA	brown	0.01857725	-0.5430545	0.09688241	0.36732117	0.93738766	0.14558005
cg09001896	FOXP1;FOXP1;FOXP1;FO	brown	-0.0282327	-0.5738952	0.11110996	0.36588953	0.91924896	0.16291914
cg06691005	RP11-521D12.5	brown	-0.3666666	-0.356167	0.10989944	0.16150185	0.39601391	-0.097019
cg08133496	EXT2;EXT2;EXT2;EXT2;EX	brown	-0.0913625	-0.22407	-0.0310264	0.21611292	0.43434847	0.09290838
cg18035323	SNX9;SNX9	brown	-0.0430987	-0.5229845	0.26815865	0.30015804	0.75884697	0.30379732
cg12513221	ABCB8;ASIC3;ASIC3;ASIC	brown	-0.1695053	-0.4253164	0.20926967	0.17631482	0.55121363	0.10443191
cg03972838	SMCR7L;SMCR7L;SMCR7	brown	-0.095648	-0.0308765	0.32821749	0.40066215	0.37244436	-0.0776138
cg08541497	C8orf76;C8orf76;C8orf76;C8	brown	-0.029963	-0.2719515	0.27662777	0.17483168	0.32522341	0.20734911
cg02061804	DOC2B;DOC2B	brown	0.03870323	-0.2820149	0.10591942	0.21750184	0.56343811	0.19054179
cg01439892	RP11-800A3.4	brown	-0.0954048	0.08884885	0.20757886	0.38667337	0.25005758	-0.013016
cg10526841	TRIM26;TRIM26;TRIM26;T	brown	-0.2701841	-0.0907125	0.08798755	0.20114588	0.26267341	-0.0767516
cg00660817	KIF3A;KIF3A;KIF3A;KIF3	brown	-0.3374168	0.05203238	-0.0656773	0.09031743	-0.1936848	-0.143352
cg00329447	PLEC;PLEC	brown	-0.0249269	-0.4142536	0.1976502	0.2964093	0.76351816	0.11988389
cg15996769	ACACB;ACACB	brown	-0.1836058	-0.359435	0.28903219	0.27367976	0.42974543	0.12985203
cg22076943	IGLV3-32	brown	-0.0399013	-0.1817466	0.15283652	0.17909791	0.34928954	0.13399177
cg01128107	TCHP;TCHP;TCHP;TCHP;T	brown	-0.1802443	-0.5027645	0.270719	0.22984987	0.66868124	0.09191438
cg11108105	RP11-960L18.1;RP11-	brown	0.03667654	-0.3632377	0.21652613	0.53498782	0.594366	0.36322023
cg03110082	ANKRD33;ANKRD33;ANK	brown	0.15675351	-0.1852497	0.21388875	0.26747409	0.3414358	0.42999293
cg14428590	RP11-37C7.1;RP11-	brown	-0.2035102	-0.2367971	0.23936568	0.37008063	0.48311272	0.07065839
cg19946091	LAIR1;LAIR1;LAIR1;LAIR1	brown	0.20231465	-0.2063277	-0.0094566	0.55793522	0.55714693	0.2244418
cg13845211	CTD-2260A17.2;CTD-	brown	0.1510299	-0.2568354	-0.0065182	0.64347652	0.6017537	0.30762642
cg11409900	PHLDA1;PHLDA1;PHLDA	brown	-0.326684	-0.5202106	0.05627302	0.04253374	0.59006303	-0.1050614
cg10772974	AVEN;CHRM5;CHRM5;CH	brown	-0.1384041	-0.6876102	-0.0214632	0.15465262	0.88649798	0.08502301
cg16052317	TTC23;TTC23;TTC23;TTC23	brown	-0.1364094	-0.4162095	0.0735341	0.02977037	0.40282534	0.02722868
cg25922970	BTBD3;BTBD3;BTBD3;BTB	brown	-0.3184037	-0.4350869	0.15411218	0.04099513	0.40550773	-0.1208814
cg03354908	ANKRD29	brown	-0.0430352	-0.2266536	0.01571701	0.19155522	0.41567319	0.06348132
cg04567302	SLC44A4;SLC44A4;SLC44	brown	0.12802701	-0.2902643	0.00487952	0.36484791	0.56668952	0.21604369
cg07150049	CCL26;CCL26	brown	0.12812731	-0.0345157	-0.1154541	0.45185116	0.43868403	0.15288683
cg06555246	CTXN1;TIMM44	brown	0.09149055	-0.1855802	0.31220141	0.52931549	0.47650164	0.30479965
cg23189410	ZIC4;ZIC4;ZIC4;ZIC4;ZIC1;	brown	-0.1824556	-0.7378191	0.19006794	0.23996979	0.80611253	0.15817187
cg01055562	COG2;COG2;COG2;COG2;	brown	0.0309244	-0.4520378	0.32102712	0.37801941	0.69428528	0.21373056
cg03100639	TGFB1I1;TGFB1I1;TGFB1I	brown	0.09998537	-0.2129925	0.20033369	0.26987532	0.44685064	0.30549098
cg05110943	DSC2;DSC2;DSC2;DSC2	brown	-0.2526203	-0.482809	0.2506675	0.21846469	0.47564404	0.01140757
cg24214470	SERPINF1;SERPINF1;SERP	brown	0.05777986	-0.4119651	0.20938967	0.340754	0.80023031	0.15353287

cg06694446	UTS2D;UTS2D;UTS2D	brown	0.20523373	0.69012879	-0.0797486	-0.1425874	-0.6834065	-0.0611248
cg16981180	RP11-315J22.5;PARL	brown	0.05167098	-0.3039986	0.22978961	0.43633842	0.68474954	0.18472143
cg18582505	LGALS7B	brown	0.18889167	-0.0005866	0.28389986	0.35165392	0.38575449	0.4741504
cg14194187	DHRS9;DHRS9;DHRS9;DH	brown	0.03167647	-0.4938267	0.12734142	0.45970191	0.90082917	0.17149613
cg15142116	FOXK2;FOXK2	brown	0.13987593	-0.3974777	0.08946934	0.43704502	0.79919514	0.18057584
cg01155426	RP4-728D4.2;RP4-728D4.2	brown	-0.0406128	-0.2506907	0.19909258	0.11374086	0.39060816	0.09080281
cg20213769	DNASE1L2;DNASE1L2;RP	brown	-0.0396821	-0.1857161	0.30015178	0.02985257	0.27573574	0.09398283
cg27564411	TJP3;TJP3	brown	-0.1193346	-0.2243491	0.17550081	0.25641863	0.38203323	0.10542216
cg11715228	CRB3;CRB3	brown	-0.054078	-0.4508726	0.19622719	0.36805181	0.6201241	0.20193295
cg10591948	GRN;GRN	brown	0.09643344	-0.3799169	-0.0228814	0.44132649	0.85747975	0.11437153
cg13250850	ACADM;RABGGTB;RABG	brown	-0.1108812	-0.4011506	0.01049863	0.26745262	0.68633733	0.01054389
cg02495743	RP11-693N9.2;RP11-	brown	-0.3405278	0.02578446	0.21473755	-0.0594559	-0.2182665	-0.0931585
cg00027712	DOCK9;DOCK9;DOCK9;D	brown	0.38738429	0.09973525	-0.1278308	0.06345303	0.28425739	0.08695492
cg13628881	CBX1;CBX1;CBX1;CBX1	brown	-0.0008378	-0.5376574	0.17072491	0.22779082	0.82149433	0.21027562
cg06138703	CNTROB;CNTROB	brown	0.22912341	-0.2326653	0.09370897	0.48219539	0.68706868	0.38904371
cg07691624	RPUSD3;RPUSD3;RPUSD3	brown	0.26818813	-0.2841655	0.08764174	0.49357917	0.78271292	0.2670543
cg21348900	DNMBP	brown	-0.3509597	-0.4028622	0.09163011	0.22808594	0.4193701	-0.158538
cg09902908	MIRLET7F1	brown	0.05483476	-0.0562989	0.00185668	0.37597513	0.31439256	0.07787297
cg07645296	ITPR2;ITPR2;ITPR2	brown	-0.2026014	-0.7616768	0.10026888	0.13535317	0.86224117	0.08530499
cg01029032	RP3-525N10.2;BAI3	brown	-0.0791603	-0.2335644	0.04343333	0.10264722	0.37936061	0.145057
cg03774275	PKNOX2;PKNOX2;PKNO	brown	0.10508548	-0.1347988	0.29701673	0.30188776	0.3470315	0.24206695
cg09052865	DAPK2;DAPK2;RP11-	brown	0.11031489	-0.326446	0.23627418	0.37400476	0.71125269	0.25688097
cg10658688	TBL2;TBL2;TBL2;TBL2;TBL	brown	0.07206039	-0.3185211	0.21639295	0.49660077	0.73873587	0.13865505
cg20663241	PNOC;RP11-380I10.4;PNOC	brown	0.10136346	-0.3821627	-0.0323713	0.27409929	0.78971945	0.20052902
cg09826056	GIPC2;GIPC2	brown	-0.3530884	-0.4105211	0.18854134	0.13037829	0.35545551	-0.0037252
cg05347914	GJD3	brown	0.22487503	0.06461584	0.41526619	0.4490476	0.43564732	0.31238241
cg14956920	VOPP1;VOPP1;VOPP1;VO	brown	0.09542407	-0.285711	0.3535084	0.64055764	0.67356049	0.23894397
cg14429919	RP1-40E16.3;RP1-	brown	0.28924746	-0.1446128	0.16028732	0.30859201	0.40850657	0.40534496
cg13828458	USP7;USP7;USP7;USP7;US	brown	0.00298363	-0.4980719	0.09612797	0.30123778	0.73647251	0.186707
cg21684564	MRPL10;MRPL10;MRPL10;	brown	0.08837336	-0.2297825	0.02604558	0.6060497	0.58457464	0.1619623
cg16864875	RP5-1187M17.10	brown	-0.0092549	-0.5184664	0.17923725	0.23676262	0.69994784	0.11442224
cg23060088	PRSS12;PRSS12	brown	0.15392184	-0.0256386	-0.142087	0.31631516	0.42039021	0.06050626
cg16046214	RANBP17;RANBP17;RANB	brown	-0.176403	-0.4733237	0.06623205	0.04538664	0.52233632	0.0597318
cg04637518	UCK1;UCK1;UCK1	brown	0.24388541	-0.1989613	0.21918581	0.44882334	0.66128341	0.34343994
cg07074571	AGPAT4;AGPAT4;AGPAT	brown	-0.099758	-0.4272703	0.02821571	0.06593102	0.52929293	0.07728444

cg14794592	ITGAL;ITGAL;ITGAL	brown	0.06623295	-0.4073815	-0.0405659	0.42090724	0.80056181	0.10482791
cg25831522	ST5;ST5;ST5;ST5;ST5;ST5;S	brown	0.03738675	-0.4511366	0.19644547	0.31313804	0.85290275	0.18524348
cg05973262	NOTCH4;NOTCH4	brown	0.02381629	-0.382932	0.17083514	0.31755289	0.73555566	0.16762935
cg10903575	NXN;NXN;NXN;NXN;NX	brown	-0.101321	-0.2538566	0.060259	0.24863853	0.50422329	0.03878379
cg13380502	GPR3	brown	-0.0315323	-0.4708775	-0.023202	0.28960506	0.76065784	0.09093555
cg12997637	NRN1	brown	-0.1934244	0.02437958	-0.3266282	-0.3239563	-0.2505107	-0.2739674
cg07165167	DDAH1;CYR61;CYR61;CY	brown	-0.1651116	-0.4366597	0.08817677	0.1366982	0.50397835	0.00643781
cg21466717	FAM82B;FAM82B;CPNE3;	brown	0.03070041	-0.5339658	0.09773817	0.32283591	0.89064217	0.19322417
cg25482382	TOMM5;RP11-613M10.9	brown	-0.028347	-0.5306952	0.07280919	0.3369428	0.88166549	0.1446068
cg00899160	GSTO2;GSTO2;GSTO2;GST	brown	-0.1982512	-0.3897691	0.03148227	0.20172056	0.45411414	0.0215915
cg05927518	TACC1;TACC1;RP11-	brown	0.00046948	-0.5467625	0.03415338	0.33280725	0.95159156	0.12498396
cg16467775	GAS8;GAS8;GAS8;GAS8;G	brown	-0.0172009	-0.4227948	0.18555416	0.36828652	0.71843604	0.0837517
cg18470229	ZNF3;ZNF3;ZNF3;ZNF3;ZN	brown	0.02774157	-0.2088823	0.23912476	0.33503826	0.34043656	0.26728807
cg14901202	LRRC43;LRRC43;LRRC43;L	brown	-0.1868741	-0.4646972	0.12166394	0.07783914	0.49633404	0.131332
cg05333719	CLK3;CLK3;CLK3;CLK3;C	brown	0.18898056	-0.2493158	0.42616767	0.3871295	0.50137205	0.56120292
cg12393201	GNB5;CTD-	brown	0.00251427	-0.547337	0.19696537	0.34054224	0.84436882	0.26323297
cg07643267	LYPD3;LYPD3;LYPD3	brown	-0.1561422	-0.4838389	0.14884233	0.20052346	0.58086217	0.11866065
cg10632566	SNX5;SNX5;SNX5;SNX5;S	brown	0.05110659	-0.5046778	0.05586737	0.37861623	0.9179633	0.18816834
cg26233209	ATG12;ATG12;ATG12;ATG	brown	-0.1771996	-0.4465479	0.22359184	0.33599662	0.59558555	0.12718503
cg06813382	RP11-40F6.2;RP11-40F6.2	brown	-0.1431638	-0.4172915	0.19090716	0.07845762	0.46918327	0.00674806
cg04349021	ANKRD33;ANKRD33;ANK	brown	0.07720898	-0.2332108	0.03910616	0.23528946	0.435816	0.05967761
cg11337758	EMID1;EMID1;EMID1;EMI	brown	-0.0640969	-0.5168566	0.09140075	0.40072647	0.89169518	0.13122329
cg21468244	CDH1;CDH1;CDH1	brown	0.31378684	-0.0806029	0.18129994	0.68273572	0.54144837	0.4866067
cg05407171	HNMT;HNMT;HNMT;HNM	brown	-0.1685637	-0.1471436	0.2148728	0.33710968	0.45962346	0.06852882
cg12055133	FOXK2;FOXK2	brown	0.01897543	-0.5224701	0.07670177	0.35969299	0.89716329	0.12094744
cg09732070	SPATS2;SPATS2;SPATS2;S	brown	-0.0536138	-0.6188816	0.01789662	0.2955022	0.9553549	0.1426771
cg23087150	CAP2;CAP2;CAP2;CAP2;C	brown	-0.3917992	-0.6028074	0.08949687	0.07030761	0.57663453	-0.0678613
cg19407392	PRNP;PRNP	brown	0.15558105	-0.4040579	0.19488084	0.4954843	0.85775581	0.28315163
cg14955420	PI4KB;PI4KB;PI4KB;PI4KB	brown	0.29126809	-0.1104944	0.01062241	0.69323451	0.58490207	0.2208264
cg06940169	TP53I11;TP53I11;TP53I11;T	brown	0.09694004	-0.1058631	0.36823243	0.38050028	0.47549665	0.28475295
cg12586933	C2orf88;C2orf88	brown	0.02545468	-0.3650311	0.05348572	0.24975485	0.48001718	0.21127282
cg03220054	RP11-644K8.1;LAPTM4A	brown	-0.089016	-0.6058023	0.12480726	0.21237899	0.66754424	0.20850544
cg13185177	GP5;GP5	brown	0.12118503	-0.3249603	0.07490031	0.30620896	0.65627722	0.14555371
cg00995986	MEIS1;MEIS1;MEIS1;MEIS	brown	-0.1425939	-0.6970235	0.07859227	0.16384785	0.8202201	0.10124298
cg22589165	RP11-21B21.4	brown	0.14091093	0.02782535	0.3206667	0.5017702	0.36326218	0.15481965

cg25783125	RNY3P6;TM9SF2;TM9SF2	brown	0.22656864	-0.2538287	-0.1380525	0.57619354	0.72250164	0.13920201
cg14773011	CTD-	brown	0.5998935	0.06130773	-0.0134965	0.38994667	0.49104962	0.2264859
cg26196104	AC005356.1;GLIS2	brown	0.12499037	-0.431084	0.19176082	0.21653377	0.79362243	0.41910194
cg22522886	IGFBP5;IGFBP5;IGFBP5;A	brown	0.01176495	-0.315381	0.10748756	0.30472224	0.51118033	0.15689852
cg20976526	RGS22;RGS22;RGS22;RGS	brown	-0.054672	-0.2691712	0.02478826	0.19212383	0.34866767	0.12377511
cg08424722	ETV7;RP1-	brown	-0.1981691	-0.4020615	0.18087911	0.15111495	0.47975037	-0.0157135
cg15644413	C1orf216;C1orf216;CLSPN	brown	0.0390772	-0.4962981	0.00407947	0.30784966	0.91057426	0.15573843
cg03474822	MEI1;MEI1;MEI1;MEI1;MEI	brown	-0.14709	0.07998989	0.31263375	0.21045286	0.19149035	0.03920777
cg17125270	PLD3;PLD3;PLD3;PLD3;PL	brown	-0.1270215	-0.4552262	0.29709374	0.05859269	0.50900506	0.10522673
cg13306032	AP001171.1	brown	-0.1844816	-0.3883477	-0.0087286	0.27211191	0.53694504	0.01909065
cg16680677	RP11-90J7.3;RP11-	brown	-0.1658177	-0.4646594	-0.0890947	0.08823213	0.47066939	0.14258539
cg01063003	RXRA;RXRA	brown	0.09847326	-0.3862677	0.17157566	0.2900624	0.7710027	0.18869502
cg07637213	SLC18A3;CHAT;CHAT;SLC	brown	-0.2200856	-0.6688911	0.27641909	0.08337791	0.6378216	0.15639008
cg23027596	UBAC1;UBAC1	brown	-0.1628766	-0.1913812	0.20098943	0.33055951	0.58905981	0.00324638
cg00888605	ZNF19;AC010547.9;ZNF19;	brown	0.16205734	-0.0785527	0.10751409	0.42082119	0.42473768	0.11610291
cg02335376	GDPD3;GDPD3;GDPD3	brown	0.18080652	-0.1860002	0.24947838	0.51096385	0.64930344	0.2302597
cg11176053	ANGPT2;ANGPT2;ANGPT	brown	0.12070208	-0.393092	0.0557853	0.35234275	0.76191726	0.16103431
cg13358761	DPPA4;DPPA4;DPPA4;DPP	brown	-0.1321482	-0.5015822	0.08664503	0.18914083	0.6761474	-0.0172708
cg19465850	FTSJD2;FTSJD2;FTSJD2;FT	brown	0.18800783	-0.1626274	0.25906137	0.47714963	0.54218987	0.5094749
cg18347626	SUV420H2;SUV420H2;SU	brown	0.20518122	-0.0258134	-0.0082529	0.27867927	0.38594825	0.3054856
cg12686273	RP11-	brown	-0.011318	-0.1364153	0.00961559	0.20944917	0.45250503	0.09858887
cg24258295	CTD-2016O11.1;RP11-	brown	-0.2046141	-0.2792723	0.16085816	0.08225073	0.38309545	0.1082154
cg08794720	RUSC2;RUSC2;RUSC2;RU	brown	0.09052307	-0.4958157	0.28712514	0.43807066	0.85981991	0.31011306
cg12761421	LGR6;LGR6	brown	0.13014146	-0.2524558	0.07312685	0.50966106	0.59598817	0.24573884
cg24259228	SSC5D;SSC5D	brown	0.02310866	-0.3477579	-0.0257672	0.32780799	0.71648798	0.05567473
cg04863746	PNKD;AC021016.8	brown	0.07856046	-0.2225191	-0.0817008	0.11617305	0.45019733	0.11137531
cg11562037	SDK2;SDK2	brown	0.15869023	-0.1645319	0.24047377	0.21017654	0.38857365	0.29735326
cg11200858	UNC93B1;UNC93B1;UNC9	brown	-0.1618091	-0.4412167	0.02279058	0.21904379	0.62250391	0.0531618
cg18862171	ZDHHC24;ZDHHC24;ZDH	brown	-0.3954613	-0.3603909	0.16136331	0.14453828	0.29405524	-0.1384197
cg05549717	HYDIN;HYDIN;HYDIN;H	brown	-0.0175387	-0.409813	0.10284889	0.20787201	0.69097211	0.27817708
cg05495351	NRXN1;NRXN1;NRXN1;A	brown	-0.2292261	-0.6098098	0.12185548	0.05216611	0.6327683	0.06742712
cg09580336	ATP1A1;ATP1A1;ATP1A1;	brown	-0.1699829	-0.5012038	0.13927805	0.18873723	0.55903478	0.01936939
cg11045332	TTLL1;TTLL1;TTLL1	brown	0.07733097	-0.5006311	0.15825046	0.41209505	0.9087733	0.30188559
cg07732735	TTC23;TTC23;TTC23;TTC23	brown	-0.0044324	-0.409175	0.04712855	0.14700776	0.5878356	0.06797879
cg13933070	LINC00484;RP11-	brown	-0.2879094	0.0146597	0.39400522	0.0014077	-0.1531735	0.06632423

cg05698970	KIAA0090;KIAA0090;KIA	brown	0.2590477	-0.2603406	0.22085311	0.62145097	0.74149497	0.27380321
cg00548989	ADAM33;ADAM33;ADAM	brown	0.09049052	-0.1706524	0.05012653	0.31630803	0.31497299	0.26627795
cg18906520	OGFOD2;OGFOD2;OGFO	brown	0.18635171	-0.1759446	0.47586903	0.5694605	0.52065817	0.35208593
cg14984781	DOC2B;DOC2B	brown	0.10600806	-0.2708048	0.04005906	0.25977359	0.63038985	0.22189909
cg07941983	RP11-	brown	-0.1224676	0.15281911	-0.2798863	-0.2919534	-0.3103083	-0.2492799
cg26279928	ANKRD33;ANKRD33;ANK	brown	-0.144358	-0.3806909	0.0829922	0.15796467	0.44805038	0.11079365
cg13860325	AFAP1;AFAP1	brown	0.02392942	-0.3671128	-0.1808063	0.15453735	0.68173573	0.08952274
cg10050661	SMARCE1;KRT222	brown	-0.0849659	-0.7084148	0.08949512	0.20059632	0.74273095	0.24277991
cg08632909	FADD	brown	-0.0007952	-0.5580604	0.14095138	0.31560091	0.86788529	0.27262359
cg04361103	HVCN1;HVCN1;HVCN1;H	brown	-0.2999846	0.13501023	0.22596989	0.07407261	-0.3942959	0.02344288
cg25932329	DDX39A;DDX39A;DDX39	brown	-0.2602155	0.18846232	0.30579164	0.08842017	-0.2604763	-0.0018932
cg08468449	RP11-70P17.1	brown	-0.0514373	-0.5950818	0.09991766	0.31935653	0.96097271	0.13226669
cg16084409	TGFA-IT1;TGFA	brown	0.24322567	-0.1958619	-0.0025192	0.45915529	0.7232555	0.24465339
cg06502285	METTL20	brown	0.03988959	-0.2425227	-0.0111248	0.18747101	0.47350444	0.09932135
cg02800986	SHROOM1;SHROOM1	brown	-0.1657159	-0.2071287	0.19649922	0.065047	0.30558776	0.16958056
cg00082310	AC078953.1;PLD1;PLD1;PL	brown	-0.1119805	-0.5169973	0.09409526	0.18807946	0.62532436	0.12731143
cg10296773	HIST1H1E	brown	-0.0030378	-0.3026781	0.23169467	0.26934485	0.54545277	0.20641296
cg04127455	GPATCH4;GPATCH4;GPA	brown	0.03087754	-0.3206939	0.1581152	0.2943375	0.51007573	0.26780168
cg18053607	INPP5J;INPP5J;INPP5J;INP	brown	0.20985638	-0.1874246	0.28388897	0.36405018	0.6462543	0.32770065
cg01602139	Y_RNA;PI16	brown	-0.0399296	-0.2981819	0.14921684	0.12914581	0.47544541	0.11786509
cg08833432	PSMA7;PSMA7;SS18L1	brown	-0.0518112	-0.2885866	0.24724937	0.13674943	0.43454685	0.22221557
cg03531237	ABI2;ABI2;ABI2;ABI2;ABI	brown	-0.0633372	-0.6741816	0.11319588	0.24176276	0.87738993	0.19347813
cg12968598	CD2AP	brown	-0.0210688	-0.2740486	0.38697538	0.15985162	0.31507483	0.18720945
cg05553956	IGSF10	brown	-0.0319528	-0.5272064	0.17489076	0.16681006	0.83695525	0.11934489
cg03471593	LPCAT2;LPCAT2	brown	0.28443218	-0.1364246	0.2045764	0.63163715	0.5834011	0.31781417
cg12047944	SPNS3;SPNS3;SPNS3;SPN	brown	0.29400422	-0.2198702	0.09802027	0.51919133	0.71546315	0.40090723
cg22143856	ZNF192P1;ZNF192P1;ZNF1	brown	-0.0981265	-0.3634045	0.12265029	0.11520484	0.39251892	0.12225269
cg09225308	NOL6;NOL6	brown	0.06158908	-0.0484756	0.30122434	0.32900198	0.24998218	0.26144804
cg18028808	S100A5;S100A5	brown	-0.2157177	0.05710413	0.33395334	-0.0149179	-0.1639683	0.05284911
cg05155595	ANXA4;AC092431.1;ANXA	brown	0.11165284	-0.4057588	-0.1137622	0.47115639	0.79061189	0.10877405
cg23695135	DTNB;DTNB;DTNB;DTNB;	brown	0.10314472	-0.411401	-0.0138686	0.49815879	0.85636191	0.14668483
cg15718594	BATF3	brown	-0.2357828	-0.2300984	0.23524995	0.24539131	0.33978784	-0.102374
cg25198495	VKORC1;VKORC1	brown	-0.0195676	-0.4568589	0.07856009	0.3034374	0.86127864	0.10018286
cg18675097	ZKSCAN4;NKAPL;NKAPL	brown	0.1292842	-0.3217946	0.07551643	0.21234107	0.48827511	0.25051717
cg19905880	ADRBK1;ADRBK1;ADRB	brown	0.40260431	-0.20188	0.22473895	0.52000825	0.64879225	0.65404961

cg07018853	KANSL3;KANSL3;KANSL	brown	-0.2838123	-0.3320644	0.2648542	0.11782914	0.30927877	-0.0085225
cg10395101	ANGPT2;ANGPT2;ANGPT	brown	0.12352937	-0.3624408	0.0543952	0.45388465	0.78094755	0.27072954
cg18530645	MAN2B1;MAN2B1;MAN2	brown	0.20234289	-0.209605	0.18619108	0.27143111	0.49509243	0.34425145
cg08493959	INHA;OBSL1;OBSL1;OBSL	brown	0.15651543	-0.2869644	0.3026987	0.41979249	0.58368532	0.27494143
cg15818019	HOPX;HOPX;HOPX;HOPX	brown	0.12390004	-0.2092114	-0.119165	0.30748359	0.55644701	0.09534067
cg26210449	MAPK9;MAPK9;MAPK9;M	brown	-0.1758842	-0.3383544	0.03412717	0.1386929	0.5101001	-0.1012928
cg08438690	PPM1M;PPM1M;PPM1M;P	brown	-0.1815563	-0.1583728	0.2819553	0.0947915	0.15547587	0.11647416
cg25929976	PPARG;PPARG;PPARG;PP	brown	0.12991356	-0.1484637	-0.2070226	0.03974727	0.34168803	0.09643732
cg22672242	CTD-	brown	-0.3905756	-0.2967712	0.08855389	0.01267153	0.34338997	-0.075312
cg14442689	CTD-2335A18.1;CTD-	brown	0.07407844	-0.5185114	0.11689337	0.21985719	0.87955766	0.18919701
cg13385114	TMEM201	brown	0.04633836	-0.470639	0.06119553	0.39135967	0.88845562	0.169752
cg22786811	AC005027.4;AC005027.4;A	brown	0.11762459	-0.2175281	0.17365261	0.52947054	0.62477055	0.32256905
cg08708079	SPEF1	brown	0.13242506	-0.4109488	0.00916072	0.38699785	0.87914536	0.18712161
cg12479651	RHOC;RHOC;RHOC;RHOC	brown	0.26081493	-0.104573	0.36180898	0.2907229	0.4080293	0.37042545
cg04818845	PLEKHG5;PLEKHG5	brown	0.04575772	-0.1980691	0.33815893	0.23107049	0.32170058	0.25677276
cg19081503	MYBBP1A;MYBBP1A;MY	brown	-0.1484924	-0.5554148	0.10509442	0.25523595	0.70908378	0.09571286
cg01260219	ADAMTS8;ADAMTS8	brown	-0.1800468	-0.2988043	0.2074838	0.23255286	0.46971497	0.10679941
cg18580117	SNAP23;SNAP23;SNAP23;	brown	-0.1846782	-0.4348002	0.17533085	0.17866594	0.55136414	0.079877
cg08973950	GPR146;GPR146;AC073957.	brown	0.00324396	-0.417763	0.05263346	0.37023627	0.81071393	-0.0756376
cg21864105	FRMD8;FRMD8;FRMD8;F	brown	-0.3213211	-0.3963078	0.08864153	0.17818588	0.4318447	-0.1872705
cg11644586	CCDC87;CCS;CCS;CCS;CC	brown	-0.3172898	-0.5589665	0.2460501	0.11294787	0.53620126	0.03737802
cg04174927	IFT140;IFT140;LA16c-	brown	0.23389345	-0.3572025	0.10697476	0.36683506	0.82140874	0.34262601
cg25769566	LY6G5C;LY6G5C	brown	-0.0513468	-0.1933877	-0.000637	0.1568342	0.34527822	0.14794338
cg26717696	FITM1;FITM1	brown	0.48664113	0.04565581	-0.1703565	0.22971923	0.44293882	0.06141107
cg24796852	GMFG	brown	-0.285405	-0.3327311	0.21263154	0.18617357	0.54504046	-0.056367
cg18403361	CLEC14A;CLEC14A	brown	-0.0139427	-0.2658222	-0.3403825	-0.0723188	0.23632948	0.02068137
cg06123361	KIAA0930;KIAA0930;KIA	brown	0.16528625	-0.319962	0.04622467	0.44954176	0.74524209	0.23136369
cg10395685	CCDC81;CCDC81;CCDC81	brown	-0.2096397	-0.5796113	0.32242339	0.11174212	0.52305874	0.03057149
cg11304234	IL18;IL18;IL18;IL18;IL18;AP	brown	-0.217268	-0.6977159	0.1151505	0.09515713	0.71162687	0.01550178
cg02544979	TESPA1;TESPA1;TESPA1;T	brown	0.07451749	-0.3910221	0.02676041	0.57451416	0.7868743	0.15339303
cg19650983	CTD-2314G24.2	brown	-0.1330099	-0.488365	0.1554124	0.17423895	0.50051771	0.16829712
cg02003117	SLC9A8;SLC9A8;SLC9A8;S	brown	0.10579654	-0.389443	0.06461029	0.27330812	0.78763343	0.21284758
cg04876758	NUP160;NUP160;NUP160;	brown	-0.1260357	-0.5375252	0.11612959	0.22678404	0.78388047	0.14127526
cg13658683	RGL4;RGL4;GUSBP11;RGL	brown	0.09417626	-0.4817336	0.15653886	0.30039852	0.91097261	0.24351255
cg00500037	GIPR;GIPR	brown	-0.0469105	-0.4238654	0.12343795	0.33091607	0.79272209	0.19865185

cg10347032	MAP6D1;MAP6D1;MAP6D	brown	-0.2327058	-0.5733796	-0.0742729	0.15276189	0.61430376	-0.0092421
cg03505273	RP11-945A11.2	brown	-0.1338541	-0.2093179	0.21324244	0.20589919	0.34243572	0.08086564
cg15056348	INPP5A;INPP5A	brown	-0.0343019	-0.4510203	0.25155925	0.38462075	0.72245021	0.23130798
cg04891645	AC068138.1	brown	-0.1027359	-0.5446394	0.10443596	0.18084285	0.7330841	0.06505075
cg09146471	TTC23;TTC23;TTC23;TTC23	brown	-0.0845279	-0.3780106	0.06268884	0.05821421	0.46720023	0.06098738
cg16301176	NDUFA5;NDUFA5;NDUF	brown	-0.3145861	0.00600716	0.24403641	0.02578237	-0.1674697	-0.0392066
cg22697136	FBXO2;FBXO2;FBXO44;F	brown	-0.0563315	-0.4263012	0.27840357	0.21404176	0.4874275	0.24587708
cg26825412	ZNF512B;SOX18;TCEA2	brown	-0.0544652	-0.3343584	0.17889257	0.29733831	0.57885263	0.10679816
cg13010299	ERGIC3;ERGIC3;ERGIC3;E	brown	-0.2727055	-0.3691446	0.2613472	0.00392565	0.37362302	-0.0322605
cg13309415	BAHCC1	brown	0.22937816	-0.0645528	0.36302996	0.38484647	0.38719628	0.35589211
cg00337187	RP1-40E16.3;RP1-	brown	0.04479892	-0.2712279	0.17106224	0.23617644	0.46277198	0.23822225
cg05806864	MAN2B1;MAN2B1;MAN2	brown	0.06981222	-0.283679	0.13285707	0.16183567	0.4658525	0.2361953
cg02022181	MIR339;C7orf50	brown	-0.0126039	-0.4878736	0.15451863	0.46561041	0.78997617	0.17990903
cg03700483	KXD1	brown	-0.1716321	-0.6588689	0.08359892	0.22111979	0.80066475	0.10079258
cg09523275	NKAPL;ZKSCAN4	brown	-0.1429915	-0.4365578	0.0923013	0.0588993	0.47875816	0.04225
cg12324629	ULK2;ULK2	brown	-0.0943672	-0.5098725	0.14480589	0.21878057	0.67585304	0.0471286
cg27655607	FDXACB1;RP11-	brown	0.2044899	-0.2840531	-0.0413858	0.43567133	0.74452639	0.1928185
cg00536532	LINC00336	brown	0.00770313	-0.2477907	0.30739827	0.39489134	0.49184098	0.26761365
cg23663627	C16orf93;RNF40;C16orf93;R	brown	0.15212457	-0.3290914	0.17350161	0.63907382	0.7099898	0.29835736
cg09116474	RP1-	brown	-0.0874634	-0.2517357	-0.0086986	0.13590412	0.39800069	0.14181113
cg07974441	RP11-96C23.8;RP11-	brown	0.18029098	-0.0838814	0.14276595	0.48127015	0.44631137	0.12812052
cg02434059	ADAP2	brown	0.00070898	-0.4244061	-0.1999099	0.18042042	0.66779552	0.06216737
cg03141756	LY6G5C;LY6G5C	brown	0.23877793	-0.0293172	0.20633474	0.19684792	0.38480475	0.2818887
cg10771480	SYNE2;SYNE2;SYNE2;SY	brown	-0.2727068	-0.7637846	0.06886483	0.13908446	0.74678514	-0.0503733
cg20721921	NECAB3;NECAB3;NECAB	brown	0.20188619	-0.3223609	0.20385892	0.33279937	0.75848905	0.28970087
cg09856074	RP11-255G12.2	brown	-0.0079471	-0.3161768	0.15547681	0.39701089	0.62707664	0.15286505
cg03732014	BACH1;BACH1;BACH1;BA	brown	-0.0599167	-0.539205	-0.0611006	0.36181184	0.91620253	0.04728761
cg23279681	DAGLA;DAGLA	brown	0.03965303	-0.210258	0.20284372	0.26229126	0.54984918	0.18747612
cg22016947	RP3-368B9.2	brown	-0.0893171	0.16240777	0.28708172	0.17513995	-0.1776649	0.11092448
cg18378267	MED13L;MED13L	brown	0.03276508	-0.5198724	0.0851503	0.33502034	0.94833349	0.1336826
cg11540476	LY6G5C;LY6G5C	brown	-0.1546871	-0.3034547	0.04340373	0.03847176	0.37704275	0.09318366
cg25405284	DNAJC18;DNAJC18;DNAJ	brown	0.04314926	-0.5217686	-0.0375272	0.38419611	0.91413373	0.17006122
cg04258124	CCDC19;CCDC19;CCDC19	brown	0.15919452	-0.1195693	-0.0090127	0.60657648	0.55342144	0.1389223
cg04747322	SNCAIP;SNCAIP;SNCAIP;	brown	-0.2462119	-0.5315703	0.20085651	0.10425708	0.52040404	0.02597599
cg07122519	RP11-	brown	-0.0496732	-0.2142889	0.2096821	0.20797238	0.43231576	0.0865462

cg17048213	VAMP4;VAMP4;VAMP4;V	brown	-0.064524	-0.1456103	0.02303275	0.26011489	0.34907309	0.03669206
cg15982419	RALBP1;RALBP1	brown	-0.3013327	-0.6024409	-0.023613	0.0697546	0.56959717	-0.0822197
cg09551152	RP11-	brown	0.08606589	-0.3369415	-0.0357234	0.45363761	0.79065268	0.15910498
cg20660182	BCL2L11;BCL2L11;BCL2L1	brown	-0.2410199	-0.5584111	0.03565137	0.13701704	0.53572765	0.07313129
cg22318514	LY6G5C;LY6G5C;LY6G5C;	brown	-0.1448697	-0.2990498	0.15014961	0.09607619	0.33346673	0.08273149
cg26366092	Y_RNA	brown	-0.1919609	-0.4784916	0.07223697	0.15807967	0.75483478	0.0400435
cg13017848	RP11-443B7.2	brown	0.09465505	-0.5020229	0.09056225	0.3862913	0.91468397	0.1845259
cg01031251	RPS6KA1;RPS6KA1;RPS6	brown	0.16646507	-0.4047355	0.05658059	0.40967096	0.87753531	0.25701907
cg10376398	CTSK;CTSK	brown	0.24433222	-0.0215546	-0.2048175	0.54792976	0.43069744	0.34365193
cg13215087	MEGF9;MEGF9	brown	0.08968169	-0.3273339	0.22861676	0.40628733	0.74878844	0.26552452
cg23192873	LRRD1;LRRD1;LRRD1;LRR	brown	-0.2652035	-0.5352881	0.04863503	0.15880223	0.58550375	0.00651857
cg10313047	DNAH1;DNAH1;DNAH1	brown	-0.0025072	-0.2514773	0.22012271	0.21141796	0.40529888	0.08755757
cg17022671	DNAJC7;DNAJC7;DNAJC7	brown	-0.2062359	-0.5825837	0.26312386	0.15730449	0.5809029	0.0900545
cg13381089	AIFM2;AIFM2	brown	0.14226418	-0.3411187	-0.0934024	0.32022299	0.76827648	0.2878337
cg19118014	RP11-433A10.1;RP11-	brown	0.07922578	-0.4417412	0.03868504	0.38900434	0.87138272	0.14085975
cg04180275	RP11-	brown	-0.2078115	-0.4167727	0.25323241	0.23222738	0.50657349	0.18436219
cg09151120	SLC16A10;SLC16A10	brown	0.49031474	0.0891178	-0.2012731	0.31227527	0.461616	0.04965152
cg22766963	SLC25A1;SLC25A1;SLC25	brown	0.31302643	-0.1559326	0.4114567	0.43486322	0.63460434	0.25776025
cg10183150	C4orf3	brown	-0.2440759	-0.5336691	0.01952214	0.06068812	0.53754676	-0.0577787
cg16253634	CALD1;CALD1;CALD1;CA	brown	0.094788	-0.1689326	0.19707893	0.13978292	0.38497636	0.10802154
cg22824730	MCTP1	brown	0.01617205	-0.1381844	-0.1480456	0.03078748	0.32129181	-0.0338287
cg05261702	HMG20B;HMG20B;HMG20	brown	-0.2139584	-0.5618046	0.02816208	0.08515924	0.607709	-0.0221963
cg24293739	DICER1;DICER1;DICER1-	brown	-0.2001613	-0.3401086	0.17445551	0.33221622	0.61721874	0.02502069
cg05650674	CDH3;CDH3;CDH3;CDH3	brown	-0.1822346	-0.4827908	0.07458946	0.25154305	0.64678687	0.11309965
cg01955239	OXGR1;OXGR1;OXGR1;O	brown	-0.2757321	-0.4237243	0.00462336	0.12482714	0.4707184	-0.0170312
cg06588870	RAB25;RAB25;RAB25;RAB	brown	0.10502326	-0.3053693	0.33748539	0.34002027	0.53780444	0.28890778
cg07496190	ADAP1;ADAP1;ADAP1;A	brown	0.00111569	-0.2551517	0.01573046	0.14339645	0.46756373	0.03467744
cg20069688	STK19;DOM3Z;DOM3Z;DO	brown	0.04714626	0.52634427	0.30674305	-0.0619877	-0.6177119	0.05029399
cg19388710	NUTF2;EDC4;EDC4;EDC4	brown	0.1013855	-0.4560518	0.18796459	0.33874024	0.83478954	0.30135005
cg19833103	LRCH1;LRCH1;LRCH1;LRC	brown	-0.2588197	-0.4248654	0.12417788	0.23877053	0.4503898	0.07967532
cg09224529	SLC35B1;SLC35B1;SLC35B	brown	0.05163562	-0.526313	0.03757925	0.27822909	0.92425928	0.19296585
cg22549408	PMAIP1;PMAIP1	brown	-0.0265053	-0.4121607	0.02790714	0.27281925	0.578742	0.13219379
cg26830053	RP11-	brown	-0.0103316	-0.5688282	-0.0588896	0.3046893	0.89631922	0.11224541
cg10019467	RBM47;RBM47;RBM47;RB	brown	0.01768048	-0.5250555	0.07910012	0.33650656	0.90930596	0.16600335
cg17761940	DST;DST;DST;DST	brown	-0.0331297	-0.3109765	0.12389722	0.28534113	0.56837598	0.10874698

cg16121470	SMAD4;ELAC1	brown	-0.3269767	-0.0194377	0.1826347	-0.0002128	-0.1923534	0.0498
cg05944099	ADAR;ADAR;ADAR;ADA	brown	0.21700358	-0.3292568	0.06623928	0.49039228	0.75170227	0.25292425
cg23605244	SLC45A3	brown	0.11785115	-0.4453944	0.11814665	0.34112682	0.87906083	0.2707997
cg18013529	LZTR1;AIFM3;LZTR1;AIF	brown	-0.251611	-0.7529637	0.1287069	0.12218153	0.77777351	0.00676479
cg13263950	RP4-728D4.2;RP4-728D4.2	brown	0.03520561	-0.2866737	0.24995498	0.15776415	0.4895756	0.12659676
cg08490387	RP11-488C13.4	brown	0.22816579	-0.1226926	0.02950071	0.33458048	0.37449979	0.29651096
cg23834034	NADK;NADK;NADK	brown	0.14353751	-0.3657928	0.27755968	0.40256733	0.80217242	0.23129507
cg17343088	RP11-136O12.2	brown	-0.1601265	-0.7309334	0.07588436	0.20040759	0.87726357	0.08119853
cg26149485	LMNB2;TIMM13;LMNB2;L	brown	0.06771844	-0.1448504	0.08010548	0.31448704	0.38556215	0.09444575
cg04501839	CYP2C18;CYP2C18;CYP2C	brown	-0.0769619	-0.4498959	0.13814695	0.19546931	0.58470324	0.37436786
cg05758783	ATP5G2;ATP5G2	brown	0.0906384	-0.4164664	-0.0813396	0.35109076	0.82609681	0.16858172
cg19916364	DEPDC1;RP4-	brown	-0.0315932	-0.0428271	0.02640199	0.27045915	0.35592529	0.07421568
cg12149609	JPH2;JPH2	brown	-0.1817047	-0.4951945	0.2322974	0.28886476	0.58757811	-0.1142275
cg04742810	POLA2;POLA2;POLA2;POL	brown	0.04505092	-0.0557693	0.24548006	0.41732705	0.30741911	0.28797975
cg02091284	MAN2C1;MAN2C1;MAN2	brown	-0.4499923	-0.0770662	0.18499513	-0.1465645	-0.2406413	-0.1188918
cg00327322	SLC17A5	brown	-0.1130533	-0.2962997	0.18928431	0.22590825	0.44017314	0.13222364
cg21032100	FLT3;FLT3	brown	0.1218159	-0.4328637	0.12977498	0.33134946	0.80203442	0.27075977
cg20702870	TADA2B;TADA2B;TADA2	brown	0.16688298	-0.3241218	0.07538564	0.62247482	0.73667411	0.27812038
cg02293759	TAGLN;TAGLN;TAGLN;T	brown	0.25769036	-0.1442206	-0.0323577	0.33316785	0.54235124	0.23192653
cg03068051	CACHD1;CACHD1	brown	-0.2250312	-0.2037734	0.09855399	0.17830509	0.35141255	-0.0199058
cg02270514	CACNB1;CACNB1	brown	0.22625163	-0.1720114	-0.1315611	0.35210964	0.59956093	0.18710899
cg08186447	ZFYVE19;ZFYVE19;DNAJ	brown	0.02753892	-0.3482419	0.12120919	0.23883564	0.58307867	0.18307253
cg08495870	NUAK2	brown	-0.1458013	-0.2457041	0.25618826	0.28570003	0.32615577	0.05261689
cg27364231	ESAM;RP11-677M14.3	brown	-0.2414568	-0.3629097	0.27185347	0.08250839	0.3264456	-0.0143906
cg06330675	TM7SF3;TM7SF3;TM7SF3;	brown	-0.0085356	-0.4692251	-0.0029643	0.40067309	0.80784168	0.07740228
cg16385069	MPST;MPST;MPST;MPST;	brown	-0.3944029	-0.0178168	0.34131732	-0.0704659	-0.1448422	-0.0535961
cg18094999	USP28;USP28;USP28;USP2	brown	-0.3142126	-0.5253504	0.21655049	0.04738603	0.5409942	0.0213356
cg10107382	ZCCHC3	brown	0.0865152	-0.3929054	0.20586231	0.39167341	0.69652186	0.2521963
cg11601400	RN7SK;GSTA4;GSTA4;GS	brown	-0.1101008	-0.6697349	0.0629886	0.22649572	0.8903429	0.00301827
cg03130816	AKIRIN1;AKIRIN1;AKIRI	brown	0.1462314	-0.2916831	0.03362313	0.36611623	0.55731499	0.18248179
cg05180756	IPO13;IPO13	brown	-0.0197366	-0.5055953	0.01147927	0.23788972	0.85172432	0.0738595
cg26040735	RP11-672A2.5	brown	0.13528864	-0.4557462	0.37107401	0.35700434	0.77511067	0.26135188
cg14319451	RCOR2	brown	-0.0747147	-0.4663259	0.27874237	0.3149295	0.70276026	0.20452743
cg21362968	ESAM;ESAM;RP11-	brown	-0.3341092	-0.3717338	0.22689048	0.14205141	0.38463469	-0.074117
cg25757472	AC002456.2;AC002456.2;A	brown	-0.0516284	-0.5409962	0.08986756	0.11542214	0.54705903	0.17983031

cg20481312	RP11-30K9.5;RNF111	brown	0.06544364	-0.3845602	0.19660652	0.24959213	0.70864258	0.24599671
cg01679043	SYNE1;SYNE1;SYNE1;SY	brown	-0.1638801	-0.3542558	0.2148076	0.17167003	0.36486954	0.05447858
cg07712977	CD151;CD151;CD151;CD15	brown	-0.1188577	-0.4093494	0.24373323	0.38710686	0.61941579	0.05270496
cg00043800	DCTN1;AC005041.9;AC005	brown	0.12500769	-0.1748098	0.11320514	0.61816308	0.56858927	0.20212753
cg04267772	CARHSP1;CARHSP1;CAR	brown	0.03917377	-0.5106333	0.14670047	0.29010785	0.81480721	0.21710286
cg25213362	TMPRSS12;TMPRSS12	brown	0.03672068	-0.2015239	0.08117895	0.16861729	0.37133563	0.16785118
cg01310557	KCNAB2;KCNAB2;KCNA	brown	0.15819405	-0.3283078	0.01098295	0.39235401	0.80368907	0.2720731
cg05136169	AVP	brown	0.0346689	-0.0909146	0.36751638	0.1025803	0.15114727	0.33874584
cg22270609	RP11-461A8.1	brown	-0.0279564	-0.5608905	0.08498774	0.23176042	0.91086192	0.19317454
cg14848635	SMARCA2	brown	0.04831626	-0.5484372	0.10253958	0.32585536	0.93055321	0.303024
cg02532442	RN5S478	brown	0.21117916	-0.1254962	0.27173549	0.35201855	0.55757903	0.24296432
cg25417405	SLC27A2;SLC27A2;ATP8B	brown	-0.1563631	-0.4349192	0.29107318	0.29945283	0.60555342	0.02548635
cg01074657	GIPC2;GIPC2	brown	-0.3863359	-0.5416586	0.08045103	0.01475037	0.35212549	-0.0813157
cg09852495	FGF17;FGF17	brown	-0.1480148	-0.5889161	0.13845439	0.20409301	0.69848173	0.04566016
cg27114815	CES4A;CES4A;CES4A;CES	brown	-0.2614363	-0.3301329	0.26155264	0.00688729	0.33780981	-0.0934096
cg03437540	PRR5;PRR5;PRR5;PRR5-	brown	0.16513149	-0.4111245	0.1270551	0.44201713	0.88048707	0.27758996
cg23594693	TFEB;TFEB;TFEB;TFEB;TF	brown	0.03600016	-0.168214	0.18500603	0.33085318	0.53645715	0.12075542
cg13221796	RB1;RB1;RB1;LINC00441;L	brown	0.19672157	-0.2109918	0.0131714	0.60910554	0.66098611	0.27315472
cg01689657	CYP51A1;CYP51A1;CTB-	brown	-0.3120357	-0.5699711	0.19513135	0.04183387	0.52016475	-0.0463059
cg01809281	UBXN2A;UBXN2A;UBXN	brown	-0.0609425	-0.5605234	-0.0524511	0.14325858	0.75200532	0.10153289
cg02201766	RAD50;IL5;IL5;RAD50;RA	brown	-0.2406103	0.09648541	0.34692683	0.01138412	-0.2516499	0.12415399
cg24391460	MAGI2;MAGI2;MAGI2;MA	brown	0.09339787	-0.1232494	0.12025366	0.25343657	0.38109843	-0.0867011
cg25693597	P4HB;P4HB;P4HB;P4HB;P4	brown	0.09977925	-0.4259164	0.09029677	0.50217727	0.87411691	0.21580132
cg09463917	SCARF2;SCARF2	brown	-0.1839927	-0.3532594	0.17141845	0.08302588	0.34225334	-0.0768029
cg02512226	RP11-218D6.4;ARMC4	brown	0.00290348	-0.4477654	0.0758183	0.17586824	0.61037739	0.11522637
cg08813787	SENP2;SENP2;SENP2;SEN	brown	-0.0130966	-0.4062548	0.13091482	0.12313192	0.50420361	0.23828717
cg08799878	AL138795.1	brown	0.14151194	-0.3894663	0.07170125	0.31706308	0.8654196	0.17676866
cg10836848	RP11-447M4.1	brown	0.22361481	-0.2178672	0.44423591	0.43532819	0.66900333	0.31568756
cg08639497	RNPEPL1;RNPEPL1;RNPEP	brown	0.17194995	-0.1866445	0.57882557	0.36522743	0.51204953	0.54745788
cg16023331	ACACB;RP11-443D10.3	brown	-0.1749146	-0.6371364	0.04843445	0.21221515	0.83497898	0.02084329
cg08218437	PHACTR1;PHACTR1;PHA	brown	0.01207691	-0.4640941	0.11944627	0.22298605	0.82339266	0.17006294
cg26530341	RP11-	brown	0.07519038	0.48579092	-0.05745	-0.1784169	-0.5464235	-0.1414291
cg19917412	NAALADL1;NAALADL1;N	brown	-0.1882613	-0.6611674	0.00946835	0.168484	0.6833095	0.1048116
cg19478381	U6;HCG11	brown	-0.0108817	-0.1146283	0.05573434	0.17672077	0.32301674	0.0825796
cg08173709	FOXP1;FOXP1;FOXP1;FO	brown	0.06136306	-0.5021119	0.09299158	0.37662	0.90094541	0.18268771

cg13364410	HAPLN4	brown	-0.0880629	-0.2620211	0.24085391	0.09248039	0.26508525	0.13595457
cg15403218	LRCH4;LRCH4;LRCH4;FB	brown	0.11939065	-0.1999271	0.47396842	0.4070049	0.42065802	0.30608291
cg12683929	ITGA7;ITGA7;BLOC1S1;BL	brown	-0.1210816	-0.675116	0.02652578	0.11280398	0.73219464	0.1382708
cg22197416	STAT3;STAT3	brown	0.34304018	-0.0444925	-0.1056296	0.45625211	0.56872777	0.27411549
cg12412252	PSMG1;PSMG1;PSMG1;T	brown	0.08266279	-0.3547806	-0.0373722	0.4716999	0.71829818	0.08071413
cg04055158	PHF19;PHF19;PHF19;PHF1	brown	0.22678545	-0.1425668	0.29116925	0.5010746	0.55374959	0.29252482
cg18948913	RP11-	brown	0.18946273	-0.1245186	0.08188691	0.58096728	0.49904023	0.33216032
cg26304038	RASGRP2;RASGRP2;PYG	brown	0.10445648	-0.4103009	0.1363267	0.35097225	0.86827789	0.20879347
cg17848367	FOXP1;FOXP1;FOXP1	brown	0.04749401	-0.3746895	0.00538074	0.31774567	0.70594594	0.22361162
cg05157251	PMAIP1;PMAIP1	brown	-0.1469932	-0.3057325	0.07298686	0.16792337	0.44417806	-0.0235421
cg23595516	KIRREL3-AS1	brown	-0.36414	-0.4008935	0.09710505	0.16285757	0.41942733	-0.0465239
cg26815958	RAB5C;RAB5C;RAB5C	brown	0.16832249	-0.4032866	0.14113661	0.31233635	0.85386699	0.31132629
cg13183251	AP000356.2;SNRPD3;SNRP	brown	0.07717432	-0.4586953	0.01859089	0.24880184	0.87012528	0.13501689
cg13265524	ALS2CR11;ALS2CR11;ALS	brown	-0.2207982	-0.5489078	0.07862526	0.22146646	0.73316622	0.03241714
cg24860990	RP11-302J23.1	brown	-0.0618175	-0.0790482	0.04170674	0.28414983	0.34620265	0.06376971
cg17342071	MYEOV2;MYEOV2;MYEO	brown	-0.1317307	-0.2789587	0.14018159	0.10205095	0.34486418	0.06569692
cg13939745	CRYGD	brown	-0.1321332	-0.5230475	0.12453929	0.24106137	0.65050947	-0.0016585
cg23478008	TSPAN31;TSPAN31;TSPA	brown	0.11568905	-0.4018649	-0.0396784	0.4350705	0.85926359	0.20060061
cg08664487	BEND6	brown	-0.2206289	-0.3407757	0.19431155	0.1373529	0.40190633	-0.0175009
cg12883767	ARHGEF25;AC025165.8;AC	brown	-0.0956701	-0.397806	0.22431655	0.15764046	0.43121598	0.12753659
cg01323801	DNASE1;DNASE1;DNASE	brown	0.21819363	-0.1503191	0.37909255	0.33197932	0.4824626	0.41920726
cg22731751	ADAP1;ADAP1;COX19	brown	-0.0301579	-0.3359645	0.03061229	0.18657399	0.52577943	0.14615734
cg05620036	UROC1;UROC1	brown	0.02558436	-0.3559817	0.15846665	0.37804546	0.69350882	0.14186907
cg20006715	EFHB;EFHB;EFHB;EFHB;E	brown	-0.1266261	-0.2194436	-0.0077183	0.12335633	0.3233973	0.04074179
cg27635797	FAM5C;FAM5C;FAM5C;F	brown	-0.2053595	-0.5907161	-0.0227333	0.1851562	0.65182552	0.06929641
cg23126099	KCNS3;KCNS3;KCNS3;KC	brown	0.19326606	-0.0624111	0.00418922	0.20639114	0.34266138	0.22977958
cg14295110	SLCO3A1;SLCO3A1;SLCO	brown	0.00837526	-0.2846406	0.19566733	0.44116171	0.53236403	0.13680052
cg05593775	PDZD7;PDZD7	brown	0.15562308	-0.2509538	0.18684335	0.37374712	0.49022865	0.20495624
cg02835981	BNIP3;BNIP3	brown	-0.1250646	-0.4749832	0.02291661	0.09863008	0.58097065	0.03469162
cg23295636	Y_RNA;RP11-	brown	0.13943616	-0.1677536	0.05861019	0.30440566	0.51421141	0.2099217
cg03333737	MBNL1;MBNL1;MBNL1;M	brown	-0.1700086	-0.4506362	0.11354214	0.24677453	0.65069657	-0.087995
cg07021228	ZNF619;ZNF619;ZNF619;Z	brown	0.16094208	-0.3986635	0.17450606	0.34977336	0.75762866	0.22348513
cg08867413	PITX2;PITX2	brown	-0.1841124	-0.3384383	0.1376353	0.06285272	0.34538346	-0.0206115
cg19731122	MYO1C;MYO1C;MYO1C;	brown	-0.0483796	-0.26017	0.4401242	0.3774477	0.41638827	0.22842755
cg00904258	ADAP1;ADAP1;ADAP1;A	brown	0.04447582	-0.4454646	0.07245827	0.20968556	0.78487847	0.13809253

cg26224354	GPR146;GPR146;C7orf50;G	brown	0.13193838	-0.1943862	0.18767505	0.21463612	0.43253811	0.41250802
cg04644846	CHKB-	brown	0.10582194	-0.4468239	0.10903111	0.33215826	0.85835458	0.25402459
cg03952568	CLDN12;CLDN12;CLDN12;	brown	-0.1234029	-0.3364228	0.12058006	0.20172979	0.45869501	0.15856171
cg02133084	MIR101-2	brown	0.36228514	0.03426285	0.15669578	0.60686631	0.45245068	0.30044268
cg26705986	RP11-	brown	-0.1362477	-0.4935768	0.19762935	0.21153477	0.622143	0.10583887
cg22117172	CYP51A1;CYP51A1;CTB-	brown	-0.3177289	-0.5152677	0.12263876	0.03229619	0.53030991	-0.1260945
cg19034770	RP11-211C9.1;MFHAS1	brown	0.05792056	-0.3580398	0.09877322	0.22731883	0.52311551	0.18059041
cg18127680	LPP;LPP;LPP;LPP;LPP	brown	-0.0113347	-0.5392979	0.13319864	0.238174	0.73430944	0.2049338
cg10633911	AC004041.2;IL13;AC004041	brown	0.09985575	0.01610633	0.42405264	0.39471096	0.30180068	0.2410612
cg14091353	ITGAL;ITGAL;ITGAL;RP11-	brown	0.18241555	-0.2198612	0.18404904	0.40339126	0.61007171	0.34478942
cg15855900	CA7;CA7;NAE1;RP11-	brown	0.11305461	-0.2755864	0.35587123	0.3640701	0.49450308	0.28478365
cg14485996	PRMT8	brown	0.06598107	0.58269648	0.17731245	-0.1357233	-0.7243229	0.0086395
cg03919092	VDAC2	brown	0.14249857	-0.4141421	0.07125362	0.40155941	0.86912999	0.28344751
cg02972988	YPEL4;YPEL4;AP000662.4;	brown	0.0445178	-0.4006844	0.06943164	0.27034621	0.60920959	0.16812651
cg25935178	CCDC19;CCDC19;CCDC19;	brown	0.03437099	-0.1933449	0.20446428	0.25967837	0.36771102	0.04702207
cg18413900	RP11-	brown	0.16997784	-0.4185632	0.14875803	0.324636	0.88322754	0.29206718
cg04993267	GRPEL2;GRPEL2;GRPEL2	brown	-0.2280509	0.06774569	0.46517155	-0.0742407	-0.2460931	0.06554356
cg11835024	ATXN7L1;ATXN7L1;ATXN	brown	0.21716422	-0.2522529	0.07503014	0.26229295	0.69563605	0.31993911
cg17552829	C14orf164;C14orf164;SLC7	brown	0.08104266	-0.4244624	0.00231699	0.28564401	0.66918812	0.12680653
cg16335858	GYLTL1B;GYLTL1B	brown	0.21368532	-0.1243853	0.09728484	0.43046006	0.49175606	0.26387835
cg23301164	NFATC3;NFATC3;NFATC3	brown	0.18352489	-0.2377439	0.25232418	0.35552382	0.5956946	0.37395598
cg05455433	F12;F12;GRK6;GRK6;F12	brown	0.20884733	-0.1395113	0.44190218	0.43973469	0.53343269	0.29000155
cg14776134	GALNT6;GALNT6;SLC4A8	brown	0.13867626	-0.4232682	0.13206034	0.42946272	0.77152419	0.23095706
cg09684846	FAM18A;FAM18A;FAM18	brown	0.13781067	-0.29353	0.20420135	0.39276081	0.6067947	0.17681475
cg06593258	OSBPL1A	brown	-0.0735827	-0.6351547	-0.114215	0.10093516	0.74840056	0.15689182
cg15071067	RN5S97;DGUOK-	brown	0.21162833	-0.2687949	0.07245786	0.53486735	0.37515662	0.29529421
cg13130783	SLC24A6;SLC24A6	brown	0.02424406	-0.4886456	0.19216362	0.35277283	0.82043598	0.18075036
cg00435064	PGS1;PGS1	brown	-0.0848507	-0.0795606	0.29618734	0.30492282	0.33740386	0.0827023
cg04328562	CNN1;CNN1;CNN1;CNN1;	brown	-0.2292792	-0.6048839	0.01348334	0.11984849	0.67355612	-0.0005717
cg25888160	PHF21A;PHF21A	brown	0.38822932	-0.1511008	0.01247476	0.55595474	0.64513746	0.38508907
cg24921262	SMPD3;SMPD3;SMPD3;S	brown	-0.3056323	-0.0390277	0.26117837	0.00818011	-0.3366055	0.19556084
cg08158976	MRPL55;MRPL55;MRPL55;	brown	-0.1582696	-0.4945276	0.10576206	0.1957112	0.70678168	0.03773348
cg13468667	TGIF1;TGIF1;TGIF1;TGIF1;	brown	-0.170099	-0.2296581	0.25402108	0.00339909	0.30682951	0.02224138
cg24089970	SIL1;SIL1;SIL1;SIL1;SIL1;SI	brown	0.02348134	-0.5078364	0.15141552	0.38183865	0.9154086	0.18133894
cg08643131	MUM1;MUM1;MUM1	brown	0.12612785	-0.3390722	0.17594692	0.33993242	0.70250302	0.31389027

cg08980255	SNORA64;MYRIP;MYRIP;	brown	0.36773905	-0.0341303	-0.2907184	0.12148596	0.32327054	-0.0220459
cg24241662	RXRA;RXRA;RXRA	brown	-0.0096581	-0.4412281	0.27469856	0.2173915	0.74265584	0.17054747
cg05725721	B3GALNT1;B3GALNT1;B3	brown	-0.3414603	-0.6182763	0.04234614	0.11515594	0.6267474	-0.0337351
cg02321886	PFKFB4;PFKFB4;PFKFB4;	brown	0.14911817	-0.2402952	0.08827485	0.43558824	0.6285424	0.2591062
cg07257300	NEK6;NEK6;NEK6	brown	-0.0892932	-0.4905573	0.07309571	0.12880752	0.60246836	0.12910654
cg16987606	GPRC5C;GPRC5C;GPRC5C	brown	-0.1187852	-0.2426256	-0.0141175	0.34444452	0.50572961	-0.0573675
cg17453778	CASR;CASR;CASR	brown	-0.2331602	-0.6437653	0.2477441	0.09177497	0.63042281	0.10985951
cg17796656	CTD-	brown	0.20607264	0.18393364	0.35211785	0.31461695	0.30000992	0.21847737
cg19515035	RTKN;RTKN;RTKN;RTKN;	brown	-0.1490237	-0.4279564	0.09819124	0.05186116	0.52746576	-0.0451303
cg15231233	DOK4;DOK4;DOK4;DOK4;	brown	0.34398067	-0.0370903	0.24584221	0.54561719	0.487789	0.37358634
cg22486834	RN7SK;GSTA4;GSTA4;GS	brown	0.13296889	-0.385717	0.16217212	0.30829658	0.70417914	0.24476273
cg09476130	CCDC19;CCDC19;CCDC19	brown	0.09436048	-0.3506732	0.12496629	0.32434137	0.59597184	0.15130678
cg24615088	TGFB3;TGFB3;IFT43	brown	-0.1465889	-0.3808624	0.02376375	0.23521351	0.55287408	-0.0065376
cg00148411	FBXO18;ANKRD16	brown	-0.0136791	-0.3254666	0.15109899	0.22430686	0.49308184	0.17360757
cg02565235	MIAT;MIAT	brown	0.16499261	-0.0183621	0.34649587	0.43951489	0.356498	0.21571229
cg20157666	DPH1;DPH1;DPH1;DPH1;D	brown	0.19707255	-0.263615	0.1446176	0.29068492	0.67305301	0.30649922
cg02458589	YY1AP1;YY1AP1;YY1AP1	brown	0.08167114	-0.382592	-0.0439158	0.50419272	0.72789948	0.29260535
cg17695841	SLC35C1;SLC35C1;SLC35C	brown	0.19416414	-0.3679388	0.23497291	0.47707085	0.79294022	0.24711464
cg11360546	GPR146;GPR146;GPR146;G	brown	0.17011111	-0.2325912	0.20249112	0.46557863	0.63292085	0.3598083
cg11580702	ARPC1B;ARPC1B;ARPC1B	brown	0.06267682	-0.4454645	0.15743621	0.37110795	0.88163986	0.18470599
cg22705929	AP000662.4;AP000662.4;AP	brown	-7.89E-05	-0.5092503	-0.0231044	0.23412025	0.89535978	0.17070417
cg04632887	AVP	brown	0.14861241	-0.3119963	0.16804148	0.24405548	0.57941773	0.24363554
cg05259872	PALLD;PALLD;PALLD	brown	-0.0871567	-0.3287996	0.22921673	0.19589539	0.40260299	0.09908605
cg00064020	AP2M1;AP2M1;AP2M1;AP	brown	0.10675503	-0.2377346	0.10896766	0.14041215	0.40206422	0.24784366
cg25249383	TIMM10;TIMM10;TIMM10	brown	0.0511332	-0.2340575	0.12750893	0.4388092	0.47920842	0.11596681
cg03292675	EPB49;EPB49;EPB49;EPB49	brown	-0.0807808	-0.4297545	0.08582738	0.25226215	0.55003805	0.18453494
cg23572813	ADAMTSL5;ADAMTSL5;A	brown	0.24453619	-0.0774032	0.35712251	0.22462814	0.37303236	0.3685455
cg19997577	DBN1;DBN1;DBN1	brown	0.10396792	-0.424127	0.07230886	0.3446063	0.86133893	0.18292081
cg13399816	GNG12;GNG12;RP11-	brown	-0.2470896	-0.3969409	0.17400606	0.16143393	0.41020275	0.05787303
cg02503303	PICALM;PICALM;PICALM	brown	0.28102854	-0.1758608	0.08305765	0.69662436	0.66738038	0.2476778
cg12600901	TPM3;MIR190B	brown	0.01311644	-0.0833612	0.10481369	0.24620808	0.52595324	0.14224713
cg07531374	SSH1;SSH1;SSH1;DAO	brown	-0.1178526	-0.7121583	0.03539858	0.18192644	0.85166684	0.12688196
cg10302347	IFT140;TMEM204;IFT140;IF	brown	0.25629055	-0.1734973	0.15941701	0.46432099	0.67569753	0.24856282
cg24778858	RAPGEFL1;RAPGEFL1;RA	brown	0.13255587	-0.141637	0.22779359	0.46363622	0.57749373	0.25257531
cg23579062	DNAI1;DNAI1;DNAI1;DN	brown	0.07215611	-0.4600534	0.38354208	0.31734964	0.72975915	0.29218703

cg01329511	SMYD5;SMYD5;SMYD5;S	brown	0.0306778	-0.2639734	0.09906092	0.25240163	0.57492948	0.32276956
cg02844030	CORO1C;CORO1C;CORO1	brown	0.17979734	-0.2879085	0.24059864	0.38000436	0.67944371	0.25281219
cg14760424	LGALS8;LGALS8;LGALS8;	brown	0.08382238	-0.2509734	0.22343352	0.3181656	0.47344341	0.2938247
cg20726646	GRTP1;GRTP1;GRTP1	brown	-0.2336196	-0.3444289	0.20106365	0.03265548	0.35910894	-0.0568913
cg04494517	MCMBP;MCMBP;MCMBP;	brown	0.07048757	-0.350942	-0.0223427	0.49515641	0.74108181	0.05181611
cg17741866	ZFYVE1;ZFYVE1;ZFYVE1	brown	-0.1208955	-0.3696639	0.09438011	0.16197501	0.54837141	0.01913162
cg22662233	ZNF708	brown	0.09869064	0.17590779	-0.0590741	-0.1586293	-0.2825593	0.01485463
cg21978242	LNX1;LNX1-AS1;LNX1-	brown	0.16298372	-0.3875747	0.05545075	0.32060014	0.84846856	0.23872913
cg10667794	CORO1C;CORO1C;CORO1	brown	-0.0832979	-0.5301849	-0.0614632	0.26039994	0.68572863	0.13934371
cg05995607	MS4A6A;MS4A6A;MS4A6	brown	-0.1923956	-0.5797892	0.00859694	0.19046277	0.81110563	0.03655422
cg10820206	SH3GLB2;SH3GLB2;SH3G	brown	0.29485579	-0.0661459	0.27930309	0.35103819	0.50423157	0.21729316
cg15640925	TRIOBP;TRIOBP;NOL12;T	brown	0.30542707	-0.1498084	0.25204186	0.4393696	0.59482981	0.38192222
cg05058246	CTD-2195M18.1;CTD-	brown	-0.1718561	-0.3094869	0.20649061	0.08274896	0.3404916	0.10315482
cg08373624	AIG1;AIG1	brown	-0.002417	-0.5475061	-0.0918615	0.27780091	0.85996305	0.1600573
cg01777397	CLIP4;CLIP4;CLIP4;CLIP4;	brown	-0.220356	-0.3855474	0.18505074	0.11079484	0.39966165	0.09561157
cg26514552	C4BPA;C4BPA;C4BPA	brown	-0.0293108	-0.5229019	-0.0879404	0.23371604	0.77737483	0.11074511
cg22442730	SRMS	brown	-0.0698613	-0.338168	0.17785233	0.3128872	0.57742146	0.09427657
cg17975763	ZNF296;ZNF296	brown	0.24072011	-0.2475771	0.1583419	0.53905707	0.73070716	0.33292872
cg19388776	PROK2;PROK2	brown	-0.1944886	-0.6119373	0.10542927	0.08438872	0.68796676	0.03917108
cg10611365	EPB49;EPB49;EPB49;EPB49	brown	-0.1561156	-0.5312366	0.12165311	0.16956297	0.61640612	0.02627348
cg13027595	TAPBP;TAPBP;TAPBP;TAP	brown	0.14846859	-0.1896887	0.25465982	0.32375473	0.53031116	0.31763856
cg04972046	C6orf123;C6orf123	brown	-0.020661	-0.2971489	0.22879401	0.14798747	0.34905548	0.23837518
cg08613448	PPP2R1B;PPP2R1B	brown	0.24145913	-0.1781581	0.25754866	0.30747752	0.5922395	0.19839904
cg20830848	C7orf49;C7orf49;C7orf49;C7	brown	0.21797113	-0.1690557	0.2095863	0.36619578	0.6385668	0.2930027
cg06969845	HRH2	brown	0.06999061	-0.1447561	0.10524679	0.40960711	0.50389951	0.28089854
cg05835416	CYP2C18;CYP2C18;CYP2C	brown	0.22744268	-0.1012777	0.13362537	0.35942362	0.43969869	0.40625784
cg04701420	S1PR1;S1PR1;S1PR1;RP4-	brown	-0.4025206	0.00949601	0.06394032	-0.0814291	-0.3075188	-0.2007187
cg17691598	JUP;JUP;JUP;JUP;JUP	brown	0.082186	-0.3300388	0.14923779	0.23465102	0.59734456	0.22431899
cg03468827	TLE6;TLE6;TLE6;TLE6	brown	0.00234679	-0.1807367	0.08925876	0.18396784	0.3689475	0.18783962
cg13789015	LINC00094;LINC00094;LIN	brown	-0.0046125	-0.1775983	0.18509854	0.21963254	0.32132958	0.2052867
cg11245806	RP11-644K8.1;RP11-	brown	0.0055879	-0.3916863	-0.0522169	0.20133545	0.46862582	0.11074142
cg11590860	RP5-1187M17.10	brown	0.042108	-0.4605621	0.1188502	0.24303832	0.66436295	0.1152551
cg12803592	DEF8;DEF8;DEF8;DEF8;DE	brown	0.21424955	-0.2987605	0.00717362	0.34569082	0.70124104	0.34316082
cg24059033	TPTE;TPTE;TPTE;TPTE	brown	0.09681047	-0.2592544	0.01551687	0.06629487	0.3498287	0.056595
cg00151675	GLYCTK;GLYCTK;GLYCT	brown	0.24419427	0.00281527	0.47789367	0.40301879	0.37361336	0.35067168

cg13646005	MUM1;MUM1;MUM1;MU	brown	0.22655992	-0.2022612	-0.0189123	0.47557815	0.63478509	0.26655396
cg08521322	BVES;BVES;BVES-	brown	-0.0334954	-0.3391441	0.00867373	0.13973425	0.38121852	0.08757932
cg01270444	LRRD1;LRRD1;LRRD1;LRR	brown	-0.0420518	-0.4711538	0.0179882	0.24336504	0.69016083	0.10082376
cg01916632	PDSS2;PDSS2;PDSS2	brown	0.0648386	-0.5154503	0.08738048	0.31017116	0.82053314	0.21580157
cg22708150	LY6G5C;LY6G5C;LY6G5C;	brown	0.22616396	-0.2541048	0.04632837	0.42382254	0.71969988	0.19195424
cg15569276	CTD-	brown	0.19509486	-0.0445474	0.02924113	0.40714556	0.37232075	0.20720321
cg10628205	NFIA;NFIA;NFIA;NFIA;NF	brown	-0.1404221	-0.3391089	0.14722625	0.30968677	0.50794015	0.1000312
cg13396318	RIN2	brown	0.32728802	0.12961157	-0.0070642	0.17946428	0.26254375	0.09204345
cg04859800	IL1A	brown	0.17956531	-0.2507164	0.15000359	0.28697323	0.52543188	0.30551292
cg22201537	SNX19;SNX19;SNX19;SNX	brown	0.22803507	-0.2203086	0.07236868	0.41672718	0.73257215	0.25150339
cg21535253	MAN2B1;MAN2B1;MAN2	brown	0.09978309	-0.222535	0.08348527	0.13783565	0.43006181	0.26542158
cg12922892	KIAA1324;KIAA1324;KIA	brown	-0.0151389	-0.5396106	-0.0549516	0.2544164	0.88201949	0.05666167
cg23579306	SLC11A2;SLC11A2	brown	-0.0852469	0.26492531	0.08090125	0.13497092	-0.371979	0.01993013
cg25453800	AC034228.2;ACSL6;ACSL6;	brown	0.26300849	-0.0968868	0.43465493	0.49808776	0.47167434	0.31700809
cg24714082	RP3-455J7.4	brown	0.22064227	-0.3291546	0.01663204	0.57195001	0.78915355	0.24850794
cg15718572	CR1L;CR1L	brown	-0.2053827	-0.2890377	0.17687115	0.25555894	0.35599652	0.04900816
cg07948034	ZIC2;ZIC2;ZIC2	brown	0.09420864	-0.2130225	-0.3179993	-0.0416462	0.29552255	-0.004103
cg13168774	PFKP;PFKP;PFKP	brown	-0.0998089	-0.5843638	0.22065962	0.21520874	0.68560773	0.25590089
cg18144560	TAPBP;TAPBP;TAPBP;TAP	brown	0.01940787	-0.2862822	0.30972358	0.31131721	0.4973165	0.23575629
cg18960218	SLC7A7;SLC7A7;SLC7A7;S	brown	0.18824208	-0.1994325	0.21008872	0.26341372	0.61395031	0.27786576
cg24409634	CCDC153;CCDC153;CCDC	brown	0.27442947	-0.3000587	0.04359287	0.55552324	0.83745363	0.27068233
cg02106174	CYBASC3;CYBASC3;CYB	brown	-0.0294973	-0.4463818	0.05589622	0.17421041	0.7306625	0.08529881
cg07979387	GTPBP1;GTPBP1;GTPBP1;	brown	-0.4001949	0.10838843	0.20487805	0.13957123	-0.1883086	-0.1108914
cg15677294	TUBB4A;TUBB4A;TUBB4	brown	0.13637043	-0.1088294	0.34839112	0.43183932	0.44476262	0.34442398
cg00439384	PDIA5;PDIA5;PDIA5	brown	0.04801739	-0.3296082	0.08492378	0.28494624	0.67480019	0.07917134
cg10467021	ZMYND8;ZMYND8;ZMYN	brown	0.02388914	-0.5240685	0.20723726	0.39052881	0.87087305	0.18436751
cg11904114	AL451051.1	brown	0.10503991	-0.123569	-0.0357506	0.49806568	0.61076831	0.15950767
cg17014757	CHI3L1	brown	-0.076274	-0.2235181	0.10171153	0.10224576	0.35029425	-0.0268398
cg12188860	TOP1MT;TOP1MT;TOP1M	brown	0.14844339	-0.2046482	0.22252904	0.26334454	0.3556494	0.36222413
cg16693881	SETD1A;SETD1A	brown	0.18693224	-0.2755158	0.04470389	0.37781768	0.70720768	0.27220921
cg22806403	LMAN1L;LMAN1L;LMAN1	brown	0.06670719	-0.0734288	0.25021314	0.37546465	0.52389539	0.29052841
cg03080569	INHBC;INHBC	brown	0.15082124	-0.2317017	0.23765796	0.30593241	0.67173258	0.28752017
cg25563983	CECR7;CECR7	brown	-0.1502215	-0.3302601	0.26767689	0.15767099	0.35645792	0.20253881
cg26655325	SERPINB2	brown	0.17314241	-0.2397404	-0.1209174	0.41993976	0.76408869	0.15485737
cg17931078	SFTPB	brown	-0.0195155	-0.2861672	0.14071911	0.33100428	0.64050674	0.18742533

cg23310078	INPP5B;INPP5B;INPP5B;IN	brown	0.11052474	-0.2112517	0.12557528	0.1426003	0.59396774	0.21082441
cg03340041	RP11-96L14.7;RP11-	brown	0.23628707	-0.236387	0.35070047	0.34707153	0.70184523	0.41249055
cg22077691	PRKRIR	brown	0.20512882	-0.3069071	-0.0400261	0.28615821	0.80034822	0.26212847
cg10690887	ABHD3	brown	-0.3595615	0.044419	0.09675548	-0.1596532	-0.3687642	-0.0778791
cg04027040	SUPT3H;SUPT3H;SUPT3H	brown	0.13344629	-0.4142087	0.40470662	0.29305144	0.68193585	0.30823376
cg13476778	FRMD6;FRMD6;FRMD6;F	brown	0.04276318	-0.2215422	0.15413739	0.15716063	0.30777729	0.18961676
cg21500300	RP11-	brown	0.11919987	-0.2689773	0.03705632	0.33099496	0.73933683	0.19460029
cg00508575	ATP2B1;ATP2B1;ATP2B1;	brown	0.17146799	-0.2275372	-0.091	0.20625561	0.51972881	0.16748924
cg20564709	XXYLT1;XXYLT1	brown	0.01855082	-0.5289228	-0.1048582	0.33356374	0.92046969	0.05828157
cg21416022	TTC23;TTC23;TTC23;TTC23	brown	-0.029776	-0.3552092	0.04672586	0.05073244	0.44039366	0.08469403
cg13687422	DYRK4;DYRK4;DYRK4;D	brown	-0.03101	-0.5275679	0.00893251	0.26855167	0.83575976	0.10668022
cg04728863	RP11-517O13.3	brown	0.11254982	-0.3275794	0.03244953	0.31660693	0.7169518	0.1806542
cg15560375	SCPEP1;SCPEP1;RP5-	brown	0.07944535	-0.4724607	0.10735976	0.41951016	0.82091242	0.18934576
cg26511386	TMEM139;TMEM139;TME	brown	0.17073817	-0.3848304	0.15297319	0.28402391	0.82998944	0.19741922
cg13523587	NISCH;NISCH;NISCH;TNN	brown	-0.4504425	-0.012745	0.25584283	0.00588411	-0.2294041	-0.1470105
cg13876222	NOTCH1	brown	0.16791135	-0.3687731	0.16085664	0.32878418	0.71403973	0.33535219
cg09824731	GRM4	brown	-0.0646876	-0.4652788	0.16845445	0.20377948	0.49370869	0.29645371
cg17161421	PPT2;PRRT1;PPT2;PPT2;PP	brown	-0.1118049	-0.4348426	0.18583557	0.17826922	0.54217151	0.10573388
cg26686009	C10orf25;ZNF22	brown	0.16791753	-0.2566258	-0.0045453	0.2167422	0.54354721	0.16930284
cg19836999	ESRP2;ESRP2;ESRP2;ESRP	brown	0.12353474	-0.413089	0.27407303	0.33184525	0.61164126	0.28665414
cg23214480	APBB1;APBB1;APBB1;AP	brown	-0.2715706	0.04007065	-0.2858806	-0.2517764	-0.2471063	-0.3432626
cg13126552	DNAH1;DNAH1;DNAH1	brown	-0.0072292	-0.1803042	0.14968013	0.21740445	0.35120856	0.15335724
cg17628249	C17orf64;C17orf64;C17orf64	brown	0.1359763	-0.4213224	0.1446658	0.33074574	0.78522752	0.25523622
cg20205586	SRSF7;SRSF7;SRSF7;SRSF	brown	-0.1398629	0.21240255	0.31139034	0.10164831	-0.4102435	0.10132813
cg26486466	ZNF300;ZNF300;ZNF300	brown	-0.0645124	-0.4948007	0.35093963	0.12193256	0.49135252	0.10026397
cg11499386	PPAP2C;PPAP2C	brown	0.01929292	-0.2605516	0.34340076	0.15206394	0.35164426	0.16861003
cg27553890	SSH1;SSH1;SSH1;SSH1	brown	0.20284709	-0.1158288	0.310864	0.37660778	0.37706702	0.26858034
cg26556011	ANKRD13D;ANKRD13D;A	brown	0.1933367	-0.3712181	0.15661172	0.42310833	0.79769439	0.33130209
cg00099427	CCDC19;CCDC19;CCDC19	brown	0.27524492	-0.1504678	0.09023887	0.43097848	0.59041976	0.2331624
cg12729822	RPS6KA1;RPS6KA1;RPS6	brown	0.13506965	-0.4375148	0.12892888	0.32727828	0.84653007	0.26135724
cg05124136	THNSL2;THNSL2;THNSL2	brown	0.2196645	0.18414083	0.23531147	0.38356187	0.25559343	0.25230499
cg03763997	MSRB2	brown	0.06920219	-0.1697798	0.16993642	0.27483866	0.44122536	0.15446549
cg16795804	EIF2AK2;EIF2AK2;EIF2AK	brown	-0.2611131	-0.3657877	0.14475434	0.17138107	0.41156257	-0.0286707
cg27522205	GALNT6;GALNT6;SLC4A8	brown	-0.1051947	-0.3820421	-0.0265172	0.25318235	0.47753352	0.04488352
cg11591636	ZIC5	brown	-0.0767519	-0.4344132	-0.0746457	0.01993231	0.4371789	0.0356967

cg09662920	GIPC2;GIPC2	brown	-0.2682938	-0.2821587	0.17080687	0.05837388	0.20927782	-0.2415113
cg07697815	ANKRD24	brown	-0.0985623	0.30558792	0.35078053	0.06931031	-0.2598533	-0.0198913
cg07741676	POMGNT1;POMGNT1;PO	brown	0.353067	-0.1981417	0.32626653	0.62080726	0.63826942	0.40059868
cg15493222	COX6C;COX6C;COX6C;CO	brown	-0.073277	-0.1553381	0.21133935	0.28998773	0.30313937	0.10604729
cg15982002	FDFT1;FDFT1;FDFT1;FDF	brown	-0.0752322	-0.3854055	0.1292482	0.05595572	0.45024842	0.18774348
cg24174962	LONP1;LONP1;LONP1;CA	brown	-0.0883844	-0.2757133	0.13379527	0.21334681	0.32394797	0.17341508
cg20939768	TRIM17;TRIM17;TRIM17;T	brown	-0.0023976	-0.3216739	0.30983653	0.25562901	0.43047293	0.20946366
cg15773296	SYNE2;SYNE2;SYNE2;ESR	brown	-0.0013806	-0.2544796	-0.1796826	0.21183423	0.52070988	0.01151017
cg25150199	SNRNP25;POLR3K;SNRNP	brown	-0.294497	0.06726165	0.15446105	0.00753292	-0.2332342	-0.1954387
cg12123199	CALML4;CALML4;CALML	brown	0.04431643	-0.4993268	0.17715502	0.33914885	0.80252768	0.19489109
cg23152216	KRAS;KRAS;KRAS;KRAS	brown	0.18243818	-0.1919646	-0.2879991	0.20297256	0.67032367	0.12661009
cg17417856	PRMT1;PRMT1;PRMT1;C19	brown	0.13686416	0.01812301	0.17897003	0.33350583	0.38461336	0.28044065
cg08699196	ITGB7;ITGB7;ITGB7;ITGB7	brown	0.15066541	-0.4384227	0.23635948	0.27937336	0.78661226	0.30996208
cg17168157	RGS22;RGS22;RGS22;RGS	brown	0.01044546	-0.2153947	-0.0206681	0.22468076	0.45490395	0.12324409
cg17822007	PLCH1	brown	0.04174644	-0.3565081	0.21094449	0.2382204	0.47214614	0.0900193
cg01291856	FAM99B	brown	-0.2500244	0.26305206	0.42786604	0.07789068	-0.3190022	-0.0755694
cg09112636	RP11-231P20.2;RP11-	brown	-0.0700135	-0.2734433	0.05551039	0.24085179	0.48726387	0.07512681
cg20659967	RP4-788L20.3	brown	-0.0076907	0.29736787	0.02451599	0.00359502	-0.318697	-0.1301462
cg14599440	MPPE1;MPPE1;MPPE1;MP	brown	-0.1010153	-0.1999253	-0.0294149	0.23517849	0.50131727	0.06979156
cg14541311	MYH6;MYH6;MYH6	brown	0.13972695	0.00867146	0.35813152	0.39015197	0.33113797	0.27641078
cg06843131	SLC2A8;SLC2A8;SLC2A8;S	brown	0.14971314	-0.2622954	0.01479045	0.60151764	0.67455004	0.2182345
cg06991565	SAPCD1-AS1	brown	0.20796669	0.00983333	0.38127194	0.33052487	0.26934891	0.26698478
cg00637015	PUM1;PUM1;PUM1;PUM1;	brown	0.17769826	-0.3665069	0.01165834	0.27354813	0.7822458	0.24694965
cg25538809	MAP2K3;MAP2K3;MAP2K	brown	0.30920104	-0.0194455	0.39236422	0.40284686	0.49410816	0.43181894
cg07127410	ZNRF3-AS1;ZNRF3	brown	0.13607201	-0.0066522	0.21507643	0.20867415	0.31745058	0.25895489
cg05773743	NR5A1;NR5A1	brown	0.18697719	-0.4004189	0.01513254	0.44690503	0.83797272	0.28506924
cg24845165	LPIN3;LPIN3	brown	0.21422304	-0.2387598	0.19278004	0.35730445	0.56326959	0.36262378
cg18060905	IGHJ3;IGHJ2;IGHJ1;IGHD7-	brown	0.40755851	0.08342524	-0.0563982	0.15062938	0.37552714	0.06421697
cg04632310	AL590138.1	brown	0.17197564	-0.2874123	0.18224816	0.54126423	0.71854523	0.32851983
cg15439725	ZNF732	brown	0.09173962	-0.2903832	0.13563155	0.09198636	0.47481083	0.13203702
cg18305324	PRRC2A;SNORA38	brown	0.2756306	-0.2913902	0.16238601	0.52064962	0.78007976	0.37590268
cg20825023	RP11-54O7.1	brown	0.00975045	0.19809341	0.40307974	0.07507023	-0.2148551	0.23438474
cg04617914	FOXP4;FOXP4;RP11-	brown	0.18086298	-0.0638188	0.30572917	0.43156341	0.50689287	0.24204404
cg03242458	TRIM15;TRIM15	brown	0.07719588	0.04606195	-0.1919716	0.30402571	0.27431234	0.16344826
cg07138491	FAM183A;FAM183A;FAM	brown	-0.1274798	-0.2503814	0.15573918	0.08439137	0.33863435	0.03135833

cg04823169	TRIM14;TRIM14	brown	0.11304065	-0.3515514	0.23760802	0.13714378	0.56450869	0.16512782
cg11492403	RUNDC1;AARSD1;AARSD	brown	0.37845993	0.09051807	0.49526572	0.54351373	0.32750388	0.47723267
cg07068322	C8orf45;C8orf45;C8orf45;C8	brown	-0.1132953	-0.1366416	-0.2297702	0.22100634	0.32332668	0.0545879
cg04887468	OSBPL5;OSBPL5;OSBPL5;	brown	0.22832217	-0.0391925	0.52270968	0.44489407	0.41777918	0.32682419
cg20485874	LRRC17;LRRC17;LRRC17;F	brown	0.12656627	-0.2816943	0.06522237	0.14682584	0.46145074	0.09106925
cg04767060	PIK3C2B;RP11-739N20.2	brown	0.21392857	-0.1889389	0.06275457	0.40300288	0.56194814	0.24837877
cg07700552	RP11-613M10.6	brown	0.24318946	-0.1669777	0.36703224	0.55213571	0.51608162	0.43242544
cg11240129	CCDC17;CCDC17;CCDC17;	brown	0.2144776	-0.0582065	0.35189739	0.24751617	0.31475578	0.19116967
cg08557347	CTC-527H23.2;N4BP1	brown	-0.0302721	-0.5455396	-0.0347934	0.34431726	0.9216293	0.09254016
cg16194834	PAIP1;PAIP1;PAIP1;PAIP1;	brown	-0.1357842	-0.3750463	0.12416322	0.29298229	0.60622384	-0.05243
cg09654136	CAMTA2;CAMTA2;CAMT	brown	0.15939563	-0.3415727	0.1431507	0.25371085	0.6325691	0.28832558
cg09614479	RP11-40F6.2;RP11-	brown	-0.0367623	-0.2094805	-0.0464346	0.14970749	0.36973133	-0.0031377
cg20808711	GPSM3;GPSM3;GPSM3;GP	brown	-0.0207016	-0.4267263	0.23001009	0.26135727	0.61931411	0.22440804
cg25413538	RP11-	brown	0.17290451	-0.1700801	0.17121867	0.24658767	0.32949613	0.14846724
cg17279839	RARRES2;RARRES2;RP4-	brown	-0.057053	-0.3919353	0.05893977	0.14157779	0.51777203	0.0281382
cg06246950	SLC35E4;SLC35E4;SLC35E	brown	-0.0868774	-0.3044787	0.20052659	0.22304267	0.51911572	0.00997418
cg14091103	LPP;LPP;LPP;LPP;LPP	brown	0.16178619	-0.3184992	0.13096624	0.39100569	0.67568185	0.25723988
cg14602697	RAD9A;RAD9A;RAD9A;R	brown	0.0625531	-0.0624725	0.35080357	0.45964589	0.38649351	0.23955438
cg17462329	P4HB;P4HB;P4HB;P4HB;P4	brown	0.24156305	-0.2926094	0.05933387	0.47377759	0.7317519	0.22226328
cg05210497	TIGD3	brown	-0.1164185	-0.4994426	0.13819942	0.14317656	0.59004603	0.09538999
cg19620086	SLC38A2;SLC38A2;SLC38	brown	0.23841691	-0.2836159	-0.0687013	0.35753441	0.59183908	0.24897357
cg12022583	WIPF3	brown	-0.0948214	-0.1565236	0.13594075	0.23701897	0.32168333	-0.0061472
cg16446736	ZBTB46;ZBTB46	brown	-0.0152402	-0.2170863	0.32497595	0.27880469	0.40786179	0.41147408
cg23509369	PSMD3;PSMD3;PSMD3	brown	0.2305492	-0.1242958	0.27802028	0.50534781	0.55545887	0.30811072
cg01878807	DHRS4;DHRS4;DHRS4;DH	brown	-0.0063735	-0.4637406	0.21738248	0.33715493	0.73611449	0.17976936
cg14654731	TMEM155;AC079341.1;TM	brown	0.02305617	-0.3663583	0.07346875	0.18169931	0.47434559	0.24433263
cg23266308	IL17RE;IL17RE;IL17RE;IL17	brown	0.02953011	-0.3652237	0.46471681	0.14557939	0.44146956	0.3486016
cg22998028	ASB16	brown	-0.1128678	-0.1280099	0.13223533	0.04904082	0.07584509	0.00740043
cg03948048	MIR107;PANK1	brown	0.46879617	0.00471117	-0.1610892	0.19601937	0.44059945	0.14845104
cg09765245	RNF149;RNF149	brown	0.16516214	-0.0462956	0.16554367	0.52179068	0.42623269	0.2973874
cg06420834	MYL9;MYL9;RP5-	brown	0.30925191	-0.3121629	0.12608658	0.35857419	0.74159747	0.42452254
cg06878361	ZNF577;ZNF577;ZNF577;Z	brown	-0.1484946	-0.5580499	0.1883087	0.17782682	0.57505267	0.16475983
cg10775230	TMIE	brown	0.26072603	-0.0860907	0.09292057	0.43213966	0.56752117	-0.0022738
cg07966572	ZNF777	brown	0.27696415	-0.0389871	0.05834899	0.22185031	0.33211207	0.37374913
cg01109337	U1;TEX14	brown	-0.1845395	-0.2337821	0.17404974	0.2014974	0.30487072	0.03889853

cg08634161	SNORD112	brown	0.22105751	0.06442988	0.11941065	0.40741978	0.335666	0.19072936
cg15674748	RP11-112L6.4;RP11-112L6.4	brown	0.18254649	-0.0636335	0.08652184	0.42256909	0.54129898	0.13674288
cg06058170	HOMER1;HOMER1;HOME	brown	-0.1929641	-0.7206044	0.00836794	0.0841926	0.72712646	0.07815407
cg19788934	C12orf43;C12orf43;C12orf43	brown	0.18917964	-0.17958	-0.0776427	0.50621006	0.56320347	0.15552447
cg03991307	MYF5	brown	-0.0160323	-0.3178366	0.03736374	0.08440962	0.41868679	0.11425621
cg11807356	NUMA1;NUMA1;NUMA1;	brown	0.3299013	-0.0899402	0.05617384	0.41773565	0.6446381	0.41054771
cg15650376	PIP5KL1;PIP5KL1;PIP5KL1	brown	0.11767536	-0.0009313	0.04313034	0.45797704	0.34919238	0.03108919
cg21931419	SYNDIG1L	brown	-0.0524877	-0.413215	0.2402237	0.18142768	0.42952356	0.21861168
cg15596666	RP11-	brown	-0.0367367	-0.197824	0.07877961	0.24564801	0.3833308	0.09321928
cg03371384	AP000439.2	brown	0.21849543	-0.2635335	0.08658546	0.45310027	0.77964851	0.20897829
cg18105767	CYP2S1;CYP2S1	brown	-0.02989	-0.355571	0.06396624	0.2846885	0.60250729	0.1607471
cg03832522	RUFY4;RUFY4;RUFY4;RU	brown	0.01723338	-0.3654865	0.0589295	0.2798915	0.59405765	-0.0010619
cg26931208	RASIP1	brown	0.12262603	-0.1404659	0.08887283	0.40559765	0.33574845	0.30173614
cg19627033	RN7SK;GSTA4;GSTA4;GS	brown	-0.0355277	-0.5853491	0.10084238	0.25031859	0.85265669	0.10378095
cg26336264	C2orf63;C2orf63;C2orf63;C2	brown	-0.0773392	-0.3232215	0.25167633	0.24536471	0.37612432	0.11457084
cg07056448	SERPINB2	brown	0.0732851	-0.4794082	0.00280151	0.36875261	0.84895929	0.21171566
cg25098500	SPAG4	brown	-0.0348799	-0.2619514	0.08953965	0.05721649	0.46241437	0.28453998
cg09555673	AP003774.6	brown	-0.2314417	0.15702419	0.26762792	-0.0315995	-0.2918208	-0.0253585
cg03920024	ADM2;MIOX;MIOX;MIOX;	brown	0.20343117	-0.2170896	0.18612936	0.25937951	0.62658208	0.18096509
cg12850707	PMEPA1;PMEPA1;PMEPA	brown	0.32060855	-0.0169842	0.19397277	0.61845712	0.53628868	0.34342852
cg26908123	MYO1C;MYO1C;MYO1C;	brown	-0.1446316	-0.3292372	0.21958373	0.25850199	0.44968842	0.12417927
cg19875973	CPT1C;CPT1C;CPT1C;CPT1	brown	0.12690052	-0.147093	0.28000167	0.2087833	0.34668714	0.27198367
cg21695020	DPEP3;DPEP3;DPEP3	brown	0.33649708	-0.018975	0.11622552	0.29045207	0.33591576	0.3822176
cg27400689	NDUFS3;PTPMT1;PTPMT1;	brown	0.30605899	0.0110728	-0.1166494	0.30588142	0.43497288	0.11527186
cg03763518	C1orf54;C1orf54;C1orf54	brown	-0.0685709	-0.6123016	-0.0239735	0.16965819	0.83160068	0.04007055
cg04364892	U6;RASA1	brown	0.08640168	-0.083071	-0.02669	0.39357168	0.4802541	-0.0026235
cg14286234	U6	brown	0.20484092	-0.1947932	-0.1044128	0.61616137	0.64017205	0.20126133
cg25828496	RN5S448	brown	0.03052374	-0.1768031	0.04482026	0.49847871	0.39276755	0.13910637
cg25317746	NOTCH4;NOTCH4	brown	0.15244519	-0.1892902	0.17667836	0.30283075	0.58455607	0.26176018
cg17713161	C17orf28;C17orf28	brown	0.23705747	-0.0451512	0.17729466	0.59787159	0.55029805	0.1410881
cg13871633	ZNF662;ZNF662;ZNF662;Z	brown	-0.0684703	-0.5421326	0.19357799	0.41565319	0.69752196	0.11631328
cg23181800	AKT3-	brown	0.36175189	0.19614256	-0.4330619	0.2225071	0.22379579	-0.0492104
cg15845821	NWD1;NWD1;NWD1	brown	-0.044151	-0.0410716	0.08119106	0.28880671	0.33031808	0.12777093
cg20350981	GIT1;GIT1;GIT1;ANKRD13	brown	-0.0017712	-0.2731696	0.14450815	0.25834904	0.47587965	0.18564474
cg21069258	TGFBR2;TGFBR2	brown	0.24204273	-0.1502832	-0.3072127	-0.2171861	0.37681083	0.03371482

cg20248866	PCYT2;PCYT2;PCYT2;PCY	brown	-0.0519795	-0.4984316	0.3164619	0.23521246	0.63811977	0.1294606
cg26466145	TPRG1-AS1;TPRG1	brown	-0.1445103	-0.611543	0.06453796	0.2120406	0.73069201	0.06110225
cg22151217	ZNF131;ZNF131;ZNF131;Z	brown	0.13362163	-0.3648338	-0.0277034	0.36100744	0.8472405	0.15573986
cg21843594	HORMAD2;CTA-	brown	-0.0331152	-0.6246755	0.05543068	0.19347282	0.71842405	0.16972216
cg04632023	RP11-8L8.2	brown	-0.0480167	-0.3282568	0.10865498	0.22709962	0.52009931	0.14619221
cg15014458	LYPD3;LYPD3;LYPD3	brown	-0.0097527	-0.3738866	0.18565079	0.24572734	0.58508705	0.19837076
cg08608126	CD59;CD59;CD59;CD59;CD	brown	0.11756119	-0.1031981	-0.0223988	0.56527508	0.47914457	0.09513944
cg04258133	TNNT3;TNNT3	brown	0.33232309	-0.1092238	0.05954998	0.38175294	0.52479999	0.30582084
cg01982627	BRD4;BRD4	brown	0.0969549	-0.3748395	0.040457	0.41289198	0.86291948	0.14647723
cg04190237	RP11-195F19.5;RP11-	brown	-0.0064021	-0.2278661	0.0722255	0.22152168	0.40528018	0.10607848
cg23713900	CLUL1;CLUL1	brown	-0.0570133	-0.1494529	-0.1365533	0.18770093	0.32586312	0.00149605
cg09916174	CALD1;CALD1;CALD1;CA	brown	0.22182312	-0.0309944	-0.2073973	0.30223223	0.42679501	0.15413818
cg06540876	ZBTB12;C2;C2;C2	brown	0.06620866	-0.1183117	-0.090663	0.31282254	0.36141959	0.00113509
cg00124684	IDH2;IDH2	brown	0.17532155	0.0674734	0.04414548	0.41655963	0.46491307	0.1442142
cg26145333	HMBS;HMBS;HMBS;HMB	brown	0.26120588	-0.1930957	0.26108301	0.582566	0.48102358	0.31130256
cg17340043	MCM10;MCM10	brown	-0.0204061	0.09448201	0.03137928	0.32461004	0.17892259	-0.012597
cg12444861	PLAGL1;PLAGL1	brown	0.09092432	-0.0791567	0.24562195	0.22586252	0.43614209	0.24977175
cg20376277	CTC-461F20.1;RP11-	brown	0.02571197	-0.3133841	0.01888846	0.1469379	0.37620322	0.10391527
cg25378281	CTA-109P11.4	brown	0.19097285	-0.1322785	0.01338324	0.5503923	0.54253971	0.23918519
cg10638734	GPRC5B;GPRC5B;GPRC5B	brown	-0.1310377	-0.4897381	0.08040373	0.20906349	0.59010306	0.02054189
cg13504931	ITSN2;ITSN2;ITSN2;ITSN2;	brown	-0.0297818	-0.5362478	-0.1425812	0.20931107	0.83200601	0.02563645
cg00128506	RP11-211C9.1;MFHAS1	brown	0.0006608	-0.3923746	0.02013612	0.21427137	0.51532753	0.11042551
cg18479961	MIR671	brown	0.18912884	-0.1465048	0.26391216	0.24179215	0.33344588	0.318306
cg04055835	PHLDA3;PHLDA3;PHLDA	brown	0.06341436	-0.2142143	0.12767248	0.38592726	0.40420337	0.08986808
cg20711299	RP4-781K5.5;RP4-	brown	0.39377868	0.04790668	-0.1261459	0.13426975	0.38289404	0.04167892
cg00650977	RP5-	brown	0.17768577	-0.3357931	-0.0164326	0.24147992	0.70297172	0.13985957
cg02299602	Y_RNA;CMBL;CMBL;CMB	brown	0.21147708	-0.0666695	-0.0190369	0.59615087	0.54239192	0.06115565
cg12315353	GABBR1;GABBR1;GABBR	brown	0.08364472	0.01852511	0.1293097	0.27734667	0.46033189	0.11268397
cg26556091	RP3-508I15.18;RP3-	brown	0.38639503	0.14404866	0.33276544	0.42850047	0.37133912	0.45625466
cg14904905	SIRPB2;SIRPB2;RP4-	brown	0.11238941	0.03142738	-0.0066965	0.45055771	0.29456298	0.20184384
cg18737650	PDZD8	brown	0.13852172	-0.4187086	0.08399894	0.3380265	0.83434188	0.27694857
cg06312848	KBTBD2;KBTBD2;KBTBD	brown	0.26559699	-0.0433989	-0.4955446	0.08960009	0.41503821	-0.0726993
cg23771366	PRSS23;PRSS23;PRSS23;P	brown	-0.1990217	-0.3742718	0.02170715	0.18380338	0.44820279	-0.0266756
cg19095000	FBXO2;FBXO2;FBXO2;FB	brown	0.16528381	0.05686102	0.49412766	0.4254621	0.37649984	0.26959224
cg14547393	RP11-712B9.2;RP11-	brown	-0.0192215	-0.2674601	0.19189093	0.20459036	0.34406491	0.10847212

cg06797665	RP11-67C2.2	brown	0.25383171	-0.1260977	0.28879046	0.71277998	0.61383691	0.24055966
cg14726652	GPRC5B;GPRC5B;GPRC5B	brown	-0.1284853	-0.3259146	0.09810085	0.18306433	0.32643698	-0.0333219
cg05350176	SNORA74	brown	0.02112208	-0.5332002	-0.0039611	0.34896085	0.94715805	0.1337859
cg04413853	C17orf64;C17orf64;C17orf64	brown	0.2246517	-0.2473807	0.08627449	0.29278788	0.60076929	0.24635728
cg08161931	PLEC;PLEC	brown	0.05779499	-0.1728936	-0.0359446	0.24973893	0.3909203	0.11146037
cg01111041	PRRT1;PPT2;PPT2;PPT2;PR	brown	-0.1281007	-0.1420335	0.08201447	0.39783538	0.32335915	0.05267956
cg02163846	TRAF2;TRAF2	brown	0.15806833	0.22266478	0.17695678	0.28222098	0.25740441	0.17549072
cg24600306	CHRNA4;RP11-	brown	-0.1415842	-0.1719084	0.28398684	0.21480046	0.31088383	0.04187042
cg23197073	HDAC4;HDAC4;HDAC4	brown	-0.0789525	-0.3641524	0.27908194	0.04060771	0.45351721	0.23355721
cg18759081	COL9A3;COL9A3;COL9A3;	brown	0.10995133	0.28886999	0.0448538	-0.0510176	-0.2714579	0.08271759
cg01475538	RRAS2;RRAS2;RRAS2;RR	brown	-0.1454994	0.20962682	0.45176129	0.17118989	-0.1654277	0.08379995
cg00013530	GNAI2	brown	0.26239901	-0.286184	0.19203726	0.48222592	0.74534071	0.34085566
cg16365756	RSPRY1;RSPRY1	brown	0.11067102	-0.4478264	-0.0146835	0.33089853	0.88340712	0.18504426
cg16808301	RP4-800J21.3;RBM38	brown	-0.3910334	-0.0430982	0.11240105	-0.028194	-0.2173893	-0.1895364
cg20603222	GPR146;GPR146;C7orf50;G	brown	0.17342397	-0.1060895	0.1566763	0.19640663	0.33022274	0.34711142
cg09489128	HSPBP1;HSPBP1	brown	0.11345084	-0.291653	0.10365522	0.31480862	0.59156274	0.15615884
cg04834097	ZNF736;ZNF736;ZNF736;Z	brown	-0.4248095	-0.0027793	0.33750999	0.05079699	-0.22516	-0.1005498
cg19536610	CS;CS;CS;CS;CS;CS;CS;CS;	brown	0.09325859	-0.4741481	0.03824152	0.40448753	0.90076292	0.15052157
cg18694169	NKAPL;ZKSCAN4	brown	-0.0546118	-0.3384253	0.06012301	0.04856924	0.46235802	-0.0263944
cg03890167	CHIT1;CHIT1;CHIT1	brown	-0.1780846	-0.3400109	0.34045	0.12328135	0.34655656	0.06622377
cg16553272	SLC44A4;SLC44A4;SLC44	brown	0.06286484	-0.2533085	0.24494457	0.42344785	0.4360545	0.28333463
cg01528492	TMEM64;TMEM64;RP11-	brown	0.13336021	-0.3535889	0.03792717	0.45389362	0.81775379	0.15884607
cg15236082	C1orf229	brown	0.04876946	-0.3948999	0.14269473	0.29180448	0.62488776	0.27744108
cg01778872	PEA15;PEA15;PEA15	brown	0.17963365	-0.1100752	0.44903272	0.33643472	0.43438262	0.4864953
cg11625897	SPICE1;SPICE1;SPICE1;SPI	brown	-0.2089319	-0.2956964	-0.0067794	0.08331482	0.27286444	-0.1016889
cg10470887	ENSA;ENSA;ENSA;ENSA;	brown	0.1603225	-0.2319334	-0.0531583	0.39130725	0.68367254	0.07324304
cg05295388	AC009404.2;AC009404.2;A	brown	0.13166738	-0.2772644	0.29495072	0.32465846	0.56292182	0.26630734
cg03149448	RP11-228B15.4;RP11-	brown	0.36985774	0.04392241	0.38752345	0.37734206	0.49268928	0.24580628
cg27525756	SEPT5;SEPT5;SEPT5;GP1B	brown	0.09234794	-0.2069381	0.18528019	0.30067787	0.48540668	0.11443187
cg08563601	BARD1;BARD1;BARD1;BA	brown	-0.1202743	-0.4235261	-0.1061154	0.17524019	0.52978417	0.095486
cg08979515	ABHD10;ABHD10;ABHD1	brown	-0.0061366	-0.2912384	0.05722045	0.19262138	0.33658196	0.27074125
cg25996835	BBS4;BBS4;BBS4;BBS4;BB	brown	-0.2800185	-0.3735646	0.13133252	-0.0066204	0.34981668	-0.0425558
cg10375954	ACOT7;ACOT7	brown	-0.1322688	0.16857265	0.21622618	0.03172282	-0.3362733	0.10578415
cg17353890	MTMR11;MTMR11;MTMR	brown	0.24050179	-0.0653597	0.03471036	0.24275711	0.32154422	0.26021633
cg26952936	ERN2;ERN2;ERN2;ERN2	brown	-0.0283085	-0.2732883	0.23650004	0.16373435	0.321014	0.23205802

cg11601000	CTA-250D10.15	brown	-0.2758491	0.0962774	0.38822185	0.1883739	-0.1989468	0.03863543
cg20059377	SH2D7	brown	-0.2289005	0.14571083	0.26688584	-0.065497	-0.2640404	-0.0558208
cg00405086	MED23;MED23;MED23;ME	brown	-0.1468919	0.27847569	0.21049063	0.07545683	-0.327246	-0.0061583
cg15047582	RP4-728D4.2;RP4-	brown	-0.0863234	-0.4166943	0.20618945	-0.0220739	0.28113144	0.1132708
cg03453179	COL9A3;COL9A3;COL9A3;	brown	0.01518997	-0.2514185	0.00449722	0.11495214	0.44772528	0.11387041
cg20783223	RP11-	brown	0.04093074	-0.3277727	-0.0976657	0.19202087	0.62417637	0.07262658
cg22365023	PKN3	brown	0.20663444	-0.3713306	0.03385916	0.34034392	0.86185416	0.25263253
cg02952078	LEMD1;LEMD1	brown	-0.2921185	0.04725745	0.39072088	0.02448872	-0.2041766	0.11310725
cg03676180	EDC4;EDC4;CTC-	brown	0.27905284	-0.0938993	0.20504601	0.55972576	0.51131971	0.43722101
cg26775678	RP11-496I9.1;RP11-	brown	0.20270782	-0.0948194	0.04297557	0.35075218	0.50405596	0.26047624
cg25998666	ARL2;ARL2;ARL2;SNX15;	brown	0.20507198	-0.0536426	-0.0810669	0.3425812	0.5246769	0.23388989
cg21483092	RGS12;RGS12;RGS12;RGS	brown	-0.26138	0.01221318	0.2049173	0.01752555	-0.2580975	0.00758187
cg26293570	FBXO24;FBXO24;FBXO24;	brown	0.32125187	0.04881977	-0.0912716	0.35242331	0.4621511	0.21196646
cg22729821	FLVCR2;FLVCR2;FLVCR2;	brown	0.00579242	-0.4595316	0.12125807	0.17097761	0.67618814	0.24266964
cg06757381	USP48;USP48;USP48;USP4	brown	-0.0680639	-0.5735499	-0.0434126	0.18032634	0.81079443	0.02364992
cg12320395	FGFR1;FGFR1;FGFR1;FGF	brown	0.17642921	-0.2545828	0.11284936	0.35607988	0.74005871	0.21379146
cg19458257	Y_RNA;SPAG1;SPAG1;SP	brown	-0.0872632	-0.1155623	-0.0381122	0.24662668	0.34542705	-0.0706588
cg05825759	MLL3;MLL3;MLL3;MLL3	brown	0.27148238	-0.0466902	0.20016487	0.72380312	0.45557425	0.32938698
cg08272268	ZNF281;ZNF281;ZNF281;Z	brown	0.16576924	-0.1741761	-0.12012	0.35623592	0.58595849	0.00447332
cg22088248	ZADH2	brown	0.01897659	-0.418691	0.1154035	0.22616087	0.58528826	0.15024436
cg08137948	LGALS8;LGALS8;LGALS8;	brown	-0.1768067	-0.3470114	0.11288215	0.11333431	0.40960224	-0.109046
cg09810593	RASIP1	brown	-0.2166543	-0.3671461	0.04534548	0.17842813	0.3959453	0.00558425
cg09895325	SRRM1;SRRM1	brown	-0.1238539	-0.3515553	0.18628014	0.19891945	0.39803079	0.09519184
cg08209691	LY6D;LY6D;LY6D;RP11-	brown	0.27077909	0.08640894	0.45343105	0.42290199	0.28914182	0.4147965
cg09575472	RP11-267A15.3	brown	0.27492848	-0.0132021	0.08700212	0.42920631	0.49176646	0.21437554
cg04837750	SLC9C2;SLC9C2;SLC9C2;R	brown	0.1056819	-0.3103885	0.14237048	0.28207388	0.53105022	0.25858099
cg04259566	LDB3;LDB3;LDB3;LDB3	brown	0.3523619	-0.1427312	0.15541177	0.7276094	0.57438827	0.37632895
cg12796135	DENND4B;DENND4B	brown	0.17171444	-0.2342841	0.02973994	0.25017094	0.64525978	0.16790036
cg09935994	ARL4C;ARL4C	brown	0.12092485	-0.3759401	0.00738734	0.25820688	0.64480488	0.30689992
cg19677893	CNPY2;CNPY2;CNPY2;CN	brown	-0.0520572	-0.0730967	0.30830931	0.21989738	0.28788868	0.14259519
cg23082424	U6	brown	0.22186507	-0.289625	0.03760758	0.51232702	0.75607483	0.2075537
cg11766101	BEAN1;BEAN1;BEAN1;CT	brown	0.30246616	-0.0158413	-0.205663	-0.0017445	0.32185535	0.05549997
cg13559553	DPEP3;DPEP3;DPEP3	brown	0.17775734	-0.0885738	-0.1008069	0.21200881	0.32992085	0.14914293
cg08199262	RGL4;GUSBP11;RGL4;RGL	brown	0.22832636	-0.3027676	0.006262	0.16121186	0.69177176	0.38311334
cg24684067	BEAN1;BEAN1;BEAN1;BE	brown	-0.1200284	-0.3804671	0.29794625	0.15257337	0.49110256	0.0894661

cg00444719	RP11-798M19.3	brown	0.42622925	0.07798369	-0.2632781	0.35416625	0.46578157	0.04697915
cg13987674	RP11-10N16.3;RP11-	brown	0.28013353	-0.0639456	-0.2207559	0.13891032	0.43092914	0.24252714
cg23987137	PDE4D;PDE4D;PDE4D;PDE	brown	0.25150378	-0.1585422	-0.0757453	0.58497439	0.53015788	0.17550652
cg06617876	LCN8;LCN8;LCN8;LCN8	brown	-0.3873814	0.17537237	0.30586441	-0.0626158	-0.3223917	-0.0823788
cg23184690	APOC1P1;APOC1P1	brown	0.27541258	0.05672416	0.30647335	0.29692661	0.35288123	0.32382238
cg00275686	NINJ2;NINJ2;NINJ2;RP11-	brown	-0.1799844	-0.3156398	0.09446075	0.16408149	0.51485653	0.02839184
cg22068467	SLC16A5;SLC16A5	brown	0.08632551	-0.2982571	0.16627356	0.2646625	0.51144761	0.15260799
cg11660018	PRSS23;PRSS23;PRSS23;P	brown	-0.1063629	-0.3282337	0.08866391	0.31105637	0.59963063	-0.0902794
cg16014085	SEPT1;SEPT1;ZNF48;ZNF4	brown	-0.0018445	-0.5127536	0.02936786	0.37667003	0.74910079	0.01865248
cg07529339	RP11-290F5.2;RP11-290F5.2	brown	0.36460739	-0.00312	0.15373657	0.61919675	0.48071794	0.43001871
cg20667514	SNORA68;RP11-463M16.4	brown	-0.1132805	0.31463799	0.25042388	0.12042406	-0.3346192	0.12707781
cg20027894	SGSM2;SGSM2	brown	0.16052667	-0.3320097	0.10431743	0.34371238	0.80532686	0.16968649
cg24777351	SLC38A11;SLC38A11;SLC3	brown	-0.1101568	-0.3100949	0.16185676	0.22268209	0.39852569	0.11534904
cg05118960	HYAL2;HYAL2;HYAL2;H	brown	0.33292678	-0.2051934	0.15589144	0.40553418	0.59208099	0.43944883
cg24468890	HDAC1;HDAC1;HDAC1;H	brown	-0.3795077	-0.0316791	0.31011009	0.08382351	-0.1957357	-0.035725
cg13228876	KLF7;KLF7;KLF7	brown	0.36286449	-0.0953348	0.22537543	0.27545559	0.40606506	0.11357479
cg04855697	AC009677.1	brown	0.14821175	-0.3315985	-0.0548653	0.23320781	0.77363147	0.08603037
cg14128973	CNN1;CNN1;CNN1	brown	-0.1393718	-0.3335588	0.05109214	0.16911083	0.51492158	0.00589041
cg08216808	ARPC2;ARPC2;ARPC2;AR	brown	-0.0261701	-0.1196237	0.31912553	0.23202214	0.35544844	0.06288323
cg20201647	ACADVL	brown	0.20201712	-0.1208076	0.14207532	0.26162309	0.41271073	0.36047031
cg03263792	TSPAN16;TSPAN16	brown	0.19658582	0.00507804	0.08239638	0.29565465	0.39961897	0.11059924
cg11097416	SKI;SKI	brown	0.34822361	0.05693364	0.24829051	0.48821734	0.42777964	0.37995166
cg07604700	FAM211A;FAM211A	brown	0.01900653	-0.3153487	0.13822129	0.2603505	0.58327471	0.06564279
cg02491794	TTC7A;TTC7A;TTC7A;TTC	brown	0.23702472	-0.2774159	0.1860887	0.26745244	0.63572393	0.51242452
cg24872431	ZDHHC7	brown	0.12538918	-0.2696963	0.12229652	0.56614141	0.48741261	0.2317583
cg10763547	GP1BA;GP1BA	brown	0.05153893	-0.3950016	0.15596658	0.34308974	0.65536093	0.25253357
cg00575846	CD63;CD63;CD63;CD63;CD	brown	0.17140498	-0.1964175	-0.0729473	0.52517627	0.60054357	0.18611716
cg11159295	PPM1M;PPM1M;PPM1M;P	brown	-0.3804979	-0.0364733	0.16740863	-0.1117224	-0.2348305	-0.1308461
cg05914150	PIK3R1;PIK3R1;PIK3R1;PI	brown	0.1730445	-0.3627445	0.00952524	0.48804702	0.8064325	0.19845035
cg24355400	USH1C;USH1C	brown	0.2657517	-0.1180595	0.10490003	0.44272708	0.70594416	0.23072199
cg19272266	DDHD2;DDHD2;PPAPDC1	brown	0.07980924	-0.1338128	0.1458705	0.24019294	0.34013071	0.27177981
cg24359706	AC034228.7;AC034228.7;A	brown	0.06410612	-0.3975021	-0.053985	0.4031322	0.81006324	0.09291142
cg18960660	RP11-136O12.2	brown	-0.0820736	-0.2420368	0.09973116	0.20796845	0.33981428	0.2266467
cg19533386	CLCN1	brown	0.40079949	0.15327711	-0.2437198	0.23108	0.32458847	0.13595784
cg13291394	CTD-	brown	0.07664772	-0.199519	0.02455242	0.39683881	0.51086697	0.11712433

cg27182761	PHLDA1;PHLDA1;PHLDA	brown	0.01803063	-0.3188693	-0.0294677	0.1637264	0.50819108	0.14615574
cg11340260	GP1BA;GP1BA	brown	0.09240675	-0.3728529	0.33002799	0.36766353	0.64138813	0.35694755
cg02203833	CDK5;CDK5;CDK5;SLC4A	brown	0.13536951	-0.3050756	0.26942081	0.31189697	0.66302552	0.14870876
cg15447535	DHX9;DHX9;DHX9	brown	-0.3980925	0.00196713	0.18006629	0.03214122	-0.1946346	-0.1513454
cg13432524	TMEM106A-AS1	brown	0.34639034	-0.0030546	0.16101105	0.42813052	0.42213168	0.40236431
cg07402711	KCNAB2;KCNAB2;KCNA	brown	0.33040282	0.01207337	0.10044797	0.19162321	0.30862115	0.27316036
cg10874468	WDR1;WDR1;WDR1;WDR1	brown	-0.2195233	-0.0608008	0.39146384	0.23880361	-0.1742872	-0.0222671
cg20129838	OR2AE1	brown	0.17381839	-0.3273615	0.11752937	0.45043866	0.73384788	0.19022974
cg16724148	AGL	brown	0.4867102	0.03054828	-0.1120684	0.2708745	0.44593975	0.1942683
cg13401703	TTC23;TTC23;TTC23;TTC23	brown	0.01177882	-0.3649927	0.03785491	0.06102641	0.44483998	0.07482015
cg11487663	IRAK3;IRAK3;IRAK3;IRA	brown	0.02901407	-0.1649616	-0.2325748	-0.0244196	0.28719893	-0.3560608
cg08039193	SLC12A4;SLC12A4;SLC12	brown	-0.3770061	0.10085863	0.25808226	-0.0756883	-0.35445	-0.0366562
cg24740850	CPEB4;CPEB4;CPEB4;CPE	brown	0.26937551	-0.0584588	0.27460674	0.23926072	0.32119161	0.488762
cg03154412	SPSB1	brown	0.1142708	-0.158327	0.13108982	0.33887706	0.45531679	0.12041981
cg05008895	LA16c-	brown	-0.3135743	-0.1802211	0.03004662	-0.0122679	-0.0569778	-0.054285
cg06094482	HPCAL4;PPIE	brown	0.16657096	-0.0234741	-0.0978859	0.30307461	0.28109212	0.12247147
cg21231302	RNF208;RNF208	brown	0.19942435	-0.2108354	0.22066422	0.3686715	0.62554667	0.17532648
cg05096467	U1;TEX14	brown	0.01156658	-0.2652062	-0.0438225	0.25025693	0.57450379	0.16442883
cg09852127	PCDHA12	brown	-0.0177101	-0.3309119	0.20326882	0.19451087	0.34986659	0.24176492
cg20087519	SYCE1;SYCE1;SYCE1;SYC	brown	-0.0707137	-0.395234	0.03339552	-0.0014264	0.37308885	0.11084351
cg16097030	PYCR1;PYCR1	brown	0.31291355	-0.254241	0.21903197	0.5920903	0.72266155	0.36901245
cg24637276	TNFRSF10B;TNFRSF10B;T	brown	0.41631898	-0.0133169	0.41176315	0.35325288	0.50050092	0.54295419
cg04009363	SFXN5;SFXN5;SFXN5;SFX	brown	-0.1251713	-0.530768	0.27066498	0.15991344	0.59725454	0.1023627
cg18063278	PPARG;PPARG;PPARG;PP	brown	0.25847037	-0.1495245	0.30279731	0.17645622	0.34000823	0.22493388
cg06526676	GYLTL1B;GYLTL1B	brown	0.17615979	-0.400476	0.08954492	0.34027787	0.81599234	0.29188297
cg18638434	ENTPD5;ENTPD5;ENTPD5;	brown	0.21459936	-0.2977665	-0.004751	0.46265936	0.68838053	0.26582671
cg05870200	SLC16A3	brown	0.22470771	-0.1628225	0.2563445	0.34681532	0.5209049	0.38620401
cg16764848	GNPNAT1;GNPNAT1;GNP	brown	0.27248612	-0.1418899	0.41625676	0.3705415	0.40677897	0.32970227
cg04749507	PPT2;PRRT1;PPT2;PPT2;PP	brown	-0.0550044	-0.2392184	0.33414258	0.2372654	0.35356891	0.16209604
cg17944560	WDR81;WDR81;WDR81;W	brown	0.27395778	-0.0495319	0.09571664	0.5353776	0.42089381	0.18128294
cg11276172	ITPRIP;ITPRIP	brown	0.25981499	-0.1782507	0.20460525	0.53122265	0.58019149	0.30503577
cg23992003	DCP1B;DCP1B;DCP1B	brown	0.31445441	-0.1164651	-0.1157839	0.68609167	0.59835394	0.21481063
cg22263040	TFR2;TFR2;TFR2;TFR2	brown	0.22961901	0.15848019	-0.0220398	0.36417841	0.25399303	0.00519356
cg10635061	FHL2;FHL2	brown	-0.0115039	0.26732297	0.36094757	0.33908862	-0.3008584	0.10193793
cg27494647	RARRES2;RARRES2;RARR	brown	-0.0320206	-0.2792079	0.16262261	0.22737176	0.43887352	0.04796004

cg12239753	SAA2;RN5S333;SAA2-	brown	0.16850781	-0.1446774	0.04746765	0.20642634	0.39328047	0.29101537
cg03164561	NMUR1	brown	0.30819694	0.22108227	0.09488701	0.56226182	0.25842457	0.30002588
cg15369784	RP1-90K10.4;PI16	brown	0.41660089	0.11283386	-0.0180821	0.19940686	0.30318283	0.08539511
cg02711271	SYNPO	brown	-0.1890442	0.1520946	0.40579129	0.03113343	-0.2687513	0.08329408
cg12433386	KCNQ1OT1	brown	0.42236238	-0.1099849	0.04007652	0.42178724	0.64694379	0.39041395
cg24570378	THAP4	brown	0.18343244	-0.2084279	0.04363853	0.51182797	0.64091424	0.14345023
cg25677116	RP11-40F6.2;RP11-40F6.2	brown	0.06023441	-0.3011542	0.07092744	0.1781812	0.54905141	0.03503387
cg01322214	C7orf31;C7orf31;C7orf31;C7	brown	0.04126923	-0.3243744	0.06614878	0.29714979	0.5928266	0.08973446
cg04557883	PXDC1;PXDC1;PXDC1	brown	-0.1298096	-0.4019972	0.14174023	0.13528557	0.3995057	0.08756753
cg26835374	AL583842.3;AL583842.3;AL	brown	0.20197476	-0.0937681	0.2079651	0.25613162	0.29110289	0.34316353
cg25028832	IL11RA;IL11RA;IL11RA;IL1	brown	0.12288583	-0.4091958	0.17782866	0.37217604	0.72062651	0.21546022
cg09452027	SLC35C1;SLC35C1;SLC35C	brown	0.24544526	-0.3243844	0.18966307	0.4097153	0.75730814	0.28454685
cg07084721	PCOLCE;PCOLCE;PCOLCE	brown	0.42268632	0.0641913	0.07259446	0.20855781	0.38032666	0.20283622
cg25597805	LINC00114;LINC00114;LIN	brown	0.27631259	0.05687685	0.08349322	0.61447586	0.47062612	0.13710202
cg09269848	MYO1C;MYO1C;MYO1C;	brown	-0.0983065	-0.3672676	0.22643667	0.23915105	0.54300628	0.04236979
cg21165029	MXRA8	brown	0.12516168	-0.2151056	0.07486306	0.190652	0.52375207	0.13163378
cg10175387	UBAC1;UBAC1	brown	0.12512437	-0.1553455	-0.0228026	0.17864254	0.41839913	0.07591726
cg06278025	INPP5J;INPP5J;INPP5J;INP	brown	0.0185542	-0.1853306	0.17932217	0.20297103	0.52073709	0.098769
cg13495118	TM4SF18;TM4SF18;TM4SF	brown	0.22987009	-0.0451507	-0.1830805	0.16669544	0.34850479	-0.0111646
cg04108240	TRA2A;TRA2A;TRA2A;TR	brown	0.50135435	0.06777406	-0.0253597	0.29603715	0.48759063	0.17138845
cg14900154	ANKFY1;ANKFY1	brown	0.1613437	-0.1111252	0.01155361	0.28153519	0.45150476	0.18799762
cg03744383	CES1;CES1;CES1;CES1;CES	brown	-0.0573933	-0.2877731	0.18639825	0.24570237	0.40514227	0.15715759
cg20525917	GATA5;GATA5;GATA5	brown	0.2332695	-0.1019527	0.32077682	0.21602141	0.27447835	0.47080727
cg02505487	NR4A1	brown	0.22114763	-0.2565972	0.17418926	0.37328179	0.67946244	0.26975659
cg25339073	RP5-	brown	0.02655666	-0.4181691	0.12734547	0.17021544	0.52498876	0.2173351
cg14164099	CTD-2536I1.1;CTD-2536I1.1	brown	0.04600134	-0.3255979	0.11656093	0.0651241	0.43579015	0.04289034
cg13953976	IL1A	brown	0.07888241	-0.2626807	0.14619996	0.24134514	0.42556273	0.19746968
cg26095391	C14orf93;C14orf93;C14orf93	brown	-0.179283	-0.277207	0.1204031	0.19929434	0.40306081	-0.0521543
cg05017199	TMEM176B;TMEM176B;T	brown	-0.1765414	-0.4532634	-0.0058149	0.23200881	0.50899423	-0.1192052
cg10136008	HNMT;HNMT;HNMT;HNM	brown	-0.0893046	-0.2540127	0.02089995	0.22348059	0.41896457	0.08918338
cg27548431	NUP155;NUP155;NUP155;	brown	-0.0694824	-0.3959814	-0.0906058	0.16270324	0.51973694	0.09178636
cg09994057	FAM107B;FAM107B;FAM1	brown	0.33196873	0.2246833	0.03824509	0.29041012	0.2837728	0.07041357
cg15752292	TIMM50;TIMM50	brown	0.19580699	-0.1507597	-0.022197	0.42368936	0.54903201	0.23639432
cg15253520	FAM82B;FAM82B;FAM82	brown	-0.0721765	-0.3783474	-0.1309748	0.09672113	0.4895427	0.14318115
cg23644960	RP11-	brown	0.08065446	-0.2045428	-0.391925	-0.0358682	0.29685326	-0.1004333

cg26189401	SCN5A;SCN5A;SCN5A	brown	-0.175299	-0.2278946	0.18047125	0.23528485	0.30336708	-0.0752567
cg26949668	SPTBN2;SPTBN2;SPTBN2	brown	0.02288496	-0.2209314	-0.0014711	0.20119906	0.47884759	0.0634772
cg21419800	CTD-2034I4.2;CTD-2034I4.1	brown	0.07867272	-0.1808368	-0.113636	0.21741224	0.37189089	0.02347255
cg12707935	GLIPR2;GLIPR2;GLIPR2;G	brown	-0.0114408	-0.2408269	-0.0844097	0.32454902	0.3630223	0.326769
cg22851880	RP4-728D4.2;RP4-	brown	0.17034303	-0.213949	0.12866865	0.11401082	0.31909319	0.30954365
cg20179697	SNX9;SNX9	brown	0.13677137	-0.0606267	0.17869807	0.36182793	0.3386246	0.27315395
cg10257302	TBPL2;TBPL2	brown	0.10208576	-0.1795089	0.14040247	0.23139311	0.29705451	0.36299165
cg09264673	ACSM3;ACSM3;ACSM1;A	brown	0.12142078	0.17900477	-0.1579449	0.48613971	0.2755963	-0.0145375
cg21183455	C22orf34;C22orf34;C22orf34	brown	0.13367002	-0.2639902	0.07366612	0.19663782	0.55158791	0.19877478
cg04982921	EID1;EID1;EID1;SHC4;SHC	brown	0.28549012	0.11226087	0.21107133	0.33974479	0.30000079	0.03763713
cg21207090	CTA-109P11.4	brown	0.28959074	-0.044617	-0.0066296	0.64873284	0.5455227	0.28275131
cg09060241	AC012074.2	brown	0.25490652	-0.233789	0.11389001	0.32286635	0.52358558	0.27797268
cg20854857	TAGLN;TAGLN;TAGLN;T	brown	0.17229702	-0.0916964	0.35096814	0.30304053	0.35100368	0.37987701
cg20091636	RP11-163N6.2;RP11-	brown	0.07911697	-0.1077002	-0.07943	0.25255299	0.39288768	0.18727444
cg09508281	ENAM;ENAM	brown	0.02899927	-0.3572731	0.1601549	0.20920711	0.53629067	0.09105312
cg02179849	KANSL1	brown	0.26205758	-0.0850015	0.13595034	0.66653497	0.47029674	0.20587001
cg17662872	WFS1;WFS1;WFS1	brown	0.31949937	0.05190445	0.07952826	0.41128106	0.32183517	0.23869308
cg22802461	KRTDAP;KRTDAP;KRTDA	brown	0.27029236	0.11718871	0.09900801	0.3793878	0.45005687	0.21736593
cg10236417	RPL34;RPL34;RPL34;RPL34	brown	-0.3492412	-0.0467072	-0.014591	0.04116488	-0.1128598	-0.2328376
cg02892925	RP11-25K19.1;TOX;RP11-	brown	0.20006536	-0.199533	0.11410588	0.12884862	0.51547383	0.10324042
cg10599571	MRPS6;MRPS6;MRPS6;SL	brown	-0.1594149	-0.2875082	0.0898062	0.09400484	0.34754286	-0.010654
cg20116128	GPSM3;GPSM3;GPSM3;GP	brown	0.04248685	-0.3067515	0.14016577	0.20721733	0.50547733	0.18092731
cg06492412	SRXN1;SRXN1	brown	0.42859732	0.05268626	-0.2469578	0.22181671	0.4392926	0.09856133
cg20999247	MCM2;MCM2;MCM2;MC	brown	0.31390622	0.15919896	0.25779289	0.36571537	0.28473544	0.2384926
cg17160439	NAALADL1;NAALADL1;N	brown	0.17642238	-0.2877156	-0.0189504	0.36716881	0.69124237	0.1705425
cg17434943	SEC31A;SEC31A;SEC31A;S	brown	0.01485737	-0.3362945	-0.1120198	0.2693782	0.58875364	0.16225479
cg09701102	C11orf72;C11orf72;NDUFV	brown	0.25366189	-0.1279083	-0.003165	0.54119055	0.58328459	0.2020128
cg19669931	FAM166B;FAM166B;FAM1	brown	0.25531804	-0.0106386	0.31307944	0.38507433	0.41574022	0.32435101
cg11154555	Y_RNA;CDHR2;CDHR2;C	brown	0.32571301	0.07718379	-0.091667	-0.0260267	0.22827729	0.05753492
cg16880215	DPEP3;DPEP3;DPEP3	brown	0.27833716	-0.0235312	-0.120626	0.18218499	0.34198263	0.20294702
cg24899750	RP4-	brown	0.1470188	0.00197463	-0.0865673	0.32064667	0.278341	0.24679726
cg02102684	VASN;CORO7;CORO7;CO	brown	0.26083916	-0.3084488	0.17688232	0.26704007	0.66825142	0.42500921
cg18466965	FBXO21;FBXO21;FBXO21;	brown	0.13104882	-0.1646992	0.07637747	0.20100805	0.35671442	0.36257308
cg06021832	CITED4;RP5-1066H13.4	brown	0.30180496	0.10706238	0.07331627	0.41562041	0.24899779	0.2992818
cg26377721	RP4-684O24.5	brown	-0.2440857	-0.4103436	0.04590364	0.0128518	0.43980659	-0.22167

cg26647808	GCFC1;GCFC1;C21orf49;C2	brown	0.06146497	-0.3082401	0.10047152	0.38551613	0.57216493	0.10690913
cg19172222	TBX21	brown	-0.2743892	0.07712062	0.26287993	-0.0105872	-0.2147873	-0.0385945
cg10282491	CLDN9;CLDN6	brown	0.11904265	0.16203354	0.17159413	0.07506369	-0.1361198	0.12501949
cg22034203	LAPTM5;LAPTM5;LAPTM	brown	0.18399946	-0.1606886	0.2423008	0.26321213	0.40972209	0.33232965
cg17548261	RP11-	brown	0.26987726	-0.2271845	0.11023546	0.5796608	0.70925468	0.25473881
cg24620905	MTMR11;MTMR11;MTMR	brown	0.22212451	-0.1163919	0.13237021	0.2032267	0.3847731	0.29786409
cg27600082	CTD-2089N3.2;CTD-	brown	-0.0297266	-0.4828287	-0.0518315	0.03897247	0.49767807	0.10047851
cg03400060	BHMT2;BHMT2;BHMT2;D	brown	-0.0075352	-0.1782414	0.06813454	0.11671476	0.32724653	0.07455105
cg14458991	FDFT1;FDFT1;FDFT1;FDF	brown	0.04730572	-0.1161973	0.2081316	0.14552821	0.29680467	0.16802896
cg02255004	RP11-610O8.1;RP11-	brown	0.20368189	-0.1740381	0.1532521	0.52913632	0.60858934	0.158081
cg00255660	RP11-461A8.1	brown	0.12116055	-0.4524788	0.07118198	0.28701309	0.78995554	0.186802
cg11697038	AHDC1;AHDC1;AHDC1;A	brown	0.1397956	-0.319139	0.06453095	0.27311231	0.67319893	0.27655216
cg22881200	U6;XXYLT1	brown	0.30088115	0.07823928	-0.2325358	0.42201443	0.40956216	0.1543906
cg03415883	SEC31B;SEC31B;SEC31B;S	brown	0.22767033	-0.1986991	0.12563541	0.3598365	0.56554954	0.25296391
cg07061500	C6orf99;C6orf99	brown	-0.2212544	0.06191401	0.06447154	-0.1348598	-0.3173242	-0.0458564
cg03203040	MCM5;MCM5;MCM5;MC	brown	-0.3666016	-0.0169258	0.2500984	-0.1344047	-0.2085268	-0.0175661
cg16713264	WDR77;RP11-	brown	-0.3571275	-0.0504618	0.11651034	0.02657283	-0.3156903	-0.0588122
cg13406746	IRF7;IRF7;IRF7;IRF7;IRF7;I	brown	-0.3842737	0.06083018	-0.0073858	-0.07805	-0.3381926	-0.2713797
cg24956866	CALD1;CALD1;CALD1;CA	brown	0.09585349	-0.1689211	0.27142251	0.10637245	0.34627528	0.18317344
cg10517650	SPICE1;SPICE1;SPICE1;SPI	brown	-0.2171194	-0.3135382	-0.0715235	0.08439226	0.26642811	-0.0289956
cg05261851	SLC12A4;SLC12A4;SLC12	brown	-0.1773818	0.18313932	0.37328623	0.22699098	-0.2055939	0.01800782
cg25655096	LPAR5;LPAR5;LPAR5;LPA	brown	-0.0551638	-0.2857893	0.22258839	0.27910071	0.36377835	0.1581435
cg17330251	PON1;PON1;PON1	brown	-0.1640659	-0.0281723	0.14095969	-0.0496523	-0.1574789	-0.0218306
cg20648149	SYNE2;SYNE2;SYNE2;SY	brown	0.20485698	-0.1561389	-0.227119	0.22427656	0.48290835	0.11097353
cg02947253	ADORA3;ADORA3;ADOR	brown	0.17728921	-0.2642662	0.04024107	0.45024337	0.5696747	0.26663712
cg18919478	CTHRC1	brown	0.34345552	0.14977133	0.03929523	0.40385902	0.26530314	0.28627271
cg25136988	LY6G5C;LY6G5C;LY6G5C;	brown	0.18773018	-0.2546786	-0.0961378	0.36700526	0.66489715	0.15871341
cg09084679	PLXDC1;PLXDC1;PLXDC1	brown	-0.0032282	-0.1431661	0.27549195	0.17212273	0.37360306	0.09156042
cg15282632	CACNB3;CACNB3;CACNB	brown	0.17036634	-0.0224927	0.16482941	0.36276493	0.38392446	0.23579901
cg00047873	TMEM201;TMEM201	brown	0.23653073	0.02711866	0.28658711	0.43880327	0.30724873	0.53016282
cg06558640	LGALS8;LGALS8;LGALS8;	brown	0.04409534	-0.283401	0.25354253	0.18828832	0.44053227	0.07870653
cg16116203	PTMS;PTMS;PTMS;MLF2	brown	0.10516704	0.25205408	0.42317625	0.03939789	-0.222886	0.25105252
cg17794604	C11orf52;HSPB2-	brown	0.28211948	-0.0527164	0.00684849	0.19268259	0.33094322	0.23544914
cg12607838	PNPLA1;PNPLA1	brown	0.23832458	-0.2245575	-0.0666437	0.56940366	0.72501802	0.30870436
cg07859880	ECH1	brown	0.02654858	-0.2316765	0.01611184	0.3315277	0.4592558	0.13216069

cg21064315	SZT2;SZT2;HYI;HYI;HYI	brown	0.30451689	0.10163245	0.02608405	0.34586356	0.26855991	0.42042398
cg14390322	EGLN3;EGLN3;EGLN3;EGL	brown	0.3451374	-0.1067336	0.07755418	0.50441549	0.50833261	0.21601746
cg21935511	PRCD;CYGB	brown	-0.3932753	-0.0221928	0.31572509	-0.1036928	-0.2359244	-0.0391267
cg01844045	TBC1D1;TBC1D1;TBC1D1	brown	0.18920274	-0.2612675	-0.0206849	0.37437789	0.61547388	0.05570585
cg00499707	EPB41L1;EPB41L1;EPB41L	brown	0.33987374	-0.1390443	-0.0366993	0.46627829	0.64737966	0.22735719
cg14216694	TM4SF5;TM4SF5	brown	-0.3459938	0.18928785	0.3141127	0.02816065	-0.417602	-0.0988545
cg17833742	ARMC5;RP11-452L6.5	brown	0.08142211	-0.3223428	0.05005229	0.28372125	0.66397274	0.16374569
cg13451048	ERI3;ERI3;ERI3	brown	0.32707818	-0.230114	0.15960697	0.37579433	0.75660675	0.30511523
cg14884379	SERPINB2	brown	0.20646099	-0.1779324	0.02274497	0.64869748	0.61608676	0.18730459
cg06965798	EMILIN2	brown	0.11609822	-0.0765234	-0.2158414	0.55300988	0.41413115	0.1052289
cg24707219	SLC44A4;SLC44A4;SLC44	brown	0.174249	-0.2052745	0.30469244	0.45085138	0.46184724	0.34210017
cg24315542	DYDC1;DYDC1;DYDC1;D	brown	-0.4235996	0.00773753	0.25812002	-0.0454778	-0.3568668	-0.0746338
cg19669684	NOTCH2NL;NBPF10;NBPF	brown	0.16147103	-0.2359044	-0.1894602	0.37739875	0.68123585	0.19722961
cg25660691	RP11-40F6.2;RP11-40F6.2	brown	-0.0193968	-0.3223474	0.01360384	0.06307056	0.44917785	-0.0428044
cg15879720	NCS1	brown	0.23593909	-0.0227023	0.00758919	0.47945721	0.51536225	0.28401167
cg06875191	MECR;MECR;MECR;MECR	brown	0.38598408	0.15717079	-0.0582068	0.28876623	0.25617429	0.0255728
cg24735235	RP11-169K16.9;RP11-	brown	0.05225765	-0.1946588	-0.0342429	0.20947841	0.4944928	0.02103667
cg20785560	GIPC2;GIPC2	brown	0.00226205	-0.2779904	0.03251991	0.21436251	0.5384066	0.05563933
cg18380260	AASDHPPT;AASDHPPT;K	brown	0.03884252	-0.2037874	-0.1708014	0.31078841	0.39675067	-0.052005
cg21412128	GPR182	brown	0.18015439	-0.4043041	0.0228295	0.29903969	0.84913792	0.22079654
cg02799635	RP11-31F19.1;RP11-	brown	0.03827767	-0.1328294	-0.0286962	0.10390905	0.33704481	0.01925577
cg14941025	NFE2L3	brown	0.26246732	0.18629628	0.00885635	0.43019582	0.25831422	0.22947188
cg06246967	RP11-275I4.2;RP11-275I4.2	brown	0.17456871	0.66244234	-0.0143599	-0.1412723	-0.9107726	0.01824995
cg08718886	RABGGTA;RABGGTA;RA	brown	-0.4924821	-0.0620902	0.19037739	-0.0940188	-0.3063605	-0.1607481
cg13655803	WFIKKN2;WFIKKN2	brown	0.27931243	0.032033	0.16775467	0.39641545	0.37658357	0.30004279
cg15737090	EML2	brown	0.17850427	-0.2355128	-0.1007109	0.38349816	0.46018107	0.22645603
cg11052143	ALS2CR11;ALS2CR11;ALS	brown	0.15221201	-0.3589573	0.14982555	0.34916544	0.69640129	0.27447095
cg15710638	TBC1D16	brown	0.39864528	-0.1693438	0.05596065	0.47015627	0.63023331	0.60502766
cg08975915	ATP2B1;ATP2B1;ATP2B1;	brown	0.31791222	-0.1478624	-0.0463791	0.2338823	0.42781594	0.28052632
cg11865119	MEST;MEST;MEST;MEST;	brown	-0.0739496	-0.265174	0.02637647	0.11480046	0.41007099	0.06036601
cg01030476	LGALS8;LGALS8;LGALS8;	brown	0.14011188	-0.1438643	0.17304752	0.3222268	0.42701246	0.15093825
cg13169967	NXF1;NXF1;NXF1;NXF1;N	brown	-0.1642235	0.21701179	0.22922196	0.04887755	-0.3334396	-0.017117
cg22568153	NPHP1;NPHP1;NPHP1;NP	brown	0.42728596	-0.0008665	0.18635267	0.42800274	0.46631608	0.25805058
cg24088408	E2F1	brown	0.02834975	-0.0986453	-0.0408228	0.2956571	0.36680309	0.08243587
cg24025566	AVP	brown	0.2472589	-0.0100047	-0.0620156	0.36869	0.43337794	0.17057132

cg18246518	CKM	brown	0.29719079	-0.0888263	0.33767876	0.3245384	0.42669252	0.33076632
cg06783429	LRRC4;LRRC4;SND1;SND1	brown	0.31124457	-0.1504972	0.17489109	0.26654089	0.42601511	0.53232249
cg03096957	RP11-535C7.1	brown	0.46319752	0.0246611	-0.0473875	0.19849805	0.52060497	0.1414731
cg26223272	4-Sep	brown	-0.0316098	-0.2891152	-0.1266237	0.01054647	0.36233819	0.11982816
cg25135716	GLYCTK;GLYCTK;GLYCT	brown	0.29632059	0.0111825	0.27211192	0.45124493	0.43811899	0.25179942
cg03793277	APOC1	brown	0.13711272	-0.2637463	0.00499146	0.28372914	0.56984249	0.22458811
cg00433892	PBX4	brown	0.36570173	0.12611291	0.20583388	0.50465901	0.44291064	0.44672202
cg04520793	ASB16	brown	-0.438995	-0.2227058	0.01493675	-0.1755378	-0.1242853	-0.2737151
cg26392102	HLA-C;HLA-C;HLA-C;HLA-	brown	0.01666789	0.28360866	0.43805676	0.1253355	-0.2736491	0.21151637
cg17467243	RBPMS;RBPMS;RBPMS	brown	0.2943589	-0.0744299	0.19755859	0.23534595	0.34520682	0.47946546
cg24508633	ABHD2;ABHD2;ABHD2;A	brown	0.25083998	-0.122914	-0.0499573	0.57038504	0.54095541	0.13885955
cg23629150	TOP1MT;TOP1MT;TOP1M	brown	0.16686414	-0.222384	0.12193282	0.29319121	0.48102542	0.17646284
cg12131208	C17orf64;C17orf64;C17orf64	brown	0.35730716	-0.0827691	0.07822674	0.27154378	0.48863699	0.26857305
cg01964004	RN5S97;DGUOK-	brown	0.09209652	-0.2959881	-0.2083765	0.30826146	0.3161621	0.24104043
cg27288091	COX4I1;COX4I1;COX4I1;C	brown	0.35138222	-0.2189941	0.10509751	0.4959788	0.75398849	0.42353486
cg26019173	U6;STX5;STX5	brown	-0.0594199	-0.6054884	0.0637326	0.24339126	0.9638181	0.08214249
cg25975995	PET112;PET112;PET112;PE	brown	-0.0238499	0.40200297	0.32108686	0.41141238	-0.4846878	0.21620706
cg18979916	ZKSCAN4;NKAPL	brown	-0.0498176	-0.1916168	0.10517472	0.08059743	0.279156	0.03180906
cg20707409	CUZD1;CUZD1;CUZD1	brown	0.20695528	0.05079659	0.09149019	0.07270656	0.25824297	0.1961424
cg18062490	CCDC135;RP11-405F3.4	brown	0.26152823	0.42009964	0.14511563	0.36869618	-0.3034885	0.2435778
cg11644596	LRRC3-AS1;LRRC3;LRRC3	brown	0.21504996	-0.2189219	0.17768455	0.27256692	0.43422284	0.32300196
cg25167618	SLC12A8;SLC12A8;SLC12	brown	0.26562164	0.09937094	0.0979747	0.32889972	0.33922157	0.16386885
cg20799095	RP11-145A3.2;RP11-	brown	0.19106193	-0.2083238	0.32032543	0.28300022	0.51387691	0.13620441
cg09707395	ARHGEF15;ARHGEF15;AR	brown	0.03931565	-0.2315992	0.0401418	0.32795306	0.62983304	0.05686676
cg15222419	ITGA5;ITGA5;ITGA5;RP11-	brown	0.27820788	-0.2043612	0.18871463	0.47534359	0.74958873	0.25074635
cg27333706	MIR205HG;MIR205HG;MI	brown	-0.1952099	0.1067177	0.16439853	0.07982952	-0.2715786	-0.0890951
cg10172584	YJEFN3;YJEFN3;YJEFN3;	brown	0.377142	-0.0762385	-0.0037651	0.30282926	0.41569221	0.55862376
cg10852211	DCUN1D2;DCUN1D2;DCU	brown	0.51386154	0.07558426	-0.0242701	0.26121353	0.42095883	0.25990161
cg07953152	EMID1;EMID1;EMID1;EMI	brown	0.1859025	-0.2718732	0.07434852	0.46486194	0.74493346	0.26763226
cg04310331	MRPL23-	brown	-0.2960444	0.11416388	0.2742094	-0.1194973	-0.3326234	0.04199807
cg00787282	PLB1	brown	-0.0869565	0.1809286	0.22007952	0.25282622	-0.2288368	0.06244726
cg11084334	LHFPL4;LHFPL4	brown	0.08463442	-0.5006742	0.1224863	0.14591773	0.65073084	0.25691692
cg20758387	WDR85;WDR85;WDR85;W	brown	0.40797478	0.1016225	-0.0844138	0.59107475	0.41666665	0.31348633
cg15431042	RP11-67C2.2	brown	-0.0208861	-0.5614019	0.00590538	0.16319766	0.92773442	0.09656972
cg08880670	RP11-40F6.2;RP11-40F6.2	brown	0.0786756	-0.2060433	-0.0093207	0.11341526	0.43624339	-0.0372877

cg08024432	PIK3C2B;RP11-739N20.2	brown	0.14364989	-0.2016855	0.10862998	0.3506937	0.414151	0.20081557
cg07757887	CTHRC1;CTHRC1	brown	0.25492039	-0.0091214	-0.0649959	0.25017872	0.45487311	0.15875984
cg17169196	LTA;LTA;LTA;LTA;LTA;LT	brown	0.17849088	0.67811088	0.01585573	-0.1529899	-0.9160191	-0.0176631
cg00392001	UBR4	brown	0.33524665	-0.1960245	0.03462775	0.59645782	0.71562824	0.26403866
cg04249817	C10orf125;C10orf125;C10orf	brown	0.23751936	0.13355905	0.11546157	0.36422113	0.33823078	0.07231877
cg17220161	CHIT1;CHIT1;CHIT1	brown	-0.0982428	-0.3466134	0.32513267	0.12373218	0.37425966	0.0742829
cg14006239	AIF1L;AIF1L;AIF1L;AIF1L	brown	0.26353653	-0.0419384	-0.0631567	0.37218643	0.39139216	0.18454632
cg15361450	FADS3;FADS3;FADS3;FA	brown	-0.3704284	-0.1083882	0.14065254	-0.0703871	-0.191402	-0.0574006
cg10098541	PDIA5;PDIA5;PDIA5	brown	0.21374425	-0.1562276	0.33220264	0.31429066	0.53813235	0.262656
cg24408488	C20orf112;C20orf112;C20orf	brown	-0.3872741	-0.0460165	0.21577578	-0.1803605	-0.3511632	-0.0671728
cg08190844	RP11-	brown	0.24419165	-0.1828187	-0.0248227	0.40002962	0.62629186	0.21517531
cg15379294	CLCN2;POLR2H;POLR2H;P	brown	0.42064627	0.05197594	-0.0687615	0.31550337	0.47593419	0.13424542
cg05324191	Y_RNA;RP4-655J12.4;RP4-	brown	0.0855956	-0.2597649	-0.0239708	0.27691559	0.50593455	0.06322272
cg26392354	PSMF1;PSMF1;PSMF1;PS	brown	-0.1053852	-0.6322296	0.02821261	0.10933191	0.9412222	0.06914165
cg10571161	ABCA17P;ABCA3;ABCA17	brown	-0.0229842	-0.3500048	0.29603073	-0.0164646	0.33668592	0.12532746
cg26053291	ANKRD36BP2;ANKRD36B	brown	0.14425445	-0.3261901	0.1651744	0.1187685	0.37261285	0.25003973
cg20024687	TOP1MT;TOP1MT;TOP1M	brown	0.28471454	-0.1785341	0.09806286	0.51326428	0.50017164	0.25523663
cg07909384	MIR181B2;MIR181A2HG	brown	0.03659426	-0.5593942	0.03555315	0.20600691	0.92332951	0.12769601
cg03977449	AC104699.1;DTNB;DTNB;D	brown	0.02071949	-0.4295965	0.03655455	0.07630372	0.69559612	0.13076326
cg16634101	SASH1;RP11-631F7.1	brown	-0.2568464	0.12588222	0.08748888	0.04595868	-0.2629862	-0.1036524
cg17583667	RP4-728D4.2;RP4-728D4.2	brown	0.19913143	-0.1715271	0.21809182	0.19265318	0.37977459	0.16059167
cg20316427	RP11-	brown	-0.1453905	0.07888196	0.19992276	0.3644425	-0.2024581	0.05074427
cg18518516	NUP160;NUP160;RN5S340;	brown	0.33401238	-0.1710398	0.32587569	0.29699007	0.55843393	0.34730179
cg16617101	AC008063.2;DPP4;DPP4;DP	brown	-0.2881947	0.0943805	0.04026098	-0.0270584	-0.3253024	-0.209399
cg16988970	ASB16	brown	-0.168707	0.10466837	0.21653037	-0.086099	-0.3629733	0.00375844
cg01189606	PLXNB1;PLXNB1;PLXNB1	brown	0.12508511	-0.309595	-0.0333073	0.25055	0.65686004	0.21449515
cg20194364	HSP90AB1;HSP90AB1	brown	0.24491286	0.15998883	0.0287821	0.18351388	0.30353205	0.05948591
cg20279977	RP11-553L6.2;RP11-553L6.2	brown	-0.2895302	0.0449109	0.17845155	-0.1180059	-0.220386	-0.1306752
cg10379476	TOLLIP;TOLLIP;TOLLIP;T	brown	0.20078206	-0.3150818	-0.1016412	0.39068344	0.70844676	0.32068638
cg17064521	RP11-519G16.3;RP11-	brown	-0.1670021	-0.5908219	0.07385418	0.19850299	0.91460045	0.03518022
cg18928066	BTBD19;BTBD19;BTBD19;	brown	0.16544133	-0.1905392	0.22164014	0.22457236	0.49364207	0.36359715
cg19641644	CAMK2B;CAMK2B;CAMK	brown	-0.1384953	-0.3622202	-0.2634296	-0.085434	0.3279589	-0.4190055
cg08217285	GIT1;GIT1;GIT1;ANKRD13	brown	-0.0260481	-0.2683676	0.07279924	0.18144346	0.41690637	0.10875769
cg20116907	TPRG1;TPRG1-AS1	brown	0.0840846	-0.2270433	-0.0198374	0.34239615	0.43503079	0.2389708
cg24577159	RP11-	brown	0.27274408	-0.2904596	0.21946684	0.32070011	0.69751576	0.25530004

cg17942763	SKI	brown	-0.2117879	0.13504612	0.2802549	-0.0191216	-0.3360248	-0.0758817
cg15370819	AC008440.5;MYADM;MYA	brown	0.0347278	-0.4686841	0.14223192	0.25255293	0.63796205	0.11129737
cg05970811	SRGAP1;SRGAP1;SRGAP1	brown	0.16642917	-0.2927193	0.02045471	0.28968629	0.52992987	0.20935471
cg27263492	CIRBP;CIRBP;CIRBP	brown	0.05944868	-0.1496304	0.00035002	0.26591754	0.35747981	0.21001236
cg15932663	SLC9A9;SLC9A9	brown	0.39446498	-0.0374401	0.08016428	0.34284424	0.48312087	0.09063689
cg27016454	HPSE;HPSE;HPSE;HPSE	brown	-0.1029045	-0.6556982	0.02651841	0.13986789	0.95711986	0.0479212
cg26628031	snoU13;C8orf38;C8orf38;C8	brown	0.39727293	-0.0663554	0.15364728	0.5332444	0.60286128	0.46904891
cg00826536	Y_RNA	brown	0.28772707	-0.045282	0.08314344	0.44378973	0.48517496	0.32738175
cg10076265	FCGRT;FCGRT	brown	-0.2387872	-0.6645855	0.07051976	0.10407436	0.87847204	-0.0239518
cg24613798	snoU13	brown	0.2854885	-0.0156246	0.39805617	0.35695644	0.28334643	0.50238613
cg04981010	SLCO4A1;SLCO4A1;SLCO	brown	0.29865336	-0.0849073	0.30060663	0.34617468	0.36393797	0.31549935
cg00165394	RP11-	brown	-0.1330532	0.15196543	0.45868065	0.03507652	-0.1718584	0.23321014
cg03140521	GNG12;GNG12;RP11-	brown	-0.0462955	-0.4185444	0.25739087	0.14783836	0.45576799	0.32587235
cg26580609	U6;NT5C1B;NT5C1B;NT5C	brown	0.15602698	-0.1268907	-0.0455307	0.08260761	0.29621909	0.24703784
cg20770960	PODNL1;PODNL1;PODNL	brown	-0.1243802	-0.5558729	0.03014449	0.18798544	0.92708507	-0.003643
cg18719665	TTC39C	brown	0.16134558	0.68961256	-0.0804097	-0.1376489	-0.9015889	-0.0300225
cg01626496	HSD17B11;HSD17B11;HSD	brown	0.04782794	-0.2417705	0.05656193	0.33519474	0.50284206	0.02036295
cg18354633	AL109947.1	brown	-0.094674	-0.5924482	0.04141934	0.17717032	0.95420567	0.07820224
cg14899653	TOM1L2	brown	-0.0320852	-0.5730238	0.02510543	0.21584816	0.93464679	0.09147419
cg21539594	TIE1;TIE1;TIE1	brown	0.29068484	-0.0965789	0.0691251	0.33386993	0.55800748	0.12044998
cg06259584	PCNA;SNORA26	brown	0.38304798	-0.1022372	0.25625279	0.70984201	0.52609523	0.26483843
cg11909142	SEC23B;SEC23B	brown	-0.0341369	-0.6066717	0.05042282	0.24087914	0.96276447	0.07807226
cg05303280	GLDN;GLDN;GLDN	brown	0.20243301	-0.0161392	-0.1005436	0.19284406	0.40478722	0.10604282
cg23141183	SLC16A3	brown	0.1166557	-0.2844507	0.15120388	0.25844546	0.6351165	0.23403274
cg26238820	TMEM100;TMEM100;TME	brown	-0.0066854	-0.2408757	0.10793946	0.1345194	0.36561186	0.05931736
cg24814257	CEACAM5;CEACAM5;CEA	brown	-0.1881346	0.22040296	0.16752933	0.1385231	-0.3154902	-0.0303648
cg23432896	U6	brown	-0.4372375	0.00893645	0.19947032	-0.0847374	-0.3937783	-0.2123977
cg14522803	PNPLA2	brown	-0.0057053	-0.5688656	0.08152582	0.23145846	0.95015558	0.1469745
cg03363031	C9orf171;C9orf171;C9orf171	brown	0.04151656	-0.377605	0.02190202	0.27224323	0.6602036	0.00388625
cg10775231	RP11-40F6.2;RP11-	brown	0.0261091	-0.2621588	0.02145886	0.17041139	0.47410992	0.03126093
cg10710218	ACOT11;ACOT11;ACOT11	brown	-0.0924822	-0.6256285	0.02273371	0.22484191	0.94345078	0.05744308
cg25195673	GFPT1;GFPT1	brown	0.24713407	-0.1215473	-0.1212146	0.47111478	0.47713342	0.23658981
cg23545734	ECE1;RP5-1071N3.1;ECE1	brown	0.08898213	0.64666115	-0.0557641	-0.210939	-0.94427	-0.0623818
cg06000353	VNN1	brown	0.01753215	-0.2544208	-0.3613271	-0.086218	0.34406	-0.1055664
cg02867250	PSORS1C2;PSORS1C1	brown	0.58156554	0.10399957	0.07537987	0.27253516	0.45164955	0.20352796

cg19962349	PAIP1;PAIP1	brown	0.19849819	0.67488727	-0.0380558	-0.0569716	-0.8898861	-0.0075902
cg14409041	RP11-	brown	-0.0742197	-0.2741522	0.10103398	0.21408679	0.38773228	0.1322254
cg26776551	INTS6;INTS6;INTS6;INTS6	brown	-0.0931642	-0.6318789	0.02578386	0.13780134	0.94935977	0.05029545
cg14097631	RP11-	brown	0.02443803	-0.531663	0.05965259	0.24492869	0.94411428	0.1415898
cg22777668	LAG3;LAG3;LAG3	brown	-0.1721133	-0.5916993	0.05923841	0.14965143	0.90624218	0.03185654
cg15130132	ECE1;RP5-1071N3.1;ECE1	brown	0.12328278	0.63584221	-0.1013509	-0.2113656	-0.9404054	0.00105107
cg21890667	HORMAD2;HORMAD2;HO	brown	0.14954368	-0.4141548	0.01917663	0.31803264	0.68002814	0.10662494
cg00632587	MIR31HG	brown	0.02578567	-0.1994871	0.12424725	0.16758825	0.33679725	0.09919508
cg20968071	KANSL3;KANSL3;KANSL	brown	0.15426955	-0.1682771	0.254568	0.3707841	0.53684499	0.33192331
cg01884767	RP11-533E19.2	brown	0.52328172	0.07511829	-0.1641593	0.30004741	0.49775693	0.11287493
cg18215677	RP11-401P9.6	brown	0.10697947	-0.3296877	0.01849731	0.1850383	0.63201072	0.09338496
cg02364450	MDC1;MDC1;MDC1-AS1	brown	-0.0322109	-0.6048405	0.06762431	0.16193666	0.95181019	0.14267511
cg04084348	PLAU;PLAU;PLAU;C10orf5	brown	-0.1323787	-0.5255285	0.0546244	0.20798186	0.90460559	0.02487096
cg07101082	NCOA2	brown	0.49405546	0.03358572	0.02902352	0.24277718	0.54357926	0.14257931
cg09950128	TMEM219;TMEM219;TME	brown	0.26810856	-0.1158778	0.17170317	0.40316199	0.48190626	0.41123315
cg25189241	GORASP2;GORASP2;GOR	brown	0.32793165	-0.0182548	-0.2464583	0.0024996	0.33599063	0.02969073
cg26657550	DCLK2;DCLK2;DCLK2	brown	0.50193681	0.05696621	0.1123784	0.43152836	0.48704759	0.22649293
cg25986804	NCOA2;NCOA2;NCOA2;N	brown	-0.2767089	0.08621489	0.15882146	0.11172638	-0.3077894	-0.0624241
cg21277889	MIR550A3;AC007255.7;AC	brown	-0.0653527	-0.5781865	0.0342573	0.21795848	0.9316503	0.1127261
cg09264084	MAZ;MAZ;AC009133.14;A	brown	-0.0756034	0.22510419	0.36420405	0.1756112	-0.2232349	0.16103814
cg05336510	C9orf103;C9orf103;C9orf103	brown	0.37076914	-0.1230287	-0.1395787	0.43501866	0.6477041	0.23386922
cg15419710	SCNN1D;SCNN1D;SCNN1	brown	0.3176639	0.04825732	0.35762818	0.28599721	0.30265262	0.41962005
cg09593400	KLC2;KLC2;KLC2;KLC2;K	brown	-0.4028857	-0.0161373	0.2440634	-0.0207882	-0.2449567	-0.0931589
cg15735984	TBX2;TBX2;TBX2	brown	-0.1407641	-0.223164	-0.2656743	-0.1118836	0.25548721	-0.4330107
cg21036162	LY6G5C;LY6G5C;LY6G5C;	brown	0.30352708	-0.0602343	0.12596922	0.43659013	0.45519705	0.25920822
cg06366675	PRDM5;PRDM5;PRDM5;P	brown	0.30132457	0.01388791	0.42596791	0.34227632	0.31239092	0.3016901
cg08751913	RNF144B;RNF144B	brown	0.23076369	-0.0421286	-0.0751776	0.17522883	0.33463594	0.1164692
cg08569425	GRAMD3;GRAMD3;GRA	brown	0.4305584	0.09022158	-0.1029545	0.23753994	0.4345869	0.12514667
cg16982741	ECH1	brown	0.08664605	-0.0385217	0.02044188	0.29152747	0.31019597	0.01110907
cg18048620	AMHR2;AMHR2;AMHR2;	brown	0.09800568	-0.2286219	0.20598929	0.3084187	0.50037186	0.13712723
cg16588864	AKR1B15	brown	0.09439177	-0.0670804	-0.1712023	0.12096569	0.32141111	-0.1591403
cg06097821	U6;NVL;NVL	brown	0.45943778	0.0412278	-0.3274833	0.24263389	0.46790219	0.01119186
cg02454657	CCDC162P;CCDC162P	brown	0.21277951	-0.1002734	0.07946187	0.30904876	0.50170301	0.13521148
cg15573884	snoU13;LAMTOR1;LAMTO	brown	0.18599278	-0.2538293	0.23604877	0.28675376	0.53292419	0.3727053
cg15690822	RFFL;RFFL;RFFL;RFFL	brown	0.31411367	-0.0186201	-0.1558318	0.21218498	0.43950661	0.05310962

cg04099543	P2RY6;P2RY6;P2RY6;P2R	brown	-0.169936	-0.6576712	0.07309001	0.13138096	0.91607903	0.00692136
cg11483312	AL451051.1	brown	0.04545129	-0.5516001	0.04667976	0.29842416	0.94103007	0.13317463
cg16577651	CYP26C1;RP11-348J12.2	brown	0.10913885	-0.3134588	0.12500553	0.18112093	0.5005172	0.06258965
cg23531792	SLC44A2;SLC44A2	brown	0.09657479	0.63213954	0.06035319	-0.1821011	-0.9076696	-0.002811
cg13661182	BCAS1;BCAS1;BCAS1;BC	brown	0.0594127	-0.112171	0.36010596	0.3608829	0.32198746	0.26520765
cg19348676	WFDC5;WFDC5	brown	0.20027573	-0.0183469	-0.0470933	0.19771115	0.31310629	0.07965622
cg19898038	SH2D7;SNORA63;SH2D7	brown	0.36927049	-0.0887878	0.12132012	0.55007401	0.67975105	0.33399848
cg14835333	U6	brown	0.12320575	-0.1465888	-0.2840783	0.13283736	0.3870443	0.0884633
cg14615559	LCN2;LCN2;LCN2;LCN2;L	brown	0.02752479	-0.5393643	0.02618407	0.20218867	0.94104473	0.14795625
cg15848553	RP11-464O2.2;RP11-12J10.3	brown	-0.254815	0.00122406	0.26011562	0.11008775	-0.1523828	-0.0565026
cg19201019	B3GNT8	brown	0.37264711	0.03578421	-0.0673223	0.57231045	0.43131324	0.21559812
cg17801058	MIR22HG;MIR22HG;MIR2	brown	0.17314447	-0.2481074	0.12211761	0.27718931	0.52724332	0.01064953
cg07998581	PDGFRB;PDGFRB	brown	0.30250945	-0.032944	0.03536915	0.24127348	0.42884928	0.35315399
cg27003765	SLC44A4;SLC44A4;SLC44	brown	0.16714383	-0.208891	0.06496775	0.25865595	0.43642668	0.25623106
cg22507772	RP3-470L22.1	brown	0.4852221	-0.0678619	0.14589957	0.46858553	0.61850693	0.43482375
cg09822636	MTSS1;MTSS1	brown	0.1263335	0.59404167	-0.0012322	-0.205529	-0.9207631	-0.0252102
cg11142456	ZNF560	brown	-0.0448706	-0.2259144	-0.130543	0.03095875	0.29404467	0.15111306
cg11625933	GBE1;GBE1;GBE1	brown	0.21584645	-0.0548588	-0.1505564	0.25752839	0.48105017	-0.1953405
cg13477969	IL18RAP;IL18RAP;IL18RA	brown	-0.1920558	-0.5107889	0.07552568	0.04295309	0.83872474	-0.0155776
cg05490745	RP11-	brown	-0.0560601	-0.5857966	0.07810666	0.14964216	0.94810691	0.08205023
cg04349020	IFNGR2	brown	0.13871001	0.62611671	0.03813211	-0.1630519	-0.9135165	0.08026172
cg02268965	SLC8A1;SLC8A1;SLC8A1;S	brown	0.40801657	-0.0817167	-0.272007	0.14177955	0.58938444	0.01074024
cg03989567	LGALS8;LGALS8;LGALS8;	brown	0.19983442	-0.1994643	-0.0005578	0.39723922	0.57194514	0.20466634
cg18881380	IL1R1;IL1R1;IL1R1;IL1R1;I	brown	0.33264109	0.10799969	0.15991083	0.38139084	0.30490738	0.17143079
cg13153661	TOP1MT;TOP1MT;TOP1M	brown	0.19594052	-0.1331807	0.00529408	0.44738176	0.35071515	0.161107
cg04184394	MCF2L2;B3GNT5;MCF2L2;	brown	-0.0929737	-0.5740737	0.0059332	0.16936223	0.94159844	0.04648802
cg10149070	CTC-537E7.3	brown	0.03867472	0.60895909	0.0136437	-0.0722769	-0.8957651	-0.1770183
cg14328641	MS4A3;MS4A3;MS4A3;MS	brown	-0.1007301	-0.5805837	0.09763151	0.24950006	0.94133836	0.05616299
cg26876647	RUSC1-AS1;RUSC1-	brown	0.12510069	-0.159256	0.18645873	0.10663928	0.38098321	0.22105492
cg12919005	RP11-268G13.1	brown	0.38329655	-0.0763226	0.18012013	0.33722101	0.43303988	0.41396013
cg16077279	FBXO44;FBXO2;FBXO2;F	brown	0.10566376	-0.3671943	0.12684612	0.2618364	0.50233462	0.22200003
cg16509978	POLD4;POLD4;POLD4;POL	brown	-0.390371	0.11372774	0.08888294	0.00338667	-0.2878235	-0.2207312
cg08918564	TOLLIP;TOLLIP;TOLLIP;T	brown	0.32344171	-0.1315498	-0.1208912	0.40478205	0.56344208	0.33256989
cg23762445	Y_RNA;DNAJC27-	brown	0.12792381	-0.2334935	-0.1063985	0.12909982	0.5307353	0.10236804
cg07800806	GUSBP11;GUSBP11;GUSB	brown	0.39592007	0.014484	0.29029438	0.40443601	0.48456239	0.36765935

cg25162888	SYNPO;SYNPO;SYNPO	brown	-0.1404651	-0.5429815	-0.0005248	0.1187816	0.90799724	0.00151703
cg20702390	U6;NT5C1B;NT5C1B	brown	0.20998378	-0.1404749	-0.0041155	0.13526118	0.35584855	0.22020885
cg06203221	RP11-371F15.3	brown	0.5968124	0.06604597	-0.0863442	0.24054684	0.48908048	0.28638499
cg07285167	CSF3R;CSF3R;CSF3R;CSF3	brown	-0.0432794	-0.5920867	0.03385127	0.18657497	0.95288378	0.09741087
cg05575594	SEC23B;SEC23B	brown	0.00280443	-0.5763349	0.05631267	0.26737339	0.95487586	0.09984727
cg14315430	DDIT3;DDIT3;DDIT3;MBD	brown	0.18743339	-0.3606007	0.23700718	0.32395454	0.68750991	0.29943791
cg26452476	RP11-	brown	0.3199298	-0.1421939	0.14255335	0.45972306	0.62669257	0.24849433
cg21053989	HPD;HPD	brown	-0.0403089	-0.5872833	0.02174848	0.22553489	0.94575828	0.11484084
cg17588550	RP11-468E2.9;RP11-	brown	-0.1467196	-0.6366509	0.03514606	0.18235702	0.93679325	0.01429231
cg26580095	BTBD8;BTBD8;BTBD8	brown	-0.1265988	0.21942144	0.31040173	0.12148892	-0.1687519	0.01344677
cg20119798	PON1;PON1	brown	-0.0809525	0.23215562	0.04137319	-0.039515	-0.4073593	-0.0280539
cg08075269	RP5-	brown	0.34919503	-0.1071439	0.06526435	0.3946074	0.51990129	0.31421956
cg07773769	PPM1M;PPM1M;PPM1M;P	brown	-0.3288723	0.1458055	0.20787718	-0.1498302	-0.4399314	-0.1373005
cg03079637	BSG;BSG;BSG;BSG;BSG;B	brown	-0.1345258	0.26552096	0.31213246	0.38630043	-0.3874895	0.0315167
cg08058894	TOX2;TOX2;TOX2;TOX2	brown	-0.2298746	0.1064361	0.29600409	-0.110479	-0.3957293	0.05024177
cg00618452	RNU7-20P;EZH2;EZH2	brown	0.5085144	-0.0116169	0.32270044	0.31966918	0.53778449	0.30807035
cg01885102	SLC7A4;SLC7A4;SLC7A4;	brown	0.25591603	0.00718258	0.12911708	0.39272726	0.51233049	0.23202573
cg17139328	RNY3P6;TM9SF2;TM9SF2	brown	0.48117009	-0.0214701	0.09757059	0.61271107	0.50725349	0.49603841
cg17235008	POP5;POP5;POP5;POP5;PO	brown	0.39003827	0.00522228	0.14903726	0.31329618	0.3711461	0.18217084
cg10862944	HAL;HAL;HAL;HAL;HAL;	brown	-0.0559638	-0.5945713	0.05877184	0.22848753	0.95594993	0.09876983
cg22409100	SLC8A1;SLC8A1;SLC8A1;S	brown	-0.1010239	-0.5438847	0.07971462	0.21368506	0.92319742	0.05143894
cg17682828	FXYD7;FXYD7;LGI4	brown	-0.4499057	-0.1265376	0.25438792	-0.0684324	-0.2777841	-0.1854094
cg10031849	HRH1;HRH1;HRH1	brown	-0.2035042	-0.6801668	0.04150021	0.18668341	0.92409503	0.01827327
cg14054393	ZMYND10;ZMYND10-	brown	0.30227198	-0.0275257	0.30568518	0.66539822	0.45423804	0.43310946
cg11025974	CACNB4;CACNB4;CACNB	brown	0.01376972	0.1959832	0.05205252	-0.007108	-0.2292389	0.01052983
cg23982607	GNB1;GNB1;GNB1;GNB1;	brown	-0.0654864	-0.2714445	0.1372605	0.21466146	0.35205335	0.04361845
cg10951224	C10orf26	brown	0.27980135	-0.0683915	0.11629852	0.22931217	0.32478068	0.3517935
cg01649837	NWD1;NWD1;NWD1	brown	0.05646512	0.03296758	0.0493085	0.31003604	0.31844186	0.15148594
cg19976948	BCL11A;BCL11A;BCL11A;	brown	0.24591651	-0.0903012	-0.297574	0.0849504	0.55083978	0.01063539
cg12410283	SOS2;SOS2;SOS2;SOS2;SO	brown	0.2205479	-0.2390067	-0.1779959	0.30313674	0.66732678	0.06569337
cg18329967	LA16c-	brown	0.33046883	0.09731795	-0.1941331	0.03021571	0.28117957	0.11948508
cg23586766	MCFD2;MCFD2;MCFD2;M	brown	-0.026525	-0.5944962	0.00530066	0.22604495	0.96144402	0.10238454
cg25033715	SNX20;SNX20;SNX20;SNX	brown	-0.3477546	0.08765121	0.1640192	0.04410074	-0.2242088	-0.1635497
cg03389538	GP9	brown	-0.0659352	-0.6213444	0.09244479	0.20221904	0.93844683	0.08265122
cg04458969	FRZB	brown	-0.0197283	0.30318158	0.03728017	0.12477095	-0.2998958	-0.0101701

cg02000541	RP11-145A3.2;RP11-	brown	0.16454682	-0.1376899	-0.0609599	0.19260395	0.44460319	0.15390322
cg02402368	ANGPT2;ANGPT2;ANGPT	brown	0.22234595	0.00460784	0.02874634	0.48228408	0.35786123	0.19915234
cg14578677	TLR6;TLR1	brown	-0.0199462	-0.57393	0.03336761	0.16883487	0.93771987	0.1078907
cg07628442	IFNGR2	brown	0.17369093	0.67913755	-0.0338776	-0.1343476	-0.9071082	0.00546468
cg11881599	CLLU1;CLLU1OS	brown	-0.0340234	0.13693886	-0.1931619	0.44112973	-0.26823	-0.0668342
cg18086635	FAM59A;FAM59A	brown	0.02896932	-0.1708581	0.11528708	0.25351025	0.3242944	0.14826753
cg02390806	RP11-	brown	-0.0805413	-0.2194066	0.21425747	0.20619786	0.33926746	0.12730249
cg22068770	MOV10;MOV10;MOV10;M	brown	-0.0979197	-0.6369254	-0.0073682	0.19797594	0.91640604	0.05865835
cg18246603	SH3TC1;SH3TC1;SH3TC1;S	brown	0.26983869	0.04610774	-0.1440438	0.1681838	0.37229518	-0.057732
cg03734611	DNAJC11;DNAJC11;DNAJ	brown	0.32199114	0.00642609	-0.2157505	0.5400379	0.50959117	0.19391475
cg26532320	IRF8;IRF8;IRF8;IRF8;IRF8;I	brown	0.16096641	-0.1017355	-0.2312353	-0.1191873	0.33559369	-0.0008457
cg01185080	ZNF710	brown	0.17667415	-0.3911729	0.18620142	0.30886118	0.64500724	0.19171356
cg03753670	DNAJC11;DNAJC11;DNAJ	brown	0.44320527	-0.1396909	0.1793477	0.49133452	0.67190862	0.4423142
cg15505615	COL9A3;COL9A3;COL9A3	brown	0.32348331	-0.0189328	0.08373245	0.2536287	0.3112971	0.33404167
cg06168149	GPD1;GPD1;GPD1;GPD1;G	brown	-0.0571183	0.13167226	0.28956335	-0.0658612	-0.248905	0.18663809
cg23217262	RP11-488I20.8;RP11-	brown	0.05459953	-0.2875965	-0.0110322	-0.0195137	0.40399821	0.01530322
cg00078513	SNORD115-40;SNHG14	brown	-0.140275	0.20526721	0.33513691	0.09480553	-0.29567	0.09598531
cg13857119	PLEKHG5;PLEKHG5	brown	0.17046288	-0.0799456	-0.2430115	0.24371215	0.29581912	0.15307443
cg03631294	CD79B	brown	0.05496214	0.24916102	0.35534676	-0.0897057	-0.2963388	0.28899388
cg09084613	RP11-515O17.2	brown	-0.1359173	-0.5999072	0.07936571	0.17718614	0.91090121	-0.0138002
cg19038540	S1PR1;S1PR1;S1PR1;RP4-	brown	-0.1246314	0.31471271	0.18512673	-0.0178497	-0.52024	-0.0557221
cg19291926	CDC6	brown	-0.3197701	0.21341726	0.17268078	-0.0730576	-0.456617	-0.1082187
cg26625316	ACACB;ACACB	brown	0.17255556	-0.2118327	-0.2233929	0.26518177	0.47858695	-0.0649207
cg20362821	RP11-505E24.2	brown	0.19995209	-0.2522412	0.0355435	0.6367005	0.6300948	0.21797187
cg08403095	RP11-396O20.1	brown	0.35078348	-0.0722891	-0.1394913	0.17827863	0.47733332	0.01176583
cg03739402	AD000671.1;AD000671.1;Z	brown	0.10223691	0.65446125	0.04941093	-0.0876908	-0.9196605	-0.0068206
cg21888602	SET;SET	brown	0.16104386	0.65129372	-0.0201412	-0.1304696	-0.9083648	0.01464647
cg07617736	RP11-160A10.3;RP11-	brown	-0.0975353	0.17708687	0.04042551	0.03664723	-0.2595125	0.02122562
cg07429188	GPR56;GPR56;GPR56;GPR	brown	-0.1522485	-0.0375715	0.23649111	-0.1233958	-0.2507005	0.0967878
cg24924577	SEMA4B;SEMA4B;SEMA4	brown	-0.1945719	-0.044832	0.12589256	-0.1892513	-0.2239532	-0.0864027
cg04677723	MOGS;MOGS;MOGS;MOG	brown	-0.1000331	-0.5899361	0.02461624	0.23494269	0.94335281	0.06256409
cg09084200	NCAPD3;NCAPD3;NCAPD	brown	-0.4086468	-0.1056729	0.23179738	0.08940027	-0.2140893	-0.0607444
cg19999116	RP11-213G21.2	brown	0.00293165	-0.5635721	0.03624915	0.19453666	0.95457724	0.05141762
cg27340001	TP53BP1;TP53BP1;TP53BP	brown	0.26415228	0.11438031	-0.1935504	0.42316105	0.28755452	0.06903967
cg25899923	RP11-7F17.3;RP11-	brown	0.48548351	0.04537241	-0.006107	0.27639386	0.4776378	0.24941282

cg00441638	MLH3;MLH3;MLH3;MLH3;	brown	0.6126632	0.08257512	-0.0174493	0.32474565	0.51143599	0.23380842
cg02329527	EFHB;EFHB;EFHB;EFHB;E	brown	-0.1141124	0.2633835	0.18251713	0.07669402	-0.3402165	0.12816611
cg20161791	LFNG;LFNG	brown	-0.2974318	0.01747659	0.08132667	-0.0990922	-0.2619048	-0.4412621
cg01697902	CTSG;CTSG	brown	-0.0207045	-0.6031286	0.07459287	0.21173658	0.95982642	0.12074834
cg07952576	RP11-362A9.3	brown	0.00607985	-0.534717	-0.0375405	0.26375495	0.94154093	0.07721502
cg08425278	CDC25B;CDC25B	brown	0.10466664	0.52976577	0.09431833	-0.175577	-0.8770233	-0.0161692
cg02712165	FAM211A;FAM211A	brown	0.24262028	-0.1810938	0.24917008	0.28216636	0.45809659	0.17138501
cg24774354	CORO7;CORO7;CORO7;CO	brown	0.19421106	-0.0824028	0.05335406	0.33284662	0.33928034	0.0444996
cg10604555	PRAP1;RP11-122K13.7	brown	-0.0126163	-0.2212509	0.20284961	0.32203497	0.41694682	0.16056317
cg02822894	AC012360.4;TGFBRAP1	brown	-0.0455907	-0.6116568	-0.0129658	0.20019994	0.93628267	0.06963857
cg12929333	LRMP;RP11-	brown	0.11519361	-0.2057329	-0.2467109	0.10375601	0.45232094	-0.007196
cg09485398	GJD3	brown	-0.0668922	-0.2830043	0.14074929	0.03181812	0.30751178	0.06340422
cg22613968	MDS2;MDS2	brown	0.13945204	0.60383913	-0.0743921	-0.1873128	-0.88676	0.00829164
cg23378736	MDS2;MDS2	brown	0.05853194	0.49852741	0.06635787	-0.2135171	-0.9012507	-0.0154281
cg04903089	HLA-DMB;HLA-DMB;HLA-	brown	-0.1441825	-0.6404029	0.02298275	0.18612308	0.93733841	0.01395139
cg11723452	IGSF3;IGSF3	brown	0.39354027	-0.1194854	0.14515413	0.47689111	0.67753683	0.23342575
cg15419081	KCNE3;KCNE3;KCNE3;KC	brown	-0.1782044	-0.5930447	0.02345762	0.18194479	0.89785599	-0.0134623
cg22098683	HVCN1;HVCN1;HVCN1;H	brown	-0.0304093	0.31697926	0.28969304	0.23989961	-0.4528463	0.16561795
cg05940538	RP11-302J23.1	brown	0.22834259	-0.2297088	-0.079802	0.33067522	0.58918857	0.24755037
cg26930374	TRAJ1	brown	0.06799004	0.64663335	-0.034269	-0.1750904	-0.9026295	-0.0498811
cg00492348	MFSD7;MFSD7;MFSD7	brown	-0.0557778	-0.5973582	0.06516562	0.12201229	0.93440423	0.12560247
cg12657464	SETD2;SETD2;SETD2;SET	brown	0.02578508	-0.5462507	-0.0531604	0.16502935	0.93303231	0.08017411
cg10518739	KB-173C10.1;KB-	brown	0.01922932	0.59110315	0.13251564	-0.206299	-0.9258525	-0.0322958
cg23531488	NDUFA5;NDUFA5;NDUF	brown	-0.2280243	0.17975258	0.2536083	0.01285924	-0.3747557	-0.0203911
cg16990516	GIMAP8	brown	0.10506669	0.63383241	0.00786877	-0.2438711	-0.9186085	-0.0549536
cg01966891	CTD-2536I1.1;CTD-2536I1.1	brown	-0.0337787	-0.2950502	0.17287928	-0.0629572	0.29420671	0.06105151
cg23468927	PTPRCAP;CORO1B;CORO	brown	0.25542714	0.6774188	0.20268586	-0.0466468	-0.8459022	0.05034084
cg16145216	HIVEP3;HIVEP3;HIVEP3;H	brown	0.01381641	0.13666905	0.17026987	-0.1100363	-0.3668725	0.03323343
cg09694086	ARF1;ARF1;ARF1;ARF1;A	brown	-0.0506095	-0.3171031	0.00694895	0.47927182	0.43387836	-0.0088408
cg08397633	SNAP23;SNAP23;SNAP23;	brown	0.14017108	-0.1027225	-0.1727417	0.38429307	0.43834088	0.10111957
cg19772215	TPP1;TPP1;TPP1;TPP1;TPP	brown	-0.0081685	-0.5665572	-0.0078155	0.11893426	0.93893687	0.11490164
cg22038059	C19orf60	brown	-0.1717747	-0.3300661	0.12451852	0.09275341	0.34532187	-0.040086
cg02287214	AP003774.6	brown	-0.1016233	-0.5204459	0.0079205	0.21574798	0.90306379	0.02404847
cg00669330	CXXC11;CXXC11	brown	0.26670155	0.74147978	-0.0002328	-0.064246	-0.8390253	0.08064372
cg02930866	EAF1;EAF1-AS1	brown	-0.1442727	-0.6159149	0.06898118	0.09165116	0.88348443	0.00350816

cg14950616	MKNK1;MKNK1;MKNK1;	brown	-0.0083623	-0.5854675	0.07702785	0.14332277	0.94981257	0.11743989
cg08040565	C10orf95	brown	-0.0262536	-0.5857159	0.02765948	0.10095358	0.94101068	0.09065082
cg01908976	MYO1C;MYO1C;MYO1C;	brown	0.2678286	-0.1273372	0.22547244	0.45868591	0.56033303	0.23904993
cg06744540	CLEC5A;CLEC5A;CLEC5A;	brown	-0.105924	-0.4675945	0.04974992	0.04280255	0.86175318	0.06956226
cg11501059	RARA;RARA;RARA	brown	-0.2883209	0.1813868	0.13291899	-5.85E-06	-0.2088723	-0.1477012
cg14338521	OSBPL5;OSBPL5;OSBPL5;	brown	0.00913607	-0.4899005	0.03464378	0.11803831	0.91061745	0.11832886
cg14058848	PTRH1;PTRH1;RP11-	brown	-0.1828442	0.39029751	0.18068086	0.07673193	-0.3613825	-0.000362
cg11713658	ARHGAP10	brown	-0.183316	0.1200598	-0.1087961	0.01041533	-0.2373975	-0.1043236
cg17050687	RHBDD2;RHBDD2	brown	-0.0317123	-0.5796759	0.02116357	0.22068256	0.94029006	0.13113211
cg11570271	CECR3	brown	-0.2024166	0.03171312	0.08838725	-0.2817429	-0.459163	0.0541366
cg24558747	ERGIC1;ERGIC1;ERGIC1;E	brown	0.26440129	-0.1238941	-0.093661	0.23638485	0.61543952	0.10740435
cg03773647	AP000662.4;AP000662.4;AP	brown	0.25142847	-0.0698075	-0.1655227	0.17113873	0.28974813	0.27016673
cg00443981	C17orf64;C17orf64;C17orf64	brown	0.29271856	0.10001499	0.08909724	0.208535	0.14013243	0.25248891
cg00272709	LAX1;LAX1	brown	-0.3239556	0.17390122	0.13209576	0.06058466	-0.5201116	-0.1765511
cg22595230	CENPB;CENPB;CDC25B;C	brown	0.22617407	-0.1255957	0.13402454	0.26721013	0.41225191	0.27662739
cg24271538	PPARG;PPARG;PPARG;PP	brown	-0.1210862	-0.3098219	0.19096097	-0.0354141	0.3380904	-0.0841355
cg09844336	PPP2R1A;PPP2R1A;PPP2R	brown	-0.2470607	-0.0031509	0.11843496	-0.1055731	-0.224801	-0.1624934
cg02544454	C7orf31;C7orf31;C7orf31;C7	brown	0.24790373	-0.1411902	0.16587756	0.39427846	0.51935252	0.22109578
cg22955741	RP11-98L5.5	brown	-0.3766872	0.08531576	0.30874277	-0.107207	-0.4073848	-0.0433612
cg21553977	BTN2A2;BTN2A2	brown	-0.0658496	-0.5991632	-0.0003916	0.11579845	0.94783889	0.05128243
cg16708012	TMEM119;TMEM119;TME	brown	0.35896631	-0.0622248	0.13286942	0.32149875	0.48301277	0.38454063
cg03366145	LYVE1;MRVI1;MRVI1;MR	brown	-0.0355859	-0.5383885	-0.0243624	0.18761631	0.91000672	0.06763758
cg03295928	RTKN;RTKN;RTKN;RTKN;	brown	-0.0674609	-0.3616401	0.09672505	0.09160177	0.40610412	-0.1032607
cg13666617	IFFO2;IFFO2	brown	0.06619143	0.24308048	0.49003763	0.0576525	-0.2774527	0.29514013
cg26742866	DNAJB4;DNAJB4;DNAJB4	brown	0.12149579	-0.0536272	-0.3385032	0.02058337	0.26571629	-0.0954588
cg01803180	PELP1;PELP1;PELP1;PELP1	brown	-0.0151249	-0.5813131	0.0511649	0.19317777	0.93958206	0.08084298
cg19766489	GIPC2;GIPC2	brown	-0.008711	-0.1351761	0.0928161	0.217424	0.2789952	0.11911223
cg08239375	SUPT7L;SUPT7L;SUPT7L;S	brown	0.43663441	-0.0039393	-0.1472216	0.24268878	0.53488294	0.11367586
cg26857677	STRN4;STRN4;FKRP;STRN	brown	-0.4627346	-0.0049768	0.20926526	-0.0963534	-0.3777452	-0.2405347
cg14097859	MAML3	brown	-0.0168605	-0.548411	0.06057318	0.15835507	0.9439219	0.084205
cg12421481	ACAD10;ACAD10;ACAD1	brown	0.27456253	-0.2056617	-0.0555999	0.37787562	0.58596015	0.28443593
cg10752508	CPOX;CPOX;CPOX	brown	-0.0262364	-0.591267	-0.0143249	0.23318331	0.95300388	0.11232225
cg10672754	U8	brown	-0.1623718	0.22209236	0.09527594	-0.0546582	-0.4053479	-0.0450035
cg19530728	BTBD19;BTBD19;BTBD19;	brown	0.22486796	-0.1401107	0.10435349	0.24408444	0.46558774	0.20562981
cg12134559	TRRAP	brown	0.32732374	-0.0521521	-0.1544316	0.11135942	0.41544872	0.04879955

cg08036492	COX10;COX10;COX10	brown	0.01195982	-0.5712019	0.01189419	0.14909661	0.94705129	0.13233617
cg12286415	LINC00426;LINC00426;LIN	brown	-0.2708772	0.06941465	0.24539356	-0.0326423	-0.3911757	-0.1253653
cg14680496	MAD1L1;MAD1L1;MAD1L	brown	0.32366814	-0.0599237	-0.0140223	0.26964569	0.39379718	0.42407184
cg07976603	RAVER1;ICAM3	brown	-0.0545066	0.21659127	0.22230527	-0.0745191	-0.4173636	0.02700451
cg18503912	FXYD1;FXYD1;LGI4	brown	-0.0156871	-0.5757154	0.02434144	0.19735238	0.95687773	0.13881711
cg07120158	FGD4;FGD4	brown	0.03994772	-0.5560772	0.03810154	0.26360902	0.94785143	0.13566806
cg08934272	WHSC1L1;WHSC1L1;WHS	brown	0.03663953	-0.5417775	-0.0082247	0.32365757	0.94039999	0.11057819
cg06278080	SNAPC5;MAP2K1;SNAPC5	brown	0.04064945	-0.5337925	0.02805296	0.17071958	0.9379442	0.10121673
cg21578596	HLA-DMB;HLA-DMB;HLA-	brown	-0.0932742	-0.5822713	-0.0052228	0.19290614	0.93880488	0.01456049
cg21187770	KIF3C;KIF3C;KIF3C	brown	0.30854512	-0.1085289	0.0395972	0.33740601	0.57067283	0.12346248
cg06685244	CSAD;RP11-	brown	-0.2238069	0.18788339	0.17318931	-0.0227679	-0.2420443	-0.1732456
cg13860869	C3orf24;C3orf24;C3orf24	brown	0.1867622	-0.0835133	-0.145423	0.11367438	0.35277274	0.1195702
cg16303241	SDSL;SDSL;SDSL;SDSL	brown	0.43834831	0.00172985	0.26625049	0.47337724	0.49727635	0.60597997
cg04590344	ZNF517;ZNF517;ZNF517;Z	brown	-0.0241811	-0.5669609	-0.0001558	0.1904339	0.94092118	0.0726901
cg09197274	HK1;HK1;HK1;HK1;HK1;H	brown	-0.0218643	-0.6006156	0.02845189	0.24950188	0.94787778	0.08717463
cg27650859	ZNF335;ZNF335;ZNF335	brown	-0.0853469	0.25413023	0.11708762	0.03960261	-0.2647194	-0.0277319
cg17081586	GRPEL2;GRPEL2;GRPEL2	brown	-0.2992796	0.02737047	0.3733632	-0.1509707	-0.3621859	-0.0276643
cg24621453	Z98044.1	brown	0.00338241	-0.5927192	0.02898176	0.15507196	0.94976069	0.14485039
cg01699630	ARG1;ARG1;ARG1;ARG1;	brown	0.07618324	-0.4961586	-0.0396256	0.1908326	0.91931658	0.13949543
cg13703437	FYB;FYB;FYB;FYB;FYB;F	brown	-0.0369192	-0.6338156	0.05079981	0.19560936	0.93956935	0.12938839
cg00420390	MAD1L1;MAD1L1	brown	0.27998856	-0.0119132	-0.1032443	0.09510038	0.43866147	0.19183017
cg14626483	ZNF236	brown	0.19825083	-0.0609153	0.06864974	0.29235524	0.36882207	0.22859322
cg03301240	SPI1;SPI1;SPI1;SPI1	brown	-0.1333877	-0.5850419	0.04446591	0.18549174	0.88593711	0.02236339
cg20080231	ZNF337;ZNF337	brown	0.01083174	0.16605547	0.30665171	0.11057049	-0.2524431	0.05112305
cg19344626	NWD1;NWD1;NWD1	brown	0.28933353	0.1647076	-0.0227143	0.36655946	0.30817252	0.21011653
cg23964444	FOXP1;FOXP1;FOXP1;FO	brown	0.19538681	-0.3338147	0.04550669	0.46255998	0.71935405	0.21446784
cg07077697	CORO6;CORO6;CORO6;CO	brown	0.25401985	0.01040161	0.06415761	0.36300153	0.35161387	0.22356022
cg20398633	RP11-321E2.4	brown	0.02295048	-0.5542613	0.03287553	0.21923919	0.95049133	0.12303629
cg19595244	AHCYL1;AHCYL1	brown	0.03838086	-0.5331523	0.00297418	0.23732234	0.95137867	0.11393535
cg04604347	NBL1;NBL1;NBL1;NBL1;N	brown	0.17193515	-0.1989227	0.20488421	0.29727577	0.51583239	0.21379032
cg13723441	RP11-998D10.4	brown	0.42184189	-0.026777	0.33754801	0.57426107	0.45532896	0.38764294
cg06468347	ITGAE;CTD-3195I5.5	brown	-0.0394265	-0.5780828	0.05946716	0.24767619	0.94224229	0.09070142
cg24755915	ZFP64;ZFP64;ZFP64	brown	0.01183289	-0.5157187	-0.0443771	0.26109806	0.93683473	0.11303971
cg25930786	TARM1	brown	-0.0567617	-0.5901645	-0.0048474	0.08867765	0.91901278	0.06060555
cg08117235	ZNF692;ZNF692;ZNF692;Z	brown	-0.4541986	-0.0413714	0.16711559	-0.1405994	-0.2655152	-0.2721771

cg19573490	PCYT2;PCYT2;PCYT2;PCY	brown	-0.0556603	-0.5933479	-0.0169753	0.21989386	0.93250808	0.10053631
cg06133360	KCNA10	brown	0.40492741	-0.0356194	-0.1814152	0.25554188	0.59737827	0.10808885
cg22981993	ALS2CR11;ALS2CR11;ALS	brown	-0.0279241	-0.3632043	0.1583542	0.22426562	0.50952598	0.0349541
cg24496915	CCDC34;CCDC34;CCDC34	brown	0.04461299	-0.2110513	-0.0237111	0.27542316	0.44312048	-0.0295122
cg22107476	CHST12;CHST12	brown	-0.2777461	0.09728484	0.17968206	0.08542041	-0.2943687	-0.077754
cg13769596	RP11-285G1.9;TMEM72-	brown	-0.1424152	-0.5009828	0.03519473	0.12711629	0.89115161	-0.0075974
cg01135584	AOAH-IT1	brown	0.03959734	-0.5239083	0.03589658	0.18899561	0.91105291	0.14063903
cg27210863	SH2D4B	brown	0.1254118	0.59133201	0.07515143	-0.1720175	-0.859185	-0.0044067
cg08597832	TOP1MT;TOP1MT;TOP1M	brown	0.18796787	-0.1024036	0.01065173	0.18874302	0.25791303	0.15350703
cg18747276	XBP1;XBP1	brown	0.27834972	-0.1943452	0.12618672	0.32849488	0.65485416	0.11212685
cg01173441	AC015971.2;AC015971.2;A	brown	0.0610834	-0.5179239	-0.0173883	0.27495688	0.9247051	0.06284364
cg02739870	SLC12A7;SLC12A7	brown	-0.1312838	-0.5956918	-0.0093461	0.16842609	0.8966331	0.08075135
cg01087239	KIAA1683	brown	0.18113641	0.6021622	0.15744933	-0.1344919	-0.8587498	0.18925181
cg07162320	RUSC1;RUSC1;RUSC1;RU	brown	0.2992856	-0.035401	0.11828857	0.37815651	0.42675852	0.21072873
cg15717205	ANXA7;ANXA7;ANXA7	brown	0.32055128	0.08395937	0.00664719	0.3811494	0.29584544	0.31498926
cg02646470	GBAP1;GBAP1;GBAP1;GB	brown	-0.0574205	-0.4606141	0.13181017	0.20128269	0.87826314	0.09962713
cg21204860	SEPT9;SEPT9	brown	-0.0486251	0.52088656	0.11753161	-0.2290579	-0.9123237	-0.0740212
cg11746924	CD79B	brown	-0.000125	0.39612387	-0.0039442	-0.1548229	-0.422898	-0.0559891
cg15424045	U7	brown	0.04103195	-0.5388553	-0.0477818	0.27828842	0.93224226	0.09217464
cg06451577	PERP;PERP	brown	0.08530163	-0.1293207	-0.3205945	0.06599201	0.33323714	-0.1246375
cg24408573	NISCH;NISCH;NISCH;NIS	brown	-0.0124282	-0.5867371	0.03168282	0.10113267	0.9337575	0.15502875
cg09866923	GKAP1;GKAP1;GKAP1;G	brown	0.07691439	-0.2106299	0.01965429	0.21029462	0.34165629	0.19580245
cg16727877	CLIP1;CLIP1;CLIP1;CLIP1	brown	0.28637382	0.0033551	-0.1220699	0.27791569	0.31948944	0.06168917
cg14506192	DIABLO;DIABLO;DIABLO	brown	0.19577555	0.66941911	0.13602407	-0.107593	-0.8708776	0.08141113
cg04388919	RP11-143I21.1	brown	0.22150427	-0.2480329	-0.0309401	0.30855442	0.72099449	0.12491175
cg16873386	RP3-434P1.6	brown	-0.1144274	-0.4817962	0.03836807	0.23422271	0.87268157	0.01795267
cg20000185	TMEM30A;TMEM30A	brown	-0.0166471	-0.5800282	-0.0217029	0.16702988	0.93957084	0.09826513
cg20317872	DENND2D;DENND2D;DE	brown	0.05087804	0.54158059	0.1027446	-0.2228952	-0.805709	-0.0045761
cg21461649	CFTR;CFTR;CFTR;CFTR	brown	-0.127104	-0.2794675	-0.4866111	-0.2202581	0.20792418	-0.1806035
cg26504509	AC079767.2;AC079767.2;CR	brown	0.04927578	-0.4877212	-0.113427	0.24691911	0.90397467	0.01284158
cg12265929	TRPV4;TRPV4;TRPV4;TRP	brown	0.28701698	-0.0551801	0.12625578	0.36183292	0.38681034	0.30133992
cg02988947	LIMD2	brown	-0.2938847	0.09519028	0.18679643	-0.271847	-0.4943819	-0.121435
cg01869128	MIOS;MIOS;MIOS;MIOS;	brown	-0.3557586	0.10774622	0.19097177	-0.0664819	-0.3748743	-0.0580522
cg18973954	TAF6;TAF6;TAF6;TAF6;TA	brown	-0.4198745	-0.1052314	0.20751661	-0.0717818	-0.159699	-0.11985
cg17469039	MAFK;MAFK	brown	0.26633946	-0.0699724	0.07330468	0.14545867	0.42928607	0.16215948

cg19764841	LIME1;LIME1;LIME1;LIME	brown	-0.482326	0.03811946	0.08814296	-0.1653657	-0.5102815	-0.1938994
cg02956542	PGLYRP4;PGLYRP4;PGLY	brown	-0.10716	-0.5048267	0.05747751	0.14466112	0.89323066	0.01777043
cg01448863	SLC10A1	brown	0.00786906	-0.5257266	0.032953	0.28480787	0.9395717	0.12838941
cg11197422	Y_RNA;PDE6A	brown	-0.1189339	-0.4389148	0.08008722	0.23183958	0.84913031	0.04861627
cg25953688	CELF1;CELF1;CELF1;CELF	brown	0.21453433	-0.2016082	0.03888455	0.2338243	0.44862785	0.23429281
cg07863022	SEPT9;SEPT9;SEPT9	brown	-0.0538803	-0.6266729	0.0656762	0.16123336	0.91836113	0.08013311
cg13134916	IFLTD1;IFLTD1;IFLTD1;IF	brown	-0.0285455	-0.5425542	0.03447392	0.2425996	0.92682911	0.08414448
cg00337361	APBB1;APBB1;APBB1;AP	brown	0.17055873	0.60803205	0.25081822	-0.0895623	-0.8507647	0.13234589
cg00446123	LIME1;LIME1;LIME1;LIME	brown	0.08710549	0.58310545	0.0640621	-0.1789493	-0.9044832	-0.0095251
cg14001824	CCNE2;CCNE2;CCNE2	brown	-0.1136491	-0.650051	0.05736761	0.14052748	0.90883259	0.05367089
cg11647108	RP11-40F6.2;RP11-	brown	0.08917698	-0.2714269	0.00655632	0.17504156	0.50838013	0.09071332
cg26425555	CTC-461F20.1;RP11-	brown	-0.3899206	0.05703258	0.22008742	-0.1021167	-0.4627504	-0.1578069
cg06729209	RP11-264B17.3;RP11-	brown	0.08976302	0.61211401	0.05834769	-0.1841658	-0.9180319	-0.0300025
cg07424807	PPARG;PPARG;PPARG;PP	brown	0.46651201	-0.0617588	0.35772363	0.30480479	0.52721834	0.33609212
cg03340036	PIGY;HERC3	brown	0.02572799	-0.5460284	0.00589842	0.2986323	0.93589219	0.11682129
cg24395387	PIGG;PIGG;PIGG;PIGG;PI	brown	0.43380215	-0.0407125	-0.0944974	0.37182345	0.56377853	0.13266418
cg03853968	P4HA2;P4HA2;SLC22A4;SL	brown	-0.2302724	-0.6377696	0.05448754	0.08350335	0.83396603	-0.061981
cg23536675	WSB1;WSB1;WSB1;WSB1	brown	0.28437181	-0.0962136	-0.1801079	0.50775402	0.5359892	0.04352598
cg11956003	ANGEL1;ANGEL1;ANGEL	brown	0.28919084	-0.0513332	-0.0752595	0.31633897	0.39934467	0.23386333
cg03048029	GCFC1-AS1;GCFC1-	brown	-0.0172791	-0.5949254	0.01232373	0.21921925	0.93108036	0.08839936
cg22916645	TRAF3IP3;TRAF3IP3;TRAF	brown	0.17085954	0.67903169	-0.0613476	-0.0426517	-0.8396202	0.00520466
cg20644981	RPS3A;RPS3A;RPS3A;RPS	brown	-0.2968253	0.11362948	0.20827859	-0.1349991	-0.4404255	-0.1508066
cg16259605	RP11-774O3.2;RP11-	brown	-0.0803299	-0.5926493	0.06168866	0.18952263	0.91337517	0.07318525
cg07511565	AC007390.5;AC007390.5;A	brown	-0.2723727	0.0451807	0.25740935	0.09828503	-0.2595629	-0.0818006
cg16472011	TOLLIP;TOLLIP;TOLLIP;T	brown	0.30305924	-0.1286427	-0.2078999	0.32445155	0.48011621	0.42122119
cg17004025	PRTN3	brown	-0.14623	-0.4334464	0.13801167	0.1038366	0.82424499	0.03855181
cg19401149	DEFA4	brown	0.07613378	-0.4658941	-0.003199	0.15877744	0.91156151	0.14895536
cg11015597	RAC2	brown	-0.3892671	0.07624668	0.28081832	-0.1345626	-0.4575435	-0.1124107
cg18646784	AL390776.1;ERI3;ERI3	brown	0.07364749	-0.5120788	-0.0744125	0.15413903	0.91828266	0.13848166
cg00429622	FZR1	brown	0.02079601	-0.5705226	0.0023888	0.21903104	0.94564893	0.12470806
cg20647285	HPD;HPD	brown	-0.0644528	-0.5917021	0.03066045	0.2183165	0.92500918	0.0871439
cg08415138	TNKS	brown	-0.0156837	-0.6013271	-0.0332585	0.20354645	0.92980561	0.10072878
cg16450918	USHBP1;USHBP1;USHBP1	brown	0.34819274	-0.0427226	0.13825648	0.36681794	0.54156332	0.18165838
cg02184418	LCK;LCK;LCK;LCK;LCK;L	brown	0.06788474	0.58868302	0.02331039	-0.160755	-0.9052062	0.03203755
cg02997962	C20orf26;C20orf26;C20orf26	brown	-0.0387468	-0.5969658	-0.0038177	0.20398506	0.94191832	0.0727531

cg21725986	FCRL6;FCRL6;FCRL6	brown	-0.2369572	0.09060573	0.07210392	-0.1548736	-0.2797691	-0.0685744
cg26265234	CCKBR;CCKBR;CCKBR;C	brown	-0.195446	-0.438624	-0.2304372	-0.0216233	0.42471912	-0.4126183
cg18637746	CUL3	brown	-0.0483987	-0.5333921	0.04055258	0.24618171	0.91640671	0.08766706
cg27663181	ADORA3;ADORA3;ADOR	brown	-0.0081119	-0.5734821	0.01974206	0.2411785	0.95111414	0.10011033
cg04902126	SLC39A10;SLC39A10;SLC3	brown	-0.0406001	-0.597111	-0.0139543	0.15783131	0.95091198	0.06268527
cg12209737	ST3GAL4;ST3GAL4;ST3GA	brown	-0.0654222	-0.6274621	-0.0078046	0.1748568	0.93072604	0.09203449
cg22984446	FAM216B;FAM216B	brown	0.01322603	-0.5340564	-0.0145323	0.27141703	0.92957294	0.06555507
cg10178523	FNIP2;FNIP2;FNIP2	brown	0.35031372	-0.1329636	-0.0576141	0.31176663	0.64143661	0.1304099
cg25514993	INPP5D;INPP5D;INPP5D	brown	0.08883155	-0.0212397	0.30855611	0.27742322	0.29202381	-0.0496474
cg13083127	PTPN18;PTPN18;PTPN18;P	brown	0.06414602	0.56383	0.13701317	-0.0665504	-0.8999507	0.00857916
cg00463732	ITGAE	brown	-0.0258372	-0.6064203	0.12283829	0.18163203	0.94635184	0.12997778
cg07409200	FAM216B;FAM216B	brown	0.06778472	-0.39161	0.08627245	0.30631327	0.88399127	0.12968224
cg16918625	TGFBR2;TGFBR2	brown	0.16693283	-0.339263	-0.2560793	-0.1857689	0.56843412	0.02454463
cg03328727	SEMA4D;SEMA4D;SEMA4	brown	-0.0309919	0.26275983	0.06225905	-0.0390881	-0.3784114	-0.019868
cg12172984	PSTPIP1;PSTPIP1	brown	0.0609036	-0.5346193	0.00783117	0.21552278	0.93836017	0.15241677
cg00412815	Y_RNA;SEPT9	brown	0.13633316	0.67225532	0.05406166	0.05727803	-0.8563168	-0.0843352
cg03254865	BDP1;BDP1;BDP1	brown	0.10578575	-0.4988714	-0.018701	0.22744129	0.92503174	0.16321618
cg14187466	SNUPN;SNUPN	brown	0.02703083	-0.5681223	0.02126009	0.27014137	0.92797791	0.13662751
cg08199758	CLDN9;CLDN6	brown	0.28117371	0.17115449	-0.0362479	0.11344024	-0.0092998	0.10622614
cg26763948	SLC26A9;SLC26A9	brown	-0.1034809	0.47829022	0.33068068	0.08730359	-0.4718111	0.01849966
cg11181843	ANAPC7;ANAPC7;ANAPC	brown	0.08154134	-0.5270613	0.13115676	0.27105834	0.90356203	0.16544745
cg09398924	WDR48;WDR48;WDR48;W	brown	-0.1695728	-0.3303397	0.05571108	-0.0917047	0.33213199	-0.3245121
cg25137372	RP11-	brown	-0.0134638	-0.4945731	0.06996426	0.24374546	0.9311457	0.09774294
cg25967384	1-Mar	brown	-0.0076897	-0.5625074	-0.0266474	0.21599113	0.94225577	0.08089369
cg05920921	CTA-31J9.2;RP11-557H15.4	brown	0.02156456	-0.5377792	0.01389946	0.22130673	0.93778847	0.12791334
cg02838542	QSOX2	brown	0.01295932	-0.5440496	0.03430987	0.16456681	0.90849383	0.12367708
cg04824767	FADS3;FADS3;FADS3;FA	brown	-0.3717161	-0.0604926	0.09523627	-0.1369481	-0.2932284	-0.1057996
cg17571559	HRH1;HRH1;HRH1	brown	0.00100097	-0.5756118	0.03117266	0.18673796	0.9547464	0.11795326
cg19507032	OXER1	brown	-0.1571283	-0.7065237	0.00301442	0.07111944	0.86641779	0.04078932
cg10719447	PDE4D;PDE4D;PDE4D;PDE	brown	0.07252254	-0.4751419	-0.0783591	0.23285697	0.91296463	0.09429801
cg27232174	XXYLT1;XXYLT1;XXYLT	brown	-0.0588211	-0.5992437	0.05966248	0.22503677	0.9254147	0.14392886
cg06021353	CTD-2292M14.1	brown	0.05789647	-0.5256465	0.02997873	0.17114719	0.93630265	0.12443274
cg11285173	COX5BP2	brown	-0.0068723	-0.5930983	0.05188908	0.19550459	0.93499949	0.11077377
cg14405088	RP11-165H23.1	brown	0.06333166	-0.5032573	-0.0378837	0.21489845	0.92328048	0.127815
cg18974455	ASPH;ASPH;ASPH	brown	-0.0321888	-0.6091555	-0.0290084	0.15821213	0.93851559	0.05146696

cg23028178	NID1;NID1	brown	0.34876782	-0.0851332	0.07164534	0.36671859	0.420758	0.22738439
cg23449757	CTD-2116N20.1	brown	0.51725141	0.0042228	-0.0205087	0.3668411	0.60685095	0.18951083
cg03453618	RAC2;RAC2;RAC2	brown	-0.0365368	-0.5762433	-0.0171001	0.23177504	0.93024489	0.06067849
cg27421385	RNF145;RNF145;RNF145;R	brown	-0.0231936	-0.5740223	0.04827486	0.22895491	0.93959826	0.07978305
cg18691862	LRRC3C	brown	0.06612076	0.61216649	-0.0433725	-0.174337	-0.9159069	-0.0488792
cg01500402	MLST8;MLST8;MLST8;ML	brown	-0.1938561	-0.5791181	0.02293836	0.11035115	0.87578727	-0.0651263
cg07757091	MLL3	brown	0.35616891	-0.1362228	-0.1894257	0.20127436	0.56356236	0.08590816
cg23916460	RP11-264B17.3;RP11-	brown	0.1299423	0.62211618	0.21819449	-0.1320246	-0.8816455	0.05153348
cg20246452	MARK3;MARK3;MARK3;	brown	0.10143065	-0.499683	0.00172873	0.29612103	0.9141018	0.20413153
cg20348374	C17orf108;C17orf108;C17orf	brown	-0.3968583	0.11052644	0.13012908	-0.1359682	-0.4877345	-0.1643892
cg08149223	C10orf71;RP11-	brown	-0.3737817	0.22408151	0.10384016	-0.0217223	-0.4293276	-0.1577673
cg14023009	ARHGEF10;ARHGEF10;AR	brown	-0.0885783	-0.6084919	0.0882735	0.18081297	0.89307311	0.03294622
cg23397030	RP11-1078H9.5;RP11-	brown	-0.0063297	-0.5753802	0.03382915	0.24480847	0.95094338	0.11637407
cg27553955	KCNG3;KCNG3;MTA3	brown	-0.3348149	-0.1372146	0.24596522	-0.1174308	-0.2731093	-0.0874382
cg17451586	BRD2;BRD2;BRD2	brown	-0.1455937	0.09534869	0.23783481	0.06365279	-0.2984016	0.07967484
cg13914531	IRF5;IRF5;IRF5;IRF5;IRF5;I	brown	-0.2309954	-0.645906	0.01536053	0.0578722	0.87369862	-0.0033705
cg11683242	LCK;LCK;LCK;LCK;LCK;L	brown	0.1039819	0.6194298	0.07068928	-0.1719309	-0.9088897	0.10617051
cg06840062	DTNB;DTNB;DTNB;DTNB;	brown	0.04043155	-0.5332529	-0.0136261	0.10516328	0.91912144	0.11713928
cg12542099	RP1-286D6.2;RP1-	brown	0.2565821	0.00037746	0.16843003	0.54212375	0.35878017	0.23796754
cg07225906	MPST;TST;TST;MPST;TST;	brown	-0.2018683	-0.6125335	0.0983449	0.07758266	0.81850326	0.0285914
cg03173141	CD68;CD68	brown	0.22100281	-0.0656016	-0.2404307	0.12148015	0.43108907	0.14906704
cg10611016	C11orf42	brown	0.02763939	-0.54094	-0.029852	0.23174177	0.94631904	0.0866585
cg04353474	AC105750.1;AC105750.1	brown	-0.0361632	-0.5881856	-0.0064725	0.19229486	0.94812957	0.04640649
cg11502375	LRG1;LRG1	brown	-0.0301733	-0.58885	0.02999277	0.16117479	0.94047025	0.09977661
cg08783988	ARID5B	brown	0.11001784	0.67287969	0.13032996	-0.0618547	-0.8868676	-0.0157275
cg20843475	MAP7	brown	0.40119519	-0.0984082	0.18397563	0.33592026	0.5494068	0.17568791
cg00423969	FER1L5;FER1L5;FER1L5;FE	brown	-0.0503427	-0.5875078	0.13094211	0.15836375	0.92564848	0.12715006
cg13765621	CD1D	brown	-0.1034482	-0.5191967	-0.0096248	0.09693871	0.90654703	0.01133724
cg05796704	FBXO2;FBXO44;FBXO2;F	brown	0.25430986	-0.1362326	0.10173125	0.38611025	0.40641055	0.14592347
cg01507019	MT1G;MT1G;MT1G;MT1H;	brown	0.06380216	-0.3397095	0.14036853	0.18376749	0.40313625	0.19561854
cg25350859	INPP5J;INPP5J;INPP5J;INP	brown	-0.093663	0.43062159	0.32684811	0.08181325	-0.4318957	0.08650733
cg10683527	NEIL3;NEIL3	brown	-0.0460993	0.28191157	0.22953552	0.07518414	-0.4258323	0.17372968
cg09085347	KIAA0319L;KIAA0319L;KI	brown	-0.1075478	-0.496686	0.06498832	0.19384676	0.87610928	0.0415301
cg09674170	CLDN9;CLDN6	brown	0.23216499	0.10947113	0.09059281	0.14729038	-0.051396	0.29208094
cg19826663	U3	brown	0.02865809	-0.4624486	0.0141791	0.14246484	0.9059045	0.12274412

cg03066525	TRIM24;TRIM24;TRIM24;T	brown	0.43500079	-0.0706147	0.07835511	0.33465334	0.57339521	0.10731387
cg03836521	SNORA30;SRCAP;SRCAP	brown	0.02214117	-0.5441314	0.0240307	0.27676612	0.93372524	0.07469431
cg16170874	DPEP3;DPEP3;DPEP3	brown	0.30708279	-0.0524537	0.19079887	0.25593392	0.31005429	0.50952799
cg13763027	DARS;DARS;DARS;DARS;	brown	0.07753262	-0.0314155	-0.360282	0.02357234	0.38682952	-0.0935032
cg04355430	AP000265.1	brown	-0.0393159	-0.5425258	-0.0182938	0.24946744	0.93012446	0.03555278
cg19217739	MAST4;MAST4;MAST4	brown	-0.1013779	-0.0401325	0.2357118	-0.0951838	-0.2937011	0.21035138
cg17412005	TOE1;MUTYH;MUTYH;M	brown	-0.0390386	-0.3695883	0.1577105	0.28916787	0.82420593	0.06322728
cg26056693	RP11-561C5.5;RP11-	brown	-0.1330467	0.3156527	0.03327235	-0.1209808	-0.5147639	-0.0889296
cg02251680	FGD4;FGD4	brown	0.04513025	-0.5514878	0.06057359	0.2397317	0.93815864	0.13320362
cg05840650	CD81;CD81;CD81	brown	0.19305048	0.35349814	0.32107226	0.41196186	-0.2911829	0.22311842
cg10098414	PIGY;HERC3	brown	0.08310765	-0.4998446	-0.0538378	0.28531656	0.92656632	0.12959745
cg15209200	RP11-213G21.1;RP11-	brown	0.00360454	0.59102246	-0.0152122	-0.2333435	-0.948003	-0.098892
cg05088356	MAST4;MAST4;MAST4	brown	0.08888124	0.46089585	0.08691996	-0.178255	-0.8587328	0.00851582
cg07149948	UBL7;UBL7;UBL7;UBL7;U	brown	0.13499771	0.47434759	0.44081591	0.23113923	-0.3936838	0.18889024
cg13267916	GORASP2;GORASP2;GOR	brown	-0.0081048	-0.5560951	-0.0092884	0.23038494	0.92165025	0.10395573
cg16408081	PTPRCAP;CORO1B;CORO	brown	0.14863818	0.61453264	0.20886979	-0.0916759	-0.8327021	0.05224032
cg11179544	LGR5;LGR5	brown	-0.0024213	-0.4289253	0.03297585	0.23728484	0.87729655	0.08388942
cg16891376	FLT3LG	brown	-0.2493294	0.166816	0.14278061	-0.1478824	-0.5306547	0.01267281
cg25693439	ADCY9	brown	-0.0269816	-0.5836858	0.07556418	0.22511223	0.94053238	0.10064424
cg23468816	ARID5B	brown	0.12931305	0.68466485	0.12845484	-0.0411522	-0.8896783	0.01906626
cg09604501	AC098869.1;RBM47;RBM47	brown	0.18694055	-0.2269451	-0.3094641	0.12369488	0.57754031	0.00981798
cg12146213	RP11-111K18.2	brown	0.04069469	-0.5421075	-0.025765	0.2555971	0.93323414	0.08985594
cg13672525	MDM4;MDM4;MDM4;MD	brown	0.22897847	0.1627626	0.13857393	0.45395021	0.27042678	0.07906348
cg22820233	FCAR;FCAR;FCAR;FCAR;	brown	0.0311429	-0.5577484	0.00617219	0.23295554	0.938248	0.15534424
cg01275030	RNF13;ANKUB1;RNF13;R	brown	-0.1346478	-0.4905892	0.10493234	0.18298483	0.86353004	0.03997803
cg26725604	U3	brown	0.04544688	-0.523795	0.02787108	0.23103944	0.94223787	0.11494275
cg12134894	HNRPLL;HNRPLL	brown	0.04210324	-0.4197778	0.04985795	0.31290476	0.87507155	0.16465262
cg20065449	OR2M5	brown	0.31417572	-0.1779728	-0.1948163	0.16720444	0.59890703	0.04969221
cg11993173	RGS20;RGS20;RGS20;RGS	brown	-0.0543744	-0.5462611	0.00348401	0.20031343	0.88605301	0.04785758
cg20994007	RBX1;RBX1;XPNPEP3;XP	brown	-0.2013742	0.14270888	0.16860773	0.02568609	-0.3111741	-0.0688596
cg16028117	ATG7	brown	-0.0708639	0.22256838	0.27039831	0.01657845	-0.3516317	-0.1492389
cg03001246	RP11-	brown	0.0173692	-0.3133739	-0.426806	-0.0233924	0.41843976	-0.0863333
cg21491092	HIF1A;HIF1A;HIF1A;HIF1	brown	-0.0315569	-0.2297807	0.16093987	0.15389185	0.30739488	0.11672964
cg18270217	RHOBTB2;RP11-	brown	0.00549929	-0.5795693	-0.0059287	0.15791521	0.95188296	0.05611335
cg13828018	GEN1;SMC6;SMC6	brown	-0.0013798	-0.5618028	-0.0565976	0.21569801	0.94776643	0.07806381

cg04323387	RP11-58E21.3;RP11-	brown	-0.1351156	-0.5356634	0.02898238	0.10050556	0.86423822	-0.091719
cg22793645	CD79B;CD79B;CD79B;CD7	brown	0.08783045	0.31114966	0.21322843	-0.0579884	-0.3235603	0.12892842
cg15391464	SNORD42B;RPL23A;RPL23	brown	-0.344458	0.07308133	0.23852557	-0.1115615	-0.4140108	-0.0917565
cg03499017	ACOT7;ACOT7	brown	0.49842	-0.058972	0.01074803	0.41210605	0.60296858	0.52415468
cg27554551	VASN;CORO7;CORO7;CO	brown	0.52817726	0.02657142	0.01785822	0.38455313	0.48172931	0.29176763
cg05586855	NR1H3;NR1H3;NR1H3;NR1	brown	-0.0433226	-0.587902	-0.0036748	0.1721747	0.91437252	0.05294792
cg05260783	EZR-AS1;EZR	brown	-0.2094634	0.14625131	0.00439821	-0.0465721	-0.3402376	-0.1089834
cg13937155	SCARF1;SCARF1;SCARF1;	brown	-0.1188006	-0.677461	-0.0004572	0.13029512	0.90657074	-0.0191423
cg02771489	IL7R;IL7R	brown	-0.3162456	0.16110021	0.21832739	-0.1443507	-0.426922	-0.1262601
cg09731767	RNH1;RNH1;RNH1;RNH1;	brown	-0.1265249	-0.4872109	0.07950528	0.22745711	0.86617978	0.03085863
cg03025830	FGF17;FGF17;EPB49;EPB4	brown	0.06350328	-0.4090862	-0.0150543	0.23775415	0.4882169	0.09195369
cg03960698	TTC32	brown	0.05096452	-0.5244614	-0.0584585	0.26505435	0.93021515	0.11414286
cg17313269	TRIM38;TRIM38;TRIM38	brown	0.04867159	-0.5417701	0.04129098	0.22836856	0.94017441	0.13200926
cg17310897	OSBPL5;OSBPL5;OSBPL5;	brown	0.03923679	-0.4692951	0.06424361	0.13356674	0.91474992	0.12592363
cg10016364	FCRL6;FCRL6;FCRL6	brown	-0.0742925	0.18416848	0.20726084	-0.0796875	-0.3100874	0.11700276
cg25654945	AC100802.3	brown	0.08037519	0.34953222	-0.075886	0.01074789	-0.3657309	0.06510999
cg04316051	MARS;ARHGAP9;ARHGA	brown	-0.2289466	0.13927956	0.34498718	0.13602112	-0.4141948	-0.0197623
cg04742550	ITGAX;ITGAX;ITGAX;ITG	brown	-0.2924375	-0.4467713	0.03761322	0.09989138	0.74469901	-0.2091209
cg21031775	Y_RNA;SNTB2;RP11-	brown	-0.05573	-0.4179006	0.12922116	0.22407364	0.82574672	0.07977311
cg08907799	RP11-558A11.3	brown	0.07031313	-0.5190743	-0.0559961	0.19973594	0.92923348	0.15647438
cg19607845	FBXO9;FBXO9;FBXO9;FB	brown	0.06227952	-0.5010798	0.02196781	0.24577753	0.91410615	0.12954419
cg18884413	FAM13A;FAM13A;FAM13	brown	0.32910855	-0.144916	0.17743973	0.23987636	0.49687662	0.1437287
cg04730794	DOCK2;DOCK2	brown	0.01560184	-0.555806	-0.0472145	0.12744856	0.93586358	0.11055176
cg10584828	RFWD2;RP11-	brown	-0.099143	-0.6346717	-0.0624363	0.19508237	0.92010753	0.02051522
cg08390622	GALK2;GALK2;GALK2;G	brown	-0.2175487	0.2050382	0.33296619	0.07168733	-0.4741929	-0.0736446
cg05063632	COL4A2;COL4A2-AS1	brown	0.08824173	-0.0518233	-0.061202	0.24457383	0.31955382	0.1205311
cg12931148	RTN4R;RTN4R;RTN4R;RT	brown	0.15988768	0.63106005	0.15255807	-0.1332661	-0.8376578	0.06127183
cg04858110	SCRN1;SCRN1;SCRN1;SCR	brown	0.1875135	0.64976275	0.06183287	-0.0680111	-0.8658426	0.11332775
cg21284750	CMTM5;CMTM5;CMTM5;C	brown	0.23084005	0.43104611	0.09322353	0.11468402	-0.3854508	0.13666396
cg14716166	AC012652.1;AC012652.1;A	brown	0.05862554	-0.5262116	-0.0481692	0.25998885	0.93531833	0.12104097
cg00842918	GAS7;GAS7;GAS7	brown	0.02213328	-0.5338719	0.00106938	0.20846572	0.92505216	0.04435222
cg01440934	MKNK1;MKNK1;MKNK1;	brown	0.0109116	-0.5583574	0.00072802	0.2425013	0.94187409	0.12906061
cg10725937	TREM2;TREM2;TREM2	brown	0.00010282	-0.5243558	0.02174868	0.09989691	0.91411494	0.12248254
cg27642691	INSRR;NTRK1;NTRK1	brown	0.03885468	-0.3585485	-0.0505274	0.10034003	0.52396282	-0.1341722
cg08841239	RP11-160H22.3;RC3H1	brown	-0.1994523	0.1100907	0.24987834	-0.0244455	-0.2913071	0.08923085

cg19789589	SORBS3;SORBS3;SORBS3;	brown	-0.2106015	0.09963703	0.1205653	-0.0530835	-0.3430647	-0.0735644
cg21530469	NLRC4;NLRC4;NLRC4;NL	brown	0.08996171	-0.5042848	-0.0035842	0.12070573	0.91343254	0.17111137
cg22356324	HK1;HK1;HK1;HK1;HK1;H	brown	-0.3643183	0.07581399	0.08485582	0.00292295	-0.3411453	-0.189563
cg26987302	RP5-1096D14.3	brown	-0.1456313	-0.6663117	0.0436099	0.14625067	0.90167066	0.02874782
cg13723983	C9orf50;METTL11A	brown	0.18888363	-0.0614064	-0.0777708	0.21055061	0.26555402	0.19297822
cg09043230	AP1G1;AP1G1;AP1G1;AP1	brown	-0.0019715	-0.5619979	0.02818941	0.13847642	0.94427198	0.09120784
cg23459144	PODNL1;PODNL1	brown	-0.1802274	-0.264194	-0.0737788	-0.1433617	0.28517708	-0.3952389
cg06196379	TREM1	brown	-0.0234834	-0.4470188	0.05117784	0.11648877	0.88771504	0.09467691
cg15149219	AE000662.92	brown	0.03724345	0.6129608	0.12515866	-0.0623349	-0.8618768	-0.0311974
cg05008701	OLR1;OLR1;OLR1	brown	-0.0042142	-0.5627057	-0.0508652	0.22605658	0.92707775	0.0830351
cg14797308	POU2F1;POU2F1;POU2F1;	brown	-0.0583112	-0.6573366	-0.0182858	0.17121635	0.8935604	0.08253172
cg26604499	DHODH;DHODH;DHODH;	brown	0.16567194	0.64901849	0.07111175	-0.0663935	-0.8289525	0.11117828
cg02311193	RP11-	brown	-0.1106801	-0.7069459	0.0211327	0.15109193	0.91086105	0.03827807
cg13692972	OTUB1;OTUB1;OTUB1;OT	brown	0.03728034	-0.4972714	-0.0465133	0.1574294	0.9218755	0.12536943
cg18107006	HNRPLL;HNRPLL;HNRPL	brown	-0.0021501	-0.4882643	0.03653541	0.27511159	0.91097149	0.07882626
cg21640508	IL4R;IL4R;IL4R;IL4R;IL4R;I	brown	-0.1553687	0.29868793	0.22596468	0.07979959	-0.4106905	-0.0409373
cg14364242	CHRNA3;CHRNA3;CHRN	brown	0.09739122	0.63777284	0.13273384	-0.1279416	-0.883776	-0.0552186
cg06036677	C1orf159;C1orf159;C1orf159	brown	0.07208361	-0.1649756	0.12898579	0.09357103	0.29778421	0.0851147
cg08958168	SLC12A4;SLC12A4;SLC12	brown	-0.1308816	0.29029631	0.2899801	0.03029025	-0.499946	0.04810843
cg00519069	CD97	brown	-0.0948725	0.31540276	0.26114836	0.12050909	-0.4298698	-0.101588
cg18560638	MIR623;UBAC2;UBAC2;U	brown	0.11393985	0.54129548	0.02235666	-0.0308037	-0.8150774	-0.0677118
cg06406619	ELANE	brown	-0.1769711	-0.6073848	0.08652683	0.14824814	0.87583752	0.00124465
cg02929855	CACNA2D4;CACNA2D4;C	brown	0.04574541	-0.5043711	0.06242895	0.27926504	0.91021949	0.24628632
cg19006608	CTC-537E7.3	brown	-0.049576	0.50411277	0.08752785	-0.0697637	-0.8547426	-0.030238
cg15210526	MPZL1;MPZL1;MPZL1;MP	brown	0.02986849	-0.51695	-0.0474145	0.26066914	0.92944791	0.09606929
cg14310772	RP11-290F5.1	brown	-0.0589371	-0.6352252	-0.0050155	0.16166161	0.90616487	0.05987841
cg16176984	RP11-941F15.1;RP11-	brown	0.06481862	-0.4412408	-0.0332937	0.22595994	0.87849007	0.07914415
cg25634693	CREB1;CREB1;CREB1;AC0	brown	0.06696982	-0.512165	0.04248009	0.24991152	0.91044657	0.11349278
cg03764274	CCDC137;C17orf90;C17orf9	brown	-0.0928558	0.157977	0.28822522	0.01913566	-0.3342382	0.22195124
cg15615312	LAT;RP11-	brown	0.13791121	0.64625041	0.21391182	-0.1178963	-0.8814331	0.03825132
cg12741420	IRF4;IRF4;IRF4;IRF4;IRF4	brown	-0.4871065	-0.0579928	-0.1308279	-0.2317742	-0.4038091	-0.2024684
cg03999085	TRPM1;TRPM1	brown	0.16766247	0.38590411	0.17207944	0.14233495	-0.3660774	0.26453315
cg21859019	C1orf130	brown	-0.1941769	-0.7111854	-0.0134724	0.12527508	0.86435809	-0.1012072
cg00854392	SEPT1;SEPT1;SEPT1;SEPT1	brown	-0.0462204	0.2703128	0.3819257	-0.0417157	-0.541094	0.4208069
cg12836863	ZAR1L;BRCA2;BRCA2;BR	brown	-0.0140539	-0.571623	0.04890474	0.24876468	0.94295893	0.12012762

cg16622018	EXOSC4;CTD-3065J16.9	brown	0.21504398	0.21593855	0.39323287	0.46954365	-0.2090427	0.30363672
cg14082278	U6	brown	0.07961642	-0.5311614	-0.0012815	0.25079729	0.93591269	0.13489885
cg07677892	AC097495.2;AC097495.3;A	brown	0.01426239	-0.5785325	-0.0524441	0.20750108	0.91396222	0.11046277
cg25683268	TMEM100;TMEM100	brown	0.15207807	-0.0237648	-0.0715536	0.23036839	0.34464007	0.10660513
cg21877714	IMPDH2;IMPDH2;IMPDH2	brown	-0.0434716	0.4026803	0.39903406	0.23244033	-0.3768464	0.13934636
cg18761133	STAT1;STAT1;STAT1;STA	brown	-0.1365154	0.10655177	0.17526516	-0.0022975	-0.2713339	0.0357889
cg19197419	UBE2C;UBE2C;UBE2C;UB	brown	-0.3117531	0.15283475	0.20106206	-0.0202306	-0.3360524	-0.1607493
cg02913140	MEF2C;MEF2C;MEF2C;ME	brown	0.01828628	-0.4568686	-0.0689557	0.16235807	0.88725551	0.10415111
cg19284751	MARCO;MARCO;MARCO;	brown	0.0067241	-0.5598374	0.01805455	0.16233907	0.94367882	0.10487908
cg05485462	CLDN9;CLDN6	brown	0.32175103	0.24098143	0.13233021	0.11783298	-0.117206	0.19225449
cg19254378	IKZF4;IKZF4;IKZF4;IKZF4;	brown	0.02660583	-0.5160509	0.01107983	0.24789713	0.8879591	0.1355499
cg14593639	GPR126;GPR126;GPR126;G	brown	0.04554226	-0.1740996	-0.1354847	0.09158407	0.39204582	-0.1191997
cg14999035	ANKRD28;ANKRD28;ANK	brown	0.01766362	-0.5553295	-0.1492697	0.18900011	0.90798979	0.10502993
cg11230346	RGS3;RGS3;RGS3;RGS3;R	brown	-0.2507374	0.33550633	0.26753466	-0.0620231	-0.5080477	-0.1018574
cg05941378	PPIB;PPIB;PPIB	brown	0.01397743	-0.5543103	-0.0033055	0.24008362	0.93296038	0.09754405
cg11244291	USP36	brown	0.04255448	-0.5445449	-0.0777072	0.15713303	0.9225324	0.10564103
cg04051212	PNPLA8;PNPLA8;PNPLA8;	brown	0.02689958	-0.551717	-0.0184748	0.21621841	0.90338468	0.1069688
cg04602039	SEC62-	brown	-0.1115848	-0.6979109	0.08471485	0.10678644	0.88257289	0.0775747
cg14187687	SAMD4A;SAMD4A;SAMD	brown	0.05123988	-0.535523	-0.0279396	0.23888717	0.92883577	0.12173268
cg12987701	VDAC2;VDAC2;VDAC2;V	brown	-0.2237726	0.14682331	0.21632049	0.19029027	-0.2430953	-0.1197449
cg07575056	MAPKAP1;MAPKAP1;MA	brown	0.23853149	-0.1257355	-0.0478259	0.22350239	0.33202405	0.17112565
cg05867620	CTB-11I22.2;CTB-11I22.1	brown	0.09859233	-0.5184813	0.05566325	0.26568132	0.91473556	0.16142566
cg01241390	ZCCHC9;ZCCHC9;ZCCHC9	brown	0.08550742	-0.5097844	-0.0522526	0.21449424	0.90960039	0.12265991
cg22282062	AC012307.2;DUSP2	brown	0.02777556	0.50943087	0.15389779	-0.1384618	-0.8794898	0.06564918
cg18856300	ENPP4;ENPP4;RP1-	brown	-0.043485	-0.5700985	0.0945136	0.2101007	0.92371047	0.09194278
cg24824128	C1orf38;C1orf38;C1orf38	brown	-0.1577927	-0.6181809	-0.0178381	0.15296105	0.88404057	-0.0225613
cg04346064	STX16;STX16;STX16;STX1	brown	0.38730084	-0.1202772	0.00104802	0.33637186	0.59514753	0.18282134
cg04524040	CREB3L3	brown	-0.0315739	0.19444167	0.19485702	0.24853029	-0.2741616	0.11029559
cg05396087	TDRD12;TDRD12	brown	0.32368627	-0.0951096	-0.0747552	0.14705095	0.45201255	0.02931973
cg20925954	DUSP5;RP11-	brown	0.05565034	0.39444689	0.23130237	0.12257685	-0.3740783	0.03204948
cg07944900	SNORD112	brown	0.16078647	-0.0176163	-0.0755722	0.20106108	0.33004216	0.15588278
cg15334250	EPHB3	brown	-0.0783938	-0.5414152	0.03279186	0.18677122	0.89421483	0.0192212
cg02258175	NCOR2	brown	0.2882	0.06020037	0.17821324	0.18971473	0.26875975	0.17628816
cg05904013	IRF5;IRF5;IRF5;IRF5;IRF5;I	brown	-0.0675329	-0.5867126	0.01653845	0.05709369	0.92461192	0.08308064
cg15780361	ALS2CR11;ALS2CR11;ALS	brown	0.14475028	-0.1615001	-0.0441209	0.2534675	0.50116733	0.13994845

cg15361231	GLRX2;GLRX2;GLRX2;GL	brown	0.0610789	-0.5314519	0.02383053	0.26041055	0.92781547	0.10639057
cg05186458	TFDP2;TFDP2;TFDP2;TFD	brown	0.50176195	-0.0059392	-0.1798318	0.32184206	0.5667593	0.11331454
cg01033844	CDC25B;CDC25B	brown	0.0718814	0.55273111	0.11690505	-0.1800224	-0.8675039	0.09057477
cg18945039	OVGP1;OVGP1;OVGP1;O	brown	0.08331088	0.47674939	0.16523356	0.05261924	-0.3766352	0.11272453
cg26246049	RP11-253D19.1	brown	0.03535301	-0.5504737	0.06296699	0.2635583	0.91581729	0.12779466
cg02150868	GM2A	brown	-0.0229639	-0.5864595	0.07804578	0.1236157	0.92762451	0.19293469
cg15284102	BTN3A1;BTN3A1;BTN3A1	brown	0.40558142	-0.1631866	-0.1859224	0.20964124	0.63145144	0.10952198
cg17346017	SNORA63	brown	0.02981549	0.54369541	0.11447305	-0.0897659	-0.8336181	-0.089971
cg00382999	NCK1;NCK1;NCK1	brown	0.03057334	-0.5501166	-0.0073935	0.23325583	0.94175024	0.1082453
cg05784862	KSR1	brown	0.09468683	0.68338045	-0.0442746	-0.0665102	-0.8112341	-0.0659299
cg01792666	SRCAP;SRCAP;RP11-	brown	-0.4620464	-0.0048197	0.15484624	-0.2019161	-0.3890537	-0.2185998
cg12044599	PTPRCAP;CORO1B;CORO	brown	0.24985625	0.65864455	0.21625632	-0.0239568	-0.8003868	0.13523966
cg06474207	RP11-69H7.2;RP11-69H7.2	brown	-0.2689317	0.20175223	0.22098772	-0.0663487	-0.2823309	-0.0765208
cg25678125	ST3GAL5;ST3GAL5;ST3GA	brown	-0.1183044	-0.0160781	0.1562264	-0.0598136	-0.2402084	-0.0716985
cg20819482	TNKS	brown	0.09058208	-0.4966263	-0.0055031	0.28953828	0.9172819	0.06849592
cg15784124	PITX2;PITX2	brown	-0.1813224	-0.4842079	-0.0972487	-0.0854659	0.42542713	-0.2517382
cg17980508	IFI44L;IFI44L;IFI44L;IFI44L	brown	-0.0503168	-0.6052395	-0.0280797	0.16388125	0.90810813	0.1106028
cg26225827	ZAP70;ZAP70	brown	0.04320013	0.62159468	0.15827351	-0.1689404	-0.8832243	-0.0191146
cg17875481	ADRBK1;ADRBK1	brown	0.16871367	0.33928139	0.48795669	0.23640355	-0.2355325	0.39261534
cg20772590	SEPT9;SEPT9	brown	0.00290728	0.5536427	0.12526316	-0.1944954	-0.9087955	-0.0264719
cg27432516	RP11-558A11.3	brown	0.0661776	-0.4918107	-0.0616723	0.10323032	0.90004659	0.12022193
cg10135341	CAV2;AC073130.1;CAV2;C	brown	-0.002716	-0.5592871	-0.0306281	0.17779119	0.91959723	0.08480379
cg21544713	RP11-163N6.2;RP11-	brown	0.32425945	0.03231766	-0.0645957	0.36818747	0.30928498	0.26181674
cg14957346	JUP;JUP;JUP;JUP;JUP	brown	0.3713241	-0.0282499	-0.0494012	0.26576758	0.41235826	0.17558227
cg10237171	PPARG;PPARG;PPARG;PP	brown	0.06020445	-0.5217428	-0.0024552	0.23312766	0.9360764	0.11255148
cg17075729	COQ9;COQ9;AC009052.12	brown	0.45956908	0.03957667	0.04731208	0.31069817	0.44831589	0.2478163
cg10056280	U7	brown	0.0355215	-0.5515712	0.00204118	0.23620725	0.93523865	0.10291888
cg23687322	CHN2;CHN2;CHN2;CHN2	brown	0.21917958	-0.1365036	-0.0790281	0.05896554	0.4429567	0.06433105
cg05837922	SFT2D3	brown	0.24436066	0.48993002	0.0555942	-0.0444412	-0.4934594	0.0813886
cg21205978	APOL6	brown	-0.2380391	0.07644282	0.20776458	-0.0989264	-0.2549087	-0.1292746
cg20033652	NIN;NIN;NIN;NIN;NIN;NI	brown	-0.0231802	0.14982975	0.17674416	0.29062595	-0.2347643	0.05143388
cg04170334	ZC3H8;ZC3H8;ZC3H8;ZC3	brown	0.01100953	-0.5637834	-0.0521141	0.19354605	0.92753344	0.09216575
cg04753771	GPR126;GPR126;GPR126	brown	-0.1152189	-0.3207719	-0.0421607	0.00329168	0.35785528	-0.122878
cg13221508	APP;APP;APP;APP	brown	0.03492568	-0.5282954	0.03768481	0.26758484	0.9315922	0.11854653
cg21835874	AC130689.5;SMG6	brown	-0.3026644	0.09140125	0.1533104	-0.1882873	-0.5009403	-0.0705552

cg05265015	7SK;PFKFB3;PFKFB3	brown	-0.2272812	-0.7158192	-0.0529535	0.04088021	0.79853217	-0.060267
cg13394762	MARK3;MARK3;MARK3;	brown	0.09837857	-0.5197351	0.07024742	0.28004377	0.92486801	0.13071189
cg21044995	MAP6	brown	0.06113729	0.60815044	-0.0792952	-0.1304927	-0.8401441	-0.0121739
cg16814786	NCOA4;NCOA4;NCOA4;N	brown	-0.0526392	-0.6490561	0.02014848	0.19357521	0.91417986	0.0842796
cg07740110	TRPV3;TRPV3	brown	0.12864755	-0.1762924	0.09323132	0.35667755	0.47204964	0.09694771
cg06204222	RP11-	brown	-0.008117	-0.5568391	-0.060496	0.24991869	0.93806873	0.05472068
cg09153826	RP11-344B5.4;RP11-	brown	-0.1969884	0.05558017	0.2220924	-0.0807898	-0.1823964	-0.0710919
cg16598508	LA16c-444G7.2;RP11-	brown	-0.008082	-0.5773448	0.01355857	0.21200157	0.90586409	0.14189431
cg15080495	ADGB;ADGB;ADGB;ADG	brown	0.01316048	-0.5579242	0.01579444	0.10289858	0.93130615	0.09773571
cg00054047	AP001625.6	brown	0.227705	0.57571253	-0.1257047	-0.0496962	-0.7148364	-0.0218291
cg13885437	PECR;PECR;PECR	brown	-0.2094769	0.12054202	0.10578924	-0.1030824	-0.3977432	-0.1743569
cg02608914	GORASP2;GORASP2;GOR	brown	-0.0828774	-0.6320091	0.02326767	0.15755254	0.93579474	0.01726092
cg06495586	LNX1	brown	-0.1086617	-0.5657461	0.09786298	0.19464822	0.89373842	0.05975326
cg24494617	RP11-87E22.2;RP11-	brown	-0.0215255	0.15763741	0.16780802	0.02673092	-0.2684422	0.06250984
cg13287659	MAP1S	brown	-0.291173	0.10696618	0.17286821	-0.0748661	-0.3589029	-0.1244938
cg27158141	ST6GALNAC3;ST6GALNA	brown	-0.2382101	-0.4220165	-0.2706751	-0.0376918	0.39857195	-0.3963433
cg11650948	METTL20	brown	0.35762166	0.28769206	-0.0082365	0.14429141	0.05533022	0.26883457
cg20259191	SCPEP1;SCPEP1;RP5-	brown	0.37609314	-0.0537137	-0.0835895	0.56158295	0.45827695	0.23805208
cg15142796	MRPL3;MRPL3;MRPL3;MR	brown	0.04231395	-0.5347071	-0.079921	0.11140445	0.91989761	0.12885725
cg19025685	CYB5RL;AL357673.1;RP11-	brown	-0.063693	-0.5427393	0.01611036	0.1823272	0.92454719	0.06159743
cg08563773	ID3	brown	0.00955905	-0.5442161	-0.0277569	0.23717718	0.91427156	0.08788989
cg09565754	ADCY9	brown	0.04633618	-0.529774	-0.0257763	0.199915	0.92627878	0.12142565
cg00598013	CTLA4;CTLA4	brown	0.03860451	0.62339837	0.13805949	-0.152588	-0.8925295	0.00100995
cg04205769	DPH5;DPH5;DPH5;DPH5;D	brown	0.03820865	-0.5403998	-0.0219194	0.23653012	0.93186979	0.10253629
cg04645556	PLCB1	brown	0.01842904	-0.5645648	0.00061184	0.220674	0.94915712	0.09148058
cg21027759	IL19	brown	0.05481129	-0.5144948	-0.0295802	0.09538206	0.92096247	0.11934009
cg18233010	TCF7L2;TCF7L2;TCF7L2;T	brown	-0.0386026	-0.5738183	0.10066471	0.20475187	0.90937523	0.09929213
cg20326258	KCNH3	brown	0.1766641	0.45301004	0.27489612	0.11276947	-0.4107672	0.34273643
cg20318536	MAP3K6;MAP3K6	brown	0.05865927	0.30260757	0.49980169	0.16345546	-0.4110937	0.27938144
cg24955845	SLFN13;SLFN13;SLFN13;S	brown	-0.2345484	-0.7554995	0.00106787	0.04546914	0.8019609	-0.0500729
cg06130949	UPP1;UPP1;UPP1;UPP1;UP	brown	-0.073466	-0.4150882	0.07116764	0.23656277	0.82757082	-0.0044349
cg26898932	NSRP1;NSRP1;NSRP1	brown	0.06642013	-0.5039633	0.00779817	0.26189767	0.89009024	0.13632537
cg18158438	SLC22A11;SLC22A11;SLC2	brown	0.06083513	0.45955779	-0.0872479	-0.0799352	-0.7053815	-0.0600834
cg23220439	RP11-	brown	0.12616503	0.6636868	0.0761926	-0.0874774	-0.8180697	0.07552852
cg04182865	RNF14;RNF14;RNF14;PCD	brown	0.10937129	-0.4628845	-0.0084062	0.24618633	0.92086108	0.1108755

cg19777783	SLC15A4;SLC15A4;SLC15	brown	0.34211501	0.03309774	0.11558129	0.31924407	0.29458987	0.23758006
cg23467627	ABC12-	brown	0.00733763	-0.4295646	0.12178638	0.30635503	0.88893343	0.11764761
cg18042806	AGXT2L2;AGXT2L2;AGXT	brown	-0.3099618	0.13642027	0.2007553	-0.211455	-0.5676413	-0.1477269
cg25056645	VAMP1;VAMP1;VAMP1;V	brown	0.02048835	-0.5480888	-0.0457473	0.15754365	0.92724014	0.11038588
cg20431939	GPR56;GPR56;GPR56;GPR	brown	0.18711773	0.38997738	0.24702652	0.16094543	-0.3134457	0.14994881
cg24102749	CLEC5A;CLEC5A;CLEC5A;	brown	-0.0071098	-0.3920762	0.07785219	0.05973142	0.84887212	0.05831705
cg07291836	TP53RK;TP53RK	brown	0.03037824	-0.5442406	0.00728986	0.24288919	0.91912119	0.11620802
cg23599224	RP11-584P21.2	brown	0.07969255	-0.5095372	0.04681135	0.20300717	0.90884739	0.13812574
cg13992911	AC116351.2	brown	-0.1404362	0.2225661	0.17199653	-0.1303015	-0.3805985	0.02937386
cg03769206	AC010376.1	brown	0.04261749	-0.5501806	-0.0353991	0.127621	0.93412387	0.11244056
cg22108311	LRRC41;LRRC41;LRRC41;L	brown	0.04893837	-0.5504479	-0.0269776	0.2487593	0.93319639	0.10462914
cg11894057	RP11-134P9.3;RP11-134P9.3	brown	0.01180114	-0.5510547	0.00433547	0.07386244	0.92037761	0.07144548
cg16567676	HPGDS;HPGDS	brown	-0.0163292	-0.6152841	-0.0054546	0.201602	0.90789057	0.09849557
cg02641539	TM7SF4;TM7SF4;TM7SF4;	brown	-0.1766685	-0.3550689	0.00996871	0.01629373	0.74622924	-0.0572439
cg25278941	RP11-12A2.3	brown	0.01738217	-0.340194	0.08521816	0.27793415	0.82123203	0.14157357
cg18027122	ABCC1;ABCC1	brown	-0.1507887	0.15263266	-0.0385801	-0.1872869	-0.4520025	-0.1026374
cg09774932	WNT5B;WNT5B;WNT5B;W	brown	-0.3398232	-0.7548356	0.01913514	0.03640179	0.82234976	-0.1070155
cg06418013	UBXN2A;UBXN2A;UBXN	brown	0.05817261	-0.5278254	-0.0462159	0.07812874	0.91234833	0.13948613
cg20100970	SH3TC2;SH3TC2;SH3TC2;S	brown	-0.1321833	-0.4313107	0.01955679	0.16495907	0.82535077	-0.0838031
cg08825489	RP11-	brown	-0.1887619	-0.5925135	0.02243652	0.13705162	0.83408133	-0.0257187
cg22176040	SORBS1;SORBS1;SORBS1;	brown	-0.3568112	0.07919672	0.0334238	-0.2246654	-0.4094669	-0.1451602
cg08584521	RNF145;RNF145;RNF145;R	brown	-0.2572918	0.04548457	0.31906062	0.01468842	-0.2560709	-0.1108791
cg10325497	LCK;LCK;LCK;LCK;LCK;L	brown	-0.0262282	0.41433012	0.22918956	-0.1273849	-0.8414719	-0.0173225
cg12387699	RP11-792D21.2;RP11-	brown	0.33535466	0.05913235	-0.0321766	0.26077946	0.48145564	0.07630538
cg07628474	CTSG;CTSG	brown	0.0407861	-0.5229082	-0.0499057	0.11851879	0.92993689	0.10358334
cg01222160	CRB1;CRB1	brown	0.3340346	-0.0131227	0.11836838	0.17389101	0.3940658	0.20750881
cg17682392	TRAJ1;TRAJ2	brown	0.01332162	0.56002254	0.12995306	-0.1263297	-0.8746154	-0.0021084
cg24210717	GPD1;GPD1;GPD1;GPD1;G	brown	0.13204618	0.15343481	0.4497625	0.05594467	-0.1712702	0.24935017
cg19520710	DDIT3;DDIT3;DDIT3;MBD	brown	0.3140615	-0.1942701	0.17010256	0.37665036	0.58575222	0.32431637
cg14148107	RP11-404F10.2	brown	-0.0974119	0.23665852	0.08824238	0.01169284	-0.2983307	-0.033522
cg18620155	MARVELD1;MARVELD1;	brown	-0.2112696	-0.7065047	0.02389052	0.05356777	0.82205588	-0.1352655
cg05862597	FAM179A;Y_RNA;FAM179	brown	0.07190604	0.60794049	0.07693077	-0.1498667	-0.8817532	0.05133103
cg00387445	RP11-	brown	-0.1797018	-0.692003	-0.0284138	0.10990362	0.814721	0.08408543
cg15063381	FARP1-AS1	brown	0.1631646	0.63509488	0.09969219	0.02367145	-0.8312912	0.06796847
cg21761853	Y_RNA;FLOT1;FLOT1	brown	-0.1768904	0.41120989	0.09559689	-0.2009465	-0.8402069	-0.1336708

cg04238983	MIR612;NEAT1	brown	-0.0398859	-0.6248128	0.00529	0.22530621	0.91373039	0.11063913
cg00103115	CCL24	brown	-0.0617935	0.4196309	0.36628138	-0.0030938	-0.5104054	0.17681654
cg25426467	FAM167A	brown	0.03269861	-0.5191713	0.02050919	0.17407295	0.92350893	0.10072413
cg07872499	TET2;TET2;TET2;TET2;TET	brown	-0.1547619	-0.681085	0.01397697	0.0523012	0.81922523	0.05607293
cg08198817	SLC8A1;SLC8A1;SLC8A1	brown	0.09897763	-0.4995505	0.09804048	0.27036813	0.90704099	0.14973756
cg12118504	RGL2;RGL2;RGL2;RGL2;R	brown	-0.032833	-0.6478458	0.0231184	0.19019808	0.9233304	0.05563581
cg04846203	MPZL1;MPZL1;MPZL1;MP	brown	-0.0978043	-0.6629122	0.04155185	0.13765689	0.90089608	0.05919812
cg18994841	ARHGEF3;ARHGEF3;ARH	brown	0.1589551	0.67561864	0.12380118	-0.0920802	-0.8351426	0.07944939
cg09151516	RGL3;RGL3;RGL3;RGL3;R	brown	-0.0568288	-0.4944519	-0.0016014	0.2297389	0.89923924	0.01453609
cg09557913	ZCCHC8;ZCCHC8;ZCCHC8	brown	-0.1013898	-0.6822151	0.05890024	0.14026535	0.88792185	0.0727736
cg18377670	SPEG;SPEG;SPEG;SPEG;SP	brown	-0.1362382	0.17127897	0.09583975	-0.0834444	-0.2633447	0.01079983
cg03106245	SPI1;SPI1;SPI1;SPI1;SPI1;S	brown	-0.0570083	-0.6569771	0.05408261	0.20039762	0.90703471	0.11705121
cg21201760	INPP4A;INPP4A	brown	0.06362594	0.40181597	0.06491711	-0.2300935	-0.8354714	0.01494168
cg07884764	CCDC88B;CCDC88B	brown	-0.2377186	0.14973975	0.18325345	-0.287393	-0.5536677	-0.1197603
cg26284800	CDC42-	brown	0.02913436	-0.572501	-0.0372645	0.21130703	0.91607804	0.13904928
cg24379812	RP11-711M9.1;AC073046.25	brown	-0.0380946	0.51813789	0.15125501	-0.1810414	-0.8665564	-0.0319559
cg10828914	RAP1GAP;RAP1GAP;RAP1	brown	-0.1523232	0.22866753	0.34377241	0.10054901	-0.3382326	0.19515674
cg24550026	BCL2L15;BCL2L15;BCL2L1	brown	0.10639641	-0.4887131	-0.0280051	0.22042958	0.883276	0.11948038
cg12859943	SNX14;SYNCRIP;SNX14;S	brown	0.11124682	-0.4925482	0.05248048	0.24897829	0.9102059	0.13321873
cg16526526	NLRC4;NLRC4;NLRC4;NL	brown	0.05052198	-0.5400709	-0.0453103	0.10931778	0.9173861	0.10263327
cg03753385	SLC52A2;SLC52A2;GS1-	brown	-0.2259172	-0.485963	0.01230775	0.08683593	0.76610036	-0.0579474
cg02723617	SLC4A2;CDK5;CDK5;SLC4	brown	0.05114638	0.61725132	-0.0031481	0.00504563	-0.8013258	-0.0464561
cg06193788	DPY19L3	brown	-0.1321476	-0.612641	0.04164303	0.09884224	0.91294361	-0.0257434
cg06989393	snoU13;AC019118.2	brown	-0.1339867	-0.4448943	0.04293502	0.10109483	0.79235642	-0.0299366
cg08423714	RP1-	brown	-0.4564182	0.02469374	0.25508564	-0.0855636	-0.4120884	-0.1906275
cg20618771	TTC38	brown	-0.2404766	-0.4473954	-0.0274986	0.09375717	0.74234528	-0.1574544
cg22757101	TBXA2R	brown	-0.1896975	-0.6404665	-0.085742	0.04852464	0.80156841	0.03358125
cg20825710	KRTAP5-2;RP13-25N22.1	brown	0.10411765	0.34795345	0.17186076	-0.0275291	-0.4211754	0.22125459
cg20151982	MMRN1	brown	0.07811291	-0.3951691	0.0369174	0.29517767	0.88971142	0.14452883
cg10393581	ELANE	brown	0.06002922	-0.5320113	0.10151611	0.24759868	0.8946294	0.13177239
cg18169682	snoU13;SH3BP5;SH3BP5;S	brown	0.12543304	-0.4832103	0.00773429	0.25517991	0.91325499	0.19489102
cg04988978	MPO	brown	-0.1017561	-0.3499299	0.02614534	-0.007307	0.76690973	-0.0632943
cg17839611	GNGT2;ABI3;ABI3;GNGT2;	brown	0.04858941	0.57261799	0.19186104	-0.1695301	-0.859463	-0.015854
cg13721397	FAM73B;FAM73B;FAM73	brown	0.03704777	0.09579913	0.32421735	0.00687056	-0.4269623	0.23811582
cg12655939	DPY19L3	brown	0.03579642	-0.5547504	0.02578572	0.18952982	0.93457526	0.11814107

cg08977295	WDR43;Y_RNA	brown	0.08089175	-0.5046017	0.08705897	0.26962281	0.91363398	0.09920598
cg26812411	ARHGAP32	brown	-0.1889995	-0.2020057	-0.1136516	-0.1588633	0.18783427	-0.2233936
cg23789402	RP11-	brown	0.38968692	0.05175997	-0.1154074	0.27472526	0.41578462	0.15739433
cg04325862	C9orf100;C9orf100;CA9	brown	0.18511444	-0.1459925	0.11537075	0.24366927	0.3869976	0.32299473
cg20112357	DHRS9;DHRS9;DHRS9;DH	brown	0.12484214	-0.3849345	0.01816251	0.25844439	0.88715912	0.10807004
cg24903314	ARHGAP1;ARHGAP1;ARH	brown	0.03280347	-0.5240182	0.03465656	0.26337707	0.89897307	0.12970565
cg15647654	HEXB;HEXB	brown	0.04767525	-0.5884544	0.03352783	0.23757194	0.90579743	0.12664785
cg21031026	SREBF1;SREBF1;SREBF1	brown	-0.0516949	-0.6281337	-0.0346929	0.11531223	0.85074058	0.06015287
cg16195752	FMO1;FMO1;RP1-45C12.1	brown	0.01876445	0.47693093	0.08011364	-0.2058497	-0.8422683	0.01110869
cg08866535	PIK3C2B;RP11-739N20.2	brown	0.33832713	0.09499358	-0.0062459	0.45711079	0.18936209	0.38011932
cg17330765	CNKSR1;CNKSR1;CNKSR	brown	0.16544267	-0.1048445	-0.0514562	0.27636935	0.31515996	0.23649994
cg26005232	NUDC;NUDC	brown	-0.1724311	-0.0476231	0.0520697	-0.1127738	-0.235383	0.00723992
cg03573795	SPATA13;SPATA13;SPAT	brown	0.08368454	-0.4585629	-0.0575747	0.23976611	0.89531931	0.10836393
cg20601288	RP11-758P17.2	brown	-0.321868	0.1712185	0.12100078	-0.0091302	-0.4988699	-0.1976027
cg01532849	TCF7L2;TCF7L2;TCF7L2;T	brown	-0.00688	-0.5799576	0.04261057	0.21151052	0.89796008	0.1133836
cg02991207	RP11-165H23.1	brown	0.06510084	-0.5121015	-0.00472	0.18039004	0.91555668	0.10293932
cg05000446	C9orf131;C9orf131;C9orf131	brown	0.03422012	-0.5427678	-0.0701879	0.11127477	0.91918524	0.10581767
cg23062385	PHACTR2;PHACTR2	brown	0.21186229	-0.0601667	0.21024018	0.11681423	0.33098631	0.12644356
cg00986133	KCNMA1;KCNMA1;RP11-	brown	0.21184037	-0.2854273	0.0194791	0.27876576	0.70357739	-0.0397568
cg14161241	PLAGL1;PLAGL1;PLAGL1;	brown	0.25295907	-0.2508779	0.22207791	0.17641507	0.49015354	0.25014669
cg27518211	RP11-111K18.2	brown	0.06374543	-0.5043026	0.12851249	0.2732705	0.89611091	0.11386018
cg03211711	NLRC4;NLRC4;NLRC4	brown	0.05462679	-0.5366663	-0.0461382	0.20034378	0.93933281	0.12157406
cg03998212	NRXN2;NRXN2;NRXN2	brown	-0.137992	0.34282611	0.2461504	0.07546912	-0.3636003	-0.0091667
cg26188803	SNX27;SNX27	brown	0.06773695	-0.5357713	-0.0053588	0.22153667	0.92747312	0.08674793
cg11862874	UGGT1;UGGT1	brown	-0.0360762	-0.2965486	-0.2360929	0.05968047	0.51901096	-0.0992209
cg09123760	DIABLO;DIABLO;DIABLO	brown	0.13866212	0.57305181	0.27705292	-0.0352736	-0.7762467	0.0881774
cg19271640	BSG;BSG;BSG;BSG;BSG;B	brown	0.09963756	0.35817793	0.41776616	0.45201284	-0.3886868	0.1680634
cg18843330	LGALS1;LGALS1;LGALS1;	brown	-0.3151424	0.21895668	0.20776127	-0.0269892	-0.4152523	-0.0790038
cg21497653	UBA7;UBA7;UBA7;UBA7;	brown	-0.1003174	-0.6597388	0.03687483	0.10179737	0.88832965	0.04060798
cg20896049	CLEC7A;CLEC7A;CLEC7A;	brown	0.08907125	-0.5229615	0.15940633	0.27954649	0.90487691	0.17017306
cg25292745	RP11-561C5.5;RP11-	brown	-0.1142769	0.27476446	0.02301075	-0.0919876	-0.4813432	-0.0458519
cg04886642	USH1G;OTOP2	brown	-0.1843742	-0.6011371	-0.1540226	-0.0506533	0.52358101	-0.3012476
cg02695390	GYG1;GYG1;GYG1;GYG1;	brown	0.02766515	-0.4810746	0.01608692	0.22337542	0.89605317	0.0908349
cg16939977	GPR56;GPR56;GPR56;GPR	brown	-0.1231199	0.04847764	0.03218188	-0.0496785	-0.230644	0.0103881
cg00510842	Y_RNA	brown	0.04459013	-0.5196365	-0.0205718	0.2157427	0.92740942	0.11957126

cg11595214	HEXB;HEXB;HEXB;HEXB	brown	0.02590917	0.45236293	-0.0018068	-0.0096889	-0.4731507	-0.1019098
cg23381867	AC016912.3	brown	0.07296662	-0.5075152	-0.0341886	0.26813542	0.92275016	0.13749856
cg18634760	CPB2;CPB2-AS1	brown	0.06334018	-0.5027985	-0.0848199	0.21246889	0.92627656	0.09586242
cg21376136	CACNB4;CACNB4;CACNB	brown	0.20678904	0.27073905	0.15080679	0.04699808	-0.2029821	0.14415612
cg16238026	ZNRF2;ZNRF2;ZNRF2	brown	-0.2214258	0.04885529	0.26200122	-0.0669815	-0.2763099	-0.0691484
cg19035578	FAM102A;FAM102A;FAM	brown	-0.3413991	0.19849299	0.25474503	0.06333739	-0.4615883	-0.1001523
cg02064830	FAM129C;FAM129C	brown	-0.0230707	0.47357897	0.14258535	-0.2125463	-0.8426662	0.01236889
cg15458313	GPLD1;GPLD1;GPLD1;GPL	brown	0.03353354	-0.5425876	-0.05827	0.14793531	0.93608133	0.09398459
cg08939373	C11orf41;C11orf41;C11orf41	brown	0.09519139	-0.4321725	0.02601731	0.1552026	0.88236673	0.12792201
cg03678274	MIR1537;LYST;LYST;LYST	brown	0.08493135	-0.458109	-0.0505788	0.11915071	0.89036464	0.10206999
cg14985891	CASQ2;CASQ2	brown	0.05627179	-0.5291424	-0.011365	0.16684534	0.93335918	0.09464244
cg06373940	ERCC3;ERCC3;ERCC3;ERC	brown	0.04833351	-0.5479326	-0.0466922	0.26618413	0.92936346	0.1343411
cg00009088	VPS37C;VPS37C;VPS37C;	brown	0.0562714	-0.5204177	-0.0256202	0.28321653	0.92676364	0.1485486
cg18847562	NAB1;NAB1;NAB1;NAB1;	brown	0.10856644	-0.4815868	0.02460071	0.24160886	0.90933283	0.14801212
cg12503394	TMEM184A;TMEM184A;T	brown	-0.1049927	0.129273	0.05724867	-0.0876417	-0.3216046	-0.0467483
cg04737087	AC007040.11;ANKRD53;A	brown	-0.1791621	0.11482962	0.32075005	0.10222531	-0.3136772	0.00468476
cg07407658	ABI1;ABI1;ABI1;ABI1;ABI	brown	-0.1129624	0.31998119	0.05076561	0.03274574	-0.3886752	-0.0484591
cg10752406	AZU1	brown	0.03644052	-0.5321991	0.04377126	0.27001324	0.89843414	0.11813907
cg06235649	PISD;PISD;PISD;PISD;PIS	brown	0.02352921	0.27267534	0.36127558	0.09945029	-0.408543	0.25706405
cg02778694	SEMA4D;SEMA4D;SEMA4	brown	0.07303547	0.38376073	0.22089964	0.05009816	-0.4045685	0.2255031
cg23456595	AP003419.16	brown	0.31313467	0.72585368	0.23819383	0.03158266	-0.7518961	0.22980974
cg10155715	RP11-135J2.3	brown	0.07485062	-0.4514939	-0.0531281	0.19345402	0.90001751	0.05626344
cg26647549	POR;POR;POR;POR	brown	-0.0394949	-0.6392108	0.07237825	0.15337175	0.87019138	0.02517981
cg13443575	SLFN13;SLFN13;SLFN13;S	brown	-0.0467269	-0.6357752	0.0238938	0.16202249	0.87512424	0.05163761
cg01013627	AC090587.4;STIM1;AC0905	brown	-0.3953205	0.15696163	0.04993748	-0.1561153	-0.4703946	-0.2026672
cg12453401	AJ009632.3	brown	0.01517473	-0.5104819	-0.076148	0.10426755	0.92008385	0.0386308
cg05558046	GAS7;GAS7	brown	-0.1054263	0.40693745	0.32108083	0.01053215	-0.6618504	0.0786092
cg01565438	SLFN13;SLFN13;SLFN13;S	brown	-0.1570784	-0.6763606	0.0807098	0.1499609	0.85062077	-0.0420858
cg08816602	MAMDC2;RP11-195E11.3	brown	0.13862712	-0.348523	0.03923656	0.26414103	0.86334467	0.14153311
cg13753423	EGR3;RP11-459E5.1	brown	0.08876182	-0.5308993	0.05553193	0.24926324	0.90948985	0.10682613
cg10713515	COMMD3;COMMD3;COM	brown	0.04483162	-0.4377247	-0.091366	0.17585482	0.87277047	0.0592434
cg14510967	C11orf21;C11orf21;TSPAN3	brown	-0.2735829	-0.613295	-0.1430075	0.00642969	0.69020154	-0.10609
cg02205401	SEC31A;SEC31A;SEC31A;S	brown	0.05880835	-0.5306218	0.07502225	0.26452967	0.9228568	0.15120028
cg20760846	H2AFV;H2AFV;H2AFV;H2	brown	0.27627126	0.07045012	-0.1450323	0.40872142	0.32802264	0.21266464
cg07268239	CCDC28B;CCDC28B;CCDC	brown	-0.3531729	0.17704997	0.14125128	-0.1239279	-0.5406145	-0.2133337

cg09507934	ORAI2;ORAI2;ORAI2;ORA	brown	-0.0526043	0.12032139	0.32041792	0.04579931	-0.383163	0.11711526
cg16306654	ACOT7;ACOT7	brown	-0.0946483	0.41509975	0.31361322	0.22652351	-0.4848205	0.0708829
cg13926377	ADD1;ADD1;ADD1;ADD1;	brown	0.1215872	-0.48939	0.01925693	0.22339116	0.91743323	0.13712797
cg02302574	SLC46A3	brown	0.07294956	-0.5060839	-0.0171229	0.28513447	0.93296163	0.09644537
cg09860323	ADGB;ADGB;ADGB;ADG	brown	0.10561334	-0.4935739	-0.0242477	0.10598339	0.91623961	0.1190102
cg09833738	IL1RAP	brown	0.0535511	-0.5042636	-0.1333536	0.20393757	0.88468019	-0.0200086
cg04256521	U6;LIMK2;LIMK2	brown	0.02583387	-0.5351524	0.00203389	0.1223379	0.93014785	0.06068729
cg12959840	CASP1;CASP1;CASP1;CAS	brown	-0.0982452	-0.5027061	0.11917634	0.20354923	0.85919197	0.06149923
cg26371324	RNF31;RNF31;RNF31;RNF	brown	-0.0135283	0.36125095	0.09642766	0.08330161	-0.3554389	6.57E-05
cg22807026	RCAN1;RCAN1;RCAN1;RC	brown	0.08224848	-0.4937291	-0.0773713	0.12663564	0.91375881	0.10133572
cg26336059	RAB13;RAB13;RAB13;RAB	brown	-0.1252292	-0.6893347	-0.0447593	0.10019533	0.84541868	-0.0475525
cg13119003	OR6P1	brown	0.09568933	-0.5135756	0.01609401	0.2343992	0.88899198	0.14047384
cg11367172	VOPP1;VOPP1;VOPP1;VO	brown	0.05463438	-0.4574897	0.05292646	0.27155445	0.8964788	0.11398048
cg07412002	TMEM30A;TMEM30A	brown	0.00562218	-0.5595776	-0.052367	0.15102012	0.92844517	0.09759247
cg13442459	ARHGAP22;ARHGAP22;A	brown	0.0440039	0.1911479	0.19267877	-0.069883	-0.2987114	0.06471109
cg05644028	PANK2;PANK2;PANK2;PA	brown	0.05382647	-0.4505749	0.02358866	0.2479252	0.89599566	0.13744758
cg09516349	SSBP4	brown	-0.2806429	0.22996644	0.15030028	-0.1044983	-0.4966208	-0.1190702
cg05913072	RP11-412B14.1	brown	0.13236332	0.57811753	0.00296649	0.00494616	-0.8169196	0.01219586
cg02451018	C21orf7;C21orf7;C21orf7;C2	brown	0.0280647	0.44145693	0.14199322	0.32726957	-0.5159264	0.04954544
cg03417903	C20orf112	brown	0.39334762	0.13215896	0.00096898	0.3861026	0.22459358	0.26153285
cg14480116	SPRED2;SPRED2	brown	0.07265849	-0.5163625	-0.0300861	0.12671301	0.92137728	0.1320937
cg06157991	MKL1;MKL1;MKL1;MKL1;	brown	-0.0913446	-0.4048741	0.12890166	0.13652028	0.78224098	0.09603127
cg12412636	NEK9;NEK9;NEK9;NEK9;N	brown	-0.0703851	-0.5085185	-0.0194963	0.18149019	0.85355673	-0.017329
cg21048416	RP11-	brown	-0.1995384	0.03373133	0.2277794	-0.0883444	-0.2197811	-0.1013618
cg08399831	DLD;DLD;DLD;DLD;DLD;	brown	-0.2134939	0.15720274	0.20821341	-0.0206499	-0.4476089	-0.0297449
cg24727216	ASXL1	brown	-0.0149814	0.5233638	0.18235635	-0.0653254	-0.8178708	0.01249533
cg23018755	MAFG;MAFG;MAFG	brown	-0.0449071	-0.6394131	0.0264862	0.11134243	0.85038083	0.00781343
cg27114796	U7;IGF2BP3;IGF2BP3;IGF2	brown	0.07351569	-0.5129648	-0.0046852	0.24050354	0.93412682	0.12411722
cg05464506	AC105393.2;AC105393.2	brown	0.05119629	0.59431813	-0.0387437	-0.1787781	-0.8586333	0.08090447
cg19902228	AGXT2L2;AGXT2L2;AGXT	brown	0.01574476	0.2989054	0.49010517	0.07041041	-0.3734404	0.21746453
cg21524602	RP11-436D10.3;ADO	brown	0.10704087	-0.4765213	0.20259875	0.20543426	0.88016153	0.15377937
cg20451589	ATP5G3;ATP5G3;ATP5G3;	brown	-0.0993494	0.39227304	0.27090061	0.07950143	-0.4900963	0.01563833
cg02876332	MAST4-	brown	0.02656758	-0.5581281	0.03537162	0.21391766	0.8987872	0.0707286
cg17640894	BSG;BSG;BSG;BSG;BSG;B	brown	-0.3058552	0.19251607	0.23240943	0.1158435	-0.4624974	-0.0406413
cg23523937	EDEM2;EDEM2;EDEM2;ED	brown	-0.0113678	-0.5177445	0.02354412	0.20650657	0.85142902	0.1093458

cg07069934	ISG20;ISG20;ISG20;ISG20;I	brown	0.01741588	0.54455656	0.11652202	-0.2170256	-0.8912999	0.02675901
cg10446869	KRTCAP2;TRIM46;TRIM46	brown	-0.1506336	-0.6886001	0.04915175	0.06279597	0.84011619	-0.016606
cg14508198	U2SURP;U2SURP;U2SURP	brown	0.00090155	-0.552182	-0.0555184	0.24328575	0.93575886	0.06762572
cg24619709	IRF5;IRF5;IRF5;IRF5;IRF5;I	brown	-0.1635224	-0.6874841	0.00046384	0.0831953	0.84244927	0.01781796
cg12726743	CAMK2G;CAMK2G;CAM	brown	-0.2720879	0.25298648	0.07578801	-0.1545347	-0.4661078	-0.1474811
cg14449059	RP11-50C13.2;LIPC	brown	-0.2023354	-0.656042	0.02109518	0.05520856	0.79968231	-0.022814
cg04878480	RP1-228P16.3;RP1-228P16.4	brown	0.11219609	-0.4761745	0.09145868	0.25001846	0.88996025	0.12575578
cg14869215	ANKRD32;ANKRD32;ANK	brown	0.0930937	-0.5047261	0.00905938	0.24288111	0.91713595	0.16199956
cg02622890	PRSS3;PRSS3;PRSS3	brown	-0.2322765	0.11307654	0.23933804	-0.0879317	-0.4261375	-0.1097786
cg17685731	POU2F1;POU2F1;POU2F1;	brown	0.06034902	-0.5555691	-0.022009	0.20903666	0.89533749	0.11099831
cg15155922	MEMO1P1	brown	0.09921672	-0.5048779	0.01467634	0.21390317	0.90291238	0.14872725
cg23494744	PHKG2;PHKG2;PHKG2;PH	brown	0.10134221	-0.5130326	0.06115414	0.25579802	0.91231948	0.13861147
cg04277040	RP11-18F14.1;RP11-	brown	-0.089901	0.36160402	0.22918969	0.01192934	-0.5905567	0.07044381
cg15447231	RP11-421L21.3;RP11-	brown	-0.0194145	-0.6249486	-0.0012117	0.22911409	0.91471532	0.09769389
cg14008271	RP11-81H14.2;RP11-	brown	-0.1272271	-0.4497953	0.00251921	0.1212306	0.75302024	-0.0309697
cg10713461	CTD-3195I5.5	brown	0.05595729	-0.5336803	-0.0252826	0.24280162	0.92875236	0.13202687
cg05716556	GNGT2;ABI3;GNGT2;GNG	brown	-0.1725268	0.3332196	0.26828719	0.09040981	-0.4166427	-0.077408
cg13599613	SH2D3C;SH2D3C;SH2D3C;	brown	0.0806199	-0.5116872	-0.0146771	0.22706771	0.91415767	0.15008634
cg18835672	LINC00494;RP1-	brown	-0.1945925	0.19821574	0.13410654	-0.0312121	-0.4458632	-0.0472436
cg22742001	GIT2;GIT2;GIT2;GIT2;GIT2;	brown	0.02922678	-0.5672598	-0.0335593	0.22334224	0.9222736	0.07089404
cg02796279	AC007879.3;AC007879.2;A	brown	0.06470267	-0.5112725	-0.0137256	0.1771763	0.91567497	0.13543807
cg24219822	CD244	brown	0.01377796	0.51508335	0.03000189	-0.2294282	-0.8522456	-0.0757505
cg01008102	RGS18;RGS18;RGS18;RGS	brown	0.15157051	-0.3618085	-0.2895791	0.08214381	0.68756897	-0.0534891
cg08293124	CD63;CD63;CD63;RP11-	brown	-0.0010311	-0.5010838	-0.0557338	0.2230841	0.88217914	-0.0173851
cg20835863	RP11-1070N10.3	brown	-0.1373373	-0.1621668	-0.1577572	-0.0002665	0.33846612	-0.291003
cg15114651	SLC1A5;SLC1A5	brown	-0.0929608	0.41281651	0.35879899	0.04995175	-0.3985963	0.07181372
cg00105570	RAG1;RAG1	brown	0.13246753	0.58860431	-0.0167356	-0.1507113	-0.8857266	0.05792159
cg05807690	CHST10;CHST10;CHST10;C	brown	0.02918901	-0.1863852	-0.4897148	-0.0564928	0.37723247	-0.2719104
cg22697477	RUNX1;RUNX1	brown	0.0787137	-0.4953121	-0.0208145	0.17134134	0.91560026	0.12657687
cg08827572	TRAF3IP3;TRAF3IP3;TRAF	brown	0.26362589	0.45376195	0.19138027	-0.0272509	-0.6128183	0.3402999
cg19931348	PI3	brown	0.10703503	-0.4817132	0.02150655	0.24823151	0.90209336	0.13628613
cg13948857	AC116366.5;C5orf56	brown	-0.0575007	-0.4644893	0.05022878	0.19899493	0.88454908	0.03375418
cg20535781	CHFR;CHFR;CHFR;CHFR;	brown	0.25077536	-0.0533307	-0.1083449	0.23818972	0.34009981	0.10275954
cg13481904	AC012442.5;CHCHD5;CHC	brown	0.13226593	-0.5205726	0.05010708	0.20679906	0.91614539	0.23769536
cg07358876	CAPN13;CAPN13	brown	-0.2322419	0.25120711	0.17088071	-0.1565732	-0.5138789	-0.0247855

cg11416918	VPS37C;VPS37C;VPS37C;	brown	0.09632278	-0.4971052	0.10053549	0.29980847	0.9139605	0.10291137
cg00405670	GPR126;GPR126;GPR126;G	brown	-0.1498521	-0.6361597	-0.0063645	0.12880599	0.86957866	-0.0489422
cg09287387	TNPO1;TNPO1;TNPO1;TNP	brown	0.14603188	-0.1131725	-0.4444831	0.12687665	0.43970208	-0.0706293
cg03219996	RP11-	brown	0.09635932	-0.4407776	-0.0031455	0.2483696	0.89235264	0.09766109
cg03508526	ZDHHC2	brown	-0.0947078	-0.2571826	-0.2178331	0.07208393	0.31328035	0.08109357
cg05246522	KSR1	brown	0.11199325	0.68062759	-0.0740212	-0.0155268	-0.8321639	-0.0306556
cg01306540	CDC42BPB	brown	0.08196085	-0.487799	0.01050122	0.23320173	0.9008183	0.11626248
cg24168413	FXYD1;FXYD1;LGI4	brown	0.05391435	-0.5154258	0.054555	0.2269098	0.92375592	0.09460355
cg24574692	AC104809.2	brown	0.0537516	-0.5176427	-0.0247292	0.22589992	0.90440402	0.03974801
cg12405599	RPN1;RPN1;RPN1;RPN1;R	brown	0.05116902	-0.5532387	-0.0075635	0.16678179	0.9244867	0.10038079
cg05767652	AL035610.2	brown	0.0733506	-0.500341	-0.0527231	0.23566901	0.90097231	0.1061516
cg24184014	AC109826.2;AC109826.1	brown	0.32969149	-0.1377421	-0.0811456	0.17549568	0.67440613	0.25989629
cg00872580	PFKFB3;PFKFB3;PFKFB3;	brown	0.13541219	0.22215028	0.12160363	-0.0765013	-0.4421917	0.57989204
cg11260662	TDP1;TDP1;TDP1	brown	0.09881003	-0.5126999	0.00107372	0.21555699	0.91389537	0.16458091
cg27072996	LPPR3;LPPR3;LPPR3;LPPR	brown	-0.2303015	-0.7654868	0.01444011	0.02350832	0.74055777	-0.0126759
cg14310212	AC156455.1;AC156455.1	brown	-0.3226472	0.07272181	0.19478555	-0.0020728	-0.3791176	-0.0526013
cg14158827	ACSL6;ACSL6	brown	0.02042799	0.5705274	0.13789806	-0.1535197	-0.8941969	-0.0161572
cg08824121	RP4-781K5.5;RP4-	brown	0.14879121	-0.2090904	-0.270651	0.14885066	0.55873496	-0.0462054
cg13901779	MYB;MYB	brown	-0.0551913	-0.661887	-0.0196335	0.1594422	0.88829054	0.10134488
cg04711454	RP11-	brown	-0.0679237	-0.5918184	0.11300816	0.21748755	0.85709288	-0.0448773
cg01543184	MAFG;MAFG;MAFG	brown	-0.0211836	-0.6219171	0.0410815	0.16755768	0.90067723	0.0242135
cg17110052	ETFB	brown	0.06150453	0.51147532	-0.0562182	0.11248879	-0.7630468	-0.0446012
cg26084614	RP11-446H18.5;RP11-	brown	-0.0308265	0.58106588	0.1214627	-0.16217	-0.8899397	-0.0681016
cg06291743	DLGAP1;DLGAP1;DLGAP	brown	0.06823835	-0.3590752	-0.051511	0.26172791	0.84343236	0.0692226
cg00410753	DPEP2;DPEP2;DPEP2;DPE	brown	-0.1329397	0.12211474	0.16447487	0.17355405	-0.4121174	0.06941427
cg07688618	U6	brown	-0.1020427	0.33626455	0.12470422	-0.1084415	-0.7470275	-0.0673321
cg01837846	KLHL29;KLHL29	brown	-0.0492141	0.4951165	0.08965728	-0.2394831	-0.8777397	-0.0654941
cg07841875	HEATR7A;HEATR7A;HEA	brown	0.12400265	0.52342269	0.21133866	-0.0653781	-0.7851253	0.17006159
cg25360761	CD22;CD22;CD22	brown	-0.1334113	-0.6757354	0.02933266	0.09975983	0.81678293	-0.0364193
cg17816488	TNIP3;TNIP3	brown	0.0889295	-0.4826445	0.03247523	0.28506738	0.90891524	0.10615839
cg20470583	FGD4;FGD4	brown	0.12443993	-0.4897942	-0.0049701	0.27035985	0.92001918	0.13692757
cg25146434	RP11-194G10.1	brown	0.11581742	-0.4583565	0.00752254	0.30259976	0.85048765	0.12173219
cg19476647	LTBR;LTBR;LTBR;LTBR;LT	brown	-0.111549	-0.6276851	-0.0474131	0.1597023	0.85233721	-0.1653808
cg19021885	snoU13	brown	0.08827258	0.52139745	-0.063237	-0.1920876	-0.7741715	-0.0042523
cg06836736	ME1;ME1;ME1	brown	-0.0151523	-0.4981648	0.03939474	0.04154465	0.57342432	0.11710315

cg04657382	RP11-699L21.1;RP11-	brown	-0.0671014	-0.5998482	0.11360809	0.18143487	0.83474523	0.1144458
cg13199620	SERPINF1;SERPINF1;SERP	brown	0.14526622	0.5550727	0.10653201	-0.163373	-0.7359784	0.11606787
cg07195330	RP11-284F21.8	brown	-0.2036618	0.11545515	0.28444322	-0.0930232	-0.285656	0.1284833
cg10250177	LCK;LCK;LCK;LCK;LCK;L	brown	-0.0007103	0.42700709	0.11076329	-0.1756118	-0.7703087	0.0959249
cg00376294	SPRED2;SPRED2	brown	0.10376068	-0.4991045	0.05223639	0.19945226	0.90600995	0.1435561
cg16619193	IKBKE;IKBKE;IKBKE;IKB	brown	-0.3831484	0.23112632	0.15947121	-0.1531859	-0.5755991	-0.1145451
cg07905847	NOL6;NOL6;NOL6	brown	-0.2611774	-0.3990522	0.060819	0.01354498	0.44773301	-0.1595273
cg14156592	BCAP29;BCAP29;BCAP29;	brown	0.06044179	-0.5311188	0.04485903	0.24021903	0.92190458	0.11674041
cg21648245	RPL34;RPL34;RPL34;RPL34	brown	-0.2379383	0.18330972	0.16833299	0.02590338	-0.3687696	-0.1768822
cg16009632	TNS4;TNS4	brown	-0.3037299	0.18107483	0.12798879	-0.1982373	-0.4941961	-0.1013554
cg07474825	KRI1;KRI1;KRI1;KRI1;KRI	brown	-0.111417	0.3644113	0.27995075	0.1807661	-0.4369118	0.01928702
cg21201401	LIME1;LIME1;LIME1;LIME	brown	0.07972405	0.49772924	0.0921417	-0.106721	-0.7828183	0.07293573
cg01650347	C3orf65;C3orf65;C3orf65;IG	brown	0.03636627	-0.533195	-0.0857622	0.14665031	0.92291869	0.06053585
cg13784559	PTPN18;PTPN18;PTPN18;P	brown	-0.0103474	0.52686806	0.13340012	-0.023896	-0.8808151	0.0110015
cg07841371	TTLL10;TTLL10;TTLL10-	brown	0.38489624	0.54152847	0.02682166	-0.1079372	-0.6163109	0.13925269
cg18689504	NOTCH4;NOTCH4;GPSM3	brown	-0.2741003	-0.7060548	-0.0319649	0.07110132	0.82464342	-0.1035566
cg18363008	TM4SF5;TM4SF5	brown	-0.2501495	0.2974308	0.3476286	0.05042317	-0.5093919	-0.1064485
cg26189818	AC093911.1	brown	0.08478978	-0.4589705	-0.0612871	0.1252353	0.89208728	0.08087082
cg20716932	COMMD7;COMMD7	brown	-0.2175	0.27483203	0.17169938	-0.0806725	-0.5679849	-0.0262066
cg07170708	GORASP2;GORASP2;GOR	brown	0.06716372	-0.4951939	-0.1153329	0.15762433	0.87141693	0.09952596
cg04236294	TARM1	brown	0.00728976	-0.504683	-0.008937	0.1045185	0.90148279	0.09578576
cg24647339	IL18R1;IL18R1;IL18R1;IL18	brown	0.09773626	-0.502672	0.02941874	0.25818456	0.91560978	0.09654847
cg13891009	ITGB1BP1;ITGB1BP1;ITGB	brown	-0.0274529	-0.407651	0.07942889	0.23191222	0.78941148	0.04677815
cg12733607	C10orf140;C10orf140	brown	-0.0079574	-0.3644856	-0.5180394	-0.1129478	0.44483825	-0.1196611
cg08891071	GPX3;GPX3;GPX3;GPX3;G	brown	-0.243362	-0.7766712	-0.0719458	0.02509892	0.81428794	-0.1512953
cg10426076	PRG2;PRG2;PRG2;PRG2;R	brown	0.10642015	-0.4823958	0.03618244	0.28654129	0.91371242	0.13118394
cg26192769	PSTPIP1;PSTPIP1;PSTPIP1;	brown	-0.118432	-0.4641846	0.12263845	0.18224722	0.73315359	0.03130225
cg22059755	UBN2;UBN2	brown	0.38106013	-0.0856617	0.00259426	0.26079623	0.63196544	0.13402482
cg17250929	S100A5;S100A5	brown	0.14915259	0.57826822	-0.0892217	-0.0980924	-0.7420997	-0.0176891
cg20547005	RPS15A;RPS15A;RPS15A	brown	0.11298252	0.67073501	0.12319553	-0.0246467	-0.7412697	0.1532228
cg26809643	ZNF680;ZNF680;ZNF680;Z	brown	-0.2385217	-0.3240199	-0.4270509	-0.0144497	0.34349425	-0.3458503
cg27027427	IFIT3;IFIT3;LIPA;LIPA;LIP	brown	-0.045574	0.25764338	0.10440094	0.04809896	-0.354744	-0.087532
cg21305096	CTD-2126E3.1	brown	0.12949605	0.27203918	-0.0469346	0.05571459	-0.2645634	0.04732869
cg09096833	SMOX;SMOX;SMOX;SMO	brown	0.11158463	0.48958628	0.26911804	-0.1107845	-0.8047928	0.1029315
cg20388732	STAT5A;STAT5A;STAT5A;	brown	-0.1197151	-0.2349456	-0.2790671	-0.0455288	0.37607536	0.08512558

cg24615189	KCNIP1;KCNIP1	brown	-0.2323537	-0.6278822	-0.0074233	0.07652096	0.80970443	-0.0983943
cg11009736	MARCO;MARCO;MARCO;	brown	-0.0639377	-0.4227868	0.04244156	0.05556923	0.84749201	0.04532957
cg10973881	TMOD4;TMOD4;TMOD4;T	brown	-0.1100422	-0.6324544	-0.0106064	0.14149181	0.84159605	0.05741286
cg08941317	RP11-727A23.5;RP11-	brown	-0.0830963	-0.4807727	0.17320317	0.15958223	0.81029821	0.0727121
cg08310116	NEK9;NEK9;NEK9;NEK9;N	brown	-0.0331872	-0.4852568	0.0244367	0.18371789	0.86184015	0.06985641
cg22761077	ELANE	brown	-0.1991573	-0.6029096	-0.0157429	-0.0101276	0.84214486	-0.098041
cg18553399	LA16c-	brown	-0.1556956	0.18212535	0.01338029	-0.1073079	-0.4267946	-0.1026402
cg14557699	PCDHA12	brown	-0.0517965	-0.3614306	0.14203762	-0.1196779	0.37528986	0.0747107
cg09781514	PRKD3;PRKD3;PRKD3	brown	0.09716787	-0.5199468	0.03855841	0.18071799	0.91418334	0.11478319
cg06922393	RP11-45F15.2	brown	-0.0035828	0.65057364	0.08892957	-0.1180798	-0.7423419	-0.0728543
cg27478579	MTIF2;MTIF2;MTIF2;MTIF	brown	0.02978159	0.21997161	-0.0591903	0.12706848	-0.2640903	0.07080891
cg11612617	IGF1	brown	-0.1871055	0.204848	0.27589984	-0.0578032	-0.468156	-0.0017546
cg18854765	LRRIQ3;FPGT;FPGT-	brown	0.04678466	-0.6059288	-0.046153	0.16531027	0.87822145	0.12247593
cg14784845	MEF2C;MEF2C;MEF2C;ME	brown	0.13700713	-0.4420387	0.00552237	0.19181171	0.863579	0.07901409
cg10452229	PCM1;PCM1	brown	0.14146506	-0.4009266	0.03672044	0.32160085	0.88767126	0.08857537
cg26976492	U1	brown	0.03325989	-0.4985622	-0.0142868	0.16478437	0.92391972	0.07199029
cg04748516	AP003064.1;AHNAK	brown	0.09074494	0.61550335	0.04905434	0.02492216	-0.6653869	0.04132291
cg21187669	ATPAF2;ATPAF2;ATPAF2;	brown	0.1605373	0.10689155	0.01790421	0.0006609	0.29915438	0.17602581
cg05827190	MFSD7;MFSD7;MFSD7	brown	0.04598852	-0.2473128	0.11061875	0.0959909	0.5174672	0.00095798
cg01881664	COPS3;COPS3;COPS3;COP	brown	0.01322275	-0.5250574	-0.0553782	0.26126182	0.90157281	0.10781039
cg02098075	EFCAB1;EFCAB1;EFCAB1;	brown	0.0822242	-0.4936669	-0.0289963	0.21767897	0.90160994	0.10871791
cg04864877	AC083900.1	brown	0.15698055	-0.447426	-0.0699233	0.11670908	0.89382576	0.15096154
cg22845525	MYO1A	brown	0.1588084	0.39670783	-0.1872505	0.19382691	-0.4732339	0.07411539
cg03237401	MIR1278;CDC73;CDC73	brown	0.09695772	-0.3225688	0.09874281	0.18794489	0.66613483	-0.0058463
cg03217204	ARG1;ARG1;ARG1;ARG1;	brown	0.10010288	-0.4800635	-0.0617421	0.14945288	0.90421315	0.1206275
cg10418276	ANKRD12	brown	0.06650447	-0.5019432	-0.0272438	0.19104143	0.90657194	0.06387785
cg12521157	FCHSD2;FCHSD2;FCHSD2	brown	0.30215365	-0.2523535	0.20544405	0.29747852	0.64258377	0.19279858
cg24313724	TKT;TKT;TKT;TKT;TKT;TK	brown	0.39282627	-0.1181967	0.26040097	0.23609575	0.58437687	0.28566763
cg13811379	RP11-161M6.3	brown	0.34676633	0.39810251	0.18617329	0.06282656	-0.3571149	0.21183532
cg12232470	BCL9L;BCL9L;BCL9L;BCL9	brown	-0.1085048	0.28317337	0.31109591	0.01870712	-0.5126016	0.06312116
cg18234296	SERTAD4;C1orf133;C1orf13	brown	0.19219501	0.57188026	-0.0123045	-0.1353373	-0.7467316	0.0885538
cg12911648	KRT17;KRT17;KRT17;KRT	brown	0.07501425	-0.5054268	0.09876453	0.15537681	0.9133301	0.06852871
cg04752257	TOMM20L;TOMM20L;RP1	brown	0.05158786	0.36945726	0.11893916	0.09784396	-0.3384891	0.09575311
cg21750689	IGF1;IGF1;IGF1;IGF1;IGF1	brown	0.13806314	-0.2760461	-0.3763118	0.04713144	0.49390577	0.18007168
cg09895920	CREB3L4;SLC39A1;CREB3	brown	-0.0842183	-0.5965992	0.04573584	0.17853768	0.86082848	-0.0436492

cg22806068	RBBP8;RBBP8;RBBP8	brown	-0.0915928	-0.5822139	-0.0635545	0.01449569	0.59229666	-0.0463947
cg16705546	IRF8;IRF8;IRF8	brown	-0.090446	0.16073654	0.20705653	-0.1583479	-0.428194	0.05706562
cg23875059	PRR5	brown	0.01822061	0.5832049	0.15250109	-0.1096518	-0.8068645	-0.0152007
cg20807852	ANKRD40;ANKRD40	brown	-0.2133035	-0.5320069	0.03333957	0.08310323	0.75865499	-0.0308588
cg00157962	FAM102B;FAM102B	brown	0.03045083	0.21280996	-0.0570261	0.175097	-0.3897724	0.07396065
cg13928634	NLRC4;NLRC4;NLRC4	brown	0.38233	-0.2037064	-0.1851628	0.18491224	0.70011721	0.16301277
cg27206867	WDR1;WDR1;WDR1;WDR1	brown	0.02999345	0.09981318	0.14867987	0.10203275	-0.3636763	0.08428223
cg22076474	BACH1;BACH1;BACH1;BA	brown	0.02284852	-0.5652406	-0.005244	0.21613656	0.91368759	0.07323656
cg05480350	MIR1205;PVT1;PVT1;PVT1	brown	0.21770428	0.36179764	0.25681369	0.47014391	-0.3513886	0.32132055
cg25664606	PACSIN2;PACSIN2;PACSI	brown	0.10266721	-0.5011903	0.08509896	0.2295519	0.89590557	0.13705468
cg18771857	MATR3;MATR3;MATR3;M	brown	-0.2479211	-0.4182799	-0.0518616	0.02392175	0.46823087	-0.3001264
cg10592592	DDIT4;DDIT4	brown	-0.3163103	0.08844169	0.14650516	-0.1984047	-0.4858347	-0.0867309
cg19429405	C10orf105;CDH23	brown	0.01190638	-0.5736208	-0.0749052	0.17427085	0.90725361	0.0609636
cg04321197	IL10;IL10;IL10	brown	-0.0382335	-0.5750488	0.04611595	0.19519601	0.88746661	0.06962541
cg25341726	IL27;IL27	brown	0.09865453	-0.418375	0.11891837	0.19357201	0.8748371	0.21501611
cg14289658	NUDC;NUDC	brown	0.00957516	0.10710632	-0.0486962	-0.0162408	-0.2063354	-0.0040063
cg01771416	NFKBIZ;NFKBIZ;NFKBIZ;	brown	0.25588004	-0.1276607	-0.190509	0.29775375	0.62766105	0.02334138
cg23105068	C3orf37;C3orf37;C3orf37;C3	brown	0.21206392	0.46874343	-0.071789	-0.0787672	-0.6153998	0.06136227
cg19438764	ABCA9;ABCA9;ABCA9	brown	0.06402855	-0.5021205	-0.1117462	0.09082325	0.91308054	0.1056585
cg25082710	IVL;IVL	brown	0.01209306	0.362506	0.16127993	0.02122407	-0.4639384	0.04012717
cg16053299	ITGAL;ITGAL;RP11-	brown	0.14868415	-0.4292397	0.06393259	0.36058219	0.90519948	0.11425069
cg16609995	PBX2;PBX2;GPSM3;GPSM	brown	-0.0237625	0.48126586	0.20738206	-0.0216303	-0.7510045	-0.0103906
cg23014216	PPCDC;PPCDC;PPCDC;PP	brown	-0.4289267	-0.4194898	-0.2032026	-0.0796321	0.32976829	-0.6100354
cg04447288	CPLX1;CPLX1	brown	-0.349002	0.05232592	0.21531393	-0.0752246	-0.3789019	-0.1012311
cg01782486	ZBTB7B;ZBTB7B;ZBTB7B;	brown	0.0910662	0.35246125	0.14816566	-0.0785457	-0.7006594	0.1088064
cg01248273	MARCH7;MARCH7	brown	0.11592864	-0.4975466	0.13870779	0.22664582	0.8907794	0.16668721
cg05759148	HIST1H2AJ	brown	0.03215732	0.24695593	0.01554532	-0.1039724	-0.2757746	0.04351991
cg20979153	ZNF217;ZNF217;ZNF217;Z	brown	-0.0471915	-0.191533	-0.0234666	-0.0927297	0.42582986	-0.0366541
cg18019929	D2HGDH;D2HGDH;D2HG	brown	-0.126487	-0.4749724	0.17568684	0.19611311	0.80539273	0.04613491
cg19040264	CLIP1;CLIP1;CLIP1;CLIP1	brown	0.07648643	-0.496593	-0.0447117	0.23273208	0.9047583	0.0756806
cg09423126	RP11-87E22.2;RP11-	brown	-0.1694784	0.16063813	0.17037616	-0.0518789	-0.3636593	-0.0982981
cg21510967	AC109826.2	brown	-0.1633484	0.15074604	0.02875616	-0.1626557	-0.3345693	-0.1496377
cg06127112	SYT17	brown	-0.1049203	0.0768232	0.21219603	-0.1336303	-0.251228	0.09157903
cg19271162	GZMA	brown	0.06153952	0.32019523	0.19822171	0.02188642	-0.3806368	0.03865476
cg17742642	BAI2;BAI2;BAI2;BAI2	brown	0.27310059	0.70782604	0.17991728	0.00893606	-0.7486712	0.16356589

cg00732810	RASA3-IT1	brown	0.37578421	0.60371887	0.18154486	0.05285942	-0.6266504	0.33199332
cg03638686	RORC	brown	-0.2353072	0.27329166	0.24783954	-0.1744563	-0.6460919	-0.0592666
cg08804892	TRAK1;TRAK1;TRAK1;TR	brown	-0.0320619	0.08140761	0.22670872	0.0578166	-0.291111	0.07457288
cg25310780	STAMBP	brown	0.14125908	-0.4988868	0.04559554	0.13382177	0.83099392	0.18219149
cg16232289	KIAA1109;KIAA1109;KIA	brown	0.24322513	-0.1671095	0.2637719	0.29087505	0.64596443	0.13959918
cg03751527	GHRL;GHRL;GHRL;GHRL;	brown	0.10663898	-0.4401228	0.06538197	0.26662676	0.8810061	0.08255538
cg13441156	GHRL;GHRL;GHRL;GHRL;	brown	0.15280261	-0.4654504	0.06143774	0.2148539	0.89981815	0.09885449
cg24058132	GALC;GALC;GALC;GALC;	brown	-0.0910891	0.43376292	0.23467494	0.02567287	-0.5534423	0.02102853
cg22784697	AL162502.1;LCOR;LCOR;L	brown	0.1704624	0.63779241	0.09570105	-0.0839401	-0.7679788	0.07619589
cg01574571	IL12RB1	brown	-0.17819	0.32229392	0.07531123	-0.2309345	-0.5122784	-0.0884976
cg27246361	IGLV3-9	brown	-0.1379645	0.44087139	0.06456998	-0.2822638	-0.7992998	-0.1693012
cg08001909	AC009506.1;BAZ2B;BAZ2B	brown	0.06067887	-0.5692251	-0.1311957	0.1282226	0.8531443	0.07293841
cg16294143	NAPRT1;RP11-	brown	-0.0656134	-0.363092	-0.0588707	0.05610944	0.55531053	-0.0969802
cg04094865	USP31	brown	0.12893821	0.61039341	-0.0502992	0.11148878	-0.6289843	0.01441947
cg07641746	AL023096.1;RUNX3	brown	-0.0131361	0.52319789	0.24186796	0.12731591	-0.5249945	-0.0048016
cg26429850	CORO7;CORO7;CORO7;CO	brown	-0.091576	0.48546025	0.22831817	-0.1750548	-0.8573241	-0.0469063
cg00615241	PRTN3	brown	0.00584605	-0.489025	0.09245496	0.05440709	0.83245552	0.09273218
cg00874230	TESC;TESC;TESC;TESC;TE	brown	-0.3507101	0.07718184	0.04265539	-0.2081998	-0.4106064	-0.2070843
cg21920303	SLAMF1;SLAMF1;SLAMF	brown	-0.0302377	0.56261932	0.02458762	-0.0585485	-0.8048547	-0.0235184
cg13651137	DBH-AS1;DBH	brown	0.11938242	0.43732166	0.19410419	0.09385953	-0.4158891	0.21092163
cg22489074	SLC35E3;SLC35E3;SLC35E	brown	0.08076719	0.35285329	0.23956336	0.00175952	-0.6309946	0.0247907
cg02901136	S100A12;S100A12	brown	0.08456674	-0.4673441	-0.1045935	0.15324651	0.89094538	0.0867598
cg10696060	GPR56;GPR56;GPR56;GPR	brown	-0.0564071	0.10666787	0.11012304	-0.0748871	-0.3961399	0.01843564
cg08719380	CACNB4;CACNB4;CACNB	brown	0.20447333	0.43905091	0.0506514	0.04180727	-0.4345322	-0.0301589
cg14680792	USP4;USP4	brown	0.11106457	-0.4808824	0.23482048	0.24885469	0.8417948	0.17616958
cg08335107	AC011286.1	brown	0.0836056	0.52468302	0.07924566	0.10742601	-0.4937709	0.06338218
cg26366091	CHI3L2;CHI3L2;CHI3L2;CH	brown	-0.083963	-0.5334907	0.04785268	-0.0741783	0.81757085	0.04432384
cg15164958	VSTM2A;VSTM2A;VSTM2	brown	-0.2764463	-0.4558819	-0.3958107	-0.0842452	0.37296046	-0.2284827
cg25486322	Y_RNA;AC019221.4	brown	0.13095402	0.47759302	-0.1609611	-0.0958558	-0.6673671	0.00160156
cg08467128	GPR84;GPR84;RP11-	brown	0.37888248	-0.0970602	-0.1136776	0.32339919	0.65754432	0.16912919
cg09281528	RP11-	brown	0.10219039	0.5421533	-0.0124471	-0.1163664	-0.7413759	0.02604855
cg04738877	GALC;GALC;GALC;GALC;	brown	0.19395828	0.72497549	-0.0848579	0.1852217	-0.7131249	-0.0819591
cg10073706	NPEPL1;NPEPL1;NPEPL1;S	brown	-0.2386329	-0.546535	-0.1657302	-0.0631469	0.49377135	-0.2679186
cg00944421	ESRP2;ESRP2;ESRP2;ESRP	brown	0.37668084	0.01487073	0.05852831	0.29663841	0.28488905	0.27867537
cg01366692	SCYL1;SCYL1;SCYL1;SCY	brown	0.08735319	0.09681022	0.39413313	0.16960429	-0.29321	0.18334006

cg21212591	TMEM212-	brown	0.10143438	-0.4538909	-0.0894418	0.14459626	0.88814433	0.08699818
cg11984636	MBP;MBP;MBP;MBP	brown	0.03071972	-0.5096432	-0.0191285	0.15148147	0.83728433	-0.0131947
cg01276321	AIM1;AIM1;AIM1;AIM1	brown	0.10317965	-0.5028665	0.07605486	0.15633696	0.90710324	0.11877678
cg21818059	ANAPC5;RNF34;RNF34;RN	brown	-0.1115276	0.26733923	0.18100822	0.05958516	-0.4104223	-0.0505041
cg19176897	LGALS8;LGALS8;LGALS8;	brown	0.30760924	0.08274304	0.06856475	0.34236355	0.25955105	0.19558495
cg12009710	Y_RNA;C7orf23	brown	-0.0976943	0.4536008	0.05972025	-0.1508873	-0.8040799	-0.0963316
cg08550353	SGK1;SGK1;SGK1;SGK1;S	brown	0.00663591	-0.6052565	0.03424571	0.17413553	0.83681409	0.02239957
cg26101277	CCL1	brown	-0.0719248	-0.3930604	0.08450972	0.2405055	0.81345594	0.05656631
cg14265321	AMICA1;AMICA1;AMICA	brown	0.02054648	0.62244074	0.10607077	-0.1404149	-0.794473	-0.041141
cg13849515	TRIM25;TRIM25	brown	-0.1099469	0.22663955	0.1480032	0.04568377	-0.3600914	0.10518106
cg21070941	DNAJC14;RP11-762I7.5	brown	-0.0166846	0.52741514	0.30097699	0.00530176	-0.587606	0.06132212
cg25218152	RP11-744N12.3;FLI1;RP11-	brown	0.18104383	-0.439354	0.06164073	0.26081324	0.8845921	0.14291717
cg27118196	CTNNBIP1;CTNNBIP1;CTN	brown	0.3147817	-0.2147436	0.20690121	0.20384435	0.65369773	0.27803616
cg01701207	SF3B3;SF3B3;SF3B3	brown	0.15215781	-0.4583078	0.13595785	0.15545203	0.87324487	0.18589954
cg13549082	METTL11A	brown	-0.1514412	-0.6316099	-0.1769981	0.05242945	0.81274617	-0.1990599
cg22145857	SNORA24	brown	0.10024009	0.50400142	0.03985965	-0.0281988	-0.7014065	0.04836763
cg10821511	GNGT2;GNGT2;GNGT2;G	brown	0.0244023	0.47821801	0.32184795	-0.0223113	-0.7904037	0.12257402
cg13282195	NAPRT1;NAPRT1;NAPRT1	brown	-0.0341427	-0.3801945	-0.0033415	0.1080868	0.59817275	0.01687679
cg12410124	AC124944.5;AC124944.5	brown	0.03850774	-0.4747419	-0.0336183	0.23901799	0.91189069	0.09950546
cg18759728	PGM1;PGM1;PGM1;PGM1;	brown	-0.1963463	-0.3747168	0.01454784	0.0098067	0.41344151	-0.2067747
cg21836919	RP11-164O23.7	brown	0.17502796	-0.3338208	0.08383313	0.33289674	0.86296333	0.14934349
cg08119452	OLFM4;OLFM4	brown	0.10908649	-0.2754826	-0.0029752	0.13188526	0.79060193	0.13788017
cg21667061	NARG2;NARG2;NARG2;N	brown	0.18344037	0.304269	0.28803173	0.19945496	-0.3134516	0.08496798
cg09456782	DCUN1D2;DCUN1D2;DCU	brown	-0.2221088	-0.722446	-0.005732	0.05654568	0.80723444	-0.093237
cg20572153	GAS7;GAS7	brown	-0.0922271	0.39110164	0.37504634	0.03078065	-0.5788497	0.11300807
cg11563400	RP11-	brown	0.12150078	0.4628986	0.20593621	0.10227695	-0.3920513	0.11578747
cg08192639	RN7SKP9;UBAC2;UBAC2	brown	-0.0994594	0.44551218	0.25785788	-0.1839605	-0.8084397	-0.0192286
cg14482811	LCN8;LCN8;LCN8;LCN8	brown	0.43015763	0.54878333	0.06521206	0.03486705	-0.5356236	0.42742584
cg25399158	INSRR;NTRK1;NTRK1	brown	-0.1584435	-0.6252603	0.00117966	0.08883604	0.6191756	-0.0434023
cg04072563	RP3-523C21.1	brown	0.2895168	0.62308239	-0.0077043	-0.0519702	-0.6133584	0.10132282
cg11874426	BCL9L;BCL9L;BCL9L;BCL9	brown	0.07728772	0.53874188	0.24659233	-0.0947211	-0.8032356	0.07360453
cg22851870	ZNF740;ZNF740;ZNF740;C	brown	-0.2361571	0.21997589	0.10622185	-0.0311763	-0.5071605	-0.2007333
cg02837301	DOK2;DOK2;DOK2;DOK2	brown	-0.2897762	0.1402451	0.21561834	-0.0341276	-0.4920825	-0.0647022
cg27254852	INPP5K;INPP5K;INPP5K;I	brown	0.07739067	-0.5108534	0.08836824	0.24356552	0.8908913	0.16001772
cg22629727	RP11-203B7.1	brown	0.11498025	-0.4030198	-0.0268024	0.26106982	0.89853564	0.1227712

cg20996897	LARP1;LARP1	brown	0.11941192	-0.4679811	0.0658459	0.30827621	0.89873966	0.17751892
cg11460824	AC073130.3;AC073130.3;TE	brown	0.03300196	-0.6134027	0.00697078	0.16209005	0.84976746	0.10421494
cg02434267	TRABD;TRABD;TRABD;T	brown	-0.1123244	0.34889785	0.16122603	-0.1645202	-0.8027199	0.03705644
cg24156826	RP11-452L6.1	brown	0.08835054	-0.2620339	-0.2538833	0.18275029	0.6475609	-0.3471381
cg22887000	VSTM1;VSTM1;VSTM1	brown	0.08902082	-0.2778979	0.11504561	0.29572485	0.5896742	0.03799906
cg21475150	RPL31;RPL31;RPL31;RPL31	brown	0.40407799	-0.0803553	-0.1475984	0.23199794	0.36223734	0.24788812
cg04412883	RP11-	brown	0.00149507	0.23273261	0.10759572	0.04612394	-0.3162299	0.07871416
cg14901029	GHRHR;GHRHR;GHRHR;	brown	0.10456728	0.57933635	0.27701769	0.10334194	-0.5251924	0.17461843
cg09154591	VIT;VIT;VIT;VIT;VIT;VIT;	brown	0.06208728	-0.2044468	-0.0190959	0.03573962	0.57703002	0.00871641
cg08362785	MKL1;MKL1	brown	0.18459253	0.29337654	0.12793154	-0.118036	-0.665297	0.11782172
cg19428589	RP11-161M6.3	brown	0.24452577	0.40315061	0.24351898	-0.041245	-0.4742698	0.23007806
cg16062877	KSR1	brown	0.10919742	0.42496385	0.53167575	0.29040739	-0.3992168	0.28091687
cg17847724	PLCB1	brown	0.11420748	-0.4594406	0.03564154	0.20047309	0.89523471	0.10733321
cg24100560	SSBP3;SSBP3;SSBP3;SSBP	brown	-0.0005957	0.37214585	0.27330219	0.31638405	-0.4825939	0.11959939
cg15740575	AC104650.2;SH3TC1;SH3T	brown	-0.0756098	-0.562743	-0.0356752	0.16000844	0.85113079	-0.1468856
cg09090006	CTD-	brown	-0.0200157	0.46689689	0.06419706	-0.1826569	-0.7796529	-0.0320615
cg18858676	OR10G8	brown	0.29763087	-0.1566709	0.03627475	0.27728801	0.62976942	0.12903116
cg18098722	RP11-496I9.1;C11orf35	brown	0.35831477	0.0053369	0.01406694	0.23138246	0.29727322	0.32574321
cg12816748	PRKAR1B;PRKAR1B;PRK	brown	-0.0767423	-0.5902124	0.08864925	0.09918052	0.81233525	0.07963599
cg10716641	RP11-700H13.1;RP11-	brown	0.13584501	-0.3896427	0.03157805	0.26734347	0.82358406	0.17936638
cg08905487	LAIR2;LAIR2	brown	0.10400112	-0.4531278	0.0310486	0.20072867	0.86627883	0.1895198
cg10471548	RIOK1;RIOK1;RIOK1;RIO	brown	0.10202315	-0.4722804	-0.0147972	0.15287361	0.90731713	0.11372602
cg11294761	CD7	brown	-0.2758498	0.23452596	0.24866859	-0.1977845	-0.6043555	-0.0130531
cg17989259	DUSP16	brown	0.14905527	-0.4329231	0.22840152	0.13623352	0.79618352	0.1467531
cg16948489	LAX1;LAX1	brown	0.02568979	0.46742444	0.20269942	0.05564297	-0.7600362	0.07717167
cg12727605	DAXX;DAXX;DAXX;DAX	brown	0.05611417	-0.4603685	-0.409558	-0.0439299	0.56092247	-0.0302705
cg16015622	C3orf37;C3orf37;C3orf37;C3	brown	0.19167602	0.2197734	0.07627467	-0.0061824	-0.3615528	0.29959659
cg20833652	RP11-672A2.3	brown	0.30677172	0.41384713	-0.1514207	0.01632679	-0.4579553	0.13660102
cg07631828	CTSZ;CTSZ;CTSZ;CTSZ	brown	-0.2774755	-0.6634068	-0.2340503	-0.0909097	0.56610422	-0.3153509
cg13841742	OCIAD2;OCIAD2;OCIAD2;	brown	0.14197528	0.66258042	0.19838768	0.01391939	-0.765676	0.03195248
cg07306426	SYS1;SYS1	brown	0.073377	-0.3317193	-0.0876637	0.15429441	0.83778432	0.05923662
cg08279189	TRIM27;TRIM27	brown	0.09769776	-0.5127504	0.11003435	0.27942594	0.91767684	0.10779982
cg07763337	LCN8;LCN8;LCN8;LCN8;L	brown	0.12145511	0.33558582	0.32420799	0.13886085	-0.3493892	0.22253737
cg05768044	CHD9;CHD9;CHD9;CHD9	brown	0.12001758	-0.5186724	-0.0454258	0.17937387	0.85670274	0.1806313
cg26882827	RP11-398A8.3;RP11-	brown	-0.2203304	-0.7203089	-0.0521985	0.04721314	0.78200887	-0.1697127

cg19028179	AL591428.1	brown	0.13682368	0.45961841	0.15572498	0.17983581	-0.3976468	0.06684139
cg09607047	MIR365A;RP11-	brown	0.13411744	0.13527979	0.10604462	0.27769657	-0.221303	0.0837204
cg06196145	PNPLA2	brown	0.03027304	-0.424362	0.04877237	0.12911525	0.82524926	0.05038106
cg02243522	MIR1207;PVT1;PVT1;PVT1;	brown	0.09241792	0.22444145	0.15699512	0.04079235	-0.2912629	0.09988625
cg17126963	CPEB4	brown	-0.0759527	0.42087226	0.25274338	0.09440079	-0.546938	-0.0244687
cg02894251	RP11-982M15.8	brown	-2.53E-06	0.31636934	0.15028655	-0.0138036	-0.4202086	0.12410408
cg00570498	CTC-	brown	-0.0086698	0.38885275	0.16008381	0.06135927	-0.5772206	0.09580393
cg23725139	CYTH1;CYTH1;CYTH1	brown	0.11622598	0.24899122	0.12773224	0.01599403	-0.2902245	0.16549386
cg20403541	RGS12;RGS12;RGS12;RGS	brown	-0.1386899	-0.6307244	-0.0246819	0.07287772	0.79584848	-0.1623912
cg14127336	TCL1A;TCL1A;TCL1A;TCL	brown	0.10504724	0.30754235	0.20180297	-0.0779704	-0.3953583	0.10855338
cg06814966	RAB2A;RAB2A;RAB2A;RA	brown	0.26674253	-0.23756	-0.2215031	0.12511036	0.69215079	0.05251022
cg12608507	NDUFS1;EEF1B2;NDUFS1;	brown	0.05432264	0.56830732	-0.0115267	0.03399241	-0.6169693	0.00720881
cg12564406	RP11-	brown	-0.1784659	0.36517765	0.19342781	0.08858652	-0.5806038	-0.1433172
cg04619509	RP11-356I2.1	brown	0.37642802	-0.162042	-0.1046427	0.28750512	0.71227805	0.09301542
cg22902340	ITGB2;ITGB2;ITGB2;ITGB2	brown	-0.2691839	-0.0072101	0.16105235	-0.0731166	-0.3438751	-0.0584966
cg18717554	STRA6;RP11-60L3.1;RP11-	brown	0.30521801	0.45783312	0.25406891	0.04482015	-0.4403469	0.21172571
cg24292665	MYEOV;MYEOV	brown	-0.2905093	-0.342143	-0.1857196	-0.0277654	0.43984377	-0.4863476
cg19866584	AC008592.5;AC008592.5	brown	0.34716152	-0.1908989	-0.0922165	0.34640101	0.72967304	0.13214529
cg14080566	KNCN;KNCN	brown	0.06069288	0.5418363	0.3078819	-0.0300503	-0.743457	0.13078969
cg02597092	RP11-16E12.2;RP11-16E12.2	brown	0.13159525	0.64057244	0.06446348	-0.0828006	-0.7239886	0.08023695
cg18712352	ZBTB25;ZBTB25;ZBTB1;ZB	brown	-0.131854	0.22294571	0.20618863	-0.0059364	-0.4869277	0.04353276
cg02378355	FAM120A	brown	0.27150262	-0.2725937	-0.1375902	0.23010835	0.75268216	0.03450614
cg11683482	DMAP1;DMAP1;DMAP1;D	brown	-0.0205497	0.47350432	0.29749939	0.37998462	-0.5318172	0.10942905
cg06386920	FXYD1;LGI4	brown	0.03946929	0.44322534	0.39626712	0.19266903	-0.3998695	0.21634106
cg11494508	HEPACAM2;HEPACAM2	brown	0.04164691	-0.4967168	-0.2075225	0.17305621	0.84224826	0.04221182
cg26180383	MIR1207;PVT1;PVT1;PVT1;	brown	-0.1094505	0.28516751	0.05931209	-0.1084554	-0.6536032	-0.128122
cg01935086	DCDC2B;DCDC2B;IQCC	brown	0.07019034	0.35215646	-0.0560753	-0.1729933	-0.6201939	0.02025311
cg08390169	IGHV6-1	brown	0.11402739	0.65632478	0.06304035	-0.0139576	-0.7163012	0.06636443
cg16860308	MFSD1;MFSD1;MFSD1;M	brown	0.09372016	-0.4892007	-0.0083133	0.27096839	0.89674923	0.09238678
cg19225308	TBXA2R	brown	-0.1612183	-0.6813134	-0.1281169	0.07662164	0.80045075	-0.0109887
cg10597669	RP11-7M8.2;HPD	brown	-0.0257575	0.50495892	0.10151886	-0.0574058	-0.6909426	-0.0630546
cg13846195	U6	brown	0.08639272	-0.4891428	0.11079536	0.17698904	0.88632064	0.10007778
cg25651219	ASCC1	brown	0.09892235	0.21329642	0.11760068	-0.0256603	-0.4850299	0.06067634
cg06710577	CEACAM21;AC005626.1;C	brown	-0.2872899	-0.4361372	0.00201326	0.0384412	0.396413	-0.5669781
cg26388730	NRXN2;NRXN2	brown	0.23887426	0.29942969	0.33083979	0.10148428	-0.3507685	0.25251598

cg22496986	CAMKK1;CAMKK1;CAM	brown	0.15001476	0.58256428	-0.0553352	-0.0411498	-0.6740614	0.01590226
cg11300443	NSMCE1;NSMCE1;NSMCE	brown	-0.0982816	0.43161344	0.05118474	-0.1775176	-0.8166321	-0.0197122
cg15838394	PLEKHB1;PLEKHB1;PLEK	brown	-0.0876084	0.4219097	0.19602958	-0.0566651	-0.7170033	-0.0310063
cg04354393	SLFN13;SLFN13;SLFN13;S	brown	-0.0422128	-0.6305922	0.01592126	0.15064255	0.82393864	-0.0177292
cg27383124	RP11-	brown	0.09289346	0.42279518	0.22014595	0.08080698	-0.4158682	0.49109402
cg09970967	PSEN1;PSEN1;PSEN1;PSE	brown	0.23389465	0.25082507	0.36517944	0.11253047	-0.2503214	0.32465067
cg09442240	CIB4	brown	-0.0475175	0.54376097	0.23795871	0.01938191	-0.5743225	8.27E-05
cg06585203	EZR-AS1;EZR	brown	-0.1336715	0.20845701	0.17864028	0.12622605	-0.3870747	0.00087565
cg13149833	EHD3;CAPN14	brown	0.12232398	-0.4330381	-0.1011763	0.26157848	0.85722058	-0.018899
cg16074988	CCND3;CCND3;CCND3;CC	brown	0.27330577	0.43735298	-0.023722	0.06111043	-0.4774888	0.12945424
cg14661234	TNFRSF4;TNFRSF4	brown	-0.0286966	0.19159461	0.11528233	-0.0111576	-0.2765083	-0.0319114
cg19623226	RP13-631K18.5	brown	0.07032997	0.60564408	0.08947341	-0.0558745	-0.6808751	0.07132306
cg10269078	RORC	brown	-0.1183189	0.30208291	0.1871101	-0.0684247	-0.5570511	-0.0122235
cg21251018	ZKSCAN4;NKAPL	brown	0.20887746	0.10321687	-0.2205989	0.15663148	0.13568278	0.15490889
cg14042308	RP11-	brown	0.05484048	0.5719761	0.22623943	-0.0483722	-0.7684054	0.10790516
cg25790531	RP1-124C6.1	brown	0.1563491	-0.4481885	-0.0223441	0.14993933	0.89143308	0.17167666
cg08646654	IFNA14	brown	0.08762748	-0.3816319	-0.1243888	0.18341945	0.83453698	0.08707428
cg08053370	RP11-429J17.8;RP11-	brown	0.08811721	-0.3400584	0.09674271	0.14608723	0.81066627	0.0809884
cg24855514	RP11-443B7.1;RP11-	brown	-0.0219252	0.47700636	0.14844368	0.35123125	-0.5588593	0.09892458
cg15572575	Y_RNA;SEPT9	brown	-0.1510181	0.41613568	0.10871658	0.00298046	-0.7647868	-0.1034715
cg24758110	ITPR1;ITPR1;ITPR1;ITPR1;I	brown	0.07845502	-0.4611633	0.02726113	0.1651173	0.80382187	0.16019528
cg19996272	TRIM59;TRIM59;TRIM59;T	brown	-0.1132035	0.25772205	0.20625218	-0.0461918	-0.4348126	0.0223317
cg03508765	GIMAP5;GIMAP5;GIMAP5	brown	0.10343198	-0.3521661	-0.0120129	-0.1026752	0.70254683	0.12295131
cg05840553	RPL12;RPL12;RPL12;RPL12	brown	-0.4042074	-0.3178169	-0.0829945	-0.0548955	0.32142046	-0.2535925
cg24070137	SDSL;SDSL;SDSL;SDSL;S	brown	-0.3674024	-0.6471084	-0.185164	-0.0672886	0.65674118	-0.2146776
cg04525252	TAF6;TAF6;TAF6;TAF6;TA	brown	-0.1361981	0.40720489	0.0092751	-0.0437618	-0.7025317	-0.1016319
cg16192333	SNORA70;TMX1	brown	0.15686308	-0.4170279	0.06698205	0.28165866	0.86119448	0.17343617
cg10575075	ZNF438;ZNF438;ZNF438;Z	brown	0.13930348	-0.4467012	0.1327309	0.12793087	0.83487526	0.15717459
cg11058631	IDI1;U7;IDI1;IDI1;IDI2-	brown	0.14672329	-0.4744763	0.23081607	0.18581251	0.83495555	0.18191846
cg23194271	TMEM235;AC087645.3	brown	0.03309273	0.47663516	0.10431591	0.03619631	-0.6640257	0.05864546
cg08116238	C12orf10;C12orf10;C12orf10	brown	-0.0171497	0.25076774	0.09983895	0.04780792	-0.3756138	0.02067118
cg02614847	TSPAN31;TSPAN31;TSPA	brown	0.01265867	0.55285737	0.11877612	-0.1012303	-0.8612237	0.02735182
cg01244006	SCNN1A;SCNN1A;SCNN1	brown	-0.0579117	-0.6283351	-0.0358792	0.10217213	0.83481105	-0.0667489
cg24522183	QARS;QARS;QARS;QARS;	brown	0.11366615	0.57372028	0.2437907	0.05167348	-0.6057101	0.16078302
cg08059358	AC007879.3;AC007879.2;A	brown	0.1729815	-0.3688195	-0.0056633	0.12756204	0.83751596	0.07577493

cg08028554	FLT3;FLT3	brown	0.15550053	-0.2566255	-0.0783406	0.1669495	0.7805323	0.10229739
cg19699106	COG2;COG2	brown	0.11434941	0.34214415	0.0098698	-0.0278204	-0.3428976	-0.0289665
cg06749985	TRPM2;TRPM2;TRPM2;TR	brown	-0.2888835	-0.5054986	-0.1222395	-0.1444591	0.64163658	-0.19686
cg10398799	EPC1;AL391839.1;EPC1;EP	brown	-0.2461362	-0.4146083	-0.1731994	-0.071421	0.33585189	-0.1167341
cg03996090	HSD11B1;HSD11B1;RP1-	brown	0.08119302	0.51559978	0.15399146	-0.1414239	-0.6571861	0.06589474
cg25436157	TMEM241;TMEM241;TME	brown	-0.1442967	0.46199399	0.05814515	-0.0159741	-0.58189	-0.0648276
cg21002456	AC100788.1;TIMP2	brown	-0.115976	0.35602142	0.23723092	-0.1528473	-0.6511433	0.02252627
cg21050890	CTC-295J13.3	brown	0.20681054	-0.3478559	-0.1573502	0.22003379	0.82436919	0.06232659
cg24746726	SULT1E1;SULT1E1	brown	0.13412428	-0.4565067	0.0781337	0.18944407	0.86535754	0.13644163
cg02108623	PRDM1;PRDM1;PRDM1;P	brown	0.19599375	-0.1851592	-0.2969692	0.17351287	0.63327875	0.01054672
cg26953232	BRD2;BRD2	brown	-0.1587547	0.14965939	0.25066432	-0.0050968	-0.4201406	0.07426447
cg16873443	AC114730.2	brown	-0.1946372	-0.2479355	-0.0946127	-0.0776281	0.40114662	-0.1748087
cg12549087	GSTO1;GSTO1;GSTO1;GST	brown	0.20058071	0.0587309	0.04795586	0.13719057	-0.3116375	0.29982048
cg07461273	MCM7;MCM7;MCM7;MC	brown	0.03819225	0.36164166	0.16894203	-0.0750484	-0.7078885	0.13269933
cg17589597	RP4-800J21.3;RBM38	brown	-0.2535978	0.17585286	0.0582268	-0.0729453	-0.5544355	-0.0832922
cg26770947	ANKRD13A;ANKRD13A;A	brown	0.11301525	-0.3758027	-0.0213435	0.14931684	0.84852538	0.1451489
cg10548102	MGST1;MGST1;MGST1;M	brown	0.10842296	-0.4800234	0.04033679	0.20541236	0.88209591	0.1439705
cg02780919	NRIP3;NRIP3;RP11-	brown	-0.0031735	0.05586684	0.2092544	-0.0383607	-0.244663	0.11487011
cg11469587	ST6GALNAC1;ST6GALNA	brown	0.00672354	0.34207097	0.11426792	-0.1478668	-0.6477189	0.04867186
cg25629126	AC130689.5;SMG6	brown	-0.0890051	0.28320957	0.23242455	-0.1697395	-0.5282893	0.03362483
cg12389043	CCDC92;CCDC92;CCDC92;	brown	0.20586294	-0.3012467	-0.1014152	0.11362079	0.72797756	0.06281985
cg03821474	SCARF1;SCARF1;SCARF1;	brown	-0.2364704	-0.4811765	0.06032862	0.07218891	0.72765064	-0.0661281
cg15162876	STRA6;STRA6;STRA6;STR	brown	0.19149186	0.63939306	0.19886649	-0.0443247	-0.6797882	0.12958179
cg13267195	ALDH3B1;ALDH3B1;ALD	brown	0.08399623	-0.3354734	-0.2800733	0.17094938	0.6927239	-0.078699
cg18234441	RP11-818F20.5;TXNRD1	brown	0.18185476	-0.2738198	-0.3489234	0.16281969	0.64769546	0.04168442
cg22594808	TANK;TANK;TANK;TANK	brown	-0.3753359	-0.3879502	-0.1035098	0.0527413	0.56661495	-0.4998287
cg27506442	LINC00426;LINC00426;LIN	brown	-0.2333453	0.21724356	0.05992033	-0.0968205	-0.545894	-0.1375041
cg04461802	GPR126;GPR126;GPR126;G	brown	-0.301317	-0.5727265	-0.0223544	-0.0683686	0.47976142	-0.1800683
cg25119406	RN7SKP9;UBAC2;UBAC2	brown	-0.0667957	0.43650824	0.27699719	-0.2142145	-0.8041971	0.01604367
cg14525270	TIE1;TIE1;TIE1;TIE1	brown	-0.0405495	-0.4541319	0.04287151	0.13699901	0.75818837	-0.0625895
cg20342998	BCL9L;BCL9L;BCL9L;BCL9	brown	0.11382936	0.31440015	0.39217706	0.00920525	-0.5612379	0.15320717
cg17585031	KSR1	brown	-0.0974938	0.51073693	0.25872381	0.03574585	-0.7502505	-0.021747
cg00741848	AC102953.6	brown	0.33865118	0.43101177	-0.0199276	0.09303385	-0.3838431	0.27231777
cg11993583	ZBP1;ZBP1;ZBP1;ZBP1;ZBP	brown	-0.3544652	0.09720715	0.0490854	-0.2746859	-0.494353	-0.1651421
cg12926341	AC016700.3	brown	0.12940737	0.49909451	0.05843946	-0.0927085	-0.6372928	0.0051662

cg03339288	AC002511.1;AC002511.4	brown	0.06821107	0.66329964	0.05499324	0.22544953	-0.6094444	-0.0393544
cg25786937	NAPRT1;RP11-	brown	-0.1341681	-0.4582139	0.05308511	0.1590587	0.718441	0.01141536
cg22517705	CD52;CD52;UBXN11;UBX	brown	-0.1878725	0.30061237	0.08262661	-0.1797291	-0.6631756	-0.0806837
cg14248680	SERPINB6;SERPINB6;SER	brown	-0.2424984	-0.4213653	-0.1542116	-0.1124441	0.44737872	-0.3098292
cg22164891	ZNF217;ZNF217;ZNF217;Z	brown	-0.0861908	-0.2498871	-0.1662155	0.11741571	0.486728	-0.0559956
cg05962517	ADORA2A;ADORA2A;AD	brown	0.14124166	0.28746116	0.07912954	-0.1032194	-0.6131732	-0.0153233
cg11701312	RPS5	brown	-0.0549715	0.37418436	0.21374314	0.06469688	-0.3997056	-0.0260699
cg25576364	CLEC3B;EXOSC7;CLEC3B	brown	0.02962045	-0.3146946	0.09409887	0.22902	0.62037946	0.05358704
cg06692957	GNAQ	brown	-0.2447442	-0.6012314	-0.1133442	-0.0149092	0.57108711	-0.2544237
cg17487617	METRNL;METRNL	brown	0.00209863	0.48973631	0.2321077	0.19735809	-0.573256	0.08259107
cg09445967	ZP1	brown	0.22582567	0.43505573	0.31958829	-0.0679521	-0.6298699	0.29491801
cg04050646	SLC25A20;SLC25A20;SLC2	brown	-0.0049929	0.30289624	0.10713346	-0.0642475	-0.3396135	0.00026761
cg17141212	TAF6;TAF6;TAF6;TAF6;TA	brown	-0.1492636	0.36217601	0.01012643	-0.0810608	-0.6389766	-0.1284279
cg25421594	SOAT2;SOAT2	brown	0.13808948	0.49095464	0.19302447	0.2358137	-0.5796126	0.0832098
cg21593373	AC093391.2;AC093391.2	brown	0.36184437	-0.2541593	0.05682162	0.34042511	0.74638205	0.20872299
cg24514600	PVT1;PVT1;PVT1;PVT1	brown	0.15702604	-0.449232	-0.0019996	0.11247893	0.85184676	0.10771228
cg25484698	USP18	brown	0.01494964	0.42077429	0.32361538	-0.0597006	-0.5925174	0.47471454
cg04093736	AC084082.3;AC084082.3	brown	0.37840088	0.13986616	-0.1264028	0.36714603	0.23642563	0.16235331
cg27426267	FAM118A;FAM118A;FAM	brown	-0.083138	0.49720062	0.11256184	-0.0468761	-0.8577149	-0.0625042
cg03516514	DDX24;DDX24;DDX24;DD	brown	0.41520061	-0.1724546	-0.0500408	0.3047213	0.68502229	0.29406689
cg06145370	UBE2H;UBE2H;UBE2H	brown	0.12430164	-0.4952064	-0.0801185	0.16704482	0.80840606	0.11265781
cg15265586	MEF2D;MEF2D;MEF2D;M	brown	-0.0846004	0.23189895	0.23023142	-0.2170697	-0.4356044	-0.0489301
cg13481413	C1QTNF3;C1QTNF3;C1QT	brown	0.20246548	-0.3120309	-0.0889546	0.13525165	0.7165499	0.08888128
cg09934892	CLUAP1;CLUAP1;CLUAP1	brown	0.01092022	0.5217799	0.23487029	-0.068231	-0.800445	-3.16E-05
cg06729247	AP001625.6	brown	-0.0818811	0.33898587	-0.00902	-0.0246063	-0.5741815	-0.0607046
cg22833204	STAT1;STAT1;STAT1;STA	brown	-0.0200574	0.49856071	0.11780428	-0.0664804	-0.7408516	0.02030181
cg06997537	CCR9;CCR9;CCR9;CCR9;C	brown	0.08405336	0.28531385	0.00940131	-0.1693015	-0.6735039	0.11359989
cg10443920	BCL2L2;PPP1R3E	brown	-0.0204133	0.25382653	0.07927967	0.0953198	-0.306738	-0.0977036
cg22216242	RP11-12D16.2	brown	0.22685338	-0.3432335	-0.0535335	0.30076093	0.82611643	0.14342975
cg13559810	ITPRIP	brown	0.00330058	-0.4268615	0.07346549	0.13266099	0.69749028	0.08737364
cg04101478	ZBTB7B;ZBTB7B;ZBTB7B;	brown	0.23860796	0.38486485	-0.0870728	0.00990948	-0.5293618	0.05520761
cg12559906	DENND2D	brown	0.04279216	0.47409494	-0.0848496	0.08521728	-0.7887582	-0.0440723
cg09012125	KCNJ5;KCNJ5;KCNJ5;KCN	brown	0.06661419	-0.2945414	-0.1389073	0.23681678	0.64682992	-0.1314229
cg01942558	TNFAIP6	brown	0.06041681	-0.5295671	0.00422079	0.17089671	0.91907458	0.07483731
cg24198765	ARHGEF38;ARHGEF38;AR	brown	-0.2407766	0.14408869	0.14978503	-0.210595	-0.5533868	-0.0279384

cg06547966	GRM7;GRM7;GRM7;GRM7	brown	-0.2417462	-0.7665452	-0.0071278	-0.0490573	0.67163664	-0.1093851
cg24119798	RP11-430H10.1;RP11-	brown	-0.2957987	0.1964972	0.19257761	-0.2081294	-0.6291517	-0.1025454
cg16118387	SERTAD4;C1orf133;C1orf13	brown	-0.2049021	0.21422555	0.21034582	-0.1422687	-0.5549509	-0.0782971
cg03190891	SORBS1;SORBS1;SORBS1;	brown	-0.007937	0.40712016	0.10327496	-0.1492822	-0.6868089	0.08185125
cg07746081	ASH2L;ASH2L	brown	-0.1804723	-0.5961468	-0.0432588	0.10350764	0.72286752	-0.0621097
cg25151013	RP11-1055B8.4	brown	0.00449445	0.39537674	0.44863694	0.01341258	-0.6025839	0.1237276
cg22126095	TECPR2;TECPR2	brown	0.14571548	0.6519846	0.17580008	0.07072729	-0.5866912	0.12295223
cg14915060	MAT1A;MAT1A;MAT1A	brown	0.13267039	0.70756313	0.21661993	0.04130945	-0.6911065	0.09571941
cg20248774	RP11-87E22.2;RP11-	brown	0.03170016	0.60349954	0.105525	-0.0166872	-0.6578545	-0.0764827
cg12717944	FADS3;FADS3;FADS3;FA	brown	0.10129478	0.51594222	0.11697401	-0.0908512	-0.6783721	0.05297087
cg23144473	TRGV10	brown	0.06920421	-0.2894261	-0.4868287	0.13636201	0.60757145	-0.0576058
cg08923922	AMPD3;AMPD3;AMPD3;A	brown	0.1989029	-0.310921	-0.1029667	0.29056073	0.77702188	0.04775175
cg18299860	SMG6;RP11-	brown	0.17200812	0.56071773	0.11933481	0.15263288	-0.5264369	0.08829697
cg18697636	SLC9A3;SLC9A3;RP11-	brown	-0.0677376	-0.5797517	0.05109556	-0.0100805	0.5494219	0.09114714
cg25157073	PDHX;PDHX	brown	0.16745096	-0.4778769	-0.125075	0.19241078	0.77752205	0.10712483
cg14038949	PFKFB3;PFKFB3;PFKFB3;	brown	-0.0335131	0.33224446	0.14790942	-0.1742029	-0.7579448	0.07660578
cg11810462	ASIC1;ASIC1	brown	-0.0302513	0.40578546	0.09603965	-0.0915878	-0.6924653	-0.0159573
cg13015534	ST6GALNAC1;ST6GALNA	brown	0.10302439	0.35633762	0.04455449	-0.021268	-0.5221908	-0.0031595
cg12870052	BLM;BLM;BLM;BLM	brown	0.11004719	-0.5116242	0.05599173	0.23696594	0.90472097	0.16689382
cg24980428	RP11-108M21.1	brown	-0.0467574	0.60706201	0.11116199	-0.1384731	-0.8129434	-0.0251654
cg15975802	PTPN6;PTPN6;PTPN6;PTP	brown	-0.237024	-0.7068545	0.00562077	0.02534062	0.73525811	-0.0484953
cg14943837	TBC1D10A;TBC1D10A;MT	brown	-0.0247216	0.44768596	0.18441611	0.16549227	-0.7341601	0.01116406
cg00491948	MAK;MAK;MAK;TMEM14	brown	0.05740748	0.47165087	0.1056316	-0.0342545	-0.5653357	-0.0697929
cg11931463	RP11-6F2.6;RP11-6F2.4	brown	-0.0212197	0.45844072	0.21459608	0.01062357	-0.6216054	0.08189426
cg24815934	ITGB2;ITGB2;ITGB2;ITGB2	brown	-0.3214104	0.13584315	0.03245567	-0.1791099	-0.5499722	-0.1388649
cg09858862	ID3	brown	0.09040624	-0.4761268	0.07471682	0.28788808	0.85762113	0.14759734
cg26066951	CYTH1;CYTH1;CYTH1	brown	0.04604586	0.37181693	0.16893358	-0.1094481	-0.6698742	0.19041139
cg15665309	CTB-99A3.1	brown	0.11585172	0.59520035	0.42875729	0.1212109	-0.5214242	0.06928028
cg18346693	STMN3	brown	0.40740742	0.37310863	-0.0006562	0.1372524	-0.3123648	0.15012938
cg14332490	HNRNPC;HNRNPC;HNRN	brown	-0.0792501	0.39383345	0.10532694	-0.0514667	-0.6757127	-0.1083636
cg27204405	FCRLA;FCRLA	brown	-0.0849031	0.26161446	0.36926392	-0.0753767	-0.5526784	0.03118819
cg20114528	ASB8;ASB8;ASB8;ASB8;A	brown	0.21581008	0.70355944	-0.1066799	0.2107716	-0.691669	0.03206229
cg10469818	ZNF333;ZNF333;ZNF333;Z	brown	0.06605471	0.32646379	0.16709256	0.29866754	-0.445141	0.13320511
cg21748271	EDEM2;EDEM2;EDEM2;ED	brown	-0.0600208	-0.4691956	0.01988053	0.13769242	0.70262907	0.01978654
cg21675036	RP11-	brown	-0.1504351	-0.6069325	-0.0580919	0.03434114	0.61964719	-0.1262046

cg09862861	LCN8;LCN8;LCN8	brown		0.14123485	0.39133707	0.00121066	0.05586396	-0.4657479	-0.0737938
cg18383660	HCRTR1;HCRTR1	brown		-0.0250715	0.5237672	0.25097587	-0.0080754	-0.6528762	0.1133795
cg14755717	CD81;CD81;CD81;CD81;CD	brown		0.06847048	0.43866253	0.16019242	-0.1282087	-0.703337	-0.0020387
cg07298397	AC007381.3	brown		0.05913117	-0.3090928	-0.1005488	0.17324265	0.55637857	0.10005572
cg19563510	MAFG;MAFG;MAFG	brown		-0.0438277	-0.6435568	0.06142848	0.07982582	0.75948059	0.02790872
cg16075139	CD81;CD81;CD81;CD81;CD	brown		0.24028642	0.66001267	0.20743244	-0.0347425	-0.6801399	0.05180089
cg05088285	SETD9;SETD9;SETD9;SET	brown		-0.2085751	-0.5489471	-0.085518	-0.034598	0.58799854	-0.1774693
cg15288326	KIF21B;RP11-	brown		0.15209124	-0.4078835	-0.0839751	0.11817508	0.83311812	0.16911994
cg16994041	PRKAG2;RP13-	brown		0.11093471	-0.3679253	-0.0593478	0.1797022	0.71223319	-0.0689468
cg16837379	CLDN9;CLDN6	brown		0.27432085	0.49571513	0.25778046	0.02074514	-0.4486877	0.08923857
cg20795401	ITGA6;ITGA6;ITGA6;ITGA	brown		-0.2117794	0.17335934	0.2186639	-0.0537006	-0.5423625	-0.0045174
cg15418246	NDUFS2;ADAMTS4;ADA	brown		0.27824666	-0.1565197	0.00946414	0.22006091	0.6163636	0.16858113
cg21124491	TMEM30A;TMEM30A	brown		0.21921319	-0.199931	-0.2495484	0.25570659	0.6427207	0.05832551
cg17029023	FAM129C;FAM129C	brown		-0.1280529	0.43227291	0.29008871	-0.1789827	-0.7457171	-0.0205397
cg01433291	CORO7;CORO7;CORO7;CO	brown		0.23647031	0.39409468	-0.0548174	0.20838401	-0.3368376	0.18118736
cg27407935	SREBF1;SREBF1;SREBF1;S	brown		-0.1686438	-0.3977458	0.17808268	-0.0159378	0.67912022	0.01260847
cg04549162	GPR26	brown		-0.1548433	-0.6491282	0.11992646	-0.0457111	0.61634774	0.05662622
cg09488881	RP11-439C8.2	brown		0.24787935	-0.0966248	-0.1100458	0.15783668	0.64857608	0.08736551
cg07677192	snoU13;RP11-629N8.3	brown		0.13202145	-0.457116	0.06040384	0.16031809	0.78013162	0.10219288
cg15610510	IFIT3;IFIT3;LIPA;LIPA;LIP	brown		-0.0305517	0.29712394	0.054836	-0.032351	-0.4184942	-0.0393579
cg01392760	DFNA5;DFNA5;DFNA5;DF	brown		0.00511402	0.14343992	0.13218057	0.02996623	-0.2456765	0.14317688
cg22643165	IGHV1-2	brown		0.26155594	0.60990229	-0.1150795	-0.0192237	-0.6072344	0.11968749
cg03620325	C2CD3;C2CD3;C2CD3	brown		0.09914033	-0.4554357	-0.1016662	0.14971283	0.83467374	0.11151634
cg13263397	ATP1B1;ATP1B1;ATP1B1	brown		0.05037576	-0.3710008	-0.0968716	0.15189588	0.72047546	0.00166084
cg10203076	AC005355.1;RP11-215P8.4	brown		0.17995593	0.39895817	0.040165	-0.0314558	-0.4648253	0.18515494
cg07828990	3-Mar	brown		-0.0537804	0.35912168	0.25702563	-0.0984928	-0.5513435	-0.0381237
cg04488758	USP44	brown		0.10857487	0.2985028	0.08335727	-0.0514268	-0.4665283	0.0383732
cg09251557	ALDH5A1	brown		-0.0643483	0.49381602	-0.0780572	-0.0573355	-0.5274097	-0.1706987
cg07173112	TTLL10;TTLL10;TTLL10;TT	brown		-0.033841	0.53246499	0.1091042	-0.0637947	-0.6395005	-0.0200015
cg21048162	BATF;BATF;BATF	brown		-0.0512695	0.50521137	0.0481895	-0.1087046	-0.7717402	-0.0442129
cg23564253	S100B;S100B	brown		0.11810009	-0.3088613	-0.1334769	0.22688082	0.68984134	-0.0327896
cg10223809	AC091814.3;CLEC12A	brown		0.10432643	0.47445646	-0.0792654	-0.1491829	-0.6138102	0.0012045
cg00094764	USP7;USP7;USP7;USP7;US	brown		0.25026025	0.62129847	-0.0530319	0.17194968	-0.5478667	0.21093783
cg25451120	GNGT2;ABI3;GNGT2;GNG	brown		-0.0175427	0.56174513	0.20286243	-0.0440518	-0.7400667	0.00140854
cg18582408	SLC4A7;SLC4A7;SLC4A7	brown		0.09935078	-0.375463	-0.2254864	0.18489824	0.78780817	-0.0443784

cg12584702	RP1-50J22.4;ETV7	brown	-0.200171	-0.643433	-0.1311085	-0.03377	0.6790661	-0.1925791
cg08853318	SLC39A11	brown	0.0994962	0.51941603	0.18687607	-0.033251	-0.7110054	0.10655228
cg00183186	HTR1B	brown	-0.1687857	-0.7379364	-0.0006062	0.00789441	0.67360812	-0.0569324
cg05870818	FCER2;FCER2	brown	0.18128967	0.57406292	-0.1395521	-0.0326354	-0.6810399	0.08826883
cg21770784	AL844908.5;AL844908.5;IT	brown	-0.2951897	0.21051022	0.11041713	-0.2034062	-0.6707495	-0.1564076
cg06468672	COBLL1;AC019181.2;COBL	brown	0.14043656	-0.2299491	0.31875538	0.1461736	0.55526347	0.1546795
cg16498634	IRF8;IRF8;IRF8;IRF8;IRF8;I	brown	0.0574455	-0.3628875	-0.1490196	0.1113554	0.75693556	0.05824755
cg01886988	RP11-	brown	-0.2324466	0.30483018	0.18911399	-0.0886191	-0.6478249	-0.0769602
cg11623855	C11orf41;C11orf41;C11orf41	brown	-0.0128768	0.60839256	0.24918954	-0.0027317	-0.7145367	0.00221575
cg14475636	C5orf42;C5orf42	brown	-0.2068019	0.21817938	0.00933059	-0.1591928	-0.5395816	-0.1186676
cg13903164	SAMD14;SAMD14;SAMD1	brown	-0.0894778	0.30990373	0.25321767	-0.0679804	-0.6900012	0.11704855
cg26828058	SAMD3;SAMD3;SAMD3;S	brown	0.0780702	0.44172387	0.50663776	0.01161108	-0.6367245	0.2234921
cg21829166	MICAL2;MICAL2;MICAL2;	brown	0.1352876	-0.4970526	-0.0085051	0.12391879	0.82802127	0.1562388
cg01792532	S100PBP;S100PBP;YARS;S	brown	0.05568256	0.38453758	0.16278228	0.04898895	-0.5685107	0.10152425
cg06376426	GRHL3;RP11-10N16.3;RP11-	brown	-0.1768864	0.15159063	0.35721213	-0.1517002	-0.5319939	0.03816765
cg21291431	SORBS3;SORBS3;SORBS3;	brown	-0.2446181	-0.4199409	-0.1448187	0.08117058	0.54147423	-0.4990014
cg11537677	AC007381.2	brown	-0.0815984	0.45595133	0.05692578	-0.1592065	-0.7466312	-0.0206533
cg02233041	TNIP3;TNIP3;TNIP3	brown	0.20756938	-0.4376633	-0.0084371	0.1551812	0.84517471	0.16356136
cg06926818	CHN2;CHN2;CHN2;CHN2	brown	0.17488299	-0.3817426	-0.0506876	0.16981649	0.81292378	0.12548934
cg04771042	SORBS3;SORBS3;SORBS3;	brown	-0.025773	0.2019511	0.2988617	-0.156756	-0.3981142	0.07998694
cg17478888	MBTPS1;MBTPS1	brown	0.09974802	0.41982433	-0.115684	0.15061442	-0.6278719	-0.0119193
cg03490138	CD27;LOC678655;LOC6786	brown	0.06561211	0.31286971	0.15840212	-0.1220566	-0.5552367	0.0685137
cg06180354	SCYL2;SCYL2;SCYL2;SCY	brown	0.21483631	-0.3758494	-0.036843	0.19848915	0.85176254	0.10330648
cg06627114	TCF4;TCF4;TCF4;TCF4	brown	0.19115443	0.3444486	0.16912286	-0.0522507	-0.5027938	0.39230727
cg19810953	RPL12;RPL12;SNORA65;RP	brown	-0.0099621	0.55509534	0.2738456	0.08503503	-0.5623755	0.07583272
cg05044173	CD4;CD4;CD4;CD4;CD4;C	brown	0.02697152	0.50955654	0.24479387	-0.0983687	-0.6949579	0.12315267
cg06967105	MIR429	brown	-0.0080102	0.52973551	0.25747387	-0.0755862	-0.7246498	0.08900615
cg03535732	TOP1MT;TOP1MT;U6;TOP	brown	0.21618813	0.40395843	0.33442522	0.08576534	-0.5502503	0.20473311
cg00318699	AC104782.3;AC104782.3	brown	0.20181956	0.39704566	-0.0462152	-0.0712704	-0.3932447	0.07286021
cg18460107	CLEC4C;CLEC4C	brown	-0.1186808	0.48013658	0.06050425	0.10549932	-0.6760092	-0.1013565
cg17836177	RP11-87E22.2;RP11-	brown	-0.1136598	0.38272429	0.19542019	-0.1117393	-0.6789144	-0.0081959
cg11976790	DMBT1;DMBT1	brown	0.05274035	-0.3415174	-0.3899681	0.01782529	0.58027255	0.00045557
cg00662956	ETV5;DGKG	brown	0.12117342	0.50238069	0.15018682	-0.1015948	-0.480172	0.02758715
cg14750778	PIGS;PIGS;PIGS	brown	0.01241131	0.35430004	0.18666199	0.22419286	-0.5335574	0.05416482
cg23869295	MGLL;MGLL;MGLL;MGLL	brown	-0.0066136	0.32398084	0.18025465	0.05573939	-0.586796	0.09065002

cg11566441	DIXDC1;DIXDC1;DIXDC1;	brown	0.15196965	0.49904633	-0.1385659	0.20820168	-0.5102403	0.14320804
cg15562617	RP11-18F14.1;RP11-	brown	-0.2339146	0.34095379	0.12938531	-0.1117607	-0.629263	-0.0860678
cg09489811	ARHGEF38;ARHGEF38;AR	brown	-0.2280688	0.24026793	0.15675146	-0.2211019	-0.6638305	-0.0583783
cg06005133	ZNF705A;ZNF705A;ZNF70	brown	-0.0059873	-0.3987512	-0.4081166	0.11037767	0.67417704	-0.0306889
cg13207340	MTA2;MTA2;MTA2;MTA2	brown	-0.0072774	0.57098017	0.20278986	0.08587829	-0.8020947	0.06299683
cg10780517	PER2	brown	-0.228553	0.27134295	0.08387709	-0.174598	-0.575191	-0.114659
cg07743451	TPM3;TPM3;TPM3;TPM3;T	brown	0.10855649	0.47403108	-0.0297461	0.29477558	-0.4880079	0.02873582
cg05283385	RAPGEF1;RAPGEF1	brown	-0.1609939	0.45348279	0.17057891	-0.0100171	-0.6694123	-0.0441793
cg25992672	RP11-638I2.2	brown	0.17905051	0.39161264	0.05308587	-0.0562976	-0.5607266	0.5381574
cg15746849	RP11-255B23.3	brown	0.18915946	-0.4078909	0.08694953	0.20442299	0.84594971	0.1340501
cg13549362	IGLV4-	brown	-0.0630636	0.43390723	0.21000435	-0.203215	-0.6706311	-0.0700524
cg21580043	ATPAF2;ATPAF2;ATPAF2;	brown	-0.0524793	-0.3087727	0.08674374	0.00282987	0.73377273	0.03547474
cg17100513	RP5-1092A11.5;TP73;TP73	brown	-0.1365609	-0.5871762	0.03560093	0.11666205	0.73643662	-0.0905359
cg16353907	IDI1;U7;IDI2-AS1;IDI2-	brown	0.20666671	-0.3975105	0.23348088	0.16750238	0.78741522	0.22333031
cg24361265	ELL3;ELL3;SERF2;SERF2;E	brown	0.28194914	0.42435802	0.08016726	-0.0030363	-0.3672477	0.03926322
cg04190651	C19orf59	brown	0.14256553	0.43901459	0.15736654	0.49476292	-0.510226	0.12837566
cg11416297	RP11-862G15.1	brown	-0.0927324	0.5021589	0.17539696	-0.0808866	-0.6368135	-0.0409724
cg09991306	SNED1;SNED1;AC005237.4	brown	0.22063066	0.51183398	0.16594041	-0.019944	-0.6290245	0.13348798
cg14032729	MEI1;MEI1;MEI1;MEI1;MEI	brown	-0.2369969	0.31921279	0.19143034	-0.1949409	-0.6721813	-0.134055
cg13353113	NREP;NREP-AS1;NREP-	brown	0.22456258	-0.359926	-0.0533976	0.22745002	0.84686603	0.14625101
cg08419648	FBXW4;FBXW4;FBXW4;F	brown	-0.0291192	0.49328919	0.22771653	-0.0993519	-0.6911352	0.00245426
cg14899065	IL16;IL16;IL16	brown	0.13316235	-0.4283645	0.0287205	0.20360708	0.80374985	0.17029539
cg12513294	STIM1;STIM1;STIM1;STIM	brown	0.12765852	0.27606893	-0.0232489	0.25938356	-0.4877126	0.0894765
cg16764781	TP63;TP63;TP63;TP63	brown	0.1649166	-0.3502838	-0.0387688	0.1959673	0.78027626	0.08609641
cg21916114	AP003419.15	brown	-0.0604727	0.43338404	0.18686764	-0.1147362	-0.6404738	-0.0774551
cg03407567	MYL6B;RP11-	brown	-0.2201054	0.23406072	0.10867868	-0.0397981	-0.5200666	-0.0686811
cg24848696	C11orf42	brown	0.17016637	-0.3700029	-0.0882286	0.10878068	0.79562864	0.01236065
cg10236261	IFT81	brown	-0.1075083	0.22350362	-0.1870821	-0.1740554	-0.4608427	-0.1874298
cg25075135	MAP7;MAP7;MAP7	brown	-0.0365317	-0.6079717	-0.0430578	0.09392984	0.70308592	0.02181033
cg04822495	RP11-1105G2.3	brown	-0.0210936	-0.314708	0.19500115	0.1673991	0.69640393	0.11071833
cg04925604	MANBAL;MANBAL;MAN	brown	0.06900877	-0.4756146	-0.3480951	0.05012459	0.67734649	0.00403766
cg12141090	C21orf7;C21orf7;C21orf7;C2	brown	0.17189507	0.48571943	0.23400932	0.22241326	-0.6175076	0.04268817
cg24037916	WSB2;WSB2;WSB2;WSB2;	brown	-0.1880185	-0.6395115	-0.1766112	-0.1017453	0.55835793	-0.1057641
cg02284273	FRMD4A	brown	0.13310314	0.35337397	0.13637314	0.09004023	-0.3972995	0.19647122
cg10526478	IRF8;IRF8;IRF8;IRF8;IRF8;I	brown	-0.1716572	0.33625784	0.28526441	-0.0791749	-0.6244084	-0.0064007

cg26599122	RP3-340N1.5;UBXN10	brown	0.08561661	0.51486435	-0.1665443	0.17446351	-0.4392739	-0.0874939
cg04118190	AC012307.2;DUSP2	brown	0.02833951	0.23566347	0.34505563	-0.0267754	-0.52277	0.23901258
cg02513379	IL21R;IL21R;IL21R	brown	-0.1363708	0.32922801	0.07586556	-0.0609272	-0.5457303	-0.1174358
cg25104637	NRXN2;NRXN2	brown	0.22365373	0.31679521	-0.0719678	0.02633209	-0.3667306	0.24041231
cg13280041	NHSL1	brown	0.40448518	0.40356144	0.10326789	0.16401333	-0.326019	0.3901749
cg09857555	TRBV6-1	brown	0.01456018	-0.3690596	0.00035378	-0.0205698	0.66586216	0.03000431
cg18616757	IKBKE;IKBKE;IKBKE;IKB	brown	-0.1213216	0.4576322	0.10846129	-0.1363819	-0.5852671	-0.004922
cg01475000	IFNAR2;IFNAR2;IFNAR2;I	brown	0.05293479	0.36877104	0.15101589	0.0342622	-0.4094119	0.07160769
cg23872046	RP11-725G5.2	brown	-0.1219266	0.33363854	0.16183993	0.01936854	-0.4897559	0.01754139
cg16368519	SUN1;SUN1;SUN1;SUN1;S	brown	-0.0123448	0.2494583	-0.041235	-0.0899689	-0.4895239	-0.0182526
cg03995631	BTLA;BTLA	brown	-0.0580991	0.17973191	0.09113065	-0.1730756	-0.5101503	0.11741382
cg05449217	WDR59	brown	-0.1035125	-0.6276027	-0.0389918	0.07228706	0.76196384	-0.1931739
cg21307484	IL2RB;IL2RB;IL2RB	brown	-0.1626381	0.32586891	0.1963672	-0.1360735	-0.6667529	0.01093667
cg19159092	RP11-54O7.3	brown	-0.1656607	0.30769497	0.12699017	-0.1268233	-0.5822778	-0.0166831
cg01883408	CCNI2	brown	0.112612	0.25073813	0.2115958	-0.0992885	-0.3101508	0.12555644
cg20981515	PDGFRB;PDGFRB;PDGFR	brown	0.06745809	-0.3555062	-0.0032056	0.21898638	0.8462899	0.06509137
cg26231961	FADS3;FADS3;FADS3;FA	brown	0.18345563	0.47423153	0.11688204	-0.0538404	-0.4881838	0.08187977
cg14801565	KIF1A	brown	0.07699576	0.68774806	0.02120701	-0.0193392	-0.6496134	0.01231878
cg19396218	RP11-124O11.2	brown	0.01298322	0.61976967	0.25156431	0.19734059	-0.5920952	0.00090634
cg00227093	SYNPO	brown	0.21665091	-0.0796098	-0.0609232	0.12121295	0.64678142	0.07885531
cg15564579	ADPRH;ADPRH;ADPRH;A	brown	0.08862059	-0.5245593	0.232011	0.26523723	0.81721479	0.19598304
cg13318911	CECR3	brown	0.17969682	0.358921	-0.0140668	-0.1292744	-0.5182321	0.10893165
cg02650122	APBB1;APBB1;APBB1;AP	brown	0.23198093	0.3875862	0.33074367	0.11558959	-0.468678	0.26564746
cg17611742	DENND1B;DENND1B;DEN	brown	-0.2162613	0.33923264	0.1720215	-0.1054073	-0.6418635	-0.1209681
cg00966098	RP11-146F11.5;ZNF785	brown	-0.1399658	-0.6592208	-0.0410032	0.01550308	0.76134399	-0.1034783
cg08364283	PXN;PXN;PXN;PXN;PXN;	brown	0.01937509	0.31992675	0.03387497	0.24949039	-0.5594206	0.0157287
cg19675778	SORBS3;SORBS3;SORBS3;	brown	-0.0662029	-0.6207058	0.09424409	0.03027392	0.62697387	-0.0078772
cg26582754	RP3-468K18.5;PHACTR2	brown	0.12758549	-0.2403364	-0.1345781	0.23467656	0.74170136	0.05390445
cg09696044	RP4-800J21.3;RBM38	brown	-0.114927	0.34790492	0.08061986	-0.0739084	-0.666683	-0.1005835
cg01491603	TMEM66;TMEM66;TMEM6	brown	-0.140587	0.22509238	0.10882082	-0.122059	-0.5469344	-0.0718688
cg10203699	MICALL1;MICALL1	brown	0.22408612	0.54878692	0.20516311	0.0263468	-0.5318875	0.09197686
cg06295676	PSPC1-	brown	0.12468692	-0.4119157	0.38766738	0.24688989	0.72007178	0.22720723
cg10401268	FPGS;FPGS;FPGS;FPGS;FP	brown	0.27742309	0.36162711	-0.0083387	0.04277519	-0.4256799	0.28607322
cg20187011	ADAMTS1	brown	0.0303469	-0.3704803	0.13962483	0.24685848	0.67400343	0.03381349
cg03398090	TNFRSF25;TNFRSF25;TNF	brown	-0.2120493	0.28177588	0.18458374	-0.1482663	-0.6306825	-0.0844479

cg08036668	GALK2;GALK2;GALK2;G	brown	0.12295523	0.51188156	0.27038785	0.19215858	-0.592783	0.08772653
cg21113478	DCLK3	brown	-0.0040089	0.44861094	-0.0008896	0.16401028	-0.5866777	0.02299833
cg13241077	FAM194B;FAM194B	brown	-0.2464545	-0.6851476	-0.0219549	-0.0502762	0.68916891	-0.1823985
cg10117369	LAX1;LAX1	brown	-0.0267324	0.41592088	0.2119951	0.11203849	-0.6895593	-0.0008456
cg01930676	MRPL1;MRPL1;MRPL1;MR	brown	-0.0283648	0.32869103	0.07685349	-0.1724902	-0.5940653	-0.0912098
cg07335019	HTT;HTT	brown	-0.0968107	0.33911018	0.18815218	-0.0667766	-0.5613668	-0.0004115
cg13408636	BCL2L2;PPP1R3E	brown	0.01485288	0.45575993	0.26370513	0.07822433	-0.6388688	-0.0242305
cg10632672	DCDC2B;DCDC2B;IQCC	brown	0.10296888	0.38767541	0.11625339	-0.1005227	-0.5775239	0.10102195
cg11824827	AC135050.5	brown	0.15196959	0.54419374	0.37736002	0.03242036	-0.6280259	0.19867139
cg23946686	C10orf27;C10orf27	brown	0.17139535	-0.3450385	0.17950682	0.20163567	0.71932548	0.16140446
cg10156042	DEPDC5;DEPDC5;DEPDC5	brown	-0.175719	-0.5945083	-0.0882633	0.04366403	0.70349282	-0.1169505
cg04317686	ASB10;ASB10;ASB10;ASB	brown	-0.103506	0.42696706	0.0996163	-0.1480547	-0.7312835	-0.0367916
cg12872357	RAB32	brown	0.27154576	-0.3535292	-0.0131579	0.1719527	0.7811353	0.15709602
cg16790524	IGLV3-9	brown	-0.2129587	0.28964656	0.11224465	-0.3097346	-0.6878807	-0.1144609
cg05871405	USP10;USP10;USP10;USP1	brown	-0.1452707	-0.5468764	-0.097971	-0.1842676	0.53926445	-0.0192886
cg10440286	ELFN2;ELFN2;RP1-	brown	0.32625863	0.57174625	0.19769968	0.04290154	-0.5793705	0.28452995
cg19829001	ST6GALNAC1;ST6GALNA	brown	0.28438269	0.33030375	-0.1848046	0.23473594	-0.2591616	0.11723524
cg02191305	NPHP4;NPHP4;NPHP4	brown	0.00114918	0.33134924	0.374755	0.00893705	-0.6557162	0.08940844
cg17569842	RGS12;RGS12;RGS12;RGS	brown	0.04183975	0.27445596	0.11239005	-0.0049027	-0.4227904	0.12863013
cg03847895	ANKRD52;ANKRD52	brown	0.10661445	-0.3312589	-0.200533	0.18944945	0.73562674	-0.0089151
cg12018809	LPAR5;LPAR5;LPAR5;LPA	brown	-0.1811853	0.30784513	0.14773378	-0.1330833	-0.6064738	-0.19277
cg25753024	SSH3;SSH3;SSH3;SSH3;SS	brown	-0.0470296	-0.3615938	0.18973986	0.1305328	0.49244977	0.11515678
cg25754958	PTPN7;PTPN7;PTPN7;PTP	brown	-0.0347015	-0.3866807	0.03108527	0.15301634	0.73042518	-0.0088494
cg06762457	ZC3H12D;ZC3H12D;ZC3H1	brown	0.12088876	0.55983352	0.21482173	0.14482887	-0.5130527	0.09427823
cg03363633	TYROBP	brown	-0.2247957	-0.5065549	-0.1032347	0.03679978	0.61355493	-0.4941412
cg01586609	HTR3A;HTR3A	brown	0.10763743	-0.4254596	-0.0693171	0.0906601	0.80094881	0.13058457
cg26035676	RP11-	brown	0.10379478	-0.4205782	-0.0747738	0.24088641	0.72448431	0.06980704
cg05406621	ZNF683;ZNF683;ZNF683;Z	brown	-0.0297924	0.48302524	0.1171473	-0.1705922	-0.7332638	0.01583896
cg20696806	SLC4A4	brown	0.14036045	-0.444007	0.10535968	0.22501961	0.81286881	0.12699537
cg20892847	ARHGEF25;SLC26A10;AC0	brown	0.23892332	0.27430719	0.11661695	0.23921774	-0.2345361	0.29101276
cg17745693	TPRG1;TPRG1;TPRG1;TPR	brown	0.23931265	-0.2729931	-0.1089894	0.2517183	0.80183251	0.15103008
cg25101092	AC109826.2;AC109826.1	brown	-0.0081335	0.43348156	0.32277573	0.00639289	-0.7469202	0.10117358
cg16544318	RP11-474L11.5	brown	0.05267143	-0.328325	-0.1991677	-0.0113291	0.71631476	0.0521952
cg05880138	RP11-5P18.5;C1orf64	brown	-0.0158352	0.18852808	0.01847867	-0.0539601	-0.4062059	0.03899643
cg15633128	SLC16A9;SLC16A9;SLC16	brown	-0.0535808	0.34731805	0.06769547	-0.0218613	-0.5166476	-0.1030166

cg17155829	CTB-133G6.1	brown	0.0573523	0.38192208	0.09514734	-0.006736	-0.5853447	0.11299723
cg22578433	CCND3;CCND3;CCND3;CC	brown	0.23945907	0.53330546	0.12264297	0.02878427	-0.6044591	0.25438856
cg10211626	C22orf46;NHP2L1;C22orf46;	brown	0.18185319	0.29018687	0.22483514	0.18923402	-0.2227956	0.24826432
cg24287110	KLF6;KLF6	brown	-0.0856417	0.48925333	0.01056958	-0.0735191	-0.6417557	-0.0571548
cg00933182	FMNL1;FMNL1	brown	0.11627673	0.43820272	0.09557868	0.04799811	-0.5456098	0.10747654
cg05884711	ATP6V0C;ATP6V0C;RP11-	brown	0.14272683	0.31455659	0.07626653	-0.1414763	-0.4819291	0.14768575
cg00659077	HSBP1L1	brown	0.21108792	0.5041746	0.07305297	0.0681844	-0.5536659	0.30167656
cg24699026	TAGAP	brown	0.24702646	-0.1745052	0.0048689	0.2585787	0.7117903	0.12291021
cg25667337	AC012307.2	brown	0.02188059	0.40923669	0.30809229	0.18777791	-0.7015032	0.12047857
cg25123866	PYGM;PYGM	brown	0.02026284	0.488281	0.10443979	0.00823861	-0.5145683	-0.0584108
cg03229930	7SK	brown	0.20788364	-0.3926881	0.15512158	0.12106804	0.79272484	0.18191654
cg17439633	SLC43A3;RP11-	brown	-0.0517628	0.10774959	-0.1853436	-0.1063693	-0.2918415	-0.0062888
cg11667763	AC016700.3	brown	0.20183085	0.43394133	0.15664507	-0.054797	-0.4619742	0.12472717
cg19658600	TSPAN32;TSPAN32;TSPA	brown	0.02480126	0.29089082	0.36137193	-0.0555458	-0.4973938	0.14603511
cg08467103	SPRED2;SPRED2	brown	0.10924645	-0.2152445	-0.0234778	0.03705109	0.7029462	0.15716426
cg08508845	RP11-300J18.1;RP11-	brown	-0.0938399	0.33858373	0.07287156	0.01695077	-0.6439955	-0.1089109
cg18104968	LTBP3;LTBP3;LTBP3;LTBP	brown	0.10765675	0.33214539	-0.0412863	-0.2701605	-0.4451989	0.04650724
cg19801025	ST7-OT4;ST7-OT4;ST7-	brown	0.04143864	-0.3956299	-0.3671562	0.1047104	0.66814897	-0.0825749
cg00169122	RP1-	brown	0.18412065	0.44231264	-0.0742238	-0.0397234	-0.413048	0.2371555
cg04851848	CTC-461F20.1;RP11-	brown	0.05066031	0.14661342	-2.17E-05	-0.0750332	-0.4719689	0.13396383
cg06387582	ABR;AC016292.1;ABR;ABR	brown	-0.1157712	0.45037602	0.13264459	-0.1142575	-0.7010741	-0.0599074
cg14216634	TRIB1;TRIB1;TRIB1	brown	-0.1164878	-0.5567073	0.02100176	0.14106179	0.80732862	-0.0892024
cg08264507	PCTP;PCTP;PCTP;PCTP;PC	brown	0.27132714	-0.3473865	0.10867213	0.1813921	0.77779523	0.11960773
cg24662231	AOC3;AOC2	brown	-0.0456663	0.55935928	0.18528364	-0.1043664	-0.7088977	0.07576325
cg11403831	FER;FER;FER	brown	0.2079563	-0.3968871	-0.0332627	0.17603403	0.84772703	0.11513511
cg14174912	KLF6;KLF6	brown	0.07005081	0.52232929	0.16280505	0.11823358	-0.5380748	0.04167932
cg22616379	LIME1;LIME1;LIME1;LIME	brown	-0.1774279	0.28843738	0.21267578	-0.1878695	-0.691783	-0.0451876
cg22728178	CTC-461F20.1;RP11-	brown	0.0244647	0.24451554	0.10633671	-0.0741663	-0.551906	0.07883945
cg22581187	IQGAP2;IQGAP2;IQGAP2	brown	0.21580542	-0.3684133	-0.0800593	0.21912218	0.78463382	0.12194111
cg20021790	RP11-1260E13.4;RP11-	brown	-0.0051255	0.20910342	0.1525504	-0.1363057	-0.4515436	0.10217607
cg01839993	DDIT4;DDIT4	brown	-0.1828716	0.22389249	0.20989518	-0.1738617	-0.5878578	-0.0388568
cg18169610	CD81;CD81;CD81;CD81	brown	0.0435929	0.4283705	-0.0270156	-0.1367775	-0.5911761	-0.0115454
cg04903269	CELF6;CELF6;RP11-	brown	-0.1957407	-0.3613501	0.02462273	0.11662114	0.63572527	-0.1907817
cg05726208	PRDM10;PRDM10;PRDM1	brown	0.19470022	0.36556218	-0.2919376	0.0173177	-0.4074401	-0.0552908
cg18326313	PSPC1-	brown	0.18245192	-0.3978378	0.03135232	0.19045231	0.85064746	0.12213338

cg19188021	ODF3B;ODF3B;ODF3B;OD	brown	-0.1136183	-0.6456971	-0.0582054	-0.0670256	0.71658079	-0.118332
cg13668327	CYTH1;CYTH1;CYTH1	brown	-0.1841782	0.28304412	0.08959984	-0.2295774	-0.653506	-0.1191465
cg06736444	CTD-2270P14.3;CTD-	brown	0.0052429	0.56923721	0.19926526	0.23816884	-0.6353652	0.03293847
cg04346683	UBE2Q2	brown	0.10253091	-0.3929415	-0.2764126	0.19266418	0.76100199	-0.0169717
cg00607630	USP7;USP7;USP7;USP7;US	brown	0.15063599	0.343311	0.01598241	-0.0278072	-0.5583033	0.10306275
cg15161691	B3GAT1;B3GAT1;B3GAT1	brown	0.26892652	0.57914573	0.21768776	0.06941736	-0.5009823	0.20326255
cg10690747	UBASH3A;UBASH3A;UBA	brown	0.03060824	0.39853672	0.25197876	0.01373818	-0.6404253	0.14407522
cg15282665	ST6GAL1;ST6GAL1;ST6GA	brown	0.0913627	-0.4377054	-0.3070746	0.18201335	0.72752822	0.00681358
cg04671853	TTC16;TTC16;TTC16;PTRH	brown	0.00410092	0.47644391	0.29565739	-0.0334545	-0.6116197	0.05520998
cg05014732	RP11-33I11.2	brown	0.15017091	-0.4293171	0.02725488	0.24104647	0.82854713	0.0576387
cg10823980	RAPGEF1;RAPGEF1	brown	-0.0562647	0.55719099	0.15966946	0.06789224	-0.6996112	0.02331869
cg19878838	FAM47E;FAM47E;SCARB2	brown	-0.3306917	-0.5810199	-0.0450577	-0.0734942	0.57909962	-0.2168023
cg09684262	TIPARP-AS1;TIPARP-	brown	-0.110739	0.32443571	0.09753578	-0.0893015	-0.6180297	-0.0568015
cg18356679	HEY2;RP11-624M8.1;RP11-	green	0.05654052	0.19280791	-0.560046	-0.1638624	-0.1732653	-0.2367617
cg00730266	PPP1R9A;PPP1R9A;PPP1R	green	0.23267901	0.16365681	-0.4881093	-0.1046517	-0.0206276	-0.0531634
cg06207823	CD36;CD36;CD36;CD36;CD	green	0.38192862	0.57040358	-0.6269541	-0.0635709	-0.3770503	-0.19179
cg09916684	GCNT2;GCNT2;GCNT2;GC	green	0.59706987	0.57517807	-0.4215995	0.05483466	-0.1707316	0.01813844
cg09355232	SNORD56;ITGB8;ITGB8;IT	green	0.51940395	0.47949496	-0.6057215	0.011776	-0.1631799	-0.0674971
cg03094728	AKAP9;AKAP9;AKAP9	green	0.21849381	0.1021995	-0.5738459	-0.0570381	0.04382773	-0.1867473
cg10207609	CD36;CD36;CD36;CD36;CD	green	0.29965337	0.2044934	-0.4142417	-0.2054276	-0.0066573	-0.1297616
cg13383842	SECTM1	green	-0.1113433	-0.0395812	0.46281805	0.15634209	0.08285033	0.20217834
cg25939954	TBC1D4	green	0.56120589	0.58695117	-0.4519472	0.12184098	-0.2393205	-0.043084
cg07691874	SERPINB13;SERPINB13;SE	green	0.50215057	0.49226158	-0.5302844	0.11278482	-0.1573558	0.04016652
cg04346128	KCTD16;KCTD16	green	-0.2522517	-0.1859888	0.34901835	0.16511441	0.14031461	0.1551171
cg20844118	LETMD1;LETMD1;LETMD	green	0.47057709	0.40570615	-0.5470906	0.05968733	-0.1084024	0.00071791
ch.4.12604R	PIGG;PIGG;PIGG;PIGG;PI	green	0.16976744	0.24269916	-0.4254958	-0.1360134	-0.1983705	-0.1568462
cg06017028	PGGT1B;PGGT1B;PGGT1B	green	0.22200546	0.20875544	-0.4958641	-0.1241152	-0.1295766	-0.0868386
cg10185299	SAMM50;SAMM50;SAMM	green	0.51266714	0.47269184	-0.546932	0.02721714	-0.1475026	0.04007021
cg10289177	ASIC3;ASIC3	green	-0.0141283	-0.0006735	0.32989692	0.26513445	0.05467245	0.2712091
cg08082792	FBRSL1;FBRSL1;FBRSL1;F	green	-0.1076536	-0.1582694	0.61896794	0.15084561	0.10565688	0.21263342
cg00361146	SLC7A11	green	-0.0060763	0.14862655	-0.7285998	-0.1560434	-0.1611427	-0.3772666
cg21124196	PRRX1;PRRX1;PRRX1;PRR	green	0.14845609	0.20370189	-0.7021923	-0.0746928	-0.0087345	-0.2220517
cg03954573	FAM50B;FAM50B;RP11-	green	0.0147381	-0.1264254	0.57442134	0.22129061	0.16105418	0.42686862
cg22233082	MIR627;VPS39;VPS39	green	0.49617063	0.31114188	-0.5265056	0.02113242	0.01473108	-0.0424086
cg17447147	CENPC1;CENPC1;CENPC1	green	-0.0013804	0.15388595	-0.4875743	-0.1980556	-0.2034997	-0.1985834

cg17157592	DENND1A;DENND1A;DE	green	0.4163665	0.32843176	-0.5885742	0.02478346	-0.0719793	0.00495735
cg07011006	GPD1;GPD1;GPD1;GPD1	green	0.04069815	-0.1755454	0.59952517	0.46576291	0.32152027	0.3658081
cg17969427	RP11-437J19.1	green	0.49120434	0.50633064	-0.4473933	0.14189145	-0.2417404	0.0732434
cg10129944	PHACTR1;PHACTR1;PHA	green	0.14430139	0.15144638	-0.4924957	-0.1282762	-0.065298	-0.1237391
cg04122324	NUFIP1;KIAA1704;KIAA17	green	-0.5489254	-0.4935002	0.5827861	-0.0116262	0.16598365	-0.1506876
cg07333510	ENO3;ENO3;ENO3;ENO3;E	green	-0.0146769	-0.2807193	0.64386838	0.38076443	0.49053238	0.267986
cg22278522	GPR56;GPR56;GPR56;GPR	green	-0.1910385	-0.0763598	0.46685494	0.28267868	0.1409397	0.26376937
cg17020695	RP11-31F15.1;RP11-	green	0.28436417	0.1357621	-0.3454892	-0.0162604	0.01224894	0.08496686
cg07380086	CHN1;CHN1;CHN1;CHN1	green	0.49100321	0.4171432	-0.5013827	-0.0045765	-0.1492382	-0.0939079
cg17144305	PCNXL2	green	0.51207235	0.39212737	-0.6097719	0.06799507	-0.0055494	-0.0505227
cg20528127	C11orf36;C11orf36;C11orf36	green	-0.0883952	-0.0314672	0.48565872	0.09932965	0.10594703	0.22344537
cg04842116	BMPER	green	0.44950272	0.59571732	-0.6143216	-0.0669367	-0.2986626	-0.0477415
cg25475056	COX6B1;COX6B1	green	0.02413899	0.10632625	-0.4907562	-0.0318844	-0.1635524	-0.0939737
cg19713057	HRASLS	green	-0.0209614	0.02080297	-0.6301929	-0.1131287	0.03903655	-0.3237801
cg12815829	FAM107B;FAM107B;FAM1	green	0.15439354	0.1281907	-0.6440524	0.05142165	-0.0016046	-0.2391146
cg15848147	USP53;USP53;USP53;USP5	green	0.46998214	0.39624381	-0.5379432	0.04826811	-0.0834392	-0.0615055
cg01186006	MEF2C	green	0.13493536	-0.0585209	0.82935147	0.37243296	0.22490556	0.43311851
cg05016698	EPN2-	green	0.12717886	0.18974414	0.62538272	0.54541188	-0.004863	0.32602153
cg06693488	KCNK7;KCNK7	green	0.08577029	-0.0441428	0.51872883	0.32054207	0.24312928	0.41054914
cg07036465	LRP6;LRP6	green	0.48467832	0.45752237	-0.4591624	0.10220529	-0.1077983	0.01031014
cg18484488	MIR380;MIR299	green	0.44610981	0.45295961	-0.4214253	0.04685201	-0.117874	0.06156394
cg21499782	ST7L;ST7L;ST7L;ST7L;ST7	green	0.11926665	0.15132233	-0.5733378	-0.1459018	-0.0701344	-0.2262706
cg14142550	AC005301.8	green	0.43656656	0.38689854	-0.1161027	-0.0248444	-0.2994545	0.25374901
cg00027729	ITGAM	green	0.45251036	0.40774782	-0.585751	0.05571557	-0.1155597	-0.0209455
cg10800960	RP11-2E17.1	green	0.11068503	0.16182021	-0.7076556	-0.0950172	-0.0258376	-0.3036826
cg24337863	RP11-256L11.1	green	0.35195704	0.338687	-0.6086865	-0.0739223	-0.1033814	-0.0581458
cg23239588	RP1-	green	-0.1118458	-0.1230249	0.72692639	0.1548525	0.06266757	0.28094918
cg19476312	NREP;NREP;NREP;NREP;N	green	0.07740436	-0.3019378	0.70882679	0.27498612	0.41898173	0.34283163
cg05379675	ELAVL2;ELAVL2;ELAVL2;	green	0.23571572	0.06800499	-0.4021798	-0.1668676	-0.0092133	-0.0054041
cg27511413	ASGR1;ASGR1;ASGR1;AS	green	-0.0478745	0.0218164	0.52050452	0.2222952	0.20680269	0.14172185
cg12677723	MIR1470;WIZ	green	-0.1451751	-0.1577384	0.61269367	0.24123392	0.25861456	0.10368814
cg09734798	STAP1	green	0.04106009	-0.2583668	0.84468078	0.23491066	0.34233726	0.44461393
cg26618989	MRPS30;RP11-	green	0.03721962	0.1684098	-0.4647267	-0.145224	-0.1084648	-0.1236381
cg08743715	CEACAM1;CEACAM1;CEA	green	-0.1370952	-0.0681039	0.68067807	0.29535989	0.05711212	0.25447366
cg04106489	WSB2;WSB2;WSB2;WSB2;	green	0.12653147	0.22134276	-0.4753869	-0.0309458	-0.0493447	-0.3874398

cg27542454	HNRNPA1;RP11-968A15.8	green	0.19996279	0.17249975	-0.6469326	0.07722958	0.12099196	-0.168307
cg25994584	C4orf22;C4orf22;C4orf22;C4	green	0.18623144	0.21526512	-0.8051748	-0.0879118	-0.1371279	-0.3006618
cg24685761	TMEM79;TMEM79;TMEM7	green	0.15280777	0.23544661	-0.4917865	-0.0322173	-0.0761446	-0.010881
cg09526889	SIAH2-AS1	green	0.15305968	0.11107619	-0.4163718	-0.0710645	-0.0217517	-0.1401747
cg22367004	RP11-	green	0.65956833	0.55556622	-0.3940487	0.16322352	-0.1809002	0.14409547
cg06609282	U6	green	0.13888759	0.0741468	-0.7700664	-0.0827739	0.03736312	-0.2483833
cg17775713	TF;TF;TF;TF;TF;TF;TFP1	green	-0.3046374	-0.2752647	0.45222631	0.10066833	0.16641477	0.13214433
cg13344757	ALDOC;ALDOC;ALDOC;A	green	-0.0176072	-0.1268261	0.51183493	0.25053065	0.38351665	0.22246818
cg21594918	DEGS2;DEGS2	green	-0.1668772	-0.0304933	0.51691864	0.21350118	0.07638528	0.13927148
cg02284258	AC132068.1;ZFHX3;ZFHX3	green	-0.0537132	0.14600327	0.49924163	0.20165427	-0.0640096	0.15626867
cg23186959	MEIS2;MEIS2;MEIS2;MEIS	green	0.1641113	0.13539978	-0.737301	-0.0775796	0.0487805	-0.2995602
cg27220549	SEPT2;SEPT2	green	0.45175744	0.39550598	-0.5427697	0.05786551	-0.1176479	-0.0161972
cg17447791	ULK2;ULK2	green	0.31595472	0.30291241	-0.6784949	-0.0498072	-0.0659695	-0.180844
cg07511070	KIRREL3;KIRREL3;KIRRE	green	-0.0933662	-0.0082847	0.34988493	0.23967995	0.1274051	0.11827736
cg08440736	POFUT1;RP11-392M18.5	green	0.46967301	0.3370577	-0.4882067	0.05589512	0.0074181	-0.0155489
cg02864225	TBR1;TBR1;TBR1	green	0.21106487	0.01450316	-0.4230602	-0.1085678	0.06086186	-0.1352311
cg10071662	GPHN;GPHN;GPHN;GPHN	green	0.14166114	0.09696765	-0.6519153	-0.1223255	-0.0164373	-0.0560975
cg03214303	C19orf66;C19orf66	green	-0.1414464	0.01453764	0.50412772	0.27777081	-0.0191797	0.18682015
cg25195522	RP11-519G16.3;RP11-	green	0.30636492	0.3680519	-0.5630776	-0.0596178	-0.1697837	-0.2900269
cg09330012	EHBP1;EHBP1	green	-0.022121	-0.1159059	0.73304732	0.1810179	0.12590589	0.25012673
cg08116386	TXNDC15;TXNDC15;TXN	green	0.39163127	0.35658378	-0.6929878	0.02644409	-0.0609544	-0.1263411
cg24327813	AP001024.1	green	0.0063934	-0.1300698	0.50799177	0.19138553	0.13009396	0.50175074
cg23704802	CTD-2284J15.1;CTD-	green	0.19300919	0.1794201	-0.4946414	-0.1742346	-0.1340009	-0.119451
cg26766272	SGK1;SGK1;SGK1;SGK1;S	green	0.06995761	0.26577451	-0.7639362	-0.120819	-0.1922591	-0.3534642
cg25377313	GALC;GALC;GALC;GALC;	green	0.00363193	-0.1196457	0.50286463	0.41573081	0.23653697	0.1535464
cg12445693	LMNB1;LMNB1;LMNB1;L	green	0.14614937	0.21457554	-0.3632915	-0.0801023	-0.0350662	-0.1348102
cg06920962	PLD4;PLD4;PLD4;PLD4	green	-0.1162829	0.08280026	0.52096457	0.28527164	-0.0225515	0.21609981
cg16678283	ARL3;SFXN2;SFXN2	green	-0.1575013	-0.1248481	0.53748134	0.07169737	0.09768569	0.45436279
cg09459339	CD96;CD96;CD96;CD96;CD	green	0.09782342	0.13659311	-0.4225644	-0.1422556	-0.1178411	-0.152398
cg23867562	BEST2;BEST2;BEST2	green	-0.2627767	-0.0195892	0.493663	0.22740932	-0.0698238	0.1195639
cg20264568	PTPRB;PTPRB;PTPRB;PTP	green	-0.2084127	-0.0685562	0.43598221	0.1143771	0.05947939	0.00537778
cg14439324	UFM1;UFM1;UFM1;UFM1;	green	0.1374057	0.3209186	-0.5167493	-0.1198317	-0.2867072	-0.2430131
cg26526503	C7orf53;C7orf53;C7orf53	green	0.49527302	0.3692254	-0.5417958	-2.53E-05	-0.0500955	0.06749471
cg01754828	BAI3;RP3-525N10.2	green	0.21838606	0.19995278	-0.6494573	-0.1321332	-0.0529128	-0.2357686
cg10150125	U6	green	-0.1773204	-0.2153307	0.8586175	0.1554747	0.1056906	0.24099991

cg15102770	ADAMTS3;ADAMTS3	green	-0.0332587	0.01047559	-0.3832606	-0.1207151	0.03191059	-0.4022066
cg15748240	C1orf173;C1orf173;RP11-	green	-0.0752981	-0.1596786	0.71699149	0.20811001	0.15795906	0.08321033
cg23697780	CYP7B1	green	0.46430116	0.52207013	-0.5008826	0.08285001	-0.1841728	-0.006098
cg11875994	GLYCTK-AS1;GLYCTK-	green	-0.0317676	-0.2960182	0.75981454	0.33298379	0.40614161	0.27257207
cg13528778	U4	green	0.44111627	0.484705	-0.5957159	0.01383051	-0.1592596	-0.1404265
cg00645755	INTS1;INTS1;INTS1	green	-0.151886	-0.0593701	0.32811075	0.12073834	0.05993924	0.21478887
cg14448515	SCIN;SCIN;SCIN	green	0.57875768	0.4841423	-0.5115511	0.17203786	-0.0533795	0.04800193
cg25361092	WWTR1;WWTR1	green	-0.0856358	-0.2886683	0.78670092	0.12618323	0.20295027	0.22154035
cg16325378	WT1-AS;WT1-AS;WT1-	green	0.04440133	-0.0419161	-0.3490106	-0.0730937	0.02443311	0.11225902
cg05020125	RP11-863K10.4;ERLIN2	green	-0.0096214	-0.0931565	0.41918995	0.23409598	0.15934728	0.39007922
cg06772933	RP11-352D3.2	green	0.03315877	-0.2900131	0.73600577	0.21071652	0.44691704	0.35689809
cg08738600	RUVBL1;EEFSEC	green	0.03752459	0.01564955	-0.357533	-0.1859463	-0.0231367	-0.1619373
cg09379151	GPR18;GPR18;GPR18;UBA	green	0.27599714	0.32163908	-0.497295	-0.0571326	-0.179955	-0.0971167
cg01008011	RP11-3B12.1;RP11-	green	0.08288404	0.3075824	-0.5974087	-0.2008782	-0.2367873	-0.262068
cg20945738	TNFSF13B;TNFSF13B;TNF	green	0.16873731	0.18201968	-0.5720394	-0.1397663	-0.0258085	-0.2124793
cg06624369	RP11-4O1.2;RP11-4O1.2	green	0.09213365	0.24810311	-0.6257693	-0.2148211	-0.2028734	-0.3045024
cg05839875	FYB;FYB;FYB;FYB;FYB;F	green	0.10944599	0.20122977	-0.5303494	-0.0311238	-0.1793564	-0.1952412
cg14016200	ICAM4;ICAM4;ICAM4;ICA	green	-0.1002915	-0.1373663	0.35216409	0.14687082	0.11139062	0.21624065
cg22490405	MEIS1;MEIS1;MEIS1;MEIS	green	0.25735103	-0.0103758	-0.6252197	0.03545164	0.23846192	-0.1044842
cg22759096	ATAT1;ATAT1;ATAT1;AT	green	-0.0894682	-0.160927	0.57173115	0.47451084	0.32902921	0.32500144
cg03697708	CACNA1A;CACNA1A;CA	green	0.16058878	0.21453942	-0.5628632	-0.0415637	-0.0879682	-0.2041989
cg09830360	ARHGAP42	green	0.44912308	0.43963647	-0.6336779	0.00772199	-0.0739944	0.01582496
cg26247335	PCDHA7	green	0.15050592	0.160482	-0.5835402	-0.064494	-0.0785333	-0.2813243
cg17783509	PHOX2B;RP11-	green	-0.3847129	-0.310835	0.45978013	0.0675713	0.14431006	0.02059461
cg20327862	C8orf47;C8orf47	green	-0.2610262	-0.2070798	0.35431316	0.06196354	0.1149335	0.16131533
cg14093610	CCDC140	green	-0.148311	-0.1778314	-0.40521	-0.1801399	0.13009736	-0.2061359
cg14462572	RP1-65J11.1	green	0.09244904	0.09762849	0.56820855	0.15517589	0.04654116	0.20713431
cg23063036	NDST3;NDST3;NDST3	green	0.19282263	0.14727026	-0.381892	-0.0501524	-0.0536999	-0.0637662
cg01485196	CNOT8;CNOT8;CNOT8;CN	green	0.18585476	0.14310951	-0.4615306	-0.0401226	0.01452104	-0.0082004
cg02056924	AC006557.2	green	-0.1374045	-0.2765928	0.73297808	0.00966587	0.12467676	0.26989501
cg15225701	FER;FER;FER;FER;FER	green	0.1392481	0.16573776	-0.5055855	-0.1603712	-0.1800211	0.0269338
cg11182225	TAC1;TAC1;TAC1	green	0.11551647	0.22906386	-0.6708115	-0.1193043	-0.1173114	-0.1581905
cg27168398	DET1;DET1;DET1;DET1;DE	green	0.35902866	0.26690641	-0.629422	-0.0144801	-0.0238745	0.04263876
cg23835083	FAM50B;FAM50B;RP11-	green	0.05560979	-0.04323	0.45632725	0.18178135	0.16433927	0.26139813
cg27281549	RAD51C;RAD51C;RAD51C	green	0.33760829	0.23168198	-0.5227856	0.01985334	0.05093151	-0.1620662

cg03739354	RP11-338C15.3	green	-0.0122922	0.13529263	-0.7488703	-0.2114297	-0.1294293	-0.3925915
cg26889241	RP13-44L19.2;MANEA	green	0.0779264	0.22851591	-0.6690938	-0.3249092	-0.2194968	-0.3093398
cg02493167	OTP	green	0.11346556	-0.0640941	-0.4189214	-0.1677374	0.13835395	-0.1113325
cg25782610	RAD23B;RAD23B;RAD23B	green	-0.1298461	0.0141778	-0.6186124	-0.2041811	-0.0990289	-0.3586071
cg04355791	MIR129-	green	0.14642617	0.16059943	-0.3318055	0.04004941	-0.0093099	-0.1113103
cg08786026	HEY2;RP11-624M8.1;RP11-	green	-0.0958627	0.095234	-0.6742688	-0.258852	-0.1159637	-0.1896506
cg25450482	PDCD2L	green	0.09169638	0.33554637	-0.4767092	-0.1677348	-0.2584074	-0.3406236
cg26577002	XRCC4;XRCC4;XRCC4;XR	green	0.08708491	0.06684943	-0.3629623	-0.0336447	0.02261584	-0.0046638
cg00154652	U3	green	-0.2013656	-0.0626501	0.31764097	0.0495999	0.04512385	0.07862917
cg11777890	RNF26	green	0.08024202	0.02861873	-0.3362399	-0.1135475	-0.0006602	-0.0004543
cg03658244	MAPRE2;MAPRE2;MAPRE	green	0.10956002	0.2072247	-0.617984	-0.0307291	-0.0602067	-0.0365348
cg09300914	OXSM;OXSM;OXSM;OXS	green	-0.0207817	0.121707	-0.3983709	-0.2672146	-0.1558891	-0.3312051
cg26557993	EXOSC9;EXOSC9;EXOSC9	green	0.00388843	-0.0627732	0.80970386	0.22154745	0.03476437	0.36213108
cg17609028	PCDHB3	green	0.1907018	0.11302048	-0.5817421	0.02163272	0.03468359	-0.0750677
cg18159478	RP4-754E20__A.5	green	0.02247949	-0.0992724	0.732846	0.24422502	0.16009233	0.24360567
cg09385414	UTP20;UTP20	green	-0.0191889	0.08807098	-0.4993619	-0.2317851	-0.1566202	-0.3123426
cg11964154	LBR;LBR;LBR;LBR	green	0.22735091	0.18048697	-0.4848174	-0.0408259	0.00248332	-0.087933
cg19641667	LRRC72;LRRC72;LRRC72;S	green	-0.0307338	0.08210806	-0.6572958	-0.2132716	-0.0097328	-0.3179725
cg27303409	PJA2;PJA2;PJA2	green	0.27217513	0.23777989	-0.4019941	-0.0306213	-0.0770293	0.02110359
cg00286510	DBF4;SLC25A40;SLC25A40	green	0.19938938	0.30403545	-0.6553	-0.1989551	-0.1307685	-0.2148625
cg02529547	HILS1	green	-0.2062247	-0.1135988	0.42206425	0.36823017	0.12294494	0.19370533
cg02079236	LYVE1;MRVI1;MRVI1;MR	green	0.21200773	0.17845839	-0.5976242	-0.0064665	0.13937738	-0.2058174
cg01289020	FTSJD1;FTSJD1;FTSJD1;FT	green	0.09224262	0.10828431	-0.4579859	-0.1316484	-0.0616152	-0.1814205
cg14273320	PABPN1;PABPN1;PABPN1	green	0.02493131	-0.2800025	0.75744413	0.21867214	0.33701934	0.29290577
cg23353945	RP4-541C22.5	green	-0.122782	0.00197244	0.49675908	0.27828918	0.02118359	0.1410111
cg01810137	RBM19;RBM19	green	0.14236918	0.19824414	0.37776866	0.37553097	0.10536658	0.25600642
cg18430295	NRN1	green	0.17248093	0.25673303	-0.3982391	-0.0571657	-0.216282	-0.0797619
cg16567044	MEG3;MEG3;MEG3;MEG3;	green	-0.226214	-0.0466583	0.53006301	0.14738398	0.02005116	0.13045016
cg19297726	NRXN1;NRXN1;NRXN1	green	0.18150168	0.18604729	-0.6175543	-0.0855904	-0.0130153	-0.1486066
cg23092714	CRYBA2;CRYBA2	green	0.02317901	-0.0156603	0.39633798	0.27789338	0.12805463	0.27902943
cg22772528	RP11-335O4.1	green	0.14772966	0.06056453	-0.5454284	0.07635901	-0.0244922	-0.1875134
cg11522654	PEX3;PEX3;PEX3	green	0.06625139	0.14962306	-0.6991894	-0.185225	-0.1139234	-0.2681863
cg18820312	CTD-2341M24.1;CTD-	green	-0.0052065	-0.1151536	0.81384478	0.21510703	0.02243886	0.33968059
cg08936487	AL445309.1;TUBB2B;TUBB	green	0.05062407	-0.0578152	0.78835718	0.1778251	0.03409943	0.29127374
cg16364095	CYP26B1;CYP26B1;CYP26	green	-0.2565763	-0.244329	0.36566882	0.09383207	0.10734309	-0.0100869

cg13677416	TESC;TESC	green	-0.1196418	-0.1153268	0.72278282	0.2404808	0.18394796	0.10564079
cg22710189	RP11-2G1.1	green	0.04172091	-0.0979309	0.78216727	0.16994617	0.13364875	0.35711942
cg05316703	PLEKHB2;PLEKHB2;PLEK	green	0.03078601	0.03291643	-0.4174763	-0.1659759	-0.0547535	-0.087269
cg20439103	ABCE1;ABCE1;ABCE1;AN	green	0.10214552	0.19511619	-0.7877858	-0.190672	-0.1039624	-0.3689551
cg12797413	THRA;THRA	green	0.24666729	0.05277375	0.72919593	0.37089992	0.13329335	0.45677138
cg09229115	RP11-51B13.1	green	-0.117816	0.02570187	0.77316212	0.08457245	-0.0819706	0.04031839
cg12185681	HSF2;HSF2	green	-0.0221817	-0.0804207	0.70680488	0.2067732	0.00223993	0.24903421
cg25519099	PSMD7;PSMD7;PSMD7;PS	green	-0.1557204	-0.0326048	-0.4681357	-0.2281737	-0.0076157	-0.4455152
cg17164498	SPINK4	green	0.06392911	-0.1300651	0.78252843	0.18163559	0.13356596	0.27634202
cg25934800	RP11-436F21.1	green	0.11357839	0.03047963	0.8052959	0.27311649	0.08545941	0.36344341
cg10647101	TBX1	green	-0.2317594	-0.1195741	0.32094862	0.04792621	0.04609806	-0.0086378
cg23658739	AP001628.6	green	-0.0833803	-0.0148325	0.39011647	0.20215478	0.09435962	0.13066413
cg18656763	FAM50B;FAM50B;RP11-	green	-0.046633	-0.1556274	0.38364851	0.12494757	0.20383074	0.09173533
cg04365308	TAS2R42	green	0.01644261	0.04939367	0.73220153	0.28090797	0.04394385	0.26880273
cg07993132	ZC3H15;ZC3H15;ZC3H15;Z	green	0.16138608	0.08395077	-0.328954	-0.181704	0.00605328	0.00013266
cg15846488	TCF4;TCF4;TCF4;TCF4;TCF	green	0.26368372	0.18514108	-0.3611291	0.03143787	0.05578906	-0.1529728
cg19554304	RIN1;RIN1;RIN1;RIN1;RP1	green	-0.0434038	-0.0299879	0.66965564	0.25219266	0.11235065	0.20604378
cg14365081	KLHDC3;KLHDC3;KLHDC	green	-0.0456689	0.10054694	0.62803465	0.31494713	0.06439455	0.33287686
cg02135304	MEF2C	green	0.11958373	0.10543476	-0.638897	-0.13028	-0.0209427	-0.1105324
cg14525832	SLIRP;ALKBH1;ALKBH1;	green	-0.0354886	0.0228633	-0.6070351	-0.1842536	-0.0323454	-0.4094673
cg04209949	TBC1D5;TBC1D5;TBC1D5;	green	0.00897553	-0.2575398	0.84880087	0.28804771	0.25351714	0.37240306
cg10898833	CD36	green	-0.0458057	-0.0854821	0.88344355	0.1469041	0.03215093	0.31642015
cg02808051	SCN2A;SCN2A;SCN2A;SC	green	0.17641149	-0.0961262	0.52366649	0.24612564	0.28952705	0.37402092
cg22072966	RP11-260O18.1;RP11-	green	0.07132984	-0.1051338	0.8407258	0.19167656	0.1230157	0.35945199
cg09277389	IFT57;IFT57;IFT57;IFT57	green	0.08022125	0.25134083	-0.6520838	-0.1209464	-0.1459687	-0.3090454
cg11533763	WAPAL;WAPAL;WAPAL	green	0.02176067	-0.111135	0.69938082	0.16688636	0.16006658	0.16741818
cg22079042	SDR39U1;SDR39U1;SDR39	green	-0.026441	0.07882148	-0.4027943	-0.1846453	-0.1307292	-0.1860865
cg24655284	ADAMTS6;ADAMTS6;AD	green	-0.0658356	0.02915473	-0.5416154	-0.0725104	-0.031228	-0.192139
cg08206898	FAM160B1;FAM160B1	green	0.30231606	0.22572341	-0.4395577	-0.0196623	-0.0587902	-0.0279318
cg14261863	ELMO2;ELMO2;ELMO2;EL	green	-0.038127	-0.0521971	-0.4782647	-0.0397313	0.14615796	-0.1903334
cg02470595	MIR615;HOXC5;HOXC5;H	green	0.22425296	0.10286461	-0.5159524	0.02178251	0.00419565	-0.103662
cg03069031	NKAIN1;NKAIN1;NKAIN1	green	-0.0773899	0.02469034	0.43909057	0.20654338	0.10999368	0.22037616
cg10702309	HES1;HES1	green	0.33537973	0.24744215	-0.5224608	-0.0124298	-0.0337242	0.16709568
cg09272879	RCHY1;RCHY1;RCHY1;RC	green	0.02516489	-0.2421468	0.84570592	0.32867444	0.32281142	0.31822817
cg06214728	U6atac;CHSY3	green	0.05504347	-0.0116064	0.85935454	0.16707359	0.04210028	0.39573011

cg01101647	EPHA5;EPHA5;EPHA5;EPH	green	0.04213792	0.12734269	-0.6524985	-0.0877974	-0.056861	-0.4677123
cg14672108	ST5;ST5;ST5;ST5;ST5;ST5;S	green	-0.0855459	0.07599609	0.47757029	0.15020113	0.13157844	0.18578761
cg09035349	OLIG1;OLIG1;AP000282.2	green	0.26386669	0.05917306	-0.4093901	0.01007107	0.16457821	0.01183726
cg27049123	Y_RNA	green	-0.0865831	-0.1471301	0.84707542	0.19641182	0.15211646	0.27662917
cg14775560	FOLH1;FOLH1;FOLH1;FOL	green	0.12891115	0.11947781	-0.4607068	-0.086754	-0.0163316	-0.2661784
cg14428911	SEMA6D;SEMA6D;SEMA6	green	0.26293741	0.11967118	-0.5955583	-0.0126235	0.01916744	0.13581766
cg07484813	MDM2;MDM2;MDM2;MD	green	0.11917367	0.13922445	-0.7692311	-0.153781	-0.0616397	-0.0488555
cg18197332	FAM50B;FAM50B;RP11-	green	0.14441364	0.01846461	0.30761564	0.26131835	0.14414505	0.15160862
cg20411884	RP11-661P17.1	green	0.20636643	0.09184664	-0.6207039	-0.1283481	0.00394486	-0.1614787
cg06875669	SAAL1;SAAL1	green	0.07073877	-0.1497522	0.80947165	0.12008142	0.10781644	0.3551836
cg14172596	PTPRF;PTPRF;PTPRF;PTPR	green	0.09157778	0.22721189	-0.5684272	-0.1489734	-0.1442855	-0.1027745
cg01285144	OR52A1;OR52A1	green	0.17277962	0.08648489	-0.3701007	0.09719226	0.07963143	-0.1598435
cg00164390	U6;OSGIN2;OSGIN2;OSGI	green	-0.0776548	-0.0089641	-0.7164299	-0.1853219	0.03030235	-0.3195983
cg26270094	RNY4	green	0.10310753	0.26022738	-0.787108	-0.1508745	-0.1620802	-0.279318
cg22755301	SCAF11;SCAF11;SCAF11	green	0.14451149	-0.114376	0.85064448	0.27858475	0.17330665	0.39053732
cg01655210	TAS2R1;CTD-	green	-0.0149293	-0.0823434	0.6903167	0.16598301	0.12346834	0.03802549
cg00669363	FNDC3A;FNDC3A;FNDC3	green	0.07820634	0.15522915	-0.8025556	-0.1552031	-0.105718	-0.3030691
cg26579171	GTPBP8;GTPBP8;GTPBP8;	green	0.08917212	0.04877664	-0.4379324	-0.1284265	-0.0660638	0.15900291
cg10149357	U6	green	0.36610319	0.26050195	-0.4790139	0.17199269	0.16648183	-0.014786
cg01247301	AC073071.1	green	-0.1130949	-0.1942157	0.71115314	0.08707963	0.12168969	0.06574229
cg19511605	USP7;USP7;USP7;USP7	green	-0.3676909	-0.2346173	0.46493145	0.27216297	0.09313679	0.07712946
cg08078832	RP11-572C21.1	green	-0.0349709	-0.1913862	0.78062039	0.16535974	0.17384088	0.19349939
cg04858318	FER;FER;FER;FER;FER	green	0.2347206	0.23839215	-0.5539768	-0.0667987	-0.1222866	0.25138043
cg15463457	MMRN1	green	0.10949405	0.14994698	-0.7120037	-0.0418471	-0.0302107	-0.2742673
cg06445316	MLLT10;MLLT10;MLLT10;	green	0.0925008	0.07438405	-0.3934059	-0.0915148	-0.0017281	-0.2159775
cg03016981	Z99129.1;SERINC1;PKIB	green	-0.1152537	-0.1954638	0.71183215	0.1332296	0.13321249	0.139355
cg23271234	VAX2	green	0.38464588	0.25827527	-0.4875386	0.03558489	-0.0553013	0.13106831
cg17155208	SLC9A9;SLC9A9;SLC9A9;S	green	0.22006294	0.20111424	-0.5983921	-0.1131736	-0.1378257	-0.1102929
cg02426940	NEIL1;NEIL1;MIR631;NEIL	green	0.11066458	-0.0888961	0.62972179	0.20562381	0.16261376	0.51169376
cg25529370	RP11-361H10.4;RP11-	green	-0.0250743	-0.0643538	0.7549128	0.17692246	0.08324301	0.17196167
cg20624787	AC104387.1;LRRC4C;LRRC	green	0.35651146	0.17463939	-0.4727641	0.06091475	0.14942775	0.020613
cg19234736	PCBP4;PCBP4;PCBP4;PCB	green	-0.0391943	0.08188764	0.61746727	0.28564256	0.15442116	0.26259284
cg17079352	NDUFB3;NDUFB3;NDUFB	green	-0.0356028	0.07037972	-0.3422511	-0.1239364	-0.0906289	-0.317474
cg19860663	FBXW8;FBXW8	green	-0.119005	0.15165949	-0.6206771	-0.2848477	-0.3188158	-0.3533089
cg23503584	IKZF2;IKZF2;IKZF2;IKZF2	green	-0.0476091	0.0178482	-0.6188208	-0.0771134	0.01262956	-0.2500112

cg10511110	MIR548P	green	-0.0058308	-0.1426866	0.88417321	0.23670394	0.17971539	0.20376576
cg09821214	FAM50B;FAM50B;RP11-	green	-0.1268566	-0.2057014	0.46302889	0.09611689	0.1294407	0.39552631
cg19852097	RP11-239A17.1	green	0.01080966	-0.0635497	0.89775426	0.20290478	0.03575829	0.2781376
cg06610641	ZNF527;ZNF527;ZNF527	green	-0.1581863	-0.0086013	-0.4884225	-0.232125	-0.0386103	-0.2116365
cg13245809	RP11-13N12.2	green	0.02550337	0.13025055	-0.6264793	-0.1159438	-0.1601825	-0.2774063
cg17367708	RP11-14O22.1	green	0.05675804	0.12219234	-0.7824486	-0.2144674	-0.0318077	-0.3826619
cg01970802	RP11-386O9.2;ZRANB2-	green	0.01883254	-0.1150342	0.75463304	0.10000427	0.15407534	0.26056942
cg09597335	PLEKHG5;PLEKHG5;PLEK	green	0.1188667	0.03263146	0.50077687	0.30388198	0.25384417	0.32778439
cg17747924	BATF;BATF;BATF	green	0.14398682	0.14820709	-0.3464223	-0.1405305	-0.1296616	-0.0568829
cg22703284	CPEB4	green	0.01922615	0.15418184	-0.6359951	-0.2597384	-0.1163266	-0.4012698
cg01051381	COPB1;COPB1;COPB1;CO	green	0.1030445	0.14713529	-0.4234694	-0.1001704	-0.0655849	-0.2553607
cg09992055	DAXX;DAXX;DAXX;DAX	green	0.05356927	0.15138901	-0.4509826	-0.0508431	-0.0846586	-0.0989468
cg19435206	LIG4;ABHD13	green	0.0630462	0.18704535	-0.7619479	-0.0986712	-0.0909211	-0.3631796
cg23051598	ATXN2L;RP11-	green	-0.1261666	-0.0610147	0.48209994	0.14176906	0.0672234	0.13145711
cg10100093	TRIM23;TRIM23;TRIM23;C	green	0.17525663	0.20099522	-0.628241	-0.066003	-0.0662998	-0.1242093
cg22896071	CROT;CROT	green	-8.48E-05	-0.0936564	0.71320866	0.12385129	0.11708001	0.24227651
cg12558755	SNX10;SNX10;SNX10;SNX	green	-0.027147	0.1070867	-0.7329016	-0.1719733	-0.0660019	-0.455256
cg18187762	COMMD6;COMMD6;COM	green	-0.0060507	0.04829051	-0.5867009	-0.2380651	-0.0860641	-0.1220757
cg06381665	ADAP1;ADAP1;ADAP1;A	green	0.29991358	0.07242978	0.57332274	0.38165715	0.19134776	0.51219765
cg22251614	FAM81B	green	-0.0252014	-0.2046534	0.64053436	0.06162158	0.18824538	0.18828367
cg04208803	CDK6;AC002454.1;AC0024	green	0.17072639	0.04756298	-0.7334141	-0.0596591	0.14395428	-0.0719821
cg10073231	SLAIN2;SLAIN2	green	0.16138976	0.00385974	0.72435441	0.18184241	0.0160996	0.35499649
cg14402950	AC105399.2	green	0.06284851	-0.0927135	0.86868914	0.19659327	0.13884788	0.40552236
cg10311324	RIMS2;RIMS2;RIMS2	green	0.00630772	-0.1557568	0.85787347	0.28170953	0.10986798	0.43362508
cg03383178	KCTD3;KCTD3	green	-0.0066441	-0.1850623	0.93203321	0.29086652	0.19096745	0.37674182
cg07474312	CTD-	green	0.14685613	0.10806265	-0.5852563	-0.0909716	-0.0943668	-0.0437886
cg12221623	RP11-132M7.1;TBX18;RP11-	green	0.41408888	0.31964225	-0.4376834	0.09824918	0.07757063	-0.0329539
cg27508071	C7orf26;C7orf26;AC079742.	green	0.10732203	0.00251297	0.55892331	0.33649204	0.19731585	0.34117621
cg08426604	RP11-521E5.1	green	-0.0357156	-0.0748323	0.86367754	0.21263962	0.13932302	0.18466741
cg19988790	RN5S117	green	0.01214512	-0.1111261	0.85275534	0.13084416	0.07820833	0.25439717
cg08687163	CTD-	green	0.24365101	-0.0109227	0.54914996	0.37561	0.36939812	0.3346765
cg01947523	RP11-443B7.1;RP11-	green	0.11462403	0.15425661	-0.6365217	-0.0441323	-0.0567428	-0.1889455
cg26066413	SNORA31	green	-0.0839046	-0.0984161	0.71713953	0.10552052	0.05080122	0.0136726
cg21676369	PCDHA12	green	-0.0345683	-0.1905549	0.77893288	0.22989922	0.15181797	0.28564228
cg23630813	MTRF1L;MTRF1L	green	0.23268726	0.03748916	-0.5778027	-0.0225002	0.16274722	-0.0511336

cg20517050	HOXA5;HOXA5;HOXA3;H	green	0.10891958	-0.036438	0.57628929	0.27865773	0.133348	0.16182805
cg06895885	SPAG1;SPAG1;SPAG1;SPA	green	-0.0003449	-0.1254468	0.87166517	0.17070554	0.0755644	0.25615363
cg07735220	AC007386.4	green	-0.0326379	0.09185646	-0.6915281	-0.1915795	-0.1151978	-0.1567107
cg16358438	THRAP3;THRAP3;THRAP3	green	-0.049078	3.86E-05	-0.3832533	-0.1544464	-0.1028682	-0.2871542
cg21325324	PDE7B	green	-0.0739275	-0.0299657	0.72069958	0.09947812	-0.0036176	0.04944069
cg04163687	ABR;ABR;ABR;ABR;ABR;	green	0.0388718	0.01868227	0.7037338	0.22373873	0.05371218	0.36333281
cg25132230	PTPRC;PTPRC;PTPRC;PTP	green	0.11491608	0.12181318	-0.4682155	-0.0972864	-0.1503854	-0.1924622
cg20801464	TGIF1;TGIF1;TGIF1;TGIF1;	green	0.05945897	0.11452185	-0.3762872	-0.1661927	-0.0896926	-0.172961
cg08963455	AGTR1;AGTR1;AGTR1	green	-0.0382213	-0.1474675	0.78878831	0.11040684	0.06733497	0.33188393
cg05530568	RP11-175K6.1;RP11-	green	0.08563226	0.12654428	-0.6764279	-0.1866901	-0.0377725	-0.3069814
cg03445103	PTPRR;PTPRR;PTPRR;PTP	green	-0.0047089	-0.048718	0.82306906	0.08483511	-0.0183757	0.35384438
cg25315624	PBX1;PBX1;PBX1;PBX1;P	green	-0.038581	0.04256958	-0.7031484	-0.1595072	0.01403962	-0.319656
cg01922269	AC005187.2	green	-0.0348189	-0.1935596	0.84472226	0.17435369	0.13707881	0.30867725
cg17590835	U4;NAALADL2	green	0.01913255	-0.1506306	0.82360284	0.18372799	0.19415691	0.26639332
cg16581032	U6;LINC00293	green	-0.3704311	-0.2353215	0.50010008	-0.0243609	-0.0555936	0.22086992
cg21414813	NBEA;NBEA	green	-0.2468525	-0.04231	0.4312042	0.02728552	-0.062757	-0.0321312
cg24431734	AGAP1-IT1	green	0.17127724	-0.0176397	0.69749738	0.33154742	0.10601107	0.38596973
cg18808765	OR5K4;RP11-325B23.2	green	-0.052161	-0.2130087	0.76205461	0.11575006	0.16811786	0.22944121
cg20778348	PDS5B;PDS5B;PDS5B;PDS	green	0.00421845	-0.0992668	-0.5476911	-0.0167509	0.2071252	-0.3289691
cg18424033	FLII;FLII;FLII;SMCR7;SMC	green	-0.0309301	0.04970262	0.40331592	0.31506172	0.07005864	0.10397432
cg25259085	SNORD98;CCAR1;CCAR1	green	-0.0472223	-0.1869485	0.84174266	0.13443654	0.11581018	0.28536651
cg11363040	SMARCC1;SMARCC1;SM	green	0.15621286	0.26055608	-0.6108229	-0.1323086	-0.1299256	-0.1965063
cg21185075	RP11-480G7.1	green	-0.0406372	-0.1934975	0.7702205	0.12910465	0.14260626	0.32800596
cg18295068	SCN3A;SCN3A;SCN3A;SC	green	0.02530343	0.12476704	-0.5703437	-0.157218	-0.0079689	-0.2603973
cg15112475	ZFAND2A;ZFAND2A;ZFA	green	0.05247549	-0.0312775	0.55560802	0.17203466	0.12819333	0.14779757
cg00200074	ORC5;ORC5;ORC5;ORC5	green	0.06897856	-0.0314315	-0.3369257	-0.0116511	0.06785571	0.00132709
cg24704593	MACC1;AC005083.1;AC005	green	0.01322492	-0.0172688	-0.4843335	-0.0884384	0.0715281	-0.078296
cg11841113	ADAMTS3;ADAMTS3	green	-0.0525712	0.03488869	-0.47724	-0.0427009	-0.0593643	-0.2826593
cg22084605	AC006150.1;DGKB;DGKB;	green	0.0812621	-0.1378062	0.90045903	0.26453944	0.13984004	0.47582282
cg25882643	MYEF2;MYEF2;MYEF2;M	green	0.01543368	-0.0503419	0.8867925	0.20771154	0.07842725	0.30252083
cg22078571	AMN;AMN	green	-0.1422044	-0.1741984	0.44520007	0.2275884	0.16884982	0.11136183
cg04962994	RP11-257A22.1	green	-0.0335458	-0.0975767	0.80414304	0.21002428	0.158998	0.47163058
cg24899978	PKD1;PKD1;PKD1;PKD1;P	green	0.10827253	0.01485477	0.58181068	0.38841471	0.2045499	0.27606807
cg16207673	ASCL4	green	0.13222254	0.22934303	-0.603825	-0.0518566	-0.1443277	-0.1117418
cg07118069	MAGOHB;MAGOHB;MAG	green	-0.1477222	0.01278016	-0.5918485	-0.1604756	-0.057271	-0.3501069

cg24101790	DPP10	green	0.04866498	-0.0592533	0.85886807	0.21036533	0.09070048	0.34409896
cg15379412	NR2F2;NR2F2;NR2F2;NR2	green	0.02363449	-0.1155748	-0.3408401	-0.1585623	0.08468215	-0.2670281
cg24456633	RUFY2;RUFY2;RUFY2;RU	green	0.19255612	0.21326014	-0.4922873	-0.0196877	-0.0454159	-0.0095418
cg18032908	AL583832.1;CNTN2;CNTN2	green	0.069201	0.09740687	0.49946942	0.2393635	0.07412512	0.38892941
cg09611363	SDAD1;SDAD1;SDAD1;SD	green	0.07588321	0.04014811	-0.5641068	-0.0779647	-0.0145889	0.06667278
cg19389852	TXNIP;TXNIP;TXNIP;TXNI	green	-0.0527672	0.05648432	-0.6691304	-0.2096558	-0.062051	-0.2206994
ch.2.2130426	C2orf29;C2orf29	green	0.00275873	0.19340687	-0.4573122	-0.156608	-0.1169724	-0.1805868
cg03542643	ZNF146;ZNF565;ZNF565	green	0.06688613	0.08296627	-0.3556626	-0.0761106	-0.003526	-0.0887753
cg08485488	AC006041.1	green	0.05935985	0.012149	0.82752836	0.16489695	0.0603493	0.27304404
cg18113332	B4GALNT4;B4GALNT4	green	0.12005329	-0.0533374	0.43664108	0.31054915	0.15941017	0.29879303
cg07870003	MTERFD3;MTERFD3;MTE	green	-0.0502656	0.05312711	-0.3583594	-0.1055149	-0.0889328	-0.0307441
cg26653400	AC007092.1;AC007092.1;A	green	0.07553513	0.04317651	-0.5204116	-0.019189	-0.0570674	-0.03089
cg00188206	TSPAN18;TSPAN18;RP11-	green	0.3931705	0.20828734	-0.4422874	0.13348672	0.21139967	0.05036751
cg26021597	CTC-	green	0.10814173	0.15355887	-0.7402841	-0.0474705	0.00072012	-0.2034001
cg05965693	DHRS1;DHRS1;DHRS1;DH	green	0.31442674	-0.0292446	0.56437844	0.24005109	0.19066039	0.59776367
cg11084449	AC012668.3;AC012668.2;A	green	-0.019471	-0.1597815	0.80154818	0.24425857	0.13568669	0.36194792
cg18661205	PCDHB18	green	0.31991092	0.0321193	-0.5496084	0.01897307	0.19384813	-0.0635898
cg09258911	MGA;MGA	green	-0.4789108	-0.4518763	0.52094431	-0.0477961	0.14058845	-0.1920084
cg14678786	PIK3C3;PIK3C3	green	0.10926424	0.24237161	-0.5634888	-0.0195209	-0.0772575	-0.212289
cg17018649	FSTL1;FSTL1;FSTL1;FSTL1	green	-0.0422075	0.04885123	0.55489888	0.2528336	0.00904134	0.36552224
cg13141983	U6	green	-0.0076122	-0.1628087	0.88769569	0.23722883	0.18498834	0.28508647
cg00219756	MACC1;MACC1;AC005083.	green	0.06440357	-0.0151821	-0.6284826	-0.0484951	0.10341719	-0.2905479
cg10217311	OR13C8	green	-0.098361	-0.1887451	0.64087972	0.09806742	0.09539277	0.16889022
cg26680132	TFAP2D	green	-0.0732006	0.04200332	-0.6448116	-0.1904585	-0.0975841	-0.254074
cg13115661	ASTN1;ASTN1;ASTN1;AST	green	0.01813923	0.14503952	-0.7968058	-0.244023	-0.1079086	-0.2338299
cg01947066	PML;PML	green	0.11795003	0.13218442	-0.5118483	-0.0205681	-0.0519257	-0.0982107
cg20528165	OR2T8	green	0.31235146	0.05362238	0.76348607	0.31879916	0.16533483	0.57317171
cg06690989	WAPAL;WAPAL;WAPAL	green	0.07559391	-0.0816068	0.74128972	0.22683827	0.16999436	0.34506411
cg10221617	HAS2-AS1;HAS2-	green	-0.2243742	-0.1837317	-0.4425483	-0.1357568	0.13274815	-0.2402689
cg18457264	IFLTD1;IFLTD1;IFLTD1;IF	green	0.00654525	-0.2501567	0.78430348	0.21699716	0.33550153	0.39596669
cg22063680	CLEC2B;RP11-	green	0.04249747	0.09931922	-0.4722678	-0.1488008	-0.0414288	-0.1210454
cg18107960	WDR41;WDR41;WDR41;W	green	0.07860779	0.07725153	-0.5404825	-0.0059857	0.05300668	-0.2523528
cg02069738	NBAS	green	-0.1663612	-0.2002346	0.31191412	0.11298888	0.08716453	0.117536
cg05694098	DHRS7B	green	0.13657088	0.15363049	-0.5098247	-0.1124144	0.00820786	-0.1824075
cg01437709	CHAC1	green	0.29874477	0.00952127	0.49258433	0.71119321	0.2737251	0.31135283

cg11299856	RN5S335	green	-0.0124815	-0.1164807	0.77764304	0.19520627	0.19697014	0.17351369
cg27395288	MAPRE1	green	-0.0127673	0.13903372	-0.5875678	-0.2570905	-0.096154	-0.3503099
cg13615966	U6	green	-0.0740308	-0.227511	0.80491039	0.11058289	0.19381638	0.36691889
cg13607699	UBTF;UBTF;UBTF;UBTF;U	green	-0.035962	-0.0586538	-0.3511955	-0.0935102	0.1334685	-0.176356
cg17191813	NAA60;NAA60;NAA60;NA	green	0.10577156	-0.122333	0.56102457	0.34520247	0.31203699	0.31093893
cg03222868	KCNH3	green	-0.0689158	-0.0826893	0.46419122	0.27100881	0.0886519	0.17851868
cg07638358	AC093171.1	green	0.06377554	0.10719769	-0.6855073	-0.1766405	-0.0539339	-0.2244086
cg19543005	C8orf83;C8orf83;C8orf83;C8	green	0.07407965	0.17529784	-0.7438925	-0.109541	-0.0573563	-0.4067322
cg04043517	CCDC112;CCDC112	green	-0.003676	-0.1502069	0.8146048	0.17674769	0.19172327	0.28057085
cg04758387	TMEM52;TMEM52;TMEM5	green	-0.0164591	-0.2217091	0.55686138	0.28689639	0.29697406	0.23335734
cg01912330	KBTBD6	green	-0.0471441	0.05505466	-0.6566241	-0.150778	0.04596556	-0.4518585
cg18054408	AK5;AK5;AK5;AK5	green	0.05718008	-0.0340423	0.86948977	0.26262871	0.07030788	0.27313841
cg17427102	RBM27;RBM27	green	0.10451892	0.23674099	-0.6468081	-0.0774447	-0.0352149	-0.3084612
cg06730718	PAPD5;SNORA70;PAPD5	green	-0.2702759	-0.0262662	-0.4291328	-0.3095687	-0.1338083	-0.1870968
cg24074685	TIA1;TIA1;TIA1;TIA1;C2orf	green	0.00538882	-0.0065793	-0.5421629	-0.1352423	0.06641924	-0.2371384
cg00705908	ASAH1;ASAH1;ASAH1;AS	green	0.04327712	0.10445817	-0.4761223	-0.0124026	-0.0349173	-0.163365
cg14356946	EIF2S2	green	0.08025254	0.17215177	-0.3831354	-0.1117647	-0.1244458	-0.114485
cg19379244	HHLA1	green	-0.0099472	-0.0948911	0.58321599	0.18812557	0.01853334	0.45962985
cg21476388	RP11-269F21.1	green	0.07481549	-0.1274812	0.79229929	0.31430049	0.33282462	0.34751136
cg18407966	CASP9;CASP9;CASP9;CAS	green	0.1973127	0.25421338	-0.2943911	-0.041649	-0.1264952	0.00250218
cg20679767	RP11-725D20.1	green	0.03992455	-0.1721615	0.91521018	0.21676997	0.19654177	0.48188359
cg21573611	GTPBP10;GTPBP10;GTPBP	green	-0.0648932	0.0921102	-0.6917066	-0.2044934	-0.1179108	-0.3445514
cg14653548	ATXN7L3B	green	0.10301788	0.13191076	-0.3187339	-0.0654058	-0.0937794	-0.0935444
cg24347838	CCDC39;CCDC39;CCDC39-	green	0.06873893	0.02858289	-0.6771045	-0.1143308	0.08777641	-0.0046784
cg11488290	PTPRR;PTPRR;PTPRR;PTP	green	-0.0377759	0.0045282	0.83097707	0.16436185	-0.0503603	0.19442127
cg05000960	C8orf59;C8orf59;C8orf59;C8	green	0.07165523	0.11029903	-0.5873249	-0.1138816	-0.0743325	-0.1782975
cg21813112	TRIM33	green	-0.1582456	0.05776254	-0.3830471	-0.1212555	-0.1278814	-0.3075788
cg20012224	RP11-1B20.1;CDC42BPA	green	0.05488737	-0.0859507	0.88365879	0.24809815	0.08948089	0.48513481
cg13403615	LMAN1	green	-0.2827655	-0.0774775	-0.731353	-0.2814109	-0.0310393	-0.5017388
cg12062611	TMCC1;TMCC1;TMCC1;T	green	0.08402118	-0.1119896	0.74008332	0.12604534	0.10886649	0.22524308
cg20478264	HIST1H2BM;HIST1H2AJ	green	-0.0266506	0.09077381	-0.4570284	-0.0637072	-0.0401159	-0.4354846
cg19071452	NMI;NMI;NMI	green	0.14436272	0.05205249	-0.3815536	-0.1189754	0.0819799	-0.056508
cg11086820	IRF1;IRF1;IRF1;IRF1;IRF1;I	green	-0.0691122	0.18491781	-0.6131315	-0.2600849	-0.2140601	-0.2014808
cg20478181	TFB1M;TFB1M;TFB1M;TF	green	0.12519628	0.20073979	-0.6358681	-0.1536883	-0.0985227	-0.1772436
cg03011941	CFDP1;CFDP1;CFDP1;CFD	green	0.01621325	-0.0805892	0.36129081	0.28051829	0.13119647	0.33895549

cg04936872	SNORA75;RP11-	green	-0.0320722	-0.1440566	0.7167823	0.20406871	0.13129187	0.34462947
cg20569839	TAF13;TAF13	green	-0.2686832	-0.2132684	0.38954154	-0.0176537	0.1240498	-0.0330659
cg06723404	PREPL;PREPL;PREPL;PREP	green	-0.2238026	0.01519032	-0.7828299	-0.194057	-0.08261	-0.4671706
cg01371498	SHC4	green	-0.1461781	-0.0479798	-0.4887657	-0.1786283	-0.0121945	-0.0975547
cg25634234	CA1;CA1;CA1;CA1;CA1;C	green	0.02098149	-0.1516743	0.86653184	0.36536698	0.23464887	0.38400621
cg00153990	OR7D2	green	-0.0384788	-0.1515318	0.77116793	0.10327822	0.04277734	0.26448853
cg02074891	SLC36A1;SLC36A1;SLC36	green	0.07065737	0.24912858	-0.5575935	-0.1251506	-0.0775081	-0.1906957
cg09942546	LINC00543	green	0.04065705	-0.0586931	-0.4978399	-0.221463	0.09908655	-0.1567537
cg04299170	HNRNPK;MIR7-1;RP11-	green	-0.0720001	-0.1685183	0.75751	0.06672356	0.02413164	0.29132667
cg16056150	AC060835.1	green	-0.0177407	-0.1440327	0.87156134	0.14817575	0.10695664	0.32932007
cg24975979	BAI2;BAI2;BAI2	green	-0.1183528	0.05492519	0.49757641	0.09654562	-0.0521141	0.20117302
cg02045294	MAMSTR;MAMSTR	green	0.03286816	0.02848492	0.36344757	0.09892594	0.13033663	0.2084127
cg00465058	SNHG5;SNHG5;SNHG5;SN	green	-0.0375359	0.08257241	-0.5906802	-0.2654641	-0.1624734	0.06906055
cg12853765	RP11-55K22.2;PEX7	green	-0.0200139	-0.1751992	0.84898178	0.12421586	0.09623353	0.32196736
cg17582400	SNORD115-	green	0.03186003	-0.0346346	0.76917246	0.0845116	-0.0032937	0.27757597
cg17558214	PHF14;PHF14;PHF14;PHF1	green	0.01287516	-0.1567755	0.77778958	0.16797685	0.15918501	0.42347801
cg15671708	FAM188A;FAM188A	green	0.16777456	0.05299968	-0.3908486	0.03775432	0.11385465	-0.0934074
cg16236725	DLEC1;DLEC1;DLEC1	green	0.12657251	0.21880925	-0.147292	-0.0467338	-0.1981258	-0.0370168
cg06653004	TFAP2B;TFAP2B	green	0.11853857	0.04612085	-0.5982282	-0.079248	0.08280311	-0.0914815
cg12521055	PAM16;PAM16;PAM16;PA	green	-0.2059643	0.0257786	-0.5334091	-0.1685461	-0.0731818	-0.4574589
cg25985748	NPHP3;NPHP3;NPHP3;NP	green	-0.0405094	-0.2038465	0.88065436	0.21016311	0.16733649	0.31714676
cg05251415	ERMP1;ERMP1;ERMP1;ER	green	0.15711112	0.03474308	-0.6711679	-0.1023661	0.08374686	-0.1223695
cg19087530	RN5S269;C8orf34;C8orf34	green	0.00351079	-0.1510081	0.75803464	0.16067049	0.13895288	0.29191422
cg02998566	RP11-138I17.1;RP11-	green	0.07269947	-0.1145856	0.85886095	0.25746021	0.19467566	0.29759603
cg09577417	RNF149;SNORD89	green	0.03726524	-0.0573822	0.68815143	0.13784687	0.0818741	0.29350077
cg00754027	KIF14	green	0.13543385	0.12420057	-0.6343107	-0.0847826	-0.0729205	-0.1886012
cg17121254	U6;CDH9	green	0.02579419	-0.0653438	0.84573245	0.20634257	0.10449582	0.34690875
cg20150306	CACNG6;CACNG6	green	0.11242034	0.16035542	0.37956492	0.25248188	-0.0549636	0.39183333
cg26568662	C7orf50;C7orf50;C7orf50	green	0.16534926	0.1374678	-0.3326299	0.11340988	0.08509117	-0.1364336
cg18274744	RP11-367J11.2;TADA2B	green	0.17135621	-0.0907751	0.55677856	0.43763956	0.27972862	0.32646812
cg02156356	ZNF883	green	0.07190297	0.02738914	-0.4149758	-0.0488629	0.01899633	-0.2048324
cg01577070	PRSS12	green	-0.0561196	-0.1664947	0.75814223	0.14790948	0.10507291	0.32912065
cg10922215	SLU7;SLU7	green	0.02527413	-0.132246	0.76892431	0.17975816	0.15559839	0.37498639
cg06395296	snoU13	green	0.13285923	0.06103275	0.57614929	0.2758446	0.11662215	0.15986204
cg03760060	SNHG14;SNHG14;SNHG14	green	0.06446815	-0.1007896	0.48788137	0.25704035	0.20014489	0.26796395

cg06098761	SERTAD4;C1orf133;C1orf13	green	-0.0525066	0.10919958	-0.4280081	-0.1374342	-0.0498112	-0.3946819
cg08859858	MBD1;MBD1;MBD1;MBD1	green	0.07987979	0.10463961	-0.3264312	-0.0940427	-0.1200987	-0.1267664
cg08393116	NBN;NBN;NBN;NBN;NBN;	green	-0.0552397	0.12622667	-0.4379754	-0.0542796	-0.1274399	-0.4503061
cg03437096	RP11-521J24.1	green	-0.0140108	-0.1622949	0.93537509	0.2069573	0.16337124	0.32953633
ch.10.666708	RP11-5N23.2;RP11-5N23.3	green	0.13504697	0.16309816	-0.3779166	-0.1343019	-0.0613457	-0.1236818
cg18926042	EDC4;EDC4;EDC4;EDC4;C	green	-0.0141129	-0.0002694	0.47139137	0.2778143	0.14941498	0.27172125
cg14480394	CPT1C;CPT1C;C19orf76;CP	green	-0.034972	-0.1856762	0.36230915	0.10603977	0.19572975	0.18752389
cg25139843	ANO3;ANO3;ANO3;ANO3;	green	-0.0220662	0.01942406	0.65163061	0.17959282	0.01438369	0.15211171
cg09417586	ATL2;ATL2;ATL2;ATL2;AT	green	0.04591653	-0.0236843	0.83942879	0.23636168	0.03434481	0.19750675
cg22317649	RALGAPA1;SNORA31;RA	green	-0.0892713	-0.1797722	0.67682043	0.17723758	0.19492933	0.00925264
cg04459413	HSPH1;HSPH1;HSPH1;HSP	green	0.09113605	0.24519612	-0.6087864	-0.1010702	-0.1265945	-0.1120117
cg09299770	RP11-731N10.1;CTD-	green	0.00882113	-0.1892166	0.85012799	0.20962338	0.14763515	0.40172158
cg26019412	RN5S265;TMEM68	green	0.06060053	-0.1194997	0.83764705	0.24386637	0.16185456	0.39545771
cg13206850	ATXN7L1;ATXN7L1;ATXN	green	0.02495741	-0.1145358	0.80341536	0.13729623	0.03713247	0.37166758
cg05478553	STK39	green	0.02753215	-0.1197187	0.82532908	0.19875436	0.06636407	0.25332181
cg10111450	PPP1R9A;PPP1R9A;PPP1R	green	-0.1450686	-0.100073	-0.7131987	-0.2631773	0.05332938	-0.1568563
cg01427957	CELF3;CELF3;CELF3;CELF	green	-0.1602891	-0.1965842	0.4134324	-0.055573	0.07913206	0.08304647
cg11793332	ELF1;ELF1;ELF1;ELF1;ELF	green	-0.1586195	0.01988407	-0.8581725	-0.2313205	-0.078825	-0.3757089
cg16243402	OPRM1;OPRM1;OPRM1;O	green	-0.0592851	-0.0257153	0.81227421	0.15957178	0.01105683	0.20483907
cg11127022	ARHGEF4;ARHGEF4;ARH	green	0.26588077	0.19338428	0.43131425	0.32517865	0.07363062	0.33785911
cg08994981	AC012501.2;AC012501.2;SN	green	-0.0411922	-0.066388	0.8360926	0.16648445	0.04556815	0.06742298
cg25669230	TPH2;TPH2;TPH2	green	-0.1043187	-0.0674095	-0.5535817	-0.2331619	-0.0544828	-0.1896103
cg14508379	SCG2;SCG2;SCG2	green	0.04491347	-0.1954494	0.83657935	0.34660088	0.26079513	0.34939266
cg04212239	SMC4;SMC4;SMC4;SMC4;	green	-0.1266189	-0.058393	0.35688483	0.00082495	-0.0762357	0.11543553
cg15476410	COL3A1;MIR1245A	green	-0.020738	-0.1730512	0.78995314	0.16721209	0.16466513	0.30027815
cg12089985	HIST1H4C	green	-0.0813028	-0.0511919	-0.4289709	-0.0598769	0.082289	-0.2626341
cg09316492	AC104820.2;AC068196.1	green	0.05073589	-0.1293971	0.77531226	0.17868367	0.18976962	0.31752601
cg14613617	VWA2	green	0.08626348	0.06108153	-0.378472	-0.0558114	-0.0071521	0.01236817
cg04600199	NME7;NME7;NME7;NME7;	green	0.06963935	-0.2303073	0.84221995	0.33136758	0.3058111	0.42415036
cg20521702	ST3GAL6-AS1;ST3GAL6-	green	-0.087663	0.07626966	-0.7835784	-0.1818088	-0.1057366	-0.2820659
cg13688257	OR5K2	green	-0.0273742	-0.11385	0.77360541	0.1422132	0.05514764	0.3615255
cg02466442	TMEM144	green	0.01031577	-0.0845081	0.86696989	0.13080311	0.07807921	0.44056203
cg23358787	PNLIPRP3	green	0.10074307	0.01342784	0.8952595	0.25289109	0.04619539	0.42255335
cg13615350	AF127936.7;AF127936.7;AF	green	0.08959882	-0.1040953	0.88301772	0.31211993	0.14666137	0.41022014
cg11700453	KPNA2;KPNA2;BPTF	green	-0.2220281	0.017768	-0.6486848	-0.2234558	-0.0640906	-0.406127

cg12273194	FANCM;FANCM;FANCM;	green	-0.0252548	0.05386367	-0.4852716	-0.1905504	-0.0471554	-0.1978599
cg17168830	ZNF195;ZNF195;ZNF195;Z	green	-0.1733012	-0.0231483	-0.6349707	-0.1414712	-0.0075201	-0.4263814
cg04438374	RP11-807H17.1	green	0.16340248	-0.0544511	0.71217861	0.21556644	0.07412759	0.60756298
cg03301084	HEY1;HEY1;HEY1;HEY1;H	green	0.02205964	0.00223266	-0.5916534	-0.0231457	0.02829257	-0.0958565
cg17291467	OR5D13	green	0.1038755	0.08946771	0.64148308	0.35072193	-0.0157254	0.31130613
cg08753678	ANKRD12	green	-0.0575112	-0.1287562	0.77941005	0.10938139	0.08545878	0.23441696
cg03424584	CDK8;CDK8;CDK8;CDK8	green	0.03829935	-0.1255239	0.81257618	0.26248175	0.09772611	0.37113313
cg27288424	ABCD2	green	-0.0160647	-0.0123696	-0.5555871	-0.1109455	0.03800772	-0.1050074
cg06712980	RP11-114G22.1;RP11-	green	-0.0184197	-0.0076233	0.83647105	0.22815781	0.00872025	0.32802214
cg08587073	WDR63;WDR63;WDR63;W	green	-0.0407026	0.06400401	-0.3239499	-0.1027897	-0.0507099	-0.1068188
cg16425355	Y_RNA	green	0.03605213	-0.1067623	0.85487682	0.22689751	0.16932116	0.24095449
cg25881170	CD47;CD47	green	0.00590175	-0.217491	0.45070253	0.09711236	0.19215544	0.28517073
cg22182854	C10orf100;RP11-202D18.2	green	0.08402117	0.02417788	0.87367509	0.19984936	0.01293761	0.48504181
cg13397390	FAM19A1	green	0.0483636	-0.1504197	0.84438006	0.27980603	0.1859631	0.28118731
cg00477577	SRFBP1	green	0.02629416	-0.161183	0.7862033	0.18588539	0.13857887	0.35496584
cg00981692	CTD-2532K18.2;CTD-	green	0.05324408	-0.0824789	0.89207967	0.18763994	0.0965193	0.3147244
cg15304707	KANSL1L;KANSL1L;KAN	green	0.01924423	-0.1443622	0.87090288	0.19880502	0.14103429	0.25442264
cg06751430	MYCT1;MYCT1;MYCT1	green	-0.0177545	-0.1331689	0.88703846	0.22732113	0.15121088	0.34387843
cg14756071	C6orf120;C6orf120;WDR27;	green	0.0025957	0.04825791	-0.6355957	-0.1206814	-0.0838287	-0.1811511
cg03182029	COBLL1;AC019181.2;COBL	green	0.04488576	0.11419921	-0.3188424	0.00089414	-0.1040696	-0.0156119
cg07872519	RUNDC3B;ABCB1;ABCB1;	green	0.03347853	-0.1139507	0.59497601	0.19551272	0.24924294	0.37691739
cg09535474	AGAP1-IT1	green	0.05572978	0.10348905	0.3734927	0.25398271	-0.0647755	0.34433057
cg00451635	EMP2;EMP2	green	0.0130672	0.13508871	0.39799287	0.2863266	0.06943102	0.23590362
cg27486909	RP11-	green	0.09113811	-0.0373284	0.78560303	0.16338122	0.03150904	0.38162504
cg20573702	OR6C6	green	0.17435334	0.02413673	0.8785009	0.31961536	0.12325075	0.47784572
cg04492963	HEMGN;HEMGN	green	0.1813431	0.21796161	0.63803752	0.35082296	-0.084765	0.40605921
cg13617074	RP11-29M5.1;MEIS2	green	0.06410992	-0.0698418	0.89553053	0.17079406	0.08384099	0.33963587
cg07978718	RP11-327J17.2;RP11-	green	0.15301045	0.02635568	0.51006605	0.27656696	0.09316065	0.37520146
cg23982237	FBXO9;FBXO9;FBXO9;FB	green	-0.2881114	-0.0392106	-0.453256	-0.1985006	-0.0582564	-0.5429177
cg03990478	HHLA3;HHLA3;HHLA3;H	green	-0.0027297	0.15748569	-0.4651575	-0.1854112	-0.2121326	-0.2430169
cg21248296	AC013727.2;AC013727.1	green	0.12008096	-0.0813043	0.92333697	0.25391331	0.12089041	0.46675055
cg10162403	IFLTD1;IFLTD1;IFLTD1;IF	green	0.07313562	-0.1285753	0.83114922	0.20293236	0.17864385	0.35249683
cg07015028	TMEM117;TMEM117;TME	green	0.16381392	-0.0406203	0.84552321	0.2211415	0.12309398	0.41911415
cg17990295	RP11-916O13.1;C12orf12	green	0.06684795	-0.0654289	0.88896927	0.27916497	0.11560315	0.40885968
cg04112602	HERC4	green	0.04574963	-0.2441967	0.77176015	0.16024922	0.24977576	0.26212988

cg00295744	CELF3;CELF3;CELF3;CELF	green	-0.1568091	-0.107858	0.31554678	-0.0426795	0.01400032	-0.0627247
cg13461104	PKIA;PKIA;PKIA	green	0.0196763	-0.1015983	0.82009895	0.25374012	0.14148854	0.33509724
cg00253847	CAMSAP1;CAMSAP1;CA	green	0.18384606	-0.112947	0.51899171	0.41219463	0.36637975	0.3462579
cg05155307	SH2D6;SH2D6;SH2D6;SH2	green	0.20970221	0.1294488	0.62747723	0.35776249	0.12467855	0.37450388
cg27498171	FGD4;FGD4;FGD4;FGD4;F	green	0.31922483	0.02113884	0.65397047	0.51310099	0.38707129	0.36838727
cg16974342	HOMER1;HOMER1;HOME	green	-0.0238662	-0.0629166	-0.6809035	-0.2318512	0.09371408	-0.3439523
cg20801778	RP11-	green	0.08884448	0.09189902	0.40268673	0.40585564	-0.0525958	0.2338197
cg12410930	KB-1208A12.3;RPL30	green	0.11491291	-0.1497704	0.80712371	0.26093276	0.18435386	0.48102502
cg27517719	KTN1;KTN1	green	-0.054978	-0.1593353	0.6947434	0.1659644	0.09686563	0.13669691
cg22403811	RALYL;RALYL;RALYL;RA	green	0.2890871	-0.1221571	-0.3713896	0.08655432	0.30910486	0.01885158
cg09032750	U7;UBAP2;UBAP2;UBAP2;	green	0.01014503	-0.1361518	0.75829461	0.17119952	0.11741849	0.15969577
cg11118271	RP11-710F7.2;RP11-	green	0.09362858	-0.039576	0.84276705	0.19143554	0.07361497	0.44144378
cg16234747	ITPR1;ITPR1;ITPR1	green	0.28626633	0.03405174	0.55183428	0.30490858	0.20687079	0.31258012
cg11718675	OR2L8;OR2L13	green	-0.0628419	-0.1636927	0.72037744	0.09458901	0.08362505	0.12120034
cg07741616	TINAGL1;TINAGL1;TINAG	green	0.24795423	0.06364072	0.46420302	0.30882898	0.15796449	0.36156898
cg04071089	TRAPPC11;TRAPPC11;TRA	green	-0.0507085	-0.1505693	0.67716922	0.16778583	0.09276226	0.20309776
cg17665419	MFSD1;MFSD1;MFSD1;M	green	0.11935071	-0.102702	0.74789755	0.19746102	0.15522949	0.39649157
cg09728223	U6	green	0.01081094	-0.1924476	0.68048299	0.15295841	0.17497071	0.3568554
cg13952377	RP11-279L11.1;CSMD1	green	-0.0298139	-0.0674479	0.89963505	0.15622248	0.03436711	0.27827354
cg09960211	PHLDA3;PHLDA3;PHLDA	green	-0.1568977	-0.0027662	0.29625596	0.07180196	-0.0192147	-0.4608909
cg09238384	NR3C2;NR3C2;NR3C2;NR3	green	0.0628699	-0.1530535	0.89268711	0.20461492	0.14905874	0.35804367
cg00066942	EPC2;EPC2;EPC2	green	-0.1364117	0.03713983	0.32687572	0.15255503	-0.0604188	0.04871553
cg03380207	USO1;USO1;G3BP2;G3BP2;	green	-0.0605527	0.07567047	-0.3637709	-0.2216227	-0.046406	-0.3131439
cg11909310	GTPBP10;GTPBP10;GTPBP	green	-0.2004793	-0.0461648	-0.5522188	-0.28285	-0.0651418	-0.3300602
cg26184501	ITGA3;ITGA3;ITGA3;ITGA	green	0.40966134	0.23251894	0.55010772	0.19635442	-0.0041757	0.2732025
cg20839257	AC068535.2;AC068535.3	green	0.07134474	-0.0764979	0.86481389	0.26451902	0.10047198	0.39791173
cg21657521	C19orf59;TRAPPC5;TRAPP	green	-0.0735494	0.14754294	-0.7077775	-0.1363778	-0.1432706	-0.2495263
cg06092921	MAPK10;MAPK10;RP11-	green	0.04751722	-0.1402889	0.83322589	0.1362124	0.14878435	0.42706119
cg04788221	C20orf20	green	-0.1546452	-0.0347265	-0.4389173	-0.2209746	-0.0163491	-0.244607
cg14701880	CNOT2;CNOT2;CNOT2;CN	green	0.09043269	-0.1003747	0.72825389	0.14440781	0.10218042	0.30037236
cg11686689	RP11-226L15.5;PIGM;PIGM	green	0.06081679	-0.1748782	0.72903329	0.24480816	0.21192461	0.32759172
cg24860775	GABRA1;GABRA1;GABR	green	-0.0466499	0.0393284	-0.7794002	-0.1877246	0.00198604	-0.359726
cg22511605	HRC;HRC;HRC;HRC	green	0.01175214	0.08449926	0.53503285	0.14151311	-0.027173	0.25833443
cg08414370	RP11-475A13.1;RP11-	green	0.06136431	-0.0412268	0.90264257	0.20250735	0.05484571	0.37476201
cg01824746	AP000473.5;AP000473.5	green	-0.0902625	0.00313193	0.83506871	0.15239683	-0.0176931	0.06827067

cg18515229	KLHL26	green	-0.2176457	0.08237584	-0.4647231	-0.2596908	-0.1381189	-0.4368462
cg15291475	SERPINH1;SERPINH1;SER	green	-0.0227388	0.11101753	-0.4505612	-0.1132872	0.02061159	-0.311365
cg01521154	RP11-877E17.2;RP11-	green	-0.34653	-0.2261737	-0.6337313	-0.2341237	0.0979515	-0.4104021
cg19240021	TIPRL;TIPRL	green	0.13439896	0.06781948	0.5643601	0.17464866	-0.0054435	0.19129185
cg09988663	CDKN3;CDKN3;CDKN3;C	green	-0.0707592	0.00112682	-0.5650182	-0.1767824	-0.0584254	-0.2746221
cg20222611	HOXA13;HOXA13	green	-0.1640969	0.0214973	-0.5999201	-0.2898089	-0.161517	-0.2553263
cg06731192	HCG14	green	-0.0882408	0.06627732	-0.5659755	-0.2070132	-0.0533183	-0.198436
cg06809970	BEND7;BEND7	green	-0.0034533	-0.0306137	0.74652302	0.13645603	0.03198941	0.23238184
cg23718415	SNX19;SNX19;SNX19	green	-0.0417314	0.02468273	-0.5872955	-0.2098448	-0.0162435	-0.0618065
cg26585428	PNLIPRP1;PNLIPRP1;PNLI	green	0.07379643	-0.0461459	0.89276265	0.18122957	0.02832163	0.48036613
cg26120680	RPP25L	green	-0.1577275	-0.0302957	-0.359962	-0.1918681	-0.0929886	-0.1848423
cg00403661	RN5S217;ECHDC1;ECHDC	green	0.14614639	-0.0984369	0.8525838	0.41528456	0.15382958	0.363747
cg18648427	RP11-294O2.2;RP11-	green	0.03725535	-0.0326147	0.82931345	0.18894456	0.05970115	0.34377059
cg19049152	THSD1P1;THSD1P1	green	-0.12304	-0.0762398	0.35983173	0.10162221	0.03661842	0.14425093
cg03801404	C21orf91-	green	-0.1774887	-0.0916112	-0.4837342	-0.1767539	0.07352524	-0.2563112
cg01570069	HELLS;HELLS;HELLS;HEL	green	-0.034235	-0.0083575	-0.3701351	-0.0807704	0.11735059	-0.172099
cg22585269	CHEK2;HSCB;HSCB;HSCB	green	-0.0025839	0.06375694	-0.3458449	-0.2754372	-0.1352578	-0.2498452
cg09194099	CEP44;CEP44;CEP44;CEP44	green	0.16411041	0.09831102	0.67649756	0.33474695	-0.0551674	0.37962249
cg19198472	LLGL1	green	0.28412976	0.09764541	0.73119907	0.4289547	0.11510046	0.49339929
cg00981832	KIAA1598;KIAA1598;KIA	green	0.04822243	-0.1438918	0.65370889	0.12313452	0.15272811	0.10042188
cg10405256	RP11-893F2.5	green	0.28365533	0.25944138	0.54564804	0.59066311	0.05778259	0.28876853
cg26252703	FAM102B;FAM102B	green	-0.2214835	0.03038885	-0.4831691	-0.153661	-0.0942089	-0.3444012
cg11517848	RP11-47I22.3	green	0.07700418	-0.0745355	0.79914703	0.20113004	0.07431387	0.23496199
cg06131526	FBXO5;RP1-	green	-0.1953388	0.02546174	-0.7046274	-0.2545103	-0.1168945	-0.2382266
cg25865672	STATH	green	0.06910907	-0.0826902	0.90422785	0.22382826	0.09665474	0.47915755
cg18382401	RP11-348J24.1;RP11-	green	0.09553079	-0.1000231	0.90938476	0.24182996	0.12807116	0.50858011
cg12867312	BRAF;BRAF	green	-0.0431108	-0.12656	0.75613384	0.13283115	0.08924523	0.30774786
cg07808773	MFF;MFF;MFF;MFF;MFF;	green	-0.163326	0.00883134	-0.4889074	-0.230086	-0.1181811	-0.2896849
cg18588204	RAD21;RAD21;RAD21-AS1	green	0.02686599	-0.0052464	0.78351234	0.20372676	0.0205447	0.34186975
cg04471409	RPS6KA2;RPS6KA2;RPS6	green	-0.2022439	0.01548479	-0.3231576	-0.2354995	-0.1453959	-0.2643571
cg14593650	AC011306.2	green	0.015163	-0.1452701	0.89003513	0.18595117	0.15784981	0.21942129
cg23920293	KRIT1;KRIT1;KRIT1;KRIT1	green	-0.0461023	-0.1624734	0.83208801	0.16913933	0.14760438	0.29654361
cg03417912	LINC00492	green	0.09981273	-0.0712016	0.88717594	0.17314699	0.08876636	0.41981636
cg19470294	RP1-28O17.1;RP1-28O17.1	green	0.09880212	-0.0139246	0.88675341	0.270616	0.05304478	0.42138289
cg17082662	C3orf38;C3orf38;C3orf38;CG	green	-0.2331315	0.00685455	-0.4852105	-0.0933709	-0.1090335	-0.362195

cg01938189	ANKRD34C;RP11-	green	-0.2159677	-0.1990259	0.36802744	0.03516545	0.07032209	-0.0089727
cg14312557	HECW2	green	-0.048109	0.08050476	-0.5285268	-0.1673031	-0.0677956	-0.2167943
cg13976259	RP4-705F19.1	green	0.30718762	0.17822888	0.60094828	0.299307	0.07822861	0.49544975
cg21916555	NAF1;NAF1	green	0.13208235	-0.0823177	0.92184484	0.25405276	0.13128993	0.47200432
cg03047277	NAAA;NAAA;NAAA;NAA	green	0.22963315	0.0599776	0.59541235	0.22071814	0.05971336	0.45968708
cg12476823	SNORD114-25;SNORD114-	green	-0.0143078	0.02278594	0.52064332	0.09721679	-0.0542277	0.14718668
cg23413954	CTC-458I2.2	green	0.13074025	-0.1194364	0.85191559	0.24442465	0.14689206	0.36416965
cg19548283	RP11-	green	0.0273834	0.09092478	-0.4572866	-0.0595995	-0.0453924	-0.2253458
cg15006883	PRPF40A	green	0.00378872	-0.1744485	0.73093105	0.16374914	0.16084248	0.21869811
cg17513708	OR2G3;RP11-	green	0.07992379	0.1776522	0.81310277	0.19282066	-0.1415832	0.31862451
cg10532188	AC097721.2;AC097721.2;SP	green	-0.05875	-0.0574295	-0.7954993	-0.2427166	0.05281974	-0.2261288
cg22317498	U6	green	-0.0188553	-0.1170322	0.67463907	0.12317589	0.08617505	0.11011271
cg27124774	PCDHB10	green	0.00212201	0.0783264	-0.5657675	-0.1210719	0.02984514	-0.1762659
cg08636145	TFAP2A;TFAP2A;TFAP2A;	green	0.0647411	0.08581571	-0.4433121	-0.1569168	-0.1264795	-0.1168476
cg27404696	KIAA0430;C16orf45;KIAA0	green	0.29068963	0.11504189	0.68045394	0.48770414	0.26639365	0.38147101
cg04880751	KMO;KMO;KMO;KMO;K	green	0.06552679	0.173739	-0.651393	-0.1529332	-0.1085833	-0.3530978
cg07604651	RP11-	green	0.16103443	-0.0242673	-0.4267431	-0.0318797	0.1629031	0.03766838
cg10958362	C5orf38;IRX2;C5orf38;C5orf	green	-0.1853014	-0.0971958	-0.6479402	-0.1865511	0.09237321	-0.421204
cg12196780	KLHL9;KLHL9	green	0.14144055	0.06677247	-0.4854337	0.03252065	0.14322526	-0.2250968
cg15239238	SOX6;SOX6;SOX6;SOX6	green	0.05372797	0.05296742	-0.4861431	-0.1689662	0.01126452	-0.406251
cg05548403	CAST;CAST;CAST;CAST;C	green	-0.0348168	0.15927141	-0.7290779	-0.1931251	-0.1702551	-0.4623615
cg19400055	PRRC2A;PRRC2A;PRRC2A	green	-0.1083353	0.03576006	-0.5331719	-0.2991068	-0.1339312	0.04498571
cg19219778	TESPA1;TESPA1;TESPA1;T	green	-0.2106389	0.00352441	-0.7070067	-0.1860806	-0.0703886	-0.3186795
cg18006794	AGPAT3;AGPAT3;AGPAT	green	0.15809038	0.05510718	0.34270422	0.12596473	0.0205861	0.34320297
cg03334213	C4orf26;C4orf26	green	0.01401817	0.16119777	0.63525901	0.15876231	-0.2213903	-0.0021635
cg22088096	TINAGL1;TINAGL1;TINAG	green	0.18772522	0.09639366	0.32517943	0.29644651	0.11040232	0.35258892
cg25191416	RP11-78C6.1	green	0.05820863	-0.1437297	0.79190387	0.19440118	0.19095619	0.35299303
cg06410536	AP5M1;AP5M1;AP5M1;AP	green	-0.027705	0.18613347	-0.336961	-0.1621335	-0.15341	-0.2468122
cg15824062	GPHN;RP11-72M17.1	green	0.17992256	-0.055491	0.88016821	0.34757656	0.1834152	0.32933145
cg26913033	GBE1;GBE1	green	-0.0359279	-0.0545339	0.86090764	0.1495042	-0.0122891	0.33754815
cg06133339	FAM163B;FAM163B	green	0.18158604	-0.0949912	0.83576501	0.27215919	0.21101092	0.43952701
cg04783624	RANBP9	green	0.13207562	-0.0827401	0.82567111	0.23603923	0.11554082	0.54367931
cg13714403	SIK3;SIK3;SIK3;SIK3;SIK3;	green	0.04767934	0.17899812	-0.5044903	-0.0438003	-0.0450584	-0.1895409
cg21796999	SGSM2;RP1-	green	0.33746564	0.07472276	0.54193525	0.60366145	0.21919197	0.53291659
cg06422757	KCTD20;KCTD20;KCTD20;	green	0.10901799	0.09435399	-0.6131347	0.00491221	0.14996278	-0.1487939

cg03980224	RAI14;RAI14;RAI14;RAI14;	green	0.20711438	0.1133243	0.75930841	0.25358953	-0.0179878	0.32107131
cg23945243	RP11-335O4.1	green	0.01610543	0.00910499	-0.6666102	-0.1378679	0.03026445	-0.3036251
cg13500074	BCL11A;BCL11A;BCL11A;	green	-0.0581416	-0.1083932	-0.6266993	-0.1004581	0.07945581	-0.0644822
cg12933901	UGGT2-IT1	green	0.06194775	0.01489779	0.78351298	0.1687526	0.01080514	0.33781495
cg00427601	N4BP2L1;N4BP2L1;N4BP2	green	0.0539004	0.00885296	0.79566597	0.12574095	-0.0043381	0.06933844
cg04830221	VWC2L-	green	0.09292458	0.09950913	0.8441331	0.1975984	-0.0381063	0.41735772
cg07677319	AL157359.4	green	0.05477008	-0.0954632	0.91945557	0.19988085	0.11864058	0.302021
cg01823709	GPBP1;GPBP1;GPBP1;GPB	green	-0.1494523	0.04269585	-0.5256105	-0.2127486	-0.0963338	-0.30486
cg09110662	AF165138.7	green	0.04201582	-0.1209085	0.85098086	0.19505522	0.08092759	0.28633434
cg06354975	LIMCH1;LIMCH1;LIMCH1;	green	0.03278695	0.07747156	0.5600193	0.13531671	-0.0316831	0.06418869
cg18382261	TFIP11;TFIP11;TFIP11;TFIP	green	-0.1973367	-0.0950331	-0.3450458	-0.1353812	0.05395495	-0.3088775
cg18986489	IKZF2;IKZF2;IKZF2;IKZF2	green	0.05117466	-0.0530812	-0.4729969	-0.0377039	0.16730761	-0.1390816
cg01532321	EIF4G3;EIF4G3;EIF4G3;EIF	green	0.10910805	-0.1209734	0.88630759	0.17522311	0.15688604	0.41013046
cg12704417	SATB1;SATB1;SATB1;SAT	green	-0.1817661	0.09045634	-0.6195738	-0.2128477	-0.1774376	-0.5714036
cg10830493	U6	green	0.17621295	0.00334747	0.83409896	0.24742839	0.08962553	0.31015993
cg25037788	U6	green	0.08146222	-0.0161254	0.65006262	0.17486244	-0.0152489	0.32982379
cg11578462	DMXL1;DMXL1;DMXL1	green	0.08046379	-0.1111642	0.89514564	0.18609559	0.10031959	0.52728427
cg26782876	TPP2;TPP2;TPP2	green	-0.0138321	0.04037184	-0.5973709	-0.1127178	0.00656768	-0.1328805
cg05098432	PLCE1;PLCE1;RP11-	green	0.20764442	0.18607417	0.60349433	0.24827034	0.02772091	0.24541066
cg05596621	RP11-507P23.3;RP11-	green	0.16928332	-0.1161063	0.38105886	0.09495102	0.23775877	0.28957311
cg24594819	CXXC4;CXXC4;AC004053.	green	-0.1652868	-0.0633839	-0.5428953	-0.1873133	0.00946394	-0.4236582
cg01695677	EIF4G3;EIF4G3;EIF4G3;EIF	green	0.04519722	-0.11743	0.84351359	0.25546817	0.08737033	0.36462752
cg11324445	ZC3H14;ZC3H14;ZC3H14;Z	green	0.16624498	-0.0611246	0.72291533	0.3438748	0.09597832	0.32425623
cg19107894	AC073218.2;AC073218.2	green	0.07215723	-0.1312519	0.78805454	0.18092487	0.15688339	0.32265381
cg07705639	Y_RNA;MRPS14;MRPS14	green	-0.2083175	0.02185198	0.31904903	0.04077245	-0.065428	0.11210371
cg19688118	CCDC112;CCDC112;CCDC	green	-0.0787924	-0.052438	-0.317153	-0.106479	-0.0180605	0.21030248
cg25142260	CHD1;CHD1	green	0.04792569	-0.1755864	0.78295267	0.23985189	0.15446865	0.41515136
cg26005146	NUP205;NUP205;NUP205;	green	0.04568798	0.00289517	-0.3426232	-0.0878748	0.06911804	0.21271647
cg24535176	EYA4;EYA4;EYA4;EYA4;E	green	0.15331634	-0.1466991	0.66724403	0.2682772	0.26493232	0.32098927
cg05089849	FAT4	green	0.15588673	-0.0565668	0.90688338	0.38248971	0.13390286	0.39707203
cg21827013	SLC16A7;SLC16A7;SLC16	green	0.11869472	-0.1507792	0.68056289	0.20580728	0.27807107	0.26114698
cg08685854	ZNF488;ZNF488;ZNF488;Z	green	0.03406704	-0.0266352	-0.680805	-0.0512359	0.11312302	-0.210034
cg16682960	NAALADL2-	green	0.06096308	0.02856345	0.87088538	0.20593876	0.02407657	0.49334156
cg21509296	RP11-294O2.2;RP11-	green	0.04476545	-0.0042883	0.65805882	0.11119977	0.02906928	0.10592093
cg25687611	NDUFAF2	green	-0.0247452	-0.055565	0.62036692	0.08222209	-0.0096905	0.06905486

cg21839244	CILP2;YJEFN3	green	0.37537984	0.08720476	0.56393516	0.38803912	0.35285405	0.43451869
cg00354692	VCAN;VCAN-AS1	green	0.01941913	0.01241736	0.81327741	0.15021789	-0.0464243	0.44782565
cg12469756	AGL;AGL;AGL;AGL	green	0.13969128	-0.142205	-0.5656299	0.01054899	0.28385574	-0.2204692
cg10154200	AC021749.1	green	0.01093284	0.02843263	0.80213366	0.11765667	-0.0167811	0.1747915
cg17740568	RP11-537P22.1;SLC25A21	green	0.17353733	-0.0469424	-0.5096411	0.042811	0.26253647	-0.1473307
cg07974719	EDNRB;EDNRB;EDNRB;E	green	-0.0818974	-0.1635998	0.36293154	0.23056874	0.20812549	0.21426236
cg27050750	C15orf42;C15orf42;RP11-	green	0.03343189	0.08761966	-0.3205652	0.02836702	0.00963305	-0.0883143
cg23170347	PRICKLE2	green	0.08649115	-0.0058424	0.53039696	0.16486519	0.10630569	0.24498547
cg02902110	ABRACL;ABRACL	green	-0.1649579	-0.1898902	-0.3926045	-0.1588589	0.12952513	-0.4158579
cg05959720	CTD-2003C8.2;CTD-	green	-0.086096	-0.091996	0.49111853	0.14204905	0.08276356	0.09132594
cg05224573	COPS8;AC105760.2;AC1057	green	-0.2319831	-0.1021955	-0.6292682	-0.2432752	-0.0114865	-0.3978682
cg08527179	C2orf89;C2orf89;TMSB10	green	0.09301633	0.01367261	-0.3818881	-0.1162878	0.0350927	0.20511682
cg02905326	LZTR1;LZTR1;AIFM3;AIF	green	0.30622748	-0.0804223	0.66767923	0.41099497	0.33258455	0.37025931
cg12311154	RP11-436A7.1	green	0.04140471	-0.1212567	0.87717192	0.19674095	0.12782302	0.41636668
cg22188627	RP11-115D19.1;RP11-	green	0.21126277	-0.0115573	0.8756867	0.32663922	0.12548438	0.43737947
cg08265548	PHOX2A;PHOX2A	green	0.20316838	0.07125037	0.69958711	0.5377736	0.05676975	0.41566635
cg25449399	CYYR1;CYYR1	green	0.03171089	-0.0348429	0.50343342	0.16280119	0.08550974	0.25005848
cg02166179	ZNF93;ZNF93	green	0.21982167	-0.093311	0.82247009	0.31749782	0.24106622	0.53538063
cg22476711	TBC1D4	green	-0.1348887	0.06146871	-0.6932424	-0.2046942	-0.0992822	-0.4268665
cg24339092	PCDHA8	green	0.13005039	-0.0616615	-0.6401767	-0.0969239	0.13494251	-0.0378744
cg11346890	BTBD7;BTBD7;BTBD7;BTB	green	0.05157746	-0.1018467	0.89285994	0.22086169	0.09038484	0.34186518
cg00054301	MIR328	green	0.15362982	0.18838642	0.37892464	0.25361365	-0.0429266	0.11562163
cg01945274	RP11-181C3.1	green	-0.041496	0.05356049	-0.3888468	-0.1122257	0.00446007	-0.1500501
cg21214753	TP63;TP63;TP63;TP63	green	4.85E-05	-0.0743238	0.86919143	0.1402674	0.02275761	0.28320064
cg11503011	RP11-175K6.1;RP11-	green	-0.2937609	-0.0218843	-0.6001906	-0.3552951	-0.194383	-0.5259112
cg16803128	FAM26D;FAM26D;FAM26	green	0.12442998	-0.0254445	0.77971976	0.18840317	0.02061189	0.35902197
cg04799831	RP5-	green	0.2084547	0.05888198	0.65356216	0.28086855	0.08756951	0.42157257
cg26006767	TRIM27;TRIM27;TRIM27	green	0.16156427	0.00332558	0.74880214	0.25401698	0.08671807	0.36848901
cg20938830	ATR;ATR	green	0.12508741	-0.0728343	0.85892954	0.20588245	0.09513365	0.39420316
cg00769068	AKR1CL1	green	0.19575615	0.24227961	0.57508387	0.3785061	-0.0307544	0.24930809
cg13214943	SNORD56	green	0.20530661	-0.0799081	0.74716714	0.21245234	0.18749987	0.66152537
cg04998805	ARHGDIB;ARHGDIB;ARH	green	-0.073277	0.0398489	-0.2813367	0.18800869	0.13786699	-0.1355923
cg16554192	NUDCD3;NUDCD3	green	0.21963946	-0.0047202	0.70796035	0.26076735	0.08991586	0.42939719
cg25716314	CTD-2193G5.1	green	0.09254045	-0.1220629	0.89261701	0.255348	0.14191535	0.45185979
cg17886959	MT2A;MT2A;MT2A;MT2A;	green	0.12250943	0.12293256	0.37971318	0.41323665	0.14259915	0.23103645

cg23953279	AL110292.1;RP11-	green	0.16219043	-0.0718755	0.79191523	0.17703796	0.16630699	0.51488807
cg07490133	CELSR2;CELSR2;CELSR2	green	0.21633048	0.09477795	0.76269105	0.29868667	0.05645724	0.41736558
cg06692169	LYPLAL1;LYPLAL1;LYPL	green	0.09228488	-0.1322792	0.77260124	0.20508522	0.20726523	0.28123585
cg25863892	RP11-	green	0.13094461	-0.0493289	0.87564777	0.24543385	0.12069889	0.42988937
cg06340043	RP11-	green	0.35995003	0.1313547	0.56651719	0.33836449	0.08814423	0.59774596
cg05546525	TMPRSS11E	green	0.12132218	-0.0745933	0.89835863	0.27543982	0.14434522	0.38651925
cg11661235	THEM5	green	0.06870621	-0.0810321	0.76229401	0.16361169	0.01754535	0.27868292
cg21643316	AC010105.1	green	0.03424344	-0.0598756	0.49989503	0.26759201	0.14739096	0.09685048
cg09928046	COL11A1;COL11A1	green	0.02744618	0.13267818	0.78324782	0.20958068	-0.1667788	0.32089791
cg21817791	RP11-361F15.2;STXBP5	green	0.01665866	-0.0556931	0.89264097	0.16974759	0.00028444	0.46848559
cg01451877	U2	green	0.05556658	0.04891467	0.72368546	0.30537639	-0.0188604	0.42744372
cg07827420	SEPT9;SEPT9;SEPT9	green	-0.1623107	-0.0998142	0.43467347	0.11429403	-0.0460967	0.34064222
cg26121257	RP1-257I9.2	green	0.12779599	0.00743332	0.89121397	0.20637588	0.06694965	0.33942873
cg23116041	GAL3ST1;GAL3ST1;GAL3S	green	0.01352294	0.09145009	0.36174451	0.07844654	-0.0408041	0.27751686
cg11417212	RN5S381	green	0.10784199	0.00218016	0.86411474	0.17620865	-0.0043433	0.50288734
cg11638850	NELL2;NELL2;NELL2	green	0.04032895	0.02734263	0.82236634	0.12046628	-0.09162	0.3667286
cg16150605	ATP5B;ATP5B;ATP5B;ATP	green	0.11655194	-0.018861	0.65414891	0.18341545	0.00057786	0.35191057
cg26357453	DHRS7B	green	-0.0228189	-0.0453858	-0.3400837	-0.0706773	0.02907583	-0.037194
cg11872859	RP1-97G4.1;RP1-34H18.1	green	0.17013658	0.00999192	0.93700785	0.29219033	0.07582297	0.42859829
cg21591274	ANKRD17;ANKRD17	green	0.04784894	-0.1794319	-0.6932013	0.01492122	0.35455172	-0.2596822
cg09108752	snoU13	green	0.05219745	-0.0714925	0.77747541	0.20428467	0.0751109	0.19346918
cg05954779	KLHDC1;KLHDC1;KLHDC	green	-0.0342961	-0.1111607	0.75839882	0.16838254	0.03359927	0.14550772
cg12426896	CDH19;CDH19;CDH19	green	0.11989717	-0.1160423	0.87209055	0.28085458	0.18960831	0.5922072
cg09878033	OR5AR1	green	-0.0617559	-0.0541619	0.68907819	0.16754077	0.07471256	-0.0948536
cg18191688	PDE4DIP;PDE4DIP;PDE4DI	green	0.1361865	0.03837024	0.37939877	0.13952311	0.09692293	0.11539319
cg02730326	ELTD1	green	0.15794722	0.06719034	0.47226461	0.14494621	-0.0321108	0.32874551
cg16610086	SLC1A3;SLC1A3;SLC1A3;S	green	0.01527055	-0.0500512	-0.4617427	-0.0571564	0.14667888	-0.2197393
cg10358206	CORIN;CORIN	green	0.00845202	0.0356891	0.80400752	0.0935129	-0.0941918	0.2241853
cg23719290	U6	green	0.13119757	-0.0562881	0.81874494	0.17495729	0.10425711	0.34756511
cg06523556	CHRNA6;CHRNA6;CHRN	green	-0.1469132	0.0094862	-0.3449749	-0.0345676	-0.0740132	-0.0980931
cg04263383	ANGPTL3;ANGPTL3	green	0.09575232	-0.0433416	0.89284831	0.18731817	0.03562449	0.42379495
cg16396891	U6	green	0.2084385	0.00937045	0.72854889	0.23984023	0.11377928	0.46563747
cg06588683	SLC2A8;SLC2A8;SLC2A8;S	green	0.25035959	0.24438016	0.48916176	0.31389571	-0.0423693	0.5366646
cg00267054	VPS54;VPS54;VPS54	green	-0.1456015	-0.102798	-0.4546178	-0.1727954	-0.0161501	-0.0422588
cg26216557	IFNA14	green	0.08237418	-0.1353859	0.91415601	0.24015191	0.15491372	0.40919488

cg02404744	RP11-382E9.1	green	0.19397902	-0.1077766	0.86632437	0.22854601	0.21199616	0.39483566
cg16954004	RAB6B;RAB6B;RAB6B;RA	green	-0.0474247	0.00263628	0.33582308	0.07339524	-0.0533622	0.05727773
cg08460080	GABRG3;GABRG3;GABR	green	0.12782303	-0.0969914	0.59809009	0.16636387	0.13268789	0.57929574
cg10802555	HMCN1	green	0.03823041	-0.0270332	0.83868928	0.17663523	0.04709222	0.29618804
cg00504034	COMMD6;COMMD6;COM	green	-0.1008802	-0.0501413	-0.3304066	-0.278608	-0.1302258	0.07671111
cg11873500	PPFIA2;PPFIA2;PPFIA2;PP	green	0.13371355	0.10347553	0.82328404	0.19290134	0.04399845	0.27020021
cg01745599	DEFB126;DEFB126	green	0.06317196	0.11845016	0.61922003	0.27121477	-0.0889819	0.34008607
cg14403634	RP11-341A22.2;RP11-	green	0.08514661	0.14483717	0.82327862	0.18447192	-0.0875151	0.30768203
cg09447808	SLC39A8;SLC39A8;SLC39	green	-0.0820981	-0.2292226	-0.4364096	-0.0872817	0.17425364	-0.0826114
cg19118077	U8;AKR1C3;AKR1C3;AKR	green	0.10904733	0.00715707	-0.5955194	-0.0087189	0.15656215	-0.1255513
cg21502834	TFAP2B;TFAP2B	green	0.05312097	0.01910573	-0.4709035	-0.1587146	0.08509806	-0.108855
cg07632208	RP11-298O21.5;RP11-	green	0.06956135	-0.0349538	0.91642729	0.18646886	0.02095607	0.30691478
cg12833292	FAM3C;FAM3C;FAM3C;F	green	0.1741041	-0.0643486	0.93076753	0.22911413	0.1083826	0.43885893
cg16225168	MEF2C;MEF2C;MEF2C;ME	green	-0.1063539	-0.2591259	-0.6443135	-0.2460136	0.17650487	-0.2172116
cg04133410	PTCH1;PTCH1	green	-0.2544588	-0.0983915	-0.5438293	-0.2280814	0.03493758	-0.3817542
cg08250541	MTFR1;MTFR1;MTFR1;MT	green	0.12227117	0.06027222	0.84593524	0.2215799	-0.0381043	0.37363949
cg06633037	PCDHB15	green	0.08472318	-0.0854141	0.62842314	0.17251133	0.13391588	0.24205609
cg01438434	WSCD1;WSCD1;WSCD1;W	green	0.02282108	-0.0188207	-0.4293316	-0.0085184	0.14855116	-0.0822941
cg00134611	SNRNP48	green	0.2173249	-0.0843727	0.76461754	0.2339484	0.17280234	0.35280872
cg23305428	MLL3	green	0.11126991	-0.1166973	0.78063886	0.23502419	0.20813705	0.22601703
cg06412210	AK4;AK4;AK4;AK4;AK4	green	0.27804567	0.08051133	0.53480575	0.26857306	0.12773217	0.57278978
cg15615683	STOML2;STOML2;STOML	green	0.27178277	0.16918749	0.43246741	0.33835821	0.09028092	0.43712859
cg00247599	HIST1H3H	green	-0.110799	0.00500151	-0.2958861	-0.0748266	-0.0716005	-0.174397
cg00907661	ZDHHC20;ZDHHC20;ZDH	green	0.34020237	0.07268263	0.49770001	0.24351983	0.10565236	0.41048243
cg01946480	TSPAN15;TSPAN15	green	-0.2124679	-0.0510942	0.35835789	0.02254044	0.03708583	-0.0528051
cg06488135	PPP4R4;PPP4R4;PPP4R4;PP	green	-0.0737063	-0.063055	-0.4164543	-0.1205033	0.03614469	-0.1956797
cg01438216	AC092415.1;AC092415.1	green	0.18062422	-0.0456824	0.77932793	0.20967087	0.11476786	0.49592152
cg12501949	CCDC141	green	0.21086637	-0.0115733	0.87506103	0.24011291	0.11927402	0.3371331
cg04844516	CDH18;CDH18;CDH18;CD	green	0.13076045	-0.0188701	0.86339973	0.25838406	0.0894514	0.33173695
cg23033619	RP5-1185K9.1	green	0.19839949	-0.0362991	0.69766345	0.25698971	0.11593584	0.40827563
cg02941068	SLC9B2;SLC9B2;SLC9B2;S	green	0.08652789	-0.0721845	0.79394918	0.19084385	0.10127366	0.224883
cg26804004	SCAPER;SCAPER;SCAPER	green	0.23176252	-0.0970298	0.84871044	0.28367566	0.22459241	0.51137569
cg14771658	SCN7A;SCN7A	green	-0.0804506	-0.0128513	0.35739898	-0.1126105	-0.1066919	0.09247928
cg05085162	FRRS1;FRRS1;FRRS1	green	0.0793359	-0.1001817	0.84572618	0.14172589	0.11208411	0.21868981
cg01953591	F5;F5;F5	green	-0.1811278	-0.0759258	-0.3557711	-0.1961774	-0.0483647	-0.1425965

cg24960006	DPYD-AS2;DPYD-AS2	green	0.16679815	-0.0264857	0.91613452	0.22475892	0.08660669	0.46846855
cg19676479	CTD-2050E21.2	green	0.18166115	0.06049354	0.83582955	0.22833757	0.03953987	0.41438795
cg21408445	PTPN13	green	0.14094601	0.01348813	0.77388143	0.10633708	0.03270477	0.40793723
cg02347356	RP11-	green	0.17952859	-0.0100668	0.40506073	0.2480813	0.19176438	0.22334968
cg12041010	ING3;ING3;ING3;ING3;ING	green	-0.1862373	-0.0074649	-0.3970905	-0.1686608	-0.0565851	-0.3127479
cg00761968	LECT1;LECT1;LECT1	green	0.09219031	0.02028905	0.44606233	0.20603458	0.09650165	0.21231876
cg04773714	NHLH2;NHLH2;NHLH2;N	green	0.00440578	-0.0112242	0.49817684	0.1623538	-0.0511072	0.25447396
cg08357850	MLKL;MLKL;MLKL;MLKL	green	0.03382815	0.06870725	-0.3708012	-0.0539915	-0.0485531	-0.1753338
cg14352267	CCDC68	green	0.19373781	-0.0698225	0.78610727	0.20426333	0.09916852	0.68471573
cg27401196	RP11-257A22.1	green	0.06084066	0.0850811	0.83785386	0.19581325	-0.0577235	0.49752558
cg18190847	HRH1;HRH1	green	-0.3285011	-0.1768068	0.36268224	-0.0289058	-0.0531633	-0.0219176
cg13294509	GLE1;GLE1;GLE1	green	-0.1219982	-0.0540054	-0.4383711	-0.0666797	0.0095568	-0.1859728
cg23576842	SETBP1;RP11-	green	-0.0876299	0.00411866	-0.4903014	-0.1855415	0.00669417	-0.334496
cg14402194	PRMT5;PRMT5;PRMT5;PR	green	0.08250579	-0.0817287	0.821677	0.18167317	0.11701731	0.39891398
cg05077768	U6;ADAMTS12	green	0.08733783	-0.0714467	0.79963388	0.12654425	0.0977209	0.23888928
cg00828713	RALGAPB	green	0.07357155	-0.0790771	0.777713	0.1742563	0.03186797	0.41589401
cg19359099	HK2;HK2	green	0.27829233	0.25524682	0.40305586	0.24515619	0.0230953	0.24815758
cg19868031	TRAM1L1	green	0.20464662	0.0967296	0.86178612	0.28035509	0.03978186	0.42530554
cg10437902	EXOC5;EXOC5;EXOC5;EX	green	0.36947978	-0.1260165	0.57722439	0.62018885	0.52340146	0.49946247
cg08982380	EPB41L4A;CTC-	green	0.02275387	-0.0275677	0.52824596	0.04860705	0.08826892	-0.1791298
cg16637427	MFGE8;MFGE8;MFGE8;M	green	-0.0505735	0.00738786	-0.4066638	-0.0386683	0.03518702	-0.2431077
cg09262491	RP11-166N17.3;EPC1	green	0.30464199	0.04266707	0.63212135	0.38191378	0.07116867	0.38097632
cg25308022	CD44;CD44;CD44;CD44;CD	green	0.08516261	-0.0569363	0.71042409	0.16207194	0.04330122	0.32602888
cg00791207	SRSF8	green	0.18354898	-0.0376925	0.88205475	0.28373573	0.10497835	0.41361888
cg16560433	RP11-2H8.2	green	0.01779847	-0.0906236	0.70838909	0.2076856	0.07107868	0.24111628
cg14672173	AC010105.1	green	0.15365983	-0.0209227	0.46257563	0.2752048	0.19619467	0.17810851
cg24373647	PALLD;PALLD;PALLD	green	0.27345077	-0.0338947	0.62320764	0.30922184	0.23702932	0.27884581
cg18964261	TUBE1;TUBE1;C6orf225	green	-0.296388	-0.1957869	-0.5085281	-0.1865414	0.05851499	-0.376435
cg19212222	Y_RNA	green	0.10956796	-0.0175136	0.74367284	0.21595346	0.03431846	0.20161926
cg05015635	GAS1;RP11-276H19.1	green	-0.143997	0.06253837	-0.4825897	-0.1851493	-0.14589	-0.2561448
cg20018396	GS1-18A18.2	green	0.22603451	0.05437224	0.86670552	0.24864428	0.08732218	0.39624958
cg07058144	MICALCL	green	0.16326295	0.20897978	0.53068316	0.21634608	-0.1543644	0.29041829
cg14888864	CDH19;CDH19;CDH19	green	0.05346606	0.03640129	0.87967616	0.17579383	-0.0202388	0.40999507
cg22596847	FRS2;FRS2;FRS2;FRS2;FRS	green	0.00368558	-0.1121472	0.73254207	0.13141793	0.05027786	0.2417354
cg14079243	AGTPBP1;AGTPBP1;AGTP	green	0.3096675	0.21447091	0.61873887	0.33225014	-0.0647344	0.53906143

cg20944970	PIK3C2G;PIK3C2G;RERGL	green	0.03399696	0.05702704	0.88045872	0.1658592	-0.0900121	0.35128469
cg25647648	RP11-219E24.1;RP11-	green	0.35699056	0.15106763	0.81682858	0.29015968	0.05011181	0.58578535
cg26590064	UBAP1L	green	-0.0827108	0.00773368	0.24111724	0.10199115	-0.0058507	-0.2530306
cg14696931	OR5AK2	green	0.16243079	0.19227895	0.83736713	0.29521746	-0.0960877	0.35010039
cg07418518	BHMT;BHMT;DMGDH	green	0.19902415	-0.055552	0.85143964	0.2609214	0.13549598	0.53963009
cg23952952	CTR9;CTR9	green	-0.0177184	0.0559026	-0.4179388	-0.0699197	-0.1025501	0.04190038
cg01816720	GALNT13;AC009227.2;GAL	green	0.1709526	-0.0039435	0.87445492	0.24470811	0.13129711	0.46087691
cg20988589	DUSP19;DUSP19;DUSP19	green	0.11104023	0.01776553	-0.5561093	0.00625552	0.20315256	-0.2150245
cg21075228	RP11-65D13.1;RP11-	green	0.01550476	0.09652289	0.69853886	0.13463364	-0.1725657	0.31695377
cg04847551	ADAMTS6;ADAMTS6;AD	green	-0.1659167	-0.0803439	-0.4648488	-0.1112854	0.04059217	-0.3581046
cg09063787	ITFG2;ITFG2;ITFG2;ITFG2;	green	-0.3060564	-0.1214361	-0.4229027	-0.2611895	-0.0372731	-0.3872419
cg22510266	CBLL1;CBLL1;CBLL1;CBL	green	0.15926251	-0.0823771	0.77351823	0.23002606	0.13459518	0.38848495
cg06852111	DNAJB6;DNAJB6;DNAJB6	green	0.16112805	0.04059258	0.88005043	0.23824367	-0.0109034	0.39342794
cg17355153	KIAA1462;KIAA1462	green	0.25617466	0.20838536	0.70479567	0.26223813	-0.1102182	0.61092579
cg22737985	H2AFY2;H2AFY2;H2AFY2	green	-0.1036957	-0.1575006	-0.5525876	-0.1389699	0.12441125	-0.1209555
cg22548804	PCOLCE2;PCOLCE2;PCOL	green	0.19422593	0.05648565	0.72855662	0.15119818	0.0175336	0.39001671
cg13448816	LRRC7;LRRC7	green	-0.2799863	-0.2586776	-0.4304534	-0.1339866	0.13292568	-0.4401151
cg27473842	RP1-296L11.1	green	-0.0009802	0.12166851	0.79349132	0.18129727	-0.1794845	0.25770297
cg18131837	MIR548U;HS3ST5;RP3-	green	0.04895618	0.04871925	-0.3717836	0.07660075	0.08867112	0.01448918
cg26682425	ANKRD40;ANKRD40	green	-0.3792728	-0.1052681	-0.5940507	-0.213488	-0.0980765	-0.3758953
cg24529144	LINC00301;LINC00301	green	0.17936141	-0.0211262	0.89412945	0.19728988	0.05846844	0.47694037
cg09739586	RP11-1E3.1	green	0.19144123	-0.0213054	0.81365616	0.23510858	0.12267763	0.49801106
cg18239738	RNPC3;RNPC3;RNPC3;RN	green	0.09669087	0.17225374	0.60698705	0.26930424	-0.0428696	0.19942474
cg09998787	U6;TXK	green	0.01490732	-0.0341169	0.72892493	0.23162906	0.0014968	0.24315117
cg20496695	GRIA2;GRIA2;GRIA2	green	0.09468223	-0.0496447	0.86076976	0.2162935	0.07054182	0.45650294
cg12441104	TNR	green	0.27700333	0.08595553	0.56797746	0.19629613	0.03701992	0.35389712
cg14552744	LAMP3;LAMP3;LAMP3	green	0.21819601	-0.0627131	0.61714221	0.30704371	0.27516381	0.21456822
cg01367204	KLF1	green	0.34754714	0.08972997	0.72750782	0.66366826	0.15287164	0.48907966
cg21826610	U4	green	0.30796475	0.11123932	0.7584445	0.2849196	0.0547346	0.38585482
cg10002564	HAS2-AS1;HAS2-	green	0.05938478	0.04429031	-0.5261005	0.04346139	0.11994389	-0.2100186
cg10156861	MFF;MFF;MFF;MFF;MFF;	green	-0.3309538	-0.1664107	-0.4681271	-0.2266457	0.0065193	-0.4092836
cg19547370	PCOLCE2;PCOLCE2;PCOL	green	0.11989336	-0.006581	0.67920857	0.2444801	0.0401261	0.18020678
cg10596956	OR52M1	green	-0.0278694	-0.1107546	0.54624551	0.16390136	0.04961356	0.05184533
cg18583918	RP3-359N14.2	green	0.2039122	-0.0697009	0.75943504	0.13450257	0.0913466	0.48168753
cg19051933	LSS;LSS;AP001468.1	green	0.20953945	0.00141109	0.74785127	0.19901061	0.13226883	0.11779236

cg11868507	CTXN3	green	0.25085352	0.11188305	0.53895777	0.23068814	0.09207583	0.2534264
cg26335127	LGALS3;LGALS3;LGALS3;	green	0.04751893	0.10443577	-0.5021571	0.06371168	0.12148728	-0.1773065
cg20303490	TECRL;TECRL;TECRL;TEC	green	0.1753323	0.01566934	0.88279934	0.22376659	0.08640343	0.51271428
cg01758993	SLC35C2;SLC35C2;SLC35C	green	-0.2456489	-0.0595699	-0.5558237	-0.1585137	-0.0035822	-0.2747656
cg05608612	RPS7;RPS7;RPS7;RPS7;RPS	green	0.14489617	-0.0326912	0.47273703	0.18524831	0.14434163	-0.0107996
cg23847843	ZSCAN23;ZSCAN23;ZSCA	green	0.22030404	-0.0755264	0.32315274	0.05683365	0.17712608	0.24293585
cg06510950	CCDC129	green	0.18511867	0.16248886	0.47404265	0.30628404	0.01231123	0.11662609
cg01883825	AC022415.1;ZNF788	green	0.15093487	0.12795751	0.38606462	0.30522767	0.12432248	0.13939493
cg17249170	EMX2OS;EMX2;EMX2	green	-0.2523231	-0.1967686	0.35454253	0.03726331	0.01213724	0.10846231
cg14872260	PDGFRA;PDGFRA;PDGFR	green	-0.0270807	-0.0830952	-0.3307843	-0.0418884	0.12003555	-0.0165673
cg02592999	RP11-100L22.2;RP11-	green	0.06011512	-0.0902215	0.82986348	0.17636491	0.07171354	0.3727759
cg11793796	ZNF891;ZNF10;ZNF10;ZNF	green	0.17809054	-0.0842377	0.74332458	0.24806367	0.16017823	0.29313296
cg24851818	WNT5B;WNT5B;WNT5B;W	green	-0.0016396	-0.002907	0.46349974	0.25867753	0.08467197	0.10082773
cg15212266	ALPL;ALPL;ALPL;ALPL;A	green	-0.1654194	-0.0360023	-0.5299617	-0.1487264	0.0563797	-0.2791335
cg17971798	MRPL13;MTBP;MTBP;MTB	green	0.18356038	-0.0975645	0.68691817	0.27954741	0.20914683	0.3880401
cg23943415	MSR1;MSR1;MSR1;MSR1	green	0.23176391	0.07910076	0.78376513	0.2302403	0.08323706	0.22834182
cg01172802	GRM7-AS3;GRM7-	green	0.13296006	0.05519803	0.803393	0.25425979	0.07029586	0.40109987
cg20819150	TFIP11	green	0.23998256	0.04491833	0.50110833	0.1792538	0.00460702	0.37558166
cg12965311	RP1-104O17.1;RP1-	green	0.218186	-0.0765282	0.87089116	0.21794916	0.15524806	0.58508976
cg19890459	HIF1AN;NDUFB8;RP11-	green	0.39301038	0.06457491	0.71128025	0.31353935	0.12624558	0.53371854
cg02098293	PRKAR2B	green	0.12346148	0.03906727	0.78841534	0.15654391	-0.0717019	0.44703355
cg14629189	CD2AP	green	0.34714824	0.01547685	0.83118308	0.33640391	0.16363827	0.58134147
cg00188503	CYP4V2;CYP4V2	green	-0.1160084	0.02510869	-0.3609118	-0.1457968	-0.1028375	-0.0560647
cg03612345	CCDC58;CCDC58	green	0.22244449	0.00367002	0.84899273	0.31544048	0.09075969	0.53278212
cg24305122	U6;PPA2;PPA2;PPA2;PPA2;	green	-0.0094379	-0.1116777	0.77211214	0.08414055	0.05708861	0.35695409
cg00528520	SLC25A46;SLC25A46;SLC2	green	0.30490189	0.00941892	0.84074788	0.3616344	0.13266526	0.54052475
cg24340565	VGLL4;VGLL4;VGLL4	green	0.24176708	0.10888115	0.70287919	0.13648879	-0.1108253	0.2994172
cg09191490	SNHG14;SNHG14	green	0.08203154	0.23091214	0.52390862	0.06884956	-0.1578515	0.34133085
cg03853987	CHST10;CHST10;CHST10;C	green	-0.004285	-0.100181	-0.3877894	-0.1102494	0.18858735	-0.2283999
cg16814895	VPS8;VPS8;VPS8;VPS8;VP	green	-0.4228438	-0.1265717	-0.6323871	-0.2809827	-0.054928	-0.5214002
cg24479317	CRISP1	green	0.15313153	0.19598204	0.81012843	0.17069226	-0.1843751	0.44226717
cg27428016	snoU13;DISP1;DISP1	green	0.0807716	0.01942378	0.8520672	0.24106931	0.00590139	0.23534237
cg02408103	CTDSP2;CTDSP2;CTDSP2	green	-0.1978427	-0.1284249	0.31509333	0.02878383	-0.043889	0.06675116
cg02508455	RP13-870H17.3;RP13-	green	0.32590717	0.29191121	0.56354633	0.50760334	0.1513677	0.40520434
cg21971465	C12orf76;C12orf76;C12orf76	green	-0.1578925	-0.1417578	-0.3427801	-0.1393201	0.06336435	0.01837214

cg06579736	ERVMER61-1	green	0.25893945	0.05031738	0.78883365	0.31341118	0.14948705	0.22928142
cg00020191	SYT1;SYT1;SYT1;SYT1	green	-0.0638821	-0.1012867	-0.7063455	-0.0738482	0.09489978	-0.3221576
cg10048824	CLNK;CLNK;RP11-61G19.1	green	0.41234484	-0.0052783	0.77201694	0.52675637	0.24950659	0.56493168
cg09349409	MIR192	green	0.20492044	0.13759195	0.35902997	0.1424613	-0.0629672	0.39790676
cg11155432	CDH9;CDH9;CDH9	green	0.19535605	0.14416553	0.61992396	0.22934319	-0.0577809	0.34877482
cg10435384	NAV2;NAV2;NAV2;NAV2;	green	0.04888589	0.13153657	0.68882586	0.07840088	-0.1106721	0.0255216
cg00897805	Y_RNA;CTD-2384A14.1	green	-0.0206769	0.15034742	0.77571148	0.1543371	-0.1814381	0.28528094
cg06479653	NAPG;NAPG	green	-0.179165	0.00018463	-0.5805856	-0.2256619	-0.0492386	-0.5029341
cg25423563	TGDS;TGDS	green	0.06110688	0.1125144	-0.4792799	0.04594371	0.04197518	-0.2734519
cg24803326	C10orf137;C10orf137;C10orf	green	0.21322874	-0.0689583	0.89342999	0.27436441	0.14546138	0.36089015
cg01483249	ATG3;ATG3;ATG3;ATG3;S	green	-0.1860168	-0.100272	-0.3830152	-0.1374033	0.05303874	-0.360146
cg00770784	CFDP1;CFDP1;CFDP1;CFD	green	0.09343467	0.00297432	0.32518939	0.27242458	0.07767773	0.28768322
cg00555094	NANP;RP4-694B14.5;RP4-	green	0.31702688	-0.0066507	0.76904365	0.24396409	0.07003231	0.55973531
cg01989857	DYNC2LI1;DYNC2LI1;DY	green	-0.1603395	-0.0338401	-0.4964604	-0.1479511	0.00535525	-0.1800403
cg27015942	RP11-802H3.2	green	0.0972302	0.09824208	0.80273419	0.22601122	-0.0810538	0.1785732
cg02134660	FAM83B	green	0.33299211	0.05661987	0.7580743	0.2794402	0.13859876	0.39940127
cg18872973	FAM50B;FAM50B;RP11-	green	0.24736256	0.18006007	0.41879041	0.26738027	0.12522438	0.36058813
cg08203569	ZNF836	green	-0.2852613	-0.0327806	-0.603942	-0.2389599	-0.0652336	-0.5155277
cg27357647	RNU6-57;NAA16	green	0.21791089	0.03834236	0.86469451	0.33635699	0.08912008	0.4466201
cg18057637	GTPBP1;GTPBP1;GTPBP1	green	0.39911251	0.02419347	0.66543458	0.3291071	0.21130323	0.43847633
cg14121003	RUNX1T1;RUNX1T1;RUN	green	0.315893	0.02066879	0.63996946	0.39013103	0.24173962	0.44920045
cg10631684	PLBD2;PLBD2;PLBD2;SLC	green	0.10693233	-0.0371521	0.72027428	0.14278425	0.01109665	0.13094062
cg24568245	ATM;ATM;ATM;ATM;AT	green	-0.1320499	0.02352616	-0.3479987	-0.1240845	-0.0508678	-0.2007142
cg02868222	SLC33A1;SLC33A1	green	-0.1597583	-0.1088198	-0.7247071	-0.1109427	0.11335431	-0.533682
cg17582199	SNAP23;SNAP23;SNAP23;	green	-0.1387365	-0.117401	-0.3090126	-0.1673887	0.02446873	0.01187136
cg09678707	DNAH5	green	0.17801879	0.17771829	0.82600938	0.19415032	-0.0861115	0.30940008
cg24373369	MTFMT;MTFMT;MTFMT	green	0.04541943	0.01652405	0.40686386	0.2536254	0.12111611	0.0931041
cg13559772	FRY;FRY;FRY	green	0.20517282	0.10862647	0.72001953	0.25928996	-0.0319191	0.42451625
cg03674061	KIAA0146;KIAA0146;KIA	green	0.29138469	0.05478825	0.71849065	0.23747281	0.09981044	0.30622408
cg08238791	RUNX2;RUNX2;RUNX2;R	green	-0.1698466	-0.1091951	-0.3053055	-0.0557496	0.05141028	-0.023071
cg00092426	UBR1;UBR1;UBR1	green	0.38421516	0.1565696	0.6474684	0.6933955	0.10558707	0.39449402
cg12695537	RP13-487K5.1	green	0.34436185	0.11495183	0.76876257	0.59193762	0.16285089	0.4038293
cg17428659	TMEM72;TMEM72;RP11-	green	0.33894512	0.17761469	0.78444086	0.23463593	-0.0320502	0.51813717
cg16268412	LYPD3;LYPD3	green	0.24418382	-0.0335213	0.4998185	0.3045401	0.16016065	0.51112259
cg20244343	ANGPTL6	green	0.22048606	0.03570101	0.79501094	0.23521101	0.02824265	0.47194012

cg24823791	GSTCD;GSTCD;GSTCD;GS	green	0.08038343	-0.0153344	0.78408947	0.150181	0.00039377	0.20755609
cg12098674	ATOX1;G3BP1;G3BP1;G3B	green	-0.2784843	-0.014983	-0.5055148	-0.20717	-0.1612324	-0.1633846
cg08928646	RP11-	green	-0.3023709	-0.0711632	-0.5245237	-0.2110393	-0.0800559	-0.4320937
cg21195162	XCL2	green	0.32719291	0.02629769	0.60167936	0.15223117	0.05114983	0.62198425
cg15949111	PPM1J;PPM1J;PPM1J	green	0.20207562	0.13282177	0.30573806	0.21555021	0.09572351	0.30601828
cg14612560	NFKB2	green	0.36076485	0.17142734	0.66164055	0.31397943	0.08107532	0.48535386
cg07678594	JUP;JUP;JUP;JUP;JUP;JUP;	green	0.29337775	0.26099737	0.46805426	0.27243212	-0.0426661	0.4597828
cg03371545	RP3-388N13.3	green	0.02748018	-0.0604166	-0.74654	-0.0271581	0.25522045	-0.2337471
cg13740574	LACTB;LACTB	green	0.33295309	0.07420814	0.82464974	0.33487376	0.09606589	0.37194526
cg10660865	GAPVD1;GAPVD1;GAPV	green	-0.2546472	-0.1466552	-0.4046839	-0.1913393	0.06622093	-0.4712708
cg13537346	COL6A3	green	0.25579065	0.02978483	0.63164079	0.18138049	0.13051864	0.23601495
cg07350977	CDH9;CDH9;CDH9	green	0.16650676	0.2047505	0.21134305	0.13035176	-0.1528037	0.20507797
cg13263208	C20orf62;C20orf62	green	0.41831376	0.26788062	0.69997965	0.36169274	0.06548118	0.42659485
cg06820725	RN5S259	green	0.25337626	-0.0116858	0.75477569	0.24702592	0.09668519	0.6255012
cg04263575	HGF;HGF	green	0.23472483	-0.0352563	0.80700045	0.24014884	0.1575212	0.4530434
cg12663039	C16orf45;C16orf45;C16orf45	green	0.1706284	0.20365209	0.46952545	0.16232242	-0.0945555	0.25760329
cg08661752	EML5;EML5;EML5;EML5	green	0.36933713	0.09833474	0.75899844	0.30245598	0.09439196	0.5720153
cg15371509	INTS3;INTS3;INTS3;INTS3;	green	-0.2190705	-0.1449278	-0.3208815	-0.1802127	0.0389714	-0.1895672
cg22635982	ARPC2;ARPC2;ARPC2;AR	green	0.33676586	0.0440088	0.81516229	0.25242057	0.096946	0.46874399
cg17748504	EGLN1	green	0.31529257	-0.0097427	0.55066188	0.19222074	0.23689581	0.19109678
cg08949856	TRAV39	green	-0.01284	-0.1263217	-0.6121015	0.03582395	0.357585	-0.2801951
cg12915421	AC109586.1	green	0.20652229	0.29190179	0.67395016	0.15210668	-0.2317256	0.36088062
cg22209520	ADORA2A;ADORA2A;AD	green	0.19685335	0.12439085	0.46092517	0.11082684	-0.0333342	0.46092782
cg11041457	PBRM1;PBRM1;PBRM1;PB	green	-0.1067899	-0.0766466	-0.4042998	-0.1538069	-0.0007532	-0.1051021
cg25922163	GGN;GGN	green	-0.1316699	-0.0328284	-0.3103503	-0.0760788	0.02865187	-0.0552172
cg09823492	FAM149A;FAM149A;FAM	green	0.30470915	-0.0083352	0.78560662	0.25122736	0.13928122	0.49676326
cg04634417	PCDHA8	green	0.2809377	0.11048146	0.40847452	0.14159638	0.0457725	0.2836542
cg07144984	ZBTB20;ZBTB20;ZBTB20	green	0.15891045	0.14849186	0.33068106	0.283474	-0.0794171	0.16057658
cg04773679	ECHDC1;ECHDC1;ECHDC	green	0.32851659	0.02931945	0.84233008	0.28991115	0.10815501	0.62341133
cg17872796	LARP7;LARP7;LARP7;LAR	green	0.02612095	-0.131095	0.51578613	0.10583823	0.12308297	-0.0179
cg25814283	TCTN1;TCTN1;TCTN1;TCT	green	0.26400695	0.07940224	0.67211868	0.13148876	-0.0297939	0.35749267
cg17302069	CHD9;CHD9;CHD9;CHD9;	green	0.22823911	0.05481755	0.6344679	0.22659422	0.05891571	0.12733175
cg20026499	RP11-770G2.4;OVOL1;RP11-	green	0.32371821	0.20805166	0.47602324	0.25362517	0.08240713	0.33771176
cg07476963	RABGAP1L;RABGAP1L;R	green	0.18078278	0.18175864	0.46847386	0.3467686	0.1166482	0.19570322
cg26902259	NCOA7;NCOA7	green	-0.2064899	-0.0766545	-0.3439694	-0.2298436	-0.0334238	-0.3654073

cg01040719	AL353997.1	green	0.38450701	-0.0206422	0.55224482	0.37789992	0.30441576	0.55464384
cg14540557	AC092958.1;RP11-639B1.1	green	0.41818252	0.09707158	0.82336881	0.34624822	0.12526104	0.6493645
cg09797892	ZNF254;ZNF254;ZNF254	green	0.17621968	-0.0148899	0.70934029	0.18611527	-0.0266616	0.54409084
cg01983105	NFKB1;NFKB1;NFKB1;NF	green	0.27681745	0.06742978	0.79367038	0.230765	0.03436172	0.47238889
cg26043240	AL451164.1	green	0.46988108	0.17820837	0.75678963	0.33262117	0.0933047	0.62124466
cg19527543	FRYL;FRYL	green	0.29105596	0.00348458	0.75382391	0.23566425	0.11371907	0.66816308
cg26103369	KIAA0317;KIAA0317;KIA	green	0.54005547	0.19259889	0.52070189	0.76327597	0.16652571	0.39573081
cg26785346	SORBS2;SORBS2;SORBS2;	green	0.09027044	0.11946268	0.34805635	0.11489972	-0.0292846	0.22386956
cg22987789	TMEM8A;TMEM8A;TMEM	green	0.3430069	0.26243472	0.56871766	0.29756485	-0.1450758	0.46092156
cg18597415	CLC	green	0.42252012	0.29584173	0.57930778	0.52819531	-0.0413186	0.33712019
cg05362677	LINC00163;LINC00163;AP0	green	0.26808696	-0.0091524	0.63244582	0.16070231	0.06624548	0.38517175
cg03668051	PSMA1;PSMA1;PSMA1;PS	green	0.40601314	0.0734134	0.7928298	0.33736549	0.09737661	0.53873681
cg06369005	CDH9;CDH9;CDH9	green	0.2032691	0.25844673	0.09806634	0.12059973	-0.1467242	0.13550025
cg14784877	GALNT5;7SK	green	0.16695784	0.34330431	0.72953697	0.17720738	-0.2447954	0.19695033
cg06376718	C1orf101;C1orf101;C1orf101	green	-0.1258276	-0.0315034	-0.3165433	-0.1865115	-0.0133747	-0.2541741
cg26566848	SEZ6	green	0.19424009	0.00056274	0.279847	0.05214903	0.10799507	0.27782219
cg11111952	CDK19;CDK19	green	0.35893486	0.05293876	0.79309071	0.3707845	0.13706208	0.44759132
cg25145979	ACADL;AC006994.2	green	0.18105953	0.11206948	0.85049657	0.23193644	0.02054887	0.29615408
cg00404142	NADSYN1;NADSYN1;NA	green	0.28075329	0.09515074	0.6113665	0.21926438	-0.0696236	0.34717817
cg21220750	TRAF3IP2;TRAF3IP2-	green	0.40285626	0.15271854	0.75984411	0.38946404	0.03941704	0.51601238
cg02794150	RP11-644F5.16	green	0.36972666	0.06350589	0.74050502	0.28130419	0.11037138	0.49899072
cg14456900	MRPS24;MRPS24;MRPS24;	green	-0.1232191	-0.297624	-0.6005599	0.01247629	0.39890474	-0.3367752
cg25625233	MYSM1;MYSM1;MYSM1;	green	0.17597028	0.00979926	0.51393865	0.21124379	0.0111855	0.35740147
cg06421734	RIF1;RIF1	green	0.555072	0.18958496	0.5182458	0.7112187	0.1853702	0.4569606
cg06911238	FAAH;FAAH;FAAH;FAAH	green	0.36969509	0.0930557	0.58168354	0.28933201	0.14212735	0.24461737
cg24906129	RP3-495K2.3	green	0.3414255	0.10351705	0.81000169	0.25229765	0.05158268	0.439208
cg12864235	CDH9;CDH9;CDH9	green	0.29308347	0.28732811	0.00545056	0.1158153	-0.1332481	0.1747445
cg19772066	ZNF229	green	0.24821465	0.32974185	0.58508061	0.12261903	-0.2269238	0.33713347
cg05403411	TIAL1;TIAL1;TIAL1;TIAL1;	green	0.4527504	0.18193411	0.73889539	0.35674694	0.05770697	0.49951298
cg21351219	SGOL1;SGOL1;SGOL1;SG	green	-0.3161695	-0.0943611	-0.6151912	-0.2811385	-0.0300662	-0.4114898
cg05327268	TBRG4;TBRG4;TBRG4;TBR	green	0.30132744	0.15984147	0.73955748	0.16625563	-0.0586345	0.29499543
cg14976170	AC005008.2	green	0.54461207	0.42118314	0.69447203	0.37399136	-0.0624715	0.48177118
cg02477395	TM7SF4;TM7SF4;TM7SF4;	green	0.21975406	0.40698399	0.72377844	0.17535889	-0.3459873	0.34993861
cg21631918	STRA6;STRA6;STRA6;STR	green	0.12063011	0.12591929	0.46502732	0.15071506	-0.0998575	0.21892429
cg19305527	CUL3	green	0.58184443	0.24012583	0.6581425	0.55319994	0.07978563	0.48054577

cg02653559	ZIC4;ZIC4;ZIC4;ZIC1	green	-0.5270692	-0.3411671	-0.6557637	-0.2721391	0.14346693	-0.57629
cg17430535	DNAH12;DNAH12;DNAH1	green	0.49207799	0.1570852	0.76862466	0.30052689	0.06011141	0.63684227
cg16964521	CTPS;CTPS;CTPS	green	0.20589464	0.22137163	0.41032472	0.13252648	-0.1802878	-0.0281683
cg04131958	RP11-277P12.7;RP11-	green	0.28770659	0.23878854	0.70910403	0.25245675	-0.0753523	0.29553422
cg12052101	CCNB1IP1;CCNB1IP1;CCN	green	0.41328	0.09854368	0.63046957	0.30939461	0.09094367	0.53882571
cg21092030	ASPG;ASPG;ASPG;ASPG	green	0.14549773	0.16162129	0.32749248	0.09302091	-0.0972832	-0.0085896
cg02160692	FBXO11;FBXO11;FBXO11;	green	0.60081135	0.24231856	0.67039074	0.26216368	0.00108419	0.58407215
cg11366178	LNX1;LNX1-AS2;LNX1-	grey	-0.2960838	-0.3207867	0.17153996	0.13371126	0.30046935	0.01307495
cg05186787	PRUNE2;PRUNE2;PRUNE2	grey	-0.1254865	-0.0128957	0.17960597	0.05529799	-0.0358976	0.11494022
cg22025478	RP11-	grey	-0.1445742	-0.3328432	0.19450028	0.18356584	0.32057373	0.01834613
cg12954619	GANAB;GANAB;GANAB;	grey	-0.2436224	-0.2062809	0.20813731	0.3012021	0.17660537	0.21542301
cg12463140	GLT25D1;GLT25D1	grey	0.15720193	0.23795423	-0.2160481	-0.1531437	-0.1196249	-0.1338066
cg01914428	SHANK2	grey	-0.0920179	-0.1594075	0.19746011	0.11173905	0.27072306	0.17490593
cg20397086	SIRT7;SIRT7;SIRT7;SIRT7;	grey	0.13419903	0.14606853	-0.3080965	-0.0612378	-0.1944049	-0.0597283
cg23332328	CYP2W1;CYP2W1;CYP2W	grey	-0.0541331	0.02226817	0.3264508	0.19664466	0.1067459	0.21569376
cg12474691	C1orf61;C1orf61;C1orf61;C1	grey	-0.2797579	-0.3160528	0.19932132	0.08356317	0.27823178	0.07250837
cg03832374	MAGI2-AS3;MAGI2-	grey	0.21557068	0.26388003	-0.3332275	0.01356905	-0.1523338	-0.290798
cg24765602	HSPA2;HSPA2;HSPA2;RP1	grey	-0.1813168	-0.2529442	0.16560279	0.19839836	0.22976413	0.19468031
cg00026554	NXF1;NXF1;NXF1;NXF1;N	grey	-0.2840197	-0.137818	0.2342248	0.22921703	0.12685259	-0.0203149
cg01097465	KCNH2;KCNH2	grey	-0.2167313	-0.1282726	0.25183534	0.19874865	0.15965548	0.09268522
cg03218370	OLFML2A;OLFML2A	grey	-0.3589509	-0.318394	0.21668628	0.08158835	0.23057124	-0.0092796
cg24136700	RAI1;RAI1;RAI1	grey	-0.3606746	-0.3564509	0.28873006	0.08708504	0.15773626	0.06559705
cg05766064	MIR320C1	grey	-0.3043549	-0.2831989	0.12082775	0.03957838	0.19902215	-0.02485
cg04507075	C20orf26;C20orf26;C20orf26	grey	0.19555129	0.190568	-0.134377	-0.115111	-0.1595121	-0.0585201
cg24777028	RNF14;RNF14;RNF14;RNF	grey	-0.2158131	-0.2491472	0.23997314	0.15246024	0.14670296	0.23179352
cg25764931	CACNG6;CACNG6	grey	0.33969021	0.32927645	-0.0509753	0.086159	-0.1005454	0.03259292
cg18699242	USP18	grey	-0.2626651	-0.3250416	0.05226976	0.06578463	0.24085118	4.17E-05
cg18618355	ZNF599;ZNF599	grey	-0.3320648	-0.3069795	0.03445981	-0.0722263	0.10873395	-0.0305974
cg13476950	DNAH1;DNAH1;DNAH1	grey	-0.1389802	-0.3071567	0.21208176	0.11119784	0.31338104	0.12987964
cg03780875	IDH2;IDH2;IDH2;CTD-	grey	-0.1612593	0.05149964	0.10803806	0.16653302	0.04807303	-0.0749371
cg06634862	LNX1;LNX1-	grey	-0.0698747	-0.1384759	0.17844424	0.06993351	0.09974896	0.05950038
cg05570491	HIVEP3;HIVEP3;HIVEP3;H	grey	-0.1256039	-0.1769727	0.13725512	0.09691185	0.14676649	-0.0479459
cg11769885	HIVEP3;HIVEP3;HIVEP3;H	grey	-0.0979698	-0.1284484	0.13672319	0.10186602	0.10065317	-0.0293527
cg21137052	RPL3;RPL3;RPL3;RPL3;RPL	grey	-0.1487151	-0.1787556	0.07524254	0.07717959	0.07302848	0.02220911
cg17345081	NMBR;RP11-137J7.2	grey	0.10167978	-0.0590708	-0.158125	-0.0301515	0.12597708	-0.0132434

cg20039814	TP53INP1;TP53INP1;RP11-	grey	-0.1425006	-0.3771105	0.19709982	0.04817035	0.32824218	0.18929939
cg13502414	C17orf51;C17orf51;C17orf51	grey	-0.1801764	-0.1454247	0.08907974	0.19286812	0.09352296	0.08174574
cg18161904	RP1-222E13.10;RP1-	grey	-0.3684349	-0.31495	0.30172344	0.11258714	0.24713404	-0.0422845
cg04434328	PLEKHG5;PLEKHG5	grey	-0.2159629	-0.2396298	0.20990381	0.13643768	0.25072756	0.04275383
cg10242541	ESRRB;ESRRB	grey	-0.4016742	-0.3801023	0.30993052	0.05178368	0.13392642	-0.0750249
cg00006301	ANK3;ANK3;ANK3;ANK3	grey	-0.1344681	-0.1864253	0.14840583	0.07340098	0.1981616	0.06427786
cg01347529	EPHX4	grey	-0.2549628	-0.0809725	0.27938212	0.03061929	-0.0312703	-0.0290733
cg19943580	ZNF385C;ZNF385C	grey	-0.2116552	0.0117456	0.42086554	0.2242047	0.12555823	0.1983076
cg07448457	IL34;IL34	grey	-0.2948371	-0.1717832	0.36814476	0.08652637	0.17588496	0.04665364
cg21442335	ANO4;ANO4;ANO4;ANO4;	grey	-0.2915096	-0.3470714	0.4280079	0.05121347	0.16793103	0.08482515
cg03699904	SLC2A2;SLC2A2	grey	-0.3065691	-0.3269308	0.17209047	0.00506283	0.23623912	0.02022979
cg21968765	ABI3BP;ABI3BP;ABI3BP;A	grey	-0.209394	-0.0450482	0.07348458	0.266685	0.14124209	0.00943642
cg17679621	SLC5A7;SLC5A7;SLC5A7	grey	-0.2051376	-0.2811443	0.22374116	0.13584293	0.2562633	0.09254799
cg07019075	AC017116.8;AC017116.8;A	grey	-0.1951147	-0.247532	0.02344727	0.17223825	0.26619658	0.0394694
cg11251215	RNF220;RNF220;RNF220;R	grey	0.05494951	-0.066978	0.38602274	0.35496729	0.1984983	0.22402475
cg04791732	TTC39B;TTC39B;TTC39B	grey	-0.2398354	-0.1393379	0.10292823	0.02180497	0.21678257	-0.0777436
cg00359661	BMP6;BMP6;BMP6	grey	-0.325553	-0.2904358	0.21686784	0.20669705	0.21418521	-0.0184125
cg05075236	RP1-293L8.2;RP11-	grey	0.1237481	0.21268115	0.2362204	-0.0068575	-0.1893016	-0.1729304
cg17042037	INPP5K;INPP5K;INPP5K;I	grey	-0.0109629	-0.2598207	0.05902051	0.18330346	0.26933875	0.25668189
cg21717724	RP11-27I1.2;PSMD5	grey	0.16029694	0.25357419	-0.0732484	-0.1717244	-0.2302794	0.07782005
cg06621309	HPCAL1;HPCAL1;HPCAL1	grey	-0.1561879	-0.0340381	0.08327717	-0.0722565	-0.0875538	0.13079372
cg12308054	HSPA4;HSPA4	grey	0.15022035	0.28895417	-0.0051928	-0.1706024	-0.2372893	0.02713445
cg02271488	MAST1;MAST1	grey	0.39901006	0.36170968	-0.1055789	0.12270512	-0.1238209	-0.0434744
cg12451679	ZC3H4	grey	-0.3580859	-0.3549974	-0.0226716	0.02679497	0.20201254	0.0221219
cg05777089	FAM5C;FAM5C;FAM5C;F	grey	-0.3965875	-0.3507848	0.28058925	-0.0524346	0.12883134	-0.0360095
cg11280542	HOTAIR;HOTAIR;HOTAIR	grey	-0.2791967	-0.253214	-0.0055153	0.08115269	0.15347649	0.04670367
cg10558013	ZNF503;ZNF503-	grey	-0.1006677	-0.0859551	0.09913214	0.08243012	0.18030478	0.19370459
cg17035174	NELL2;NELL2;NELL2;NEL	grey	-0.2169633	-0.2293989	0.40509692	0.16192073	0.29368063	0.07620206
cg13595604	VIP	grey	0.07348495	0.14222704	-0.0805322	-0.0653232	-0.1872933	-0.1135302
cg20632567	MDS2	grey	0.01891652	-0.0521233	0.29052103	0.30902743	0.26217582	0.08773998
cg21698295	RP11-53M11.3;RP11-	grey	-0.0505515	0.1132599	0.20666416	0.18081844	-0.0095394	0.05924357
cg00433654	INO80B;INO80B;INO80B;I	grey	-0.3904437	-0.3394261	0.20627647	0.02915407	0.2431218	0.02660805
cg03884592	HIVEP3;HIVEP3;HIVEP3;H	grey	-0.1004949	-0.1351959	0.11238024	0.10593543	0.11418649	-0.0236149
cg13408347	C12orf12;C12orf12;C12orf12	grey	-0.211391	-0.22426	0.17310761	0.03416037	0.14559354	0.0401922
cg14929207	DHRS13;DHRS13	grey	-0.2861383	-0.0957342	0.25606529	0.00190608	-0.0524019	0.11283433

cg03044200	IGLV3-32	grey	-0.1473729	-0.1777158	0.1925212	0.15694896	0.29073917	0.0723012
cg00851518	SYTL1;SYTL1;SYTL1;SYT	grey	-0.0106658	-0.1096966	0.16610464	0.0385867	0.20935449	0.11191611
cg11487037	MATR3;MATR3;MATR3;M	grey	-0.4128653	-0.3782753	0.19045611	0.06649491	0.16164048	-0.0062298
cg04474832	ABHD14B;ABHD14B;RP11-	grey	-0.4498603	-0.2163198	0.25023047	0.22230552	0.26671435	-0.1424971
cg19562969	C20orf24;C20orf24;C20orf24	grey	-0.2422403	-0.2984988	0.19026068	0.18280538	0.27437235	0.04494184
cg11742103	MTA2;MTA2;MTA2;EML3;	grey	-0.092119	-0.1172534	0.05589777	0.00784562	0.07235361	0.10326147
cg19880015	RP11-574O7.1	grey	-0.2683667	-0.1452726	0.18846452	0.10044459	0.24371755	0.03576727
cg07836518	RP3-523C21.1	grey	-0.3631302	-0.3143686	0.25935594	-0.0703643	0.15179875	-0.0634699
cg12219789	NEURL4;NEURL4;NEURL4	grey	-0.0031391	-0.0918679	0.24996498	0.19104479	0.29893817	0.05277122
cg27127492	C8orf38;C8orf38;C8orf38;C8	grey	-0.2426711	-0.2548805	0.24473601	0.08490549	0.18702432	0.19875238
cg14921939	MATN4;MATN4;MATN4;R	grey	-0.3188603	-0.3670945	0.07577384	0.09863487	0.33102864	-0.051307
cg20504242	FAH;FAH;FAH;FAH	grey	-0.1119103	-0.0248201	0.35911361	0.21010803	0.17118042	0.13582634
cg05913141	PEX11A;PEX11A;PEX11A;	grey	-0.2451154	-0.1609773	0.31562957	0.27549865	0.19084078	0.06358764
cg07112210	MGAT5B;MGAT5B;MGAT	grey	-0.1387626	-0.1975695	0.2407584	0.28766245	0.26962115	0.1342118
cg05593411	PDE4D;PDE4D;PDE4D;PDE	grey	-0.2499172	-0.3314601	0.26615461	0.11785287	0.21029654	0.04691885
cg11145399	TP53BP2;TP53BP2;TP53BP	grey	-0.0906526	-0.0341852	0.16328371	0.19363137	0.15607756	0.05806008
cg13939777	RP11-358H18.3;RP11-	grey	0.09490396	0.24262136	-0.2732165	-0.2648887	-0.1248127	-0.0957848
cg07596299	C11orf51;C11orf51;C11orf51	grey	-0.3568592	-0.3108472	0.19401814	0.04323986	0.2377843	-0.1061584
cg12793044	MEGF6;MEGF6;MEGF6;M	grey	0.29336934	0.31246368	0.26366754	0.08580407	-0.1240587	0.04181878
cg06988187	BVES;BVES;BVES;BVES-	grey	-0.21128	-0.2166716	0.15134275	0.19598106	0.17671637	0.09038961
cg08196561	RP11-	grey	0.01198003	-0.106187	0.26825638	0.19326813	0.12807015	0.30359771
cg03949642	CLUAP1;CLUAP1;CLUAP1	grey	-0.25487	-0.284455	0.23316406	0.11110284	0.1687891	0.07791283
cg03168947	TRAK1;TRAK1;TRAK1;TR	grey	-0.1746882	-0.1884069	0.19807009	0.17103739	0.19762654	-0.028156
cg08174179	ZNF749;ZNF749	grey	-0.2642158	-0.3335673	0.1562845	-0.0502682	0.18857734	-0.0506262
cg08793894	NEURL4;NEURL4;RP11-	grey	-0.2022692	-0.0357161	0.27587704	0.03477646	-0.0201895	0.00053507
cg22333888	ADAM33;ADAM33;ADAM	grey	-0.3031989	-0.2673755	0.01268392	0.1091512	0.07346153	0.01764541
cg01650399	PCNXL3	grey	-0.2011978	0.09717804	0.36113721	0.14533494	-0.062873	0.17006877
cg22882506	ITPRIP;ITPRIP	grey	-0.1212435	-0.1132167	0.43003687	0.42505973	0.2003824	0.1497072
cg21982982	XX-C283C717.1;FAM116B	grey	-0.0083679	0.0066889	0.14058109	0.23675622	0.18714421	0.10475983
cg23743252	RNF14;RNF14;RNF14;RNF	grey	-0.2676171	-0.3581755	0.22894129	0.14628497	0.2978195	0.06222934
cg25883020	NKX6-3	grey	0.29491117	0.25613728	-0.1093205	0.05797161	-0.0846024	0.09125301
cg07862738	SERPINA5;SERPINA5;SER	grey	-0.3165924	-0.3484355	0.18371384	0.04525663	0.27803518	0.00595106
cg11045303	GNPNAT1;GNPNAT1;GNP	grey	-0.292538	-0.3160036	0.1275326	0.09099927	0.23158968	0.04688825
cg23333501	FAM108A1;FAM108A1	grey	0.17878526	0.15939661	-0.2806721	-0.0462276	-0.0475057	0.00589776
cg12614318	PRLHR;PRLHR	grey	-0.2853557	-0.2466838	0.32876135	0.10989285	0.15168402	0.07460025

cg14159576	AC095067.1	grey	0.28762462	0.31993627	-0.2407599	0.00712666	-0.253847	0.03505105
cg18335505	FAM123C;FAM123C;AC14	grey	-0.2442728	-0.2707922	0.14294565	0.14989334	0.21843602	0.15460807
cg17720880	SLC35B2;SLC35B2;SLC35B	grey	-0.2964742	-0.1645441	0.38317161	0.16096166	0.19426438	0.00688878
cg16676775	KCNK1;KCNK1	grey	0.31081233	0.31291862	-0.1479803	-0.071375	-0.1384692	-0.0459053
cg12474759	HIST1H4B	grey	-0.0847988	-0.009545	-0.2788755	-0.0733921	-0.0289735	-0.0048021
cg02998698	SS18;SS18;SS18;SS18;SS18	grey	-0.3282885	-0.3124291	0.15772189	0.10305537	0.27614086	-0.0055444
cg18716679	IDO2;RP11-44K6.3;IDO2	grey	-0.234445	-0.2759858	0.28684949	0.04914483	0.16817016	0.12238222
cg18829521	KIAA1217;KIAA1217;KIA	grey	0.21285206	0.2480006	-0.3131524	0.03222627	-0.0526896	-0.0263902
cg25028289	SLC11A2;SLC11A2	grey	-0.1296984	-0.1660597	-0.0212583	0.32084372	0.20308286	0.03063586
cg23957311	ZIC4;ZIC4;ZIC4;ZIC4;ZIC4;	grey	0.30627988	0.25814243	-0.2456237	-0.050251	-0.1614827	-0.083718
cg13033722	PTK7;PTK7;PTK7;PTK7;PT	grey	-0.2132569	-0.1696056	0.09635143	-0.0678534	0.04173795	0.08700339
cg07904727	RP11-77I22.3;RP11-	grey	-0.2226985	-0.2583777	0.27843795	0.19445532	0.24859548	0.02995276
cg27247554	IZUMO2;IZUMO2;IZUMO2	grey	-0.0317658	-0.1920276	0.26594249	0.10095685	0.22139724	0.32850135
cg07164659	BIN3;BIN3;BIN3;BIN3;BIN	grey	-0.1368245	0.07139802	0.13646439	0.08471833	-0.1427492	0.06085271
cg08347626	CTD-2194D22.1	grey	-0.0060441	-0.0256507	0.07712427	0.09956514	0.06470107	0.17723351
cg09300179	SEMA7A;SEMA7A;SEMA7	grey	-0.2587231	-0.1501975	0.31903831	0.1851116	0.25171884	0.12907981
cg02946570	C2orf43;C2orf43;C2orf43;C2	grey	0.1648236	0.14000977	-0.1330703	-0.0974481	-0.0459942	-0.0877592
cg06809604	YAP1	grey	-0.2580193	-0.2990592	0.28226082	0.14292618	0.18132491	0.06525025
cg04601137	ADAMTSL5;ADAMTSL5;A	grey	-0.2643657	-0.303657	0.32215641	0.04588899	0.21576191	0.1275299
cg04845579	SULF1;SULF1;SULF1	grey	-0.173442	-0.1527551	0.19329614	0.15771421	0.13472538	0.08652339
cg25631853	TTC9B	grey	0.25251133	0.31099829	-0.1288567	-0.0127382	-0.0839031	-0.0457924
cg24284752	VPS28;VPS28;VPS28;VPS2	grey	-0.3032548	-0.2375349	0.19995742	0.23967425	0.26014035	-0.0980151
cg25336746	CYP2J2;CYP2J2;CYP2J2	grey	-0.2074053	-0.0809248	0.19330266	0.22149878	0.21906278	0.04301534
cg06341179	ATL1;ATL1;ATL1;ATL1;AT	grey	-0.2051324	-0.1279499	0.01626705	0.14274919	0.23437816	-0.0074146
cg07298431	PRDM10;PRDM10;PRDM1	grey	0.02273457	-0.1340822	0.33672247	0.29272802	0.22591494	0.35061356
cg21016666	RP11-332J15.3	grey	-0.3045903	-0.0425288	0.29399793	0.15223652	0.15794686	-0.1133862
cg00982984	CTD-	grey	-0.3817229	-0.2930675	0.19942596	0.09563065	0.24453486	-0.0678831
cg22862634	MTA2;MTA2;MTA2;EML3;	grey	-0.0799606	-0.0937452	-0.0181021	0.00995138	0.0985713	0.04895757
cg08743812	CARTPT;CARTPT	grey	-0.1409777	-0.2182832	0.25059981	0.12357878	0.2465441	0.02965457
cg11712936	CNTNAP5;AC079154.1	grey	-0.2874229	-0.3590364	0.25096234	-0.018416	0.23845948	0.02494849
cg26796245	ANK2;ANK2;ANK2;ANK2;	grey	-0.4153414	-0.3702288	0.34355572	0.05973321	0.14961729	-0.0419118
cg12651412	HIST1H2BC;HIST1H2AC	grey	0.10982065	0.23198479	-0.3114918	-0.1491951	-0.2028052	-0.0969995
cg24942142	FAM57B;FAM57B;FAM57	grey	-0.0527313	-0.1458226	0.38145827	0.35990078	0.19932601	0.1646308
cg09955450	CTNND2;CTNND2;CTNND	grey	-0.1644951	-0.1429947	0.18897186	0.00299527	0.14405755	0.0133734
cg13073141	CERS2;CERS2;CERS2;CER	grey	0.04957444	0.07678788	0.10553025	0.34975162	0.23239827	-0.0724036

cg26120476	SNX30;RP11-276E15.4	grey	-0.2383942	-0.1362288	0.15498898	0.02126746	-0.0974532	0.15537856
cg07034329	MAST4;MAST4;MAST4	grey	-0.342182	-0.3044229	0.09826012	0.03669907	0.10566665	0.02814989
cg18753958	COL9A3;COL9A3	grey	-0.1357344	-0.2748093	0.26657761	0.08888483	0.26100247	0.15205386
cg19921829	POLR2D;POLR2D;POLR2D	grey	-0.3439271	-0.353063	0.2369316	0.1190393	0.27290175	-0.0226741
cg01996091	CCDC94	grey	-0.3125551	-0.3478104	0.16735706	0.07604656	0.25781968	0.04985613
cg10088405	SCHIP1;SCHIP1;SCHIP1;S	grey	-0.1869541	-0.174706	0.15891489	0.18844091	0.16550579	-0.047694
cg03668469	RP11-290F20.1	grey	-0.0045823	0.14015	0.13750255	0.33838236	0.10451678	0.01165048
cg18852096	MIB2;MIB2;MIB2;MIB2;MI	grey	-0.1545298	-0.10186	0.25474431	0.04238496	0.02779097	0.17826961
cg10031516	CTD-2506J14.1;CTD-	grey	-0.3227302	-0.3148113	0.16948598	0.07906638	0.27709432	0.07671356
cg21205305	C19orf76	grey	0.02816331	-0.0626732	0.25704106	0.22849749	0.2573703	0.27637573
cg08491582	TMOD1	grey	0.29499464	0.20193296	0.08370563	0.46947935	0.05138847	0.40691851
cg21387281	OGFR-AS1;OGFR	grey	-0.3230556	-0.3111429	0.21517031	0.01705511	0.19333994	-0.0151337
cg02608124	MRGPRX4	grey	0.27510528	0.27283221	-0.1356821	0.05325298	-0.0416661	0.0427732
cg13092523	MSC;MSC;RP11-	grey	-0.3726761	-0.3728471	0.17680739	0.0511914	0.23614662	-0.0456875
cg19492274	TM9SF3;TM9SF3	grey	0.02748424	0.06455166	0.18863131	0.23396909	0.0211817	0.2280683
cg12218079	PTCHD4;PTCHD4;PTCHD4	grey	-0.1067203	-0.0450854	0.17240589	0.157379	0.19490073	0.12349681
cg01963623	USP18	grey	-0.1798137	-0.2488702	0.0617351	0.06403096	0.20864011	-0.0399988
cg04255201	AC011484.1;AC011484.1;PN	grey	-0.3906731	-0.3435094	0.20794508	-0.0819768	0.02136259	0.02778634
cg19895135	IFT140;TMEM204;TMEM20	grey	-0.1209775	0.12097312	0.26287536	0.06421032	0.05851275	0.04583458
cg20879768	TCEB3B;KATNAL2;KATN	grey	0.34352032	0.32769123	-0.1129053	-0.0784575	-0.2566366	0.14640999
cg11267527	CXCL12;CXCL12;CXCL12;	grey	-0.2992189	-0.2850806	0.21172146	0.07251742	0.19051419	0.02616864
cg05377391	IFT172;IFT172;IFT172;IFT1	grey	-0.3360646	-0.3064236	0.1240338	0.09707386	0.22179425	0.02026548
cg24923430	MFHAS1;MFHAS1;MFHA	grey	0.09160411	0.14851011	-0.074544	0.04748693	-0.0871425	-0.1530178
cg00217859	NOB1;NOB1;NOB1;NOB1;	grey	0.25446073	0.32200444	0.04213473	-0.0248906	-0.1768433	-0.0335792
cg24177832	BVES;BVES;BVES;BVES-	grey	-0.0597822	-0.2808722	0.06943741	0.25192025	0.31754481	0.23141258
cg00655184	CTAGE1	grey	-0.1557986	-0.2698061	0.19410763	0.01850678	0.20601455	0.06490021
cg09444160	LARP1B;LARP1B;LARP1B;	grey	-0.1060658	-0.167521	-0.0836633	0.12465324	0.18667509	-0.0095979
cg01669689	EXO1;EXO1;EXO1;EXO1;E	grey	0.07081928	0.15025687	-0.0210203	-0.0226408	-0.1594981	-0.1032199
cg03959515	RP11-89K10.1;RP11-	grey	-0.1690039	-0.2838926	0.22779998	0.0760866	0.30346449	0.20247839
cg15627771	AC092614.2;MYO1B;MYO1	grey	-0.3392739	-0.3237383	0.37582529	-0.063281	0.14075862	-0.0436718
cg17481219	CYP2U1;CYP2U1;CYP2U1;	grey	-0.3316329	-0.3275098	0.06447834	0.1178973	0.28086222	-0.1140706
cg05573014	RP11-244K5.4	grey	0.17163617	0.15422254	-0.3214607	-0.1722883	-0.0525785	-0.039449
cg23979598	NPM2;NPM2;NPM2;NPM2;	grey	-0.2114269	-0.1586871	0.29453336	0.19625213	0.22088635	0.13532737
cg22519184	RP5-857K21.5;RP5-	grey	0.19192365	0.25163792	-0.0501332	-0.0491105	-0.2788793	-0.013957
cg07062043	PHYHIPL;PHYHIPL;PHYH	grey	0.20252915	0.07615928	-0.2729487	0.04982415	0.03727551	-0.0540695

cg14624668	RAMP2;AC100793.1	grey	-0.1235439	-0.1204025	0.17206125	0.22816342	0.27598177	0.25415476
cg23884418	RAB8A	grey	-0.0913827	-0.1320911	0.22288273	0.25019753	0.21855832	0.1371344
cg20434856	MYO10;MYO10;MYO10;M	grey	-0.0390873	-0.2720547	0.25540327	0.1979439	0.26648536	0.25167546
cg17828445	MARCH9;MARCH9;CDK4	grey	-0.3109978	-0.2689011	0.30882315	-0.0425062	0.22285319	-0.0525583
cg09805252	RP13-554M15.6;RP13-	grey	-0.2034462	-0.2098096	0.09289972	0.09056416	0.16494385	0.1043101
cg01730072	AC144450.2	grey	0.26277071	0.24728939	-0.3198205	-0.047891	-0.0454527	-0.0374985
cg06569300	SF3B2;SF3B2;GAL3ST3;GA	grey	-0.1602518	-0.2947836	0.2038278	0.20418169	0.2742451	0.15761251
cg21907521	FUT1;FUT1	grey	-0.2377402	-0.136907	0.30653493	0.03744064	0.19091037	0.05188508
cg22928599	AC011484.1;AC011484.1;PN	grey	-0.3325811	0.04438064	0.32598759	0.07729999	-0.1001898	-0.0259246
cg24940007	RP11-418I22.2	grey	0.3319119	0.32556812	-0.2286761	-0.0382299	-0.090568	0.12300125
cg13393036	TP53INP1;TP53INP1;RP11-	grey	-0.1245292	-0.366536	0.19373496	0.05324033	0.33314994	0.18122159
cg01937840	CTNND2;CTNND2;CTNND	grey	-0.0748528	-0.1664929	0.10926204	0.07807166	0.24018386	0.0432354
cg06607307	RP11-798K23.1	grey	-0.1669561	-0.2761269	0.29975825	0.25640267	0.24186182	0.36225735
cg01921918	KLHL23;KLHL23;KLHL23	grey	-0.3901297	-0.3765103	0.21522312	0.11947934	0.27900484	-0.1084114
cg10052118	MMD;MMD;MMD	grey	-0.2421217	-0.2272589	0.12932538	0.28066504	0.2029522	-0.0194688
cg12037819	ABCC1;ABCC1	grey	0.00658847	-0.0556564	0.22041591	0.24675284	0.19435902	0.17096334
cg00624400	AC116553.1	grey	0.03664378	0.02729323	0.24489808	0.2933732	0.1238512	0.19597764
cg10578938	CYFIP2;CYFIP2;CYFIP2;C	grey	-0.2507071	-0.1846426	0.17801777	0.10203813	-0.0019092	0.03230416
cg03760190	CTD-2296D1.2	grey	0.34198062	0.29634614	-0.1319103	0.04382039	0.02970233	0.02048163
cg17207545	RASA4B	grey	-0.1516907	-0.2068078	0.04546629	0.10079084	0.13461091	0.03766022
cg00772566	FGF12	grey	0.13616448	0.20251013	-0.130957	-0.0411923	-0.1398356	-0.0130469
cg00979931	ATG16L2;ATG16L2;ATG16	grey	-0.0783833	-0.2483882	0.25402026	-0.093607	0.16405638	0.25369761
cg05719902	SVOPL;SVOPL;SVOPL	grey	-0.0968814	-0.2435096	0.27489153	0.05821297	0.21240341	0.13084501
cg25676905	VAT1	grey	0.15849461	0.06689177	-0.1900226	-0.0226327	0.00013138	0.17661454
cg07336299	PODNL1	grey	-0.2366076	-0.2166461	0.32129012	0.23035946	0.2504461	0.05194469
cg19528790	TTC24;TTC24;TTC24	grey	-0.2466903	-0.2226552	0.15271424	0.08714909	0.12119157	0.03633386
cg22584172	ZNF451;ZNF451;ZNF451;Z	grey	0.02799412	0.09730136	-0.2552572	-0.2131322	-0.0818477	-0.1786963
cg01154254	AC007040.11;ANKRD53;A	grey	-0.1696763	-0.2858442	0.3160774	0.21048262	0.3172747	0.11538664
cg00042156	RP11-178A10.1;RP11-	grey	0.39483718	0.361656	-0.0693079	0.22496449	0.01919631	0.17961265
cg10871071	CCDC129	grey	0.27228837	0.28993426	-0.0966544	0.04709329	-0.1750296	0.22702262
cg00230895	PGAP1;PGAP1;PGAP1;PG	grey	-0.0735775	-0.1347538	0.17831585	0.26465884	0.17739467	0.39601991
cg15703585	RSPH10B2;RSPH10B2;RSP	grey	0.21009618	0.2199416	-0.0025125	-0.0803368	-0.0894663	-0.035636
cg07591253	GRIFIN;GRIFIN	grey	-0.1070452	-0.0762552	0.11783547	0.31073123	0.28294414	0.15862655
cg04469165	MBIP;MBIP;MBIP;MBIP;M	grey	-0.0400641	0.07321443	-0.2305624	-0.1700646	-0.1900687	-0.1328292
cg17380689	GAL3ST1;GAL3ST1;GAL3S	grey	-0.0054873	-0.1357046	0.35804402	0.13753088	0.20469838	0.23833959

cg13682809	AC010969.1	grey	-0.2034082	-0.1875876	0.0557896	0.01305457	0.1533884	0.06482121
cg09560365	XRCC2	grey	0.13503526	0.08041381	-0.243583	0.13200327	0.02476669	-0.0201788
cg06211713	LCTL;LCTL;LCTL;LCTL	grey	-0.2692685	-0.2753733	0.24100793	0.07217864	0.29109047	-0.0847989
cg13744677	RP11-	grey	-0.0732487	-0.2798447	0.01238342	0.01042738	0.23941816	0.12808748
cg09653424	DTNA;DTNA;DTNA;DTNA	grey	-0.2177867	-0.2269271	0.22482823	0.16558457	0.22000209	0.04653867
cg17428744	UTP11L;UTP11L;UTP11L;U	grey	-0.3627521	-0.3666216	0.07292904	0.06432131	0.14401765	-0.0399321
cg17444709	RP11-253E3.1	grey	0.15939962	0.12346973	-0.1342631	-0.0330912	-0.0710586	0.1345146
cg14229732	C18orf25	grey	0.17384482	0.1525166	-0.2635395	0.05923353	-0.1017681	0.0016278
cg11427329	SLC29A1;SLC29A1;SLC29	grey	0.02485968	0.25405727	0.0535038	0.43451874	0.08739212	0.14226301
cg23715029	TRAK1;TRAK1;TRAK1;TR	grey	-0.0006363	-0.0711607	0.23342715	0.22964273	0.14598477	0.11498949
cg18611122	CERS2;CERS2;CERS2;CER	grey	-0.114733	-0.0707753	0.19600618	0.18302356	0.11651633	0.09880688
cg16681199	COL9A1	grey	-0.2128379	-0.2054236	0.17758537	0.00466116	0.10229879	0.1412571
cg04701817	VWA2	grey	0.23078686	0.1156993	-0.0940096	0.06394401	0.14107837	-0.0973244
cg23195510	RLTPR;RLTPR	grey	-0.3696874	-0.3129548	0.10392295	0.08479832	0.27532778	-0.1593236
cg11303738	KRBOX1-	grey	0.05347384	-0.1251778	0.18860498	0.12530966	0.28368203	0.05698061
cg12681948	ATP13A3;AC046143.7;ATP	grey	-0.1109348	-0.0812846	0.04081818	0.19070432	0.21188086	0.04158786
cg12238416	FAM47E;FAM47E;FAM47E	grey	-0.2942051	-0.2826754	0.15814213	0.06174994	0.30574013	-0.1104831
cg26038582	HIVEP3;HIVEP3;HIVEP3;H	grey	0.1233806	-0.0786446	0.20831605	0.1859263	0.16427623	0.17138901
cg09946253	CLVS2;CLVS2	grey	-0.1489906	-0.2746988	0.22538263	0.02698248	0.19659858	0.11212919
cg06861037	ADCY7;ADCY7	grey	0.06608111	0.10715428	0.24259093	0.37237265	0.17221251	0.20329002
cg01808072	IAH1;IAH1;IAH1;IAH1;IAH	grey	-0.2285057	-0.260329	0.16456148	0.04441848	0.24751016	0.02007617
cg02274315	SLC6A6;SLC6A6;SLC6A6;S	grey	-0.4223549	-0.3724673	0.24886638	0.09915748	0.28272123	-0.2584042
cg14440600	CPT1C;CPT1C;C19orf76;CP	grey	-0.2711206	-0.2933898	0.27912931	0.12499603	0.26812613	0.04589702
cg10695490	HRC;HRC	grey	-0.2280846	-0.0234342	0.31089586	0.2846154	0.1261266	0.1439669
cg21090298	MCF2L;MCF2L;MCF2L-	grey	-0.2752111	-0.2899999	0.15053373	0.03029375	0.22741901	0.00170701
cg16342423	RP11-6I2.4	grey	-0.3191469	-0.3613069	0.15528426	0.03782982	0.23761702	-0.1075366
cg01001422	NFIX;NFIX	grey	-0.2567103	-0.2436748	0.25431646	0.07965547	0.22639114	0.04445936
cg13655341	NPB;NPB;NPB;PCYT2	grey	-0.3204486	-0.0479343	0.2603221	0.05886711	-0.0716309	0.061357
cg13030790	RUNX1;RUNX1;RUNX1;R	grey	0.03817651	0.06720859	-0.1701946	-0.2128392	-0.1103743	-0.2722327
cg05941025	SPIRE2;SPIRE2	grey	-0.1365468	0.04444089	0.29043744	0.20708707	-0.0734602	0.19690382
cg14468013	DLG2;DLG2;DLG2;DLG2;D	grey	0.27794393	0.26047487	-0.2271799	0.07273333	0.02058609	0.01092094
cg11939399	REST;REST	grey	-0.3754328	-0.3279702	0.08393591	0.02520191	0.17840169	-0.0883733
cg06272239	ATG12;ATG12;ATG12;ATG	grey	0.11344334	0.25754527	-0.4292139	-0.0280511	-0.0953261	-0.1583502
cg01730666	CD300LG	grey	-0.2346489	-0.0895939	0.14480699	0.12159541	0.20308547	0.05284109
cg21436456	ZNF385D;ZNF385D;ZNF38	grey	-0.3643559	-0.3725317	0.15589609	0.03702268	0.18875005	-0.0401741

cg25164777	PXDNL	grey	0.36073888	0.31515381	-0.0966317	0.05206813	-0.0575453	-0.0156278
cg15742063	FAM43B	grey	-0.2369578	-0.3233942	0.27180811	0.1894426	0.25955136	0.06527211
cg17333051	SGTA	grey	-0.3004651	-0.3067415	0.19511279	0.11973141	0.25547914	-0.0100181
cg03832575	RGS12;RGS12;RGS12;RGS	grey	-0.2973176	-0.1027619	0.29482837	0.04617403	-0.1310853	0.10173328
cg01532172	RP11-478H13.3	grey	-0.1127155	-0.1744327	-0.0337701	0.07383438	0.25458699	-0.0910811
cg08648042	AC091172.1	grey	-0.3231561	-0.2408746	0.23751216	0.10240625	0.28740055	-0.0084682
cg26432918	CTD-2562J17.4;CTD-	grey	-0.1599777	-0.277356	0.17576317	0.21441516	0.28023051	0.16729845
cg09323728	TP53INP1;TP53INP1;RP11-	grey	0.0026586	-0.2293428	0.24939478	0.14974643	0.31902019	0.1785025
cg24100140	RP11-137L10.6;RP11-	grey	0.2010822	0.23163395	-0.315132	0.01447438	-0.0565385	-0.0171713
cg18356276	AHR;AHR;AC003075.4;AC0	grey	0.33816141	0.34474752	-0.2091966	0.08563523	-0.1482869	-0.0490836
cg10538151	GRIN1;GRIN1;GRIN1;GRI	grey	-0.4026688	-0.3688088	0.11621828	-0.1523359	0.09813863	0.05151763
cg07969622	PRAME;PRAME;PRAME;P	grey	-0.1380532	0.08868933	0.20145434	0.16859314	-0.0724702	0.12321902
cg13349269	ZNF33A;ZNF33A;ZNF33A;	grey	0.09184226	0.18509343	-0.3339032	-0.1550228	-0.2477455	-0.004729
cg05009295	RP11-433C9.2;RP11-	grey	-0.1867144	0.08553299	0.21918197	0.25015773	0.03949853	-0.1360918
cg05614333	GABRA5;GABRA5;GABR	grey	-0.2063054	-0.2164362	0.11424538	-0.0209848	0.15607521	0.22904081
cg03383941	WFDC2	grey	0.12630602	-0.0517915	0.01998838	0.03073915	0.11099768	0.08038403
cg03045080	GNG5	grey	-0.3054061	-0.1798921	0.26286113	0.38944252	0.17721825	-0.023883
cg15700776	CYP2U1;CYP2U1;CYP2U1;	grey	-0.2299321	-0.2498571	0.1074044	0.1165055	0.21964106	-0.0127976
cg14132536	DPM2;DPM2;DPM2;DPM2;	grey	-0.1795259	-0.2309793	0.18080732	0.02323625	0.21462033	0.04846313
cg05674784	TBX18;TBX18	grey	-0.0407252	0.04395111	-0.3036348	-0.1227255	-0.0534216	-0.1211077
cg13251913	GPR56;GPR56;GPR56;GPR	grey	-0.2033649	-0.2332867	0.21787983	0.03489092	0.06312853	0.10082808
cg17961975	SLC7A7;SLC7A7;SLC7A7;S	grey	-0.0131801	-0.0091272	0.25083465	0.16187604	0.18894634	0.16137196
cg17936862	PUS10;PUS10;PEX13;PEX1	grey	0.2099306	0.20017035	-0.2857134	-0.0281489	-0.1225923	-0.0748312
cg25593520	C18orf21;C18orf21	grey	-0.2291497	-0.1431754	0.06221526	0.14697205	0.24288489	-0.074033
cg11991824	LY6G5C;LY6G5C	grey	-0.2644537	-0.3500664	0.02834738	-0.0218066	0.32086313	0.01071337
cg25875617	GFER;AC005606.14;AC0056	grey	0.19340401	0.04417941	0.35065067	0.28264002	0.24906959	0.44223764
cg06231148	ANXA1;ANXA1	grey	-0.0380504	-0.04648	-0.0498142	0.14387668	0.16825584	-0.0522518
cg00188627	SCHIP1;SCHIP1;SCHIP1;S	grey	-0.1634041	-0.1094063	0.11667387	0.18801712	0.10157888	-0.1088042
cg11264363	CPLX2;CPLX2;CPLX2;CPL	grey	0.26077286	0.226422	-0.191716	0.01185407	0.00967685	0.03829523
cg02467092	LIMK1;LIMK1;LIMK1;LIM	grey	0.03803865	0.21254375	0.00745908	0.33178725	0.02978211	0.09437069
cg03257475	AC024940.1;AC024940.1;FA	grey	0.07638672	0.03286997	-0.0962176	-0.0454957	-0.0432929	0.14186698
cg25715909	SLC12A5;RP11-	grey	-0.0554181	0.09991139	0.09845749	0.12148951	0.03208675	-0.0237447
cg18326616	C2orf78	grey	0.14990249	0.15036302	-0.0426228	-0.0984168	-0.0978225	0.00185378
cg18261909	GTF2H4;GTF2H4;VARS2;V	grey	-0.1170887	-0.2113438	0.37762329	0.1913811	0.29004948	0.20896079
cg04284128	TRIM14;TRIM14;TRIM14;T	grey	-0.2923529	-0.2974333	0.14946033	-0.0040123	0.15631252	0.05127219

cg03369965	LRRC34;LRRC34;LRRC34;L	grey	-0.1667781	-0.2883097	0.14646112	0.0646422	0.27725461	0.03030695
cg20927523	RFX5;RFX5;RFX5;RFX5;R	grey	-0.2817714	0.07221692	0.31542041	0.12396811	-0.0172109	-0.0284575
cg19379302	SLC6A11;SLC6A11	grey	0.22270415	0.30337982	0.04147266	-0.0113404	-0.2332486	0.07198962
cg09329151	YAF2;YAF2;YAF2;YAF2;Y	grey	0.23477522	0.00765997	-0.235076	-0.0182361	0.13754825	0.24363778
cg01551013	EML1;EML1	grey	-0.2753442	-0.3314954	0.12821984	0.07941452	0.24367681	0.28402338
cg12357115	RP11-89K10.1;RP11-	grey	-0.2200619	-0.2551118	0.36109134	0.01712971	0.07912621	0.12765616
cg25806524	AC011484.1;AC011484.1;PN	grey	-0.1239358	-0.2064552	0.26579124	0.14647127	0.12529896	0.07652337
cg26614578	C12orf52;C12orf52;RP11-	grey	0.09915496	0.12614366	-0.1487866	-0.079449	-0.0504676	-0.3169746
cg08667275	RP11-334E6.10	grey	-0.3424972	-0.2000881	0.16913167	-0.0589095	0.22724684	-0.1110812
cg10689249	RP5-1100I6.1	grey	-0.0696703	-0.0485745	0.3833035	0.42825067	0.13532386	0.17707495
cg23927974	METTL5;METTL5;METTL5;	grey	0.35878355	0.33932862	-0.2220989	0.08754352	-0.1207361	-0.0798409
cg13068285	STMN2;STMN2;STMN2;ST	grey	0.23256119	0.22800028	-0.1563804	0.09832144	-0.0157522	-0.0700511
cg27420687	RP11-	grey	-0.2173623	-0.3423944	0.09734058	0.12713889	0.26570274	0.20729925
cg04378774	WRB;WRB;WRB;WRB;WR	grey	-0.1400979	-0.1031964	0.20746579	0.01949883	0.02403126	0.06353924
cg22012583	CERS2;CERS2;CERS2;CER	grey	-0.0504975	0.07428164	0.15774194	0.24575613	0.04966661	-0.0362403
cg23762517	HIVEP3;HIVEP3;HIVEP3;H	grey	0.08701599	-0.0304445	0.11035066	0.15906076	0.16036314	0.02358422
cg11265417	TIFA;TIFA;ALPK1	grey	-0.2424841	-0.213488	0.26298278	0.12999804	0.24149042	0.08210577
cg15565110	TMEM67;TMEM67;TMEM6	grey	0.16568526	0.11738703	-0.1626965	0.02390673	0.04352111	-0.1187554
cg22658283	RP11-738B7.1	grey	-0.021483	0.02806019	-0.2036128	-0.1742076	0.00031737	-0.2912016
cg12324144	LRRC34;LRRC34;LRRC34;L	grey	-0.2644387	-0.3329634	0.19461135	0.03518255	0.25636037	-0.0506684
cg14215392	PCDHA8	grey	-0.2675031	-0.3104973	0.05081395	0.15989467	0.27620805	-0.0661032
cg14426911	CTD-2201E9.4	grey	0.0294532	-0.1545871	-0.1491293	0.13604809	0.23029344	0.09142623
cg12158483	BAIAP2L2	grey	-0.1987159	-0.3354137	0.26579885	-0.0355285	0.2190581	0.09585896
cg09131317	PON3;PON3	grey	-0.3720618	-0.3384514	0.01953929	-0.1091791	0.21622291	-0.0840048
cg23464360	MAFA	grey	0.03452054	-0.0021423	0.01760268	0.21231555	0.19079594	0.04538765
cg27051533	PPARG;PPARG;PPARG;PP	grey	0.07655603	-0.1541781	-0.1361067	0.04265717	0.2421689	0.02555824
cg11230818	BMPER	grey	-0.3049015	-0.3073479	0.32671108	0.04324059	0.25502243	-0.0337586
cg21047206	PDE4DIP;PDE4DIP;PDE4DI	grey	-0.041003	-0.2063038	0.34705939	0.12057432	0.27350852	0.27922615
cg17977679	C11orf85;C11orf85;C11orf85	grey	-0.2879988	-0.3344549	0.28026052	-0.0492527	0.12832173	0.01954792
cg20343048	CLIC6	grey	-0.2770154	-0.3328304	0.25705025	0.07599393	0.24028437	-0.0053257
cg22252041	SNRPC;SNRPC;SNRPC;SN	grey	-0.136677	-0.2443488	0.06253189	0.11112649	0.22707235	0.11193053
cg01518631	NFIB;NFIB;NFIB;NFIB;NFI	grey	-0.0213761	0.01262113	-0.308271	-0.0961706	-0.0617541	-0.2217898
cg19008784	PNPT1;PNPT1;PNPT1;PNP	grey	0.11904123	0.09073936	-0.0972335	-0.0657109	0.00838684	-0.0501832
cg17560967	EXOC6B;EXOC6B;EXOC6	grey	-0.043069	0.05309072	-0.3475023	-0.1339927	-0.1707376	-0.2452162
cg09399176	INSC	grey	-0.1930118	-0.2050252	0.25301839	0.09359979	0.18438249	0.05650879

cg05181282	PAFAH1B1;PAFAH1B1;PA	grey	-0.0704761	-0.0020549	-0.1336004	-0.2112475	-0.1036162	-0.286486
cg26927427	KIFC3;KIFC3;KIFC3;CTD-	grey	0.08891973	-0.0277962	0.12393463	0.23098157	0.2149997	0.23185873
cg01686420	NOS1;NOS1	grey	0.2575196	0.30297387	-0.1993132	-0.0356621	-0.1131653	-0.0854163
cg22370379	XXbac-BPG154L12.4	grey	-0.3481877	-0.3525868	0.19283071	0.01634642	0.14850901	0.02511254
cg01402735	ELTD1	grey	-0.2333003	-0.1009521	0.23795416	0.16667833	0.01806441	0.28301901
cg13203634	ZNF214;ZNF214;ZNF214;Z	grey	-0.3034448	-0.2686131	0.13872807	0.13874214	0.27126366	-0.0362023
cg22579103	RP11-673E11.2	grey	-0.0102313	-0.1657136	0.00440912	0.14557351	0.22316557	0.21125799
cg26338427	C14orf167;DHRS4;DHRS4;	grey	-0.167458	-0.274344	0.18940108	0.13929059	0.26774085	0.06963215
cg16601294	SERP1;SERP1;EIF2A;SERP	grey	0.10511388	0.1365305	-0.3030755	-0.100791	-0.0592633	-0.0356295
cg22404498	RP1-	grey	-0.08493	-0.2199667	0.24721888	0.18846161	0.28963591	0.09613797
cg14484793	OLFM1;OLFM1;OLFM1;OL	grey	-0.0563569	-0.187127	0.25375658	0.09023552	0.2057356	0.20974809
cg01002690	UGDH;UGDH;UGDH;UGD	grey	0.03673329	-0.0210551	0.18168981	-0.0648713	0.02139887	0.1294753
cg08969794	TKT;TKT;TKT;TKT;TKT;TK	grey	-0.3703653	-0.2924401	0.35003225	0.18207956	0.26943357	-0.1121017
cg14238325	CRIP3;CRIP3;CRIP3	grey	-0.3580967	-0.3162633	0.1203897	-0.1194107	0.13416762	-0.1425183
cg21106065	SMAD3;SMAD3	grey	-0.2658001	-0.2431498	0.18101358	0.01776304	0.2231029	0.06488477
cg26421140	HSPA2;HSPA2;HSPA2;RP1	grey	-0.1643503	-0.1525931	0.11571804	0.15981573	0.13258704	-0.0122476
cg27400205	SCHIP1;SCHIP1;SCHIP1;S	grey	-0.1191917	-0.1769357	0.09154865	0.20893615	0.24755701	-0.1019279
cg18271071	CASP9;CASP9;CASP9;CAS	grey	-0.0828335	0.02202339	0.0595974	0.16968448	0.0247158	-0.0822955
cg04767753	PSMG4;PSMG4;PSMG4;PS	grey	0.20878851	0.16539911	-0.1381163	0.07852536	0.05646234	0.05697325
cg02977320	ZNF503-	grey	-0.1471367	-0.1936945	0.22443769	0.01701387	0.18929139	0.13325827
cg22714811	KRBOX1-	grey	0.10541388	-0.1347688	0.19017804	0.1407444	0.31740244	0.15000205
cg14145159	RP11-	grey	-0.2773816	-0.1837584	0.16716663	0.06503629	0.2778054	-0.0567888
cg09648269	ANO4;ANO4;ANO4;ANO4;	grey	-0.2877402	-0.2925153	0.29957085	0.06107917	0.15611699	-0.0054852
cg09460013	PTCHD4;PTCHD4;PTCHD4	grey	-0.2244548	-0.2393273	0.07769074	0.06185337	0.25630268	0.13063945
cg25989075	NHSL1;NHSL1;NHSL1	grey	-0.1052864	-0.2393935	-0.0872851	0.02256227	0.20066795	0.16405651
cg18981477	BMP2	grey	0.14055513	0.2605266	-0.2793698	-0.0494961	-0.1785473	-0.0615758
cg02864688	MAD2L2;MAD2L2;MAD2L	grey	-0.186455	-0.3019777	0.31880437	0.12781259	0.2712688	0.16985337
cg25106815	RP11-148B6.2	grey	-0.0821008	-0.205018	0.18133612	0.14134348	0.1867348	0.1085769
cg03749037	MANBA;MANBA;MANBA	grey	-0.3864313	-0.358356	0.25843722	0.15975328	0.23318929	-0.033976
cg27307729	CTD-2089N3.3	grey	-0.156094	-0.2067558	0.06973227	0.21161754	0.1966497	0.07321418
cg04535048	HSF5;HSF5	grey	0.31353059	0.30661881	0.11760985	0.08670427	-0.1020343	0.11278063
cg00914125	HS6ST3	grey	0.17569916	0.04009975	-0.025044	0.15323038	0.05391298	0.17388356
cg22855255	CA10;CA10;CA10;CA10;CA	grey	-0.1776618	-0.3107764	0.15508418	0.1131735	0.23084344	0.02450123
cg24745940	GKN1;GKN1	grey	0.1632378	0.28939195	-0.0409936	-0.087017	-0.195931	-0.3048345
cg19122723	C18orf1	grey	-0.2260756	-0.0114287	0.14887772	-0.0415266	-0.1021066	0.07823954

cg03126821	C8orf47;C8orf47	grey	-0.2816518	-0.2997301	0.38087658	0.07993536	0.17781037	0.12337065
cg10271365	MYOM2;MYOM2	grey	-0.2666425	-0.3473692	0.0887965	-0.0315282	0.10409509	0.12156542
cg03292284	OPN3;OPN3;OPN3;OPN3;O	grey	0.04597395	-0.0947615	0.35448837	0.18515039	0.22337943	0.29908921
cg00669966	RP11-	grey	-0.2688573	-0.172659	0.18370333	0.13264564	0.18439889	0.12144822
cg04242499	PCDHGB3	grey	-0.1514905	-0.2639342	0.09664303	-0.0504475	0.14535992	0.05872693
cg13559366	GRIN1	grey	0.23537757	0.2107448	0.17847877	-0.1402118	-0.1469017	0.0597006
cg16882373	NFE2L3	grey	-0.2947131	-0.1142063	0.22374189	0.21624302	0.21874104	-0.1942818
cg25591664	SERINC5;SERINC5;SERIN	grey	-0.1698732	-0.1983113	0.15844444	-0.03161	0.07339072	0.06684523
cg21196201	SULT2B1	grey	-0.0898494	0.00420448	0.19502346	0.16188774	0.10255538	0.01683008
cg07054208	DCDC2;DCDC2	grey	-0.1842194	-0.1185955	0.0758614	0.13624695	0.13425108	0.0110123
cg20629183	MPP2;MPP2;MPP2;MPP2;	grey	-0.3323584	-0.3174002	0.00262687	-0.0113813	0.14849204	-0.0869194
cg02247430	GNPNAT1;GNPNAT1;GNP	grey	0.03708427	0.05549853	0.27566664	0.41773651	0.13774268	0.17955774
cg14087524	SNORA63;U6	grey	-0.0136742	-0.1742695	0.04020353	0.12624591	0.2294586	0.06924489
cg01456368	DNAH1;DNAH1;DNAH1	grey	-0.0257454	-0.292011	0.08135606	0.09346495	0.2790851	0.10557293
cg00364457	BTBD9;BTBD9;BTBD9	grey	-0.1629437	-0.1061603	0.25747381	0.11915409	0.23120532	0.09086059
cg09816693	VSX2	grey	-0.3783337	-0.3585214	0.27916424	-0.0012181	0.14197416	-0.019744
cg01422274	TSPAN4;TSPAN4;TSPAN4;	grey	-0.1663513	-0.100612	0.25433376	0.11333454	0.14488314	0.09146271
cg22511684	RP11-291L15.2;RP11-	grey	0.09753881	0.07272906	-0.2939384	-0.1029471	-0.0481658	0.00256383
cg01277202	RNMT;RNMT;RNMT;RNM	grey	0.19608798	0.27248055	-0.2608371	0.06832964	-0.0977281	0.04367298
cg21852429	C17orf98	grey	0.12143466	-0.0451945	0.06939962	0.35944665	0.25104193	0.25041011
cg01559901	ZNF709;ZNF709	grey	0.05200689	-0.2257291	0.34166275	0.13039713	0.26117519	0.29710591
cg06206357	MYL10	grey	-0.0651004	-0.1767844	0.00485665	0.0285619	0.26956079	0.00076612
cg25085537	OXTR;OXTR;OXTR;OXTR;	grey	0.31270359	0.29593292	0.16886255	0.03832047	-0.1496421	0.0260674
cg09135866	ZNF611;ZNF611	grey	-0.3431759	-0.3624857	0.0888134	0.02087532	0.14530605	-0.1174265
cg21012455	WRAP73;WRAP73;WRAP7	grey	0.05308403	-0.1029377	0.20345904	0.28011173	0.17284387	0.25559298
cg13252075	PPARA;PPARA;PPARA;PP	grey	-0.4226488	-0.2985385	0.19077243	0.17024706	0.28692903	-0.2800467
cg06877840	RBP4;RBP4;RBP4;RBP4;RB	grey	0.38714321	0.32441047	-0.2087603	0.02915344	-0.0126992	-0.0694895
cg19980771	CTA-	grey	-0.0285707	-0.1447707	0.08411731	0.18299035	0.2544366	0.11363498
cg26336453	NAV2-IT1	grey	0.34001234	0.34532356	-0.069619	0.12837282	-0.0550578	-0.0143011
cg21104412	KRBOX1-	grey	0.10005085	-0.1455753	0.12073736	0.16788385	0.32199339	0.24803439
cg10098193	ACA59	grey	0.15908059	0.01499959	-0.2088557	-0.0143112	0.06612875	-0.107662
cg02557865	C16orf45;C16orf45;C16orf45	grey	-0.3344208	-0.3605879	0.40797113	0.08332564	0.18462079	0.10534003
cg02640005	ST5;ST5;ST5;ST5;ST5;ST5;S	grey	0.16780211	-0.0402939	0.20952256	0.29792091	0.22899072	0.35881951
cg06417831	MAD2L1BP;GTPBP2;GTPB	grey	0.10211452	-0.0216245	0.02217052	-0.0525779	0.12179964	0.08695639
cg24929696	RP11-823P9.1;RP11-	grey	0.1637468	-0.0911059	0.1881745	0.12724641	0.14963658	0.34911821

cg08760829	STK32A;STK32A;STK32A;	grey	-0.3985248	-0.3619548	0.26292579	-0.0455085	0.13200628	-0.0029202
cg22014708	FAM120AOS;FAM120AOS	grey	0.15574582	0.27698648	0.15697323	-0.031879	-0.1776099	0.07268541
cg20207973	BAIAP2L2;BAIAP2L2	grey	0.05807077	0.10650124	0.15768672	0.21613237	0.02861496	0.2327213
cg17665193	GAD2	grey	-0.4066988	-0.3873667	0.07359991	-0.1027107	0.13222185	-0.0868752
cg01681396	SYTL1;SYTL1;SYTL1;SYT	grey	0.15307638	0.07606715	0.30186094	0.09523869	0.02612014	0.29371067
cg10986945	DNASE1;DNASE1;DNASE	grey	-0.0676291	-0.0735185	0.29699682	0.16360232	0.12936669	0.18113615
cg18059933	TP53INP1;TP53INP1;RP11-	grey	-0.0451627	-0.3142651	0.18328336	0.0939752	0.35636849	0.16904391
cg12398520	COL9A1	grey	-0.0154413	-0.1146948	0.15572366	0.15358458	0.17256761	0.20314986
cg02509569	SNTG2;SNTG2;SNTG2;SNT	grey	-0.3385318	-0.1533581	0.24622465	-0.0149616	0.16143551	0.01948575
cg04178340	AC002398.5;AC002398.5;A	grey	0.05264333	-0.063771	0.13167269	0.26915954	0.24045843	0.3639918
cg01213447	RP11-327J17.3;RP11-	grey	-0.0988086	-0.1283097	-0.0664182	0.00324785	0.04346742	-0.0232168
cg11086566	ASIC3;ASIC3;ASIC3;ASIC3	grey	0.19179721	0.02097518	0.20113958	0.26000255	0.25882275	0.15690622
cg11172367	TPD52L3;TPD52L3;TPD52L	grey	0.3064283	0.35310305	-0.1706858	0.00298938	-0.1671724	0.12969841
cg09315367	SLC44A2;SLC44A2	grey	0.11270309	-0.1071795	0.19443698	0.02062237	0.20626903	0.23262485
cg17312492	RP11-758N13.1;RP11-	grey	-0.364229	-0.355728	0.20677116	0.03282784	0.176884	-0.0953879
cg23222061	PRKAG2;PRKAG2;PRKAG	grey	-0.0533546	-0.1890251	0.18170152	0.08204491	0.30985181	0.12403662
cg25116709	AC106786.1	grey	0.14883797	0.06904397	-0.3203656	-0.0048229	0.03261912	-0.061658
cg22029157	IRF6;IRF6	grey	-0.2111101	-0.3288574	0.04759624	-0.0214965	0.23141653	0.06486021
cg22832231	RP11-107C16.2	grey	0.24653323	0.26522163	-0.0705805	0.02729574	-0.1176786	-0.0082387
cg05552307	YOD1;PFKFB2	grey	-0.1541005	-0.2247017	0.17978338	0.01299522	0.12097209	0.17690536
cg07219955	PLA2G7;PLA2G7;PLA2G7;	grey	-0.2914607	-0.2798557	0.25812639	-0.089015	0.11306146	-0.1274514
cg18817664	TLE6;TLE6;TLE6;TLE6;TLE	grey	-0.4186263	-0.3251638	0.17102874	-0.0108558	0.28442444	-0.1689032
cg14996491	HIVEP3;HIVEP3;HIVEP3;H	grey	0.08042893	0.05190939	0.25351798	0.19187009	0.02916467	0.13005582
cg00121525	Y_RNA;FAM53A;FAM53A;	grey	-0.3244042	-0.3248983	-0.2480462	-0.0733647	0.1803628	-0.0848223
cg14419560	DSCAML1;DSCAML1;DSC	grey	-0.2580484	-0.2262085	0.14595421	0.09176215	0.27041458	-0.128654
cg00414572	PEX13;PEX13;PEX13;PEX1	grey	0.25797802	0.30116569	0.21694599	0.17289651	-0.1841368	0.11137852
cg26103798	RN5S222	grey	0.00503308	-0.0659336	0.08082377	0.1989047	0.23306805	0.10602989
cg21256257	RNF14;RNF14;RNF14;RNF	grey	-0.1511939	-0.1829553	-0.0623117	0.01357609	0.12128361	0.16038237
cg07063745	CPS1;CPS1;CPS1;LANCL1;	grey	0.02936901	0.13302656	-0.3067555	-0.0825481	-0.1794067	-0.0028228
cg14762670	CACNA2D1;CACNA2D1;C	grey	-0.0866187	-0.2622499	0.40681585	0.17606944	0.24874931	0.16501145
cg14651919	ZACN;ZACN	grey	-0.0066991	-0.1327839	0.19484504	0.1472193	0.20364441	0.18040181
cg05816193	HIST1H1A	grey	-0.1223818	-0.1446383	0.08475446	0.01919258	0.08780592	-0.0480337
cg27312338	KIFC3;KIFC3;KIFC3;KIFC3	grey	0.14087714	-0.1054895	0.16707204	0.17658509	0.30670937	0.20845812
cg14125353	RP11-472N13.2	grey	-0.1797775	-0.2141259	0.14166669	0.07675346	0.24813546	0.01007486
cg11081690	ACVR1C	grey	0.12680765	0.03850332	-0.0904552	-0.0381004	0.07988116	-0.0656788

cg17788570	SNX24;SNX24;SNX24;SNX	grey	-0.2513379	-0.3419185	0.1799584	0.13636025	0.25284993	-0.0081307
cg07422880	CERS2;CERS2;CERS2;CER	grey	0.19974678	0.29229499	0.37007886	0.45355835	0.0708609	0.39539111
cg06062514	LY6G5B;LY6G5B	grey	0.2975856	0.27059522	0.04809773	0.0648313	-0.0289732	-0.0716798
cg14228987	CHST5;CHST5	grey	-0.1670018	-0.1879465	0.2070527	0.09164065	0.24393564	0.12539604
cg09809672	EDARADD;EDARADD;ED	grey	-0.0448864	-0.0543461	0.20414934	0.2871211	0.27233213	0.09377347
cg01493106	SCARF2;SCARF2	grey	0.05081338	0.23964711	0.4169842	0.29915958	0.03498472	0.27379769
cg10515956	MCOLN3;MCOLN3;MCOL	grey	-0.138471	-0.2245832	0.09748399	0.15153845	0.27592037	0.05573738
cg25071744	APOB;APOB;APOB	grey	-0.1471462	-0.2785123	0.06663481	-0.0221064	0.1160576	0.01109387
cg11560910	IQCJ;IQCJ-SCHIP1	grey	0.22683969	0.32286036	-0.0445099	0.03695583	-0.09026	-0.0062502
cg20915728	AL031963.1;RP1-40E16.11	grey	0.33229498	0.30391259	-0.0832347	0.00725935	-0.0387792	0.01240859
cg03750567	SYTL1;SYTL1;SYTL1;SYT	grey	0.21906874	0.07019735	0.151237	0.15834359	0.13321355	0.34529265
cg13527620	Y_RNA	grey	0.01970842	-0.0567025	-0.0401823	0.11368045	0.19402748	-0.0894522
cg17976829	GLRX3	grey	0.07250888	0.0673125	-0.1414843	-0.1604534	-0.0426424	-0.2940015
cg21789599	AIMP1;AIMP1;AIMP1;AIM	grey	-0.0572646	-0.0724793	-0.0552469	-0.1515377	-0.0142968	-0.1445874
cg11369867	ALOX15;ALOX15;ALOX15	grey	-0.2024823	-0.2391138	0.30099652	0.23616754	0.22102726	0.0125194
cg18877737	NTS;NTS	grey	0.12459027	0.21091492	0.24435371	0.19761236	0.03737547	0.11465371
cg07790718	MNT;MNT;MNT;RP1-	grey	0.04584587	-0.0457116	0.35156284	0.40029525	0.25042255	0.23236261
cg12256845	PTCHD4;PTCHD4;PTCHD4	grey	-0.19769	-0.1349658	0.07570576	0.0470717	0.13738257	0.06629023
cg01173186	NRSN1;NRSN1;NRSN1;NR	grey	-0.1894542	-0.2663395	0.28678743	0.14758206	0.21297324	-0.0272525
cg07771316	HOXC12	grey	0.1218606	0.04286433	-0.1040031	-0.0480252	-0.0075453	-0.0270659
cg23358572	ALOX15;ALOX15;ALOX15	grey	-0.2855372	-0.368755	0.19298534	0.10593552	0.22324775	0.01029939
cg22191765	MFI2	grey	-0.0972163	-0.1009006	0.12185067	0.14026656	0.05214287	0.18273243
cg12936232	JPH4;JPH4;JPH4;JPH4;JPH	grey	-0.2977054	-0.3314985	0.2388582	0.04035861	0.18119038	0.07134584
cg11785192	LHX6;LHX6	grey	-0.0627198	-0.1417329	0.1146252	0.28762431	0.27211327	0.07958834
cg19242174	LAMB1;LAMB1;LAMB1;L	grey	0.22051228	0.1555653	-0.2170295	-0.0926835	-0.0379223	-0.0918045
cg10184431	HIST1H2AJ;HIST1H2BM;H	grey	-0.0967863	-0.1002016	0.25281597	-0.0102867	-0.0025511	0.16971422
cg21300318	MIR7-3HG;MIR7-	grey	0.0965295	-0.0380821	0.19606077	0.02763556	0.05748746	0.31105813
cg18681905	ATP13A2;ATP13A2;ATP13	grey	0.01963467	-0.0052247	0.18737577	0.13447021	0.01437557	0.29286628
cg24454109	FGF19	grey	-0.2819655	-0.2672918	0.01574975	0.14519766	0.18036201	-0.0636136
cg17702370	LINC00482	grey	-0.2525893	-0.3389083	0.32029883	0.12097631	0.2567437	0.10453996
cg01010328	TTC24;TTC24;TTC24;TTC24	grey	-0.1934473	-0.1563625	0.12602413	-0.0044737	-0.0254829	0.1034502
cg05830480	ACP6;ACP6	grey	0.10171233	0.25203318	-0.0413883	0.38455916	-0.0909757	0.10261349
cg02518135	RP11-634B7.4;RP11-	grey	-0.2057168	-0.2747218	0.08700221	0.06493975	0.16661909	0.02205408
cg25299750	TCP11;TCP11;TCP11;TCP11	grey	-0.3290524	-0.3221055	0.04740204	-0.0795072	0.14382947	-0.2417177
cg21431091	TMEM9;TMEM9;TMEM9;T	grey	-0.2045996	-0.2973246	0.20181554	0.03420161	0.24565223	0.12060402

cg05572037	PCOLCE;RP13-	grey	-0.3542159	-0.299144	0.29007417	0.12555842	0.25575519	0.08483669
cg16572908	SSR2;SSR2;SSR2;SSR2;SSR	grey	0.1730853	0.05632103	-0.08498	-0.0196302	-0.0226469	0.24995945
cg14095048	TMEM211	grey	-0.173504	0.09658933	0.18192729	0.18992518	-0.0335381	-0.0781362
cg17997138	FBXL16;FBXL16;FBXL16;	grey	-0.374226	-0.3727606	0.23595059	-0.0170368	0.12279955	-0.0947248
cg22918476	SYT8;SYT8;SYT8;SYT8;SY	grey	-0.3622907	-0.3129219	0.18026449	-0.0689743	0.21591037	-0.0097569
cg13337095	LRRC34;LRRC34;LRRC34;L	grey	-0.1927435	-0.2040391	0.14186358	0.04510158	0.20512692	-0.0717441
cg17242608	ZIC2;ZIC2;ZIC2;ZIC2	grey	0.04345667	0.02372816	-0.276022	-0.1602249	-0.0452676	-0.0490071
cg25565192	NPAS4;NPAS4	grey	0.00944838	0.03520275	0.25889656	0.08298095	-0.0153768	0.22486851
cg20181251	CREB3L1;CREB3L1	grey	-0.2461943	-0.3336288	0.0530618	0.10480243	0.29693287	-0.0489435
cg08111629	KRBOX1-	grey	0.11052629	-0.1035318	-0.0094929	0.12829564	0.31655728	0.10990184
cg06623151	ASCL2	grey	-0.2634874	-0.2928316	0.10427349	0.0893317	0.1896954	-0.014617
cg21951975	IRF6;IRF6	grey	-0.2372135	-0.2897269	0.03945679	-0.0362107	0.12928402	0.04711751
cg27014732	C10orf112;C10orf112	grey	-0.2758628	-0.2465449	0.08213686	0.0278328	0.16191099	0.01677825
cg00234261	RP4-555L14.5;RP4-	grey	-0.1943057	-0.1838683	0.1438949	0.2163819	0.17301724	0.1208646
cg25893545	EFCAB6;EFCAB6	grey	0.25792562	0.31594508	-0.1112623	0.01996734	-0.1254226	0.06265091
cg08508763	TRAK1;TRAK1;TRAK1;TR	grey	-0.0691018	0.03822244	0.3630447	0.157077	-0.152107	0.19252446
cg11424828	MYOM2;MYOM2;MYOM2	grey	0.0295365	-0.0590963	0.07355477	0.00054118	0.03501249	0.12923105
cg05583659	SPOCD1;SPOCD1;SPOCD1	grey	-0.2119568	-0.1275745	0.19328378	0.09913432	0.20448487	0.02914542
cg21951391	WWTR1-AS1;WWTR1-	grey	-0.3004975	-0.3596628	0.30653049	-0.0850378	0.11062209	-0.0196448
cg06981391	FRMD4A	grey	-0.1217345	0.01057807	0.18719757	0.18184167	0.0846568	0.00749115
cg09496906	RP11-91I20.1	grey	-0.1013583	-0.1395019	0.20473479	-0.0052085	0.17724695	0.1186355
cg25656836	RP11-	grey	-0.0601087	-0.0063308	0.12194978	0.17939373	0.16460068	0.18056029
cg01512721	PRSS48	grey	-0.2676545	-0.0676193	0.39126605	0.1879691	-0.0968261	0.04783518
cg18200280	KLK4;KLK4	grey	-0.101862	-0.0551505	0.24721518	0.13624594	0.0952788	0.22712844
cg09641885	AC073283.4;EPCAM;EPCA	grey	-0.2486415	-0.2320355	0.09239763	0.06678569	0.14830932	-0.1036539
cg08905895	RP11-	grey	-0.2285421	-0.3159526	0.2427604	0.05445688	0.06611373	0.04748354
cg03321553	LTK;LTK;LTK;LTK;LTK	grey	0.04782231	-0.178227	0.1205345	0.10772962	0.18702069	0.17199491
cg23115674	AC016768.1	grey	0.19816522	0.30661777	-0.0737428	-0.0358021	-0.1573777	-0.0828187
cg03762817	TTC9;CTD-2540L5.5;CTD-	grey	0.14471157	0.27234115	-0.2224663	0.00467773	-0.0918888	-0.0234034
cg09847289	ASB18;ASB18;ASB18	grey	0.3826791	0.32084177	-0.1669994	0.1846947	0.0379733	0.10963676
cg13066963	HES2	grey	-0.2377287	-0.2275646	0.34981626	0.16445621	0.09437798	0.15305535
cg20895058	C14orf39;RP11-1042B17.3	grey	-0.2492248	-0.2780376	-0.124533	0.10772213	0.25012688	-0.0396441
cg18008766	SRSF7;SRSF7;GEMIN6;SR	grey	-0.3150957	-0.3034491	0.2771217	-0.0421056	-0.0145176	0.01838969
cg21756194	CALCR;CALCR	grey	-0.1253381	-0.1539696	0.21687197	0.24752718	0.10420377	0.14310378
cg00386141	LEMD1;LEMD1	grey	-0.1433179	-0.0245419	0.18328157	0.06931581	0.05615298	-0.0447012

cg17603758	RP3-402G11.5	grey	0.06300148	0.00801261	0.10628987	0.23923253	0.07784602	0.19786158
cg07120993	PDGFRL;PDGFRL	grey	-0.1106643	-0.1195598	0.06189055	0.12367107	0.07595516	0.01200453
cg08487455	CNTNAP4;CNTNAP4	grey	0.11167597	-0.0146098	0.00262531	-0.0683596	0.11333324	-0.0578863
cg19262963	ARHGEF25;SLC26A10;AC0	grey	-0.049457	-0.1404262	0.27679917	0.40423988	0.08270194	0.23049268
cg17998343	EFCAB4B	grey	-0.1088322	-0.0503587	0.18922223	0.14618668	0.1850032	-0.0516619
cg24587879	USP44;USP44;USP44;USP4	grey	-0.0775774	-0.1769335	0.01029473	0.17904655	0.17262033	0.12675875
cg04097078	KLHL22;SCARF2	grey	-0.1734704	-0.1916842	0.05416107	0.10056982	0.19003917	-0.0235011
cg01282852	NAV2	grey	0.18295941	0.10614345	0.04739168	0.0438563	0.02857999	0.09566357
cg08577187	Y_RNA	grey	-0.3942526	-0.3778773	0.02279367	0.0083509	0.16477687	-0.159024
cg12951726	SLC24A6;SLC24A6;SLC24	grey	-0.2289225	-0.0314181	0.26710707	0.17576461	0.11161085	0.0890996
cg22051166	LVRN;LVRN;LVRN;LVRN;	grey	0.31150901	0.16607599	-0.3322169	0.06517019	0.16050555	-0.0259186
cg13605615	DIRAS3;DIRAS3;DIRAS3;R	grey	-0.0864857	-0.0465249	0.3212769	0.08865904	0.05819477	0.08815478
cg21140145	ZC2HC1A;ZC2HC1A;ZC2H	grey	-0.1162165	-0.3360008	0.18153379	0.01677178	0.31549074	0.06287902
cg13026729	EZR-AS1;EZR;EZR	grey	-0.1808987	-0.1642415	0.27201457	-0.011158	-0.0110784	0.11976705
cg00397283	RAVER1;ICAM3	grey	-0.3078405	-0.078412	0.11005379	-0.1182835	-0.168762	-0.1098991
cg01885635	ZNF621;ZNF621;ZNF621;Z	grey	-0.3706469	0.00609038	0.26888269	0.05265222	-0.1337971	-0.0812218
cg02060584	ANK2;ANK2;ANK2;ANK2;	grey	-0.1514155	-0.3094264	0.09444876	0.06052381	0.2552629	0.10418644
cg02873442	RP11-62I21.1	grey	0.30960617	0.1344433	-0.2287351	0.15169078	0.1786035	-0.013171
cg26608174	HOXB7;RP11-	grey	-0.2126831	-0.2678142	0.25170668	0.17448565	0.12179621	-0.0400917
cg19655195	DNAH7;DNAH7	grey	-0.0937254	-0.2438756	0.14001517	0.15841816	0.29403424	0.03745037
cg22833618	HRC;HRC;HRC;HRC	grey	-0.3499366	-0.3430804	0.36490101	0.00614542	0.23716955	0.01275268
cg27020253	HLA-L;HLA-L;HCG17	grey	-0.2031886	-0.2419044	0.27587806	0.22267292	0.28105638	0.1143689
cg18301423	PDLIM4;PDLIM4;PDLIM4;	grey	-0.2747576	-0.2442355	0.23226602	-0.0527758	0.23631805	-0.2074068
cg01664925	LZTS1;LZTS1;LZTS1;LZTS1	grey	0.37893709	0.32606492	-0.029479	0.24061474	0.09723719	0.11996783
cg17826679	SLC44A2;SLC44A2	grey	0.0950292	-0.1123066	0.16121349	0.02287564	0.18305549	0.24822474
cg02575448	PCDHGB3	grey	-0.0680447	-0.1801212	-0.0267971	-0.0937696	0.08746772	0.04735194
cg07957031	RP11-	grey	0.04909847	0.09255087	0.14141366	0.32092701	0.17722931	0.18376437
cg08904363	NAPEPLD;NAPEPLD;NAP	grey	-0.1987991	-0.2803716	0.3192269	-0.004791	0.08151495	0.07683155
cg02055483	NPAS4;NPAS4	grey	-0.229111	-0.3112617	0.16515121	0.06671858	0.23086576	-0.0062605
cg02324021	RASA1;RASA1;RASA1;RA	grey	-0.1031227	-0.0480363	-0.2662529	-0.310392	-0.1035094	-0.2599034
cg13884907	SERTAD4;C1orf133;C1orf13	grey	0.40756571	0.18638087	-0.3117129	0.09409796	0.20883207	-0.0212469
cg04287289	FANCA;FANCA;FANCA;F	grey	-0.1703707	-0.1218713	-0.0228441	0.03007411	-0.0206395	0.06301655
cg04127044	PPYR1;XXyac-YR14BB7.1	grey	-0.2968266	-0.3279292	0.13109963	0.13579238	0.26426329	-0.1294196
cg06023039	SNAPC5;SNAPC5;SNAPC5	grey	-0.0795793	-0.0967031	-0.2263974	-0.1694082	0.03233563	0.08606781
cg21735461	TPST2	grey	-0.2463507	0.04264249	0.18472024	0.23999553	-0.1022137	0.06481658

cg13750905	KIFC3;KIFC3;KIFC3;CTD-	grey	-0.1374022	-0.1393256	-0.0078568	0.02827737	0.14846938	0.03324084
cg02887458	GATAD2A;GATAD2A;GA	grey	-0.0015469	-0.1264501	0.0747408	0.08516752	0.27342806	0.04282436
cg16579641	STARD13;RP11-	grey	0.16013131	0.03146624	-0.2648574	-0.0052247	0.12522434	-0.0416203
cg20863339	EIF4E3;GPR27;EIF4E3;EIF4	grey	-0.2957372	-0.3303525	0.11807559	0.00874257	0.14240265	-0.0959948
cg08490155	SSTR1	grey	-0.063655	-0.2026607	0.29210833	0.27754002	0.22186325	0.09847893
cg08517826	CLASP2;CLASP2;CLASP2	grey	0.08892256	0.13367502	-0.2449319	-0.1538856	-0.0861477	-0.1006614
cg23446861	NUCB1;NUCB1;NUCB1;N	grey	-0.1219896	-0.071844	0.24841323	0.31224634	0.18961396	0.16304637
cg17451176	PCDHGA5	grey	-0.2326558	-0.3397967	0.18977197	-0.0471839	0.19335946	0.10769724
cg05022225	TEAD2;TEAD2;DKKL1	grey	0.12560349	-0.0395135	-0.1810614	-0.0109053	0.08518039	0.1541129
cg01346718	CSNK1E;CSNK1E;CSNK1E	grey	0.00472329	-0.0491754	0.37019135	0.27428527	0.23391462	0.11381095
cg19611570	ORAI2;ORAI2;ORAI2;ORA	grey	-0.2818733	-0.2232272	0.15275879	0.19935044	0.12321589	-0.0448255
cg15437444	FBXL2;FBXL2;FBXL2;FBX	grey	-0.1117354	0.05228306	-0.2260538	-0.1966367	-0.1994164	-0.2053184
cg03955711	GADL1;GADL1	grey	-0.0465802	0.02858956	0.08380743	0.1982088	0.0715259	0.06561582
cg15416643	NEFL	grey	-0.1806119	-0.1344856	-0.012898	0.09422927	0.10450184	-0.1196509
cg02356993	GABRA5;GABRA5;GABR	grey	-0.2091671	-0.3365318	0.08317382	-0.0337933	0.22689518	0.16080443
cg02661687	ST6GALNAC4;ST6GALNA	grey	-0.3109462	-0.3217957	0.22704439	-0.0315666	0.06266912	0.0798436
cg25147026	PTGS2;PTGS2;PTGS2	grey	-0.1185676	-0.1865324	0.13318654	0.26404643	0.3157312	0.0524484
cg16488649	CTD-3088G3.2;CTD-	grey	0.27536905	0.23354355	0.11693933	0.20205191	0.12914604	0.2411916
cg27069921	RP11-528L24.3;RP11-	grey	0.02250418	0.01396915	0.20832383	0.15420888	0.16416746	0.17178443
cg22020709	AC004920.3	grey	0.09459245	0.06468772	-0.0466511	0.3092453	0.12486794	0.01697249
cg00643392	RTBDN;RTBDN	grey	-0.1444237	-0.2336833	0.1513062	0.007374	0.18379666	-0.0124764
cg01764105	SMC4;SMC4;SMC4;SMC4;	grey	-0.1950441	-0.2486761	0.3438926	0.04478555	0.19672887	0.12163902
cg14070755	MLPH;MLPH;MLPH;MLPH	grey	0.21324454	-0.0177013	0.13387035	0.21887607	0.23803186	0.11347556
cg15507942	CD36;CD36;CD36;CD36;CD	grey	0.14375395	0.27754464	-0.0695929	-0.0454326	-0.0688812	-0.0535915
cg20862309	ZC3H14;ZC3H14;ZC3H14;Z	grey	-0.1525992	-0.0917461	0.20456202	0.14076782	-0.0190084	0.12293356
cg15655138	PIM1;PIM1;PIM1	grey	-0.147572	-0.0947152	-0.0476308	-0.2080892	-0.0123591	-0.194427
cg05202629	C17orf97;C17orf97;C17orf97	grey	-0.0766326	-0.0184093	0.1941898	0.17239354	0.12678817	0.04969768
cg21413492	COL9A1	grey	-0.2001061	-0.2772651	0.18217739	0.02784118	0.15848299	0.05381513
cg16339402	HOTAIR;HOTAIR;HOTAIR	grey	-0.3126407	-0.3375419	0.26147948	0.08019871	0.16923688	-0.0556464
cg05318777	RP11-	grey	-0.2055442	-0.17011	0.19733541	0.17474007	0.14847709	-0.0656682
cg19645880	AC073283.4;AC073283.4;A	grey	-0.1688742	-0.2505925	0.11976631	0.16345119	0.26434472	-0.0359206
cg22324523	RP11-236J17.3	grey	-0.1722471	0.05309135	0.13455539	0.18893129	0.06954399	-0.0169069
cg01977082	COX6C;COX6C;COX6C;CO	grey	-0.2498142	-0.2305615	0.23162646	0.19321453	0.187185	-0.0590629
cg15655714	ASPDH;ASPDH	grey	-0.1354506	-0.2018862	0.27776457	0.31672687	0.24225468	0.15754391
cg22105158	C19orf77	grey	0.04460091	-0.1785781	0.12288824	0.04777927	0.21405056	0.25402019

cg06292784	PTPRT	grey	0.20656647	0.10456987	-0.2792503	0.01701563	0.03039832	-0.0257998
cg01005080	LHX8	grey	-0.2543361	-0.3395066	0.10932284	0.06488596	0.26368793	0.02193142
cg07873154	TRIM10	grey	0.19826145	0.32996161	-0.1938032	-0.0252234	-0.1778397	-0.0591804
cg01739167	CHRNE;CHRNE;CHRNE	grey	-0.1854006	0.01217179	0.24167583	-0.017549	-0.1241198	0.21009214
cg24709000	DENND3	grey	-0.1084211	-0.1294207	0.09733328	0.13673031	0.26785445	0.00804924
cg19101186	OBSL1;OBSL1	grey	-0.2507588	0.06751551	0.12780705	0.02146871	-0.1071739	-0.0694927
cg02968629	MIR497HG;MIR497HG;MI	grey	0.34017643	0.31953783	0.04486331	0.2572669	0.04235497	0.19961131
cg17417600	GNPNAT1;GNPNAT1;GNP	grey	-0.1886345	-0.122529	0.09576597	0.07732603	0.10108367	0.00641867
cg04846508	SYT8;SYT8;SYT8;SYT8;SY	grey	-0.1663186	-0.0768451	0.20477885	-0.0461777	0.07033596	0.06600786
cg09766820	LHX8	grey	-0.2505531	-0.2627549	0.09016878	0.02929741	0.19781259	0.02194282
cg25808082	HUS1B;EXOC2;EXOC2;EX	grey	0.18068034	-0.1143007	0.14237698	0.14503567	0.27230867	0.37956371
cg03065168	EPHA2	grey	-0.030027	-0.1747138	0.45131782	0.30763141	0.26838113	0.16470985
cg14074486	TOMM40L;TOMM40L;TO	grey	0.0856739	-0.0450457	0.13330286	0.1529776	0.26488831	0.11887017
cg24222546	STRA6;STRA6;STRA6;STR	grey	0.15100687	0.09571901	0.25527415	0.37889573	0.25182966	0.24150438
cg02462868	HOXB7;RP11-	grey	0.14758486	-0.094734	0.01711991	0.30026321	0.24214087	0.28926262
cg04556868	GADL1;GADL1	grey	-0.2886556	-0.3038555	0.11175606	-0.0197753	0.16157422	-0.0355368
cg17372806	RTN3;RTN3;RTN3;RTN3;R	grey	0.00939244	0.20505751	0.10489333	0.43549159	0.01802431	0.16953987
cg02840199	ADCY9;ADCY9	grey	-0.0791246	-0.1381557	0.17336563	0.07418478	0.10310337	0.0518043
cg14743534	AC017116.8;AC004985.13;A	grey	-0.2616939	-0.2824184	-0.0596468	0.05755559	0.21622591	-0.155219
cg14374861	RP11-324L3.3;RP11-324L3.3	grey	-0.2217969	-0.3023504	0.14986467	0.09597631	0.12596034	0.11243599
cg21613259	EIF4B;EIF4B;EIF4B;EIF4B;	grey	0.29609545	0.16450303	-0.2881933	0.10512007	0.17119886	-0.0101945
cg13782866	DISC1	grey	-0.1883741	-0.2890983	0.26091721	0.13495455	0.20315255	0.10628544
cg17457090	NDRG4;NDRG4;NDRG4;N	grey	-0.1299872	-0.1011795	0.03735999	0.04395365	0.11376619	0.04560778
cg08121984	APOC1P1;APOC1P1	grey	-0.0915028	0.2149213	0.4792438	0.23744156	-0.1048015	0.14065752
cg02142074	SRC;SRC;SRC	grey	-0.1952444	-0.3154832	0.20495769	0.03087928	0.25754549	0.01544459
cg24470734	SLFN12;SLFN12;SLFN12;S	grey	-0.0537999	-0.0383501	0.11392493	0.03510328	-0.035587	0.26817932
cg26320663	FLG-AS1	grey	-0.1420853	-0.0370126	0.03263168	0.05150599	0.10416035	0.01945822
cg13606421	GALNTL2;GALNTL2;GAL	grey	-0.115632	-0.0912902	0.0490805	0.08897004	0.16175152	0.06150827
cg23504411	HIST1H3J;HIST1H3J	grey	-0.0252127	0.04455625	-0.0513148	0.08903263	0.05382097	0.09652826
cg05704547	FHIT	grey	-0.1825475	-0.2738692	0.19690601	0.04032145	0.09599767	-0.0850392
cg01225565	SUV420H1;SUV420H1;SU	grey	-0.348964	-0.3412984	0.11837717	0.11133495	0.26852149	-0.1306838
cg14767647	NUP153;NUP153;NUP153	grey	-0.0589142	-0.0652891	-0.0380823	-0.0037448	0.00868108	0.02965076
cg05938007	MRPL33;MRPL33;MRPL33;	grey	-0.1129604	0.02363587	0.09682982	0.25720788	-0.0098267	0.02598569
cg09873702	PLD3;PLD3;PLD3;PLD3	grey	0.06974278	0.10734502	-0.0156057	0.19188949	0.09591525	0.34499215
cg02147797	PRICKLE2;PRICKLE2	grey	-0.2256418	-0.2228294	0.14857812	0.019068	0.15758307	-0.0662991

cg06917450	C1orf109;C1orf109;C1orf109	grey	0.00568769	0.0044889	0.00975317	0.01237751	-0.099385	-0.0850566
cg24366383	SLC24A2	grey	0.0195268	-0.0481409	-0.2413532	0.15919441	0.11568021	-0.0749194
cg03102788	NPBWR2	grey	-0.3051955	0.01777454	0.10104636	-0.1065234	-0.1699164	-0.0710807
cg01889010	AC114730.2	grey	-0.0630997	0.00497704	0.22539095	0.20665161	0.14149199	0.16882372
cg21365805	CHRNA4;CHRNA4;CHRN	grey	-0.1343999	-0.3073076	0.1818046	0.06523099	0.27341668	-0.0025372
cg09073799	MEX3B;MEX3B;AC026956.	grey	0.03335292	-0.0738144	-0.0075542	0.04825844	0.13260392	0.0640113
cg11397548	DNALI1;DNALI1;DNALI1;	grey	-0.099923	-0.2128244	0.06870468	0.11260132	0.31499604	0.05802788
cg03659817	MTMR11;MTMR11;MTMR	grey	-0.0358642	0.15411083	0.13734526	0.1485312	0.0256281	0.03251186
cg14032572	SLC35F4;SLC35F4;SLC35F	grey	-0.27226	-0.2627194	0.18172828	0.10074088	0.13735411	0.01593866
cg03805019	ZBTB46	grey	0.05346272	-0.0029315	0.2129155	0.17365258	0.08809521	0.29025509
cg17788468	RP11-313D6.3	grey	-0.3568401	-0.0239906	0.03054252	-0.0489097	-0.2141237	-0.1222737
cg08267002	TLX2;TLX2	grey	-0.0238763	-0.0177365	-0.1931657	-0.1397124	-0.0043955	-0.2175654
cg08448379	TFCP2;TFCP2;TFCP2;TFCP	grey	-0.1860439	-0.1727642	0.10251881	0.13031561	0.15228108	0.08410932
cg16831065	DBF4B;DBF4B;DBF4B;DB	grey	-0.0139456	0.1965151	0.17584889	0.19672027	-0.1564397	0.31581277
cg21035892	DLG2;DLG2;DLG2;RP11-	grey	-0.2147296	-0.2594221	0.25835297	0.10967858	0.25521297	0.05358752
cg09233315	SERHL2;SERHL2;SERHL2;	grey	-0.0300563	-0.1804859	0.19480082	0.09531756	0.18642146	0.1313239
cg07050946	HEXDC	grey	-0.1813647	-0.0234084	0.24475739	0.12618298	0.04679672	0.04548638
cg03136323	SYT17;SYT17	grey	-0.0750913	0.08497216	0.32293488	0.2227448	0.07190746	0.07260482
cg18658046	RP11-359J23.1	grey	-0.1475256	-0.1618628	0.0953909	0.04830805	0.17123235	0.09116106
cg23395404	RABGAP1L;RABGAP1L	grey	-0.2064784	-0.2080552	-0.0675698	0.23479673	0.27490784	-0.0120779
cg06424894	SORD;SORD;SORD	grey	-0.143289	-0.0309409	0.15884418	0.07978647	0.02916187	0.10625016
cg08364654	KRBOX1-AS1;KRBOX1-	grey	-0.0194793	-0.1827049	0.11538318	0.0672911	0.30514063	0.04899493
cg17081027	C1orf65	grey	-0.011627	-0.090807	0.21055344	0.26047178	0.11516513	0.13693187
cg04077175	PDE4A;PDE4A	grey	-0.133633	-0.0782961	0.33466837	0.2646101	0.17467719	0.0780081
cg25323137	RP11-425E13.1	grey	-0.1830435	-0.2774829	0.21290698	0.08612868	0.2136094	0.01914242
cg07529715	CTHRC1;CTHRC1;CTHRC1	grey	-0.058149	-0.0442713	0.06721387	0.07263261	0.0641159	0.0405907
cg00886211	RP11-217B7.3	grey	-0.074308	-0.011793	0.05275015	-0.0410626	0.04979444	0.05328813
cg18931939	TEX30;TEX30;TEX30;TEX3	grey	-0.3505407	-0.3202182	0.0346205	0.03837757	0.25989369	-0.0550506
cg22237261	OR3A2;RP11-64J4.2	grey	0.28723559	0.32645874	0.07719752	0.16594728	-0.1599405	-0.1650734
cg05094984	C11orf9;C11orf9;RP11-	grey	-0.060274	-0.1712454	0.02687249	0.09319692	0.2000119	0.13043045
cg01165781	SYTL1;SYTL1;SYTL1	grey	0.1111568	0.03314976	0.29554894	0.06599279	0.03602782	0.2908758
cg07173760	CLC	grey	0.11642895	0.15448996	-0.0639074	0.48376851	0.01043169	0.11751232
cg05344026	LRRC34;LRRC34;LRRC34;L	grey	-0.1333052	-0.229508	0.21127578	0.03210835	0.29926612	-0.0505913
cg13466284	SFN;SFN;SFN	grey	0.18264654	0.00157909	0.15890692	0.3945417	0.24140793	0.22447447
cg14588439	COMMD1;COMMD1;COM	grey	-0.1766455	-0.0621372	0.17659306	0.18582628	0.09580501	0.01932032

cg03754907	KIAA0930;KIAA0930	grey	-0.2633621	-0.2876246	0.170046	-0.0583294	0.29086994	-0.1409453
cg19263659	RP11-646E18.4	grey	0.00393641	-0.0766769	0.0776941	0.1449365	0.20220266	0.11296091
cg12570166	LRTM2;LRTM2;LRTM2;LR	grey	-0.2238477	-0.312056	0.23726218	0.00684327	0.1824568	-0.0030953
cg00752478	ZDHHC1;ZDHHC1;ZDHHC	grey	-0.1698489	0.05293903	0.26818682	0.15923227	0.00849181	0.03154402
cg05800738	RP11-168K9.2	grey	0.2042279	0.29623091	-0.1535634	0.16113998	-0.0875447	-0.0425601
cg05725404	NDRG4;NDRG4;NDRG4;N	grey	-0.0626331	-0.1547012	0.03380997	0.06471063	0.1907924	0.07642782
cg06273882	TMEM63C;TMEM63C;TME	grey	-0.1026354	-0.0176688	0.07859901	0.11877894	0.04885938	-0.0375181
cg05738030	EPS8L1;EPS8L1	grey	-0.0517301	0.11300537	0.25465466	0.11261257	-0.0170137	0.21956467
cg18124413	EPG5	grey	-0.162414	-0.2218821	0.25416959	0.1540512	0.24000048	0.04465823
cg23716779	AC011484.1;AC011484.1;PN	grey	-0.2433707	-0.335219	0.12291686	0.05272664	0.15136943	0.02642408
cg17959580	RP1-63G5.5;ELFN2	grey	0.09240669	-0.0013652	0.03736916	0.27835561	0.07461227	0.20031806
cg01025836	ARHGAP40	grey	-0.2012641	-0.0023582	0.19063402	-0.027825	-0.1145463	-0.0403178
cg08995661	BAIAP2;BAIAP2;BAIAP2;B	grey	0.34148253	0.33383989	-0.0611674	0.05241758	0.06396676	0.24320912
cg01579024	RANBP17;RANBP17;RANB	grey	-0.0236939	-0.0312592	-0.0514565	-0.09101	0.02308435	-0.0898795
cg11093583	CTC-786C10.1	grey	0.13932565	0.01736003	0.3554722	0.30773924	0.21501536	0.15752843
cg12597066	ZNF287;ZNF287;ZNF287	grey	0.22974116	0.21307645	-0.1883656	0.00589398	0.00760394	-0.0362839
cg22189019	FGF22	grey	-0.1915258	-0.0372914	0.26188738	0.05110498	-0.0457469	0.10258125
cg01057532	SNORD109B;SNHG14;SNH	grey	-0.0671144	0.10916974	0.00023751	0.25193284	0.0287805	0.03649069
cg05580800	TOP1MT;TOP1MT;TOP1M	grey	0.13549426	0.03821328	0.1890833	0.32074829	0.19180562	0.43242345
cg13602816	ARHGEF4;ARHGEF4;ARH	grey	-0.2654794	-0.3362313	0.04629466	0.04690245	0.17378469	0.01170732
cg27543040	RP11-634B7.4;RP11-	grey	-0.1895178	-0.2343211	0.07565185	0.06630927	0.12472475	0.03861082
cg15102118	DIO2;DIO2;DIO2;DIO2;DIO	grey	-0.2023099	-0.2236609	0.10272716	-0.00319	0.17549909	0.03436028
cg24528447	NR2F1;RP11-65F13.2	grey	-0.1626754	-0.2132742	-0.1101729	0.04642312	0.25433508	-0.0756634
cg02425416	INS-	grey	-0.1499023	-0.1067278	0.123177	-0.0003377	0.04625709	-0.0277122
cg21859425	CXADR;CXADR;CXADR	grey	-0.1097067	-0.2566728	-0.029856	0.08546991	0.27634724	0.16880421
cg25576711	CIDEB;CIDEB;CIDEB;LTB4	grey	-0.0085266	-0.1910084	0.13085207	0.15527278	0.27313896	0.15433075
cg14552404	SNHG14;SNORD116-	grey	-0.0293085	0.02839293	0.21167029	0.18328035	0.04807455	0.05663147
cg11137578	ACOT12;ACOT12	grey	-0.2345277	-0.2933145	0.13679744	-0.0618949	0.17717852	-0.0102356
cg09506037	NCKAP5	grey	0.1116236	0.05923375	-0.0821378	0.05126453	0.04735797	0.09794336
cg13708645	KDM2B;KDM2B;KDM2B;	grey	-0.279943	-0.2852293	0.12940827	-0.0236851	0.07164681	0.07074476
cg13263137	MARC2;MARC2;MARC2	grey	-0.2041898	-0.1352177	0.23098074	0.23288485	0.20059165	-0.0433808
cg17557766	TFAP2A;TFAP2A;TFAP2A;	grey	-0.0522259	-0.1652717	0.16259422	0.07655845	0.14303547	0.2313746
cg11175836	KLHDC7A;KLHDC7A	grey	0.089335	0.07030518	0.1644716	0.42886683	0.01716805	0.21537811
cg05171021	DHRS4;DHRS4;DHRS4;DH	grey	-0.0604783	-0.1857839	0.22669916	0.17472352	0.21013071	0.1810404
cg03511754	GPATCH2;SPATA17	grey	-0.0606313	-0.0539384	-0.101673	0.22698524	0.06857799	0.04986766

cg01323542	AIRE;AIRE;AIRE	grey	-0.0414163	-0.1448353	0.16188055	0.16455757	0.25399953	-0.0036606
cg00056578	KSR2;KSR2	grey	-0.2821307	-0.3297603	0.2706165	0.14075979	0.2519401	-0.0049838
cg15878909	FAM90A1;FAM90A1;FAM	grey	-0.0827609	-0.104995	0.12102901	0.24063205	0.13717103	0.03542497
cg13396289	ANKH;ANKH;ANKH	grey	0.03784874	0.07976962	-0.2749865	-0.1581684	-0.1295014	-0.0021648
cg23481564	MAFF;MAFF;MAFF;MAFF	grey	-0.3345522	-0.045889	0.2648525	0.06095229	-0.1006997	0.08030339
cg13126319	C8orf55;C8orf55;CTD-	grey	0.11330695	-0.1648202	0.16913982	0.28659922	0.28400791	0.22024533
cg01849085	RP11-3N2.13	grey	-0.1461021	-0.2543404	0.12777128	0.05385695	0.16069918	0.08577192
cg21736411	WRB;WRB;WRB;WRB;WR	grey	-0.1085758	-0.085999	0.32965138	0.0539042	-0.0197146	0.07603379
cg04697454	RGMA	grey	-0.1667137	-0.2416684	0.028157	0.06841927	0.13842618	-0.0312034
cg18728950	CTD-2184D3.4;CTD-	grey	0.08804975	0.15763321	0.23487	-0.1102045	-0.117522	-0.0114072
cg09454315	AHRR;AHRR;AHRR;AHRR	grey	0.04790366	-0.1127737	0.08363406	0.12181022	0.23648869	0.23587815
cg18797653	VTRNA2-1	grey	-0.0451189	-0.0788988	0.25004375	0.0401378	0.04323841	0.15451718
cg24606651	LRP2;LRP2	grey	0.11893056	0.14220238	-0.226003	0.00375052	-0.125729	0.15970979
cg04338966	HMBS;HMBS;HMBS;HMB	grey	-0.1841163	-0.1286283	0.10917704	0.13560194	0.09602469	-0.031869
cg15023006	KIF1A;KIF1A;KIF1A	grey	-0.0041643	0.01588168	-0.2456812	-0.1348848	-0.0934434	0.19522262
cg20591307	BCAR1;BCAR1;BCAR1	grey	-0.1458714	0.01876608	0.30024179	0.09552254	0.09247177	-0.0122931
cg18806912	MAPKBP1;MAPKBP1;MA	grey	-0.0258596	-0.0647284	0.25185177	0.32304782	0.23701965	0.11707488
cg04495619	AC022148.1	grey	0.28629023	0.32228432	-0.1983518	0.11162504	-0.0385619	0.09381049
cg21305381	ANGPTL4;ANGPTL4	grey	0.23319313	0.0179385	0.29410471	0.26171557	0.17417028	0.26692139
cg24424815	CLMN	grey	-0.1635659	0.06181518	0.22413316	0.06367912	-0.1768092	0.01235406
cg08152839	ST8SIA2;ST8SIA2;ST8SIA2	grey	0.1310773	0.05375443	-0.0880279	0.05405712	0.07161935	-0.1608034
cg01977347	CTD-2277K2.1;CTD-	grey	0.19043398	0.240086	0.11583002	0.2054136	-0.0756343	0.20213927
cg04522531	SNORA21;RPL23;RPL23;RP	grey	0.1097622	0.19814435	-0.0904263	0.03190475	-0.2259581	0.05674048
cg17093267	XPNPEP1;XPNPEP1;XPNP	grey	-0.0559309	0.05116008	0.02685629	0.17159345	0.15188843	-0.0438865
cg04100190	COL9A2;COL9A2;COL9A2;	grey	0.11375332	0.00990748	0.06713378	0.10649555	0.06976143	0.0580055
cg10922280	DPEP2;DPEP2;DPEP2;DPE	grey	-0.292659	-0.2654952	0.10367656	0.01647779	-0.1053306	-0.0498164
cg02292342	C18orf1	grey	-0.0131783	0.01481139	0.09512745	0.09449236	0.02196017	0.11007568
cg01855070	TRAK1;TRAK1;TRAK1;TR	grey	-0.162951	-0.0842488	0.27737096	0.06110633	-0.0140444	0.04572014
cg10861146	EFNA1;EFNA1;EFNA1	grey	-0.3350219	-0.339469	0.22299048	0.07269314	0.24091261	-0.1560112
cg06588028	LPAR5;LPAR5;LPAR5;LPA	grey	-0.2000826	-0.1132151	0.195406	0.00340486	0.05054666	0.07231999
cg16014136	GTPBP10;GTPBP10;GTPBP	grey	0.05348398	0.054635	-0.2762707	-0.0576639	-0.017731	0.04071288
cg20797485	RP11-	grey	0.05650001	0.23928556	0.19922092	0.30253343	0.14958307	0.09416233
cg02602007	MLLT4-AS1;MLLT4-	grey	-0.1360659	-0.183245	0.27638669	0.03167742	0.10833188	0.07043389
cg07584331	ZNF434;ZNF434;ZNF434;Z	grey	-0.1362571	-0.2377635	0.07623289	0.06466347	0.21625676	0.1175036
cg18922793	RP11-123G5.1	grey	0.07002565	0.24012142	0.44575445	0.25861035	-0.0953587	0.11185043

cg00147160	CNKSR1;CNKSR1;CNKSR	grey	-0.234742	-0.1990807	0.09091864	0.13475615	0.20465854	-0.0423527
cg24655016	LRRC34;LRRC34;LRRC34;L	grey	-0.2101664	-0.285266	0.11913092	0.01396179	0.27917001	-0.0892935
cg16742003	GJD4	grey	0.18045138	0.04822408	-0.054276	0.01430247	0.1324486	0.08872183
cg19149522	ZDHHC4;ZDHHC4;ZDHHC	grey	0.01200999	0.02897934	0.21948183	0.12393081	0.06884091	0.18694777
cg16081285	CSRNP3;CSRNP3;CSRNP3;	grey	-0.1860784	-0.1253554	0.28512994	0.15482641	0.08976698	0.13831604
cg19585816	ANK1	grey	-0.2018688	-0.243281	-0.101138	-0.1993473	0.08616565	-0.0949771
cg27651885	LURAP1;POMGNT1;POMG	grey	-0.2618611	-0.2708012	0.05293152	0.14812248	0.21721135	-0.0696381
cg23255963	NRG3;NRG3	grey	-0.1785595	-0.1399236	0.18522964	0.11228733	0.0946939	0.12140362
cg02024077	CNTNAP2	grey	0.10413432	-0.0730662	0.00689452	-0.0030155	0.08876983	0.14405334
cg21885218	RP11-383H13.1	grey	0.18327064	0.33214503	-0.0296326	0.01764221	-0.1137414	-0.0730336
cg21115004	SLC35D1	grey	-0.2224462	-0.1533844	0.21738686	0.10116958	0.14064996	0.01416004
cg24348114	USF2;USF2;LSR;LSR	grey	0.0118428	-0.0162326	-0.1222701	-0.1305453	-0.0121043	0.01071788
cg18638931	MOV10L1;MOV10L1;MOV	grey	0.02317775	-0.1379375	0.08258283	0.07010572	0.14083408	0.20475172
cg19832381	HIST1H2BL;HIST1H3H	grey	-0.1433503	0.11474671	0.13981558	0.12684791	0.11011301	-0.0504487
cg06388871	METTL11A;METTL11A;ME	grey	0.06049982	0.05950965	-0.240703	-0.1322702	-0.0473343	-0.2204967
cg05973792	DCTN4;DCTN4;DCTN4;DC	grey	0.0560622	-0.0363665	0.24316492	0.24297809	0.24298758	0.16039476
cg07997936	HIST1H2BE	grey	-0.1372877	-0.2584943	0.00112575	-0.1347542	0.1801476	0.0814875
cg20490578	RP1-116K23.1	grey	0.14289853	0.1390504	0.18550851	0.04037865	0.02758226	-0.0525876
cg08094206	TRIM10	grey	0.18183782	0.32371622	-0.18461	0.02235338	-0.1660942	-0.0091513
cg14174317	PTCHD3	grey	0.04153223	0.14229127	0.18589386	0.07638762	-0.0721702	-0.0164443
cg02040583	DUT;DUT;DUT;DUT;DUT;	grey	-0.1433841	0.01481336	-0.2835613	-0.2816239	-0.114873	-0.1147646
cg11937664	LRRC36;LRRC36;LRRC36;L	grey	-0.129308	-0.1333153	0.24902481	0.23122518	0.17855332	0.17222626
cg27387222	DNM1L;DNM1L;DNM1L;D	grey	0.37107699	0.30375033	-0.126647	0.27390491	0.12609035	0.00809627
cg12438392	TSN;TSN;TSN;TSN;TSN;TS	grey	0.1416378	-0.017994	-0.0058786	-0.0079673	0.09070814	0.09304064
cg21835643	MATN4;MATN4;MATN4;R	grey	-0.1862153	-0.2454891	0.18563229	0.13125095	0.32064582	0.05381799
cg14404466	CYP46A1;CYP46A1	grey	-0.2505614	-0.2365442	0.20331171	0.17554593	0.22434653	-0.0334331
cg08615372	C19orf60	grey	-0.1503987	-0.1916017	0.25350606	0.10388152	0.18817776	0.00434935
cg01749651	LINC00051	grey	-0.26605	-0.0331508	0.17976929	-0.1241416	-0.1061011	-0.0629782
cg24869588	PCDHAC2	grey	-0.0685101	-0.2403694	-0.1299876	0.03672899	0.20982005	0.29568095
cg10071605	AC135050.1	grey	0.27338021	0.08501406	0.13304447	0.1552256	0.23512636	0.11634825
cg06742341	MAP6;MAP6;MAP6;MAP6	grey	-0.1631198	-0.3315582	0.18246338	-0.0033928	0.14661438	0.10043343
cg06225328	PSTPIP1;PSTPIP1;PSTPIP1;	grey	-0.0836725	-0.0189948	0.19626843	0.13682992	0.01482535	0.07118844
cg26317285	PHLPP1;PHLPP1	grey	-0.3675015	0.067888	0.27244023	0.06604837	-0.1538171	-0.1414478
cg03180724	RP5-1100I6.1	grey	0.10362443	0.15936016	0.38971223	0.53367265	-0.0026649	0.24813882
cg16413785	C14orf64;C14orf64;C14orf64	grey	0.08800499	-0.068547	-0.0235671	0.0546392	0.09274065	-0.0042146

cg20473545	HIST1H3D;HIST1H3D	grey	0.04443807	0.11181197	-0.1461885	-0.1031218	-0.1286707	0.17419061
cg16116973	PROCR;PROCR;EDEM2	grey	0.06362429	-0.0861701	-0.1168204	0.02360443	0.22239605	-0.1059288
cg11187739	U6	grey	-0.3413073	-0.0744655	0.15983542	0.01270199	-0.1474732	-0.0936841
cg05650586	C10orf112;C10orf112	grey	-0.1891495	-0.1606077	0.03880905	0.03637886	0.12887683	-0.0130161
cg04052013	TNNT3	grey	-0.2442303	0.05050507	0.23451447	0.08697649	-0.0642499	0.08976536
cg07212818	DRD4	grey	0.02996047	0.12033042	0.13843344	0.04367584	-0.072868	0.03245331
cg23478293	RP11-1077A2.1;RP11-	grey	-0.1541418	-0.2028843	-0.0365333	0.02211007	0.20180451	0.00031495
cg11547984	AC104655.3;AC104655.3;A	grey	0.25125846	0.21572607	0.2736547	0.37860314	0.21978964	0.24435212
cg21998208	GPR98	grey	-0.094069	0.00607428	0.16632928	0.08325956	-0.0229421	0.00011266
cg22645719	LRRC4C;LRRC4C;LRRC4C;	grey	0.1204852	-0.018887	-0.26127	-0.0526927	0.07348589	-0.0066702
cg11554543	GATAD2A;GATAD2A;GA	grey	0.18295257	-0.0256832	0.01560586	-0.0848293	0.25306536	-0.0154928
cg07549373	VPS52;VPS52;VPS52;VPS5	grey	0.05262207	0.06315715	-0.0884921	0.20046086	0.11874103	0.12516395
cg27599788	PDLIM3;PDLIM3;PDLIM3;	grey	-0.0972292	-0.019293	0.08038348	0.22806153	0.03965501	0.07036797
cg08927643	PAPPA2;PAPPA2;PAPPA2	grey	0.18523402	0.1861152	-0.0695707	-0.1162016	-0.0985035	-0.0243195
cg13819250	DHX8;DHX8	grey	-0.0022312	0.03824514	-0.1684779	-0.1724938	-0.0134278	-0.1979621
cg09336589	DLG4;DLG4;DLG4;DLG4	grey	-0.1432048	0.14058264	0.04267232	0.04692787	-0.1816153	0.01050557
cg19453818	CAV2;CAV2;CAV2;AC0020	grey	-0.1109793	-0.1721196	0.217573	0.17660287	0.20939055	0.09984832
cg01774108	IKZF2;IKZF2;IKZF2;IKZF2;	grey	0.19196834	0.1987911	-0.269096	-0.0106788	-0.1541162	-0.0644898
cg09319828	TTC24;TTC24;TTC24;TTC24	grey	-0.0645831	-0.1535065	0.14046803	0.12298428	0.07363026	0.1622409
cg08541612	RP1-163G9.1;RP1-	grey	0.08255886	0.01085773	-0.0671525	-0.0779237	0.00463384	-0.0066307
cg07826936	FAM160B2;FAM160B2;FA	grey	0.1459601	0.19082551	0.03220886	0.20401256	0.03773109	0.20207874
cg11157584	CELF3;CELF3;CELF3;CELF	grey	-0.1279423	-0.1037962	0.19204304	0.17016992	0.06172843	0.03674976
cg02341923	MAPK8IP2;MAPK8IP2;CH	grey	-0.165332	-0.1353391	0.18738067	0.11435833	0.03793636	0.06340071
cg04689048	AC011484.1;AC011484.1;PN	grey	-0.1338157	-0.1205705	0.29343563	0.01773613	-0.079615	0.12179358
cg26451735	STRA6;RP11-60L3.1;RP11-	grey	0.08602414	-0.076478	0.15776556	0.13570837	0.12148458	0.33954014
cg09706243	POLD4;POLD4;POLD4;POL	grey	-0.3144397	0.06574885	0.23060849	0.1948573	-0.0778916	-0.1330069
cg02933909	PTCHD3	grey	0.13859539	0.04738251	0.15095928	0.14228887	0.07543448	0.19637985
cg00910115	DCTN3;DCTN3;DCTN3;DC	grey	-0.1063156	-0.1211546	0.23439255	0.16295022	0.13010473	0.35681122
cg12615334	CALCR;CALCR	grey	-0.2236337	-0.326428	0.25965118	0.17947948	0.25238647	0.10339378
cg26030713	C8orf45;C8orf45;C8orf45;C8	grey	-0.3069711	-0.2994563	0.01015491	0.0586402	0.22564726	0.04949049
cg10698635	AL138765.1;PIK3AP1;PIK3	grey	-0.0581586	-0.0155112	0.11717024	0.21571187	0.19965544	-0.067781
cg24636969	ZNF704;ZNF704	grey	-0.0121393	0.17133119	-0.1010118	0.15437112	0.11462386	-0.0527516
cg06536614	VTRNA2-1	grey	-0.1087236	-0.1415901	0.19020624	-0.0327143	0.03838069	0.08379298
cg09419670	RP11-27I1.2;PSMD5;RP11-	grey	-0.017077	0.21480439	-0.1602885	-0.2051076	-0.2719292	-0.0525553
cg24049348	PDGFRL;PDGFRL	grey	-0.3234729	-0.2907732	0.00331176	0.02704559	0.14152972	-0.2153475

cg23548151	PIWIL1;PIWIL1;PIWIL1;PI	grey	-0.1920684	-0.282998	0.21884118	0.0096788	0.16846437	0.12095722
cg02174634	ADRB3;ADRB3;ADRB3	grey	-0.1674512	-0.0767899	0.15913305	-0.0738218	0.04062537	-0.0149138
cg27443416	CCKBR;CCKBR	grey	-0.0466344	-0.1324409	0.27109054	0.15062174	0.2128252	0.12387563
cg26233227	LPAR5;LPAR5;LPAR5;LPA	grey	-0.2865546	-0.2544493	0.27828478	0.0165603	0.22083844	0.05979534
cg26395594	UFL1;UFL1;UFL1-AS1	grey	0.01972695	0.06140793	-0.230996	-0.0268633	0.05317468	-0.0529413
cg10066139	RP11-736N17.8	grey	0.15029228	0.27009477	0.29225887	0.1578427	-0.0930151	0.17292474
cg01869186	SLC12A9;EPHB4;EPHB4;EP	grey	-0.1610901	-0.0444745	0.18323465	0.16788971	0.00928937	0.08976569
cg25017620	RP11-634B7.4;RP11-	grey	-0.1368164	-0.2753374	0.09334175	0.07701358	0.19185371	0.04781997
cg12858902	GSTM5;GSTM5;GSTM5;GS	grey	-0.1508038	-0.1794521	0.05995872	0.03079336	0.13758347	-0.0235178
cg07730329	PCDHGA12	grey	-0.0363114	-0.2584501	0.22707882	-0.0763198	0.18732006	0.20811924
cg21746969	LRTM2;LRTM2;LRTM2;LR	grey	-0.1058392	-0.1034192	0.24219366	0.10132919	0.01319603	0.0679425
cg20137554	MCF2L	grey	-0.2965082	-0.2610748	0.05674155	-0.0071421	0.19029149	-0.1546705
cg11659317	PLEKHH2;PLEKHH2;PLEK	grey	-0.1918694	0.02535515	0.18863597	-0.0886965	-0.1833859	0.09220861
cg00574740	LMO7;LMO7;RP11-	grey	-0.0471391	-0.1007561	0.23121806	0.01113208	0.00512584	0.06262166
cg26803100	RP11-	grey	-0.0068759	0.09894826	-0.2851442	-0.1621194	-0.1212819	-0.0858852
cg02959759	CACNA1C;CACNA1C;CAC	grey	-0.1000637	-0.0013597	0.24616242	-0.0532061	-0.099552	0.18158683
cg08431884	MSH4	grey	0.10941505	0.17837303	0.14165142	0.11611411	-0.0839029	0.0420424
cg14562086	COL1A1	grey	-0.2256178	-0.0534083	0.26403967	0.21650855	0.20117	0.00444451
cg06183947	AHI1;AHI1;AHI1;AHI1;AHI	grey	0.05599706	0.1047042	-0.2733846	-0.0516837	-0.1098407	0.05273356
cg22500706	AC007353.1;RP11-488I20.8	grey	0.14452551	0.11089415	-0.0665808	0.22733016	-0.0090437	0.1587379
cg14341289	FSD1L;FSD1L;FSD1L;FSD1	grey	-0.0023023	0.04553551	0.16508935	0.15314759	0.06357997	0.2054205
cg08098382	ZFR2;ZFR2	grey	-0.1146883	-0.0908584	0.14402544	0.2276091	0.06486296	0.10874671
cg20900524	PPT1;PPT1;PPT1;PPT1;PPT	grey	-0.30877	-0.2715998	0.1568017	0.06457752	0.12075218	0.00499737
cg06491590	RADIL;RADIL;RADIL;RAD	grey	-0.1280483	0.12537155	-0.0132098	-0.1338259	-0.2735657	-0.0466596
cg26093148	CELF3;CELF3;CELF3;AL58	grey	-0.0039395	-0.1291203	0.17151173	0.27498481	0.26560859	0.1318063
cg22424214	GARNL3;GARNL3;GARNL	grey	-0.2344332	-0.2548778	0.12207583	-0.2152469	0.05383381	0.07420554
cg03983883	ZC2HC1A;ZC2HC1A;ZC2H	grey	-0.0767851	-0.2607775	0.150145	-0.0201641	0.20790054	0.07584539
cg11097954	TRDV1	grey	-0.2810005	-0.2778347	-0.0664737	0.01068519	0.0193609	-0.0451225
cg10700718	MYOM2;MYOM2	grey	-0.2720449	-0.0969152	0.0768153	-0.0794123	-0.1146916	0.00197859
cg09109520	GPR56;GPR56;GPR56;GPR	grey	-0.1644144	-0.0829958	0.19235199	-0.0086698	-0.1784195	0.11845298
cg23283495	IRF6;IRF6	grey	-0.1550788	-0.2329712	0.04002745	-0.0064159	0.12900259	0.06238093
cg04687486	FUT10;FUT10;FUT10;FUT1	grey	-0.2377469	-0.1969238	0.26240616	-0.0403838	0.23792984	0.02504006
cg24847621	TRIM10	grey	0.19152968	0.21411329	-0.1681484	0.00559829	-0.0555794	-0.0278159
cg21174817	FAM57B;FAM57B;FAM57	grey	-0.0363098	-0.1607368	0.40282873	0.22884913	0.23822009	0.23098281
cg27113419	NDRG4;NDRG4;NDRG4;N	grey	-0.1546826	-0.0999147	0.0601987	-0.0148597	0.05982821	0.02212508

cg14550066	NCR1	grey	-0.1272196	-0.0673368	0.21549193	0.05698894	0.0272174	0.1488788
cg15779323	PDGFRL;PDGFRL	grey	-0.1567063	-0.2015687	0.0697891	0.0622981	0.12129706	-0.0247067
cg11356485	PCDHA6;PCDHA6	grey	-0.0778556	-0.221458	0.08691221	0.0581255	0.2010997	0.10685824
cg17322242	LENG8;LENG8;LENG8-	grey	0.20577599	0.31808265	0.14936207	0.08244072	-0.1691231	0.12375015
cg15495372	ZNF579	grey	-7.44E-06	0.11349404	0.16508383	0.34740147	0.09546599	0.11502265
cg21367232	SCAMP2;SCAMP2;SCAMP	grey	-0.0250231	0.01324632	0.11901392	-0.0428629	0.05397457	0.01651367
cg12339802	C1orf109;C1orf109;C1orf109	grey	-0.1256957	-0.0029024	0.00419432	-0.0338788	-0.1327602	-0.1759016
cg08033217	USP2;USP2;USP2;RP11-	grey	-0.0205456	-0.0655094	0.05778251	-0.0269847	0.05819625	-0.0407756
cg07123170	PSMD6;PSMD6;PSMD6;PS	grey	0.07454152	-0.0441433	-0.1542207	0.27005081	0.24839203	0.16384454
cg18170071	PAX4;PAX4;PAX4;PAX4	grey	-0.1251889	-0.0821829	0.02615517	0.16703225	0.06477152	0.08528999
cg00373499	RP11-	grey	-0.2517161	-0.1751117	0.15265724	0.10077774	0.20268803	0.00106519
cg14377951	GSTM5;GSTM5;GSTM5;GS	grey	-0.2030586	-0.2460675	0.08593093	0.01071144	0.16101091	0.06211761
cg08976386	IGSF5;TMPRSS3	grey	-0.1874719	-0.1173464	0.28883139	0.1169524	0.16312416	0.00220186
cg25537033	SDK1;SDK1	grey	-0.0800887	-0.0850668	0.26201633	0.13081826	0.09021848	0.09300228
cg15488912	LTK;LTK;LTK;LTK;LTK	grey	-0.0342747	-0.0549904	0.23058076	0.16239623	0.07131241	0.23894907
cg24473270	EEPD1;EEPD1	grey	-0.1977941	-0.151085	0.02804515	-0.0955222	0.03048964	0.21167092
cg16901648	CHST12;CHST12	grey	-0.2736108	0.00096746	0.18308917	0.04339494	-0.1800478	-0.0727102
cg27151561	MDGA1;MDGA1	grey	-0.1512848	-0.1502817	0.00140924	0.10661708	0.13505031	-0.0702586
cg01224234	CTHRC1	grey	0.13582206	-0.0596758	-0.1515905	0.18760489	0.27847241	0.08722478
cg13277380	SNX31;SNX31	grey	-0.1625169	-0.3072956	0.1532546	0.04504577	0.23492314	0.1624154
cg13111919	CCDC111;CCDC111;CCDC	grey	-0.1380107	-0.017276	0.0466204	-0.1613183	-0.1273157	-0.1837656
cg12256550	CREB3L1;CREB3L1	grey	0.00650377	-0.1572814	0.05787319	0.32731642	0.29597983	0.22757092
cg08217526	C14orf64;C14orf64;C14orf64	grey	-0.0970105	0.06612937	-0.0546925	-0.1339868	-0.1691715	-0.0773136
cg07547279	PRKAG2;PRKAG2;PRKAG	grey	0.02781185	-0.1042681	-0.0012895	0.08217002	0.25954085	0.06414806
cg04656811	DTNA;DTNA;DTNA;DTNA	grey	0.11557018	0.01596484	0.14148754	0.18224844	0.11829758	0.19944122
cg01573222	ERP44;INVS;INVS;INVS;IN	grey	0.16604038	0.05650026	0.06208042	0.01384366	0.13852527	0.09233843
cg27119318	WRB;WRB;WRB;WRB;WR	grey	-0.0794077	-0.0704228	0.26456235	-0.0087291	0.0126322	0.05938835
cg20488627	CTD-2561B21.11	grey	0.08164966	0.21733213	0.19807154	0.26370746	0.09663836	0.20074783
cg06636831	RNU5B-	grey	0.01315584	-0.1962506	0.06163276	-0.0091254	0.14599078	0.0501257
cg01908551	METRNL	grey	-0.074611	-0.0095804	0.31098958	0.15800599	0.07878282	0.12683028
cg04085076	HOPX;HOPX;HOPX;HOPX	grey	-0.1833387	0.03790496	0.06734372	-0.026206	-0.1156219	-0.0666196
cg02203922	HUS1B;EXOC2;EXOC2	grey	0.12126092	-0.1574181	0.10157757	0.10616532	0.26977496	0.338589
cg03350299	APOB;APOB;APOB	grey	-0.1456296	-0.2733021	0.03028024	-0.0560261	0.10148134	0.00983458
cg25572968	U6;NDRG4;NDRG4;NDRG	grey	0.01232106	-0.1000315	0.01857833	0.05762759	0.17968663	0.21643393
cg00224202	NHSL1;NHSL1;NHSL1;NH	grey	-0.1717742	-0.1158974	0.18356879	-0.0060071	-0.0404998	0.14102831

cg24380824	LRRTM4;LRRTM4;LRRTM	grey	0.06155392	0.02804286	-0.21556	-0.1825403	0.02457583	-0.2278325
cg08745965	VTRNA2-1	grey	-0.0572817	-0.068174	0.24691422	0.03332539	0.0294714	0.14701818
cg23736297	CTD-2561B21.4;RPTOR	grey	0.19059794	-0.04156	0.26061224	0.37933778	0.2230885	0.0685055
cg21602768	ABHD12;ABHD12;ABHD1	grey	-0.2530461	0.00522802	-0.2909777	-0.2845167	-0.1749273	-0.400112
cg20495040	SLC50A1;SLC50A1;SLC50	grey	-0.1515359	0.03583968	-0.1148695	0.01951168	-0.0625182	-0.110823
cg23528751	UTS2D;UTS2D;UTS2D;CC	grey	-0.2920189	-0.0460033	0.2215953	0.04296844	-0.1729107	0.00987523
cg08533932	PDGFRL;PDGFRL	grey	-0.1938429	-0.2535522	-0.0400997	0.02636496	0.20951171	-0.1130756
cg16024214	LRRC34;LRRC34;LRRC34;L	grey	-0.1568291	-0.2743133	0.20091479	-0.0148846	0.27816578	-0.0451454
cg25912517	PDGFRL;PDGFRL	grey	-0.1188793	-0.2468313	0.05326787	0.07006841	0.21479577	0.05366553
cg06485706	LAT;LAT;RP11-	grey	-0.0629813	-0.0225433	-0.124545	-0.1053844	0.05464263	-0.1456633
cg22132520	EIF4H;EIF4H;EIF4H	grey	-0.2591492	0.08713501	0.2726576	0.27645668	-0.094356	0.02815891
cg06602546	TCEA2;ZNF512B	grey	-0.0515995	-0.3065555	0.28210286	0.02190984	0.31343709	0.06191217
cg12211471	RN5S139;KBTBD12;KBTB	grey	0.35531233	0.31115222	0.12780551	0.25147359	0.21140167	0.02776844
cg25974617	TRAM1;TRAM1;TRAM1;T	grey	-0.07905	-0.0134026	0.19284514	0.16400786	0.02490428	-0.0541294
cg14279329	RP11-646E18.4	grey	0.06013668	0.10615544	-0.0495335	-0.2085283	-0.2425834	-0.076918
cg11469778	ARHGEF4;ARHGEF4;ARH	grey	-0.210528	-0.3044278	0.07410493	-0.0062388	0.1447692	0.06755459
cg13784833	ARHGEF7;ARHGEF7;ARH	grey	-0.1024873	-0.2677608	-0.1326648	-0.029217	0.26300946	-0.0838802
cg16151451	TSPAN4;TSPAN4;TSPAN4;	grey	-0.0410535	-0.0122998	0.27035294	0.14295452	0.00159499	0.07909854
cg03760072	SRPRB	grey	-0.0273067	-0.1678062	0.10729812	0.12666217	0.15293989	0.24585927
cg04006327	ATG16L2;ATG16L2;ATG16	grey	0.01637673	-0.157519	0.33371235	-0.0997902	0.11229401	0.19762109
cg09009771	MORC3;CHAF1B;CHAF1B;	grey	-0.0888851	0.03873827	0.22430089	0.16168831	-0.0530932	0.16044333
cg16991868	GPATCH3;GPATCH3;GPA	grey	-0.1408879	-0.2676752	0.05578821	0.07723541	0.20941792	0.14483216
cg18167492	RAB23;RAB23	grey	-0.0719353	-0.178594	0.05243345	0.30849365	0.31912261	0.1533247
cg19971049	RAB43;RAB43;RAB43;RAB	grey	0.12795202	-0.053415	0.22293052	0.36088166	0.25409488	0.33510385
cg22921096	FAM131A;FAM131A;FAM	grey	0.08802269	-0.0584571	0.21076518	0.24835175	0.25346108	0.14608448
cg13795252	DEFB121	grey	0.11286839	0.27932668	-0.0004534	0.06208856	-0.1792915	-0.0297717
cg07648456	WNT5A	grey	0.15656973	0.07152139	0.10748257	0.01435111	0.09800509	0.0069522
cg12848831	LIMCH1;LIMCH1;LIMCH1;	grey	-0.1691726	-0.1641776	0.19586441	0.05678085	0.22086031	-0.0339536
cg22174935	SORBS1;SORBS1;SORBS1	grey	0.12010297	-0.0498248	0.10169025	0.28922113	0.2369529	0.15334781
cg08725083	CHMP3;CHMP3;CHMP3;C	grey	-0.0829487	-0.134051	0.11782414	0.25400569	0.24495161	0.01076812
cg26127836	H2AFJ;H2AFJ;H2AFJ;H2A	grey	-0.2143921	-0.346397	0.10273017	0.03809177	0.14446099	0.08312634
cg23018448	QTRT1;QTRT1	grey	0.04224487	0.06614278	0.03805701	0.18856547	0.09023638	0.23568778
cg04714994	LRRC34;LRRC34;LRRC34;L	grey	-0.0531343	-0.1747403	0.28553097	0.01580918	0.2292177	0.05336072
cg03860311	CAMK2B;CAMK2B;CAMK	grey	-0.1094941	-0.11905	0.13557272	0.05351327	-0.0059586	0.05368193
cg11625868	ZFP90;ZFP90;ZFP90;ZFP90;	grey	-0.2177933	0.00807732	0.26276908	0.09000908	-0.1466772	-0.1643081

cg03465499	MRGPRF;MRGPRF;MRGP	grey	-0.1265468	-0.2165749	0.11671397	-0.0072621	0.20492168	0.0432109
cg00260114	CAST;CAST;CAST;CAST;C	grey	-0.2009781	-0.2033407	-0.0942755	-0.1863857	-0.0124714	0.10090313
cg22805436	RP11-429J17.6;RP11-	grey	-0.1029359	-0.0419565	0.1685019	0.08043588	0.13194114	-0.0305593
cg14217495	FCRL6;FCRL6;FCRL6	grey	-0.3429637	-0.0281844	0.10179141	-0.0413807	-0.1465514	-0.02759
cg01554410	IRX6;RP11-26L20.3	grey	-0.0445833	-0.1939257	0.14642281	0.05194081	0.19759485	0.12803682
cg14560040	SURF6	grey	0.09776885	-0.0773203	0.23688997	0.19835874	0.25560252	0.32334766
cg20816757	CX3CL1;CX3CL1;CX3CL1	grey	-0.0121062	-0.064197	0.38453037	0.22540603	0.26864035	0.15363317
cg21605781	APPL2;APPL2;APPL2;APP	grey	-0.1185962	0.0582726	0.12577412	0.03295323	0.07543033	0.04372936
cg03335125	CCDC17;CCDC17;CCDC17;	grey	0.1097044	-0.0884348	0.10168376	0.14408995	0.23580061	0.00757847
cg10309005	CYP4V2;CYP4V2;CYP4V2	grey	0.01006544	-0.0544035	0.20797971	0.11160123	0.06895432	0.1539628
cg05155922	HOPX;HOPX;HOPX;HOPX	grey	-0.1444719	0.07201659	0.11046225	-0.0135946	-0.1690854	-0.0017989
cg02445972	CLDN7;RP1-	grey	-0.1393742	-0.2285109	-0.2351544	-0.1419267	0.21364072	-0.4555931
cg21627409	PCDHGA12	grey	-0.0440386	-0.282649	0.27204832	-0.0326814	0.22778932	0.20413602
cg24622817	MKL1;MKL1;MKL1;MKL1;	grey	0.01366719	0.09006276	-0.2423676	-0.0136716	-0.0128332	-0.0872537
cg02509370	KCNH3	grey	-0.0486319	-0.0377673	-0.1077171	-0.0880392	0.00107215	0.05756695
cg24334507	WISP2;WISP2;WISP2;WISP	grey	-0.0268049	0.24547412	0.18542571	0.17050754	-0.0030321	0.03115847
cg02918577	RGMA;RGMA;RGMA	grey	-0.257569	-0.2987439	0.0376965	0.10515998	0.13239692	-0.0150344
cg19761502	WRB;WRB;WRB;WRB;WR	grey	-0.1229114	-0.094757	0.29384447	0.01714967	0.03062596	0.02250968
cg19453250	SLC39A6;SLC39A6;ELP2;E	grey	0.06437703	0.09611542	0.10486591	-0.0779647	-0.0876782	0.01170911
cg15545541	LTB4R2;CIDEB;CIDEB;CID	grey	0.04460436	-0.1542394	0.05353935	0.18326863	0.28785346	0.00495274
cg20540428	PPP4R2;PPP4R2;PPP4R2;PP	grey	0.04587969	0.04240368	0.07012307	0.09111475	0.08896859	-0.072608
cg01801283	CGN;CGN;CGN;CGN	grey	-0.1986916	-0.253713	-0.0339073	0.04793157	0.20912089	-0.0062174
cg21909996	SRRM3;SRRM3;SRRM3	grey	-0.2017989	0.08611486	0.34143951	0.09365765	-0.1506548	-0.0064522
cg00718959	CTC-558O2.2	grey	-0.0077156	0.03283988	0.17914957	0.04472397	0.07594462	0.05672902
cg26900558	TTC24;TTC24;TTC24;TTC24	grey	0.01221315	-0.1718636	0.17264165	0.1080648	0.14513066	0.20841579
cg00398510	ATF7IP2;ATF7IP2;ATF7IP2	grey	-0.2450558	-0.0938734	0.06233428	-0.1610765	-0.1959597	-0.0457128
cg27467234	LRRC34;LRRC34;LRRC34;L	grey	-0.0462304	-0.1632962	0.19247555	0.03625431	0.23668071	0.02128501
cg17385452	TMF1;TMF1;TMF1	grey	-0.3030063	-0.1139104	0.13240979	0.24510305	0.20954706	-0.0492504
cg16150451	EMX1	grey	-0.1213379	-0.2312232	-0.2310121	-0.2368523	0.18438956	-0.3965447
cg22010712	RP11-474N8.5	grey	0.10732533	0.00815566	0.30472289	0.23613417	0.17698303	0.47582168
cg09045655	HOXC9;HOXC9;HOXC-	grey	-0.0962038	-0.2495457	0.18975556	0.08583649	0.24729981	0.09553794
cg00105512	SYCE1L;SYCE1L;RP11-	grey	0.05957615	-0.0215803	-0.0142529	0.20400555	0.20857409	-0.0478613
cg19684207	WRB;WRB;WRB;WRB;WR	grey	-0.0635076	-0.0491863	0.24883832	-0.0017163	0.01290414	0.01454586
cg14208374	AL031963.2	grey	0.23352528	0.24581124	0.35272114	0.35703305	0.0678824	0.3853363
cg17361154	ENTPD8;ENTPD8	grey	-0.1755385	-0.2985331	0.16239632	-0.00947	0.29340306	-0.006644

cg16313343	BRF1;BRF1;BRF1;BRF1;BR	grey	-0.2076159	-0.2372027	0.24391038	-0.0386596	0.15871029	0.00413692
cg10431713	SLFN12;SLFN12;SLFN12;S	grey	-0.0241196	-0.0610818	0.08645427	0.04541214	0.00940937	0.06687422
cg00066816	IL12B;AC008697.1;AC00869	grey	-0.2997385	-0.2750005	0.08599376	0.06890284	0.15961063	-0.0023859
cg26257814	CTD-2126E3.1	grey	-0.1811765	-0.2549176	0.17180834	0.15737973	0.21002117	-0.0235862
cg23772011	C1QTNF6;C1QTNF6;C1QT	grey	-0.1261829	0.16999882	0.18083115	-0.0929951	-0.18371	-0.1802431
cg18460466	TPM3;TPM3;TPM3	grey	0.13845817	0.2568961	0.16504436	0.21122215	0.04593662	0.17511119
cg23231670	RAET1L;RAET1L	grey	-0.1481441	-0.1728867	0.21138066	-0.1146592	0.11151404	0.15581515
cg25856644	TRAK1;TRAK1;TRAK1;TR	grey	-0.1011627	-0.2765971	0.01745264	0.19203355	0.22855555	0.05983751
cg01877814	POLG;RP11-	grey	-0.2794055	-0.2880034	0.11711141	0.03714058	0.08779433	-0.0977751
cg13084335	HSPB2;CRYAB;CRYAB;CR	grey	-0.103003	0.20859283	0.04411892	0.06570015	-0.1517789	0.08568235
cg00654322	CTD-3193O13.1;SNAPC2	grey	-0.00418	-0.2324902	0.22745137	0.13475058	0.25583223	0.26536476
cg05994982	PI4K2A	grey	-0.160724	-0.3173813	0.10576823	0.13363024	0.28104488	0.07818938
cg25898146	MYOM2;MYOM2	grey	-0.0029664	-0.0860337	0.18887546	0.03770917	0.06689652	0.10635983
cg00668749	HAS3;HAS3;HAS3;HAS3	grey	-0.0785365	-0.1376619	-0.1565661	0.16139183	0.15961148	-0.0153369
cg25021939	CLCNKA;CLCNKA;HSPB7;	grey	-0.0016575	0.20659263	0.16685997	0.1649493	-0.0621944	0.2247032
cg17014785	ANKRD45;ANKRD45	grey	-0.0161537	-0.0669526	-0.2197321	0.06888639	0.14974475	-0.1817284
cg10102384	PACRG;PACRG;PACRG;P	grey	-0.0273635	-0.1867987	0.14366217	0.28683782	0.2529939	0.19621751
cg03213860	PAK1;PAK1;PAK1;PAK1;P	grey	-0.1635461	-0.1379876	0.09362122	0.09617238	0.0466892	0.06480151
cg04375683	PPP2R1A;PPP2R1A;PPP2R	grey	0.07014975	-0.0165714	0.15296308	0.04988893	0.08149531	0.09501618
cg06023345	RP11-	grey	-0.1526716	-0.1926004	0.27677431	0.02523867	0.14852164	-0.0259372
cg11065385	B4GALNT3;B4GALNT3	grey	-0.3075655	-0.259254	0.15480202	-0.0145262	0.09327867	-0.1031605
cg04663285	B4GALNT4;B4GALNT4	grey	0.07150154	0.01767097	0.00982641	0.03218252	-0.0703563	0.10486589
cg15734436	HLA-DPB1;HLA-	grey	0.20927765	0.1300057	0.07451419	0.22684021	0.18079906	0.33110371
cg05494220	HSD17B12;HSD17B12;HSD	grey	-0.2399074	-0.0938105	0.15674792	0.09681529	0.13095148	-0.0751929
cg19508671	CTD-2184D3.4;CTD-	grey	0.04488936	0.15747748	0.02849722	-0.1326436	-0.0963521	-0.1210193
cg25340688	VTRNA2-1	grey	-0.0826319	-0.0998147	0.20855539	-0.008848	0.02137977	0.08928435
cg19118791	C9orf174;C9orf174;C9orf174	grey	-0.1224396	-0.1691342	0.1826626	0.06370719	0.12939566	0.08863487
cg21593676	KLHL8;KLHL8;KLHL8;KL	grey	-0.1361969	-0.1215592	0.13632188	0.21654674	0.1899296	0.11386176
cg24309555	APOB;APOB;APOB	grey	-0.1336223	-0.2277357	0.02189541	-0.0445912	0.09193662	0.01252371
cg23035274	SYNRG;SYNRG;SYNRG;S	grey	-0.2680952	-0.0606199	0.26382515	0.19564707	-0.0829109	0.06981254
cg21705215	RP4-669H2.1;TSHZ2;TSHZ2	grey	-0.1362629	-0.1737386	0.19011339	0.20076992	0.20609695	-0.0290738
cg27438304	C11orf9;C11orf9;RP11-	grey	-0.0869774	-0.1827884	-0.009083	0.07743475	0.18256478	0.06753807
cg23448153	NANOS2	grey	0.11310445	0.0407562	0.03286563	0.32030837	0.17434801	0.06585718
cg24041556	SLC44A2;SLC44A2	grey	0.15672291	-0.0604019	0.16094442	-0.0087634	0.15042571	0.24596171
cg05628902	C12orf44;C12orf44;C12orf44	grey	0.07524835	0.00190352	0.06219812	0.03240724	0.06046093	-0.0015803

cg14816899	DISC1	grey	-0.2763028	-0.2624592	0.14625265	0.1096361	0.17310215	-0.1381958
cg03967504	SLC11A1;SLC11A1;SLC11	grey	-0.2056199	-0.0919273	0.27533942	-0.0350593	0.09656784	0.05417479
cg27524531	WDR90;WDR90;WDR90;W	grey	-0.0070267	0.01126127	0.08180431	0.19537826	0.16377766	0.0862822
cg04004590	PRKG1;PRKG1	grey	-0.0934978	-0.1000752	0.03607138	0.0906731	0.20272845	0.11140437
cg27380999	KSR2;KSR2	grey	-0.366561	-0.3687584	0.0787847	-0.045714	0.18990793	-0.1072845
cg00879495	GTPBP2;GTPBP2;GTPBP2;	grey	-0.0560016	-0.0988932	0.28207148	0.23553352	0.26002361	0.19387294
cg12181083	SULF1;SULF1;SULF1	grey	-0.1162281	-0.0179234	0.14122681	-0.0050712	0.00733642	0.0529671
cg08770761	ATP6V0C;ATP6V0C;RP11-	grey	-0.3195986	-0.295454	0.04490539	-0.2432406	-0.0252491	-0.020635
cg15182643	EGFLAM;EGFLAM	grey	-0.1319045	-0.2912046	-0.0649647	-0.1044024	0.1818781	-0.3397269
cg17129400	NCOA6;NCOA6	grey	-0.2348588	-0.052802	0.27061117	0.1891833	-0.002808	0.1125124
cg14861570	MMD;MMD;MMD	grey	-0.158265	-0.1103404	0.09005587	0.20023893	0.21562722	-0.0191442
cg10349685	DLC1;DLC1;DLC1;DLC1	grey	-0.0421635	-0.0436578	0.14879059	0.04803943	0.11705546	0.00238115
cg17037355	SLC4A11;SLC4A11;SLC4A	grey	-0.0774715	-0.0963065	0.26399113	0.13218552	0.12281113	0.01889077
cg13385408	CHN2;CHN2;CHN2;CHN2	grey	0.31596549	0.12472533	0.0272806	-0.0052905	0.10931363	0.15872087
cg11515438	KLK12;KLK12;KLK12;KLK	grey	0.04779098	0.14303011	0.2197748	0.20020632	-0.0038647	0.22988456
cg19978055	KIAA1875;SHARPIN	grey	0.16695978	0.06366566	0.22930104	0.33111875	0.24282899	0.19198464
cg08427977	VSTM4	grey	0.06253727	-0.0565231	0.23129582	0.20348427	0.08907649	0.11812055
cg18174404	NMU;NMU;NMU;NMU;N	grey	-0.2579176	-0.2848248	0.35037626	-0.0276939	0.16831097	0.04093681
cg21779786	H1F0;H1F0	grey	-0.0170406	-0.1382528	0.12039101	0.11654247	0.14500351	0.08885959
cg14253327	RP11-35N6.1	grey	-0.0109784	0.19929654	0.01684357	-0.1185641	-0.1360499	-0.1235413
cg23671319	POU6F2;POU6F2;POU6F2-	grey	-0.1827802	-0.0523864	0.14426531	0.07026646	0.03711962	0.0585126
cg05076576	SPEG;AC053503.11	grey	-0.1927584	-0.2029048	0.19093545	0.13507363	0.06397073	0.13606723
cg13698714	DHRS3;DHRS3;DHRS3	grey	-0.3006751	-0.3044508	0.18631921	0.06739621	0.06721583	-0.0221401
cg22283921	TP53INP1;TP53INP1;RP11-	grey	-0.0938449	0.06999483	-0.1544658	-0.169332	-0.1727181	-0.087918
cg23966736	LIMCH1;LIMCH1;LIMCH1;	grey	0.13075671	0.09546699	0.0222056	0.10738444	0.06539998	0.07588275
cg21974102	ADRA2B	grey	-0.1653614	-0.0803968	-0.0063487	0.14139005	0.08644799	-0.0303515
cg25210835	GSTM5;GSTM5;GSTM5;GS	grey	-0.1671033	-0.2134675	0.08067834	0.02330055	0.13920472	0.04770293
cg05177637	MATN3;MATN3	grey	0.12310023	-0.0697731	-0.0095556	0.17473544	0.28000922	0.12091181
cg06230848	RP11-89K10.1;RP11-	grey	-0.1104915	-0.1521307	0.26107895	-0.0375635	0.07351747	0.07102274
cg24576494	RP11-347H15.4;RP11-	grey	0.0928185	-0.1311659	0.28179906	0.13894203	0.1711521	0.22702453
cg17105597	HCN4	grey	0.06583457	-0.0078287	0.18582452	0.10344305	0.06106625	0.22117787
cg06143905	FGF20;FGF20;FGF20	grey	-0.14316	-0.2300789	0.26720141	0.07582006	0.26411473	0.08454722
cg15741433	HIST1H1A	grey	0.11301527	-0.1173122	0.20481185	0.07253859	0.11393288	0.16031958
cg15686942	IQSEC1;IQSEC1;IQSEC1	grey	0.07233651	0.26235727	0.07553133	0.2707392	-0.0560762	0.16929221
cg24255896	AGXT2L1;AGXT2L1;AGXT	grey	-0.042504	-0.1745281	-0.0882658	-0.034517	0.16488442	0.07929856

cg19234738	RP11-200A1.1	grey	-0.0338587	0.02352879	0.14748628	0.11174604	0.08004814	-0.0129814
cg07534843	RBPJL;MATN4;MATN4;M	grey	-0.2419705	-0.2659047	0.04333933	0.02440122	0.22795342	-0.087238
cg22899561	BCAR1;BCAR1;BCAR1;BC	grey	0.00715653	0.20791158	0.43753289	0.27861227	-0.0367659	0.2631165
cg12396970	THBS4;THBS4;CTD-	grey	0.09112048	0.02022929	-0.1955095	0.04243795	0.09135412	-0.0937966
cg13816655	CD320	grey	0.31179806	0.16645272	0.07591437	0.38048384	0.18983287	0.25361371
cg21964762	RAB37;RAB37;RAB37;RAB	grey	-0.211512	-0.1737825	0.28008759	0.09261547	0.072924	-0.0103083
cg01209566	RP11-1082L8.2;RP11-	grey	0.06109405	0.1329036	0.0389494	0.07442938	-0.0499187	0.14576137
cg19374305	DNAH7;DNAH7	grey	0.09054241	-0.0966296	0.0990579	0.18191812	0.30846224	0.10532501
cg02996131	SYNE1;SYNE1;SYNE1;SY	grey	0.04564318	-0.0942173	0.11034813	0.25114374	0.24916118	0.1618676
cg27251412	AIRE;AIRE;AIRE;AIRE;AIR	grey	0.13081584	-0.1159839	0.15421236	0.21366192	0.25387838	0.1309276
cg00579402	FUT6;FUT6;FUT6;FUT6;FU	grey	0.14328602	-0.0644181	0.28185653	0.17145697	0.16744031	0.2770685
cg03217528	RP11-	grey	-0.0576163	-0.0945178	0.26026628	0.18440654	0.20571335	-0.0043876
cg04472261	STON2;STON2;STON2	grey	0.140696	0.14763162	-0.1983351	0.2789941	0.13480381	0.13588653
cg13239252	LRRIQ1;TSPAN19;TSPAN1	grey	0.03779128	-0.0127775	-0.204782	-0.2048369	0.00473958	0.08153617
cg22577520	ARGLU1;ARGLU1;ARGLU	grey	-0.1513394	0.10842537	0.15117029	0.28820995	0.00937572	-0.0190453
cg08672999	ZNF836	grey	-0.1780171	-0.201061	0.11619427	0.06779523	0.12101517	0.06337247
[truncated: 773,025 more chars]
