# Supplementary material for: Blood epigenome-wide association studies of suicide attempt in adults with bipolar disorder
Source: Transl Psychiatry. 2024 Jan 31;14:70. doi: 10.1038/s41398-024-02760-y (PMC10831084; doi:10.1038/s41398-024-02760-y)
Supplement: Supplementary file 1 — Supplementary Figures S1-S10 [file 41398_2024_2760_MOESM1_ESM.pdf]

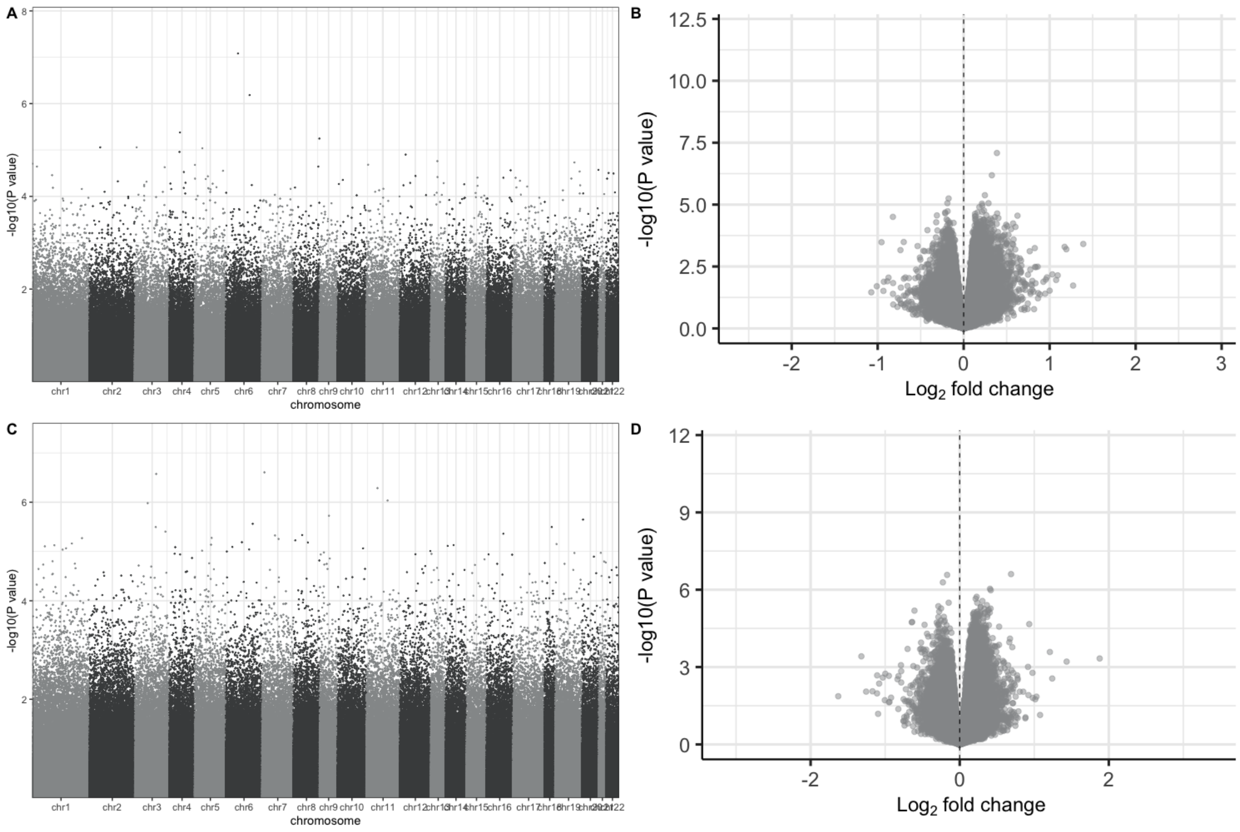

Supplementary Figure S1. A shows the Manhattan plot for the BD/SA vs. CON EWAS, **B** shows the Volcano plot for the BD/SA vs. CON EWAS, **C** shows the Manhattan plot for the BD/non-SA vs. CON EWAS, and **D** shows the Volcano plot for the BD/non-SA vs. CON EWAS. None of the differentially methylated positions passed false discovery rate (FDR) $q <$ 0.05. BD - bipolar disorder; CON - controls; EWAS - epigenome-wide association study; FDR - false discovery rate; SA - suicide attempt.

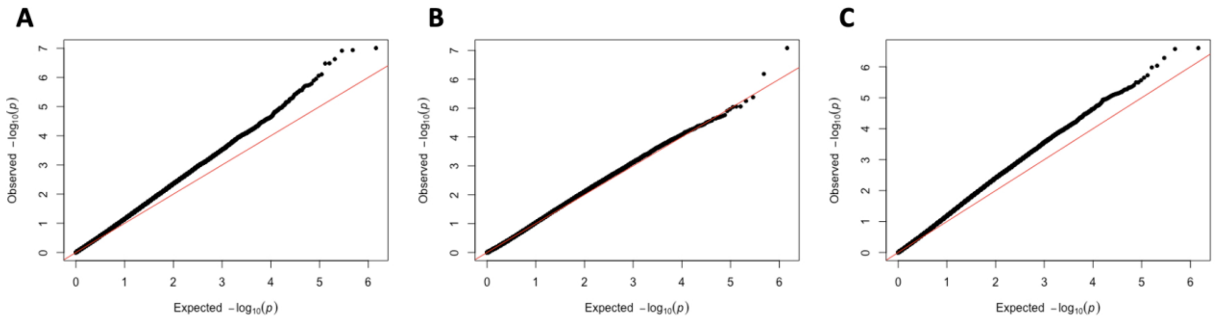

Supplementary Figure S2. Quantile-quantile (QQ) plots for the discovery cohort EWAS. **A** shows the QQ plot for the BD/SA vs. BD/non-SA EWAS (lambda inflation factor = 1.20), **B** shows the QQ plot for the BD/SA vs. CON EWAS (lambda inflation factor = 0.90), and **C** shows the QQ plot for the BD/non-SA vs. CON EWAS (lambda inflation factor = 1.21). BD - bipolar disorder; CON - controls; EWAS - epigenome-wide association study; SA - suicide attempt.

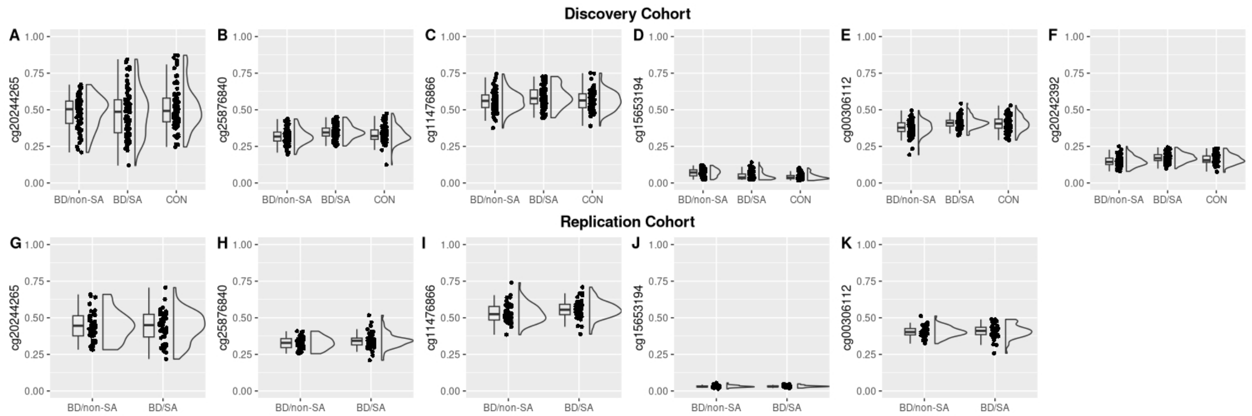

Supplementary Figure S3. Beta-value methylation levels for each discovery cohort FDR-significant DMP in the discovery cohort (**A-F**) and the replication cohort (**G-K**), stratified by sub-group (BD/non-SA, BD/SA, CON). CON are not present in the replication cohort. Also, cg20242392 failed quality controls in the replication cohort so is not included there. Findings demonstrate that there is not a consistent stepwise pattern in methylation associated with stepwise changes in severity (CON -> BD/non-SA -> BD/SA). This could suggest nonlinear/discontinuous methylation differences between groups, and a specific epigenetic architecture of SA which is separable from BD. Further, there is some consistency in sub-group differences across cohorts but also some inter-cohort heterogeneity. BD - bipolar disorder; CON - controls; DMP - differentially methylated positions; FDR - false discovery rate; SA - suicide attempt.

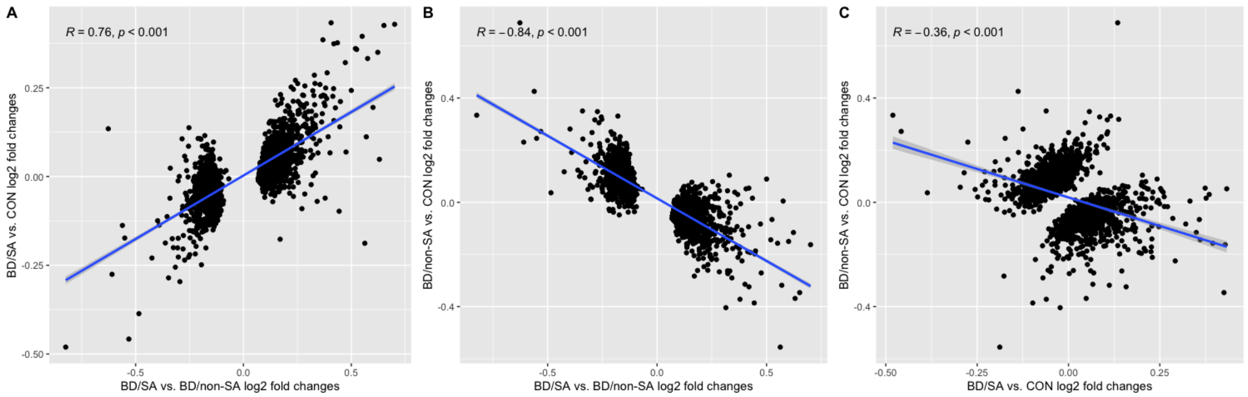

Supplementary Figure S4. Correlations among log2 fold changes for the three EWAS in the discovery cohort, focused on the DMPs at $p < 0.001$ in the BD/SA vs. BD/non-SA EWAS. **A** shows a strong positive correlation among log2 fold changes between BD/SA vs. BD/non-SA and BD/SA vs. CON EWAS, suggesting similar epigenetic patterns distinguishing BD/SA from BD/non-SA and BD/SA from CON. **B** shows a strong negative correlation between BD/SA vs. BD/non-SA and BD/non-SA vs. CON EWAS, suggesting differing patterns distinguishing BD/SA from BD/non-SA and BD/non-SA from CON and possibly indicating that BD/SA is not an exacerbation of BD/non-SA pathophysiology relative to CON. **C** shows a weak negative correlation between BD/SA vs. CON and BD/non-SA vs. CON EWAS, again suggesting that the differentiation between BD/SA and CON, and BD/non-SA and CON, is separable at the DMPs, which is expected given the results of the main EWAS. BD - bipolar disorder; CON - controls; DMP - differentially methylated probes, EWAS - epigenome-wide association study.

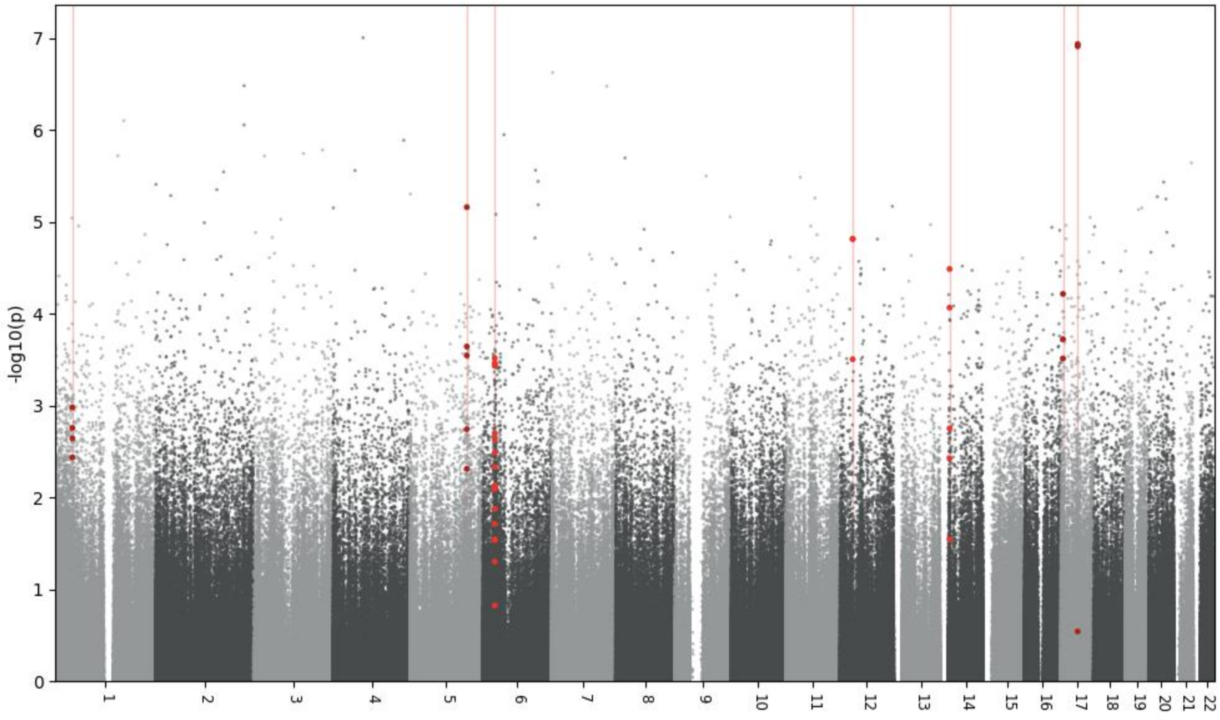

Supplementary Figure S5. Manhattan plot for DMRs, with Šidák-significant DMRs highlighted by red vertical lines, where individual points highlighted in red represent the associated CpG probes. The -log10 *p*-value for each individual probe can be seen on the y-axis. DMR - differentially methylated region.

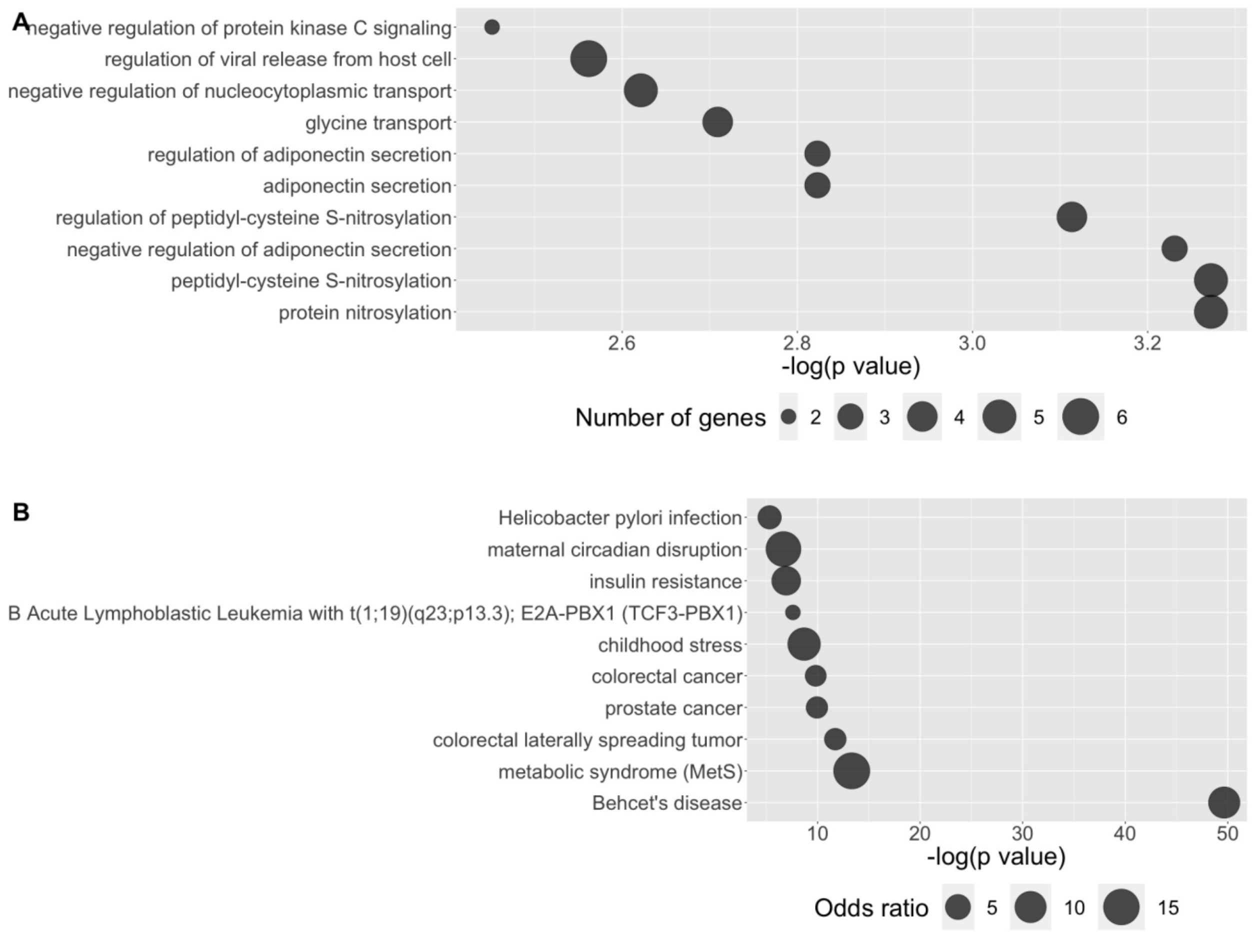

Supplementary Figure S6. Gene ontology and trait enrichment results for the discovery cohort BD/SA vs. CON EWAS using the DMPs at nominal $p < 0.001$. **A)** shows the top ten gene ontology pathways ordered by -log(p value), with the number of genes assigned to each pathway represented by the size of the point. **B)** shows the top ten traits ordered by -log(p value), with the odds ratio for each trait represented by the size of the point.

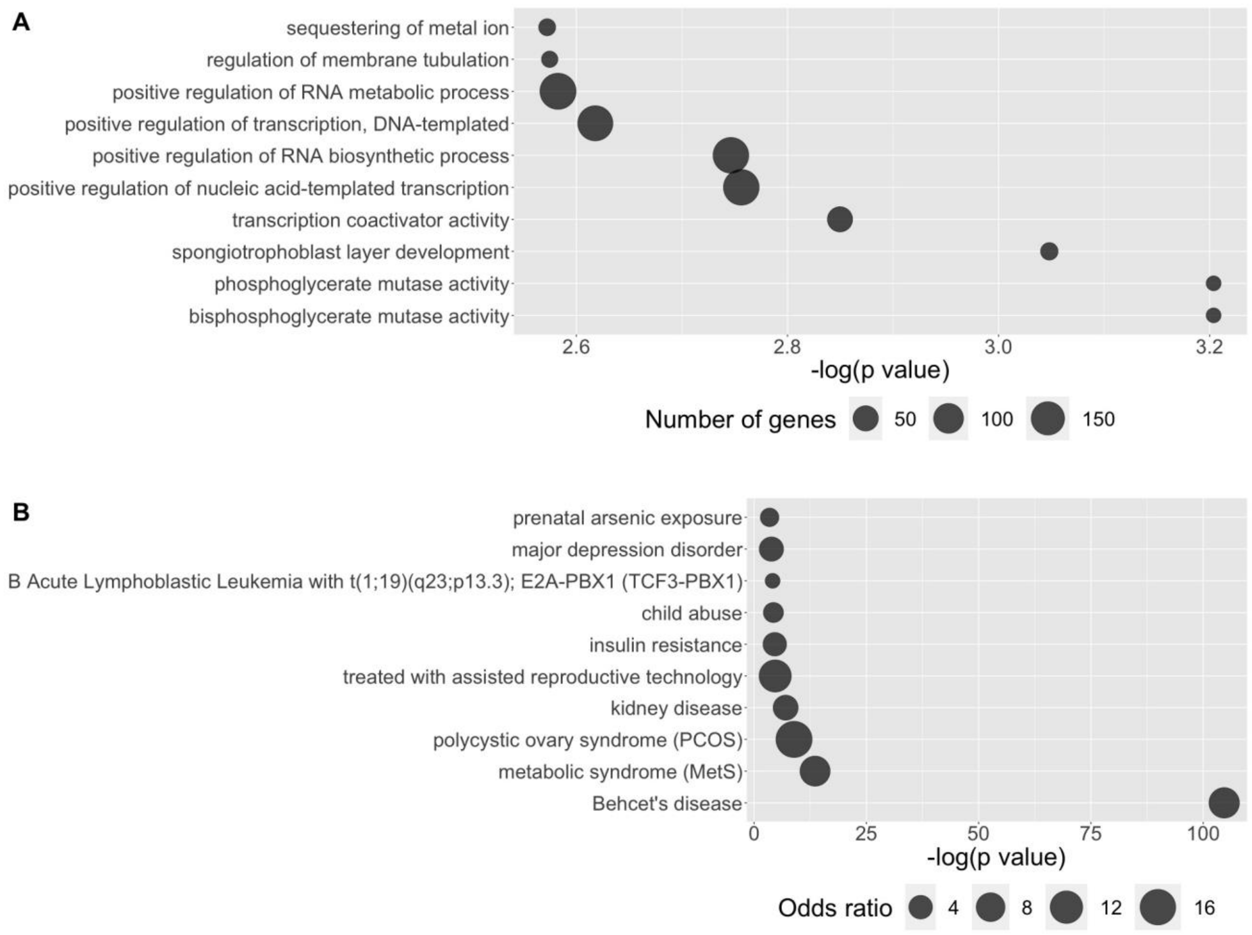

Supplementary Figure S7. Gene ontology and trait enrichment results for the discovery cohort BD/non-SA vs. CON EWAS using the DMPs at nominal $p < 0.001$. **A)** shows the top ten gene ontology pathways ordered by -log(p value), with the number of genes assigned to each pathway represented by the size of the point. **B)** shows the top ten traits ordered by -log(p value), with the odds ratio for each trait represented by the size of the point.

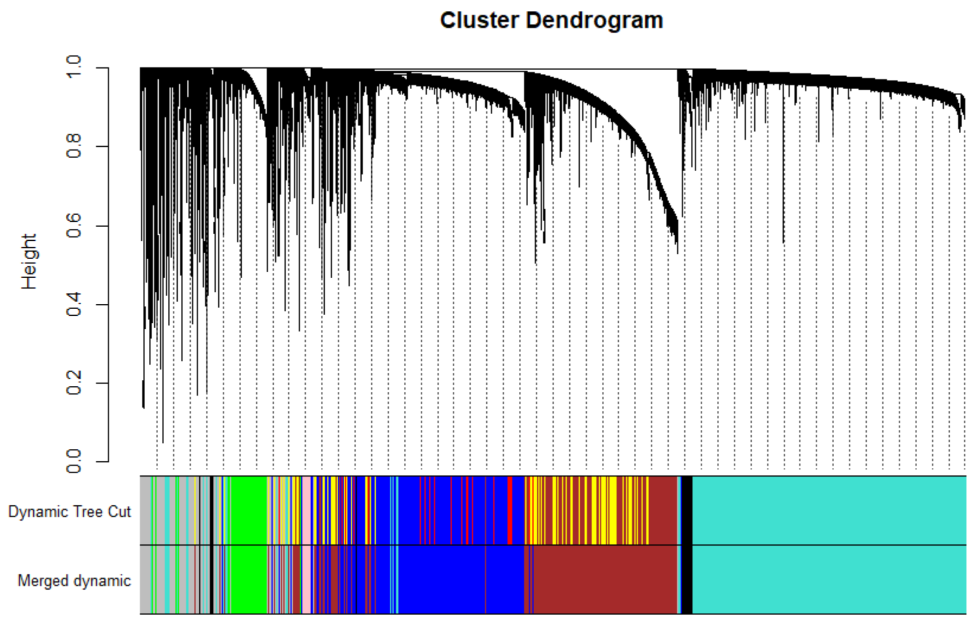

Supplementary Figure S8. Cluster dendrogram for the weighted gene co-methylation network analysis (WGCNA).

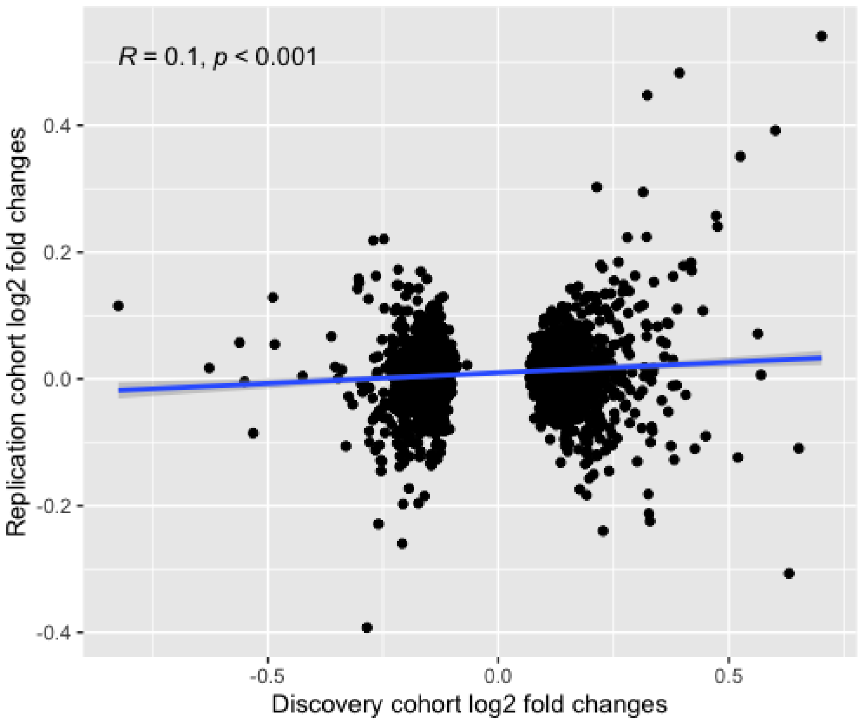

Supplementary Figure S9. Attempt to replicate patterns of log2 fold changes in the BD/SA vs. BD/non-SA contrast across the discovery and replication cohorts. Scatter plot of the log2 fold changes for the 1,958 discovery cohort sites with a nominal $p < 0.001$ (missing 114 probes which failed quality control in replication cohort) across both cohorts, with the blue line showing the linear fit ($r = 0.10$). BD - bipolar disorder; SA - suicide attempt.

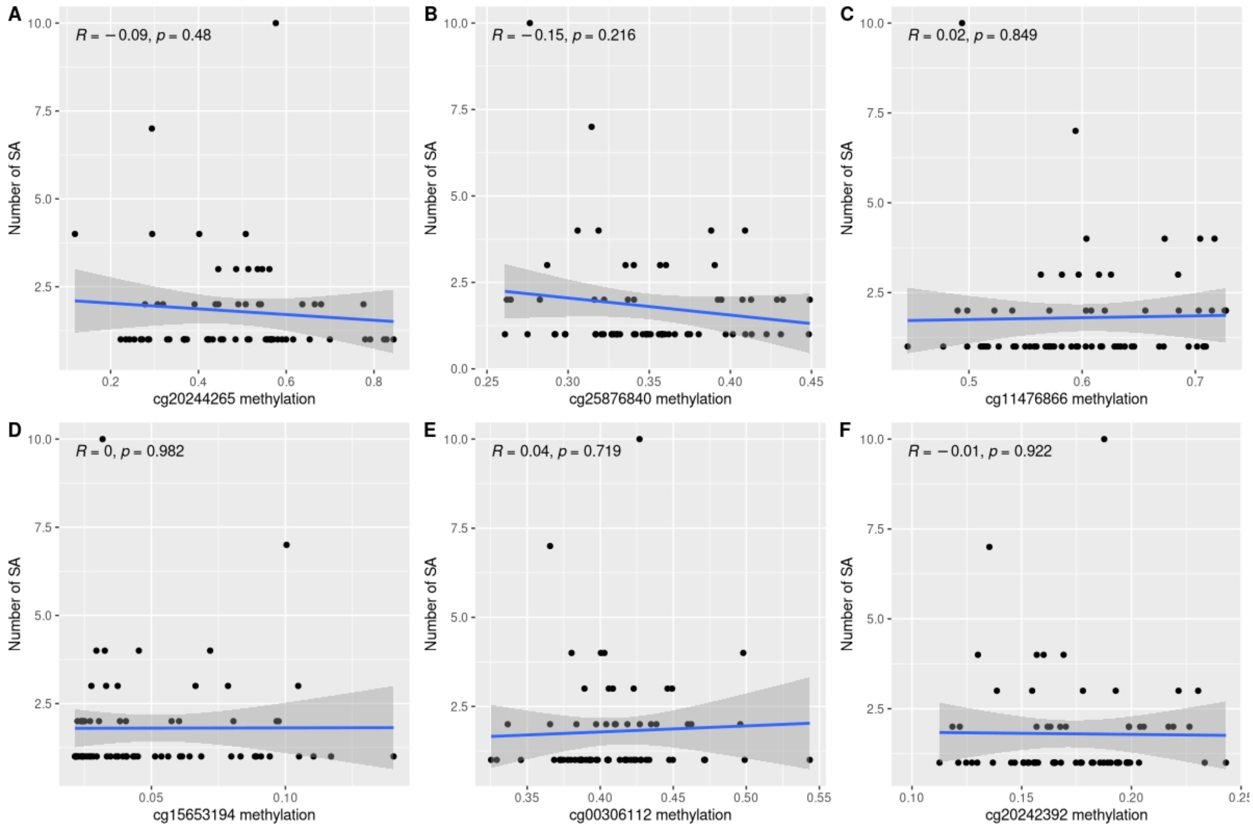

Supplementary Figure S10. Scatter plots depict relationships between discovery cohort FDR-significant DMP methylation (beta-values) and the total number of lifetime SAs within the BD/SA group. Correlation coefficients and *P*-values are presented in the upper left of each plot. FDR - false discovery rate; DMP - differentially methylated position; SA - suicide attempt; BD - bipolar disorder.
